# Gene fusion as an important mechanism to generate new genes in the genus *Oryza*

**DOI:** 10.1186/s13059-022-02696-w

**Published:** 2022-06-15

**Authors:** Yanli Zhou, Chengjun Zhang, Li Zhang, Qiannan Ye, Ningyawen Liu, Muhua Wang, Guangqiang Long, Wei Fan, Manyuan Long, Rod A. Wing

**Affiliations:** 1 Germplasm Bank of Wild species, Kunming Institute of Botany, Chinese Academy of Science, Kunming, Yunnan 650201 China; 2 Department of Ecology and Evolution, The University of Chicago, 1101 E. 57th Street, Chicago, IL 60637 USA; 3 Chinese Institute for Brain Research, (CIBR), Beijing, 102206 China; 4 Arizona Genomics Institute, School of Plant Sciences, University of Arizona, Tucson, AZ 85721 USA; 5 State Key Laboratory for Biocontrol, School of Marine Sciences, Sun Yat-sen University, Zhuhai, 519000 China; 6 Key Laboratory of Medicinal Plant Biology of Yunnan Province, Yunnan Agricultural University, Kunming, Yunnan 650201 China; 7 Center for Desert Agriculture, King Abdullah University of Science & Technology, Thuwal, 23955-6900 Kingdom of Saudi Arabia

**Keywords:** Fusion gene, *Oryza*, Evolutionary patterns, Phenotype

## Abstract

**Background:**

Events of gene fusion have been reported in several organisms. However, the general role of gene fusion as part of new gene origination remains unknown.

**Results:**

We conduct genome-wide interrogations of four Oryza genomes by designing and implementing novel pipelines to detect fusion genes. Based on the phylogeny of ten plant species, we detect 310 fusion genes across four Oryza species. The estimated rate of origination of fusion genes in the Oryza genus is as high as 63 fusion genes per species per million years, which is fixed at 16 fusion genes per species per million years and much higher than that in flies. By RNA sequencing analysis, we find more than 44% of the fusion genes are expressed and 90% of gene pairs show strong signals of purifying selection. Further analysis of CRISPR/Cas9 knockout lines indicates that newly formed fusion genes regulate phenotype traits including seed germination, shoot length and root length, suggesting the functional significance of these genes.

**Conclusions:**

We detect new fusion genes that may drive phenotype evolution in Oryza. This study provides novel insights into the genome evolution of Oryza.

**Supplementary Information:**

The online version contains supplementary material available at 10.1186/s13059-022-02696-w.

## Background

New genes, which have been shown to be critical in the understanding of phenotypic evolution [1, 2], can be generated by a number of molecular mechanisms [3, 4], including gene duplication, de novo evolution, and gene fusion. Thus, investigation of the magnitude, origination, and corresponding evolutionary processes underlying the formation and fixation of new genes is critical to our understanding of the evolution of genome complexity that contributes to protein diversity. New genes formed by gene fusion can occur between 2 ancestrally neighboring genes, and sometimes lead to the evolution of novel complex domain structures [4–7]. In terms of the parental materials (DNA or RNA sequence) that produced to process, the fusion event results into fusion gene and retro-fusion gene, respectively.

Gene fusion was found to be a potent process for evolutionary novelties in bacteria. For example, in 18 bacteria species, including *Escherichia coli*, it was found that 0.2–1% of the genes in each genome were fusion genes [8]. However, it has been a challenge to understand the role of gene fusion in multicellular eukaryotic organisms. Early medical genetic studies revealed that some cancer-genesis mutations have arisen from the fusion of 2 adjacent genes in the genomes of cancer patients [9], implicating the role of gene fusion as a mechanism for new gene evolution, even though such fused genes may have deleterious consequences upon their birth. Analyses of the origination of fusion genes in humans and other hominoids have shown that they can form at both the DNA (i.e., alternative splicing site skip or mutation, transposon element related movement or recombination) and RNA levels (i.e., retroposition with subsequent flanking sequence recruitment [5, 9, 10]), thereby revealing the diverse molecular processes that can lead to gene fusions.

Whether or not gene fusion is a general mechanism for new gene evolution in plants remains unknown. However, recent sequencing efforts in several model organisms have predicted that gene fusion is likely an active molecular process in eukaryotes. For example, in human it has been shown that at least 4–5% of tandemly duplicated genes are transcribed into single putative fusion transcripts [11, 12]. With alternative splicing and deletion mechanisms, a fusion gene that encodes a chimeric protein can be generated during evolution [5]. This prediction was supported by the high proportion of chimeric proteins identified in several organisms [7, 10, 13], and although it was unknown how often the gene fusion mechanisms were invoked, ~30% of new genes recruited various genomic sequences and formed chimeric gene structures in *Drosophila* [14]. In rice, ~50% of new genes on the short arm of chromosome 3 may have been formed by chimeric mechanisms [15]. These chimeric new genes, which could have been formed by any number of the recombination-based molecular processes that result in gene fusion, inspired a search for the global distribution of gene fusion events across the genomes of a set of closely related species.

The major technical hurdles that have prevented the detection and study of gene fusions in the past have been (1) the lack of transcriptome data derived from multiple and recently diverged species; (2) the inclusion of low expressed transcripts; and (3) the lack of a suitable out-group to infer fusion gene origination processes. The low expression associated with recently evolved genes often makes the identification of new genes difficult, including those created by gene fusion mechanisms [16]. With the advent of high coverage RNA sequencing (RNA-seq), however, it recently became feasible to identify and investigate fusion genes at the transcriptome level in multiple species. The DNA and RNA sequencing of multiple *Oryza* species which have diverged over a recent evolutionary time frame provide an excellent opportunity to study new gene evolution through gene fusion.

Here we report a computational search method, assembled into a pipeline named GriffinDetector (scripts and documentation are available online: http://longlab.uchicago.edu/?q=GriffinDetector), which was designed to identify fusion genes in several recently diverged species. We applied this pipeline to interrogate 8 *Oryza* species/varietal groups and 2 additional out-group species, to detect the presence of fusion genes in four focus genomes *Oryza sativa* L. v.g. *japonica*, *O. sativa* L. v.g. *indica*, *O. barthii*, and *O. glaberrima*. *O. glaberrima* is a cultivated African rice species which originated from *O. barthii* that diverged from each other within 0.40 million years [17–20]. Using these comprehensive genomic resources and GriffinDetector, we detected a surprisingly high number of new gene fusion events that led to a high rate of fused protein formation in the recent history of *Oryza*, suggesting frequent adaptive events in these species are triggered by new fusion genes.

## Results

### Predicting new gene fusions

We developed a set of computational methods to process the genome sequence data of four recently diverged *Oryza* species, *O. sativa* L v.g. *japonica*, *O. sativa* L v.g. *indica*, *O. glaberrima*, and *O. barthii.* Generally, a fusion gene has at least 2 distinct parts originated from 2 independent parental genes. The algorithm used to identify fusion genes needs to resolve the major question of whether 2 different gene parts fuse to become one single gene. In single genome studies, analyses are limited to the identification of fusion genes through comparisons of the candidate gene to its paralogous genes [7] or to different stages of transcriptome data [21]. Without out-group support, it can be hard to distinguish gene fusion events from gene fission events—which occur when a longer homologous parental gene is split into 2 short homologous daughter genes. Benefiting from next-generation sequencing technology, it is possible to detect fusion genes based on multiple genome comparisons [4, 15]. Our pipeline, GriffinDetector, detects fusion genes using protein sequences annotated in several genomes and considers the phylogenetic relationships of homologous gene structures.

**Fig. 1:**
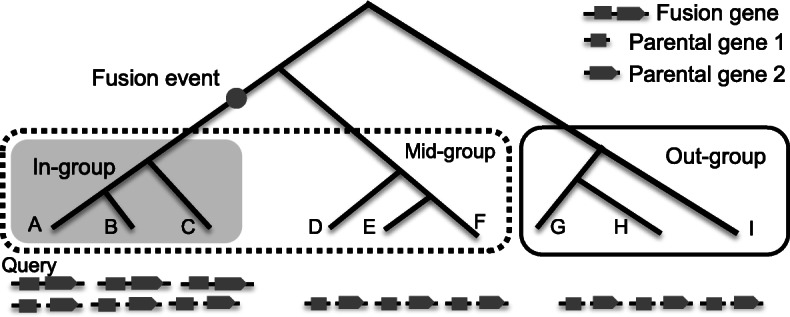
The basic design for detecting fusion genes. The three species in gray box have both long homologous gene hits (fusion gene candidates) and short homologous gene hits (parental genes), while the other six species in the mid-group and out-group only have the parental genes. Thus, we can deduce a fusion event (gray dot) occurred in the lineage leading to the in-group

The basic design of GriffinDetector is to classify BLAST alignment hits of a query gene from a focus species (the species where we want to detect fusion genes) to several species into 2 groups, the long homologous gene group, i.e., potential fusion genes (e.g., two exons structure in Fig. 1 below the in-group), and the short homologous gene group, i.e., potential parent genes (e.g., one exon per gene in Fig. 1). If the query gene (e.g., from focus species A) has at least 2 short homologous gene hits across all species (all groups) while it has the long homologous gene only in the closely related species (species B and C in in-group in Fig. 1), then, most likely the short homologous genes have been integrated into one fusion gene along the lineage (gray dot in Fig. 1). Thus, we assess the query gene as a fusion gene in focus species A.

**Fig. 2:**
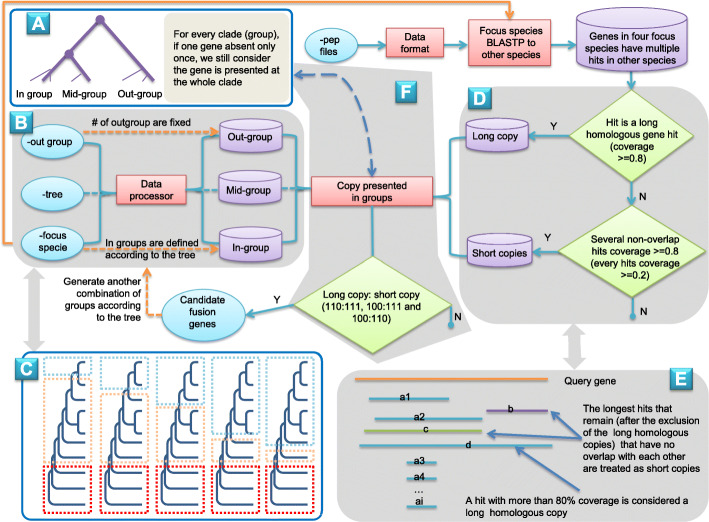
GriffinDetector pipeline flowchart. The light blue ovals indicate the data input or final output data, the green diamonds indicate the thresholds used, the purple cylinders indicate the data generated, and the red boxes indicate data processing. A, F Whether the gene is present in the species /group or not. B, C All the species are categorized into three groups according to their phylogeny: species in the out-group are fixed based on the control file (red dashed box) while the other two groups are dynamic (green and yellow dashed box); the focus species is the initial species in the in-group, then, the closest node or clade will be added into the in-group gradually; the remaining species belong to the mid-group; the process will be stop when only one species remains in the mid-group. D, E When the species belonging to the three groups are clear, BLASTP hits from the focus species query gene are categorized into long hit copies or short hit copies for each species: hits having more than 80% sequence coverage of the query gene are recorded as long copies (line d); the remaining longest hits which have no overlap with each other indicate short copies (lines b and c); other hits (lines a_i_) are ignored

GriffinDetector consists of 3 main parts. First, the species used in analysis (input data including out-group species, phylogeny, and focus species—which is defined in the control file) are categorized into three groups: “in-group,” “mid-group,” and “out-group” (Fig. 2 A–C). Next, all query genes in the focus species are aligned to the other species with BLASTP [22], followed by an alignment assortment into long homologous copies and short homologous copies for each species (Fig. 2 D, E) and group (Fig. 2 A, F). Finally, a query gene is classified as a candidate fusion gene if it meets additional threshold criteria as outlined in more detail in the “Methods” section.

### Abundance of fusion genes in the Oryza genomes

**Fig. 3:**
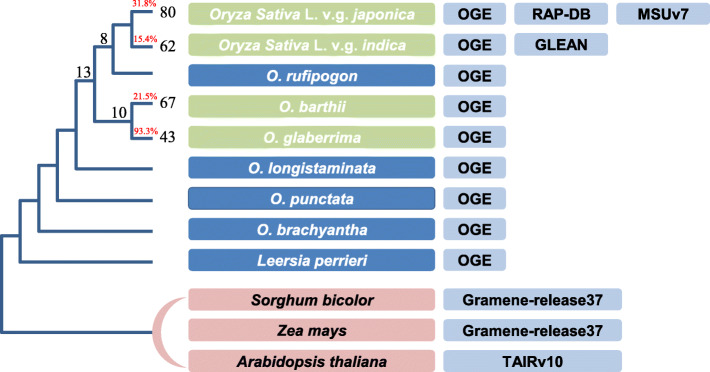
The phylogeny and relative annotation versions used in the analysis. Species in the light gray box are focus species, fusion genes are detected in all these four species for all six combinations (Additional file 2). The dark box represents the most distantly related species

Using the OGE/IOMAP annotation dataset for *O. sativa* v.g. *japonica*, *O. sativa* v.g. indica, and *Arabidopsis thaliana* as the most distant out-group species within the phylogeny (Fig. 3, *Zea mays* and *Sorghum bicolor* are also used as out-groups in the pipeline’s robust analysis, more detail in the “Methods” section), we detected 157, 107, 122, and 100 candidate fusion genes (isoforms) in *japonica*, *indica*, *O. barthii*, and *O. glaberrima*, respectively (Additional file 1), which correspond to 114, 100, 109, and 82 genomic loci, respectively. Since some of the genes among these species may be orthologous to one another, we removed all redundant copies by comparing the candidate fusion genes *via* a BLASTP alignment [22], where hits having more than 80% identity over 100 bp or greater were classified as orthologous gene pairs. This resulted in a final candidate gene fusion list of 310 across the 4 focused genomes (Table 1, more details in Additional file 2, notes that the orthologous genes in different species are considered as one fusion gene), most of which were species-specific genes.

**Table 1:** Fusion gene number detected in four focus species

Shared species	Detail species	Gene numbers	Total gene numbers
Species specific	*Sativa* v.g. *japonica*	80	/
	*Sativa* v.g. *indica*	62	/
	*Glaberrima*	43	/
	*Barthii*	67	/
Shared by four species	*Sativa* v.g. *japonica, sativa* v.g. *indica, glaberrima, barthii*	13	13
Shared by three species*	*Sativa* v.g. *indica, glaberrima, barthii*	6	11
	*Sativa* v.g. *japonica, sativa* v.g. *indica, glaberrima,*	1	
	*Sativa* v.g. *japonica, sativa* v.g. *indica, barthii*	2	
	*Sativa* v.g. *japonica, glaberrima, barthii*	2	
Shared by two species	*Sativa* v.g. *japonica, sativa* v.g. *indica,*	8	34
	*Glaberrima, barthii*	10	
	*Sativa* v.g. *indica, glaberrima*^*#*^	1	
	*Sativa* v.g. *indica, barthii*^*#*^	7	
	*Sativa* v.g. *japonica, glaberrima*^*#*^	6	
	*Sativa* v.g. *japonica, barthii*^*#*^	2	

^*^Gene loss events may be happened

^#^Gene loss or gene gain may be operated

### A high rate of fusion gene origination

Of the 310 candidate fusion genes detected in this study, 58 were shared between 2 and 4 species by simple cross intersections (IDs in Additional file 2). However, when phylogeny was take into consideration, only 31 shared fusion genes could be properly matched onto the species topology tree, suggesting the episodic origination of these fusion genes (Fig. 3). The other 252 fusion genes were categorized as being specie-specific and must have originated very recently in evolutionary time (<1 million years, MY) [17–19]. Thus, the simple estimation of the fusion gene birth rate can be as high as 63 (252/4) per MY per species (Table 1 and Fig. 3). However, for individual species, they showed a great variation of fixation rate (Additional file 3). Analysis of resequencing data from 110 *O. glaberrima* accessions showed a mean fixation frequency of 93.3%*,* with 35 of the 43 *O. glaberrima-*specific fusion genes being 100% fixed (Additional file 3), while 94 *O. barthii* accessions held a mean fixation frequency of 21.5%, with only 2 genes being 100% fixed. On the other hand, by retrieving CDS from vcf files in the 3000 Rice Genomes Project (http://iric.irri.org/resources/3000-genomes-project), *O. sativa v.g. japonica* and *O. sativa v.g. indica* were estimated to have mean fixation frequencies of 31.8% and 15.4%, respectively (Additional file 3). If we take a frequency of 80% as our gene fixation threshold, then we estimate that the fusion gene fixation rate in rice can be as high as 16 (i.e., 64 species-specific fusion genes/4 species) new fusion genes per MY per species, which is nearly 1~2 orders of magnitude greater than that documented for flies and humans [23].

### One third of fusion genes show specific expression patterns, while another one third of fusion genes exhibit similar expression patterns to their parental genes

To determine if some of the candidate fusion genes identified in this study are potentially functional, we analyzed 157 *japonica* fusion gene isoforms for evidence of gene expression and signatures of selection.

Using RNA-seq data from root, leaf, and mixed stage panicle tissues of *O. sativa* v.g. *japonica*, *O. barthii*, *O. glaberrima*, *O. punctata*, *O. brachyantha*, and *L. perrieri* [24], fragments per kilobase of exon per million fragments (FPKM) [25] values were retrieved for the 157 *O. sativa* v.g. *japonica* candidate fusion gene isoforms and their homologous copies (see “Methods” section, Additional file 4). First, we observed that the long homologous copies of 37 fusion gene isoforms (23.6%), identified in all six species, did not produce detectable FPKM signals. However, of the short homologous copies, all but one group of genes (related to fusion gene isoform Osjap05g14570.1) produced detectable FPKM signals (Additional file 4, Additional file 5: Figure S1). Next, among the 69 fusion genes (loci) that had detectable expression signals (FPKM value >0) in *O. sativa* v.g. *japonica*, we noticed 2 fusion genes (Osjap05g04120 and Osjap01g42770) that shared the same expression patterns as their short homologous copies in all three tissues (Additional file 6, Pearson’s *r* range from 0.864 to 0.996, *p* value range from 0.071 to 7.55E−05), 16 fusion genes that shared the same expression patterns as one of their short homologous copies in all three tissues with significant *p* values (Additional file 6, *p*<0.05), as well as an additional 6 with weak *p* values (Additional file 6, 0.05<*p*<0.1). Lastly, we selected the maximum FPKM value from three tissues for each species to represent the expression levels of both the long homologous and short homologous copies. The expression level from the former was significantly less than the latter (Additional file 7, Additional file 5: Figure S2, *p*=2.57 × 10^−8^); this narrowed expression level of new fusion gene is in accord with the phenomenon in de novo genes [26].

Based on FPKM expression data for the 69 fusion gene pairs, 24 gene pairs shared the same/similar expression patterns as their parental genes, 26 gene pairs had novel expression patterns, and the remaining 19 lacked data for their parental copies (Additional file 6). These results indicate that over one third of the candidate fusion genes may have undergone neofunctionalization or subfunctionalization, which has also been suggested by previous studies in human and *Drosophila* [7, 27]. Previous studies support that the new genes in *Drosophila* and human tend to move off their sex chromosomes and tend to be expressed in testis [23, 28–30]. Since the rice tribe does not have sex chromosomes, but does have reproductive tissues (i.e., panicles), we compared FPKM expression data of the fusion genes across three tissues and detected signals for 48, 27, and 52 genes in root, leaf, and panicle tissues, respectively (Additional file 6). There was no significant difference between panicle and root tissue (Fisher’s exact test, *p* = 0.4948), but the number of expressed fusion genes in leaf tissue were significantly lower than in root and panicle (*p* = 1.22 × 10^−4^ and 2.00 × 10^−3^). These expression patterns suggest that fusion genes may play specific roles in root and panicle biology as compared with leaf tissue.

### Fusion genes may drive phenotype evolution

To further investigate whether these expressed fusion genes are associated with adaptive phenotypes, we chose a new gene that represented “neofunctionalization” (i.e., Osjap07g28390 have a novel expression pattern, whose expression level did not correlate with two parental genes), and another that represented “subfunctionalization” (i.e., Osjap09g15430, which has a highly similar expression pattern with that of one of its parental genes), for gene editing experiments using CRISPR/Cas9.

**Fig 4:**
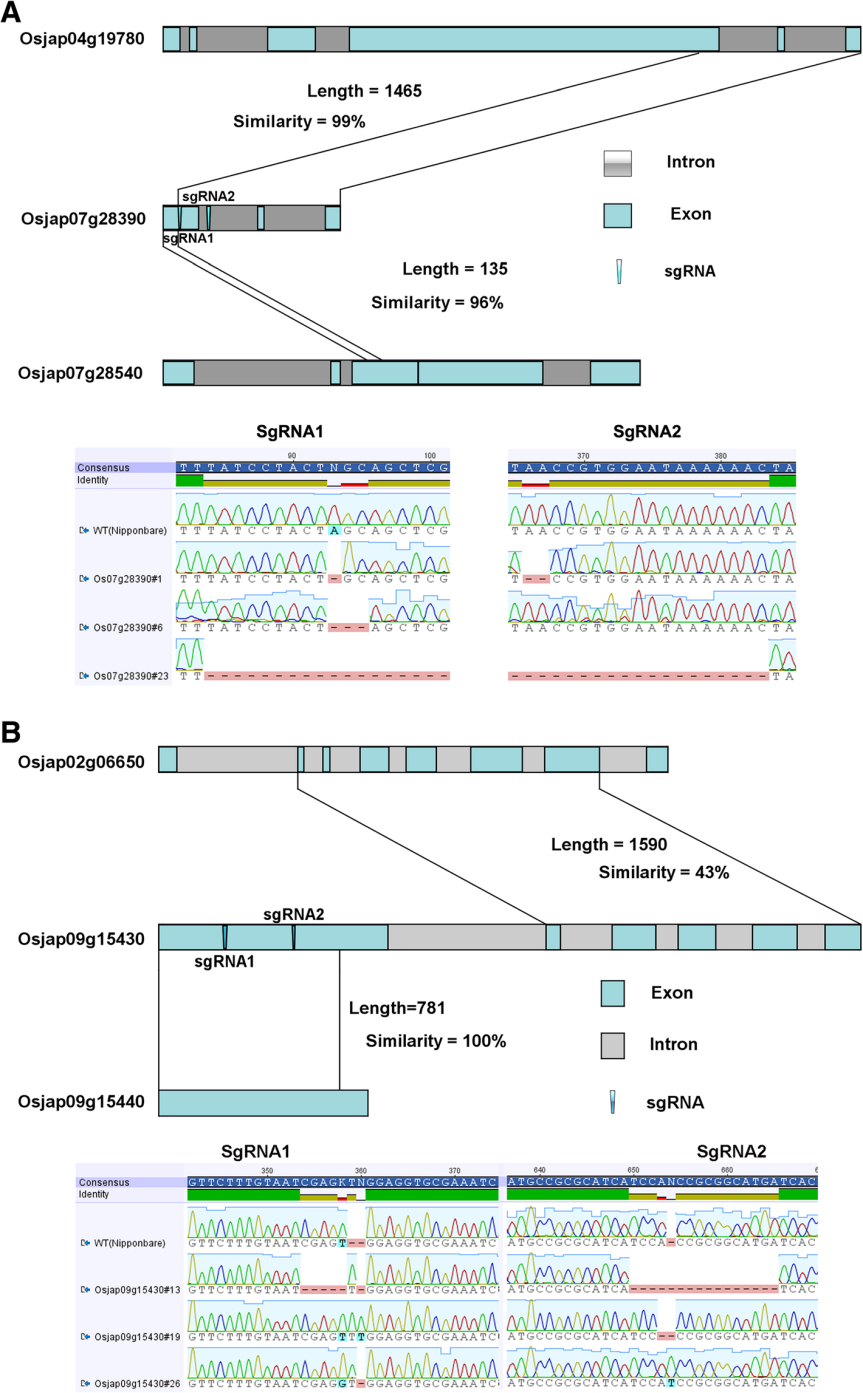
Location of sgRNA on the chimeric diagram and editing informations of three homozygous lines for these two fusion genes. Chimeric structure was drawn based on pairwise blast results, two sgRNAs of each fusion gene were marked by green triangle and their edit types for three selected lines were aligned chromatographically

A total of 30 lines were obtained for the Osjap07g28390 gene editing experiment, of which 14 were shown to be edited, and included 3 homozygous mutants. One Osjap07g28390#1 edited line had a single-nucleotide deletion (A) for the sgRNA1 and a two nucleotide (AA) deletion at sgRNA2; transformant Osjap07g28390#6 contained a three nucleotide (AGC) deletion at the sgRNA1; while transformant Osjap07g28390#23 contained a large fragment deletion between the two sgRNAs (Fig. 4 A).

A total of 29 putative transgenic lines were generated for the Osjap09g15430 fusion gene, of which 13 were shown to be edited. Selected homozygous lines are as follows: Osjap09g15430#13 contained a 5-nucleotide deletion at sgRNA1 and a 15-nucleotide deletion at sgRNA2; Osjap09g15430#19 contained a 2-nucleotide (TT) insertion at sgRNA1, and single-nucleotide (A) deletion at sgRNA2; while Osjap09g15430#26 contained a one-nucleotide (G) insertion at sgRNA1 and one-nucleotide (T) insertion at sgRNA2 (Fig. 4 B).

**Fig. 5:**
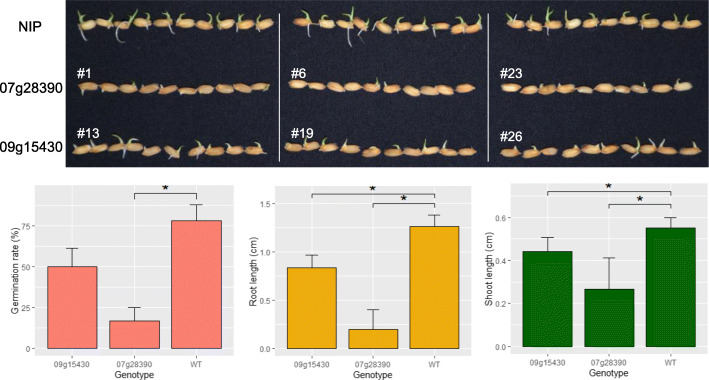
Phenotypic comparison between CRISPR/Cas9 knockout mutants and wild type plants. **A** Morphological characteristics donated by knockout of two fusion genes, the photo were taken at the fifth day after sowing. **B** Germination rate of different genotype. **C** Root length of germinated seedlings. **D** Shoot length of germinated seedlings. All these traits were observed at five days after sowing, with 3 homozygous lines of each fusion gene showed similar phenotype. The same Nipponbare line was used as control. The statistical analysis between transgenic lines and Nipponbare was done by Wilcox test. Asterisks indicate significant at *p*< 0.05

We found that the germination rate and root length of the three Osjap07g28390 lines was much lower that of the same Nipponbare line and that root and shoot length of the three Osjap09g15430 edited lines were shorter (Fig. 5) than the same Nipponbare line grown under identical growing conditions. These results are consistent with the expression preponderance in panicle and root. When we searched these two genes against rice stress database BAR (http://bar.utoronto.ca/efprice/cgi-bin/efpWeb.cgi), they both mainly response to drought stress (Additional file 5: Figure S3). As reported in other species, drought often reduced germination rate, root length, and shoot length [31, 32]; thus, the shorter root and lower germination in CRISPR mutant possibly reflect a result of drought adaptation.

### Duplication of parental gene subsequent with fusion event is the main origination structural pattern

**Fig 6:**
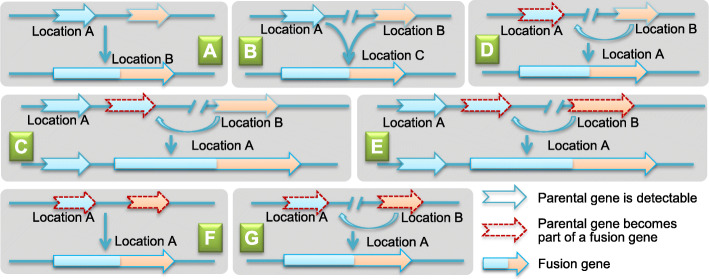
Origination patterns of fusion genes. A–C Parental copies are detectable in focus species (Type I); D, E only one parental copy is detectable (Type II); F, G none of the parental genes are detectable (Type III)

To better understand how gene fusions that are formed in rice, we investigated the structures of the 69 *japonica* expressed fusion genes we identified. First, the 69 expressed fusion genes were categorized into 3 major types based on the parental gene presence and locus in the genome of the focus species [33]. Type I fusion genes have both parental genes that are detectable (Type I) and are formed under the following 3 scenarios where the parental genes are located in the same region of the chromosome, and a segmental duplication generated a novel copy which was then fused together by alternative splicing site mutations or skip (Fig. 6 A); the parental genes are located in different regions of the genome, but duplicate copies are generated, inserted into an adjacent region, and then fused (Fig. 6B); or, one of the parental genes generated a tandemly duplicated copy while the other parental gene (unlinked) generated a duplicate copy that inserted nearby and then fused (Fig. 6C). Type II fusion genes are ones when only one parental gene is detectable. Here, one of the parental genes generates a novel duplicated copy that is inserted adjacent to another parental gene, which has not been duplicated and they fuse together (Fig. 6 D); or one of the parental genes generates a tandemly duplicated copy, and another parental gene, elsewhere in the genome, is moved to an adjacent region and then they fuse together (Fig. 6E). (3) Type III fusion genes are ones where no original parental gene is detectable. Here, one parental gene is transposed to an adjacent region of another parental gene, and then becomes a fusion gene (Fig. 6G), or the parental genes are located in the same region, and an alternative splicing site mutation or skip leads to the fusion of the 2 parental genes (Fig. 6F).

Following the guidelines above, we categorized the 69 expressed fusion genes into 35 Type I, 19 Type II, and 15 Type III (Additional file 8). In Type I, 2 of 35 fusion genes had parental genes in the same region in the focus species (Fig. 6A), 4 were located near one of the parental genes (Fig. 6C), and the other 29 were located in different regions of the genome (Fig. 6B). Of the 19 Type II fusion genes, only one was located in the adjacent region to one parental gene (Fig. 6E) while the others followed the hypotheses outlined in Fig. 6D. Lastly, of the 15 Type III fusion genes, and considering the location of parental genes in other species, 11 of them had the parental homologous copies in the same syntenic regions (Fig. 6G), while parental genes in the remaining 4 cases were located in different chromosomal regions (Fig. 6F).

Our data suggests there are 13 fusion genes that have only one short homologous copy in *L. perrieri* and *A. thaliana* while they have two parental copies in other species (Additional file 8). This result reveals that some of these parental genes are also newly evolved. Since the lack of homologous copies that have more than 20% coverage of the fusion gene in *L. perrieri* and *A. thaliana*, these young parental genes may have originated de novo. These results are consistent with our previous works that showed that the genus *Oryza* has a high rate of de novo gene [26] origination and the neofunctionalization in gene fission and gene fusion is often dependent upon a duplication of parental gene [33, 34].

## Discussion

Here, we presented a new pipeline, GriffinDetector, to scan for candidate fusion genes in focus species utilizing several closely related species, a given phylogeny, and three group species. We then applied GriffinDetector to the OGE/IOMAP rice tribe genome data set, which showed to be on the top of published genome data [24] especially for the analysis of new gene evolution [26]. We detected 310 fusion genes in 4 focus species, *O. sativa* L. v.g. *japonica*, *O. sativa* L. v.g. *indica*, *O. barthii*, and *O. glaberrima* (Additional file 2). The abundance of fusion genes detected in these AA genome species reveals a high birth rate of functional fusion genes, of which around 1/3 share the same expression pattern with at least one parental gene, and another 1/3 developed totally new expression patterns. Further, almost 1/2 of the fusion genes detected were formed first by gene duplication followed by gene fusion. CRISPR/Cas9 knockout mutants of 2 randomly selected fusion genes demonstrated that fusion genes contribute to phenotypes such as root length and seed germination, irrespective of expression novelty, which implies that fusion genes may play important roles in rice genome evolution even if they share redundancy with their parental genes. As Ohno (1970) pointed out, gene duplication serve as the most important mechanism for origination of new gene copies. Compared with other patterns of new gene like orphan gene, fusion gene derived from gene duplication or exon shuffling could make use of existing nucleotide sequences and was estimated to affect a wider range of phenotypic outcomes for the potential greater genetic diversity [13]. Works in humans and *Drosophila* also suggested that ~80% of new genes are formed by DNA-based duplication, 5–10% by de novo duplication, and ~10% by retroposition [4].

As a pipeline, GriffinDetector has several novel attributes compared with previous analytical tools used to detect gene fusions [35–37]. First, GriffinDetector is the first pipeline that is able to integrate data from several genomes to scan for fusion genes automatically. Second, GriffinDetector allows the use of a maximum amount of genomic information to make judgments about whether a query gene is absent from one group or not. This strategy largely reduces the number of false positives that can arise from imperfections of an assembly or annotation. Third, since GriffinDetector works on several closely related genomes, possible biased data present in one genome will not have a significant effect on the final results, unless the bias is in the focus species. Lastly, our results show that GriffinDetector has great potential to become a gateway tool that will inspire innovative projects for those working on integrated analyses of multiple, closely related, genomes to detect and interrogate fusion genes.

**Fig. 7:**
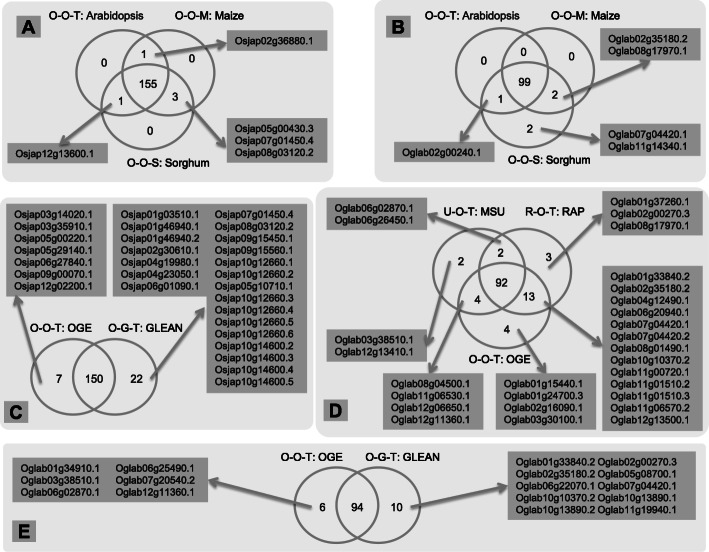
Comparisons of candidate fusion gene isoforms among six combinations. Three-letter vectors denote the various annotations and most distantly related species. Letters in the first position: O represents OGE/IOMAP, U for MSU, R for RAP-DB annotations. In the second position, O stands for OGE/IOMAP, and G for GLEAN; Letters in third position: T stands for TAIR, S stands for *Sorghum bicolor*, and Z stands for *Zea mays*. Venn diagrams were used to display the number of fusion genes that were shared or unique across different species or annotations. **A, B** Most distantly related species shift, **A** *sativa* v.g. *japonica* IDs, **B** *glaberrima* IDs, 155 and 99 candidate fusion gene isoforms are shared by three combinations respectively; **C, E** *sativa* v.g. *indica* annotation shift, **C** *sativa* v.g. *japonica* IDs, **E** *glaberrima* IDs, 150 and 94 candidate fusion gene isoforms are shared by two combinations respectively; **D** *sativa* v.g. *japonica* annotation shift, **D** *glaberrima* IDs (*sativa* v.g. *japonica* IDs are not comparable), 92 candidate fusion gene isoforms are shared by the three combinations

GriffinDetector is an efficient pipeline. Our analyses indicates that changing the out-group species, or annotation of a sister species, will not significantly affect the results, thereby demonstrating the robustness of the detection methodology (see “Methods”). We observed that the vast majority (i.e., 76.7%) of the fusion genes detected by GriffinDetector were shared among different out-group and non-focus annotation combinations (Table 2, Fig. 7, “Methods”). However, we also observed that utilization of alternative annotations of focus genomes had a significant impact on the detection of fusion genes (Table 2). For example, when we focused on the detection of fusion genes in *O. sativa* L. v.g. *japonica* rice, and then switched the OGE/IOMAP-MAKER annotation to the MSU or RAP-DB annotation, the fusion gene number decreased from 157 to 24 or 7, respectively. Such large variations are not unexpected. For example, when Wang et al. [38] and Sakai et al. [39]  scanned the *indica* and *japonica* genome assemblies for retrocopies, they detected 1235 and 150, respectively. One major reason is that the public annotations, like the MSU- and RAP-DB annotations, tend to use very strict parameters to maintain a conservative gene pool, which was shown to be unsuitable for new gene analysis [16]. In our analysis, we chose the OGE/IOMAP annotations for all subsequent analysis, since most of the other species used in the analysis were annotated in a similar manner [24, 26] which used the newest MAKER-P [40, 41] annotation pipeline to generate a reliable and consistent annotation data set.

**Table 2.** Number of candidate fusion gene isoforms

Combinations	O-O-T^*^	O-O-S	O-O-M	U-O-T	R-O-T	O-G-T
*Sativa* v.g. *japonica*	157	159	159	24	7	172
*Sativa* v.g. *indica*	107	109	108	113	113	178
*O. barthii*	122	124	123	134	119	114
*O. glaberrima*	100	104	101	113	110	104

^*^Letters in the first position indicate the annotation version for *japonica*, O represents OGE/IOMAP annotation (generate by the *O*ryza Genome Evolution and International *Oryza* Map Alignment Projects), U stands for MSU, R stands for RAP-DB. Letters in the second position indicate the annotation version for *indica*, O stands for OGE/IOMAP, G stands for GLEAN. Letters in third position indicate the different species, T for *Arabidopsis thaliana* (TAIR annotation), S for *Sorghum bicolor*, and Z for *Zea mays*.

### High rate of fusion gene origination in Oryza

Many groups have demonstrated that fusion genes play an important role in eukaryotic new gene origination which has led to major contributions of adaptive evolutionary novelties [42]. Here, we demonstrated that several species in the *Oryza* genus exhibit high rates of fusion gene origination.

The total number of fusion genes detected by GriffinDetector was 310 among 4 *Oryza* species (Additional file 2) which diverged very recently (<1 MY) [17–19]. Excluding fusion genes that may have originated earlier, and fusion genes that may not be fixed in the population, we estimated that the fixation rate of fusion genes in *Oryza* was as high as 16 fusion gene per MY per species. This rate is much higher than that found in other eukaryotic systems such as *Drosophila*, where it has been estimated to be fixed at around 0.16 fusion genes per MY per species [7]. Among them, *O. glaberrima* showed an especially high fixation frequency (Additional file 3), which is consistent with the fact that *O. glaberrima* underwent a genetic bottleneck effect leading to its low level of genetic diversity [43–46].

**Fig. 8:**
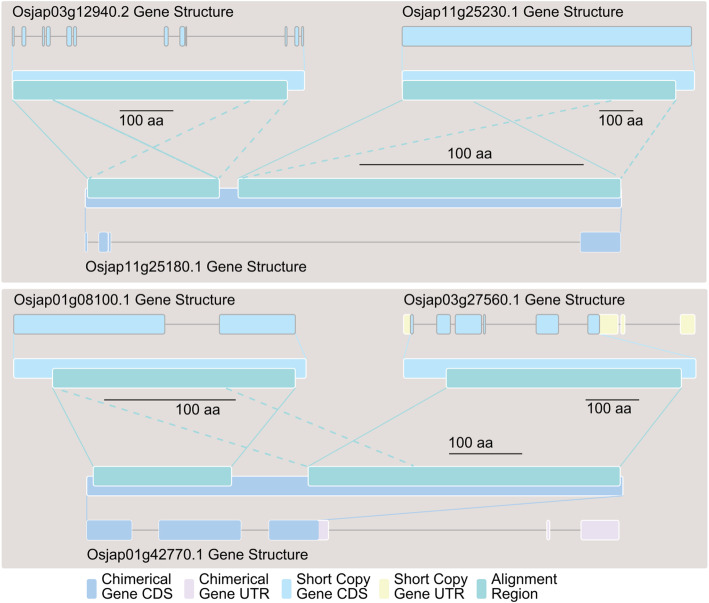
Two retro-fusion gene structures. Osjap0312940 was retroposed to be the second exon of Osjap11g25180; Osjap01g08100 was retroposed to be the first exon of Osjap01g42770, while Osjap03g27560 was retroposed to become the second and third exon. The second intron of Osjap01g42770 may be retained from the parental gene or is newly evolved. Tandem repeats were detected for both gene pairs (dash lines)

Among the 69 fusion genes in *O. sativa* L. v.g. *japonica* that have an expression signal in the tissues tested, only 2 cases were confirmed to have an intron-loss structure (Fig. 8), Osjap01g42770 was shared with *indica*, while Osjap11g25180 was *japonica* specific. Thus, we estimate the retro-fusion gene birth rate in rice to be around 2 per MY (1.77 per MY if weighted, 1 + 1 × 33/43 = 1.77), which is very close to 1.7 (197/(1.5/2/6.5 × 10^3^) = 1.7) per MY estimated in a previous study [38]. This rate is more than 10 times the rate found in human and *Drosophila*, which was estimated to be 0.14 [23, 30] and 0.16 [47] per MY, respectively, and two times the rate in zebrafish, estimated at 1 per MY [48]. It is interesting to note that the estimates of birth rates of new genes seem to increase over time. Our rate (16 fusion genes per MY and 2 retro-fusion genes per MY) is far higher than previous studies [7, 23, 30], but close to the results reported for rice [38] and zebrafish [48]. This phenomenon can be explained in two ways: (1) due to a fast decay rate, many old fusion genes will be eliminated from the genome, while very young fusion genes can still be observed. Both our data and previous studies support this hypothesis. Our data suggests that among the 4 focus species which diverged within 1 MY, at least 11 fusion gene loss events have occurred (Table 1). Previous studies indicate that the estimated half-life of fusion genes was 0.44 MY [7], which means that older fusion genes have a much higher chance of being deleted. The distributions of fusion genes according to *Ks* values (or age) clearly show that more fusion genes are enriched at a young age [7, 48]. Wang et al. [38] estimated the birth rate of retro-fusion genes using young genes which were less than 1 MY (*Ks* ≤ 0.013), with a rate as high as 7 genes per MY, but when all the 197 genes are used (the *Ks* ≤ 1.5, which indicate the oldest age is 115.4 MY), this rate decreases to 1.7 gene per MY. (2) The mechanisms or strategies for genome evolution of plants, primates, fish, and insects may have large differences, so fusion genes might play a more important role in rice and zebrafish compared with their roles in human or *Drosophila*. Our recent work suggests that plants do have large differences in gene structure evolution as compared to human and fly systems [49], which therefore may contribute to the differences we observed in gene fusion birth rates.

### The patterns of fusion gene formation

The progenitor of cereal crops was shown to be an ancient aneuploid that experienced a whole genome duplication ~70 MY ago [18]. It has been suggested that genome content and gene expression quickly change after whole genome duplication events (WGDs) [50]. Since all the fusion genes we detected were very young, most of them being less than 1 MY old, these fusion genes have likely originated on a case-by-case basis and are not related to this large-scale duplication event(s).

In our analysis, we classified the formation of fusion genes into 3 categories: (type I) both parental copies are detectable in the focus species; (type II) only one parental copy is detectable in the focus species; and (type III) none of parental short copies are detectable. Overall, these 3 categories correspond with 3 types of molecular evolution events: (1) sequences with in the fusion gene are copies of parental genes; (2) one part of a fusion gene sequence was from a parental gene duplication, and the other part of the fusion gene was a parental gene, itself; and finally, (3) the fusion gene sequence was a fused unit of 2 parental genes. We identified 35 fusion genes belong to the type I category, 19 to the type II category, and 15 to the type III category. Although many previous studies have pointed out that the rate of duplicated gene loss is lower than expected, and several theories have been proposed to explain these lower rates of gene loss [51], we cannot guarantee that all fusion genes detected in this study are indexed in the correct group, possibly because of visible recent gene loss in the focus species. For example, Additional file 2 and Fig. 6 show that fusion gene loss events have occurred 11 times. However, this obviously does not change our estimate for the high origination rate of new fusion genes, given the high number of young fusion genes that were detected herein.

It is well known that the two primary mechanisms responsible for the majority of gene fusion events in eukaryotes are DNA-level recombination [52] and retroposition [23, 30, 38]. Our *Oryza* fusion gene data did not reveal the presence of a large amount of TEs (23 in total, Additional file 9) like LINEs, LTRs, Pack-Mules, and *Helitrons*, which previous studies have suggested may contribute to fusion gene origination [53–55]. Here, we only detected 2 cases of retro-fusion genes (Fig. 8) out of a total of 69 fusion genes which have transcription evidence. The low retro-fusion gene birth rate observed in our study coincides with previous studies [56] and may be due to the finding that retrogenes favor the recruitment of promoter regions, and not additional protein coding genes/exons, en route to fusion gene formation [4]. We note here that GriffinDetector was designed specifically to focus on protein coding gene fusions.

Williford and Betrán recently classified the formation of a fusion gene into two phases [57]. First, sequences from two sources were placed in adjacent regions, and second, sequence insertion, deletion, mutation, or recruitment of noncoding regions occurs, which provides suitable splicing sites for the two sequence sources to become a fusion gene. This simple but important statement may help us to explain the data we observed, such as the case of Osjap04g02830 (Additional file 8), where the parental genes in *O. sativa* L. v.g. *japonica* were located near to one another, while in the other species they were not, which means the parental gene may have been re-located recently and, in a very short time, allowed for a new fusion gene to form.

In an analysis of the formation of fusion genes in *Drosophila*, fusion genes were classified into 3 major types: retro-fusion genes, ectopic recombination fusion genes, and tandem duplicated fusion genes [13]. These types are supported by previous studies: Rogers and colleagues detected 14 fusion genes, 8 of them related to the tandem duplication mechanisms; Shuang Yang and Arguello et al. detected 17 duplicated genes which were generated through ectopic recombination in a 12 MY time frame, most of which are functional and evolved diverse functions and chimerical structural [58]. In contrast, our data showed very little evidence of tandem duplication fusion genes (Additional file 8), which raises interesting new questions of origination to explore in future studies.

### Phenotypic effects of new genes and biological diversity

Phenotypic evolution in morphology and development leads to biological diversity. Whether or not the gene evolution participates in the evolution of genetic basis underlying phenotypic evolution is a new direction to explore, besides the better known role of regulatory systems for genes [3]. Huang et al. [59] show that a species-specific gene in *Arabidopsis thaliana* evolved important phenotypic effects in development and morphology. In this study, we used the CRISPR knockout technique to create mutant lines for the fusion genes Osjap07g28390 and Osjap09g15430. We found that both genes evolved significant phenotypic effects in three phenotypic traits, germination rates, root length, and shoot length. Regardless of possible neofunctionalization (Osjap07g28390) or subfunctionalization (Osjap09g15430) in molecular functions, they both are involved in drought resistance, an important trait adaptive to frequent environmental changes where rice often encountered. These data reveal that the genetic basis of the morphology and organs in Oryza evolved rapidly by acquiring new genes recently.

## Conclusions

We detect new fusion genes that may drive phenotype evolution in *Oryza*. This study provides novel insights into the genome evolution of *Oryza*.

## Methods

### Development of GriffinDetector

GriffinDetector is a pipeline script written in Perl and requires two assistant programs (makeblastdb and blastp) from the BLAST+ package [22]. GriffinDetector is available at http://longlab.uchicago.edu/?q=GriffinDetector with detailed documentation, which also can be download at GitHub repository (https://github.com/zhangcj2022/GriffinDetector).

The GriffinDetector pipeline uses a control file to set the main parameters including information regarding the necessary input data (you can get the example in package). It divides all of the species listed in the control file into 3 groups: in-group, mid-group, and out-group. The out-group is fixed (e.g., Fig. 2C, the three species in red dashed box), while the in-group and mid-group are dynamic (e.g., Fig. 2C, the species in green and yellow dashed boxes). In Fig. 2C, from left to the right, the number of species belonging to the in-group gradually increases, while the number of species in mid-group decreases. In the initial round, GriffinDetector will take one species from the focus species listed in the control file, as the initial in-group (Fig. 2C 1^st^) and in the following rounds, GriffinDetector will add one species (Fig. 2C 2^nd^, 3^th^, 5^th^) or one clade (Fig. 2C 4^th^) which is the nearest one to the current in-group clade into the in-group to generate a new in-group. This dynamic progress will not stop until only one species remains in the mid-group. Thus, GriffinDetector sort alternative combinations of species with associated with each focus species.

Based on the in-group combinations, protein sequences of each focus species are aligned using BLASTP [22] to protein sequences of other species (including itself). GriffinDetector will inspect the BLASTP results and categorize the hits into short homologous copies and long homologous copies (Fig. 2D, E). Hits that have more than 80% coverage of the query gene will be assigned as “long homologous copies” immediately (Fig. 2E line d), then GriffinDetector will compare the remaining hits to each other and filter the shorter hits (Fig. 2E line a_i_) which overlap with other longer hits (Fig. 2E line c). Finally, hits that overlap more than 20% with the query gene (Fig. 2E line b & c) will be assigned as short homologous copies. If the final remaining short homologous copies in one species are more than two (including two) and the total coverage of the query gene is larger than 80%, GriffinDetector identifies that species as having the short copies. Thus, GriffinDetector is able to determine whether each species has the long homologous copy or not, as well as short copies.

If one homologous copy is present in one group, it will be denoted as 1; otherwise, it will be denoted as 0. GriffinDetector defines the homologous copy as present in one group if the homologous copy is present in all species of the group, but if the group has two or more species, the homologous copy is allowed to be absent in one species at most (Fig. 2A). For example, if the query gene has a long homologous copy present in the in-group and mid-group, but is absent from the out-group, we use a vector to denote it as L-110. If the short homologous copies are present in all three groups, we record it as S-111 (e.g., Fig. 1). Generally, we have 2^3^ combinations for each long homologous copy and 2^3^ combinations for each short homologous copy, where the total number of possible combinations is 64. GriffinDetector only recognizes three combinations as fusion genes: L-110 and S-111, L-100 and S-111, L-100 and S-110.

### Data and resources

In our analysis, we used 12 species and 15 annotation versions. All related data, including protein sequences and GFF files of Oryza species, can be accessed through OGE/IOMAP website (http://oge.gramene.org/, more detail in http://bioinfor.kib.ac.cn/?q=node/10), which are required by the pipeline. The fragments per kilobase of transcript per million mapped reads (FPKM) values were downloaded from the OGE/IOMAP website, as well as the TE annotation of *O. japonica*.

RNA-seq data from leaf root and mixed stage panicle tissues were as described in Stein et al. [24]. The RNA-seq reads were aligned to the respected genomes using Tophat software [60]. The alignment files were inputted into Cufflink software [61] to generate the FPKM value.

The OGE/IOMAP annotation was generated using the MAKER annotation pipeline v2.2.8a [40, 41] as describe in Stein et al. [24]. The de novo transcript assembly, reference guide transcript assembly, the cDNA, and protein annotations from *O. sativa* L v.g. *japonica*, *O. glaberrima*, *Brachypodium distachyon* [62], and *Oryza* cDNA database (NCBI taxonomy ID 4527) were used as evidence for annotation.

### Efficiency of GriffinDetector

Since GriffinDetector is the first automated pipeline designed to detect fusion genes across multiple genomes, it is important to determine its efficiency in detecting such genes. Here, we applied the GriffinDetector to the rice tribe and its closely related out-group species to estimate its robustness. First, we set *O. sativa* L. v.g. *japonica*, *O. sativa* L. v.g. *indica*, *O. barthii*, and *O. glaberrima* as focus species respectively; *O. brachyantha*, *Leersia perrieri*, and *Arabidopsis thaliana* as out group species; and *O. rufipogon*, *O. longistaminata*, and *O. punctate* as other species, to search for fusion genes in the 4 focus species respectively based on their phylogeny (Fig. 3). In addition, we utilized different versions of annotations for the *japonica* and *indica* genome, and adjusted the most distantly related out-group from *A. thaliana* to *Sorghum bicolor* and *Zea mays* to estimate the influences of annotation and out-group distance on gene fusion detection, respectively. For the other 7 species, we only used the OGE/IOMAP MAKER annotations [24]. We use three-letter vectors to denote the 6 different combinations of annotations and out-groups utilized for these analyses: O-O-T, O-O-S, O-O-M, U-O-T, R-O-T, O-G-T (Table 2). The letter in the first position indicates the annotation version for *japonica*: O = OGE/IOMAP, U = MSU [63, 64], and R = RAP-DB [64, 65]. The second letter position indicates the annotation version for indica: O = OGE/IOMAP and G = GLEAN [66]. The third letter position indicates the most distantly related out-group species: T = *A. thaliana* TAIR annotation [67], S = *S. bicolor* [68], and Z = *Z. mays* [69].

The fusion gene data sets identified using these 6 combinations were compared to each other (Fig. 7), and we reached the following conclusions: (1) changing the most distantly related out-group species did not significantly change the results (i.e., >95% of the fusion genes detected were shared); (2) changing the annotation of which is close to focus species due to the phylogeny will slightly affect the final results (i.e., >76.7% of the fusion genes were shared); and (3) changing the annotation version of the focus species will greatly affect the results (i.e., the number of fusion genes detected decreased from 157 to 7). In Table 2, columns O-O-T, O-O-S, and O-O-M, which display the results from the analyses in which the most distantly related species was changed to either *A. thaliana*, *S. bicolor*, or *Z. mays*, the final number of candidate fusion gene isoforms barely changed in *japonica*, *indica*, *O. barthii*, and *O. glaberrima*. A more detailed comparison between candidate fusion gene isoforms of *japonica* (Fig. 7A) and *O. glaberrima* (Fig. 7B) indicates that most (>95%) of the detected fusion genes are shared among these three combinations, which means changing the most distantly related out-group species hardly has any effect on the fusion genes identified in each focus species. The O-O-T, U-O-T, and R-O-T columns, which differed in the *japonica* annotation used, showed a slight change in the number of fusion gene candidates detected for *indica* (*N* = 6), *O. barthii* (*N* = 15), and *O. glaberrima* (*N* = 13). Similar results were seen by varying the *indica* genome annotations (i.e., columns O-O-T and O-G-T), where the number of fusion gene candidates in *japonica*, *O. barthii*, and *O. glaberrima* changed by 15, 8, and 4. A more detailed comparison between candidate fusion gene isoforms of *japonica* (Fig. 7C) and *O. glaberrima* (Fig. 7D, E) indicate that over three fourths (>76.7%) of the results are shared among these combinations; this rate (>76.7%) is lower than the previous one (>95%) and indicates that changing closely related species (e.g., while treating *japonica* as focus species which means we focus on detecting fusion genes in *japonica*, but changing the *indica* annotation from Version A to B, the final results of detected fusion genes will be different) annotations will somewhat affect the final results. On the other hand, when the *japonica* annotation changed from OGE/IOMAP to MSU to RAP-DB, the candidate fusion gene isoforms in *japonica* decreased from 157 to 24 to 7. Further, when the *indica* annotation changed from OGE/IOMAP to GLEAN, the candidate fusion gene isoforms in *indica* increased from 107 to 178. Both results show that the annotation used plays an important role in fusion gene detection.

Since the annotations of most species used were generated by OGE/ IOMAP (Fig. 3), we decide to use the O-O-T combination for all subsequent analyses which yield representative results as described above.

### The origination rate and fixation frequency of fusion genes

Origination of Oryza species were based on species-specific fusion gene. For the calculation of fixation frequency on species-specific fusion genes, we collected *O. glaberrima* and *O. barthii* population SRA data from Meyer et al. [20] and Wang et al. [45], and the SNPs were called by Genome Analysis Toolkit (GATK 4.1.9.0) [70]. The variation of coding sequence was retrieved by vcf2fasta (https://github.com/santiagosnchez/vcf2fasta). On the other hand, we directly download vcf files for 100 *O. japonica* individuals and 101 *O. indica* individuals from Rice SNP-seek database (https://snpseek.irri.org/). Based on the re-annotated gff of species-specific fusion genes in four focus species (Additional file 10), we extracted coding sequence (CDS) of each fusion gene.

We mapped the SNPs on the reference and checked the exact sites in the CDS region, if the SNPs changed the frame of the CDS or it become a stop codon, then we defined the fusion gene was not fixed in the individual. Thus, we counted the total individual numbers of fusion genes not fixed among each species and defined the fixation frequency as the percentage of fixed individual to total individual number in each species. Finally, we define the fixation rate as the total fixed fusion gene number against species number.

### RNA-seq data analysis

RNA-seq data derived from three tissues (i.e., leaf, root, mixed state panicles) from 6 species: *japonica, O. barthii*, *O. glaberrima*, *O. punctata*, *O. brachyantha*, and *L. perrieri*, were generated by the OGE/IOMAP project [24]. Using the same reference genome coordinate, if the region for an FPKM value had more than 50% sequence overlap to a fusion gene or homologous short copies, the FPKM value was used as the expression signal measurement for the relative genes. The FPKM values for the 157 fusion genes (isoforms) in *japonica*, as well as for all the homologous short copies, are presented in Additional file 4. The reference gene LOC_Os08g03290, which was recommended by previous work [71], was also used as a control to present the expression levels. Among these, 69 fusion genes (loci) have expression signals and were used in the following analysis.

Using the natural logarithm of the FPKM values and two R libraries, limma and ggplot2, we generated the heat maps (Additional file 5: Figure S4) to visualize the gene expression data employed in the analysis. This reference gene was the only gene studied previously in two separate analyses [71, 72] and has expression signals in all 3 tissues in *japonica*. For cases where there was more than one homologous copy in one species, we took the highest FPKM value as the representative for the expression level of the short homologous copies.

### Knockout mutant generated by CRISPR/Cas9

Vector system of CRISPR/Cas9 was kindly obtained from Professor yaoguang Liu’s lab. Two candidate sgRNA were designed for each selected fusion gene. The two sgRNA were tandemly fused by paired adapters Uctcg-B1’&gRctga-B2 and Uctga-B2’&gRcggt-BL, and taken effect under the control of rice promoter OsU3 and OsU6a, respectively. All these constructs were introduced into *E. coli* and confirmed by sequencing. Positive single clone of each fusion gene construct were rejuvenated and used for plasmid extraction. Qualified plasmids were introduced into *Agrobacterium tumefaciens* EHA105 with electroporation method, and then transformed into *Japonica* rice Nipponbare for genome editing.

For the Agrobacterium-mediated transformation in *Japonica* rice variety Nipponbare efficiently, tissue culture-based method was adopted. Embryonic callus cells induced from the mature seeds scutella serve as starting material (Additional file 5: Figure S5A), then high potential ones were inoculated onto subculture medium for callus propagation for 20 days (Additional file 5: Figure S5B). After shaking one single colony Agrobacterium harboring CRISPR/Cas9 vector in suspension medium with antibiotics, glucose, and acetosyringone, target callus source co-cultivated with the fresh activated agrobacterium for transformation and then dried callus particles were transferred to co-culture medium for another 3 days (Additional file 5: Figure S5C). The transformed callus was rinsed with ddH_2_O and subjected to resistance screen on selective culture medium containing carbenicillin and hygromycin B (Additional file 5: Figure S5D). The potential positive transformants were further selected for proliferation on differentiation medium (Additional file 5: Figure S5E). Finally, the regenerated seedlings were processed for rooting before transplantation (Additional file 5: Figure S5F).

The mutants potentially edited by Cas9 protein were first screened by hygromycin gene-specific primer. Editing information of T0 transformants were obtained by PCR sequencing with gene-specific primers flanking sgRNA, and the homozygous lines were obtained by analyzing each chromatograms against reference sequence in DSDecodeM (http://skl.scau.edu.cn/dsdecode/). Seeds of homozygous transformants were propagated to T2 generation for phenotype observation.

## Supplementary Information

**Additional file 1.** Chimeric gene output of four focus species.

**Additional file 2.** Candidate fusion gene presented in four focus species.

**Additional file 3.** Fixation frequency of species-specific fusion gene included in four focus species.

**Additional file 4.** FPKM of fusion gene and short copy homologs in *O. sativa* v.g. *japonica*, *O. barthii*, *O. glaberrima*, *O. punctate, O. branchyantha* and *Leersia*.

**Additional file 5: Supplemental figures.** **Figure S1.** Expression profiles of fusion genes and their parent genes in *O. sativa* v.g. *japonica* and other sibling species like *O. barthii*, *O. glaberrima*, *O. punctata*, *O. brchyantha* and *O. Leersia*; **Figure S2.** Statistics of expression levels between long homolog and short homolog; **Figure S3.** Expression heat map of two selected fusion genes in response to abiotic stress; **Figure S4.** Spatial expression pattern of 69 expressed fusion genes; **Figure S5.** Generation of transgenic rice plants. (A) Embryogenic callus derived from the scutella of mature seeds. (B) Subculture elite callus for proliferation. (C) Inoculation of callus with *Agrobacterium* carrying CRISPR/Cas9 vector by co-culture. (D) Screen hygromycin-resistant callus by transferring to selective medium. (E) Regeneration of hygromycin-resistant callus on MS medium for 1 month. (F) Rooting and growth of transgenic rice plants.

**Additional file 6.** Correlation of *O. japonica* fusion gene and its short copies FPKM value in different tissues.

**Additional file 7.** Correlation between the maximum FPKM value of long-homologous copies and the short-homologous copies in four *Oryza* species.

**Additional file 8.** The generation patterns of fusion genes.

**Additional file 9.** Transposable element retrieved from *O. sativa* v.g. *japonica* fusion genes*.*

**Additional file 10.** Gene structure re-annotation of species-specific fusion gene in four focus species.

**Additional file 11.** Review history.

## Data Availability

All the data feed into pipeline was available at website http://bioinfor.kib.ac.cn/?q=node/10. The raw RNA-seq data are available in NCBI under the accession number PRJNA13141, PRJNA13765, PRJNA13770, PRJNA163065, PRJNA30379, and PRJNA70533. The generated vcf files for *O. barthii* and *O. glaberrima* population data were deposited at https://data.cyverse.org/dav-anon/iplant/home/zhouyanli11/ricedata. Other datasets generated during the current study are included in this article and its supplementary information files. Additional information and resources in the manuscript are available from the corresponding authors on reasonable request. The codes of GrrifinDetector are available under a GPL-3.0 License on GitHub https://github.com/zhangcj2022/GriffinDetector.git [73] and Zenodo [74].
