# Supplementary material for: Gene fusion as an important mechanism to generate new genes in the genus Oryza
Source: Genome Biol. 2022 Jun 15;23:130. doi: 10.1186/s13059-022-02696-w (PMC9199173; doi:10.1186/s13059-022-02696-w)

Figure S1 Expression profiles of fusion genes and their parent genes in O. sativa v.g. japonica and other sibling species like O. barthii, O. glaberrima, O. punctata, O. brchyantha and O. Leersia

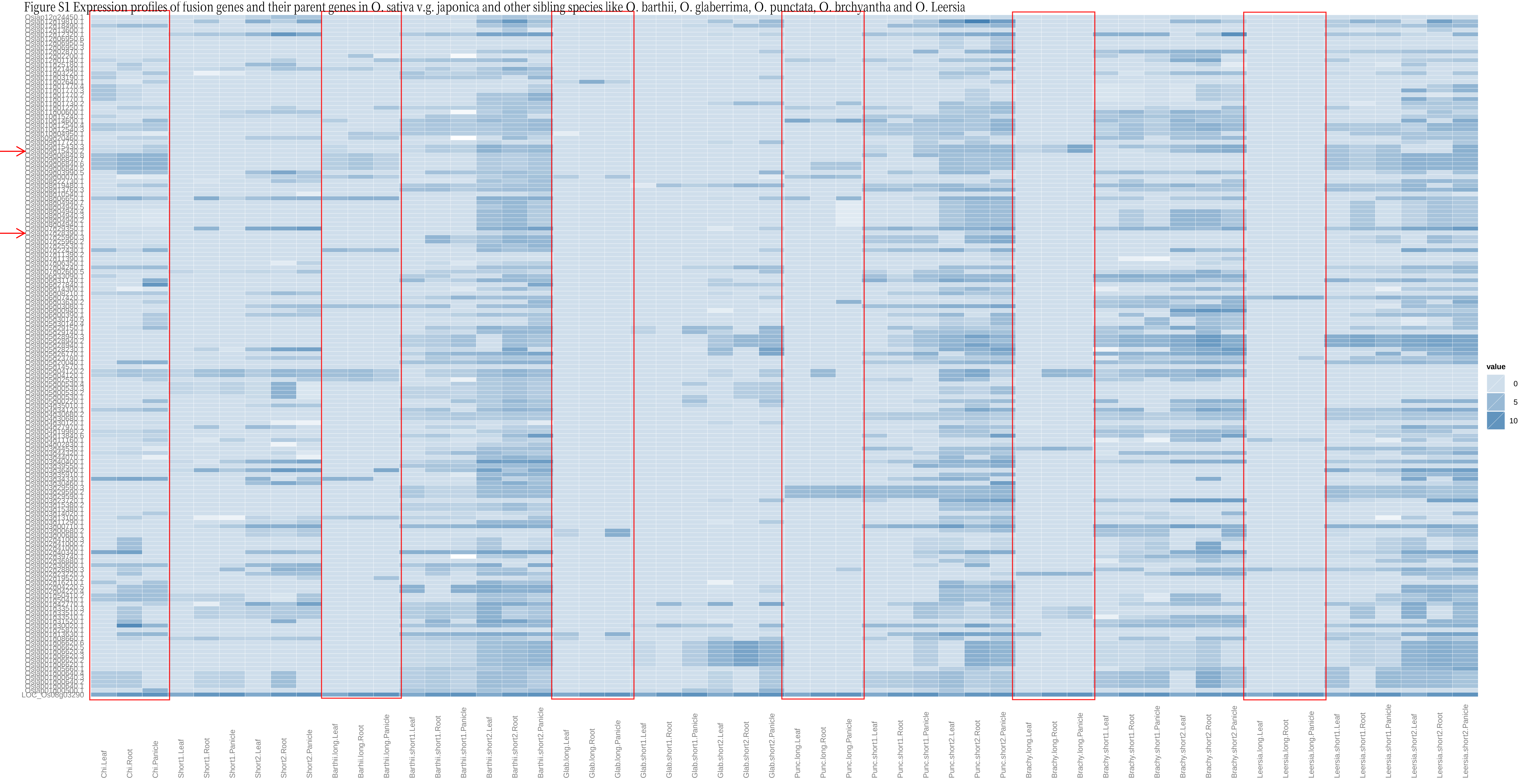

Figure S2 Statistics of expression levels between long homolog and short homolog.

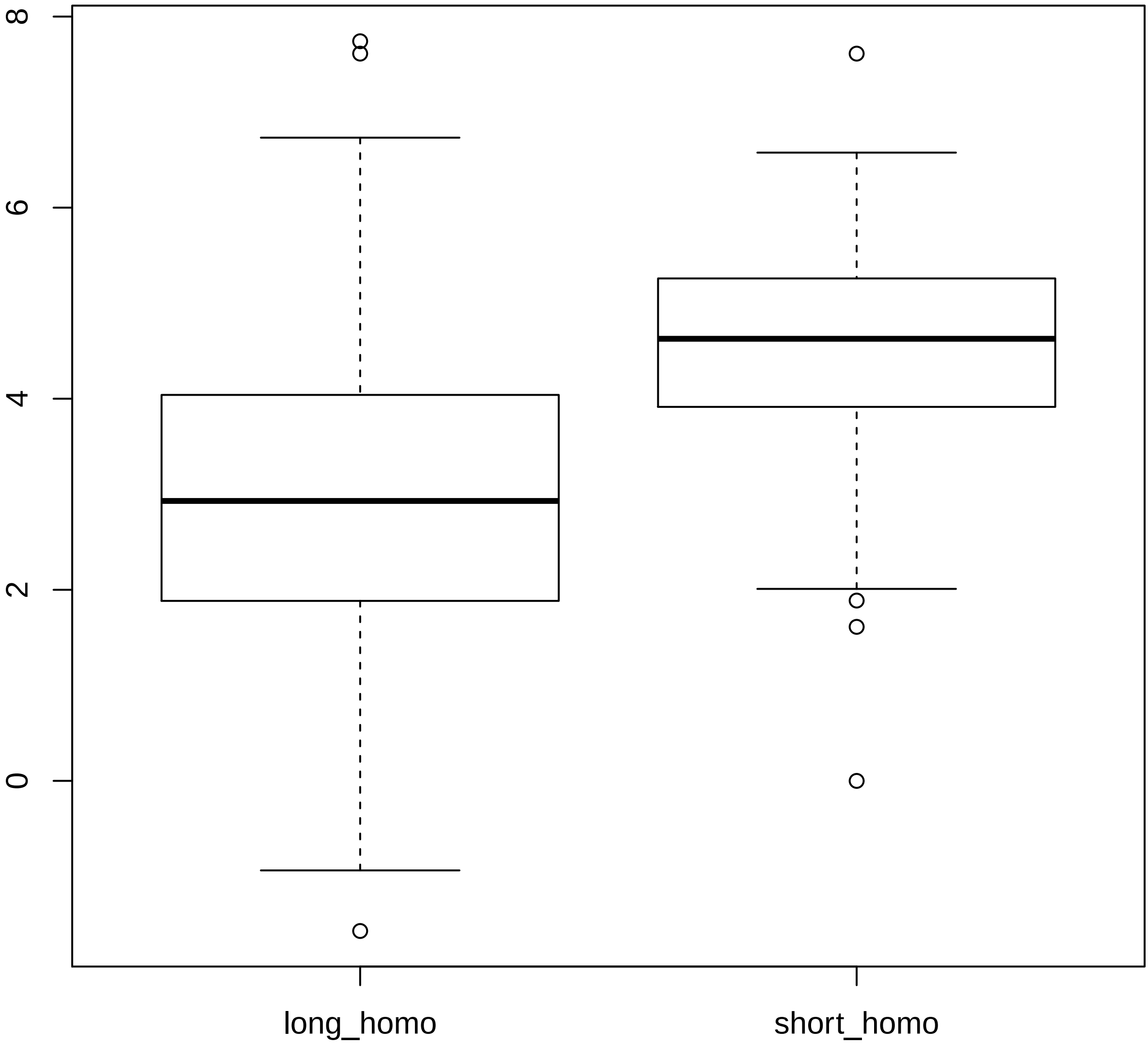

Figure S3 Expression heat map of two selected fusion genes.

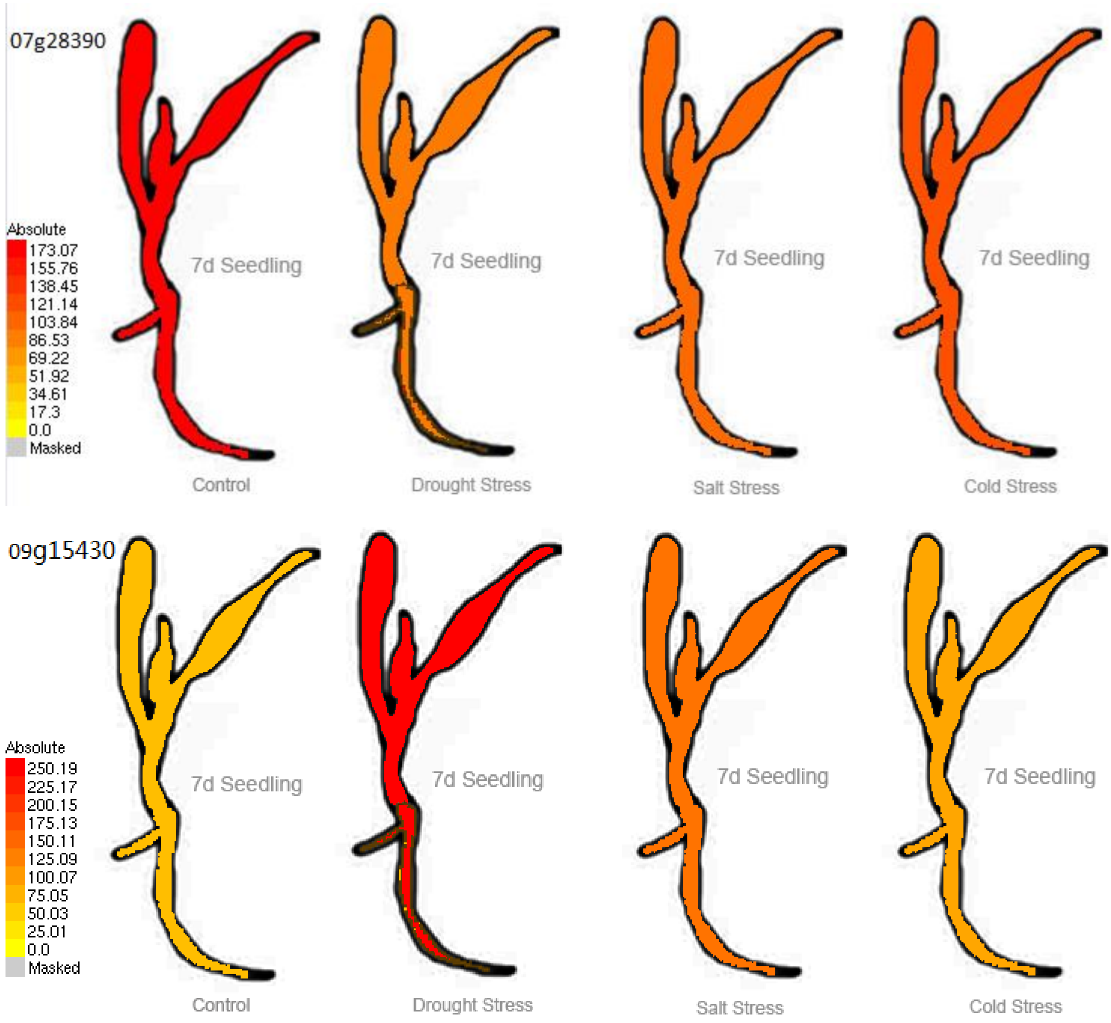

Figure S4 Spatial expression pattern of 69 expressed fusion genes.

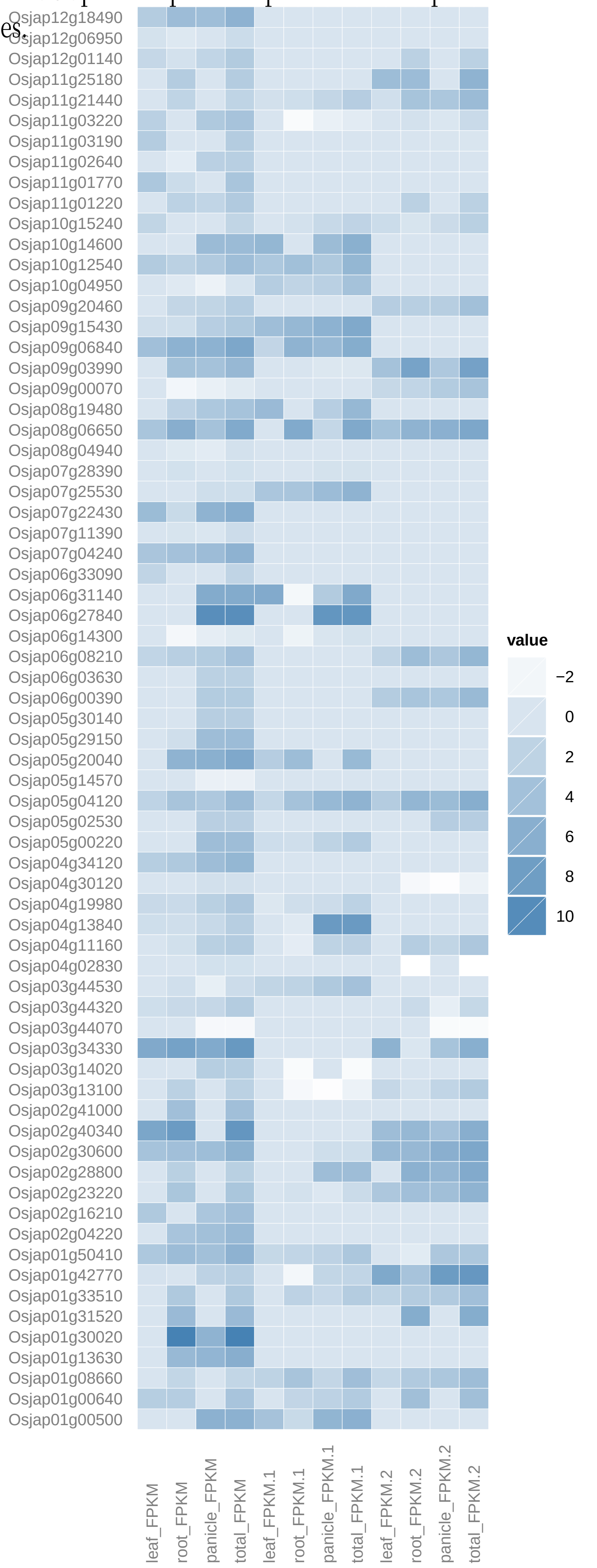

Figure S5 Generation of transgenic rice plants. (A) Embryogenic callus derived from the scutella of mature seeds. (B) Subculture elite callus for proliferation. (C) Inoculation of callus with Agrobacterium carrying CRISPR/Cas9 vector by co-culture. (D) Screen hygromycin-resistant callus by transferring to selective medium. (E) Regeneration of hygromycin-resistant callus on MS medium for 1 month. (F) Rooting and growth of transgenic rice plants.

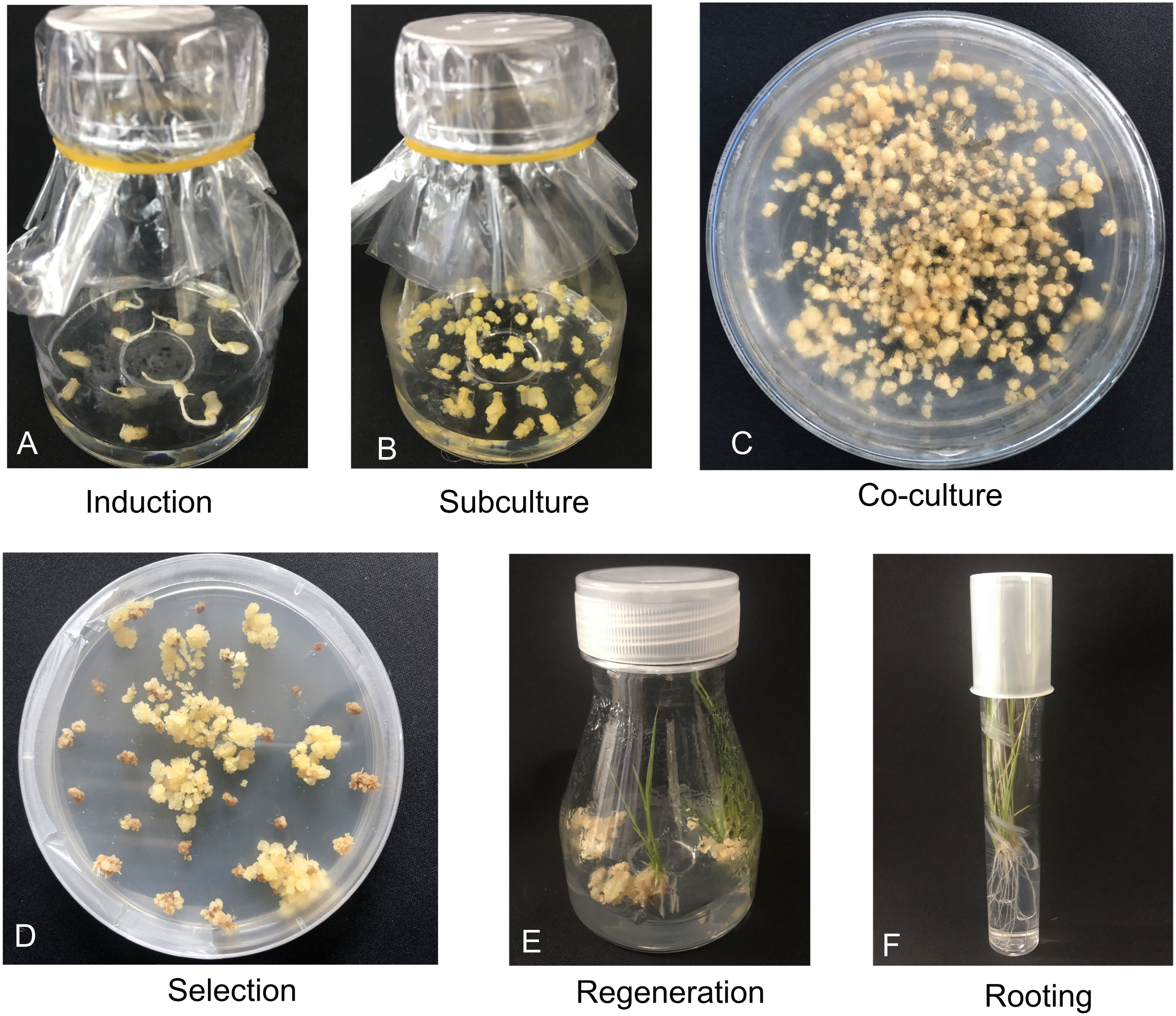

Supplement: Supplementary file 5 — Additional file 5: Supplemental figures. Figure S1. Expression profiles of fusion genes and their parent genes in O. sativa v.g. japonica and other sibling species like O. barthii, O. glaberrima, O. punctata, O. brchyantha and O. Leersia; Figure S2. Statistics of expression levels between long homolog and short homolog; Figure S3. Expression heat map of two selected fusion genes in response to abiotic stress; Figure S4. Spatial expression pattern of 69 expressed fusion genes; Figure S5. Generation of transgenic rice plants. (A) Embryogenic callus derived from the scutella of mature seeds. (B) Subculture elite callus for proliferation. (C) Inoculation of callus with Agrobacterium carrying CRISPR/Cas9 vector by co-culture. (D) Screen hygromycin-resistant callus by transferring to selective medium. (E) Regeneration of hygromycin-resistant callus on MS medium for 1 month. (F) Rooting and growth of transgenic rice plants. [file 13059_2022_2696_MOESM5_ESM.pdf]
