# Supplementary material for: Gene fusion as an important mechanism to generate new genes in the genus Oryza
Source: Genome Biol. 2022 Jun 15;23:130. doi: 10.1186/s13059-022-02696-w (PMC9199173; doi:10.1186/s13059-022-02696-w)
Supplement: Supplementary file 1 — Additional file 1. Chimeric gene output of four focus species. [file 13059_2022_2696_MOESM1_ESM.pdf]

Supplemental data 1

((((((((jap,indica),rufi),(glab,barthii)),longi),punctata),brachy),leersia),tair);

chimeric gene id	INGROUP	MIDGROUP						OUTGROUP		
	jap	indica	rufi	glab	barthii	longi	punctata	brachy	leersia	tair
Osjap01g00640.1	1<br>Osjap01g00640.1 0.001 1 0.999 0.001 1<br>Osjap01g00640.2 0.001 1 0.999 0.001 1<br>Osjap01g00640.3 0.001 1 0.999 0.001 1<br>Osjap01g00640.4 0.001 1 0.999 0.001 1	1<br>Osind01g00770.1 0.105 1 0.895 0.022 1<br>Osind01g00770.2 0.105 1 0.895 0.027 1	0	0	0	0	0	0	0	0
	1<br>Osjap04g25390.2 0.57 1 0.43 0.019 1<br>Osjap05g00080.1 0.001 0.451 0.45 0.007 1	1<br>Osind04g24430.1 0.57 1 0.43 0.019 1<br>Osind05g00060.1 0.001 0.451 0.45 0.007 1	1<br>Orufi01g00590.1 0.565 1 0.435 0.001 1<br>Orufi05g00070.1 0.001 0.451 0.45 0.007 1	1<br>Oglab01g00510.1 0.565 1 0.435 0.001 1<br>Oglab05g00040.1 0.001 0.451 0.45 0.007 1	1<br>Obart01g00590.1 0.565 1 0.435 0.001 1<br>Obart05g00050.1 0.001 0.451 0.45 0.007 1	1<br>Olong_015014-R1 0.49 1 0.51 0.055 1<br>Olong_015017-R1 0.001 0.451 0.45 0.001 1	1<br>Opunc01g00510.1 0.001 0.451 0.45 0.078 1<br>Opunc09g01380.1 0.554 1 0.446 0.03 1	1<br>Obrac05g00060.2 0.001 0.451 0.45 0.008 1<br>Obrac09g00870.1 0.564 1 0.436 0.052 1	1<br>Lperr01g00510.1 0.001 0.451 0.45 0.007 1<br>Lperr09g00030.1 0.564 1 0.436 0.031 1	1<br>AT1G20380.1 0.565 1 0.435 0.001 1<br>AT5G08440.2 0.002 0.45 0.448 0.012 0.997
Osjap01g00640.2	1<br>Osjap01g00640.1 0.001 1 0.999 0.001 1<br>Osjap01g00640.2 0.001 1 0.999 0.001 1<br>Osjap01g00640.3 0.001 1 0.999 0.001 1<br>Osjap01g00640.4 0.001 1 0.999 0.001 1	1<br>Osind01g00770.1 0.107 1 0.893 0.022 1<br>Osind01g00770.2 0.107 1 0.893 0.027 1	0	0	0	0	0	0	0	0
	1<br>Osjap04g25390.2 0.565 1 0.435 0.019 1<br>Osjap05g00080.1 0.001 0.457 0.456 0.007 1	1<br>Osind04g24430.1 0.565 1 0.435 0.019 1<br>Osind05g00060.1 0.001 0.457 0.456 0.007 1	1<br>Orufi01g00590.1 0.558 1 0.442 0.001 1<br>Orufi05g00070.1 0.001 0.457 0.456 0.007 1	1<br>Oglab01g00510.1 0.558 1 0.442 0.001 1<br>Oglab05g00040.1 0.001 0.457 0.456 0.007 1	1<br>Obart01g00590.1 0.558 1 0.442 0.001 1<br>Obart05g00050.1 0.001 0.457 0.456 0.007 1	1<br>Olong_015014-R1 0.493 1 0.507 0.07 1<br>Olong_015017-R1 0.001 0.457 0.456 0.001 1	1<br>Opunc01g00510.1 0.001 0.457 0.456 0.078 1<br>Opunc09g01380.1 0.548 1 0.452 0.03 1	1<br>Obrac05g00060.2 0.001 0.457 0.456 0.008 1<br>Obrac09g00870.1 0.558 1 0.442 0.052 1	1<br>Lperr01g00510.1 0.001 0.457 0.456 0.007 1<br>Lperr09g00030.1 0.558 1 0.442 0.031 1	1<br>AT1G20380.1 0.558 1 0.442 0.001 1<br>AT5G08440.2 0.002 0.457 0.455 0.012 0.997
Osjap01g00640.3	1<br>Osjap01g00640.1 0.001 1 0.999 0.001 1<br>Osjap01g00640.2 0.001 1 0.999 0.001 1<br>Osjap01g00640.3 0.001 1 0.999 0.001 1<br>Osjap01g00640.4 0.001 1 0.999 0.001 1	1<br>Osind01g00770.1 0.107 1 0.893 0.022 1<br>Osind01g00770.2 0.107 1 0.893 0.027 1	0	0	0	0	0	0	0	0
	1<br>Osjap04g25390.2 0.564 1 0.436 0.019 1<br>Osjap05g00080.1 0.001 0.458 0.457 0.007 0.999	1<br>Osind04g24430.1 0.564 1 0.436 0.019 1<br>Osind05g00060.1 0.001 0.458 0.457 0.007 0.999	1<br>Orufi01g00590.1 0.558 1 0.442 0.001 1<br>Orufi05g00070.1 0.001 0.458 0.457 0.007 0.999	1<br>Oglab01g00510.1 0.558 1 0.442 0.001 1<br>Oglab05g00040.1 0.001 0.458 0.457 0.007 0.999	1<br>Obart01g00590.1 0.558 1 0.442 0.001 1<br>Obart05g00050.1 0.001 0.458 0.457 0.007 0.999	1<br>Olong_015014-R1 0.459 1 0.541 0.001 1<br>Olong_015017-R1 0.001 0.458 0.457 0.001 0.999	1<br>Opunc01g00510.1 0.001 0.458 0.457 0.078 0.999<br>Opunc09g01380.1 0.547 1 0.453 0.03 1	1<br>Obrac05g00060.2 0.001 0.458 0.457 0.008 0.998<br>Obrac09g00870.1 0.557 1 0.443 0.052 1	1<br>Lperr01g00510.1 0.001 0.458 0.457 0.007 0.998<br>Lperr09g00030.1 0.557 1 0.443 0.031 1	1<br>AT1G20380.1 0.558 1 0.442 0.001 1<br>AT5G08440.2 0.002 0.458 0.456 0.012 0.999
Osjap01g00640.4	1<br>Osjap01g00640.1 0.001 1 0.999 0.001 1<br>Osjap01g00640.2 0.001 1 0.999 0.001 1<br>Osjap01g00640.3 0.001 1 0.999 0.001 1<br>Osjap01g00640.4 0.001 1 0.999 0.001 1	1<br>Osind01g00770.1 0.109 1 0.891 0.022 1<br>Osind01g00770.2 0.109 1 0.891 0.027 1	0	0	0	0	0	0	0	0
	1<br>Osjap04g25390.2 0.555 1 0.445 0.019 1<br>Osjap05g00080.1 0.001 0.467 0.466 0.007 0.999	1<br>Osind04g24430.1 0.555 1 0.445 0.019 1<br>Osind05g00060.1 0.001 0.467 0.466 0.007 0.999	1<br>Orufi01g00590.1 0.549 1 0.451 0.001 1<br>Orufi05g00070.1 0.001 0.467 0.466 0.007 0.999	1<br>Oglab01g00510.1 0.549 1 0.451 0.001 1<br>Oglab05g00040.1 0.001 0.467 0.466 0.007 0.999	1<br>Obart01g00590.1 0.549 1 0.451 0.001 1<br>Obart05g00050.1 0.001 0.467 0.466 0.007 0.999	1<br>Olong_015014-R1 0.468 1 0.532 0.001 1<br>Olong_015017-R1 0.001 0.467 0.466 0.001 0.999	1<br>Opunc01g00510.1 0.001 0.467 0.466 0.078 0.999<br>Opunc09g01380.1 0.538 1 0.462 0.03 1	1<br>Obrac05g00060.2 0.001 0.467 0.466 0.008 0.998<br>Obrac09g00870.1 0.548 1 0.452 0.052 1	1<br>Lperr01g00510.1 0.001 0.467 0.466 0.007 0.998<br>Lperr09g00030.1 0.548 1 0.452 0.031 1	1<br>AT1G20380.1 0.549 1 0.451 0.001 1<br>AT5G08440.2 0.002 0.467 0.465 0.012 0.999
Osjap01g42770.1	1	1	1	0	0	1	0	0	0	0

	Osjap01g42770.1 0.001 1 0.999 0.001 1	Osind01g44140.1 0.001 1 0.999 0.001 1	Orufi01g40790.1 0.001 1 0.999 0.001 1<br>Orufi01g40790.2 0.001 1 0.999 0.001 1<br>Orufi01g40790.3 0.001 1 0.999 0.001 1			Olong_024830-R1 0.001 1 0.999 0.001 1				
	1<br>Osjap01g08100.1 0.012 0.27 0.258 0.133 0.963<br>Osjap03g27560.1 0.413 0.995 0.582 0.146 0.95	1<br>Osind01g08020.1 0.012 0.27 0.258 0.133 0.963<br>Osind03g28340.1 0.413 0.995 0.582 0.146 0.95	1<br>Orufi02g20240.3 0.012 0.259 0.247 0.044 0.951<br>Orufi03g26160.3 0.413 0.995 0.582 0.146 0.95	1<br>Oglab01g32220.1 0.001 0.258 0.257 0.004 0.934<br>Oglab01g32230.1 0.352 0.999 0.647 0.002 0.955	1<br>Obart01g37410.1 0.001 0.258 0.257 0.004 0.934<br>Obart01g37420.1 0.352 1 0.648 0.002 1	1<br>Olong_010570-R1 0.413 0.995 0.582 0.133 0.915<br>Olong_011675-R1 0.012 0.258 0.246 0.517 0.972	1<br>Opunc01g36330.1 0.001 0.258 0.257 0.005 0.975<br>Opunc01g36340.1 0.352 0.999 0.647 0.002 0.969	1<br>Obrac01g28830.1 0.001 0.257 0.256 0.004 0.978<br>Obrac01g28840.1 0.355 1 0.645 0.002 1	1<br>Lperr01g32130.1 0.001 0.258 0.257 0.005 0.977<br>Lperr01g32140.1 0.352 1 0.648 0.002 1	0<br>AT2G37970.1 0.001 0.258 0.257 0.049 0.978<br>AT3G54090.1 0.461 1 0.539 0.123 1
Osjap01g50410.1	1<br>Osjap01g50410.1 0.001 1 0.999 0.001 1<br>Osjap01g50410.2 0.005 1 0.995 0.005 1	0	0	0	0	0	0	0	0	0
	1<br>Osjap05g17010.3 0.078 0.362 0.284 0.258 0.997<br>Osjap05g22890.1 0.439 0.998 0.559 0.089 0.979	1<br>Osind01g51960.1 0.009 0.362 0.353 0.022 0.997<br>Osind01g51970.1 0.425 1 0.575 0.001 1	1<br>Orufi01g47830.2 0.009 0.362 0.353 0.022 0.997<br>Orufi03g16980.1 0.439 0.994 0.555 0.031 0.991	1<br>Oglab03g03400.1 0.44 0.999 0.559 0.028 0.983<br>Oglab05g14070.1 0.084 0.362 0.278 0.227 0.997	1<br>Obart01g44360.2 0.02 0.362 0.342 0.024 0.997<br>Obart01g44370.1 0.425 1 0.575 0.001 1	1<br>Olong_021838-R1 0.039 0.362 0.323 0.002 0.996<br>Olong_021839-R1 0.463 1 0.537 0.001 1	1<br>Opunc01g43300.2 0.039 0.366 0.327 0.002 0.995<br>Opunc01g43310.1 0.425 1 0.575 0.001 1	1<br>Obrac01g34770.2 0.007 0.362 0.355 0.062 0.997<br>Obrac01g34780.1 0.445 1 0.555 0.001 0.999	1<br>Lperr01g38790.2 0.039 0.363 0.324 0.002 0.998<br>Lperr03g14360.1 0.427 0.994 0.567 0.008 0.991	1<br>AT3G21070.1 0.083 0.362 0.279 0.062 1<br>AT3G56370.1 0.428 0.996 0.568 0.004 0.994
Osjap01g50410.2	1<br>Osjap01g50410.1 0.005 1 0.995 0.005 1<br>Osjap01g50410.2 0.001 1 0.999 0.001 1	0	0	0	0	0	0	0	0	0
	1<br>Osjap05g17010.3 0.058 0.348 0.29 0.258 0.997<br>Osjap05g22890.1 0.426 0.998 0.572 0.089 0.979	1<br>Osind01g51960.1 0.009 0.348 0.339 0.022 0.997<br>Osind01g51970.1 0.412 1 0.588 0.001 1	1<br>Orufi01g47830.2 0.009 0.348 0.339 0.022 0.997<br>Orufi03g16980.1 0.426 0.994 0.568 0.031 0.991	1<br>Oglab03g03400.1 0.427 0.999 0.572 0.028 0.983<br>Oglab05g14070.1 0.063 0.348 0.285 0.227 0.997	1<br>Obart01g44360.2 0.021 0.348 0.327 0.024 0.997<br>Obart01g44370.1 0.412 1 0.588 0.001 1	1<br>Olong_021838-R1 0.041 0.348 0.307 0.072 0.996<br>Olong_021839-R1 0.451 1 0.549 0.001 1	1<br>Opunc01g43300.4 0.04 0.351 0.311 0.002 0.994<br>Opunc01g43310.1 0.412 1 0.588 0.001 1	1<br>Obrac01g34770.2 0.008 0.348 0.34 0.062 0.997<br>Obrac01g34780.1 0.432 1 0.568 0.001 0.999	1<br>Lperr01g38790.4 0.04 0.349 0.309 0.002 0.998<br>Lperr03g14360.1 0.414 0.994 0.58 0.008 0.991	1<br>AT3G21070.1 0.062 0.348 0.286 0.062 1<br>AT3G56370.1 0.415 0.996 0.581 0.004 0.994
Osjap02g23220.1	1<br>Osjap02g23220.1 0.001 1 0.999 0.001 1	0	0	0	0	0	1<br>Opunc02g18320.1 0.001 0.998 0.997 0.001 0.989<br>Opunc02g18320.2 0.001 1 0.999 0.001 1	1<br>Obrac02g14310.1 0.001 0.987 0.986 0.001 1<br>Obrac02g14310.2 0.001 0.987 0.986 0.001 1<br>Obrac02g14310.3 0.001 0.987 0.986 0.001 1	0	0
	1<br>Osjap03g34460.2 0.001 0.351 0.35 0.003 0.918<br>Osjap04g16230.4 0.423 0.99 0.567 0.015 0.983	1<br>Osind03g35700.1 0.001 0.351 0.35 0.003 0.918<br>Osind04g14780.2 0.423 0.99 0.567 0.016 0.983	1<br>Orufi02g21480.1 0.372 0.998 0.626 0.039 0.983<br>Orufi02g21490.1 0.001 0.336 0.335 0.003 0.948	1<br>Oglab03g30900.1 0.001 0.351 0.35 0.221 0.936<br>Oglab04g11660.2 0.423 0.99 0.567 0.015 0.983	1<br>Obart02g20410.1 0.412 1 0.588 0.002 1<br>Obart03g31870.1 0.001 0.351 0.35 0.281 0.941	1<br>Olong_017754-R1 0.378 0.967 0.589 0.071 1<br>Olong_017755-R1 0.001 0.329 0.328 0.003 0.931	1<br>Opunc02g18320.3 0.001 0.357 0.356 0.003 0.957<br>Opunc04g11260.2 0.423 0.991 0.568 0.015 0.987	1<br>Obrac03g23560.1 0.001 0.314 0.313 0.004 0.94<br>Obrac04g07710.2 0.42 0.985 0.565 0.017 0.977	1<br>Lperr02g16420.1 0.412 0.987 0.575 0.002 0.994<br>Lperr02g16430.2 0.001 0.356 0.355 0.003 0.941	1<br>AT1G21750.1 0.411 0.968 0.557 0.004 0.964<br>AT5G22370.1 0.001 0.333 0.332 0.003 0.909
Osjap02g28800.3	1<br>Osjap02g28800.1 0.001 1 0.999 0.001 1<br>Osjap02g28800.2 0.001 1 0.999 0.001 1<br>Osjap02g28800.3 0.001 1 0.999 0.001 1	0	1<br>Orufi02g26700.1 0.001 0.99 0.989 0.001 0.965	0	0	1<br>Olong_027102-R1 0.144 1 0.856 0.033 1	1<br>Opunc02g23140.1 0.001 1 0.999 0.001 1	0	1<br>Lperr02g20690.1 0.001 1 0.999 0.001 1	0
	1<br>Osjap04g33800.1 0.049 0.673 0.624 0.039 0.974<br>Osjap11g07330.1 0.688 0.998 0.31 0.005 0.995	1<br>Osind02g28600.2 0.684 1 0.316 0.003 1<br>Osind02g28610.1 0.001 0.673 0.672 0.001 0.982	1<br>Orufi02g26700.2 0.001 0.673 0.672 0.001 0.982<br>Orufi11g06530.1 0.688 1 0.312 0.005 1	1<br>Oglab03g04200.1 0.039 0.673 0.634 0.004 0.987<br>Oglab11g05820.1 0.688 0.998 0.31 0.005 0.995	1<br>Obart02g25330.2 0.684 1 0.316 0.003 1<br>Obart02g25340.1 0.001 0.673 0.672 0.001 0.982	1<br>Olong_010559-R1 0.675 0.996 0.321 0.126 0.991<br>Olong_017224-R1 0.068 0.673 0.605 0.014 0.986	0<br>Opunc02g23140.2 0.001 0.673 0.672 0.001 0.982	1<br>Obrac02g18260.1 0.684 1 0.316 0.003 1<br>Obrac02g18270.1 0.001 0.673 0.672 0.001 0.982	1<br>Lperr02g11790.1 0.051 0.673 0.622 0.079 0.981<br>Lperr10g02180.1 0.683 0.998 0.315 0.035 1	1<br>AT1G22440.1 0.686 1 0.314 0.01 1<br>AT5G23390.1 0.001 0.673 0.672 0.016 0.981
Osjap02g36880.1	1<br>Osjap02g36880.1 0.001 1 0.999 0.001 1	0	0	0	0	0	0	1<br>Obrac02g23910.1 0.001 1 0.999 0.136 1	0	0
	1	1	1	1	0	1	1	1	1	1

	Osjap06g08840.1 0.384 0.982 0.598 0.111 0.978<br>Osjap06g08870.1 0.052 0.363 0.311 0.086 0.957	Osind02g36410.1 0.001 0.363 0.362 0.264 0.969<br>Osind02g36420.1 0.363 1 0.637 0.021 1	Orufi02g34400.1 0.001 0.363 0.362 0.003 0.958<br>Orufi02g34410.1 0.363 1 0.637 0.021 1	Oglab02g30250.1 0.001 0.363 0.362 0.003 0.919<br>Oglab02g30260.1 0.363 1 0.637 0.121 1	Obart02g32720.1 0.306 1 0.694 0.014 1	Olong_003175-R1 0.363 1 0.637 0.021 1<br>Olong_003176-R2 0.001 0.363 0.362 0.184 0.961	Opunc02g30220.3 0.001 0.363 0.362 0.264 0.969<br>Opunc02g30230.1 0.363 1 0.637 0.022 1	Obrac05g14180.1 0.398 0.994 0.596 0.033 0.938<br>Obrac06g05990.1 0.001 0.363 0.362 0.003 0.953	Lperr02g26790.1 0.001 0.363 0.362 0.296 0.97<br>Lperr02g26800.1 0.363 1 0.637 0.022 1	AT1G22170.1 0.001 0.363 0.362 0.003 0.946<br>AT5G65090.1 0.413 0.998 0.585 0.081 1
Osjap02g40340.1	1<br>Osjap02g40310.1 0.003 0.982 0.979 0.162 0.926<br>Osjap02g40340.1 0.003 1 0.997 0.003 1<br>Osjap02g40370.1 0.003 0.982 0.979 0.002 0.726	1<br>Osind02g39860.1 0.003 0.985 0.982 0.002 0.659	0	0	0	0	0	0	0	0
	1<br>Osjap02g41540.1 0.655 0.997 0.342 0.434 0.806<br>Osjap03g26380.1 0.003 0.292 0.289 0.004 0.406<br>Osjap05g23970.1 0.368 0.591 0.223 0.665 0.821	1<br>Osind02g39840.1 0.003 0.292 0.289 0.007 0.809<br>Osind02g39860.2 0.332 0.985 0.653 0.015 0.571	0<br>Orufi02g37680.6 0.332 0.992 0.66 0.022 0.879	1<br>Oglab02g33340.1 0.361 1 0.639 0.042 1<br>Oglab03g24170.1 0.003 0.292 0.289 0.004 0.406	1<br>Obart02g36180.3 0.332 0.992 0.66 0.019 0.724<br>Obart03g24380.1 0.003 0.292 0.289 0.004 0.406	1<br>Olong_011081-R1 0.652 1 0.348 0.05 1<br>Olong_011082-R1 0.003 0.294 0.291 0.007 0.752<br>Olong_025373-R1 0.299 0.606 0.307 0.116 0.574	1<br>Opunc02g34450.1 0.655 0.997 0.342 0.432 0.806<br>Opunc03g22570.2 0.003 0.294 0.291 0.007 0.816<br>Opunc04g19490.1 0.368 0.588 0.22 0.521 0.726	1<br>Obrac02g27500.6 0.655 0.997 0.342 0.432 0.806<br>Obrac03g19420.1 0.003 0.292 0.289 0.007 0.81<br>Obrac05g14470.1 0.368 0.588 0.22 0.584 0.771	1<br>Lperr02g29800.1 0.018 0.292 0.274 0.234 0.846<br>Lperr02g30750.1 0.655 0.997 0.342 0.453 0.814<br>Lperr06g23460.1 0.368 0.606 0.238 0.548 0.76	1<br>AT1G76310.1 0.376 0.606 0.23 0.571 0.773<br>AT2G20360.1 0.655 0.997 0.342 0.423 0.801<br>AT5G38650.1 0.003 0.292 0.289 0.007 0.809
Osjap03g00680.1	1<br>Osjap03g00680.1 0.001 1 0.999 0.001 1<br>Osjap03g00680.2 0.001 1 0.999 0.001 1<br>Osjap03g00680.3 0.001 1 0.999 0.001 1	1<br>Osind03g00740.1 0.001 0.994 0.993 0.001 0.996	1<br>Orufi03g12660.1 0.001 1 0.999 0.001 1<br>Orufi03g12660.2 0.001 1 0.999 0.001 1	1<br>Oglab03g00390.1 0.001 1 0.999 0.001 1	1<br>Obart03g00550.1 0.001 0.994 0.993 0.001 0.996	0	0	0	0	0
	1<br>Osjap02g42090.1 0.051 0.661 0.61 0.065 1<br>Osjap12g08580.1 0.71 0.999 0.289 0.011 0.996	1<br>Osind02g41670.1 0.06 0.661 0.601 0.131 1<br>Osind03g02220.1 0.7 1 0.3 0.015 1	1<br>Orufi02g39350.1 0.06 0.661 0.601 0.131 1<br>Orufi09g02220.2 0.746 1 0.254 0.095 1	1<br>Oglab02g34920.1 0.06 0.661 0.601 0.131 1<br>Oglab03g02050.1 0.688 1 0.312 0.187 1	1<br>Obart02g37790.1 0.06 0.632 0.572 0.133 0.977<br>Obart12g06510.1 0.685 0.999 0.314 0.066 0.997	0<br>Olong_013175-R1 0.264 0.904 0.64 0.002 0.961	1<br>Opunc03g00470.1 0.001 0.783 0.782 0.002 1<br>Opunc11g04590.1 0.783 0.998 0.215 0.055 1	1<br>Obrac02g10260.1 0.712 0.999 0.287 0.265 0.995<br>Obrac02g27970.1 0.134 0.661 0.527 0.105 1	1<br>Lperr03g00570.1 0.001 0.657 0.656 0.002 0.994<br>Lperr04g11360.1 0.752 0.998 0.246 0.415 0.998	0<br>AT5G04460.1 0.017 0.661 0.644 0.081 1
Osjap03g00680.2	1<br>Osjap03g00680.1 0.001 1 0.999 0.001 1<br>Osjap03g00680.2 0.001 1 0.999 0.001 1<br>Osjap03g00680.3 0.001 1 0.999 0.001 1	1<br>Osind03g00740.1 0.001 0.994 0.993 0.001 0.996	1<br>Orufi03g12660.1 0.001 1 0.999 0.001 1<br>Orufi03g12660.2 0.001 1 0.999 0.001 1	1<br>Oglab03g00390.1 0.001 1 0.999 0.001 1	1<br>Obart03g00550.1 0.001 0.994 0.993 0.001 0.996	0	0	0	0	0
	1<br>Osjap02g42090.1 0.052 0.68 0.628 0.065 1<br>Osjap12g08580.1 0.702 0.999 0.297 0.011 0.996	1<br>Osind02g41670.1 0.062 0.68 0.618 0.131 1<br>Osind03g02220.1 0.691 1 0.309 0.015 1	1<br>Orufi02g39350.1 0.062 0.68 0.618 0.131 1<br>Orufi09g02220.2 0.738 1 0.262 0.095 1	1<br>Oglab02g34920.1 0.062 0.68 0.618 0.131 1<br>Oglab03g02050.1 0.691 1 0.309 0.214 1	1<br>Obart02g37790.1 0.062 0.65 0.588 0.133 0.977<br>Obart12g06510.1 0.681 0.999 0.318 0.079 0.997	0<br>Olong_013175-R1 0.272 0.902 0.63 0.002 0.961	1<br>Opunc03g00470.1 0.001 0.777 0.776 0.002 1<br>Opunc03g03130.2 0.777 0.999 0.222 0.443 0.997	1<br>Obrac02g10260.1 0.704 0.999 0.295 0.265 0.995<br>Obrac02g27970.1 0.138 0.68 0.542 0.105 1	1<br>Lperr03g00570.1 0.001 0.68 0.679 0.002 1<br>Lperr04g11360.1 0.745 0.998 0.253 0.415 0.998	0<br>AT5G04460.1 0.018 0.68 0.662 0.081 1
Osjap03g00710.1	1<br>Osjap03g00710.1 0.002 1 0.998 0.002 1	0	1<br>Orufi03g12690.1 0.002 1 0.998 0.002 1<br>Orufi03g12690.2 0.002 1 0.998 0.002 1	0	0	0	0	0	0	0
	1<br>Osjap08g14010.2 0.424 0.983 0.559 0.444 0.966<br>Osjap10g20350.1 0.002 0.245 0.243 0.15 0.969	1<br>Osind03g00760.1 0.316 0.994 0.678 0.002 0.841<br>Osind03g00790.1 0.002 0.28 0.278 0.006 0.879	1<br>Orufi08g14730.1 0.424 0.983 0.559 0.503 0.97<br>Orufi10g18610.1 0.002 0.245 0.243 0.15 0.969	0<br>Oglab03g00420.2 0.234 0.994 0.76 0.128 0.895	1<br>Obart03g00580.1 0.316 1 0.684 0.003 0.966<br>Obart03g00600.1 0.002 0.28 0.278 0.006 0.879	1<br>Olong_002892-R1 0.002 0.245 0.243 0.16 0.969<br>Olong_013172-R3 0.355 0.998 0.643 0.201 0.985	1<br>Opunc03g00510.2 0.316 0.994 0.678 0.003 0.891<br>Opunc03g00530.1 0.002 0.237 0.235 0.008 0.977	1<br>Obrac03g00430.2 0.355 0.993 0.638 0.213 0.962<br>Obrac10g11260.2 0.002 0.237 0.235 0.182 0.929	1<br>Lperr03g00610.3 0.316 0.987 0.671 0.004 0.948<br>Lperr10g13450.1 0.006 0.245 0.239 0.167 0.969	1<br>AT3G52180.1 0.316 0.993 0.677 0.003 0.982<br>AT5G03455.1 0.002 0.237 0.235 0.116 0.979
Osjap03g13100.1	1<br>Osjap03g13100.1 0.001 1 0.999 0.001 1	1<br>Osind03g12930.1 0.001 1 0.999 0.028 1	0	1<br>Oglab03g12120.1 0.001 1 0.999 0.028 1	1<br>Obart03g12120.1 0.001 1 0.999 0.028 1	0	0	0	0	0
	1<br>Osjap04g16240.1 0.632 0.998 0.366 0.082 0.921<br>Osjap12g01140.1 0.084 0.622 0.538 0.498 0.981	1<br>Osind04g14790.1 0.632 0.998 0.366 0.013 0.911<br>Osind12g01290.1 0.084 0.622 0.538 0.49 0.981	1<br>Orufi06g01460.3 0.632 0.998 0.366 0.212 0.912<br>Orufi12g01130.1 0.084 0.622 0.538 0.498 0.981	1<br>Oglab06g01620.1 0.632 0.998 0.366 0.233 0.912<br>Oglab12g00800.1 0.089 0.622 0.533 0.483 0.983	1<br>Obart06g01490.1 0.632 0.998 0.366 0.233 0.912<br>Obart11g01060.1 0.089 0.622 0.533 0.471 0.982	1<br>Olong_007714-R9 0.632 1 0.368 0.002 1<br>Olong_007715-R1 0.1 0.623 0.523 0.007 0.965	1<br>Opunc01g28650.1 0.636 0.998 0.362 0.094 0.923<br>Opunc03g11680.1 0.001 0.623 0.622 0.001 0.971	1<br>Obrac03g09720.2 0.001 0.623 0.622 0.001 0.97<br>Obrac04g09430.1 0.63 0.998 0.368 0.014 0.863	1<br>Lperr03g10720.1 0.001 0.623 0.622 0.001 0.934<br>Lperr03g17470.1 0.623 0.998 0.375 0.119 0.828	1<br>AT1G20230.1 0.635 0.998 0.363 0.104 0.833<br>AT3G46740.1 0.097 0.623 0.526 0.158 0.971

Osjap03g30460.1	1<br>Osjap03g30460.1 0.001 1 0.999 0.001 1	1<br>Osind03g31500.1 0.018 1 0.982 0.031 1	1<br>Orufi03g29110.1 0.018 1 0.982 0.296 1	0	1<br>Obart03g27980.1 0.08 1 0.92 0.016 1<br>Obart03g27980.2 0.08 1 0.92 0.015 1	0	0	0	0	0
	1<br>Osjap03g30380.1 0.547 0.999 0.452 0.059 0.866<br>Osjap12g24400.2 0.083 0.517 0.434 0.012 0.988	1<br>Osind08g14630.1 0.547 0.999 0.452 0.069 0.96<br>Osind12g20440.1 0.083 0.517 0.434 0.01 0.99	1<br>Orufi03g29970.1 0.547 0.999 0.452 0.035 0.998<br>Orufi12g21550.1 0.083 0.517 0.434 0.01 0.99	1<br>Oglab03g27330.1 0.547 1 0.453 0.036 1<br>Oglab12g17460.4 0.083 0.517 0.434 0.012 0.996	1<br>Obart03g27900.1 0.547 0.999 0.452 0.053 0.865<br>Obart12g19300.1 0.083 0.517 0.434 0.01 0.99	1<br>Olong_002926-R1 0.088 0.519 0.431 0.002 0.996<br>Olong_007449-R1 0.547 0.999 0.452 0.058 0.881	1<br>Opunc03g25490.1 0.014 0.528 0.514 0.486 0.8<br>Opunc03g26870.1 0.548 0.999 0.451 0.053 1	0<br>Obrac03g20710.1 0.085 0.519 0.434 0.028 0.997	1<br>Lperr03g23190.1 0.547 0.999 0.452 0.03 0.867<br>Lperr03g23200.1 0.07 0.519 0.449 0.002 0.997	0<br>AT5G36940.1 0.084 0.52 0.436 0.003 0.998
Osjap03g34330.1	1<br>Osjap03g34330.1 0.001 1 0.999 0.001 1	1<br>Osind03g35550.1 0.001 1 0.999 0.039 1	1<br>Orufi03g33060.1 0.001 1 0.999 0.001 1	1<br>Oglab03g30830.1 0.001 1 0.999 0.001 1	1<br>Obart03g31750.1 0.001 1 0.999 0.046 1	0	0	0	0	0
	1<br>Osjap03g39110.1 0.634 1 0.366 0.036 1<br>Osjap10g05370.1 0.012 0.609 0.597 0.1 0.963	1<br>Osind01g46540.1 0.634 0.999 0.365 0.065 0.994<br>Osind10g03770.1 0.012 0.609 0.597 0.11 0.96	1<br>Orufi10g04850.1 0.031 0.609 0.578 0.093 0.963<br>Orufi11g12520.1 0.635 1 0.365 0.038 0.991	1<br>Oglab03g34460.1 0.609 0.997 0.388 0.016 0.993<br>Oglab10g04340.1 0.012 0.609 0.597 0.1 0.963	1<br>Obart03g36200.1 0.634 1 0.366 0.016 0.997<br>Obart10g04600.1 0.014 0.609 0.595 0.106 0.962	1<br>Olong_014796-R1 0.001 0.516 0.515 0.002 0.95<br>Olong_027495-R1 0.609 0.994 0.385 0.005 0.998	1<br>Opunc01g42030.1 0.634 1 0.366 0.28 0.872<br>Opunc03g28940.2 0.001 0.609 0.608 0.002 0.962	1<br>Obrac02g16280.1 0.628 0.981 0.353 0.063 0.949<br>Obrac03g23440.1 0.001 0.609 0.608 0.002 0.964	1<br>Lperr03g26400.1 0.001 0.609 0.608 0.002 0.962<br>Lperr03g30520.1 0.626 1 0.374 0.003 0.997	0<br>AT1G70730.3 0.001 0.609 0.608 0.118 0.967
Osjap03g40400.1	1<br>Osjap03g40400.1 0.001 1 0.999 0.001 1	0	0	0	0	0	1<br>Opunc03g34420.1 0.025 0.985 0.96 0.001 0.985	0	0	0
	1<br>Osjap07g05740.1 0.583 0.999 0.416 0.003 1<br>Osjap07g05830.1 0.001 0.559 0.558 0.002 0.99	1<br>Osind03g41490.1 0.001 0.559 0.558 0.002 0.99<br>Osind07g04980.1 0.583 0.999 0.416 0.003 1	1<br>Orufi07g04840.1 0.583 0.999 0.416 0.003 1<br>Orufi07g04930.1 0.001 0.559 0.558 0.002 0.99	1<br>Oglab07g04840.1 0.631 0.999 0.368 0.021 1<br>Oglab07g04940.1 0.001 0.559 0.558 0.002 0.99	1<br>Obart03g37340.1 0.001 0.559 0.558 0.003 0.99<br>Obart07g05050.1 0.583 0.999 0.416 0.003 1	0<br>Olong_017306-R1 0.541 0.985 0.444 0.171 0.972	1<br>Opunc07g04870.1 0.583 0.999 0.416 0.003 1<br>Opunc07g04960.1 0.001 0.559 0.558 0.002 0.99	1<br>Obrac03g27810.1 0.001 0.559 0.558 0.002 0.99<br>Obrac07g03140.1 0.583 0.999 0.416 0.003 1	1<br>Lperr03g31580.1 0.575 0.985 0.41 0.203 0.971<br>Lperr03g31590.1 0.001 0.559 0.558 0.002 0.99	1<br>AT3G09630.1 0.003 0.559 0.556 0.007 0.99<br>AT5G20030.1 0.639 0.997 0.358 0.172 0.975
Osjap03g44070.1	1<br>Osjap03g44070.1 0.001 1 0.999 0.001 1	0	1<br>Orufi03g42420.1 0.001 1 0.999 0.001 1	0	0	1<br>Olong_021918-R1 0.036 0.967 0.931 0.001 0.957<br>Olong_021918-R2 0.036 0.967 0.931 0.001 0.958	1<br>Opunc03g37840.1 0.001 1 0.999 0.001 0.995	0	0	0
	1<br>Osjap03g44260.1 0.569 1 0.431 0.062 1<br>Osjap05g21490.1 0.016 0.535 0.519 0.057 0.81	1<br>Osind01g39050.1 0.567 1 0.433 0.029 0.996<br>Osind03g45110.2 0.001 0.54 0.539 0.002 0.991	1<br>Orufi03g42640.1 0.569 1 0.431 0.056 1<br>Orufi05g20190.1 0.016 0.535 0.519 0.058 0.817	1<br>Oglab03g38640.1 0.001 0.54 0.539 0.001 0.991<br>Oglab03g38820.1 0.569 0.97 0.401 0.063 0.949	1<br>Obart03g40680.1 0.001 0.54 0.539 0.001 0.991<br>Obart03g40860.1 0.569 1 0.431 0.056 1	1<br>Olong_002818-R1 0.567 0.999 0.432 0.026 0.996<br>Olong_017812-R1 0.032 0.542 0.51 0.001 0.964	1<br>Opunc06g00320.1 0.569 1 0.431 0.083 0.875<br>Opunc12g13740.1 0.029 0.549 0.52 0.001 1	0<br>Obrac03g30660.1 0.001 0.539 0.538 0.001 0.989	1<br>Lperr01g38560.1 0.569 1 0.431 0.099 1<br>Lperr08g09550.1 0.014 0.536 0.522 0.05 0.981	0<br>AT4G13650.1 0.02 0.537 0.517 0.069 0.907
Osjap04g02830.1	1<br>Osjap04g02830.1 0.001 1 0.999 0.001 1	0	1<br>Orufi04g02580.1 0.054 0.859 0.805 0.403 0.971<br>Orufi04g02650.1 0.001 0.988 0.987 0.254 0.883	0	0	0	0	0	0	0
	1<br>Osjap04g02720.1 0.001 0.531 0.53 0.001 1<br>Osjap04g02730.1 0.545 1 0.455 0.049 1	1<br>Osind01g20610.1 0.545 1 0.455 0.052 1<br>Osind04g02110.1 0.07 0.509 0.439 0.629 1	1<br>Orufi04g02720.1 0.563 0.988 0.425 0.135 0.82<br>Orufi10g04890.1 0.001 0.503 0.502 0.001 0.96	0<br>Oglab12g09560.1 0.05 0.507 0.457 0.619 0.987	1<br>Obart04g01960.1 0.632 1 0.368 0.004 1<br>Obart10g04670.1 0.001 0.503 0.502 0.001 0.931	1<br>Olong_015999-R1 0.563 0.988 0.425 0.135 0.82<br>Olong_024256-R1 0.054 0.528 0.474 0.309 0.998	1<br>Opunc04g01960.1 0.545 0.991 0.446 0.13 0.994<br>Opunc04g02050.1 0.006 0.531 0.525 0.004 1	0<br>Obrac04g01260.1 0.054 0.509 0.455 0.502 1	0<br>Lperr04g14110.1 0.001 0.497 0.496 0.001 0.963	0<br>AT5G45480.1 0.095 0.509 0.414 0.701 0.987
Osjap04g11160.1	1<br>Osjap04g11160.1 0.001 1 0.999 0.001 1	1<br>Osind09g02760.1 0.001 1 0.999 0.001 1	1<br>Orufi04g09840.1 0.001 1 0.999 0.001 1	0	0	0	1<br>Opunc04g07220.1 0.017 0.988 0.971 0.005 1	0	1<br>Lperr04g05910.1 0.064 0.993 0.929 0.022 0.997	0
	1<br>Osjap10g21270.2 0.556 0.988 0.432 0.059 0.976<br>Osjap11g14300.1 0.007 0.518 0.511 0.04 0.878	1<br>Osind08g20650.1 0.007 0.518 0.511 0.04 0.878<br>Osind09g05810.1 0.548 0.978 0.43 0.004 0.868	1<br>Orufi10g19500.1 0.556 0.988 0.432 0.017 0.976<br>Orufi11g12980.1 0.007 0.518 0.511 0.039 0.881	1<br>Oglab09g05260.1 0.548 0.978 0.43 0.004 0.868<br>Oglab11g10800.1 0.007 0.518 0.511 0.04 0.878	1<br>Obart04g08610.1 0.538 1 0.462 0.001 1<br>Obart11g12300.1 0.007 0.518 0.511 0.04 0.878	1<br>Olong_024054-R1 0.577 1 0.423 0.002 1<br>Olong_024055-R1 0.009 0.541 0.532 0.001 0.96	1<br>Opunc10g16660.1 0.556 0.988 0.432 0.053 0.976<br>Opunc11g10540.3 0.007 0.518 0.511 0.087 0.889	1<br>Obrac04g04850.1 0.007 0.549 0.542 0.043 0.975<br>Obrac10g11900.3 0.559 0.983 0.424 0.029 0.935	1<br>Lperr10g14190.1 0.556 0.988 0.432 0.114 0.977<br>Lperr11g10010.2 0.007 0.518 0.511 0.037 0.886	1<br>AT4G18750.1 0.564 0.999 0.435 0.069 0.882<br>AT5G44240.1 0.008 0.518 0.51 0.066 0.889

Osjap04g27970.1	1<br>Osjap04g27970.1 0.001 1 0.999 0.001 1	0	1<br>Orufi04g25730.1 0.014 0.941 0.927 0.016 0.995	0	0	0	0	0	1<br>Lperr04g19670.1 0.033 0.941 0.908 0.042 0.995	0
	1<br>Osjap02g33010.1 0.481 0.938 0.457 0.087 0.989<br>Osjap02g40270.1 0.073 0.442 0.369 0.204 0.995	1<br>Osind04g27100.1 0.001 0.442 0.441 0.002 1<br>Osind04g27110.1 0.481 0.939 0.458 0.089 0.987	1<br>Orufi02g30690.1 0.481 0.938 0.457 0.087 0.989<br>Orufi02g37650.1 0.073 0.442 0.369 0.204 0.995	1<br>Oglab04g22120.1 0.014 0.442 0.428 0.032 1<br>Oglab04g22130.1 0.481 0.939 0.458 0.089 0.987	1<br>Obart04g23980.1 0.001 0.442 0.441 0.002 1<br>Obart04g23990.1 0.481 0.939 0.458 0.089 0.987	1<br>Olong_003249-R1 0.481 0.938 0.457 0.087 0.989<br>Olong_008382-R1 0.02 0.414 0.394 0.064 0.969	1<br>Opunc04g21520.1 0.014 0.442 0.428 0.032 1<br>Opunc04g21530.1 0.481 0.939 0.458 0.089 0.987	1<br>Obrac04g16300.1 0.016 0.442 0.426 0.039 1<br>Obrac04g16310.1 0.481 0.939 0.458 0.089 0.987	1<br>Lperr02g23670.1 0.481 0.938 0.457 0.087 0.989<br>Lperr02g29790.1 0.073 0.442 0.369 0.23 0.995	1<br>AT1G80480.1 0.073 0.442 0.369 0.194 1<br>AT5G27080.1 0.474 0.935 0.461 0.017 0.927
Osjap05g00530.1	1<br>Osjap05g00530.1 0.001 1 0.999 0.001 1<br>Osjap05g00530.2 0.001 1 0.999 0.001 1<br>Osjap05g00530.3 0.001 1 0.999 0.001 1<br>Osjap05g00530.4 0.001 1 0.999 0.001 1	1<br>Osind05g00510.1 0.131 1 0.869 0.001 1	1<br>Orufi05g00500.1 0.001 1 0.999 0.001 1	0	1<br>Obart05g00520.1 0.084 0.999 0.915 0.001 0.993<br>Obart05g00520.2 0.084 1 0.916 0.001 1	1<br>Olong_022405-R1 0.131 1 0.869 0.001 1	0	0	0	0
	1<br>Osjap02g42390.1 0.422 0.993 0.571 0.001 0.983<br>Osjap07g27300.1 0.068 0.363 0.295 0.072 0.834	1<br>Osind02g41800.1 0.389 0.995 0.606 0.004 0.994<br>Osind06g29470.1 0.058 0.363 0.305 0.1 0.846	1<br>Orufi02g39480.2 0.389 0.994 0.605 0.004 0.992<br>Orufi07g24760.1 0.068 0.363 0.295 0.072 0.834	1<br>Oglab05g00490.2 0.375 1 0.625 0.099 0.996<br>Oglab05g00500.1 0.001 0.365 0.364 0.002 0.982	1<br>Obart06g01490.1 0.1 0.367 0.267 0.2 0.99<br>Obart09g15270.1 0.407 1 0.593 0.029 1	1<br>Olong_023813-R1 0.401 0.997 0.596 0.001 0.976<br>Olong_026414-R1 0.085 0.363 0.278 0.071 0.826	1<br>Opunc05g00480.1 0.397 0.999 0.602 0.007 0.998<br>Opunc05g00490.1 0.008 0.367 0.359 0.037 0.986	0<br>Obrac05g00480.2 0.062 0.363 0.301 0.093 0.998	1<br>Lperr04g17270.1 0.392 1 0.608 0.002 1<br>Lperr07g20540.1 0.052 0.363 0.311 0.079 0.819	0<br>AT4G37380.1 0.009 0.363 0.354 0.043 0.85
Osjap05g00530.2	1<br>Osjap05g00530.1 0.001 1 0.999 0.001 1<br>Osjap05g00530.2 0.001 1 0.999 0.001 1<br>Osjap05g00530.3 0.001 1 0.999 0.001 1<br>Osjap05g00530.4 0.001 1 0.999 0.001 1	1<br>Osind05g00510.1 0.16 1 0.84 0.001 1	1<br>Orufi05g00500.1 0.001 1 0.999 0.001 1	0	1<br>Obart05g00520.1 0.103 0.999 0.896 0.001 0.993<br>Obart05g00520.2 0.103 1 0.897 0.001 1	1<br>Olong_022405-R1 0.16 1 0.84 0.001 1	0	0	0	0
	1<br>Osjap07g27300.1 0.084 0.445 0.361 0.072 0.834<br>Osjap09g15690.3 0.487 1 0.513 0.003 1	1<br>Osind06g29470.1 0.071 0.446 0.375 0.1 0.846<br>Osind09g15830.1 0.487 1 0.513 0.029 1	1<br>Orufi07g24760.1 0.084 0.445 0.361 0.072 0.834<br>Orufi09g16480.1 0.487 1 0.513 0.029 1	1<br>Oglab05g00500.1 0.001 0.449 0.448 0.002 0.982<br>Oglab09g13820.1 0.486 1 0.514 0.093 1	1<br>Obart06g01490.1 0.123 0.451 0.328 0.2 0.99<br>Obart09g15270.1 0.487 1 0.513 0.029 1	1<br>Olong_020432-R1 0.074 0.445 0.371 0.013 0.62<br>Olong_025239-R1 0.486 1 0.514 0.029 1	1<br>Opunc02g35220.2 0.487 0.993 0.506 0.294 0.984<br>Opunc05g00490.1 0.01 0.45 0.44 0.037 0.986	0<br>Obrac05g00480.2 0.077 0.446 0.369 0.093 0.998	1<br>Lperr05g00500.1 0.486 1 0.514 0.022 0.996<br>Lperr07g20540.1 0.064 0.445 0.381 0.079 0.819	0<br>AT4G37380.1 0.011 0.445 0.434 0.043 0.85
Osjap05g00530.3	1<br>Osjap05g00530.1 0.001 1 0.999 0.001 1<br>Osjap05g00530.2 0.001 1 0.999 0.001 1<br>Osjap05g00530.3 0.001 1 0.999 0.001 1<br>Osjap05g00530.4 0.001 1 0.999 0.001 1	1<br>Osind05g00510.1 0.161 1 0.839 0.001 1	1<br>Orufi05g00500.1 0.001 1 0.999 0.001 1	0	1<br>Obart05g00520.1 0.104 0.999 0.895 0.001 0.993<br>Obart05g00520.2 0.104 1 0.896 0.001 1	1<br>Olong_022405-R1 0.161 1 0.839 0.001 1	0	0	0	0
	1<br>Osjap07g27300.1 0.084 0.46 0.376 0.072 0.873<br>Osjap09g15690.3 0.487 1 0.513 0.003 1	1<br>Osind08g09120.1 0.078 0.456 0.378 0.168 0.613<br>Osind09g15830.1 0.487 1 0.513 0.029 1	1<br>Orufi07g24760.1 0.084 0.46 0.376 0.072 0.873<br>Orufi09g16480.1 0.487 1 0.513 0.029 1	1<br>Oglab05g00500.1 0.001 0.456 0.455 0.002 0.993<br>Oglab09g13820.1 0.487 1 0.513 0.093 1	1<br>Obart06g01490.1 0.123 0.45 0.327 0.2 0.982<br>Obart09g15270.1 0.487 1 0.513 0.029 1	1<br>Olong_020432-R1 0.074 0.481 0.407 0.013 0.666<br>Olong_023813-R1 0.482 0.997 0.515 0.004 0.976	1<br>Opunc02g35220.2 0.485 0.993 0.508 0.294 0.984<br>Opunc05g00490.1 0.01 0.456 0.446 0.037 0.993	0<br>Obrac05g00480.2 0.077 0.448 0.371 0.093 0.998	1<br>Lperr05g00500.1 0.487 1 0.513 0.022 0.996<br>Lperr07g20540.1 0.064 0.46 0.396 0.079 0.857	0<br>AT4G37380.1 0.011 0.447 0.436 0.043 0.85
Osjap05g00530.4	1<br>Osjap05g00530.1 0.001 1 0.999 0.001 1<br>Osjap05g00530.2 0.001 1 0.999 0.001 1<br>Osjap05g00530.3 0.001 1 0.999 0.001 1<br>Osjap05g00530.4 0.001 1 0.999 0.001 1	1<br>Osind05g00510.1 0.142 1 0.858 0.001 1	1<br>Orufi05g00500.1 0.001 1 0.999 0.001 1	0	1<br>Obart05g00520.1 0.091 0.999 0.908 0.001 0.993<br>Obart05g00520.2 0.091 1 0.909 0.001 1	1<br>Olong_022405-R1 0.142 1 0.858 0.001 1	0	0	0	0
	1	1	1	1	1	1	1	0	1	0

	Osjap07g27300.1 0.074 0.394 0.32 0.072 0.834<br>Osjap09g15690.3 0.507 1 0.493 0.003 1	Osind06g29470.1 0.063 0.395 0.332 0.1 0.846<br>Osind09g15830.1 0.442 1 0.558 0.029 1	Orufi07g24760.1 0.074 0.394 0.32 0.072 0.834<br>Orufi09g16480.1 0.442 1 0.558 0.029 1	Oglab05g00490.2 0.407 1 0.593 0.099 0.996<br>Oglab05g00500.1 0.001 0.397 0.396 0.002 0.982	Obart02g34000.1 0.092 0.388 0.296 0.188 0.958<br>Obart09g15270.1 0.442 1 0.558 0.029 1	Olong_018361-R1 0.442 1 0.558 0.002 1<br>Olong_020432-R1 0.066 0.394 0.328 0.013 0.62	Opunc05g00480.1 0.431 0.999 0.568 0.007 0.998<br>Opunc05g00490.1 0.009 0.398 0.389 0.037 0.986	Obrac05g00480.2 0.068 0.395 0.327 0.093 0.998	Lperr04g17270.1 0.439 1 0.561 0.028 1<br>Lperr07g20540.1 0.056 0.394 0.338 0.079 0.819	AT4G37380.1 0.009 0.394 0.385 0.043 0.85
Osjap05g02530.1	1<br>Osjap05g02530.1 0.001 1 0.999 0.001 1	1<br>Osind05g02510.1 0.001 0.998 0.997 0.001 0.987	0	1<br>Oglab05g02190.1 0.002 1 0.998 0.079 1	1<br>Obart05g02340.1 0.002 1 0.998 0.076 1	0	0	0	0	0
	1<br>Osjap03g00960.1 0.459 0.994 0.535 0.132 0.996<br>Osjap06g18010.1 0.001 0.338 0.337 0.184 1	1<br>Osind03g01020.1 0.459 0.994 0.535 0.136 0.996<br>Osind06g17540.1 0.001 0.324 0.323 0.003 0.865	1<br>Orufi05g02460.2 0.459 1 0.541 0.003 0.982<br>Orufi06g16520.1 0.001 0.324 0.323 0.002 0.884	1<br>Oglab03g00660.1 0.459 0.994 0.535 0.124 0.996<br>Oglab05g07310.1 0.001 0.324 0.323 0.003 0.935	1<br>Obart03g00820.2 0.459 0.994 0.535 0.022 0.995<br>Obart06g15630.1 0.001 0.324 0.323 0.002 0.955	1<br>Olong_016162-R1 0.001 0.324 0.323 0.002 0.954<br>Olong_026687-R1 0.427 1 0.573 0.038 0.983	1<br>Opunc05g02220.2 0.459 0.999 0.54 0.003 0.979<br>Opunc07g20770.2 0.001 0.324 0.323 0.002 0.832	1<br>Obrac05g01840.1 0.458 0.998 0.54 0.168 1<br>Obrac06g15870.1 0.001 0.326 0.325 0.003 0.84	0<br>Lperr05g02100.1 0.457 0.998 0.541 0.015 0.978	0<br>AT5G15380.1 0.449 0.992 0.543 0.066 0.987
Osjap05g04120.1	1<br>Osjap05g04120.1 0.001 1 0.999 0.001 1<br>Osjap05g04120.2 0.001 1 0.999 0.001 1	1<br>Osind05g04070.1 0.008 1 0.992 0.001 1<br>Osind05g04070.2 0.008 1 0.992 0.001 1	1<br>Orufi05g03950.1 0.001 1 0.999 0.001 1<br>Orufi05g03950.2 0.001 1 0.999 0.001 1	1<br>Oglab05g03490.1 0.001 1 0.999 0.001 1<br>Oglab05g03490.2 0.001 1 0.999 0.001 1	1<br>Obart05g03830.1 0.001 1 0.999 0.001 1<br>Obart05g03830.2 0.001 1 0.999 0.001 1	1<br>Olong_016739-R1 0.074 1 0.926 0.001 1	1<br>Opunc05g03540.1 0.001 1 0.999 0.001 1<br>Opunc05g03540.2 0.001 1 0.999 0.001 1	1<br>Obrac05g02930.1 0.074 1 0.926 0.001 1	0	0
	1<br>Osjap01g27410.1 0.09 0.723 0.633 0.066 0.996<br>Osjap03g15140.5 0.743 1 0.257 0.061 1	1<br>Osind01g28630.1 0.09 0.723 0.633 0.066 0.996<br>Osind03g15230.1 0.79 1 0.21 0.03 1	1<br>Orufi01g26130.1 0.09 0.723 0.633 0.066 0.996<br>Orufi03g14720.1 0.79 1 0.21 0.03 1	1<br>Oglab01g19490.1 0.09 0.723 0.633 0.066 0.996<br>Oglab03g14060.1 0.79 1 0.21 0.03 1	1<br>Obart01g23310.1 0.09 0.723 0.633 0.066 0.996<br>Obart03g14050.1 0.79 1 0.21 0.03 1	1<br>Olong_003347-R2 0.09 0.723 0.633 0.047 0.996<br>Olong_009943-R1 0.79 1 0.21 0.03 1	1<br>Opunc01g23460.1 0.09 0.723 0.633 0.064 0.996<br>Opunc03g13590.1 0.79 1 0.21 0.002 0.545	1<br>Obrac01g17590.1 0.09 0.723 0.633 0.067 0.996<br>Obrac03g11330.1 0.79 1 0.21 0.03 1	0<br>Lperr05g03460.1 0.001 0.721 0.72 0.001 0.993	1<br>AT2G47580.1 0.775 1 0.225 0.008 1<br>AT3G16630.1 0.003 0.721 0.718 0.031 0.992
Osjap05g04120.2	1<br>Osjap05g04120.1 0.001 1 0.999 0.001 1<br>Osjap05g04120.2 0.001 1 0.999 0.001 1	1<br>Osind05g04070.1 0.008 1 0.992 0.001 1<br>Osind05g04070.2 0.008 1 0.992 0.001 1	1<br>Orufi05g03950.1 0.001 1 0.999 0.001 1<br>Orufi05g03950.2 0.001 1 0.999 0.001 1	1<br>Oglab05g03490.1 0.001 1 0.999 0.001 1<br>Oglab05g03490.2 0.001 1 0.999 0.001 1	1<br>Obart05g03830.1 0.001 1 0.999 0.001 1<br>Obart05g03830.2 0.001 1 0.999 0.001 1	1<br>Olong_016739-R1 0.073 1 0.927 0.001 1	1<br>Opunc05g03540.1 0.001 1 0.999 0.001 1<br>Opunc05g03540.2 0.001 1 0.999 0.001 1	1<br>Obrac05g02930.1 0.073 1 0.927 0.001 1	0	0
	1<br>Osjap01g27410.1 0.089 0.726 0.637 0.066 0.996<br>Osjap03g15140.5 0.747 1 0.253 0.061 1	1<br>Osind01g28630.1 0.089 0.726 0.637 0.066 0.996<br>Osind03g15230.1 0.792 1 0.208 0.03 1	1<br>Orufi01g26130.1 0.089 0.726 0.637 0.066 0.996<br>Orufi03g14720.1 0.792 1 0.208 0.03 1	1<br>Oglab01g19490.1 0.089 0.726 0.637 0.066 0.996<br>Oglab03g14060.1 0.792 1 0.208 0.03 1	1<br>Obart01g23310.1 0.089 0.726 0.637 0.066 0.996<br>Obart03g14050.1 0.792 1 0.208 0.03 1	1<br>Olong_003347-R2 0.089 0.726 0.637 0.047 0.996<br>Olong_009943-R1 0.792 1 0.208 0.03 1	1<br>Opunc01g23460.1 0.089 0.726 0.637 0.064 0.996<br>Opunc03g13590.1 0.792 1 0.208 0.002 0.545	1<br>Obrac01g17590.1 0.089 0.726 0.637 0.067 0.996<br>Obrac03g11330.1 0.792 1 0.208 0.03 1	0<br>Lperr05g03460.1 0.001 0.725 0.724 0.001 0.993	1<br>AT2G47580.1 0.778 1 0.222 0.008 1<br>AT3G16630.1 0.003 0.725 0.722 0.031 0.992
Osjap05g14570.1	1<br>Osjap05g14570.1 0.002 1 0.998 0.002 1	0	1<br>Orufi05g13730.1 0.002 1 0.998 0.002 1	0	0	0	1<br>Opunc05g10890.1 0.002 0.832 0.83 0.005 0.949	0	0	0
	1<br>Osjap01g31830.1 0.687 0.937 0.25 0.055 0.334<br>Osjap05g14540.1 0.034 0.683 0.649 0.309 1	1<br>Osind02g39040.1 0.616 0.948 0.332 0.127 0.415<br>Osind05g15150.1 0.002 0.615 0.613 0.009 1	1<br>Orufi09g08190.1 0.029 0.616 0.587 0.071 0.938<br>Orufi12g06770.1 0.626 0.954 0.328 0.154 0.383	0<br>Oglab02g28070.1 0.63 0.973 0.343 0.002 0.402<br>Oglab05g11780.1 0.344 0.611 0.267 0.172 1	1<br>Obart02g30540.1 0.63 0.973 0.343 0.002 0.402<br>Obart05g12690.1 0.002 0.594 0.592 0.005 0.973	1<br>Olong_008352-R1 0.034 0.611 0.577 0.004 1<br>Olong_023139-R1 0.626 0.937 0.311 0.01 0.432	1<br>Opunc04g20130.1 0.626 0.945 0.319 0.007 0.261<br>Opunc05g11350.1 0.032 0.615 0.583 0.147 0.998	0	0<br>Lperr05g09990.1 0.233 0.443 0.21 0.072 0.928	0<br>AT2G02650.1 0.282 0.573 0.291 0.562 1
Osjap05g23780.1	1<br>Osjap05g23780.1 0.001 1 0.999 0.001 1	0	1<br>Orufi05g22360.1 0.001 1 0.999 0.001 1	0	0	0	0	0	1<br>Lperr05g16920.1 0.029 0.999 0.97 0.015 0.977<br>Lperr05g16920.2 0.029 0.999 0.97 0.016 0.975	0
	1<br>Osjap01g39920.1 0.388 0.981 0.593 0.001 0.472<br>Osjap06g31930.1 0.017 0.374 0.357 0.147 0.902	0<br>Osind05g24290.1 0.349 1 0.651 0.113 1	1<br>Orufi01g38030.1 0.388 0.981 0.593 0.001 0.472<br>Orufi06g29370.1 0.017 0.374 0.357 0.147 0.902	1<br>Oglab05g19890.1 0.388 1 0.612 0.002 1<br>Oglab05g19900.1 0.001 0.374 0.373 0.003 0.894	1<br>Obart01g34670.1 0.388 0.981 0.593 0.001 0.465<br>Obart06g27420.1 0.017 0.374 0.357 0.141 0.901	1<br>Olong_004247-R1 0.017 0.374 0.357 0.147 0.902<br>Olong_020609-R1 0.388 0.981 0.593 0.001 0.483	1<br>Opunc05g18780.1 0.375 1 0.625 0.004 1<br>Opunc05g18790.1 0.001 0.374 0.373 0.003 0.895	0<br>Obrac01g26700.1 0.571 0.981 0.41 0.109 0.448<br>Obrac05g14330.1 0.084 0.374 0.29 0.041 0.873	1<br>Lperr01g29930.1 0.388 0.981 0.593 0.001 0.501<br>Lperr06g22970.1 0.017 0.374 0.357 0.164 0.892	0<br>AT2G32760.1 0.017 0.374 0.357 0.091 0.889
Osjap05g28230.1	1<br>Osjap05g28230.1 0.001 1 0.999 0.001 1	1<br>Osind05g28520.1 0.001 1 0.999 0.001 1	1<br>Orufi05g26870.1 0.001 1 0.999 0.001 1	1<br>Oglab05g23160.1 0.001 1 0.999 0.001 1	1<br>Obart05g25070.1 0.001 1 0.999 0.001 1	0	1<br>Opunc05g22640.1 0.001 1 0.999 0.001 1	0	1<br>Lperr01g22990.1 0.001 1 0.999 0.027 1	0
	1	1	1	1	1	1	1	1	1	1

	Osjap01g31140.1 0.006 0.432 0.426 0.093 0.997<br>Osjap01g31150.1 0.487 0.999 0.512 0.009 0.999	Osind01g32600.1 0.001 0.431 0.43 0.002 0.995<br>Osind01g32610.1 0.487 0.999 0.512 0.009 0.999	Orufi01g29880.1 0.001 0.432 0.431 0.075 0.997<br>Orufi01g29890.1 0.487 0.999 0.512 0.009 0.999	Oglab01g22740.1 0.027 0.432 0.405 0.061 0.996<br>Oglab01g22750.1 0.487 0.999 0.512 0.009 0.999	Obart01g26870.1 0.005 0.432 0.427 0.117 0.997<br>Obart01g26880.1 0.487 0.999 0.512 0.009 0.999	Olong_000367-R1 0.1 0.432 0.332 0.002 0.995<br>Olong_000368-R1 0.462 0.999 0.537 0.069 0.999	Opunc01g26820.1 0.047 0.432 0.385 0.002 0.996<br>Opunc01g26830.1 0.487 1 0.513 0.009 1	Obrac01g20400.1 0.044 0.432 0.388 0.004 0.996<br>Obrac05g17560.2 0.432 1 0.568 0.109 1	Lperr05g20850.2 0.491 1 0.509 0.002 1<br>Lperr05g20860.1 0.001 0.432 0.431 0.002 0.996	AT5G08690.1 0.011 0.431 0.42 0.034 0.995<br>AT5G51600.1 0.487 0.999 0.512 0.008 0.996
Osjap06g00390.1	1<br>Osjap06g00390.1 0.002 1 0.998 0.002 1	0	0	0	0	0	0	0	0	0
	1<br>Osjap06g28340.1 0.002 0.285 0.283 0.523 1<br>Osjap07g16770.1 0.342 0.998 0.656 0.078 0.992	0<br>Osind11g16460.1 0.207 0.992 0.785 0.03 0.982	1<br>Orufi06g00350.1 0.326 1 0.674 0.003 1<br>Orufi06g00360.1 0.002 0.285 0.283 0.007 1	1<br>Oglab06g00360.1 0.326 1 0.674 0.003 1<br>Oglab06g00370.1 0.002 0.285 0.283 0.007 1	1<br>Obart06g00340.1 0.326 1 0.674 0.003 1<br>Obart06g00350.1 0.002 0.289 0.287 0.007 0.987	1<br>Olong_019467-R3 0.002 0.289 0.287 0.054 0.988<br>Olong_019620-R1 0.377 1 0.623 0.061 0.953	1<br>Opunc06g00310.1 0.326 1 0.674 0.003 1<br>Opunc06g00330.1 0.002 0.289 0.287 0.057 0.987	1<br>Obrac06g00310.2 0.326 1 0.674 0.017 1<br>Obrac06g16850.1 0.002 0.285 0.283 0.515 1	1<br>Lperr06g00340.1 0.326 1 0.674 0.003 1<br>Lperr06g20160.1 0.002 0.285 0.283 0.508 0.994	0<br>AT5G47470.1 0.346 1 0.654 0.06 1
Osjap06g00940.1	1<br>Osjap06g00940.1 0.002 1 0.998 0.002 1	0	1<br>Orufi06g00860.1 0.118 1 0.882 0.021 1	1<br>Oglab06g00840.1 0.085 1 0.915 0.04 1	1<br>Obart06g00880.1 0.085 1 0.915 0.04 1	0	0	0	0	0
	1<br>Osjap06g00950.1 0.415 0.99 0.575 0.132 0.99<br>Osjap08g13790.1 0.083 0.327 0.244 0.005 1	1<br>Osind06g00910.1 0.411 1 0.589 0.068 1<br>Osind07g02910.1 0.091 0.325 0.234 0.012 1	1<br>Orufi06g00690.1 0.415 1 0.585 0.099 1<br>Orufi07g02700.1 0.091 0.325 0.234 0.012 1	1<br>Oglab06g00730.1 0.411 0.987 0.576 0.257 0.968<br>Oglab07g02750.1 0.1 0.325 0.225 0.007 1	1<br>Obart06g00690.1 0.415 1 0.585 0.099 1<br>Obart08g12830.1 0.104 0.324 0.22 0.012 0.988	1<br>Olong_006603-R1 0.04 0.324 0.284 0.034 0.987<br>Olong_022685-R1 0.415 1 0.585 0.074 1	1<br>Opunc06g00800.1 0.325 1 0.675 0.025 1<br>Opunc06g00830.1 0.097 0.325 0.228 0.003 1	1<br>Obrac02g00120.1 0.1 0.325 0.225 0.006 1<br>Obrac06g00660.1 0.411 1 0.589 0.034 0.999	0<br>Lperr01g05300.1 0.108 0.325 0.217 0.013 1<br>Lperr06g00740.1 0.415 0.998 0.583 0.073 0.997	0<br>AT1G20140.1 0.112 0.325 0.213 0.018 1
Osjap06g03080.1	1<br>Osjap06g03080.1 0.001 1 0.999 0.001 1	0	0	1<br>Oglab06g02870.1 0.001 1 0.999 0.001 1	1<br>Obart06g02710.1 0.001 1 0.999 0.03 1	1<br>Olong_013693-R1 0.043 1 0.957 0.069 1	0	0	0	0
	1<br>Osjap01g37620.1 0.041 0.738 0.697 0.099 0.998<br>Osjap04g34350.1 0.75 0.966 0.216 0.466 0.888	1<br>Osind03g45110.1 0.019 0.732 0.713 0.155 0.984<br>Osind04g33620.1 0.75 0.966 0.216 0.438 0.875	0<br>Orufi03g42420.1 0.041 0.732 0.691 0.602 0.997	1<br>Oglab02g27640.1 0.041 0.697 0.656 0.125 0.964<br>Oglab04g28300.1 0.75 0.966 0.216 0.466 0.888	1<br>Obart02g30110.1 0.041 0.697 0.656 0.125 0.964<br>Obart04g30240.1 0.75 0.966 0.216 0.466 0.888	1<br>Olong_001861-R1 0.75 0.966 0.216 0.381 0.87<br>Olong_002818-R1 0.041 0.694 0.653 0.04 0.956	1<br>Opunc04g27750.1 0.75 0.966 0.216 0.426 0.879<br>Opunc07g23960.4 0.041 0.697 0.656 0.072 0.993	1<br>Obrac04g21390.1 0.75 0.966 0.216 0.381 0.87<br>Obrac06g14370.1 0.056 0.689 0.633 0.049 0.963	1<br>Lperr04g03130.1 0.012 0.697 0.685 0.117 1<br>Lperr12g00960.1 0.75 0.966 0.216 0.389 0.871	0<br>AT2G37990.1 0.75 0.966 0.216 0.425 0.893
Osjap06g08210.1	1<br>Osjap06g08210.1 0.002 1 0.998 0.002 1	0	0	0	0	0	0	0	0	0
	1<br>Osjap02g37570.1 0.002 0.498 0.496 0.003 0.997<br>Osjap09g13400.1 0.506 0.998 0.492 0.207 0.995	1<br>Osind06g08300.1 0.002 0.5 0.498 0.003 1<br>Osind09g13560.1 0.506 0.998 0.492 0.121 0.995	1<br>Orufi06g07430.1 0.002 0.5 0.498 0.003 1<br>Orufi09g14020.1 0.506 0.998 0.492 0.121 0.995	1<br>Oglab06g07110.1 0.002 0.5 0.498 0.003 1<br>Oglab09g11950.1 0.506 0.998 0.492 0.121 0.995	0<br>Obart09g12990.1 0.497 0.997 0.5 0.055 0.978	1<br>Olong_012584-R1 0.521 0.997 0.476 0.003 0.995<br>Olong_014884-R1 0.002 0.5 0.498 0.003 1	1<br>Opunc06g06900.1 0.002 0.5 0.498 0.003 1<br>Opunc09g11410.1 0.52 1 0.48 0.009 1	0<br>Obrac09g07500.1 0.443 0.992 0.549 0.133 0.973	0<br>Lperr09g09380.2 0.44 0.998 0.558 0.032 0.995	0<br>AT5G16080.1 0.495 0.991 0.496 0.009 0.971
Osjap07g02600.5	1<br>Osjap07g02600.3 0.002 0.978 0.976 0.478 0.853<br>Osjap07g02600.4 0.002 1 0.998 0.555 1<br>Osjap07g02600.5 0.002 1 0.998 0.002 1	1<br>Osind07g02300.3 0.002 1 0.998 0.002 1<br>Osind07g02300.4 0.002 1 0.998 0.002 1	0	0	0	0	0	0	0	0
	1<br>Osjap01g07190.1 0.002 0.254 0.252 0.692 0.935<br>Osjap07g02840.1 0.298 0.976 0.678 0.411 0.823	1<br>Osind01g07120.1 0.002 0.254 0.252 0.692 0.935<br>Osind07g02530.1 0.298 0.976 0.678 0.411 0.823	1<br>Orufi07g02320.1 0.298 0.976 0.678 0.411 0.823<br>Orufi12g02700.1 0.007 0.259 0.252 0.518 0.746	1<br>Oglab01g05790.1 0.002 0.254 0.252 0.692 0.935<br>Oglab07g02390.2 0.298 0.976 0.678 0.41 0.823	1<br>Obart01g06210.1 0.002 0.254 0.252 0.692 0.935<br>Obart07g02450.1 0.298 0.976 0.678 0.377 0.809	1<br>Olong_003501-R1 0.002 0.254 0.252 0.634 0.923<br>Olong_009970-R1 0.298 0.976 0.678 0.411 0.823	1<br>Opunc01g05980.1 0.002 0.254 0.252 0.689 0.934<br>Opunc07g02500.8 0.298 0.976 0.678 0.472 0.79	1<br>Obrac05g04770.1 0.002 0.254 0.252 0.594 0.923<br>Obrac07g01520.1 0.298 0.99 0.692 0.386 0.893	1<br>Lperr07g02310.1 0.298 0.976 0.678 0.431 0.845<br>Lperr11g02330.1 0.007 0.257 0.25 0.682 0.933	0<br>AT4G36380.1 0.002 0.308 0.306 0.691 0.966
Osjap07g04240.1	1<br>Osjap07g04240.1 0.001 1 0.999 0.001 1	0	0	0	0	0	0	0	0	0
	1<br>Osjap04g16300.1 0.376 0.999 0.623 0.003 0.997	1<br>Osind06g16400.1 0.001 0.3 0.299 0.725 0.998	1<br>Orufi07g03520.1 0.376 1 0.624 0.002 1	1<br>Oglab06g10580.1 0.002 0.299 0.297 0.504 0.995	1<br>Obart05g11940.1 0.005 0.276 0.271 0.029 1	1<br>Olong_011562-R1 0.376 1 0.624 0.002 1	1<br>Opunc07g03760.1 0.376 1 0.624 0.002 1	0<br>Obrac07g02280.1 0.376 1 0.624 0.002 1	1<br>Lperr05g15860.1 0.001 0.289 0.288 0.292 0.978	0<br>AT5G21990.1 0.375 0.999 0.624 0.007 0.998

	Osjap12g10970.1 0.045 0.302 0.257 0.005 1	Osind07g03740.1 0.376 1 0.624 0.002 1	Orufi10g08890.2 0.001 0.302 0.301 0.542 1	Oglab07g03710.1 0.376 1 0.624 0.002 1	Obart07g03860.1 0.376 1 0.624 0.16 1	Olong_023745-R3 0.001 0.241 0.24 0.385 0.93	Opunc12g18050.2 0.001 0.302 0.301 0.004 1		Lperr07g03350.1 0.376 1 0.624 0.002 1	
Osjap07g06350.1	1<br>Osjap07g06350.1 0.003 1 0.997 0.003 1	0	0	0	0	0	0	0	0	0
	1<br>Osjap07g09830.1 0.009 0.597 0.588 0.382 0.959<br>Osjap08g03480.1 0.704 0.948 0.244 0.32 0.424	1<br>Osind05g05600.1 0.687 0.933 0.246 0.328 0.433<br>Osind07g05530.1 0.003 0.655 0.652 0.006 0.989	1<br>Orufi07g05450.1 0.003 0.328 0.325 0.007 0.979<br>Orufi07g05460.1 0.33 0.942 0.612 0.07 0.989	1<br>Oglab04g03880.1 0.672 0.93 0.258 0.32 0.43<br>Oglab07g08200.1 0.009 0.597 0.588 0.332 0.955	1<br>Obart07g08500.1 0.009 0.597 0.588 0.33 0.955<br>Obart09g03030.1 0.687 0.933 0.246 0.328 0.433	1<br>Olong_015067-R1 0.687 0.933 0.246 0.31 0.41<br>Olong_016234-R1 0.014 0.672 0.658 0.197 0.676	1<br>Opunc06g23810.1 0.687 0.933 0.246 0.317 0.419<br>Opunc07g05240.1 0.003 0.583 0.58 0.273 0.967	1<br>Obrac07g03440.1 0.69 0.98 0.29 0.346 0.515<br>Obrac07g03880.2 0.003 0.583 0.58 0.46 0.946	1<br>Lperr07g04680.1 0.006 0.583 0.577 0.356 0.988<br>Lperr08g03240.1 0.704 0.948 0.244 0.351 0.463	0<br>AT5G63020.1 0.467 0.945 0.478 0.226 0.421
Osjap07g29350.1	1<br>Osjap07g29350.1 0.002 1 0.998 0.002 1	1<br>Osind07g26050.1 0.013 1 0.987 0.157 1	1<br>Orufi07g26420.1 0.106 1 0.894 0.002 1	0	0	1<br>Olong_024157-R2 0.011 0.989 0.978 0.044 0.998	0	0	0	0
	1<br>Osjap03g18810.1 0.719 1 0.281 0.007 1<br>Osjap07g29380.2 0.013 0.629 0.616 0.158 0.994	1<br>Osind01g39050.1 0.004 0.637 0.633 0.07 0.971<br>Osind08g27680.1 0.723 0.996 0.273 0.289 0.986	1<br>Orufi03g18050.1 0.717 1 0.283 0.437 1<br>Orufi07g26440.5 0.013 0.629 0.616 0.164 0.994	1<br>Oglab06g19010.1 0.119 0.605 0.486 0.058 0.967<br>Oglab07g22020.1 0.635 1 0.365 0.021 1	1<br>Obart06g20980.1 0.028 0.605 0.577 0.043 0.968<br>Obart07g25530.1 0.635 1 0.365 0.021 1	1<br>Olong_015865-R1 0.717 1 0.283 0.364 1<br>Olong_024157-R3 0.011 0.715 0.704 0.056 0.975	1<br>Opunc07g23940.1 0.719 1 0.281 0.007 1<br>Opunc07g23960.1 0.011 0.626 0.615 0.053 0.995	1<br>Obrac06g14370.1 0.026 0.635 0.609 0.05 0.99<br>Obrac07g16760.2 0.708 1 0.292 0.101 1	0<br>Lperr07g22320.1 0.719 1 0.281 0.007 1<br>Lperr11g01590.1 0.149 0.57 0.421 0.083 0.878	0<br>AT5G18100.1 0.711 0.998 0.287 0.018 0.957
Osjap08g06650.1	1<br>Osjap08g06650.1 0.002 1 0.998 0.002 1	1<br>Osind08g06790.1 0.002 1 0.998 0.002 1	1<br>Orufi08g06840.1 0.002 1 0.998 0.002 1	1<br>Oglab08g05640.1 0.002 1 0.998 0.002 1	1<br>Obart08g06070.1 0.002 1 0.998 0.002 1	0	1<br>Opunc08g05850.1 0.047 1 0.953 0.068 1	0	0	0
	1<br>Osjap04g21220.1 0.047 0.275 0.228 0.21 0.936<br>Osjap09g20870.1 0.359 0.984 0.625 0.208 0.966	1<br>Osind04g20250.1 0.047 0.275 0.228 0.198 0.94<br>Osind09g20960.1 0.359 0.984 0.625 0.208 0.966	1<br>Orufi04g19250.2 0.047 0.275 0.228 0.21 0.936<br>Orufi09g21960.1 0.359 0.984 0.625 0.2 0.965	1<br>Oglab02g26360.1 0.348 0.996 0.648 0.01 0.994<br>Oglab04g16180.1 0.047 0.275 0.228 0.198 0.94	1<br>Obart02g28440.1 0.348 0.996 0.648 0.093 0.994<br>Obart04g17770.1 0.047 0.275 0.228 0.198 0.94	1<br>Olong_008030-R1 0.047 0.275 0.228 0.232 0.937<br>Olong_008031-R2 0.277 1 0.723 0.106 1	1<br>Opunc04g15550.1 0.047 0.275 0.228 0.206 0.938<br>Opunc04g18740.1 0.295 0.984 0.689 0.088 0.994	1<br>Obrac08g04780.1 0.277 1 0.723 0.098 1<br>Obrac08g04800.1 0.002 0.275 0.273 0.006 0.943	1<br>Lperr08g05690.1 0.277 1 0.723 0.1 1<br>Lperr08g05700.1 0.002 0.275 0.273 0.006 0.943	1<br>AT1G05310.1 0.33 0.982 0.652 0.183 0.99<br>AT4G30800.1 0.047 0.275 0.228 0.208 0.943
Osjap08g10540.1	1<br>Osjap08g10540.1 0.001 1 0.999 0.001 1	1<br>Osind08g11830.1 0.001 1 0.999 0.006 1	1<br>Orufi08g11060.1 0.001 1 0.999 0.001 1	0	0	0	0	0	0	0
	1<br>Osjap08g10560.1 0.001 0.248 0.247 0.02 0.683<br>Osjap10g16570.1 0.315 0.993 0.678 0.229 0.992	1<br>Osind05g10830.1 0.309 0.995 0.686 0.057 0.966<br>Osind07g12020.1 0.001 0.247 0.246 0.003 0.758	1<br>Orufi05g09510.1 0.309 0.995 0.686 0.066 0.964<br>Orufi08g11080.2 0.001 0.248 0.247 0.021 0.71	1<br>Oglab02g32050.1 0.248 0.967 0.719 0.001 0.99<br>Oglab08g09010.1 0.001 0.248 0.247 0.028 0.635	1<br>Obart02g04890.1 0.266 0.986 0.72 0.148 0.991<br>Obart07g11970.1 0.001 0.248 0.247 0.003 0.764	1<br>Olong_015028-R1 0.001 0.248 0.247 0.032 0.684<br>Olong_022719-R1 0.315 0.993 0.678 0.238 0.966	1<br>Opunc02g16130.1 0.315 0.995 0.68 0.224 1<br>Opunc08g08990.2 0.001 0.248 0.247 0.018 0.873	0<br>Obrac04g05110.2 0.003 0.248 0.245 0.025 0.656	0<br>Lperr07g03020.1 0.688 0.978 0.29 0.003 0.899<br>Lperr10g01740.2 0.01 0.273 0.263 0.042 0.596	0<br>AT4G15880.1 0.734 0.969 0.235 0.495 0.99
Osjap08g22730.1	1<br>Osjap08g22730.1 0.001 1 0.999 0.001 1	1<br>Osind08g24940.1 0.13 0.987 0.857 0.09 0.999	1<br>Orufi08g23770.1 0.02 1 0.98 0.019 1<br>Orufi08g23770.3 0.002 1 0.998 0.018 1	0	0	0	0	0	0	0
	1<br>Osjap04g34840.1 0.02 0.374 0.354 0.031 0.992<br>Osjap08g22710.1 0.43 1 0.57 0.026 1	1<br>Osind04g34100.1 0.001 0.374 0.373 0.002 0.992<br>Osind08g24940.2 0.437 1 0.563 0.006 1	1<br>Orufi04g32420.1 0.02 0.374 0.354 0.031 0.992<br>Orufi08g23750.1 0.414 1 0.586 0.002 1	0<br>Oglab04g28790.1 0.001 0.374 0.373 0.002 0.992	1<br>Obart04g30710.1 0.001 0.374 0.373 0.002 0.992<br>Obart08g21280.1 0.411 1 0.589 0.002 1	1<br>Olong_012750-R1 0.002 0.367 0.365 0.085 0.881<br>Olong_024729-R1 0.465 0.999 0.534 0.017 1	1<br>Opunc04g28230.1 0.004 0.369 0.365 0.008 0.979<br>Opunc08g19370.1 0.39 0.989 0.599 0.003 0.997	1<br>Obrac04g21850.1 0.001 0.364 0.363 0.008 0.992<br>Obrac08g14190.1 0.405 1 0.595 0.002 1	1<br>Lperr04g26140.1 0.015 0.365 0.35 0.032 0.96<br>Lperr08g18050.1 0.41 1 0.59 0.003 1	0<br>AT2G02020.1 0.001 0.36 0.359 0.007 0.994
Osjap09g00070.1	1<br>Osjap09g00070.1 0.003 1 0.997 0.003 1	0	1<br>Orufi09g00140.1 0.003 1 0.997 0.003 1<br>Orufi09g13330.1 0.175 0.984 0.809 0.402 0.983	1<br>Oglab09g00090.1 0.003 1 0.997 0.003 1<br>Oglab09g11280.1 0.175 0.984 0.809 0.365 0.982	1<br>Obart09g00120.1 0.003 1 0.997 0.003 1<br>Obart09g12390.1 0.175 0.984 0.809 0.365 0.982	0	1<br>Opunc09g00070.1 0.003 1 0.997 0.003 1<br>Opunc09g10830.1 0.175 0.984 0.809 0.37 0.982<br>Opunc10g04500.1 0.003 1 0.997 0.003 1	0	0	0
	1<br>Osjap02g38200.1 0.35 1 0.65 0.434 1	1<br>Osind02g37760.1 0.35 1 0.65 0.439 1	1<br>Orufi02g35630.1 0.35 1 0.65 0.435 1	1<br>Oglab02g31460.1 0.35 1 0.65 0.396 0.929	1<br>Obart02g34090.1 0.35 1 0.65 0.434 1	1<br>Olong_015651-R1 0.35 1 0.65 0.434 1	0<br>Opunc09g00900.1 0.35 1 0.65 0.258 1	0<br>Obrac02g24970.1 0.347 1 0.653 0.432 1	0<br>Lperr09g00310.1 0.306 0.984 0.678 0.437 0.987	0<br>AT4G00238.1 0.347 0.803 0.456 0.409 0.835

	Osjap09g04150.1 0.003 0.294 0.291 0.009 0.991	Osind09g04190.1 0.003 0.294 0.291 0.009 0.991	Orufi09g04570.1 0.003 0.294 0.291 0.009 0.991	Oglab09g03840.1 0.003 0.347 0.344 0.007 0.839	Obart09g03960.1 0.009 0.35 0.341 0.604 0.939	Olong_025390-R1 0.003 0.35 0.347 0.008 0.87				
Osjap09g03990.5	1<br>Osjap09g03990.1 0.004 1 0.996 0.543 1<br>Osjap09g03990.2 0.004 1 0.996 0.508 1<br>Osjap09g03990.5 0.004 1 0.996 0.004 1	0	1<br>Orufi09g04390.1 0.004 1 0.996 0.6 1<br>Orufi09g04390.2 0.004 1 0.996 0.559 1	0	0	0	0	0	0	0
	1<br>Osjap05g14220.1 0.507 1 0.493 0.468 0.818<br>Osjap09g14680.2 0.004 0.414 0.41 0.77 0.967	1<br>Osind09g04090.1 0.674 1 0.326 0.03 0.582<br>Osind09g04110.1 0.004 0.581 0.577 0.704 1	1<br>Orufi09g08210.2 0.507 1 0.493 0.388 0.791<br>Orufi09g15320.1 0.004 0.414 0.41 0.75 0.965	1<br>Oglab08g18120.2 0.731 1 0.269 0.391 0.77<br>Oglab09g03780.1 0.004 0.581 0.577 0.714 1	1<br>Obart07g20140.1 0.709 1 0.291 0.385 0.717<br>Obart09g03930.1 0.004 0.581 0.577 0.714 1	1<br>Olong_001796-R2 0.004 0.626 0.622 0.703 0.995<br>Olong_005713-R1 0.705 1 0.295 0.522 0.912	1<br>Opunc07g19040.1 0.709 1 0.291 0.384 0.717<br>Opunc09g03510.2 0.004 0.581 0.577 0.685 1	1<br>Obrac07g12720.1 0.709 1 0.291 0.276 0.667<br>Obrac09g02030.2 0.004 0.581 0.577 0.676 1	1<br>Lperr07g17470.1 0.709 1 0.291 0.382 0.716<br>Lperr09g02810.2 0.004 0.581 0.577 0.737 1	1<br>AT1G79610.1 0.004 0.626 0.622 0.735 0.974<br>AT5G07100.1 0.639 1 0.361 0.285 0.864
Osjap09g20460.1	1<br>Osjap09g20460.1 0.001 1 0.999 0.001 1	1<br>Osind09g20560.1 0.001 0.986 0.985 0.001 0.998	1<br>Orufi09g21510.1 0.001 0.967 0.966 0.001 0.999	1<br>Oglab09g18200.1 0.001 0.999 0.998 0.001 0.999	1<br>Obart09g19980.1 0.062 0.999 0.937 0.165 0.999<br>Obart09g19980.2 0.001 0.999 0.998 0.001 0.999	1<br>Olong_014589-R1 0.108 0.974 0.866 0.018 0.991<br>Olong_014589-R2 0.108 0.974 0.866 0.018 0.991	0	0	0	0
	1<br>Osjap02g21530.2 0.022 0.584 0.562 0.096 0.943<br>Osjap09g20430.1 0.624 1 0.376 0.085 1	1<br>Osind02g21430.2 0.022 0.584 0.562 0.096 0.943<br>Osind09g20550.1 0.624 1 0.376 0.032 1	1<br>Orufi02g19810.2 0.022 0.584 0.562 0.096 0.943<br>Orufi09g21500.1 0.624 1 0.376 0.085 1	1<br>Oglab02g17850.1 0.022 0.584 0.562 0.095 0.914<br>Oglab09g18170.1 0.624 1 0.376 0.079 1	1<br>Obart04g25370.1 0.044 0.592 0.548 0.176 0.967<br>Obart09g19950.1 0.624 1 0.376 0.079 1	1<br>Olong_014116-R1 0.059 0.592 0.533 0.069 0.957<br>Olong_027417-R1 0.626 0.991 0.365 0.135 0.745	1<br>Opunc09g18280.1 0.619 0.998 0.379 0.058 0.951<br>Opunc09g18290.2 0.001 0.592 0.591 0.001 0.97	1<br>Obrac09g13140.3 0.624 0.986 0.362 0.049 0.806<br>Obrac09g13160.3 0.001 0.593 0.592 0.001 0.974	1<br>Lperr09g15890.1 0.623 0.996 0.373 0.048 0.957<br>Lperr09g15920.1 0.001 0.592 0.591 0.001 0.957	0<br>AT1G18160.1 0.02 0.592 0.572 0.064 0.958
Osjap10g15240.1	1<br>Osjap10g15240.1 0.001 1 0.999 0.001 1	0	0	0	0	0	0	0	0	0
	1<br>Osjap03g03010.1 0.01 0.376 0.366 0.001 0.999<br>Osjap03g36880.2 0.49 0.996 0.506 0.068 0.878	1<br>Osind10g12350.1 0.415 1 0.585 0.001 1<br>Osind10g12360.1 0.001 0.376 0.375 0.001 0.999	1<br>Orufi10g13620.2 0.415 1 0.585 0.001 1<br>Orufi10g13630.1 0.001 0.376 0.375 0.001 0.999	1<br>Oglab10g12600.4 0.415 1 0.585 0.001 1<br>Oglab10g12610.1 0.001 0.376 0.375 0.001 0.999	1<br>Obart10g12900.2 0.415 1 0.585 0.036 1<br>Obart10g12910.1 0.001 0.376 0.375 0.001 0.982	1<br>Olong_005999-R1 0.004 0.376 0.372 0.119 0.999<br>Olong_018868-R1 0.469 0.994 0.525 0.001 1	1<br>Opunc10g11270.1 0.415 1 0.585 0.001 1<br>Opunc10g11280.1 0.001 0.376 0.375 0.094 0.976	1<br>Obrac10g07770.1 0.489 1 0.511 0.004 1<br>Obrac10g07780.1 0.009 0.377 0.368 0.023 0.971	1<br>Lperr10g09460.1 0.415 1 0.585 0.001 1<br>Lperr10g09470.1 0.001 0.376 0.375 0.001 0.999	1<br>AT2G35920.1 0.461 0.998 0.537 0.039 0.998<br>AT4G28600.1 0.003 0.376 0.373 0.014 0.999
Osjap11g00600.1	1<br>Osjap11g00600.1 0.002 1 0.998 0.002 1<br>Osjap12g00550.1 0.002 0.998 0.996 0.002 1	1<br>Osind12g00790.1 0.002 0.998 0.996 0.002 1	1<br>Orufi11g00440.1 0.002 1 0.998 0.002 1	1<br>Oglab11g00570.1 0.106 1 0.894 0.051 1<br>Oglab12g00270.1 0.002 0.801 0.799 0.003 0.995	1<br>Obart11g00510.1 0.106 1 0.894 0.045 1<br>Obart12g00320.1 0.002 0.998 0.996 0.003 1	0	0	0	0	0
	1<br>Osjap08g03600.1 0.748 0.984 0.236 0.201 0.274<br>Osjap10g06830.2 0.087 0.719 0.632 0.265 0.998	1<br>Osind05g03780.1 0.748 0.984 0.236 0.16 0.63<br>Osind10g04960.1 0.063 0.719 0.656 0.31 0.998	1<br>Orufi08g03710.3 0.748 0.984 0.236 0.289 0.394<br>Orufi12g00580.1 0.002 0.703 0.701 0.002 0.881	0<br>Oglab10g05540.1 0.087 0.719 0.632 0.32 0.998	1<br>Obart08g03340.1 0.748 0.984 0.236 0.191 0.268<br>Obart12g18380.1 0.069 0.717 0.648 0.137 0.977	1<br>Olong_026176-R1 0.083 0.717 0.634 0.114 0.997<br>Olong_027703-R1 0.748 0.984 0.236 0.192 0.274	1<br>Opunc08g03130.1 0.748 0.984 0.236 0.206 0.282<br>Opunc12g00560.1 0.002 0.724 0.722 0.003 0.995	1<br>Obrac08g02840.1 0.748 0.984 0.236 0.201 0.274<br>Obrac12g00030.1 0.004 0.624 0.62 0.412 0.991	1<br>Lperr12g00550.1 0.748 0.986 0.238 0.194 0.271<br>Lperr12g00630.2 0.033 0.719 0.686 0.031 0.983	0<br>AT2G40030.1 0.738 0.982 0.244 0.168 0.234
Osjap11g01220.1	1<br>Osjap11g01220.1 0.001 1 0.999 0.001 1<br>Osjap12g01140.1 0.001 1 0.999 0.001 1	1<br>Osind11g00690.1 0.001 0.955 0.954 0.001 0.999<br>Osind12g01290.1 0.001 1 0.999 0.001 1	1<br>Orufi11g01000.1 0.001 1 0.999 0.001 1<br>Orufi12g01130.1 0.001 1 0.999 0.001 1	1<br>Oglab12g00800.1 0.001 1 0.999 0.001 1	1<br>Obart11g01060.1 0.001 1 0.999 0.001 1	0	1<br>Opunc12g00940.1 0.001 1 0.999 0.001 1	0	0	0
	1<br>Osjap03g13100.1 0.489 0.982 0.493 0.097 0.622<br>Osjap07g30390.1 0.009 0.433 0.424 0.013 0.944	1<br>Osind03g12930.1 0.489 0.982 0.493 0.121 0.633<br>Osind07g27070.1 0.007 0.433 0.426 0.006 0.946	1<br>Orufi03g17220.1 0.496 1 0.504 0.027 1<br>Orufi07g27420.1 0.009 0.433 0.424 0.013 0.946	1<br>Oglab03g12120.1 0.489 0.982 0.493 0.121 0.633<br>Oglab07g22980.1 0.007 0.433 0.426 0.007 0.94	1<br>Obart03g12120.1 0.489 0.982 0.493 0.121 0.633<br>Obart12g00790.1 0.001 0.433 0.432 0.002 0.949	1<br>Olong_007715-R1 0.492 1 0.508 0.007 1<br>Olong_008211-R1 0.007 0.433 0.426 0.006 0.946	1<br>Opunc03g11680.1 0.489 1 0.511 0.148 1<br>Opunc11g00920.1 0.001 0.433 0.432 0.142 0.951	1<br>Obrac03g09720.2 0.489 1 0.511 0.142 1<br>Obrac12g00780.1 0.001 0.433 0.432 0.11 0.953	1<br>Lperr03g10720.1 0.489 0.993 0.504 0.148 0.952<br>Lperr11g00920.1 0.001 0.433 0.432 0.002 0.949	1<br>AT2G34940.1 0.001 0.433 0.432 0.002 0.955<br>AT3G46740.1 0.492 1 0.508 0.161 1
Osjap11g03190.1	1<br>Osjap11g03190.1 0.002 1 0.998 0.002 1	0	0	0	0	0	0	0	0	0

	1<br>Osjap11g03620.1 0.003 0.23 0.227 0.198 0.811<br>Osjap12g03260.1 0.372 0.995 0.623 0.046 1	1<br>Osind11g02550.1 0.372 1 0.628 0.045 1<br>Osind11g03030.1 0.003 0.23 0.227 0.016 0.766	1<br>Orufi11g02770.1 0.372 1 0.628 0.196 1<br>Orufi11g03240.1 0.003 0.23 0.227 0.016 0.768	1<br>Oglab11g02800.1 0.002 0.313 0.311 0.005 0.959<br>Oglab11g02820.1 0.332 1 0.668 0.002 1	1<br>Obart11g02890.1 0.002 0.313 0.311 0.005 0.962<br>Obart11g02910.1 0.332 1 0.668 0.002 1	0<br>Olong_004426-R1 0.372 0.995 0.623 0.042 1	1<br>Opunc11g02560.1 0.002 0.372 0.37 0.002 0.552<br>Opunc12g02460.1 0.372 0.995 0.623 0.048 1	0<br>Obrac11g01720.1 0.576 0.995 0.419 0.196 1	0<br>Lperr11g02460.1 0.381 0.995 0.614 0.075 1	0<br>AT1G51450.1 0.512 0.968 0.456 0.444 0.972
Osjap11g03220.1	1<br>Osjap11g03220.1 0.001 1 0.999 0.001 1	0	0	0	0	0	0	0	0	0
	1<br>Osjap12g03280.1 0.432 1 0.568 0.047 1<br>Osjap12g03300.1 0.001 0.302 0.301 0.003 0.991	1<br>Osind11g02580.2 0.424 1 0.576 0.032 1<br>Osind12g03310.1 0.001 0.326 0.325 0.003 0.964	1<br>Orufi11g02800.2 0.424 1 0.576 0.032 1<br>Orufi11g02820.1 0.001 0.302 0.301 0.219 0.99	1<br>Oglab11g02850.3 0.424 1 0.576 0.032 1<br>Oglab11g02870.1 0.001 0.343 0.342 0.003 0.963	1<br>Obart11g02940.2 0.424 1 0.576 0.032 1<br>Obart11g02960.2 0.001 0.35 0.349 0.167 0.926	1<br>Olong_016306-R1 0.001 0.35 0.349 0.117 0.924<br>Olong_016308-R1 0.43 1 0.57 0.041 1	1<br>Opunc11g02640.1 0.001 0.343 0.342 0.003 1<br>Opunc12g02490.1 0.43 1 0.57 0.04 1	1<br>Obrac11g01750.1 0.001 0.34 0.339 0.003 0.994<br>Obrac12g02140.1 0.432 1 0.568 0.044 1	1<br>Lperr11g02500.4 0.001 0.322 0.321 0.003 0.938<br>Lperr12g02610.1 0.43 1 0.57 0.041 1	1<br>AT2G26460.1 0.424 0.98 0.556 0.029 0.969<br>AT3G22880.1 0.001 0.326 0.325 0.003 0.977
Osjap11g21440.1	1<br>Osjap11g21440.1 0.001 1 0.999 0.001 1	1<br>Osind04g04060.1 0.001 0.934 0.933 0.072 0.988	1<br>Orufi11g19860.1 0.003 1 0.997 0.052 1	1<br>Oglab11g16480.1 0.003 1 0.997 0.052 1	1<br>Obart11g18650.1 0.001 1 0.999 0.073 1	1<br>Olong_013913-R1 0.006 0.965 0.959 0.001 0.986	0	0	0	0
	1<br>Osjap03g43210.1 0.717 0.987 0.27 0.278 0.99<br>Osjap10g09150.2 0.008 0.716 0.708 0.047 0.738	1<br>Osind03g44240.1 0.717 0.987 0.27 0.017 0.988<br>Osind10g06650.1 0.008 0.716 0.708 0.008 0.713	1<br>Orufi03g41640.1 0.717 0.987 0.27 0.018 0.987<br>Orufi10g08130.1 0.008 0.716 0.708 0.047 0.737	1<br>Oglab03g37880.1 0.717 0.987 0.27 0.278 0.99<br>Oglab11g20100.1 0.02 0.711 0.691 0.029 0.947	1<br>Obart03g02940.1 0.739 0.986 0.247 0.118 0.932<br>Obart10g07740.1 0.008 0.716 0.708 0.047 0.737	1<br>Olong_001283-R1 0.002 0.674 0.672 0.014 0.844<br>Olong_011979-R1 0.739 0.987 0.248 0.108 0.954	1<br>Opunc03g37060.1 0.717 0.987 0.27 0.151 0.989<br>Opunc05g18050.1 0.047 0.702 0.655 0.06 0.817	1<br>Obrac03g30000.2 0.705 0.979 0.274 0.031 0.937<br>Obrac05g13790.3 0.029 0.702 0.673 0.127 0.872	1<br>Lperr03g33980.1 0.717 0.974 0.257 0.008 0.411<br>Lperr11g13500.1 0.043 0.677 0.634 0.047 0.638	0<br>AT3G12620.1 0.719 0.984 0.265 0.047 0.964
Osjap11g25180.1	1<br>Osjap11g25180.1 0.001 1 0.999 0.001 1	0	0	0	0	0	0	0	0	0
	1<br>Osjap03g12940.2 0.75 0.996 0.246 0.002 0.941<br>Osjap11g25230.1 0.001 0.715 0.714 0.001 0.936	1<br>Osind03g12720.1 0.75 0.999 0.249 0.032 0.989<br>Osind11g17650.1 0.001 0.75 0.749 0.001 0.989	1<br>Orufi03g12330.1 0.75 0.996 0.246 0.029 0.947<br>Orufi11g23320.1 0.001 0.75 0.749 0.001 0.989	1<br>Oglab03g12000.1 0.75 0.996 0.246 0.036 0.944<br>Oglab11g18660.1 0.001 0.75 0.749 0.001 0.989	1<br>Obart03g11990.1 0.75 0.999 0.249 0.032 0.989<br>Obart11g21600.1 0.005 0.725 0.72 0.026 0.955	1<br>Olong_007726-R1 0.763 0.999 0.236 0.153 0.981<br>Olong_026881-R1 0.001 0.73 0.729 0.002 0.98	1<br>Opunc11g17740.1 0.001 0.742 0.741 0.003 0.975<br>Opunc11g18020.1 0.765 1 0.235 0.004 1	1<br>Obrac11g12680.1 0.775 1 0.225 0.081 1<br>Obrac11g12750.1 0.006 0.767 0.761 0.004 0.999	1<br>Lperr03g10630.5 0.765 0.999 0.234 0.004 0.967<br>Lperr11g18000.1 0.001 0.749 0.748 0.001 1	0<br>AT2G29260.1 0.755 0.999 0.244 0.121 0.975
Osjap12g01140.1	1<br>Osjap11g01220.1 0.001 1 0.999 0.001 1<br>Osjap12g01140.1 0.001 1 0.999 0.001 1	1<br>Osind11g00690.1 0.001 0.953 0.952 0.001 0.999<br>Osind12g01290.1 0.001 1 0.999 0.001 1	1<br>Orufi11g01000.1 0.001 1 0.999 0.001 1<br>Orufi12g01130.1 0.001 1 0.999 0.001 1	1<br>Oglab12g00800.1 0.001 1 0.999 0.001 1	1<br>Obart11g01060.1 0.001 1 0.999 0.001 1	0	1<br>Opunc12g00940.1 0.001 1 0.999 0.001 1	0	0	0
	1<br>Osjap03g13100.1 0.511 0.981 0.47 0.097 0.622<br>Osjap07g30390.1 0.01 0.373 0.363 0.013 0.798	1<br>Osind03g12930.1 0.511 0.981 0.47 0.121 0.633<br>Osind07g27070.1 0.007 0.377 0.37 0.006 0.783	1<br>Orufi03g17220.1 0.517 1 0.483 0.027 1<br>Orufi07g27420.1 0.01 0.377 0.367 0.013 0.783	1<br>Oglab03g12120.1 0.511 0.981 0.47 0.121 0.633<br>Oglab07g22980.1 0.007 0.373 0.366 0.007 0.783	1<br>Obart03g12120.1 0.511 0.981 0.47 0.121 0.633<br>Obart10g06750.1 0.021 0.409 0.388 0.151 0.885	1<br>Olong_007715-R1 0.514 1 0.486 0.007 1<br>Olong_008211-R1 0.007 0.377 0.37 0.006 0.783	1<br>Opunc03g11680.1 0.511 1 0.489 0.148 1<br>Opunc11g00920.1 0.001 0.377 0.376 0.142 0.816	1<br>Obrac03g09720.2 0.511 1 0.489 0.142 1<br>Obrac10g04150.1 0.02 0.405 0.385 0.036 0.868	1<br>Lperr03g10720.1 0.511 0.993 0.482 0.148 0.952<br>Lperr11g00920.1 0.001 0.377 0.376 0.002 0.792	1<br>AT2G34940.1 0.001 0.377 0.376 0.002 0.793<br>AT3G46740.1 0.514 1 0.486 0.161 1
Osjap12g12320.1	1<br>Osjap12g12320.1 0.001 1 0.999 0.001 1	1<br>Osind12g09530.1 0.001 1 0.999 0.001 1	1<br>Orufi12g10460.1 0.001 1 0.999 0.001 1	1<br>Oglab12g09470.1 0.001 1 0.999 0.001 1	1<br>Obart12g09410.1 0.001 1 0.999 0.001 1	0	0	0	0	0
	1<br>Osjap01g44780.2 0.363 0.998 0.635 0.086 0.994<br>Osjap03g07600.1 0.001 0.323 0.322 0.004 0.989	1<br>Osind01g46040.1 0.363 0.998 0.635 0.142 0.995<br>Osind03g07670.1 0.001 0.323 0.322 0.004 0.989	1<br>Orufi01g42650.3 0.363 0.998 0.635 0.186 0.995<br>Orufi03g07180.1 0.001 0.323 0.322 0.004 0.989	1<br>Oglab01g34040.1 0.425 0.998 0.573 0.203 0.995<br>Oglab03g07280.1 0.001 0.323 0.322 0.004 0.989	1<br>Obart01g39260.3 0.388 0.998 0.61 0.039 0.994<br>Obart03g07210.1 0.001 0.323 0.322 0.004 0.989	1<br>Olong_021912-R2 0.373 1 0.627 0.008 1<br>Olong_021913-R1 0.001 0.321 0.32 0.004 0.988	1<br>Opunc02g11250.1 0.001 0.323 0.322 0.372 0.993<br>Opunc12g08570.1 0.39 1 0.61 0.211 1	1<br>Obrac03g05880.1 0.001 0.323 0.322 0.004 0.992<br>Obrac12g06500.1 0.395 1 0.605 0.103 1	1<br>Lperr03g06600.1 0.001 0.323 0.322 0.004 0.992<br>Lperr12g08310.1 0.399 0.999 0.6 0.002 0.998	1<br>AT3G49880.1 0.426 0.995 0.569 0.032 0.987<br>AT4G34670.1 0.001 0.315 0.314 0.004 0.966
Osjap12g19810.1	1<br>Osjap12g19810.1 0.002 1 0.998 0.002 1	1<br>Osind12g15880.1 0.002 1 0.998 0.002 1	0	0	0	0	0	0	0	0
	1<br>Osjap07g08090.1 0.149 0.629 0.48 0.642 0.97	1<br>Osind04g18390.2 0.133 0.627 0.494 0.648 0.967	1<br>Orufi03g39910.2 0.149 0.629 0.48 0.617 0.969	1<br>Oglab06g04730.1 0.149 0.629 0.48 0.655 0.974	1<br>Obart03g38270.2 0.149 0.629 0.48 0.61 0.968	1<br>Olong_001697-R1 0.651 1 0.349 0.006 1	1<br>Opunc03g35430.1 0.149 0.629 0.48 0.637 0.97	1<br>Obrac06g03630.1 0.129 0.622 0.493 0.588 0.894	1<br>Lperr07g06070.1 0.149 0.629 0.48 0.64 0.969	0<br>AT4G01970.1 0.149 0.651 0.502 0.688 0.985

	Osjap12g19770.1 0.651 1 0.349 0.006 1	Osind12g15880.2 0.638 1 0.362 0.012 1	Orufi12g17100.1 0.629 1 0.371 0.035 1	Oglab12g14370.2 0.642 1 0.358 0.115 1	Obart12g15400.1 0.651 1 0.349 0.006 1	Olong_022562-R1 0.149 0.629 0.48 0.615 0.971	Opunc12g13920.1 0.64 1 0.36 0.003 1	Obrac12g09830.1 0.649 1 0.351 0.003 1	Lperr12g12590.1 0.651 1 0.349 0.006 1	
Osjap12g24450.1	1<br>Osjap12g24450.1 0.001 1 0.999 0.001 1	0	0	0	0	0	0	0	0	0
	1<br>Osjap03g06030.1 0.06 0.59 0.53 0.122 0.973<br>Osjap07g31120.1 0.6 0.992 0.392 0.008 0.974	1<br>Osind12g20480.1 0.04 0.591 0.551 0.076 0.975<br>Osind12g20490.1 0.599 0.996 0.397 0.003 0.982	1<br>Orufi12g21590.1 0.039 0.591 0.552 0.063 0.978<br>Orufi12g21600.1 0.599 0.996 0.397 0.003 0.982	1<br>Oglab12g17520.1 0.039 0.591 0.552 0.063 0.978<br>Oglab12g17530.1 0.599 0.996 0.397 0.003 0.982	1<br>Obart03g05850.1 0.06 0.59 0.53 0.122 0.973<br>Obart12g19340.1 0.599 0.996 0.397 0.002 0.98	1<br>Olong_000677-R1 0.325 0.589 0.264 0.093 0.96<br>Olong_002428-R1 0.06 0.315 0.255 0.188 0.726<br>Olong_008690-R1 0.6 0.992 0.392 0.008 0.974	1<br>Opunc12g17520.1 0.001 0.591 0.59 0.002 0.983<br>Opunc12g17530.1 0.599 0.995 0.396 0.001 0.985	0<br>Obrac07g18220.1 0.6 0.986 0.386 0.008 0.958<br>Obrac12g12740.1 0.07 0.4 0.33 0.004 0.986	1<br>Lperr07g23910.1 0.595 0.991 0.396 0.006 0.967<br>Lperr12g16150.1 0.008 0.591 0.583 0.003 0.979	0<br>AT5G17850.1 0.047 0.586 0.539 0.05 0.971

(((((((_a1,rufi),_a2),longi),punctata),brachy),leersia),tair);

chimeric gene id	INGROUP		MIDGROUP					OUTGROUP		
	jap	indica	rufi	glab	barthii	longi	punctata	brachy	leersia	tair
Osjap01g00500.1	1<br>Osjap01g00500.1 0.002 1 0.998 0.002 1	0	0	0	0	0	0	0	0	0
	0<br>Osjap01g00490.1 0.407 1 0.593 0.003 1	1<br>Osind01g00540.1 0.407 1 0.593 0.003 1<br>Osind01g00560.1 0.002 0.405 0.403 0.005 1	0<br>Orufi12g08600.2 0.384 1 0.616 0.006 0.961	1<br>Oglab01g00380.1 0.407 1 0.593 0.003 1<br>Oglab01g00390.1 0.002 0.405 0.403 0.005 1	1<br>Obart01g00460.1 0.002 0.405 0.403 0.005 1<br>Obart06g14780.1 0.407 1 0.593 0.003 1	1<br>Olong_014274-R1 0.002 0.405 0.403 0.004 0.964<br>Olong_018545-R1 0.407 1 0.593 0.162 1	1<br>Opunc01g00410.2 0.002 0.396 0.394 0.002 0.432<br>Opunc06g13790.1 0.407 1 0.593 0.003 1	0<br>Obrac01g00370.2 0.405 1 0.595 0.009 1	0<br>Lperr06g12190.1 0.407 1 0.593 0.146 1	0<br>AT1G32100.1 0.407 1 0.593 0.003 1
Osjap01g00640.1	1<br>Osjap01g00640.1 0.001 1 0.999 0.001 1<br>Osjap01g00640.2 0.001 1 0.999 0.001 1<br>Osjap01g00640.3 0.001 1 0.999 0.001 1<br>Osjap01g00640.4 0.001 1 0.999 0.001 1	1<br>Osind01g00770.1 0.105 1 0.895 0.022 1<br>Osind01g00770.2 0.105 1 0.895 0.027 1	0	0	0	0	0	0	0	0
	1<br>Osjap04g25390.2 0.57 1 0.43 0.019 1<br>Osjap05g00080.1 0.001 0.451 0.45 0.007 1	1<br>Osind04g24430.1 0.57 1 0.43 0.019 1<br>Osind05g00060.1 0.001 0.451 0.45 0.007 1	1<br>Orufi01g00590.1 0.565 1 0.435 0.001 1<br>Orufi05g00070.1 0.001 0.451 0.45 0.007 1	1<br>Oglab01g00510.1 0.565 1 0.435 0.001 1<br>Oglab05g00040.1 0.001 0.451 0.45 0.007 1	1<br>Obart01g00590.1 0.565 1 0.435 0.001 1<br>Obart05g00050.1 0.001 0.451 0.45 0.007 1	1<br>Olong_015014-R1 0.49 1 0.51 0.055 1<br>Olong_015017-R1 0.001 0.451 0.45 0.001 1	1<br>Opunc01g00510.1 0.001 0.451 0.45 0.078 1<br>Opunc09g01380.1 0.554 1 0.446 0.03 1	1<br>Obrac05g00060.2 0.001 0.451 0.45 0.008 1<br>Obrac09g00870.1 0.564 1 0.436 0.052 1	1<br>Lperr01g00510.1 0.001 0.451 0.45 0.007 1<br>Lperr09g00030.1 0.564 1 0.436 0.031 1	1<br>AT1G20380.1 0.565 1 0.435 0.001 1<br>AT5G08440.2 0.002 0.45 0.448 0.012 0.997
Osjap01g00640.2	1<br>Osjap01g00640.1 0.001 1 0.999 0.001 1<br>Osjap01g00640.2 0.001 1 0.999 0.001 1<br>Osjap01g00640.3 0.001 1 0.999 0.001 1<br>Osjap01g00640.4 0.001 1 0.999 0.001 1	1<br>Osind01g00770.1 0.107 1 0.893 0.022 1<br>Osind01g00770.2 0.107 1 0.893 0.027 1	0	0	0	0	0	0	0	0
	1<br>Osjap04g25390.2 0.565 1 0.435 0.019 1<br>Osjap05g00080.1 0.001 0.457 0.456 0.007 1	1<br>Osind04g24430.1 0.565 1 0.435 0.019 1<br>Osind05g00060.1 0.001 0.457 0.456 0.007 1	1<br>Orufi01g00590.1 0.558 1 0.442 0.001 1<br>Orufi05g00070.1 0.001 0.457 0.456 0.007 1	1<br>Oglab01g00510.1 0.558 1 0.442 0.001 1<br>Oglab05g00040.1 0.001 0.457 0.456 0.007 1	1<br>Obart01g00590.1 0.558 1 0.442 0.001 1<br>Obart05g00050.1 0.001 0.457 0.456 0.007 1	1<br>Olong_015014-R1 0.493 1 0.507 0.07 1<br>Olong_015017-R1 0.001 0.457 0.456 0.001 1	1<br>Opunc01g00510.1 0.001 0.457 0.456 0.078 1<br>Opunc09g01380.1 0.548 1 0.452 0.03 1	1<br>Obrac05g00060.2 0.001 0.457 0.456 0.008 1<br>Obrac09g00870.1 0.558 1 0.442 0.052 1	1<br>Lperr01g00510.1 0.001 0.457 0.456 0.007 1<br>Lperr09g00030.1 0.558 1 0.442 0.031 1	1<br>AT1G20380.1 0.558 1 0.442 0.001 1<br>AT5G08440.2 0.002 0.457 0.455 0.012 0.997
Osjap01g00640.3	1<br>Osjap01g00640.1 0.001 1 0.999 0.001 1	1<br>Osind01g00770.1 0.107 1 0.893 0.022 1	0	0	0	0	0	0	0	0

	Osjap01g00640.2 0.001 1 0.999 0.001 1<br>Osjap01g00640.3 0.001 1 0.999 0.001 1<br>Osjap01g00640.4 0.001 1 0.999 0.001 1	Osind01g00770.2 0.107 1 0.893 0.027 1								
	1<br>Osjap04g25390.2 0.564 1 0.436 0.019 1<br>Osjap05g00080.1 0.001 0.458 0.457 0.007 0.999	1<br>Osind04g24430.1 0.564 1 0.436 0.019 1<br>Osind05g00060.1 0.001 0.458 0.457 0.007 0.999	1<br>Orufi01g00590.1 0.558 1 0.442 0.001 1<br>Orufi05g00070.1 0.001 0.458 0.457 0.007 0.999	1<br>Oglab01g00510.1 0.558 1 0.442 0.001 1<br>Oglab05g00040.1 0.001 0.458 0.457 0.007 0.999	1<br>Obart01g00590.1 0.558 1 0.442 0.001 1<br>Obart05g00050.1 0.001 0.458 0.457 0.007 0.999	1<br>Olong_015014-R1 0.459 1 0.541 0.001 1<br>Olong_015017-R1 0.001 0.458 0.457 0.001 0.999	1<br>Opunc01g00510.1 0.001 0.458 0.457 0.078 0.999<br>Opunc09g01380.1 0.547 1 0.453 0.03 1	1<br>Obrac05g00060.2 0.001 0.458 0.457 0.008 0.998<br>Obrac09g00870.1 0.557 1 0.443 0.052 1	1<br>Lperr01g00510.1 0.001 0.458 0.457 0.007 0.998<br>Lperr09g00030.1 0.557 1 0.443 0.031 1	1<br>AT1G20380.1 0.558 1 0.442 0.001 1<br>AT5G08440.2 0.002 0.458 0.456 0.012 0.999
Osjap01g00640.4	1<br>Osjap01g00640.1 0.001 1 0.999 0.001 1<br>Osjap01g00640.2 0.001 1 0.999 0.001 1<br>Osjap01g00640.3 0.001 1 0.999 0.001 1<br>Osjap01g00640.4 0.001 1 0.999 0.001 1	1<br>Osind01g00770.1 0.109 1 0.891 0.022 1<br>Osind01g00770.2 0.109 1 0.891 0.027 1	0	0	0	0	0	0	0	0
	1<br>Osjap04g25390.2 0.555 1 0.445 0.019 1<br>Osjap05g00080.1 0.001 0.467 0.466 0.007 0.999	1<br>Osind04g24430.1 0.555 1 0.445 0.019 1<br>Osind05g00060.1 0.001 0.467 0.466 0.007 0.999	1<br>Orufi01g00590.1 0.549 1 0.451 0.001 1<br>Orufi05g00070.1 0.001 0.467 0.466 0.007 0.999	1<br>Oglab01g00510.1 0.549 1 0.451 0.001 1<br>Oglab05g00040.1 0.001 0.467 0.466 0.007 0.999	1<br>Obart01g00590.1 0.549 1 0.451 0.001 1<br>Obart05g00050.1 0.001 0.467 0.466 0.007 0.999	1<br>Olong_015014-R1 0.468 1 0.532 0.001 1<br>Olong_015017-R1 0.001 0.467 0.466 0.001 0.999	1<br>Opunc01g00510.1 0.001 0.467 0.466 0.078 0.999<br>Opunc09g01380.1 0.538 1 0.462 0.03 1	1<br>Obrac05g00060.2 0.001 0.467 0.466 0.008 0.998<br>Obrac09g00870.1 0.548 1 0.452 0.052 1	1<br>Lperr01g00510.1 0.001 0.467 0.466 0.007 0.998<br>Lperr09g00030.1 0.548 1 0.452 0.031 1	1<br>AT1G20380.1 0.549 1 0.451 0.001 1<br>AT5G08440.2 0.002 0.467 0.465 0.012 0.999
Osjap01g05660.1	1<br>Osjap01g05660.1 0.001 1 0.999 0.001 1	0	0	0	0	0	0	0	0	0
	0<br>Osjap04g21900.2 0.001 0.513 0.512 0.13 0.997	1<br>Osind01g05840.1 0.524 0.999 0.475 0.001 0.995<br>Osind04g21000.1 0.001 0.513 0.512 0.149 0.997	1<br>Orufi01g05280.1 0.524 0.999 0.475 0.001 0.995<br>Orufi04g19880.1 0.001 0.513 0.512 0.13 0.997	1<br>Oglab01g04570.1 0.524 0.999 0.475 0.001 0.995<br>Oglab04g16820.1 0.001 0.513 0.512 0.103 0.997	1<br>Obart01g05020.1 0.524 0.999 0.475 0.001 0.995<br>Obart04g18400.1 0.001 0.513 0.512 0.106 0.997	1<br>Olong_008851-R2 0.519 0.999 0.48 0.047 0.995<br>Olong_009541-R1 0.001 0.513 0.512 0.082 0.996	1<br>Opunc01g04830.3 0.004 0.513 0.509 0.004 0.996<br>Opunc01g04840.2 0.524 0.999 0.475 0.001 0.995	1<br>Obrac01g03780.3 0.524 0.999 0.475 0.001 0.995<br>Obrac04g11710.1 0.002 0.513 0.511 0.107 0.997	1<br>Lperr01g04710.1 0.524 0.999 0.475 0.001 0.995<br>Lperr04g14860.2 0.005 0.519 0.514 0.002 0.996	1<br>AT2G32700.7 0.005 0.513 0.508 0.004 0.996<br>AT3G20630.1 0.524 0.999 0.475 0.001 0.994
Osjap01g06620.1	1<br>Osjap01g06620.1 0.001 1 0.999 0.001 1<br>Osjap01g06620.2 0.001 1 0.999 0.001 1<br>Osjap01g06620.3 0.001 1 0.999 0.001 1<br>Osjap01g06620.4 0.001 1 0.999 0.001 1<br>Osjap01g06620.5 0.001 1 0.999 0.001 1<br>Osjap01g06620.6 0.001 1 0.999 0.001 1	0	0	0	0	0	0	0	0	0
	0<br>Osjap01g00270.3 0.404 0.998 0.594 0.002 1	1<br>Osind01g06600.1 0.399 1 0.601 0.001 1<br>Osind01g06610.1 0.009 0.344 0.335 0.03 1	1<br>Orufi01g06220.1 0.399 1 0.601 0.001 1<br>Orufi01g06230.1 0.009 0.344 0.335 0.026 1	1<br>Oglab01g05340.1 0.399 1 0.601 0.001 1<br>Oglab01g05350.1 0.001 0.344 0.343 0.002 1	1<br>Obart01g05730.1 0.399 1 0.601 0.001 1<br>Obart01g05740.1 0.001 0.344 0.343 0.002 1	0<br>Olong_013885-R1 0.047 0.344 0.297 0.038 1<br>Olong_024361-R1 0.683 0.998 0.315 0.002 1	1<br>Opunc01g00250.2 0.4 0.998 0.598 0.004 1<br>Opunc01g05590.1 0.001 0.344 0.343 0.002 1	1<br>Obrac01g23150.1 0.018 0.344 0.326 0.059 1<br>Obrac06g09410.4 0.399 0.998 0.599 0.001 0.997	1<br>Lperr01g00210.1 0.399 0.998 0.599 0.001 1<br>Lperr01g05400.1 0.001 0.344 0.343 0.124 1	1<br>AT1G79750.1 0.031 0.344 0.313 0.08 1<br>AT4G20060.1 0.399 0.998 0.599 0.001 0.999
Osjap01g06620.2	1<br>Osjap01g06620.1 0.001 1 0.999 0.001 1<br>Osjap01g06620.2 0.001 1 0.999 0.001 1<br>Osjap01g06620.3 0.001 1 0.999 0.001 1<br>Osjap01g06620.4 0.001 1 0.999 0.001 1<br>Osjap01g06620.5 0.001 1 0.999 0.001 1<br>Osjap01g06620.6 0.001 1 0.999 0.001 1	0	0	0	0	0	0	0	0	0
	0	1	1	1	1	0	1	1	1	1

	Osjap01g00270.3 0.39 0.998 0.608 0.002 1	Osind01g06600.1 0.385 1 0.615 0.001 1<br>Osind01g06610.1 0.008 0.332 0.324 0.03 1	Orufi01g06220.1 0.385 1 0.615 0.001 1<br>Orufi01g06230.1 0.008 0.332 0.324 0.026 1	Oglab01g05340.1 0.385 1 0.615 0.001 1<br>Oglab01g05350.1 0.001 0.332 0.331 0.002 1	Obart01g05730.1 0.385 1 0.615 0.001 1<br>Obart01g05740.1 0.001 0.332 0.331 0.002 1	Olong_013885-R1 0.045 0.332 0.287 0.038 1<br>Olong_024361-R1 0.673 0.998 0.325 0.002 1	Opunc01g00250.2 0.386 0.998 0.612 0.004 1<br>Opunc01g05590.1 0.001 0.332 0.331 0.002 1	Obrac01g23150.1 0.018 0.332 0.314 0.059 1<br>Obrac06g09410.4 0.385 0.998 0.613 0.001 0.997	Lperr01g00210.1 0.385 0.998 0.613 0.001 1<br>Lperr01g05400.1 0.001 0.332 0.331 0.124 1	AT1G79750.1 0.03 0.332 0.302 0.08 1<br>AT4G20060.1 0.385 0.998 0.613 0.001 0.999
Osjap01g06620.3	1<br>Osjap01g06620.1 0.001 1 0.999 0.001 1<br>Osjap01g06620.2 0.001 1 0.999 0.001 1<br>Osjap01g06620.3 0.001 1 0.999 0.001 1<br>Osjap01g06620.4 0.001 1 0.999 0.001 1<br>Osjap01g06620.5 0.001 1 0.999 0.001 1<br>Osjap01g06620.6 0.001 1 0.999 0.001 1	0	0	0	0	0	0	0	0	0
	0<br>Osjap01g00270.3 0.391 0.998 0.607 0.002 1	1<br>Osind01g06600.1 0.386 1 0.614 0.001 1<br>Osind01g06610.1 0.008 0.334 0.326 0.03 1	1<br>Orufi01g06220.1 0.386 1 0.614 0.001 1<br>Orufi01g06230.1 0.008 0.334 0.326 0.026 1	1<br>Oglab01g05340.1 0.386 1 0.614 0.001 1<br>Oglab01g05350.1 0.001 0.334 0.333 0.002 1	1<br>Obart01g05730.1 0.386 1 0.614 0.001 1<br>Obart01g05740.1 0.001 0.334 0.333 0.002 1	0<br>Olong_013885-R1 0.045 0.334 0.289 0.038 1<br>Olong_024361-R1 0.672 0.998 0.326 0.002 1	1<br>Opunc01g00250.2 0.387 0.998 0.611 0.004 1<br>Opunc01g05590.1 0.001 0.334 0.333 0.002 1	1<br>Obrac01g23150.1 0.018 0.334 0.316 0.059 1<br>Obrac06g09410.4 0.386 0.998 0.612 0.001 0.997	1<br>Lperr01g00210.1 0.386 0.998 0.612 0.001 1<br>Lperr01g05400.1 0.001 0.334 0.333 0.124 1	1<br>AT1G79750.1 0.03 0.334 0.304 0.08 1<br>AT4G20060.1 0.386 0.998 0.612 0.001 0.999
Osjap01g06620.4	1<br>Osjap01g06620.1 0.001 1 0.999 0.001 1<br>Osjap01g06620.2 0.001 1 0.999 0.001 1<br>Osjap01g06620.3 0.001 1 0.999 0.001 1<br>Osjap01g06620.4 0.001 1 0.999 0.001 1<br>Osjap01g06620.5 0.001 1 0.999 0.001 1<br>Osjap01g06620.6 0.001 1 0.999 0.001 1	0	0	0	0	0	0	0	0	0
	0<br>Osjap01g00270.3 0.384 0.998 0.614 0.002 1	1<br>Osind01g06600.1 0.379 1 0.621 0.001 1<br>Osind01g06610.1 0.008 0.326 0.318 0.03 1	1<br>Orufi01g06220.1 0.379 1 0.621 0.001 1<br>Orufi01g06230.1 0.008 0.326 0.318 0.026 1	1<br>Oglab01g05340.1 0.379 1 0.621 0.001 1<br>Oglab01g05350.1 0.001 0.326 0.325 0.002 1	1<br>Obart01g05730.1 0.379 1 0.621 0.001 1<br>Obart01g05740.1 0.001 0.326 0.325 0.002 1	0<br>Olong_013885-R1 0.046 0.326 0.28 0.038 1<br>Olong_024361-R1 0.67 0.998 0.328 0.002 1	1<br>Opunc01g00250.2 0.38 0.998 0.618 0.004 1<br>Opunc01g05590.1 0.001 0.326 0.325 0.002 1	1<br>Obrac01g23150.1 0.018 0.326 0.308 0.059 1<br>Obrac06g09410.4 0.379 0.998 0.619 0.001 0.997	1<br>Lperr01g00210.1 0.379 0.998 0.619 0.001 1<br>Lperr01g05400.1 0.001 0.326 0.325 0.124 1	1<br>AT1G79750.1 0.03 0.326 0.296 0.08 1<br>AT4G20060.1 0.379 0.998 0.619 0.001 0.999
Osjap01g06620.5	1<br>Osjap01g06620.1 0.001 1 0.999 0.001 1<br>Osjap01g06620.2 0.001 1 0.999 0.001 1<br>Osjap01g06620.3 0.001 1 0.999 0.001 1<br>Osjap01g06620.4 0.001 1 0.999 0.001 1<br>Osjap01g06620.5 0.001 1 0.999 0.001 1<br>Osjap01g06620.6 0.001 1 0.999 0.001 1	0	0	0	0	0	0	0	0	0
	0<br>Osjap01g00270.3 0.404 0.998 0.594 0.002 1	1<br>Osind01g06600.1 0.399 1 0.601 0.001 1<br>Osind01g06610.1 0.009 0.345 0.336 0.03 1	1<br>Orufi01g06220.1 0.399 1 0.601 0.001 1<br>Orufi01g06230.1 0.009 0.345 0.336 0.026 1	1<br>Oglab01g05340.1 0.399 1 0.601 0.001 1<br>Oglab01g05350.1 0.001 0.345 0.344 0.002 1	1<br>Obart01g05730.1 0.399 1 0.601 0.001 1<br>Obart01g05740.1 0.001 0.345 0.344 0.002 1	0<br>Olong_013885-R1 0.047 0.345 0.298 0.038 1<br>Olong_024361-R1 0.661 0.998 0.337 0.002 1	1<br>Opunc01g00250.2 0.4 0.998 0.598 0.004 1<br>Opunc01g05590.1 0.001 0.345 0.344 0.002 1	1<br>Obrac01g23150.1 0.018 0.345 0.327 0.059 1<br>Obrac06g09410.4 0.399 0.998 0.599 0.001 0.997	1<br>Lperr01g00210.1 0.399 0.998 0.599 0.001 1<br>Lperr01g05400.1 0.001 0.345 0.344 0.124 1	1<br>AT1G79750.1 0.031 0.345 0.314 0.08 1<br>AT4G20060.1 0.399 0.998 0.599 0.001 0.999
Osjap01g06620.6	1<br>Osjap01g06620.1 0.001 1 0.999 0.001 1<br>Osjap01g06620.2 0.001 1 0.999 0.001 1<br>Osjap01g06620.3 0.001 1 0.999 0.001 1<br>Osjap01g06620.4 0.001 1 0.999 0.001 1<br>Osjap01g06620.5 0.001 1 0.999 0.001 1	0	0	0	0	0	0	0	0	0

	Osjap01g06620.6 0.001 1 0.999 0.001 1									
	0<br>Osjap01g00270.1 0.45 0.998 0.548 0.321 1	1<br>Osind01g06600.1 0.432 1 0.568 0.001 1<br>Osind01g06610.1 0.009 0.373 0.364 0.03 1	1<br>Orufi01g06220.1 0.432 1 0.568 0.001 1<br>Orufi01g06230.1 0.009 0.373 0.364 0.026 1	1<br>Oglab01g05340.1 0.432 1 0.568 0.001 1<br>Oglab01g05350.1 0.001 0.373 0.372 0.002 1	1<br>Obart01g05730.1 0.432 1 0.568 0.001 1<br>Obart01g05740.1 0.001 0.373 0.372 0.002 1	0<br>Olong_013885-R1 0.051 0.373 0.322 0.038 1<br>Olong_024361-R1 0.633 0.998 0.365 0.002 1	1<br>Opunc01g00250.2 0.45 0.998 0.548 0.189 1<br>Opunc01g05590.1 0.001 0.373 0.372 0.002 1	1<br>Obrac01g23150.1 0.02 0.373 0.353 0.059 1<br>Obrac06g09410.2 0.45 0.998 0.548 0.196 0.998	1<br>Lperr01g00210.1 0.447 0.998 0.551 0.187 1<br>Lperr01g05400.1 0.001 0.373 0.372 0.124 1	0<br>AT1G79750.1 0.034 0.373 0.339 0.08 1<br>AT4G20060.1 0.647 0.998 0.351 0.448 0.999
Osjap01g13630.1	1<br>Osjap01g13630.1 0.001 1 0.999 0.001 1	0	1<br>Orufi01g13080.1 0.042 1 0.958 0.043 1	1<br>Oglab01g10320.1 0.042 1 0.958 0.042 1	0	0	0	1<br>Obrac01g09290.1 0.001 1 0.999 0.001 1	0	0
	0<br>Osjap01g13630.3 0.459 1 0.541 0.002 1	1<br>Osind01g13830.1 0.535 1 0.465 0.002 1<br>Osind01g13840.1 0.001 0.449 0.448 0.002 0.99	1<br>Orufi01g13080.4 0.459 1 0.541 0.002 1<br>Orufi01g13090.1 0.001 0.449 0.448 0.002 0.99	0<br>Oglab01g10320.2 0.459 1 0.541 0.002 1	1<br>Obart01g12120.1 0.535 1 0.465 0.002 1<br>Obart01g12130.1 0.001 0.487 0.486 0.002 0.998	1<br>Olong_021508-R1 0.53 1 0.47 0.062 1<br>Olong_021509-R1 0.077 0.449 0.372 0.005 0.99	1<br>Opunc01g11830.1 0.031 0.487 0.456 0.054 0.998<br>Opunc05g02420.2 0.535 1 0.465 0.002 1	0<br>Obrac05g02000.1 0.535 1 0.465 0.002 1	1<br>Lperr01g11090.1 0.001 0.458 0.457 0.002 0.984<br>Lperr05g21710.3 0.531 1 0.469 0.008 1	1<br>AT3G62130.1 0.025 0.444 0.419 0.013 0.993<br>AT4G04460.1 0.545 1 0.455 0.022 1
Osjap01g25870.1	1<br>Osjap01g25870.1 0.001 1 0.999 0.001 1	0	0	1<br>Oglab01g18120.1 0.004 1 0.996 0.149 1	0	0	0	0	0	0
	0<br>Osjap12g10880.1 0.373 0.982 0.609 0.23 0.925	1<br>Osind01g26950.1 0.001 0.326 0.325 0.003 1<br>Osind11g14850.1 0.367 0.996 0.629 0.001 0.961	0<br>Orufi01g24680.1 0.004 0.627 0.623 0.207 1	1<br>Oglab01g17930.1 0.393 0.996 0.603 0.008 0.985<br>Oglab01g18150.1 0.001 0.326 0.325 0.003 1	1<br>Obart01g21780.1 0.395 0.991 0.596 0.015 0.908<br>Obart01g21980.1 0.001 0.381 0.38 0.002 1	1<br>Olong_007897-R1 0.146 0.381 0.235 0.004 1<br>Olong_007898-R1 0.391 1 0.609 0.001 0.876	1<br>Opunc01g22160.1 0.001 0.373 0.372 0.002 0.973<br>Opunc10g14020.1 0.387 0.985 0.598 0.003 0.864	0<br>Obrac11g13690.1 0.38 0.985 0.605 0.002 0.645	1<br>Lperr01g18740.1 0.003 0.38 0.377 0.004 0.998<br>Lperr01g18750.1 0.391 1 0.609 0.001 0.901	0<br>AT2G15080.1 0.396 1 0.604 0.009 0.907
Osjap01g30020.1	1<br>Osjap01g30020.1 0.002 1 0.998 0.002 1	0	0	0	0	0	0	0	0	0
	0<br>Osjap01g30020.2 0.002 0.576 0.574 0.003 1	1<br>Osind01g31470.1 0.595 0.946 0.351 0.004 0.781<br>Osind01g31480.1 0.002 0.576 0.574 0.003 1	1<br>Orufi01g28700.1 0.595 1 0.405 0.005 1<br>Orufi01g28710.2 0.002 0.576 0.574 0.004 1	1<br>Oglab01g21750.1 0.648 1 0.352 0.005 1<br>Oglab01g21760.1 0.002 0.576 0.574 0.003 1	1<br>Obart01g25790.1 0.595 1 0.405 0.005 1<br>Obart01g25800.1 0.002 0.576 0.574 0.003 1	1<br>Olong_011926-R1 0.7 1 0.3 0.098 1<br>Olong_011927-R1 0.002 0.576 0.574 0.004 1	1<br>Opunc01g25700.1 0.648 1 0.352 0.008 1<br>Opunc01g25710.1 0.002 0.576 0.574 0.003 1	1<br>Obrac01g19510.1 0.648 1 0.352 0.006 1<br>Obrac01g19520.1 0.002 0.576 0.574 0.003 1	1<br>Lperr01g22050.1 0.648 1 0.352 0.006 1<br>Lperr01g22060.1 0.002 0.576 0.574 0.003 1	0<br>AT1G29970.2 0.656 0.964 0.308 0.003 0.446<br>AT1G53580.1 0.119 0.568 0.449 0.173 0.986
Osjap01g33510.1	1<br>Osjap01g33510.1 0.001 1 0.999 0.001 1<br>Osjap01g33510.2 0.001 1 0.999 0.001 1<br>Osjap01g33510.3 0.001 1 0.999 0.001 1<br>Osjap01g33510.4 0.001 1 0.999 0.001 1	0	0	1<br>Oglab01g24700.1 0.022 1 0.978 0.001 1<br>Oglab01g24700.2 0.022 1 0.978 0.001 1<br>Oglab01g24700.3 0.022 1 0.978 0.001 1	0	0	0	1<br>Obrac01g22030.1 0.015 1 0.985 0.018 0.999<br>Obrac01g22030.2 0.015 1 0.985 0.018 0.999	0	0
	0<br>Osjap02g13850.1 0.637 1 0.363 0.003 0.989<br>Osjap03g28950.1 0.261 0.541 0.28 0.103 0.969	1<br>Osind01g35000.1 0.078 0.625 0.547 0.002 0.94<br>Osind02g13980.2 0.637 1 0.363 0.003 0.989	1<br>Orufi01g32190.1 0.078 0.625 0.547 0.002 0.944<br>Orufi02g12700.1 0.637 1 0.363 0.003 0.989	0<br>Oglab02g12410.1 0.637 1 0.363 0.003 0.989<br>Oglab03g26370.1 0.261 0.541 0.28 0.103 0.969	1<br>Obart01g29130.1 0.078 0.625 0.547 0.002 0.94<br>Obart02g12550.1 0.637 1 0.363 0.003 0.989	1<br>Olong_006665-R1 0.151 0.625 0.474 0.012 0.926<br>Olong_027869-R1 0.637 1 0.363 0.003 0.997	1<br>Opunc01g28950.1 0.078 0.625 0.547 0.002 0.936<br>Opunc02g11230.1 0.637 1 0.363 0.003 0.997	0<br>Obrac01g23640.1 0.644 0.998 0.354 0.141 0.942<br>Obrac02g16330.1 0.282 0.505 0.223 0.21 0.922	0<br>Lperr01g08580.1 0.618 0.998 0.38 0.044 0.956<br>Lperr01g24930.2 0.106 0.332 0.226 0.439 0.992	1<br>AT3G24430.1 0.156 0.625 0.469 0.145 0.947<br>AT5G24230.1 0.632 1 0.368 0.03 0.995
Osjap01g33510.2	1<br>Osjap01g33510.1 0.001 1 0.999 0.001 1<br>Osjap01g33510.2 0.001 1 0.999 0.001 1<br>Osjap01g33510.3 0.001 1 0.999 0.001 1<br>Osjap01g33510.4 0.001 1 0.999 0.001 1	0	0	1<br>Oglab01g24700.1 0.023 1 0.977 0.001 1<br>Oglab01g24700.2 0.023 1 0.977 0.001 1<br>Oglab01g24700.3 0.023 1 0.977 0.001 1	0	0	0	1<br>Obrac01g22030.1 0.015 1 0.985 0.018 0.999<br>Obrac01g22030.2 0.015 1 0.985 0.018 0.999	0	0
	0<br>Osjap02g13850.1 0.627 1 0.373 0.003 0.989<br>Osjap03g28950.1 0.268 0.529 0.261 0.103 0.969	1<br>Osind01g35000.1 0.08 0.615 0.535 0.002 0.94<br>Osind02g13980.2 0.627 1 0.373 0.003 0.989	1<br>Orufi01g32190.1 0.08 0.615 0.535 0.002 0.944<br>Orufi02g12700.1 0.627 1 0.373 0.003 0.989	0<br>Oglab02g12410.1 0.627 1 0.373 0.003 0.989<br>Oglab03g26370.1 0.268 0.529 0.261 0.103 0.969	1<br>Obart01g29130.1 0.08 0.615 0.535 0.002 0.94<br>Obart02g12550.1 0.627 1 0.373 0.003 0.989	1<br>Olong_006665-R1 0.155 0.615 0.46 0.012 0.926<br>Olong_027869-R1 0.627 1 0.373 0.003 0.997	1<br>Opunc01g28950.1 0.08 0.615 0.535 0.002 0.936<br>Opunc02g11230.1 0.627 1 0.373 0.003 0.997	0<br>Obrac01g23640.1 0.635 0.998 0.363 0.141 0.942	0<br>Lperr01g08580.1 0.607 0.998 0.391 0.044 0.956<br>Lperr01g24930.2 0.109 0.326 0.217 0.439 0.992	1<br>AT3G24430.1 0.16 0.615 0.455 0.145 0.947<br>AT5G24230.1 0.623 1 0.377 0.03 0.995

Osjap01g33510.3	1<br>Osjap01g33510.1 0.001 1 0.999 0.001 1<br>Osjap01g33510.2 0.001 1 0.999 0.001 1<br>Osjap01g33510.3 0.001 1 0.999 0.001 1<br>Osjap01g33510.4 0.001 1 0.999 0.001 1	0	0	0	1<br>Oglab01g24700.1 0.023 1 0.977 0.001 1<br>Oglab01g24700.2 0.023 1 0.977 0.001 1<br>Oglab01g24700.3 0.023 1 0.977 0.001 1	0	0	0	1<br>Obrac01g22030.1 0.015 1 0.985 0.018 0.999<br>Obrac01g22030.2 0.015 1 0.985 0.018 0.999	0	0
	0<br>Osjap02g13850.1 0.626 1 0.374 0.003 0.989<br>Osjap03g28950.1 0.269 0.487 0.218 0.103 0.76	1<br>Osind01g35000.1 0.08 0.613 0.533 0.002 0.94<br>Osind02g13980.2 0.626 1 0.374 0.003 0.989	1<br>Orufi01g32190.1 0.08 0.613 0.533 0.002 0.944<br>Orufi02g12700.1 0.626 1 0.374 0.003 0.989	0<br>Oglab02g12410.1 0.626 1 0.374 0.003 0.989<br>Oglab03g26370.1 0.269 0.487 0.218 0.103 0.76	1<br>Obart01g29130.1 0.08 0.613 0.533 0.002 0.94<br>Obart02g12550.1 0.626 1 0.374 0.003 0.989	1<br>Olong_006665-R1 0.155 0.613 0.458 0.012 0.926<br>Olong_027869-R1 0.626 1 0.374 0.003 0.997	1<br>Opunc01g28950.1 0.08 0.613 0.533 0.002 0.936<br>Opunc02g11230.1 0.626 1 0.374 0.003 0.997	0<br>Obrac01g23640.1 0.633 0.998 0.365 0.141 0.942	0<br>Lperr01g08580.1 0.606 0.998 0.392 0.044 0.956<br>Lperr01g24930.2 0.109 0.343 0.234 0.439 0.992	1<br>AT3G24430.1 0.161 0.613 0.452 0.145 0.947<br>AT5G24230.1 0.621 1 0.379 0.03 0.995
Osjap01g42770.1	1<br>Osjap01g42770.1 0.001 1 0.999 0.001 1	1<br>Osind01g44140.1 0.001 1 0.999 0.001 1	1<br>Orufi01g40790.1 0.001 1 0.999 0.001 1<br>Orufi01g40790.2 0.001 1 0.999 0.001 1<br>Orufi01g40790.3 0.001 1 0.999 0.001 1	0	0	1<br>Olong_024830-R1 0.001 1 0.999 0.001 1	0	0	0	0
	1<br>Osjap01g08100.1 0.012 0.27 0.258 0.133 0.963<br>Osjap03g27560.1 0.413 0.995 0.582 0.146 0.95	1<br>Osind01g08020.1 0.012 0.27 0.258 0.133 0.963<br>Osind03g28340.1 0.413 0.995 0.582 0.146 0.95	1<br>Orufi02g20240.3 0.012 0.259 0.247 0.044 0.951<br>Orufi03g26160.3 0.413 0.995 0.582 0.146 0.95	1<br>Oglab01g32220.1 0.001 0.258 0.257 0.004 0.934<br>Oglab01g32230.1 0.352 0.999 0.647 0.002 0.955	1<br>Obart01g37410.1 0.001 0.258 0.257 0.004 0.934<br>Obart01g37420.1 0.352 1 0.648 0.002 1	1<br>Olong_010570-R1 0.413 0.995 0.582 0.133 0.915<br>Olong_011675-R1 0.012 0.258 0.246 0.517 0.972	1<br>Opunc01g36330.1 0.001 0.258 0.257 0.005 0.975<br>Opunc01g36340.1 0.352 0.999 0.647 0.002 0.969	1<br>Obrac01g28830.1 0.001 0.257 0.256 0.004 0.978<br>Obrac01g28840.1 0.355 1 0.645 0.002 1	1<br>Lperr01g32130.1 0.001 0.258 0.257 0.005 0.977<br>Lperr01g32140.1 0.352 1 0.648 0.002 1	0<br>AT2G37970.1 0.001 0.258 0.257 0.049 0.978<br>AT3G54090.1 0.461 1 0.539 0.123 1
Osjap01g50410.1	1<br>Osjap01g50410.1 0.001 1 0.999 0.001 1<br>Osjap01g50410.2 0.005 1 0.995 0.005 1	0	0	0	0	0	0	0	0	0
	1<br>Osjap05g17010.3 0.078 0.362 0.284 0.258 0.997<br>Osjap05g22890.1 0.439 0.998 0.559 0.089 0.979	1<br>Osind01g51960.1 0.009 0.362 0.353 0.022 0.997<br>Osind01g51970.1 0.425 1 0.575 0.001 1	1<br>Orufi01g47830.2 0.009 0.362 0.353 0.022 0.997<br>Orufi03g16980.1 0.439 0.994 0.555 0.031 0.991	1<br>Oglab03g03400.1 0.44 0.999 0.559 0.028 0.983<br>Oglab05g14070.1 0.084 0.362 0.278 0.227 0.997	1<br>Obart01g44360.2 0.02 0.362 0.342 0.024 0.997<br>Obart01g44370.1 0.425 1 0.575 0.001 1	1<br>Olong_021838-R1 0.039 0.362 0.323 0.002 0.996<br>Olong_021839-R1 0.463 1 0.537 0.001 1	1<br>Opunc01g43300.2 0.039 0.366 0.327 0.002 0.995<br>Opunc01g43310.1 0.425 1 0.575 0.001 1	1<br>Obrac01g34770.2 0.007 0.362 0.355 0.062 0.997<br>Obrac01g34780.1 0.445 1 0.555 0.001 0.999	1<br>Lperr01g38790.2 0.039 0.363 0.324 0.002 0.998<br>Lperr03g14360.1 0.427 0.994 0.567 0.008 0.991	1<br>AT3G21070.1 0.083 0.362 0.279 0.062 1<br>AT3G56370.1 0.428 0.996 0.568 0.004 0.994
Osjap01g50410.2	1<br>Osjap01g50410.1 0.005 1 0.995 0.005 1<br>Osjap01g50410.2 0.001 1 0.999 0.001 1	0	0	0	0	0	0	0	0	0
	1<br>Osjap05g17010.3 0.058 0.348 0.29 0.258 0.997<br>Osjap05g22890.1 0.426 0.998 0.572 0.089 0.979	1<br>Osind01g51960.1 0.009 0.348 0.339 0.022 0.997<br>Osind01g51970.1 0.412 1 0.588 0.001 1	1<br>Orufi01g47830.2 0.009 0.348 0.339 0.022 0.997<br>Orufi03g16980.1 0.426 0.994 0.568 0.031 0.991	1<br>Oglab03g03400.1 0.427 0.999 0.572 0.028 0.983<br>Oglab05g14070.1 0.063 0.348 0.285 0.227 0.997	1<br>Obart01g44360.2 0.021 0.348 0.327 0.024 0.997<br>Obart01g44370.1 0.412 1 0.588 0.001 1	1<br>Olong_021838-R1 0.041 0.348 0.307 0.072 0.996<br>Olong_021839-R1 0.451 1 0.549 0.001 1	1<br>Opunc01g43300.4 0.04 0.351 0.311 0.002 0.994<br>Opunc01g43310.1 0.412 1 0.588 0.001 1	1<br>Obrac01g34770.2 0.008 0.348 0.34 0.062 0.997<br>Obrac01g34780.1 0.432 1 0.568 0.001 0.999	1<br>Lperr01g38790.4 0.04 0.349 0.309 0.002 0.998<br>Lperr03g14360.1 0.414 0.994 0.58 0.008 0.991	1<br>AT3G21070.1 0.062 0.348 0.286 0.062 1<br>AT3G56370.1 0.415 0.996 0.581 0.004 0.994
Osjap02g04220.4	1<br>Osjap02g04220.4 0.002 1 0.998 0.002 1<br>Osjap02g04220.5 0.002 1 0.998 0.002 1	0	0	0	0	0	0	0	0	0
	0<br>Osjap02g04220.3 0.002 0.597 0.595 0.002 0.897	1<br>Osind02g04470.1 0.002 0.595 0.593 0.002 0.887<br>Osind05g30190.1 0.701 0.94 0.239 0.17 0.407	1<br>Orufi02g04090.1 0.002 0.597 0.595 0.002 0.897<br>Orufi07g11350.3 0.701 0.995 0.294 0.222 0.984	1<br>Oglab02g03990.2 0.002 0.597 0.595 0.214 0.911<br>Oglab11g17910.1 0.701 0.992 0.291 0.039 0.17	1<br>Obart02g03990.2 0.002 0.597 0.595 0.209 0.91<br>Obart05g26570.1 0.701 0.94 0.239 0.17 0.407	1<br>Olong_014970-R1 0.002 0.608 0.606 0.154 0.998<br>Olong_017772-R1 0.701 0.992 0.291 0.022 0.54	0<br>Opunc02g02500.1 0.217 0.593 0.376 0.681 0.945<br>Opunc02g04060.1 0.618 0.888 0.27 0.002 0.398	0<br>Obrac05g18790.1 0.701 0.94 0.239 0.122 0.373	0<br>Lperr02g08210.1 0.183 0.593 0.41 0.01 0.839<br>Lperr05g22210.1 0.701 0.94 0.239 0.174 0.41	0<br>AT3G58610.1 0.704 0.94 0.236 0.19 0.418

Osjap02g04220.5	1<br>Osjap02g04220.4 0.002 1 0.998 0.002 1<br>Osjap02g04220.5 0.002 1 0.998 0.002 1	0	0	0	0	0	0	0	0	0
	0<br>Osjap02g04220.2 0.002 0.631 0.629 0.002 0.889	1<br>Osind02g04470.1 0.002 0.631 0.629 0.002 0.889<br>Osind05g30190.1 0.739 0.942 0.203 0.204 0.407	1<br>Orufi02g04090.1 0.002 0.629 0.627 0.002 0.894<br>Orufi07g11350.3 0.739 0.995 0.256 0.33 0.984	1<br>Oglab02g03990.2 0.002 0.631 0.629 0.214 0.911<br>Oglab11g17910.1 0.739 0.992 0.253 0.057 0.17	1<br>Obart02g03990.2 0.002 0.631 0.629 0.209 0.91<br>Obart05g26570.1 0.739 0.942 0.203 0.204 0.407	1<br>Olong_014970-R1 0.002 0.642 0.64 0.154 0.998<br>Olong_017772-R1 0.739 0.992 0.253 0.111 0.54	0<br>Opunc02g02500.1 0.259 0.628 0.369 0.681 0.945<br>Opunc05g24160.1 0.739 0.942 0.203 0.201 0.405	0<br>Obrac05g18790.1 0.739 0.942 0.203 0.159 0.373	0<br>Lperr02g08210.1 0.227 0.628 0.401 0.01 0.839<br>Lperr05g22210.1 0.739 0.942 0.203 0.208 0.41	0<br>AT3G58610.1 0.739 0.942 0.203 0.22 0.418
Osjap02g16210.1	1<br>Osjap02g16210.1 0.003 1 0.997 0.003 1	0	0	0	0	0	0	0	0	0
	0<br>Osjap02g16210.2 0.003 0.781 0.778 0.003 0.941	1<br>Osind02g16490.1 0.663 1 0.337 0.009 1<br>Osind02g16500.1 0.003 0.64 0.637 0.004 0.851	1<br>Orufi01g17650.1 0.663 1 0.337 0.007 1<br>Orufi02g14920.1 0.003 0.64 0.637 0.004 0.851	0<br>Oglab02g14090.1 0.003 0.392 0.389 0.005 0.618<br>Oglab06g12810.1 0.663 1 0.337 0.01 1	1<br>Obart02g14710.1 0.003 0.64 0.637 0.004 0.851<br>Obart09g03220.1 0.663 1 0.337 0.009 1	1<br>Olong_010644-R1 0.695 0.919 0.224 0.195 0.947<br>Olong_018165-R2 0.003 0.64 0.637 0.004 0.851	1<br>Opunc01g07390.1 0.663 1 0.337 0.007 1<br>Opunc02g13160.2 0.072 0.64 0.568 0.099 0.851	0<br>Obrac02g10440.1 0.072 0.392 0.32 0.132 0.822	0<br>Lperr02g11890.1 0.009 0.64 0.631 0.008 0.846	0<br>AT1G26180.1 0.153 0.64 0.487 0.18 0.865
Osjap02g23220.1	1<br>Osjap02g23220.1 0.001 1 0.999 0.001 1	0	0	0	0	0	1<br>Opunc02g18320.1 0.001 0.998 0.997 0.001 0.989<br>Opunc02g18320.2 0.001 1 0.999 0.001 1	1<br>Obrac02g14310.1 0.001 0.987 0.986 0.001 1<br>Obrac02g14310.2 0.001 0.987 0.986 0.001 1<br>Obrac02g14310.3 0.001 0.987 0.986 0.001 1	0	0
	1<br>Osjap03g34460.2 0.001 0.351 0.35 0.003 0.918<br>Osjap04g16230.4 0.423 0.99 0.567 0.015 0.983	1<br>Osind03g35700.1 0.001 0.351 0.35 0.003 0.918<br>Osind04g14780.2 0.423 0.99 0.567 0.016 0.983	1<br>Orufi02g21480.1 0.372 0.998 0.626 0.039 0.983<br>Orufi02g21490.1 0.001 0.336 0.335 0.003 0.948	1<br>Oglab03g30900.1 0.001 0.351 0.35 0.221 0.936<br>Oglab04g11660.2 0.423 0.99 0.567 0.015 0.983	1<br>Obart02g20410.1 0.412 1 0.588 0.002 1<br>Obart03g31870.1 0.001 0.351 0.35 0.281 0.941	1<br>Olong_017754-R1 0.378 0.967 0.589 0.071 1<br>Olong_017755-R1 0.001 0.329 0.328 0.003 0.931	1<br>Opunc02g18320.3 0.001 0.357 0.356 0.003 0.957<br>Opunc04g11260.2 0.423 0.991 0.568 0.015 0.987	1<br>Obrac03g23560.1 0.001 0.314 0.313 0.004 0.94<br>Obrac04g07710.2 0.42 0.985 0.565 0.017 0.977	1<br>Lperr02g16420.1 0.412 0.987 0.575 0.002 0.994<br>Lperr02g16430.2 0.001 0.356 0.355 0.003 0.941	1<br>AT1G21750.1 0.411 0.968 0.557 0.004 0.964<br>AT5G22370.1 0.001 0.333 0.332 0.003 0.909
Osjap02g28800.3	1<br>Osjap02g28800.1 0.001 1 0.999 0.001 1<br>Osjap02g28800.2 0.001 1 0.999 0.001 1<br>Osjap02g28800.3 0.001 1 0.999 0.001 1	0	1<br>Orufi02g26700.1 0.001 0.99 0.989 0.001 0.965	0	0	1<br>Olong_027102-R1 0.144 1 0.856 0.033 1	1<br>Opunc02g23140.1 0.001 1 0.999 0.001 1	0	1<br>Lperr02g20690.1 0.001 1 0.999 0.001 1	0
	1<br>Osjap04g33800.1 0.049 0.673 0.624 0.039 0.974<br>Osjap11g07330.1 0.688 0.998 0.31 0.005 0.995	1<br>Osind02g28600.2 0.684 1 0.316 0.003 1<br>Osind02g28610.1 0.001 0.673 0.672 0.001 0.982	1<br>Orufi02g26700.2 0.001 0.673 0.672 0.001 0.982<br>Orufi11g06530.1 0.688 1 0.312 0.005 1	1<br>Oglab03g04200.1 0.039 0.673 0.634 0.004 0.987<br>Oglab11g05820.1 0.688 0.998 0.31 0.005 0.995	1<br>Obart02g25330.2 0.684 1 0.316 0.003 1<br>Obart02g25340.1 0.001 0.673 0.672 0.001 0.982	1<br>Olong_010559-R1 0.675 0.996 0.321 0.126 0.991<br>Olong_017224-R1 0.068 0.673 0.605 0.014 0.986	0<br>Opunc02g23140.2 0.001 0.673 0.672 0.001 0.982	1<br>Obrac02g18260.1 0.684 1 0.316 0.003 1<br>Obrac02g18270.1 0.001 0.673 0.672 0.001 0.982	1<br>Lperr02g11790.1 0.051 0.673 0.622 0.079 0.981<br>Lperr10g02180.1 0.683 0.998 0.315 0.035 1	1<br>AT1G22440.1 0.686 1 0.314 0.01 1<br>AT5G23390.1 0.001 0.673 0.672 0.016 0.981
Osjap02g30600.1	1<br>Osjap02g30600.1 0.003 1 0.997 0.003 1<br>Osjap02g30600.2 0.003 1 0.997 0.003 1	1<br>Osind02g30400.1 0.032 1 0.968 0.016 1<br>Osind02g30400.2 0.032 1 0.968 0.017 1	1<br>Orufi02g28440.1 0.003 1 0.997 0.003 1	0	0	0	0	0	0	0
	1<br>Osjap04g25080.3 0.508 0.994 0.486 0.153 0.992<br>Osjap04g31350.1 0.003 0.412 0.409 0.004 0.5	0<br>Osind04g24130.1 0.508 0.936 0.428 0.172 0.866<br>Osind05g14500.1 0.026 0.305 0.279 0.129 0.714	1<br>Orufi02g28440.3 0.331 1 0.669 0.005 1<br>Orufi05g00390.1 0.077 0.283 0.206 0.156 0.397	1<br>Oglab02g10070.1 0.003 0.293 0.29 0.002 0.255<br>Oglab02g25140.1 0.331 1 0.669 0.005 1	1<br>Obart02g26980.2 0.331 1 0.669 0.005 1<br>Obart05g12910.2 0.08 0.331 0.251 0.152 0.569	0<br>Olong_009904-R1 0.331 1 0.669 0.005 1	1<br>Opunc02g24620.1 0.428 1 0.572 0.141 1<br>Opunc03g26570.3 0.003 0.305 0.302 0.006 0.75	0<br>Obrac02g19560.1 0.437 1 0.563 0.133 1	0<br>Lperr02g21920.1 0.418 1 0.582 0.442 1	0<br>AT1G01730.1 0.46 0.99 0.53 0.223 0.933
Osjap02g36880.1	1<br>Osjap02g36880.1 0.001 1 0.999 0.001 1	0	0	0	0	0	0	1<br>Obrac02g23910.1 0.001 1 0.999 0.136 1	0	0
	1<br>Osjap06g08840.1 0.384 0.982 0.598 0.111 0.978	1<br>Osind02g36410.1 0.001 0.363 0.362 0.264 0.969	1<br>Orufi02g34400.1 0.001 0.363 0.362 0.003 0.958	1<br>Oglab02g30250.1 0.001 0.363 0.362 0.003 0.919	0<br>Obart02g32720.1 0.306 1 0.694 0.014 1	1<br>Olong_003175-R1 0.363 1 0.637 0.021 1	1<br>Opunc02g30220.3 0.001 0.363 0.362 0.264 0.969	1<br>Obrac05g14180.1 0.398 0.994 0.596 0.033 0.938	1<br>Lperr02g26790.1 0.001 0.363 0.362 0.296 0.97	1<br>AT1G22170.1 0.001 0.363 0.362 0.003 0.946

	Osjap06g08870.1 0.052 0.363 0.311 0.086 0.957	Osind02g36420.1 0.363 1 0.637 0.021 1	Orufi02g34410.1 0.363 1 0.637 0.021 1	Oglab02g30260.1 0.363 1 0.637 0.121 1		Olong_003176-R2 0.001 0.363 0.362 0.184 0.961	Opunc02g30230.1 0.363 1 0.637 0.022 1	Obrac06g05990.1 0.001 0.363 0.362 0.003 0.953	Lperr02g26800.1 0.363 1 0.637 0.022 1	AT5G65090.1 0.413 0.998 0.585 0.081 1
Osjap02g39740.1	1<br>Osjap02g39740.1 0.001 1 0.999 0.001 1	0	0	0	0	0	0	0	0	0
	0<br>Osjap09g20450.1 0.654 0.985 0.331 0.041 0.963	1<br>Osind02g39300.1 0.039 0.632 0.593 0.015 1<br>Osind09g20550.1 0.654 0.985 0.331 0.019 0.932	1<br>Orufi02g37130.2 0.001 0.632 0.631 0.001 1<br>Orufi09g21510.1 0.654 0.985 0.331 0.463 0.991	1<br>Oglab02g32840.2 0.039 0.632 0.593 0.016 1<br>Oglab09g18170.1 0.654 0.985 0.331 0.049 0.872	1<br>Obart02g35650.1 0.039 0.632 0.593 0.013 1<br>Obart09g19950.1 0.654 0.985 0.331 0.049 0.872	1<br>Olong_003820-R1 0.039 0.632 0.593 0.013 1<br>Olong_003821-R1 0.665 0.988 0.323 0.045 0.988	1<br>Opunc02g00500.1 0.659 0.99 0.331 0.088 0.943<br>Opunc02g32820.2 0.001 0.632 0.631 0.001 1	1<br>Obrac02g26120.1 0.001 0.632 0.631 0.001 1<br>Obrac02g26130.1 0.656 0.986 0.33 0.019 1	1<br>Lperr02g29300.1 0.001 0.632 0.631 0.001 1<br>Lperr10g08790.1 0.661 0.986 0.325 0.032 0.952	0<br>AT1G79690.1 0.005 0.629 0.624 0.004 0.999
Osjap02g40340.1	1<br>Osjap02g40310.1 0.003 0.982 0.979 0.162 0.926<br>Osjap02g40340.1 0.003 1 0.997 0.003 1<br>Osjap02g40370.1 0.003 0.982 0.979 0.002 0.726	1<br>Osind02g39860.1 0.003 0.985 0.982 0.002 0.659	0	0	0	0	0	0	0	0
	1<br>Osjap02g41540.1 0.655 0.997 0.342 0.434 0.806<br>Osjap03g26380.1 0.003 0.292 0.289 0.004 0.406<br>Osjap05g23970.1 0.368 0.591 0.223 0.665 0.821	1<br>Osind02g39840.1 0.003 0.292 0.289 0.007 0.809<br>Osind02g39860.2 0.332 0.985 0.653 0.015 0.571	0<br>Orufi02g37680.6 0.332 0.992 0.66 0.022 0.879	1<br>Oglab02g33340.1 0.361 1 0.639 0.042 1<br>Oglab03g24170.1 0.003 0.292 0.289 0.004 0.406	1<br>Obart02g36180.3 0.332 0.992 0.66 0.019 0.724<br>Obart03g24380.1 0.003 0.292 0.289 0.004 0.406	1<br>Olong_011081-R1 0.652 1 0.348 0.05 1<br>Olong_011082-R1 0.003 0.294 0.291 0.007 0.752<br>Olong_025373-R1 0.299 0.606 0.307 0.116 0.574	1<br>Opunc02g34450.1 0.655 0.997 0.342 0.432 0.806<br>Opunc03g22570.2 0.003 0.294 0.291 0.007 0.816<br>Opunc04g19490.1 0.368 0.588 0.22 0.521 0.726	1<br>Obrac02g27500.6 0.655 0.997 0.342 0.432 0.806<br>Obrac03g19420.1 0.003 0.292 0.289 0.007 0.81<br>Obrac05g14470.1 0.368 0.588 0.22 0.584 0.771	1<br>Lperr02g29800.1 0.018 0.292 0.274 0.234 0.846<br>Lperr02g30750.1 0.655 0.997 0.342 0.453 0.814<br>Lperr06g23460.1 0.368 0.606 0.238 0.548 0.76	1<br>AT1G76310.1 0.376 0.606 0.23 0.571 0.773<br>AT2G20360.1 0.655 0.997 0.342 0.423 0.801<br>AT5G38650.1 0.003 0.292 0.289 0.007 0.809
Osjap02g41000.1	1<br>Osjap02g41000.1 0.001 1 0.999 0.001 1<br>Osjap02g41000.2 0.001 1 0.999 0.001 1	0	0	0	0	0	0	0	0	0
	0<br>Osjap02g41000.3 0.001 0.654 0.653 0.001 0.954	1<br>Osind02g40480.1 0.017 0.391 0.374 0.017 1<br>Osind06g17750.1 0.402 0.977 0.575 0.006 0.993	1<br>Orufi02g38250.1 0.001 0.392 0.391 0.002 0.995<br>Orufi06g16680.1 0.402 0.977 0.575 0.006 0.993	1<br>Oglab02g33870.1 0.019 0.391 0.372 0.002 1<br>Oglab02g33890.1 0.417 0.977 0.56 0.001 0.992	1<br>Obart02g36720.1 0.001 0.391 0.39 0.002 1<br>Obart06g15760.1 0.402 0.977 0.575 0.006 0.993	1<br>Olong_019934-R1 0.079 0.391 0.312 0.127 1<br>Olong_020342-R1 0.402 0.977 0.575 0.007 0.992	1<br>Opunc02g33950.1 0.019 0.391 0.372 0.002 0.995<br>Opunc06g14370.1 0.402 0.977 0.575 0.006 0.993	1<br>Obrac02g27090.1 0.417 0.977 0.56 0.002 0.99<br>Obrac02g27110.2 0.019 0.391 0.372 0.002 1	1<br>Lperr02g30380.1 0.028 0.392 0.364 0.023 1<br>Lperr06g11770.4 0.407 0.977 0.57 0.033 0.992	1<br>AT3G23430.1 0.417 0.978 0.561 0.001 0.992<br>AT5G28910.2 0.025 0.391 0.366 0.004 1
Osjap02g41000.2	1<br>Osjap02g41000.1 0.001 1 0.999 0.001 1<br>Osjap02g41000.2 0.001 1 0.999 0.001 1	0	0	0	0	0	0	0	0	0
	0<br>Osjap02g41000.3 0.001 0.65 0.649 0.001 0.954	1<br>Osind02g40480.1 0.017 0.396 0.379 0.017 1<br>Osind06g17750.1 0.407 0.977 0.57 0.035 0.993	1<br>Orufi02g38250.2 0.001 0.396 0.395 0.002 1<br>Orufi06g16680.1 0.407 0.977 0.57 0.035 0.993	1<br>Oglab02g33870.1 0.019 0.396 0.377 0.002 1<br>Oglab02g33890.1 0.41 0.977 0.567 0.001 0.992	1<br>Obart02g36720.1 0.001 0.396 0.395 0.002 1<br>Obart06g15760.1 0.407 0.977 0.57 0.035 0.993	1<br>Olong_019934-R1 0.08 0.396 0.316 0.127 1<br>Olong_020342-R1 0.407 0.977 0.57 0.039 0.992	1<br>Opunc02g33950.1 0.019 0.396 0.377 0.002 0.995<br>Opunc06g14370.1 0.403 0.977 0.574 0.019 0.993	1<br>Obrac02g27090.1 0.41 0.977 0.567 0.002 0.99<br>Obrac02g27110.2 0.019 0.396 0.377 0.002 1	1<br>Lperr02g30380.2 0.028 0.396 0.368 0.023 0.997<br>Lperr06g11770.4 0.399 0.977 0.578 0.033 0.992	1<br>AT3G23430.1 0.41 0.978 0.568 0.001 0.992<br>AT5G28910.2 0.025 0.396 0.371 0.004 1
Osjap02g41000.3	1<br>Osjap02g41000.1 0.001 0.954 0.953 0.001 0.654<br>Osjap02g41000.2 0.001 0.954 0.953 0.001 0.65<br>Osjap02g41000.3 0.001 1 0.999 0.001 1	0	0	0	0	0	0	0	0	0
	0<br>Osjap06g18200.1 0.586 0.986 0.4 0.006 0.436	1<br>Osind02g40480.1 0.024 0.57 0.546 0.017 1<br>Osind06g17750.1 0.586 0.986 0.4 0.006 0.436	1<br>Orufi02g38250.1 0.001 0.571 0.57 0.002 0.995<br>Orufi06g16680.1 0.586 0.986 0.4 0.006 0.436	1<br>Oglab02g33870.1 0.028 0.57 0.542 0.002 1<br>Oglab02g33890.3 0.608 1 0.392 0.003 1	1<br>Obart02g36720.1 0.001 0.57 0.569 0.002 1<br>Obart06g15760.1 0.586 0.986 0.4 0.006 0.422	1<br>Olong_019934-R1 0.115 0.57 0.455 0.127 1<br>Olong_023063-R2 0.608 0.986 0.378 0.001 0.412	1<br>Opunc02g33950.1 0.028 0.57 0.542 0.002 0.995<br>Opunc06g14370.1 0.586 0.986 0.4 0.006 0.436	1<br>Obrac02g27110.2 0.028 0.57 0.542 0.002 1<br>Obrac06g10650.1 0.584 0.986 0.402 0.006 0.433	1<br>Lperr02g30380.1 0.041 0.571 0.53 0.023 1<br>Lperr06g11770.2 0.593 0.986 0.393 0.031 0.451	1<br>AT1G68740.1 0.608 0.986 0.378 0.001 0.394<br>AT5G28960.1 0.037 0.57 0.533 0.004 1

Osjap03g00680.1	1<br>Osjap03g00680.1 0.001 1 0.999 0.001 1<br>Osjap03g00680.2 0.001 1 0.999 0.001 1<br>Osjap03g00680.3 0.001 1 0.999 0.001 1	1<br>Osind03g00740.1 0.001 0.994 0.993 0.001 0.996	1<br>Orufi03g12660.1 0.001 1 0.999 0.001 1<br>Orufi03g12660.2 0.001 1 0.999 0.001 1	1<br>Oglab03g00390.1 0.001 1 0.999 0.001 1	1<br>Obart03g00550.1 0.001 0.994 0.993 0.001 0.996	0	0	0	0	0
	1<br>Osjap02g42090.1 0.051 0.661 0.61 0.065 1<br>Osjap12g08580.1 0.71 0.999 0.289 0.011 0.996	1<br>Osind02g41670.1 0.06 0.661 0.601 0.131 1<br>Osind03g02220.1 0.7 1 0.3 0.015 1	1<br>Orufi02g39350.1 0.06 0.661 0.601 0.131 1<br>Orufi09g02220.2 0.746 1 0.254 0.095 1	1<br>Oglab02g34920.1 0.06 0.661 0.601 0.131 1<br>Oglab03g02050.1 0.688 1 0.312 0.187 1	1<br>Obart02g37790.1 0.06 0.632 0.572 0.133 0.977<br>Obart12g06510.1 0.685 0.999 0.314 0.066 0.997	0<br>Olong_013175-R1 0.264 0.904 0.64 0.002 0.961	1<br>Opunc03g00470.1 0.001 0.783 0.782 0.002 1<br>Opunc11g04590.1 0.783 0.998 0.215 0.055 1	1<br>Obrac02g10260.1 0.712 0.999 0.287 0.265 0.995<br>Obrac02g27970.1 0.134 0.661 0.527 0.105 1	1<br>Lperr03g00570.1 0.001 0.657 0.656 0.002 0.994<br>Lperr04g11360.1 0.752 0.998 0.246 0.415 0.998	0<br>AT5G04460.1 0.017 0.661 0.644 0.081 1
Osjap03g00680.2	1<br>Osjap03g00680.1 0.001 1 0.999 0.001 1<br>Osjap03g00680.2 0.001 1 0.999 0.001 1<br>Osjap03g00680.3 0.001 1 0.999 0.001 1	1<br>Osind03g00740.1 0.001 0.994 0.993 0.001 0.996	1<br>Orufi03g12660.1 0.001 1 0.999 0.001 1<br>Orufi03g12660.2 0.001 1 0.999 0.001 1	1<br>Oglab03g00390.1 0.001 1 0.999 0.001 1	1<br>Obart03g00550.1 0.001 0.994 0.993 0.001 0.996	0	0	0	0	0
	1<br>Osjap02g42090.1 0.052 0.68 0.628 0.065 1<br>Osjap12g08580.1 0.702 0.999 0.297 0.011 0.996	1<br>Osind02g41670.1 0.062 0.68 0.618 0.131 1<br>Osind03g02220.1 0.691 1 0.309 0.015 1	1<br>Orufi02g39350.1 0.062 0.68 0.618 0.131 1<br>Orufi09g02220.2 0.738 1 0.262 0.095 1	1<br>Oglab02g34920.1 0.062 0.68 0.618 0.131 1<br>Oglab03g02050.1 0.691 1 0.309 0.214 1	1<br>Obart02g37790.1 0.062 0.65 0.588 0.133 0.977<br>Obart12g06510.1 0.681 0.999 0.318 0.079 0.997	0<br>Olong_013175-R1 0.272 0.902 0.63 0.002 0.961	1<br>Opunc03g00470.1 0.001 0.777 0.776 0.002 1<br>Opunc03g03130.2 0.777 0.999 0.222 0.443 0.997	1<br>Obrac02g10260.1 0.704 0.999 0.295 0.265 0.995<br>Obrac02g27970.1 0.138 0.68 0.542 0.105 1	1<br>Lperr03g00570.1 0.001 0.68 0.679 0.002 1<br>Lperr04g11360.1 0.745 0.998 0.253 0.415 0.998	0<br>AT5G04460.1 0.018 0.68 0.662 0.081 1
Osjap03g00710.1	1<br>Osjap03g00710.1 0.002 1 0.998 0.002 1	0	1<br>Orufi03g12690.1 0.002 1 0.998 0.002 1<br>Orufi03g12690.2 0.002 1 0.998 0.002 1	0	0	0	0	0	0	0
	1<br>Osjap08g14010.2 0.424 0.983 0.559 0.444 0.966<br>Osjap10g20350.1 0.002 0.245 0.243 0.15 0.969	1<br>Osind03g00760.1 0.316 0.994 0.678 0.002 0.841<br>Osind03g00790.1 0.002 0.28 0.278 0.006 0.879	1<br>Orufi08g14730.1 0.424 0.983 0.559 0.503 0.97<br>Orufi10g18610.1 0.002 0.245 0.243 0.15 0.969	0<br>Oglab03g00420.2 0.234 0.994 0.76 0.128 0.895	1<br>Obart03g00580.1 0.316 1 0.684 0.003 0.966<br>Obart03g00600.1 0.002 0.28 0.278 0.006 0.879	1<br>Olong_002892-R1 0.002 0.245 0.243 0.16 0.969<br>Olong_013172-R3 0.355 0.998 0.643 0.201 0.985	1<br>Opunc03g00510.2 0.316 0.994 0.678 0.003 0.891<br>Opunc03g00530.1 0.002 0.237 0.235 0.008 0.977	1<br>Obrac03g00430.2 0.355 0.993 0.638 0.213 0.962<br>Obrac10g11260.2 0.002 0.237 0.235 0.182 0.929	1<br>Lperr03g00610.3 0.316 0.987 0.671 0.004 0.948<br>Lperr10g13450.1 0.006 0.245 0.239 0.167 0.969	1<br>AT3G52180.1 0.316 0.993 0.677 0.003 0.982<br>AT5G03455.1 0.002 0.237 0.235 0.116 0.979
Osjap03g11290.1	1<br>Osjap03g11290.1 0.004 1 0.996 0.004 1	0	0	0	1<br>Obart03g10480.1 0.004 1 0.996 0.004 1	0	0	0	0	0
	0<br>Osjap01g06090.1 0.495 0.996 0.501 0.64 0.945<br>Osjap03g11310.1 0.004 0.283 0.279 0.005 0.398	1<br>Osind03g11120.1 0.004 0.509 0.505 0.007 1<br>Osind03g11140.1 0.527 1 0.473 0.668 0.954	1<br>Orufi03g10700.1 0.004 0.509 0.505 0.007 1<br>Orufi03g10740.1 0.527 1 0.473 0.664 0.947	1<br>Oglab01g01190.1 0.534 0.996 0.462 0.656 0.959<br>Oglab03g10450.1 0.004 0.509 0.505 0.007 1	1<br>Obart01g05330.1 0.495 0.996 0.501 0.64 0.945<br>Obart03g10500.1 0.004 0.305 0.301 0.011 0.989	1<br>Olong_001134-R1 0.53 0.828 0.298 0.477 0.77<br>Olong_001982-R1 0.004 0.509 0.505 0.007 1	1<br>Opunc03g10110.1 0.004 0.509 0.505 0.007 1<br>Opunc03g10130.1 0.527 1 0.473 0.686 0.985	0<br>Obrac03g08430.5 0.527 1 0.473 0.284 0.412	0<br>Lperr03g09350.4 0.004 0.509 0.505 0.005 1	0
Osjap03g13100.1	1<br>Osjap03g13100.1 0.001 1 0.999 0.001 1	1<br>Osind03g12930.1 0.001 1 0.999 0.028 1	0	1<br>Oglab03g12120.1 0.001 1 0.999 0.028 1	1<br>Obart03g12120.1 0.001 1 0.999 0.028 1	0	0	0	0	0
	1<br>Osjap04g16240.1 0.632 0.998 0.366 0.082 0.921<br>Osjap12g01140.1 0.084 0.622 0.538 0.498 0.981	1<br>Osind04g14790.1 0.632 0.998 0.366 0.013 0.911<br>Osind12g01290.1 0.084 0.622 0.538 0.49 0.981	1<br>Orufi06g01460.3 0.632 0.998 0.366 0.212 0.912<br>Orufi12g01130.1 0.084 0.622 0.538 0.498 0.981	1<br>Oglab06g01620.1 0.632 0.998 0.366 0.233 0.912<br>Oglab12g00800.1 0.089 0.622 0.533 0.483 0.983	1<br>Obart06g01490.1 0.632 0.998 0.366 0.233 0.912<br>Obart11g01060.1 0.089 0.622 0.533 0.471 0.982	1<br>Olong_007714-R9 0.632 1 0.368 0.002 1<br>Olong_007715-R1 0.1 0.623 0.523 0.007 0.965	1<br>Opunc01g28650.1 0.636 0.998 0.362 0.094 0.923<br>Opunc03g11680.1 0.001 0.623 0.622 0.001 0.971	1<br>Obrac03g09720.2 0.001 0.623 0.622 0.001 0.97<br>Obrac04g09430.1 0.63 0.998 0.368 0.014 0.863	1<br>Lperr03g10720.1 0.001 0.623 0.622 0.001 0.934<br>Lperr03g17470.1 0.623 0.998 0.375 0.119 0.828	1<br>AT1G20230.1 0.635 0.998 0.363 0.104 0.833<br>AT3G46740.1 0.097 0.623 0.526 0.158 0.971
Osjap03g14020.1	1<br>Osjap03g14020.1 0.001 1 0.999 0.001 1	0	0	0	0	0	0	0	0	0
	0<br>Osjap01g07780.1 0.005 0.564 0.559 0.046 0.874	1<br>Osind03g13930.1 0.621 1 0.379 0.002 1<br>Osind08g13600.1 0.006 0.57 0.564 0.02 0.948	1<br>Orufi03g13600.1 0.621 1 0.379 0.002 1<br>Orufi03g13610.2 0.001 0.571 0.57 0.002 0.955	0<br>Oglab03g20090.1 0.007 0.633 0.626 0.074 0.748	1<br>Obart01g06740.1 0.005 0.564 0.559 0.083 0.874<br>Obart03g12970.1 0.621 1 0.379 0.002 1	1<br>Olong_020502-R1 0.668 1 0.332 0.003 1<br>Olong_024721-R1 0.006 0.577 0.571 0.063 0.942	1<br>Opunc03g12540.1 0.695 1 0.305 0.22 1<br>Opunc12g06380.1 0.003 0.572 0.569 0.014 0.968	1<br>Obrac03g10420.2 0.708 0.999 0.291 0.003 1<br>Obrac09g12510.3 0.006 0.586 0.58 0.019 0.985	1<br>Lperr03g11490.1 0.709 0.999 0.29 0.009 1<br>Lperr03g30240.1 0.016 0.563 0.547 0.031 0.847	0<br>AT2G13600.1 0.001 0.597 0.596 0.01 0.983

Osjap03g15380.1	1<br>Osjap03g15380.1 0.001 1 0.999 0.001 1<br>Osjap03g15380.2 0.001 0.997 0.996 0.001 1	0	0	0	0	0	0	0	0	0
	0<br>Osjap03g15380.4 0.296 1 0.704 0.001 1	1<br>Osind03g15480.1 0.001 0.212 0.211 0.004 0.996<br>Osind03g15490.1 0.294 0.997 0.703 0.176 1	1<br>Orufi03g14960.1 0.001 0.212 0.211 0.004 0.996<br>Orufi03g14970.2 0.294 0.997 0.703 0.172 1	1<br>Oglab03g14290.1 0.012 0.213 0.201 0.051 0.934<br>Oglab03g14300.1 0.213 1 0.787 0.012 1	1<br>Obart03g14310.1 0.001 0.212 0.211 0.004 0.996<br>Obart03g14320.1 0.213 1 0.787 0.012 1	0<br>Olong_005229-R1 0.296 0.997 0.701 0.001 1	1<br>Opunc03g13810.1 0.001 0.212 0.211 0.008 0.996<br>Opunc03g13830.1 0.296 0.997 0.701 0.001 1	0<br>Obrac03g11560.3 0.296 0.997 0.701 0.001 1	0<br>Lperr03g12630.1 0.296 0.997 0.701 0.001 1	0
Osjap03g15380.2	1<br>Osjap03g15380.1 0.001 1 0.999 0.001 0.997<br>Osjap03g15380.2 0.001 1 0.999 0.001 1	0	0	0	0	0	0	0	0	0
	0<br>Osjap03g15380.3 0.296 1 0.704 0.001 1	1<br>Osind03g15480.1 0.001 0.213 0.212 0.004 0.996<br>Osind03g15490.1 0.295 1 0.705 0.176 1	1<br>Orufi03g14960.1 0.001 0.213 0.212 0.004 0.996<br>Orufi03g14970.2 0.295 1 0.705 0.172 1	1<br>Oglab03g14290.1 0.012 0.213 0.201 0.051 0.934<br>Oglab03g14300.2 0.213 1 0.787 0.012 1	1<br>Obart03g14310.1 0.001 0.213 0.212 0.004 0.996<br>Obart03g14320.1 0.213 1 0.787 0.012 0.997	0<br>Olong_005229-R1 0.296 1 0.704 0.001 1	1<br>Opunc03g13810.1 0.001 0.213 0.212 0.008 0.996<br>Opunc03g13830.1 0.296 1 0.704 0.001 1	0<br>Obrac03g11560.3 0.296 1 0.704 0.001 1	0<br>Lperr03g12630.1 0.296 1 0.704 0.001 1	0
Osjap03g23720.1	1<br>Osjap03g23720.1 0.001 1 0.999 0.001 1	0	0	0	0	0	0	0	0	0
	0<br>Osjap03g23720.4 0.395 1 0.605 0.002 1	1<br>Osind03g24110.1 0.036 0.369 0.333 0.007 0.987<br>Osind03g24160.1 0.395 1 0.605 0.002 1	1<br>Orufi03g22650.4 0.036 0.383 0.347 0.006 1<br>Orufi03g22660.1 0.395 1 0.605 0.002 1	1<br>Oglab03g21650.1 0.036 0.369 0.333 0.007 0.987<br>Oglab03g21680.1 0.408 1 0.592 0.022 1	0<br>Obart03g21960.3 0.373 1 0.627 0.005 1	1<br>Olong_010543-R1 0.408 0.992 0.584 0.027 0.96<br>Olong_019014-R1 0.064 0.371 0.307 0.009 0.991	1<br>Opunc03g20710.1 0.036 0.368 0.332 0.007 0.966<br>Opunc03g20720.1 0.408 0.978 0.57 0.02 0.949	1<br>Obrac03g17280.1 0.002 0.368 0.366 0.006 0.978<br>Obrac03g17290.1 0.414 0.991 0.577 0.039 0.952	1<br>Lperr03g18780.1 0.036 0.369 0.333 0.007 0.979<br>Lperr03g18790.2 0.428 1 0.572 0.054 1	1<br>AT2G31890.1 0.477 0.995 0.518 0.194 0.994<br>AT3G02080.1 0.039 0.368 0.329 0.014 0.972
Osjap03g29590.1	1<br>Osjap03g29590.1 0.003 1 0.997 0.003 1<br>Osjap03g29590.2 0.003 0.915 0.912 0.004 0.996<br>Osjap03g29590.3 0.003 0.98 0.977 0.003 0.965	0	0	0	0	1<br>Olong_002587-R1 0.003 1 0.997 0.003 0.986	1<br>Opunc03g24820.1 0.056 0.98 0.924 0.004 1	0	0	0
	0<br>Osjap03g29590.4 0.416 1 0.584 0.006 1	1<br>Osind03g30690.1 0.416 0.98 0.564 0.005 0.94<br>Osind03g30700.1 0.003 0.39 0.387 0.008 0.967	1<br>Orufi03g28240.1 0.003 0.39 0.387 0.009 0.965<br>Orufi03g28260.1 0.39 1 0.61 0.07 1	1<br>Oglab03g26860.1 0.416 0.98 0.564 0.005 0.94<br>Oglab03g26870.1 0.003 0.39 0.387 0.008 0.967	1<br>Obart03g27180.1 0.416 0.98 0.564 0.005 0.94<br>Obart03g27190.1 0.003 0.39 0.387 0.008 0.967	0<br>Olong_002588-R1 0.128 0.39 0.262 0.012 0.953	1<br>Opunc03g24820.2 0.416 1 0.584 0.006 1<br>Opunc03g24830.1 0.003 0.39 0.387 0.008 0.961	0<br>Obrac03g20150.5 0.416 0.98 0.564 0.005 0.929	1<br>Lperr03g22510.1 0.003 0.39 0.387 0.008 0.969<br>Lperr03g22520.1 0.423 0.98 0.557 0.011 0.941	0<br>AT1G61280.1 0.567 0.987 0.42 0.073 0.964<br>AT3G29130.2 0.033 0.39 0.357 0.333 0.982
Osjap03g29590.2	1<br>Osjap03g29590.1 0.004 0.996 0.992 0.003 0.915<br>Osjap03g29590.2 0.004 1 0.996 0.004 1<br>Osjap03g29590.3 0.004 0.996 0.992 0.003 0.9	0	0	0	0	1<br>Olong_002587-R1 0.004 0.996 0.992 0.003 0.986	1<br>Opunc03g24820.1 0.061 0.982 0.921 0.004 1	0	0	0
	0<br>Osjap03g29590.4 0.454 1 0.546 0.006 0.86	1<br>Osind03g30690.1 0.454 1 0.546 0.005 0.837<br>Osind03g30700.1 0.004 0.425 0.421 0.008 0.967	1<br>Orufi03g28240.1 0.004 0.425 0.421 0.009 0.965<br>Orufi03g28260.1 0.425 1 0.575 0.07 0.875	1<br>Oglab03g26860.1 0.454 1 0.546 0.005 0.837<br>Oglab03g26870.1 0.004 0.425 0.421 0.008 0.967	1<br>Obart03g27180.1 0.454 1 0.546 0.005 0.837<br>Obart03g27190.1 0.004 0.425 0.421 0.008 0.967	0<br>Olong_002588-R1 0.139 0.425 0.286 0.012 0.953	1<br>Opunc03g24820.4 0.454 1 0.546 0.006 1<br>Opunc03g24830.1 0.004 0.425 0.421 0.008 0.961	0<br>Obrac03g20150.5 0.454 0.996 0.542 0.005 0.831	1<br>Lperr03g22510.1 0.004 0.425 0.421 0.008 0.969<br>Lperr03g22520.1 0.461 1 0.539 0.011 0.805	0<br>AT2G39445.1 0.618 0.993 0.375 0.309 0.856<br>AT3G29130.2 0.036 0.425 0.389 0.333 0.982
Osjap03g29590.3	1<br>Osjap03g29590.1 0.003 0.965 0.962 0.003 0.98	0	0	0	0	1<br>Olong_002587-R1 0.003 0.913 0.91 0.003 0.986	1<br>Opunc03g24820.1 0.055 0.981 0.926 0.004 1	0	0	0

	Osjap03g29590.2 0.003 0.9 0.897 0.004 0.996<br>Osjap03g29590.3 0.003 1 0.997 0.003 1									
	0<br>Osjap03g29590.8 0.41 1 0.59 0.005 1	1<br>Osind03g30690.1 0.41 1 0.59 0.005 1<br>Osind03g30700.1 0.003 0.384 0.381 0.008 0.967	1<br>Orufi03g28240.1 0.003 0.384 0.381 0.009 0.965<br>Orufi03g28260.3 0.41 1 0.59 0.005 1	1<br>Oglab03g26860.1 0.41 1 0.59 0.005 1<br>Oglab03g26870.1 0.003 0.384 0.381 0.008 0.967	1<br>Obart03g27180.1 0.41 1 0.59 0.005 1<br>Obart03g27190.1 0.003 0.384 0.381 0.008 0.967	0<br>Olong_002588-R1 0.126 0.384 0.258 0.012 0.953	1<br>Opunc03g24820.3 0.41 0.968 0.558 0.006 0.989<br>Opunc03g24830.1 0.003 0.384 0.381 0.008 0.961	0<br>Obrac03g20150.5 0.41 0.997 0.587 0.005 0.984	1<br>Lperr03g22510.1 0.003 0.384 0.381 0.008 0.969<br>Lperr03g22520.1 0.416 0.997 0.581 0.011 0.995	0<br>AT2G39445.1 0.558 0.968 0.41 0.309 0.978<br>AT3G29130.2 0.032 0.384 0.352 0.333 0.982
Osjap03g30460.1	1<br>Osjap03g30460.1 0.001 1 0.999 0.001 1	1<br>Osind03g31500.1 0.018 1 0.982 0.031 1	1<br>Orufi03g29110.1 0.018 1 0.982 0.296 1	0	1<br>Obart03g27980.1 0.08 1 0.92 0.016 1<br>Obart03g27980.2 0.08 1 0.92 0.015 1	0	0	0	0	0
	1<br>Osjap03g30380.1 0.547 0.999 0.452 0.059 0.866<br>Osjap12g24400.2 0.083 0.517 0.434 0.012 0.988	1<br>Osind08g14630.1 0.547 0.999 0.452 0.069 0.96<br>Osind12g20440.1 0.083 0.517 0.434 0.01 0.99	1<br>Orufi03g29970.1 0.547 0.999 0.452 0.035 0.998<br>Orufi12g21550.1 0.083 0.517 0.434 0.01 0.99	1<br>Oglab03g27330.1 0.547 1 0.453 0.036 1<br>Oglab12g17460.4 0.083 0.517 0.434 0.012 0.996	1<br>Obart03g27900.1 0.547 0.999 0.452 0.053 0.865<br>Obart12g19300.1 0.083 0.517 0.434 0.01 0.99	1<br>Olong_002926-R1 0.088 0.519 0.431 0.002 0.996<br>Olong_007449-R1 0.547 0.999 0.452 0.058 0.881	1<br>Opunc03g25490.1 0.014 0.528 0.514 0.486 0.8<br>Opunc03g26870.1 0.548 0.999 0.451 0.053 1	0<br>Obrac03g20710.1 0.085 0.519 0.434 0.028 0.997	1<br>Lperr03g23190.1 0.547 0.999 0.452 0.03 0.867<br>Lperr03g23200.1 0.07 0.519 0.449 0.002 0.997	0<br>AT5G36940.1 0.084 0.52 0.436 0.003 0.998
Osjap03g34330.1	1<br>Osjap03g34330.1 0.001 1 0.999 0.001 1	1<br>Osind03g35550.1 0.001 1 0.999 0.039 1	1<br>Orufi03g33060.1 0.001 1 0.999 0.001 1	1<br>Oglab03g30830.1 0.001 1 0.999 0.001 1	1<br>Obart03g31750.1 0.001 1 0.999 0.046 1	0	0	0	0	0
	1<br>Osjap03g39110.1 0.634 1 0.366 0.036 1<br>Osjap10g05370.1 0.012 0.609 0.597 0.1 0.963	1<br>Osind01g46540.1 0.634 0.999 0.365 0.065 0.994<br>Osind10g03770.1 0.012 0.609 0.597 0.11 0.96	1<br>Orufi10g04850.1 0.031 0.609 0.578 0.093 0.963<br>Orufi11g12520.1 0.635 1 0.365 0.038 0.991	1<br>Oglab03g34460.1 0.609 0.997 0.388 0.016 0.993<br>Oglab10g04340.1 0.012 0.609 0.597 0.1 0.963	1<br>Obart03g36200.1 0.634 1 0.366 0.016 0.997<br>Obart10g04600.1 0.014 0.609 0.595 0.106 0.962	1<br>Olong_014796-R1 0.001 0.516 0.515 0.002 0.95<br>Olong_027495-R1 0.609 0.994 0.385 0.005 0.998	1<br>Opunc01g42030.1 0.634 1 0.366 0.28 0.872<br>Opunc03g28940.2 0.001 0.609 0.608 0.002 0.962	1<br>Obrac02g16280.1 0.628 0.981 0.353 0.063 0.949<br>Obrac03g23440.1 0.001 0.609 0.608 0.002 0.964	1<br>Lperr03g26400.1 0.001 0.609 0.608 0.002 0.962<br>Lperr03g30520.1 0.626 1 0.374 0.003 0.997	0<br>AT1G70730.3 0.001 0.609 0.608 0.118 0.967
Osjap03g35910.1	1<br>Osjap03g35910.1 0.001 1 0.999 0.001 1	0	0	0	0	0	0	0	0	0
	0<br>Osjap03g42400.1 0.379 0.982 0.603 0.035 0.841	1<br>Osind03g37100.1 0.393 1 0.607 0.008 1<br>Osind03g37110.1 0.001 0.364 0.363 0.003 1	0<br>Orufi03g34640.1 0.362 1 0.638 0.002 1	1<br>Oglab03g31660.1 0.393 1 0.607 0.008 1<br>Oglab03g31670.1 0.001 0.364 0.363 0.003 1	1<br>Obart03g33260.1 0.001 0.364 0.363 0.003 1<br>Obart03g39130.1 0.379 0.982 0.603 0.035 0.841	1<br>Olong_027249-R2 0.431 0.988 0.557 0.026 0.52<br>Olong_027324-R1 0.069 0.364 0.295 0.004 1	1<br>Opunc03g30460.1 0.001 0.364 0.363 0.004 1<br>Opunc03g36240.1 0.371 0.982 0.611 0.03 0.841	0<br>Obrac03g24640.1 0.431 1 0.569 0.083 1	1<br>Lperr01g30100.1 0.409 0.982 0.573 0.094 0.839<br>Lperr03g27810.1 0.001 0.361 0.36 0.003 0.994	0<br>AT5G40405.1 0.427 0.968 0.541 0.077 0.825
Osjap03g39550.1	1<br>Osjap03g39550.1 0.001 1 0.999 0.001 1	0	0	0	0	0	0	1<br>Obrac03g27240.1 0.001 1 0.999 0.002 1	0	0
	0<br>Osjap04g29790.2 0.453 0.993 0.54 0.042 0.989	1<br>Osind03g40580.1 0.001 0.4 0.399 0.004 1<br>Osind03g40590.1 0.433 1 0.567 0.103 1	1<br>Orufi03g38090.1 0.001 0.4 0.399 0.004 1<br>Orufi03g38100.1 0.455 1 0.545 0.003 1	1<br>Oglab03g34920.1 0.001 0.394 0.393 0.004 0.985<br>Oglab03g34930.1 0.455 1 0.545 0.003 1	1<br>Obart03g36630.1 0.001 0.394 0.393 0.004 0.985<br>Obart04g26380.4 0.444 0.993 0.549 0.03 0.989	0<br>Olong_018096-R5 0.453 0.993 0.54 0.102 0.99	1<br>Opunc03g33620.1 0.001 0.394 0.393 0.004 0.985<br>Opunc04g23380.6 0.434 0.993 0.559 0.024 0.827	0<br>Obrac04g17810.1 0.455 0.993 0.538 0.003 0.992	0<br>Lperr04g21440.4 0.455 0.993 0.538 0.003 0.992	0<br>AT3G14420.1 0.458 1 0.542 0.005 1
Osjap03g40400.1	1<br>Osjap03g40400.1 0.001 1 0.999 0.001 1	0	0	0	0	0	1<br>Opunc03g34420.1 0.025 0.985 0.96 0.001 0.985	0	0	0
	1<br>Osjap07g05740.1 0.583 0.999 0.416 0.003 1<br>Osjap07g05830.1 0.001 0.559 0.558 0.002 0.99	1<br>Osind03g41490.1 0.001 0.559 0.558 0.002 0.99<br>Osind07g04980.1 0.583 0.999 0.416 0.003 1	1<br>Orufi07g04840.1 0.583 0.999 0.416 0.003 1<br>Orufi07g04930.1 0.001 0.559 0.558 0.002 0.99	1<br>Oglab07g04840.1 0.631 0.999 0.368 0.021 1<br>Oglab07g04940.1 0.001 0.559 0.558 0.002 0.99	1<br>Obart03g37340.1 0.001 0.559 0.558 0.003 0.99<br>Obart07g05050.1 0.583 0.999 0.416 0.003 1	0<br>Olong_017306-R1 0.541 0.985 0.444 0.171 0.972	1<br>Opunc07g04870.1 0.583 0.999 0.416 0.003 1<br>Opunc07g04960.1 0.001 0.559 0.558 0.002 0.99	1<br>Obrac03g27810.1 0.001 0.559 0.558 0.002 0.99<br>Obrac07g03140.1 0.583 0.999 0.416 0.003 1	1<br>Lperr03g31580.1 0.575 0.985 0.41 0.203 0.971<br>Lperr03g31590.1 0.001 0.559 0.558 0.002 0.99	1<br>AT3G09630.1 0.003 0.559 0.556 0.007 0.99<br>AT5G20030.1 0.639 0.997 0.358 0.172 0.975
Osjap03g44070.1	1<br>Osjap03g44070.1 0.001 1 0.999 0.001 1	0	1<br>Orufi03g42420.1 0.001 1 0.999 0.001 1	0	0	1<br>Olong_021918-R1 0.036 0.967 0.931 0.001 0.957	1<br>Opunc03g37840.1 0.001 1 0.999 0.001 0.995	0	0	0

						Olong_021918-R2 0.036 0.967 0.931 0.001 0.958				
	1<br>Osjap03g44260.1 0.569 1 0.431 0.062 1<br>Osjap05g21490.1 0.016 0.535 0.519 0.057 0.81	1<br>Osind01g39050.1 0.567 1 0.433 0.029 0.996<br>Osind03g45110.2 0.001 0.54 0.539 0.002 0.991	1<br>Orufi03g42640.1 0.569 1 0.431 0.056 1<br>Orufi05g20190.1 0.016 0.535 0.519 0.058 0.817	1<br>Oglab03g38640.1 0.001 0.54 0.539 0.001 0.991<br>Oglab03g38820.1 0.569 0.97 0.401 0.063 0.949	1<br>Obart03g40680.1 0.001 0.54 0.539 0.001 0.991<br>Obart03g40860.1 0.569 1 0.431 0.056 1	1<br>Olong_002818-R1 0.567 0.999 0.432 0.026 0.996<br>Olong_017812-R1 0.032 0.542 0.51 0.001 0.964	1<br>Opunc06g00320.1 0.569 1 0.431 0.083 0.875<br>Opunc12g13740.1 0.029 0.549 0.52 0.001 1	0<br>Obrac03g30660.1 0.001 0.539 0.538 0.001 0.989	1<br>Lperr01g38560.1 0.569 1 0.431 0.099 1<br>Lperr08g09550.1 0.014 0.536 0.522 0.05 0.981	0<br>AT4G13650.1 0.02 0.537 0.517 0.069 0.907
Osjap03g44530.1	1<br>Osjap03g44530.1 0.001 1 0.999 0.001 1	1<br>Osind03g45590.1 0.001 1 0.999 0.002 1	0	0	0	1<br>Olong_013357-R1 0.001 1 0.999 0.002 1	0	1<br>Obrac03g30850.1 0.007 1 0.993 0.023 1	0	0
	0<br>Osjap07g07980.2 0.004 0.595 0.591 0.225 0.975	1<br>Osind07g07170.1 0.001 0.607 0.606 0.002 0.987<br>Osind08g23560.1 0.729 0.988 0.259 0.317 0.968	1<br>Orufi03g42810.1 0.625 1 0.375 0.004 1<br>Orufi07g06860.4 0.001 0.607 0.606 0.002 0.987	1<br>Oglab03g38980.1 0.618 1 0.382 0.004 1<br>Oglab03g38990.1 0.001 0.595 0.594 0.002 0.968	1<br>Obart03g41020.1 0.618 1 0.382 0.004 1<br>Obart03g41030.1 0.001 0.595 0.594 0.002 0.967	0<br>Olong_020188-R1 0.001 0.607 0.606 0.002 0.987	1<br>Opunc03g38110.1 0.629 1 0.371 0.007 1<br>Opunc07g06610.1 0.001 0.611 0.61 0.002 0.993	0<br>Obrac03g30850.2 0.007 0.595 0.588 0.037 0.969	1<br>Lperr03g34950.2 0.625 1 0.375 0.129 0.987<br>Lperr07g06010.1 0.001 0.611 0.61 0.002 0.991	1<br>AT3G01980.3 0.636 1 0.364 0.017 1<br>AT5G46410.2 0.129 0.592 0.463 0.206 0.991
Osjap04g02830.1	1<br>Osjap04g02830.1 0.001 1 0.999 0.001 1	0	1<br>Orufi04g02580.1 0.054 0.859 0.805 0.403 0.971<br>Orufi04g02650.1 0.001 0.988 0.987 0.254 0.883	0	0	0	0	0	0	0
	1<br>Osjap04g02720.1 0.001 0.531 0.53 0.001 1<br>Osjap04g02730.1 0.545 1 0.455 0.049 1	1<br>Osind01g20610.1 0.545 1 0.455 0.052 1<br>Osind04g02110.1 0.07 0.509 0.439 0.629 1	1<br>Orufi04g02720.1 0.563 0.988 0.425 0.135 0.82<br>Orufi10g04890.1 0.001 0.503 0.502 0.001 0.96	0<br>Oglab12g09560.1 0.05 0.507 0.457 0.619 0.987	1<br>Obart04g01960.1 0.632 1 0.368 0.004 1<br>Obart10g04670.1 0.001 0.503 0.502 0.001 0.931	1<br>Olong_015999-R1 0.563 0.988 0.425 0.135 0.82<br>Olong_024256-R1 0.054 0.528 0.474 0.309 0.998	1<br>Opunc04g01960.1 0.545 0.991 0.446 0.13 0.994<br>Opunc04g02050.1 0.006 0.531 0.525 0.004 1	0<br>Obrac04g01260.1 0.054 0.509 0.455 0.502 1	0<br>Lperr04g14110.1 0.001 0.497 0.496 0.001 0.963	0<br>AT5G45480.1 0.095 0.509 0.414 0.701 0.987
Osjap04g11160.1	1<br>Osjap04g11160.1 0.001 1 0.999 0.001 1	1<br>Osind09g02760.1 0.001 1 0.999 0.001 1	1<br>Orufi04g09840.1 0.001 1 0.999 0.001 1	0	0	0	1<br>Opunc04g07220.1 0.017 0.988 0.971 0.005 1	0	1<br>Lperr04g05910.1 0.064 0.993 0.929 0.022 0.997	0
	1<br>Osjap10g21270.2 0.556 0.988 0.432 0.059 0.976<br>Osjap11g14300.1 0.007 0.518 0.511 0.04 0.878	1<br>Osind08g20650.1 0.007 0.518 0.511 0.04 0.878<br>Osind09g05810.1 0.548 0.978 0.43 0.004 0.868	1<br>Orufi10g19500.1 0.556 0.988 0.432 0.017 0.976<br>Orufi11g12980.1 0.007 0.518 0.511 0.039 0.881	1<br>Oglab09g05260.1 0.548 0.978 0.43 0.004 0.868<br>Oglab11g10800.1 0.007 0.518 0.511 0.04 0.878	1<br>Obart04g08610.1 0.538 1 0.462 0.001 1<br>Obart11g12300.1 0.007 0.518 0.511 0.04 0.878	1<br>Olong_024054-R1 0.577 1 0.423 0.002 1<br>Olong_024055-R1 0.009 0.541 0.532 0.001 0.96	1<br>Opunc10g16660.1 0.556 0.988 0.432 0.053 0.976<br>Opunc11g10540.3 0.007 0.518 0.511 0.087 0.889	1<br>Obrac04g04850.1 0.007 0.549 0.542 0.043 0.975<br>Obrac10g11900.3 0.559 0.983 0.424 0.029 0.935	1<br>Lperr10g14190.1 0.556 0.988 0.432 0.114 0.977<br>Lperr11g10010.2 0.007 0.518 0.511 0.037 0.886	1<br>AT4G18750.1 0.564 0.999 0.435 0.069 0.882<br>AT5G44240.1 0.008 0.518 0.51 0.066 0.889
Osjap04g13840.6	1<br>Osjap04g13840.1 0.043 1 0.957 0.638 1<br>Osjap04g13840.2 0.043 1 0.957 0.65 1<br>Osjap04g13840.3 0.043 1 0.957 0.666 1<br>Osjap04g13840.4 0.043 1 0.957 0.648 1<br>Osjap04g13840.6 0.002 1 0.998 0.002 1	0	0	0	0	0	0	0	0	0
	0<br>Osjap09g21020.1 0.69 0.989 0.299 0.073 0.967	1<br>Osind04g12260.1 0.629 1 0.371 0.006 1<br>Osind04g12270.1 0.043 0.6 0.557 0.727 0.965	1<br>Orufi04g12300.1 0.629 1 0.371 0.004 0.637<br>Orufi04g12310.2 0.002 0.6 0.598 0.727 0.984	1<br>Oglab04g09430.1 0.629 1 0.371 0.006 1<br>Oglab04g09440.2 0.002 0.6 0.598 0.727 0.984	1<br>Obart04g11160.1 0.629 1 0.371 0.006 1<br>Obart04g11170.3 0.043 0.6 0.557 0.728 0.983	1<br>Olong_026127-R1 0.629 1 0.371 0.006 1<br>Olong_026128-R1 0.077 0.6 0.523 0.163 0.887	1<br>Opunc04g09100.1 0.629 1 0.371 0.005 0.775<br>Opunc04g09110.3 0.002 0.6 0.598 0.71 0.934	1<br>Obrac04g05960.1 0.629 1 0.371 0.005 0.865<br>Obrac04g05970.2 0.097 0.6 0.503 0.799 0.96	1<br>Lperr04g07860.1 0.077 0.536 0.459 0.82 0.965<br>Lperr09g16480.1 0.62 0.989 0.369 0.024 0.97	0<br>AT5G10130.1 0.643 1 0.357 0.037 0.988
Osjap04g19980.2	1<br>Osjap04g19980.1 0.001 1 0.999 0.001 1<br>Osjap04g19980.2 0.001 1 0.999 0.001 1	0	0	0	0	0	0	0	0	0
	0<br>Osjap07g05530.1 0.031 0.52 0.489 0.013 0.568	1<br>Osind03g01180.1 0.001 0.485 0.484 0.002 0.955<br>Osind04g18790.1 0.497 1 0.503 0.002 1	1<br>Orufi04g18080.1 0.001 0.496 0.495 0.002 0.995<br>Orufi04g18090.1 0.497 1 0.503 0.002 1	1<br>Oglab04g14980.1 0.001 0.496 0.495 0.002 0.991<br>Oglab04g14990.1 0.496 1 0.504 0.007 1	1<br>Obart04g16660.1 0.001 0.496 0.495 0.002 0.991<br>Obart04g16670.1 0.641 1 0.359 0.24 1	0<br>Olong_004277-R1 0.001 0.496 0.495 0.003 0.99	1<br>Opunc01g42400.1 0.031 0.507 0.476 0.078 0.892<br>Opunc04g14460.1 0.565 1 0.435 0.003 1	1<br>Obrac04g10330.1 0.612 1 0.388 0.046 1<br>Obrac08g01810.1 0.022 0.479 0.457 0.456 0.99	1<br>Lperr04g13310.1 0.508 1 0.492 0.029 1<br>Lperr11g13180.1 0.026 0.496 0.47 0.063 0.982	0<br>AT1G13450.1 0.575 0.995 0.42 0.148 1

Osjap04g27970.1	1<br>Osjap04g27970.1 0.001 1 0.999 0.001 1	0	1<br>Orufi04g25730.1 0.014 0.941 0.927 0.016 0.995	0	0	0	0	0	1<br>Lperr04g19670.1 0.033 0.941 0.908 0.042 0.995	0
	1<br>Osjap02g33010.1 0.481 0.938 0.457 0.087 0.989<br>Osjap02g40270.1 0.073 0.442 0.369 0.204 0.995	1<br>Osind04g27100.1 0.001 0.442 0.441 0.002 1<br>Osind04g27110.1 0.481 0.939 0.458 0.089 0.987	1<br>Orufi02g30690.1 0.481 0.938 0.457 0.087 0.989<br>Orufi02g37650.1 0.073 0.442 0.369 0.204 0.995	1<br>Oglab04g22120.1 0.014 0.442 0.428 0.032 1<br>Oglab04g22130.1 0.481 0.939 0.458 0.089 0.987	1<br>Obart04g23980.1 0.001 0.442 0.441 0.002 1<br>Obart04g23990.1 0.481 0.939 0.458 0.089 0.987	1<br>Olong_003249-R1 0.481 0.938 0.457 0.087 0.989<br>Olong_008382-R1 0.02 0.414 0.394 0.064 0.969	1<br>Opunc04g21520.1 0.014 0.442 0.428 0.032 1<br>Opunc04g21530.1 0.481 0.939 0.458 0.089 0.987	1<br>Obrac04g16300.1 0.016 0.442 0.426 0.039 1<br>Obrac04g16310.1 0.481 0.939 0.458 0.089 0.987	1<br>Lperr02g23670.1 0.481 0.938 0.457 0.087 0.989<br>Lperr02g29790.1 0.073 0.442 0.369 0.23 0.995	1<br>AT1G80480.1 0.073 0.442 0.369 0.194 1<br>AT5G27080.1 0.474 0.935 0.461 0.017 0.927
Osjap04g30120.1	1<br>Osjap04g30120.1 0.001 1 0.999 0.001 1<br>Osjap04g30120.3 0.001 1 0.999 0.001 1<br>Osjap04g30120.4 0.001 1 0.999 0.001 1	0	1<br>Orufi04g27870.1 0.001 1 0.999 0.001 1<br>Orufi04g27870.2 0.001 1 0.999 0.001 1	0	1<br>Obart04g26710.1 0.001 1 0.999 0.001 1<br>Obart04g26710.2 0.001 1 0.999 0.001 1	1<br>Olong_014711-R2 0.001 0.849 0.848 0.001 0.977	0	1<br>Obrac04g18040.3 0.001 1 0.999 0.001 1	0	0
	1<br>Osjap04g16240.1 0.007 0.623 0.616 0.07 0.895<br>Osjap05g24420.1 0.657 0.996 0.339 0.024 0.993	0<br>Osind04g29430.1 0.001 0.616 0.615 0.002 0.991	1<br>Orufi05g23020.1 0.657 0.996 0.339 0.024 0.993<br>Orufi10g14810.1 0.007 0.632 0.625 0.016 0.827	1<br>Oglab06g14840.1 0.01 0.632 0.622 0.059 0.882<br>Oglab11g19190.1 0.648 1 0.352 0.042 1	1<br>Obart05g21760.1 0.657 0.996 0.339 0.024 0.993<br>Obart12g15260.1 0.007 0.625 0.618 0.001 0.685	0<br>Olong_014711-R1 0.001 0.645 0.644 0.002 0.837	1<br>Opunc04g23700.2 0.633 1 0.367 0.003 1<br>Opunc05g09290.1 0.001 0.63 0.629 0.096 0.861	0<br>Obrac04g07720.1 0.007 0.633 0.626 0.076 0.934	1<br>Lperr07g00250.2 0.665 1 0.335 0.015 0.99<br>Lperr10g10450.1 0.007 0.632 0.625 0.009 0.826	1<br>AT3G20730.1 0.006 0.623 0.617 0.023 0.998<br>AT4G11290.1 0.643 1 0.357 0.012 1
Osjap04g30680.1	1<br>Osjap04g30680.1 0.001 1 0.999 0.001 1<br>Osjap04g30680.2 0.001 1 0.999 0.001 1	0	0	0	0	0	0	0	0	0
	0<br>Osjap11g01970.1 0.005 0.367 0.362 0.043 0.899	1<br>Osind04g29890.1 0.48 1 0.52 0.002 1<br>Osind04g29900.1 0.001 0.42 0.419 0.002 1	1<br>Orufi04g28360.1 0.48 1 0.52 0.002 1<br>Orufi04g28370.1 0.001 0.42 0.419 0.002 1	1<br>Oglab04g23190.1 0.48 1 0.52 0.002 1<br>Oglab04g23200.1 0.001 0.42 0.419 0.002 1	1<br>Obart04g25100.1 0.48 1 0.52 0.002 1<br>Obart04g25110.1 0.001 0.42 0.419 0.002 1	1<br>Olong_018824-R1 0.421 1 0.579 0.001 1<br>Olong_027971-R1 0.005 0.286 0.281 0.104 0.729	1<br>Opunc04g24220.1 0.48 1 0.52 0.002 1<br>Opunc04g24230.1 0.001 0.42 0.419 0.002 1	1<br>Obrac04g18400.1 0.462 1 0.538 0.061 1<br>Obrac04g18410.1 0.001 0.42 0.419 0.002 1	1<br>Lperr04g22400.1 0.466 1 0.534 0.122 1<br>Lperr04g22410.1 0.001 0.42 0.419 0.002 1	1<br>AT4G39690.1 0.48 1 0.52 0.002 1<br>AT5G10060.1 0.003 0.332 0.329 0.006 0.836
Osjap04g30680.2	1<br>Osjap04g30680.1 0.001 1 0.999 0.001 1<br>Osjap04g30680.2 0.001 1 0.999 0.001 1	0	0	0	0	0	0	0	0	0
	0<br>Osjap11g01970.1 0.005 0.375 0.37 0.043 0.899	1<br>Osind04g29890.1 0.491 1 0.509 0.002 1<br>Osind04g29900.1 0.001 0.43 0.429 0.002 1	1<br>Orufi04g28360.1 0.491 1 0.509 0.002 1<br>Orufi04g28370.1 0.001 0.43 0.429 0.002 1	1<br>Oglab04g23190.1 0.491 1 0.509 0.002 1<br>Oglab04g23200.1 0.001 0.43 0.429 0.002 1	1<br>Obart04g25100.1 0.491 1 0.509 0.002 1<br>Obart04g25110.1 0.001 0.43 0.429 0.002 1	1<br>Olong_018824-R1 0.431 1 0.569 0.001 1<br>Olong_027971-R1 0.005 0.292 0.287 0.104 0.729	1<br>Opunc04g24220.1 0.491 1 0.509 0.002 1<br>Opunc04g24230.1 0.001 0.43 0.429 0.002 1	1<br>Obrac04g18400.1 0.473 1 0.527 0.061 1<br>Obrac04g18410.1 0.001 0.43 0.429 0.002 1	1<br>Lperr04g22400.1 0.477 1 0.523 0.122 1<br>Lperr04g22410.1 0.001 0.43 0.429 0.002 1	1<br>AT4G39690.1 0.491 1 0.509 0.002 1<br>AT5G10060.1 0.003 0.34 0.337 0.006 0.836
Osjap04g34120.1	1<br>Osjap04g34120.1 0.001 1 0.999 0.001 1	0	0	0	0	0	0	0	0	0
	0<br>Osjap04g34120.5 0.438 1 0.562 0.056 1	1<br>Osind04g33370.1 0.33 1 0.67 0.007 1<br>Osind04g33380.1 0.001 0.329 0.328 0.004 1	1<br>Orufi04g31100.2 0.001 0.329 0.328 0.004 1<br>Orufi04g31670.1 0.33 0.986 0.656 0.007 0.973	1<br>Oglab04g28050.2 0.33 1 0.67 0.007 1<br>Oglab04g28060.1 0.001 0.329 0.328 0.004 1	1<br>Obart04g30010.1 0.33 1 0.67 0.007 1<br>Obart04g30020.1 0.001 0.329 0.328 0.004 1	1<br>Olong_001885-R1 0.001 0.329 0.328 0.007 1<br>Olong_001886-R1 0.438 1 0.562 0.061 1	1<br>Opunc04g26880.2 0.001 0.329 0.328 0.004 1<br>Opunc04g27490.1 0.352 1 0.648 0.046 1	0<br>Obrac04g20700.2 0.006 0.329 0.323 0.011 1<br>Obrac04g21200.1 0.445 0.894 0.449 0.041 0.546	1<br>Lperr04g24800.2 0.001 0.329 0.328 0.222 1<br>Lperr04g25470.1 0.447 1 0.553 0.073 1	1<br>AT3G59950.1 0.482 1 0.518 0.134 1<br>AT5G18400.2 0.001 0.329 0.328 0.015 1
Osjap04g35010.1	1<br>Osjap04g35010.1 0.003 1 0.997 0.003 1	0	0	0	0	0	0	0	0	0
	1<br>Osjap03g18110.1 0.003 0.396 0.393 0.09 0.763	0<br>Osind04g34350.1 0.149 0.941 0.792 0.071 0.987	1<br>Orufi04g32660.1 0.446 0.941 0.495 0.282 0.986	1<br>Oglab08g00300.1 0.452 0.947 0.495 0.705 0.917	1<br>Obart04g30900.1 0.446 0.977 0.531 0.725 0.975	0<br>Olong_012571-R1 0.452 0.947 0.495 0.677 0.909	1<br>Opunc02g17940.1 0.003 0.366 0.363 0.101 0.797	0<br>Obrac08g00850.1 0.452 0.947 0.495 0.704 0.917	0<br>Lperr08g01210.1 0.452 0.947 0.495 0.384 0.499	0

	Osjap08g00940.1 0.452 0.947 0.495 0.705 0.917		Orufi06g15410.1 0.003 0.409 0.406 0.004 0.525	Oglab08g09550.1 0.003 0.389 0.386 0.095 0.893	Obart06g16190.1 0.003 0.403 0.4 0.004 0.447		Opunc08g00870.1 0.452 0.947 0.495 0.372 0.485	Obrac11g05680.1 0.003 0.221 0.218 0.008 0.626		
Osjap05g00220.1	1<br>Osjap05g00220.1 0.002 1 0.998 0.002 1	0	1<br>Orufi05g00210.1 0.002 1 0.998 0.002 1	0	0	0	0	0	0	0
	0<br>Osjap06g12050.3 0.512 0.901 0.389 0.502 0.854	1<br>Osind05g00160.1 0.349 1 0.651 0.088 1<br>Osind05g00170.1 0.002 0.322 0.32 0.005 0.777	0<br>Orufi01g00190.1 0.055 0.32 0.265 0.018 0.112<br>Orufi08g24310.1 0.636 0.998 0.362 0.375 0.997	1<br>Oglab05g00170.1 0.349 1 0.651 0.081 0.919<br>Oglab05g00180.1 0.002 0.335 0.333 0.005 0.825	1<br>Obart05g00180.1 0.349 1 0.651 0.081 0.919<br>Obart05g00190.1 0.002 0.335 0.333 0.005 0.825	1<br>Olong_004875-R2 0.349 0.998 0.649 0.088 0.997<br>Olong_004876-R1 0.002 0.335 0.333 0.005 0.826	1<br>Opunc05g00160.1 0.358 0.998 0.64 0.076 0.841<br>Opunc05g00170.1 0.002 0.335 0.333 0.005 0.83	1<br>Obrac01g00170.1 0.055 0.356 0.301 0.126 0.879<br>Obrac05g00210.1 0.404 1 0.596 0.003 1	0<br>Lperr05g00200.1 0.36 0.857 0.497 0.122 0.996<br>Lperr05g00210.2 0.053 0.335 0.282 0.135 0.829	0<br>AT5G14030.1 0.017 0.356 0.339 0.046 0.882<br>AT5G50915.1 0.486 0.838 0.352 0.213 0.762
Osjap05g00530.1	1<br>Osjap05g00530.1 0.001 1 0.999 0.001 1<br>Osjap05g00530.2 0.001 1 0.999 0.001 1<br>Osjap05g00530.3 0.001 1 0.999 0.001 1<br>Osjap05g00530.4 0.001 1 0.999 0.001 1	1<br>Osind05g00510.1 0.131 1 0.869 0.001 1	1<br>Orufi05g00500.1 0.001 1 0.999 0.001 1	0	1<br>Obart05g00520.1 0.084 0.999 0.915 0.001 0.993<br>Obart05g00520.2 0.084 1 0.916 0.001 1	1<br>Olong_022405-R1 0.131 1 0.869 0.001 1	0	0	0	0
	1<br>Osjap02g42390.1 0.422 0.993 0.571 0.001 0.983<br>Osjap07g27300.1 0.068 0.363 0.295 0.072 0.834	1<br>Osind02g41800.1 0.389 0.995 0.606 0.004 0.994<br>Osind06g29470.1 0.058 0.363 0.305 0.1 0.846	1<br>Orufi02g39480.2 0.389 0.994 0.605 0.004 0.992<br>Orufi07g24760.1 0.068 0.363 0.295 0.072 0.834	1<br>Oglab05g00490.2 0.375 1 0.625 0.099 0.996<br>Oglab05g00500.1 0.001 0.365 0.364 0.002 0.982	1<br>Obart06g01490.1 0.1 0.367 0.267 0.2 0.99<br>Obart09g15270.1 0.407 1 0.593 0.029 1	1<br>Olong_023813-R1 0.401 0.997 0.596 0.001 0.976<br>Olong_026414-R1 0.085 0.363 0.278 0.071 0.826	1<br>Opunc05g00480.1 0.397 0.999 0.602 0.007 0.998<br>Opunc05g00490.1 0.008 0.367 0.359 0.037 0.986	0<br>Obrac05g00480.2 0.062 0.363 0.301 0.093 0.998	1<br>Lperr04g17270.1 0.392 1 0.608 0.002 1<br>Lperr07g20540.1 0.052 0.363 0.311 0.079 0.819	0<br>AT4G37380.1 0.009 0.363 0.354 0.043 0.85
Osjap05g00530.2	1<br>Osjap05g00530.1 0.001 1 0.999 0.001 1<br>Osjap05g00530.2 0.001 1 0.999 0.001 1<br>Osjap05g00530.3 0.001 1 0.999 0.001 1<br>Osjap05g00530.4 0.001 1 0.999 0.001 1	1<br>Osind05g00510.1 0.16 1 0.84 0.001 1	1<br>Orufi05g00500.1 0.001 1 0.999 0.001 1	0	1<br>Obart05g00520.1 0.103 0.999 0.896 0.001 0.993<br>Obart05g00520.2 0.103 1 0.897 0.001 1	1<br>Olong_022405-R1 0.16 1 0.84 0.001 1	0	0	0	0
	1<br>Osjap07g27300.1 0.084 0.445 0.361 0.072 0.834<br>Osjap09g15690.3 0.487 1 0.513 0.003 1	1<br>Osind06g29470.1 0.071 0.446 0.375 0.1 0.846<br>Osind09g15830.1 0.487 1 0.513 0.029 1	1<br>Orufi07g24760.1 0.084 0.445 0.361 0.072 0.834<br>Orufi09g16480.1 0.487 1 0.513 0.029 1	1<br>Oglab05g00500.1 0.001 0.449 0.448 0.002 0.982<br>Oglab09g13820.1 0.486 1 0.514 0.093 1	1<br>Obart06g01490.1 0.123 0.451 0.328 0.2 0.99<br>Obart09g15270.1 0.487 1 0.513 0.029 1	1<br>Olong_020432-R1 0.074 0.445 0.371 0.013 0.62<br>Olong_025239-R1 0.486 1 0.514 0.029 1	1<br>Opunc02g35220.2 0.487 0.993 0.506 0.294 0.984<br>Opunc05g00490.1 0.01 0.45 0.44 0.037 0.986	0<br>Obrac05g00480.2 0.077 0.446 0.369 0.093 0.998	1<br>Lperr05g00500.1 0.486 1 0.514 0.022 0.996<br>Lperr07g20540.1 0.064 0.445 0.381 0.079 0.819	0<br>AT4G37380.1 0.011 0.445 0.434 0.043 0.85
Osjap05g00530.3	1<br>Osjap05g00530.1 0.001 1 0.999 0.001 1<br>Osjap05g00530.2 0.001 1 0.999 0.001 1<br>Osjap05g00530.3 0.001 1 0.999 0.001 1<br>Osjap05g00530.4 0.001 1 0.999 0.001 1	1<br>Osind05g00510.1 0.161 1 0.839 0.001 1	1<br>Orufi05g00500.1 0.001 1 0.999 0.001 1	0	1<br>Obart05g00520.1 0.104 0.999 0.895 0.001 0.993<br>Obart05g00520.2 0.104 1 0.896 0.001 1	1<br>Olong_022405-R1 0.161 1 0.839 0.001 1	0	0	0	0
	1<br>Osjap07g27300.1 0.084 0.46 0.376 0.072 0.873<br>Osjap09g15690.3 0.487 1 0.513 0.003 1	1<br>Osind08g09120.1 0.078 0.456 0.378 0.168 0.613<br>Osind09g15830.1 0.487 1 0.513 0.029 1	1<br>Orufi07g24760.1 0.084 0.46 0.376 0.072 0.873<br>Orufi09g16480.1 0.487 1 0.513 0.029 1	1<br>Oglab05g00500.1 0.001 0.456 0.455 0.002 0.993<br>Oglab09g13820.1 0.487 1 0.513 0.093 1	1<br>Obart06g01490.1 0.123 0.45 0.327 0.2 0.982<br>Obart09g15270.1 0.487 1 0.513 0.029 1	1<br>Olong_020432-R1 0.074 0.481 0.407 0.013 0.666<br>Olong_023813-R1 0.482 0.997 0.515 0.004 0.976	1<br>Opunc02g35220.2 0.485 0.993 0.508 0.294 0.984<br>Opunc05g00490.1 0.01 0.456 0.446 0.037 0.993	0<br>Obrac05g00480.2 0.077 0.448 0.371 0.093 0.998	1<br>Lperr05g00500.1 0.487 1 0.513 0.022 0.996<br>Lperr07g20540.1 0.064 0.46 0.396 0.079 0.857	0<br>AT4G37380.1 0.011 0.447 0.436 0.043 0.85
Osjap05g00530.4	1<br>Osjap05g00530.1 0.001 1 0.999 0.001 1<br>Osjap05g00530.2 0.001 1 0.999 0.001 1<br>Osjap05g00530.3 0.001 1 0.999 0.001 1<br>Osjap05g00530.4 0.001 1 0.999 0.001 1	1<br>Osind05g00510.1 0.142 1 0.858 0.001 1	1<br>Orufi05g00500.1 0.001 1 0.999 0.001 1	0	1<br>Obart05g00520.1 0.091 0.999 0.908 0.001 0.993<br>Obart05g00520.2 0.091 1 0.909 0.001 1	1<br>Olong_022405-R1 0.142 1 0.858 0.001 1	0	0	0	0

	1<br>Osjap07g27300.1 0.074 0.394 0.32 0.072 0.834<br>Osjap09g15690.3 0.507 1 0.493 0.003 1	1<br>Osind06g29470.1 0.063 0.395 0.332 0.1 0.846<br>Osind09g15830.1 0.442 1 0.558 0.029 1	1<br>Orufi07g24760.1 0.074 0.394 0.32 0.072 0.834<br>Orufi09g16480.1 0.442 1 0.558 0.029 1	1<br>Oglab05g00490.2 0.407 1 0.593 0.099 0.996<br>Oglab05g00500.1 0.001 0.397 0.396 0.002 0.982	1<br>Obart02g34000.1 0.092 0.388 0.296 0.188 0.958<br>Obart09g15270.1 0.442 1 0.558 0.029 1	1<br>Olong_018361-R1 0.442 1 0.558 0.002 1<br>Olong_020432-R1 0.066 0.394 0.328 0.013 0.62	1<br>Opunc05g00480.1 0.431 0.999 0.568 0.007 0.998<br>Opunc05g00490.1 0.009 0.398 0.389 0.037 0.986	0<br>Obrac05g00480.2 0.068 0.395 0.327 0.093 0.998	1<br>Lperr04g17270.1 0.439 1 0.561 0.028 1<br>Lperr07g20540.1 0.056 0.394 0.338 0.079 0.819	0<br>AT4G37380.1 0.009 0.394 0.385 0.043 0.85
Osjap05g02530.1	1<br>Osjap05g02530.1 0.001 1 0.999 0.001 1	1<br>Osind05g02510.1 0.001 0.998 0.997 0.001 0.987	0	1<br>Oglab05g02190.1 0.002 1 0.998 0.079 1	1<br>Obart05g02340.1 0.002 1 0.998 0.076 1	0	0	0	0	0
	1<br>Osjap03g00960.1 0.459 0.994 0.535 0.132 0.996<br>Osjap06g18010.1 0.001 0.338 0.337 0.184 1	1<br>Osind03g01020.1 0.459 0.994 0.535 0.136 0.996<br>Osind06g17540.1 0.001 0.324 0.323 0.003 0.865	1<br>Orufi05g02460.2 0.459 1 0.541 0.003 0.982<br>Orufi06g16520.1 0.001 0.324 0.323 0.002 0.884	1<br>Oglab03g00660.1 0.459 0.994 0.535 0.124 0.996<br>Oglab05g07310.1 0.001 0.324 0.323 0.003 0.935	1<br>Obart03g00820.2 0.459 0.994 0.535 0.022 0.995<br>Obart06g15630.1 0.001 0.324 0.323 0.002 0.955	1<br>Olong_016162-R1 0.001 0.324 0.323 0.002 0.954<br>Olong_026687-R1 0.427 1 0.573 0.038 0.983	1<br>Opunc05g02220.2 0.459 0.999 0.54 0.003 0.979<br>Opunc07g20770.2 0.001 0.324 0.323 0.002 0.832	1<br>Obrac05g01840.1 0.458 0.998 0.54 0.168 1<br>Obrac06g15870.1 0.001 0.326 0.325 0.003 0.84	0<br>Lperr05g02100.1 0.457 0.998 0.541 0.015 0.978	0<br>AT5G15380.1 0.449 0.992 0.543 0.066 0.987
Osjap05g04120.1	1<br>Osjap05g04120.1 0.001 1 0.999 0.001 1<br>Osjap05g04120.2 0.001 1 0.999 0.001 1	1<br>Osind05g04070.1 0.008 1 0.992 0.001 1<br>Osind05g04070.2 0.008 1 0.992 0.001 1	1<br>Orufi05g03950.1 0.001 1 0.999 0.001 1<br>Orufi05g03950.2 0.001 1 0.999 0.001 1	1<br>Oglab05g03490.1 0.001 1 0.999 0.001 1<br>Oglab05g03490.2 0.001 1 0.999 0.001 1	1<br>Obart05g03830.1 0.001 1 0.999 0.001 1<br>Obart05g03830.2 0.001 1 0.999 0.001 1	1<br>Olong_016739-R1 0.074 1 0.926 0.001 1	1<br>Opunc05g03540.1 0.001 1 0.999 0.001 1<br>Opunc05g03540.2 0.001 1 0.999 0.001 1	1<br>Obrac05g02930.1 0.074 1 0.926 0.001 1	0	0
	1<br>Osjap01g27410.1 0.09 0.723 0.633 0.066 0.996<br>Osjap03g15140.5 0.743 1 0.257 0.061 1	1<br>Osind01g28630.1 0.09 0.723 0.633 0.066 0.996<br>Osind03g15230.1 0.79 1 0.21 0.03 1	1<br>Orufi01g26130.1 0.09 0.723 0.633 0.066 0.996<br>Orufi03g14720.1 0.79 1 0.21 0.03 1	1<br>Oglab01g19490.1 0.09 0.723 0.633 0.066 0.996<br>Oglab03g14060.1 0.79 1 0.21 0.03 1	1<br>Obart01g23310.1 0.09 0.723 0.633 0.066 0.996<br>Obart03g14050.1 0.79 1 0.21 0.03 1	1<br>Olong_003347-R2 0.09 0.723 0.633 0.047 0.996<br>Olong_009943-R1 0.79 1 0.21 0.03 1	1<br>Opunc01g23460.1 0.09 0.723 0.633 0.064 0.996<br>Opunc03g13590.1 0.79 1 0.21 0.002 0.545	1<br>Obrac01g17590.1 0.09 0.723 0.633 0.067 0.996<br>Obrac03g11330.1 0.79 1 0.21 0.03 1	0<br>Lperr05g03460.1 0.001 0.721 0.72 0.001 0.993	1<br>AT2G47580.1 0.775 1 0.225 0.008 1<br>AT3G16630.1 0.003 0.721 0.718 0.031 0.992
Osjap05g04120.2	1<br>Osjap05g04120.1 0.001 1 0.999 0.001 1<br>Osjap05g04120.2 0.001 1 0.999 0.001 1	1<br>Osind05g04070.1 0.008 1 0.992 0.001 1<br>Osind05g04070.2 0.008 1 0.992 0.001 1	1<br>Orufi05g03950.1 0.001 1 0.999 0.001 1<br>Orufi05g03950.2 0.001 1 0.999 0.001 1	1<br>Oglab05g03490.1 0.001 1 0.999 0.001 1<br>Oglab05g03490.2 0.001 1 0.999 0.001 1	1<br>Obart05g03830.1 0.001 1 0.999 0.001 1<br>Obart05g03830.2 0.001 1 0.999 0.001 1	1<br>Olong_016739-R1 0.073 1 0.927 0.001 1	1<br>Opunc05g03540.1 0.001 1 0.999 0.001 1<br>Opunc05g03540.2 0.001 1 0.999 0.001 1	1<br>Obrac05g02930.1 0.073 1 0.927 0.001 1	0	0
	1<br>Osjap01g27410.1 0.089 0.726 0.637 0.066 0.996<br>Osjap03g15140.5 0.747 1 0.253 0.061 1	1<br>Osind01g28630.1 0.089 0.726 0.637 0.066 0.996<br>Osind03g15230.1 0.792 1 0.208 0.03 1	1<br>Orufi01g26130.1 0.089 0.726 0.637 0.066 0.996<br>Orufi03g14720.1 0.792 1 0.208 0.03 1	1<br>Oglab01g19490.1 0.089 0.726 0.637 0.066 0.996<br>Oglab03g14060.1 0.792 1 0.208 0.03 1	1<br>Obart01g23310.1 0.089 0.726 0.637 0.066 0.996<br>Obart03g14050.1 0.792 1 0.208 0.03 1	1<br>Olong_003347-R2 0.089 0.726 0.637 0.047 0.996<br>Olong_009943-R1 0.792 1 0.208 0.03 1	1<br>Opunc01g23460.1 0.089 0.726 0.637 0.064 0.996<br>Opunc03g13590.1 0.792 1 0.208 0.002 0.545	1<br>Obrac01g17590.1 0.089 0.726 0.637 0.067 0.996<br>Obrac03g11330.1 0.792 1 0.208 0.03 1	0<br>Lperr05g03460.1 0.001 0.725 0.724 0.001 0.993	1<br>AT2G47580.1 0.778 1 0.222 0.008 1<br>AT3G16630.1 0.003 0.725 0.722 0.031 0.992
Osjap05g14570.1	1<br>Osjap05g14570.1 0.002 1 0.998 0.002 1	0	1<br>Orufi05g13730.1 0.002 1 0.998 0.002 1	0	0	0	1<br>Opunc05g10890.1 0.002 0.832 0.83 0.005 0.949	0	0	0
	1<br>Osjap01g31830.1 0.687 0.937 0.25 0.055 0.334<br>Osjap05g14540.1 0.034 0.683 0.649 0.309 1	1<br>Osind02g39040.1 0.616 0.948 0.332 0.127 0.415<br>Osind05g15150.1 0.002 0.615 0.613 0.009 1	1<br>Orufi09g08190.1 0.029 0.616 0.587 0.071 0.938<br>Orufi12g06770.1 0.626 0.954 0.328 0.154 0.383	0<br>Oglab02g28070.1 0.63 0.973 0.343 0.002 0.402<br>Oglab05g11780.1 0.344 0.611 0.267 0.172 1	1<br>Obart02g30540.1 0.63 0.973 0.343 0.002 0.402<br>Obart05g12690.1 0.002 0.594 0.592 0.005 0.973	1<br>Olong_008352-R1 0.034 0.611 0.577 0.004 1<br>Olong_023139-R1 0.626 0.937 0.311 0.01 0.432	1<br>Opunc04g20130.1 0.626 0.945 0.319 0.007 0.261<br>Opunc05g11350.1 0.032 0.615 0.583 0.147 0.998	0	0<br>Lperr05g09990.1 0.233 0.443 0.21 0.072 0.928	0<br>AT2G02650.1 0.282 0.573 0.291 0.562 1
Osjap05g20040.1	1<br>Osjap05g20040.1 0.001 1 0.999 0.001 1	0	0	0	0	0	0	0	0	0
	0<br>Osjap08g00350.1 0.79 1 0.21 0.111 1	1<br>Osind05g20720.1 0.104 0.744 0.64 0.002 1<br>Osind08g00300.1 0.79 1 0.21 0.111 1	1<br>Orufi05g18840.1 0.104 0.744 0.64 0.002 1<br>Orufi05g18850.1 0.788 1 0.212 0.006 1	1<br>Oglab05g16550.1 0.104 0.744 0.64 0.002 1<br>Oglab05g16560.1 0.788 1 0.212 0.006 1	1<br>Obart05g17640.1 0.076 0.744 0.668 0.156 1<br>Obart05g17650.1 0.788 1 0.212 0.006 1	1<br>Olong_017021-R1 0.788 1 0.212 0.006 1<br>Olong_017022-R1 0.104 0.744 0.64 0.003 1	1<br>Opunc05g15550.1 0.088 0.744 0.656 0.137 1<br>Opunc05g15560.3 0.784 1 0.216 0.253 1	1<br>Obrac05g11810.1 0.104 0.744 0.64 0.002 1<br>Obrac05g11820.1 0.788 1 0.212 0.006 1	1<br>Lperr05g14030.1 0.104 0.744 0.64 0.002 1<br>Lperr08g00390.1 0.788 1 0.212 0.085 1	1<br>AT1G34350.2 0.788 1 0.212 0.005 1<br>AT4G24330.1 0.118 0.742 0.624 0.023 0.994
Osjap05g23780.1	1<br>Osjap05g23780.1 0.001 1 0.999 0.001 1	0	1<br>Orufi05g22360.1 0.001 1 0.999 0.001 1	0	0	0	0	0	1<br>Lperr05g16920.1 0.029 0.999 0.97 0.015 0.977<br>Lperr05g16920.2 0.029 0.999 0.97 0.016 0.975	0

	1<br>Osjap01g39920.1 0.388 0.981 0.593 0.001 0.472<br>Osjap06g31930.1 0.017 0.374 0.357 0.147 0.902	0<br>Osind05g24290.1 0.349 1 0.651 0.113 1	1<br>Orufi01g38030.1 0.388 0.981 0.593 0.001 0.472<br>Orufi06g29370.1 0.017 0.374 0.357 0.147 0.902	1<br>Oglab05g19890.1 0.388 1 0.612 0.002 1<br>Oglab05g19900.1 0.001 0.374 0.373 0.003 0.894	1<br>Obart01g34670.1 0.388 0.981 0.593 0.001 0.465<br>Obart06g27420.1 0.017 0.374 0.357 0.141 0.901	1<br>Olong_004247-R1 0.017 0.374 0.357 0.147 0.902<br>Olong_020609-R1 0.388 0.981 0.593 0.001 0.483	1<br>Opunc05g18780.1 0.375 1 0.625 0.004 1<br>Opunc05g18790.1 0.001 0.374 0.373 0.003 0.895	0<br>Obrac01g26700.1 0.571 0.981 0.41 0.109 0.448<br>Obrac05g14330.1 0.084 0.374 0.29 0.041 0.873	1<br>Lperr01g29930.1 0.388 0.981 0.593 0.001 0.501<br>Lperr06g22970.1 0.017 0.374 0.357 0.164 0.892	0<br>AT2G32760.1 0.017 0.374 0.357 0.091 0.889
Osjap05g26770.1	1<br>Osjap05g26770.1 0.003 1 0.997 0.003 1	0	1<br>Orufi05g25440.1 0.003 1 0.997 0.003 1	0	0	0	0	0	0	0
	0<br>Osjap05g26770.3 0.003 0.609 0.606 0.005 1	1<br>Osind01g35040.1 0.003 0.609 0.606 0.005 1<br>Osind05g27210.1 0.63 1 0.37 0.008 1	1<br>Orufi01g24400.1 0.639 1 0.361 0.008 1<br>Orufi01g32230.1 0.003 0.609 0.606 0.005 1	1<br>Oglab01g24720.1 0.657 1 0.343 0.25 1<br>Oglab01g24730.1 0.003 0.609 0.606 0.005 1	1<br>Obart01g29160.1 0.003 0.609 0.606 0.005 1<br>Obart05g23830.1 0.63 1 0.37 0.008 1	0<br>Olong_006107-R1 0.63 1 0.37 0.008 1<br>Olong_006661-R1 0.185 0.609 0.424 0.007 1	1<br>Opunc05g21240.1 0.003 0.609 0.606 0.005 1<br>Opunc05g21250.2 0.624 1 0.376 0.34 1	0<br>Obrac01g16370.1 0.639 1 0.361 0.008 1<br>Obrac05g16520.1 0.185 0.609 0.424 0.007 1	1<br>Lperr01g24980.1 0.003 0.609 0.606 0.005 1<br>Lperr05g19670.1 0.63 1 0.37 0.008 1	1<br>AT4G26210.1 0.639 0.997 0.358 0.008 0.992<br>AT5G11770.1 0.143 0.609 0.466 0.284 1
Osjap05g28230.1	1<br>Osjap05g28230.1 0.001 1 0.999 0.001 1	1<br>Osind05g28520.1 0.001 1 0.999 0.001 1	1<br>Orufi05g26870.1 0.001 1 0.999 0.001 1	1<br>Oglab05g23160.1 0.001 1 0.999 0.001 1	1<br>Obart05g25070.1 0.001 1 0.999 0.001 1	0	1<br>Opunc05g22640.1 0.001 1 0.999 0.001 1	0	1<br>Lperr01g22990.1 0.001 1 0.999 0.027 1	0
	1<br>Osjap01g31140.1 0.006 0.432 0.426 0.093 0.997<br>Osjap01g31150.1 0.487 0.999 0.512 0.009 0.999	1<br>Osind01g32600.1 0.001 0.431 0.43 0.002 0.995<br>Osind01g32610.1 0.487 0.999 0.512 0.009 0.999	1<br>Orufi01g29880.1 0.001 0.432 0.431 0.075 0.997<br>Orufi01g29890.1 0.487 0.999 0.512 0.009 0.999	1<br>Oglab01g22740.1 0.027 0.432 0.405 0.061 0.996<br>Oglab01g22750.1 0.487 0.999 0.512 0.009 0.999	1<br>Obart01g26870.1 0.005 0.432 0.427 0.117 0.997<br>Obart01g26880.1 0.487 0.999 0.512 0.009 0.999	1<br>Olong_000367-R1 0.1 0.432 0.332 0.002 0.995<br>Olong_000368-R1 0.462 0.999 0.537 0.069 0.999	1<br>Opunc01g26820.1 0.047 0.432 0.385 0.002 0.996<br>Opunc01g26830.1 0.487 1 0.513 0.009 1	1<br>Obrac01g20400.1 0.044 0.432 0.388 0.004 0.996<br>Obrac05g17560.2 0.432 1 0.568 0.109 1	1<br>Lperr05g20850.2 0.491 1 0.509 0.002 1<br>Lperr05g20860.1 0.001 0.432 0.431 0.002 0.996	1<br>AT5G08690.1 0.011 0.431 0.42 0.034 0.995<br>AT5G51600.1 0.487 0.999 0.512 0.008 0.996
Osjap05g28940.1	1<br>Osjap05g28940.1 0.002 1 0.998 0.002 1<br>Osjap05g28940.2 0.002 1 0.998 0.002 1<br>Osjap05g28940.3 0.002 1 0.998 0.002 1<br>Osjap05g28940.4 0.002 0.802 0.8 0.002 0.986	0	0	0	0	0	0	0	0	0
	0<br>Osjap01g10270.1 0.002 0.576 0.574 0.003 0.997	1<br>Osind05g29240.1 0.691 1 0.309 0.004 1<br>Osind05g29250.1 0.002 0.577 0.575 0.003 1	1<br>Orufi05g27610.1 0.691 1 0.309 0.004 1<br>Orufi05g27620.1 0.002 0.577 0.575 0.003 1	1<br>Oglab05g23750.1 0.691 1 0.309 0.004 1<br>Oglab05g23760.1 0.002 0.577 0.575 0.003 1	1<br>Obart01g26130.1 0.691 1 0.309 0.004 1<br>Obart05g25720.1 0.002 0.577 0.575 0.003 1	1<br>Olong_013304-R1 0.002 0.576 0.574 0.003 0.997<br>Olong_014925-R1 0.691 1 0.309 0.004 1	1<br>Opunc05g23320.1 0.691 1 0.309 0.004 1<br>Opunc05g23330.1 0.002 0.577 0.575 0.003 1	1<br>Obrac05g18130.1 0.691 1 0.309 0.004 1<br>Obrac05g18140.1 0.002 0.577 0.575 0.003 1	1<br>Lperr01g22340.3 0.691 1 0.309 0.004 1<br>Lperr05g21370.1 0.002 0.577 0.575 0.003 1	0<br>AT1G59725.1 0.002 0.579 0.577 0.003 1
Osjap05g28940.2	1<br>Osjap05g28940.1 0.002 1 0.998 0.002 1<br>Osjap05g28940.2 0.002 1 0.998 0.002 1<br>Osjap05g28940.3 0.002 1 0.998 0.002 1	0	0	0	0	0	0	0	0	0
	0<br>Osjap05g28940.4 0.002 0.764 0.762 0.002 0.986	1<br>Osind05g29240.1 0.658 1 0.342 0.004 1<br>Osind05g29250.1 0.002 0.55 0.548 0.003 1	1<br>Orufi05g27610.1 0.658 1 0.342 0.004 1<br>Orufi05g27620.1 0.002 0.55 0.548 0.003 1	1<br>Oglab05g23750.1 0.658 1 0.342 0.004 1<br>Oglab05g23760.1 0.002 0.55 0.548 0.003 1	1<br>Obart01g26130.1 0.658 1 0.342 0.004 1<br>Obart05g25720.1 0.002 0.55 0.548 0.003 1	1<br>Olong_013304-R1 0.002 0.549 0.547 0.003 0.997<br>Olong_014925-R1 0.658 1 0.342 0.004 1	1<br>Opunc01g26010.1 0.658 1 0.342 0.004 1<br>Opunc05g23330.1 0.002 0.55 0.548 0.003 1	1<br>Obrac05g18130.1 0.658 1 0.342 0.004 1<br>Obrac05g18140.1 0.002 0.55 0.548 0.003 1	1<br>Lperr01g22340.3 0.658 1 0.342 0.004 1<br>Lperr05g21370.1 0.002 0.55 0.548 0.003 1	0<br>AT1G59725.1 0.002 0.552 0.55 0.003 1
Osjap05g28940.3	1<br>Osjap05g28940.1 0.002 1 0.998 0.002 1<br>Osjap05g28940.2 0.002 1 0.998 0.002 1<br>Osjap05g28940.3 0.002 1 0.998 0.002 1	0	0	0	0	0	0	0	0	0
	0<br>Osjap05g28940.4 0.002 0.796 0.794 0.002 0.986	1<br>Osind05g29240.1 0.685 1 0.315 0.004 1<br>Osind05g29250.1 0.002 0.573 0.571 0.003 1	1<br>Orufi05g27610.1 0.685 1 0.315 0.004 1<br>Orufi05g27620.1 0.002 0.573 0.571 0.003 1	1<br>Oglab05g23750.1 0.685 1 0.315 0.004 1<br>Oglab05g23760.1 0.002 0.573 0.571 0.003 1	1<br>Obart01g26130.1 0.685 1 0.315 0.004 1<br>Obart05g25720.1 0.002 0.573 0.571 0.003 1	1<br>Olong_013304-R1 0.002 0.571 0.569 0.003 0.997<br>Olong_014925-R1 0.685 1 0.315 0.004 1	1<br>Opunc05g23320.1 0.685 1 0.315 0.004 1<br>Opunc05g23330.1 0.002 0.573 0.571 0.003 1	1<br>Obrac05g18130.1 0.685 1 0.315 0.004 1<br>Obrac05g18140.1 0.002 0.573 0.571 0.003 1	1<br>Lperr01g22340.3 0.685 1 0.315 0.004 1<br>Lperr05g21370.1 0.002 0.573 0.571 0.003 1	0<br>AT1G59725.1 0.002 0.574 0.572 0.003 1

Osjap05g29140.1	1<br>Osjap05g29140.1 0.002 1 0.998 0.002 1	0	0	0	0	0	0	0	1<br>Lperr05g21550.1 0.054 1 0.946 0.008 1	0
	0<br>Osjap01g29980.1 0.048 0.499 0.451 0.095 0.992	1<br>Osind05g29480.1 0.002 0.503 0.501 0.004 1<br>Osind05g29520.1 0.673 1 0.327 0.017 1	1<br>Orufi05g27820.1 0.002 0.503 0.501 0.004 1<br>Orufi05g27830.1 0.524 1 0.476 0.209 1	1<br>Oglab05g23960.1 0.002 0.503 0.501 0.004 1<br>Oglab05g23970.1 0.524 1 0.476 0.209 1	1<br>Obart05g25940.1 0.002 0.503 0.501 0.004 1<br>Obart05g25950.1 0.524 1 0.476 0.137 1	1<br>Olong_021573-R1 0.513 1 0.487 0.1 1<br>Olong_021574-R1 0.025 0.503 0.478 0.004 1	1<br>Opunc01g25660.1 0.002 0.499 0.497 0.004 0.97<br>Opunc05g23520.1 0.513 1 0.487 0.119 1	1<br>Obrac01g19470.3 0.002 0.505 0.503 0.004 1<br>Obrac05g18290.1 0.53 1 0.47 0.259 1	0<br>Lperr01g22010.1 0.012 0.505 0.493 0.107 1	1<br>AT2G34470.2 0.513 1 0.487 0.087 1<br>AT4G18260.1 0.062 0.503 0.441 0.07 0.492
Osjap05g29150.1	1<br>Osjap05g29150.1 0.002 1 0.998 0.002 1	0	0	0	0	0	0	0	0	0
	0<br>Osjap05g29150.3 0.002 0.51 0.508 0.003 0.934	1<br>Osind05g29530.1 0.516 1 0.484 0.003 1<br>Osind05g29540.1 0.002 0.51 0.508 0.003 1	1<br>Orufi05g27840.1 0.516 1 0.484 0.003 1<br>Orufi05g27850.1 0.002 0.51 0.508 0.003 1	1<br>Oglab01g25570.1 0.021 0.503 0.482 0.044 0.972<br>Oglab05g23980.1 0.516 1 0.484 0.003 1	1<br>Obart05g25960.1 0.516 1 0.484 0.003 1<br>Obart05g25970.1 0.002 0.51 0.508 0.003 1	1<br>Olong_006430-R1 0.033 0.503 0.47 0.124 0.871<br>Olong_021571-R1 0.516 1 0.484 0.003 1	1<br>Opunc05g23530.1 0.516 1 0.484 0.003 1<br>Opunc05g23540.1 0.013 0.51 0.497 0.056 1	1<br>Obrac05g18300.1 0.516 1 0.484 0.003 1<br>Obrac06g07360.6 0.029 0.508 0.479 0.279 0.49	1<br>Lperr05g21560.1 0.516 1 0.484 0.003 0.993<br>Lperr05g21570.1 0.002 0.51 0.508 0.004 1	0<br>AT1G71170.1 0.016 0.507 0.491 0.017 0.997
Osjap05g30140.4	1<br>Osjap05g30140.4 0.001 1 0.999 0.001 1<br>Osjap05g30140.5 0.001 1 0.999 0.001 1	0	0	0	0	0	0	0	0	0
	0<br>Osjap05g30140.1 0.001 0.629 0.628 0.002 0.862	1<br>Osind05g30480.1 0.63 1 0.37 0.074 1<br>Osind05g30490.1 0.001 0.629 0.628 0.002 0.969	1<br>Orufi01g27170.1 0.63 1 0.37 0.063 1<br>Orufi05g28830.1 0.001 0.629 0.628 0.002 0.969	1<br>Oglab01g20290.1 0.63 1 0.37 0.063 1<br>Oglab05g24830.1 0.001 0.629 0.628 0.002 0.969	1<br>Obart05g26820.1 0.63 1 0.37 0.11 1<br>Obart05g26830.1 0.001 0.629 0.628 0.002 0.969	0<br>Olong_025420-R1 0.16 0.632 0.472 0.139 0.969<br>Olong_027851-R1 0.685 1 0.315 0.137 0.989	1<br>Opunc01g24270.1 0.63 1 0.37 0.064 1<br>Opunc05g24450.1 0.094 0.629 0.535 0.002 0.964	0<br>Obrac04g18520.1 0.762 1 0.238 0.415 1<br>Obrac06g19100.1 0.144 0.632 0.488 0.078 0.969	0<br>Lperr01g10070.1 0.759 1 0.241 0.425 1<br>Lperr05g22520.1 0.094 0.633 0.539 0.003 0.97	1<br>AT3G25640.1 0.692 1 0.308 0.206 1<br>AT5G15860.1 0.095 0.634 0.539 0.028 0.974
Osjap05g30140.5	1<br>Osjap05g30140.4 0.001 1 0.999 0.001 1<br>Osjap05g30140.5 0.001 1 0.999 0.001 1	0	0	0	0	0	0	0	0	0
	0<br>Osjap05g30140.1 0.001 0.604 0.603 0.002 0.862	1<br>Osind05g30480.1 0.605 1 0.395 0.074 1<br>Osind05g30490.1 0.001 0.604 0.603 0.002 0.969	1<br>Orufi01g27170.1 0.605 1 0.395 0.063 1<br>Orufi05g28830.1 0.001 0.604 0.603 0.002 0.969	1<br>Oglab01g20290.1 0.605 1 0.395 0.063 1<br>Oglab05g24830.1 0.001 0.604 0.603 0.002 0.969	1<br>Obart05g26820.1 0.605 1 0.395 0.11 1<br>Obart05g26830.1 0.001 0.604 0.603 0.002 0.969	0<br>Olong_025420-R1 0.171 0.607 0.436 0.139 0.969<br>Olong_027851-R1 0.664 1 0.336 0.137 0.989	1<br>Opunc01g24270.1 0.605 1 0.395 0.064 1<br>Opunc05g24450.1 0.1 0.604 0.504 0.002 0.964	0<br>Obrac04g18520.1 0.746 1 0.254 0.415 1<br>Obrac06g19100.1 0.154 0.607 0.453 0.078 0.969	1<br>Lperr01g20770.1 0.605 1 0.395 0.066 1<br>Lperr05g22520.1 0.1 0.604 0.504 0.003 0.949	1<br>AT3G25640.1 0.671 1 0.329 0.206 1<br>AT5G15860.1 0.102 0.61 0.508 0.028 0.974
Osjap06g00390.1	1<br>Osjap06g00390.1 0.002 1 0.998 0.002 1	0	0	0	0	0	0	0	0	0
	1<br>Osjap06g28340.1 0.002 0.285 0.283 0.523 1<br>Osjap07g16770.1 0.342 0.998 0.656 0.078 0.992	0<br>Osind11g16460.1 0.207 0.992 0.785 0.03 0.982	1<br>Orufi06g00350.1 0.326 1 0.674 0.003 1<br>Orufi06g00360.1 0.002 0.285 0.283 0.007 1	1<br>Oglab06g00360.1 0.326 1 0.674 0.003 1<br>Oglab06g00370.1 0.002 0.285 0.283 0.007 1	1<br>Obart06g00340.1 0.326 1 0.674 0.003 1<br>Obart06g00350.1 0.002 0.289 0.287 0.007 0.987	1<br>Olong_019467-R3 0.002 0.289 0.287 0.054 0.988<br>Olong_019620-R1 0.377 1 0.623 0.061 0.953	1<br>Opunc06g00310.1 0.326 1 0.674 0.003 1<br>Opunc06g00330.1 0.002 0.289 0.287 0.057 0.987	1<br>Obrac06g00310.2 0.326 1 0.674 0.017 1<br>Obrac06g16850.1 0.002 0.285 0.283 0.515 1	1<br>Lperr06g00340.1 0.326 1 0.674 0.003 1<br>Lperr06g20160.1 0.002 0.285 0.283 0.508 0.994	0<br>AT5G47470.1 0.346 1 0.654 0.06 1
Osjap06g00940.1	1<br>Osjap06g00940.1 0.002 1 0.998 0.002 1	0	1<br>Orufi06g00860.1 0.118 1 0.882 0.021 1	1<br>Oglab06g00840.1 0.085 1 0.915 0.04 1	1<br>Obart06g00880.1 0.085 1 0.915 0.04 1	0	0	0	0	0
	1<br>Osjap06g00950.1 0.415 0.99 0.575 0.132 0.99<br>Osjap08g13790.1 0.083 0.327 0.244 0.005 1	1<br>Osind06g00910.1 0.411 1 0.589 0.068 1<br>Osind07g02910.1 0.091 0.325 0.234 0.012 1	1<br>Orufi06g00690.1 0.415 1 0.585 0.099 1<br>Orufi07g02700.1 0.091 0.325 0.234 0.012 1	1<br>Oglab06g00730.1 0.411 0.987 0.576 0.257 0.968<br>Oglab07g02750.1 0.1 0.325 0.225 0.007 1	1<br>Obart06g00690.1 0.415 1 0.585 0.099 1<br>Obart08g12830.1 0.104 0.324 0.22 0.012 0.988	1<br>Olong_006603-R1 0.04 0.324 0.284 0.034 0.987<br>Olong_022685-R1 0.415 1 0.585 0.074 1	1<br>Opunc06g00800.1 0.325 1 0.675 0.025 1<br>Opunc06g00830.1 0.097 0.325 0.228 0.003 1	1<br>Obrac02g00120.1 0.1 0.325 0.225 0.006 1<br>Obrac06g00660.1 0.411 1 0.589 0.034 0.999	0<br>Lperr01g05300.1 0.108 0.325 0.217 0.013 1<br>Lperr06g00740.1 0.415 0.998 0.583 0.073 0.997	0<br>AT1G20140.1 0.112 0.325 0.213 0.018 1

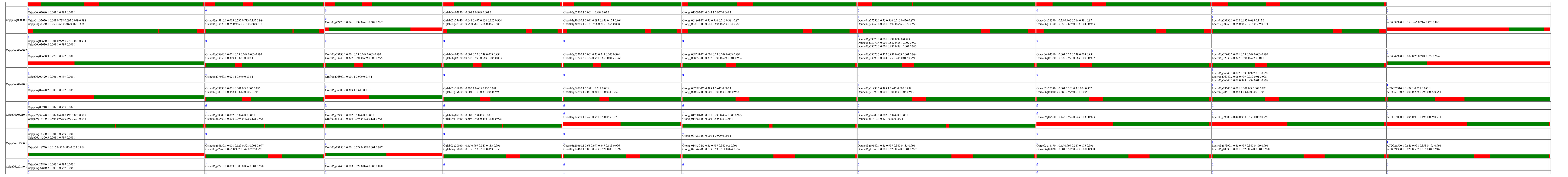

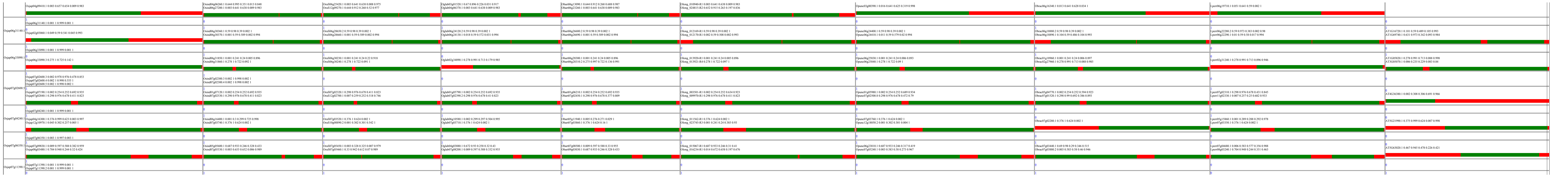

	Osjap07g11390.5 0.001 0.674 0.673 0.001 0.978	Osind07g10300.1 0.001 0.584 0.583 0.001 0.987<br>Osind09g02420.1 0.586 0.889 0.303 0.002 1	Orufi07g09980.2 0.585 0.979 0.394 0.094 0.957<br>Orufi07g09990.1 0.001 0.584 0.583 0.001 0.987	Oglab07g09530.1 0.703 0.979 0.276 0.041 0.938<br>Oglab07g09550.1 0.001 0.584 0.583 0.001 0.987	Obart03g24150.1 0.014 0.566 0.552 0.01 0.834<br>Obart07g09900.1 0.586 0.979 0.393 0.001 0.955	Olong_014668-R1 0.584 0.977 0.393 0.022 0.925<br>Olong_014669-R2 0.001 0.584 0.583 0.151 0.994	Opunc05g18930.2 0.027 0.571 0.544 0.031 0.81<br>Opunc07g09130.1 0.584 0.99 0.406 0.017 0.965	Obrac03g18620.1 0.013 0.566 0.553 0.013 0.829<br>Obrac07g05900.2 0.586 0.978 0.392 0.082 0.939	Lperr01g35150.2 0.011 0.567 0.556 0.01 0.892	AT1G65470.1 0.601 0.968 0.367 0.032 0.87<br>AT3G14470.1 0.025 0.557 0.532 0.032 0.851
Osjap07g11390.2	1<br>Osjap07g11390.1 0.001 1 0.999 0.001 1<br>Osjap07g11390.2 0.001 1 0.999 0.001 1	0	0	0	0	0	0	0	0	0
	0<br>Osjap07g11390.4 0.001 0.67 0.669 0.001 0.98	1<br>Osind07g10300.1 0.001 0.579 0.578 0.001 0.962<br>Osind09g02420.1 0.579 0.887 0.308 0.002 1	1<br>Orufi07g09980.2 0.579 0.979 0.4 0.094 0.957<br>Orufi07g09990.1 0.001 0.579 0.578 0.001 0.962	1<br>Oglab07g09530.1 0.698 0.979 0.281 0.041 0.938<br>Oglab07g09550.1 0.001 0.579 0.578 0.001 0.962	1<br>Obart03g24150.1 0.014 0.575 0.561 0.01 0.834<br>Obart07g09900.1 0.579 0.979 0.4 0.001 0.955	1<br>Olong_014668-R1 0.579 0.977 0.398 0.025 0.925<br>Olong_014669-R2 0.001 0.579 0.578 0.151 0.983	1<br>Opunc05g18930.2 0.028 0.574 0.546 0.031 0.801<br>Opunc07g09130.1 0.579 0.99 0.411 0.019 0.965	0<br>Obrac03g18620.1 0.013 0.575 0.562 0.013 0.829	0<br>Lperr01g35150.2 0.011 0.576 0.565 0.01 0.892	0<br>AT3G14460.1 0.029 0.596 0.567 0.025 0.654
Osjap07g22430.1	1<br>Osjap07g22430.1 0.003 1 0.997 0.003 1	0	1<br>Orufi07g20080.1 0.003 1 0.997 0.099 1	0	1<br>Obart07g19310.1 0.003 1 0.997 0.094 1	0	0	0	0	0
	0<br>Osjap07g22430.2 0.393 1 0.607 0.133 1	1<br>Osind01g03960.1 0.136 0.39 0.254 0.756 0.882<br>Osind07g19410.1 0.393 1 0.607 0.243 1	1<br>Orufi01g30120.1 0.111 0.375 0.264 0.641 0.935<br>Orufi07g20080.2 0.393 1 0.607 0.26 1	1<br>Oglab04g15310.1 0.399 0.991 0.592 0.272 0.988<br>Oglab12g04490.5 0.09 0.387 0.297 0.524 0.987	1<br>Obart04g26170.2 0.111 0.384 0.273 0.201 0.246<br>Obart07g19310.2 0.393 1 0.607 0.243 1	0<br>Olong_001427-R1 0.467 1 0.533 0.006 1<br>Olong_024445-R1 0.115 0.353 0.238 0.413 0.957	1<br>Opunc03g01390.1 0.115 0.372 0.257 0.685 0.907<br>Opunc07g18240.1 0.393 1 0.607 0.319 1	1<br>Obrac03g26550.1 0.115 0.337 0.222 0.068 0.886<br>Obrac07g12120.1 0.393 1 0.607 0.216 1	1<br>Lperr07g01300.1 0.133 0.35 0.217 0.488 0.527<br>Lperr07g16720.1 0.393 1 0.607 0.129 1	0<br>AT3G63190.1 0.378 1 0.622 0.269 1
Osjap07g25530.1	1<br>Osjap07g25530.1 0.002 1 0.998 0.002 1<br>Osjap07g25530.2 0.002 1 0.998 0.005 1	0	0	0	0	0	0	0	0	0
	0<br>Osjap03g05920.1 0.368 0.998 0.63 0.012 0.988	1<br>Osind07g22250.1 0.364 1 0.636 0.003 1<br>Osind07g22260.1 0.002 0.337 0.335 0.002 0.92	1<br>Orufi07g22950.1 0.364 1 0.636 0.003 1<br>Orufi07g22960.1 0.002 0.337 0.335 0.002 0.92	1<br>Oglab07g18760.1 0.364 1 0.636 0.003 1<br>Oglab07g18770.1 0.002 0.337 0.335 0.002 0.929	1<br>Obart03g05770.1 0.368 0.998 0.63 0.014 0.986<br>Obart07g21920.1 0.002 0.337 0.335 0.002 0.929	1<br>Olong_006816-R1 0.061 0.337 0.276 0.002 0.918<br>Olong_016841-R1 0.378 0.984 0.606 0.035 0.958	0<br>Opunc03g05560.1 0.297 0.996 0.699 0.032 0.99	0<br>Obrac07g14080.1 0.364 1 0.636 0.005 1	0<br>Lperr03g05070.1 0.352 0.996 0.644 0.084 0.997	0<br>AT5G21950.1 0.37 0.988 0.618 0.019 0.984
Osjap07g25960.2	1<br>Osjap07g25960.2 0.002 1 0.998 0.002 1<br>Osjap07g25960.3 0.002 1 0.998 0.002 1	0	0	0	0	0	0	0	0	0
	0<br>Osjap07g25960.1 0.002 0.51 0.508 0.004 0.904	1<br>Osind07g22730.1 0.002 0.51 0.508 0.179 0.928<br>Osind07g22740.1 0.512 0.935 0.423 0.004 0.917	1<br>Orufi07g23420.1 0.002 0.51 0.508 0.004 0.904<br>Orufi07g23430.2 0.512 1 0.488 0.004 1	1<br>Oglab03g20050.1 0.56 0.927 0.367 0.023 0.85<br>Oglab07g19210.1 0.002 0.51 0.508 0.166 0.927	1<br>Obart07g22390.1 0.016 0.51 0.494 0.131 0.926<br>Obart07g22400.3 0.512 1 0.488 0.004 1	1<br>Olong_014424-R1 0.006 0.51 0.504 0.003 0.912<br>Olong_014425-R1 0.512 0.921 0.409 0.005 0.99	1<br>Opunc03g19170.1 0.56 0.895 0.335 0.022 0.786<br>Opunc07g21000.2 0.002 0.51 0.508 0.191 0.926	0<br>Obrac07g14400.1 0.508 0.946 0.438 0.286 0.914	0<br>Lperr03g17430.1 0.56 0.891 0.331 0.023 0.751<br>Lperr07g19400.1 0.097 0.508 0.411 0.699 0.992	0<br>AT2G26210.1 0.232 0.51 0.278 0.163 0.889
Osjap07g25960.3	1<br>Osjap07g25960.2 0.002 1 0.998 0.002 1<br>Osjap07g25960.3 0.002 1 0.998 0.002 1	0	0	0	0	0	0	0	0	0
	0<br>Osjap07g25960.1 0.002 0.521 0.519 0.004 0.904	1<br>Osind07g22730.1 0.002 0.521 0.519 0.179 0.928<br>Osind07g22740.1 0.523 0.922 0.399 0.004 0.848	1<br>Orufi07g23420.1 0.002 0.521 0.519 0.004 0.904<br>Orufi07g23430.2 0.523 1 0.477 0.004 1	1<br>Oglab03g20050.1 0.572 0.914 0.342 0.023 0.775<br>Oglab07g19210.1 0.002 0.521 0.519 0.166 0.927	1<br>Obart07g22390.1 0.016 0.521 0.505 0.131 0.926<br>Obart07g22400.3 0.523 1 0.477 0.004 1	1<br>Olong_014424-R1 0.006 0.521 0.515 0.003 0.912<br>Olong_014425-R1 0.523 0.922 0.399 0.005 0.944	1<br>Opunc03g19170.1 0.572 0.914 0.342 0.022 0.786<br>Opunc07g21000.2 0.002 0.521 0.519 0.191 0.926	0<br>Obrac07g14400.1 0.519 0.944 0.425 0.286 0.914	0<br>Lperr03g17430.1 0.572 0.909 0.337 0.023 0.751<br>Lperr07g19400.1 0.099 0.519 0.42 0.699 0.992	0<br>AT2G26210.1 0.237 0.521 0.284 0.163 0.889
Osjap07g28390.1	1<br>Osjap07g28390.1 0.006 1 0.994 0.006 1	0	0	0	0	0	0	0	0	0

	0<br>Osjap04g19780.1 0.269 1 0.731 0.91 1	1<br>Osind04g18570.1 0.269 1 0.731 0.905 1<br>Osind10g04680.1 0.006 0.263 0.257 0.091 0.484	1<br>Orufi04g17880.1 0.269 1 0.731 0.914 1<br>Orufi07g25690.5 0.006 0.263 0.257 0.258 0.318	1<br>Oglab04g14790.1 0.269 1 0.731 0.9 1<br>Oglab07g21200.1 0.006 0.263 0.257 0.267 0.31	1<br>Obart04g16460.1 0.269 1 0.731 0.91 1<br>Obart07g24510.2 0.012 0.263 0.251 0.539 0.629	1<br>Olong_023809-R1 0.269 1 0.731 0.907 1<br>Olong_025722-R1 0.006 0.263 0.257 0.294 0.959	1<br>Opunc04g14330.1 0.269 1 0.731 0.91 1<br>Opunc07g23200.1 0.006 0.263 0.257 0.374 0.41	0<br>Obrac04g10180.1 0.269 1 0.731 0.908 1	0<br>Lperr04g13170.1 0.257 0.994 0.737 0.889 1	0
Osjap07g29350.1	1<br>Osjap07g29350.1 0.002 1 0.998 0.002 1	1<br>Osind07g26050.1 0.013 1 0.987 0.157 1	1<br>Orufi07g26420.1 0.106 1 0.894 0.002 1	0	0	1<br>Olong_024157-R2 0.011 0.989 0.978 0.044 0.998	0	0	0	0
	1<br>Osjap03g18810.1 0.719 1 0.281 0.007 1<br>Osjap07g29380.2 0.013 0.629 0.616 0.158 0.994	1<br>Osind01g39050.1 0.004 0.637 0.633 0.07 0.971<br>Osind08g27680.1 0.723 0.996 0.273 0.289 0.986	1<br>Orufi03g18050.1 0.717 1 0.283 0.437 1<br>Orufi07g26440.5 0.013 0.629 0.616 0.164 0.994	1<br>Oglab06g19010.1 0.119 0.605 0.486 0.058 0.967<br>Oglab07g22020.1 0.635 1 0.365 0.021 1	1<br>Obart06g20980.1 0.028 0.605 0.577 0.043 0.968<br>Obart07g25530.1 0.635 1 0.365 0.021 1	1<br>Olong_015865-R1 0.717 1 0.283 0.364 1<br>Olong_024157-R3 0.011 0.715 0.704 0.056 0.975	1<br>Opunc07g23940.1 0.719 1 0.281 0.007 1<br>Opunc07g23960.1 0.011 0.626 0.615 0.053 0.995	1<br>Obrac06g14370.1 0.026 0.635 0.609 0.05 0.99<br>Obrac07g16760.2 0.708 1 0.292 0.101 1	0<br>Lperr07g22320.1 0.719 1 0.281 0.007 1<br>Lperr11g01590.1 0.149 0.57 0.421 0.083 0.878	0<br>AT5G18100.1 0.711 0.998 0.287 0.018 0.957
Osjap08g04940.1	1<br>Osjap08g04840.3 0.006 0.999 0.993 0.079 0.997<br>Osjap08g04840.4 0.006 0.999 0.993 0.08 0.997<br>Osjap08g04940.1 0.001 1 0.999 0.001 1<br>Osjap08g04940.2 0.001 1 0.999 0.001 1<br>Osjap08g04940.3 0.001 1 0.999 0.001 1<br>Osjap08g04940.4 0.001 1 0.999 0.001 1<br>Osjap08g04940.5 0.143 1 0.857 0.089 1<br>Osjap08g04940.6 0.001 0.844 0.843 0.001 0.965<br>Osjap08g04940.7 0.147 1 0.853 0.08 1<br>Osjap08g04940.8 0.131 0.939 0.808 0.005 0.998	1<br>Osind08g04780.1 0.001 1 0.999 0.087 0.993	1<br>Orufi08g05010.2 0.001 0.986 0.985 0.001 0.995<br>Orufi08g05010.3 0.001 0.986 0.985 0.001 0.995<br>Orufi08g05110.1 0.005 1 0.995 0.064 0.978<br>Orufi08g05110.2 0.001 1 0.999 0.001 0.977<br>Orufi08g05110.3 0.001 1 0.999 0.001 0.977<br>Orufi08g05110.4 0.001 1 0.999 0.001 0.976<br>Orufi08g05110.5 0.001 1 0.999 0.001 0.977<br>Orufi08g05110.6 0.131 0.986 0.855 0.004 0.981	0	0	0	1<br>Opunc08g04470.1 0.001 0.977 0.976 0.001 1	0	0	0
	0	1<br>Osind04g16520.1 0.542 1 0.458 0.295 1<br>Osind08g04870.1 0.001 0.533 0.532 0.001 0.93	0<br>Orufi08g05010.1 0.131 0.849 0.718 0.006 0.995	1<br>Oglab08g04140.1 0.542 1 0.458 0.287 1<br>Oglab08g04220.1 0.001 0.533 0.532 0.002 0.939	1<br>Obart04g14760.1 0.542 1 0.458 0.295 1<br>Obart08g04630.1 0.001 0.533 0.532 0.001 0.924	1<br>Olong_024915-R1 0.001 0.533 0.532 0.001 0.997<br>Olong_026200-R1 0.542 1 0.458 0.258 1	1<br>Opunc08g04400.1 0.542 1 0.458 0.255 1<br>Opunc08g04480.1 0.001 0.533 0.532 0.001 0.981	1<br>Obrac04g08790.1 0.542 1 0.458 0.257 1<br>Obrac11g12090.1 0.001 0.528 0.527 0.003 0.974	1<br>Lperr08g04410.1 0.542 1 0.458 0.258 1<br>Lperr08g04480.1 0.001 0.529 0.528 0.001 0.947	0<br>AT1G34210.1 0.538 1 0.462 0.42 1
Osjap08g04940.2	1<br>Osjap08g04840.3 0.006 0.999 0.993 0.079 0.997<br>Osjap08g04840.4 0.006 0.999 0.993 0.08 0.997<br>Osjap08g04940.1 0.001 1 0.999 0.001 1<br>Osjap08g04940.2 0.001 1 0.999 0.001 1<br>Osjap08g04940.3 0.001 1 0.999 0.001 1<br>Osjap08g04940.4 0.001 1 0.999 0.001 1<br>Osjap08g04940.5 0.11 1 0.89 0.059 1<br>Osjap08g04940.6 0.001 0.841 0.84 0.001 0.965<br>Osjap08g04940.7 0.138 1 0.862 0.08 1<br>Osjap08g04940.8 0.122 0.937 0.815 0.005 0.998	1<br>Osind08g04780.1 0.001 1 0.999 0.087 0.993	1<br>Orufi08g05010.2 0.001 0.985 0.984 0.001 0.995<br>Orufi08g05010.3 0.001 0.985 0.984 0.001 0.995<br>Orufi08g05110.1 0.005 1 0.995 0.064 0.978<br>Orufi08g05110.2 0.001 1 0.999 0.001 0.977<br>Orufi08g05110.3 0.001 1 0.999 0.001 0.977<br>Orufi08g05110.4 0.001 1 0.999 0.001 0.976<br>Orufi08g05110.5 0.001 1 0.999 0.001 0.977<br>Orufi08g05110.6 0.122 0.985 0.863 0.004 0.981	0	0	0	1<br>Opunc08g04470.1 0.001 0.977 0.976 0.001 1	0	0	0
	0	1<br>Osind04g16520.1 0.542 1 0.458 0.295 1<br>Osind08g04870.1 0.001 0.533 0.532 0.001 0.93	0<br>Orufi08g05010.1 0.122 0.846 0.724 0.006 0.995	1<br>Oglab08g04140.1 0.542 1 0.458 0.287 1<br>Oglab08g04220.1 0.001 0.533 0.532 0.002 0.939	1<br>Obart04g14760.1 0.542 1 0.458 0.295 1<br>Obart08g04630.1 0.001 0.533 0.532 0.001 0.924	1<br>Olong_015528-R1 0.001 0.533 0.532 0.002 0.955<br>Olong_026200-R1 0.542 1 0.458 0.258 1	1<br>Opunc08g04400.1 0.542 1 0.458 0.255 1<br>Opunc08g04480.1 0.001 0.533 0.532 0.001 0.981	1<br>Obrac04g08790.1 0.542 1 0.458 0.257 1<br>Obrac11g12090.1 0.001 0.528 0.527 0.003 0.974	1<br>Lperr08g04410.1 0.542 1 0.458 0.258 1<br>Lperr08g04480.1 0.001 0.529 0.528 0.001 0.947	0<br>AT1G34210.1 0.537 1 0.463 0.42 1
Osjap08g04940.3	1<br>Osjap08g04840.3 0.006 0.999 0.993 0.079 0.997	1<br>Osind08g04780.1 0.001 1 0.999 0.087 0.993	1<br>Orufi08g05010.2 0.001 0.985 0.984 0.001 0.995	0	0	0	1<br>Opunc08g04470.1 0.001 0.977 0.976 0.001 1	0	0	0

	Osjap08g04840.4 0.006 0.999 0.993 0.08 0.997<br>Osjap08g04940.1 0.001 1 0.999 0.001 1<br>Osjap08g04940.2 0.001 1 0.999 0.001 1<br>Osjap08g04940.3 0.001 1 0.999 0.001 1<br>Osjap08g04940.4 0.001 1 0.999 0.001 1<br>Osjap08g04940.5 0.112 1 0.888 0.059 1<br>Osjap08g04940.6 0.001 0.838 0.837 0.001 0.965<br>Osjap08g04940.7 0.14 1 0.86 0.08 1<br>Osjap08g04940.8 0.123 0.936 0.813 0.005 0.998		Orufi08g05010.3 0.001 0.985 0.984 0.001 0.995<br>Orufi08g05110.1 0.006 1 0.994 0.064 0.978<br>Orufi08g05110.2 0.001 1 0.999 0.001 0.977<br>Orufi08g05110.3 0.001 1 0.999 0.001 0.977<br>Orufi08g05110.4 0.001 1 0.999 0.001 0.976<br>Orufi08g05110.5 0.001 1 0.999 0.001 0.977<br>Orufi08g05110.6 0.123 0.985 0.862 0.004 0.981							
	0	1<br>Osind04g16520.1 0.549 1 0.451 0.295 1<br>Osind08g04870.1 0.001 0.54 0.539 0.001 0.93	0<br>Orufi08g05010.1 0.123 0.844 0.721 0.006 0.995	1<br>Oglab08g04140.1 0.549 1 0.451 0.287 1<br>Oglab08g04220.1 0.001 0.54 0.539 0.002 0.939	1<br>Obart04g14760.1 0.549 1 0.451 0.295 1<br>Obart08g04630.1 0.001 0.54 0.539 0.001 0.924	1<br>Olong_015528-R1 0.001 0.54 0.539 0.002 0.955<br>Olong_026200-R1 0.549 1 0.451 0.258 1	1<br>Opunc08g04400.1 0.549 1 0.451 0.255 1<br>Opunc08g04480.1 0.001 0.54 0.539 0.001 0.981	1<br>Obrac04g08790.1 0.549 1 0.451 0.257 1<br>Obrac11g12090.1 0.001 0.535 0.534 0.003 0.974	1<br>Lperr08g04410.1 0.549 1 0.451 0.258 1<br>Lperr08g04480.1 0.001 0.536 0.535 0.001 0.947	0<br>AT1G34210.1 0.545 1 0.455 0.42 1
Osjap08g04940.4	1<br>Osjap08g04840.3 0.006 0.999 0.993 0.079 0.997<br>Osjap08g04840.4 0.006 0.999 0.993 0.08 0.997<br>Osjap08g04940.1 0.001 1 0.999 0.001 1<br>Osjap08g04940.2 0.001 1 0.999 0.001 1<br>Osjap08g04940.3 0.001 1 0.999 0.001 1<br>Osjap08g04940.4 0.001 1 0.999 0.001 1<br>Osjap08g04940.5 0.112 1 0.888 0.059 1<br>Osjap08g04940.6 0.001 0.858 0.857 0.001 0.965<br>Osjap08g04940.7 0.14 1 0.86 0.08 1<br>Osjap08g04940.8 0.124 0.936 0.812 0.005 0.998	1<br>Osind08g04780.1 0.001 1 0.999 0.087 0.993	1<br>Orufi08g05010.2 0.001 0.985 0.984 0.001 0.995<br>Orufi08g05010.3 0.001 0.985 0.984 0.001 0.995<br>Orufi08g05110.1 0.006 1 0.994 0.064 0.978<br>Orufi08g05110.2 0.001 1 0.999 0.001 0.977<br>Orufi08g05110.3 0.001 1 0.999 0.001 0.977<br>Orufi08g05110.4 0.001 1 0.999 0.001 0.976<br>Orufi08g05110.5 0.001 1 0.999 0.001 0.977<br>Orufi08g05110.6 0.124 0.985 0.861 0.004 0.981	0	0	0	1<br>Opunc08g04470.1 0.001 0.976 0.975 0.001 1	0	0	0
	0	1<br>Osind04g16520.1 0.553 1 0.447 0.295 1<br>Osind08g04870.1 0.001 0.544 0.543 0.001 0.93	0<br>Orufi08g05010.1 0.124 0.864 0.74 0.006 0.995	1<br>Oglab08g04140.1 0.553 1 0.447 0.287 1<br>Oglab08g04220.1 0.001 0.544 0.543 0.002 0.939	1<br>Obart04g14760.1 0.553 1 0.447 0.295 1<br>Obart08g04630.1 0.001 0.544 0.543 0.001 0.924	1<br>Olong_015528-R1 0.001 0.544 0.543 0.002 0.955<br>Olong_026200-R1 0.553 1 0.447 0.258 1	1<br>Opunc08g04400.1 0.553 1 0.447 0.255 1<br>Opunc08g04480.1 0.001 0.544 0.543 0.001 0.981	1<br>Obrac04g08790.1 0.553 1 0.447 0.257 1<br>Obrac11g12090.1 0.001 0.539 0.538 0.003 0.974	1<br>Lperr08g04410.1 0.553 1 0.447 0.258 1<br>Lperr08g04480.1 0.001 0.54 0.539 0.001 0.947	0<br>AT1G34210.1 0.549 1 0.451 0.42 1
Osjap08g04940.5	1<br>Osjap08g04840.3 0.089 0.999 0.91 0.079 0.997<br>Osjap08g04840.8 0.089 1 0.911 0.098 0.996<br>Osjap08g04940.1 0.089 1 0.911 0.143 1<br>Osjap08g04940.2 0.059 1 0.941 0.11 1<br>Osjap08g04940.3 0.059 1 0.941 0.112 1<br>Osjap08g04940.4 0.059 1 0.941 0.112 1<br>Osjap08g04940.5 0.001 1 0.999 0.001 1<br>Osjap08g04940.7 0.001 1 0.999 0.001 1<br>Osjap08g04940.8 0.089 0.935 0.846 0.027 0.998	1<br>Osind08g04780.1 0.068 1 0.932 0.087 0.993	1<br>Orufi08g05010.2 0.068 0.985 0.917 0.159 0.995<br>Orufi08g05010.3 0.068 0.985 0.917 0.163 0.995<br>Orufi08g05110.1 0.001 1 0.999 0.001 0.978<br>Orufi08g05110.2 0.089 1 0.911 0.142 0.977<br>Orufi08g05110.3 0.059 1 0.941 0.108 0.977<br>Orufi08g05110.4 0.059 1 0.941 0.109 0.976<br>Orufi08g05110.5 0.059 1 0.941 0.107 0.977<br>Orufi08g05110.6 0.089 0.985 0.896 0.02 0.981	0	0	0	1<br>Opunc08g04470.1 0.089 0.976 0.887 0.25 1	0	0	0
	0<br>Osjap08g04940.6 0.059 0.834 0.775 0.179 0.965	1<br>Osind04g16520.1 0.513 1 0.487 0.295 1<br>Osind08g04870.1 0.089 0.503 0.414 0.129 0.93	0<br>Orufi08g05010.1 0.089 0.84 0.751 0.033 0.995	1<br>Oglab08g04140.1 0.509 1 0.491 0.287 1<br>Oglab08g04220.1 0.089 0.503 0.414 0.13 0.939	1<br>Obart08g04530.1 0.509 1 0.491 0.258 1<br>Obart08g04630.1 0.089 0.503 0.414 0.122 0.924	1<br>Olong_015528-R1 0.089 0.503 0.414 0.134 0.955<br>Olong_026200-R1 0.509 1 0.491 0.258 1	1<br>Opunc08g04400.1 0.509 1 0.491 0.255 1<br>Opunc08g04480.1 0.092 0.503 0.411 0.255 0.981	0<br>Obrac04g08790.1 0.513 1 0.487 0.257 1	1<br>Lperr08g04410.1 0.509 1 0.491 0.258 1<br>Lperr08g04480.1 0.089 0.5 0.411 0.265 0.947	0<br>AT1G34210.1 0.509 1 0.491 0.42 1
Osjap08g04940.7	1	1	1	0	0	0	1	0	0	0

	Osjap08g04840.3 0.08 0.999 0.919 0.127 0.996<br>Osjap08g04840.8 0.08 1 0.92 0.154 0.995<br>Osjap08g04940.1 0.08 1 0.92 0.147 1<br>Osjap08g04940.2 0.08 1 0.92 0.138 1<br>Osjap08g04940.3 0.08 1 0.92 0.14 1<br>Osjap08g04940.4 0.08 1 0.92 0.14 1<br>Osjap08g04940.5 0.001 1 0.999 0.001 1<br>Osjap08g04940.7 0.001 1 0.999 0.001 1<br>Osjap08g04940.8 0.08 0.932 0.852 0.033 0.998	Osind08g04780.1 0.08 1 0.92 0.14 0.992	Orufi08g05010.2 0.08 0.984 0.904 0.188 0.993<br>Orufi08g05010.3 0.08 0.984 0.904 0.193 0.993<br>Orufi08g05110.1 0.001 1 0.999 0.001 0.978<br>Orufi08g05110.2 0.08 1 0.92 0.145 0.977<br>Orufi08g05110.3 0.08 1 0.92 0.134 0.977<br>Orufi08g05110.4 0.08 1 0.92 0.136 0.976<br>Orufi08g05110.5 0.08 1 0.92 0.133 0.977<br>Orufi08g05110.6 0.08 0.984 0.904 0.025 0.981				Opunc08g04470.1 0.08 0.975 0.895 0.255 1			
	0<br>Osjap08g04940.6 0.08 0.827 0.747 0.224 0.965	1<br>Osind04g16520.1 0.518 1 0.482 0.444 1<br>Osind08g04870.1 0.08 0.508 0.428 0.132 0.93	0<br>Orufi08g05010.1 0.08 0.833 0.753 0.039 0.995	1<br>Oglab08g04140.1 0.518 1 0.482 0.439 1<br>Oglab08g04220.1 0.08 0.508 0.428 0.13 0.939	1<br>Obart04g14760.1 0.518 1 0.482 0.444 1<br>Obart08g04630.1 0.08 0.508 0.428 0.122 0.924	1<br>Olong_015528-R1 0.08 0.508 0.428 0.136 0.955<br>Olong_026200-R1 0.518 1 0.482 0.417 1	1<br>Opunc08g04400.1 0.514 1 0.486 0.402 1<br>Opunc08g04480.1 0.08 0.508 0.428 0.255 0.981	0<br>Obrac04g08790.1 0.518 1 0.482 0.414 1	1<br>Lperr08g04410.1 0.518 1 0.482 0.417 1<br>Lperr08g04480.1 0.08 0.504 0.424 0.27 0.947	0<br>AT1G34210.1 0.514 1 0.486 0.432 1
Osjap08g06650.1	1<br>Osjap08g06650.1 0.002 1 0.998 0.002 1	1<br>Osind08g06790.1 0.002 1 0.998 0.002 1	1<br>Orufi08g06840.1 0.002 1 0.998 0.002 1	1<br>Oglab08g05640.1 0.002 1 0.998 0.002 1	1<br>Obart08g06070.1 0.002 1 0.998 0.002 1	0	1<br>Opunc08g05850.1 0.047 1 0.953 0.068 1	0	0	0
	1<br>Osjap04g21220.1 0.047 0.275 0.228 0.21 0.936<br>Osjap09g20870.1 0.359 0.984 0.625 0.208 0.966	1<br>Osind04g20250.1 0.047 0.275 0.228 0.198 0.94<br>Osind09g20960.1 0.359 0.984 0.625 0.208 0.966	1<br>Orufi04g19250.2 0.047 0.275 0.228 0.21 0.936<br>Orufi09g21960.1 0.359 0.984 0.625 0.2 0.965	1<br>Oglab02g26360.1 0.348 0.996 0.648 0.01 0.994<br>Oglab04g16180.1 0.047 0.275 0.228 0.198 0.94	1<br>Obart02g28440.1 0.348 0.996 0.648 0.093 0.994<br>Obart04g17770.1 0.047 0.275 0.228 0.198 0.94	1<br>Olong_008030-R1 0.047 0.275 0.228 0.232 0.937<br>Olong_008031-R2 0.277 1 0.723 0.106 1	1<br>Opunc04g15550.1 0.047 0.275 0.228 0.206 0.938<br>Opunc04g18740.1 0.295 0.984 0.689 0.088 0.994	1<br>Obrac08g04780.1 0.277 1 0.723 0.098 1<br>Obrac08g04800.1 0.002 0.275 0.273 0.006 0.943	1<br>Lperr08g05690.1 0.277 1 0.723 0.1 1<br>Lperr08g05700.1 0.002 0.275 0.273 0.006 0.943	1<br>AT1G05310.1 0.33 0.982 0.652 0.183 0.99<br>AT4G30800.1 0.047 0.275 0.228 0.208 0.943
Osjap08g10540.1	1<br>Osjap08g10540.1 0.001 1 0.999 0.001 1	1<br>Osind08g11830.1 0.001 1 0.999 0.006 1	1<br>Orufi08g11060.1 0.001 1 0.999 0.001 1	0	0	0	0	0	0	0
	1<br>Osjap08g10560.1 0.001 0.248 0.247 0.02 0.683<br>Osjap10g16570.1 0.315 0.993 0.678 0.229 0.992	1<br>Osind05g10830.1 0.309 0.995 0.686 0.057 0.966<br>Osind07g12020.1 0.001 0.247 0.246 0.003 0.758	1<br>Orufi05g09510.1 0.309 0.995 0.686 0.066 0.964<br>Orufi08g11080.2 0.001 0.248 0.247 0.021 0.71	1<br>Oglab02g32050.1 0.248 0.967 0.719 0.001 0.99<br>Oglab08g09010.1 0.001 0.248 0.247 0.028 0.635	1<br>Obart02g04890.1 0.266 0.986 0.72 0.148 0.991<br>Obart07g11970.1 0.001 0.248 0.247 0.003 0.764	1<br>Olong_015028-R1 0.001 0.248 0.247 0.032 0.684<br>Olong_022719-R1 0.315 0.993 0.678 0.238 0.966	1<br>Opunc02g16130.1 0.315 0.995 0.68 0.224 1<br>Opunc08g08990.2 0.001 0.248 0.247 0.018 0.873	0<br>Obrac04g05110.2 0.003 0.248 0.245 0.025 0.656	0<br>Lperr07g03020.1 0.688 0.978 0.29 0.003 0.899<br>Lperr10g01740.2 0.01 0.273 0.263 0.042 0.596	0<br>AT4G15880.1 0.734 0.969 0.235 0.495 0.99
Osjap08g13760.3	1<br>Osjap08g13760.3 0.003 1 0.997 0.003 1	0	1<br>Orufi08g14470.3 0.023 1 0.977 0.081 1	0	0	0	0	0	0	0
	0<br>Osjap03g41020.1 0.431 1 0.569 0.179 1	1<br>Osind08g15490.1 0.003 0.247 0.244 0.002 0.172<br>Osind08g15500.1 0.314 1 0.686 0.005 1	1<br>Orufi03g39540.2 0.431 1 0.569 0.179 1<br>Orufi08g14470.1 0.003 0.247 0.244 0.031 0.195	1<br>Oglab08g11850.1 0.003 0.247 0.244 0.002 0.172<br>Oglab08g11860.1 0.314 1 0.686 0.005 1	1<br>Obart08g12780.1 0.003 0.247 0.244 0.002 0.172<br>Obart08g12790.1 0.314 1 0.686 0.005 1	0	1<br>Opunc03g34960.1 0.418 1 0.582 0.171 1<br>Opunc05g25720.1 0.037 0.311 0.274 0.016 0.217	1<br>Obrac03g28290.1 0.418 1 0.582 0.15 1<br>Obrac09g02930.1 0.023 0.247 0.224 0.011 0.186	1<br>Lperr03g32080.1 0.431 1 0.569 0.205 1<br>Lperr08g10650.1 0.003 0.247 0.244 0.002 0.18	1<br>AT1G20890.1 0.361 0.95 0.589 0.036 0.97<br>AT1G64440.1 0.037 0.271 0.234 0.006 0.195
Osjap08g19480.1	1<br>Osjap08g19480.1 0.001 1 0.999 0.001 1	0	0	0	0	0	0	0	0	0
	0<br>Osjap05g21810.1 0.044 0.36 0.316 0.142 0.996	1<br>Osind08g21600.1 0.001 0.346 0.345 0.002 0.98<br>Osind08g21610.1 0.364 0.959 0.595 0.02 0.976	1<br>Orufi08g20540.1 0.037 0.364 0.327 0.137 1<br>Orufi08g20550.1 0.364 0.959 0.595 0.021 0.975	1<br>Oglab08g16980.1 0.037 0.364 0.327 0.137 1<br>Oglab08g17000.1 0.364 0.959 0.595 0.022 0.974	1<br>Obart08g18360.1 0.037 0.364 0.327 0.137 1<br>Obart08g18370.1 0.364 0.959 0.595 0.02 0.975	1<br>Olong_010651-R1 0.364 0.959 0.595 0.01 0.973<br>Olong_010652-R1 0.037 0.364 0.327 0.137 1	1<br>Opunc08g16280.1 0.037 0.364 0.327 0.137 1<br>Opunc08g16290.1 0.364 0.959 0.595 0.019 0.973	1<br>Obrac08g12010.1 0.037 0.364 0.327 0.139 1<br>Obrac08g12020.1 0.364 0.984 0.62 0.013 0.973	1<br>Lperr08g15260.1 0.037 0.357 0.32 0.14 1<br>Lperr08g15270.1 0.364 0.959 0.595 0.02 0.98	1<br>AT5G06220.2 0.411 0.96 0.549 0.078 0.988<br>AT5G64300.1 0.042 0.348 0.306 0.166 0.98
Osjap08g22730.1	1<br>Osjap08g22730.1 0.001 1 0.999 0.001 1	1<br>Osind08g24940.1 0.13 0.987 0.857 0.09 0.999	1<br>Orufi08g23770.1 0.02 1 0.98 0.019 1<br>Orufi08g23770.3 0.002 1 0.998 0.018 1	0	0	0	0	0	0	0

	1<br>Osjap04g34840.1 0.02 0.374 0.354 0.031 0.992<br>Osjap08g22710.1 0.43 1 0.57 0.026 1	1<br>Osind04g34100.1 0.001 0.374 0.373 0.002 0.992<br>Osind08g24940.2 0.437 1 0.563 0.006 1	1<br>Orufi04g32420.1 0.02 0.374 0.354 0.031 0.992<br>Orufi08g23750.1 0.414 1 0.586 0.002 1	0<br>Oglab04g28790.1 0.001 0.374 0.373 0.002 0.992	1<br>Obart04g30710.1 0.001 0.374 0.373 0.002 0.992<br>Obart08g21280.1 0.411 1 0.589 0.002 1	1<br>Olong_012750-R1 0.002 0.367 0.365 0.085 0.881<br>Olong_024729-R1 0.465 0.999 0.534 0.017 1	1<br>Opunc04g28230.1 0.004 0.369 0.365 0.008 0.979<br>Opunc08g19370.1 0.39 0.989 0.599 0.003 0.997	1<br>Obrac04g21850.1 0.001 0.364 0.363 0.008 0.992<br>Obrac08g14190.1 0.405 1 0.595 0.002 1	1<br>Lperr04g26140.1 0.015 0.365 0.35 0.032 0.96<br>Lperr08g18050.1 0.41 1 0.59 0.003 1	0<br>AT2G02020.1 0.001 0.36 0.359 0.007 0.994
Osjap09g03990.5	1<br>Osjap09g03990.1 0.004 1 0.996 0.543 1<br>Osjap09g03990.2 0.004 1 0.996 0.508 1<br>Osjap09g03990.5 0.004 1 0.996 0.004 1	0	1<br>Orufi09g04390.1 0.004 1 0.996 0.6 1<br>Orufi09g04390.2 0.004 1 0.996 0.559 1	0	0	0	0	0	0	0
	1<br>Osjap05g14220.1 0.507 1 0.493 0.468 0.818<br>Osjap09g14680.2 0.004 0.414 0.41 0.77 0.967	1<br>Osind09g04090.1 0.674 1 0.326 0.03 0.582<br>Osind09g04110.1 0.004 0.581 0.577 0.704 1	1<br>Orufi09g08210.2 0.507 1 0.493 0.388 0.791<br>Orufi09g15320.1 0.004 0.414 0.41 0.75 0.965	1<br>Oglab08g18120.2 0.731 1 0.269 0.391 0.77<br>Oglab09g03780.1 0.004 0.581 0.577 0.714 1	1<br>Obart07g20140.1 0.709 1 0.291 0.385 0.717<br>Obart09g03930.1 0.004 0.581 0.577 0.714 1	1<br>Olong_001796-R2 0.004 0.626 0.622 0.703 0.995<br>Olong_005713-R1 0.705 1 0.295 0.522 0.912	1<br>Opunc07g19040.1 0.709 1 0.291 0.384 0.717<br>Opunc09g03510.2 0.004 0.581 0.577 0.685 1	1<br>Obrac07g12720.1 0.709 1 0.291 0.276 0.667<br>Obrac09g02030.2 0.004 0.581 0.577 0.676 1	1<br>Lperr07g17470.1 0.709 1 0.291 0.382 0.716<br>Lperr09g02810.2 0.004 0.581 0.577 0.737 1	1<br>AT1G79610.1 0.004 0.626 0.622 0.735 0.974<br>AT5G07100.1 0.639 1 0.361 0.285 0.864
Osjap09g06840.5	1<br>Osjap09g06840.1 0.002 1 0.998 0.001 1<br>Osjap09g06840.2 0.002 1 0.998 0.001 1<br>Osjap09g06840.3 0.002 1 0.998 0.001 1<br>Osjap09g06840.4 0.002 1 0.998 0.001 1<br>Osjap09g06840.5 0.002 1 0.998 0.002 1<br>Osjap09g06840.6 0.002 1 0.998 0.001 1<br>Osjap09g06840.7 0.002 1 0.998 0.001 1<br>Osjap09g06840.8 0.002 1 0.998 0.001 1	0	0	1<br>Oglab09g06250.1 0.002 1 0.998 0.004 1<br>Oglab09g06250.2 0.002 1 0.998 0.004 1	1<br>Obart09g06850.1 0.002 1 0.998 0.004 1<br>Obart09g06850.2 0.002 1 0.998 0.005 1	1<br>Olong_018324-R1 0.084 0.995 0.911 0.322 0.91	1<br>Opunc09g05880.2 0.005 1 0.995 0.007 1	0	0	0
	0<br>Osjap03g08670.1 0.399 1 0.601 0.284 1	1<br>Osind09g06740.1 0.347 1 0.653 0.067 1<br>Osind09g06760.1 0.002 0.31 0.308 0.005 0.985	1<br>Orufi09g07360.3 0.343 1 0.657 0.2 1<br>Orufi09g07380.1 0.002 0.31 0.308 0.006 0.983	1<br>Oglab09g06170.1 0.002 0.304 0.302 0.005 0.721<br>Oglab09g14590.1 0.378 1 0.622 0.068 1	1<br>Obart02g37100.1 0.005 0.29 0.285 0.268 0.773<br>Obart09g06770.4 0.296 1 0.704 0.115 1	0<br>Olong_000874-R3 0.38 1 0.62 0.113 1	1<br>Opunc02g34270.1 0.005 0.29 0.285 0.009 0.83<br>Opunc09g14540.1 0.394 1 0.606 0.091 1	0<br>Obrac09g03560.2 0.399 1 0.601 0.261 1	1<br>Lperr02g30550.1 0.006 0.264 0.258 0.013 0.711<br>Lperr03g07450.1 0.399 1 0.601 0.103 1	1<br>AT1G29310.1 0.399 1 0.601 0.101 1<br>AT5G55810.2 0.008 0.29 0.282 0.004 0.765
Osjap09g06840.6	1<br>Osjap09g06840.1 0.001 1 0.999 0.001 1<br>Osjap09g06840.2 0.001 1 0.999 0.001 1<br>Osjap09g06840.3 0.001 1 0.999 0.001 1<br>Osjap09g06840.4 0.001 1 0.999 0.001 1<br>Osjap09g06840.5 0.001 1 0.999 0.002 1<br>Osjap09g06840.6 0.001 1 0.999 0.001 1<br>Osjap09g06840.7 0.001 1 0.999 0.001 1<br>Osjap09g06840.8 0.001 1 0.999 0.001 1	0	0	1<br>Oglab09g06250.1 0.001 1 0.999 0.004 1	1<br>Obart09g06850.1 0.001 1 0.999 0.004 1<br>Obart09g06850.2 0.001 1 0.999 0.005 1	1<br>Olong_018324-R1 0.079 0.996 0.917 0.322 0.91	1<br>Opunc09g05880.2 0.004 1 0.996 0.007 1	0	0	0
	0<br>Osjap03g08670.1 0.375 1 0.625 0.284 1	1<br>Osind09g06740.1 0.326 1 0.674 0.067 1<br>Osind09g06760.1 0.001 0.292 0.291 0.005 0.985	1<br>Orufi09g07360.1 0.327 1 0.673 0.107 1<br>Orufi09g07380.1 0.001 0.292 0.291 0.006 0.983	1<br>Oglab09g06170.1 0.001 0.286 0.285 0.005 0.721<br>Oglab09g14590.1 0.356 1 0.644 0.068 1	1<br>Obart02g37100.1 0.004 0.272 0.268 0.268 0.773<br>Obart09g06770.4 0.278 1 0.722 0.115 1	0<br>Olong_000874-R3 0.357 1 0.643 0.113 1	1<br>Opunc02g34270.1 0.004 0.272 0.268 0.009 0.83<br>Opunc09g14540.1 0.371 1 0.629 0.091 1	0<br>Obrac09g03560.2 0.375 1 0.625 0.261 1	1<br>Lperr02g30550.1 0.006 0.249 0.243 0.013 0.711<br>Lperr03g07450.1 0.375 1 0.625 0.103 1	1<br>AT1G29310.1 0.375 1 0.625 0.101 1<br>AT5G55810.2 0.007 0.272 0.265 0.004 0.765
Osjap09g06840.7	1<br>Osjap09g06840.1 0.001 1 0.999 0.001 1<br>Osjap09g06840.2 0.001 1 0.999 0.001 1<br>Osjap09g06840.3 0.001 1 0.999 0.001 1	0	0	1<br>Oglab09g06250.1 0.001 1 0.999 0.004 1	1<br>Obart09g06850.1 0.001 1 0.999 0.004 1<br>Obart09g06850.2 0.001 1 0.999 0.005 1	1<br>Olong_018324-R1 0.079 0.996 0.917 0.322 0.91	0	0	0	0

	Osjap09g06840.4 0.001 1 0.999 0.001 1<br>Osjap09g06840.5 0.001 1 0.999 0.002 1<br>Osjap09g06840.6 0.001 1 0.999 0.001 1<br>Osjap09g06840.7 0.001 1 0.999 0.001 1<br>Osjap09g06840.8 0.001 1 0.999 0.001 1									
	0<br>Osjap03g08670.1 0.375 1 0.625 0.284 1	1<br>Osind09g06740.1 0.326 1 0.674 0.067 1<br>Osind09g06760.1 0.001 0.292 0.291 0.005 0.985	1<br>Orufi09g07360.1 0.327 1 0.673 0.107 1<br>Orufi09g07380.1 0.001 0.292 0.291 0.006 0.983	1<br>Oglab09g06170.1 0.001 0.286 0.285 0.005 0.721<br>Oglab09g14590.1 0.356 1 0.644 0.068 1	1<br>Obart02g37100.1 0.004 0.272 0.268 0.268 0.773<br>Obart09g06770.4 0.278 1 0.722 0.115 1	0<br>Olong_000874-R3 0.357 1 0.643 0.113 1	1<br>Opunc02g34270.1 0.004 0.272 0.268 0.009 0.83<br>Opunc09g14540.1 0.371 1 0.629 0.091 1	0<br>Obrac09g03560.2 0.375 1 0.625 0.261 1	1<br>Lperr02g30550.1 0.006 0.249 0.243 0.013 0.711<br>Lperr03g07450.1 0.375 1 0.625 0.103 1	1<br>AT1G29310.1 0.375 1 0.625 0.101 1<br>AT5G55810.2 0.007 0.272 0.265 0.004 0.765
Osjap09g06840.8	1<br>Osjap09g06840.1 0.001 1 0.999 0.001 1<br>Osjap09g06840.2 0.001 1 0.999 0.001 1<br>Osjap09g06840.3 0.001 1 0.999 0.001 1<br>Osjap09g06840.4 0.001 1 0.999 0.001 1<br>Osjap09g06840.5 0.001 1 0.999 0.002 1<br>Osjap09g06840.6 0.001 1 0.999 0.001 1<br>Osjap09g06840.7 0.001 1 0.999 0.001 1<br>Osjap09g06840.8 0.001 1 0.999 0.001 1	0	0	1<br>Oglab09g06250.1 0.001 1 0.999 0.004 1	1<br>Obart09g06850.1 0.001 1 0.999 0.004 1<br>Obart09g06850.2 0.001 1 0.999 0.005 1	1<br>Olong_018324-R1 0.079 0.996 0.917 0.322 0.91	0	0	0	0
	0<br>Osjap03g08670.1 0.324 1 0.676 0.223 1	1<br>Osind03g08730.1 0.324 1 0.676 0.025 1<br>Osind09g06760.1 0.001 0.29 0.289 0.005 0.985	1<br>Orufi03g08240.1 0.324 1 0.676 0.025 1<br>Orufi09g07380.1 0.001 0.29 0.289 0.006 0.983	1<br>Oglab09g06150.1 0.29 1 0.71 0.213 1<br>Oglab09g06170.1 0.001 0.284 0.283 0.005 0.721	1<br>Obart02g37100.1 0.004 0.271 0.267 0.268 0.773<br>Obart09g06770.4 0.277 1 0.723 0.078 1	0<br>Olong_005924-R1 0.324 1 0.676 0.132 1	1<br>Opunc02g34270.1 0.004 0.271 0.267 0.009 0.83<br>Opunc03g07900.2 0.324 1 0.676 0.025 1	0<br>Obrac09g03560.2 0.289 1 0.711 0.126 1	1<br>Lperr02g30550.1 0.006 0.247 0.241 0.013 0.711<br>Lperr03g07450.1 0.324 1 0.676 0.026 1	1<br>AT1G29310.1 0.324 1 0.676 0.025 1<br>AT5G55810.2 0.007 0.271 0.264 0.004 0.765
Osjap09g15430.2	1<br>Osjap09g15430.2 0.002 1 0.998 0.002 1<br>Osjap09g15430.3 0.002 1 0.998 0.002 1	1<br>Osind09g15570.1 0.002 1 0.998 0.002 1<br>Osind09g15570.2 0.002 1 0.998 0.002 1	1<br>Orufi09g16160.1 0.002 1 0.998 0.002 1<br>Orufi09g16160.2 0.002 1 0.998 0.002 1	1<br>Oglab09g13510.1 0.002 1 0.998 0.002 1<br>Oglab09g13510.2 0.002 1 0.998 0.002 1<br>Oglab09g13510.3 0.002 1 0.998 0.002 1<br>Oglab09g13510.4 0.002 1 0.998 0.002 1	1<br>Obart09g14980.1 0.002 1 0.998 0.002 1<br>Obart09g14980.2 0.002 1 0.998 0.002 1	0	1<br>Opunc09g13420.1 0.002 1 0.998 0.002 1<br>Opunc09g13420.2 0.002 1 0.998 0.002 1	1<br>Obrac09g09090.1 0.002 1 0.998 0.002 1	0	0
	0<br>Osjap02g06650.1 0.01 0.565 0.555 0.01 0.997	1<br>Osind02g06940.1 0.01 0.565 0.555 0.01 0.997<br>Osind08g23920.1 0.569 0.993 0.424 0.131 0.988	1<br>Orufi04g24220.1 0.014 0.567 0.553 0.165 1<br>Orufi08g22880.1 0.569 0.993 0.424 0.131 0.988	1<br>Oglab08g19240.1 0.569 0.993 0.424 0.143 0.986<br>Oglab12g18680.1 0.002 0.567 0.565 0.003 1	1<br>Obart02g06180.1 0.01 0.565 0.555 0.01 0.997<br>Obart08g20390.1 0.569 0.993 0.424 0.631 0.988	1<br>Olong_008093-R2 0.596 0.991 0.395 0.25 0.996<br>Olong_024186-R1 0.002 0.567 0.565 0.003 1	1<br>Opunc04g20190.1 0.007 0.567 0.56 0.006 1<br>Opunc08g18560.1 0.569 0.993 0.424 0.128 0.969	1<br>Obrac01g09090.1 0.605 0.998 0.393 0.299 0.982<br>Obrac02g04550.3 0.009 0.565 0.556 0.214 0.997	1<br>Lperr04g18350.1 0.002 0.567 0.565 0.021 1<br>Lperr09g11120.1 0.569 1 0.431 0.156 1	1<br>AT1G68540.1 0.567 0.991 0.424 0.112 1<br>AT2G21270.3 0.003 0.565 0.562 0.059 0.997
Osjap09g15430.3	1<br>Osjap09g15430.2 0.002 1 0.998 0.002 1<br>Osjap09g15430.3 0.002 1 0.998 0.002 1	1<br>Osind09g15570.1 0.002 1 0.998 0.002 1<br>Osind09g15570.2 0.002 1 0.998 0.002 1	1<br>Orufi09g16160.1 0.002 1 0.998 0.002 1<br>Orufi09g16160.2 0.002 1 0.998 0.002 1	1<br>Oglab09g13510.1 0.002 1 0.998 0.002 1<br>Oglab09g13510.2 0.002 1 0.998 0.002 1<br>Oglab09g13510.3 0.002 1 0.998 0.002 1<br>Oglab09g13510.4 0.002 1 0.998 0.002 1	1<br>Obart09g14980.1 0.002 1 0.998 0.002 1<br>Obart09g14980.2 0.002 1 0.998 0.002 1	0	1<br>Opunc09g13420.1 0.002 1 0.998 0.002 1<br>Opunc09g13420.2 0.002 1 0.998 0.002 1	1<br>Obrac09g09090.1 0.002 1 0.998 0.002 1	0	0
	0<br>Osjap02g06650.1 0.01 0.54 0.53 0.01 0.997	1<br>Osind02g06940.1 0.01 0.54 0.53 0.01 0.997<br>Osind08g23920.1 0.544 0.993 0.449 0.131 0.988	1<br>Orufi04g24220.1 0.013 0.542 0.529 0.165 1<br>Orufi08g22880.1 0.544 0.993 0.449 0.131 0.988	1<br>Oglab08g19240.1 0.544 0.993 0.449 0.143 0.986<br>Oglab12g18680.1 0.002 0.542 0.54 0.003 1	1<br>Obart02g06180.1 0.01 0.54 0.53 0.01 0.997<br>Obart08g20390.1 0.544 0.993 0.449 0.631 0.988	1<br>Olong_001228-R1 0.548 0.992 0.444 0.03 0.947<br>Olong_024186-R1 0.002 0.542 0.54 0.003 1	1<br>Opunc04g20190.1 0.007 0.542 0.535 0.006 1<br>Opunc08g18560.1 0.544 0.993 0.449 0.128 0.969	1<br>Obrac02g04550.3 0.008 0.54 0.532 0.214 0.997<br>Obrac09g00560.1 0.548 0.998 0.45 0.164 0.966	1<br>Lperr04g18350.1 0.002 0.542 0.54 0.021 1<br>Lperr09g11120.1 0.544 1 0.456 0.156 1	1<br>AT1G68540.1 0.542 0.992 0.45 0.112 1<br>AT2G21270.3 0.003 0.54 0.537 0.059 0.997
Osjap09g17720.1	1<br>Osjap09g17720.1 0.001 1 0.999 0.001 1	1<br>Osind09g17930.1 0.001 1 0.999 0.001 0.995	0	0	0	0	0	0	0	0

	0<br>Osjap10g13350.2 0.212 0.99 0.778 0.015 0.984	1<br>Osind06g11050.1 0.002 0.207 0.205 0.268 0.883<br>Osind06g25720.1 0.208 0.992 0.784 0.029 0.989	1<br>Orufi09g18680.1 0.004 0.209 0.205 0.291 0.895<br>Orufi10g11850.1 0.212 0.99 0.778 0.016 0.984	1<br>Oglab06g09730.1 0.002 0.207 0.205 0.268 0.883<br>Oglab06g20260.1 0.207 0.992 0.785 0.014 0.981	1<br>Obart02g06450.1 0.218 0.992 0.774 0.019 0.994<br>Obart09g17370.1 0.001 0.209 0.208 0.003 0.852	1<br>Olong_005351-R1 0.001 0.209 0.208 0.003 0.853<br>Olong_007875-R1 0.218 0.99 0.772 0.014 0.967	1<br>Opunc08g00450.2 0.216 0.99 0.774 0.048 0.856<br>Opunc09g15680.1 0.004 0.209 0.205 0.301 0.895	0<br>Obrac01g21100.1 0.194 0.984 0.79 0.011 0.984	1<br>Lperr02g25740.1 0.002 0.207 0.205 0.277 0.888<br>Lperr06g18550.1 0.213 0.99 0.777 0.025 0.975	1<br>AT3G48280.1 0.218 0.99 0.772 0.002 0.998<br>AT4G39010.1 0.002 0.207 0.205 0.282 0.899
Osjap09g20460.1	1<br>Osjap09g20460.1 0.001 1 0.999 0.001 1	1<br>Osind09g20560.1 0.001 0.986 0.985 0.001 0.998	1<br>Orufi09g21510.1 0.001 0.967 0.966 0.001 0.999	1<br>Oglab09g18200.1 0.001 0.999 0.998 0.001 0.999	1<br>Obart09g19980.1 0.062 0.999 0.937 0.165 0.999<br>Obart09g19980.2 0.001 0.999 0.998 0.001 0.999	1<br>Olong_014589-R1 0.108 0.974 0.866 0.018 0.991<br>Olong_014589-R2 0.108 0.974 0.866 0.018 0.991	0	0	0	0
	1<br>Osjap02g21530.2 0.022 0.584 0.562 0.096 0.943<br>Osjap09g20430.1 0.624 1 0.376 0.085 1	1<br>Osind02g21430.2 0.022 0.584 0.562 0.096 0.943<br>Osind09g20550.1 0.624 1 0.376 0.032 1	1<br>Orufi02g19810.2 0.022 0.584 0.562 0.096 0.943<br>Orufi09g21500.1 0.624 1 0.376 0.085 1	1<br>Oglab02g17850.1 0.022 0.584 0.562 0.095 0.914<br>Oglab09g18170.1 0.624 1 0.376 0.079 1	1<br>Obart04g25370.1 0.044 0.592 0.548 0.176 0.967<br>Obart09g19950.1 0.624 1 0.376 0.079 1	1<br>Olong_014116-R1 0.059 0.592 0.533 0.069 0.957<br>Olong_027417-R1 0.626 0.991 0.365 0.135 0.745	1<br>Opunc09g18280.1 0.619 0.998 0.379 0.058 0.951<br>Opunc09g18290.2 0.001 0.592 0.591 0.001 0.97	1<br>Obrac09g13140.3 0.624 0.986 0.362 0.049 0.806<br>Obrac09g13160.3 0.001 0.593 0.592 0.001 0.974	1<br>Lperr09g15890.1 0.623 0.996 0.373 0.048 0.957<br>Lperr09g15920.1 0.001 0.592 0.591 0.001 0.957	0<br>AT1G18160.1 0.02 0.592 0.572 0.064 0.958
Osjap10g04950.1	1<br>Osjap10g04950.1 0.01 1 0.99 0.01 1<br>Osjap10g21210.1 0.01 1 0.99 0.477 1	1<br>Osind10g03420.1 0.01 1 0.99 0.558 1<br>Osind10g17700.1 0.01 1 0.99 0.477 1	1<br>Orufi10g04520.1 0.01 1 0.99 0.01 1<br>Orufi10g19420.1 0.01 1 0.99 0.477 1	1<br>Oglab10g03910.1 0.01 1 0.99 0.01 1	1<br>Obart10g18220.1 0.01 1 0.99 0.392 0.823	1<br>Olong_009744-R1 0.01 1 0.99 0.651 1<br>Olong_009744-R2 0.01 1 0.99 0.641 1	0	0	0	0
	0<br>Osjap07g13660.9 0.361 1 0.639 0.733 0.947	1<br>Osind02g38620.1 0.361 1 0.639 0.835 0.923<br>Osind06g00010.1 0.01 0.361 0.351 0.609 0.978	1<br>Orufi02g36440.1 0.361 1 0.639 0.835 0.923<br>Orufi08g26670.5 0.01 0.351 0.341 0.583 0.749	1<br>Oglab02g32260.1 0.361 1 0.639 0.838 0.924<br>Oglab04g27900.1 0.01 0.33 0.32 0.14 0.208	1<br>Obart02g34980.1 0.361 1 0.639 0.838 0.924<br>Obart04g29850.1 0.01 0.33 0.32 0.131 0.196	1<br>Olong_008835-R2 0.361 1 0.639 0.809 0.91<br>Olong_022843-R2 0.01 0.33 0.32 0.14 0.208	1<br>Opunc02g32260.1 0.361 1 0.639 0.84 0.925<br>Opunc04g27380.2 0.01 0.33 0.32 0.14 0.208	1<br>Obrac02g25650.1 0.381 1 0.619 0.839 0.923<br>Obrac04g21100.1 0.01 0.33 0.32 0.145 0.217	1<br>Lperr02g28770.1 0.381 1 0.619 0.834 0.92<br>Lperr04g25370.2 0.01 0.33 0.32 0.151 0.225	0
Osjap10g12540.3	1<br>Osjap08g08740.1 0.004 0.96 0.956 0.002 0.513<br>Osjap10g12540.1 0.004 1 0.996 0.003 1<br>Osjap10g12540.2 0.004 1 0.996 0.004 1<br>Osjap10g12540.3 0.004 1 0.996 0.004 1<br>Osjap10g12540.4 0.004 1 0.996 0.004 1	0	0	0	1<br>Obart04g26170.1 0.115 0.96 0.845 0.066 0.174<br>Obart04g26170.2 0.115 0.96 0.845 0.067 0.165	0	0	0	0	0
	0<br>Osjap02g39060.3 0.411 0.988 0.577 0.756 0.958	1<br>Osind02g38620.1 0.435 0.988 0.553 0.782 0.961<br>Osind03g12490.1 0.004 0.308 0.304 0.002 0.137	1<br>Orufi02g36440.1 0.435 0.988 0.553 0.782 0.961<br>Orufi03g12130.3 0.004 0.308 0.304 0.002 0.137	1<br>Oglab02g32260.1 0.435 0.988 0.553 0.786 0.961<br>Oglab03g11800.1 0.004 0.308 0.304 0.002 0.137	1<br>Obart02g34980.1 0.435 0.988 0.553 0.786 0.961<br>Obart03g11790.2 0.004 0.308 0.304 0.002 0.137	0<br>Olong_008835-R2 0.435 0.988 0.553 0.747 0.954	1<br>Opunc02g32260.1 0.431 0.988 0.557 0.787 0.962<br>Opunc03g11340.1 0.004 0.308 0.304 0.002 0.131	1<br>Obrac02g25650.1 0.431 0.988 0.557 0.783 0.961<br>Obrac03g09470.2 0.004 0.3 0.296 0.002 0.142	1<br>Lperr02g28770.1 0.431 0.988 0.557 0.775 0.959<br>Lperr03g10490.2 0.004 0.423 0.419 0.002 0.209	0<br>AT2G35620.1 0.016 0.304 0.288 0.008 0.132
Osjap10g12540.4	1<br>Osjap08g08740.1 0.004 0.957 0.953 0.002 0.513<br>Osjap10g12540.1 0.004 1 0.996 0.003 1<br>Osjap10g12540.2 0.004 1 0.996 0.004 1<br>Osjap10g12540.3 0.004 1 0.996 0.004 1<br>Osjap10g12540.4 0.004 1 0.996 0.004 1	0	0	0	1<br>Obart04g26170.1 0.126 0.957 0.831 0.066 0.174<br>Obart04g26170.2 0.126 0.957 0.831 0.067 0.165	0	0	0	0	0
	0<br>Osjap02g39060.3 0.355 0.987 0.632 0.756 0.958	1<br>Osind02g38620.1 0.381 0.987 0.606 0.782 0.961<br>Osind03g12490.1 0.004 0.359 0.355 0.002 0.146	1<br>Orufi02g36440.1 0.381 0.987 0.606 0.782 0.961<br>Orufi03g12130.3 0.004 0.359 0.355 0.002 0.146	1<br>Oglab02g32260.1 0.381 0.987 0.606 0.786 0.961<br>Oglab03g11800.1 0.004 0.359 0.355 0.002 0.146	1<br>Obart02g34980.1 0.381 0.987 0.606 0.786 0.961<br>Obart03g11790.2 0.004 0.359 0.355 0.002 0.146	0<br>Olong_008835-R2 0.381 0.987 0.606 0.747 0.954	1<br>Opunc02g32260.1 0.377 0.987 0.61 0.787 0.962<br>Opunc03g11340.1 0.004 0.359 0.355 0.002 0.14	1<br>Obrac02g25650.1 0.377 0.987 0.61 0.783 0.961<br>Obrac03g09470.1 0.004 0.359 0.355 0.002 0.14	1<br>Lperr02g28770.1 0.377 0.987 0.61 0.775 0.959<br>Lperr03g10490.1 0.004 0.359 0.355 0.002 0.14	0<br>AT2G35620.1 0.017 0.359 0.342 0.008 0.143
Osjap10g14600.1	1<br>Osjap10g14600.1 0.002 1 0.998 0.002 1	1<br>Osind10g11660.1 0.002 0.995 0.993 0.003 0.958	1<br>Orufi08g13110.1 0.002 0.987 0.985 0.003 0.99	1<br>Oglab10g11970.1 0.002 0.982 0.98 0.007 1	1<br>Obart10g12400.1 0.002 0.98 0.978 0.004 0.996	0	1<br>Opunc10g10650.1 0.002 0.995 0.993 0.003 0.974	0	0	0

	Osjap10g14600.2 0.002 1 0.998 0.002 1<br>Osjap10g14600.3 0.002 1 0.998 0.002 1<br>Osjap10g14600.4 0.002 1 0.998 0.002 1<br>Osjap10g14600.5 0.002 1 0.998 0.002 1<br>Osjap10g14600.6 0.002 1 0.998 0.002 1						Opunc10g10650.2 0.002 0.995 0.993 0.003 0.974			
	0<br>Osjap08g12520.1 0.277 0.98 0.703 0.226 0.86	1<br>Osind08g14090.1 0.664 0.987 0.323 0.346 0.989<br>Osind10g11740.1 0.16 0.638 0.478 0.002 0.08	1<br>Orufi08g13110.2 0.664 0.987 0.323 0.346 0.989<br>Orufi10g13050.2 0.002 0.658 0.656 0.003 0.627	1<br>Oglab03g12700.1 0.701 0.975 0.274 0.29 0.977<br>Oglab10g11980.2 0.04 0.649 0.609 0.016 0.42	1<br>Obart03g12670.1 0.701 0.975 0.274 0.29 0.977<br>Obart10g12410.2 0.04 0.653 0.613 0.046 0.639	1<br>Olong_005116-R1 0.658 0.975 0.317 0.171 0.982<br>Olong_028277-R1 0.002 0.651 0.649 0.006 0.975	1<br>Opunc10g10640.1 0.04 0.638 0.598 0.049 0.61<br>Opunc12g14410.1 0.7 0.975 0.275 0.253 0.984	1<br>Obrac10g07370.1 0.117 0.638 0.521 0.003 0.101<br>Obrac12g10200.1 0.66 0.975 0.315 0.237 0.985	1<br>Lperr03g19720.1 0.653 0.982 0.329 0.285 1<br>Lperr10g09020.1 0.117 0.638 0.521 0.001 0.059	1<br>AT3G04260.1 0.117 0.638 0.521 0.075 0.193<br>AT3G55330.1 0.653 0.975 0.322 0.2 0.991
Osjap10g15240.1	1<br>Osjap10g15240.1 0.001 1 0.999 0.001 1	0	0	0	0	0	0	0	0	0
	1<br>Osjap03g03010.1 0.01 0.376 0.366 0.001 0.999<br>Osjap03g36880.2 0.49 0.996 0.506 0.068 0.878	1<br>Osind10g12350.1 0.415 1 0.585 0.001 1<br>Osind10g12360.1 0.001 0.376 0.375 0.001 0.999	1<br>Orufi10g13620.2 0.415 1 0.585 0.001 1<br>Orufi10g13630.1 0.001 0.376 0.375 0.001 0.999	1<br>Oglab10g12600.4 0.415 1 0.585 0.001 1<br>Oglab10g12610.1 0.001 0.376 0.375 0.001 0.999	1<br>Obart10g12900.2 0.415 1 0.585 0.036 1<br>Obart10g12910.1 0.001 0.376 0.375 0.001 0.982	1<br>Olong_005999-R1 0.004 0.376 0.372 0.119 0.999<br>Olong_018868-R1 0.469 0.994 0.525 0.001 1	1<br>Opunc10g11270.1 0.415 1 0.585 0.001 1<br>Opunc10g11280.1 0.001 0.376 0.375 0.094 0.976	1<br>Obrac10g07770.1 0.489 1 0.511 0.004 1<br>Obrac10g07780.1 0.009 0.377 0.368 0.023 0.971	1<br>Lperr10g09460.1 0.415 1 0.585 0.001 1<br>Lperr10g09470.1 0.001 0.376 0.375 0.001 0.999	1<br>AT2G35920.1 0.461 0.998 0.537 0.039 0.998<br>AT4G28600.1 0.003 0.376 0.373 0.014 0.999
Osjap11g00600.1	1<br>Osjap11g00600.1 0.002 1 0.998 0.002 1<br>Osjap12g00550.1 0.002 0.998 0.996 0.002 1	1<br>Osind12g00790.1 0.002 0.998 0.996 0.002 1	1<br>Orufi11g00440.1 0.002 1 0.998 0.002 1	1<br>Oglab11g00570.1 0.106 1 0.894 0.051 1<br>Oglab12g00270.1 0.002 0.801 0.799 0.003 0.995	1<br>Obart11g00510.1 0.106 1 0.894 0.045 1<br>Obart12g00320.1 0.002 0.998 0.996 0.003 1	0	0	0	0	0
	1<br>Osjap08g03600.1 0.748 0.984 0.236 0.201 0.274<br>Osjap10g06830.2 0.087 0.719 0.632 0.265 0.998	1<br>Osind05g03780.1 0.748 0.984 0.236 0.16 0.63<br>Osind10g04960.1 0.063 0.719 0.656 0.31 0.998	1<br>Orufi08g03710.3 0.748 0.984 0.236 0.289 0.394<br>Orufi12g00580.1 0.002 0.703 0.701 0.002 0.881	0<br>Oglab10g05540.1 0.087 0.719 0.632 0.32 0.998	1<br>Obart08g03340.1 0.748 0.984 0.236 0.191 0.268<br>Obart12g18380.1 0.069 0.717 0.648 0.137 0.977	1<br>Olong_026176-R1 0.083 0.717 0.634 0.114 0.997<br>Olong_027703-R1 0.748 0.984 0.236 0.192 0.274	1<br>Opunc08g03130.1 0.748 0.984 0.236 0.206 0.282<br>Opunc12g00560.1 0.002 0.724 0.722 0.003 0.995	1<br>Obrac08g02840.1 0.748 0.984 0.236 0.201 0.274<br>Obrac12g00030.1 0.004 0.624 0.62 0.412 0.991	1<br>Lperr12g00550.1 0.748 0.986 0.238 0.194 0.271<br>Lperr12g00630.2 0.033 0.719 0.686 0.031 0.983	0<br>AT2G40030.1 0.738 0.982 0.244 0.168 0.234
Osjap11g01220.1	1<br>Osjap11g01220.1 0.001 1 0.999 0.001 1<br>Osjap12g01140.1 0.001 1 0.999 0.001 1	1<br>Osind11g00690.1 0.001 0.955 0.954 0.001 0.999<br>Osind12g01290.1 0.001 1 0.999 0.001 1	1<br>Orufi11g01000.1 0.001 1 0.999 0.001 1<br>Orufi12g01130.1 0.001 1 0.999 0.001 1	1<br>Oglab12g00800.1 0.001 1 0.999 0.001 1	1<br>Obart11g01060.1 0.001 1 0.999 0.001 1	0	1<br>Opunc12g00940.1 0.001 1 0.999 0.001 1	0	0	0
	1<br>Osjap03g13100.1 0.489 0.982 0.493 0.097 0.622<br>Osjap07g30390.1 0.009 0.433 0.424 0.013 0.944	1<br>Osind03g12930.1 0.489 0.982 0.493 0.121 0.633<br>Osind07g27070.1 0.007 0.433 0.426 0.006 0.946	1<br>Orufi03g17220.1 0.496 1 0.504 0.027 1<br>Orufi07g27420.1 0.009 0.433 0.424 0.013 0.946	1<br>Oglab03g12120.1 0.489 0.982 0.493 0.121 0.633<br>Oglab07g22980.1 0.007 0.433 0.426 0.007 0.94	1<br>Obart03g12120.1 0.489 0.982 0.493 0.121 0.633<br>Obart12g00790.1 0.001 0.433 0.432 0.002 0.949	1<br>Olong_007715-R1 0.492 1 0.508 0.007 1<br>Olong_008211-R1 0.007 0.433 0.426 0.006 0.946	1<br>Opunc03g11680.1 0.489 1 0.511 0.148 1<br>Opunc11g00920.1 0.001 0.433 0.432 0.142 0.951	1<br>Obrac03g09720.2 0.489 1 0.511 0.142 1<br>Obrac12g00780.1 0.001 0.433 0.432 0.11 0.953	1<br>Lperr03g10720.1 0.489 0.993 0.504 0.148 0.952<br>Lperr11g00920.1 0.001 0.433 0.432 0.002 0.949	1<br>AT2G34940.1 0.001 0.433 0.432 0.002 0.955<br>AT3G46740.1 0.492 1 0.508 0.161 1
Osjap11g01730.2	1<br>Osjap11g01730.2 0.004 1 0.996 0.004 1	0	1<br>Orufi11g01440.2 0.004 1 0.996 0.004 1	1<br>Oglab11g01340.2 0.004 1 0.996 0.004 1	0	0	1<br>Opunc11g01300.1 0.004 0.989 0.985 0.309 1	0	0	0
	0<br>Osjap11g01730.3 0.48 1 0.52 0.004 1	1<br>Osind07g17820.1 0.004 0.401 0.397 0.486 0.909<br>Osind11g01160.1 0.468 1 0.532 0.004 1	0<br>Orufi11g01440.3 0.48 1 0.52 0.004 1	1<br>Oglab11g01330.1 0.524 1 0.476 0.213 1<br>Oglab12g01230.1 0.004 0.476 0.472 0.573 0.975	1<br>Obart12g01240.1 0.498 0.993 0.495 0.002 0.566<br>Obart12g01250.1 0.004 0.476 0.472 0.57 0.975	1<br>Olong_020639-R1 0.476 1 0.524 0.036 1<br>Olong_025830-R1 0.004 0.476 0.472 0.502 0.97	1<br>Opunc11g01280.1 0.517 1 0.483 0.084 0.91<br>Opunc11g01300.2 0.004 0.476 0.472 0.538 0.973	0<br>Obrac11g03920.2 0.004 0.465 0.461 0.631 0.986	0<br>Lperr11g01230.1 0.651 0.859 0.208 0.245 0.765<br>Lperr12g01420.1 0.004 0.476 0.472 0.547 0.973	0<br>AT1G04590.2 0.011 0.628 0.617 0.539 0.997
Osjap11g01770.1	1<br>Osjap11g01770.1 0.001 1 0.999 0.001 1<br>Osjap11g01770.2 0.001 1 0.999 0.039 1<br>Osjap11g01770.3 0.001 1 0.999 0.001 1<br>Osjap11g01770.4 0.001 1 0.999 0.001 1	0	0	0	0	0	0	0	0	0

	0<br>Osjap11g01890.3 0.001 0.353 0.352 0.001 0.67	1<br>Osind11g01200.1 0.348 0.994 0.646 0.02 1<br>Osind12g02160.1 0.001 0.348 0.347 0.002 0.949	1<br>Orufi11g01480.1 0.348 0.998 0.65 0.002 1<br>Orufi12g01730.1 0.001 0.348 0.347 0.002 0.949	1<br>Oglab11g01510.1 0.002 0.353 0.351 0.005 0.686<br>Oglab12g01260.1 0.357 1 0.643 0.012 1	1<br>Obart11g01710.2 0.001 0.353 0.352 0.001 0.685<br>Obart11g12030.1 0.355 0.998 0.643 0.141 0.996	1<br>Olong_024204-R6 0.001 0.348 0.347 0.002 0.947<br>Olong_026748-R2 0.363 1 0.637 0.001 1	1<br>Opunc12g01360.1 0.363 1 0.637 0.017 1<br>Opunc12g01530.2 0.001 0.363 0.362 0.002 0.959	1<br>Obrac12g01170.2 0.355 0.999 0.644 0.002 0.998<br>Obrac12g01310.1 0.001 0.348 0.347 0.002 0.948	1<br>Lperr12g01430.1 0.357 0.999 0.642 0.002 0.998<br>Lperr12g01570.1 0.001 0.348 0.347 0.002 0.948	0<br>AT5G44050.1 0.002 0.355 0.353 0.006 0.99
Osjap11g01770.2	1<br>Osjap11g01770.1 0.039 1 0.961 0.001 1<br>Osjap11g01770.2 0.001 1 0.999 0.001 1<br>Osjap11g01770.3 0.039 1 0.961 0.001 1<br>Osjap11g01770.4 0.039 1 0.961 0.001 1	0	0	0	0	0	0	0	0	0
	0<br>Osjap11g01890.3 0.039 0.378 0.339 0.001 0.67	1<br>Osind11g01200.1 0.373 0.994 0.621 0.02 1<br>Osind12g02160.1 0.039 0.373 0.334 0.002 0.949	1<br>Orufi11g01480.1 0.373 0.999 0.626 0.002 1<br>Orufi12g01730.1 0.039 0.373 0.334 0.002 0.949	1<br>Oglab11g01510.1 0.015 0.378 0.363 0.005 0.686<br>Oglab12g01260.1 0.381 1 0.619 0.012 1	1<br>Obart11g01710.2 0.015 0.378 0.363 0.001 0.685<br>Obart11g12030.1 0.38 0.999 0.619 0.141 0.996	1<br>Olong_024204-R6 0.039 0.373 0.334 0.002 0.947<br>Olong_026748-R2 0.387 1 0.613 0.001 1	1<br>Opunc12g01360.1 0.387 1 0.613 0.017 1<br>Opunc12g01520.8 0.014 0.375 0.361 0.001 0.972	1<br>Obrac12g01170.2 0.38 0.999 0.619 0.002 0.998<br>Obrac12g01310.1 0.039 0.373 0.334 0.002 0.948	1<br>Lperr11g01370.1 0.032 0.373 0.341 0.001 0.966<br>Lperr12g01430.1 0.381 0.999 0.618 0.002 0.998	0<br>AT5G44050.1 0.04 0.38 0.34 0.006 0.99
Osjap11g01770.3	1<br>Osjap11g01770.1 0.001 1 0.999 0.001 1<br>Osjap11g01770.2 0.001 1 0.999 0.039 1<br>Osjap11g01770.3 0.001 1 0.999 0.001 1<br>Osjap11g01770.4 0.001 1 0.999 0.001 1	0	0	0	0	0	0	0	0	0
	0<br>Osjap11g01890.3 0.001 0.339 0.338 0.001 0.67	1<br>Osind11g01200.1 0.334 0.994 0.66 0.02 1<br>Osind12g02160.1 0.001 0.334 0.333 0.002 0.949	1<br>Orufi11g01480.1 0.334 0.998 0.664 0.002 1<br>Orufi12g01730.1 0.001 0.334 0.333 0.002 0.949	1<br>Oglab11g01510.1 0.002 0.339 0.337 0.005 0.686<br>Oglab12g01260.1 0.343 1 0.657 0.012 1	1<br>Obart11g01710.2 0.001 0.339 0.338 0.001 0.685<br>Obart12g01280.1 0.343 1 0.657 0.013 1	1<br>Olong_024204-R2 0.001 0.334 0.333 0.002 0.945<br>Olong_026748-R2 0.349 1 0.651 0.001 1	1<br>Opunc12g01360.1 0.349 1 0.651 0.017 1<br>Opunc12g01520.8 0.001 0.336 0.335 0.001 0.972	1<br>Obrac12g01170.2 0.342 0.999 0.657 0.002 0.998<br>Obrac12g01310.1 0.001 0.334 0.333 0.002 0.948	1<br>Lperr12g01430.1 0.343 0.999 0.656 0.002 0.998<br>Lperr12g01570.1 0.001 0.334 0.333 0.002 0.948	1<br>AT3G04080.1 0.349 0.994 0.645 0.13 1<br>AT5G44050.1 0.002 0.342 0.34 0.006 0.99
Osjap11g01770.4	1<br>Osjap11g01770.1 0.001 1 0.999 0.001 1<br>Osjap11g01770.2 0.001 1 0.999 0.039 1<br>Osjap11g01770.3 0.001 1 0.999 0.001 1<br>Osjap11g01770.4 0.001 1 0.999 0.001 1	0	0	0	0	0	0	0	0	0
	0<br>Osjap11g01890.3 0.001 0.552 0.551 0.001 0.67	1<br>Osind02g25830.1 0.001 0.552 0.551 0.002 0.962<br>Osind05g02000.1 0.555 0.998 0.443 0.202 0.996	1<br>Orufi11g01590.1 0.001 0.552 0.551 0.002 0.962<br>Orufi12g01550.4 0.569 0.992 0.423 0.102 0.997	1<br>Oglab11g01510.1 0.002 0.552 0.55 0.005 0.686<br>Oglab12g01260.1 0.558 1 0.442 0.058 1	1<br>Obart11g01710.2 0.001 0.552 0.551 0.001 0.685<br>Obart12g01280.1 0.558 1 0.442 0.063 1	1<br>Olong_024204-R6 0.001 0.543 0.542 0.002 0.947<br>Olong_026748-R2 0.567 1 0.433 0.001 1	0<br>Opunc12g01530.2 0.001 0.568 0.567 0.002 0.959	1<br>Obrac12g01170.2 0.555 0.999 0.444 0.156 0.998<br>Obrac12g01310.1 0.001 0.543 0.542 0.002 0.948	1<br>Lperr11g01290.1 0.597 0.999 0.402 0.262 0.998<br>Lperr12g01560.1 0.001 0.596 0.595 0.001 0.709	0<br>AT5G44050.1 0.002 0.555 0.553 0.006 0.99
Osjap11g02640.1	1<br>Osjap11g02640.1 0.002 1 0.998 0.002 1<br>Osjap12g02610.1 0.002 1 0.998 0.002 1	1<br>Osind11g01990.1 0.002 1 0.998 0.002 1<br>Osind12g02780.1 0.002 1 0.998 0.002 1	1<br>Orufi11g02320.1 0.002 1 0.998 0.003 1<br>Orufi12g02480.1 0.002 1 0.998 0.002 1	1<br>Oglab11g02200.1 0.002 1 0.998 0.002 1<br>Oglab12g02130.1 0.002 1 0.998 0.002 1	1<br>Obart12g02080.1 0.024 1 0.976 0.002 1	0	0	0	0	0
	1<br>Osjap07g04360.1 0.034 0.683 0.649 0.018 0.926<br>Osjap12g12870.1 0.685 0.948 0.263 0.106 0.417	0<br>Osind07g03860.1 0.034 0.706 0.672 0.032 0.959	1<br>Orufi07g03650.1 0.034 0.683 0.649 0.019 0.978<br>Orufi12g10910.1 0.685 0.948 0.263 0.106 0.417	1<br>Oglab07g03810.1 0.034 0.683 0.649 0.019 0.988<br>Oglab12g09880.1 0.685 0.948 0.263 0.097 0.385	0<br>Obart11g02350.1 0.022 0.681 0.659 0.004 0.993	1<br>Olong_012584-R1 0.067 0.633 0.566 0.002 0.965<br>Olong_021330-R1 0.665 0.935 0.27 0.153 0.616	1<br>Opunc11g02190.1 0.002 0.681 0.679 0.003 0.989<br>Opunc12g08740.1 0.685 0.948 0.263 0.081 0.324	1<br>Obrac05g10860.1 0.052 0.681 0.629 0.097 0.987<br>Obrac12g07170.1 0.685 0.938 0.253 0.142 0.57	1<br>Lperr12g02190.1 0.002 0.681 0.679 0.003 0.989<br>Lperr12g08370.1 0.688 0.948 0.26 0.095 0.391	0<br>AT5G27320.1 0.014 0.655 0.641 0.003 0.98
Osjap11g03190.1	1<br>Osjap11g03190.1 0.002 1 0.998 0.002 1	0	0	0	0	0	0	0	0	0

	1<br>Osjap11g03620.1 0.003 0.23 0.227 0.198 0.811<br>Osjap12g03260.1 0.372 0.995 0.623 0.046 1	1<br>Osind11g02550.1 0.372 1 0.628 0.045 1<br>Osind11g03030.1 0.003 0.23 0.227 0.016 0.766	1<br>Orufi11g02770.1 0.372 1 0.628 0.196 1<br>Orufi11g03240.1 0.003 0.23 0.227 0.016 0.768	1<br>Oglab11g02800.1 0.002 0.313 0.311 0.005 0.959<br>Oglab11g02820.1 0.332 1 0.668 0.002 1	1<br>Obart11g02890.1 0.002 0.313 0.311 0.005 0.962<br>Obart11g02910.1 0.332 1 0.668 0.002 1	0<br>Olong_004426-R1 0.372 0.995 0.623 0.042 1	1<br>Opunc11g02560.1 0.002 0.372 0.37 0.002 0.552<br>Opunc12g02460.1 0.372 0.995 0.623 0.048 1	0<br>Obrac11g01720.1 0.576 0.995 0.419 0.196 1	0<br>Lperr11g02460.1 0.381 0.995 0.614 0.075 1	0<br>AT1G51450.1 0.512 0.968 0.456 0.444 0.972
Osjap11g03220.1	1<br>Osjap11g03220.1 0.001 1 0.999 0.001 1	0	0	0	0	0	0	0	0	0
	1<br>Osjap12g03280.1 0.432 1 0.568 0.047 1<br>Osjap12g03300.1 0.001 0.302 0.301 0.003 0.991	1<br>Osind11g02580.2 0.424 1 0.576 0.032 1<br>Osind12g03310.1 0.001 0.326 0.325 0.003 0.964	1<br>Orufi11g02800.2 0.424 1 0.576 0.032 1<br>Orufi11g02820.1 0.001 0.302 0.301 0.219 0.99	1<br>Oglab11g02850.3 0.424 1 0.576 0.032 1<br>Oglab11g02870.1 0.001 0.343 0.342 0.003 0.963	1<br>Obart11g02940.2 0.424 1 0.576 0.032 1<br>Obart11g02960.2 0.001 0.35 0.349 0.167 0.926	1<br>Olong_016306-R1 0.001 0.35 0.349 0.117 0.924<br>Olong_016308-R1 0.43 1 0.57 0.041 1	1<br>Opunc11g02640.1 0.001 0.343 0.342 0.003 1<br>Opunc12g02490.1 0.43 1 0.57 0.04 1	1<br>Obrac11g01750.1 0.001 0.34 0.339 0.003 0.994<br>Obrac12g02140.1 0.432 1 0.568 0.044 1	1<br>Lperr11g02500.4 0.001 0.322 0.321 0.003 0.938<br>Lperr12g02610.1 0.43 1 0.57 0.041 1	1<br>AT2G26460.1 0.424 0.98 0.556 0.029 0.969<br>AT3G22880.1 0.001 0.326 0.325 0.003 0.977
Osjap11g21440.1	1<br>Osjap11g21440.1 0.001 1 0.999 0.001 1	1<br>Osind04g04060.1 0.001 0.934 0.933 0.072 0.988	1<br>Orufi11g19860.1 0.003 1 0.997 0.052 1	1<br>Oglab11g16480.1 0.003 1 0.997 0.052 1	1<br>Obart11g18650.1 0.001 1 0.999 0.073 1	1<br>Olong_013913-R1 0.006 0.965 0.959 0.001 0.986	0	0	0	0
	1<br>Osjap03g43210.1 0.717 0.987 0.27 0.278 0.99<br>Osjap10g09150.2 0.008 0.716 0.708 0.047 0.738	1<br>Osind03g44240.1 0.717 0.987 0.27 0.017 0.988<br>Osind10g06650.1 0.008 0.716 0.708 0.008 0.713	1<br>Orufi03g41640.1 0.717 0.987 0.27 0.018 0.987<br>Orufi10g08130.1 0.008 0.716 0.708 0.047 0.737	1<br>Oglab03g37880.1 0.717 0.987 0.27 0.278 0.99<br>Oglab11g20100.1 0.02 0.711 0.691 0.029 0.947	1<br>Obart03g02940.1 0.739 0.986 0.247 0.118 0.932<br>Obart10g07740.1 0.008 0.716 0.708 0.047 0.737	1<br>Olong_001283-R1 0.002 0.674 0.672 0.014 0.844<br>Olong_011979-R1 0.739 0.987 0.248 0.108 0.954	1<br>Opunc03g37060.1 0.717 0.987 0.27 0.151 0.989<br>Opunc05g18050.1 0.047 0.702 0.655 0.06 0.817	1<br>Obrac03g30000.2 0.705 0.979 0.274 0.031 0.937<br>Obrac05g13790.3 0.029 0.702 0.673 0.127 0.872	1<br>Lperr03g33980.1 0.717 0.974 0.257 0.008 0.411<br>Lperr11g13500.1 0.043 0.677 0.634 0.047 0.638	0<br>AT3G12620.1 0.719 0.984 0.265 0.047 0.964
Osjap11g25180.1	1<br>Osjap11g25180.1 0.001 1 0.999 0.001 1	0	0	0	0	0	0	0	0	0
	1<br>Osjap03g12940.2 0.75 0.996 0.246 0.002 0.941<br>Osjap11g25230.1 0.001 0.715 0.714 0.001 0.936	1<br>Osind03g12720.1 0.75 0.999 0.249 0.032 0.989<br>Osind11g17650.1 0.001 0.75 0.749 0.001 0.989	1<br>Orufi03g12330.1 0.75 0.996 0.246 0.029 0.947<br>Orufi11g23320.1 0.001 0.75 0.749 0.001 0.989	1<br>Oglab03g12000.1 0.75 0.996 0.246 0.036 0.944<br>Oglab11g18660.1 0.001 0.75 0.749 0.001 0.989	1<br>Obart03g11990.1 0.75 0.999 0.249 0.032 0.989<br>Obart11g21600.1 0.005 0.725 0.72 0.026 0.955	1<br>Olong_007726-R1 0.763 0.999 0.236 0.153 0.981<br>Olong_026881-R1 0.001 0.73 0.729 0.002 0.98	1<br>Opunc11g17740.1 0.001 0.742 0.741 0.003 0.975<br>Opunc11g18020.1 0.765 1 0.235 0.004 1	1<br>Obrac11g12680.1 0.775 1 0.225 0.081 1<br>Obrac11g12750.1 0.006 0.767 0.761 0.004 0.999	1<br>Lperr03g10630.5 0.765 0.999 0.234 0.004 0.967<br>Lperr11g18000.1 0.001 0.749 0.748 0.001 1	0<br>AT2G29260.1 0.755 0.999 0.244 0.121 0.975
Osjap12g01140.1	1<br>Osjap11g01220.1 0.001 1 0.999 0.001 1<br>Osjap12g01140.1 0.001 1 0.999 0.001 1	1<br>Osind11g00690.1 0.001 0.953 0.952 0.001 0.999<br>Osind12g01290.1 0.001 1 0.999 0.001 1	1<br>Orufi11g01000.1 0.001 1 0.999 0.001 1<br>Orufi12g01130.1 0.001 1 0.999 0.001 1	1<br>Oglab12g00800.1 0.001 1 0.999 0.001 1	1<br>Obart11g01060.1 0.001 1 0.999 0.001 1	0	1<br>Opunc12g00940.1 0.001 1 0.999 0.001 1	0	0	0
	1<br>Osjap03g13100.1 0.511 0.981 0.47 0.097 0.622<br>Osjap07g30390.1 0.01 0.373 0.363 0.013 0.798	1<br>Osind03g12930.1 0.511 0.981 0.47 0.121 0.633<br>Osind07g27070.1 0.007 0.377 0.37 0.006 0.783	1<br>Orufi03g17220.1 0.517 1 0.483 0.027 1<br>Orufi07g27420.1 0.01 0.377 0.367 0.013 0.783	1<br>Oglab03g12120.1 0.511 0.981 0.47 0.121 0.633<br>Oglab07g22980.1 0.007 0.373 0.366 0.007 0.783	1<br>Obart03g12120.1 0.511 0.981 0.47 0.121 0.633<br>Obart10g06750.1 0.021 0.409 0.388 0.151 0.885	1<br>Olong_007715-R1 0.514 1 0.486 0.007 1<br>Olong_008211-R1 0.007 0.377 0.37 0.006 0.783	1<br>Opunc03g11680.1 0.511 1 0.489 0.148 1<br>Opunc11g00920.1 0.001 0.377 0.376 0.142 0.816	1<br>Obrac03g09720.2 0.511 1 0.489 0.142 1<br>Obrac10g04150.1 0.02 0.405 0.385 0.036 0.868	1<br>Lperr03g10720.1 0.511 0.993 0.482 0.148 0.952<br>Lperr11g00920.1 0.001 0.377 0.376 0.002 0.792	1<br>AT2G34940.1 0.001 0.377 0.376 0.002 0.793<br>AT3G46740.1 0.514 1 0.486 0.161 1
Osjap12g02200.1	1<br>Osjap11g02160.1 0.024 1 0.976 0.05 1<br>Osjap12g02200.1 0.001 1 0.999 0.001 1	0	1<br>Orufi11g01900.1 0.002 1 0.998 0.032 0.997	1<br>Oglab11g01770.1 0.002 1 0.998 0.068 1	1<br>Obart11g01960.3 0.107 1 0.893 0.023 1	0	0	0	1<br>Lperr12g01900.1 0.114 0.993 0.879 0.041 0.983	0
	0<br>Osjap06g22240.1 0.479 1 0.521 0.001 0.976	1<br>Osind11g01570.1 0.002 0.331 0.329 0.192 0.912<br>Osind12g02490.1 0.476 1 0.524 0.001 1	1<br>Orufi12g02050.1 0.001 0.357 0.356 0.002 1<br>Orufi12g02070.1 0.476 1 0.524 0.002 1	1<br>Oglab06g17410.1 0.471 0.999 0.528 0.017 0.999<br>Oglab12g01850.1 0.134 0.461 0.327 0.052 0.99	1<br>Obart12g01850.1 0.476 1 0.524 0.001 1<br>Obart12g01860.1 0.118 0.457 0.339 0.077 0.945	1<br>Olong_015795-R1 0.002 0.356 0.354 0.218 0.997<br>Olong_026769-R1 0.476 0.997 0.521 0.001 0.986	0<br>Opunc12g01810.1 0.22 0.999 0.779 0.023 0.999	0<br>Obrac11g01280.1 0.114 0.356 0.242 0.006 0.888<br>Obrac12g01600.1 0.476 0.997 0.521 0.001 0.999	0<br>Lperr11g01750.3 0.107 0.331 0.224 0.076 0.849<br>Lperr11g01770.1 0.476 1 0.524 0.001 1	0<br>AT5G45790.2 0.113 0.338 0.225 0.112 0.891<br>AT5G60900.1 0.497 0.996 0.499 0.043 0.96
Osjap12g02870.1	1<br>Osjap12g02870.1 0.001 1 0.999 0.001 1	0	0	0	0	0	0	0	0	0
	0	1	1	1	1	0	1	1	0	1

	Osjap12g02870.3 0.001 0.491 0.49 0.002 0.979	Osind12g02900.1 0.001 0.492 0.491 0.002 0.984<br>Osind12g02910.1 0.523 1 0.477 0.002 1	Orufi11g02580.1 0.523 1 0.477 0.002 1<br>Orufi12g02680.3 0.013 0.492 0.479 0.034 0.98	Oglab12g02350.1 0.013 0.492 0.479 0.027 0.984<br>Oglab12g02360.1 0.523 0.988 0.465 0.004 1	Obart11g02600.1 0.013 0.492 0.479 0.027 0.984<br>Obart12g02320.1 0.523 0.988 0.465 0.004 1	Olong_021395-R1 0.523 0.988 0.465 0.003 0.99<br>Olong_021396-R1 0.057 0.261 0.204 0.004 0.96	Opunc12g02350.2 0.001 0.492 0.491 0.001 0.615<br>Opunc12g02360.1 0.523 0.985 0.462 0.005 0.885	Obrac03g28840.1 0.607 1 0.393 0.573 0.882<br>Obrac12g02000.1 0.058 0.492 0.434 0.002 0.982	Lperr03g32810.1 0.607 1 0.393 0.608 0.882<br>Lperr12g02430.1 0.018 0.382 0.364 0.057 1	AT1G74040.1 0.06 0.484 0.424 0.114 0.97<br>AT3G13430.1 0.591 0.988 0.397 0.419 0.927
Osjap12g06950.3	1<br>Osjap12g06950.1 0.002 0.996 0.994 0.001 0.961<br>Osjap12g06950.2 0.002 0.996 0.994 0.002 0.833<br>Osjap12g06950.3 0.002 1 0.998 0.002 1<br>Osjap12g06950.4 0.002 1 0.998 0.002 1<br>Osjap12g06950.5 0.002 1 0.998 0.002 1<br>Osjap12g06950.6 0.076 1 0.924 0.002 1	0	0	0	0	0	0	0	0	0
	0<br>Osjap12g06950.7 0.017 0.744 0.727 0.066 0.925	1<br>Osind03g35000.1 0.761 0.981 0.22 0.065 0.374<br>Osind12g06720.1 0.076 0.74 0.664 0.002 0.827	1<br>Orufi01g21010.1 0.748 0.981 0.233 0.073 0.423<br>Orufi12g06430.2 0.017 0.744 0.727 0.001 0.956	1<br>Oglab11g11670.1 0.744 0.987 0.243 0.023 0.402<br>Oglab12g05700.2 0.017 0.732 0.715 0.003 0.987	0<br>Obart12g05660.3 0.002 0.744 0.742 0.002 1	1<br>Olong_002657-R2 0.108 0.738 0.63 0.081 0.94<br>Olong_028100-R1 0.772 0.981 0.209 0.05 0.371	1<br>Opunc07g20590.1 0.744 0.981 0.237 0.036 0.623<br>Opunc12g05420.1 0.002 0.736 0.734 0.003 0.985	0	0	0
Osjap12g06950.5	1<br>Osjap12g06950.1 0.002 0.996 0.994 0.001 0.966<br>Osjap12g06950.2 0.002 0.996 0.994 0.002 0.833<br>Osjap12g06950.3 0.002 1 0.998 0.002 1<br>Osjap12g06950.4 0.002 1 0.998 0.002 1<br>Osjap12g06950.5 0.002 1 0.998 0.002 1<br>Osjap12g06950.6 0.074 1 0.926 0.022 1	0	0	0	0	0	0	0	0	0
	0<br>Osjap12g06950.7 0.016 0.752 0.736 0.066 0.934	1<br>Osind03g35000.1 0.768 0.982 0.214 0.065 0.374<br>Osind12g06720.1 0.074 0.747 0.673 0.021 0.827	1<br>Orufi01g21010.1 0.756 0.982 0.226 0.073 0.423<br>Orufi12g06430.2 0.016 0.752 0.736 0.011 0.957	1<br>Oglab11g11670.1 0.752 0.988 0.236 0.023 0.402<br>Oglab12g05700.2 0.016 0.739 0.723 0.003 0.987	0<br>Obart12g05660.3 0.002 0.752 0.75 0.002 1	1<br>Olong_002657-R2 0.066 0.745 0.679 0.081 0.994<br>Olong_028100-R1 0.778 0.982 0.204 0.05 0.371	1<br>Opunc07g20590.1 0.752 0.982 0.23 0.036 0.623<br>Opunc12g05420.1 0.002 0.743 0.741 0.003 0.985	0	0	0
Osjap12g06950.6	1<br>Osjap12g06950.1 0.002 0.996 0.994 0.035 0.966<br>Osjap12g06950.2 0.002 0.996 0.994 0.061 0.833<br>Osjap12g06950.3 0.002 1 0.998 0.076 1<br>Osjap12g06950.4 0.002 1 0.998 0.073 1<br>Osjap12g06950.5 0.022 1 0.978 0.074 1<br>Osjap12g06950.6 0.002 1 0.998 0.002 1	0	0	0	0	0	0	0	0	0
	0<br>Osjap12g06950.7 0.002 0.738 0.736 0.139 0.934	1<br>Osind03g35000.1 0.755 0.98 0.225 0.065 0.374<br>Osind12g06720.1 0.002 0.733 0.731 0.002 0.827	1<br>Orufi01g21010.1 0.742 0.98 0.238 0.073 0.423<br>Orufi12g06430.5 0.002 0.738 0.736 0.003 1	1<br>Oglab11g11670.1 0.738 0.987 0.249 0.023 0.402<br>Oglab12g05700.2 0.002 0.725 0.723 0.028 0.987	1<br>Obart11g13140.1 0.779 0.98 0.201 0.015 0.47<br>Obart12g05660.3 0.002 0.738 0.736 0.055 1	1<br>Olong_002657-R2 0.035 0.731 0.696 0.081 0.994<br>Olong_028100-R1 0.766 0.98 0.214 0.05 0.371	1<br>Opunc07g20590.1 0.738 0.98 0.242 0.036 0.623<br>Opunc12g05420.1 0.024 0.729 0.705 0.116 0.985	0	0	0
Osjap12g12320.1	1<br>Osjap12g12320.1 0.001 1 0.999 0.001 1	1<br>Osind12g09530.1 0.001 1 0.999 0.001 1	1<br>Orufi12g10460.1 0.001 1 0.999 0.001 1	1<br>Oglab12g09470.1 0.001 1 0.999 0.001 1	1<br>Obart12g09410.1 0.001 1 0.999 0.001 1	0	0	0	0	0
	1<br>Osjap01g44780.2 0.363 0.998 0.635 0.086 0.994<br>Osjap03g07600.1 0.001 0.323 0.322 0.004 0.989	1<br>Osind01g46040.1 0.363 0.998 0.635 0.142 0.995<br>Osind03g07670.1 0.001 0.323 0.322 0.004 0.989	1<br>Orufi01g42650.3 0.363 0.998 0.635 0.186 0.995<br>Orufi03g07180.1 0.001 0.323 0.322 0.004 0.989	1<br>Oglab01g34040.1 0.425 0.998 0.573 0.203 0.995<br>Oglab03g07280.1 0.001 0.323 0.322 0.004 0.989	1<br>Obart01g39260.3 0.388 0.998 0.61 0.039 0.994<br>Obart03g07210.1 0.001 0.323 0.322 0.004 0.989	1<br>Olong_021912-R2 0.373 1 0.627 0.008 1<br>Olong_021913-R1 0.001 0.321 0.32 0.004 0.988	1<br>Opunc02g11250.1 0.001 0.323 0.322 0.372 0.993<br>Opunc12g08570.1 0.39 1 0.61 0.211 1	1<br>Obrac03g05880.1 0.001 0.323 0.322 0.004 0.992<br>Obrac12g06500.1 0.395 1 0.605 0.103 1	1<br>Lperr03g06600.1 0.001 0.323 0.322 0.004 0.992<br>Lperr12g08310.1 0.399 0.999 0.6 0.002 0.998	1<br>AT3G49880.1 0.426 0.995 0.569 0.032 0.987<br>AT4G34670.1 0.001 0.315 0.314 0.004 0.966

Osjap12g18490.1	1<br>Osjap12g18490.1 0.001 1 0.999 0.001 1	0	0	1<br>Oglab12g13500.1 0.007 1 0.993 0.001 1	0	0	0	0	0	0
	0<br>Osjap12g18490.3 0.001 0.678 0.677 0.002 1	1<br>Osind03g14600.1 0.678 0.993 0.315 0.127 0.978<br>Osind12g14860.1 0.007 0.678 0.671 0.002 1	1<br>Orufi09g14200.1 0.706 0.993 0.287 0.221 0.981<br>Orufi12g15970.1 0.007 0.678 0.671 0.002 1	1<br>Oglab01g13480.1 0.025 0.637 0.612 0.114 0.97<br>Oglab08g16770.1 0.667 0.993 0.326 0.049 0.982	1<br>Obart09g13250.1 0.706 0.993 0.287 0.191 0.981<br>Obart12g14470.1 0.007 0.678 0.671 0.002 1	1<br>Olong_005042-R1 0.672 0.993 0.321 0.037 0.987<br>Olong_022838-R1 0.099 0.637 0.538 0.002 0.982	1<br>Opunc01g16700.1 0.021 0.637 0.616 0.124 0.971<br>Opunc08g16120.1 0.667 0.993 0.326 0.049 0.982	0<br>Obrac12g09310.2 0.059 0.678 0.619 0.068 1	1<br>Lperr01g14290.1 0.025 0.637 0.612 0.006 0.965<br>Lperr12g11870.1 0.673 1 0.327 0.112 1	1<br>AT3G06350.1 0.019 0.64 0.621 0.148 0.978<br>AT4G24570.1 0.647 0.993 0.346 0.22 0.99
Osjap12g19810.1	1<br>Osjap12g19810.1 0.002 1 0.998 0.002 1	1<br>Osind12g15880.1 0.002 1 0.998 0.002 1	0	0	0	0	0	0	0	0
	1<br>Osjap07g08090.1 0.149 0.629 0.48 0.642 0.97<br>Osjap12g19770.1 0.651 1 0.349 0.006 1	1<br>Osind04g18390.2 0.133 0.627 0.494 0.648 0.967<br>Osind12g15880.2 0.638 1 0.362 0.012 1	1<br>Orufi03g39910.2 0.149 0.629 0.48 0.617 0.969<br>Orufi12g17100.1 0.629 1 0.371 0.035 1	1<br>Oglab06g04730.1 0.149 0.629 0.48 0.655 0.974<br>Oglab12g14370.2 0.642 1 0.358 0.115 1	1<br>Obart03g38270.2 0.149 0.629 0.48 0.61 0.968<br>Obart12g15400.1 0.651 1 0.349 0.006 1	1<br>Olong_001697-R1 0.651 1 0.349 0.006 1<br>Olong_022562-R1 0.149 0.629 0.48 0.615 0.971	1<br>Opunc03g35430.1 0.149 0.629 0.48 0.637 0.97<br>Opunc12g13920.1 0.64 1 0.36 0.003 1	1<br>Obrac06g03630.1 0.129 0.622 0.493 0.588 0.894<br>Obrac12g09830.1 0.649 1 0.351 0.003 1	1<br>Lperr07g06070.1 0.149 0.629 0.48 0.64 0.969<br>Lperr12g12590.1 0.651 1 0.349 0.006 1	0<br>AT4G01970.1 0.149 0.651 0.502 0.688 0.985
Osjap12g24450.1	1<br>Osjap12g24450.1 0.001 1 0.999 0.001 1	0	0	0	0	0	0	0	0	0
	1<br>Osjap03g06030.1 0.06 0.59 0.53 0.122 0.973<br>Osjap07g31120.1 0.6 0.992 0.392 0.008 0.974	1<br>Osind12g20480.1 0.04 0.591 0.551 0.076 0.975<br>Osind12g20490.1 0.599 0.996 0.397 0.003 0.982	1<br>Orufi12g21590.1 0.039 0.591 0.552 0.063 0.978<br>Orufi12g21600.1 0.599 0.996 0.397 0.003 0.982	1<br>Oglab12g17520.1 0.039 0.591 0.552 0.063 0.978<br>Oglab12g17530.1 0.599 0.996 0.397 0.003 0.982	1<br>Obart03g05850.1 0.06 0.59 0.53 0.122 0.973<br>Obart12g19340.1 0.599 0.996 0.397 0.002 0.98	1<br>Olong_000677-R1 0.325 0.589 0.264 0.093 0.96<br>Olong_002428-R1 0.06 0.315 0.255 0.188 0.726<br>Olong_008690-R1 0.6 0.992 0.392 0.008 0.974	1<br>Opunc12g17520.1 0.001 0.591 0.59 0.002 0.983<br>Opunc12g17530.1 0.599 0.995 0.396 0.001 0.985	0<br>Obrac07g18220.1 0.6 0.986 0.386 0.008 0.958<br>Obrac12g12740.1 0.07 0.4 0.33 0.004 0.986	1<br>Lperr07g23910.1 0.595 0.991 0.396 0.006 0.967<br>Lperr12g16150.1 0.008 0.591 0.583 0.003 0.979	0<br>AT5G17850.1 0.047 0.586 0.539 0.05 0.971

((((((_b1,_a2),longi),punctata),brachy),leersia),tair);

chimeric gene id	INGROUP			MIDGROUP				OUTGROUP		
	jap	indica	rufi	glab	barthii	longi	punctata	brachy	leersia	tair
Osjap01g00640.1	1<br>Osjap01g00640.1 0.001 1 0.999 0.001 1<br>Osjap01g00640.2 0.001 1 0.999 0.001 1<br>Osjap01g00640.3 0.001 1 0.999 0.001 1<br>Osjap01g00640.4 0.001 1 0.999 0.001 1	1<br>Osind01g00770.1 0.105 1 0.895 0.022 1<br>Osind01g00770.2 0.105 1 0.895 0.027 1	0	0	0	0	0	0	0	0
	1<br>Osjap04g25390.2 0.57 1 0.43 0.019 1<br>Osjap05g00080.1 0.001 0.451 0.45 0.007 1	1<br>Osind04g24430.1 0.57 1 0.43 0.019 1<br>Osind05g00060.1 0.001 0.451 0.45 0.007 1	1<br>Orufi01g00590.1 0.565 1 0.435 0.001 1<br>Orufi05g00070.1 0.001 0.451 0.45 0.007 1	1<br>Oglab01g00510.1 0.565 1 0.435 0.001 1<br>Oglab05g00040.1 0.001 0.451 0.45 0.007 1	1<br>Obart01g00590.1 0.565 1 0.435 0.001 1<br>Obart05g00050.1 0.001 0.451 0.45 0.007 1	1<br>Olong_015014-R1 0.49 1 0.51 0.055 1<br>Olong_015017-R1 0.001 0.451 0.45 0.001 1	1<br>Opunc01g00510.1 0.001 0.451 0.45 0.078 1<br>Opunc09g01380.1 0.554 1 0.446 0.03 1	1<br>Obrac05g00060.2 0.001 0.451 0.45 0.008 1<br>Obrac09g00870.1 0.564 1 0.436 0.052 1	1<br>Lperr01g00510.1 0.001 0.451 0.45 0.007 1<br>Lperr09g00030.1 0.564 1 0.436 0.031 1	1<br>AT1G20380.1 0.565 1 0.435 0.001 1<br>AT5G08440.2 0.002 0.45 0.448 0.012 0.997
Osjap01g00640.2	1<br>Osjap01g00640.1 0.001 1 0.999 0.001 1<br>Osjap01g00640.2 0.001 1 0.999 0.001 1<br>Osjap01g00640.3 0.001 1 0.999 0.001 1<br>Osjap01g00640.4 0.001 1 0.999 0.001 1	1<br>Osind01g00770.1 0.107 1 0.893 0.022 1<br>Osind01g00770.2 0.107 1 0.893 0.027 1	0	0	0	0	0	0	0	0
	1<br>Osjap04g25390.2 0.565 1 0.435 0.019 1	1<br>Osind04g24430.1 0.565 1 0.435 0.019 1	1<br>Orufi01g00590.1 0.558 1 0.442 0.001 1	1<br>Oglab01g00510.1 0.558 1 0.442 0.001 1	1<br>Obart01g00590.1 0.558 1 0.442 0.001 1	1<br>Olong_015014-R1 0.493 1 0.507 0.07 1	1<br>Opunc01g00510.1 0.001 0.457 0.456 0.078 1	1<br>Obrac05g00060.2 0.001 0.457 0.456 0.008 1	1<br>Lperr01g00510.1 0.001 0.457 0.456 0.007 1	1<br>AT1G20380.1 0.558 1 0.442 0.001 1

	Osjap05g00080.1 0.001 0.457 0.456 0.007 1	Osind05g00060.1 0.001 0.457 0.456 0.007 1	Orufi05g00070.1 0.001 0.457 0.456 0.007 1	Oglab05g00040.1 0.001 0.457 0.456 0.007 1	Obart05g00050.1 0.001 0.457 0.456 0.007 1	Olong_015017-R1 0.001 0.457 0.456 0.001 1	Opunc09g01380.1 0.548 1 0.452 0.03 1	Obrac09g00870.1 0.558 1 0.442 0.052 1	Lperr09g00030.1 0.558 1 0.442 0.031 1	AT5G08440.2 0.002 0.457 0.455 0.012 0.997
Osjap01g00640.3	1<br>Osjap01g00640.1 0.001 1 0.999 0.001 1<br>Osjap01g00640.2 0.001 1 0.999 0.001 1<br>Osjap01g00640.3 0.001 1 0.999 0.001 1<br>Osjap01g00640.4 0.001 1 0.999 0.001 1	1<br>Osind01g00770.1 0.107 1 0.893 0.022 1<br>Osind01g00770.2 0.107 1 0.893 0.027 1	0	0	0	0	0	0	0	0
	1<br>Osjap04g25390.2 0.564 1 0.436 0.019 1<br>Osjap05g00080.1 0.001 0.458 0.457 0.007 0.999	1<br>Osind04g24430.1 0.564 1 0.436 0.019 1<br>Osind05g00060.1 0.001 0.458 0.457 0.007 0.999	1<br>Orufi01g00590.1 0.558 1 0.442 0.001 1<br>Orufi05g00070.1 0.001 0.458 0.457 0.007 0.999	1<br>Oglab01g00510.1 0.558 1 0.442 0.001 1<br>Oglab05g00040.1 0.001 0.458 0.457 0.007 0.999	1<br>Obart01g00590.1 0.558 1 0.442 0.001 1<br>Obart05g00050.1 0.001 0.458 0.457 0.007 0.999	1<br>Olong_015014-R1 0.459 1 0.541 0.001 1<br>Olong_015017-R1 0.001 0.458 0.457 0.001 0.999	1<br>Opunc01g00510.1 0.001 0.458 0.457 0.078 0.999<br>Opunc09g01380.1 0.547 1 0.453 0.03 1	1<br>Obrac05g00060.2 0.001 0.458 0.457 0.008 0.998<br>Obrac09g00870.1 0.557 1 0.443 0.052 1	1<br>Lperr01g00510.1 0.001 0.458 0.457 0.007 0.998<br>Lperr09g00030.1 0.557 1 0.443 0.031 1	1<br>AT1G20380.1 0.558 1 0.442 0.001 1<br>AT5G08440.2 0.002 0.458 0.456 0.012 0.999
Osjap01g00640.4	1<br>Osjap01g00640.1 0.001 1 0.999 0.001 1<br>Osjap01g00640.2 0.001 1 0.999 0.001 1<br>Osjap01g00640.3 0.001 1 0.999 0.001 1<br>Osjap01g00640.4 0.001 1 0.999 0.001 1	1<br>Osind01g00770.1 0.109 1 0.891 0.022 1<br>Osind01g00770.2 0.109 1 0.891 0.027 1	0	0	0	0	0	0	0	0
	1<br>Osjap04g25390.2 0.555 1 0.445 0.019 1<br>Osjap05g00080.1 0.001 0.467 0.466 0.007 0.999	1<br>Osind04g24430.1 0.555 1 0.445 0.019 1<br>Osind05g00060.1 0.001 0.467 0.466 0.007 0.999	1<br>Orufi01g00590.1 0.549 1 0.451 0.001 1<br>Orufi05g00070.1 0.001 0.467 0.466 0.007 0.999	1<br>Oglab01g00510.1 0.549 1 0.451 0.001 1<br>Oglab05g00040.1 0.001 0.467 0.466 0.007 0.999	1<br>Obart01g00590.1 0.549 1 0.451 0.001 1<br>Obart05g00050.1 0.001 0.467 0.466 0.007 0.999	1<br>Olong_015014-R1 0.468 1 0.532 0.001 1<br>Olong_015017-R1 0.001 0.467 0.466 0.001 0.999	1<br>Opunc01g00510.1 0.001 0.467 0.466 0.078 0.999<br>Opunc09g01380.1 0.538 1 0.462 0.03 1	1<br>Obrac05g00060.2 0.001 0.467 0.466 0.008 0.998<br>Obrac09g00870.1 0.548 1 0.452 0.052 1	1<br>Lperr01g00510.1 0.001 0.467 0.466 0.007 0.998<br>Lperr09g00030.1 0.548 1 0.452 0.031 1	1<br>AT1G20380.1 0.549 1 0.451 0.001 1<br>AT5G08440.2 0.002 0.467 0.465 0.012 0.999
Osjap01g08660.1	1<br>Osjap01g08660.1 0.001 1 0.999 0.001 1	1<br>Osind01g08490.1 0.001 1 0.999 0.001 1	1<br>Orufi01g08070.1 0.001 1 0.999 0.001 1	1<br>Oglab01g06470.1 0.001 1 0.999 0.001 1	1<br>Obart01g07430.1 0.017 1 0.983 0.001 1	1<br>Olong_011567-R1 0.068 1 0.932 0.001 1	0	0	0	0
	1<br>Osjap01g29230.2 0.111 0.579 0.468 0.25 0.983<br>Osjap05g00560.2 0.656 0.997 0.341 0.146 1	1<br>Osind01g30690.1 0.111 0.579 0.468 0.267 0.982<br>Osind05g00540.1 0.656 0.997 0.341 0.126 1	0<br>Orufi01g27950.1 0.111 0.579 0.468 0.25 0.983<br>Orufi01g48560.2 0.656 0.98 0.324 0.085 0.984	1<br>Oglab01g21070.2 0.111 0.579 0.468 0.249 0.983<br>Oglab05g00530.1 0.656 0.997 0.341 0.146 1	1<br>Obart01g25090.1 0.111 0.579 0.468 0.219 0.982<br>Obart05g00540.1 0.656 0.997 0.341 0.146 1	0<br>Olong_003039-R1 0.656 0.997 0.341 0.128 1<br>Olong_003692-R2 0.149 0.579 0.43 0.244 0.979	1<br>Opunc01g07160.1 0.589 1 0.411 0.111 1<br>Opunc01g07170.1 0.001 0.58 0.579 0.001 0.984	1<br>Obrac01g05760.3 0.594 1 0.406 0.082 1<br>Obrac01g05770.3 0.001 0.58 0.579 0.001 0.985	1<br>Lperr01g07030.1 0.594 1 0.406 0.018 1<br>Lperr01g07040.2 0.001 0.58 0.579 0.001 0.989	1<br>AT1G54350.1 0.592 0.993 0.401 0.076 0.975<br>AT5G53430.1 0.023 0.579 0.556 0.09 0.984
Osjap01g13630.1	1<br>Osjap01g13630.1 0.001 1 0.999 0.001 1	0	1<br>Orufi01g13080.1 0.042 1 0.958 0.043 1	1<br>Oglab01g10320.1 0.042 1 0.958 0.042 1	0	0	0	1<br>Obrac01g09290.1 0.001 1 0.999 0.001 1	0	0
	0<br>Osjap01g13630.3 0.459 1 0.541 0.002 1	1<br>Osind01g13830.1 0.535 1 0.465 0.002 1<br>Osind01g13840.1 0.001 0.449 0.448 0.002 0.99	1<br>Orufi01g13080.4 0.459 1 0.541 0.002 1<br>Orufi01g13090.1 0.001 0.449 0.448 0.002 0.99	0<br>Oglab01g10320.2 0.459 1 0.541 0.002 1	1<br>Obart01g12120.1 0.535 1 0.465 0.002 1<br>Obart01g12130.1 0.001 0.487 0.486 0.002 0.998	1<br>Olong_021508-R1 0.53 1 0.47 0.062 1<br>Olong_021509-R1 0.077 0.449 0.372 0.005 0.99	1<br>Opunc01g11830.1 0.031 0.487 0.456 0.054 0.998<br>Opunc05g02420.2 0.535 1 0.465 0.002 1	0<br>Obrac05g02000.1 0.535 1 0.465 0.002 1	1<br>Lperr01g11090.1 0.001 0.458 0.457 0.002 0.984<br>Lperr05g21710.3 0.531 1 0.469 0.008 1	1<br>AT3G62130.1 0.025 0.444 0.419 0.013 0.993<br>AT4G04460.1 0.545 1 0.455 0.022 1
Osjap01g31520.1	1<br>Osjap01g31520.1 0.001 1 0.999 0.001 1	0	1<br>Orufi01g30230.1 0.006 0.972 0.966 0.014 0.955<br>Orufi01g30230.2 0.006 0.972 0.966 0.014 0.953<br>Orufi01g30230.3 0.006 0.972 0.966 0.015 0.952	1<br>Oglab01g23070.1 0.018 0.974 0.956 0.011 0.935	1<br>Obart01g27180.1 0.01 0.974 0.964 0.002 0.926	0	0	1<br>Obrac01g20650.1 0.021 0.96 0.939 0.109 0.926	0	0
	1<br>Osjap01g14430.1 0.722 0.966 0.244 0.019 1<br>Osjap01g31460.1 0.007 0.707 0.7 0.001 0.735	1<br>Osind01g32720.1 0.721 0.964 0.243 0.003 0.99<br>Osind05g28330.1 0.001 0.707 0.706 0.001 0.899	0<br>Orufi01g30230.4 0.018 0.707 0.689 0.016 0.905	0<br>Oglab01g09850.1 0.722 0.966 0.244 0.019 1<br>Oglab01g23050.1 0.031 0.556 0.525 0.081 0.754	1<br>Obart01g25360.1 0.723 0.966 0.243 0.017 1<br>Obart01g27130.1 0.031 0.707 0.676 0.032 0.604	1<br>Olong_000377-R1 0.721 0.964 0.243 0.003 0.99<br>Olong_000393-R1 0.007 0.707 0.7 0.002 0.78	1<br>Opunc01g27140.1 0.007 0.707 0.7 0.001 1<br>Opunc01g36860.1 0.73 0.979 0.249 0.02 1	1<br>Obrac01g20500.1 0.721 0.964 0.243 0.003 0.99<br>Obrac01g20630.1 0.007 0.707 0.7 0.001 0.801	1<br>Lperr01g11680.1 0.722 0.966 0.244 0.007 1<br>Lperr01g23240.1 0.006 0.707 0.701 0.01 0.803	1<br>AT1G67000.1 0.036 0.707 0.671 0.285 0.843<br>AT5G24090.1 0.724 0.966 0.242 0.036 1

Osjap01g42770.1	1<br>Osjap01g42770.1 0.001 1 0.999 0.001 1	1<br>Osind01g44140.1 0.001 1 0.999 0.001 1	1<br>Orufi01g40790.1 0.001 1 0.999 0.001 1<br>Orufi01g40790.2 0.001 1 0.999 0.001 1<br>Orufi01g40790.3 0.001 1 0.999 0.001 1	0	0	1<br>Olong_024830-R1 0.001 1 0.999 0.001 1	0	0	0	0
	1<br>Osjap01g08100.1 0.012 0.27 0.258 0.133 0.963<br>Osjap03g27560.1 0.413 0.995 0.582 0.146 0.95	1<br>Osind01g08020.1 0.012 0.27 0.258 0.133 0.963<br>Osind03g28340.1 0.413 0.995 0.582 0.146 0.95	1<br>Orufi02g20240.3 0.012 0.259 0.247 0.044 0.951<br>Orufi03g26160.3 0.413 0.995 0.582 0.146 0.95	1<br>Oglab01g32220.1 0.001 0.258 0.257 0.004 0.934<br>Oglab01g32230.1 0.352 0.999 0.647 0.002 0.955	1<br>Obart01g37410.1 0.001 0.258 0.257 0.004 0.934<br>Obart01g37420.1 0.352 1 0.648 0.002 1	1<br>Olong_010570-R1 0.413 0.995 0.582 0.133 0.915<br>Olong_011675-R1 0.012 0.258 0.246 0.517 0.972	1<br>Opunc01g36330.1 0.001 0.258 0.257 0.005 0.975<br>Opunc01g36340.1 0.352 0.999 0.647 0.002 0.969	1<br>Obrac01g28830.1 0.001 0.257 0.256 0.004 0.978<br>Obrac01g28840.1 0.355 1 0.645 0.002 1	1<br>Lperr01g32130.1 0.001 0.258 0.257 0.005 0.977<br>Lperr01g32140.1 0.352 1 0.648 0.002 1	0<br>AT2G37970.1 0.001 0.258 0.257 0.049 0.978<br>AT3G54090.1 0.461 1 0.539 0.123 1
Osjap02g28800.3	1<br>Osjap02g28800.1 0.001 1 0.999 0.001 1<br>Osjap02g28800.2 0.001 1 0.999 0.001 1<br>Osjap02g28800.3 0.001 1 0.999 0.001 1	0	1<br>Orufi02g26700.1 0.001 0.99 0.989 0.001 0.965	0	0	1<br>Olong_027102-R1 0.144 1 0.856 0.033 1	1<br>Opunc02g23140.1 0.001 1 0.999 0.001 1	0	1<br>Lperr02g20690.1 0.001 1 0.999 0.001 1	0
	1<br>Osjap04g33800.1 0.049 0.673 0.624 0.039 0.974<br>Osjap11g07330.1 0.688 0.998 0.31 0.005 0.995	1<br>Osind02g28600.2 0.684 1 0.316 0.003 1<br>Osind02g28610.1 0.001 0.673 0.672 0.001 0.982	1<br>Orufi02g26700.2 0.001 0.673 0.672 0.001 0.982<br>Orufi11g06530.1 0.688 1 0.312 0.005 1	1<br>Oglab03g04200.1 0.039 0.673 0.634 0.004 0.987<br>Oglab11g05820.1 0.688 0.998 0.31 0.005 0.995	1<br>Obart02g25330.2 0.684 1 0.316 0.003 1<br>Obart02g25340.1 0.001 0.673 0.672 0.001 0.982	1<br>Olong_010559-R1 0.675 0.996 0.321 0.126 0.991<br>Olong_017224-R1 0.068 0.673 0.605 0.014 0.986	0<br>Opunc02g23140.2 0.001 0.673 0.672 0.001 0.982	1<br>Obrac02g18260.1 0.684 1 0.316 0.003 1<br>Obrac02g18270.1 0.001 0.673 0.672 0.001 0.982	1<br>Lperr02g11790.1 0.051 0.673 0.622 0.079 0.981<br>Lperr10g02180.1 0.683 0.998 0.315 0.035 1	1<br>AT1G22440.1 0.686 1 0.314 0.01 1<br>AT5G23390.1 0.001 0.673 0.672 0.016 0.981
Osjap02g30600.1	1<br>Osjap02g30600.1 0.003 1 0.997 0.003 1<br>Osjap02g30600.2 0.003 1 0.997 0.003 1	1<br>Osind02g30400.1 0.032 1 0.968 0.016 1<br>Osind02g30400.2 0.032 1 0.968 0.017 1	1<br>Orufi02g28440.1 0.003 1 0.997 0.003 1	0	0	0	0	0	0	0
	1<br>Osjap04g25080.3 0.508 0.994 0.486 0.153 0.992<br>Osjap04g31350.1 0.003 0.412 0.409 0.004 0.5	0<br>Osind04g24130.1 0.508 0.936 0.428 0.172 0.866<br>Osind05g14500.1 0.026 0.305 0.279 0.129 0.714	1<br>Orufi02g28440.3 0.331 1 0.669 0.005 1<br>Orufi05g00390.1 0.077 0.283 0.206 0.156 0.397	1<br>Oglab02g10070.1 0.003 0.293 0.29 0.002 0.255<br>Oglab02g25140.1 0.331 1 0.669 0.005 1	1<br>Obart02g26980.2 0.331 1 0.669 0.005 1<br>Obart05g12910.2 0.08 0.331 0.251 0.152 0.569	0<br>Olong_009904-R1 0.331 1 0.669 0.005 1	1<br>Opunc02g24620.1 0.428 1 0.572 0.141 1<br>Opunc03g26570.3 0.003 0.305 0.302 0.006 0.75	0<br>Obrac02g19560.1 0.437 1 0.563 0.133 1	0<br>Lperr02g21920.1 0.418 1 0.582 0.442 1	0<br>AT1G01730.1 0.46 0.99 0.53 0.223 0.933
Osjap02g40340.1	1<br>Osjap02g40310.1 0.003 0.982 0.979 0.162 0.926<br>Osjap02g40340.1 0.003 1 0.997 0.003 1<br>Osjap02g40370.1 0.003 0.982 0.979 0.002 0.726	1<br>Osind02g39860.1 0.003 0.985 0.982 0.002 0.659	0	0	0	0	0	0	0	0
	1<br>Osjap02g41540.1 0.655 0.997 0.342 0.434 0.806<br>Osjap03g26380.1 0.003 0.292 0.289 0.004 0.406<br>Osjap05g23970.1 0.368 0.591 0.223 0.665 0.821	1<br>Osind02g39840.1 0.003 0.292 0.289 0.007 0.809<br>Osind02g39860.2 0.332 0.985 0.653 0.015 0.571	0<br>Orufi02g37680.6 0.332 0.992 0.66 0.022 0.879	1<br>Oglab02g33340.1 0.361 1 0.639 0.042 1<br>Oglab03g24170.1 0.003 0.292 0.289 0.004 0.406	1<br>Obart02g36180.3 0.332 0.992 0.66 0.019 0.724<br>Obart03g24380.1 0.003 0.292 0.289 0.004 0.406	1<br>Olong_011081-R1 0.652 1 0.348 0.05 1<br>Olong_011082-R1 0.003 0.294 0.291 0.007 0.752<br>Olong_025373-R1 0.299 0.606 0.307 0.116 0.574	1<br>Opunc02g34450.1 0.655 0.997 0.342 0.432 0.806<br>Opunc03g22570.2 0.003 0.294 0.291 0.007 0.816<br>Opunc04g19490.1 0.368 0.588 0.22 0.521 0.726	1<br>Obrac02g27500.6 0.655 0.997 0.342 0.432 0.806<br>Obrac03g19420.1 0.003 0.292 0.289 0.007 0.81<br>Obrac05g14470.1 0.368 0.588 0.22 0.584 0.771	1<br>Lperr02g29800.1 0.018 0.292 0.274 0.234 0.846<br>Lperr02g30750.1 0.655 0.997 0.342 0.453 0.814<br>Lperr06g23460.1 0.368 0.606 0.238 0.548 0.76	1<br>AT1G76310.1 0.376 0.606 0.23 0.571 0.773<br>AT2G20360.1 0.655 0.997 0.342 0.423 0.801<br>AT5G38650.1 0.003 0.292 0.289 0.007 0.809
Osjap03g00680.1	1<br>Osjap03g00680.1 0.001 1 0.999 0.001 1<br>Osjap03g00680.2 0.001 1 0.999 0.001 1<br>Osjap03g00680.3 0.001 1 0.999 0.001 1	1<br>Osind03g00740.1 0.001 0.994 0.993 0.001 0.996	1<br>Orufi03g12660.1 0.001 1 0.999 0.001 1<br>Orufi03g12660.2 0.001 1 0.999 0.001 1	1<br>Oglab03g00390.1 0.001 1 0.999 0.001 1	1<br>Obart03g00550.1 0.001 0.994 0.993 0.001 0.996	0	0	0	0	0
	1<br>Osjap02g42090.1 0.051 0.661 0.61 0.065 1<br>Osjap12g08580.1 0.71 0.999 0.289 0.011 0.996	1<br>Osind02g41670.1 0.06 0.661 0.601 0.131 1<br>Osind03g02220.1 0.7 1 0.3 0.015 1	1<br>Orufi02g39350.1 0.06 0.661 0.601 0.131 1<br>Orufi09g02220.2 0.746 1 0.254 0.095 1	1<br>Oglab02g34920.1 0.06 0.661 0.601 0.131 1<br>Oglab03g02050.1 0.688 1 0.312 0.187 1	1<br>Obart02g37790.1 0.06 0.632 0.572 0.133 0.977<br>Obart12g06510.1 0.685 0.999 0.314 0.066 0.997	0<br>Olong_013175-R1 0.264 0.904 0.64 0.002 0.961	1<br>Opunc03g00470.1 0.001 0.783 0.782 0.002 1<br>Opunc11g04590.1 0.783 0.998 0.215 0.055 1	1<br>Obrac02g10260.1 0.712 0.999 0.287 0.265 0.995<br>Obrac02g27970.1 0.134 0.661 0.527 0.105 1	1<br>Lperr03g00570.1 0.001 0.657 0.656 0.002 0.994<br>Lperr04g11360.1 0.752 0.998 0.246 0.415 0.998	0<br>AT5G04460.1 0.017 0.661 0.644 0.081 1

Osjap03g00680.2	1<br>Osjap03g00680.1 0.001 1 0.999 0.001 1<br>Osjap03g00680.2 0.001 1 0.999 0.001 1<br>Osjap03g00680.3 0.001 1 0.999 0.001 1	1<br>Osind03g00740.1 0.001 0.994 0.993 0.001 0.996	1<br>Orufi03g12660.1 0.001 1 0.999 0.001 1<br>Orufi03g12660.2 0.001 1 0.999 0.001 1	1<br>Oglab03g00390.1 0.001 1 0.999 0.001 1	1<br>Obart03g00550.1 0.001 0.994 0.993 0.001 0.996	0	0	0	0	0
	1<br>Osjap02g42090.1 0.052 0.68 0.628 0.065 1<br>Osjap12g08580.1 0.702 0.999 0.297 0.011 0.996	1<br>Osind02g41670.1 0.062 0.68 0.618 0.131 1<br>Osind03g02220.1 0.691 1 0.309 0.015 1	1<br>Orufi02g39350.1 0.062 0.68 0.618 0.131 1<br>Orufi09g02220.2 0.738 1 0.262 0.095 1	1<br>Oglab02g34920.1 0.062 0.68 0.618 0.131 1<br>Oglab03g02050.1 0.691 1 0.309 0.214 1	1<br>Obart02g37790.1 0.062 0.65 0.588 0.133 0.977<br>Obart12g06510.1 0.681 0.999 0.318 0.079 0.997	0<br>Olong_013175-R1 0.272 0.902 0.63 0.002 0.961	1<br>Opunc03g00470.1 0.001 0.777 0.776 0.002 1<br>Opunc03g03130.2 0.777 0.999 0.222 0.443 0.997	1<br>Obrac02g10260.1 0.704 0.999 0.295 0.265 0.995<br>Obrac02g27970.1 0.138 0.68 0.542 0.105 1	1<br>Lperr03g00570.1 0.001 0.68 0.679 0.002 1<br>Lperr04g11360.1 0.745 0.998 0.253 0.415 0.998	0<br>AT5G04460.1 0.018 0.68 0.662 0.081 1
Osjap03g00710.1	1<br>Osjap03g00710.1 0.002 1 0.998 0.002 1	0	1<br>Orufi03g12690.1 0.002 1 0.998 0.002 1<br>Orufi03g12690.2 0.002 1 0.998 0.002 1	0	0	0	0	0	0	0
	1<br>Osjap08g14010.2 0.424 0.983 0.559 0.444 0.966<br>Osjap10g20350.1 0.002 0.245 0.243 0.15 0.969	1<br>Osind03g00760.1 0.316 0.994 0.678 0.002 0.841<br>Osind03g00790.1 0.002 0.28 0.278 0.006 0.879	1<br>Orufi08g14730.1 0.424 0.983 0.559 0.503 0.97<br>Orufi10g18610.1 0.002 0.245 0.243 0.15 0.969	0<br>Oglab03g00420.2 0.234 0.994 0.76 0.128 0.895	1<br>Obart03g00580.1 0.316 1 0.684 0.003 0.966<br>Obart03g00600.1 0.002 0.28 0.278 0.006 0.879	1<br>Olong_002892-R1 0.002 0.245 0.243 0.16 0.969<br>Olong_013172-R3 0.355 0.998 0.643 0.201 0.985	1<br>Opunc03g00510.2 0.316 0.994 0.678 0.003 0.891<br>Opunc03g00530.1 0.002 0.237 0.235 0.008 0.977	1<br>Obrac03g00430.2 0.355 0.993 0.638 0.213 0.962<br>Obrac10g11260.2 0.002 0.237 0.235 0.182 0.929	1<br>Lperr03g00610.3 0.316 0.987 0.671 0.004 0.948<br>Lperr10g13450.1 0.006 0.245 0.239 0.167 0.969	1<br>AT3G52180.1 0.316 0.993 0.677 0.003 0.982<br>AT5G03455.1 0.002 0.237 0.235 0.116 0.979
Osjap03g13100.1	1<br>Osjap03g13100.1 0.001 1 0.999 0.001 1	1<br>Osind03g12930.1 0.001 1 0.999 0.028 1	0	1<br>Oglab03g12120.1 0.001 1 0.999 0.028 1	1<br>Obart03g12120.1 0.001 1 0.999 0.028 1	0	0	0	0	0
	1<br>Osjap04g16240.1 0.632 0.998 0.366 0.082 0.921<br>Osjap12g01140.1 0.084 0.622 0.538 0.498 0.981	1<br>Osind04g14790.1 0.632 0.998 0.366 0.013 0.911<br>Osind12g01290.1 0.084 0.622 0.538 0.49 0.981	1<br>Orufi06g01460.3 0.632 0.998 0.366 0.212 0.912<br>Orufi12g01130.1 0.084 0.622 0.538 0.498 0.981	1<br>Oglab06g01620.1 0.632 0.998 0.366 0.233 0.912<br>Oglab12g00800.1 0.089 0.622 0.533 0.483 0.983	1<br>Obart06g01490.1 0.632 0.998 0.366 0.233 0.912<br>Obart11g01060.1 0.089 0.622 0.533 0.471 0.982	1<br>Olong_007714-R9 0.632 1 0.368 0.002 1<br>Olong_007715-R1 0.1 0.623 0.523 0.007 0.965	1<br>Opunc01g28650.1 0.636 0.998 0.362 0.094 0.923<br>Opunc03g11680.1 0.001 0.623 0.622 0.001 0.971	1<br>Obrac03g09720.2 0.001 0.623 0.622 0.001 0.97<br>Obrac04g09430.1 0.63 0.998 0.368 0.014 0.863	1<br>Lperr03g10720.1 0.001 0.623 0.622 0.001 0.934<br>Lperr03g17470.1 0.623 0.998 0.375 0.119 0.828	1<br>AT1G20230.1 0.635 0.998 0.363 0.104 0.833<br>AT3G46740.1 0.097 0.623 0.526 0.158 0.971
Osjap03g30460.1	1<br>Osjap03g30460.1 0.001 1 0.999 0.001 1	1<br>Osind03g31500.1 0.018 1 0.982 0.031 1	1<br>Orufi03g29110.1 0.018 1 0.982 0.296 1	0	1<br>Obart03g27980.1 0.08 1 0.92 0.016 1<br>Obart03g27980.2 0.08 1 0.92 0.015 1	0	0	0	0	0
	1<br>Osjap03g30380.1 0.547 0.999 0.452 0.059 0.866<br>Osjap12g24400.2 0.083 0.517 0.434 0.012 0.988	1<br>Osind08g14630.1 0.547 0.999 0.452 0.069 0.96<br>Osind12g20440.1 0.083 0.517 0.434 0.01 0.99	1<br>Orufi03g29970.1 0.547 0.999 0.452 0.035 0.998<br>Orufi12g21550.1 0.083 0.517 0.434 0.01 0.99	1<br>Oglab03g27330.1 0.547 1 0.453 0.036 1<br>Oglab12g17460.4 0.083 0.517 0.434 0.012 0.996	1<br>Obart03g27900.1 0.547 0.999 0.452 0.053 0.865<br>Obart12g19300.1 0.083 0.517 0.434 0.01 0.99	1<br>Olong_002926-R1 0.088 0.519 0.431 0.002 0.996<br>Olong_007449-R1 0.547 0.999 0.452 0.058 0.881	1<br>Opunc03g25490.1 0.014 0.528 0.514 0.486 0.8<br>Opunc03g26870.1 0.548 0.999 0.451 0.053 1	0<br>Obrac03g20710.1 0.085 0.519 0.434 0.028 0.997	1<br>Lperr03g23190.1 0.547 0.999 0.452 0.03 0.867<br>Lperr03g23200.1 0.07 0.519 0.449 0.002 0.997	0<br>AT5G36940.1 0.084 0.52 0.436 0.003 0.998
Osjap03g34330.1	1<br>Osjap03g34330.1 0.001 1 0.999 0.001 1	1<br>Osind03g35550.1 0.001 1 0.999 0.039 1	1<br>Orufi03g33060.1 0.001 1 0.999 0.001 1	1<br>Oglab03g30830.1 0.001 1 0.999 0.001 1	1<br>Obart03g31750.1 0.001 1 0.999 0.046 1	0	0	0	0	0
	1<br>Osjap03g39110.1 0.634 1 0.366 0.036 1<br>Osjap10g05370.1 0.012 0.609 0.597 0.1 0.963	1<br>Osind01g46540.1 0.634 0.999 0.365 0.065 0.994<br>Osind10g03770.1 0.012 0.609 0.597 0.11 0.96	1<br>Orufi10g04850.1 0.031 0.609 0.578 0.093 0.963<br>Orufi11g12520.1 0.635 1 0.365 0.038 0.991	1<br>Oglab03g34460.1 0.609 0.997 0.388 0.016 0.993<br>Oglab10g04340.1 0.012 0.609 0.597 0.1 0.963	1<br>Obart03g36200.1 0.634 1 0.366 0.016 0.997<br>Obart10g04600.1 0.014 0.609 0.595 0.106 0.962	1<br>Olong_014796-R1 0.001 0.516 0.515 0.002 0.95<br>Olong_027495-R1 0.609 0.994 0.385 0.005 0.998	1<br>Opunc01g42030.1 0.634 1 0.366 0.28 0.872<br>Opunc03g28940.2 0.001 0.609 0.608 0.002 0.962	1<br>Obrac02g16280.1 0.628 0.981 0.353 0.063 0.949<br>Obrac03g23440.1 0.001 0.609 0.608 0.002 0.964	1<br>Lperr03g26400.1 0.001 0.609 0.608 0.002 0.962<br>Lperr03g30520.1 0.626 1 0.374 0.003 0.997	0<br>AT1G70730.3 0.001 0.609 0.608 0.118 0.967
Osjap03g44070.1	1<br>Osjap03g44070.1 0.001 1 0.999 0.001 1	0	1<br>Orufi03g42420.1 0.001 1 0.999 0.001 1	0	0	1<br>Olong_021918-R1 0.036 0.967 0.931 0.001 0.957<br>Olong_021918-R2 0.036 0.967 0.931 0.001 0.958	1<br>Opunc03g37840.1 0.001 1 0.999 0.001 0.995	0	0	0
	1<br>Osjap03g44260.1 0.569 1 0.431 0.062 1	1<br>Osind01g39050.1 0.567 1 0.433 0.029 0.996	1<br>Orufi03g42640.1 0.569 1 0.431 0.056 1	1<br>Oglab03g38640.1 0.001 0.54 0.539 0.001 0.991	1<br>Obart03g40680.1 0.001 0.54 0.539 0.001 0.991	1<br>Olong_002818-R1 0.567 0.999 0.432 0.026 0.996	1<br>Opunc06g00320.1 0.569 1 0.431 0.083 0.875	0<br>Obrac03g30660.1 0.001 0.539 0.538 0.001 0.989	1<br>Lperr01g38560.1 0.569 1 0.431 0.099 1	0<br>AT4G13650.1 0.02 0.537 0.517 0.069 0.907

	Osjap05g21490.1 0.016 0.535 0.519 0.057 0.81	Osind03g45110.2 0.001 0.54 0.539 0.002 0.991	Orufi05g20190.1 0.016 0.535 0.519 0.058 0.817	Oglab03g38820.1 0.569 0.97 0.401 0.063 0.949	Obart03g40860.1 0.569 1 0.431 0.056 1	Olong_017812-R1 0.032 0.542 0.51 0.001 0.964	Opunc12g13740.1 0.029 0.549 0.52 0.001 1		Lperr08g09550.1 0.014 0.536 0.522 0.05 0.981	
Osjap03g44320.1	1<br>Osjap03g44320.1 0.004 1 0.996 0.004 1	0	1<br>Orufi03g42680.1 0.004 0.975 0.971 0.004 0.96<br>Orufi03g42680.2 0.004 0.975 0.971 0.004 0.96	0	0	0	0	0	0	0
	1<br>Osjap04g33070.2 0.07 0.676 0.606 0.058 0.563<br>Osjap05g18460.1 0.684 0.947 0.263 0.428 0.554	1<br>Osind04g32300.1 0.078 0.676 0.598 0.063 0.544<br>Osind05g19170.1 0.684 0.947 0.263 0.428 0.554	0<br>Orufi04g30630.1 0.07 0.525 0.455 0.055 0.42<br>Orufi05g17410.1 0.684 0.947 0.263 0.428 0.554	1<br>Oglab04g26980.1 0.078 0.676 0.598 0.063 0.544<br>Oglab06g18840.1 0.697 0.947 0.25 0.499 0.59	1<br>Obart04g28950.3 0.004 0.676 0.672 0.003 0.524<br>Obart06g20790.1 0.697 0.947 0.25 0.422 0.527	0<br>Olong_003439-R1 0.684 0.947 0.263 0.471 0.636<br>Olong_020808-R1 0.168 0.656 0.488 0.008 0.916	1<br>Opunc04g26460.2 0.004 0.676 0.672 0.003 0.58<br>Opunc05g14360.1 0.684 0.947 0.263 0.426 0.552	0<br>Obrac04g20350.1 0.168 0.676 0.508 0.004 0.467<br>Obrac06g08740.1 0.709 0.947 0.238 0.411 0.51	0<br>Lperr04g24470.1 0.135 0.676 0.541 0.142 0.558<br>Lperr06g10840.1 0.709 0.947 0.238 0.404 0.503	1<br>AT4G22260.1 0.152 0.676 0.524 0.154 0.536<br>AT5G55230.2 0.701 0.988 0.287 0.393 0.498
Osjap03g44530.1	1<br>Osjap03g44530.1 0.001 1 0.999 0.001 1	1<br>Osind03g45590.1 0.001 1 0.999 0.002 1	0	0	0	1<br>Olong_013357-R1 0.001 1 0.999 0.002 1	0	1<br>Obrac03g30850.1 0.007 1 0.993 0.023 1	0	0
	0<br>Osjap07g07980.2 0.004 0.595 0.591 0.225 0.975	1<br>Osind07g07170.1 0.001 0.607 0.606 0.002 0.987<br>Osind08g23560.1 0.729 0.988 0.259 0.317 0.968	1<br>Orufi03g42810.1 0.625 1 0.375 0.004 1<br>Orufi07g06860.4 0.001 0.607 0.606 0.002 0.987	1<br>Oglab03g38980.1 0.618 1 0.382 0.004 1<br>Oglab03g38990.1 0.001 0.595 0.594 0.002 0.968	1<br>Obart03g41020.1 0.618 1 0.382 0.004 1<br>Obart03g41030.1 0.001 0.595 0.594 0.002 0.967	0<br>Olong_020188-R1 0.001 0.607 0.606 0.002 0.987	1<br>Opunc03g38110.1 0.629 1 0.371 0.007 1<br>Opunc07g06610.1 0.001 0.611 0.61 0.002 0.993	0<br>Obrac03g30850.2 0.007 0.595 0.588 0.037 0.969	1<br>Lperr03g34950.2 0.625 1 0.375 0.129 0.987<br>Lperr07g06010.1 0.001 0.611 0.61 0.002 0.991	1<br>AT3G01980.3 0.636 1 0.364 0.017 1<br>AT5G46410.2 0.129 0.592 0.463 0.206 0.991
Osjap04g02830.1	1<br>Osjap04g02830.1 0.001 1 0.999 0.001 1	0	1<br>Orufi04g02580.1 0.054 0.859 0.805 0.403 0.971<br>Orufi04g02650.1 0.001 0.988 0.987 0.254 0.883	0	0	0	0	0	0	0
	1<br>Osjap04g02720.1 0.001 0.531 0.53 0.001 1<br>Osjap04g02730.1 0.545 1 0.455 0.049 1	1<br>Osind01g20610.1 0.545 1 0.455 0.052 1<br>Osind04g02110.1 0.07 0.509 0.439 0.629 1	1<br>Orufi04g02720.1 0.563 0.988 0.425 0.135 0.82<br>Orufi10g04890.1 0.001 0.503 0.502 0.001 0.96	0<br>Oglab12g09560.1 0.05 0.507 0.457 0.619 0.987	1<br>Obart04g01960.1 0.632 1 0.368 0.004 1<br>Obart10g04670.1 0.001 0.503 0.502 0.001 0.931	1<br>Olong_015999-R1 0.563 0.988 0.425 0.135 0.82<br>Olong_024256-R1 0.054 0.528 0.474 0.309 0.998	1<br>Opunc04g01960.1 0.545 0.991 0.446 0.13 0.994<br>Opunc04g02050.1 0.006 0.531 0.525 0.004 1	0<br>Obrac04g01260.1 0.054 0.509 0.455 0.502 1	0<br>Lperr04g14110.1 0.001 0.497 0.496 0.001 0.963	0<br>AT5G45480.1 0.095 0.509 0.414 0.701 0.987
Osjap04g11160.1	1<br>Osjap04g11160.1 0.001 1 0.999 0.001 1	1<br>Osind09g02760.1 0.001 1 0.999 0.001 1	1<br>Orufi04g09840.1 0.001 1 0.999 0.001 1	0	0	0	1<br>Opunc04g07220.1 0.017 0.988 0.971 0.005 1	0	1<br>Lperr04g05910.1 0.064 0.993 0.929 0.022 0.997	0
	1<br>Osjap10g21270.2 0.556 0.988 0.432 0.059 0.976<br>Osjap11g14300.1 0.007 0.518 0.511 0.04 0.878	1<br>Osind08g20650.1 0.007 0.518 0.511 0.04 0.878<br>Osind09g05810.1 0.548 0.978 0.43 0.004 0.868	1<br>Orufi10g19500.1 0.556 0.988 0.432 0.017 0.976<br>Orufi11g12980.1 0.007 0.518 0.511 0.039 0.881	1<br>Oglab09g05260.1 0.548 0.978 0.43 0.004 0.868<br>Oglab11g10800.1 0.007 0.518 0.511 0.04 0.878	1<br>Obart04g08610.1 0.538 1 0.462 0.001 1<br>Obart11g12300.1 0.007 0.518 0.511 0.04 0.878	1<br>Olong_024054-R1 0.577 1 0.423 0.002 1<br>Olong_024055-R1 0.009 0.541 0.532 0.001 0.96	1<br>Opunc10g16660.1 0.556 0.988 0.432 0.053 0.976<br>Opunc11g10540.3 0.007 0.518 0.511 0.087 0.889	1<br>Obrac04g04850.1 0.007 0.549 0.542 0.043 0.975<br>Obrac10g11900.3 0.559 0.983 0.424 0.029 0.935	1<br>Lperr10g14190.1 0.556 0.988 0.432 0.114 0.977<br>Lperr11g10010.2 0.007 0.518 0.511 0.037 0.886	1<br>AT4G18750.1 0.564 0.999 0.435 0.069 0.882<br>AT5G44240.1 0.008 0.518 0.51 0.066 0.889
Osjap04g27970.1	1<br>Osjap04g27970.1 0.001 1 0.999 0.001 1	0	1<br>Orufi04g25730.1 0.014 0.941 0.927 0.016 0.995	0	0	0	0	0	1<br>Lperr04g19670.1 0.033 0.941 0.908 0.042 0.995	0
	1<br>Osjap02g33010.1 0.481 0.938 0.457 0.087 0.989<br>Osjap02g40270.1 0.073 0.442 0.369 0.204 0.995	1<br>Osind04g27100.1 0.001 0.442 0.441 0.002 1<br>Osind04g27110.1 0.481 0.939 0.458 0.089 0.987	1<br>Orufi02g30690.1 0.481 0.938 0.457 0.087 0.989<br>Orufi02g37650.1 0.073 0.442 0.369 0.204 0.995	1<br>Oglab04g22120.1 0.014 0.442 0.428 0.032 1<br>Oglab04g22130.1 0.481 0.939 0.458 0.089 0.987	1<br>Obart04g23980.1 0.001 0.442 0.441 0.002 1<br>Obart04g23990.1 0.481 0.939 0.458 0.089 0.987	1<br>Olong_003249-R1 0.481 0.938 0.457 0.087 0.989<br>Olong_008382-R1 0.02 0.414 0.394 0.064 0.969	1<br>Opunc04g21520.1 0.014 0.442 0.428 0.032 1<br>Opunc04g21530.1 0.481 0.939 0.458 0.089 0.987	1<br>Obrac04g16300.1 0.016 0.442 0.426 0.039 1<br>Obrac04g16310.1 0.481 0.939 0.458 0.089 0.987	1<br>Lperr02g23670.1 0.481 0.938 0.457 0.087 0.989<br>Lperr02g29790.1 0.073 0.442 0.369 0.23 0.995	1<br>AT1G80480.1 0.073 0.442 0.369 0.194 1<br>AT5G27080.1 0.474 0.935 0.461 0.017 0.927
Osjap04g30120.1	1<br>Osjap04g30120.1 0.001 1 0.999 0.001 1<br>Osjap04g30120.3 0.001 1 0.999 0.001 1<br>Osjap04g30120.4 0.001 1 0.999 0.001 1	0	1<br>Orufi04g27870.1 0.001 1 0.999 0.001 1<br>Orufi04g27870.2 0.001 1 0.999 0.001 1	0	1<br>Obart04g26710.1 0.001 1 0.999 0.001 1<br>Obart04g26710.2 0.001 1 0.999 0.001 1	1<br>Olong_014711-R2 0.001 0.849 0.848 0.001 0.977	0	1<br>Obrac04g18040.3 0.001 1 0.999 0.001 1	0	0
	1	0	1	1	1	0	1	0	1	1

	Osjap04g16240.1 0.007 0.623 0.616 0.07 0.895<br>Osjap05g24420.1 0.657 0.996 0.339 0.024 0.993	Osind04g29430.1 0.001 0.616 0.615 0.002 0.991	Orufi05g23020.1 0.657 0.996 0.339 0.024 0.993<br>Orufi10g14810.1 0.007 0.632 0.625 0.016 0.827	Oglab06g14840.1 0.01 0.632 0.622 0.059 0.882<br>Oglab11g19190.1 0.648 1 0.352 0.042 1	Obart05g21760.1 0.657 0.996 0.339 0.024 0.993<br>Obart12g15260.1 0.007 0.625 0.618 0.001 0.685	Olong_014711-R1 0.001 0.645 0.644 0.002 0.837	Opunc04g23700.2 0.633 1 0.367 0.003 1<br>Opunc05g09290.1 0.001 0.63 0.629 0.096 0.861	Obrac04g07720.1 0.007 0.633 0.626 0.076 0.934	Lperr07g00250.2 0.665 1 0.335 0.015 0.99<br>Lperr10g10450.1 0.007 0.632 0.625 0.009 0.826	AT3G20730.1 0.006 0.623 0.617 0.023 0.998<br>AT4G11290.1 0.643 1 0.357 0.012 1
Osjap05g00530.1	1<br>Osjap05g00530.1 0.001 1 0.999 0.001 1<br>Osjap05g00530.2 0.001 1 0.999 0.001 1<br>Osjap05g00530.3 0.001 1 0.999 0.001 1<br>Osjap05g00530.4 0.001 1 0.999 0.001 1	1<br>Osind05g00510.1 0.131 1 0.869 0.001 1	1<br>Orufi05g00500.1 0.001 1 0.999 0.001 1	0	1<br>Obart05g00520.1 0.084 0.999 0.915 0.001 0.993<br>Obart05g00520.2 0.084 1 0.916 0.001 1	1<br>Olong_022405-R1 0.131 1 0.869 0.001 1	0	0	0	0
	1<br>Osjap02g42390.1 0.422 0.993 0.571 0.001 0.983<br>Osjap07g27300.1 0.068 0.363 0.295 0.072 0.834	1<br>Osind02g41800.1 0.389 0.995 0.606 0.004 0.994<br>Osind06g29470.1 0.058 0.363 0.305 0.1 0.846	1<br>Orufi02g39480.2 0.389 0.994 0.605 0.004 0.992<br>Orufi07g24760.1 0.068 0.363 0.295 0.072 0.834	1<br>Oglab05g00490.2 0.375 1 0.625 0.099 0.996<br>Oglab05g00500.1 0.001 0.365 0.364 0.002 0.982	1<br>Obart06g01490.1 0.1 0.367 0.267 0.2 0.99<br>Obart09g15270.1 0.407 1 0.593 0.029 1	1<br>Olong_023813-R1 0.401 0.997 0.596 0.001 0.976<br>Olong_026414-R1 0.085 0.363 0.278 0.071 0.826	1<br>Opunc05g00480.1 0.397 0.999 0.602 0.007 0.998<br>Opunc05g00490.1 0.008 0.367 0.359 0.037 0.986	0<br>Obrac05g00480.2 0.062 0.363 0.301 0.093 0.998	1<br>Lperr04g17270.1 0.392 1 0.608 0.002 1<br>Lperr07g20540.1 0.052 0.363 0.311 0.079 0.819	0<br>AT4G37380.1 0.009 0.363 0.354 0.043 0.85
Osjap05g00530.2	1<br>Osjap05g00530.1 0.001 1 0.999 0.001 1<br>Osjap05g00530.2 0.001 1 0.999 0.001 1<br>Osjap05g00530.3 0.001 1 0.999 0.001 1<br>Osjap05g00530.4 0.001 1 0.999 0.001 1	1<br>Osind05g00510.1 0.16 1 0.84 0.001 1	1<br>Orufi05g00500.1 0.001 1 0.999 0.001 1	0	1<br>Obart05g00520.1 0.103 0.999 0.896 0.001 0.993<br>Obart05g00520.2 0.103 1 0.897 0.001 1	1<br>Olong_022405-R1 0.16 1 0.84 0.001 1	0	0	0	0
	1<br>Osjap07g27300.1 0.084 0.445 0.361 0.072 0.834<br>Osjap09g15690.3 0.487 1 0.513 0.003 1	1<br>Osind06g29470.1 0.071 0.446 0.375 0.1 0.846<br>Osind09g15830.1 0.487 1 0.513 0.029 1	1<br>Orufi07g24760.1 0.084 0.445 0.361 0.072 0.834<br>Orufi09g16480.1 0.487 1 0.513 0.029 1	1<br>Oglab05g00500.1 0.001 0.449 0.448 0.002 0.982<br>Oglab09g13820.1 0.486 1 0.514 0.093 1	1<br>Obart06g01490.1 0.123 0.451 0.328 0.2 0.99<br>Obart09g15270.1 0.487 1 0.513 0.029 1	1<br>Olong_020432-R1 0.074 0.445 0.371 0.013 0.62<br>Olong_025239-R1 0.486 1 0.514 0.029 1	1<br>Opunc02g35220.2 0.487 0.993 0.506 0.294 0.984<br>Opunc05g00490.1 0.01 0.45 0.44 0.037 0.986	0<br>Obrac05g00480.2 0.077 0.446 0.369 0.093 0.998	1<br>Lperr05g00500.1 0.486 1 0.514 0.022 0.996<br>Lperr07g20540.1 0.064 0.445 0.381 0.079 0.819	0<br>AT4G37380.1 0.011 0.445 0.434 0.043 0.85
Osjap05g00530.3	1<br>Osjap05g00530.1 0.001 1 0.999 0.001 1<br>Osjap05g00530.2 0.001 1 0.999 0.001 1<br>Osjap05g00530.3 0.001 1 0.999 0.001 1<br>Osjap05g00530.4 0.001 1 0.999 0.001 1	1<br>Osind05g00510.1 0.161 1 0.839 0.001 1	1<br>Orufi05g00500.1 0.001 1 0.999 0.001 1	0	1<br>Obart05g00520.1 0.104 0.999 0.895 0.001 0.993<br>Obart05g00520.2 0.104 1 0.896 0.001 1	1<br>Olong_022405-R1 0.161 1 0.839 0.001 1	0	0	0	0
	1<br>Osjap07g27300.1 0.084 0.46 0.376 0.072 0.873<br>Osjap09g15690.3 0.487 1 0.513 0.003 1	1<br>Osind08g09120.1 0.078 0.456 0.378 0.168 0.613<br>Osind09g15830.1 0.487 1 0.513 0.029 1	1<br>Orufi07g24760.1 0.084 0.46 0.376 0.072 0.873<br>Orufi09g16480.1 0.487 1 0.513 0.029 1	1<br>Oglab05g00500.1 0.001 0.456 0.455 0.002 0.993<br>Oglab09g13820.1 0.487 1 0.513 0.093 1	1<br>Obart06g01490.1 0.123 0.45 0.327 0.2 0.982<br>Obart09g15270.1 0.487 1 0.513 0.029 1	1<br>Olong_020432-R1 0.074 0.481 0.407 0.013 0.666<br>Olong_023813-R1 0.482 0.997 0.515 0.004 0.976	1<br>Opunc02g35220.2 0.485 0.993 0.508 0.294 0.984<br>Opunc05g00490.1 0.01 0.456 0.446 0.037 0.993	0<br>Obrac05g00480.2 0.077 0.448 0.371 0.093 0.998	1<br>Lperr05g00500.1 0.487 1 0.513 0.022 0.996<br>Lperr07g20540.1 0.064 0.46 0.396 0.079 0.857	0<br>AT4G37380.1 0.011 0.447 0.436 0.043 0.85
Osjap05g00530.4	1<br>Osjap05g00530.1 0.001 1 0.999 0.001 1<br>Osjap05g00530.2 0.001 1 0.999 0.001 1<br>Osjap05g00530.3 0.001 1 0.999 0.001 1<br>Osjap05g00530.4 0.001 1 0.999 0.001 1	1<br>Osind05g00510.1 0.142 1 0.858 0.001 1	1<br>Orufi05g00500.1 0.001 1 0.999 0.001 1	0	1<br>Obart05g00520.1 0.091 0.999 0.908 0.001 0.993<br>Obart05g00520.2 0.091 1 0.909 0.001 1	1<br>Olong_022405-R1 0.142 1 0.858 0.001 1	0	0	0	0
	1<br>Osjap07g27300.1 0.074 0.394 0.32 0.072 0.834<br>Osjap09g15690.3 0.507 1 0.493 0.003 1	1<br>Osind06g29470.1 0.063 0.395 0.332 0.1 0.846<br>Osind09g15830.1 0.442 1 0.558 0.029 1	1<br>Orufi07g24760.1 0.074 0.394 0.32 0.072 0.834<br>Orufi09g16480.1 0.442 1 0.558 0.029 1	1<br>Oglab05g00490.2 0.407 1 0.593 0.099 0.996<br>Oglab05g00500.1 0.001 0.397 0.396 0.002 0.982	1<br>Obart02g34000.1 0.092 0.388 0.296 0.188 0.958<br>Obart09g15270.1 0.442 1 0.558 0.029 1	1<br>Olong_018361-R1 0.442 1 0.558 0.002 1<br>Olong_020432-R1 0.066 0.394 0.328 0.013 0.62	1<br>Opunc05g00480.1 0.431 0.999 0.568 0.007 0.998<br>Opunc05g00490.1 0.009 0.398 0.389 0.037 0.986	0<br>Obrac05g00480.2 0.068 0.395 0.327 0.093 0.998	1<br>Lperr04g17270.1 0.439 1 0.561 0.028 1<br>Lperr07g20540.1 0.056 0.394 0.338 0.079 0.819	0<br>AT4G37380.1 0.009 0.394 0.385 0.043 0.85
Osjap05g02530.1	1	1	0	1	1	0	0	0	0	0

	Osjap05g02530.1 0.001 1 0.999 0.001 1	Osind05g02510.1 0.001 0.998 0.997 0.001 0.987		Oglab05g02190.1 0.002 1 0.998 0.079 1	Obart05g02340.1 0.002 1 0.998 0.076 1					
	1<br>Osjap03g00960.1 0.459 0.994 0.535 0.132 0.996<br>Osjap06g18010.1 0.001 0.338 0.337 0.184 1	1<br>Osind03g01020.1 0.459 0.994 0.535 0.136 0.996<br>Osind06g17540.1 0.001 0.324 0.323 0.003 0.865	1<br>Orufi05g02460.2 0.459 1 0.541 0.003 0.982<br>Orufi06g16520.1 0.001 0.324 0.323 0.002 0.884	1<br>Oglab03g00660.1 0.459 0.994 0.535 0.124 0.996<br>Oglab05g07310.1 0.001 0.324 0.323 0.003 0.935	1<br>Obart03g00820.2 0.459 0.994 0.535 0.022 0.995<br>Obart06g15630.1 0.001 0.324 0.323 0.002 0.955	1<br>Olong_016162-R1 0.001 0.324 0.323 0.002 0.954<br>Olong_026687-R1 0.427 1 0.573 0.038 0.983	1<br>Opunc05g02220.2 0.459 0.999 0.54 0.003 0.979<br>Opunc07g20770.2 0.001 0.324 0.323 0.002 0.832	1<br>Obrac05g01840.1 0.458 0.998 0.54 0.168 1<br>Obrac06g15870.1 0.001 0.326 0.325 0.003 0.84	0<br>Lperr05g02100.1 0.457 0.998 0.541 0.015 0.978	0<br>AT5G15380.1 0.449 0.992 0.543 0.066 0.987
Osjap05g04120.1	1<br>Osjap05g04120.1 0.001 1 0.999 0.001 1<br>Osjap05g04120.2 0.001 1 0.999 0.001 1	1<br>Osind05g04070.1 0.008 1 0.992 0.001 1<br>Osind05g04070.2 0.008 1 0.992 0.001 1	1<br>Orufi05g03950.1 0.001 1 0.999 0.001 1<br>Orufi05g03950.2 0.001 1 0.999 0.001 1	1<br>Oglab05g03490.1 0.001 1 0.999 0.001 1<br>Oglab05g03490.2 0.001 1 0.999 0.001 1	1<br>Obart05g03830.1 0.001 1 0.999 0.001 1<br>Obart05g03830.2 0.001 1 0.999 0.001 1	1<br>Olong_016739-R1 0.074 1 0.926 0.001 1	1<br>Opunc05g03540.1 0.001 1 0.999 0.001 1<br>Opunc05g03540.2 0.001 1 0.999 0.001 1	1<br>Obrac05g02930.1 0.074 1 0.926 0.001 1	0	0
	1<br>Osjap01g27410.1 0.09 0.723 0.633 0.066 0.996<br>Osjap03g15140.5 0.743 1 0.257 0.061 1	1<br>Osind01g28630.1 0.09 0.723 0.633 0.066 0.996<br>Osind03g15230.1 0.79 1 0.21 0.03 1	1<br>Orufi01g26130.1 0.09 0.723 0.633 0.066 0.996<br>Orufi03g14720.1 0.79 1 0.21 0.03 1	1<br>Oglab01g19490.1 0.09 0.723 0.633 0.066 0.996<br>Oglab03g14060.1 0.79 1 0.21 0.03 1	1<br>Obart01g23310.1 0.09 0.723 0.633 0.066 0.996<br>Obart03g14050.1 0.79 1 0.21 0.03 1	1<br>Olong_003347-R2 0.09 0.723 0.633 0.047 0.996<br>Olong_009943-R1 0.79 1 0.21 0.03 1	1<br>Opunc01g23460.1 0.09 0.723 0.633 0.064 0.996<br>Opunc03g13590.1 0.79 1 0.21 0.002 0.545	1<br>Obrac01g17590.1 0.09 0.723 0.633 0.067 0.996<br>Obrac03g11330.1 0.79 1 0.21 0.03 1	0<br>Lperr05g03460.1 0.001 0.721 0.72 0.001 0.993	1<br>AT2G47580.1 0.775 1 0.225 0.008 1<br>AT3G16630.1 0.003 0.721 0.718 0.031 0.992
Osjap05g04120.2	1<br>Osjap05g04120.1 0.001 1 0.999 0.001 1<br>Osjap05g04120.2 0.001 1 0.999 0.001 1	1<br>Osind05g04070.1 0.008 1 0.992 0.001 1<br>Osind05g04070.2 0.008 1 0.992 0.001 1	1<br>Orufi05g03950.1 0.001 1 0.999 0.001 1<br>Orufi05g03950.2 0.001 1 0.999 0.001 1	1<br>Oglab05g03490.1 0.001 1 0.999 0.001 1<br>Oglab05g03490.2 0.001 1 0.999 0.001 1	1<br>Obart05g03830.1 0.001 1 0.999 0.001 1<br>Obart05g03830.2 0.001 1 0.999 0.001 1	1<br>Olong_016739-R1 0.073 1 0.927 0.001 1	1<br>Opunc05g03540.1 0.001 1 0.999 0.001 1<br>Opunc05g03540.2 0.001 1 0.999 0.001 1	1<br>Obrac05g02930.1 0.073 1 0.927 0.001 1	0	0
	1<br>Osjap01g27410.1 0.089 0.726 0.637 0.066 0.996<br>Osjap03g15140.5 0.747 1 0.253 0.061 1	1<br>Osind01g28630.1 0.089 0.726 0.637 0.066 0.996<br>Osind03g15230.1 0.792 1 0.208 0.03 1	1<br>Orufi01g26130.1 0.089 0.726 0.637 0.066 0.996<br>Orufi03g14720.1 0.792 1 0.208 0.03 1	1<br>Oglab01g19490.1 0.089 0.726 0.637 0.066 0.996<br>Oglab03g14060.1 0.792 1 0.208 0.03 1	1<br>Obart01g23310.1 0.089 0.726 0.637 0.066 0.996<br>Obart03g14050.1 0.792 1 0.208 0.03 1	1<br>Olong_003347-R2 0.089 0.726 0.637 0.047 0.996<br>Olong_009943-R1 0.792 1 0.208 0.03 1	1<br>Opunc01g23460.1 0.089 0.726 0.637 0.064 0.996<br>Opunc03g13590.1 0.792 1 0.208 0.002 0.545	1<br>Obrac01g17590.1 0.089 0.726 0.637 0.067 0.996<br>Obrac03g11330.1 0.792 1 0.208 0.03 1	0<br>Lperr05g03460.1 0.001 0.725 0.724 0.001 0.993	1<br>AT2G47580.1 0.778 1 0.222 0.008 1<br>AT3G16630.1 0.003 0.725 0.722 0.031 0.992
Osjap05g14570.1	1<br>Osjap05g14570.1 0.002 1 0.998 0.002 1	0	1<br>Orufi05g13730.1 0.002 1 0.998 0.002 1	0	0	0	1<br>Opunc05g10890.1 0.002 0.832 0.83 0.005 0.949	0	0	0
	1<br>Osjap01g31830.1 0.687 0.937 0.25 0.055 0.334<br>Osjap05g14540.1 0.034 0.683 0.649 0.309 1	1<br>Osind02g39040.1 0.616 0.948 0.332 0.127 0.415<br>Osind05g15150.1 0.002 0.615 0.613 0.009 1	1<br>Orufi09g08190.1 0.029 0.616 0.587 0.071 0.938<br>Orufi12g06770.1 0.626 0.954 0.328 0.154 0.383	0<br>Oglab02g28070.1 0.63 0.973 0.343 0.002 0.402<br>Oglab05g11780.1 0.344 0.611 0.267 0.172 1	1<br>Obart02g30540.1 0.63 0.973 0.343 0.002 0.402<br>Obart05g12690.1 0.002 0.594 0.592 0.005 0.973	1<br>Olong_008352-R1 0.034 0.611 0.577 0.004 1<br>Olong_023139-R1 0.626 0.937 0.311 0.01 0.432	1<br>Opunc04g20130.1 0.626 0.945 0.319 0.007 0.261<br>Opunc05g11350.1 0.032 0.615 0.583 0.147 0.998	0	0<br>Lperr05g09990.1 0.233 0.443 0.21 0.072 0.928	0<br>AT2G02650.1 0.282 0.573 0.291 0.562 1
Osjap05g23780.1	1<br>Osjap05g23780.1 0.001 1 0.999 0.001 1	0	1<br>Orufi05g22360.1 0.001 1 0.999 0.001 1	0	0	0	0	0	1<br>Lperr05g16920.1 0.029 0.999 0.97 0.015 0.977<br>Lperr05g16920.2 0.029 0.999 0.97 0.016 0.975	0
	1<br>Osjap01g39920.1 0.388 0.981 0.593 0.001 0.472<br>Osjap06g31930.1 0.017 0.374 0.357 0.147 0.902	0<br>Osind05g24290.1 0.349 1 0.651 0.113 1	1<br>Orufi01g38030.1 0.388 0.981 0.593 0.001 0.472<br>Orufi06g29370.1 0.017 0.374 0.357 0.147 0.902	1<br>Oglab05g19890.1 0.388 1 0.612 0.002 1<br>Oglab05g19900.1 0.001 0.374 0.373 0.003 0.894	1<br>Obart01g34670.1 0.388 0.981 0.593 0.001 0.465<br>Obart06g27420.1 0.017 0.374 0.357 0.141 0.901	1<br>Olong_004247-R1 0.017 0.374 0.357 0.147 0.902<br>Olong_020609-R1 0.388 0.981 0.593 0.001 0.483	1<br>Opunc05g18780.1 0.375 1 0.625 0.004 1<br>Opunc05g18790.1 0.001 0.374 0.373 0.003 0.895	0<br>Obrac01g26700.1 0.571 0.981 0.41 0.109 0.448<br>Obrac05g14330.1 0.084 0.374 0.29 0.041 0.873	1<br>Lperr01g29930.1 0.388 0.981 0.593 0.001 0.501<br>Lperr06g22970.1 0.017 0.374 0.357 0.164 0.892	0<br>AT2G32760.1 0.017 0.374 0.357 0.091 0.889
Osjap05g26770.1	1<br>Osjap05g26770.1 0.003 1 0.997 0.003 1	0	1<br>Orufi05g25440.1 0.003 1 0.997 0.003 1	0	0	0	0	0	0	0
	0<br>Osjap05g26770.3 0.003 0.609 0.606 0.005 1	1<br>Osind01g35040.1 0.003 0.609 0.606 0.005 1<br>Osind05g27210.1 0.63 1 0.37 0.008 1	1<br>Orufi01g24400.1 0.639 1 0.361 0.008 1<br>Orufi01g32230.1 0.003 0.609 0.606 0.005 1	1<br>Oglab01g24720.1 0.657 1 0.343 0.25 1<br>Oglab01g24730.1 0.003 0.609 0.606 0.005 1	1<br>Obart01g29160.1 0.003 0.609 0.606 0.005 1<br>Obart05g23830.1 0.63 1 0.37 0.008 1	0<br>Olong_006107-R1 0.63 1 0.37 0.008 1<br>Olong_006661-R1 0.185 0.609 0.424 0.007 1	1<br>Opunc05g21240.1 0.003 0.609 0.606 0.005 1<br>Opunc05g21250.2 0.624 1 0.376 0.34 1	0<br>Obrac01g16370.1 0.639 1 0.361 0.008 1<br>Obrac05g16520.1 0.185 0.609 0.424 0.007 1	1<br>Lperr01g24980.1 0.003 0.609 0.606 0.005 1<br>Lperr05g19670.1 0.63 1 0.37 0.008 1	1<br>AT4G26210.1 0.639 0.997 0.358 0.008 0.992<br>AT5G11770.1 0.143 0.609 0.466 0.284 1
Osjap05g28230.1	1	1	1	1	1	0	1	0	1	0

	Osjap05g28230.1 0.001 1 0.999 0.001 1	Osind05g28520.1 0.001 1 0.999 0.001 1	Orufi05g26870.1 0.001 1 0.999 0.001 1	Oglab05g23160.1 0.001 1 0.999 0.001 1	Obart05g25070.1 0.001 1 0.999 0.001 1		Opunc05g22640.1 0.001 1 0.999 0.001 1		Lperr01g22990.1 0.001 1 0.999 0.027 1	
	1<br>Osjap01g31140.1 0.006 0.432 0.426 0.093 0.997<br>Osjap01g31150.1 0.487 0.999 0.512 0.009 0.999	1<br>Osind01g32600.1 0.001 0.431 0.43 0.002 0.995<br>Osind01g32610.1 0.487 0.999 0.512 0.009 0.999	1<br>Orufi01g29880.1 0.001 0.432 0.431 0.075 0.997<br>Orufi01g29890.1 0.487 0.999 0.512 0.009 0.999	1<br>Oglab01g22740.1 0.027 0.432 0.405 0.061 0.996<br>Oglab01g22750.1 0.487 0.999 0.512 0.009 0.999	1<br>Obart01g26870.1 0.005 0.432 0.427 0.117 0.997<br>Obart01g26880.1 0.487 0.999 0.512 0.009 0.999	1<br>Olong_000367-R1 0.1 0.432 0.332 0.002 0.995<br>Olong_000368-R1 0.462 0.999 0.537 0.069 0.999	1<br>Opunc01g26820.1 0.047 0.432 0.385 0.002 0.996<br>Opunc01g26830.1 0.487 1 0.513 0.009 1	1<br>Obrac01g20400.1 0.044 0.432 0.388 0.004 0.996<br>Obrac05g17560.2 0.432 1 0.568 0.109 1	1<br>Lperr05g20850.2 0.491 1 0.509 0.002 1<br>Lperr05g20860.1 0.001 0.432 0.431 0.002 0.996	1<br>AT5G08690.1 0.011 0.431 0.42 0.034 0.995<br>AT5G51600.1 0.487 0.999 0.512 0.008 0.996
Osjap06g00940.1	1<br>Osjap06g00940.1 0.002 1 0.998 0.002 1	0	1<br>Orufi06g00860.1 0.118 1 0.882 0.021 1	1<br>Oglab06g00840.1 0.085 1 0.915 0.04 1	1<br>Obart06g00880.1 0.085 1 0.915 0.04 1	0	0	0	0	0
	1<br>Osjap06g00950.1 0.415 0.99 0.575 0.132 0.99<br>Osjap08g13790.1 0.083 0.327 0.244 0.005 1	1<br>Osind06g00910.1 0.411 1 0.589 0.068 1<br>Osind07g02910.1 0.091 0.325 0.234 0.012 1	1<br>Orufi06g00690.1 0.415 1 0.585 0.099 1<br>Orufi07g02700.1 0.091 0.325 0.234 0.012 1	1<br>Oglab06g00730.1 0.411 0.987 0.576 0.257 0.968<br>Oglab07g02750.1 0.1 0.325 0.225 0.007 1	1<br>Obart06g00690.1 0.415 1 0.585 0.099 1<br>Obart08g12830.1 0.104 0.324 0.22 0.012 0.988	1<br>Olong_006603-R1 0.04 0.324 0.284 0.034 0.987<br>Olong_022685-R1 0.415 1 0.585 0.074 1	1<br>Opunc06g00800.1 0.325 1 0.675 0.025 1<br>Opunc06g00830.1 0.097 0.325 0.228 0.003 1	1<br>Obrac02g00120.1 0.1 0.325 0.225 0.006 1<br>Obrac06g00660.1 0.411 1 0.589 0.034 0.999	0<br>Lperr01g05300.1 0.108 0.325 0.217 0.013 1<br>Lperr06g00740.1 0.415 0.998 0.583 0.073 0.997	0<br>AT1G20140.1 0.112 0.325 0.213 0.018 1
Osjap06g27840.1	1<br>Osjap06g27840.1 0.003 1 0.997 0.003 1<br>Osjap06g27840.2 0.003 1 0.997 0.004 1	1<br>Osind06g27210.1 0.003 0.809 0.806 0.001 0.998	1<br>Orufi06g25440.1 0.003 0.827 0.824 0.005 0.898	0	0	0	0	0	0	0
	0<br>Osjap04g09410.1 0.003 0.657 0.654 0.009 0.983	1<br>Osind04g06260.1 0.644 0.995 0.351 0.013 0.848<br>Osind06g27200.1 0.003 0.641 0.638 0.009 0.983	1<br>Orufi06g25420.1 0.003 0.641 0.638 0.008 0.975<br>Orufi12g09270.1 0.644 0.912 0.268 0.52 0.977	1<br>Oglab03g01520.1 0.67 0.896 0.226 0.031 0.917<br>Oglab04g06370.1 0.003 0.641 0.638 0.009 0.983	1<br>Obart06g13090.1 0.644 0.912 0.268 0.688 0.987<br>Obart06g23260.1 0.003 0.641 0.638 0.009 0.983	1<br>Olong_010940-R1 0.003 0.641 0.638 0.009 0.983<br>Olong_024815-R2 0.652 0.915 0.263 0.197 0.836	0<br>Opunc03g00390.1 0.016 0.641 0.625 0.319 0.998	0<br>Obrac06g16340.1 0.013 0.641 0.628 0.034 1	0<br>Lperr06g19710.1 0.051 0.641 0.59 0.082 1	0
Osjap07g02600.5	1<br>Osjap07g02600.3 0.002 0.978 0.976 0.478 0.853<br>Osjap07g02600.4 0.002 1 0.998 0.555 1<br>Osjap07g02600.5 0.002 1 0.998 0.002 1	1<br>Osind07g02300.3 0.002 1 0.998 0.002 1<br>Osind07g02300.4 0.002 1 0.998 0.002 1	0	0	0	0	0	0	0	0
	1<br>Osjap01g07190.1 0.002 0.254 0.252 0.692 0.935<br>Osjap07g02840.1 0.298 0.976 0.678 0.411 0.823	1<br>Osind01g07120.1 0.002 0.254 0.252 0.692 0.935<br>Osind07g02530.1 0.298 0.976 0.678 0.411 0.823	1<br>Orufi07g02320.1 0.298 0.976 0.678 0.411 0.823<br>Orufi12g02700.1 0.007 0.259 0.252 0.518 0.746	1<br>Oglab01g05790.1 0.002 0.254 0.252 0.692 0.935<br>Oglab07g02390.2 0.298 0.976 0.678 0.41 0.823	1<br>Obart01g06210.1 0.002 0.254 0.252 0.692 0.935<br>Obart07g02450.1 0.298 0.976 0.678 0.377 0.809	1<br>Olong_003501-R1 0.002 0.254 0.252 0.634 0.923<br>Olong_009970-R1 0.298 0.976 0.678 0.411 0.823	1<br>Opunc01g05980.1 0.002 0.254 0.252 0.689 0.934<br>Opunc07g02500.8 0.298 0.976 0.678 0.472 0.79	1<br>Obrac05g04770.1 0.002 0.254 0.252 0.594 0.923<br>Obrac07g01520.1 0.298 0.99 0.692 0.386 0.893	1<br>Lperr07g02310.1 0.298 0.976 0.678 0.431 0.845<br>Lperr11g02330.1 0.007 0.257 0.25 0.682 0.933	0<br>AT4G36380.1 0.002 0.308 0.306 0.691 0.966
Osjap07g22430.1	1<br>Osjap07g22430.1 0.003 1 0.997 0.003 1	0	1<br>Orufi07g20080.1 0.003 1 0.997 0.099 1	0	1<br>Obart07g19310.1 0.003 1 0.997 0.094 1	0	0	0	0	0
	0<br>Osjap07g22430.2 0.393 1 0.607 0.133 1	1<br>Osind01g03960.1 0.136 0.39 0.254 0.756 0.882<br>Osind07g19410.1 0.393 1 0.607 0.243 1	1<br>Orufi01g30120.1 0.111 0.375 0.264 0.641 0.935<br>Orufi07g20080.2 0.393 1 0.607 0.26 1	1<br>Oglab04g15310.1 0.399 0.991 0.592 0.272 0.988<br>Oglab12g04490.5 0.09 0.387 0.297 0.524 0.987	1<br>Obart04g26170.2 0.111 0.384 0.273 0.201 0.246<br>Obart07g19310.2 0.393 1 0.607 0.243 1	0<br>Olong_001427-R1 0.467 1 0.533 0.006 1<br>Olong_024445-R1 0.115 0.353 0.238 0.413 0.957	1<br>Opunc03g01390.1 0.115 0.372 0.257 0.685 0.907<br>Opunc07g18240.1 0.393 1 0.607 0.319 1	1<br>Obrac03g26550.1 0.115 0.337 0.222 0.068 0.886<br>Obrac07g12120.1 0.393 1 0.607 0.216 1	1<br>Lperr07g01300.1 0.133 0.35 0.217 0.488 0.527<br>Lperr07g16720.1 0.393 1 0.607 0.129 1	0<br>AT3G63190.1 0.378 1 0.622 0.269 1
Osjap07g29350.1	1<br>Osjap07g29350.1 0.002 1 0.998 0.002 1	1<br>Osind07g26050.1 0.013 1 0.987 0.157 1	1<br>Orufi07g26420.1 0.106 1 0.894 0.002 1	0	0	1<br>Olong_024157-R2 0.011 0.989 0.978 0.044 0.998	0	0	0	0
	1<br>Osjap03g18810.1 0.719 1 0.281 0.007 1<br>Osjap07g29380.2 0.013 0.629 0.616 0.158 0.994	1<br>Osind01g39050.1 0.004 0.637 0.633 0.07 0.971<br>Osind08g27680.1 0.723 0.996 0.273 0.289 0.986	1<br>Orufi03g18050.1 0.717 1 0.283 0.437 1<br>Orufi07g26440.5 0.013 0.629 0.616 0.164 0.994	1<br>Oglab06g19010.1 0.119 0.605 0.486 0.058 0.967<br>Oglab07g22020.1 0.635 1 0.365 0.021 1	1<br>Obart06g20980.1 0.028 0.605 0.577 0.043 0.968<br>Obart07g25530.1 0.635 1 0.365 0.021 1	1<br>Olong_015865-R1 0.717 1 0.283 0.364 1<br>Olong_024157-R3 0.011 0.715 0.704 0.056 0.975	1<br>Opunc07g23940.1 0.719 1 0.281 0.007 1<br>Opunc07g23960.1 0.011 0.626 0.615 0.053 0.995	1<br>Obrac06g14370.1 0.026 0.635 0.609 0.05 0.99<br>Obrac07g16760.2 0.708 1 0.292 0.101 1	0<br>Lperr07g22320.1 0.719 1 0.281 0.007 1<br>Lperr11g01590.1 0.149 0.57 0.421 0.083 0.878	0<br>AT5G18100.1 0.711 0.998 0.287 0.018 0.957
Osjap08g06650.1	1	1	1	1	1	0	1	0	0	0

	Osjap08g06650.1 0.002 1 0.998 0.002 1	Osind08g06790.1 0.002 1 0.998 0.002 1	Orufi08g06840.1 0.002 1 0.998 0.002 1	Oglab08g05640.1 0.002 1 0.998 0.002 1	Obart08g06070.1 0.002 1 0.998 0.002 1		Opunc08g05850.1 0.047 1 0.953 0.068 1			
	1<br>Osjap04g21220.1 0.047 0.275 0.228 0.21 0.936<br>Osjap09g20870.1 0.359 0.984 0.625 0.208 0.966	1<br>Osind04g20250.1 0.047 0.275 0.228 0.198 0.94<br>Osind09g20960.1 0.359 0.984 0.625 0.208 0.966	1<br>Orufi04g19250.2 0.047 0.275 0.228 0.21 0.936<br>Orufi09g21960.1 0.359 0.984 0.625 0.2 0.965	1<br>Oglab02g26360.1 0.348 0.996 0.648 0.01 0.994<br>Oglab04g16180.1 0.047 0.275 0.228 0.198 0.94	1<br>Obart02g28440.1 0.348 0.996 0.648 0.093 0.994<br>Obart04g17770.1 0.047 0.275 0.228 0.198 0.94	1<br>Olong_008030-R1 0.047 0.275 0.228 0.232 0.937<br>Olong_008031-R2 0.277 1 0.723 0.106 1	1<br>Opunc04g15550.1 0.047 0.275 0.228 0.206 0.938<br>Opunc04g18740.1 0.295 0.984 0.689 0.088 0.994	1<br>Obrac08g04780.1 0.277 1 0.723 0.098 1<br>Obrac08g04800.1 0.002 0.275 0.273 0.006 0.943	1<br>Lperr08g05690.1 0.277 1 0.723 0.1 1<br>Lperr08g05700.1 0.002 0.275 0.273 0.006 0.943	1<br>AT1G05310.1 0.33 0.982 0.652 0.183 0.99<br>AT4G30800.1 0.047 0.275 0.228 0.208 0.943
Osjap08g10540.1	1<br>Osjap08g10540.1 0.001 1 0.999 0.001 1	1<br>Osind08g11830.1 0.001 1 0.999 0.006 1	1<br>Orufi08g11060.1 0.001 1 0.999 0.001 1	0	0	0	0	0	0	0
	1<br>Osjap08g10560.1 0.001 0.248 0.247 0.02 0.683<br>Osjap10g16570.1 0.315 0.993 0.678 0.229 0.992	1<br>Osind05g10830.1 0.309 0.995 0.686 0.057 0.966<br>Osind07g12020.1 0.001 0.247 0.246 0.003 0.758	1<br>Orufi05g09510.1 0.309 0.995 0.686 0.066 0.964<br>Orufi08g11080.2 0.001 0.248 0.247 0.021 0.71	1<br>Oglab02g32050.1 0.248 0.967 0.719 0.001 0.99<br>Oglab08g09010.1 0.001 0.248 0.247 0.028 0.635	1<br>Obart02g04890.1 0.266 0.986 0.72 0.148 0.991<br>Obart07g11970.1 0.001 0.248 0.247 0.003 0.764	1<br>Olong_015028-R1 0.001 0.248 0.247 0.032 0.684<br>Olong_022719-R1 0.315 0.993 0.678 0.238 0.966	1<br>Opunc02g16130.1 0.315 0.995 0.68 0.224 1<br>Opunc08g08990.2 0.001 0.248 0.247 0.018 0.873	0<br>Obrac04g05110.2 0.003 0.248 0.245 0.025 0.656	0<br>Lperr07g03020.1 0.688 0.978 0.29 0.003 0.899<br>Lperr10g01740.2 0.01 0.273 0.263 0.042 0.596	0<br>AT4G15880.1 0.734 0.969 0.235 0.495 0.99
Osjap08g13760.3	1<br>Osjap08g13760.3 0.003 1 0.997 0.003 1	0	1<br>Orufi08g14470.3 0.023 1 0.977 0.081 1	0	0	0	0	0	0	0
	0<br>Osjap03g41020.1 0.431 1 0.569 0.179 1	1<br>Osind08g15490.1 0.003 0.247 0.244 0.002 0.172<br>Osind08g15500.1 0.314 1 0.686 0.005 1	1<br>Orufi03g39540.2 0.431 1 0.569 0.179 1<br>Orufi08g14470.1 0.003 0.247 0.244 0.031 0.195	1<br>Oglab08g11850.1 0.003 0.247 0.244 0.002 0.172<br>Oglab08g11860.1 0.314 1 0.686 0.005 1	1<br>Obart08g12780.1 0.003 0.247 0.244 0.002 0.172<br>Obart08g12790.1 0.314 1 0.686 0.005 1	0	1<br>Opunc03g34960.1 0.418 1 0.582 0.171 1<br>Opunc05g25720.1 0.037 0.311 0.274 0.016 0.217	1<br>Obrac03g28290.1 0.418 1 0.582 0.15 1<br>Obrac09g02930.1 0.023 0.247 0.224 0.011 0.186	1<br>Lperr03g32080.1 0.431 1 0.569 0.205 1<br>Lperr08g10650.1 0.003 0.247 0.244 0.002 0.18	1<br>AT1G20890.1 0.361 0.95 0.589 0.036 0.97<br>AT1G64440.1 0.037 0.271 0.234 0.006 0.195
Osjap08g22730.1	1<br>Osjap08g22730.1 0.001 1 0.999 0.001 1	1<br>Osind08g24940.1 0.13 0.987 0.857 0.09 0.999	1<br>Orufi08g23770.1 0.02 1 0.98 0.019 1<br>Orufi08g23770.3 0.002 1 0.998 0.018 1	0	0	0	0	0	0	0
	1<br>Osjap04g34840.1 0.02 0.374 0.354 0.031 0.992<br>Osjap08g22710.1 0.43 1 0.57 0.026 1	1<br>Osind04g34100.1 0.001 0.374 0.373 0.002 0.992<br>Osind08g24940.2 0.437 1 0.563 0.006 1	1<br>Orufi04g32420.1 0.02 0.374 0.354 0.031 0.992<br>Orufi08g23750.1 0.414 1 0.586 0.002 1	0<br>Oglab04g28790.1 0.001 0.374 0.373 0.002 0.992	1<br>Obart04g30710.1 0.001 0.374 0.373 0.002 0.992<br>Obart08g21280.1 0.411 1 0.589 0.002 1	1<br>Olong_012750-R1 0.002 0.367 0.365 0.085 0.881<br>Olong_024729-R1 0.465 0.999 0.534 0.017 1	1<br>Opunc04g28230.1 0.004 0.369 0.365 0.008 0.979<br>Opunc08g19370.1 0.39 0.989 0.599 0.003 0.997	1<br>Obrac04g21850.1 0.001 0.364 0.363 0.008 0.992<br>Obrac08g14190.1 0.405 1 0.595 0.002 1	1<br>Lperr04g26140.1 0.015 0.365 0.35 0.032 0.96<br>Lperr08g18050.1 0.41 1 0.59 0.003 1	0<br>AT2G02020.1 0.001 0.36 0.359 0.007 0.994
Osjap09g03990.5	1<br>Osjap09g03990.1 0.004 1 0.996 0.543 1<br>Osjap09g03990.2 0.004 1 0.996 0.508 1<br>Osjap09g03990.5 0.004 1 0.996 0.004 1	0	1<br>Orufi09g04390.1 0.004 1 0.996 0.6 1<br>Orufi09g04390.2 0.004 1 0.996 0.559 1	0	0	0	0	0	0	0
	1<br>Osjap05g14220.1 0.507 1 0.493 0.468 0.818<br>Osjap09g14680.2 0.004 0.414 0.41 0.77 0.967	1<br>Osind09g04090.1 0.674 1 0.326 0.03 0.582<br>Osind09g04110.1 0.004 0.581 0.577 0.704 1	1<br>Orufi09g08210.2 0.507 1 0.493 0.388 0.791<br>Orufi09g15320.1 0.004 0.414 0.41 0.75 0.965	1<br>Oglab08g18120.2 0.731 1 0.269 0.391 0.77<br>Oglab09g03780.1 0.004 0.581 0.577 0.714 1	1<br>Obart07g20140.1 0.709 1 0.291 0.385 0.717<br>Obart09g03930.1 0.004 0.581 0.577 0.714 1	1<br>Olong_001796-R2 0.004 0.626 0.622 0.703 0.995<br>Olong_005713-R1 0.705 1 0.295 0.522 0.912	1<br>Opunc07g19040.1 0.709 1 0.291 0.384 0.717<br>Opunc09g03510.2 0.004 0.581 0.577 0.685 1	1<br>Obrac07g12720.1 0.709 1 0.291 0.276 0.667<br>Obrac09g02030.2 0.004 0.581 0.577 0.676 1	1<br>Lperr07g17470.1 0.709 1 0.291 0.382 0.716<br>Lperr09g02810.2 0.004 0.581 0.577 0.737 1	1<br>AT1G79610.1 0.004 0.626 0.622 0.735 0.974<br>AT5G07100.1 0.639 1 0.361 0.285 0.864
Osjap09g15430.2	1<br>Osjap09g15430.2 0.002 1 0.998 0.002 1<br>Osjap09g15430.3 0.002 1 0.998 0.002 1	1<br>Osind09g15570.1 0.002 1 0.998 0.002 1<br>Osind09g15570.2 0.002 1 0.998 0.002 1	1<br>Orufi09g16160.1 0.002 1 0.998 0.002 1<br>Orufi09g16160.2 0.002 1 0.998 0.002 1	1<br>Oglab09g13510.1 0.002 1 0.998 0.002 1<br>Oglab09g13510.2 0.002 1 0.998 0.002 1<br>Oglab09g13510.3 0.002 1 0.998 0.002 1<br>Oglab09g13510.4 0.002 1 0.998 0.002 1	1<br>Obart09g14980.1 0.002 1 0.998 0.002 1<br>Obart09g14980.2 0.002 1 0.998 0.002 1	0	1<br>Opunc09g13420.1 0.002 1 0.998 0.002 1<br>Opunc09g13420.2 0.002 1 0.998 0.002 1	1<br>Obrac09g09090.1 0.002 1 0.998 0.002 1	0	0
	0<br>Osjap02g06650.1 0.01 0.565 0.555 0.01 0.997	1<br>Osind02g06940.1 0.01 0.565 0.555 0.01 0.997<br>Osind08g23920.1 0.569 0.993 0.424 0.131 0.988	1<br>Orufi04g24220.1 0.014 0.567 0.553 0.165 1<br>Orufi08g22880.1 0.569 0.993 0.424 0.131 0.988	1<br>Oglab08g19240.1 0.569 0.993 0.424 0.143 0.986<br>Oglab12g18680.1 0.002 0.567 0.565 0.003 1	1<br>Obart02g06180.1 0.01 0.565 0.555 0.01 0.997<br>Obart08g20390.1 0.569 0.993 0.424 0.631 0.988	1<br>Olong_008093-R2 0.596 0.991 0.395 0.25 0.996<br>Olong_024186-R1 0.002 0.567 0.565 0.003 1	1<br>Opunc04g20190.1 0.007 0.567 0.56 0.006 1<br>Opunc08g18560.1 0.569 0.993 0.424 0.128 0.969	1<br>Obrac01g09090.1 0.605 0.998 0.393 0.299 0.982<br>Obrac02g04550.3 0.009 0.565 0.556 0.214 0.997	1<br>Lperr04g18350.1 0.002 0.567 0.565 0.021 1<br>Lperr09g11120.1 0.569 1 0.431 0.156 1	1<br>AT1G68540.1 0.567 0.991 0.424 0.112 1<br>AT2G21270.3 0.003 0.565 0.562 0.059 0.997

Osjap09g15430.3	1<br>Osjap09g15430.2 0.002 1 0.998 0.002 1<br>Osjap09g15430.3 0.002 1 0.998 0.002 1	1<br>Osind09g15570.1 0.002 1 0.998 0.002 1<br>Osind09g15570.2 0.002 1 0.998 0.002 1	1<br>Orufi09g16160.1 0.002 1 0.998 0.002 1<br>Orufi09g16160.2 0.002 1 0.998 0.002 1	1<br>Oglab09g13510.1 0.002 1 0.998 0.002 1<br>Oglab09g13510.2 0.002 1 0.998 0.002 1<br>Oglab09g13510.3 0.002 1 0.998 0.002 1<br>Oglab09g13510.4 0.002 1 0.998 0.002 1	1<br>Obart09g14980.1 0.002 1 0.998 0.002 1<br>Obart09g14980.2 0.002 1 0.998 0.002 1	0	1<br>Opunc09g13420.1 0.002 1 0.998 0.002 1<br>Opunc09g13420.2 0.002 1 0.998 0.002 1	1<br>Obrac09g09090.1 0.002 1 0.998 0.002 1	0	0
	0<br>Osjap02g06650.1 0.01 0.54 0.53 0.01 0.997	1<br>Osind02g06940.1 0.01 0.54 0.53 0.01 0.997<br>Osind08g23920.1 0.544 0.993 0.449 0.131 0.988	1<br>Orufi04g24220.1 0.013 0.542 0.529 0.165 1<br>Orufi08g22880.1 0.544 0.993 0.449 0.131 0.988	1<br>Oglab08g19240.1 0.544 0.993 0.449 0.143 0.986<br>Oglab12g18680.1 0.002 0.542 0.54 0.003 1	1<br>Obart02g06180.1 0.01 0.54 0.53 0.01 0.997<br>Obart08g20390.1 0.544 0.993 0.449 0.631 0.988	1<br>Olong_001228-R1 0.548 0.992 0.444 0.03 0.947<br>Olong_024186-R1 0.002 0.542 0.54 0.003 1	1<br>Opunc04g20190.1 0.007 0.542 0.535 0.006 1<br>Opunc08g18560.1 0.544 0.993 0.449 0.128 0.969	1<br>Obrac02g04550.3 0.008 0.54 0.532 0.214 0.997<br>Obrac09g00560.1 0.548 0.998 0.45 0.164 0.966	1<br>Lperr04g18350.1 0.002 0.542 0.54 0.021 1<br>Lperr09g11120.1 0.544 1 0.456 0.156 1	1<br>AT1G68540.1 0.542 0.992 0.45 0.112 1<br>AT2G21270.3 0.003 0.54 0.537 0.059 0.997
Osjap09g17720.1	1<br>Osjap09g17720.1 0.001 1 0.999 0.001 1	1<br>Osind09g17930.1 0.001 1 0.999 0.001 0.995	0	0	0	0	0	0	0	0
	0<br>Osjap10g13350.2 0.212 0.99 0.778 0.015 0.984	1<br>Osind06g11050.1 0.002 0.207 0.205 0.268 0.883<br>Osind06g25720.1 0.208 0.992 0.784 0.029 0.989	1<br>Orufi09g18680.1 0.004 0.209 0.205 0.291 0.895<br>Orufi10g11850.1 0.212 0.99 0.778 0.016 0.984	1<br>Oglab06g09730.1 0.002 0.207 0.205 0.268 0.883<br>Oglab06g20260.1 0.207 0.992 0.785 0.014 0.981	1<br>Obart02g06450.1 0.218 0.992 0.774 0.019 0.994<br>Obart09g17370.1 0.001 0.209 0.208 0.003 0.852	1<br>Olong_005351-R1 0.001 0.209 0.208 0.003 0.853<br>Olong_007875-R1 0.218 0.99 0.772 0.014 0.967	1<br>Opunc08g00450.2 0.216 0.99 0.774 0.048 0.856<br>Opunc09g15680.1 0.004 0.209 0.205 0.301 0.895	0<br>Obrac01g21100.1 0.194 0.984 0.79 0.011 0.984	1<br>Lperr02g25740.1 0.002 0.207 0.205 0.277 0.888<br>Lperr06g18550.1 0.213 0.99 0.777 0.025 0.975	1<br>AT3G48280.1 0.218 0.99 0.772 0.002 0.998<br>AT4G39010.1 0.002 0.207 0.205 0.282 0.899
Osjap09g20460.1	1<br>Osjap09g20460.1 0.001 1 0.999 0.001 1	1<br>Osind09g20560.1 0.001 0.986 0.985 0.001 0.998	1<br>Orufi09g21510.1 0.001 0.967 0.966 0.001 0.999	1<br>Oglab09g18200.1 0.001 0.999 0.998 0.001 0.999	1<br>Obart09g19980.1 0.062 0.999 0.937 0.165 0.999<br>Obart09g19980.2 0.001 0.999 0.998 0.001 0.999	1<br>Olong_014589-R1 0.108 0.974 0.866 0.018 0.991<br>Olong_014589-R2 0.108 0.974 0.866 0.018 0.991	0	0	0	0
	1<br>Osjap02g21530.2 0.022 0.584 0.562 0.096 0.943<br>Osjap09g20430.1 0.624 1 0.376 0.085 1	1<br>Osind02g21430.2 0.022 0.584 0.562 0.096 0.943<br>Osind09g20550.1 0.624 1 0.376 0.032 1	1<br>Orufi02g19810.2 0.022 0.584 0.562 0.096 0.943<br>Orufi09g21500.1 0.624 1 0.376 0.085 1	1<br>Oglab02g17850.1 0.022 0.584 0.562 0.095 0.914<br>Oglab09g18170.1 0.624 1 0.376 0.079 1	1<br>Obart04g25370.1 0.044 0.592 0.548 0.176 0.967<br>Obart09g19950.1 0.624 1 0.376 0.079 1	1<br>Olong_014116-R1 0.059 0.592 0.533 0.069 0.957<br>Olong_027417-R1 0.626 0.991 0.365 0.135 0.745	1<br>Opunc09g18280.1 0.619 0.998 0.379 0.058 0.951<br>Opunc09g18290.2 0.001 0.592 0.591 0.001 0.97	1<br>Obrac09g13140.3 0.624 0.986 0.362 0.049 0.806<br>Obrac09g13160.3 0.001 0.593 0.592 0.001 0.974	1<br>Lperr09g15890.1 0.623 0.996 0.373 0.048 0.957<br>Lperr09g15920.1 0.001 0.592 0.591 0.001 0.957	0<br>AT1G18160.1 0.02 0.592 0.572 0.064 0.958
Osjap10g04950.1	1<br>Osjap10g04950.1 0.01 1 0.99 0.01 1<br>Osjap10g21210.1 0.01 1 0.99 0.477 1	1<br>Osind10g03420.1 0.01 1 0.99 0.558 1<br>Osind10g17700.1 0.01 1 0.99 0.477 1	1<br>Orufi10g04520.1 0.01 1 0.99 0.01 1<br>Orufi10g19420.1 0.01 1 0.99 0.477 1	1<br>Oglab10g03910.1 0.01 1 0.99 0.01 1	1<br>Obart10g18220.1 0.01 1 0.99 0.392 0.823	1<br>Olong_009744-R1 0.01 1 0.99 0.651 1<br>Olong_009744-R2 0.01 1 0.99 0.641 1	0	0	0	0
	0<br>Osjap07g13660.9 0.361 1 0.639 0.733 0.947	1<br>Osind02g38620.1 0.361 1 0.639 0.835 0.923<br>Osind06g00010.1 0.01 0.361 0.351 0.609 0.978	1<br>Orufi02g36440.1 0.361 1 0.639 0.835 0.923<br>Orufi08g26670.5 0.01 0.351 0.341 0.583 0.749	1<br>Oglab02g32260.1 0.361 1 0.639 0.838 0.924<br>Oglab04g27900.1 0.01 0.33 0.32 0.14 0.208	1<br>Obart02g34980.1 0.361 1 0.639 0.838 0.924<br>Obart04g29850.1 0.01 0.33 0.32 0.131 0.196	1<br>Olong_008835-R2 0.361 1 0.639 0.809 0.91<br>Olong_022843-R2 0.01 0.33 0.32 0.14 0.208	1<br>Opunc02g32260.1 0.361 1 0.639 0.84 0.925<br>Opunc04g27380.2 0.01 0.33 0.32 0.14 0.208	1<br>Obrac02g25650.1 0.381 1 0.619 0.839 0.923<br>Obrac04g21100.1 0.01 0.33 0.32 0.145 0.217	1<br>Lperr02g28770.1 0.381 1 0.619 0.834 0.92<br>Lperr04g25370.2 0.01 0.33 0.32 0.151 0.225	0
Osjap10g14600.1	1<br>Osjap10g14600.1 0.002 1 0.998 0.002 1<br>Osjap10g14600.2 0.002 1 0.998 0.002 1<br>Osjap10g14600.3 0.002 1 0.998 0.002 1<br>Osjap10g14600.4 0.002 1 0.998 0.002 1<br>Osjap10g14600.5 0.002 1 0.998 0.002 1<br>Osjap10g14600.6 0.002 1 0.998 0.002 1	1<br>Osind10g11660.1 0.002 0.995 0.993 0.003 0.958	1<br>Orufi08g13110.1 0.002 0.987 0.985 0.003 0.99	1<br>Oglab10g11970.1 0.002 0.982 0.98 0.007 1	1<br>Obart10g12400.1 0.002 0.98 0.978 0.004 0.996	0	1<br>Opunc10g10650.1 0.002 0.995 0.993 0.003 0.974<br>Opunc10g10650.2 0.002 0.995 0.993 0.003 0.974	0	0	0
	0<br>Osjap08g12520.1 0.277 0.98 0.703 0.226 0.86	1<br>Osind08g14090.1 0.664 0.987 0.323 0.346 0.989<br>Osind10g11740.1 0.16 0.638 0.478 0.002 0.08	1<br>Orufi08g13110.2 0.664 0.987 0.323 0.346 0.989<br>Orufi10g13050.2 0.002 0.658 0.656 0.003 0.627	1<br>Oglab03g12700.1 0.701 0.975 0.274 0.29 0.977<br>Oglab10g11980.2 0.04 0.649 0.609 0.016 0.42	1<br>Obart03g12670.1 0.701 0.975 0.274 0.29 0.977<br>Obart10g12410.2 0.04 0.653 0.613 0.046 0.639	1<br>Olong_005116-R1 0.658 0.975 0.317 0.171 0.982<br>Olong_028277-R1 0.002 0.651 0.649 0.006 0.975	1<br>Opunc10g10640.1 0.04 0.638 0.598 0.049 0.61<br>Opunc12g14410.1 0.7 0.975 0.275 0.253 0.984	1<br>Obrac10g07370.1 0.117 0.638 0.521 0.003 0.101<br>Obrac12g10200.1 0.66 0.975 0.315 0.237 0.985	1<br>Lperr03g19720.1 0.653 0.982 0.329 0.285 1<br>Lperr10g09020.1 0.117 0.638 0.521 0.001 0.059	1<br>AT3G04260.1 0.117 0.638 0.521 0.075 0.193<br>AT3G55330.1 0.653 0.975 0.322 0.2 0.991

Osjap11g00600.1	1<br>Osjap11g00600.1 0.002 1 0.998 0.002 1<br>Osjap12g00550.1 0.002 0.998 0.996 0.002 1	1<br>Osind12g00790.1 0.002 0.998 0.996 0.002 1	1<br>Orufi11g00440.1 0.002 1 0.998 0.002 1	1<br>Oglab11g00570.1 0.106 1 0.894 0.051 1<br>Oglab12g00270.1 0.002 0.801 0.799 0.003 0.995	1<br>Obart11g00510.1 0.106 1 0.894 0.045 1<br>Obart12g00320.1 0.002 0.998 0.996 0.003 1	0	0	0	0	0
	1<br>Osjap08g03600.1 0.748 0.984 0.236 0.201 0.274<br>Osjap10g06830.2 0.087 0.719 0.632 0.265 0.998	1<br>Osind05g03780.1 0.748 0.984 0.236 0.16 0.63<br>Osind10g04960.1 0.063 0.719 0.656 0.31 0.998	1<br>Orufi08g03710.3 0.748 0.984 0.236 0.289 0.394<br>Orufi12g00580.1 0.002 0.703 0.701 0.002 0.881	0<br>Oglab10g05540.1 0.087 0.719 0.632 0.32 0.998	1<br>Obart08g03340.1 0.748 0.984 0.236 0.191 0.268<br>Obart12g18380.1 0.069 0.717 0.648 0.137 0.977	1<br>Olong_026176-R1 0.083 0.717 0.634 0.114 0.997<br>Olong_027703-R1 0.748 0.984 0.236 0.192 0.274	1<br>Opunc08g03130.1 0.748 0.984 0.236 0.206 0.282<br>Opunc12g00560.1 0.002 0.724 0.722 0.003 0.995	1<br>Obrac08g02840.1 0.748 0.984 0.236 0.201 0.274<br>Obrac12g00030.1 0.004 0.624 0.62 0.412 0.991	1<br>Lperr12g00550.1 0.748 0.986 0.238 0.194 0.271<br>Lperr12g00630.2 0.033 0.719 0.686 0.031 0.983	0<br>AT2G40030.1 0.738 0.982 0.244 0.168 0.234
Osjap11g01220.1	1<br>Osjap11g01220.1 0.001 1 0.999 0.001 1<br>Osjap12g01140.1 0.001 1 0.999 0.001 1	1<br>Osind11g00690.1 0.001 0.955 0.954 0.001 0.999<br>Osind12g01290.1 0.001 1 0.999 0.001 1	1<br>Orufi11g01000.1 0.001 1 0.999 0.001 1<br>Orufi12g01130.1 0.001 1 0.999 0.001 1	1<br>Oglab12g00800.1 0.001 1 0.999 0.001 1	1<br>Obart11g01060.1 0.001 1 0.999 0.001 1	0	1<br>Opunc12g00940.1 0.001 1 0.999 0.001 1	0	0	0
	1<br>Osjap03g13100.1 0.489 0.982 0.493 0.097 0.622<br>Osjap07g30390.1 0.009 0.433 0.424 0.013 0.944	1<br>Osind03g12930.1 0.489 0.982 0.493 0.121 0.633<br>Osind07g27070.1 0.007 0.433 0.426 0.006 0.946	1<br>Orufi03g17220.1 0.496 1 0.504 0.027 1<br>Orufi07g27420.1 0.009 0.433 0.424 0.013 0.946	1<br>Oglab03g12120.1 0.489 0.982 0.493 0.121 0.633<br>Oglab07g22980.1 0.007 0.433 0.426 0.007 0.94	1<br>Obart03g12120.1 0.489 0.982 0.493 0.121 0.633<br>Obart12g00790.1 0.001 0.433 0.432 0.002 0.949	1<br>Olong_007715-R1 0.492 1 0.508 0.007 1<br>Olong_008211-R1 0.007 0.433 0.426 0.006 0.946	1<br>Opunc03g11680.1 0.489 1 0.511 0.148 1<br>Opunc11g00920.1 0.001 0.433 0.432 0.142 0.951	1<br>Obrac03g09720.2 0.489 1 0.511 0.142 1<br>Obrac12g00780.1 0.001 0.433 0.432 0.11 0.953	1<br>Lperr03g10720.1 0.489 0.993 0.504 0.148 0.952<br>Lperr11g00920.1 0.001 0.433 0.432 0.002 0.949	1<br>AT2G34940.1 0.001 0.433 0.432 0.002 0.955<br>AT3G46740.1 0.492 1 0.508 0.161 1
Osjap11g02640.1	1<br>Osjap11g02640.1 0.002 1 0.998 0.002 1<br>Osjap12g02610.1 0.002 1 0.998 0.002 1	1<br>Osind11g01990.1 0.002 1 0.998 0.002 1<br>Osind12g02780.1 0.002 1 0.998 0.002 1	1<br>Orufi11g02320.1 0.002 1 0.998 0.003 1<br>Orufi12g02480.1 0.002 1 0.998 0.002 1	1<br>Oglab11g02200.1 0.002 1 0.998 0.002 1<br>Oglab12g02130.1 0.002 1 0.998 0.002 1	1<br>Obart12g02080.1 0.024 1 0.976 0.002 1	0	0	0	0	0
	1<br>Osjap07g04360.1 0.034 0.683 0.649 0.018 0.926<br>Osjap12g12870.1 0.685 0.948 0.263 0.106 0.417	0<br>Osind07g03860.1 0.034 0.706 0.672 0.032 0.959	1<br>Orufi07g03650.1 0.034 0.683 0.649 0.019 0.978<br>Orufi12g10910.1 0.685 0.948 0.263 0.106 0.417	1<br>Oglab07g03810.1 0.034 0.683 0.649 0.019 0.988<br>Oglab12g09880.1 0.685 0.948 0.263 0.097 0.385	0<br>Obart11g02350.1 0.022 0.681 0.659 0.004 0.993	1<br>Olong_012584-R1 0.067 0.633 0.566 0.002 0.965<br>Olong_021330-R1 0.665 0.935 0.27 0.153 0.616	1<br>Opunc11g02190.1 0.002 0.681 0.679 0.003 0.989<br>Opunc12g08740.1 0.685 0.948 0.263 0.081 0.324	1<br>Obrac05g10860.1 0.052 0.681 0.629 0.097 0.987<br>Obrac12g07170.1 0.685 0.938 0.253 0.142 0.57	1<br>Lperr12g02190.1 0.002 0.681 0.679 0.003 0.989<br>Lperr12g08370.1 0.688 0.948 0.26 0.095 0.391	0<br>AT5G27320.1 0.014 0.655 0.641 0.003 0.98
Osjap11g21440.1	1<br>Osjap11g21440.1 0.001 1 0.999 0.001 1	1<br>Osind04g04060.1 0.001 0.934 0.933 0.072 0.988	1<br>Orufi11g19860.1 0.003 1 0.997 0.052 1	1<br>Oglab11g16480.1 0.003 1 0.997 0.052 1	1<br>Obart11g18650.1 0.001 1 0.999 0.073 1	1<br>Olong_013913-R1 0.006 0.965 0.959 0.001 0.986	0	0	0	0
	1<br>Osjap03g43210.1 0.717 0.987 0.27 0.278 0.99<br>Osjap10g09150.2 0.008 0.716 0.708 0.047 0.738	1<br>Osind03g44240.1 0.717 0.987 0.27 0.017 0.988<br>Osind10g06650.1 0.008 0.716 0.708 0.008 0.713	1<br>Orufi03g41640.1 0.717 0.987 0.27 0.018 0.987<br>Orufi10g08130.1 0.008 0.716 0.708 0.047 0.737	1<br>Oglab03g37880.1 0.717 0.987 0.27 0.278 0.99<br>Oglab11g20100.1 0.02 0.711 0.691 0.029 0.947	1<br>Obart03g02940.1 0.739 0.986 0.247 0.118 0.932<br>Obart10g07740.1 0.008 0.716 0.708 0.047 0.737	1<br>Olong_001283-R1 0.002 0.674 0.672 0.014 0.844<br>Olong_011979-R1 0.739 0.987 0.248 0.108 0.954	1<br>Opunc03g37060.1 0.717 0.987 0.27 0.151 0.989<br>Opunc05g18050.1 0.047 0.702 0.655 0.06 0.817	1<br>Obrac03g30000.2 0.705 0.979 0.274 0.031 0.937<br>Obrac05g13790.3 0.029 0.702 0.673 0.127 0.872	1<br>Lperr03g33980.1 0.717 0.974 0.257 0.008 0.411<br>Lperr11g13500.1 0.043 0.677 0.634 0.047 0.638	0<br>AT3G12620.1 0.719 0.984 0.265 0.047 0.964
Osjap12g01140.1	1<br>Osjap11g01220.1 0.001 1 0.999 0.001 1<br>Osjap12g01140.1 0.001 1 0.999 0.001 1	1<br>Osind11g00690.1 0.001 0.953 0.952 0.001 0.999<br>Osind12g01290.1 0.001 1 0.999 0.001 1	1<br>Orufi11g01000.1 0.001 1 0.999 0.001 1<br>Orufi12g01130.1 0.001 1 0.999 0.001 1	1<br>Oglab12g00800.1 0.001 1 0.999 0.001 1	1<br>Obart11g01060.1 0.001 1 0.999 0.001 1	0	1<br>Opunc12g00940.1 0.001 1 0.999 0.001 1	0	0	0
	1<br>Osjap03g13100.1 0.511 0.981 0.47 0.097 0.622<br>Osjap07g30390.1 0.01 0.373 0.363 0.013 0.798	1<br>Osind03g12930.1 0.511 0.981 0.47 0.121 0.633<br>Osind07g27070.1 0.007 0.377 0.37 0.006 0.783	1<br>Orufi03g17220.1 0.517 1 0.483 0.027 1<br>Orufi07g27420.1 0.01 0.377 0.367 0.013 0.783	1<br>Oglab03g12120.1 0.511 0.981 0.47 0.121 0.633<br>Oglab07g22980.1 0.007 0.373 0.366 0.007 0.783	1<br>Obart03g12120.1 0.511 0.981 0.47 0.121 0.633<br>Obart10g06750.1 0.021 0.409 0.388 0.151 0.885	1<br>Olong_007715-R1 0.514 1 0.486 0.007 1<br>Olong_008211-R1 0.007 0.377 0.37 0.006 0.783	1<br>Opunc03g11680.1 0.511 1 0.489 0.148 1<br>Opunc11g00920.1 0.001 0.377 0.376 0.142 0.816	1<br>Obrac03g09720.2 0.511 1 0.489 0.142 1<br>Obrac10g04150.1 0.02 0.405 0.385 0.036 0.868	1<br>Lperr03g10720.1 0.511 0.993 0.482 0.148 0.952<br>Lperr11g00920.1 0.001 0.377 0.376 0.002 0.792	1<br>AT2G34940.1 0.001 0.377 0.376 0.002 0.793<br>AT3G46740.1 0.514 1 0.486 0.161 1
Osjap12g02200.1	1<br>Osjap11g02160.1 0.024 1 0.976 0.05 1<br>Osjap12g02200.1 0.001 1 0.999 0.001 1	0	1<br>Orufi11g01900.1 0.002 1 0.998 0.032 0.997	1<br>Oglab11g01770.1 0.002 1 0.998 0.068 1	1<br>Obart11g01960.3 0.107 1 0.893 0.023 1	0	0	0	1<br>Lperr12g01900.1 0.114 0.993 0.879 0.041 0.983	0
	0	1	1	1	1	1	0	0	0	0

	Osjap06g22240.1 0.479 1 0.521 0.001 0.976	Osind11g01570.1 0.002 0.331 0.329 0.192 0.912<br>Osind12g02490.1 0.476 1 0.524 0.001 1	Orufi12g02050.1 0.001 0.357 0.356 0.002 1<br>Orufi12g02070.1 0.476 1 0.524 0.002 1	Oglab06g17410.1 0.471 0.999 0.528 0.017 0.999<br>Oglab12g01850.1 0.134 0.461 0.327 0.052 0.99	Obart12g01850.1 0.476 1 0.524 0.001 1<br>Obart12g01860.1 0.118 0.457 0.339 0.077 0.945	Olong_015795-R1 0.002 0.356 0.354 0.218 0.997<br>Olong_026769-R1 0.476 0.997 0.521 0.001 0.986	Opunc12g01810.1 0.22 0.999 0.779 0.023 0.999	Obrac11g01280.1 0.114 0.356 0.242 0.006 0.888<br>Obrac12g01600.1 0.476 0.997 0.521 0.001 0.999	Lperr11g01750.3 0.107 0.331 0.224 0.076 0.849<br>Lperr11g01770.1 0.476 1 0.524 0.001 1	AT5G45790.2 0.113 0.338 0.225 0.112 0.891<br>AT5G60900.1 0.497 0.996 0.499 0.043 0.96
Osjap12g12320.1	1<br>Osjap12g12320.1 0.001 1 0.999 0.001 1	1<br>Osind12g09530.1 0.001 1 0.999 0.001 1	1<br>Orufi12g10460.1 0.001 1 0.999 0.001 1	1<br>Oglab12g09470.1 0.001 1 0.999 0.001 1	1<br>Obart12g09410.1 0.001 1 0.999 0.001 1	0	0	0	0	0
	1<br>Osjap01g44780.2 0.363 0.998 0.635 0.086 0.994<br>Osjap03g07600.1 0.001 0.323 0.322 0.004 0.989	1<br>Osind01g46040.1 0.363 0.998 0.635 0.142 0.995<br>Osind03g07670.1 0.001 0.323 0.322 0.004 0.989	1<br>Orufi01g42650.3 0.363 0.998 0.635 0.186 0.995<br>Orufi03g07180.1 0.001 0.323 0.322 0.004 0.989	1<br>Oglab01g34040.1 0.425 0.998 0.573 0.203 0.995<br>Oglab03g07280.1 0.001 0.323 0.322 0.004 0.989	1<br>Obart01g39260.3 0.388 0.998 0.61 0.039 0.994<br>Obart03g07210.1 0.001 0.323 0.322 0.004 0.989	1<br>Olong_021912-R2 0.373 1 0.627 0.008 1<br>Olong_021913-R1 0.001 0.321 0.32 0.004 0.988	1<br>Opunc02g11250.1 0.001 0.323 0.322 0.372 0.993<br>Opunc12g08570.1 0.39 1 0.61 0.211 1	1<br>Obrac03g05880.1 0.001 0.323 0.322 0.004 0.992<br>Obrac12g06500.1 0.395 1 0.605 0.103 1	1<br>Lperr03g06600.1 0.001 0.323 0.322 0.004 0.992<br>Lperr12g08310.1 0.399 0.999 0.6 0.002 0.998	1<br>AT3G49880.1 0.426 0.995 0.569 0.032 0.987<br>AT4G34670.1 0.001 0.315 0.314 0.004 0.966
Osjap12g13600.1	1<br>Osjap01g05590.3 0.014 0.943 0.929 0.035 0.313<br>Osjap01g31400.1 0.007 0.915 0.908 0.006 0.818<br>Osjap12g13600.1 0.007 1 0.993 0.007 1	1<br>Osind01g32860.1 0.007 0.915 0.908 0.007 0.854<br>Osind12g10660.1 0.007 1 0.993 0.007 1<br>Osind12g10670.1 0.007 0.993 0.986 0.007 0.993	1<br>Orufi01g30130.1 0.007 0.915 0.908 0.007 0.854	1<br>Oglab01g22960.1 0.071 0.915 0.844 0.194 0.874	1<br>Obart01g27080.1 0.071 0.915 0.844 0.194 0.874<br>Obart12g10510.1 0.007 1 0.993 0.012 1	0	1<br>Opunc10g03560.1 0.035 0.844 0.809 0.016 0.248	0	0	0
	1<br>Osjap01g05590.1 0.014 0.326 0.312 0.017 0.556<br>Osjap06g06030.1 0.404 0.915 0.511 0.667 0.815	1<br>Osind01g05750.1 0.028 0.326 0.298 0.019 0.964<br>Osind06g06210.1 0.404 0.915 0.511 0.667 0.815	0<br>Orufi12g11520.1 0.007 0.794 0.787 0.008 0.882	0<br>Oglab10g03770.1 0.035 0.702 0.667 0.013 0.177	1<br>Obart01g16250.1 0.411 0.908 0.497 0.765 0.869<br>Obart03g17930.1 0.007 0.411 0.404 0.002 0.126	1<br>Olong_000447-R1 0.298 0.908 0.61 0.671 0.824<br>Olong_002147-R1 0.035 0.298 0.263 0.428 0.471	1<br>Opunc03g17210.1 0.007 0.326 0.319 0.002 0.093<br>Opunc06g05170.1 0.404 0.915 0.511 0.649 0.802	0<br>Obrac01g31700.1 0.135 0.908 0.773 0.834 0.924	1<br>Lperr01g04610.1 0.014 0.411 0.397 0.02 0.566<br>Lperr03g05500.1 0.411 0.915 0.504 0.585 0.769	1<br>AT2G36780.1 0.035 0.319 0.284 0.028 0.109<br>AT5G50380.1 0.355 0.908 0.553 0.706 0.82
Osjap12g19810.1	1<br>Osjap12g19810.1 0.002 1 0.998 0.002 1	1<br>Osind12g15880.1 0.002 1 0.998 0.002 1	0	0	0	0	0	0	0	0
	1<br>Osjap07g08090.1 0.149 0.629 0.48 0.642 0.97<br>Osjap12g19770.1 0.651 1 0.349 0.006 1	1<br>Osind04g18390.2 0.133 0.627 0.494 0.648 0.967<br>Osind12g15880.2 0.638 1 0.362 0.012 1	1<br>Orufi03g39910.2 0.149 0.629 0.48 0.617 0.969<br>Orufi12g17100.1 0.629 1 0.371 0.035 1	1<br>Oglab06g04730.1 0.149 0.629 0.48 0.655 0.974<br>Oglab12g14370.2 0.642 1 0.358 0.115 1	1<br>Obart03g38270.2 0.149 0.629 0.48 0.61 0.968<br>Obart12g15400.1 0.651 1 0.349 0.006 1	1<br>Olong_001697-R1 0.651 1 0.349 0.006 1<br>Olong_022562-R1 0.149 0.629 0.48 0.615 0.971	1<br>Opunc03g35430.1 0.149 0.629 0.48 0.637 0.97<br>Opunc12g13920.1 0.64 1 0.36 0.003 1	1<br>Obrac06g03630.1 0.129 0.622 0.493 0.588 0.894<br>Obrac12g09830.1 0.649 1 0.351 0.003 1	1<br>Lperr07g06070.1 0.149 0.629 0.48 0.64 0.969<br>Lperr12g12590.1 0.651 1 0.349 0.006 1	0<br>AT4G01970.1 0.149 0.651 0.502 0.688 0.985

(((((_c1,longi),punctata),brachy),leersia),tair);

chimeric gene id	INGROUP					MIDGROUP		OUTGROUP		
	jap	indica	rufi	glab	barthii	longi	punctata	brachy	leersia	tair
Osjap01g08660.1	1<br>Osjap01g08660.1 0.001 1 0.999 0.001 1	1<br>Osind01g08490.1 0.001 1 0.999 0.001 1	1<br>Orufi01g08070.1 0.001 1 0.999 0.001 1	1<br>Oglab01g06470.1 0.001 1 0.999 0.001 1	1<br>Obart01g07430.1 0.017 1 0.983 0.001 1	1<br>Olong_011567-R1 0.068 1 0.932 0.001 1	0	0	0	0
	1<br>Osjap01g29230.2 0.111 0.579 0.468 0.25 0.983<br>Osjap05g00560.2 0.656 0.997 0.341 0.146 1	1<br>Osind01g30690.1 0.111 0.579 0.468 0.267 0.982<br>Osind05g00540.1 0.656 0.997 0.341 0.126 1	0<br>Orufi01g27950.1 0.111 0.579 0.468 0.25 0.983<br>Orufi01g48560.2 0.656 0.98 0.324 0.085 0.984	1<br>Oglab01g21070.2 0.111 0.579 0.468 0.249 0.983<br>Oglab05g00530.1 0.656 0.997 0.341 0.146 1	1<br>Obart01g25090.1 0.111 0.579 0.468 0.219 0.982<br>Obart05g00540.1 0.656 0.997 0.341 0.146 1	0<br>Olong_003039-R1 0.656 0.997 0.341 0.128 1<br>Olong_003692-R2 0.149 0.579 0.43 0.244 0.979	1<br>Opunc01g07160.1 0.589 1 0.411 0.111 1<br>Opunc01g07170.1 0.001 0.58 0.579 0.001 0.984	1<br>Obrac01g05760.3 0.594 1 0.406 0.082 1<br>Obrac01g05770.3 0.001 0.58 0.579 0.001 0.985	1<br>Lperr01g07030.1 0.594 1 0.406 0.018 1<br>Lperr01g07040.2 0.001 0.58 0.579 0.001 0.989	1<br>AT1G54350.1 0.592 0.993 0.401 0.076 0.975<br>AT5G53430.1 0.023 0.579 0.556 0.09 0.984
Osjap02g19520.2	1<br>Osjap02g19520.1 0.002 1 0.998 0.015 1<br>Osjap02g19520.2 0.001 1 0.999 0.001 1	1<br>Osind02g19490.1 0.001 0.998 0.997 0.002 1	1<br>Orufi02g18010.1 0.001 1 0.999 0.015 1	1<br>Oglab02g16690.1 0.001 0.992 0.991 0.002 0.973	1<br>Obart02g17220.1 0.101 0.992 0.891 0.002 0.965	0	1<br>Opunc02g15310.1 0.007 1 0.993 0.041 0.994	0	0	0
	1<br>Osjap02g06360.1 0.009 0.338 0.329 0.036 0.995<br>Osjap03g32140.1 0.341 1 0.659 0.035 0.996	1<br>Osind01g26310.1 0.007 0.339 0.332 0.235 0.996<br>Osind03g33240.1 0.346 1 0.654 0.001 0.984	1<br>Orufi01g24210.2 0.007 0.344 0.337 0.003 0.999<br>Orufi02g17950.1 0.353 0.999 0.646 0.001 0.995	0<br>Oglab02g16620.2 0.334 1 0.666 0.002 1	1<br>Obart01g21490.2 0.013 0.323 0.31 0.053 0.934<br>Obart02g17150.1 0.334 1 0.666 0.002 1	0<br>Olong_017606-R3 0.346 1 0.654 0.001 1	1<br>Opunc01g21720.2 0.007 0.342 0.335 0.002 0.815<br>Opunc03g27000.1 0.342 1 0.658 0.028 0.994	1<br>Obrac01g16240.1 0.026 0.338 0.312 0.02 0.986<br>Obrac03g21720.2 0.346 1 0.654 0.016 0.994	1<br>Lperr01g18440.1 0.007 0.319 0.312 0.038 0.985<br>Lperr02g13790.2 0.335 0.993 0.658 0.008 0.995	1<br>AT1G62740.1 0.348 0.999 0.651 0.004 0.921<br>AT4G13985.1 0.04 0.296 0.256 0.022 0.961

Osjap03g00680.1	1<br>Osjap03g00680.1 0.001 1 0.999 0.001 1<br>Osjap03g00680.2 0.001 1 0.999 0.001 1<br>Osjap03g00680.3 0.001 1 0.999 0.001 1	1<br>Osind03g00740.1 0.001 0.994 0.993 0.001 0.996	1<br>Orufi03g12660.1 0.001 1 0.999 0.001 1<br>Orufi03g12660.2 0.001 1 0.999 0.001 1	1<br>Oglab03g00390.1 0.001 1 0.999 0.001 1	1<br>Obart03g00550.1 0.001 0.994 0.993 0.001 0.996	0	0	0	0	0
	1<br>Osjap02g42090.1 0.051 0.661 0.61 0.065 1<br>Osjap12g08580.1 0.71 0.999 0.289 0.011 0.996	1<br>Osind02g41670.1 0.06 0.661 0.601 0.131 1<br>Osind03g02220.1 0.7 1 0.3 0.015 1	1<br>Orufi02g39350.1 0.06 0.661 0.601 0.131 1<br>Orufi09g02220.2 0.746 1 0.254 0.095 1	1<br>Oglab02g34920.1 0.06 0.661 0.601 0.131 1<br>Oglab03g02050.1 0.688 1 0.312 0.187 1	1<br>Obart02g37790.1 0.06 0.632 0.572 0.133 0.977<br>Obart12g06510.1 0.685 0.999 0.314 0.066 0.997	0<br>Olong_013175-R1 0.264 0.904 0.64 0.002 0.961	1<br>Opunc03g00470.1 0.001 0.783 0.782 0.002 1<br>Opunc11g04590.1 0.783 0.998 0.215 0.055 1	1<br>Obrac02g10260.1 0.712 0.999 0.287 0.265 0.995<br>Obrac02g27970.1 0.134 0.661 0.527 0.105 1	1<br>Lperr03g00570.1 0.001 0.657 0.656 0.002 0.994<br>Lperr04g11360.1 0.752 0.998 0.246 0.415 0.998	0<br>AT5G04460.1 0.017 0.661 0.644 0.081 1
Osjap03g00680.2	1<br>Osjap03g00680.1 0.001 1 0.999 0.001 1<br>Osjap03g00680.2 0.001 1 0.999 0.001 1<br>Osjap03g00680.3 0.001 1 0.999 0.001 1	1<br>Osind03g00740.1 0.001 0.994 0.993 0.001 0.996	1<br>Orufi03g12660.1 0.001 1 0.999 0.001 1<br>Orufi03g12660.2 0.001 1 0.999 0.001 1	1<br>Oglab03g00390.1 0.001 1 0.999 0.001 1	1<br>Obart03g00550.1 0.001 0.994 0.993 0.001 0.996	0	0	0	0	0
	1<br>Osjap02g42090.1 0.052 0.68 0.628 0.065 1<br>Osjap12g08580.1 0.702 0.999 0.297 0.011 0.996	1<br>Osind02g41670.1 0.062 0.68 0.618 0.131 1<br>Osind03g02220.1 0.691 1 0.309 0.015 1	1<br>Orufi02g39350.1 0.062 0.68 0.618 0.131 1<br>Orufi09g02220.2 0.738 1 0.262 0.095 1	1<br>Oglab02g34920.1 0.062 0.68 0.618 0.131 1<br>Oglab03g02050.1 0.691 1 0.309 0.214 1	1<br>Obart02g37790.1 0.062 0.65 0.588 0.133 0.977<br>Obart12g06510.1 0.681 0.999 0.318 0.079 0.997	0<br>Olong_013175-R1 0.272 0.902 0.63 0.002 0.961	1<br>Opunc03g00470.1 0.001 0.777 0.776 0.002 1<br>Opunc03g03130.2 0.777 0.999 0.222 0.443 0.997	1<br>Obrac02g10260.1 0.704 0.999 0.295 0.265 0.995<br>Obrac02g27970.1 0.138 0.68 0.542 0.105 1	1<br>Lperr03g00570.1 0.001 0.68 0.679 0.002 1<br>Lperr04g11360.1 0.745 0.998 0.253 0.415 0.998	0<br>AT5G04460.1 0.018 0.68 0.662 0.081 1
Osjap03g13100.1	1<br>Osjap03g13100.1 0.001 1 0.999 0.001 1	1<br>Osind03g12930.1 0.001 1 0.999 0.028 1	0	1<br>Oglab03g12120.1 0.001 1 0.999 0.028 1	1<br>Obart03g12120.1 0.001 1 0.999 0.028 1	0	0	0	0	0
	1<br>Osjap04g16240.1 0.632 0.998 0.366 0.082 0.921<br>Osjap12g01140.1 0.084 0.622 0.538 0.498 0.981	1<br>Osind04g14790.1 0.632 0.998 0.366 0.013 0.911<br>Osind12g01290.1 0.084 0.622 0.538 0.49 0.981	1<br>Orufi06g01460.3 0.632 0.998 0.366 0.212 0.912<br>Orufi12g01130.1 0.084 0.622 0.538 0.498 0.981	1<br>Oglab06g01620.1 0.632 0.998 0.366 0.233 0.912<br>Oglab12g00800.1 0.089 0.622 0.533 0.483 0.983	1<br>Obart06g01490.1 0.632 0.998 0.366 0.233 0.912<br>Obart11g01060.1 0.089 0.622 0.533 0.471 0.982	1<br>Olong_007714-R9 0.632 1 0.368 0.002 1<br>Olong_007715-R1 0.1 0.623 0.523 0.007 0.965	1<br>Opunc01g28650.1 0.636 0.998 0.362 0.094 0.923<br>Opunc03g11680.1 0.001 0.623 0.622 0.001 0.971	1<br>Obrac03g09720.2 0.001 0.623 0.622 0.001 0.97<br>Obrac04g09430.1 0.63 0.998 0.368 0.014 0.863	1<br>Lperr03g10720.1 0.001 0.623 0.622 0.001 0.934<br>Lperr03g17470.1 0.623 0.998 0.375 0.119 0.828	1<br>AT1G20230.1 0.635 0.998 0.363 0.104 0.833<br>AT3G46740.1 0.097 0.623 0.526 0.158 0.971
Osjap03g30460.1	1<br>Osjap03g30460.1 0.001 1 0.999 0.001 1	1<br>Osind03g31500.1 0.018 1 0.982 0.031 1	1<br>Orufi03g29110.1 0.018 1 0.982 0.296 1	0	1<br>Obart03g27980.1 0.08 1 0.92 0.016 1<br>Obart03g27980.2 0.08 1 0.92 0.015 1	0	0	0	0	0
	1<br>Osjap03g30380.1 0.547 0.999 0.452 0.059 0.866<br>Osjap12g24400.2 0.083 0.517 0.434 0.012 0.988	1<br>Osind08g14630.1 0.547 0.999 0.452 0.069 0.96<br>Osind12g20440.1 0.083 0.517 0.434 0.01 0.99	1<br>Orufi03g29970.1 0.547 0.999 0.452 0.035 0.998<br>Orufi12g21550.1 0.083 0.517 0.434 0.01 0.99	1<br>Oglab03g27330.1 0.547 1 0.453 0.036 1<br>Oglab12g17460.4 0.083 0.517 0.434 0.012 0.996	1<br>Obart03g27900.1 0.547 0.999 0.452 0.053 0.865<br>Obart12g19300.1 0.083 0.517 0.434 0.01 0.99	1<br>Olong_002926-R1 0.088 0.519 0.431 0.002 0.996<br>Olong_007449-R1 0.547 0.999 0.452 0.058 0.881	1<br>Opunc03g25490.1 0.014 0.528 0.514 0.486 0.8<br>Opunc03g26870.1 0.548 0.999 0.451 0.053 1	0<br>Obrac03g20710.1 0.085 0.519 0.434 0.028 0.997	1<br>Lperr03g23190.1 0.547 0.999 0.452 0.03 0.867<br>Lperr03g23200.1 0.07 0.519 0.449 0.002 0.997	0<br>AT5G36940.1 0.084 0.52 0.436 0.003 0.998
Osjap03g34330.1	1<br>Osjap03g34330.1 0.001 1 0.999 0.001 1	1<br>Osind03g35550.1 0.001 1 0.999 0.039 1	1<br>Orufi03g33060.1 0.001 1 0.999 0.001 1	1<br>Oglab03g30830.1 0.001 1 0.999 0.001 1	1<br>Obart03g31750.1 0.001 1 0.999 0.046 1	0	0	0	0	0
	1<br>Osjap03g39110.1 0.634 1 0.366 0.036 1<br>Osjap10g05370.1 0.012 0.609 0.597 0.1 0.963	1<br>Osind01g46540.1 0.634 0.999 0.365 0.065 0.994<br>Osind10g03770.1 0.012 0.609 0.597 0.11 0.96	1<br>Orufi10g04850.1 0.031 0.609 0.578 0.093 0.963<br>Orufi11g12520.1 0.635 1 0.365 0.038 0.991	1<br>Oglab03g34460.1 0.609 0.997 0.388 0.016 0.993<br>Oglab10g04340.1 0.012 0.609 0.597 0.1 0.963	1<br>Obart03g36200.1 0.634 1 0.366 0.016 0.997<br>Obart10g04600.1 0.014 0.609 0.595 0.106 0.962	1<br>Olong_014796-R1 0.001 0.516 0.515 0.002 0.95<br>Olong_027495-R1 0.609 0.994 0.385 0.005 0.998	1<br>Opunc01g42030.1 0.634 1 0.366 0.28 0.872<br>Opunc03g28940.2 0.001 0.609 0.608 0.002 0.962	1<br>Obrac02g16280.1 0.628 0.981 0.353 0.063 0.949<br>Obrac03g23440.1 0.001 0.609 0.608 0.002 0.964	1<br>Lperr03g26400.1 0.001 0.609 0.608 0.002 0.962<br>Lperr03g30520.1 0.626 1 0.374 0.003 0.997	0<br>AT1G70730.3 0.001 0.609 0.608 0.118 0.967
Osjap03g36400.1	1<br>Osjap03g36400.1 0.003 1 0.997 0.003 1	1<br>Osind03g37600.1 0.003 1 0.997 0.003 1	1<br>Orufi03g35150.1 0.003 1 0.997 0.003 1	1<br>Oglab03g32170.1 0.003 1 0.997 0.003 1<br>Oglab03g32170.2 0.003 1 0.997 0.003 1	1<br>Obart03g33760.1 0.003 1 0.997 0.003 1	1<br>Olong_002225-R1 0.003 1 0.997 0.136 1<br>Olong_002225-R2 0.003 1 0.997 0.003 1	1<br>Opunc03g30920.1 0.003 1 0.997 0.003 1	0	0	0

	1<br>Osjap01g12980.2 0.547 0.979 0.432 0.032 0.961<br>Osjap03g04690.1 0.003 0.414 0.411 0.007 1	1<br>Osind03g04960.1 0.003 0.414 0.411 0.007 1<br>Osind12g02610.1 0.538 0.991 0.453 0.005 0.759	1<br>Orufi03g04350.1 0.003 0.414 0.411 0.007 1<br>Orufi05g22410.2 0.547 0.997 0.45 0.031 0.969	0<br>Oglab05g19940.1 0.32 0.991 0.671 0.015 0.997	1<br>Obart01g34520.1 0.547 0.991 0.444 0.027 0.818<br>Obart03g04650.1 0.003 0.414 0.411 0.007 1	1<br>Olong_020510-R2 0.523 0.991 0.468 0.291 0.873<br>Olong_025165-R1 0.003 0.414 0.411 0.007 1	0<br>Opunc12g01920.1 0.526 0.973 0.447 0.663 0.947	0<br>Obrac03g25020.5 0.003 0.535 0.532 0.439 0.972	1<br>Lperr01g29830.2 0.532 0.991 0.459 0.203 0.858<br>Lperr03g28250.1 0.003 0.414 0.411 0.007 1	1<br>AT1G52740.1 0.003 0.414 0.411 0.007 1<br>AT4G14640.1 0.544 0.979 0.435 0.033 0.987
Osjap05g02530.1	1<br>Osjap05g02530.1 0.001 1 0.999 0.001 1	1<br>Osind05g02510.1 0.001 0.998 0.997 0.001 0.987	0	1<br>Oglab05g02190.1 0.002 1 0.998 0.079 1	1<br>Obart05g02340.1 0.002 1 0.998 0.076 1	0	0	0	0	0
	1<br>Osjap03g00960.1 0.459 0.994 0.535 0.132 0.996<br>Osjap06g18010.1 0.001 0.338 0.337 0.184 1	1<br>Osind03g01020.1 0.459 0.994 0.535 0.136 0.996<br>Osind06g17540.1 0.001 0.324 0.323 0.003 0.865	1<br>Orufi05g02460.2 0.459 1 0.541 0.003 0.982<br>Orufi06g16520.1 0.001 0.324 0.323 0.002 0.884	1<br>Oglab03g00660.1 0.459 0.994 0.535 0.124 0.996<br>Oglab05g07310.1 0.001 0.324 0.323 0.003 0.935	1<br>Obart03g00820.2 0.459 0.994 0.535 0.022 0.995<br>Obart06g15630.1 0.001 0.324 0.323 0.002 0.955	1<br>Olong_016162-R1 0.001 0.324 0.323 0.002 0.954<br>Olong_026687-R1 0.427 1 0.573 0.038 0.983	1<br>Opunc05g02220.2 0.459 0.999 0.54 0.003 0.979<br>Opunc07g20770.2 0.001 0.324 0.323 0.002 0.832	1<br>Obrac05g01840.1 0.458 0.998 0.54 0.168 1<br>Obrac06g15870.1 0.001 0.326 0.325 0.003 0.84	0<br>Lperr05g02100.1 0.457 0.998 0.541 0.015 0.978	0<br>AT5G15380.1 0.449 0.992 0.543 0.066 0.987
Osjap05g04120.1	1<br>Osjap05g04120.1 0.001 1 0.999 0.001 1<br>Osjap05g04120.2 0.001 1 0.999 0.001 1	1<br>Osind05g04070.1 0.008 1 0.992 0.001 1<br>Osind05g04070.2 0.008 1 0.992 0.001 1	1<br>Orufi05g03950.1 0.001 1 0.999 0.001 1<br>Orufi05g03950.2 0.001 1 0.999 0.001 1	1<br>Oglab05g03490.1 0.001 1 0.999 0.001 1<br>Oglab05g03490.2 0.001 1 0.999 0.001 1	1<br>Obart05g03830.1 0.001 1 0.999 0.001 1<br>Obart05g03830.2 0.001 1 0.999 0.001 1	1<br>Olong_016739-R1 0.074 1 0.926 0.001 1	1<br>Opunc05g03540.1 0.001 1 0.999 0.001 1<br>Opunc05g03540.2 0.001 1 0.999 0.001 1	1<br>Obrac05g02930.1 0.074 1 0.926 0.001 1	0	0
	1<br>Osjap01g27410.1 0.09 0.723 0.633 0.066 0.996<br>Osjap03g15140.5 0.743 1 0.257 0.061 1	1<br>Osind01g28630.1 0.09 0.723 0.633 0.066 0.996<br>Osind03g15230.1 0.79 1 0.21 0.03 1	1<br>Orufi01g26130.1 0.09 0.723 0.633 0.066 0.996<br>Orufi03g14720.1 0.79 1 0.21 0.03 1	1<br>Oglab01g19490.1 0.09 0.723 0.633 0.066 0.996<br>Oglab03g14060.1 0.79 1 0.21 0.03 1	1<br>Obart01g23310.1 0.09 0.723 0.633 0.066 0.996<br>Obart03g14050.1 0.79 1 0.21 0.03 1	1<br>Olong_003347-R2 0.09 0.723 0.633 0.047 0.996<br>Olong_009943-R1 0.79 1 0.21 0.03 1	1<br>Opunc01g23460.1 0.09 0.723 0.633 0.064 0.996<br>Opunc03g13590.1 0.79 1 0.21 0.002 0.545	1<br>Obrac01g17590.1 0.09 0.723 0.633 0.067 0.996<br>Obrac03g11330.1 0.79 1 0.21 0.03 1	0<br>Lperr05g03460.1 0.001 0.721 0.72 0.001 0.993	1<br>AT2G47580.1 0.775 1 0.225 0.008 1<br>AT3G16630.1 0.003 0.721 0.718 0.031 0.992
Osjap05g04120.2	1<br>Osjap05g04120.1 0.001 1 0.999 0.001 1<br>Osjap05g04120.2 0.001 1 0.999 0.001 1	1<br>Osind05g04070.1 0.008 1 0.992 0.001 1<br>Osind05g04070.2 0.008 1 0.992 0.001 1	1<br>Orufi05g03950.1 0.001 1 0.999 0.001 1<br>Orufi05g03950.2 0.001 1 0.999 0.001 1	1<br>Oglab05g03490.1 0.001 1 0.999 0.001 1<br>Oglab05g03490.2 0.001 1 0.999 0.001 1	1<br>Obart05g03830.1 0.001 1 0.999 0.001 1<br>Obart05g03830.2 0.001 1 0.999 0.001 1	1<br>Olong_016739-R1 0.073 1 0.927 0.001 1	1<br>Opunc05g03540.1 0.001 1 0.999 0.001 1<br>Opunc05g03540.2 0.001 1 0.999 0.001 1	1<br>Obrac05g02930.1 0.073 1 0.927 0.001 1	0	0
	1<br>Osjap01g27410.1 0.089 0.726 0.637 0.066 0.996<br>Osjap03g15140.5 0.747 1 0.253 0.061 1	1<br>Osind01g28630.1 0.089 0.726 0.637 0.066 0.996<br>Osind03g15230.1 0.792 1 0.208 0.03 1	1<br>Orufi01g26130.1 0.089 0.726 0.637 0.066 0.996<br>Orufi03g14720.1 0.792 1 0.208 0.03 1	1<br>Oglab01g19490.1 0.089 0.726 0.637 0.066 0.996<br>Oglab03g14060.1 0.792 1 0.208 0.03 1	1<br>Obart01g23310.1 0.089 0.726 0.637 0.066 0.996<br>Obart03g14050.1 0.792 1 0.208 0.03 1	1<br>Olong_003347-R2 0.089 0.726 0.637 0.047 0.996<br>Olong_009943-R1 0.792 1 0.208 0.03 1	1<br>Opunc01g23460.1 0.089 0.726 0.637 0.064 0.996<br>Opunc03g13590.1 0.792 1 0.208 0.002 0.545	1<br>Obrac01g17590.1 0.089 0.726 0.637 0.067 0.996<br>Obrac03g11330.1 0.792 1 0.208 0.03 1	0<br>Lperr05g03460.1 0.001 0.725 0.724 0.001 0.993	1<br>AT2G47580.1 0.778 1 0.222 0.008 1<br>AT3G16630.1 0.003 0.725 0.722 0.031 0.992
Osjap05g28230.1	1<br>Osjap05g28230.1 0.001 1 0.999 0.001 1	1<br>Osind05g28520.1 0.001 1 0.999 0.001 1	1<br>Orufi05g26870.1 0.001 1 0.999 0.001 1	1<br>Oglab05g23160.1 0.001 1 0.999 0.001 1	1<br>Obart05g25070.1 0.001 1 0.999 0.001 1	0	1<br>Opunc05g22640.1 0.001 1 0.999 0.001 1	0	1<br>Lperr01g22990.1 0.001 1 0.999 0.027 1	0
	1<br>Osjap01g31140.1 0.006 0.432 0.426 0.093 0.997<br>Osjap01g31150.1 0.487 0.999 0.512 0.009 0.999	1<br>Osind01g32600.1 0.001 0.431 0.43 0.002 0.995<br>Osind01g32610.1 0.487 0.999 0.512 0.009 0.999	1<br>Orufi01g29880.1 0.001 0.432 0.431 0.075 0.997<br>Orufi01g29890.1 0.487 0.999 0.512 0.009 0.999	1<br>Oglab01g22740.1 0.027 0.432 0.405 0.061 0.996<br>Oglab01g22750.1 0.487 0.999 0.512 0.009 0.999	1<br>Obart01g26870.1 0.005 0.432 0.427 0.117 0.997<br>Obart01g26880.1 0.487 0.999 0.512 0.009 0.999	1<br>Olong_000367-R1 0.1 0.432 0.332 0.002 0.995<br>Olong_000368-R1 0.462 0.999 0.537 0.069 0.999	1<br>Opunc01g26820.1 0.047 0.432 0.385 0.002 0.996<br>Opunc01g26830.1 0.487 1 0.513 0.009 1	1<br>Obrac01g20400.1 0.044 0.432 0.388 0.004 0.996<br>Obrac05g17560.2 0.432 1 0.568 0.109 1	1<br>Lperr05g20850.2 0.491 1 0.509 0.002 1<br>Lperr05g20860.1 0.001 0.432 0.431 0.002 0.996	1<br>AT5G08690.1 0.011 0.431 0.42 0.034 0.995<br>AT5G51600.1 0.487 0.999 0.512 0.008 0.996
Osjap06g00940.1	1<br>Osjap06g00940.1 0.002 1 0.998 0.002 1	0	1<br>Orufi06g00860.1 0.118 1 0.882 0.021 1	1<br>Oglab06g00840.1 0.085 1 0.915 0.04 1	1<br>Obart06g00880.1 0.085 1 0.915 0.04 1	0	0	0	0	0
	1<br>Osjap06g00950.1 0.415 0.99 0.575 0.132 0.99<br>Osjap08g13790.1 0.083 0.327 0.244 0.005 1	1<br>Osind06g00910.1 0.411 1 0.589 0.068 1<br>Osind07g02910.1 0.091 0.325 0.234 0.012 1	1<br>Orufi06g00690.1 0.415 1 0.585 0.099 1<br>Orufi07g02700.1 0.091 0.325 0.234 0.012 1	1<br>Oglab06g00730.1 0.411 0.987 0.576 0.257 0.968<br>Oglab07g02750.1 0.1 0.325 0.225 0.007 1	1<br>Obart06g00690.1 0.415 1 0.585 0.099 1<br>Obart08g12830.1 0.104 0.324 0.22 0.012 0.988	1<br>Olong_006603-R1 0.04 0.324 0.284 0.034 0.987<br>Olong_022685-R1 0.415 1 0.585 0.074 1	1<br>Opunc06g00800.1 0.325 1 0.675 0.025 1<br>Opunc06g00830.1 0.097 0.325 0.228 0.003 1	1<br>Obrac02g00120.1 0.1 0.325 0.225 0.006 1<br>Obrac06g00660.1 0.411 1 0.589 0.034 0.999	0<br>Lperr01g05300.1 0.108 0.325 0.217 0.013 1<br>Lperr06g00740.1 0.415 0.998 0.583 0.073 0.997	0<br>AT1G20140.1 0.112 0.325 0.213 0.018 1
Osjap08g06650.1	1<br>Osjap08g06650.1 0.002 1 0.998 0.002 1	1<br>Osind08g06790.1 0.002 1 0.998 0.002 1	1<br>Orufi08g06840.1 0.002 1 0.998 0.002 1	1<br>Oglab08g05640.1 0.002 1 0.998 0.002 1	1<br>Obart08g06070.1 0.002 1 0.998 0.002 1	0	1<br>Opunc08g05850.1 0.047 1 0.953 0.068 1	0	0	0
	1	1	1	1	1	1	1	1	1	1

	Osjap04g21220.1 0.047 0.275 0.228 0.21 0.936<br>Osjap09g20870.1 0.359 0.984 0.625 0.208 0.966	Osind04g20250.1 0.047 0.275 0.228 0.198 0.94<br>Osind09g20960.1 0.359 0.984 0.625 0.208 0.966	Orufi04g19250.2 0.047 0.275 0.228 0.21 0.936<br>Orufi09g21960.1 0.359 0.984 0.625 0.2 0.965	Oglab02g26360.1 0.348 0.996 0.648 0.01 0.994<br>Oglab04g16180.1 0.047 0.275 0.228 0.198 0.94	Obart02g28440.1 0.348 0.996 0.648 0.093 0.994<br>Obart04g17770.1 0.047 0.275 0.228 0.198 0.94	Olong_008030-R1 0.047 0.275 0.228 0.232 0.937<br>Olong_008031-R2 0.277 1 0.723 0.106 1	Opunc04g15550.1 0.047 0.275 0.228 0.206 0.938<br>Opunc04g18740.1 0.295 0.984 0.689 0.088 0.994	Obrac08g04780.1 0.277 1 0.723 0.098 1<br>Obrac08g04800.1 0.002 0.275 0.273 0.006 0.943	Lperr08g05690.1 0.277 1 0.723 0.1 1<br>Lperr08g05700.1 0.002 0.275 0.273 0.006 0.943	AT1G05310.1 0.33 0.982 0.652 0.183 0.99<br>AT4G30800.1 0.047 0.275 0.228 0.208 0.943
Osjap09g15430.2	1<br>Osjap09g15430.2 0.002 1 0.998 0.002 1<br>Osjap09g15430.3 0.002 1 0.998 0.002 1	1<br>Osind09g15570.1 0.002 1 0.998 0.002 1<br>Osind09g15570.2 0.002 1 0.998 0.002 1	1<br>Orufi09g16160.1 0.002 1 0.998 0.002 1<br>Orufi09g16160.2 0.002 1 0.998 0.002 1	1<br>Oglab09g13510.1 0.002 1 0.998 0.002 1<br>Oglab09g13510.2 0.002 1 0.998 0.002 1<br>Oglab09g13510.3 0.002 1 0.998 0.002 1<br>Oglab09g13510.4 0.002 1 0.998 0.002 1	1<br>Obart09g14980.1 0.002 1 0.998 0.002 1<br>Obart09g14980.2 0.002 1 0.998 0.002 1	0	1<br>Opunc09g13420.1 0.002 1 0.998 0.002 1<br>Opunc09g13420.2 0.002 1 0.998 0.002 1	1<br>Obrac09g09090.1 0.002 1 0.998 0.002 1	0	0
	0<br>Osjap02g06650.1 0.01 0.565 0.555 0.01 0.997	1<br>Osind02g06940.1 0.01 0.565 0.555 0.01 0.997<br>Osind08g23920.1 0.569 0.993 0.424 0.131 0.988	1<br>Orufi04g24220.1 0.014 0.567 0.553 0.165 1<br>Orufi08g22880.1 0.569 0.993 0.424 0.131 0.988	1<br>Oglab08g19240.1 0.569 0.993 0.424 0.143 0.986<br>Oglab12g18680.1 0.002 0.567 0.565 0.003 1	1<br>Obart02g06180.1 0.01 0.565 0.555 0.01 0.997<br>Obart08g20390.1 0.569 0.993 0.424 0.631 0.988	1<br>Olong_008093-R2 0.596 0.991 0.395 0.25 0.996<br>Olong_024186-R1 0.002 0.567 0.565 0.003 1	1<br>Opunc04g20190.1 0.007 0.567 0.56 0.006 1<br>Opunc08g18560.1 0.569 0.993 0.424 0.128 0.969	1<br>Obrac01g09090.1 0.605 0.998 0.393 0.299 0.982<br>Obrac02g04550.3 0.009 0.565 0.556 0.214 0.997	1<br>Lperr04g18350.1 0.002 0.567 0.565 0.021 1<br>Lperr09g11120.1 0.569 1 0.431 0.156 1	1<br>AT1G68540.1 0.567 0.991 0.424 0.112 1<br>AT2G21270.3 0.003 0.565 0.562 0.059 0.997
Osjap09g15430.3	1<br>Osjap09g15430.2 0.002 1 0.998 0.002 1<br>Osjap09g15430.3 0.002 1 0.998 0.002 1	1<br>Osind09g15570.1 0.002 1 0.998 0.002 1<br>Osind09g15570.2 0.002 1 0.998 0.002 1	1<br>Orufi09g16160.1 0.002 1 0.998 0.002 1<br>Orufi09g16160.2 0.002 1 0.998 0.002 1	1<br>Oglab09g13510.1 0.002 1 0.998 0.002 1<br>Oglab09g13510.2 0.002 1 0.998 0.002 1<br>Oglab09g13510.3 0.002 1 0.998 0.002 1<br>Oglab09g13510.4 0.002 1 0.998 0.002 1	1<br>Obart09g14980.1 0.002 1 0.998 0.002 1<br>Obart09g14980.2 0.002 1 0.998 0.002 1	0	1<br>Opunc09g13420.1 0.002 1 0.998 0.002 1<br>Opunc09g13420.2 0.002 1 0.998 0.002 1	1<br>Obrac09g09090.1 0.002 1 0.998 0.002 1	0	0
	0<br>Osjap02g06650.1 0.01 0.54 0.53 0.01 0.997	1<br>Osind02g06940.1 0.01 0.54 0.53 0.01 0.997<br>Osind08g23920.1 0.544 0.993 0.449 0.131 0.988	1<br>Orufi04g24220.1 0.013 0.542 0.529 0.165 1<br>Orufi08g22880.1 0.544 0.993 0.449 0.131 0.988	1<br>Oglab08g19240.1 0.544 0.993 0.449 0.143 0.986<br>Oglab12g18680.1 0.002 0.542 0.54 0.003 1	1<br>Obart02g06180.1 0.01 0.54 0.53 0.01 0.997<br>Obart08g20390.1 0.544 0.993 0.449 0.631 0.988	1<br>Olong_001228-R1 0.548 0.992 0.444 0.03 0.947<br>Olong_024186-R1 0.002 0.542 0.54 0.003 1	1<br>Opunc04g20190.1 0.007 0.542 0.535 0.006 1<br>Opunc08g18560.1 0.544 0.993 0.449 0.128 0.969	1<br>Obrac02g04550.3 0.008 0.54 0.532 0.214 0.997<br>Obrac09g00560.1 0.548 0.998 0.45 0.164 0.966	1<br>Lperr04g18350.1 0.002 0.542 0.54 0.021 1<br>Lperr09g11120.1 0.544 1 0.456 0.156 1	1<br>AT1G68540.1 0.542 0.992 0.45 0.112 1<br>AT2G21270.3 0.003 0.54 0.537 0.059 0.997
Osjap09g20460.1	1<br>Osjap09g20460.1 0.001 1 0.999 0.001 1	1<br>Osind09g20560.1 0.001 0.986 0.985 0.001 0.998	1<br>Orufi09g21510.1 0.001 0.967 0.966 0.001 0.999	1<br>Oglab09g18200.1 0.001 0.999 0.998 0.001 0.999	1<br>Obart09g19980.1 0.062 0.999 0.937 0.165 0.999<br>Obart09g19980.2 0.001 0.999 0.998 0.001 0.999	1<br>Olong_014589-R1 0.108 0.974 0.866 0.018 0.991<br>Olong_014589-R2 0.108 0.974 0.866 0.018 0.991	0	0	0	0
	1<br>Osjap02g21530.2 0.022 0.584 0.562 0.096 0.943<br>Osjap09g20430.1 0.624 1 0.376 0.085 1	1<br>Osind02g21430.2 0.022 0.584 0.562 0.096 0.943<br>Osind09g20550.1 0.624 1 0.376 0.032 1	1<br>Orufi02g19810.2 0.022 0.584 0.562 0.096 0.943<br>Orufi09g21500.1 0.624 1 0.376 0.085 1	1<br>Oglab02g17850.1 0.022 0.584 0.562 0.095 0.914<br>Oglab09g18170.1 0.624 1 0.376 0.079 1	1<br>Obart04g25370.1 0.044 0.592 0.548 0.176 0.967<br>Obart09g19950.1 0.624 1 0.376 0.079 1	1<br>Olong_014116-R1 0.059 0.592 0.533 0.069 0.957<br>Olong_027417-R1 0.626 0.991 0.365 0.135 0.745	1<br>Opunc09g18280.1 0.619 0.998 0.379 0.058 0.951<br>Opunc09g18290.2 0.001 0.592 0.591 0.001 0.97	1<br>Obrac09g13140.3 0.624 0.986 0.362 0.049 0.806<br>Obrac09g13160.3 0.001 0.593 0.592 0.001 0.974	1<br>Lperr09g15890.1 0.623 0.996 0.373 0.048 0.957<br>Lperr09g15920.1 0.001 0.592 0.591 0.001 0.957	0<br>AT1G18160.1 0.02 0.592 0.572 0.064 0.958
Osjap10g04950.1	1<br>Osjap10g04950.1 0.01 1 0.99 0.01 1<br>Osjap10g21210.1 0.01 1 0.99 0.477 1	1<br>Osind10g03420.1 0.01 1 0.99 0.558 1<br>Osind10g17700.1 0.01 1 0.99 0.477 1	1<br>Orufi10g04520.1 0.01 1 0.99 0.01 1<br>Orufi10g19420.1 0.01 1 0.99 0.477 1	1<br>Oglab10g03910.1 0.01 1 0.99 0.01 1	1<br>Obart10g18220.1 0.01 1 0.99 0.392 0.823	1<br>Olong_009744-R1 0.01 1 0.99 0.651 1<br>Olong_009744-R2 0.01 1 0.99 0.641 1	0	0	0	0
	0<br>Osjap07g13660.9 0.361 1 0.639 0.733 0.947	1<br>Osind02g38620.1 0.361 1 0.639 0.835 0.923<br>Osind06g00010.1 0.01 0.361 0.351 0.609 0.978	1<br>Orufi02g36440.1 0.361 1 0.639 0.835 0.923<br>Orufi08g26670.5 0.01 0.351 0.341 0.583 0.749	1<br>Oglab02g32260.1 0.361 1 0.639 0.838 0.924<br>Oglab04g27900.1 0.01 0.33 0.32 0.14 0.208	1<br>Obart02g34980.1 0.361 1 0.639 0.838 0.924<br>Obart04g29850.1 0.01 0.33 0.32 0.131 0.196	1<br>Olong_008835-R2 0.361 1 0.639 0.809 0.91<br>Olong_022843-R2 0.01 0.33 0.32 0.14 0.208	1<br>Opunc02g32260.1 0.361 1 0.639 0.84 0.925<br>Opunc04g27380.2 0.01 0.33 0.32 0.14 0.208	1<br>Obrac02g25650.1 0.381 1 0.619 0.839 0.923<br>Obrac04g21100.1 0.01 0.33 0.32 0.145 0.217	1<br>Lperr02g28770.1 0.381 1 0.619 0.834 0.92<br>Lperr04g25370.2 0.01 0.33 0.32 0.151 0.225	0
Osjap10g14600.1	1<br>Osjap10g14600.1 0.002 1 0.998 0.002 1<br>Osjap10g14600.2 0.002 1 0.998 0.002 1<br>Osjap10g14600.3 0.002 1 0.998 0.002 1<br>Osjap10g14600.4 0.002 1 0.998 0.002 1	1<br>Osind10g11660.1 0.002 0.995 0.993 0.003 0.958	1<br>Orufi08g13110.1 0.002 0.987 0.985 0.003 0.99	1<br>Oglab10g11970.1 0.002 0.982 0.98 0.007 1	1<br>Obart10g12400.1 0.002 0.98 0.978 0.004 0.996	0	1<br>Opunc10g10650.1 0.002 0.995 0.993 0.003 0.974<br>Opunc10g10650.2 0.002 0.995 0.993 0.003 0.974	0	0	0

	Osjap10g14600.5 0.002 1 0.998 0.002 1<br>Osjap10g14600.6 0.002 1 0.998 0.002 1									
	0<br>Osjap08g12520.1 0.277 0.98 0.703 0.226 0.86	1<br>Osind08g14090.1 0.664 0.987 0.323 0.346 0.989<br>Osind10g11740.1 0.16 0.638 0.478 0.002 0.08	1<br>Orufi08g13110.2 0.664 0.987 0.323 0.346 0.989<br>Orufi10g13050.2 0.002 0.658 0.656 0.003 0.627	1<br>Oglab03g12700.1 0.701 0.975 0.274 0.29 0.977<br>Oglab10g11980.2 0.04 0.649 0.609 0.016 0.42	1<br>Obart03g12670.1 0.701 0.975 0.274 0.29 0.977<br>Obart10g12410.2 0.04 0.653 0.613 0.046 0.639	1<br>Olong_005116-R1 0.658 0.975 0.317 0.171 0.982<br>Olong_028277-R1 0.002 0.651 0.649 0.006 0.975	1<br>Opunc10g10640.1 0.04 0.638 0.598 0.049 0.61<br>Opunc12g14410.1 0.7 0.975 0.275 0.253 0.984	1<br>Obrac10g07370.1 0.117 0.638 0.521 0.003 0.101<br>Obrac12g10200.1 0.66 0.975 0.315 0.237 0.985	1<br>Lperr03g19720.1 0.653 0.982 0.329 0.285 1<br>Lperr10g09020.1 0.117 0.638 0.521 0.001 0.059	1<br>AT3G04260.1 0.117 0.638 0.521 0.075 0.193<br>AT3G55330.1 0.653 0.975 0.322 0.2 0.991
Osjap11g00600.1	1<br>Osjap11g00600.1 0.002 1 0.998 0.002 1<br>Osjap12g00550.1 0.002 0.998 0.996 0.002 1	1<br>Osind12g00790.1 0.002 0.998 0.996 0.002 1	1<br>Orufi11g00440.1 0.002 1 0.998 0.002 1	1<br>Oglab11g00570.1 0.106 1 0.894 0.051 1<br>Oglab12g00270.1 0.002 0.801 0.799 0.003 0.995	1<br>Obart11g00510.1 0.106 1 0.894 0.045 1<br>Obart12g00320.1 0.002 0.998 0.996 0.003 1	0	0	0	0	0
	1<br>Osjap08g03600.1 0.748 0.984 0.236 0.201 0.274<br>Osjap10g06830.2 0.087 0.719 0.632 0.265 0.998	1<br>Osind05g03780.1 0.748 0.984 0.236 0.16 0.63<br>Osind10g04960.1 0.063 0.719 0.656 0.31 0.998	1<br>Orufi08g03710.3 0.748 0.984 0.236 0.289 0.394<br>Orufi12g00580.1 0.002 0.703 0.701 0.002 0.881	0<br>Oglab10g05540.1 0.087 0.719 0.632 0.32 0.998	1<br>Obart08g03340.1 0.748 0.984 0.236 0.191 0.268<br>Obart12g18380.1 0.069 0.717 0.648 0.137 0.977	1<br>Olong_026176-R1 0.083 0.717 0.634 0.114 0.997<br>Olong_027703-R1 0.748 0.984 0.236 0.192 0.274	1<br>Opunc08g03130.1 0.748 0.984 0.236 0.206 0.282<br>Opunc12g00560.1 0.002 0.724 0.722 0.003 0.995	1<br>Obrac08g02840.1 0.748 0.984 0.236 0.201 0.274<br>Obrac12g00030.1 0.004 0.624 0.62 0.412 0.991	1<br>Lperr12g00550.1 0.748 0.986 0.238 0.194 0.271<br>Lperr12g00630.2 0.033 0.719 0.686 0.031 0.983	0<br>AT2G40030.1 0.738 0.982 0.244 0.168 0.234
Osjap11g01220.1	1<br>Osjap11g01220.1 0.001 1 0.999 0.001 1<br>Osjap12g01140.1 0.001 1 0.999 0.001 1	1<br>Osind11g00690.1 0.001 0.955 0.954 0.001 0.999<br>Osind12g01290.1 0.001 1 0.999 0.001 1	1<br>Orufi11g01000.1 0.001 1 0.999 0.001 1<br>Orufi12g01130.1 0.001 1 0.999 0.001 1	1<br>Oglab12g00800.1 0.001 1 0.999 0.001 1	1<br>Obart11g01060.1 0.001 1 0.999 0.001 1	0	1<br>Opunc12g00940.1 0.001 1 0.999 0.001 1	0	0	0
	1<br>Osjap03g13100.1 0.489 0.982 0.493 0.097 0.622<br>Osjap07g30390.1 0.009 0.433 0.424 0.013 0.944	1<br>Osind03g12930.1 0.489 0.982 0.493 0.121 0.633<br>Osind07g27070.1 0.007 0.433 0.426 0.006 0.946	1<br>Orufi03g17220.1 0.496 1 0.504 0.027 1<br>Orufi07g27420.1 0.009 0.433 0.424 0.013 0.946	1<br>Oglab03g12120.1 0.489 0.982 0.493 0.121 0.633<br>Oglab07g22980.1 0.007 0.433 0.426 0.007 0.94	1<br>Obart03g12120.1 0.489 0.982 0.493 0.121 0.633<br>Obart12g00790.1 0.001 0.433 0.432 0.002 0.949	1<br>Olong_007715-R1 0.492 1 0.508 0.007 1<br>Olong_008211-R1 0.007 0.433 0.426 0.006 0.946	1<br>Opunc03g11680.1 0.489 1 0.511 0.148 1<br>Opunc11g00920.1 0.001 0.433 0.432 0.142 0.951	1<br>Obrac03g09720.2 0.489 1 0.511 0.142 1<br>Obrac12g00780.1 0.001 0.433 0.432 0.11 0.953	1<br>Lperr03g10720.1 0.489 0.993 0.504 0.148 0.952<br>Lperr11g00920.1 0.001 0.433 0.432 0.002 0.949	1<br>AT2G34940.1 0.001 0.433 0.432 0.002 0.955<br>AT3G46740.1 0.492 1 0.508 0.161 1
Osjap11g21440.1	1<br>Osjap11g21440.1 0.001 1 0.999 0.001 1	1<br>Osind04g04060.1 0.001 0.934 0.933 0.072 0.988	1<br>Orufi11g19860.1 0.003 1 0.997 0.052 1	1<br>Oglab11g16480.1 0.003 1 0.997 0.052 1	1<br>Obart11g18650.1 0.001 1 0.999 0.073 1	1<br>Olong_013913-R1 0.006 0.965 0.959 0.001 0.986	0	0	0	0
	1<br>Osjap03g43210.1 0.717 0.987 0.27 0.278 0.99<br>Osjap10g09150.2 0.008 0.716 0.708 0.047 0.738	1<br>Osind03g44240.1 0.717 0.987 0.27 0.017 0.988<br>Osind10g06650.1 0.008 0.716 0.708 0.008 0.713	1<br>Orufi03g41640.1 0.717 0.987 0.27 0.018 0.987<br>Orufi10g08130.1 0.008 0.716 0.708 0.047 0.737	1<br>Oglab03g37880.1 0.717 0.987 0.27 0.278 0.99<br>Oglab11g20100.1 0.02 0.711 0.691 0.029 0.947	1<br>Obart03g02940.1 0.739 0.986 0.247 0.118 0.932<br>Obart10g07740.1 0.008 0.716 0.708 0.047 0.737	1<br>Olong_001283-R1 0.002 0.674 0.672 0.014 0.844<br>Olong_011979-R1 0.739 0.987 0.248 0.108 0.954	1<br>Opunc03g37060.1 0.717 0.987 0.27 0.151 0.989<br>Opunc05g18050.1 0.047 0.702 0.655 0.06 0.817	1<br>Obrac03g30000.2 0.705 0.979 0.274 0.031 0.937<br>Obrac05g13790.3 0.029 0.702 0.673 0.127 0.872	1<br>Lperr03g33980.1 0.717 0.974 0.257 0.008 0.411<br>Lperr11g13500.1 0.043 0.677 0.634 0.047 0.638	0<br>AT3G12620.1 0.719 0.984 0.265 0.047 0.964
Osjap12g01140.1	1<br>Osjap11g01220.1 0.001 1 0.999 0.001 1<br>Osjap12g01140.1 0.001 1 0.999 0.001 1	1<br>Osind11g00690.1 0.001 0.953 0.952 0.001 0.999<br>Osind12g01290.1 0.001 1 0.999 0.001 1	1<br>Orufi11g01000.1 0.001 1 0.999 0.001 1<br>Orufi12g01130.1 0.001 1 0.999 0.001 1	1<br>Oglab12g00800.1 0.001 1 0.999 0.001 1	1<br>Obart11g01060.1 0.001 1 0.999 0.001 1	0	1<br>Opunc12g00940.1 0.001 1 0.999 0.001 1	0	0	0
	1<br>Osjap03g13100.1 0.511 0.981 0.47 0.097 0.622<br>Osjap07g30390.1 0.01 0.373 0.363 0.013 0.798	1<br>Osind03g12930.1 0.511 0.981 0.47 0.121 0.633<br>Osind07g27070.1 0.007 0.377 0.37 0.006 0.783	1<br>Orufi03g17220.1 0.517 1 0.483 0.027 1<br>Orufi07g27420.1 0.01 0.377 0.367 0.013 0.783	1<br>Oglab03g12120.1 0.511 0.981 0.47 0.121 0.633<br>Oglab07g22980.1 0.007 0.373 0.366 0.007 0.783	1<br>Obart03g12120.1 0.511 0.981 0.47 0.121 0.633<br>Obart10g06750.1 0.021 0.409 0.388 0.151 0.885	1<br>Olong_007715-R1 0.514 1 0.486 0.007 1<br>Olong_008211-R1 0.007 0.377 0.37 0.006 0.783	1<br>Opunc03g11680.1 0.511 1 0.489 0.148 1<br>Opunc11g00920.1 0.001 0.377 0.376 0.142 0.816	1<br>Obrac03g09720.2 0.511 1 0.489 0.142 1<br>Obrac10g04150.1 0.02 0.405 0.385 0.036 0.868	1<br>Lperr03g10720.1 0.511 0.993 0.482 0.148 0.952<br>Lperr11g00920.1 0.001 0.377 0.376 0.002 0.792	1<br>AT2G34940.1 0.001 0.377 0.376 0.002 0.793<br>AT3G46740.1 0.514 1 0.486 0.161 1
Osjap12g02200.1	1<br>Osjap11g02160.1 0.024 1 0.976 0.05 1<br>Osjap12g02200.1 0.001 1 0.999 0.001 1	0	1<br>Orufi11g01900.1 0.002 1 0.998 0.032 0.997	1<br>Oglab11g01770.1 0.002 1 0.998 0.068 1	1<br>Obart11g01960.3 0.107 1 0.893 0.023 1	0	0	0	1<br>Lperr12g01900.1 0.114 0.993 0.879 0.041 0.983	0
	0<br>Osjap06g22240.1 0.479 1 0.521 0.001 0.976	1<br>Osind11g01570.1 0.002 0.331 0.329 0.192 0.912<br>Osind12g02490.1 0.476 1 0.524 0.001 1	1<br>Orufi12g02050.1 0.001 0.357 0.356 0.002 1<br>Orufi12g02070.1 0.476 1 0.524 0.002 1	1<br>Oglab06g17410.1 0.471 0.999 0.528 0.017 0.999<br>Oglab12g01850.1 0.134 0.461 0.327 0.052 0.99	1<br>Obart12g01850.1 0.476 1 0.524 0.001 1<br>Obart12g01860.1 0.118 0.457 0.339 0.077 0.945	1<br>Olong_015795-R1 0.002 0.356 0.354 0.218 0.997<br>Olong_026769-R1 0.476 0.997 0.521 0.001 0.986	0<br>Opunc12g01810.1 0.22 0.999 0.779 0.023 0.999	0<br>Obrac11g01280.1 0.114 0.356 0.242 0.006 0.888<br>Obrac12g01600.1 0.476 0.997 0.521 0.001 0.999	0<br>Lperr11g01750.3 0.107 0.331 0.224 0.076 0.849<br>Lperr11g01770.1 0.476 1 0.524 0.001 1	0<br>AT5G45790.2 0.113 0.338 0.225 0.112 0.891<br>AT5G60900.1 0.497 0.996 0.499 0.043 0.96

Osjap12g12320.1	1<br>Osjap12g12320.1 0.001 1 0.999 0.001 1	1<br>Osind12g09530.1 0.001 1 0.999 0.001 1	1<br>Orufi12g10460.1 0.001 1 0.999 0.001 1	1<br>Oglab12g09470.1 0.001 1 0.999 0.001 1	1<br>Obart12g09410.1 0.001 1 0.999 0.001 1	0	0	0	0	0
	1<br>Osjap01g44780.2 0.363 0.998 0.635 0.086 0.994<br>Osjap03g07600.1 0.001 0.323 0.322 0.004 0.989	1<br>Osind01g46040.1 0.363 0.998 0.635 0.142 0.995<br>Osind03g07670.1 0.001 0.323 0.322 0.004 0.989	1<br>Orufi01g42650.3 0.363 0.998 0.635 0.186 0.995<br>Orufi03g07180.1 0.001 0.323 0.322 0.004 0.989	1<br>Oglab01g34040.1 0.425 0.998 0.573 0.203 0.995<br>Oglab03g07280.1 0.001 0.323 0.322 0.004 0.989	1<br>Obart01g39260.3 0.388 0.998 0.61 0.039 0.994<br>Obart03g07210.1 0.001 0.323 0.322 0.004 0.989	1<br>Olong_021912-R2 0.373 1 0.627 0.008 1<br>Olong_021913-R1 0.001 0.321 0.32 0.004 0.988	1<br>Opunc02g11250.1 0.001 0.323 0.322 0.372 0.993<br>Opunc12g08570.1 0.39 1 0.61 0.211 1	1<br>Obrac03g05880.1 0.001 0.323 0.322 0.004 0.992<br>Obrac12g06500.1 0.395 1 0.605 0.103 1	1<br>Lperr03g06600.1 0.001 0.323 0.322 0.004 0.992<br>Lperr12g08310.1 0.399 0.999 0.6 0.002 0.998	1<br>AT3G49880.1 0.426 0.995 0.569 0.032 0.987<br>AT4G34670.1 0.001 0.315 0.314 0.004 0.966

((((_d1,punctata),brachy),leersia),tair);

chimeric gene id	INGROUP						MIDGROUP	OUTGROUP		
	jap	indica	rufi	glab	barthii	longi	punctata	brachy	leersia	tair
Osjap03g00680.1	1<br>Osjap03g00680.1 0.001 1 0.999 0.001 1<br>Osjap03g00680.2 0.001 1 0.999 0.001 1<br>Osjap03g00680.3 0.001 1 0.999 0.001 1	1<br>Osind03g00740.1 0.001 0.994 0.993 0.001 0.996	1<br>Orufi03g12660.1 0.001 1 0.999 0.001 1<br>Orufi03g12660.2 0.001 1 0.999 0.001 1	1<br>Oglab03g00390.1 0.001 1 0.999 0.001 1	1<br>Obart03g00550.1 0.001 0.994 0.993 0.001 0.996	0	0	0	0	0
	1<br>Osjap02g42090.1 0.051 0.661 0.61 0.065 1<br>Osjap12g08580.1 0.71 0.999 0.289 0.011 0.996	1<br>Osind02g41670.1 0.06 0.661 0.601 0.131 1<br>Osind03g02220.1 0.7 1 0.3 0.015 1	1<br>Orufi02g39350.1 0.06 0.661 0.601 0.131 1<br>Orufi09g02220.2 0.746 1 0.254 0.095 1	1<br>Oglab02g34920.1 0.06 0.661 0.601 0.131 1<br>Oglab03g02050.1 0.688 1 0.312 0.187 1	1<br>Obart02g37790.1 0.06 0.632 0.572 0.133 0.977<br>Obart12g06510.1 0.685 0.999 0.314 0.066 0.997	0<br>Olong_013175-R1 0.264 0.904 0.64 0.002 0.961	1<br>Opunc03g00470.1 0.001 0.783 0.782 0.002 1<br>Opunc11g04590.1 0.783 0.998 0.215 0.055 1	1<br>Obrac02g10260.1 0.712 0.999 0.287 0.265 0.995<br>Obrac02g27970.1 0.134 0.661 0.527 0.105 1	1<br>Lperr03g00570.1 0.001 0.657 0.656 0.002 0.994<br>Lperr04g11360.1 0.752 0.998 0.246 0.415 0.998	0<br>AT5G04460.1 0.017 0.661 0.644 0.081 1
Osjap03g00680.2	1<br>Osjap03g00680.1 0.001 1 0.999 0.001 1<br>Osjap03g00680.2 0.001 1 0.999 0.001 1<br>Osjap03g00680.3 0.001 1 0.999 0.001 1	1<br>Osind03g00740.1 0.001 0.994 0.993 0.001 0.996	1<br>Orufi03g12660.1 0.001 1 0.999 0.001 1<br>Orufi03g12660.2 0.001 1 0.999 0.001 1	1<br>Oglab03g00390.1 0.001 1 0.999 0.001 1	1<br>Obart03g00550.1 0.001 0.994 0.993 0.001 0.996	0	0	0	0	0
	1<br>Osjap02g42090.1 0.052 0.68 0.628 0.065 1<br>Osjap12g08580.1 0.702 0.999 0.297 0.011 0.996	1<br>Osind02g41670.1 0.062 0.68 0.618 0.131 1<br>Osind03g02220.1 0.691 1 0.309 0.015 1	1<br>Orufi02g39350.1 0.062 0.68 0.618 0.131 1<br>Orufi09g02220.2 0.738 1 0.262 0.095 1	1<br>Oglab02g34920.1 0.062 0.68 0.618 0.131 1<br>Oglab03g02050.1 0.691 1 0.309 0.214 1	1<br>Obart02g37790.1 0.062 0.65 0.588 0.133 0.977<br>Obart12g06510.1 0.681 0.999 0.318 0.079 0.997	0<br>Olong_013175-R1 0.272 0.902 0.63 0.002 0.961	1<br>Opunc03g00470.1 0.001 0.777 0.776 0.002 1<br>Opunc03g03130.2 0.777 0.999 0.222 0.443 0.997	1<br>Obrac02g10260.1 0.704 0.999 0.295 0.265 0.995<br>Obrac02g27970.1 0.138 0.68 0.542 0.105 1	1<br>Lperr03g00570.1 0.001 0.68 0.679 0.002 1<br>Lperr04g11360.1 0.745 0.998 0.253 0.415 0.998	0<br>AT5G04460.1 0.018 0.68 0.662 0.081 1
Osjap03g34330.1	1<br>Osjap03g34330.1 0.001 1 0.999 0.001 1	1<br>Osind03g35550.1 0.001 1 0.999 0.039 1	1<br>Orufi03g33060.1 0.001 1 0.999 0.001 1	1<br>Oglab03g30830.1 0.001 1 0.999 0.001 1	1<br>Obart03g31750.1 0.001 1 0.999 0.046 1	0	0	0	0	0
	1<br>Osjap03g39110.1 0.634 1 0.366 0.036 1<br>Osjap10g05370.1 0.012 0.609 0.597 0.1 0.963	1<br>Osind01g46540.1 0.634 0.999 0.365 0.065 0.994<br>Osind10g03770.1 0.012 0.609 0.597 0.11 0.96	1<br>Orufi10g04850.1 0.031 0.609 0.578 0.093 0.963<br>Orufi11g12520.1 0.635 1 0.365 0.038 0.991	1<br>Oglab03g34460.1 0.609 0.997 0.388 0.016 0.993<br>Oglab10g04340.1 0.012 0.609 0.597 0.1 0.963	1<br>Obart03g36200.1 0.634 1 0.366 0.016 0.997<br>Obart10g04600.1 0.014 0.609 0.595 0.106 0.962	1<br>Olong_014796-R1 0.001 0.516 0.515 0.002 0.95<br>Olong_027495-R1 0.609 0.994 0.385 0.005 0.998	1<br>Opunc01g42030.1 0.634 1 0.366 0.28 0.872<br>Opunc03g28940.2 0.001 0.609 0.608 0.002 0.962	1<br>Obrac02g16280.1 0.628 0.981 0.353 0.063 0.949<br>Obrac03g23440.1 0.001 0.609 0.608 0.002 0.964	1<br>Lperr03g26400.1 0.001 0.609 0.608 0.002 0.962<br>Lperr03g30520.1 0.626 1 0.374 0.003 0.997	0<br>AT1G70730.3 0.001 0.609 0.608 0.118 0.967
Osjap05g00530.1	1<br>Osjap05g00530.1 0.001 1 0.999 0.001 1<br>Osjap05g00530.2 0.001 1 0.999 0.001 1<br>Osjap05g00530.3 0.001 1 0.999 0.001 1<br>Osjap05g00530.4 0.001 1 0.999 0.001 1	1<br>Osind05g00510.1 0.131 1 0.869 0.001 1	1<br>Orufi05g00500.1 0.001 1 0.999 0.001 1	0	1<br>Obart05g00520.1 0.084 0.999 0.915 0.001 0.993<br>Obart05g00520.2 0.084 1 0.916 0.001 1	1<br>Olong_022405-R1 0.131 1 0.869 0.001 1	0	0	0	0
	1<br>Osjap02g42390.1 0.422 0.993 0.571 0.001 0.983	1<br>Osind02g41800.1 0.389 0.995 0.606 0.004 0.994	1<br>Orufi02g39480.2 0.389 0.994 0.605 0.004 0.992	1<br>Oglab05g00490.2 0.375 1 0.625 0.099 0.996	1<br>Obart06g01490.1 0.1 0.367 0.267 0.2 0.99	1<br>Olong_023813-R1 0.401 0.997 0.596 0.001 0.976	1<br>Opunc05g00480.1 0.397 0.999 0.602 0.007 0.998	0<br>Obrac05g00480.2 0.062 0.363 0.301 0.093 0.998	1<br>Lperr04g17270.1 0.392 1 0.608 0.002 1	0<br>AT4G37380.1 0.009 0.363 0.354 0.043 0.85

	Osjap07g27300.1 0.068 0.363 0.295 0.072 0.834	Osind06g29470.1 0.058 0.363 0.305 0.1 0.846	Orufi07g24760.1 0.068 0.363 0.295 0.072 0.834	Oglab05g00500.1 0.001 0.365 0.364 0.002 0.982	Obart09g15270.1 0.407 1 0.593 0.029 1	Olong_026414-R1 0.085 0.363 0.278 0.071 0.826	Opunc05g00490.1 0.008 0.367 0.359 0.037 0.986		Lperr07g20540.1 0.052 0.363 0.311 0.079 0.819	
Osjap05g00530.2	1<br>Osjap05g00530.1 0.001 1 0.999 0.001 1<br>Osjap05g00530.2 0.001 1 0.999 0.001 1<br>Osjap05g00530.3 0.001 1 0.999 0.001 1<br>Osjap05g00530.4 0.001 1 0.999 0.001 1	1<br>Osind05g00510.1 0.16 1 0.84 0.001 1	1<br>Orufi05g00500.1 0.001 1 0.999 0.001 1	0	1<br>Obart05g00520.1 0.103 0.999 0.896 0.001 0.993<br>Obart05g00520.2 0.103 1 0.897 0.001 1	1<br>Olong_022405-R1 0.16 1 0.84 0.001 1	0	0	0	0
	1<br>Osjap07g27300.1 0.084 0.445 0.361 0.072 0.834<br>Osjap09g15690.3 0.487 1 0.513 0.003 1	1<br>Osind06g29470.1 0.071 0.446 0.375 0.1 0.846<br>Osind09g15830.1 0.487 1 0.513 0.029 1	1<br>Orufi07g24760.1 0.084 0.445 0.361 0.072 0.834<br>Orufi09g16480.1 0.487 1 0.513 0.029 1	1<br>Oglab05g00500.1 0.001 0.449 0.448 0.002 0.982<br>Oglab09g13820.1 0.486 1 0.514 0.093 1	1<br>Obart06g01490.1 0.123 0.451 0.328 0.2 0.99<br>Obart09g15270.1 0.487 1 0.513 0.029 1	1<br>Olong_020432-R1 0.074 0.445 0.371 0.013 0.62<br>Olong_025239-R1 0.486 1 0.514 0.029 1	1<br>Opunc02g35220.2 0.487 0.993 0.506 0.294 0.984<br>Opunc05g00490.1 0.01 0.45 0.44 0.037 0.986	0<br>Obrac05g00480.2 0.077 0.446 0.369 0.093 0.998	1<br>Lperr05g00500.1 0.486 1 0.514 0.022 0.996<br>Lperr07g20540.1 0.064 0.445 0.381 0.079 0.819	0<br>AT4G37380.1 0.011 0.445 0.434 0.043 0.85
Osjap05g00530.3	1<br>Osjap05g00530.1 0.001 1 0.999 0.001 1<br>Osjap05g00530.2 0.001 1 0.999 0.001 1<br>Osjap05g00530.3 0.001 1 0.999 0.001 1<br>Osjap05g00530.4 0.001 1 0.999 0.001 1	1<br>Osind05g00510.1 0.161 1 0.839 0.001 1	1<br>Orufi05g00500.1 0.001 1 0.999 0.001 1	0	1<br>Obart05g00520.1 0.104 0.999 0.895 0.001 0.993<br>Obart05g00520.2 0.104 1 0.896 0.001 1	1<br>Olong_022405-R1 0.161 1 0.839 0.001 1	0	0	0	0
	1<br>Osjap07g27300.1 0.084 0.46 0.376 0.072 0.873<br>Osjap09g15690.3 0.487 1 0.513 0.003 1	1<br>Osind08g09120.1 0.078 0.456 0.378 0.168 0.613<br>Osind09g15830.1 0.487 1 0.513 0.029 1	1<br>Orufi07g24760.1 0.084 0.46 0.376 0.072 0.873<br>Orufi09g16480.1 0.487 1 0.513 0.029 1	1<br>Oglab05g00500.1 0.001 0.456 0.455 0.002 0.993<br>Oglab09g13820.1 0.487 1 0.513 0.093 1	1<br>Obart06g01490.1 0.123 0.45 0.327 0.2 0.982<br>Obart09g15270.1 0.487 1 0.513 0.029 1	1<br>Olong_020432-R1 0.074 0.481 0.407 0.013 0.666<br>Olong_023813-R1 0.482 0.997 0.515 0.004 0.976	1<br>Opunc02g35220.2 0.485 0.993 0.508 0.294 0.984<br>Opunc05g00490.1 0.01 0.456 0.446 0.037 0.993	0<br>Obrac05g00480.2 0.077 0.448 0.371 0.093 0.998	1<br>Lperr05g00500.1 0.487 1 0.513 0.022 0.996<br>Lperr07g20540.1 0.064 0.46 0.396 0.079 0.857	0<br>AT4G37380.1 0.011 0.447 0.436 0.043 0.85
Osjap05g00530.4	1<br>Osjap05g00530.1 0.001 1 0.999 0.001 1<br>Osjap05g00530.2 0.001 1 0.999 0.001 1<br>Osjap05g00530.3 0.001 1 0.999 0.001 1<br>Osjap05g00530.4 0.001 1 0.999 0.001 1	1<br>Osind05g00510.1 0.142 1 0.858 0.001 1	1<br>Orufi05g00500.1 0.001 1 0.999 0.001 1	0	1<br>Obart05g00520.1 0.091 0.999 0.908 0.001 0.993<br>Obart05g00520.2 0.091 1 0.909 0.001 1	1<br>Olong_022405-R1 0.142 1 0.858 0.001 1	0	0	0	0
	1<br>Osjap07g27300.1 0.074 0.394 0.32 0.072 0.834<br>Osjap09g15690.3 0.507 1 0.493 0.003 1	1<br>Osind06g29470.1 0.063 0.395 0.332 0.1 0.846<br>Osind09g15830.1 0.442 1 0.558 0.029 1	1<br>Orufi07g24760.1 0.074 0.394 0.32 0.072 0.834<br>Orufi09g16480.1 0.442 1 0.558 0.029 1	1<br>Oglab05g00490.2 0.407 1 0.593 0.099 0.996<br>Oglab05g00500.1 0.001 0.397 0.396 0.002 0.982	1<br>Obart02g34000.1 0.092 0.388 0.296 0.188 0.958<br>Obart09g15270.1 0.442 1 0.558 0.029 1	1<br>Olong_018361-R1 0.442 1 0.558 0.002 1<br>Olong_020432-R1 0.066 0.394 0.328 0.013 0.62	1<br>Opunc05g00480.1 0.431 0.999 0.568 0.007 0.998<br>Opunc05g00490.1 0.009 0.398 0.389 0.037 0.986	0<br>Obrac05g00480.2 0.068 0.395 0.327 0.093 0.998	1<br>Lperr04g17270.1 0.439 1 0.561 0.028 1<br>Lperr07g20540.1 0.056 0.394 0.338 0.079 0.819	0<br>AT4G37380.1 0.009 0.394 0.385 0.043 0.85
Osjap05g04120.1	1<br>Osjap05g04120.1 0.001 1 0.999 0.001 1<br>Osjap05g04120.2 0.001 1 0.999 0.001 1	1<br>Osind05g04070.1 0.008 1 0.992 0.001 1<br>Osind05g04070.2 0.008 1 0.992 0.001 1	1<br>Orufi05g03950.1 0.001 1 0.999 0.001 1<br>Orufi05g03950.2 0.001 1 0.999 0.001 1	1<br>Oglab05g03490.1 0.001 1 0.999 0.001 1<br>Oglab05g03490.2 0.001 1 0.999 0.001 1	1<br>Obart05g03830.1 0.001 1 0.999 0.001 1<br>Obart05g03830.2 0.001 1 0.999 0.001 1	1<br>Olong_016739-R1 0.074 1 0.926 0.001 1	1<br>Opunc05g03540.1 0.001 1 0.999 0.001 1<br>Opunc05g03540.2 0.001 1 0.999 0.001 1	1<br>Obrac05g02930.1 0.074 1 0.926 0.001 1	0	0
	1<br>Osjap01g27410.1 0.09 0.723 0.633 0.066 0.996<br>Osjap03g15140.5 0.743 1 0.257 0.061 1	1<br>Osind01g28630.1 0.09 0.723 0.633 0.066 0.996<br>Osind03g15230.1 0.79 1 0.21 0.03 1	1<br>Orufi01g26130.1 0.09 0.723 0.633 0.066 0.996<br>Orufi03g14720.1 0.79 1 0.21 0.03 1	1<br>Oglab01g19490.1 0.09 0.723 0.633 0.066 0.996<br>Oglab03g14060.1 0.79 1 0.21 0.03 1	1<br>Obart01g23310.1 0.09 0.723 0.633 0.066 0.996<br>Obart03g14050.1 0.79 1 0.21 0.03 1	1<br>Olong_003347-R2 0.09 0.723 0.633 0.047 0.996<br>Olong_009943-R1 0.79 1 0.21 0.03 1	1<br>Opunc01g23460.1 0.09 0.723 0.633 0.064 0.996<br>Opunc03g13590.1 0.79 1 0.21 0.002 0.545	1<br>Obrac01g17590.1 0.09 0.723 0.633 0.067 0.996<br>Obrac03g11330.1 0.79 1 0.21 0.03 1	0<br>Lperr05g03460.1 0.001 0.721 0.72 0.001 0.993	1<br>AT2G47580.1 0.775 1 0.225 0.008 1<br>AT3G16630.1 0.003 0.721 0.718 0.031 0.992
Osjap05g04120.2	1<br>Osjap05g04120.1 0.001 1 0.999 0.001 1<br>Osjap05g04120.2 0.001 1 0.999 0.001 1	1<br>Osind05g04070.1 0.008 1 0.992 0.001 1<br>Osind05g04070.2 0.008 1 0.992 0.001 1	1<br>Orufi05g03950.1 0.001 1 0.999 0.001 1<br>Orufi05g03950.2 0.001 1 0.999 0.001 1	1<br>Oglab05g03490.1 0.001 1 0.999 0.001 1<br>Oglab05g03490.2 0.001 1 0.999 0.001 1	1<br>Obart05g03830.1 0.001 1 0.999 0.001 1<br>Obart05g03830.2 0.001 1 0.999 0.001 1	1<br>Olong_016739-R1 0.073 1 0.927 0.001 1	1<br>Opunc05g03540.1 0.001 1 0.999 0.001 1<br>Opunc05g03540.2 0.001 1 0.999 0.001 1	1<br>Obrac05g02930.1 0.073 1 0.927 0.001 1	0	0
	1	1	1	1	1	1	1	1	0	1

	Osjap01g27410.1 0.089 0.726 0.637 0.066 0.996<br>Osjap03g15140.5 0.747 1 0.253 0.061 1	Osind01g28630.1 0.089 0.726 0.637 0.066 0.996<br>Osind03g15230.1 0.792 1 0.208 0.03 1	Orufi01g26130.1 0.089 0.726 0.637 0.066 0.996<br>Orufi03g14720.1 0.792 1 0.208 0.03 1	Oglab01g19490.1 0.089 0.726 0.637 0.066 0.996<br>Oglab03g14060.1 0.792 1 0.208 0.03 1	Obart01g23310.1 0.089 0.726 0.637 0.066 0.996<br>Obart03g14050.1 0.792 1 0.208 0.03 1	Olong_003347-R2 0.089 0.726 0.637 0.047 0.996<br>Olong_009943-R1 0.792 1 0.208 0.03 1	Opunc01g23460.1 0.089 0.726 0.637 0.064 0.996<br>Opunc03g13590.1 0.792 1 0.208 0.002 0.545	Obrac01g17590.1 0.089 0.726 0.637 0.067 0.996<br>Obrac03g11330.1 0.792 1 0.208 0.03 1	Lperr05g03460.1 0.001 0.725 0.724 0.001 0.993	AT2G47580.1 0.778 1 0.222 0.008 1<br>AT3G16630.1 0.003 0.725 0.722 0.031 0.992
Osjap05g28230.1	1<br>Osjap05g28230.1 0.001 1 0.999 0.001 1	1<br>Osind05g28520.1 0.001 1 0.999 0.001 1	1<br>Orufi05g26870.1 0.001 1 0.999 0.001 1	1<br>Oglab05g23160.1 0.001 1 0.999 0.001 1	1<br>Obart05g25070.1 0.001 1 0.999 0.001 1	0	1<br>Opunc05g22640.1 0.001 1 0.999 0.001 1	0	1<br>Lperr01g22990.1 0.001 1 0.999 0.027 1	0
	1<br>Osjap01g31140.1 0.006 0.432 0.426 0.093 0.997<br>Osjap01g31150.1 0.487 0.999 0.512 0.009 0.999	1<br>Osind01g32600.1 0.001 0.431 0.43 0.002 0.995<br>Osind01g32610.1 0.487 0.999 0.512 0.009 0.999	1<br>Orufi01g29880.1 0.001 0.432 0.431 0.075 0.997<br>Orufi01g29890.1 0.487 0.999 0.512 0.009 0.999	1<br>Oglab01g22740.1 0.027 0.432 0.405 0.061 0.996<br>Oglab01g22750.1 0.487 0.999 0.512 0.009 0.999	1<br>Obart01g26870.1 0.005 0.432 0.427 0.117 0.997<br>Obart01g26880.1 0.487 0.999 0.512 0.009 0.999	1<br>Olong_000367-R1 0.1 0.432 0.332 0.002 0.995<br>Olong_000368-R1 0.462 0.999 0.537 0.069 0.999	1<br>Opunc01g26820.1 0.047 0.432 0.385 0.002 0.996<br>Opunc01g26830.1 0.487 1 0.513 0.009 1	1<br>Obrac01g20400.1 0.044 0.432 0.388 0.004 0.996<br>Obrac05g17560.2 0.432 1 0.568 0.109 1	1<br>Lperr05g20850.2 0.491 1 0.509 0.002 1<br>Lperr05g20860.1 0.001 0.432 0.431 0.002 0.996	1<br>AT5G08690.1 0.011 0.431 0.42 0.034 0.995<br>AT5G51600.1 0.487 0.999 0.512 0.008 0.996
Osjap08g06650.1	1<br>Osjap08g06650.1 0.002 1 0.998 0.002 1	1<br>Osind08g06790.1 0.002 1 0.998 0.002 1	1<br>Orufi08g06840.1 0.002 1 0.998 0.002 1	1<br>Oglab08g05640.1 0.002 1 0.998 0.002 1	1<br>Obart08g06070.1 0.002 1 0.998 0.002 1	0	1<br>Opunc08g05850.1 0.047 1 0.953 0.068 1	0	0	0
	1<br>Osjap04g21220.1 0.047 0.275 0.228 0.21 0.936<br>Osjap09g20870.1 0.359 0.984 0.625 0.208 0.966	1<br>Osind04g20250.1 0.047 0.275 0.228 0.198 0.94<br>Osind09g20960.1 0.359 0.984 0.625 0.208 0.966	1<br>Orufi04g19250.2 0.047 0.275 0.228 0.21 0.936<br>Orufi09g21960.1 0.359 0.984 0.625 0.2 0.965	1<br>Oglab02g26360.1 0.348 0.996 0.648 0.01 0.994<br>Oglab04g16180.1 0.047 0.275 0.228 0.198 0.94	1<br>Obart02g28440.1 0.348 0.996 0.648 0.093 0.994<br>Obart04g17770.1 0.047 0.275 0.228 0.198 0.94	1<br>Olong_008030-R1 0.047 0.275 0.228 0.232 0.937<br>Olong_008031-R2 0.277 1 0.723 0.106 1	1<br>Opunc04g15550.1 0.047 0.275 0.228 0.206 0.938<br>Opunc04g18740.1 0.295 0.984 0.689 0.088 0.994	1<br>Obrac08g04780.1 0.277 1 0.723 0.098 1<br>Obrac08g04800.1 0.002 0.275 0.273 0.006 0.943	1<br>Lperr08g05690.1 0.277 1 0.723 0.1 1<br>Lperr08g05700.1 0.002 0.275 0.273 0.006 0.943	1<br>AT1G05310.1 0.33 0.982 0.652 0.183 0.99<br>AT4G30800.1 0.047 0.275 0.228 0.208 0.943
Osjap09g15430.2	1<br>Osjap09g15430.2 0.002 1 0.998 0.002 1<br>Osjap09g15430.3 0.002 1 0.998 0.002 1	1<br>Osind09g15570.1 0.002 1 0.998 0.002 1<br>Osind09g15570.2 0.002 1 0.998 0.002 1	1<br>Orufi09g16160.1 0.002 1 0.998 0.002 1<br>Orufi09g16160.2 0.002 1 0.998 0.002 1	1<br>Oglab09g13510.1 0.002 1 0.998 0.002 1<br>Oglab09g13510.2 0.002 1 0.998 0.002 1<br>Oglab09g13510.3 0.002 1 0.998 0.002 1<br>Oglab09g13510.4 0.002 1 0.998 0.002 1	1<br>Obart09g14980.1 0.002 1 0.998 0.002 1<br>Obart09g14980.2 0.002 1 0.998 0.002 1	0	1<br>Opunc09g13420.1 0.002 1 0.998 0.002 1<br>Opunc09g13420.2 0.002 1 0.998 0.002 1	1<br>Obrac09g09090.1 0.002 1 0.998 0.002 1	0	0
	0<br>Osjap02g06650.1 0.01 0.565 0.555 0.01 0.997	1<br>Osind02g06940.1 0.01 0.565 0.555 0.01 0.997<br>Osind08g23920.1 0.569 0.993 0.424 0.131 0.988	1<br>Orufi04g24220.1 0.014 0.567 0.553 0.165 1<br>Orufi08g22880.1 0.569 0.993 0.424 0.131 0.988	1<br>Oglab08g19240.1 0.569 0.993 0.424 0.143 0.986<br>Oglab12g18680.1 0.002 0.567 0.565 0.003 1	1<br>Obart02g06180.1 0.01 0.565 0.555 0.01 0.997<br>Obart08g20390.1 0.569 0.993 0.424 0.631 0.988	1<br>Olong_008093-R2 0.596 0.991 0.395 0.25 0.996<br>Olong_024186-R1 0.002 0.567 0.565 0.003 1	1<br>Opunc04g20190.1 0.007 0.567 0.56 0.006 1<br>Opunc08g18560.1 0.569 0.993 0.424 0.128 0.969	1<br>Obrac01g09090.1 0.605 0.998 0.393 0.299 0.982<br>Obrac02g04550.3 0.009 0.565 0.556 0.214 0.997	1<br>Lperr04g18350.1 0.002 0.567 0.565 0.021 1<br>Lperr09g11120.1 0.569 1 0.431 0.156 1	1<br>AT1G68540.1 0.567 0.991 0.424 0.112 1<br>AT2G21270.3 0.003 0.565 0.562 0.059 0.997
Osjap09g15430.3	1<br>Osjap09g15430.2 0.002 1 0.998 0.002 1<br>Osjap09g15430.3 0.002 1 0.998 0.002 1	1<br>Osind09g15570.1 0.002 1 0.998 0.002 1<br>Osind09g15570.2 0.002 1 0.998 0.002 1	1<br>Orufi09g16160.1 0.002 1 0.998 0.002 1<br>Orufi09g16160.2 0.002 1 0.998 0.002 1	1<br>Oglab09g13510.1 0.002 1 0.998 0.002 1<br>Oglab09g13510.2 0.002 1 0.998 0.002 1<br>Oglab09g13510.3 0.002 1 0.998 0.002 1<br>Oglab09g13510.4 0.002 1 0.998 0.002 1	1<br>Obart09g14980.1 0.002 1 0.998 0.002 1<br>Obart09g14980.2 0.002 1 0.998 0.002 1	0	1<br>Opunc09g13420.1 0.002 1 0.998 0.002 1<br>Opunc09g13420.2 0.002 1 0.998 0.002 1	1<br>Obrac09g09090.1 0.002 1 0.998 0.002 1	0	0
	0<br>Osjap02g06650.1 0.01 0.54 0.53 0.01 0.997	1<br>Osind02g06940.1 0.01 0.54 0.53 0.01 0.997<br>Osind08g23920.1 0.544 0.993 0.449 0.131 0.988	1<br>Orufi04g24220.1 0.013 0.542 0.529 0.165 1<br>Orufi08g22880.1 0.544 0.993 0.449 0.131 0.988	1<br>Oglab08g19240.1 0.544 0.993 0.449 0.143 0.986<br>Oglab12g18680.1 0.002 0.542 0.54 0.003 1	1<br>Obart02g06180.1 0.01 0.54 0.53 0.01 0.997<br>Obart08g20390.1 0.544 0.993 0.449 0.631 0.988	1<br>Olong_001228-R1 0.548 0.992 0.444 0.03 0.947<br>Olong_024186-R1 0.002 0.542 0.54 0.003 1	1<br>Opunc04g20190.1 0.007 0.542 0.535 0.006 1<br>Opunc08g18560.1 0.544 0.993 0.449 0.128 0.969	1<br>Obrac02g04550.3 0.008 0.54 0.532 0.214 0.997<br>Obrac09g00560.1 0.548 0.998 0.45 0.164 0.966	1<br>Lperr04g18350.1 0.002 0.542 0.54 0.021 1<br>Lperr09g11120.1 0.544 1 0.456 0.156 1	1<br>AT1G68540.1 0.542 0.992 0.45 0.112 1<br>AT2G21270.3 0.003 0.54 0.537 0.059 0.997
Osjap09g20460.1	1<br>Osjap09g20460.1 0.001 1 0.999 0.001 1	1<br>Osind09g20560.1 0.001 0.986 0.985 0.001 0.998	1<br>Orufi09g21510.1 0.001 0.967 0.966 0.001 0.999	1<br>Oglab09g18200.1 0.001 0.999 0.998 0.001 0.999	1<br>Obart09g19980.1 0.062 0.999 0.937 0.165 0.999<br>Obart09g19980.2 0.001 0.999 0.998 0.001 0.999	1<br>Olong_014589-R1 0.108 0.974 0.866 0.018 0.991<br>Olong_014589-R2 0.108 0.974 0.866 0.018 0.991	0	0	0	0
	1<br>Osjap02g21530.2 0.022 0.584 0.562 0.096 0.943<br>Osjap09g20430.1 0.624 1 0.376 0.085 1	1<br>Osind02g21430.2 0.022 0.584 0.562 0.096 0.943<br>Osind09g20550.1 0.624 1 0.376 0.032 1	1<br>Orufi02g19810.2 0.022 0.584 0.562 0.096 0.943<br>Orufi09g21500.1 0.624 1 0.376 0.085 1	1<br>Oglab02g17850.1 0.022 0.584 0.562 0.095 0.914<br>Oglab09g18170.1 0.624 1 0.376 0.079 1	1<br>Obart04g25370.1 0.044 0.592 0.548 0.176 0.967<br>Obart09g19950.1 0.624 1 0.376 0.079 1	1<br>Olong_014116-R1 0.059 0.592 0.533 0.069 0.957<br>Olong_027417-R1 0.626 0.991 0.365 0.135 0.745	1<br>Opunc09g18280.1 0.619 0.998 0.379 0.058 0.951<br>Opunc09g18290.2 0.001 0.592 0.591 0.001 0.97	1<br>Obrac09g13140.3 0.624 0.986 0.362 0.049 0.806<br>Obrac09g13160.3 0.001 0.593 0.592 0.001 0.974	1<br>Lperr09g15890.1 0.623 0.996 0.373 0.048 0.957<br>Lperr09g15920.1 0.001 0.592 0.591 0.001 0.957	0<br>AT1G18160.1 0.02 0.592 0.572 0.064 0.958

Osjap10g04950.1	1<br>Osjap10g04950.1 0.01 1 0.99 0.01 1<br>Osjap10g21210.1 0.01 1 0.99 0.477 1	1<br>Osind10g03420.1 0.01 1 0.99 0.558 1<br>Osind10g17700.1 0.01 1 0.99 0.477 1	1<br>Orufi10g04520.1 0.01 1 0.99 0.01 1<br>Orufi10g19420.1 0.01 1 0.99 0.477 1	1<br>Oglab10g03910.1 0.01 1 0.99 0.01 1	1<br>Obart10g18220.1 0.01 1 0.99 0.392 0.823	1<br>Olong_009744-R1 0.01 1 0.99 0.651 1<br>Olong_009744-R2 0.01 1 0.99 0.641 1	0	0	0	0
	0<br>Osjap07g13660.9 0.361 1 0.639 0.733 0.947	1<br>Osind02g38620.1 0.361 1 0.639 0.835 0.923<br>Osind06g00010.1 0.01 0.361 0.351 0.609 0.978	1<br>Orufi02g36440.1 0.361 1 0.639 0.835 0.923<br>Orufi08g26670.5 0.01 0.351 0.341 0.583 0.749	1<br>Oglab02g32260.1 0.361 1 0.639 0.838 0.924<br>Oglab04g27900.1 0.01 0.33 0.32 0.14 0.208	1<br>Obart02g34980.1 0.361 1 0.639 0.838 0.924<br>Obart04g29850.1 0.01 0.33 0.32 0.131 0.196	1<br>Olong_008835-R2 0.361 1 0.639 0.809 0.91<br>Olong_022843-R2 0.01 0.33 0.32 0.14 0.208	1<br>Opunc02g32260.1 0.361 1 0.639 0.84 0.925<br>Opunc04g27380.2 0.01 0.33 0.32 0.14 0.208	1<br>Obrac02g25650.1 0.381 1 0.619 0.839 0.923<br>Obrac04g21100.1 0.01 0.33 0.32 0.145 0.217	1<br>Lperr02g28770.1 0.381 1 0.619 0.834 0.92<br>Lperr04g25370.2 0.01 0.33 0.32 0.151 0.225	0
Osjap10g14600.1	1<br>Osjap10g14600.1 0.002 1 0.998 0.002 1<br>Osjap10g14600.2 0.002 1 0.998 0.002 1<br>Osjap10g14600.3 0.002 1 0.998 0.002 1<br>Osjap10g14600.4 0.002 1 0.998 0.002 1<br>Osjap10g14600.5 0.002 1 0.998 0.002 1<br>Osjap10g14600.6 0.002 1 0.998 0.002 1	1<br>Osind10g11660.1 0.002 0.995 0.993 0.003 0.958	1<br>Orufi08g13110.1 0.002 0.987 0.985 0.003 0.99	1<br>Oglab10g11970.1 0.002 0.982 0.98 0.007 1	1<br>Obart10g12400.1 0.002 0.98 0.978 0.004 0.996	0	1<br>Opunc10g10650.1 0.002 0.995 0.993 0.003 0.974<br>Opunc10g10650.2 0.002 0.995 0.993 0.003 0.974	0	0	0
	0<br>Osjap08g12520.1 0.277 0.98 0.703 0.226 0.86	1<br>Osind08g14090.1 0.664 0.987 0.323 0.346 0.989<br>Osind10g11740.1 0.16 0.638 0.478 0.002 0.08	1<br>Orufi08g13110.2 0.664 0.987 0.323 0.346 0.989<br>Orufi10g13050.2 0.002 0.658 0.656 0.003 0.627	1<br>Oglab03g12700.1 0.701 0.975 0.274 0.29 0.977<br>Oglab10g11980.2 0.04 0.649 0.609 0.016 0.42	1<br>Obart03g12670.1 0.701 0.975 0.274 0.29 0.977<br>Obart10g12410.2 0.04 0.653 0.613 0.046 0.639	1<br>Olong_005116-R1 0.658 0.975 0.317 0.171 0.982<br>Olong_028277-R1 0.002 0.651 0.649 0.006 0.975	1<br>Opunc10g10640.1 0.04 0.638 0.598 0.049 0.61<br>Opunc12g14410.1 0.7 0.975 0.275 0.253 0.984	1<br>Obrac10g07370.1 0.117 0.638 0.521 0.003 0.101<br>Obrac12g10200.1 0.66 0.975 0.315 0.237 0.985	1<br>Lperr03g19720.1 0.653 0.982 0.329 0.285 1<br>Lperr10g09020.1 0.117 0.638 0.521 0.001 0.059	1<br>AT3G04260.1 0.117 0.638 0.521 0.075 0.193<br>AT3G55330.1 0.653 0.975 0.322 0.2 0.991
Osjap11g00600.1	1<br>Osjap11g00600.1 0.002 1 0.998 0.002 1<br>Osjap12g00550.1 0.002 0.998 0.996 0.002 1	1<br>Osind12g00790.1 0.002 0.998 0.996 0.002 1	1<br>Orufi11g00440.1 0.002 1 0.998 0.002 1	1<br>Oglab11g00570.1 0.106 1 0.894 0.051 1<br>Oglab12g00270.1 0.002 0.801 0.799 0.003 0.995	1<br>Obart11g00510.1 0.106 1 0.894 0.045 1<br>Obart12g00320.1 0.002 0.998 0.996 0.003 1	0	0	0	0	0
	1<br>Osjap08g03600.1 0.748 0.984 0.236 0.201 0.274<br>Osjap10g06830.2 0.087 0.719 0.632 0.265 0.998	1<br>Osind05g03780.1 0.748 0.984 0.236 0.16 0.63<br>Osind10g04960.1 0.063 0.719 0.656 0.31 0.998	1<br>Orufi08g03710.3 0.748 0.984 0.236 0.289 0.394<br>Orufi12g00580.1 0.002 0.703 0.701 0.002 0.881	0<br>Oglab10g05540.1 0.087 0.719 0.632 0.32 0.998	1<br>Obart08g03340.1 0.748 0.984 0.236 0.191 0.268<br>Obart12g18380.1 0.069 0.717 0.648 0.137 0.977	1<br>Olong_026176-R1 0.083 0.717 0.634 0.114 0.997<br>Olong_027703-R1 0.748 0.984 0.236 0.192 0.274	1<br>Opunc08g03130.1 0.748 0.984 0.236 0.206 0.282<br>Opunc12g00560.1 0.002 0.724 0.722 0.003 0.995	1<br>Obrac08g02840.1 0.748 0.984 0.236 0.201 0.274<br>Obrac12g00030.1 0.004 0.624 0.62 0.412 0.991	1<br>Lperr12g00550.1 0.748 0.986 0.238 0.194 0.271<br>Lperr12g00630.2 0.033 0.719 0.686 0.031 0.983	0<br>AT2G40030.1 0.738 0.982 0.244 0.168 0.234
Osjap11g01220.1	1<br>Osjap11g01220.1 0.001 1 0.999 0.001 1<br>Osjap12g01140.1 0.001 1 0.999 0.001 1	1<br>Osind11g00690.1 0.001 0.955 0.954 0.001 0.999<br>Osind12g01290.1 0.001 1 0.999 0.001 1	1<br>Orufi11g01000.1 0.001 1 0.999 0.001 1<br>Orufi12g01130.1 0.001 1 0.999 0.001 1	1<br>Oglab12g00800.1 0.001 1 0.999 0.001 1	1<br>Obart11g01060.1 0.001 1 0.999 0.001 1	0	1<br>Opunc12g00940.1 0.001 1 0.999 0.001 1	0	0	0
	1<br>Osjap03g13100.1 0.489 0.982 0.493 0.097 0.622<br>Osjap07g30390.1 0.009 0.433 0.424 0.013 0.944	1<br>Osind03g12930.1 0.489 0.982 0.493 0.121 0.633<br>Osind07g27070.1 0.007 0.433 0.426 0.006 0.946	1<br>Orufi03g17220.1 0.496 1 0.504 0.027 1<br>Orufi07g27420.1 0.009 0.433 0.424 0.013 0.946	1<br>Oglab03g12120.1 0.489 0.982 0.493 0.121 0.633<br>Oglab07g22980.1 0.007 0.433 0.426 0.007 0.94	1<br>Obart03g12120.1 0.489 0.982 0.493 0.121 0.633<br>Obart12g00790.1 0.001 0.433 0.432 0.002 0.949	1<br>Olong_007715-R1 0.492 1 0.508 0.007 1<br>Olong_008211-R1 0.007 0.433 0.426 0.006 0.946	1<br>Opunc03g11680.1 0.489 1 0.511 0.148 1<br>Opunc11g00920.1 0.001 0.433 0.432 0.142 0.951	1<br>Obrac03g09720.2 0.489 1 0.511 0.142 1<br>Obrac12g00780.1 0.001 0.433 0.432 0.11 0.953	1<br>Lperr03g10720.1 0.489 0.993 0.504 0.148 0.952<br>Lperr11g00920.1 0.001 0.433 0.432 0.002 0.949	1<br>AT2G34940.1 0.001 0.433 0.432 0.002 0.955<br>AT3G46740.1 0.492 1 0.508 0.161 1
Osjap11g21440.1	1<br>Osjap11g21440.1 0.001 1 0.999 0.001 1	1<br>Osind04g04060.1 0.001 0.934 0.933 0.072 0.988	1<br>Orufi11g19860.1 0.003 1 0.997 0.052 1	1<br>Oglab11g16480.1 0.003 1 0.997 0.052 1	1<br>Obart11g18650.1 0.001 1 0.999 0.073 1	1<br>Olong_013913-R1 0.006 0.965 0.959 0.001 0.986	0	0	0	0
	1<br>Osjap03g43210.1 0.717 0.987 0.27 0.278 0.99<br>Osjap10g09150.2 0.008 0.716 0.708 0.047 0.738	1<br>Osind03g44240.1 0.717 0.987 0.27 0.017 0.988<br>Osind10g06650.1 0.008 0.716 0.708 0.008 0.713	1<br>Orufi03g41640.1 0.717 0.987 0.27 0.018 0.987<br>Orufi10g08130.1 0.008 0.716 0.708 0.047 0.737	1<br>Oglab03g37880.1 0.717 0.987 0.27 0.278 0.99<br>Oglab11g20100.1 0.02 0.711 0.691 0.029 0.947	1<br>Obart03g02940.1 0.739 0.986 0.247 0.118 0.932<br>Obart10g07740.1 0.008 0.716 0.708 0.047 0.737	1<br>Olong_001283-R1 0.002 0.674 0.672 0.014 0.844<br>Olong_011979-R1 0.739 0.987 0.248 0.108 0.954	1<br>Opunc03g37060.1 0.717 0.987 0.27 0.151 0.989<br>Opunc05g18050.1 0.047 0.702 0.655 0.06 0.817	1<br>Obrac03g30000.2 0.705 0.979 0.274 0.031 0.937<br>Obrac05g13790.3 0.029 0.702 0.673 0.127 0.872	1<br>Lperr03g33980.1 0.717 0.974 0.257 0.008 0.411<br>Lperr11g13500.1 0.043 0.677 0.634 0.047 0.638	0<br>AT3G12620.1 0.719 0.984 0.265 0.047 0.964
Osjap12g01140.1	1	1	1	1	1	0	1	0	0	0

	Osjap11g01220.1 0.001 1 0.999 0.001 1<br>Osjap12g01140.1 0.001 1 0.999 0.001 1	Osind11g00690.1 0.001 0.953 0.952 0.001 0.999<br>Osind12g01290.1 0.001 1 0.999 0.001 1	Orufi11g01000.1 0.001 1 0.999 0.001 1<br>Orufi12g01130.1 0.001 1 0.999 0.001 1	Oglab12g00800.1 0.001 1 0.999 0.001 1	Obart11g01060.1 0.001 1 0.999 0.001 1		Opunc12g00940.1 0.001 1 0.999 0.001 1			
	1<br>Osjap03g13100.1 0.511 0.981 0.47 0.097 0.622<br>Osjap07g30390.1 0.01 0.373 0.363 0.013 0.798	1<br>Osind03g12930.1 0.511 0.981 0.47 0.121 0.633<br>Osind07g27070.1 0.007 0.377 0.37 0.006 0.783	1<br>Orufi03g17220.1 0.517 1 0.483 0.027 1<br>Orufi07g27420.1 0.01 0.377 0.367 0.013 0.783	1<br>Oglab03g12120.1 0.511 0.981 0.47 0.121 0.633<br>Oglab07g22980.1 0.007 0.373 0.366 0.007 0.783	1<br>Obart03g12120.1 0.511 0.981 0.47 0.121 0.633<br>Obart10g06750.1 0.021 0.409 0.388 0.151 0.885	1<br>Olong_007715-R1 0.514 1 0.486 0.007 1<br>Olong_008211-R1 0.007 0.377 0.37 0.006 0.783	1<br>Opunc03g11680.1 0.511 1 0.489 0.148 1<br>Opunc11g00920.1 0.001 0.377 0.376 0.142 0.816	1<br>Obrac03g09720.2 0.511 1 0.489 0.142 1<br>Obrac10g04150.1 0.02 0.405 0.385 0.036 0.868	1<br>Lperr03g10720.1 0.511 0.993 0.482 0.148 0.952<br>Lperr11g00920.1 0.001 0.377 0.376 0.002 0.792	1<br>AT2G34940.1 0.001 0.377 0.376 0.002 0.793<br>AT3G46740.1 0.514 1 0.486 0.161 1
Osjap12g12320.1	1<br>Osjap12g12320.1 0.001 1 0.999 0.001 1	1<br>Osind12g09530.1 0.001 1 0.999 0.001 1	1<br>Orufi12g10460.1 0.001 1 0.999 0.001 1	1<br>Oglab12g09470.1 0.001 1 0.999 0.001 1	1<br>Obart12g09410.1 0.001 1 0.999 0.001 1	0	0	0	0	0
	1<br>Osjap01g44780.2 0.363 0.998 0.635 0.086 0.994<br>Osjap03g07600.1 0.001 0.323 0.322 0.004 0.989	1<br>Osind01g46040.1 0.363 0.998 0.635 0.142 0.995<br>Osind03g07670.1 0.001 0.323 0.322 0.004 0.989	1<br>Orufi01g42650.3 0.363 0.998 0.635 0.186 0.995<br>Orufi03g07180.1 0.001 0.323 0.322 0.004 0.989	1<br>Oglab01g34040.1 0.425 0.998 0.573 0.203 0.995<br>Oglab03g07280.1 0.001 0.323 0.322 0.004 0.989	1<br>Obart01g39260.3 0.388 0.998 0.61 0.039 0.994<br>Obart03g07210.1 0.001 0.323 0.322 0.004 0.989	1<br>Olong_021912-R2 0.373 1 0.627 0.008 1<br>Olong_021913-R1 0.001 0.321 0.32 0.004 0.988	1<br>Opunc02g11250.1 0.001 0.323 0.322 0.372 0.993<br>Opunc12g08570.1 0.39 1 0.61 0.211 1	1<br>Obrac03g05880.1 0.001 0.323 0.322 0.004 0.992<br>Obrac12g06500.1 0.395 1 0.605 0.103 1	1<br>Lperr03g06600.1 0.001 0.323 0.322 0.004 0.992<br>Lperr12g08310.1 0.399 0.999 0.6 0.002 0.998	1<br>AT3G49880.1 0.426 0.995 0.569 0.032 0.987<br>AT4G34670.1 0.001 0.315 0.314 0.004 0.966

(((((((jap,indica),rufi),(glab,barthii)),longi),punctata),brachy),leersia),tair);

chimeric gene id	INGROUP	MIDGROUP						OUTGROUP		
	indica	jap	rufi	glab	barthii	longi	punctata	brachy	leersia	tair
Osind01g02220.1	1<br>Osind01g02220.1 0.001 1 0.999 0.001 1	0	0	1<br>Oglab01g01590.1 0.056 0.987 0.931 0.001 0.999	1<br>Obart01g01710.1 0.056 0.987 0.931 0.001 0.986	1<br>Olong_021362-R1 0.043 1 0.957 0.072 1	1<br>Opunc01g01490.1 0.056 0.999 0.943 0.056 0.988<br>Opunc01g01490.2 0.043 0.999 0.956 0.042 0.988	0	0	0
	1<br>Osind01g02240.1 0.007 0.498 0.491 0.073 0.999<br>Osind05g03930.1 0.505 1 0.495 0.104 1	0<br>Osjap01g02100.2 0.497 1 0.503 0.017 1	1<br>Orufi01g01810.1 0.503 1 0.497 0.002 1<br>Orufi01g01830.3 0.007 0.499 0.492 0.143 1	1<br>Oglab01g01570.1 0.035 0.499 0.464 0.101 1<br>Oglab05g03370.1 0.531 1 0.469 0.154 1	1<br>Obart01g01680.1 0.035 0.499 0.464 0.11 1<br>Obart05g03720.1 0.505 1 0.495 0.104 1	1<br>Olong_016471-R1 0.505 0.991 0.486 0.106 0.98<br>Olong_021363-R1 0.035 0.499 0.464 0.032 1	1<br>Opunc01g01500.1 0.053 0.498 0.445 0.198 0.998<br>Opunc05g03460.1 0.531 0.997 0.466 0.153 0.99	0<br>Obrac01g17910.1 0.479 1 0.521 0.202 1	1<br>Lperr01g01610.1 0.523 1 0.477 0.058 0.998<br>Lperr01g01640.1 0.023 0.498 0.475 0.157 0.998	1<br>AT1G23010.1 0.056 0.498 0.442 0.134 0.998<br>AT3G48670.1 0.503 1 0.497 0.114 0.995
Osind01g08490.1	1<br>Osind01g08490.1 0.001 1 0.999 0.001 1	1<br>Osjap01g08660.1 0.001 1 0.999 0.001 1	1<br>Orufi01g08070.1 0.001 1 0.999 0.001 1	1<br>Oglab01g06470.1 0.001 1 0.999 0.001 1	1<br>Obart01g07430.1 0.017 1 0.983 0.001 1	1<br>Olong_011567-R1 0.07 1 0.93 0.001 1	0	0	0	0
	1<br>Osind01g30690.1 0.114 0.581 0.467 0.267 0.982<br>Osind05g00540.1 0.647 0.997 0.35 0.126 1	1<br>Osjap01g29230.2 0.114 0.581 0.467 0.25 0.983<br>Osjap05g00560.2 0.647 0.997 0.35 0.146 1	1<br>Orufi01g27950.1 0.114 0.581 0.467 0.25 0.983<br>Orufi01g48560.2 0.647 0.98 0.333 0.085 0.984	1<br>Oglab01g21070.2 0.114 0.581 0.467 0.249 0.983<br>Oglab05g00530.1 0.647 0.997 0.35 0.146 1	1<br>Obart01g25090.1 0.114 0.581 0.467 0.219 0.982<br>Obart05g00540.1 0.647 0.997 0.35 0.146 1	0<br>Olong_003039-R1 0.647 0.997 0.35 0.128 1<br>Olong_003692-R2 0.153 0.581 0.428 0.244 0.979	1<br>Opunc01g07170.1 0.001 0.582 0.581 0.001 0.984<br>Opunc05g00510.2 0.647 0.997 0.35 0.15 1	1<br>Obrac01g05760.3 0.583 1 0.417 0.082 1<br>Obrac01g05770.3 0.001 0.582 0.581 0.001 0.985	1<br>Lperr01g07030.1 0.583 1 0.417 0.018 1<br>Lperr01g07040.2 0.001 0.582 0.581 0.001 0.989	1<br>AT1G54350.1 0.585 0.993 0.408 0.084 0.975<br>AT5G53430.1 0.024 0.581 0.557 0.09 0.984
Osind01g11290.1	1<br>Osind01g11290.1 0.002 1 0.998 0.002 1	0	0	0	0	0	0	0	0	0
	1<br>Osind06g08940.1 0.002 0.525 0.523 0.004 0.846<br>Osind07g16750.1 0.592 0.963 0.371 0.207 0.913	1<br>Osjap01g11200.1 0.667 1 0.333 0.257 0.925<br>Osjap06g08850.1 0.002 0.578 0.576 0.004 0.966	1<br>Orufi02g30900.1 0.002 0.557 0.555 0.004 0.938<br>Orufi03g11370.1 0.658 0.968 0.31 0.211 0.912	1<br>Oglab01g08780.1 0.654 1 0.346 0.173 1<br>Oglab06g07730.1 0.002 0.578 0.576 0.004 0.966	1<br>Obart01g09730.1 0.654 1 0.346 0.173 1<br>Obart06g07830.1 0.002 0.578 0.576 0.004 0.921	1<br>Olong_007345-R1 0.658 0.968 0.31 0.211 0.912<br>Olong_010168-R1 0.002 0.557 0.555 0.004 0.938	1<br>Opunc01g09610.1 0.665 0.998 0.333 0.208 0.995<br>Opunc02g26870.2 0.002 0.557 0.555 0.004 0.93	1<br>Obrac02g21300.2 0.002 0.557 0.555 0.004 0.941<br>Obrac03g08890.1 0.658 0.968 0.31 0.043 0.907	1<br>Lperr01g09310.1 0.002 0.548 0.546 0.004 0.928<br>Lperr07g11580.1 0.654 0.986 0.332 0.252 0.954	0<br>AT5G16830.1 0.002 0.546 0.544 0.004 0.925
Osind01g15670.1	1<br>Osind01g15670.1 0.004 1 0.996 0.004 1	0	0	0	0	0	0	0	0	0
	1<br>Osind01g29920.1 0.027 0.713 0.686 0.016 0.487<br>Osind05g01020.1 0.744 1 0.256 0.653 0.958	1<br>Osjap01g15550.1 0.713 1 0.287 0.027 1<br>Osjap01g15560.1 0.004 0.702 0.698 0.002 0.412	1<br>Orufi01g14840.1 0.004 0.702 0.698 0.002 0.412<br>Orufi02g31040.2 0.744 1 0.256 0.65 0.958	1<br>Oglab01g11340.1 0.713 1 0.287 0.027 1<br>Oglab01g11350.1 0.004 0.702 0.698 0.002 0.412	1<br>Obart01g13510.1 0.713 1 0.287 0.027 1<br>Obart01g24360.1 0.027 0.713 0.686 0.016 0.487	1<br>Olong_004710-R1 0.744 1 0.256 0.667 0.96<br>Olong_009087-R1 0.004 0.702 0.698 0.003 0.477	0<br>Opunc03g32940.1 0.039 0.298 0.259 0.069 0.197<br>Opunc10g08200.1 0.682 1 0.318 0.497 0.954	1<br>Obrac02g21400.1 0.744 1 0.256 0.739 0.969<br>Obrac03g19560.1 0.031 0.709 0.678 0.011 0.435	1<br>Lperr03g21610.1 0.027 0.702 0.675 0.019 0.411<br>Lperr04g25830.1 0.744 1 0.256 0.648 0.958	0<br>AT4G01480.1 0.744 1 0.256 0.653 0.958
Osind01g16020.1	1<br>Osind01g16020.1 0.003 1 0.997 0.003 1	0	0	0	0	0	0	1<br>Obrac06g16200.1 0.093 0.993 0.9 0.018 0.93	0	0
	1<br>Osind01g16070.1 0.021 0.3 0.279 0.002 0.165<br>Osind11g10690.1 0.372 1 0.628 0.005 0.899	1<br>Osjap01g15910.1 0.003 0.3 0.297 0.002 0.173<br>Osjap11g21520.1 0.345 1 0.655 0.014 0.92	1<br>Orufi01g15200.1 0.003 0.3 0.297 0.002 0.173<br>Orufi11g19930.1 0.345 1 0.655 0.015 0.931	1<br>Oglab01g11660.1 0.003 0.3 0.297 0.002 0.173<br>Oglab11g16540.1 0.345 1 0.655 0.035 0.995	1<br>Obart01g13840.1 0.003 0.3 0.297 0.002 0.173<br>Obart11g18730.1 0.345 1 0.655 0.016 0.895	0<br>Olong_022841-R1 0.345 0.993 0.648 0.096 0.994	1<br>Opunc01g13400.1 0.369 0.993 0.624 0.002 0.888<br>Opunc01g13470.1 0.003 0.3 0.297 0.002 0.181	0<br>Obrac06g16190.1 0.269 0.993 0.724 0.018 0.975	1<br>Lperr05g03790.1 0.003 0.3 0.297 0.002 0.181<br>Lperr11g14380.1 0.372 1 0.628 0.068 0.973	0<br>AT1G48030.1 0.003 0.3 0.297 0.002 0.179
Osind01g28070.1	1<br>Osind01g28070.1 0.002 1 0.998 0.002 1	0	1<br>Orufi01g25620.1 0.002 1 0.998 0.002 1	0	0	0	0	0	0	0
	1<br>Osind02g32380.1 0.002 0.246 0.244 0.009 1	1<br>Osjap01g26860.1 0.246 0.964 0.718 0.083 0.987	1<br>Orufi12g02050.1 0.254 0.877 0.623 0.388 0.948	1<br>Oglab11g01770.1 0.246 0.857 0.611 0.213 0.4	1<br>Obart09g10970.1 0.002 0.202 0.2 0.007 0.743	1<br>Olong_007172-R1 0.005 0.246 0.241 0.341 0.684	1<br>Opunc09g15420.1 0.246 1 0.754 0.068 1	1<br>Obrac09g10790.1 0.246 0.968 0.722 0.06 0.843	1<br>Lperr09g13070.1 0.246 0.959 0.713 0.171 0.919	1<br>AT1G77380.1 0.005 0.246 0.241 0.439 0.704

	Osind11g01570.1 0.246 0.893 0.647 0.489 0.955	Osjap08g15630.1 0.002 0.246 0.244 0.007 0.912	Orufi12g05600.1 0.002 0.246 0.244 0.459 0.725	Oglab11g05250.1 0.005 0.246 0.241 0.43 0.731	Obart12g01860.1 0.232 0.973 0.741 0.088 0.703	Olong_015795-R1 0.246 0.877 0.631 0.506 0.958	Opunc11g05440.1 0.005 0.246 0.241 0.431 0.728	Obrac12g03740.1 0.005 0.246 0.241 0.35 0.667	Lperr11g05150.1 0.005 0.246 0.241 0.444 0.715	AT4G33495.1 0.246 0.914 0.668 0.103 0.988
Osind01g44140.1	1<br>Osind01g44140.1 0.001 1 0.999 0.001 1	1<br>Osjap01g42770.1 0.001 1 0.999 0.001 1	1<br>Orufi01g40790.1 0.001 1 0.999 0.001 1<br>Orufi01g40790.2 0.001 1 0.999 0.001 1<br>Orufi01g40790.3 0.001 1 0.999 0.001 1	0	0	1<br>Olong_024830-R1 0.001 1 0.999 0.001 1	0	0	0	0
	1<br>Osind01g08020.1 0.013 0.278 0.265 0.133 0.963<br>Osind03g28340.1 0.412 0.995 0.583 0.127 0.95	1<br>Osjap01g08100.1 0.013 0.278 0.265 0.133 0.963<br>Osjap03g27560.1 0.412 0.995 0.583 0.127 0.95	1<br>Orufi02g20240.3 0.013 0.267 0.254 0.044 0.951<br>Orufi03g26160.3 0.412 0.995 0.583 0.127 0.95	1<br>Oglab01g32220.1 0.001 0.266 0.265 0.004 0.934<br>Oglab01g32230.1 0.363 0.999 0.636 0.002 0.955	1<br>Obart01g37410.1 0.001 0.266 0.265 0.004 0.934<br>Obart01g37420.1 0.363 1 0.637 0.002 1	1<br>Olong_010570-R1 0.412 0.995 0.583 0.115 0.915<br>Olong_011675-R1 0.013 0.266 0.253 0.517 0.972	1<br>Opunc01g36330.1 0.001 0.266 0.265 0.005 0.975<br>Opunc01g36340.1 0.363 0.999 0.636 0.002 0.969	1<br>Obrac01g28830.1 0.001 0.264 0.263 0.004 0.978<br>Obrac01g28840.1 0.365 1 0.635 0.002 1	1<br>Lperr01g32130.1 0.001 0.266 0.265 0.005 0.977<br>Lperr01g32140.1 0.363 1 0.637 0.002 1	0<br>AT2G37970.1 0.001 0.266 0.265 0.049 0.978<br>AT3G54090.1 0.475 1 0.525 0.123 1
Osind01g52750.1	1<br>Osind01g52750.1 0.002 1 0.998 0.002 1	1<br>Osjap01g51220.1 0.002 1 0.998 0.071 1	1<br>Orufi01g48620.1 0.043 1 0.957 0.124 1	0	0	0	0	0	0	0
	1<br>Osind02g41730.1 0.002 0.698 0.696 0.362 0.927<br>Osind05g13530.1 0.797 1 0.203 0.16 1	1<br>Osjap02g42170.1 0.002 0.698 0.696 0.362 0.927<br>Osjap05g12720.1 0.797 1 0.203 0.16 1	1<br>Orufi02g39420.1 0.002 0.698 0.696 0.362 0.927<br>Orufi05g12160.1 0.797 1 0.203 0.16 1	1<br>Oglab02g35020.1 0.002 0.698 0.696 0.352 0.901<br>Oglab05g10250.1 0.797 1 0.203 0.16 1	1<br>Obart02g37850.1 0.002 0.698 0.696 0.33 0.933<br>Obart05g11250.1 0.797 1 0.203 0.16 1	1<br>Olong_006258-R1 0.002 0.698 0.696 0.246 0.913<br>Olong_022231-R1 0.797 1 0.203 0.16 1	1<br>Opunc02g35080.1 0.002 0.698 0.696 0.366 0.926<br>Opunc05g09730.1 0.797 1 0.203 0.594 1	1<br>Obrac01g35490.1 0.797 1 0.203 0.395 1<br>Obrac02g28040.1 0.002 0.698 0.696 0.366 0.928	0<br>Lperr01g39540.1 0.797 1 0.203 0.152 1<br>Lperr02g31330.1 0.005 0.538 0.533 0.364 0.762	0<br>AT5G24900.1 0.029 0.534 0.505 0.408 0.785
Osind01g52800.1	1<br>Osind01g52800.1 0.002 1 0.998 0.002 1	0	0	0	0	0	0	0	0	0
	1<br>Osind01g52820.1 0.576 1 0.424 0.258 1<br>Osind04g34200.1 0.039 0.555 0.516 0.129 0.646	1<br>Osjap01g51320.2 0.002 0.577 0.575 0.002 0.661<br>Osjap01g51330.1 0.579 1 0.421 0.278 1	1<br>Orufi01g48670.1 0.002 0.577 0.575 0.002 0.661<br>Orufi05g26820.1 0.577 0.979 0.402 0.466 0.934	1<br>Oglab01g39850.1 0.002 0.577 0.575 0.002 0.661<br>Oglab05g23130.1 0.577 0.979 0.402 0.473 0.935	1<br>Obart01g45190.1 0.002 0.577 0.575 0.002 0.661<br>Obart11g03300.1 0.579 0.977 0.398 0.316 0.966	0<br>Olong_011545-R1 0.576 0.981 0.405 0.25 0.96<br>Olong_013976-R1 0.306 0.555 0.249 0.072 0.456	1<br>Opunc01g44160.1 0.597 1 0.403 0.206 1<br>Opunc01g44180.1 0.032 0.577 0.545 0.03 0.649	1<br>Obrac01g35530.1 0.576 1 0.424 0.337 1<br>Obrac04g21950.1 0.039 0.495 0.456 0.616 0.832	1<br>Lperr01g39620.1 0.576 1 0.424 0.14 1<br>Lperr04g26250.1 0.014 0.555 0.541 0.153 0.937	1<br>AT2G32230.1 0.004 0.555 0.551 0.124 0.673<br>AT4G27400.1 0.581 0.988 0.407 0.243 0.915
Osind02g01750.1	1<br>Osind02g01750.1 0.004 1 0.996 0.004 1	0	0	0	0	0	0	0	0	0
	1<br>Osind02g01760.1 0.013 0.239 0.226 0.624 0.686<br>Osind02g01770.1 0.307 1 0.693 0.348 1	1<br>Osjap02g01510.1 0.013 0.239 0.226 0.634 0.694<br>Osjap02g01520.1 0.307 1 0.693 0.342 1	1<br>Orufi02g01500.1 0.013 0.239 0.226 0.634 0.694<br>Orufi02g01510.1 0.307 1 0.693 0.348 1	1<br>Oglab02g01440.1 0.307 1 0.693 0.348 1<br>Oglab03g38410.1 0.013 0.218 0.205 0.612 0.671	1<br>Obart02g01520.1 0.013 0.239 0.226 0.611 0.674<br>Obart02g01530.1 0.307 1 0.693 0.335 1	1<br>Olong_000304-R1 0.307 0.945 0.638 0.367 1<br>Olong_012152-R1 0.013 0.218 0.205 0.595 0.664	1<br>Opunc02g00970.1 0.013 0.227 0.214 0.912 0.995<br>Opunc02g00990.1 0.307 1 0.693 0.348 1	1<br>Obrac02g01150.2 0.307 0.983 0.676 0.435 1<br>Obrac03g30480.1 0.013 0.218 0.205 0.617 0.675	1<br>Lperr02g01300.1 0.307 1 0.693 0.748 1<br>Lperr07g08320.1 0.013 0.218 0.205 0.657 0.713	1<br>AT1G63855.3 0.307 0.962 0.655 0.327 0.985<br>AT4G11840.1 0.017 0.223 0.206 0.62 0.677
Osind02g26640.2	1<br>Osind02g26640.2 0.001 1 0.999 0.001 1	1<br>Osjap02g26750.1 0.001 0.953 0.952 0.001 0.993<br>Osjap02g26750.2 0.001 0.953 0.952 0.001 0.993<br>Osjap02g26750.4 0.001 0.953 0.952 0.001 0.799	1<br>Orufi02g24900.1 0.01 0.958 0.948 0.06 0.859<br>Orufi02g24900.3 0.001 0.958 0.957 0.001 0.74<br>Orufi02g24900.4 0.001 0.958 0.957 0.001 0.737	1<br>Oglab02g22130.2 0.001 1 0.999 0.001 1	1<br>Obart02g23460.2 0.001 1 0.999 0.001 1	0	1<br>Opunc02g21410.1 0.01 0.975 0.965 0.116 0.996<br>Opunc02g21410.2 0.01 0.975 0.965 0.114 0.996<br>Opunc02g21410.3 0.01 0.975 0.965 0.115 0.996<br>Opunc02g21410.4 0.01 0.975 0.965 0.114 0.996	0	0	0
	1<br>Osind02g26450.1 0.497 0.986 0.489 0.023 0.992<br>Osind02g26620.1 0.01 0.413 0.403 0.495 0.978	1<br>Osjap02g26590.1 0.497 0.986 0.489 0.033 0.996<br>Osjap02g26750.5 0.01 0.413 0.403 0.238 0.968	1<br>Orufi02g24730.1 0.497 0.986 0.489 0.031 0.996<br>Orufi02g24880.2 0.01 0.413 0.403 0.18 0.964	1<br>Oglab02g21930.1 0.497 1 0.503 0.047 1<br>Oglab02g22110.2 0.01 0.413 0.403 0.532 0.976	1<br>Obart02g23290.1 0.497 1 0.503 0.029 0.997<br>Obart02g23440.1 0.01 0.413 0.403 0.492 0.978	0<br>Olong_012377-R1 0.651 0.957 0.306 0.444 0.915<br>Olong_024345-R1 0.01 0.434 0.424 0.442 0.991	1<br>Opunc02g21230.1 0.504 0.96 0.456 0.003 0.934<br>Opunc02g21240.1 0.01 0.422 0.412 0.121 0.972	1<br>Obrac02g16670.1 0.503 0.957 0.454 0.005 0.995<br>Obrac02g16700.1 0.01 0.422 0.412 0.114 0.947	1<br>Lperr02g18740.1 0.512 0.975 0.463 0.059 0.982<br>Lperr02g19120.1 0.01 0.443 0.433 0.183 0.978	0<br>AT1G47230.2 0.577 0.953 0.376 0.095 0.857<br>AT4G31860.1 0.013 0.411 0.398 0.123 0.989
Osind02g37690.1	1<br>Osind02g37690.1 0.001 1 0.999 0.001 1	0	0	0	0	0	1<br>Opunc02g31410.1 0.001 0.999 0.998 0.001 0.999	1<br>Obrac02g24890.1 0.001 0.997 0.996 0.001 0.997	0	0

	1<br>Osind06g02810.1 0.001 0.32 0.319 0.004 0.992<br>Osind10g08130.1 0.366 0.971 0.605 0.098 0.966	1<br>Osjap02g38120.1 0.321 1 0.679 0.017 1<br>Osjap02g38130.1 0.001 0.32 0.319 0.004 0.992	1<br>Orufi02g35540.1 0.321 1 0.679 0.017 1<br>Orufi06g02530.1 0.001 0.32 0.319 0.004 0.992	1<br>Oglab02g31390.1 0.321 1 0.679 0.017 1<br>Oglab06g02680.1 0.001 0.32 0.319 0.004 0.992	1<br>Obart02g34000.1 0.321 1 0.679 0.017 1<br>Obart06g02500.1 0.001 0.32 0.319 0.004 0.992	1<br>Olong_016336-R1 0.388 1 0.612 0.002 1<br>Olong_016337-R1 0.064 0.32 0.256 0.006 0.989	1<br>Opunc06g02370.1 0.001 0.32 0.319 0.004 0.992<br>Opunc07g22210.1 0.391 0.987 0.596 0.227 0.851	1<br>Obrac01g03710.2 0.385 0.987 0.602 0.199 0.936<br>Obrac06g01860.1 0.001 0.32 0.319 0.004 0.992	1<br>Lperr02g27870.1 0.355 0.999 0.644 0.002 0.998<br>Lperr06g02230.1 0.001 0.32 0.319 0.004 0.992	1<br>AT4G31300.2 0.029 0.32 0.291 0.038 0.991<br>AT4G37380.1 0.349 0.987 0.638 0.112 0.856
Osind02g38400.1	1<br>Osind02g38400.1 0.002 1 0.998 0.002 1	0	0	1<br>Oglab02g32090.1 0.002 0.989 0.987 0.438 0.998	0	0	0	0	0	0
	1<br>Osind02g38410.1 0.629 0.98 0.351 0.634 0.882<br>Osind09g11760.1 0.002 0.585 0.583 0.004 0.722	1<br>Osjap02g38830.1 0.629 0.982 0.353 0.603 0.875<br>Osjap02g38850.1 0.002 0.585 0.583 0.072 0.739	1<br>Orufi02g36250.1 0.629 0.982 0.353 0.614 0.902<br>Orufi09g12240.1 0.004 0.585 0.581 0.004 0.994	1<br>Oglab02g32090.2 0.633 0.983 0.35 0.135 0.763<br>Oglab09g10440.1 0.002 0.585 0.583 0.005 0.888	1<br>Obart02g34800.1 0.629 0.989 0.36 0.634 0.889<br>Obart09g11450.1 0.002 0.585 0.583 0.007 0.828	1<br>Olong_006227-R1 0.629 0.993 0.364 0.138 0.868<br>Olong_012584-R1 0.029 0.591 0.562 0.003 0.963	0<br>Opunc07g14160.1 0.004 0.672 0.668 0.007 0.898	1<br>Obrac02g25440.1 0.629 0.974 0.345 0.594 0.855<br>Obrac09g07580.1 0.028 0.589 0.561 0.028 0.972	1<br>Lperr02g28600.1 0.629 0.976 0.347 0.633 0.88<br>Lperr03g25050.1 0.013 0.583 0.57 0.005 0.787	1<br>AT1G47480.1 0.024 0.594 0.57 0.013 0.978<br>AT3G14067.1 0.629 0.983 0.354 0.616 0.87
Osind02g39460.2	1<br>Osind02g39460.1 0.001 1 0.999 0.001 1<br>Osind02g39460.2 0.001 1 0.999 0.001 1	0	1<br>Orufi02g37290.1 0.028 0.883 0.855 0.031 1	0	0	0	0	0	0	0
	1<br>Osind01g00350.1 0.613 0.883 0.27 0.037 1<br>Osind09g08880.1 0.02 0.567 0.547 0.201 0.99	1<br>Osjap01g00320.1 0.613 0.883 0.27 0.256 1<br>Osjap02g39920.1 0.001 0.572 0.571 0.001 0.971	1<br>Orufi01g00280.2 0.613 0.883 0.27 0.039 1<br>Orufi02g37310.1 0.001 0.572 0.571 0.002 0.995	1<br>Oglab02g32990.1 0.612 1 0.388 0.217 1<br>Oglab02g33000.1 0.001 0.606 0.605 0.001 0.986	1<br>Obart02g35820.1 0.612 1 0.388 0.223 1<br>Obart02g35830.2 0.001 0.606 0.605 0.001 0.985	1<br>Olong_003837-R1 0.614 0.883 0.269 0.003 1<br>Olong_003838-R2 0.001 0.572 0.571 0.014 1	1<br>Opunc01g00290.1 0.614 0.883 0.269 0.003 1<br>Opunc02g33020.1 0.013 0.595 0.582 0.032 0.997	1<br>Obrac01g00240.4 0.609 0.883 0.274 0.056 1<br>Obrac02g26250.1 0.015 0.573 0.558 0.059 0.992	1<br>Lperr01g00240.1 0.574 0.883 0.309 0.039 1<br>Lperr02g29510.1 0.001 0.572 0.571 0.034 1	1<br>AT3G54360.1 0.614 0.887 0.273 0.064 1<br>AT4G23370.1 0.006 0.567 0.561 0.179 0.996
Osind02g41950.1	1<br>Osind02g41950.1 0.001 1 0.999 0.001 1	0	0	0	0	0	0	0	0	0
	1<br>Osind04g22000.1 0.001 0.376 0.375 0.002 0.658<br>Osind05g11850.1 0.436 0.951 0.515 0.182 0.977	1<br>Osjap02g42560.1 0.463 0.994 0.531 0.205 0.995<br>Osjap04g22950.1 0.001 0.376 0.375 0.002 0.677	1<br>Orufi02g39760.2 0.368 0.929 0.561 0.068 0.952<br>Orufi04g20860.3 0.001 0.368 0.367 0.003 0.78	0<br>Oglab02g35180.1 0.368 1 0.632 0.067 1	1<br>Obart02g38050.1 0.368 1 0.632 0.066 1<br>Obart04g19330.2 0.117 0.362 0.245 0.062 0.759	1<br>Olong_002168-R1 0.001 0.376 0.375 0.002 0.653<br>Olong_020395-R1 0.441 0.94 0.499 0.046 0.685	1<br>Opunc03g04560.1 0.449 0.939 0.49 0.101 0.927<br>Opunc04g17040.1 0.001 0.393 0.392 0.003 0.877	0<br>Obrac02g28220.1 0.368 0.929 0.561 0.074 0.718	1<br>Lperr04g15670.1 0.001 0.369 0.368 0.003 0.747<br>Lperr07g13480.1 0.45 0.956 0.506 0.004 0.975	1<br>AT1G17940.1 0.001 0.369 0.368 0.002 0.721<br>AT3G22470.1 0.424 0.93 0.506 0.06 0.982
Osind02g42070.1	1<br>Osind02g42070.1 0.001 1 0.999 0.001 1	0	0	0	0	0	0	0	0	0
	1<br>Osind01g47650.1 0.299 0.989 0.69 0.21 0.991<br>Osind03g17680.1 0.005 0.265 0.26 0.583 0.992	1<br>Osjap02g42770.1 0.267 1 0.733 0.233 1<br>Osjap03g17490.1 0.005 0.265 0.26 0.666 0.994	1<br>Orufi02g39950.1 0.267 1 0.733 0.233 1<br>Orufi03g16810.1 0.005 0.265 0.26 0.666 0.994	1<br>Oglab02g35360.1 0.267 1 0.733 0.233 1<br>Oglab03g16010.1 0.005 0.265 0.26 0.666 0.994	1<br>Obart02g38230.1 0.267 1 0.733 0.233 1<br>Obart03g16270.2 0.005 0.265 0.26 0.666 0.994	1<br>Olong_004203-R1 0.267 1 0.733 0.226 1<br>Olong_013276-R1 0.005 0.265 0.26 0.666 0.994	1<br>Opunc02g35490.1 0.267 1 0.733 0.216 1<br>Opunc03g15730.1 0.007 0.265 0.258 0.669 0.994	0<br>Obrac06g17670.3 0.216 0.991 0.775 0.127 0.962	1<br>Lperr02g31700.1 0.267 0.999 0.732 0.222 1<br>Lperr03g14180.1 0.005 0.257 0.252 0.666 0.986	1<br>AT5G10540.1 0.267 0.999 0.732 0.136 1<br>AT5G67200.1 0.02 0.251 0.231 0.725 0.994
Osind03g00740.1	1<br>Osind03g00740.1 0.001 1 0.999 0.001 1	1<br>Osjap03g00680.1 0.001 0.996 0.995 0.001 0.994<br>Osjap03g00680.2 0.001 0.996 0.995 0.001 0.994<br>Osjap03g00680.3 0.001 0.996 0.995 0.001 0.994	1<br>Orufi03g12660.1 0.001 0.996 0.995 0.001 0.994<br>Orufi03g12660.2 0.001 0.996 0.995 0.001 0.994	1<br>Oglab03g00390.1 0.001 0.996 0.995 0.001 0.994	1<br>Obart03g00550.1 0.001 1 0.999 0.001 1	0	0	0	0	0
	1<br>Osind02g41670.1 0.062 0.678 0.616 0.131 1<br>Osind03g02220.1 0.698 0.996 0.298 0.015 0.982	1<br>Osjap02g42090.1 0.052 0.678 0.626 0.065 1<br>Osjap12g08580.1 0.708 0.996 0.288 0.011 0.977	1<br>Orufi02g39350.1 0.062 0.678 0.616 0.131 1<br>Orufi09g03230.3 0.732 0.99 0.258 0.053 0.963	1<br>Oglab02g34920.1 0.062 0.678 0.616 0.131 1<br>Oglab03g02050.1 0.698 0.996 0.298 0.214 0.986	1<br>Obart02g37790.1 0.062 0.648 0.586 0.133 0.977<br>Obart12g06510.1 0.676 0.996 0.32 0.052 0.984	0<br>Olong_013175-R1 0.271 0.904 0.633 0.002 0.961	1<br>Opunc03g00470.1 0.001 0.78 0.779 0.002 1<br>Opunc11g04590.1 0.78 0.996 0.216 0.055 0.982	0<br>Obrac02g27970.1 0.137 0.678 0.541 0.105 1<br>Obrac05g17660.1 0.743 0.995 0.252 0.425 0.997	1<br>Lperr03g00570.1 0.001 0.678 0.677 0.002 1<br>Lperr04g11360.1 0.747 0.996 0.249 0.415 0.99	0<br>AT5G04460.1 0.018 0.678 0.66 0.081 1
Osind03g03640.1	1<br>Osind03g03640.1 0.001 1 0.999 0.001 1	0	0	0	0	1<br>Olong_017738-R1 0.001 1 0.999 0.002 1	0	0	0	0

	1<br>Osind04g03980.1 0.002 0.276 0.274 0.012 0.944<br>Osind08g08840.1 0.287 0.98 0.693 0.001 0.846	1<br>Osjap11g21530.1 0.001 0.279 0.278 0.001 0.971<br>Osjap11g22580.1 0.287 1 0.713 0.001 1	1<br>Orufi02g37700.1 0.001 0.276 0.275 0.002 0.946<br>Orufi11g20920.1 0.287 1 0.713 0.001 1	0<br>Oglab11g16610.1 0.001 0.279 0.278 0.002 0.957	1<br>Obart11g19590.1 0.758 1 0.242 0.003 1<br>Obart11g19610.2 0.287 0.744 0.457 0.002 0.979<br>Obart11g19620.1 0.001 0.276 0.275 0.482 0.972	1<br>Olong_021140-R1 0.001 0.279 0.278 0.002 0.957<br>Olong_026633-R1 0.287 0.984 0.697 0.001 0.867	1<br>Opunc01g13480.1 0.003 0.279 0.276 0.039 0.959<br>Opunc11g16180.1 0.287 1 0.713 0.001 1	1<br>Obrac06g16200.1 0.001 0.279 0.278 0.009 0.902<br>Obrac11g13540.1 0.287 0.99 0.703 0.001 0.908	1<br>Lperr11g15180.1 0.004 0.275 0.271 0.033 0.94<br>Lperr11g15890.1 0.28 1 0.72 0.042 1	0<br>AT3G46710.1 0.292 0.723 0.431 0.001 0.362<br>AT4G17565.1 0.004 0.262 0.258 0.048 0.915
Osind03g07990.1	1<br>Osind03g07990.1 0.005 1 0.995 0.005 1	0	0	0	0	0	0	0	0	0
	1<br>Osind03g07970.1 0.015 0.332 0.317 0.015 0.471<br>Osind03g43640.1 0.347 0.96 0.613 0.326 0.984	0<br>Osjap03g07930.1 0.266 1 0.734 0.081 1	1<br>Orufi03g07540.1 0.005 0.302 0.297 0.008 0.492<br>Orufi03g41020.1 0.337 0.96 0.623 0.332 0.985	1<br>Oglab03g07540.1 0.03 0.332 0.302 0.057 0.604<br>Oglab11g03980.1 0.357 1 0.643 0.228 1	1<br>Obart03g07440.1 0.03 0.332 0.302 0.057 0.604<br>Obart03g39270.1 0.347 0.96 0.613 0.327 0.984	1<br>Olong_003429-R1 0.302 0.95 0.648 0.251 0.837<br>Olong_008051-R1 0.03 0.296 0.266 0.054 0.505	1<br>Opunc03g07260.1 0.005 0.302 0.297 0.012 0.714<br>Opunc03g36380.1 0.337 1 0.663 0.332 1	0<br>Obrac03g22010.1 0.452 0.869 0.417 0.87 0.993	0<br>Lperr03g33440.1 0.347 0.985 0.638 0.329 0.998	0<br>AT3G53960.1 0.342 0.975 0.633 0.332 0.987
Osind03g12930.1	1<br>Osind03g12930.1 0.001 1 0.999 0.001 1	1<br>Osjap03g13100.1 0.028 1 0.972 0.001 1	0	1<br>Oglab03g12120.1 0.001 1 0.999 0.001 1	1<br>Obart03g12120.1 0.001 1 0.999 0.001 1	0	0	0	0	0
	1<br>Osind06g01770.1 0.642 0.998 0.356 0.212 0.912<br>Osind12g01290.1 0.109 0.633 0.524 0.49 0.981	1<br>Osjap04g18730.1 0.64 0.998 0.358 0.015 0.863<br>Osjap12g01140.1 0.109 0.633 0.524 0.498 0.981	1<br>Orufi06g01460.3 0.642 0.998 0.356 0.212 0.912<br>Orufi12g01130.1 0.109 0.633 0.524 0.498 0.981	1<br>Oglab06g01620.1 0.642 0.998 0.356 0.233 0.912<br>Oglab12g00800.1 0.114 0.633 0.519 0.483 0.983	1<br>Obart06g01490.1 0.642 0.998 0.356 0.233 0.912<br>Obart11g01060.1 0.114 0.633 0.519 0.471 0.982	1<br>Olong_007714-R9 0.642 1 0.358 0.002 1<br>Olong_007715-R1 0.125 0.633 0.508 0.007 0.965	1<br>Opunc01g28650.1 0.646 0.998 0.352 0.094 0.923<br>Opunc03g11680.1 0.028 0.633 0.605 0.001 0.971	1<br>Obrac03g09720.1 0.025 0.633 0.608 0.036 0.971<br>Obrac04g09430.1 0.64 0.998 0.358 0.014 0.863	1<br>Lperr03g10720.1 0.028 0.633 0.605 0.001 0.934<br>Lperr03g17470.1 0.633 0.998 0.365 0.119 0.828	1<br>AT3G46740.1 0.122 0.633 0.511 0.158 0.971<br>AT3G46790.1 0.648 0.998 0.35 0.068 0.807
Osind03g16830.1	1<br>Osind03g16830.1 0.001 1 0.999 0.001 1	0	0	0	0	0	0	0	0	0
	1<br>Osind04g16400.1 0.715 0.999 0.284 0.01 0.997<br>Osind05g11190.1 0.001 0.67 0.669 0.001 0.988	1<br>Osjap03g16610.1 0.677 1 0.323 0.017 1<br>Osjap03g16620.1 0.001 0.677 0.676 0.001 1	1<br>Orufi03g16050.1 0.677 1 0.323 0.017 1<br>Orufi03g16060.1 0.001 0.677 0.676 0.001 1	1<br>Oglab03g15290.1 0.677 1 0.323 0.017 1<br>Oglab03g15300.1 0.001 0.677 0.676 0.116 1	1<br>Obart03g15440.1 0.677 1 0.323 0.017 1<br>Obart03g15450.2 0.001 0.677 0.676 0.112 1	1<br>Olong_001740-R1 0.713 1 0.287 0.003 1<br>Olong_001741-R1 0.001 0.677 0.676 0.001 1	1<br>Opunc03g15010.1 0.679 1 0.321 0.043 1<br>Opunc03g15020.3 0.001 0.677 0.676 0.165 1	1<br>Obrac03g12410.2 0.001 0.679 0.678 0.001 0.983<br>Obrac05g14250.4 0.704 0.998 0.294 0.206 0.996	1<br>Lperr03g13610.1 0.677 1 0.323 0.017 1<br>Lperr03g13620.2 0.001 0.677 0.676 0.014 1	1<br>AT1G22200.1 0.713 0.999 0.286 0.005 0.997<br>AT2G27090.1 0.001 0.672 0.671 0.001 0.989
Osind03g30900.1	1<br>Osind03g30900.1 0.001 1 0.999 0.001 1	0	0	0	0	0	0	0	0	0
	1<br>Osind04g17090.1 0.366 0.979 0.613 0.031 0.767<br>Osind12g19850.1 0.008 0.354 0.346 0.05 0.981	1<br>Osjap03g29750.2 0.001 0.355 0.354 0.036 0.996<br>Osjap04g18300.1 0.366 0.979 0.613 0.031 0.767	1<br>Orufi03g28480.1 0.33 1 0.67 0.036 1<br>Orufi12g21010.5 0.008 0.33 0.322 0.041 0.901	1<br>Oglab03g26970.1 0.003 0.355 0.352 0.007 0.444<br>Oglab03g34550.1 0.392 0.995 0.603 0.045 0.838	1<br>Obart03g27290.1 0.001 0.331 0.33 0.001 0.291<br>Obart03g36270.1 0.392 0.995 0.603 0.045 0.838	1<br>Olong_016768-R1 0.075 0.303 0.228 0.15 1<br>Olong_022955-R1 0.33 1 0.67 0.036 1	1<br>Opunc03g24970.1 0.33 1 0.67 0.038 1<br>Opunc12g17030.1 0.008 0.33 0.322 0.105 0.917	1<br>Obrac11g03750.1 0.354 1 0.646 0.084 1<br>Obrac12g12300.1 0.001 0.337 0.336 0.061 0.968	1<br>Lperr03g22630.2 0.005 0.331 0.326 0.022 0.964<br>Lperr03g22680.1 0.332 1 0.668 0.01 1	1<br>AT1G30300.1 0.006 0.35 0.344 0.062 0.994<br>AT4G18520.1 0.355 0.98 0.625 0.047 0.974
Osind03g31500.1	1<br>Osind03g31500.1 0.001 1 0.999 0.001 1	1<br>Osjap03g30460.1 0.025 1 0.975 0.022 1	1<br>Orufi03g29110.1 0.003 1 0.997 0.276 1	0	1<br>Obart03g27980.1 0.089 1 0.911 0.016 1<br>Obart03g27980.2 0.089 1 0.911 0.015 1	0	0	0	0	0
	1<br>Osind08g14630.1 0.557 0.999 0.442 0.069 0.96<br>Osind12g20440.1 0.092 0.528 0.436 0.01 0.99	1<br>Osjap03g30380.1 0.557 0.999 0.442 0.059 0.866<br>Osjap12g24400.2 0.092 0.528 0.436 0.012 0.988	1<br>Orufi03g29970.1 0.557 0.999 0.442 0.035 0.998<br>Orufi12g21550.1 0.092 0.528 0.436 0.01 0.99	1<br>Oglab03g27330.1 0.557 1 0.443 0.036 1<br>Oglab12g17460.4 0.092 0.528 0.436 0.012 0.996	1<br>Obart03g27900.1 0.557 0.999 0.442 0.053 0.865<br>Obart12g19300.1 0.092 0.528 0.436 0.01 0.99	1<br>Olong_002926-R1 0.097 0.529 0.432 0.002 0.996<br>Olong_002936-R1 0.557 0.999 0.442 0.059 0.866	1<br>Opunc03g25470.1 0.01 0.529 0.519 0.001 0.997<br>Opunc06g15370.1 0.557 0.999 0.442 0.035 0.871	0<br>Obrac03g20710.1 0.094 0.529 0.435 0.028 0.997	1<br>Lperr03g23190.1 0.557 0.999 0.442 0.03 0.867<br>Lperr03g23200.1 0.079 0.529 0.45 0.002 0.997	0<br>AT5G36940.1 0.093 0.531 0.438 0.003 0.998
Osind03g35550.1	1<br>Osind03g35550.1 0.001 1 0.999 0.001 1	1<br>Osjap03g34330.1 0.039 1 0.961 0.001 1	1<br>Orufi03g33060.1 0.039 1 0.961 0.001 1	1<br>Oglab03g30830.1 0.039 1 0.961 0.001 1	1<br>Obart03g31750.1 0.039 1 0.961 0.046 1	0	0	0	0	0
	1	1	1	1	1	1	1	1	1	0

	Osind01g46540.1 0.646 0.999 0.353 0.065 0.994<br>Osind10g03770.1 0.026 0.622 0.596 0.057 0.96	Osjap03g39110.1 0.646 1 0.354 0.036 1<br>Osjap10g05370.1 0.026 0.622 0.596 0.051 0.963	Orufi10g04850.1 0.026 0.622 0.596 0.054 0.963<br>Orufi11g12520.1 0.647 1 0.353 0.038 0.991	Oglab03g34460.1 0.622 0.997 0.375 0.016 0.993<br>Oglab10g04340.1 0.02 0.622 0.602 0.058 0.963	Obart03g36200.1 0.646 1 0.354 0.016 0.997<br>Obart10g04600.1 0.051 0.622 0.571 0.106 0.962	Olong_014796-R1 0.039 0.532 0.493 0.002 0.95<br>Olong_027495-R1 0.622 0.995 0.373 0.005 0.998	Opunc01g42030.1 0.646 1 0.354 0.28 0.872<br>Opunc10g04080.2 0.033 0.622 0.589 0.084 0.959	Obrac02g16280.1 0.64 0.981 0.341 0.063 0.949<br>Obrac03g23440.1 0.039 0.622 0.583 0.002 0.964	Lperr03g30520.1 0.638 1 0.362 0.003 0.997<br>Lperr10g03360.1 0.004 0.622 0.618 0.009 0.962	AT1G70730.3 0.039 0.622 0.583 0.118 0.967
Osind03g45590.1	1<br>Osind03g45590.1 0.002 1 0.998 0.002 1	1<br>Osjap03g44530.1 0.002 1 0.998 0.001 1	0	0	0	1<br>Olong_013357-R1 0.002 1 0.998 0.002 1	0	1<br>Obrac03g30850.1 0.008 1 0.992 0.023 1	0	0
	1<br>Osind03g42620.1 0.589 0.998 0.409 0.054 0.993<br>Osind07g07170.1 0.002 0.556 0.554 0.002 0.987	1<br>Osjap03g41540.2 0.589 0.998 0.409 0.064 0.993<br>Osjap07g07980.2 0.005 0.556 0.551 0.225 0.99	1<br>Orufi03g42810.1 0.577 1 0.423 0.004 1<br>Orufi07g06860.4 0.002 0.556 0.554 0.002 0.987	1<br>Oglab03g38980.1 0.569 1 0.431 0.004 1<br>Oglab03g38990.1 0.002 0.542 0.54 0.002 0.968	1<br>Obart03g41020.1 0.569 1 0.431 0.004 1<br>Obart03g41030.1 0.002 0.542 0.54 0.002 0.967	1<br>Olong_020188-R1 0.002 0.556 0.554 0.002 0.987<br>Olong_021244-R1 0.563 0.992 0.429 0.283 0.929	1<br>Opunc03g38110.1 0.581 1 0.419 0.007 1<br>Opunc07g06610.1 0.002 0.561 0.559 0.002 0.993	1<br>Obrac03g30850.2 0.008 0.542 0.534 0.037 0.969<br>Obrac04g17280.1 0.569 0.992 0.423 0.003 0.906	1<br>Lperr03g34950.2 0.577 1 0.423 0.129 0.987<br>Lperr07g06010.1 0.002 0.561 0.559 0.002 0.991	1<br>AT3G01980.3 0.589 1 0.411 0.017 1<br>AT5G46410.2 0.145 0.539 0.394 0.206 0.991
Osind04g18090.1	1<br>Osind04g18090.1 0.001 1 0.999 0.001 1	1<br>Osjap04g19350.1 0.001 1 0.999 0.001 1	1<br>Orufi04g17470.1 0.03 1 0.97 0.001 1	0	0	1<br>Olong_005904-R1 0.032 1 0.968 0.021 1	0	0	1<br>Lperr04g12800.1 0.004 0.999 0.995 0.032 0.999	0
	1<br>Osind02g23720.1 0.486 0.982 0.496 0.123 0.899<br>Osind02g25560.1 0.034 0.4 0.366 0.013 0.995	1<br>Osjap02g23820.1 0.486 0.982 0.496 0.12 0.898<br>Osjap02g25840.1 0.034 0.4 0.366 0.013 0.995	1<br>Orufi02g23890.1 0.034 0.4 0.366 0.013 0.995<br>Orufi06g19770.1 0.487 1 0.513 0.115 0.977	1<br>Oglab04g14400.1 0.45 1 0.55 0.002 1<br>Oglab04g14410.1 0.027 0.404 0.377 0.064 1	1<br>Obart04g16100.1 0.45 1 0.55 0.002 1<br>Obart04g16110.1 0.027 0.404 0.377 0.064 1	1<br>Olong_013421-R1 0.507 1 0.493 0.002 0.946<br>Olong_021848-R1 0.034 0.4 0.366 0.013 0.995	1<br>Opunc04g13970.1 0.45 0.992 0.542 0.002 0.985<br>Opunc04g13980.1 0.03 0.404 0.374 0.003 1	1<br>Obrac04g09860.1 0.032 0.404 0.372 0.008 1<br>Obrac07g00130.3 0.452 0.983 0.531 0.001 0.49	1<br>Lperr02g18380.1 0.034 0.4 0.366 0.012 0.995<br>Lperr07g00080.1 0.451 0.999 0.548 0.004 0.892	0<br>AT5G01110.1 0.405 0.992 0.587 0.111 0.996
Osind04g20860.1	1<br>Osind04g20860.1 0.012 1 0.988 0.012 1	0	0	0	0	0	0	0	0	0
	1<br>Osind04g20770.1 0.361 1 0.639 0.614 1<br>Osind04g20850.1 0.012 0.301 0.289 0.435 0.508	1<br>Osjap04g21700.2 0.012 0.301 0.289 0.608 0.652<br>Osjap04g21710.1 0.361 1 0.639 0.614 1	1<br>Orufi01g10140.2 0.012 0.301 0.289 0.478 0.537<br>Orufi04g19670.1 0.361 1 0.639 0.614 1	1<br>Oglab04g16620.2 0.012 0.301 0.289 0.619 0.662<br>Oglab04g16630.1 0.361 1 0.639 0.617 1	1<br>Obart04g18220.3 0.012 0.301 0.289 0.532 0.584<br>Obart04g18230.1 0.361 1 0.639 0.617 1	0<br>Olong_021609-R1 0.012 0.301 0.289 0.46 0.524	1<br>Opunc04g15930.1 0.012 0.301 0.289 0.541 0.592<br>Opunc04g15940.1 0.361 1 0.639 0.623 1	0<br>Obrac04g11580.1 0.012 0.313 0.301 0.458 0.528<br>Obrac04g11590.1 0.639 1 0.361 0.86 1	0<br>Lperr04g14670.1 0.012 0.301 0.289 0.519 0.573	0<br>AT1G65930.1 0.024 0.771 0.747 0.488 0.612
Osind04g33950.1	1<br>Osind04g33950.1 0.002 1 0.998 0.002 1	0	0	0	0	0	0	0	0	0
	1<br>Osind04g33960.1 0.405 1 0.595 0.008 1<br>Osind09g00580.1 0.002 0.382 0.38 0.044 0.835	1<br>Osjap04g34720.1 0.402 1 0.598 0.005 1<br>Osjap09g00600.1 0.002 0.38 0.378 0.051 0.967	1<br>Orufi04g32300.1 0.402 1 0.598 0.005 1<br>Orufi09g00610.1 0.002 0.382 0.38 0.044 0.835	1<br>Oglab04g28630.2 0.002 0.38 0.378 0.005 0.966<br>Oglab04g28670.1 0.405 1 0.595 0.036 1	1<br>Obart04g30600.1 0.405 1 0.595 0.035 1<br>Obart09g00570.1 0.002 0.38 0.378 0.051 0.967	0<br>Olong_000331-R1 0.008 0.38 0.372 0.014 0.972<br>Olong_027105-R1 0.608 1 0.392 0.004 1	1<br>Opunc02g24980.1 0.002 0.38 0.378 0.308 0.979<br>Opunc04g28070.2 0.402 0.998 0.596 0.005 0.998	0<br>Obrac04g21730.1 0.002 0.38 0.378 0.005 0.966<br>Obrac08g02870.1 0.602 0.998 0.396 0.353 0.987	0<br>Lperr04g26010.1 0.002 0.38 0.378 0.005 0.966<br>Lperr12g00280.2 0.6 0.994 0.394 0.262 1	0<br>AT1G05970.2 0.021 0.378 0.357 0.04 0.96
Osind05g00510.1	1<br>Osind05g00510.1 0.001 1 0.999 0.001 1	1<br>Osjap05g00530.1 0.001 1 0.999 0.131 1<br>Osjap05g00530.2 0.001 1 0.999 0.16 1<br>Osjap05g00530.3 0.001 1 0.999 0.161 1<br>Osjap05g00530.4 0.001 1 0.999 0.142 1	1<br>Orufi05g00500.1 0.001 1 0.999 0.131 1	0	1<br>Obart05g00520.1 0.001 0.999 0.998 0.049 0.993<br>Obart05g00520.2 0.001 1 0.999 0.049 1	1<br>Olong_022405-R1 0.001 1 0.999 0.001 1	0	0	0	0
	1<br>Osind01g11620.1 0.002 0.275 0.273 0.271 0.988<br>Osind09g15830.1 0.31 1 0.69 0.029 1	0<br>Osjap02g42390.1 0.326 0.993 0.667 0.001 0.983	1<br>Orufi07g24760.1 0.001 0.258 0.257 0.267 0.834<br>Orufi09g16480.1 0.31 1 0.69 0.029 1	1<br>Oglab05g00500.1 0.001 0.26 0.259 0.344 0.982<br>Oglab09g13820.1 0.291 1 0.709 0.064 1	1<br>Obart01g04950.1 0.001 0.258 0.257 0.193 0.858<br>Obart07g24200.1 0.302 0.998 0.696 0.005 0.907	1<br>Olong_004108-R1 0.004 0.275 0.271 0.098 0.928<br>Olong_023813-R1 0.303 0.997 0.694 0.001 0.979	1<br>Opunc05g00480.1 0.298 0.999 0.701 0.007 0.998<br>Opunc05g00490.1 0.001 0.26 0.259 0.352 0.982	1<br>Obrac02g14170.4 0.004 0.267 0.263 0.164 0.843<br>Obrac03g06070.3 0.31 0.998 0.688 0.023 0.996	1<br>Lperr02g16220.1 0.004 0.267 0.263 0.238 0.857<br>Lperr05g00500.1 0.301 1 0.699 0.009 1	0<br>AT4G14820.1 0.001 0.267 0.266 0.107 0.878
Osind05g03420.1	1<br>Osind05g03420.1 0.001 1 0.999 0.001 1	0	0	0	0	0	1<br>Opunc05g03030.1 0.001 1 0.999 0.001 1	0	0	0
	1	1	1	1	1	1	0	1	1	1

	Osind06g19850.1 0.025 0.678 0.653 0.045 0.929<br>Osind06g23670.1 0.746 0.982 0.236 0.045 0.848	Osjap05g03420.2 0.706 0.982 0.276 0.075 0.828<br>Osjap05g03430.1 0.001 0.688 0.687 0.002 0.981	Orufi05g03290.1 0.706 0.982 0.276 0.064 0.854<br>Orufi05g03300.1 0.001 0.688 0.687 0.002 0.981	Oglab05g02870.1 0.706 0.997 0.291 0.068 0.948<br>Oglab05g02880.1 0.001 0.688 0.687 0.002 0.981	Obart05g03140.1 0.706 0.997 0.291 0.068 0.948<br>Obart05g03150.1 0.001 0.688 0.687 0.002 0.98	Olong_004932-R1 0.001 0.688 0.687 0.125 0.984<br>Olong_004933-R1 0.71 0.976 0.266 0.073 0.861	Opunc05g22860.1 0.005 0.669 0.664 0.053 0.921	Obrac05g02530.1 0.729 0.976 0.247 0.035 0.831<br>Obrac05g02540.1 0.001 0.688 0.687 0.002 0.981	Lperr05g02940.1 0.001 0.688 0.687 0.002 0.981<br>Lperr08g08920.1 0.733 0.999 0.266 0.004 0.819	AT3G24110.1 0.735 0.982 0.247 0.022 0.843<br>AT5G16070.1 0.001 0.688 0.687 0.002 0.979
Osind05g04070.1	1<br>Osind05g04070.1 0.001 1 0.999 0.001 1<br>Osind05g04070.2 0.001 1 0.999 0.001 1	1<br>Osjap05g04120.1 0.001 1 0.999 0.008 1<br>Osjap05g04120.2 0.001 1 0.999 0.008 1	1<br>Orufi05g03950.1 0.001 1 0.999 0.008 1<br>Orufi05g03950.2 0.001 1 0.999 0.008 1	1<br>Oglab05g03490.1 0.001 1 0.999 0.008 1<br>Oglab05g03490.2 0.001 1 0.999 0.008 1	1<br>Obart05g03830.1 0.001 1 0.999 0.008 1<br>Obart05g03830.2 0.001 1 0.999 0.008 1	1<br>Olong_016739-R1 0.069 1 0.931 0.001 1	1<br>Opunc05g03540.1 0.001 1 0.999 0.008 1<br>Opunc05g03540.2 0.001 1 0.999 0.008 1	1<br>Obrac05g02930.1 0.069 1 0.931 0.001 1	0	0
	1<br>Osind01g28630.1 0.086 0.735 0.649 0.066 0.992<br>Osind03g15230.1 0.783 1 0.217 0.03 1	1<br>Osjap01g27410.1 0.086 0.735 0.649 0.066 0.992<br>Osjap03g15140.2 0.738 1 0.262 0.01 0.192	1<br>Orufi01g26130.1 0.086 0.735 0.649 0.066 0.992<br>Orufi03g14720.1 0.783 1 0.217 0.03 1	1<br>Oglab01g19490.1 0.086 0.735 0.649 0.066 0.992<br>Oglab03g14060.1 0.783 1 0.217 0.03 1	1<br>Obart01g23310.1 0.086 0.735 0.649 0.066 0.992<br>Obart03g14050.1 0.783 1 0.217 0.03 1	1<br>Olong_003347-R2 0.086 0.735 0.649 0.047 0.992<br>Olong_009943-R1 0.783 1 0.217 0.03 1	1<br>Opunc01g23460.1 0.086 0.735 0.649 0.064 0.991<br>Opunc03g13590.1 0.783 1 0.217 0.002 0.545	1<br>Obrac01g17590.1 0.086 0.735 0.649 0.067 0.991<br>Obrac03g11330.1 0.783 1 0.217 0.03 1	0<br>Lperr05g03460.1 0.001 0.736 0.735 0.011 0.994	1<br>AT2G47580.1 0.768 1 0.232 0.008 1<br>AT3G16630.1 0.001 0.736 0.735 0.039 0.994
Osind05g04070.2	1<br>Osind05g04070.1 0.001 1 0.999 0.001 1<br>Osind05g04070.2 0.001 1 0.999 0.001 1	1<br>Osjap05g04120.1 0.001 1 0.999 0.008 1<br>Osjap05g04120.2 0.001 1 0.999 0.008 1	1<br>Orufi05g03950.1 0.001 1 0.999 0.008 1<br>Orufi05g03950.2 0.001 1 0.999 0.008 1	1<br>Oglab05g03490.1 0.001 1 0.999 0.008 1<br>Oglab05g03490.2 0.001 1 0.999 0.008 1	1<br>Obart05g03830.1 0.001 1 0.999 0.008 1<br>Obart05g03830.2 0.001 1 0.999 0.008 1	1<br>Olong_016739-R1 0.068 1 0.932 0.001 1	1<br>Opunc05g03540.1 0.001 1 0.999 0.008 1<br>Opunc05g03540.2 0.001 1 0.999 0.008 1	1<br>Obrac05g02930.1 0.068 1 0.932 0.001 1	0	0
	1<br>Osind01g28630.1 0.084 0.738 0.654 0.066 0.992<br>Osind03g15230.1 0.786 1 0.214 0.03 1	1<br>Osjap01g27410.1 0.084 0.738 0.654 0.066 0.992<br>Osjap03g15140.2 0.742 1 0.258 0.01 0.192	1<br>Orufi01g26130.1 0.084 0.738 0.654 0.066 0.992<br>Orufi03g14720.1 0.786 1 0.214 0.03 1	1<br>Oglab01g19490.1 0.084 0.738 0.654 0.066 0.992<br>Oglab03g14060.1 0.786 1 0.214 0.03 1	1<br>Obart01g23310.1 0.084 0.738 0.654 0.066 0.992<br>Obart03g14050.1 0.786 1 0.214 0.03 1	1<br>Olong_003347-R2 0.084 0.738 0.654 0.047 0.992<br>Olong_009943-R1 0.786 1 0.214 0.03 1	1<br>Opunc01g23460.1 0.084 0.738 0.654 0.064 0.991<br>Opunc03g13590.1 0.786 1 0.214 0.002 0.545	1<br>Obrac01g17590.1 0.084 0.738 0.654 0.067 0.991<br>Obrac03g11330.1 0.786 1 0.214 0.03 1	0<br>Lperr05g03460.1 0.001 0.74 0.739 0.011 0.994	1<br>AT2G47580.1 0.771 1 0.229 0.008 1<br>AT3G16630.1 0.001 0.74 0.739 0.039 0.994
Osind05g23260.1	1<br>Osind05g23260.1 0.01 1 0.99 0.01 1	0	0	0	0	0	0	0	0	0
	1<br>Osind02g39980.1 0.01 0.344 0.334 0.005 0.16<br>Osind04g23300.1 0.344 1 0.656 0.698 1	1<br>Osjap02g40490.1 0.01 0.344 0.334 0.494 0.573<br>Osjap04g24190.1 0.344 1 0.656 0.698 1	1<br>Orufi02g37810.1 0.01 0.344 0.334 0.494 0.573<br>Orufi04g22070.1 0.344 1 0.656 0.698 1	1<br>Oglab04g18980.1 0.344 1 0.656 0.698 1<br>Oglab07g15850.1 0.01 0.344 0.334 0.348 0.525	1<br>Obart04g20490.1 0.344 1 0.656 0.698 1<br>Obart05g09150.1 0.01 0.333 0.323 0.038 0.136	0<br>Olong_006838-R1 0.344 1 0.656 0.33 1	1<br>Opunc02g33420.1 0.01 0.344 0.334 0.501 0.581<br>Opunc04g18140.1 0.344 1 0.656 0.703 1	0<br>Obrac04g13370.1 0.344 0.896 0.552 0.679 0.912	1<br>Lperr02g29870.1 0.01 0.344 0.334 0.46 0.543<br>Lperr04g16590.1 0.344 0.938 0.594 0.677 0.955	0
Osind05g28520.1	1<br>Osind05g28520.1 0.001 1 0.999 0.001 1	1<br>Osjap05g28230.1 0.001 1 0.999 0.001 1	1<br>Orufi05g26870.1 0.001 1 0.999 0.001 1	1<br>Oglab05g23160.1 0.001 1 0.999 0.001 1	1<br>Obart05g25070.1 0.001 1 0.999 0.001 1	0	1<br>Opunc05g22640.1 0.001 1 0.999 0.001 1	0	1<br>Lperr01g22990.1 0.001 1 0.999 0.027 1	0
	1<br>Osind01g32600.1 0.001 0.43 0.429 0.002 0.995<br>Osind01g32610.1 0.486 0.999 0.513 0.009 0.999	1<br>Osjap01g31140.1 0.006 0.43 0.424 0.093 0.995<br>Osjap01g31150.1 0.486 0.999 0.513 0.009 0.999	1<br>Orufi01g29880.1 0.001 0.43 0.429 0.075 0.995<br>Orufi01g29890.1 0.486 0.999 0.513 0.009 0.999	1<br>Oglab01g22740.1 0.091 0.431 0.34 0.213 0.996<br>Oglab01g22750.1 0.486 0.999 0.513 0.009 0.999	1<br>Obart01g26870.1 0.005 0.43 0.425 0.117 0.995<br>Obart01g26880.1 0.486 0.999 0.513 0.009 0.999	1<br>Olong_000367-R1 0.1 0.431 0.331 0.002 0.995<br>Olong_000368-R1 0.461 0.999 0.538 0.069 0.999	1<br>Opunc01g26820.1 0.091 0.431 0.34 0.118 0.996<br>Opunc01g26830.1 0.486 1 0.514 0.009 1	1<br>Obrac01g20400.1 0.091 0.431 0.34 0.125 0.996<br>Obrac05g17560.2 0.431 1 0.569 0.109 1	1<br>Lperr05g20850.2 0.491 1 0.509 0.002 1<br>Lperr05g20860.1 0.091 0.431 0.34 0.215 0.996	1<br>AT5G08680.1 0.022 0.43 0.408 0.064 0.995<br>AT5G51600.1 0.486 0.999 0.513 0.008 0.996
Osind05g31580.1	1<br>Osind05g31580.1 0.001 1 0.999 0.001 1	0	0	0	1<br>Obart05g27940.1 0.024 1 0.976 0.001 1	1<br>Olong_007981-R1 0.074 1 0.926 0.001 1	0	0	0	0
	1<br>Osind01g25470.1 0.024 0.579 0.555 0.061 0.967<br>Osind01g25480.1 0.635 0.951 0.316 0.063 0.877	1<br>Osjap01g24630.2 0.635 0.951 0.316 0.156 0.894<br>Osjap05g31230.3 0.001 0.627 0.626 0.006 0.982	1<br>Orufi01g23560.1 0.635 0.951 0.316 0.144 0.891<br>Orufi05g29910.3 0.001 0.627 0.626 0.001 0.982	1<br>Oglab05g25920.1 0.001 0.627 0.626 0.001 0.982<br>Oglab05g25930.1 0.635 1 0.365 0.07 1	0<br>Obart01g20890.1 0.635 0.951 0.316 0.063 0.877<br>Obart07g06670.1 0.179 0.628 0.449 0.36 0.978	1<br>Olong_011279-R1 0.635 0.991 0.356 0.063 0.978<br>Olong_022566-R2 0.179 0.626 0.447 0.313 0.975	1<br>Opunc05g25540.1 0.007 0.627 0.62 0.002 0.983<br>Opunc05g25550.1 0.635 1 0.365 0.067 1	0<br>Obrac05g19820.2 0.024 0.627 0.603 0.001 0.982	1<br>Lperr01g17840.1 0.635 0.999 0.364 0.166 0.958<br>Lperr05g23570.2 0.003 0.627 0.624 0.002 0.982	1<br>AT1G07990.1 0.635 0.912 0.277 0.061 0.782<br>AT4G05420.1 0.024 0.627 0.603 0.001 0.982
Osind06g17590.1	1<br>Osind06g17590.1 0.001 1 0.999 0.001 1<br>Osind08g03120.1 0.01 0.991 0.981 0.058 0.989	0	0	0	1<br>Obart11g22180.1 0.037 0.999 0.962 0.038 0.984	1<br>Olong_000078-R1 0.035 0.896 0.861 0.028 0.972	0	0	0	0
	1	1	1	1	1	1	1	1	1	0

	Osind01g01210.1 0.474 0.999 0.525 0.091 0.977<br>Osind06g29310.2 0.016 0.463 0.447 0.009 0.829	Osjap06g30040.4 0.016 0.463 0.447 0.009 0.829<br>Osjap11g26070.1 0.474 0.999 0.525 0.095 0.977	Orufi06g27630.3 0.016 0.463 0.447 0.009 0.829<br>Orufi11g24100.1 0.488 0.918 0.43 0.147 0.881	Oglab06g23090.2 0.016 0.463 0.447 0.009 0.829<br>Oglab11g19360.1 0.474 0.999 0.525 0.091 0.977	Obart11g22190.1 0.035 0.47 0.435 0.076 0.928<br>Obart11g22210.1 0.482 0.991 0.509 0.031 0.971	Olong_000079-R1 0.02 0.474 0.454 0.017 0.917<br>Olong_022539-R1 0.488 0.889 0.401 0.1 0.844	Opunc11g18540.2 0.018 0.466 0.448 0.045 0.912<br>Opunc11g18840.1 0.474 1 0.526 0.148 1	Obrac11g13070.1 0.474 0.992 0.518 0.016 0.963<br>Obrac11g13100.2 0.035 0.474 0.439 0.13 0.891	Lperr06g21190.1 0.01 0.463 0.453 0.004 0.899<br>Lperr11g18890.1 0.474 0.994 0.52 0.119 0.988	AT4G11460.1 0.22 0.503 0.283 0.466 0.884
Osind06g23650.1	1<br>Osind06g23650.1 0.001 1 0.999 0.001 1	0	1<br>Orufi06g22110.1 0.001 1 0.999 0.011 1	0	1<br>Obart06g20680.1 0.001 1 0.999 0.001 1	0	0	0	0	0
	1<br>Osind02g08380.1 0.714 0.999 0.285 0.031 0.997<br>Osind09g17960.1 0.005 0.693 0.688 0.008 0.978	1<br>Osjap06g24310.1 0.001 0.693 0.692 0.059 0.986<br>Osjap06g24320.3 0.713 1 0.287 0.019 1	1<br>Orufi02g07600.1 0.714 0.999 0.285 0.031 0.997<br>Orufi09g15100.1 0.01 0.693 0.683 0.029 0.975	1<br>Oglab06g18700.1 0.001 0.693 0.692 0.016 0.986<br>Oglab06g18710.1 0.713 1 0.287 0.019 1	1<br>Obart02g07500.3 0.714 0.999 0.285 0.031 0.997<br>Obart04g22320.1 0.005 0.691 0.686 0.017 0.982	1<br>Olong_021346-R1 0.001 0.693 0.692 0.01 0.982<br>Olong_021347-R1 0.715 1 0.285 0.003 1	1<br>Opunc01g29350.1 0.001 0.691 0.69 0.004 0.986<br>Opunc06g18860.1 0.713 1 0.287 0.009 0.497	1<br>Obrac04g14990.1 0.005 0.691 0.686 0.017 0.982<br>Obrac06g14090.3 0.712 1 0.288 0.338 1	1<br>Lperr01g25410.2 0.001 0.691 0.69 0.009 0.986<br>Lperr02g06450.6 0.714 0.999 0.285 0.032 0.997	1<br>AT4G21160.1 0.716 1 0.284 0.047 1<br>AT5G51750.1 0.005 0.693 0.688 0.021 0.985
Osind07g02300.3	1<br>Osind07g02300.3 0.002 1 0.998 0.002 1<br>Osind07g02300.4 0.002 1 0.998 0.002 1	1<br>Osjap07g02600.3 0.002 0.978 0.976 0.478 0.853<br>Osjap07g02600.4 0.002 1 0.998 0.555 1<br>Osjap07g02600.5 0.002 1 0.998 0.002 1	0	0	0	0	0	0	0	0
	1<br>Osind01g07120.1 0.002 0.254 0.252 0.692 0.935<br>Osind07g02530.1 0.298 0.976 0.678 0.411 0.823	1<br>Osjap01g07190.1 0.002 0.254 0.252 0.692 0.935<br>Osjap07g02840.1 0.298 0.976 0.678 0.411 0.823	1<br>Orufi07g02320.1 0.298 0.976 0.678 0.411 0.823<br>Orufi12g02700.1 0.007 0.259 0.252 0.518 0.746	1<br>Oglab01g05790.1 0.002 0.254 0.252 0.692 0.935<br>Oglab07g02390.2 0.298 0.976 0.678 0.41 0.823	1<br>Obart01g06210.1 0.002 0.254 0.252 0.692 0.935<br>Obart07g02450.1 0.298 0.976 0.678 0.377 0.809	1<br>Olong_003501-R1 0.002 0.254 0.252 0.634 0.923<br>Olong_009970-R1 0.298 0.976 0.678 0.411 0.823	1<br>Opunc01g05980.1 0.002 0.254 0.252 0.689 0.934<br>Opunc07g02500.8 0.298 0.976 0.678 0.472 0.79	1<br>Obrac05g04770.1 0.002 0.254 0.252 0.594 0.923<br>Obrac07g01520.1 0.298 0.99 0.692 0.386 0.893	1<br>Lperr07g02310.1 0.298 0.976 0.678 0.431 0.845<br>Lperr11g02330.1 0.007 0.257 0.25 0.682 0.933	0<br>AT4G36380.1 0.002 0.308 0.306 0.691 0.966
Osind07g02300.4	1<br>Osind07g02300.3 0.002 1 0.998 0.002 1<br>Osind07g02300.4 0.002 1 0.998 0.002 1	1<br>Osjap07g02600.3 0.002 0.978 0.976 0.478 0.853<br>Osjap07g02600.4 0.002 1 0.998 0.555 1<br>Osjap07g02600.5 0.002 1 0.998 0.002 1	0	0	0	0	0	0	0	0
	1<br>Osind01g07120.1 0.002 0.254 0.252 0.692 0.935<br>Osind07g02530.1 0.298 0.976 0.678 0.411 0.823	1<br>Osjap01g07190.1 0.002 0.254 0.252 0.692 0.935<br>Osjap07g02840.1 0.298 0.976 0.678 0.411 0.823	1<br>Orufi07g02320.1 0.298 0.976 0.678 0.411 0.823<br>Orufi12g02700.1 0.007 0.259 0.252 0.518 0.746	1<br>Oglab01g05790.1 0.002 0.254 0.252 0.692 0.935<br>Oglab07g02390.2 0.298 0.976 0.678 0.41 0.823	1<br>Obart01g06210.1 0.002 0.254 0.252 0.692 0.935<br>Obart07g02450.1 0.298 0.976 0.678 0.377 0.809	1<br>Olong_003501-R1 0.002 0.254 0.252 0.634 0.923<br>Olong_009970-R1 0.298 0.976 0.678 0.411 0.823	1<br>Opunc01g05980.1 0.002 0.254 0.252 0.689 0.934<br>Opunc07g02500.8 0.298 0.976 0.678 0.472 0.79	1<br>Obrac05g04770.1 0.002 0.254 0.252 0.594 0.923<br>Obrac07g01520.1 0.298 0.99 0.692 0.386 0.893	1<br>Lperr07g02310.1 0.298 0.976 0.678 0.431 0.845<br>Lperr11g02330.1 0.007 0.257 0.25 0.682 0.933	0<br>AT4G36380.1 0.002 0.308 0.306 0.691 0.966
Osind07g04880.1	1<br>Osind07g04880.1 0.002 1 0.998 0.002 1	1<br>Osjap07g05600.1 0.002 1 0.998 0.002 1	1<br>Orufi07g04740.1 0.002 0.94 0.938 0.002 0.996	0	0	0	0	0	0	0
	1<br>Osind04g13510.1 0.356 0.865 0.509 0.318 0.938<br>Osind07g04890.1 0.002 0.333 0.331 0.006 0.696	1<br>Osjap04g15050.1 0.356 0.865 0.509 0.315 0.932<br>Osjap07g05610.1 0.036 0.333 0.297 0.005 0.444	1<br>Orufi04g13400.1 0.356 0.865 0.509 0.318 0.938<br>Orufi07g04750.1 0.036 0.333 0.297 0.007 0.667	1<br>Oglab04g10600.1 0.356 0.865 0.509 0.323 0.938<br>Oglab07g04740.1 0.002 0.32 0.318 0.004 0.866	1<br>Obart02g19560.1 0.361 0.865 0.504 0.151 0.92<br>Obart07g04940.1 0.002 0.32 0.318 0.007 0.757	0<br>Olong_011399-R1 0.002 0.601 0.599 0.004 0.978	1<br>Opunc02g17460.1 0.361 0.873 0.512 0.326 0.95<br>Opunc08g05640.1 0.002 0.333 0.331 0.006 0.705	0<br>Obrac02g20740.1 0.569 0.908 0.339 0.635 0.984	0<br>Lperr02g15690.1 0.356 0.865 0.509 0.32 0.935	0<br>AT3G52450.1 0.567 0.865 0.298 0.618 0.936
Osind07g21400.1	1<br>Osind07g21400.1 0.001 1 0.999 0.001 1<br>Osind07g21400.2 0.001 0.99 0.989 0.001 0.99	0	0	0	0	0	0	0	0	0
	1<br>Osind04g27100.1 0.016 0.356 0.34 0.09 0.951<br>Osind07g09290.1 0.49 0.965 0.475 0.204 0.813	1<br>Osjap07g24560.1 0.001 0.366 0.365 0.003 0.973<br>Osjap07g24570.1 0.427 0.989 0.562 0.093 0.989	1<br>Orufi07g22110.1 0.001 0.366 0.365 0.003 0.973<br>Orufi07g22120.1 0.427 0.99 0.563 0.093 0.99	1<br>Oglab07g18190.1 0.001 0.383 0.382 0.003 0.883<br>Oglab07g18200.1 0.427 0.99 0.563 0.093 0.99	1<br>Obart07g21090.1 0.001 0.366 0.365 0.003 0.973<br>Obart07g21100.1 0.458 0.99 0.532 0.179 0.991	1<br>Olong_006539-R1 0.001 0.394 0.393 0.003 0.945<br>Olong_006540-R1 0.498 0.99 0.492 0.062 0.989	1<br>Opunc07g13090.1 0.001 0.394 0.393 0.002 0.885<br>Opunc07g13100.1 0.394 0.99 0.596 0.059 0.99	1<br>Obrac07g13360.2 0.001 0.366 0.365 0.003 0.973<br>Obrac07g13370.2 0.496 0.999 0.503 0.002 1	1<br>Lperr07g18240.1 0.394 0.99 0.596 0.061 0.994<br>Lperr07g18250.1 0.001 0.357 0.356 0.003 0.809	1<br>AT1G26460.1 0.399 0.992 0.593 0.041 0.998<br>AT1G26520.1 0.001 0.356 0.355 0.029 0.947
Osind07g21400.2	1	0	0	0	0	0	0	0	0	0

	Osind07g21400.1 0.001 0.99 0.989 0.001 0.99<br>Osind07g21400.2 0.001 1 0.999 0.001 1									
	1<br>Osind04g27100.1 0.016 0.356 0.34 0.09 0.951<br>Osind07g09290.1 0.49 0.965 0.475 0.204 0.813	1<br>Osjap07g24560.1 0.001 0.366 0.365 0.003 0.973<br>Osjap07g24570.1 0.427 0.995 0.568 0.093 0.998	1<br>Orufi07g22110.1 0.001 0.366 0.365 0.003 0.973<br>Orufi07g22120.1 0.427 0.995 0.568 0.093 0.998	1<br>Oglab07g18190.1 0.001 0.386 0.385 0.003 0.918<br>Oglab07g18200.1 0.427 0.995 0.568 0.093 0.998	1<br>Obart07g21090.1 0.001 0.366 0.365 0.003 0.973<br>Obart07g21100.1 0.458 0.995 0.537 0.179 0.998	1<br>Olong_006539-R1 0.001 0.394 0.393 0.003 0.945<br>Olong_006540-R1 0.498 0.995 0.497 0.062 0.998	1<br>Opunc07g13090.1 0.001 0.394 0.393 0.002 0.885<br>Opunc07g13100.1 0.394 0.995 0.601 0.059 0.998	1<br>Obrac07g13360.2 0.001 0.366 0.365 0.003 0.973<br>Obrac07g13370.3 0.496 0.992 0.496 0.002 0.994	1<br>Lperr07g18240.1 0.394 0.992 0.598 0.061 0.997<br>Lperr07g18250.1 0.001 0.357 0.356 0.003 0.809	1<br>AT1G26460.1 0.399 0.986 0.587 0.041 0.989<br>AT1G26520.1 0.001 0.356 0.355 0.029 0.947
Osind07g25180.1	1<br>Osind07g25180.1 0.002 1 0.998 0.002 1	0	0	0	0	0	0	0	0	0
	1<br>Osind07g25220.1 0.002 0.688 0.686 0.003 0.961<br>Osind07g25260.1 0.716 1 0.284 0.711 1	1<br>Osjap07g28590.1 0.002 0.688 0.686 0.003 0.822<br>Osjap07g28610.1 0.716 1 0.284 0.543 1	1<br>Orufi07g25740.1 0.002 0.688 0.686 0.003 0.823<br>Orufi07g25750.1 0.716 1 0.284 0.543 1	1<br>Oglab07g21250.1 0.032 0.688 0.656 0.003 0.826<br>Oglab07g21260.1 0.716 1 0.284 0.543 1	1<br>Obart07g24850.1 0.007 0.688 0.681 0.025 0.798<br>Obart07g24870.1 0.716 1 0.284 0.709 0.948	1<br>Olong_018533-R1 0.716 1 0.284 0.543 1<br>Olong_020830-R1 0.051 0.686 0.635 0.045 0.983	1<br>Opunc07g23230.1 0.032 0.688 0.656 0.003 0.816<br>Opunc07g23260.1 0.716 1 0.284 0.636 1	1<br>Obrac07g16190.1 0.032 0.688 0.656 0.003 0.817<br>Obrac07g16210.1 0.716 1 0.284 0.57 1	1<br>Lperr07g21550.1 0.032 0.688 0.656 0.003 0.829<br>Lperr07g21560.1 0.716 1 0.284 0.543 1	1<br>AT1G03260.1 0.688 0.988 0.3 0.496 0.996<br>AT2G19150.1 0.048 0.688 0.64 0.021 0.802
Osind07g27670.1	1<br>Osind07g27670.1 0.008 1 0.992 0.008 1<br>Osind07g27730.1 0.008 1 0.992 0.404 1	1<br>Osjap07g31020.1 0.008 1 0.992 0.404 1	0	1<br>Oglab07g23640.1 0.016 1 0.984 0.801 0.998<br>Oglab07g23640.2 0.016 1 0.984 0.813 0.998	1<br>Obart07g27040.1 0.016 1 0.984 0.144 0.32<br>Obart07g27040.2 0.016 1 0.984 0.118 0.299	0	0	0	0	0
	1<br>Osind07g27700.1 0.008 0.664 0.656 0.124 0.258<br>Osind07g27740.1 0.73 1 0.27 0.939 0.972	1<br>Osjap03g15620.2 0.73 1 0.27 0.934 0.97<br>Osjap07g30980.1 0.008 0.639 0.631 0.199 0.358	1<br>Orufi03g15210.1 0.73 1 0.27 0.939 0.972<br>Orufi07g27980.1 0.008 0.664 0.656 0.221 0.34	1<br>Oglab03g14540.1 0.73 1 0.27 0.938 0.971<br>Oglab07g23620.1 0.008 0.664 0.656 0.124 0.258	1<br>Obart07g27030.1 0.008 0.664 0.656 0.425 0.51<br>Obart07g27050.1 0.73 1 0.27 0.939 0.972	1<br>Olong_008697-R2 0.73 1 0.27 0.936 0.971<br>Olong_008701-R1 0.008 0.664 0.656 0.124 0.258	1<br>Opunc07g25410.1 0.008 0.664 0.656 0.129 0.268<br>Opunc07g25450.1 0.73 1 0.27 0.937 0.971	1<br>Obrac03g11770.2 0.73 1 0.27 0.931 0.968<br>Obrac07g18100.1 0.008 0.664 0.656 0.131 0.271	1<br>Lperr03g12860.1 0.73 1 0.27 0.937 0.971<br>Lperr07g23810.1 0.008 0.664 0.656 0.123 0.256	0<br>AT3G66658.2 0.008 0.754 0.746 0.124 0.273
Osind08g03120.1	1<br>Osind06g17590.1 0.058 0.989 0.931 0.01 0.991<br>Osind08g03120.1 0.001 1 0.999 0.001 1	0	0	0	1<br>Obart11g22180.1 0.059 0.995 0.936 0.028 0.98	1<br>Olong_000078-R1 0.144 1 0.856 0.049 1	0	0	0	0
	1<br>Osind01g01210.1 0.001 0.668 0.667 0.002 0.971<br>Osind06g17600.1 0.717 1 0.283 0.054 0.335	1<br>Osjap11g26070.1 0.001 0.668 0.667 0.002 0.97<br>Osjap11g26110.1 0.717 1 0.283 0.046 0.285	1<br>Orufi11g24100.1 0.037 0.655 0.618 0.067 0.972<br>Orufi11g24120.1 0.717 1 0.283 0.386 0.554	1<br>Oglab11g19360.1 0.001 0.668 0.667 0.002 0.971<br>Oglab11g19410.2 0.717 1 0.283 0.009 0.413	0<br>Obart11g22150.1 0.037 0.655 0.618 0.067 0.972	1<br>Olong_022539-R1 0.005 0.668 0.663 0.018 0.968<br>Olong_028213-R1 0.717 1 0.283 0.308 0.494	1<br>Opunc11g18800.1 0.001 0.667 0.666 0.002 0.987<br>Opunc11g18810.1 0.717 0.998 0.281 0.027 0.308	1<br>Obrac11g13070.1 0.052 0.655 0.603 0.002 0.949<br>Obrac11g13130.1 0.717 0.999 0.282 0.036 0.876	1<br>Lperr11g18900.3 0.717 1 0.283 0.003 0.298<br>Lperr12g12660.2 0.041 0.621 0.58 0.058 0.883	0<br>AT2G17780.1 0.716 0.999 0.283 0.06 0.346<br>AT2G18750.1 0.19 0.593 0.403 0.153 0.812
Osind08g06790.1	1<br>Osind08g06790.1 0.002 1 0.998 0.002 1	1<br>Osjap08g06650.1 0.002 1 0.998 0.002 1	1<br>Orufi08g06840.1 0.002 1 0.998 0.002 1	1<br>Oglab08g05640.1 0.002 1 0.998 0.002 1	1<br>Obart08g06070.1 0.002 1 0.998 0.002 1	0	1<br>Opunc08g05850.1 0.047 1 0.953 0.068 1	0	0	0
	1<br>Osind04g20250.1 0.047 0.275 0.228 0.198 0.94<br>Osind09g20960.1 0.359 0.984 0.625 0.208 0.966	1<br>Osjap04g21220.1 0.047 0.275 0.228 0.21 0.936<br>Osjap09g20870.1 0.359 0.984 0.625 0.208 0.966	1<br>Orufi04g19250.2 0.047 0.275 0.228 0.21 0.936<br>Orufi09g21960.1 0.359 0.984 0.625 0.2 0.965	1<br>Oglab02g26360.1 0.348 0.996 0.648 0.01 0.994<br>Oglab04g16180.1 0.047 0.275 0.228 0.198 0.94	1<br>Obart02g28440.1 0.348 0.996 0.648 0.093 0.994<br>Obart04g17770.1 0.047 0.275 0.228 0.198 0.94	1<br>Olong_008030-R1 0.047 0.275 0.228 0.232 0.937<br>Olong_008031-R2 0.277 1 0.723 0.106 1	1<br>Opunc04g15550.1 0.047 0.275 0.228 0.206 0.938<br>Opunc04g18740.1 0.295 0.984 0.689 0.088 0.994	1<br>Obrac08g04780.1 0.277 1 0.723 0.098 1<br>Obrac08g04800.1 0.002 0.275 0.273 0.006 0.943	1<br>Lperr08g05690.1 0.277 1 0.723 0.1 1<br>Lperr08g05700.1 0.002 0.275 0.273 0.006 0.943	1<br>AT1G05310.1 0.33 0.982 0.652 0.183 0.99<br>AT4G30800.1 0.047 0.275 0.228 0.208 0.943
Osind08g08550.1	1<br>Osind08g08550.1 0.003 1 0.997 0.003 1	0	0	0	0	0	0	0	0	0
	1<br>Osind08g08530.1 0.465 1 0.535 0.161 1<br>Osind08g08570.1 0.003 0.422 0.419 0.011 0.188	1<br>Osjap08g08130.3 0.531 1 0.469 0.072 1<br>Osjap08g08150.1 0.003 0.422 0.419 0.011 0.188	1<br>Orufi08g08460.1 0.465 1 0.535 0.303 1<br>Orufi08g08480.2 0.003 0.422 0.419 0.011 0.19	1<br>Oglab08g06850.1 0.465 1 0.535 0.303 1<br>Oglab08g06860.1 0.003 0.422 0.419 0.014 0.236	1<br>Obart08g07340.1 0.465 1 0.535 0.303 1<br>Obart08g07350.1 0.003 0.422 0.419 0.014 0.236	0<br>Olong_007960-R1 0.465 1 0.535 0.233 1	1<br>Opunc08g07070.1 0.465 1 0.535 0.303 1<br>Opunc08g07080.1 0.003 0.422 0.419 0.011 0.187	0<br>Obrac08g05540.1 0.465 0.997 0.532 0.303 0.997	1<br>Lperr08g06550.1 0.459 1 0.541 0.294 1<br>Lperr08g06560.1 0.003 0.422 0.419 0.011 0.182	1<br>AT3G11470.2 0.465 0.97 0.505 0.3 0.983<br>AT4G24520.1 0.05 0.422 0.372 0.012 0.158
Osind08g22530.1	1	1	1	0	0	1	0	0	0	0

	Osind08g22530.1 0.002 1 0.998 0.002 1<br>Osind08g22680.1 0.111 0.994 0.883 0.199 0.933	Osjap08g20640.1 0.111 0.994 0.883 0.199 0.933	Orufi08g21480.1 0.002 1 0.998 0.001 0.97<br>Orufi08g21480.2 0.002 1 0.998 0.003 0.952<br>Orufi08g21640.1 0.111 0.994 0.883 0.199 0.933			Olong_020048-R1 0.002 1 0.998 0.003 0.94				
	1<br>Osind12g07100.1 0.009 0.471 0.462 0.042 0.813<br>Osind12g11660.1 0.491 0.927 0.436 0.003 1	1<br>Osjap05g14370.1 0.491 0.912 0.421 0.004 1<br>Osjap12g00550.1 0.091 0.48 0.389 0.127 0.648	1<br>Orufi05g13530.1 0.491 0.912 0.421 0.004 1<br>Orufi12g00580.1 0.091 0.48 0.389 0.145 0.74	1<br>Oglab02g26530.1 0.491 0.927 0.436 0.003 1<br>Oglab09g10810.1 0.009 0.486 0.477 0.017 0.871	1<br>Obart09g11820.1 0.009 0.486 0.477 0.017 0.871<br>Obart11g19340.1 0.491 0.927 0.436 0.004 1	1<br>Olong_005330-R1 0.002 0.486 0.484 0.003 0.821<br>Olong_028575-R1 0.514 0.927 0.413 0.004 1	0<br>Opunc08g17290.1 0.204 1 0.796 0.002 0.47	1<br>Obrac03g12080.1 0.551 0.923 0.372 0.173 0.986<br>Obrac12g00030.1 0.026 0.486 0.46 0.436 0.966	0<br>Lperr08g16100.3 0.005 0.569 0.564 0.013 0.982	0
Osind08g27620.1	1<br>Osind08g27620.1 0.009 1 0.991 0.009 1	0	0	1<br>Oglab08g20310.1 0.009 1 0.991 0.007 1	1<br>Obart08g23660.1 0.009 1 0.991 0.007 1	1<br>Olong_020567-R1 0.009 1 0.991 0.008 1	0	0	0	0
	1<br>Osind03g42330.1 0.009 0.377 0.368 0.211 0.258<br>Osind03g44220.1 0.387 0.991 0.604 0.809 0.993	1<br>Osjap03g41270.1 0.009 0.387 0.378 0.191 0.227<br>Osjap08g25130.1 0.387 1 0.613 0.519 1	1<br>Orufi03g39770.1 0.009 0.387 0.378 0.204 0.242<br>Orufi08g26140.1 0.387 1 0.613 0.519 1	1<br>Oglab03g36140.1 0.009 0.387 0.378 0.191 0.227<br>Oglab03g37860.1 0.387 0.991 0.604 0.814 0.993	1<br>Obart03g38160.1 0.009 0.387 0.378 0.203 0.242<br>Obart03g39810.1 0.387 0.991 0.604 0.814 0.993	1<br>Olong_007014-R3 0.009 0.377 0.368 0.217 0.257<br>Olong_025249-R1 0.387 0.953 0.566 0.791 0.984	1<br>Opunc03g35260.1 0.009 0.377 0.368 0.248 0.293<br>Opunc03g37040.1 0.387 0.953 0.566 0.814 0.984	1<br>Obrac03g29980.1 0.387 0.943 0.556 0.802 0.981<br>Obrac08g08500.1 0.009 0.377 0.368 0.058 0.115	1<br>Lperr03g33960.1 0.387 0.953 0.566 0.826 0.985<br>Lperr08g10620.1 0.009 0.377 0.368 0.249 0.295	0
Osind09g01630.1	1<br>Osind09g01630.1 0.002 1 0.998 0.002 1	0	0	0	0	0	0	0	0	0
	1<br>Osind02g13100.1 0.52 1 0.48 0.664 1<br>Osind03g00750.1 0.002 0.435 0.433 0.004 1	1<br>Osjap03g00690.5 0.002 0.435 0.433 0.082 1<br>Osjap04g01220.1 0.52 1 0.48 0.654 1	1<br>Orufi03g12670.1 0.002 0.435 0.433 0.118 1<br>Orufi11g09320.2 0.52 1 0.48 0.686 1	1<br>Oglab02g11710.1 0.52 1 0.48 0.675 1<br>Oglab03g00400.1 0.002 0.435 0.433 0.004 1	1<br>Obart03g00560.3 0.002 0.485 0.483 0.107 1<br>Obart04g01130.3 0.52 1 0.48 0.67 1	0<br>Olong_019815-R1 0.414 1 0.586 0.222 1	1<br>Opunc02g10410.1 0.509 1 0.491 0.668 1<br>Opunc11g05910.3 0.002 0.435 0.433 0.004 1	1<br>Obrac03g00410.1 0.002 0.431 0.429 0.079 0.988<br>Obrac04g00850.1 0.516 0.998 0.482 0.654 1	1<br>Lperr02g09810.1 0.518 1 0.482 0.329 1<br>Lperr12g13320.1 0.002 0.435 0.433 0.004 1	1<br>AT2G17695.1 0.012 0.42 0.408 0.01 0.985<br>AT4G10510.1 0.52 0.992 0.472 0.668 0.995
Osind09g02760.1	1<br>Osind09g02760.1 0.001 1 0.999 0.001 1	1<br>Osjap04g11160.1 0.001 1 0.999 0.001 1	1<br>Orufi04g09840.1 0.001 1 0.999 0.001 1	0	0	0	1<br>Opunc04g07220.1 0.017 0.988 0.971 0.005 1	0	1<br>Lperr04g05910.1 0.066 0.993 0.927 0.022 0.997	0
	1<br>Osind01g17770.1 0.585 0.999 0.414 0.006 0.928<br>Osind08g20650.1 0.008 0.524 0.516 0.04 0.878	1<br>Osjap09g02370.1 0.585 0.999 0.414 0.006 0.928<br>Osjap11g14300.1 0.008 0.524 0.516 0.04 0.878	1<br>Orufi10g19500.1 0.563 0.988 0.425 0.022 0.976<br>Orufi11g12980.1 0.008 0.524 0.516 0.039 0.881	1<br>Oglab01g12700.1 0.585 0.999 0.414 0.006 0.928<br>Oglab11g10800.1 0.008 0.524 0.516 0.04 0.878	1<br>Obart04g08610.1 0.544 1 0.456 0.001 1<br>Obart11g12300.1 0.008 0.524 0.516 0.04 0.878	1<br>Olong_024054-R1 0.585 1 0.415 0.002 1<br>Olong_024055-R1 0.01 0.548 0.538 0.001 0.96	1<br>Opunc08g10560.1 0.563 0.986 0.423 0.07 0.993<br>Opunc11g10540.3 0.008 0.524 0.516 0.087 0.889	1<br>Obrac04g04850.1 0.008 0.556 0.548 0.043 0.975<br>Obrac10g11900.4 0.567 0.982 0.415 0.03 0.966	1<br>Lperr02g04400.1 0.555 0.967 0.412 0.048 0.961<br>Lperr11g10010.2 0.008 0.524 0.516 0.037 0.886	1<br>AT1G25360.1 0.57 0.996 0.426 0.019 0.914<br>AT5G44240.1 0.008 0.524 0.516 0.066 0.889
Osind09g03780.1	1<br>Osind09g03780.1 0.003 1 0.997 0.003 1	0	0	0	0	0	0	0	0	0
	1<br>Osind09g03790.1 0.003 0.678 0.675 0.002 0.578<br>Osind10g07140.1 0.702 0.921 0.219 0.016 0.313	1<br>Osjap01g42620.1 0.699 0.909 0.21 0.023 0.341<br>Osjap09g03660.1 0.003 0.678 0.675 0.002 0.578	1<br>Orufi03g41030.1 0.699 0.909 0.21 0.023 0.344<br>Orufi09g04010.1 0.003 0.678 0.675 0.002 0.578	0<br>Oglab05g09200.1 0.693 0.909 0.216 0.014 0.341	1<br>Obart01g37280.1 0.699 0.909 0.21 0.023 0.343<br>Obart09g03600.1 0.003 0.678 0.675 0.002 0.578	1<br>Olong_003911-R1 0.699 0.909 0.21 0.023 0.344<br>Olong_007585-R1 0.009 0.678 0.669 0.164 0.596	1<br>Opunc08g19220.1 0.702 0.909 0.207 0.033 0.352<br>Opunc09g03240.1 0.003 0.675 0.672 0.123 0.577	1<br>Obrac03g29450.2 0.699 0.909 0.21 0.023 0.333<br>Obrac09g01930.1 0.003 0.675 0.672 0.002 0.531	0<br>Lperr09g02590.1 0.003 0.717 0.714 0.135 0.61	1<br>AT3G12160.1 0.69 0.909 0.219 0.023 0.347<br>AT3G52120.1 0.009 0.672 0.663 0.005 0.485
Osind09g09040.1	1<br>Osind09g09040.1 0.001 1 0.999 0.001 1	1<br>Osjap11g06710.1 0.001 1 0.999 0.067 0.999<br>Osjap11g06720.1 0.001 0.999 0.998 0.054 0.981	1<br>Orufi11g05930.1 0.001 1 0.999 0.09 1<br>Orufi11g05940.1 0.001 1 0.999 0.002 1	1<br>Oglab11g05440.1 0.001 0.995 0.994 0.001 0.998	1<br>Obart11g05910.1 0.001 0.88 0.879 0.05 1	0	0	0	0	0
	1<br>Osind09g09050.1 0.001 0.481 0.48 0.104 0.994<br>Osind11g05650.1 0.522 1 0.478 0.002 1	1<br>Osjap01g24690.1 0.525 0.989 0.464 0.014 0.934<br>Osjap11g06740.8 0.022 0.484 0.462 0.013 0.907	1<br>Orufi11g05890.1 0.522 1 0.478 0.003 0.997<br>Orufi11g05900.2 0.001 0.481 0.48 0.002 0.978	1<br>Oglab08g10820.1 0.522 0.955 0.433 0.002 0.867<br>Oglab11g05430.1 0.001 0.503 0.502 0.049 0.944	1<br>Obart01g20930.1 0.525 0.989 0.464 0.006 0.498<br>Obart11g05930.1 0.001 0.481 0.48 0.002 0.998	1<br>Olong_000002-R1 0.001 0.517 0.516 0.117 0.996<br>Olong_005787-R1 0.522 1 0.478 0.002 1	0<br>Opunc11g05960.1 0.425 1 0.575 0.002 1	1<br>Obrac01g15800.1 0.521 0.989 0.468 0.004 0.915<br>Obrac11g03930.1 0.001 0.482 0.481 0.002 0.939	1<br>Lperr05g13220.1 0.522 1 0.478 0.002 1<br>Lperr11g05360.1 0.001 0.481 0.48 0.044 0.995	0<br>AT4G38130.1 0.522 0.955 0.433 0.002 0.782
Osind09g09810.2	1	1	1	0	0	0	1	0	1	0

	Osind09g09810.1 0.001 0.984 0.983 0.001 0.605<br>Osind09g09810.2 0.001 1 0.999 0.001 1	Osjap09g09660.1 0.001 0.984 0.983 0.001 0.615<br>Osjap09g09660.2 0.001 1 0.999 0.001 1<br>Osjap09g09660.3 0.001 1 0.999 0.001 1<br>Osjap09g09660.4 0.001 1 0.999 0.001 1	Orufi09g10340.1 0.001 0.984 0.983 0.001 0.67<br>Orufi09g10340.2 0.001 1 0.999 0.001 1<br>Orufi09g10340.3 0.001 1 0.999 0.001 1				Opunc09g08150.1 0.001 1 0.999 0.001 1		Lperr09g06610.1 0.001 1 0.999 0.001 0.802<br>Lperr09g06610.2 0.001 1 0.999 0.001 1	
	1<br>Osind02g00030.1 0.593 0.846 0.253 0.188 0.689<br>Osind08g18230.1 0.001 0.563 0.562 0.002 0.984	1<br>Osjap02g00030.1 0.593 0.846 0.253 0.196 0.692<br>Osjap08g16360.1 0.001 0.563 0.562 0.002 0.984	1<br>Orufi02g00020.1 0.593 0.846 0.253 0.282 0.725<br>Orufi08g17320.1 0.001 0.563 0.562 0.002 0.984	1<br>Oglab01g06450.1 0.656 0.997 0.341 0.674 0.999<br>Oglab08g14350.1 0.001 0.563 0.562 0.002 0.984	1<br>Obart08g15380.1 0.001 0.563 0.562 0.002 0.984<br>Obart09g09660.3 0.566 1 0.434 0.136 1	1<br>Olong_009135-R1 0.001 0.564 0.563 0.086 0.862<br>Olong_009135-R6 0.592 0.99 0.398 0.003 0.983	0<br>Opunc08g12760.1 0.001 0.563 0.562 0.002 0.984<br>Opunc11g05680.1 0.593 0.818 0.225 0.151 0.422	1<br>Obrac09g05000.3 0.592 1 0.408 0.174 1<br>Obrac09g05010.1 0.001 0.564 0.563 0.002 0.988	1<br>Lperr02g00030.2 0.593 0.846 0.253 0.27 0.728<br>Lperr08g12650.1 0.001 0.56 0.559 0.003 0.96	0<br>AT3G17880.1 0.608 1 0.392 0.195 0.997
Osind09g11870.1	1<br>Osind09g11870.1 0.004 1 0.996 0.004 1	0	0	0	0	0	0	0	0	0
	1<br>Osind08g19910.1 0.004 0.377 0.373 0.001 0.086<br>Osind09g11900.1 0.381 1 0.619 0.033 1	1<br>Osjap08g17850.1 0.004 0.377 0.373 0.001 0.086<br>Osjap10g04710.1 0.377 1 0.623 0.026 1	1<br>Orufi03g37310.1 0.377 0.97 0.593 0.112 0.689<br>Orufi09g12390.3 0.004 0.377 0.373 0.034 0.124	1<br>Oglab07g16860.1 0.381 1 0.619 0.021 1<br>Oglab09g10530.1 0.004 0.377 0.373 0.001 0.088	0<br>Obart07g18590.1 0.381 1 0.619 0.021 1	1<br>Olong_013737-R1 0.381 1 0.619 0.033 1<br>Olong_017956-R1 0.004 0.369 0.365 0.002 0.133	1<br>Opunc06g17610.1 0.381 1 0.619 0.082 1<br>Opunc09g10100.1 0.004 0.377 0.373 0.083 0.164	0<br>Obrac04g00470.1 0.419 0.89 0.471 0.121 0.983	0<br>Lperr05g07610.1 0.458 0.767 0.309 0.258 0.797<br>Lperr08g13980.1 0.004 0.377 0.373 0.001 0.093	0
Osind09g15570.1	1<br>Osind09g15570.1 0.002 1 0.998 0.002 1<br>Osind09g15570.2 0.002 1 0.998 0.002 1	1<br>Osjap09g15430.2 0.002 1 0.998 0.002 1<br>Osjap09g15430.3 0.002 1 0.998 0.002 1	1<br>Orufi09g16160.1 0.002 1 0.998 0.002 1<br>Orufi09g16160.2 0.002 1 0.998 0.002 1	1<br>Oglab09g13510.1 0.002 1 0.998 0.002 1<br>Oglab09g13510.2 0.002 1 0.998 0.002 1<br>Oglab09g13510.3 0.002 1 0.998 0.002 1<br>Oglab09g13510.4 0.002 1 0.998 0.002 1	1<br>Obart09g14980.1 0.002 1 0.998 0.002 1<br>Obart09g14980.2 0.002 1 0.998 0.002 1	0	1<br>Opunc09g13420.1 0.002 1 0.998 0.002 1<br>Opunc09g13420.2 0.002 1 0.998 0.002 1	1<br>Obrac09g09090.1 0.002 1 0.998 0.002 1	0	0
	1<br>Osind02g06940.1 0.01 0.54 0.53 0.01 0.997<br>Osind08g23920.1 0.544 0.993 0.449 0.131 0.988	1<br>Osjap02g06650.1 0.01 0.54 0.53 0.01 0.997<br>Osjap09g15430.1 0.544 1 0.456 0.131 1	1<br>Orufi04g24220.1 0.013 0.542 0.529 0.165 1<br>Orufi08g22880.1 0.544 0.993 0.449 0.131 0.988	1<br>Oglab08g19240.1 0.544 0.993 0.449 0.143 0.986<br>Oglab12g18680.1 0.002 0.542 0.54 0.003 1	1<br>Obart02g06180.1 0.01 0.54 0.53 0.01 0.997<br>Obart08g20390.1 0.544 0.993 0.449 0.631 0.988	1<br>Olong_013054-R1 0.547 0.992 0.445 0.149 0.979<br>Olong_024186-R1 0.002 0.542 0.54 0.003 1	1<br>Opunc04g20190.1 0.007 0.542 0.535 0.006 1<br>Opunc08g18560.1 0.544 0.993 0.449 0.128 0.969	1<br>Obrac02g04550.3 0.008 0.54 0.532 0.214 0.997<br>Obrac09g00560.1 0.548 0.998 0.45 0.164 0.966	1<br>Lperr04g18350.1 0.002 0.542 0.54 0.021 1<br>Lperr09g11120.1 0.544 1 0.456 0.156 1	1<br>AT1G68540.1 0.542 0.992 0.45 0.112 1<br>AT2G21270.3 0.003 0.54 0.537 0.059 0.997
Osind09g15570.2	1<br>Osind09g15570.1 0.002 1 0.998 0.002 1<br>Osind09g15570.2 0.002 1 0.998 0.002 1	1<br>Osjap09g15430.2 0.002 1 0.998 0.002 1<br>Osjap09g15430.3 0.002 1 0.998 0.002 1	1<br>Orufi09g16160.1 0.002 1 0.998 0.002 1<br>Orufi09g16160.2 0.002 1 0.998 0.002 1	1<br>Oglab09g13510.1 0.002 1 0.998 0.002 1<br>Oglab09g13510.2 0.002 1 0.998 0.002 1<br>Oglab09g13510.3 0.002 1 0.998 0.002 1<br>Oglab09g13510.4 0.002 1 0.998 0.002 1	1<br>Obart09g14980.1 0.002 1 0.998 0.002 1<br>Obart09g14980.2 0.002 1 0.998 0.002 1	0	1<br>Opunc09g13420.1 0.002 1 0.998 0.002 1<br>Opunc09g13420.2 0.002 1 0.998 0.002 1	1<br>Obrac09g09090.1 0.002 1 0.998 0.002 1	0	0
	1<br>Osind02g06940.1 0.01 0.565 0.555 0.01 0.997<br>Osind08g23920.1 0.569 0.993 0.424 0.131 0.988	1<br>Osjap02g06650.1 0.01 0.565 0.555 0.01 0.997<br>Osjap09g15430.1 0.569 1 0.431 0.131 1	1<br>Orufi04g24220.1 0.014 0.567 0.553 0.165 1<br>Orufi08g22880.1 0.569 0.993 0.424 0.131 0.988	1<br>Oglab08g19240.1 0.569 0.993 0.424 0.143 0.986<br>Oglab12g18680.1 0.002 0.567 0.565 0.003 1	1<br>Obart02g06180.1 0.01 0.565 0.555 0.01 0.997<br>Obart08g20390.1 0.569 0.993 0.424 0.631 0.988	1<br>Olong_008093-R2 0.596 0.991 0.395 0.25 0.996<br>Olong_024186-R1 0.002 0.567 0.565 0.003 1	1<br>Opunc04g20190.1 0.007 0.567 0.56 0.006 1<br>Opunc08g18560.1 0.569 0.993 0.424 0.128 0.969	1<br>Obrac01g09090.1 0.605 0.998 0.393 0.299 0.982<br>Obrac02g04550.3 0.009 0.565 0.556 0.214 0.997	1<br>Lperr04g18350.1 0.002 0.567 0.565 0.021 1<br>Lperr09g11120.1 0.569 1 0.431 0.156 1	1<br>AT1G68540.1 0.567 0.991 0.424 0.112 1<br>AT2G21270.3 0.003 0.565 0.562 0.059 0.997
Osind09g15930.1	1<br>Osind09g15930.1 0.001 1 0.999 0.001 1	0	1<br>Orufi09g16580.1 0.001 1 0.999 0.061 1	1<br>Oglab09g13920.1 0.001 1 0.999 0.061 1	1<br>Obart09g15360.1 0.057 1 0.943 0.001 1	0	0	0	0	0
	1<br>Osind08g24670.1 0.004 0.544 0.54 0.138 0.995<br>Osind09g02290.1 0.544 0.887 0.343 0.018 0.769	1<br>Osjap03g04840.3 0.544 0.887 0.343 0.018 0.769<br>Osjap09g15790.1 0.001 0.544 0.543 0.155 0.993	1<br>Orufi03g04550.5 0.544 0.887 0.343 0.017 0.733<br>Orufi08g23480.1 0.004 0.544 0.54 0.146 0.995	1<br>Oglab02g15910.1 0.037 0.512 0.475 0.001 0.988<br>Oglab09g02290.1 0.544 0.887 0.343 0.02 0.789	1<br>Obart03g04820.1 0.569 0.887 0.318 0.1 0.796<br>Obart08g21020.1 0.056 0.544 0.488 0.086 0.994	1<br>Olong_025005-R2 0.571 0.98 0.409 0.001 0.955<br>Olong_025006-R1 0.105 0.544 0.439 0.031 0.99	1<br>Opunc08g19030.2 0.006 0.544 0.538 0.071 0.976<br>Opunc09g13790.1 0.544 0.98 0.436 0.055 0.958	1<br>Obrac08g14010.2 0.036 0.544 0.508 0.001 0.989<br>Obrac09g09380.2 0.544 0.98 0.436 0.01 0.954	1<br>Lperr09g11500.1 0.001 0.544 0.543 0.105 0.992<br>Lperr09g11510.2 0.544 0.98 0.436 0.01 0.958	1<br>AT2G32000.1 0.544 0.978 0.434 0.012 0.961<br>AT2G43900.1 0.002 0.519 0.517 0.083 0.843
Osind09g20560.1	1<br>Osind09g20560.1 0.001 1 0.999 0.001 1	1<br>Osjap09g20460.1 0.001 0.998 0.997 0.001 0.986	1<br>Orufi09g21510.1 0.001 0.978 0.977 0.001 0.999	1<br>Oglab09g18200.1 0.001 0.998 0.997 0.001 0.985	1<br>Obart09g19980.1 0.067 0.998 0.931 0.165 0.99	1<br>Olong_014589-R1 0.117 0.985 0.868 0.018 0.991	0	0	0	0

					Obart09g19980.2 0.001 0.998 0.997 0.001 0.984	Olong_014589-R2 0.117 0.985 0.868 0.018 0.991				
	1<br>Osind02g21430.2 0.024 0.581 0.557 0.096 0.943<br>Osind09g20550.1 0.623 0.998 0.375 0.032 0.982	1<br>Osjap02g21530.2 0.024 0.581 0.557 0.096 0.943<br>Osjap09g20430.1 0.623 0.998 0.375 0.085 0.962	1<br>Orufi02g19810.2 0.024 0.581 0.557 0.096 0.943<br>Orufi09g21500.1 0.623 0.998 0.375 0.085 0.962	1<br>Oglab02g17850.1 0.024 0.581 0.557 0.095 0.914<br>Oglab09g18170.1 0.623 0.998 0.375 0.079 0.966	1<br>Obart04g25370.1 0.047 0.589 0.542 0.176 0.967<br>Obart09g19950.1 0.623 0.998 0.375 0.079 0.966	1<br>Olong_014116-R1 0.064 0.589 0.525 0.069 0.957<br>Olong_027417-R1 0.625 0.982 0.357 0.135 0.711	1<br>Opunc09g18280.1 0.618 0.998 0.38 0.058 0.841<br>Opunc09g18290.2 0.001 0.59 0.589 0.001 0.97	1<br>Obrac09g13140.3 0.623 0.998 0.375 0.049 0.806<br>Obrac09g13160.3 0.001 0.596 0.595 0.001 0.996	1<br>Lperr09g15890.1 0.622 0.999 0.377 0.048 0.95<br>Lperr09g15920.1 0.001 0.59 0.589 0.001 0.957	0<br>AT1G18160.1 0.021 0.589 0.568 0.064 0.958
Osind10g02110.1	1<br>Osind10g02110.1 0.001 1 0.999 0.001 1	0	0	1<br>Oglab10g02440.1 0.001 0.972 0.971 0.001 0.974<br>Oglab10g02440.2 0.001 0.952 0.951 0.001 0.972	1<br>Obart10g02510.1 0.001 0.972 0.971 0.001 0.972<br>Obart10g02510.2 0.001 0.952 0.951 0.001 0.971	0	0	0	0	0
	1<br>Osind10g00350.1 0.001 0.541 0.54 0.001 0.914<br>Osind11g07740.1 0.546 0.952 0.406 0.003 0.934	1<br>Osjap10g00540.1 0.001 0.541 0.54 0.001 0.924<br>Osjap10g02950.1 0.546 0.896 0.35 0.002 0.928	1<br>Orufi10g02500.1 0.027 0.541 0.514 0.028 0.958<br>Orufi11g08730.5 0.655 0.952 0.297 0.212 0.943	0<br>Oglab10g02440.4 0.001 0.541 0.54 0.002 0.774	1<br>Obart10g00500.1 0.001 0.541 0.54 0.001 0.925<br>Obart11g08380.1 0.655 0.92 0.265 0.194 0.911	1<br>Olong_016648-R3 0.355 0.972 0.617 0.007 0.993<br>Olong_028710-R1 0.037 0.348 0.311 0.004 0.979	1<br>Opunc10g02120.1 0.001 0.541 0.54 0.001 0.921<br>Opunc11g08150.1 0.546 0.92 0.374 0.003 0.898	1<br>Obrac03g10950.1 0.001 0.541 0.54 0.001 0.916<br>Obrac11g05340.1 0.546 0.919 0.373 0.002 0.909	1<br>Lperr10g00410.1 0.001 0.541 0.54 0.003 0.929<br>Lperr10g01950.1 0.546 0.92 0.374 0.003 0.882	0<br>AT1G21230.1 0.006 0.391 0.385 0.01 0.419<br>AT4G00830.1 0.648 0.919 0.271 0.194 0.891
Osind10g19230.1	1<br>Osind10g19230.1 0.002 1 0.998 0.002 1	0	1<br>Orufi10g20960.1 0.002 1 0.998 0.002 1	1<br>Oglab10g16090.1 0.002 0.992 0.99 0.001 0.861	1<br>Obart10g19720.1 0.002 1 0.998 0.002 1	0	1<br>Opunc10g18180.1 0.002 0.992 0.99 0.002 0.85	0	0	0
	1<br>Osind01g20890.1 0.519 0.992 0.473 0.154 0.668<br>Osind08g03990.1 0.046 0.464 0.418 0.057 0.95	1<br>Osjap10g22750.1 0.509 0.992 0.483 0.061 0.836<br>Osjap10g22760.1 0.002 0.486 0.484 0.003 0.993	1<br>Orufi01g19490.1 0.519 0.992 0.473 0.171 0.72<br>Orufi08g04140.1 0.046 0.464 0.418 0.057 0.95	1<br>Oglab01g14760.1 0.519 0.992 0.473 0.2 0.827<br>Oglab08g03250.2 0.046 0.464 0.418 0.052 0.955	1<br>Obart01g17550.1 0.519 0.992 0.473 0.174 0.721<br>Obart08g03750.1 0.046 0.464 0.418 0.057 0.95	1<br>Olong_006312-R1 0.516 0.986 0.47 0.067 0.835<br>Olong_006313-R1 0.002 0.486 0.484 0.003 0.993	1<br>Opunc01g18040.1 0.519 0.992 0.473 0.174 0.822<br>Opunc08g03700.1 0.017 0.464 0.447 0.011 0.951	1<br>Obrac01g13330.1 0.525 0.992 0.467 0.196 0.825<br>Obrac10g13120.1 0.002 0.486 0.484 0.004 0.993	1<br>Lperr10g15590.1 0.516 0.992 0.476 0.07 0.79<br>Lperr10g15600.1 0.002 0.482 0.48 0.003 0.979	1<br>AT1G32080.1 0.521 0.988 0.467 0.188 0.82<br>AT2G32260.1 0.044 0.465 0.421 0.078 0.825
Osind11g00690.1	1<br>Osind11g00690.1 0.001 1 0.999 0.001 1<br>Osind12g01290.1 0.001 0.999 0.998 0.001 0.954	1<br>Osjap11g01220.1 0.001 0.999 0.998 0.001 0.955<br>Osjap12g01140.1 0.001 0.999 0.998 0.001 0.953	1<br>Orufi11g01000.1 0.001 0.999 0.998 0.001 0.951<br>Orufi12g01130.1 0.001 0.999 0.998 0.001 0.953	1<br>Oglab12g00800.1 0.001 0.999 0.998 0.001 0.956	1<br>Obart11g01060.1 0.001 0.999 0.998 0.001 0.954	0	1<br>Opunc12g00940.1 0.001 0.999 0.998 0.001 0.937	0	0	0
	1<br>Osind03g12930.1 0.523 0.999 0.476 0.121 0.604<br>Osind07g27070.1 0.007 0.462 0.455 0.006 0.946	1<br>Osjap03g13100.1 0.523 0.999 0.476 0.097 0.593<br>Osjap07g30390.1 0.01 0.462 0.452 0.013 0.944	1<br>Orufi03g17220.1 0.53 0.999 0.469 0.027 0.912<br>Orufi07g27420.1 0.01 0.462 0.452 0.013 0.946	1<br>Oglab03g12120.1 0.523 0.999 0.476 0.121 0.604<br>Oglab07g22980.1 0.007 0.462 0.455 0.007 0.94	1<br>Obart03g12120.1 0.523 0.999 0.476 0.121 0.604<br>Obart12g00790.1 0.001 0.462 0.461 0.002 0.949	1<br>Olong_007715-R1 0.527 0.999 0.472 0.009 0.911<br>Olong_008211-R1 0.007 0.462 0.455 0.006 0.946	1<br>Opunc03g11680.1 0.523 0.999 0.476 0.148 0.924<br>Opunc11g00920.1 0.001 0.462 0.461 0.142 0.951	1<br>Obrac03g09720.2 0.523 0.999 0.476 0.142 0.923<br>Obrac12g00780.1 0.001 0.462 0.461 0.11 0.953	0<br>Lperr11g00920.1 0.001 0.462 0.461 0.002 0.949	1<br>AT2G34940.1 0.001 0.462 0.461 0.002 0.955<br>AT3G46740.1 0.526 1 0.474 0.161 0.928
Osind11g01990.1	1<br>Osind11g01990.1 0.002 1 0.998 0.002 1<br>Osind12g02780.1 0.002 1 0.998 0.002 1	1<br>Osjap11g02640.1 0.002 1 0.998 0.002 1<br>Osjap12g02610.1 0.002 1 0.998 0.002 1	1<br>Orufi11g02320.1 0.002 1 0.998 0.013 1<br>Orufi12g02480.1 0.002 1 0.998 0.002 1	1<br>Oglab11g02200.1 0.002 1 0.998 0.002 1<br>Oglab12g02130.1 0.002 1 0.998 0.002 1	1<br>Obart12g02080.1 0.023 1 0.977 0.002 1	0	0	0	0	0
	1<br>Osind11g02010.1 0.014 0.669 0.655 0.001 0.994<br>Osind12g10020.1 0.673 0.949 0.276 0.055 0.417	1<br>Osjap07g04360.1 0.033 0.669 0.636 0.018 0.925<br>Osjap12g12870.1 0.673 0.949 0.276 0.055 0.417	1<br>Orufi12g02470.1 0.045 0.669 0.624 0.04 0.988<br>Orufi12g10750.1 0.671 0.949 0.278 0.045 0.364	1<br>Oglab07g03810.1 0.033 0.669 0.636 0.019 0.987<br>Oglab12g09880.1 0.673 0.949 0.276 0.051 0.385	0<br>Obart11g02350.1 0.021 0.669 0.648 0.024 0.993	1<br>Olong_012584-R1 0.066 0.622 0.556 0.002 0.965<br>Olong_017316-R1 0.671 0.949 0.278 0.045 0.365	1<br>Opunc11g02190.1 0.002 0.669 0.667 0.003 0.989<br>Opunc12g08740.1 0.671 0.949 0.278 0.04 0.324	1<br>Obrac05g10860.1 0.051 0.669 0.618 0.097 0.987<br>Obrac12g07170.1 0.671 0.94 0.269 0.071 0.57	1<br>Lperr12g02190.1 0.002 0.669 0.667 0.003 0.989<br>Lperr12g08370.1 0.669 0.949 0.28 0.047 0.391	0<br>AT5G27320.1 0.014 0.643 0.629 0.003 0.98
Osind11g02250.1	1<br>Osind11g02250.1 0.002 1 0.998 0.002 1	0	0	0	0	0	0	0	0	0
	1<br>Osind08g20070.1 0.147 0.416 0.269 0.719 0.937<br>Osind11g02130.1 0.442 1 0.558 0.113 1	1<br>Osjap09g12000.1 0.071 0.404 0.333 0.655 0.926<br>Osjap12g02750.1 0.44 1 0.56 0.008 1	1<br>Orufi12g02610.1 0.44 1 0.56 0.008 1<br>Orufi12g02670.4 0.002 0.431 0.429 0.127 0.918	1<br>Oglab08g15880.1 0.064 0.416 0.352 0.648 0.937<br>Oglab11g02320.1 0.442 1 0.558 0.113 1	1<br>Obart09g02750.1 0.058 0.404 0.346 0.659 0.928<br>Obart12g02200.1 0.44 1 0.56 0.008 1	1<br>Olong_007877-R1 0.053 0.404 0.351 0.581 0.919<br>Olong_021332-R1 0.442 1 0.558 0.113 1	1<br>Opunc03g10180.1 0.1 0.418 0.318 0.367 0.968<br>Opunc11g02310.1 0.44 1 0.56 0.008 1	1<br>Obrac08g13200.1 0.149 0.46 0.311 0.609 0.964<br>Obrac11g01530.1 0.498 0.991 0.493 0.385 0.997	0<br>Lperr02g17220.1 0.06 0.46 0.4 0.632 0.986	0<br>AT3G26330.1 0.033 0.404 0.371 0.588 0.934<br>AT5G03990.1 0.427 0.678 0.251 0.089 0.467
Osind11g17120.1	1	1	1	0	0	0	0	0	0	0

	Osind11g17120.1 0.004 1 0.996 0.004 1	Osjap11g23010.1 0.004 0.992 0.988 0.002 0.589	Orufi11g21270.1 0.004 0.992 0.988 0.002 0.538							
	1<br>Osind07g22620.1 0.062 0.619 0.557 0.157 0.93<br>Osind11g17350.1 0.623 0.992 0.369 0.433 0.864	1<br>Osjap10g07190.1 0.012 0.669 0.657 0.132 0.882<br>Osjap11g23280.1 0.669 0.992 0.323 0.285 0.467	1<br>Orufi10g06540.1 0.012 0.669 0.657 0.132 0.882<br>Orufi11g21510.1 0.669 0.992 0.323 0.285 0.467	1<br>Oglab10g05750.1 0.012 0.669 0.657 0.128 0.882<br>Oglab11g17490.1 0.669 0.992 0.323 0.285 0.467	1<br>Obart04g09320.1 0.767 0.992 0.225 0.376 0.428<br>Obart10g06170.1 0.012 0.669 0.657 0.132 0.882	1<br>Olong_020397-R1 0.004 0.584 0.58 0.006 0.787<br>Olong_023942-R1 0.669 0.992 0.323 0.349 0.515	1<br>Opunc06g04020.1 0.77 0.996 0.226 0.534 0.607<br>Opunc10g05480.1 0.012 0.669 0.657 0.132 0.882	1<br>Obrac04g04990.1 0.759 0.992 0.233 0.158 0.31<br>Obrac10g03800.1 0.012 0.611 0.599 0.155 0.885	1<br>Lperr04g06250.2 0.759 0.992 0.233 0.709 0.762<br>Lperr11g04260.1 0.027 0.626 0.599 0.249 0.427	0<br>AT2G21110.1 0.031 0.615 0.584 0.129 0.941
Osind11g17390.1	1<br>Osind11g17350.1 0.07 0.98 0.91 0.001 0.992<br>Osind11g17390.1 0.002 1 0.998 0.002 1	0	1<br>Orufi11g21490.1 0.002 0.997 0.995 0.088 0.76	0	0	0	0	0	0	0
	1<br>Osind11g17360.1 0.449 0.997 0.548 0.156 0.559<br>Osind11g17370.1 0.07 0.429 0.359 0.24 0.938	1<br>Osjap11g23270.1 0.449 0.997 0.548 0.184 0.651<br>Osjap11g23280.1 0.07 0.429 0.359 0.456 0.955	1<br>Orufi10g18830.1 0.076 0.437 0.361 0.48 0.821<br>Orufi11g21400.1 0.443 0.974 0.531 0.187 0.524	1<br>Oglab11g17470.1 0.429 0.98 0.551 0.042 0.513<br>Oglab11g17490.1 0.047 0.429 0.382 0.42 0.955	1<br>Obart02g27080.1 0.049 0.437 0.388 0.303 0.902<br>Obart03g04520.1 0.46 0.983 0.523 0.004 0.325	1<br>Olong_023942-R1 0.07 0.429 0.359 0.506 0.959<br>Olong_027580-R1 0.429 0.98 0.551 0.054 0.519	1<br>Opunc10g16000.1 0.073 0.423 0.35 0.642 0.876<br>Opunc11g04080.1 0.423 0.983 0.56 0.038 0.488	1<br>Obrac09g13360.3 0.061 0.431 0.37 0.63 0.894<br>Obrac11g11670.1 0.455 0.973 0.518 0.002 0.429	0<br>Lperr11g16280.5 0.426 0.992 0.566 0.046 0.496	0<br>AT1G16760.1 0.023 0.429 0.406 0.611 0.933
Osind11g17970.1	1<br>Osind11g17970.1 0.002 1 0.998 0.002 1	0	0	0	0	0	0	0	0	0
	1<br>Osind04g07350.1 0.13 0.624 0.494 0.581 0.989<br>Osind11g17960.1 0.69 1 0.31 0.682 1	1<br>Osjap11g25490.1 0.69 0.995 0.305 0.595 0.872<br>Osjap11g25500.1 0.002 0.661 0.659 0.003 0.993	1<br>Orufi11g23670.1 0.69 1 0.31 0.682 1<br>Orufi11g23680.1 0.002 0.661 0.659 0.003 0.995	1<br>Oglab11g18980.1 0.69 1 0.31 0.682 1<br>Oglab11g18990.1 0.002 0.661 0.659 0.099 0.994	1<br>Obart11g21800.1 0.69 1 0.31 0.682 1<br>Obart11g21810.1 0.002 0.661 0.659 0.098 0.994	1<br>Olong_008005-R1 0.69 0.998 0.308 0.664 0.998<br>Olong_009981-R1 0.059 0.626 0.567 0.343 0.894	0<br>Opunc11g18100.1 0.207 0.63 0.423 0.284 0.977<br>Opunc11g18340.1 0.69 0.998 0.308 0.682 0.998	0<br>Obrac11g12550.1 0.153 0.623 0.47 0.563 0.978<br>Obrac11g12870.1 0.675 0.998 0.323 0.641 0.998	1<br>Lperr04g06180.1 0.033 0.63 0.597 0.525 0.998<br>Lperr11g18340.1 0.697 0.998 0.301 0.693 0.998	0
Osind12g01290.1	1<br>Osind11g00690.1 0.001 0.954 0.953 0.001 0.999<br>Osind12g01290.1 0.001 1 0.999 0.001 1	1<br>Osjap11g01220.1 0.001 1 0.999 0.001 1<br>Osjap12g01140.1 0.001 1 0.999 0.001 1	1<br>Orufi11g01000.1 0.001 1 0.999 0.001 1<br>Orufi12g01130.1 0.001 1 0.999 0.001 1	1<br>Oglab12g00800.1 0.001 1 0.999 0.001 1	1<br>Obart11g01060.1 0.001 1 0.999 0.001 1	0	1<br>Opunc12g00940.1 0.001 1 0.999 0.001 1	0	0	0
	1<br>Osind03g12930.1 0.502 0.981 0.479 0.121 0.633<br>Osind07g27070.1 0.007 0.37 0.363 0.006 0.783	1<br>Osjap03g13100.1 0.502 0.981 0.479 0.097 0.622<br>Osjap07g30390.1 0.01 0.366 0.356 0.013 0.798	1<br>Orufi03g17220.1 0.509 1 0.491 0.027 1<br>Orufi07g27420.1 0.01 0.37 0.36 0.013 0.783	1<br>Oglab03g12120.1 0.502 0.981 0.479 0.121 0.633<br>Oglab07g22980.1 0.007 0.366 0.359 0.007 0.783	1<br>Obart03g12120.1 0.502 0.981 0.479 0.121 0.633<br>Obart10g06750.1 0.021 0.401 0.38 0.151 0.885	1<br>Olong_007715-R1 0.505 1 0.495 0.007 1<br>Olong_008211-R1 0.007 0.37 0.363 0.006 0.783	1<br>Opunc03g11680.1 0.502 1 0.498 0.148 1<br>Opunc11g00920.1 0.001 0.37 0.369 0.142 0.816	1<br>Obrac03g09720.2 0.502 1 0.498 0.142 1<br>Obrac10g04150.1 0.019 0.398 0.379 0.036 0.868	1<br>Lperr03g10720.1 0.502 0.993 0.491 0.148 0.951<br>Lperr11g00920.1 0.001 0.37 0.369 0.002 0.792	1<br>AT2G34940.1 0.001 0.37 0.369 0.002 0.793<br>AT3G46740.1 0.505 1 0.495 0.161 1
Osind12g02780.1	1<br>Osind11g01990.1 0.002 1 0.998 0.002 1<br>Osind12g02780.1 0.002 1 0.998 0.002 1	1<br>Osjap11g02640.1 0.002 1 0.998 0.002 1<br>Osjap12g02610.1 0.002 1 0.998 0.002 1	1<br>Orufi11g02320.1 0.002 1 0.998 0.003 1<br>Orufi12g02480.1 0.002 1 0.998 0.002 1	1<br>Oglab11g02200.1 0.002 1 0.998 0.002 1<br>Oglab12g02130.1 0.002 1 0.998 0.002 1	1<br>Obart12g02080.1 0.022 1 0.978 0.002 1	0	0	0	0	0
	1<br>Osind11g02010.1 0.013 0.64 0.627 0.001 0.994<br>Osind12g10020.1 0.651 0.953 0.302 0.022 0.417	1<br>Osjap07g04360.1 0.031 0.64 0.609 0.018 0.925<br>Osjap12g12870.1 0.651 0.953 0.302 0.022 0.417	1<br>Orufi07g03650.1 0.031 0.64 0.609 0.019 0.976<br>Orufi12g10910.1 0.651 0.953 0.302 0.022 0.417	1<br>Oglab07g03810.1 0.031 0.64 0.609 0.019 0.987<br>Oglab12g09880.1 0.651 0.953 0.302 0.02 0.385	0<br>Obart11g02350.1 0.02 0.64 0.62 0.004 0.993	1<br>Olong_012584-R1 0.061 0.597 0.536 0.002 0.965<br>Olong_017316-R1 0.651 0.953 0.302 0.017 0.365	1<br>Opunc11g02190.1 0.002 0.64 0.638 0.003 0.989<br>Opunc12g08740.1 0.651 0.953 0.302 0.015 0.324	1<br>Obrac05g10860.1 0.047 0.64 0.593 0.097 0.987<br>Obrac12g07170.1 0.651 0.944 0.293 0.029 0.57	1<br>Lperr12g02190.1 0.002 0.64 0.638 0.003 0.989<br>Lperr12g08380.1 0.644 0.987 0.343 0.01 0.488	0<br>AT5G16080.1 0.047 0.644 0.597 0.081 0.994
Osind12g09530.1	1<br>Osind12g09530.1 0.001 1 0.999 0.001 1	1<br>Osjap12g12320.1 0.001 1 0.999 0.001 1	1<br>Orufi12g10460.1 0.001 1 0.999 0.001 1	1<br>Oglab12g09470.1 0.001 1 0.999 0.001 1	1<br>Obart12g09410.1 0.001 1 0.999 0.001 1	0	0	0	0	0
	1<br>Osind01g46040.1 0.453 0.998 0.545 0.151 0.995<br>Osind03g07670.1 0.001 0.292 0.291 0.004 0.996	1<br>Osjap01g44780.2 0.453 0.998 0.545 0.095 0.994<br>Osjap03g07600.1 0.001 0.292 0.291 0.004 0.996	1<br>Orufi01g42650.3 0.453 0.998 0.545 0.195 0.995<br>Orufi03g07180.1 0.001 0.292 0.291 0.004 0.996	0<br>Oglab01g34040.1 0.502 0.998 0.496 0.203 0.995<br>Oglab03g07280.1 0.001 0.292 0.291 0.004 0.996	1<br>Obart01g39260.3 0.469 0.998 0.529 0.041 0.994<br>Obart03g07210.1 0.001 0.292 0.291 0.004 0.996	1<br>Olong_021912-R1 0.47 1 0.53 0.099 1<br>Olong_021913-R1 0.001 0.291 0.29 0.004 0.996	1<br>Opunc12g08570.1 0.334 1 0.666 0.017 1<br>Opunc12g08580.1 0.001 0.291 0.29 0.004 0.996	1<br>Obrac03g05880.1 0.001 0.291 0.29 0.004 0.996<br>Obrac12g06500.1 0.475 1 0.525 0.103 1	1<br>Lperr01g33810.2 0.469 0.998 0.529 0.002 0.994<br>Lperr03g06600.1 0.001 0.291 0.29 0.004 0.996	0<br>AT4G34670.1 0.001 0.283 0.282 0.004 0.966<br>AT5G67540.2 0.502 0.993 0.491 0.053 1
Osind12g10670.1	1	1	1	1	1	0	1	0	0	0

	Osind01g32860.1 0.007 0.915 0.908 0.007 0.854<br>Osind12g10660.1 0.007 0.993 0.986 0.007 0.993<br>Osind12g10670.1 0.007 1 0.993 0.007 1	Osjap01g31400.1 0.007 0.915 0.908 0.006 0.818<br>Osjap12g13600.1 0.007 0.993 0.986 0.007 0.993	Orufi01g30130.1 0.007 0.915 0.908 0.007 0.854	Oglab01g22960.1 0.071 0.915 0.844 0.194 0.874	Obart01g27080.1 0.071 0.915 0.844 0.194 0.874<br>Obart12g10510.1 0.007 0.993 0.986 0.012 0.988		Opunc10g03560.1 0.035 0.844 0.809 0.016 0.248			
	1<br>Osind03g19770.1 0.007 0.411 0.404 0.002 0.412<br>Osind04g15200.1 0.44 0.972 0.532 0.042 0.368	1<br>Osjap01g05590.1 0.014 0.326 0.312 0.017 0.556<br>Osjap02g24490.1 0.369 0.908 0.539 0.591 0.779	0<br>Orufi12g11520.1 0.007 0.794 0.787 0.008 0.882	1<br>Oglab01g20040.1 0.035 0.34 0.305 0.022 0.11<br>Oglab06g05230.1 0.404 0.915 0.511 0.669 0.816	1<br>Obart01g16250.1 0.411 0.908 0.497 0.765 0.869<br>Obart03g17930.1 0.007 0.411 0.404 0.002 0.126	1<br>Olong_000447-R1 0.39 0.908 0.518 0.691 0.824<br>Olong_003316-R1 0.035 0.34 0.305 0.021 0.103	1<br>Opunc03g17210.1 0.007 0.333 0.326 0.002 0.095<br>Opunc06g05170.1 0.404 0.915 0.511 0.649 0.802	0<br>Obrac01g31700.1 0.184 0.908 0.724 0.829 0.924	1<br>Lperr03g05500.1 0.411 0.915 0.504 0.585 0.769<br>Lperr03g15640.1 0.007 0.333 0.326 0.002 0.09	1<br>AT2G36780.1 0.035 0.333 0.298 0.028 0.113<br>AT5G50380.1 0.355 0.908 0.553 0.706 0.82
Osind12g16420.1	1<br>Osind12g16420.1 0.002 1 0.998 0.002 1	0	0	0	0	0	0	0	0	0
	1<br>Osind02g10370.1 0.4 0.972 0.572 0.094 0.963<br>Osind06g09400.1 0.002 0.274 0.272 0.373 0.873	1<br>Osjap09g01510.1 0.002 0.212 0.21 0.397 0.804<br>Osjap12g20330.1 0.304 1 0.696 0.037 1	1<br>Orufi12g17680.1 0.002 0.265 0.263 0.005 1<br>Orufi12g17700.1 0.304 1 0.696 0.037 1	1<br>Oglab06g08180.1 0.002 0.274 0.272 0.373 0.873<br>Oglab12g14860.1 0.304 1 0.696 0.037 1	1<br>Obart09g01470.1 0.002 0.212 0.21 0.026 0.684<br>Obart12g15930.1 0.304 1 0.696 0.034 1	1<br>Olong_005120-R1 0.304 1 0.696 0.037 1<br>Olong_008551-R1 0.002 0.209 0.207 0.365 0.786	1<br>Opunc09g01310.1 0.002 0.212 0.21 0.379 0.821<br>Opunc12g14360.1 0.304 1 0.696 0.14 1	1<br>Obrac06g15120.1 0.002 0.252 0.25 0.308 0.869<br>Obrac12g10160.1 0.304 0.997 0.693 0.083 0.994	1<br>Lperr06g18350.1 0.002 0.252 0.25 0.391 0.883<br>Lperr12g13120.1 0.304 1 0.696 0.034 1	1<br>AT1G06990.1 0.005 0.265 0.26 0.386 0.908<br>AT1G09300.1 0.316 0.974 0.658 0.01 0.937

(((((((_a1,rufi),_a2),longi),punctata),brachy),leersia),tair);

chimeric gene id	INGROUP		MIDGROUP					OUTGROUP		
	jap	indica	rufi	glab	barthii	longi	punctata	brachy	leersia	tair
Osind01g02220.1	0	1<br>Osind01g02220.1 0.001 1 0.999 0.001 1	0	1<br>Oglab01g01590.1 0.056 0.987 0.931 0.001 0.999	1<br>Obart01g01710.1 0.056 0.987 0.931 0.001 0.986	1<br>Olong_021362-R1 0.043 1 0.957 0.072 1	1<br>Opunc01g01490.1 0.056 0.999 0.943 0.056 0.988<br>Opunc01g01490.2 0.043 0.999 0.956 0.042 0.988	0	0	0
	0<br>Osjap01g02100.2 0.497 1 0.503 0.017 1	1<br>Osind01g02240.1 0.007 0.498 0.491 0.073 0.999<br>Osind05g03930.1 0.505 1 0.495 0.104 1	1<br>Orufi01g01810.1 0.503 1 0.497 0.002 1<br>Orufi01g01830.3 0.007 0.499 0.492 0.143 1	1<br>Oglab01g01570.1 0.035 0.499 0.464 0.101 1<br>Oglab05g03370.1 0.531 1 0.469 0.154 1	1<br>Obart01g01680.1 0.035 0.499 0.464 0.11 1<br>Obart05g03720.1 0.505 1 0.495 0.104 1	1<br>Olong_016471-R1 0.505 0.991 0.486 0.106 0.98<br>Olong_021363-R1 0.035 0.499 0.464 0.032 1	1<br>Opunc01g01500.1 0.053 0.498 0.445 0.198 0.998<br>Opunc05g03460.1 0.531 0.997 0.466 0.153 0.99	0<br>Obrac01g17910.1 0.479 1 0.521 0.202 1	1<br>Lperr01g01610.1 0.523 1 0.477 0.058 0.998<br>Lperr01g01640.1 0.023 0.498 0.475 0.157 0.998	1<br>AT1G23010.1 0.056 0.498 0.442 0.134 0.998<br>AT3G48670.1 0.503 1 0.497 0.114 0.995
Osind01g06920.1	0	1<br>Osind01g06920.1 0.001 1 0.999 0.001 1	0	0	0	0	0	0	0	0
	1<br>Osjap01g06890.1 0.001 0.366 0.365 0.004 0.977<br>Osjap01g06970.1 0.367 1 0.633 0.205 1	0<br>Osind01g06840.1 0.001 0.366 0.365 0.249 0.982	1<br>Orufi01g06470.1 0.001 0.366 0.365 0.005 0.97<br>Orufi01g06560.1 0.367 1 0.633 0.206 1	1<br>Oglab01g05560.1 0.001 0.366 0.365 0.251 0.982<br>Oglab01g05640.1 0.367 1 0.633 0.206 1	1<br>Obart01g05980.1 0.367 1 0.633 0.205 1<br>Obart04g25710.1 0.074 0.367 0.293 0.106 0.851	0<br>Olong_013045-R1 0.359 0.889 0.53 0.245 0.716<br>Olong_027964-R1 0.001 0.21 0.209 0.004 0.581	1<br>Opunc01g05790.1 0.367 1 0.633 0.234 1<br>Opunc08g05740.1 0.073 0.367 0.294 0.099 0.854	0<br>Obrac01g04620.1 0.399 0.989 0.59 0.021 0.993	1<br>Lperr01g05690.1 0.367 0.989 0.622 0.176 0.994<br>Lperr04g20820.1 0.074 0.336 0.262 0.084 0.768	0<br>AT3G21300.1 0.367 0.999 0.632 0.22 0.998
Osind01g08490.1	1<br>Osjap01g08660.1 0.001 1 0.999 0.001 1	1<br>Osind01g08490.1 0.001 1 0.999 0.001 1	1<br>Orufi01g08070.1 0.001 1 0.999 0.001 1	1<br>Oglab01g06470.1 0.001 1 0.999 0.001 1	1<br>Obart01g07430.1 0.017 1 0.983 0.001 1	1<br>Olong_011567-R1 0.07 1 0.93 0.001 1	0	0	0	0
	1<br>Osjap01g29230.2 0.114 0.581 0.467 0.25 0.983<br>Osjap05g00560.2 0.647 0.997 0.35 0.146 1	1<br>Osind01g30690.1 0.114 0.581 0.467 0.267 0.982<br>Osind05g00540.1 0.647 0.997 0.35 0.126 1	1<br>Orufi01g27950.1 0.114 0.581 0.467 0.25 0.983<br>Orufi01g48560.2 0.647 0.98 0.333 0.085 0.984	1<br>Oglab01g21070.2 0.114 0.581 0.467 0.249 0.983<br>Oglab05g00530.1 0.647 0.997 0.35 0.146 1	1<br>Obart01g25090.1 0.114 0.581 0.467 0.219 0.982<br>Obart05g00540.1 0.647 0.997 0.35 0.146 1	0<br>Olong_003039-R1 0.647 0.997 0.35 0.128 1<br>Olong_003692-R2 0.153 0.581 0.428 0.244 0.979	1<br>Opunc01g07170.1 0.001 0.582 0.581 0.001 0.984<br>Opunc05g00510.2 0.647 0.997 0.35 0.15 1	1<br>Obrac01g05760.3 0.583 1 0.417 0.082 1<br>Obrac01g05770.3 0.001 0.582 0.581 0.001 0.985	1<br>Lperr01g07030.1 0.583 1 0.417 0.018 1<br>Lperr01g07040.2 0.001 0.582 0.581 0.001 0.989	1<br>AT1G54350.1 0.585 0.993 0.408 0.084 0.975<br>AT5G53430.1 0.024 0.581 0.557 0.09 0.984
Osind01g11290.1	0	1<br>Osind01g11290.1 0.002 1 0.998 0.002 1	0	0	0	0	0	0	0	0

	1<br>Osjap01g11200.1 0.667 1 0.333 0.257 0.925<br>Osjap06g08850.1 0.002 0.578 0.576 0.004 0.966	1<br>Osind06g08940.1 0.002 0.525 0.523 0.004 0.846<br>Osind07g16750.1 0.592 0.963 0.371 0.207 0.913	1<br>Orufi02g30900.1 0.002 0.557 0.555 0.004 0.938<br>Orufi03g11370.1 0.658 0.968 0.31 0.211 0.912	1<br>Oglab01g08780.1 0.654 1 0.346 0.173 1<br>Oglab06g07730.1 0.002 0.578 0.576 0.004 0.966	1<br>Obart01g09730.1 0.654 1 0.346 0.173 1<br>Obart06g07830.1 0.002 0.578 0.576 0.004 0.921	1<br>Olong_007345-R1 0.658 0.968 0.31 0.211 0.912<br>Olong_010168-R1 0.002 0.557 0.555 0.004 0.938	1<br>Opunc01g09610.1 0.665 0.998 0.333 0.208 0.995<br>Opunc02g26870.2 0.002 0.557 0.555 0.004 0.93	1<br>Obrac02g21300.2 0.002 0.557 0.555 0.004 0.941<br>Obrac03g08890.1 0.658 0.968 0.31 0.043 0.907	1<br>Lperr01g09310.1 0.002 0.548 0.546 0.004 0.928<br>Lperr07g11580.1 0.654 0.986 0.332 0.252 0.954	0<br>AT5G16830.1 0.002 0.546 0.544 0.004 0.925
Osind01g15670.1	0	1<br>Osind01g15670.1 0.004 1 0.996 0.004 1	0	0	0	0	0	0	0	0
	1<br>Osjap01g15550.1 0.713 1 0.287 0.027 1<br>Osjap01g15560.1 0.004 0.702 0.698 0.002 0.412	1<br>Osind01g29920.1 0.027 0.713 0.686 0.016 0.487<br>Osind05g01020.1 0.744 1 0.256 0.653 0.958	1<br>Orufi01g14840.1 0.004 0.702 0.698 0.002 0.412<br>Orufi02g31040.2 0.744 1 0.256 0.65 0.958	1<br>Oglab01g11340.1 0.713 1 0.287 0.027 1<br>Oglab01g11350.1 0.004 0.702 0.698 0.002 0.412	1<br>Obart01g13510.1 0.713 1 0.287 0.027 1<br>Obart01g24360.1 0.027 0.713 0.686 0.016 0.487	1<br>Olong_004710-R1 0.744 1 0.256 0.667 0.96<br>Olong_009087-R1 0.004 0.702 0.698 0.003 0.477	0<br>Opunc03g32940.1 0.039 0.298 0.259 0.069 0.197<br>Opunc10g08200.1 0.682 1 0.318 0.497 0.954	1<br>Obrac02g21400.1 0.744 1 0.256 0.739 0.969<br>Obrac03g19560.1 0.031 0.709 0.678 0.011 0.435	1<br>Lperr03g21610.1 0.027 0.702 0.675 0.019 0.411<br>Lperr04g25830.1 0.744 1 0.256 0.648 0.958	0<br>AT4G01480.1 0.744 1 0.256 0.653 0.958
Osind01g16020.1	0	1<br>Osind01g16020.1 0.003 1 0.997 0.003 1	0	0	0	0	0	1<br>Obrac06g16200.1 0.093 0.993 0.9 0.018 0.93	0	0
	1<br>Osjap01g15910.1 0.003 0.3 0.297 0.002 0.173<br>Osjap11g21520.1 0.345 1 0.655 0.014 0.92	1<br>Osind01g16070.1 0.021 0.3 0.279 0.002 0.165<br>Osind11g10690.1 0.372 1 0.628 0.005 0.899	1<br>Orufi01g15200.1 0.003 0.3 0.297 0.002 0.173<br>Orufi11g19930.1 0.345 1 0.655 0.015 0.931	1<br>Oglab01g11660.1 0.003 0.3 0.297 0.002 0.173<br>Oglab11g16540.1 0.345 1 0.655 0.035 0.995	1<br>Obart01g13840.1 0.003 0.3 0.297 0.002 0.173<br>Obart11g18730.1 0.345 1 0.655 0.016 0.895	0<br>Olong_022841-R1 0.345 0.993 0.648 0.096 0.994	1<br>Opunc01g13400.1 0.369 0.993 0.624 0.002 0.888<br>Opunc01g13470.1 0.003 0.3 0.297 0.002 0.181	0<br>Obrac06g16190.1 0.269 0.993 0.724 0.018 0.975	1<br>Lperr05g03790.1 0.003 0.3 0.297 0.002 0.181<br>Lperr11g14380.1 0.372 1 0.628 0.068 0.973	0<br>AT1G48030.1 0.003 0.3 0.297 0.002 0.179
Osind01g21790.2	0	1<br>Osind01g21790.2 0.001 1 0.999 0.001 1	0	0	0	0	0	0	0	0
	1<br>Osjap01g21170.1 0.023 0.639 0.616 0.022 0.614<br>Osjap04g33720.1 0.656 0.983 0.327 0.362 0.924	0<br>Osind01g21790.1 0.023 0.639 0.616 0.023 0.637	1<br>Orufi01g20250.2 0.023 0.639 0.616 0.022 0.624<br>Orufi04g31270.1 0.656 0.983 0.327 0.362 0.924	0<br>Oglab04g27610.1 0.656 0.983 0.327 0.104 0.893	1<br>Obart01g18130.1 0.023 0.639 0.616 0.022 0.614<br>Obart04g29570.1 0.656 0.983 0.327 0.104 0.893	1<br>Olong_012814-R1 0.085 0.686 0.601 0.074 0.667<br>Olong_027538-R1 0.757 0.983 0.226 0.096 0.852	1<br>Opunc01g18590.1 0.643 1 0.357 0.007 1<br>Opunc01g18630.1 0.085 0.639 0.554 0.072 0.617	1<br>Obrac01g13780.1 0.097 0.618 0.521 0.003 0.58<br>Obrac04g20890.1 0.656 0.983 0.327 0.361 0.923	0<br>Lperr01g15790.2 0.001 0.668 0.667 0.001 0.651	1<br>AT1G23180.1 0.094 0.63 0.536 0.107 0.646<br>AT1G49010.1 0.65 0.994 0.344 0.01 0.962
Osind01g28070.1	0	1<br>Osind01g28070.1 0.002 1 0.998 0.002 1	1<br>Orufi01g25620.1 0.002 1 0.998 0.002 1	0	0	0	0	0	0	0
	1<br>Osjap01g26860.1 0.246 0.964 0.718 0.083 0.987<br>Osjap08g15630.1 0.002 0.246 0.244 0.007 0.912	1<br>Osind02g32380.1 0.002 0.246 0.244 0.009 1<br>Osind11g01570.1 0.246 0.893 0.647 0.489 0.955	1<br>Orufi12g02050.1 0.254 0.877 0.623 0.388 0.948<br>Orufi12g05600.1 0.002 0.246 0.244 0.459 0.725	1<br>Oglab11g01770.1 0.246 0.857 0.611 0.213 0.4<br>Oglab11g05250.1 0.005 0.246 0.241 0.43 0.731	1<br>Obart09g10970.1 0.002 0.202 0.2 0.007 0.743<br>Obart12g01860.1 0.232 0.973 0.741 0.088 0.703	1<br>Olong_007172-R1 0.005 0.246 0.241 0.341 0.684<br>Olong_015795-R1 0.246 0.877 0.631 0.506 0.958	1<br>Opunc09g15420.1 0.246 1 0.754 0.068 1<br>Opunc11g05440.1 0.005 0.246 0.241 0.431 0.728	1<br>Obrac09g10790.1 0.246 0.968 0.722 0.06 0.843<br>Obrac12g03740.1 0.005 0.246 0.241 0.35 0.667	1<br>Lperr09g13070.1 0.246 0.959 0.713 0.171 0.919<br>Lperr11g05150.1 0.005 0.246 0.241 0.444 0.715	1<br>AT1G77380.1 0.005 0.246 0.241 0.439 0.704<br>AT4G33495.1 0.246 0.914 0.668 0.103 0.988
Osind01g44140.1	1<br>Osjap01g42770.1 0.001 1 0.999 0.001 1	1<br>Osind01g44140.1 0.001 1 0.999 0.001 1	1<br>Orufi01g40790.1 0.001 1 0.999 0.001 1<br>Orufi01g40790.2 0.001 1 0.999 0.001 1<br>Orufi01g40790.3 0.001 1 0.999 0.001 1	0	0	1<br>Olong_024830-R1 0.001 1 0.999 0.001 1	0	0	0	0
	1<br>Osjap01g08100.1 0.013 0.278 0.265 0.133 0.963<br>Osjap03g27560.1 0.412 0.995 0.583 0.127 0.95	1<br>Osind01g08020.1 0.013 0.278 0.265 0.133 0.963<br>Osind03g28340.1 0.412 0.995 0.583 0.127 0.95	1<br>Orufi02g20240.3 0.013 0.267 0.254 0.044 0.951<br>Orufi03g26160.3 0.412 0.995 0.583 0.127 0.95	1<br>Oglab01g32220.1 0.001 0.266 0.265 0.004 0.934<br>Oglab01g32230.1 0.363 0.999 0.636 0.002 0.955	1<br>Obart01g37410.1 0.001 0.266 0.265 0.004 0.934<br>Obart01g37420.1 0.363 1 0.637 0.002 1	1<br>Olong_010570-R1 0.412 0.995 0.583 0.115 0.915<br>Olong_011675-R1 0.013 0.266 0.253 0.517 0.972	1<br>Opunc01g36330.1 0.001 0.266 0.265 0.005 0.975<br>Opunc01g36340.1 0.363 0.999 0.636 0.002 0.969	1<br>Obrac01g28830.1 0.001 0.264 0.263 0.004 0.978<br>Obrac01g28840.1 0.365 1 0.635 0.002 1	1<br>Lperr01g32130.1 0.001 0.266 0.265 0.005 0.977<br>Lperr01g32140.1 0.363 1 0.637 0.002 1	0<br>AT2G37970.1 0.001 0.266 0.265 0.049 0.978<br>AT3G54090.1 0.475 1 0.525 0.123 1
Osind01g52750.1	1<br>Osjap01g51220.1 0.002 1 0.998 0.071 1	1<br>Osind01g52750.1 0.002 1 0.998 0.002 1	1<br>Orufi01g48620.1 0.043 1 0.957 0.124 1	0	0	0	0	0	0	0
	1	1	1	1	1	1	1	1	0	0

	Osjap02g42170.1 0.002 0.698 0.696 0.362 0.927<br>Osjap05g12720.1 0.797 1 0.203 0.16 1	Osind02g41730.1 0.002 0.698 0.696 0.362 0.927<br>Osind05g13530.1 0.797 1 0.203 0.16 1	Orufi02g39420.1 0.002 0.698 0.696 0.362 0.927<br>Orufi05g12160.1 0.797 1 0.203 0.16 1	Oglab02g35020.1 0.002 0.698 0.696 0.352 0.901<br>Oglab05g10250.1 0.797 1 0.203 0.16 1	Obart02g37850.1 0.002 0.698 0.696 0.33 0.933<br>Obart05g11250.1 0.797 1 0.203 0.16 1	Olong_006258-R1 0.002 0.698 0.696 0.246 0.913<br>Olong_022231-R1 0.797 1 0.203 0.16 1	Opunc02g35080.1 0.002 0.698 0.696 0.366 0.926<br>Opunc05g09730.1 0.797 1 0.203 0.594 1	Obrac01g35490.1 0.797 1 0.203 0.395 1<br>Obrac02g28040.1 0.002 0.698 0.696 0.366 0.928	Lperr01g39540.1 0.797 1 0.203 0.152 1<br>Lperr02g31330.1 0.005 0.538 0.533 0.364 0.762	AT5G24900.1 0.029 0.534 0.505 0.408 0.785
Osind01g52800.1	0	1<br>Osind01g52800.1 0.002 1 0.998 0.002 1	0	0	0	0	0	0	0	0
	1<br>Osjap01g51320.2 0.002 0.577 0.575 0.002 0.661<br>Osjap01g51330.1 0.579 1 0.421 0.278 1	1<br>Osind01g52820.1 0.576 1 0.424 0.258 1<br>Osind04g34200.1 0.039 0.555 0.516 0.129 0.646	1<br>Orufi01g48670.1 0.002 0.577 0.575 0.002 0.661<br>Orufi05g26820.1 0.577 0.979 0.402 0.466 0.934	1<br>Oglab01g39850.1 0.002 0.577 0.575 0.002 0.661<br>Oglab05g23130.1 0.577 0.979 0.402 0.473 0.935	1<br>Obart01g45190.1 0.002 0.577 0.575 0.002 0.661<br>Obart11g03300.1 0.579 0.977 0.398 0.316 0.966	0<br>Olong_011545-R1 0.576 0.981 0.405 0.25 0.96<br>Olong_013976-R1 0.306 0.555 0.249 0.072 0.456	1<br>Opunc01g44160.1 0.597 1 0.403 0.206 1<br>Opunc01g44180.1 0.032 0.577 0.545 0.03 0.649	1<br>Obrac01g35530.1 0.576 1 0.424 0.337 1<br>Obrac04g21950.1 0.039 0.495 0.456 0.616 0.832	1<br>Lperr01g39620.1 0.576 1 0.424 0.14 1<br>Lperr04g26250.1 0.014 0.555 0.541 0.153 0.937	1<br>AT2G32230.1 0.004 0.555 0.551 0.124 0.673<br>AT4G27400.1 0.581 0.988 0.407 0.243 0.915
Osind02g01750.1	0	1<br>Osind02g01750.1 0.004 1 0.996 0.004 1	0	0	0	0	0	0	0	0
	1<br>Osjap02g01510.1 0.013 0.239 0.226 0.634 0.694<br>Osjap02g01520.1 0.307 1 0.693 0.342 1	1<br>Osind02g01760.1 0.013 0.239 0.226 0.624 0.686<br>Osind02g01770.1 0.307 1 0.693 0.348 1	1<br>Orufi02g01500.1 0.013 0.239 0.226 0.634 0.694<br>Orufi02g01510.1 0.307 1 0.693 0.348 1	1<br>Oglab02g01440.1 0.307 1 0.693 0.348 1<br>Oglab03g38410.1 0.013 0.218 0.205 0.612 0.671	1<br>Obart02g01520.1 0.013 0.239 0.226 0.611 0.674<br>Obart02g01530.1 0.307 1 0.693 0.335 1	1<br>Olong_000304-R1 0.307 0.945 0.638 0.367 1<br>Olong_012152-R1 0.013 0.218 0.205 0.595 0.664	1<br>Opunc02g00970.1 0.013 0.227 0.214 0.912 0.995<br>Opunc02g00990.1 0.307 1 0.693 0.348 1	1<br>Obrac02g01150.2 0.307 0.983 0.676 0.435 1<br>Obrac03g30480.1 0.013 0.218 0.205 0.617 0.675	1<br>Lperr02g01300.1 0.307 1 0.693 0.748 1<br>Lperr07g08320.1 0.013 0.218 0.205 0.657 0.713	1<br>AT1G63855.3 0.307 0.962 0.655 0.327 0.985<br>AT4G11840.1 0.017 0.223 0.206 0.62 0.677
Osind02g25330.1	0	1<br>Osind02g25330.1 0.001 1 0.999 0.001 1	0	0	0	0	0	0	0	0
	1<br>Osjap02g25610.1 0.001 0.578 0.577 0.179 0.949<br>Osjap04g19220.1 0.586 1 0.414 0.01 1	0<br>Osind04g17940.1 0.586 1 0.414 0.01 1	1<br>Orufi02g23670.1 0.001 0.578 0.577 0.002 0.938<br>Orufi04g17350.1 0.586 1 0.414 0.01 1	1<br>Oglab02g21020.1 0.001 0.578 0.577 0.002 0.941<br>Oglab04g14280.1 0.586 1 0.414 0.01 1	1<br>Obart01g36130.1 0.584 0.997 0.413 0.131 0.973<br>Obart02g22430.1 0.035 0.578 0.543 0.119 0.942	1<br>Olong_008587-R7 0.575 0.997 0.422 0.044 0.984<br>Olong_010793-R1 0.001 0.572 0.571 0.002 0.938	1<br>Opunc02g20390.1 0.038 0.581 0.543 0.057 0.945<br>Opunc04g13840.1 0.586 1 0.414 0.01 1	1<br>Obrac01g27840.1 0.591 0.997 0.406 0.189 0.974<br>Obrac02g15870.1 0.031 0.578 0.547 0.034 0.938	1<br>Lperr02g18240.1 0.039 0.575 0.536 0.058 0.936<br>Lperr04g12690.1 0.586 0.999 0.413 0.01 0.997	1<br>AT4G03070.1 0.589 1 0.411 0.047 0.997<br>AT5G42370.2 0.046 0.578 0.532 0.053 0.94
Osind02g26640.2	1<br>Osjap02g26750.1 0.001 0.953 0.952 0.001 0.993<br>Osjap02g26750.2 0.001 0.953 0.952 0.001 0.993<br>Osjap02g26750.4 0.001 0.953 0.952 0.001 0.799	1<br>Osind02g26640.2 0.001 1 0.999 0.001 1	1<br>Orufi02g24900.1 0.01 0.958 0.948 0.06 0.859<br>Orufi02g24900.3 0.001 0.958 0.957 0.001 0.74<br>Orufi02g24900.4 0.001 0.958 0.957 0.001 0.737	1<br>Oglab02g22130.2 0.001 1 0.999 0.001 1	1<br>Obart02g23460.2 0.001 1 0.999 0.001 1	0	1<br>Opunc02g21410.1 0.01 0.975 0.965 0.116 0.996<br>Opunc02g21410.2 0.01 0.975 0.965 0.114 0.996<br>Opunc02g21410.3 0.01 0.975 0.965 0.115 0.996<br>Opunc02g21410.4 0.01 0.975 0.965 0.114 0.996	0	0	0
	1<br>Osjap02g26590.1 0.497 0.986 0.489 0.033 0.996<br>Osjap02g26750.5 0.01 0.413 0.403 0.238 0.968	1<br>Osind02g26450.1 0.497 0.986 0.489 0.023 0.992<br>Osind02g26620.1 0.01 0.413 0.403 0.495 0.978	1<br>Orufi02g24730.1 0.497 0.986 0.489 0.031 0.996<br>Orufi02g24880.2 0.01 0.413 0.403 0.18 0.964	1<br>Oglab02g21930.1 0.497 1 0.503 0.047 1<br>Oglab02g22110.2 0.01 0.413 0.403 0.532 0.976	1<br>Obart02g23290.1 0.497 1 0.503 0.029 0.997<br>Obart02g23440.1 0.01 0.413 0.403 0.492 0.978	0<br>Olong_012377-R1 0.651 0.957 0.306 0.444 0.915<br>Olong_024345-R1 0.01 0.434 0.424 0.442 0.991	1<br>Opunc02g21230.1 0.504 0.96 0.456 0.003 0.934<br>Opunc02g21240.1 0.01 0.422 0.412 0.121 0.972	1<br>Obrac02g16670.1 0.503 0.957 0.454 0.005 0.995<br>Obrac02g16700.1 0.01 0.422 0.412 0.114 0.947	1<br>Lperr02g18740.1 0.512 0.975 0.463 0.059 0.982<br>Lperr02g19120.1 0.01 0.443 0.433 0.183 0.978	0<br>AT1G47230.2 0.577 0.953 0.376 0.095 0.857<br>AT4G31860.1 0.013 0.411 0.398 0.123 0.989
Osind02g37310.1	0	1<br>Osind02g37310.1 0.001 1 0.999 0.001 1	0	0	0	0	0	0	0	0
	1<br>Osjap01g03050.1 0.001 0.225 0.224 0.51 0.973<br>Osjap11g26640.1 0.293 1 0.707 0.123 1	0<br>Osind01g03180.1 0.001 0.225 0.224 0.507 0.973<br>Osind11g16840.2 0.499 0.999 0.5 0.358 0.998	1<br>Orufi01g02680.1 0.001 0.225 0.224 0.51 0.973<br>Orufi11g24570.1 0.293 1 0.707 0.02 1	1<br>Oglab05g22910.1 0.001 0.21 0.209 0.386 0.94<br>Oglab11g19980.1 0.27 1 0.73 0.002 1	1<br>Obart01g02620.1 0.001 0.225 0.224 0.431 0.969<br>Obart11g22860.1 0.27 1 0.73 0.002 1	0<br>Olong_019867-R1 0.293 1 0.707 0.019 1	1<br>Opunc01g02270.1 0.001 0.225 0.224 0.528 0.971<br>Opunc11g19350.1 0.27 1 0.73 0.002 1	0<br>Obrac01g02090.1 0.001 0.204 0.203 0.474 0.901<br>Obrac11g12180.1 0.501 0.999 0.498 0.291 0.998	1<br>Lperr01g02380.1 0.001 0.228 0.227 0.513 0.977<br>Lperr11g19340.1 0.299 1 0.701 0.026 1	0<br>AT4G36690.1 0.491 1 0.509 0.347 1
Osind02g37690.1	0	1<br>Osind02g37690.1 0.001 1 0.999 0.001 1	0	0	0	0	1<br>Opunc02g31410.1 0.001 0.999 0.998 0.001 0.999	1<br>Obrac02g24890.1 0.001 0.997 0.996 0.001 0.997	0	0
	1	1	1	1	1	1	1	1	1	1

	Osjap02g38120.1 0.321 1 0.679 0.017 1<br>Osjap02g38130.1 0.001 0.32 0.319 0.004 0.992	Osind06g02810.1 0.001 0.32 0.319 0.004 0.992<br>Osind10g08130.1 0.366 0.971 0.605 0.098 0.966	Orufi02g35540.1 0.321 1 0.679 0.017 1<br>Orufi06g02530.1 0.001 0.32 0.319 0.004 0.992	Oglab02g31390.1 0.321 1 0.679 0.017 1<br>Oglab06g02680.1 0.001 0.32 0.319 0.004 0.992	Obart02g34000.1 0.321 1 0.679 0.017 1<br>Obart06g02500.1 0.001 0.32 0.319 0.004 0.992	Olong_016336-R1 0.388 1 0.612 0.002 1<br>Olong_016337-R1 0.064 0.32 0.256 0.006 0.989	Opunc06g02370.1 0.001 0.32 0.319 0.004 0.992<br>Opunc07g22210.1 0.391 0.987 0.596 0.227 0.851	Obrac01g03710.2 0.385 0.987 0.602 0.199 0.936<br>Obrac06g01860.1 0.001 0.32 0.319 0.004 0.992	Lperr02g27870.1 0.355 0.999 0.644 0.002 0.998<br>Lperr06g02230.1 0.001 0.32 0.319 0.004 0.992	AT4G31300.2 0.029 0.32 0.291 0.038 0.991<br>AT4G37380.1 0.349 0.987 0.638 0.112 0.856
Osind02g38400.1	0	1<br>Osind02g38400.1 0.002 1 0.998 0.002 1	0	1<br>Oglab02g32090.1 0.002 0.989 0.987 0.438 0.998	0	0	0	0	0	0
	1<br>Osjap02g38830.1 0.629 0.982 0.353 0.603 0.875<br>Osjap02g38850.1 0.002 0.585 0.583 0.072 0.739	1<br>Osind02g38410.1 0.629 0.98 0.351 0.634 0.882<br>Osind09g11760.1 0.002 0.585 0.583 0.004 0.722	1<br>Orufi02g36250.1 0.629 0.982 0.353 0.614 0.902<br>Orufi09g12240.1 0.004 0.585 0.581 0.004 0.994	1<br>Oglab02g32090.2 0.633 0.983 0.35 0.135 0.763<br>Oglab09g10440.1 0.002 0.585 0.583 0.005 0.888	1<br>Obart02g34800.1 0.629 0.989 0.36 0.634 0.889<br>Obart09g11450.1 0.002 0.585 0.583 0.007 0.828	1<br>Olong_006227-R1 0.629 0.993 0.364 0.138 0.868<br>Olong_012584-R1 0.029 0.591 0.562 0.003 0.963	0<br>Opunc07g14160.1 0.004 0.672 0.668 0.007 0.898	1<br>Obrac02g25440.1 0.629 0.974 0.345 0.594 0.855<br>Obrac09g07580.1 0.028 0.589 0.561 0.028 0.972	1<br>Lperr02g28600.1 0.629 0.976 0.347 0.633 0.88<br>Lperr03g25050.1 0.013 0.583 0.57 0.005 0.787	1<br>AT1G47480.1 0.024 0.594 0.57 0.013 0.978<br>AT3G14067.1 0.629 0.983 0.354 0.616 0.87
Osind02g39460.2	0	1<br>Osind02g39460.1 0.001 1 0.999 0.001 1<br>Osind02g39460.2 0.001 1 0.999 0.001 1	1<br>Orufi02g37290.1 0.028 0.883 0.855 0.031 1	0	0	0	0	0	0	0
	1<br>Osjap01g00320.1 0.613 0.883 0.27 0.256 1<br>Osjap02g39920.1 0.001 0.572 0.571 0.001 0.971	1<br>Osind01g00350.1 0.613 0.883 0.27 0.037 1<br>Osind09g08880.1 0.02 0.567 0.547 0.201 0.99	1<br>Orufi01g00280.2 0.613 0.883 0.27 0.039 1<br>Orufi02g37310.1 0.001 0.572 0.571 0.002 0.995	1<br>Oglab02g32990.1 0.612 1 0.388 0.217 1<br>Oglab02g33000.1 0.001 0.606 0.605 0.001 0.986	1<br>Obart02g35820.1 0.612 1 0.388 0.223 1<br>Obart02g35830.2 0.001 0.606 0.605 0.001 0.985	1<br>Olong_003837-R1 0.614 0.883 0.269 0.003 1<br>Olong_003838-R2 0.001 0.572 0.571 0.014 1	1<br>Opunc01g00290.1 0.614 0.883 0.269 0.003 1<br>Opunc02g33020.1 0.013 0.595 0.582 0.032 0.997	1<br>Obrac01g00240.4 0.609 0.883 0.274 0.056 1<br>Obrac02g26250.1 0.015 0.573 0.558 0.059 0.992	1<br>Lperr01g00240.1 0.574 0.883 0.309 0.039 1<br>Lperr02g29510.1 0.001 0.572 0.571 0.034 1	1<br>AT3G54360.1 0.614 0.887 0.273 0.064 1<br>AT4G23370.1 0.006 0.567 0.561 0.179 0.996
Osind02g41950.1	0	1<br>Osind02g41950.1 0.001 1 0.999 0.001 1	0	0	0	0	0	0	0	0
	1<br>Osjap02g42560.1 0.463 0.994 0.531 0.205 0.995<br>Osjap04g22950.1 0.001 0.376 0.375 0.002 0.677	1<br>Osind04g22000.1 0.001 0.376 0.375 0.002 0.658<br>Osind05g11850.1 0.436 0.951 0.515 0.182 0.977	1<br>Orufi02g39760.2 0.368 0.929 0.561 0.068 0.952<br>Orufi04g20860.3 0.001 0.368 0.367 0.003 0.78	0<br>Oglab02g35180.1 0.368 1 0.632 0.067 1	1<br>Obart02g38050.1 0.368 1 0.632 0.066 1<br>Obart04g19330.2 0.117 0.362 0.245 0.062 0.759	1<br>Olong_002168-R1 0.001 0.376 0.375 0.002 0.653<br>Olong_020395-R1 0.441 0.94 0.499 0.046 0.685	1<br>Opunc03g04560.1 0.449 0.939 0.49 0.101 0.927<br>Opunc04g17040.1 0.001 0.393 0.392 0.003 0.877	0<br>Obrac02g28220.1 0.368 0.929 0.561 0.074 0.718	1<br>Lperr04g15670.1 0.001 0.369 0.368 0.003 0.747<br>Lperr07g13480.1 0.45 0.956 0.506 0.004 0.975	1<br>AT1G17940.1 0.001 0.369 0.368 0.002 0.721<br>AT3G22470.1 0.424 0.93 0.506 0.06 0.982
Osind02g42070.1	0	1<br>Osind02g42070.1 0.001 1 0.999 0.001 1	0	0	0	0	0	0	0	0
	1<br>Osjap02g42770.1 0.267 1 0.733 0.233 1<br>Osjap03g17490.1 0.005 0.265 0.26 0.666 0.994	1<br>Osind01g47650.1 0.299 0.989 0.69 0.21 0.991<br>Osind03g17680.1 0.005 0.265 0.26 0.583 0.992	1<br>Orufi02g39950.1 0.267 1 0.733 0.233 1<br>Orufi03g16810.1 0.005 0.265 0.26 0.666 0.994	1<br>Oglab02g35360.1 0.267 1 0.733 0.233 1<br>Oglab03g16010.1 0.005 0.265 0.26 0.666 0.994	1<br>Obart02g38230.1 0.267 1 0.733 0.233 1<br>Obart03g16270.2 0.005 0.265 0.26 0.666 0.994	1<br>Olong_004203-R1 0.267 1 0.733 0.226 1<br>Olong_013276-R1 0.005 0.265 0.26 0.666 0.994	1<br>Opunc02g35490.1 0.267 1 0.733 0.216 1<br>Opunc03g15730.1 0.007 0.265 0.258 0.669 0.994	0<br>Obrac06g17670.3 0.216 0.991 0.775 0.127 0.962	1<br>Lperr02g31700.1 0.267 0.999 0.732 0.222 1<br>Lperr03g14180.1 0.005 0.257 0.252 0.666 0.986	1<br>AT5G10540.1 0.267 0.999 0.732 0.136 1<br>AT5G67200.1 0.02 0.251 0.231 0.725 0.994
Osind03g00740.1	1<br>Osjap03g00680.1 0.001 0.996 0.995 0.001 0.994<br>Osjap03g00680.2 0.001 0.996 0.995 0.001 0.994<br>Osjap03g00680.3 0.001 0.996 0.995 0.001 0.994	1<br>Osind03g00740.1 0.001 1 0.999 0.001 1	1<br>Orufi03g12660.1 0.001 0.996 0.995 0.001 0.994<br>Orufi03g12660.2 0.001 0.996 0.995 0.001 0.994	1<br>Oglab03g00390.1 0.001 0.996 0.995 0.001 0.994	1<br>Obart03g00550.1 0.001 1 0.999 0.001 1	0	0	0	0	0
	1<br>Osjap02g42090.1 0.052 0.678 0.626 0.065 1<br>Osjap12g08580.1 0.708 0.996 0.288 0.011 0.977	1<br>Osind02g41670.1 0.062 0.678 0.616 0.131 1<br>Osind03g02220.1 0.698 0.996 0.298 0.015 0.982	1<br>Orufi02g39350.1 0.062 0.678 0.616 0.131 1<br>Orufi09g03230.3 0.732 0.99 0.258 0.053 0.963	1<br>Oglab02g34920.1 0.062 0.678 0.616 0.131 1<br>Oglab03g02050.1 0.698 0.996 0.298 0.214 0.986	1<br>Obart02g37790.1 0.062 0.648 0.586 0.133 0.977<br>Obart12g06510.1 0.676 0.996 0.32 0.052 0.984	0<br>Olong_013175-R1 0.271 0.904 0.633 0.002 0.961	1<br>Opunc03g00470.1 0.001 0.78 0.779 0.002 1<br>Opunc11g04590.1 0.78 0.996 0.216 0.055 0.982	0<br>Obrac02g27970.1 0.137 0.678 0.541 0.105 1<br>Obrac05g17660.1 0.743 0.995 0.252 0.425 0.997	1<br>Lperr03g00570.1 0.001 0.678 0.677 0.002 1<br>Lperr04g11360.1 0.747 0.996 0.249 0.415 0.99	0<br>AT5G04460.1 0.018 0.678 0.66 0.081 1
Osind03g03640.1	0	1<br>Osind03g03640.1 0.001 1 0.999 0.001 1	0	0	0	1<br>Olong_017738-R1 0.001 1 0.999 0.002 1	0	0	0	0
	1	1	1	0	1	1	1	1	1	0

	Osjap11g21530.1 0.001 0.279 0.278 0.001 0.971<br>Osjap11g22580.1 0.287 1 0.713 0.001 1	Osind04g03980.1 0.002 0.276 0.274 0.012 0.944<br>Osind08g08840.1 0.287 0.98 0.693 0.001 0.846	Orufi02g37700.1 0.001 0.276 0.275 0.002 0.946<br>Orufi11g20920.1 0.287 1 0.713 0.001 1	Oglab11g16610.1 0.001 0.279 0.278 0.002 0.957	Obart11g19590.1 0.758 1 0.242 0.003 1<br>Obart11g19610.2 0.287 0.744 0.457 0.002 0.979<br>Obart11g19620.1 0.001 0.276 0.275 0.482 0.972	Olong_021140-R1 0.001 0.279 0.278 0.002 0.957<br>Olong_026633-R1 0.287 0.984 0.697 0.001 0.867	Opunc01g13480.1 0.003 0.279 0.276 0.039 0.959<br>Opunc11g16180.1 0.287 1 0.713 0.001 1	Obrac06g16200.1 0.001 0.279 0.278 0.009 0.902<br>Obrac11g13540.1 0.287 0.99 0.703 0.001 0.908	Lperr11g15180.1 0.004 0.275 0.271 0.033 0.94<br>Lperr11g15890.1 0.28 1 0.72 0.042 1	AT3G46710.1 0.292 0.723 0.431 0.001 0.362<br>AT4G17565.1 0.004 0.262 0.258 0.048 0.915
Osind03g07990.1	0	1<br>Osind03g07990.1 0.005 1 0.995 0.005 1	0	0	0	0	0	0	0	0
	0<br>Osjap03g07930.1 0.266 1 0.734 0.081 1	1<br>Osind03g07970.1 0.015 0.332 0.317 0.015 0.471<br>Osind03g43640.1 0.347 0.96 0.613 0.326 0.984	1<br>Orufi03g07540.1 0.005 0.302 0.297 0.008 0.492<br>Orufi03g41020.1 0.337 0.96 0.623 0.332 0.985	1<br>Oglab03g07540.1 0.03 0.332 0.302 0.057 0.604<br>Oglab11g03980.1 0.357 1 0.643 0.228 1	1<br>Obart03g07440.1 0.03 0.332 0.302 0.057 0.604<br>Obart03g39270.1 0.347 0.96 0.613 0.327 0.984	1<br>Olong_003429-R1 0.302 0.95 0.648 0.251 0.837<br>Olong_008051-R1 0.03 0.296 0.266 0.054 0.505	1<br>Opunc03g07260.1 0.005 0.302 0.297 0.012 0.714<br>Opunc03g36380.1 0.337 1 0.663 0.332 1	0<br>Obrac03g22010.1 0.452 0.869 0.417 0.87 0.993	0<br>Lperr03g33440.1 0.347 0.985 0.638 0.329 0.998	0<br>AT3G53960.1 0.342 0.975 0.633 0.332 0.987
Osind03g12930.1	1<br>Osjap03g13100.1 0.028 1 0.972 0.001 1	1<br>Osind03g12930.1 0.001 1 0.999 0.001 1	0	1<br>Oglab03g12120.1 0.001 1 0.999 0.001 1	1<br>Obart03g12120.1 0.001 1 0.999 0.001 1	0	0	0	0	0
	1<br>Osjap04g18730.1 0.64 0.998 0.358 0.015 0.863<br>Osjap12g01140.1 0.109 0.633 0.524 0.498 0.981	1<br>Osind06g01770.1 0.642 0.998 0.356 0.212 0.912<br>Osind12g01290.1 0.109 0.633 0.524 0.49 0.981	1<br>Orufi06g01460.3 0.642 0.998 0.356 0.212 0.912<br>Orufi12g01130.1 0.109 0.633 0.524 0.498 0.981	1<br>Oglab06g01620.1 0.642 0.998 0.356 0.233 0.912<br>Oglab12g00800.1 0.114 0.633 0.519 0.483 0.983	1<br>Obart06g01490.1 0.642 0.998 0.356 0.233 0.912<br>Obart11g01060.1 0.114 0.633 0.519 0.471 0.982	1<br>Olong_007714-R9 0.642 1 0.358 0.002 1<br>Olong_007715-R1 0.125 0.633 0.508 0.007 0.965	1<br>Opunc01g28650.1 0.646 0.998 0.352 0.094 0.923<br>Opunc03g11680.1 0.028 0.633 0.605 0.001 0.971	1<br>Obrac03g09720.1 0.025 0.633 0.608 0.036 0.971<br>Obrac04g09430.1 0.64 0.998 0.358 0.014 0.863	1<br>Lperr03g10720.1 0.028 0.633 0.605 0.001 0.934<br>Lperr03g17470.1 0.633 0.998 0.365 0.119 0.828	1<br>AT3G46740.1 0.122 0.633 0.511 0.158 0.971<br>AT3G46790.1 0.648 0.998 0.35 0.068 0.807
Osind03g16830.1	0	1<br>Osind03g16830.1 0.001 1 0.999 0.001 1	0	0	0	0	0	0	0	0
	1<br>Osjap03g16610.1 0.677 1 0.323 0.017 1<br>Osjap03g16620.1 0.001 0.677 0.676 0.001 1	1<br>Osind04g16400.1 0.715 0.999 0.284 0.01 0.997<br>Osind05g11190.1 0.001 0.67 0.669 0.001 0.988	1<br>Orufi03g16050.1 0.677 1 0.323 0.017 1<br>Orufi03g16060.1 0.001 0.677 0.676 0.001 1	1<br>Oglab03g15290.1 0.677 1 0.323 0.017 1<br>Oglab03g15300.1 0.001 0.677 0.676 0.116 1	1<br>Obart03g15440.1 0.677 1 0.323 0.017 1<br>Obart03g15450.2 0.001 0.677 0.676 0.112 1	1<br>Olong_001740-R1 0.713 1 0.287 0.003 1<br>Olong_001741-R1 0.001 0.677 0.676 0.001 1	1<br>Opunc03g15010.1 0.679 1 0.321 0.043 1<br>Opunc03g15020.3 0.001 0.677 0.676 0.165 1	1<br>Obrac03g12410.2 0.001 0.679 0.678 0.001 0.983<br>Obrac05g14250.4 0.704 0.998 0.294 0.206 0.996	1<br>Lperr03g13610.1 0.677 1 0.323 0.017 1<br>Lperr03g13620.2 0.001 0.677 0.676 0.014 1	1<br>AT1G22200.1 0.713 0.999 0.286 0.005 0.997<br>AT2G27090.1 0.001 0.672 0.671 0.001 0.989
Osind03g16990.1	0	1<br>Osind03g16990.1 0.001 1 0.999 0.001 1	0	0	0	0	0	0	0	0
	1<br>Osjap03g16760.1 0.001 0.709 0.708 0.001 0.974<br>Osjap03g16790.1 0.709 0.966 0.257 0.26 0.968	0<br>Osind07g25320.1 0.3 0.71 0.41 0.009 0.994	1<br>Orufi03g16200.1 0.001 0.709 0.708 0.001 0.974<br>Orufi03g16220.1 0.709 0.966 0.257 0.26 0.968	1<br>Oglab03g15460.1 0.001 0.709 0.708 0.001 0.974<br>Oglab03g15480.1 0.709 0.961 0.252 0.267 0.968	1<br>Obart03g15600.1 0.001 0.709 0.708 0.001 0.974<br>Obart03g15640.1 0.709 0.966 0.257 0.161 0.964	1<br>Olong_004353-R1 0.001 0.709 0.708 0.001 1<br>Olong_004355-R1 0.709 0.966 0.257 0.193 0.961	1<br>Opunc01g10920.3 0.709 0.977 0.268 0.153 0.972<br>Opunc03g15170.1 0.001 0.709 0.708 0.001 1	1<br>Obrac03g12510.1 0.709 0.977 0.268 0.263 0.951<br>Obrac07g16290.1 0.001 0.709 0.708 0.001 0.994	1<br>Lperr03g13710.1 0.709 0.966 0.257 0.159 0.964<br>Lperr07g21650.1 0.001 0.709 0.708 0.001 1	0<br>AT5G13630.1 0.001 0.709 0.708 0.001 1
Osind03g18420.1	0	1<br>Osind03g18420.1 0 1 1 0 1	0	0	0	0	0	0	0	0
	1<br>Osjap03g02940.1 0.01 0.412 0.402 0.114 1<br>Osjap03g18170.1 0.415 1 0.585 0.267 1	0<br>Osind03g03120.1 0.01 0.412 0.402 0.114 1	1<br>Orufi03g02640.2 0.01 0.412 0.402 0.108 1<br>Orufi03g17450.1 0.415 1 0.585 0.112 1	1<br>Oglab03g02850.1 0.01 0.412 0.402 0.114 1<br>Oglab03g16640.1 0.417 1 0.583 0.178 1	1<br>Obart03g02920.1 0.01 0.412 0.402 0.032 1<br>Obart03g16860.1 0.415 1 0.585 0.084 1	0<br>Olong_005990-R1 0.01 0.412 0.402 0.032 1	1<br>Opunc03g02660.1 0.01 0.412 0.402 0.1 1<br>Opunc03g16270.2 0.415 1 0.585 0.109 1	1<br>Obrac03g02280.1 0.01 0.412 0.402 0.03 1<br>Obrac03g13510.1 0.424 1 0.576 0.105 1	1<br>Lperr03g02650.1 0.01 0.412 0.402 0.116 1<br>Lperr03g14700.1 0.415 1 0.585 0.11 1	0<br>AT5G49110.2 0.409 0.998 0.589 0.155 0.967
Osind03g25560.1	0	1<br>Osind03g25560.1 0.002 1 0.998 0.002 1	0	0	0	0	0	0	1<br>Lperr04g02020.1 0.009 0.868 0.859 0.017 1	0
	0<br>Osjap08g10690.1 0.002 0.643 0.641 0.516 0.882	1<br>Osind05g12260.1 0.002 0.629 0.627 0.003 1<br>Osind05g12280.1 0.715 1 0.285 0.024 1	1<br>Orufi08g11190.1 0.002 0.757 0.755 0.002 0.998<br>Orufi12g10080.1 0.784 1 0.216 0.376 1	1<br>Oglab09g01010.1 0.009 0.24 0.231 0.007 0.466<br>Oglab12g09140.1 0.357 1 0.643 0.022 1	1<br>Obart03g22930.1 0.009 0.643 0.634 0.004 0.825<br>Obart08g10210.1 0.658 1 0.342 0.06 1	0<br>Olong_026402-R1 0.649 0.926 0.277 0.077 0.967	1<br>Opunc05g06970.1 0.11 0.641 0.531 0.066 0.676<br>Opunc10g04140.1 0.643 0.948 0.305 0.279 0.707	0	0	0

Osind03g30900.1	0	1<br>Osind03g30900.1 0.001 1 0.999 0.001 1	0	0	0	0	0	0	0	0
	1<br>Osjap03g29750.2 0.001 0.355 0.354 0.036 0.996<br>Osjap04g18300.1 0.366 0.979 0.613 0.031 0.767	1<br>Osind04g17090.1 0.366 0.979 0.613 0.031 0.767<br>Osind12g19850.1 0.008 0.354 0.346 0.05 0.981	1<br>Orufi03g28480.1 0.33 1 0.67 0.036 1<br>Orufi12g21010.5 0.008 0.33 0.322 0.041 0.901	1<br>Oglab03g26970.1 0.003 0.355 0.352 0.007 0.444<br>Oglab03g34550.1 0.392 0.995 0.603 0.045 0.838	1<br>Obart03g27290.1 0.001 0.331 0.33 0.001 0.291<br>Obart03g36270.1 0.392 0.995 0.603 0.045 0.838	1<br>Olong_016768-R1 0.075 0.303 0.228 0.15 1<br>Olong_022955-R1 0.33 1 0.67 0.036 1	1<br>Opunc03g24970.1 0.33 1 0.67 0.038 1<br>Opunc12g17030.1 0.008 0.33 0.322 0.105 0.917	1<br>Obrac11g03750.1 0.354 1 0.646 0.084 1<br>Obrac12g12300.1 0.001 0.337 0.336 0.061 0.968	1<br>Lperr03g22630.2 0.005 0.331 0.326 0.022 0.964<br>Lperr03g22680.1 0.332 1 0.668 0.01 1	1<br>AT1G30300.1 0.006 0.35 0.344 0.062 0.994<br>AT4G18520.1 0.355 0.98 0.625 0.047 0.974
Osind03g31500.1	1<br>Osjap03g30460.1 0.025 1 0.975 0.022 1	1<br>Osind03g31500.1 0.001 1 0.999 0.001 1	1<br>Orufi03g29110.1 0.003 1 0.997 0.276 1	0	1<br>Obart03g27980.1 0.089 1 0.911 0.016 1<br>Obart03g27980.2 0.089 1 0.911 0.015 1	0	0	0	0	0
	1<br>Osjap03g30380.1 0.557 0.999 0.442 0.059 0.866<br>Osjap12g24400.2 0.092 0.528 0.436 0.012 0.988	1<br>Osind08g14630.1 0.557 0.999 0.442 0.069 0.96<br>Osind12g20440.1 0.092 0.528 0.436 0.01 0.99	1<br>Orufi03g29970.1 0.557 0.999 0.442 0.035 0.998<br>Orufi12g21550.1 0.092 0.528 0.436 0.01 0.99	1<br>Oglab03g27330.1 0.557 1 0.443 0.036 1<br>Oglab12g17460.4 0.092 0.528 0.436 0.012 0.996	1<br>Obart03g27900.1 0.557 0.999 0.442 0.053 0.865<br>Obart12g19300.1 0.092 0.528 0.436 0.01 0.99	1<br>Olong_002926-R1 0.097 0.529 0.432 0.002 0.996<br>Olong_002936-R1 0.557 0.999 0.442 0.059 0.866	1<br>Opunc03g25470.1 0.01 0.529 0.519 0.001 0.997<br>Opunc06g15370.1 0.557 0.999 0.442 0.035 0.871	0<br>Obrac03g20710.1 0.094 0.529 0.435 0.028 0.997	1<br>Lperr03g23190.1 0.557 0.999 0.442 0.03 0.867<br>Lperr03g23200.1 0.079 0.529 0.45 0.002 0.997	0<br>AT5G36940.1 0.093 0.531 0.438 0.003 0.998
Osind03g35550.1	1<br>Osjap03g34330.1 0.039 1 0.961 0.001 1	1<br>Osind03g35550.1 0.001 1 0.999 0.001 1	1<br>Orufi03g33060.1 0.039 1 0.961 0.001 1	1<br>Oglab03g30830.1 0.039 1 0.961 0.001 1	1<br>Obart03g31750.1 0.039 1 0.961 0.046 1	0	0	0	0	0
	1<br>Osjap03g39110.1 0.646 1 0.354 0.036 1<br>Osjap10g05370.1 0.026 0.622 0.596 0.051 0.963	1<br>Osind01g46540.1 0.646 0.999 0.353 0.065 0.994<br>Osind10g03770.1 0.026 0.622 0.596 0.057 0.96	1<br>Orufi10g04850.1 0.026 0.622 0.596 0.054 0.963<br>Orufi11g12520.1 0.647 1 0.353 0.038 0.991	1<br>Oglab03g34460.1 0.622 0.997 0.375 0.016 0.993<br>Oglab10g04340.1 0.02 0.622 0.602 0.058 0.963	1<br>Obart03g36200.1 0.646 1 0.354 0.016 0.997<br>Obart10g04600.1 0.051 0.622 0.571 0.106 0.962	1<br>Olong_014796-R1 0.039 0.532 0.493 0.002 0.95<br>Olong_027495-R1 0.622 0.995 0.373 0.005 0.998	1<br>Opunc01g42030.1 0.646 1 0.354 0.28 0.872<br>Opunc10g04080.2 0.033 0.622 0.589 0.084 0.959	1<br>Obrac02g16280.1 0.64 0.981 0.341 0.063 0.949<br>Obrac03g23440.1 0.039 0.622 0.583 0.002 0.964	1<br>Lperr03g30520.1 0.638 1 0.362 0.003 0.997<br>Lperr10g03360.1 0.004 0.622 0.618 0.009 0.962	0<br>AT1G70730.3 0.039 0.622 0.583 0.118 0.967
Osind03g45590.1	1<br>Osjap03g44530.1 0.002 1 0.998 0.001 1	1<br>Osind03g45590.1 0.002 1 0.998 0.002 1	0	0	0	1<br>Olong_013357-R1 0.002 1 0.998 0.002 1	0	1<br>Obrac03g30850.1 0.008 1 0.992 0.023 1	0	0
	1<br>Osjap03g41540.2 0.589 0.998 0.409 0.064 0.993<br>Osjap07g07980.2 0.005 0.556 0.551 0.225 0.99	1<br>Osind03g42620.1 0.589 0.998 0.409 0.054 0.993<br>Osind07g07170.1 0.002 0.556 0.554 0.002 0.987	1<br>Orufi03g42810.1 0.577 1 0.423 0.004 1<br>Orufi07g06860.4 0.002 0.556 0.554 0.002 0.987	1<br>Oglab03g38980.1 0.569 1 0.431 0.004 1<br>Oglab03g38990.1 0.002 0.542 0.54 0.002 0.968	1<br>Obart03g41020.1 0.569 1 0.431 0.004 1<br>Obart03g41030.1 0.002 0.542 0.54 0.002 0.967	1<br>Olong_020188-R1 0.002 0.556 0.554 0.002 0.987<br>Olong_021244-R1 0.563 0.992 0.429 0.283 0.929	1<br>Opunc03g38110.1 0.581 1 0.419 0.007 1<br>Opunc07g06610.1 0.002 0.561 0.559 0.002 0.993	1<br>Obrac03g30850.2 0.008 0.542 0.534 0.037 0.969<br>Obrac04g17280.1 0.569 0.992 0.423 0.003 0.906	1<br>Lperr03g34950.2 0.577 1 0.423 0.129 0.987<br>Lperr07g06010.1 0.002 0.561 0.559 0.002 0.991	1<br>AT3G01980.3 0.589 1 0.411 0.017 1<br>AT5G46410.2 0.145 0.539 0.394 0.206 0.991
Osind04g18090.1	1<br>Osjap04g19350.1 0.001 1 0.999 0.001 1	1<br>Osind04g18090.1 0.001 1 0.999 0.001 1	1<br>Orufi04g17470.1 0.03 1 0.97 0.001 1	0	0	1<br>Olong_005904-R1 0.032 1 0.968 0.021 1	0	0	1<br>Lperr04g12800.1 0.004 0.999 0.995 0.032 0.999	0
	1<br>Osjap02g23820.1 0.486 0.982 0.496 0.12 0.898<br>Osjap02g25840.1 0.034 0.4 0.366 0.013 0.995	1<br>Osind02g23720.1 0.486 0.982 0.496 0.123 0.899<br>Osind02g25560.1 0.034 0.4 0.366 0.013 0.995	1<br>Orufi02g23890.1 0.034 0.4 0.366 0.013 0.995<br>Orufi06g19770.1 0.487 1 0.513 0.115 0.977	1<br>Oglab04g14400.1 0.45 1 0.55 0.002 1<br>Oglab04g14410.1 0.027 0.404 0.377 0.064 1	1<br>Obart04g16100.1 0.45 1 0.55 0.002 1<br>Obart04g16110.1 0.027 0.404 0.377 0.064 1	1<br>Olong_013421-R1 0.507 1 0.493 0.002 0.946<br>Olong_021848-R1 0.034 0.4 0.366 0.013 0.995	1<br>Opunc04g13970.1 0.45 0.992 0.542 0.002 0.985<br>Opunc04g13980.1 0.03 0.404 0.374 0.003 1	1<br>Obrac04g09860.1 0.032 0.404 0.372 0.008 1<br>Obrac07g00130.3 0.452 0.983 0.531 0.001 0.49	1<br>Lperr02g18380.1 0.034 0.4 0.366 0.012 0.995<br>Lperr07g00080.1 0.451 0.999 0.548 0.004 0.892	0<br>AT5G01110.1 0.405 0.992 0.587 0.111 0.996
Osind04g20860.1	0	1<br>Osind04g20860.1 0.012 1 0.988 0.012 1	0	0	0	0	0	0	0	0
	1<br>Osjap04g21700.2 0.012 0.301 0.289 0.608 0.652<br>Osjap04g21710.1 0.361 1 0.639 0.614 1	1<br>Osind04g20770.1 0.361 1 0.639 0.614 1<br>Osind04g20850.1 0.012 0.301 0.289 0.435 0.508	1<br>Orufi01g10140.2 0.012 0.301 0.289 0.478 0.537<br>Orufi04g19670.1 0.361 1 0.639 0.614 1	1<br>Oglab04g16620.2 0.012 0.301 0.289 0.619 0.662<br>Oglab04g16630.1 0.361 1 0.639 0.617 1	1<br>Obart04g18220.3 0.012 0.301 0.289 0.532 0.584<br>Obart04g18230.1 0.361 1 0.639 0.617 1	0<br>Olong_021609-R1 0.012 0.301 0.289 0.46 0.524	1<br>Opunc04g15930.1 0.012 0.301 0.289 0.541 0.592<br>Opunc04g15940.1 0.361 1 0.639 0.623 1	0<br>Obrac04g11580.1 0.012 0.313 0.301 0.458 0.528<br>Obrac04g11590.1 0.639 1 0.361 0.86 1	0<br>Lperr04g14670.1 0.012 0.301 0.289 0.519 0.573	0<br>AT1G65930.1 0.024 0.771 0.747 0.488 0.612

Osind04g33950.1	0	1<br>Osind04g33950.1 0.002 1 0.998 0.002 1	0	0	0	0	0	0	0	0
	1<br>Osjap04g34720.1 0.402 1 0.598 0.005 1<br>Osjap09g00600.1 0.002 0.38 0.378 0.051 0.967	1<br>Osind04g33960.1 0.405 1 0.595 0.008 1<br>Osind09g00580.1 0.002 0.382 0.38 0.044 0.835	1<br>Orufi04g32300.1 0.402 1 0.598 0.005 1<br>Orufi09g00610.1 0.002 0.382 0.38 0.044 0.835	1<br>Oglab04g28630.2 0.002 0.38 0.378 0.005 0.966<br>Oglab04g28670.1 0.405 1 0.595 0.036 1	1<br>Obart04g30600.1 0.405 1 0.595 0.035 1<br>Obart09g00570.1 0.002 0.38 0.378 0.051 0.967	0<br>Olong_000331-R1 0.008 0.38 0.372 0.014 0.972<br>Olong_027105-R1 0.608 1 0.392 0.004 1	1<br>Opunc02g24980.1 0.002 0.38 0.378 0.308 0.979<br>Opunc04g28070.2 0.402 0.998 0.596 0.005 0.998	0<br>Obrac04g21730.1 0.002 0.38 0.378 0.005 0.966<br>Obrac08g02870.1 0.602 0.998 0.396 0.353 0.987	0<br>Lperr04g26010.1 0.002 0.38 0.378 0.005 0.966<br>Lperr12g00280.2 0.6 0.994 0.394 0.262 1	0<br>AT1G05970.2 0.021 0.378 0.357 0.04 0.96
Osind05g00510.1	1<br>Osjap05g00530.1 0.001 1 0.999 0.131 1<br>Osjap05g00530.2 0.001 1 0.999 0.16 1<br>Osjap05g00530.3 0.001 1 0.999 0.161 1<br>Osjap05g00530.4 0.001 1 0.999 0.142 1	1<br>Osind05g00510.1 0.001 1 0.999 0.001 1	1<br>Orufi05g00500.1 0.001 1 0.999 0.131 1	0	1<br>Obart05g00520.1 0.001 0.999 0.998 0.049 0.993<br>Obart05g00520.2 0.001 1 0.999 0.049 1	1<br>Olong_022405-R1 0.001 1 0.999 0.001 1	0	0	0	0
	0<br>Osjap02g42390.1 0.326 0.993 0.667 0.001 0.983	1<br>Osind01g11620.1 0.002 0.275 0.273 0.271 0.988<br>Osind09g15830.1 0.31 1 0.69 0.029 1	1<br>Orufi07g24760.1 0.001 0.258 0.257 0.267 0.834<br>Orufi09g16480.1 0.31 1 0.69 0.029 1	1<br>Oglab05g00500.1 0.001 0.26 0.259 0.344 0.982<br>Oglab09g13820.1 0.291 1 0.709 0.064 1	1<br>Obart01g04950.1 0.001 0.258 0.257 0.193 0.858<br>Obart07g24200.1 0.302 0.998 0.696 0.005 0.907	1<br>Olong_004108-R1 0.004 0.275 0.271 0.098 0.928<br>Olong_023813-R1 0.303 0.997 0.694 0.001 0.979	1<br>Opunc05g00480.1 0.298 0.999 0.701 0.007 0.998<br>Opunc05g00490.1 0.001 0.26 0.259 0.352 0.982	1<br>Obrac02g14170.4 0.004 0.267 0.263 0.164 0.843<br>Obrac03g06070.3 0.31 0.998 0.688 0.023 0.996	1<br>Lperr02g16220.1 0.004 0.267 0.263 0.238 0.857<br>Lperr05g00500.1 0.301 1 0.699 0.009 1	0<br>AT4G14820.1 0.001 0.267 0.266 0.107 0.878
Osind05g02510.1	1<br>Osjap05g02530.1 0.001 0.987 0.986 0.001 0.998	1<br>Osind05g02510.1 0.001 1 0.999 0.001 1	0	1<br>Oglab05g02190.1 0.002 0.987 0.985 0.079 0.998	1<br>Obart05g02340.1 0.002 0.987 0.985 0.076 0.998	0	0	0	0	0
	0<br>Osjap03g00960.1 0.533 0.984 0.451 0.327 0.996<br>Osjap08g22320.1 0.005 0.352 0.347 0.174 0.689	1<br>Osind07g26860.1 0.391 0.987 0.596 0.035 1<br>Osind12g11780.1 0.001 0.35 0.349 0.224 0.839	1<br>Orufi05g02460.2 0.349 0.988 0.639 0.003 0.979<br>Orufi08g23390.1 0.005 0.349 0.344 0.011 0.994	0<br>Oglab03g00660.1 0.533 0.984 0.451 0.321 0.996<br>Oglab07g20370.2 0.005 0.352 0.347 0.007 0.626	1<br>Obart08g20910.1 0.005 0.352 0.347 0.006 0.539<br>Obart11g00590.1 0.408 0.963 0.555 0.146 0.917	1<br>Olong_014752-R1 0.008 0.346 0.338 0.016 0.959<br>Olong_026687-R1 0.348 0.988 0.64 0.088 0.98	1<br>Opunc02g12280.1 0.001 0.349 0.348 0.004 0.985<br>Opunc05g02220.2 0.349 0.987 0.638 0.003 0.977	0<br>Obrac05g01840.1 0.348 0.987 0.639 0.168 1	1<br>Lperr03g00840.1 0.346 0.987 0.641 0.107 0.719<br>Lperr11g18770.2 0.006 0.296 0.29 0.428 0.781	0<br>AT5G15380.1 0.369 0.981 0.612 0.066 0.987
Osind05g03420.1	0	1<br>Osind05g03420.1 0.001 1 0.999 0.001 1	0	0	0	0	1<br>Opunc05g03030.1 0.001 1 0.999 0.001 1	0	0	0
	1<br>Osjap05g03420.2 0.706 0.982 0.276 0.075 0.828<br>Osjap05g03430.1 0.001 0.688 0.687 0.002 0.981	1<br>Osind06g19850.1 0.025 0.678 0.653 0.045 0.929<br>Osind06g23670.1 0.746 0.982 0.236 0.045 0.848	1<br>Orufi05g03290.1 0.706 0.982 0.276 0.064 0.854<br>Orufi05g03300.1 0.001 0.688 0.687 0.002 0.981	1<br>Oglab05g02870.1 0.706 0.997 0.291 0.068 0.948<br>Oglab05g02880.1 0.001 0.688 0.687 0.002 0.981	1<br>Obart05g03140.1 0.706 0.997 0.291 0.068 0.948<br>Obart05g03150.1 0.001 0.688 0.687 0.002 0.98	1<br>Olong_004932-R1 0.001 0.688 0.687 0.125 0.984<br>Olong_004933-R1 0.71 0.976 0.266 0.073 0.861	0<br>Opunc05g22860.1 0.005 0.669 0.664 0.053 0.921	1<br>Obrac05g02530.1 0.729 0.976 0.247 0.035 0.831<br>Obrac05g02540.1 0.001 0.688 0.687 0.002 0.981	1<br>Lperr05g02940.1 0.001 0.688 0.687 0.002 0.981<br>Lperr08g08920.1 0.733 0.999 0.266 0.004 0.819	1<br>AT3G24110.1 0.735 0.982 0.247 0.022 0.843<br>AT5G16070.1 0.001 0.688 0.687 0.002 0.979
Osind05g04070.1	1<br>Osjap05g04120.1 0.001 1 0.999 0.008 1<br>Osjap05g04120.2 0.001 1 0.999 0.008 1	1<br>Osind05g04070.1 0.001 1 0.999 0.001 1<br>Osind05g04070.2 0.001 1 0.999 0.001 1	1<br>Orufi05g03950.1 0.001 1 0.999 0.008 1<br>Orufi05g03950.2 0.001 1 0.999 0.008 1	1<br>Oglab05g03490.1 0.001 1 0.999 0.008 1<br>Oglab05g03490.2 0.001 1 0.999 0.008 1	1<br>Obart05g03830.1 0.001 1 0.999 0.008 1<br>Obart05g03830.2 0.001 1 0.999 0.008 1	1<br>Olong_016739-R1 0.069 1 0.931 0.001 1	1<br>Opunc05g03540.1 0.001 1 0.999 0.008 1<br>Opunc05g03540.2 0.001 1 0.999 0.008 1	1<br>Obrac05g02930.1 0.069 1 0.931 0.001 1	0	0
	1<br>Osjap01g27410.1 0.086 0.735 0.649 0.066 0.992<br>Osjap03g15140.2 0.738 1 0.262 0.01 0.192	1<br>Osind01g28630.1 0.086 0.735 0.649 0.066 0.992<br>Osind03g15230.1 0.783 1 0.217 0.03 1	1<br>Orufi01g26130.1 0.086 0.735 0.649 0.066 0.992<br>Orufi03g14720.1 0.783 1 0.217 0.03 1	1<br>Oglab01g19490.1 0.086 0.735 0.649 0.066 0.992<br>Oglab03g14060.1 0.783 1 0.217 0.03 1	1<br>Obart01g23310.1 0.086 0.735 0.649 0.066 0.992<br>Obart03g14050.1 0.783 1 0.217 0.03 1	1<br>Olong_003347-R2 0.086 0.735 0.649 0.047 0.992<br>Olong_009943-R1 0.783 1 0.217 0.03 1	1<br>Opunc01g23460.1 0.086 0.735 0.649 0.064 0.991<br>Opunc03g13590.1 0.783 1 0.217 0.002 0.545	1<br>Obrac01g17590.1 0.086 0.735 0.649 0.067 0.991<br>Obrac03g11330.1 0.783 1 0.217 0.03 1	0<br>Lperr05g03460.1 0.001 0.736 0.735 0.011 0.994	1<br>AT2G47580.1 0.768 1 0.232 0.008 1<br>AT3G16630.1 0.001 0.736 0.735 0.039 0.994
Osind05g04070.2	1<br>Osjap05g04120.1 0.001 1 0.999 0.008 1<br>Osjap05g04120.2 0.001 1 0.999 0.008 1	1<br>Osind05g04070.1 0.001 1 0.999 0.001 1<br>Osind05g04070.2 0.001 1 0.999 0.001 1	1<br>Orufi05g03950.1 0.001 1 0.999 0.008 1<br>Orufi05g03950.2 0.001 1 0.999 0.008 1	1<br>Oglab05g03490.1 0.001 1 0.999 0.008 1<br>Oglab05g03490.2 0.001 1 0.999 0.008 1	1<br>Obart05g03830.1 0.001 1 0.999 0.008 1<br>Obart05g03830.2 0.001 1 0.999 0.008 1	1<br>Olong_016739-R1 0.068 1 0.932 0.001 1	1<br>Opunc05g03540.1 0.001 1 0.999 0.008 1<br>Opunc05g03540.2 0.001 1 0.999 0.008 1	1<br>Obrac05g02930.1 0.068 1 0.932 0.001 1	0	0
	1<br>Osjap01g27410.1 0.084 0.738 0.654 0.066 0.992<br>Osjap03g15140.2 0.742 1 0.258 0.01 0.192	1<br>Osind01g28630.1 0.084 0.738 0.654 0.066 0.992<br>Osind03g15230.1 0.786 1 0.214 0.03 1	1<br>Orufi01g26130.1 0.084 0.738 0.654 0.066 0.992<br>Orufi03g14720.1 0.786 1 0.214 0.03 1	1<br>Oglab01g19490.1 0.084 0.738 0.654 0.066 0.992<br>Oglab03g14060.1 0.786 1 0.214 0.03 1	1<br>Obart01g23310.1 0.084 0.738 0.654 0.066 0.992<br>Obart03g14050.1 0.786 1 0.214 0.03 1	1<br>Olong_003347-R2 0.084 0.738 0.654 0.047 0.992<br>Olong_009943-R1 0.786 1 0.214 0.03 1	1<br>Opunc01g23460.1 0.084 0.738 0.654 0.064 0.991<br>Opunc03g13590.1 0.786 1 0.214 0.002 0.545	1<br>Obrac01g17590.1 0.084 0.738 0.654 0.067 0.991<br>Obrac03g11330.1 0.786 1 0.214 0.03 1	0<br>Lperr05g03460.1 0.001 0.74 0.739 0.011 0.994	1<br>AT2G47580.1 0.771 1 0.229 0.008 1<br>AT3G16630.1 0.001 0.74 0.739 0.039 0.994

Osind05g12550.1	1<br>Osjap05g11740.1 0.005 1 0.995 0.388 1<br>Osjap05g11740.2 0.005 1 0.995 0.432 1	1<br>Osind05g12550.1 0.005 1 0.995 0.005 1	1<br>Orufi05g11190.1 0.005 1 0.995 0.005 1	1<br>Oglab05g09590.1 0.005 1 0.995 0.005 1<br>Oglab05g09590.2 0.005 1 0.995 0.005 1	0	0	0	0	0	0
	0<br>Osjap11g19150.1 0.005 0.532 0.527 0.37 0.489	1<br>Osind08g13680.1 0.537 0.886 0.349 0.748 0.825<br>Osind11g14080.1 0.005 0.532 0.527 0.003 0.309	1<br>Orufi08g12780.1 0.537 0.886 0.349 0.769 0.839<br>Orufi11g17770.2 0.005 0.532 0.527 0.003 0.356	0<br>Oglab11g01510.4 0.507 0.886 0.379 0.681 0.789	1<br>Obart09g04270.2 0.537 0.92 0.383 0.847 0.93<br>Obart11g16680.1 0.065 0.532 0.467 0.051 0.311	1<br>Olong_007188-R1 0.537 0.886 0.349 0.744 0.822<br>Olong_026954-R1 0.045 0.532 0.487 0.016 0.497	1<br>Opunc08g10590.1 0.537 0.886 0.349 0.768 0.839<br>Opunc11g13660.1 0.065 0.532 0.467 0.051 0.311	0<br>Obrac12g01300.1 0.507 0.886 0.379 0.428 0.614	1<br>Lperr08g09600.2 0.537 0.886 0.349 0.759 0.833<br>Lperr11g13130.1 0.005 0.532 0.527 0.003 0.303	1<br>AT3G47630.2 0.005 0.532 0.527 0.003 0.298<br>AT4G19710.2 0.537 0.886 0.349 0.767 0.838
Osind05g17820.1	0	1<br>Osind05g17820.1 0.002 1 0.998 0.002 1	0	0	0	0	0	0	0	0
	1<br>Osjap01g50420.1 0.025 0.305 0.28 0.53 0.846<br>Osjap05g17020.1 0.357 1 0.643 0.075 1	0<br>Osind01g51980.1 0.025 0.305 0.28 0.53 0.846<br>Osind06g20290.1 0.552 0.973 0.421 0.427 0.957	1<br>Orufi05g16090.1 0.47 1 0.53 0.292 1<br>Orufi05g16100.1 0.025 0.305 0.28 0.6 0.878	1<br>Oglab05g14080.1 0.357 1 0.643 0.075 1<br>Oglab05g14090.1 0.025 0.305 0.28 0.618 0.883	1<br>Obart05g15020.1 0.357 1 0.643 0.123 1<br>Obart05g15030.1 0.025 0.305 0.28 0.533 0.858	0<br>Olong_011187-R1 0.523 1 0.477 0.019 1<br>Olong_021840-R1 0.025 0.305 0.28 0.657 0.887	1<br>Opunc01g43320.1 0.025 0.305 0.28 0.57 0.841<br>Opunc05g13080.1 0.425 1 0.575 0.212 1	0<br>Obrac01g34790.1 0.025 0.305 0.28 0.543 0.85<br>Obrac06g11950.1 0.489 0.973 0.484 0.016 0.937	1<br>Lperr01g38810.1 0.025 0.305 0.28 0.525 0.844<br>Lperr05g11880.1 0.47 1 0.53 0.27 1	1<br>AT3G08630.1 0.025 0.305 0.28 0.451 0.794<br>AT3G63490.1 0.445 0.986 0.541 0.223 0.983
Osind05g23260.1	0	1<br>Osind05g23260.1 0.01 1 0.99 0.01 1	0	0	0	0	0	0	0	0
	1<br>Osjap02g40490.1 0.01 0.344 0.334 0.494 0.573<br>Osjap04g24190.1 0.344 1 0.656 0.698 1	1<br>Osind02g39980.1 0.01 0.344 0.334 0.005 0.16<br>Osind04g23300.1 0.344 1 0.656 0.698 1	1<br>Orufi02g37810.1 0.01 0.344 0.334 0.494 0.573<br>Orufi04g22070.1 0.344 1 0.656 0.698 1	1<br>Oglab04g18980.1 0.344 1 0.656 0.698 1<br>Oglab07g15850.1 0.01 0.344 0.334 0.348 0.525	1<br>Obart04g20490.1 0.344 1 0.656 0.698 1<br>Obart05g09150.1 0.01 0.333 0.323 0.038 0.136	0<br>Olong_006838-R1 0.344 1 0.656 0.33 1	1<br>Opunc02g33420.1 0.01 0.344 0.334 0.501 0.581<br>Opunc04g18140.1 0.344 1 0.656 0.703 1	0<br>Obrac04g13370.1 0.344 0.896 0.552 0.679 0.912	1<br>Lperr02g29870.1 0.01 0.344 0.334 0.46 0.543<br>Lperr04g16590.1 0.344 0.938 0.594 0.677 0.955	0
Osind05g28520.1	1<br>Osjap05g28230.1 0.001 1 0.999 0.001 1	1<br>Osind05g28520.1 0.001 1 0.999 0.001 1	1<br>Orufi05g26870.1 0.001 1 0.999 0.001 1	1<br>Oglab05g23160.1 0.001 1 0.999 0.001 1	1<br>Obart05g25070.1 0.001 1 0.999 0.001 1	0	1<br>Opunc05g22640.1 0.001 1 0.999 0.001 1	0	1<br>Lperr01g22990.1 0.001 1 0.999 0.027 1	0
	1<br>Osjap01g31140.1 0.006 0.43 0.424 0.093 0.995<br>Osjap01g31150.1 0.486 0.999 0.513 0.009 0.999	1<br>Osind01g32600.1 0.001 0.43 0.429 0.002 0.995<br>Osind01g32610.1 0.486 0.999 0.513 0.009 0.999	1<br>Orufi01g29880.1 0.001 0.43 0.429 0.075 0.995<br>Orufi01g29890.1 0.486 0.999 0.513 0.009 0.999	1<br>Oglab01g22740.1 0.091 0.431 0.34 0.213 0.996<br>Oglab01g22750.1 0.486 0.999 0.513 0.009 0.999	1<br>Obart01g26870.1 0.005 0.43 0.425 0.117 0.995<br>Obart01g26880.1 0.486 0.999 0.513 0.009 0.999	1<br>Olong_000367-R1 0.1 0.431 0.331 0.002 0.995<br>Olong_000368-R1 0.461 0.999 0.538 0.069 0.999	1<br>Opunc01g26820.1 0.091 0.431 0.34 0.118 0.996<br>Opunc01g26830.1 0.486 1 0.514 0.009 1	1<br>Obrac01g20400.1 0.091 0.431 0.34 0.125 0.996<br>Obrac05g17560.2 0.431 1 0.569 0.109 1	1<br>Lperr05g20850.2 0.491 1 0.509 0.002 1<br>Lperr05g20860.1 0.091 0.431 0.34 0.215 0.996	1<br>AT5G08680.1 0.022 0.43 0.408 0.064 0.995<br>AT5G51600.1 0.486 0.999 0.513 0.008 0.996
Osind05g31580.1	0	1<br>Osind05g31580.1 0.001 1 0.999 0.001 1	0	0	1<br>Obart05g27940.1 0.024 1 0.976 0.001 1	1<br>Olong_007981-R1 0.074 1 0.926 0.001 1	0	0	0	0
	1<br>Osjap01g24630.2 0.635 0.951 0.316 0.156 0.894<br>Osjap05g31230.3 0.001 0.627 0.626 0.006 0.982	1<br>Osind01g25470.1 0.024 0.579 0.555 0.061 0.967<br>Osind01g25480.1 0.635 0.951 0.316 0.063 0.877	1<br>Orufi01g23560.1 0.635 0.951 0.316 0.144 0.891<br>Orufi05g29910.3 0.001 0.627 0.626 0.001 0.982	1<br>Oglab05g25920.1 0.001 0.627 0.626 0.001 0.982<br>Oglab05g25930.1 0.635 1 0.365 0.07 1	0<br>Obart01g20890.1 0.635 0.951 0.316 0.063 0.877<br>Obart07g06670.1 0.179 0.628 0.449 0.36 0.978	1<br>Olong_011279-R1 0.635 0.991 0.356 0.063 0.978<br>Olong_022566-R2 0.179 0.626 0.447 0.313 0.975	1<br>Opunc05g25540.1 0.007 0.627 0.62 0.002 0.983<br>Opunc05g25550.1 0.635 1 0.365 0.067 1	0<br>Obrac05g19820.2 0.024 0.627 0.603 0.001 0.982	1<br>Lperr01g17840.1 0.635 0.999 0.364 0.166 0.958<br>Lperr05g23570.2 0.003 0.627 0.624 0.002 0.982	1<br>AT1G07990.1 0.635 0.912 0.277 0.061 0.782<br>AT4G05420.1 0.024 0.627 0.603 0.001 0.982
Osind06g03270.2	0	1<br>Osind06g03270.2 0.001 1 0.999 0.001 1	0	0	0	0	0	0	0	0
	1<br>Osjap06g03320.1 0.537 1 0.463 0.009 1<br>Osjap06g03400.4 0.001 0.51 0.509 0.118 0.987	0<br>Osind06g03270.3 0.001 0.514 0.513 0.082 0.991	1<br>Orufi06g02900.1 0.537 1 0.463 0.011 1<br>Orufi06g02980.1 0.001 0.534 0.533 0.105 0.989	1<br>Oglab02g08830.1 0.576 1 0.424 0.079 1<br>Oglab04g23270.1 0.005 0.553 0.548 0.002 0.85	1<br>Obart06g02920.1 0.537 1 0.463 0.009 1<br>Obart06g03030.1 0.003 0.526 0.523 0.087 0.983	0<br>Olong_023541-R1 0.166 0.485 0.319 0.094 0.991<br>Olong_027587-R1 0.595 1 0.405 0.091 1	1<br>Opunc06g02690.1 0.537 1 0.463 0.007 1<br>Opunc06g02730.1 0.003 0.526 0.523 0.1 0.971	1<br>Obrac04g18480.1 0.005 0.514 0.509 0.099 1<br>Obrac06g13140.1 0.553 1 0.447 0.028 1	1<br>Lperr02g07340.1 0.551 1 0.449 0.021 1<br>Lperr06g02620.1 0.001 0.51 0.509 0.002 0.988	0<br>AT5G15110.1 0.544 1 0.456 0.011 1

Osind06g07560.1	1<br>Osjap06g07420.1 0.038 1 0.962 0.021 1	1<br>Osind06g07560.1 0.001 1 0.999 0.001 1	1<br>Orufi06g06800.1 0.038 1 0.962 0.039 1	0	0	0	0	0	1<br>Lperr06g06040.1 0.04 0.999 0.959 0.01 0.998<br>Lperr06g06040.2 0.076 0.999 0.923 0.01 0.998<br>Lperr06g06040.3 0.076 0.999 0.923 0.011 0.998	0
	1<br>Osjap04g00260.3 0.022 0.31 0.288 0.145 0.955<br>Osjap06g07420.2 0.399 1 0.601 0.005 1	0<br>Osind02g38310.1 0.399 1 0.601 0.005 0.998	0<br>Orufi06g06800.2 0.4 1 0.6 0.01 1	1<br>Oglab02g31950.1 0.406 1 0.594 0.236 0.998<br>Oglab06g06410.2 0.001 0.313 0.312 0.004 0.961	1<br>Obart06g06500.3 0.001 0.313 0.312 0.004 0.961<br>Obart06g06510.1 0.399 1 0.601 0.005 1	1<br>Olong_007080-R2 0.399 1 0.601 0.005 1<br>Olong_007081-R3 0.001 0.294 0.293 0.005 0.943	1<br>Opunc02g31990.2 0.399 1 0.601 0.005 0.998<br>Opunc04g00320.1 0.022 0.31 0.288 0.144 0.951	1<br>Obrac04g00310.1 0.003 0.31 0.307 0.109 0.867<br>Obrac06g05010.3 0.399 0.999 0.6 0.005 1	1<br>Lperr02g28500.3 0.037 0.313 0.276 0.054 0.831<br>Lperr02g28510.3 0.399 1 0.601 0.005 0.998	0<br>AT2G26310.1 0.488 1 0.512 0.083 1<br>AT5G26667.1 0.053 0.313 0.26 0.053 0.904
Osind06g17590.1	0	1<br>Osind06g17590.1 0.001 1 0.999 0.001 1<br>Osind08g03120.1 0.01 0.991 0.981 0.058 0.989	0	0	1<br>Obart11g22180.1 0.037 0.999 0.962 0.038 0.984	1<br>Olong_000078-R1 0.035 0.896 0.861 0.028 0.972	0	0	0	0
	1<br>Osjap06g30040.4 0.016 0.463 0.447 0.009 0.829<br>Osjap11g26070.1 0.474 0.999 0.525 0.095 0.977	1<br>Osind01g01210.1 0.474 0.999 0.525 0.091 0.977<br>Osind06g29310.2 0.016 0.463 0.447 0.009 0.829	1<br>Orufi06g27630.3 0.016 0.463 0.447 0.009 0.829<br>Orufi11g24100.1 0.488 0.918 0.43 0.147 0.881	1<br>Oglab06g23090.2 0.016 0.463 0.447 0.009 0.829<br>Oglab11g19360.1 0.474 0.999 0.525 0.091 0.977	1<br>Obart11g22190.1 0.035 0.47 0.435 0.076 0.928<br>Obart11g22210.1 0.482 0.991 0.509 0.031 0.971	1<br>Olong_000079-R1 0.02 0.474 0.454 0.017 0.917<br>Olong_022539-R1 0.488 0.889 0.401 0.1 0.844	1<br>Opunc11g18540.2 0.018 0.466 0.448 0.045 0.912<br>Opunc11g18840.1 0.474 1 0.526 0.148 1	1<br>Obrac11g13070.1 0.474 0.992 0.518 0.016 0.963<br>Obrac11g13100.2 0.035 0.474 0.439 0.13 0.891	1<br>Lperr06g21190.1 0.01 0.463 0.453 0.004 0.899<br>Lperr11g18890.1 0.474 0.994 0.52 0.119 0.988	0<br>AT4G11460.1 0.22 0.503 0.283 0.466 0.884
Osind06g23650.1	0	1<br>Osind06g23650.1 0.001 1 0.999 0.001 1	1<br>Orufi06g22110.1 0.001 1 0.999 0.011 1	0	1<br>Obart06g20680.1 0.001 1 0.999 0.001 1	0	0	0	0	0
	1<br>Osjap06g24310.1 0.001 0.693 0.692 0.059 0.986<br>Osjap06g24320.3 0.713 1 0.287 0.019 1	1<br>Osind02g08380.1 0.714 0.999 0.285 0.031 0.997<br>Osind09g17960.1 0.005 0.693 0.688 0.008 0.978	1<br>Orufi02g07600.1 0.714 0.999 0.285 0.031 0.997<br>Orufi09g15100.1 0.01 0.693 0.683 0.029 0.975	1<br>Oglab06g18700.1 0.001 0.693 0.692 0.016 0.986<br>Oglab06g18710.1 0.713 1 0.287 0.019 1	1<br>Obart02g07500.3 0.714 0.999 0.285 0.031 0.997<br>Obart04g22320.1 0.005 0.691 0.686 0.017 0.982	1<br>Olong_021346-R1 0.001 0.693 0.692 0.01 0.982<br>Olong_021347-R1 0.715 1 0.285 0.003 1	1<br>Opunc01g29350.1 0.001 0.691 0.69 0.004 0.986<br>Opunc06g18860.1 0.713 1 0.287 0.009 0.497	1<br>Obrac04g14990.1 0.005 0.691 0.686 0.017 0.982<br>Obrac06g14090.3 0.712 1 0.288 0.338 1	1<br>Lperr01g25410.2 0.001 0.691 0.69 0.009 0.986<br>Lperr02g06450.6 0.714 0.999 0.285 0.032 0.997	1<br>AT4G21160.1 0.716 1 0.284 0.047 1<br>AT5G51750.1 0.005 0.693 0.688 0.021 0.985
Osind06g31620.1	1<br>Osjap09g17800.1 0.025 1 0.975 0.113 0.974<br>Osjap09g17800.2 0.025 1 0.975 0.114 0.974	1<br>Osind06g31620.1 0.001 1 0.999 0.001 1	1<br>Orufi06g29990.1 0.001 1 0.999 0.001 1<br>Orufi09g18790.1 0.025 1 0.975 0.107 0.97<br>Orufi09g18790.2 0.025 1 0.975 0.107 0.97<br>Orufi09g18790.3 0.025 1 0.975 0.108 0.969<br>Orufi09g18790.4 0.025 1 0.975 0.108 0.969<br>Orufi09g18790.6 0.025 0.996 0.971 0.172 0.937	1<br>Oglab06g25490.1 0.001 1 0.999 0.001 1<br>Oglab09g15660.1 0.025 0.999 0.974 0.154 0.95	1<br>Obart06g28060.1 0.001 1 0.999 0.001 1<br>Obart09g17460.1 0.025 1 0.975 0.076 0.969<br>Obart09g17460.2 0.025 1 0.975 0.076 0.969	1<br>Olong_016700-R1 0.001 1 0.999 0.001 1	0	0	1<br>Lperr09g13380.1 0.087 0.982 0.895 0.166 0.951	0
	0<br>Osjap06g32830.2 0.41 1 0.59 0.121 1	1<br>Osind02g01560.1 0.069 0.421 0.352 0.236 0.926<br>Osind06g30840.1 0.429 0.999 0.57 0.078 1	0<br>Orufi06g29150.4 0.45 0.999 0.549 0.122 1	1<br>Oglab02g01250.1 0.069 0.421 0.352 0.221 0.93<br>Oglab10g06880.1 0.446 1 0.554 0.229 1	1<br>Obart04g21770.1 0.073 0.433 0.36 0.232 0.933<br>Obart10g07310.1 0.446 1 0.554 0.229 1	1<br>Olong_000286-R1 0.069 0.421 0.352 0.258 0.928<br>Olong_026973-R2 0.446 1 0.554 0.229 1	1<br>Opunc06g25610.1 0.001 0.422 0.421 0.002 0.951<br>Opunc10g06480.1 0.446 0.998 0.552 0.226 0.984	1<br>Obrac06g20010.1 0.426 1 0.574 0.211 1<br>Obrac06g20020.1 0.001 0.422 0.421 0.002 0.952	1<br>Lperr06g23510.1 0.446 1 0.554 0.234 1<br>Lperr06g23520.1 0.001 0.421 0.42 0.002 0.949	1<br>AT1G76360.1 0.037 0.421 0.384 0.219 0.952<br>AT2G46480.1 0.446 0.999 0.553 0.07 1
Osind07g02300.3	1<br>Osjap07g02600.3 0.002 0.978 0.976 0.478 0.853<br>Osjap07g02600.4 0.002 1 0.998 0.555 1<br>Osjap07g02600.5 0.002 1 0.998 0.002 1	1<br>Osind07g02300.3 0.002 1 0.998 0.002 1<br>Osind07g02300.4 0.002 1 0.998 0.002 1	0	0	0	0	0	0	0	0
	1<br>Osjap01g07190.1 0.002 0.254 0.252 0.692 0.935<br>Osjap07g02840.1 0.298 0.976 0.678 0.411 0.823	1<br>Osind01g07120.1 0.002 0.254 0.252 0.692 0.935<br>Osind07g02530.1 0.298 0.976 0.678 0.411 0.823	1<br>Orufi07g02320.1 0.298 0.976 0.678 0.411 0.823<br>Orufi12g02700.1 0.007 0.259 0.252 0.518 0.746	1<br>Oglab01g05790.1 0.002 0.254 0.252 0.692 0.935<br>Oglab07g02390.2 0.298 0.976 0.678 0.41 0.823	1<br>Obart01g06210.1 0.002 0.254 0.252 0.692 0.935<br>Obart07g02450.1 0.298 0.976 0.678 0.377 0.809	1<br>Olong_003501-R1 0.002 0.254 0.252 0.634 0.923<br>Olong_009970-R1 0.298 0.976 0.678 0.411 0.823	1<br>Opunc01g05980.1 0.002 0.254 0.252 0.689 0.934<br>Opunc07g02500.8 0.298 0.976 0.678 0.472 0.79	1<br>Obrac05g04770.1 0.002 0.254 0.252 0.594 0.923<br>Obrac07g01520.1 0.298 0.99 0.692 0.386 0.893	1<br>Lperr07g02310.1 0.298 0.976 0.678 0.431 0.845<br>Lperr11g02330.1 0.007 0.257 0.25 0.682 0.933	0<br>AT4G36380.1 0.002 0.308 0.306 0.691 0.966

Osind07g02300.4	1<br>Osjap07g02600.3 0.002 0.978 0.976 0.478 0.853<br>Osjap07g02600.4 0.002 1 0.998 0.555 1<br>Osjap07g02600.5 0.002 1 0.998 0.002 1	1<br>Osind07g02300.3 0.002 1 0.998 0.002 1<br>Osind07g02300.4 0.002 1 0.998 0.002 1	0	0	0	0	0	0	0	0
	1<br>Osjap01g07190.1 0.002 0.254 0.252 0.692 0.935<br>Osjap07g02840.1 0.298 0.976 0.678 0.411 0.823	1<br>Osind01g07120.1 0.002 0.254 0.252 0.692 0.935<br>Osind07g02530.1 0.298 0.976 0.678 0.411 0.823	1<br>Orufi07g02320.1 0.298 0.976 0.678 0.411 0.823<br>Orufi12g02700.1 0.007 0.259 0.252 0.518 0.746	1<br>Oglab01g05790.1 0.002 0.254 0.252 0.692 0.935<br>Oglab07g02390.2 0.298 0.976 0.678 0.41 0.823	1<br>Obart01g06210.1 0.002 0.254 0.252 0.692 0.935<br>Obart07g02450.1 0.298 0.976 0.678 0.377 0.809	1<br>Olong_003501-R1 0.002 0.254 0.252 0.634 0.923<br>Olong_009970-R1 0.298 0.976 0.678 0.411 0.823	1<br>Opunc01g05980.1 0.002 0.254 0.252 0.689 0.934<br>Opunc07g02500.8 0.298 0.976 0.678 0.472 0.79	1<br>Obrac05g04770.1 0.002 0.254 0.252 0.594 0.923<br>Obrac07g01520.1 0.298 0.99 0.692 0.386 0.893	1<br>Lperr07g02310.1 0.298 0.976 0.678 0.431 0.845<br>Lperr11g02330.1 0.007 0.257 0.25 0.682 0.933	0<br>AT4G36380.1 0.002 0.308 0.306 0.691 0.966
Osind07g03110.1	0	1<br>Osind07g03110.1 0.001 1 0.999 0.001 1	0	0	0	0	0	0	0	0
	1<br>Osjap07g03560.2 0.335 0.937 0.602 0.001 0.949<br>Osjap07g03570.1 0.001 0.318 0.317 0.001 0.975	0<br>Osind11g11170.1 0.367 0.748 0.381 0.038 0.738	1<br>Orufi07g02900.1 0.335 1 0.665 0.001 1<br>Orufi07g02910.1 0.001 0.318 0.317 0.001 0.975	1<br>Oglab07g03120.1 0.335 0.937 0.602 0.001 0.951<br>Oglab07g03130.1 0.001 0.318 0.317 0.001 0.975	1<br>Obart07g03200.1 0.413 0.937 0.524 0.001 0.941<br>Obart07g03210.1 0.001 0.318 0.317 0.001 0.975	0<br>Olong_011063-R1 0.404 0.837 0.433 0.006 0.847<br>Olong_026333-R1 0.001 0.318 0.317 0.002 0.973	1<br>Opunc07g03100.2 0.404 0.986 0.582 0.001 0.984<br>Opunc07g03110.1 0.001 0.318 0.317 0.001 0.975	1<br>Obrac07g01870.1 0.413 0.937 0.524 0.002 0.936<br>Obrac07g01880.1 0.001 0.318 0.317 0.001 0.975	1<br>Lperr07g02700.1 0.404 0.937 0.533 0.004 0.926<br>Lperr07g02710.2 0.001 0.318 0.317 0.001 0.975	0<br>AT1G06140.1 0.433 0.851 0.418 0.016 0.998<br>AT1G30360.1 0.001 0.324 0.323 0.001 0.985
Osind07g04880.1	1<br>Osjap07g05600.1 0.002 1 0.998 0.002 1	1<br>Osind07g04880.1 0.002 1 0.998 0.002 1	1<br>Orufi07g04740.1 0.002 0.94 0.938 0.002 0.996	0	0	0	0	0	0	0
	1<br>Osjap04g15050.1 0.356 0.865 0.509 0.315 0.932<br>Osjap07g05610.1 0.036 0.333 0.297 0.005 0.444	1<br>Osind04g13510.1 0.356 0.865 0.509 0.318 0.938<br>Osind07g04890.1 0.002 0.333 0.331 0.006 0.696	1<br>Orufi04g13400.1 0.356 0.865 0.509 0.318 0.938<br>Orufi07g04750.1 0.036 0.333 0.297 0.007 0.667	1<br>Oglab04g10600.1 0.356 0.865 0.509 0.323 0.938<br>Oglab07g04740.1 0.002 0.32 0.318 0.004 0.866	1<br>Obart02g19560.1 0.361 0.865 0.504 0.151 0.92<br>Obart07g04940.1 0.002 0.32 0.318 0.007 0.757	0<br>Olong_011399-R1 0.002 0.601 0.599 0.004 0.978	1<br>Opunc02g17460.1 0.361 0.873 0.512 0.326 0.95<br>Opunc08g05640.1 0.002 0.333 0.331 0.006 0.705	0<br>Obrac02g20740.1 0.569 0.908 0.339 0.635 0.984	0<br>Lperr02g15690.1 0.356 0.865 0.509 0.32 0.935	0<br>AT3G52450.1 0.567 0.865 0.298 0.618 0.936
Osind07g21400.1	0	1<br>Osind07g21400.1 0.001 1 0.999 0.001 1<br>Osind07g21400.2 0.001 0.99 0.989 0.001 0.99	0	0	0	0	0	0	0	0
	1<br>Osjap07g24560.1 0.001 0.366 0.365 0.003 0.973<br>Osjap07g24570.1 0.427 0.989 0.562 0.093 0.989	1<br>Osind04g27100.1 0.016 0.356 0.34 0.09 0.951<br>Osind07g09290.1 0.49 0.965 0.475 0.204 0.813	1<br>Orufi07g22110.1 0.001 0.366 0.365 0.003 0.973<br>Orufi07g22120.1 0.427 0.99 0.563 0.093 0.99	1<br>Oglab07g18190.1 0.001 0.383 0.382 0.003 0.883<br>Oglab07g18200.1 0.427 0.99 0.563 0.093 0.99	1<br>Obart07g21090.1 0.001 0.366 0.365 0.003 0.973<br>Obart07g21100.1 0.458 0.99 0.532 0.179 0.991	1<br>Olong_006539-R1 0.001 0.394 0.393 0.003 0.945<br>Olong_006540-R1 0.498 0.99 0.492 0.062 0.989	1<br>Opunc07g13090.1 0.001 0.394 0.393 0.002 0.885<br>Opunc07g13100.1 0.394 0.99 0.596 0.059 0.99	1<br>Obrac07g13360.2 0.001 0.366 0.365 0.003 0.973<br>Obrac07g13370.2 0.496 0.999 0.503 0.002 1	1<br>Lperr07g18240.1 0.394 0.99 0.596 0.061 0.994<br>Lperr07g18250.1 0.001 0.357 0.356 0.003 0.809	1<br>AT1G26460.1 0.399 0.992 0.593 0.041 0.998<br>AT1G26520.1 0.001 0.356 0.355 0.029 0.947
Osind07g21400.2	0	1<br>Osind07g21400.1 0.001 0.99 0.989 0.001 0.99<br>Osind07g21400.2 0.001 1 0.999 0.001 1	0	0	0	0	0	0	0	0
	1<br>Osjap07g24560.1 0.001 0.366 0.365 0.003 0.973<br>Osjap07g24570.1 0.427 0.995 0.568 0.093 0.998	1<br>Osind04g27100.1 0.016 0.356 0.34 0.09 0.951<br>Osind07g09290.1 0.49 0.965 0.475 0.204 0.813	1<br>Orufi07g22110.1 0.001 0.366 0.365 0.003 0.973<br>Orufi07g22120.1 0.427 0.995 0.568 0.093 0.998	1<br>Oglab07g18190.1 0.001 0.386 0.385 0.003 0.918<br>Oglab07g18200.1 0.427 0.995 0.568 0.093 0.998	1<br>Obart07g21090.1 0.001 0.366 0.365 0.003 0.973<br>Obart07g21100.1 0.458 0.995 0.537 0.179 0.998	1<br>Olong_006539-R1 0.001 0.394 0.393 0.003 0.945<br>Olong_006540-R1 0.498 0.995 0.497 0.062 0.998	1<br>Opunc07g13090.1 0.001 0.394 0.393 0.002 0.885<br>Opunc07g13100.1 0.394 0.995 0.601 0.059 0.998	1<br>Obrac07g13360.2 0.001 0.366 0.365 0.003 0.973<br>Obrac07g13370.3 0.496 0.992 0.496 0.002 0.994	1<br>Lperr07g18240.1 0.394 0.992 0.598 0.061 0.997<br>Lperr07g18250.1 0.001 0.357 0.356 0.003 0.809	1<br>AT1G26460.1 0.399 0.986 0.587 0.041 0.989<br>AT1G26520.1 0.001 0.356 0.355 0.029 0.947
Osind07g25180.1	0	1<br>Osind07g25180.1 0.002 1 0.998 0.002 1	0	0	0	0	0	0	0	0
	1<br>Osjap07g28590.1 0.002 0.688 0.686 0.003 0.822<br>Osjap07g28610.1 0.716 1 0.284 0.543 1	1<br>Osind07g25220.1 0.002 0.688 0.686 0.003 0.961<br>Osind07g25260.1 0.716 1 0.284 0.711 1	1<br>Orufi07g25740.1 0.002 0.688 0.686 0.003 0.823<br>Orufi07g25750.1 0.716 1 0.284 0.543 1	1<br>Oglab07g21250.1 0.032 0.688 0.656 0.003 0.826<br>Oglab07g21260.1 0.716 1 0.284 0.543 1	1<br>Obart07g24850.1 0.007 0.688 0.681 0.025 0.798<br>Obart07g24870.1 0.716 1 0.284 0.709 0.948	1<br>Olong_018533-R1 0.716 1 0.284 0.543 1<br>Olong_020830-R1 0.051 0.686 0.635 0.045 0.983	1<br>Opunc07g23230.1 0.032 0.688 0.656 0.003 0.816<br>Opunc07g23260.1 0.716 1 0.284 0.636 1	1<br>Obrac07g16190.1 0.032 0.688 0.656 0.003 0.817<br>Obrac07g16210.1 0.716 1 0.284 0.57 1	1<br>Lperr07g21550.1 0.032 0.688 0.656 0.003 0.829<br>Lperr07g21560.1 0.716 1 0.284 0.543 1	1<br>AT1G03260.1 0.688 0.988 0.3 0.496 0.996<br>AT2G19150.1 0.048 0.688 0.64 0.021 0.802

Osind07g27190.1	0	1<br>Osind07g27190.1 0.001 1 0.999 0.001 1	0	0	0	0	0	0	0	0
	1<br>Osjap03g17260.1 0.627 0.999 0.372 0.11 0.991<br>Osjap07g30520.1 0.035 0.621 0.586 0.033 0.988	0<br>Osind03g17490.1 0.627 0.999 0.372 0.11 0.991	1<br>Orufi07g27530.1 0.047 0.621 0.574 0.603 0.995<br>Orufi07g27540.2 0.621 1 0.379 0.09 1	1<br>Oglab07g23100.1 0.001 0.621 0.62 0.001 0.987<br>Oglab07g23110.1 0.621 1 0.379 0.088 1	1<br>Obart07g26520.1 0.035 0.621 0.586 0.063 0.988<br>Obart07g26530.1 0.621 1 0.379 0.088 1	1<br>Olong_012949-R1 0.621 1 0.379 0.086 1<br>Olong_012950-R1 0.058 0.621 0.563 0.001 0.984	1<br>Opunc07g24990.1 0.058 0.621 0.563 0.001 0.988<br>Opunc07g25000.1 0.621 1 0.379 0.085 1	1<br>Obrac07g17720.2 0.061 0.62 0.559 0.001 0.986<br>Obrac07g17730.1 0.621 1 0.379 0.092 1	1<br>Lperr03g14060.1 0.621 0.999 0.378 0.08 0.997<br>Lperr07g23360.2 0.058 0.62 0.562 0.001 0.987	0<br>AT1G10930.1 0.051 0.47 0.419 0.363 0.923<br>AT2G38650.1 0.631 1 0.369 0.132 1
Osind07g27670.1	1<br>Osjap07g31020.1 0.008 1 0.992 0.404 1	1<br>Osind07g27670.1 0.008 1 0.992 0.008 1<br>Osind07g27730.1 0.008 1 0.992 0.404 1	0	1<br>Oglab07g23640.1 0.016 1 0.984 0.801 0.998<br>Oglab07g23640.2 0.016 1 0.984 0.813 0.998	1<br>Obart07g27040.1 0.016 1 0.984 0.144 0.32<br>Obart07g27040.2 0.016 1 0.984 0.118 0.299	0	0	0	0	0
	1<br>Osjap03g15620.2 0.73 1 0.27 0.934 0.97<br>Osjap07g30980.1 0.008 0.639 0.631 0.199 0.358	1<br>Osind07g27700.1 0.008 0.664 0.656 0.124 0.258<br>Osind07g27740.1 0.73 1 0.27 0.939 0.972	1<br>Orufi03g15210.1 0.73 1 0.27 0.939 0.972<br>Orufi07g27980.1 0.008 0.664 0.656 0.221 0.34	1<br>Oglab03g14540.1 0.73 1 0.27 0.938 0.971<br>Oglab07g23620.1 0.008 0.664 0.656 0.124 0.258	1<br>Obart07g27030.1 0.008 0.664 0.656 0.425 0.51<br>Obart07g27050.1 0.73 1 0.27 0.939 0.972	1<br>Olong_008697-R2 0.73 1 0.27 0.936 0.971<br>Olong_008701-R1 0.008 0.664 0.656 0.124 0.258	1<br>Opunc07g25410.1 0.008 0.664 0.656 0.129 0.268<br>Opunc07g25450.1 0.73 1 0.27 0.937 0.971	1<br>Obrac03g11770.2 0.73 1 0.27 0.931 0.968<br>Obrac07g18100.1 0.008 0.664 0.656 0.131 0.271	1<br>Lperr03g12860.1 0.73 1 0.27 0.937 0.971<br>Lperr07g23810.1 0.008 0.664 0.656 0.123 0.256	0<br>AT3G66658.2 0.008 0.754 0.746 0.124 0.273
Osind08g03120.1	0	1<br>Osind06g17590.1 0.058 0.989 0.931 0.01 0.991<br>Osind08g03120.1 0.001 1 0.999 0.001 1	0	0	1<br>Obart11g22180.1 0.059 0.995 0.936 0.028 0.98	1<br>Olong_000078-R1 0.144 1 0.856 0.049 1	0	0	0	0
	1<br>Osjap11g26070.1 0.001 0.668 0.667 0.002 0.97<br>Osjap11g26110.1 0.717 1 0.283 0.046 0.285	1<br>Osind01g01210.1 0.001 0.668 0.667 0.002 0.971<br>Osind06g17600.1 0.717 1 0.283 0.054 0.335	1<br>Orufi11g24100.1 0.037 0.655 0.618 0.067 0.972<br>Orufi11g24120.1 0.717 1 0.283 0.386 0.554	1<br>Oglab11g19360.1 0.001 0.668 0.667 0.002 0.971<br>Oglab11g19410.2 0.717 1 0.283 0.009 0.413	0<br>Obart11g22150.1 0.037 0.655 0.618 0.067 0.972	1<br>Olong_022539-R1 0.005 0.668 0.663 0.018 0.968<br>Olong_028213-R1 0.717 1 0.283 0.308 0.494	1<br>Opunc11g18800.1 0.001 0.667 0.666 0.002 0.987<br>Opunc11g18810.1 0.717 0.998 0.281 0.027 0.308	1<br>Obrac11g13070.1 0.052 0.655 0.603 0.002 0.949<br>Obrac11g13130.1 0.717 0.999 0.282 0.036 0.876	1<br>Lperr11g18900.3 0.717 1 0.283 0.003 0.298<br>Lperr12g12660.2 0.041 0.621 0.58 0.058 0.883	0<br>AT2G17780.1 0.716 0.999 0.283 0.06 0.346<br>AT2G18750.1 0.19 0.593 0.403 0.153 0.812
Osind08g06190.1	0	1<br>Osind08g06190.1 0.001 1 0.999 0.001 1<br>Osind08g06240.1 0.002 0.996 0.994 0.013 0.991	1<br>Orufi08g06360.1 0.002 1 0.998 0.014 0.984<br>Orufi08g06360.2 0.002 1 0.998 0.014 0.984	1<br>Oglab08g05190.1 0.002 0.996 0.994 0.001 0.998<br>Oglab08g05190.2 0.002 0.996 0.994 0.001 0.998<br>Oglab08g05190.3 0.002 0.996 0.994 0.001 0.998<br>Oglab08g05190.4 0.002 0.996 0.994 0.002 0.998<br>Oglab08g05190.5 0.002 0.996 0.994 0.002 0.998	0	1<br>Olong_020302-R1 0.002 0.942 0.94 0.002 0.986	0	0	0	0
	0<br>Osjap08g06250.1 0.002 0.649 0.647 0.089 0.974	1<br>Osind03g20810.1 0.011 0.625 0.614 0.145 0.862<br>Osind09g08750.1 0.634 0.994 0.36 0.112 0.893	1<br>Orufi03g23480.1 0.011 0.625 0.614 0.451 0.911<br>Orufi12g06630.1 0.636 0.969 0.333 0.308 0.98	0<br>Oglab10g08280.1 0.627 0.996 0.369 0.109 0.936	1<br>Obart03g22810.1 0.011 0.625 0.614 0.32 0.89<br>Obart09g08510.1 0.634 0.997 0.363 0.027 0.97	1<br>Olong_021247-R1 0.692 0.993 0.301 0.293 0.93<br>Olong_024935-R1 0.04 0.638 0.598 0.344 0.987	1<br>Opunc03g21490.1 0.011 0.625 0.614 0.177 0.734<br>Opunc08g04700.1 0.632 0.872 0.24 0.34 0.958	1<br>Obrac04g06380.1 0.635 0.994 0.359 0.156 0.825<br>Obrac07g13250.1 0.019 0.625 0.606 0.158 0.752	0<br>Lperr03g19020.1 0.017 0.625 0.608 0.28 0.884	0<br>AT3G42170.1 0.377 0.993 0.616 0.089 0.968
Osind08g06790.1	1<br>Osjap08g06650.1 0.002 1 0.998 0.002 1	1<br>Osind08g06790.1 0.002 1 0.998 0.002 1	1<br>Orufi08g06840.1 0.002 1 0.998 0.002 1	1<br>Oglab08g05640.1 0.002 1 0.998 0.002 1	1<br>Obart08g06070.1 0.002 1 0.998 0.002 1	0	1<br>Opunc08g05850.1 0.047 1 0.953 0.068 1	0	0	0
	1<br>Osjap04g21220.1 0.047 0.275 0.228 0.21 0.936<br>Osjap09g20870.1 0.359 0.984 0.625 0.208 0.966	1<br>Osind04g20250.1 0.047 0.275 0.228 0.198 0.94<br>Osind09g20960.1 0.359 0.984 0.625 0.208 0.966	1<br>Orufi04g19250.2 0.047 0.275 0.228 0.21 0.936<br>Orufi09g21960.1 0.359 0.984 0.625 0.2 0.965	1<br>Oglab02g26360.1 0.348 0.996 0.648 0.01 0.994<br>Oglab04g16180.1 0.047 0.275 0.228 0.198 0.94	1<br>Obart02g28440.1 0.348 0.996 0.648 0.093 0.994<br>Obart04g17770.1 0.047 0.275 0.228 0.198 0.94	1<br>Olong_008030-R1 0.047 0.275 0.228 0.232 0.937<br>Olong_008031-R2 0.277 1 0.723 0.106 1	1<br>Opunc04g15550.1 0.047 0.275 0.228 0.206 0.938<br>Opunc04g18740.1 0.295 0.984 0.689 0.088 0.994	1<br>Obrac08g04780.1 0.277 1 0.723 0.098 1<br>Obrac08g04800.1 0.002 0.275 0.273 0.006 0.943	1<br>Lperr08g05690.1 0.277 1 0.723 0.1 1<br>Lperr08g05700.1 0.002 0.275 0.273 0.006 0.943	1<br>AT1G05310.1 0.33 0.982 0.652 0.183 0.99<br>AT4G30800.1 0.047 0.275 0.228 0.208 0.943
Osind08g08550.1	0	1<br>Osind08g08550.1 0.003 1 0.997 0.003 1	0	0	0	0	0	0	0	0
	1	1	1	1	1	0	1	0	1	1

	Osjap08g08130.3 0.531 1 0.469 0.072 1<br>Osjap08g08150.1 0.003 0.422 0.419 0.011 0.188	Osind08g08530.1 0.465 1 0.535 0.161 1<br>Osind08g08570.1 0.003 0.422 0.419 0.011 0.188	Orufi08g08460.1 0.465 1 0.535 0.303 1<br>Orufi08g08480.2 0.003 0.422 0.419 0.011 0.19	Oglab08g06850.1 0.465 1 0.535 0.303 1<br>Oglab08g06860.1 0.003 0.422 0.419 0.014 0.236	Obart08g07340.1 0.465 1 0.535 0.303 1<br>Obart08g07350.1 0.003 0.422 0.419 0.014 0.236	Olong_007960-R1 0.465 1 0.535 0.233 1	Opunc08g07070.1 0.465 1 0.535 0.303 1<br>Opunc08g07080.1 0.003 0.422 0.419 0.011 0.187	Obrac08g05540.1 0.465 0.997 0.532 0.303 0.997	Lperr08g06550.1 0.459 1 0.541 0.294 1<br>Lperr08g06560.1 0.003 0.422 0.419 0.011 0.182	AT3G11470.2 0.465 0.97 0.505 0.3 0.983<br>AT4G24520.1 0.05 0.422 0.372 0.012 0.158
Osind08g20190.1	0	1<br>Osind08g20180.1 0.139 0.975 0.836 0.237 0.449<br>Osind08g20190.1 0.008 1 0.992 0.008 1	1<br>Orufi08g10060.1 0.189 0.992 0.803 0.353 0.575<br>Orufi08g19130.1 0.131 1 0.869 0.305 0.472<br>Orufi08g19130.2 0.131 1 0.869 0.315 0.487	0	1<br>Obart08g17200.1 0.148 1 0.852 0.302 0.839	1<br>Olong_011913-R1 0.131 1 0.869 0.145 0.69	0	0	0	0
	1<br>Osjap08g09660.1 0.238 0.992 0.754 0.336 0.525<br>Osjap08g18160.1 0.033 0.238 0.205 0.876 0.905	0<br>Osind08g20150.1 0.221 0.992 0.771 0.385 0.557	1<br>Orufi08g10050.1 0.238 0.992 0.754 0.395 0.567<br>Orufi08g19160.1 0.033 0.238 0.205 0.88 0.908	1<br>Oglab08g16020.1 0.238 0.992 0.754 0.596 0.856<br>Oglab08g16050.1 0.033 0.238 0.205 0.88 0.908	1<br>Obart08g08800.1 0.246 1 0.754 0.337 0.537<br>Obart08g17210.1 0.033 0.238 0.205 0.852 0.886	1<br>Olong_011909-R1 0.033 0.238 0.205 0.792 0.849<br>Olong_011910-R1 0.238 0.992 0.754 0.097 0.617	0<br>Opunc08g15040.1 0.213 1 0.787 0.345 0.531	1<br>Obrac08g11180.1 0.238 1 0.762 0.39 0.522<br>Obrac08g11210.1 0.033 0.238 0.205 0.822 0.854	0<br>Lperr08g14190.1 0.23 0.943 0.713 0.469 0.652	0
Osind08g22530.1	1<br>Osjap08g20640.1 0.111 0.994 0.883 0.199 0.933	1<br>Osind08g22530.1 0.002 1 0.998 0.002 1<br>Osind08g22680.1 0.111 0.994 0.883 0.199 0.933	1<br>Orufi08g21480.1 0.002 1 0.998 0.001 0.97<br>Orufi08g21480.2 0.002 1 0.998 0.003 0.952<br>Orufi08g21640.1 0.111 0.994 0.883 0.199 0.933	0	0	1<br>Olong_020048-R1 0.002 1 0.998 0.003 0.94	0	0	0	0
	1<br>Osjap05g14370.1 0.491 0.912 0.421 0.004 1<br>Osjap12g00550.1 0.091 0.48 0.389 0.127 0.648	1<br>Osind12g07100.1 0.009 0.471 0.462 0.042 0.813<br>Osind12g11660.1 0.491 0.927 0.436 0.003 1	1<br>Orufi05g13530.1 0.491 0.912 0.421 0.004 1<br>Orufi12g00580.1 0.091 0.48 0.389 0.145 0.74	1<br>Oglab02g26530.1 0.491 0.927 0.436 0.003 1<br>Oglab09g10810.1 0.009 0.486 0.477 0.017 0.871	1<br>Obart09g11820.1 0.009 0.486 0.477 0.017 0.871<br>Obart11g19340.1 0.491 0.927 0.436 0.004 1	1<br>Olong_005330-R1 0.002 0.486 0.484 0.003 0.821<br>Olong_028575-R1 0.514 0.927 0.413 0.004 1	0<br>Opunc08g17290.1 0.204 1 0.796 0.002 0.47	1<br>Obrac03g12080.1 0.551 0.923 0.372 0.173 0.986<br>Obrac12g00030.1 0.026 0.486 0.46 0.436 0.966	0<br>Lperr08g16100.3 0.005 0.569 0.564 0.013 0.982	0
Osind08g24880.1	0	1<br>Osind08g24880.1 0.002 1 0.998 0.002 1	0	0	0	0	0	0	0	0
	1<br>Osjap08g22620.2 0.531 1 0.469 0.005 1<br>Osjap12g20880.1 0.002 0.437 0.435 0.06 0.299	0	1<br>Orufi08g23670.1 0.439 1 0.561 0.004 1<br>Orufi12g18170.1 0.002 0.437 0.435 0.06 0.299	1<br>Oglab04g08170.2 0.531 0.975 0.444 0.005 0.995<br>Oglab12g18580.1 0.002 0.437 0.435 0.06 0.299	1<br>Obart08g21210.1 0.439 1 0.561 0.073 1<br>Obart12g16340.1 0.025 0.437 0.412 0.045 0.276	1<br>Olong_002330-R1 0.531 0.975 0.444 0.005 0.995<br>Olong_017028-R1 0.002 0.437 0.435 0.001 0.254	1<br>Opunc08g19310.1 0.531 1 0.469 0.005 1<br>Opunc12g14850.1 0.002 0.437 0.435 0.092 0.319	1<br>Obrac08g14160.1 0.531 0.96 0.429 0.004 0.929<br>Obrac12g10450.1 0.002 0.437 0.435 0.001 0.167	1<br>Lperr08g17990.1 0.531 1 0.469 0.005 1<br>Lperr12g13600.1 0.011 0.437 0.426 0.036 0.276	0<br>AT3G07170.1 0.531 0.975 0.444 0.005 0.995
Osind08g27620.1	0	1<br>Osind08g27620.1 0.009 1 0.991 0.009 1	0	1<br>Oglab08g20310.1 0.009 1 0.991 0.007 1	1<br>Obart08g23660.1 0.009 1 0.991 0.007 1	1<br>Olong_020567-R1 0.009 1 0.991 0.008 1	0	0	0	0
	1<br>Osjap03g41270.1 0.009 0.387 0.378 0.191 0.227<br>Osjap08g25130.1 0.387 1 0.613 0.519 1	1<br>Osind03g42330.1 0.009 0.377 0.368 0.211 0.258<br>Osind03g44220.1 0.387 0.991 0.604 0.809 0.993	1<br>Orufi03g39770.1 0.009 0.387 0.378 0.204 0.242<br>Orufi08g26140.1 0.387 1 0.613 0.519 1	1<br>Oglab03g36140.1 0.009 0.387 0.378 0.191 0.227<br>Oglab03g37860.1 0.387 0.991 0.604 0.814 0.993	1<br>Obart03g38160.1 0.009 0.387 0.378 0.203 0.242<br>Obart03g39810.1 0.387 0.991 0.604 0.814 0.993	1<br>Olong_007014-R3 0.009 0.377 0.368 0.217 0.257<br>Olong_025249-R1 0.387 0.953 0.566 0.791 0.984	1<br>Opunc03g35260.1 0.009 0.377 0.368 0.248 0.293<br>Opunc03g37040.1 0.387 0.953 0.566 0.814 0.984	1<br>Obrac03g29980.1 0.387 0.943 0.556 0.802 0.981<br>Obrac08g08500.1 0.009 0.377 0.368 0.058 0.115	1<br>Lperr03g33960.1 0.387 0.953 0.566 0.826 0.985<br>Lperr08g10620.1 0.009 0.377 0.368 0.249 0.295	0
Osind09g01630.1	0	1<br>Osind09g01630.1 0.002 1 0.998 0.002 1	0	0	0	0	0	0	0	0
	1<br>Osjap03g00690.5 0.002 0.435 0.433 0.082 1<br>Osjap04g01220.1 0.52 1 0.48 0.654 1	1<br>Osind02g13100.1 0.52 1 0.48 0.664 1<br>Osind03g00750.1 0.002 0.435 0.433 0.004 1	1<br>Orufi03g12670.1 0.002 0.435 0.433 0.118 1<br>Orufi11g09320.2 0.52 1 0.48 0.686 1	1<br>Oglab02g11710.1 0.52 1 0.48 0.675 1<br>Oglab03g00400.1 0.002 0.435 0.433 0.004 1	1<br>Obart03g00560.3 0.002 0.485 0.483 0.107 1<br>Obart04g01130.3 0.52 1 0.48 0.67 1	0<br>Olong_019815-R1 0.414 1 0.586 0.222 1	1<br>Opunc02g10410.1 0.509 1 0.491 0.668 1<br>Opunc11g05910.3 0.002 0.435 0.433 0.004 1	1<br>Obrac03g00410.1 0.002 0.431 0.429 0.079 0.988<br>Obrac04g00850.1 0.516 0.998 0.482 0.654 1	1<br>Lperr02g09810.1 0.518 1 0.482 0.329 1<br>Lperr12g13320.1 0.002 0.435 0.433 0.004 1	1<br>AT2G17695.1 0.012 0.42 0.408 0.01 0.985<br>AT4G10510.1 0.52 0.992 0.472 0.668 0.995
Osind09g02760.1	1<br>Osjap04g11160.1 0.001 1 0.999 0.001 1	1<br>Osind09g02760.1 0.001 1 0.999 0.001 1	1<br>Orufi04g09840.1 0.001 1 0.999 0.001 1	0	0	0	1<br>Opunc04g07220.1 0.017 0.988 0.971 0.005 1	0	1<br>Lperr04g05910.1 0.066 0.993 0.927 0.022 0.997	0

	1<br>Osjap09g02370.1 0.585 0.999 0.414 0.006 0.928<br>Osjap11g14300.1 0.008 0.524 0.516 0.04 0.878	1<br>Osind01g17770.1 0.585 0.999 0.414 0.006 0.928<br>Osind08g20650.1 0.008 0.524 0.516 0.04 0.878	1<br>Orufi10g19500.1 0.563 0.988 0.425 0.022 0.976<br>Orufi11g12980.1 0.008 0.524 0.516 0.039 0.881	1<br>Oglab01g12700.1 0.585 0.999 0.414 0.006 0.928<br>Oglab11g10800.1 0.008 0.524 0.516 0.04 0.878	1<br>Obart04g08610.1 0.544 1 0.456 0.001 1<br>Obart11g12300.1 0.008 0.524 0.516 0.04 0.878	1<br>Olong_024054-R1 0.585 1 0.415 0.002 1<br>Olong_024055-R1 0.01 0.548 0.538 0.001 0.96	1<br>Opunc08g10560.1 0.563 0.986 0.423 0.07 0.993<br>Opunc11g10540.3 0.008 0.524 0.516 0.087 0.889	1<br>Obrac04g04850.1 0.008 0.556 0.548 0.043 0.975<br>Obrac10g11900.4 0.567 0.982 0.415 0.03 0.966	1<br>Lperr02g04400.1 0.555 0.967 0.412 0.048 0.961<br>Lperr11g10010.2 0.008 0.524 0.516 0.037 0.886	1<br>AT1G25360.1 0.57 0.996 0.426 0.019 0.914<br>AT5G44240.1 0.008 0.524 0.516 0.066 0.889
Osind09g03780.1	0	1<br>Osind09g03780.1 0.003 1 0.997 0.003 1	0	0	0	0	0	0	0	0
	1<br>Osjap01g42620.1 0.699 0.909 0.21 0.023 0.341<br>Osjap09g03660.1 0.003 0.678 0.675 0.002 0.578	1<br>Osind09g03790.1 0.003 0.678 0.675 0.002 0.578<br>Osind10g07140.1 0.702 0.921 0.219 0.016 0.313	1<br>Orufi03g41030.1 0.699 0.909 0.21 0.023 0.344<br>Orufi09g04010.1 0.003 0.678 0.675 0.002 0.578	0<br>Oglab05g09200.1 0.693 0.909 0.216 0.014 0.341	1<br>Obart01g37280.1 0.699 0.909 0.21 0.023 0.343<br>Obart09g03600.1 0.003 0.678 0.675 0.002 0.578	1<br>Olong_003911-R1 0.699 0.909 0.21 0.023 0.344<br>Olong_007585-R1 0.009 0.678 0.669 0.164 0.596	1<br>Opunc08g19220.1 0.702 0.909 0.207 0.033 0.352<br>Opunc09g03240.1 0.003 0.675 0.672 0.123 0.577	1<br>Obrac03g29450.2 0.699 0.909 0.21 0.023 0.333<br>Obrac09g01930.1 0.003 0.675 0.672 0.002 0.531	0<br>Lperr09g02590.1 0.003 0.717 0.714 0.135 0.61	1<br>AT3G12160.1 0.69 0.909 0.219 0.023 0.347<br>AT3G52120.1 0.009 0.672 0.663 0.005 0.485
Osind09g09040.1	1<br>Osjap11g06710.1 0.001 1 0.999 0.067 0.999<br>Osjap11g06720.1 0.001 0.999 0.998 0.054 0.981	1<br>Osind09g09040.1 0.001 1 0.999 0.001 1	1<br>Orufi11g05930.1 0.001 1 0.999 0.09 1<br>Orufi11g05940.1 0.001 1 0.999 0.002 1	1<br>Oglab11g05440.1 0.001 0.995 0.994 0.001 0.998	1<br>Obart11g05910.1 0.001 0.88 0.879 0.05 1	0	0	0	0	0
	1<br>Osjap01g24690.1 0.525 0.989 0.464 0.014 0.934<br>Osjap11g06740.8 0.022 0.484 0.462 0.013 0.907	1<br>Osind09g09050.1 0.001 0.481 0.48 0.104 0.994<br>Osind11g05650.1 0.522 1 0.478 0.002 1	1<br>Orufi11g05890.1 0.522 1 0.478 0.003 0.997<br>Orufi11g05900.2 0.001 0.481 0.48 0.002 0.978	1<br>Oglab08g10820.1 0.522 0.955 0.433 0.002 0.867<br>Oglab11g05430.1 0.001 0.503 0.502 0.049 0.944	1<br>Obart01g20930.1 0.525 0.989 0.464 0.006 0.498<br>Obart11g05930.1 0.001 0.481 0.48 0.002 0.998	1<br>Olong_000002-R1 0.001 0.517 0.516 0.117 0.996<br>Olong_005787-R1 0.522 1 0.478 0.002 1	0<br>Opunc11g05960.1 0.425 1 0.575 0.002 1	1<br>Obrac01g15800.1 0.521 0.989 0.468 0.004 0.915<br>Obrac11g03930.1 0.001 0.482 0.481 0.002 0.939	1<br>Lperr05g13220.1 0.522 1 0.478 0.002 1<br>Lperr11g05360.1 0.001 0.481 0.48 0.044 0.995	0<br>AT4G38130.1 0.522 0.955 0.433 0.002 0.782
Osind09g09810.2	1<br>Osjap09g09660.1 0.001 0.984 0.983 0.001 0.615<br>Osjap09g09660.2 0.001 1 0.999 0.001 1<br>Osjap09g09660.3 0.001 1 0.999 0.001 1<br>Osjap09g09660.4 0.001 1 0.999 0.001 1	1<br>Osind09g09810.1 0.001 0.984 0.983 0.001 0.605<br>Osind09g09810.2 0.001 1 0.999 0.001 1	1<br>Orufi09g10340.1 0.001 0.984 0.983 0.001 0.67<br>Orufi09g10340.2 0.001 1 0.999 0.001 1<br>Orufi09g10340.3 0.001 1 0.999 0.001 1	0	0	0	1<br>Opunc09g08150.1 0.001 1 0.999 0.001 1	0	1<br>Lperr09g06610.1 0.001 1 0.999 0.001 0.802<br>Lperr09g06610.2 0.001 1 0.999 0.001 1	0
	1<br>Osjap02g00030.1 0.593 0.846 0.253 0.196 0.692<br>Osjap08g16360.1 0.001 0.563 0.562 0.002 0.984	1<br>Osind02g00030.1 0.593 0.846 0.253 0.188 0.689<br>Osind08g18230.1 0.001 0.563 0.562 0.002 0.984	1<br>Orufi02g00020.1 0.593 0.846 0.253 0.282 0.725<br>Orufi08g17320.1 0.001 0.563 0.562 0.002 0.984	1<br>Oglab01g06450.1 0.656 0.997 0.341 0.674 0.999<br>Oglab08g14350.1 0.001 0.563 0.562 0.002 0.984	1<br>Obart08g15380.1 0.001 0.563 0.562 0.002 0.984<br>Obart09g09660.3 0.566 1 0.434 0.136 1	1<br>Olong_009135-R1 0.001 0.564 0.563 0.086 0.862<br>Olong_009135-R6 0.592 0.99 0.398 0.003 0.983	0<br>Opunc08g12760.1 0.001 0.563 0.562 0.002 0.984<br>Opunc11g05680.1 0.593 0.818 0.225 0.151 0.422	1<br>Obrac09g05000.3 0.592 1 0.408 0.174 1<br>Obrac09g05010.1 0.001 0.564 0.563 0.002 0.988	1<br>Lperr02g00030.2 0.593 0.846 0.253 0.27 0.728<br>Lperr08g12650.1 0.001 0.56 0.559 0.003 0.96	0<br>AT3G17880.1 0.608 1 0.392 0.195 0.997
Osind09g11870.1	0	1<br>Osind09g11870.1 0.004 1 0.996 0.004 1	0	0	0	0	0	0	0	0
	1<br>Osjap08g17850.1 0.004 0.377 0.373 0.001 0.086<br>Osjap10g04710.1 0.377 1 0.623 0.026 1	1<br>Osind08g19910.1 0.004 0.377 0.373 0.001 0.086<br>Osind09g11900.1 0.381 1 0.619 0.033 1	1<br>Orufi03g37310.1 0.377 0.97 0.593 0.112 0.689<br>Orufi09g12390.3 0.004 0.377 0.373 0.034 0.124	1<br>Oglab07g16860.1 0.381 1 0.619 0.021 1<br>Oglab09g10530.1 0.004 0.377 0.373 0.001 0.088	0<br>Obart07g18590.1 0.381 1 0.619 0.021 1	1<br>Olong_013737-R1 0.381 1 0.619 0.033 1<br>Olong_017956-R1 0.004 0.369 0.365 0.002 0.133	1<br>Opunc06g17610.1 0.381 1 0.619 0.082 1<br>Opunc09g10100.1 0.004 0.377 0.373 0.083 0.164	0<br>Obrac04g00470.1 0.419 0.89 0.471 0.121 0.983	0<br>Lperr05g07610.1 0.458 0.767 0.309 0.258 0.797<br>Lperr08g13980.1 0.004 0.377 0.373 0.001 0.093	0
Osind09g15570.1	1<br>Osjap09g15430.2 0.002 1 0.998 0.002 1<br>Osjap09g15430.3 0.002 1 0.998 0.002 1	1<br>Osind09g15570.1 0.002 1 0.998 0.002 1<br>Osind09g15570.2 0.002 1 0.998 0.002 1	1<br>Orufi09g16160.1 0.002 1 0.998 0.002 1<br>Orufi09g16160.2 0.002 1 0.998 0.002 1	1<br>Oglab09g13510.1 0.002 1 0.998 0.002 1<br>Oglab09g13510.2 0.002 1 0.998 0.002 1<br>Oglab09g13510.3 0.002 1 0.998 0.002 1<br>Oglab09g13510.4 0.002 1 0.998 0.002 1	1<br>Obart09g14980.1 0.002 1 0.998 0.002 1<br>Obart09g14980.2 0.002 1 0.998 0.002 1	0	1<br>Opunc09g13420.1 0.002 1 0.998 0.002 1<br>Opunc09g13420.2 0.002 1 0.998 0.002 1	1<br>Obrac09g09090.1 0.002 1 0.998 0.002 1	0	0
	1<br>Osjap02g06650.1 0.01 0.54 0.53 0.01 0.997<br>Osjap09g15430.1 0.544 1 0.456 0.131 1	1<br>Osind02g06940.1 0.01 0.54 0.53 0.01 0.997<br>Osind08g23920.1 0.544 0.993 0.449 0.131 0.988	1<br>Orufi04g24220.1 0.013 0.542 0.529 0.165 1<br>Orufi08g22880.1 0.544 0.993 0.449 0.131 0.988	1<br>Oglab08g19240.1 0.544 0.993 0.449 0.143 0.986<br>Oglab12g18680.1 0.002 0.542 0.54 0.003 1	1<br>Obart02g06180.1 0.01 0.54 0.53 0.01 0.997<br>Obart08g20390.1 0.544 0.993 0.449 0.631 0.988	1<br>Olong_013054-R1 0.547 0.992 0.445 0.149 0.979<br>Olong_024186-R1 0.002 0.542 0.54 0.003 1	1<br>Opunc04g20190.1 0.007 0.542 0.535 0.006 1<br>Opunc08g18560.1 0.544 0.993 0.449 0.128 0.969	1<br>Obrac02g04550.3 0.008 0.54 0.532 0.214 0.997<br>Obrac09g00560.1 0.548 0.998 0.45 0.164 0.966	1<br>Lperr04g18350.1 0.002 0.542 0.54 0.021 1<br>Lperr09g11120.1 0.544 1 0.456 0.156 1	1<br>AT1G68540.1 0.542 0.992 0.45 0.112 1<br>AT2G21270.3 0.003 0.54 0.537 0.059 0.997

Osind09g15570.2	1<br>Osjap09g15430.2 0.002 1 0.998 0.002 1<br>Osjap09g15430.3 0.002 1 0.998 0.002 1	1<br>Osind09g15570.1 0.002 1 0.998 0.002 1<br>Osind09g15570.2 0.002 1 0.998 0.002 1	1<br>Orufi09g16160.1 0.002 1 0.998 0.002 1<br>Orufi09g16160.2 0.002 1 0.998 0.002 1	1<br>Oglab09g13510.1 0.002 1 0.998 0.002 1<br>Oglab09g13510.2 0.002 1 0.998 0.002 1<br>Oglab09g13510.3 0.002 1 0.998 0.002 1<br>Oglab09g13510.4 0.002 1 0.998 0.002 1	1<br>Obart09g14980.1 0.002 1 0.998 0.002 1<br>Obart09g14980.2 0.002 1 0.998 0.002 1	0	1<br>Opunc09g13420.1 0.002 1 0.998 0.002 1<br>Opunc09g13420.2 0.002 1 0.998 0.002 1	1<br>Obrac09g09090.1 0.002 1 0.998 0.002 1	0	0
	1<br>Osjap02g06650.1 0.01 0.565 0.555 0.01 0.997<br>Osjap09g15430.1 0.569 1 0.431 0.131 1	1<br>Osind02g06940.1 0.01 0.565 0.555 0.01 0.997<br>Osind08g23920.1 0.569 0.993 0.424 0.131 0.988	1<br>Orufi04g24220.1 0.014 0.567 0.553 0.165 1<br>Orufi08g22880.1 0.569 0.993 0.424 0.131 0.988	1<br>Oglab08g19240.1 0.569 0.993 0.424 0.143 0.986<br>Oglab12g18680.1 0.002 0.567 0.565 0.003 1	1<br>Obart02g06180.1 0.01 0.565 0.555 0.01 0.997<br>Obart08g20390.1 0.569 0.993 0.424 0.631 0.988	1<br>Olong_008093-R2 0.596 0.991 0.395 0.25 0.996<br>Olong_024186-R1 0.002 0.567 0.565 0.003 1	1<br>Opunc04g20190.1 0.007 0.567 0.56 0.006 1<br>Opunc08g18560.1 0.569 0.993 0.424 0.128 0.969	1<br>Obrac01g09090.1 0.605 0.998 0.393 0.299 0.982<br>Obrac02g04550.3 0.009 0.565 0.556 0.214 0.997	1<br>Lperr04g18350.1 0.002 0.567 0.565 0.021 1<br>Lperr09g11120.1 0.569 1 0.431 0.156 1	1<br>AT1G68540.1 0.567 0.991 0.424 0.112 1<br>AT2G21270.3 0.003 0.565 0.562 0.059 0.997
Osind09g15930.1	0	1<br>Osind09g15930.1 0.001 1 0.999 0.001 1	1<br>Orufi09g16580.1 0.001 1 0.999 0.061 1	1<br>Oglab09g13920.1 0.001 1 0.999 0.061 1	1<br>Obart09g15360.1 0.057 1 0.943 0.001 1	0	0	0	0	0
	1<br>Osjap03g04840.3 0.544 0.887 0.343 0.018 0.769<br>Osjap09g15790.1 0.001 0.544 0.543 0.155 0.993	1<br>Osind08g24670.1 0.004 0.544 0.54 0.138 0.995<br>Osind09g02290.1 0.544 0.887 0.343 0.018 0.769	1<br>Orufi03g04550.5 0.544 0.887 0.343 0.017 0.733<br>Orufi08g23480.1 0.004 0.544 0.54 0.146 0.995	1<br>Oglab02g15910.1 0.037 0.512 0.475 0.001 0.988<br>Oglab09g02290.1 0.544 0.887 0.343 0.02 0.789	1<br>Obart03g04820.1 0.569 0.887 0.318 0.1 0.796<br>Obart08g21020.1 0.056 0.544 0.488 0.086 0.994	1<br>Olong_025005-R2 0.571 0.98 0.409 0.001 0.955<br>Olong_025006-R1 0.105 0.544 0.439 0.031 0.99	1<br>Opunc08g19030.2 0.006 0.544 0.538 0.071 0.976<br>Opunc09g13790.1 0.544 0.98 0.436 0.055 0.958	1<br>Obrac08g14010.2 0.036 0.544 0.508 0.001 0.989<br>Obrac09g09380.2 0.544 0.98 0.436 0.01 0.954	1<br>Lperr09g11500.1 0.001 0.544 0.543 0.105 0.992<br>Lperr09g11510.2 0.544 0.98 0.436 0.01 0.958	1<br>AT2G32000.1 0.544 0.978 0.434 0.012 0.961<br>AT2G43900.1 0.002 0.519 0.517 0.083 0.843
Osind09g18210.1	1<br>Osjap09g17990.1 0.002 1 0.998 0.157 1	1<br>Osind09g18210.1 0.002 1 0.998 0.002 1	0	1<br>Oglab09g15840.1 0.002 1 0.998 0.002 1	1<br>Obart09g17630.1 0.002 1 0.998 0.153 1	0	0	0	0	0
	0<br>Osjap01g39390.1 0.508 0.89 0.382 0.144 0.713	1<br>Osind01g40800.1 0.508 0.89 0.382 0.104 0.917<br>Osind08g26570.1 0.002 0.476 0.474 0.004 1	1<br>Orufi09g19030.1 0.002 0.476 0.474 0.004 1<br>Orufi09g19040.1 0.489 1 0.511 0.003 1	0<br>Oglab06g16980.1 0.489 0.897 0.408 0.005 0.903	1<br>Obart06g18820.1 0.489 0.897 0.408 0.005 0.903<br>Obart08g22690.1 0.016 0.476 0.46 0.265 1	1<br>Olong_007765-R1 0.002 0.476 0.474 0.004 1<br>Olong_023444-R1 0.534 0.89 0.356 0.35 0.752	1<br>Opunc01g33270.1 0.508 0.89 0.382 0.155 0.871<br>Opunc08g20790.1 0.002 0.476 0.474 0.004 1	1<br>Obrac01g26370.4 0.508 0.884 0.376 0.313 0.74<br>Obrac09g11280.2 0.002 0.476 0.474 0.004 1	1<br>Lperr01g29580.1 0.508 0.89 0.382 0.162 0.862<br>Lperr09g13540.1 0.002 0.476 0.474 0.273 1	1<br>AT1G75760.1 0.002 0.476 0.474 0.004 1<br>AT3G48520.1 0.508 0.888 0.38 0.316 0.725
Osind09g19090.1	0	1<br>Osind09g19090.1 0.003 1 0.997 0.003 1	0	0	0	0	0	0	0	0
	1<br>Osjap09g18870.1 0.041 0.403 0.362 0.007 0.894<br>Osjap09g19010.1 0.406 0.991 0.585 0.078 0.86	0<br>Osind09g19180.1 0.406 0.991 0.585 0.087 0.965	1<br>Orufi09g20000.1 0.041 0.403 0.362 0.007 0.894<br>Orufi09g20120.1 0.406 0.991 0.585 0.078 0.86	1<br>Oglab02g23430.1 0.432 0.991 0.559 0.021 0.678<br>Oglab09g16620.2 0.003 0.417 0.414 0.002 0.907	1<br>Obart09g18500.1 0.041 0.403 0.362 0.007 0.894<br>Obart09g18580.1 0.406 0.991 0.585 0.075 0.839	1<br>Olong_014190-R1 0.041 0.403 0.362 0.007 0.893<br>Olong_014662-R1 0.472 1 0.528 0.092 0.823	1<br>Opunc02g22590.1 0.432 0.991 0.559 0.025 0.802<br>Opunc09g16870.1 0.041 0.403 0.362 0.007 0.894	1<br>Obrac02g17760.1 0.432 0.991 0.559 0.025 0.803<br>Obrac09g11940.8 0.026 0.403 0.377 0.16 0.825	1<br>Lperr09g14330.1 0.041 0.403 0.362 0.007 0.894<br>Lperr09g14410.1 0.409 0.991 0.582 0.033 0.841	1<br>AT1G29420.1 0.041 0.397 0.356 0.007 0.915<br>AT3G53450.1 0.458 0.991 0.533 0.023 0.879
Osind09g20560.1	1<br>Osjap09g20460.1 0.001 0.998 0.997 0.001 0.986	1<br>Osind09g20560.1 0.001 1 0.999 0.001 1	1<br>Orufi09g21510.1 0.001 0.978 0.977 0.001 0.999	1<br>Oglab09g18200.1 0.001 0.998 0.997 0.001 0.985	1<br>Obart09g19980.1 0.067 0.998 0.931 0.165 0.99<br>Obart09g19980.2 0.001 0.998 0.997 0.001 0.984	1<br>Olong_014589-R1 0.117 0.985 0.868 0.018 0.991<br>Olong_014589-R2 0.117 0.985 0.868 0.018 0.991	0	0	0	0
	1<br>Osjap02g21530.2 0.024 0.581 0.557 0.096 0.943<br>Osjap09g20430.1 0.623 0.998 0.375 0.085 0.962	1<br>Osind02g21430.2 0.024 0.581 0.557 0.096 0.943<br>Osind09g20550.1 0.623 0.998 0.375 0.032 0.982	1<br>Orufi02g19810.2 0.024 0.581 0.557 0.096 0.943<br>Orufi09g21500.1 0.623 0.998 0.375 0.085 0.962	1<br>Oglab02g17850.1 0.024 0.581 0.557 0.095 0.914<br>Oglab09g18170.1 0.623 0.998 0.375 0.079 0.966	1<br>Obart04g25370.1 0.047 0.589 0.542 0.176 0.967<br>Obart09g19950.1 0.623 0.998 0.375 0.079 0.966	1<br>Olong_014116-R1 0.064 0.589 0.525 0.069 0.957<br>Olong_027417-R1 0.625 0.982 0.357 0.135 0.711	1<br>Opunc09g18280.1 0.618 0.998 0.38 0.058 0.841<br>Opunc09g18290.2 0.001 0.59 0.589 0.001 0.97	1<br>Obrac09g13140.3 0.623 0.998 0.375 0.049 0.806<br>Obrac09g13160.3 0.001 0.596 0.595 0.001 0.996	1<br>Lperr09g15890.1 0.622 0.999 0.377 0.048 0.95<br>Lperr09g15920.1 0.001 0.59 0.589 0.001 0.957	0<br>AT1G18160.1 0.021 0.589 0.568 0.064 0.958
Osind09g20780.1	0	1<br>Osind09g20780.1 0.001 1 0.999 0.001 1	0	0	0	0	0	0	0	0
	1<br>Osjap09g20690.2 0.666 1 0.334 0.002 1	0<br>Osind03g45910.1 0.002 0.324 0.322 0.04 0.557	1<br>Orufi09g21760.1 0.666 1 0.334 0.002 1	1<br>Oglab09g18370.1 0.666 1 0.334 0.002 1	1<br>Obart09g20140.1 0.666 1 0.334 0.002 1	0<br>Olong_005529-R1 0.666 0.996 0.33 0.002 0.976	1<br>Opunc01g19840.3 0.001 0.644 0.643 0.001 0.989	1<br>Obrac09g13330.6 0.666 0.994 0.328 0.002 0.943	1<br>Lperr09g16140.1 0.666 1 0.334 0.002 1	1<br>AT5G23880.1 0.001 0.643 0.642 0.001 0.986

	Osjap09g20710.1 0.001 0.644 0.643 0.062 0.99	Osind06g10990.1 0.666 0.996 0.33 0.002 0.976	Orufi09g21770.1 0.001 0.644 0.643 0.062 0.99	Oglab09g18380.1 0.001 0.55 0.549 0.001 0.918	Obart09g20150.1 0.001 0.644 0.643 0.001 0.989	Olong_005579-R1 0.066 0.392 0.326 0.003 0.995	Opunc09g18520.1 0.666 0.998 0.332 0.002 0.998	Obrac09g13340.1 0.001 0.644 0.643 0.049 0.99	Lperr09g16150.1 0.001 0.644 0.643 0.002 0.988	AT5G37710.1 0.666 1 0.334 0.002 0.995
Osind10g01380.1	0	1<br>Osind10g01380.1 0.001 1 0.999 0.001 1	0	0	0	0	1<br>Opunc10g01380.1 0.006 1 0.994 0.001 1	0	0	0
	1<br>Osjap10g01910.1 0.001 0.734 0.733 0.002 0.966<br>Osjap10g01940.1 0.748 1 0.252 0.003 1	0<br>Osind01g32780.1 0.764 0.994 0.23 0.127 0.997<br>Osind10g01350.1 0.134 0.57 0.436 0.783 0.94	1<br>Orufi10g01690.2 0.001 0.737 0.736 0.001 0.985<br>Orufi10g01700.1 0.737 1 0.263 0.178 1	1<br>Oglab10g01630.1 0.001 0.737 0.736 0.001 0.985<br>Oglab10g01660.1 0.748 1 0.252 0.003 1	1<br>Obart10g01630.2 0.001 0.734 0.733 0.002 0.966<br>Obart10g01650.1 0.748 1 0.252 0.003 1	0<br>Olong_003375-R1 0.794 0.995 0.201 0.003 0.928<br>Olong_028637-R1 0.139 0.574 0.435 0.015 0.931	1<br>Opunc01g27000.1 0.764 0.994 0.23 0.117 0.997<br>Opunc10g01380.4 0.006 0.737 0.731 0.003 0.967	1<br>Obrac10g00910.1 0.006 0.737 0.731 0.071 0.916<br>Obrac10g00920.2 0.773 1 0.227 0.003 1	1<br>Lperr07g15810.2 0.008 0.742 0.734 0.097 1<br>Lperr10g01310.1 0.748 1 0.252 0.23 1	0<br>AT5G16120.2 0.773 1 0.227 0.087 0.989
Osind10g01570.1	1<br>Osjap10g02190.1 0.006 1 0.994 0.072 1	1<br>Osind10g01570.1 0.006 1 0.994 0.006 1	1<br>Orufi10g01900.1 0.006 1 0.994 0.103 1	0	1<br>Obart10g01830.1 0.006 1 0.994 0.058 0.543	1<br>Olong_016500-R1 0.006 1 0.994 0.002 0.47	0	1<br>Obrac10g01030.1 0.144 0.952 0.808 0.311 0.97	0	0
	0<br>Osjap10g02260.1 0.162 0.725 0.563 0.032 0.225	1<br>Osind10g01540.1 0.635 1 0.365 0.312 0.473<br>Osind10g01590.1 0.006 0.587 0.581 0.314 0.948	0<br>Orufi10g01980.1 0.162 0.725 0.563 0.032 0.225	1<br>Oglab10g01810.1 0.018 0.563 0.545 0.659 0.84<br>Oglab10g01850.1 0.569 0.91 0.341 0.144 0.366	1<br>Obart10g01810.1 0.018 0.563 0.545 0.666 0.879<br>Obart10g01860.1 0.569 0.91 0.341 0.144 0.366	1<br>Olong_016498-R1 0.018 0.563 0.545 0.617 0.843<br>Olong_016499-R1 0.635 0.952 0.317 0.295 0.405	1<br>Opunc10g01450.1 0.006 0.563 0.557 0.697 0.891<br>Opunc10g01540.1 0.629 1 0.371 0.516 1	0<br>Obrac10g01200.1 0.21 0.551 0.341 0.902 0.989	0<br>Lperr10g01460.1 0.03 0.599 0.569 0.72 0.921	0
Osind10g02110.1	0	1<br>Osind10g02110.1 0.001 1 0.999 0.001 1	0	1<br>Oglab10g02440.1 0.001 0.972 0.971 0.001 0.974<br>Oglab10g02440.2 0.001 0.952 0.951 0.001 0.972	1<br>Obart10g02510.1 0.001 0.972 0.971 0.001 0.972<br>Obart10g02510.2 0.001 0.952 0.951 0.001 0.971	0	0	0	0	0
	1<br>Osjap10g00540.1 0.001 0.541 0.54 0.001 0.924<br>Osjap10g02950.1 0.546 0.896 0.35 0.002 0.928	1<br>Osind10g00350.1 0.001 0.541 0.54 0.001 0.914<br>Osind11g07740.1 0.546 0.952 0.406 0.003 0.934	1<br>Orufi10g02500.1 0.027 0.541 0.514 0.028 0.958<br>Orufi11g08730.5 0.655 0.952 0.297 0.212 0.943	0<br>Oglab10g02440.4 0.001 0.541 0.54 0.002 0.774	1<br>Obart10g00500.1 0.001 0.541 0.54 0.001 0.925<br>Obart11g08380.1 0.655 0.92 0.265 0.194 0.911	1<br>Olong_016648-R3 0.355 0.972 0.617 0.007 0.993<br>Olong_028710-R1 0.037 0.348 0.311 0.004 0.979	1<br>Opunc10g02120.1 0.001 0.541 0.54 0.001 0.921<br>Opunc11g08150.1 0.546 0.92 0.374 0.003 0.898	1<br>Obrac03g10950.1 0.001 0.541 0.54 0.001 0.916<br>Obrac11g05340.1 0.546 0.919 0.373 0.002 0.909	1<br>Lperr10g00410.1 0.001 0.541 0.54 0.003 0.929<br>Lperr10g01950.1 0.546 0.92 0.374 0.003 0.882	0<br>AT1G21230.1 0.006 0.391 0.385 0.01 0.419<br>AT4G00830.1 0.648 0.919 0.271 0.194 0.891
Osind10g19230.1	0	1<br>Osind10g19230.1 0.002 1 0.998 0.002 1	1<br>Orufi10g20960.1 0.002 1 0.998 0.002 1	1<br>Oglab10g16090.1 0.002 0.992 0.99 0.001 0.861	1<br>Obart10g19720.1 0.002 1 0.998 0.002 1	0	1<br>Opunc10g18180.1 0.002 0.992 0.99 0.002 0.85	0	0	0
	1<br>Osjap10g22750.1 0.509 0.992 0.483 0.061 0.836<br>Osjap10g22760.1 0.002 0.486 0.484 0.003 0.993	1<br>Osind01g20890.1 0.519 0.992 0.473 0.154 0.668<br>Osind08g03990.1 0.046 0.464 0.418 0.057 0.95	1<br>Orufi01g19490.1 0.519 0.992 0.473 0.171 0.72<br>Orufi08g04140.1 0.046 0.464 0.418 0.057 0.95	1<br>Oglab01g14760.1 0.519 0.992 0.473 0.2 0.827<br>Oglab08g03250.2 0.046 0.464 0.418 0.052 0.955	1<br>Obart01g17550.1 0.519 0.992 0.473 0.174 0.721<br>Obart08g03750.1 0.046 0.464 0.418 0.057 0.95	1<br>Olong_006312-R1 0.516 0.986 0.47 0.067 0.835<br>Olong_006313-R1 0.002 0.486 0.484 0.003 0.993	1<br>Opunc01g18040.1 0.519 0.992 0.473 0.174 0.822<br>Opunc08g03700.1 0.017 0.464 0.447 0.011 0.951	1<br>Obrac01g13330.1 0.525 0.992 0.467 0.196 0.825<br>Obrac10g13120.1 0.002 0.486 0.484 0.004 0.993	1<br>Lperr10g15590.1 0.516 0.992 0.476 0.07 0.79<br>Lperr10g15600.1 0.002 0.482 0.48 0.003 0.979	1<br>AT1G32080.1 0.521 0.988 0.467 0.188 0.82<br>AT2G32260.1 0.044 0.465 0.421 0.078 0.825
Osind11g00690.1	1<br>Osjap11g01220.1 0.001 0.999 0.998 0.001 0.955<br>Osjap12g01140.1 0.001 0.999 0.998 0.001 0.953	1<br>Osind11g00690.1 0.001 1 0.999 0.001 1<br>Osind12g01290.1 0.001 0.999 0.998 0.001 0.954	1<br>Orufi11g01000.1 0.001 0.999 0.998 0.001 0.951<br>Orufi12g01130.1 0.001 0.999 0.998 0.001 0.953	1<br>Oglab12g00800.1 0.001 0.999 0.998 0.001 0.956	1<br>Obart11g01060.1 0.001 0.999 0.998 0.001 0.954	0	1<br>Opunc12g00940.1 0.001 0.999 0.998 0.001 0.937	0	0	0
	1<br>Osjap03g13100.1 0.523 0.999 0.476 0.097 0.593<br>Osjap07g30390.1 0.01 0.462 0.452 0.013 0.944	1<br>Osind03g12930.1 0.523 0.999 0.476 0.121 0.604<br>Osind07g27070.1 0.007 0.462 0.455 0.006 0.946	1<br>Orufi03g17220.1 0.53 0.999 0.469 0.027 0.912<br>Orufi07g27420.1 0.01 0.462 0.452 0.013 0.946	1<br>Oglab03g12120.1 0.523 0.999 0.476 0.121 0.604<br>Oglab07g22980.1 0.007 0.462 0.455 0.007 0.94	1<br>Obart03g12120.1 0.523 0.999 0.476 0.121 0.604<br>Obart12g00790.1 0.001 0.462 0.461 0.002 0.949	1<br>Olong_007715-R1 0.527 0.999 0.472 0.009 0.911<br>Olong_008211-R1 0.007 0.462 0.455 0.006 0.946	1<br>Opunc03g11680.1 0.523 0.999 0.476 0.148 0.924<br>Opunc11g00920.1 0.001 0.462 0.461 0.142 0.951	1<br>Obrac03g09720.2 0.523 0.999 0.476 0.142 0.923<br>Obrac12g00780.1 0.001 0.462 0.461 0.11 0.953	0<br>Lperr11g00920.1 0.001 0.462 0.461 0.002 0.949	1<br>AT2G34940.1 0.001 0.462 0.461 0.002 0.955<br>AT3G46740.1 0.526 1 0.474 0.161 0.928
Osind11g00780.1	0	1<br>Osind11g00780.1 0.001 1 0.999 0.001 1<br>Osind12g01380.1 0.003 0.881 0.878 0.211 0.964	1<br>Orufi11g01080.1 0.028 1 0.972 0.002 1	0	0	0	0	0	0	0
	1<br>Osjap11g01340.1 0.569 1 0.431 0.04 1	0<br>Osind05g13850.1 0.632 0.999 0.367 0.2 0.925	1<br>Orufi12g01220.1 0.624 1 0.376 0.003 1	1<br>Oglab12g00910.1 0.622 1 0.378 0.11 1	1<br>Obart12g00900.1 0.622 1 0.378 0.083 1	1<br>Olong_005170-R1 0.634 0.991 0.357 0.123 0.942	1<br>Opunc12g01050.1 0.618 0.996 0.378 0.092 0.988	0<br>Obrac12g00870.5 0.618 0.999 0.381 0.095 0.997	1<br>Lperr11g01030.1 0.003 0.488 0.485 0.098 1	1<br>AT2G30520.1 0.003 0.488 0.485 0.042 1

	Osjap12g01290.2 0.003 0.488 0.485 0.011 1	Osind08g02040.1 0.008 0.351 0.343 0.041 0.637	Orufi12g01240.1 0.028 0.488 0.46 0.002 1	Oglab12g00920.1 0.031 0.488 0.457 0.05 1	Obart12g00910.1 0.003 0.488 0.485 0.015 1	Olong_028818-R1 0.003 0.488 0.485 0.014 1	Opunc12g01060.1 0.003 0.488 0.485 0.015 1		Lperr12g01130.1 0.593 0.998 0.405 0.01 0.99	AT5G01930.1 0.625 0.992 0.367 0.085 0.915
Osind11g01990.1	1<br>Osjap11g02640.1 0.002 1 0.998 0.002 1<br>Osjap12g02610.1 0.002 1 0.998 0.002 1	1<br>Osind11g01990.1 0.002 1 0.998 0.002 1<br>Osind12g02780.1 0.002 1 0.998 0.002 1	1<br>Orufi11g02320.1 0.002 1 0.998 0.013 1<br>Orufi12g02480.1 0.002 1 0.998 0.002 1	1<br>Oglab11g02200.1 0.002 1 0.998 0.002 1<br>Oglab12g02130.1 0.002 1 0.998 0.002 1	1<br>Obart12g02080.1 0.023 1 0.977 0.002 1	0	0	0	0	0
	1<br>Osjap07g04360.1 0.033 0.669 0.636 0.018 0.925<br>Osjap12g12870.1 0.673 0.949 0.276 0.055 0.417	1<br>Osind11g02010.1 0.014 0.669 0.655 0.001 0.994<br>Osind12g10020.1 0.673 0.949 0.276 0.055 0.417	1<br>Orufi12g02470.1 0.045 0.669 0.624 0.04 0.988<br>Orufi12g10750.1 0.671 0.949 0.278 0.045 0.364	1<br>Oglab07g03810.1 0.033 0.669 0.636 0.019 0.987<br>Oglab12g09880.1 0.673 0.949 0.276 0.051 0.385	0<br>Obart11g02350.1 0.021 0.669 0.648 0.024 0.993	1<br>Olong_012584-R1 0.066 0.622 0.556 0.002 0.965<br>Olong_017316-R1 0.671 0.949 0.278 0.045 0.365	1<br>Opunc11g02190.1 0.002 0.669 0.667 0.003 0.989<br>Opunc12g08740.1 0.671 0.949 0.278 0.04 0.324	1<br>Obrac05g10860.1 0.051 0.669 0.618 0.097 0.987<br>Obrac12g07170.1 0.671 0.94 0.269 0.071 0.57	1<br>Lperr12g02190.1 0.002 0.669 0.667 0.003 0.989<br>Lperr12g08370.1 0.669 0.949 0.28 0.047 0.391	0<br>AT5G27320.1 0.014 0.643 0.629 0.003 0.98
Osind11g02250.1	0	1<br>Osind11g02250.1 0.002 1 0.998 0.002 1	0	0	0	0	0	0	0	0
	1<br>Osjap09g12000.1 0.071 0.404 0.333 0.655 0.926<br>Osjap12g02750.1 0.44 1 0.56 0.008 1	1<br>Osind08g20070.1 0.147 0.416 0.269 0.719 0.937<br>Osind11g02130.1 0.442 1 0.558 0.113 1	1<br>Orufi12g02610.1 0.44 1 0.56 0.008 1<br>Orufi12g02670.4 0.002 0.431 0.429 0.127 0.918	1<br>Oglab08g15880.1 0.064 0.416 0.352 0.648 0.937<br>Oglab11g02320.1 0.442 1 0.558 0.113 1	1<br>Obart09g02750.1 0.058 0.404 0.346 0.659 0.928<br>Obart12g02200.1 0.44 1 0.56 0.008 1	1<br>Olong_007877-R1 0.053 0.404 0.351 0.581 0.919<br>Olong_021332-R1 0.442 1 0.558 0.113 1	1<br>Opunc03g10180.1 0.1 0.418 0.318 0.367 0.968<br>Opunc11g02310.1 0.44 1 0.56 0.008 1	1<br>Obrac08g13200.1 0.149 0.46 0.311 0.609 0.964<br>Obrac11g01530.1 0.498 0.991 0.493 0.385 0.997	0<br>Lperr02g17220.1 0.06 0.46 0.4 0.632 0.986	0<br>AT3G26330.1 0.033 0.404 0.371 0.588 0.934<br>AT5G03990.1 0.427 0.678 0.251 0.089 0.467
Osind11g05410.1	0	1<br>Osind11g05410.1 0.002 1 0.998 0.002 1	0	0	0	0	0	0	0	0
	1<br>Osjap11g06260.1 0.628 1 0.372 0.002 1<br>Osjap11g06280.1 0.002 0.625 0.623 0.003 0.995	0<br>Osind11g05410.2 0.002 0.625 0.623 0.003 0.995	0<br>Orufi11g05470.1 0.002 0.625 0.623 0.003 0.995	1<br>Oglab11g05130.1 0.628 0.987 0.359 0.004 0.965<br>Oglab11g05180.1 0.002 0.625 0.623 0.005 0.99	1<br>Obart01g33570.1 0.025 0.602 0.577 0.278 0.978<br>Obart11g05600.1 0.628 0.987 0.359 0.004 0.965	1<br>Olong_000031-R1 0.002 0.625 0.623 0.003 0.995<br>Olong_000032-R1 0.628 0.987 0.359 0.005 0.963	1<br>Opunc11g05330.1 0.002 0.625 0.623 0.003 0.995<br>Opunc11g05360.1 0.628 1 0.372 0.004 1	0<br>Obrac04g18640.1 0.07 0.605 0.535 0.598 0.987	0<br>Lperr01g28980.1 0.013 0.605 0.592 0.342 0.984	0<br>AT3G06830.1 0.059 0.621 0.562 0.396 0.995
Osind11g15920.1	0	1<br>Osind11g15920.1 0.002 1 0.998 0.002 1	1<br>Orufi11g20460.1 0.109 0.985 0.876 0.348 0.916<br>Orufi11g20460.2 0.112 1 0.888 0.371 1<br>Orufi11g20460.3 0.109 1 0.891 0.366 1	0	0	0	1<br>Opunc11g15810.3 0.109 0.921 0.812 0.125 0.99	0	1<br>Lperr11g15410.3 0.109 0.985 0.876 0.298 0.998	0
	1<br>Osjap06g19970.1 0.109 0.744 0.635 0.321 0.904<br>Osjap11g22060.1 0.747 0.988 0.241 0.599 0.955	0<br>Osind11g15950.1 0.109 0.868 0.759 0.8 0.987	0<br>Orufi06g18160.1 0.109 0.744 0.635 0.053 0.931	1<br>Oglab06g15540.1 0.109 0.744 0.635 0.243 0.893<br>Oglab11g16960.1 0.747 0.985 0.238 0.419 0.931	1<br>Obart06g17130.1 0.109 0.744 0.635 0.053 0.931<br>Obart11g19100.1 0.747 0.985 0.238 0.701 0.964	1<br>Olong_009798-R1 0.109 0.744 0.635 0.358 0.955<br>Olong_028838-R1 0.747 0.988 0.241 0.473 0.941	1<br>Opunc06g15530.1 0.067 0.744 0.677 0.164 0.938<br>Opunc11g15810.2 0.747 0.988 0.241 0.664 0.873	1<br>Obrac06g11630.2 0.109 0.744 0.635 0.471 0.912<br>Obrac11g11210.1 0.747 0.988 0.241 0.599 0.955	1<br>Lperr06g14140.1 0.159 0.744 0.585 0.007 0.922<br>Lperr11g15410.2 0.749 0.985 0.236 0.597 0.94	0<br>AT5G60230.2 0.107 0.695 0.588 0.024 0.953
Osind11g17120.1	1<br>Osjap11g23010.1 0.004 0.992 0.988 0.002 0.589	1<br>Osind11g17120.1 0.004 1 0.996 0.004 1	1<br>Orufi11g21270.1 0.004 0.992 0.988 0.002 0.538	0	0	0	0	0	0	0
	1<br>Osjap10g07190.1 0.012 0.669 0.657 0.132 0.882<br>Osjap11g23280.1 0.669 0.992 0.323 0.285 0.467	1<br>Osind07g22620.1 0.062 0.619 0.557 0.157 0.93<br>Osind11g17350.1 0.623 0.992 0.369 0.433 0.864	1<br>Orufi10g06540.1 0.012 0.669 0.657 0.132 0.882<br>Orufi11g21510.1 0.669 0.992 0.323 0.285 0.467	1<br>Oglab10g05750.1 0.012 0.669 0.657 0.128 0.882<br>Oglab11g17490.1 0.669 0.992 0.323 0.285 0.467	1<br>Obart04g09320.1 0.767 0.992 0.225 0.376 0.428<br>Obart10g06170.1 0.012 0.669 0.657 0.132 0.882	1<br>Olong_020397-R1 0.004 0.584 0.58 0.006 0.787<br>Olong_023942-R1 0.669 0.992 0.323 0.349 0.515	1<br>Opunc06g04020.1 0.77 0.996 0.226 0.534 0.607<br>Opunc10g05480.1 0.012 0.669 0.657 0.132 0.882	1<br>Obrac04g04990.1 0.759 0.992 0.233 0.158 0.31<br>Obrac10g03800.1 0.012 0.611 0.599 0.155 0.885	1<br>Lperr04g06250.2 0.759 0.992 0.233 0.709 0.762<br>Lperr11g04260.1 0.027 0.626 0.599 0.249 0.427	0<br>AT2G21110.1 0.031 0.615 0.584 0.129 0.941
Osind11g17390.1	0	1<br>Osind11g17350.1 0.07 0.98 0.91 0.001 0.992<br>Osind11g17390.1 0.002 1 0.998 0.002 1	1<br>Orufi11g21490.1 0.002 0.997 0.995 0.088 0.76	0	0	0	0	0	0	0
	1	1	1	1	1	1	1	1	0	0

	Osjap11g23270.1 0.449 0.997 0.548 0.184 0.651<br>Osjap11g23280.1 0.07 0.429 0.359 0.456 0.955	Osind11g17360.1 0.449 0.997 0.548 0.156 0.559<br>Osind11g17370.1 0.07 0.429 0.359 0.24 0.938	Orufi10g18830.1 0.076 0.437 0.361 0.48 0.821<br>Orufi11g21400.1 0.443 0.974 0.531 0.187 0.524	Oglab11g17470.1 0.429 0.98 0.551 0.042 0.513<br>Oglab11g17490.1 0.047 0.429 0.382 0.42 0.955	Obart02g27080.1 0.049 0.437 0.388 0.303 0.902<br>Obart03g04520.1 0.46 0.983 0.523 0.004 0.325	Olong_023942-R1 0.07 0.429 0.359 0.506 0.959<br>Olong_027580-R1 0.429 0.98 0.551 0.054 0.519	Opunc10g16000.1 0.073 0.423 0.35 0.642 0.876<br>Opunc11g04080.1 0.423 0.983 0.56 0.038 0.488	Obrac09g13360.3 0.061 0.431 0.37 0.63 0.894<br>Obrac11g11670.1 0.455 0.973 0.518 0.002 0.429	Lperr11g16280.5 0.426 0.992 0.566 0.046 0.496	AT1G16760.1 0.023 0.429 0.406 0.611 0.933
Osind11g17970.1	0	1<br>Osind11g17970.1 0.002 1 0.998 0.002 1	0	0	0	0	0	0	0	0
	1<br>Osjap11g25490.1 0.69 0.995 0.305 0.595 0.872<br>Osjap11g25500.1 0.002 0.661 0.659 0.003 0.993	1<br>Osind04g07350.1 0.13 0.624 0.494 0.581 0.989<br>Osind11g17960.1 0.69 1 0.31 0.682 1	1<br>Orufi11g23670.1 0.69 1 0.31 0.682 1<br>Orufi11g23680.1 0.002 0.661 0.659 0.003 0.995	1<br>Oglab11g18980.1 0.69 1 0.31 0.682 1<br>Oglab11g18990.1 0.002 0.661 0.659 0.099 0.994	1<br>Obart11g21800.1 0.69 1 0.31 0.682 1<br>Obart11g21810.1 0.002 0.661 0.659 0.098 0.994	1<br>Olong_008005-R1 0.69 0.998 0.308 0.664 0.998<br>Olong_009981-R1 0.059 0.626 0.567 0.343 0.894	0<br>Opunc11g18100.1 0.207 0.63 0.423 0.284 0.977<br>Opunc11g18340.1 0.69 0.998 0.308 0.682 0.998	0<br>Obrac11g12550.1 0.153 0.623 0.47 0.563 0.978<br>Obrac11g12870.1 0.675 0.998 0.323 0.641 0.998	1<br>Lperr04g06180.1 0.033 0.63 0.597 0.525 0.998<br>Lperr11g18340.1 0.697 0.998 0.301 0.693 0.998	0
Osind12g01290.1	1<br>Osjap11g01220.1 0.001 1 0.999 0.001 1<br>Osjap12g01140.1 0.001 1 0.999 0.001 1	1<br>Osind11g00690.1 0.001 0.954 0.953 0.001 0.999<br>Osind12g01290.1 0.001 1 0.999 0.001 1	1<br>Orufi11g01000.1 0.001 1 0.999 0.001 1<br>Orufi12g01130.1 0.001 1 0.999 0.001 1	1<br>Oglab12g00800.1 0.001 1 0.999 0.001 1	1<br>Obart11g01060.1 0.001 1 0.999 0.001 1	0	1<br>Opunc12g00940.1 0.001 1 0.999 0.001 1	0	0	0
	1<br>Osjap03g13100.1 0.502 0.981 0.479 0.097 0.622<br>Osjap07g30390.1 0.01 0.366 0.356 0.013 0.798	1<br>Osind03g12930.1 0.502 0.981 0.479 0.121 0.633<br>Osind07g27070.1 0.007 0.37 0.363 0.006 0.783	1<br>Orufi03g17220.1 0.509 1 0.491 0.027 1<br>Orufi07g27420.1 0.01 0.37 0.36 0.013 0.783	1<br>Oglab03g12120.1 0.502 0.981 0.479 0.121 0.633<br>Oglab07g22980.1 0.007 0.366 0.359 0.007 0.783	1<br>Obart03g12120.1 0.502 0.981 0.479 0.121 0.633<br>Obart10g06750.1 0.021 0.401 0.38 0.151 0.885	1<br>Olong_007715-R1 0.505 1 0.495 0.007 1<br>Olong_008211-R1 0.007 0.37 0.363 0.006 0.783	1<br>Opunc03g11680.1 0.502 1 0.498 0.148 1<br>Opunc11g00920.1 0.001 0.37 0.369 0.142 0.816	1<br>Obrac03g09720.2 0.502 1 0.498 0.142 1<br>Obrac10g04150.1 0.019 0.398 0.379 0.036 0.868	1<br>Lperr03g10720.1 0.502 0.993 0.491 0.148 0.951<br>Lperr11g00920.1 0.001 0.37 0.369 0.002 0.792	1<br>AT2G34940.1 0.001 0.37 0.369 0.002 0.793<br>AT3G46740.1 0.505 1 0.495 0.161 1
Osind12g02780.1	1<br>Osjap11g02640.1 0.002 1 0.998 0.002 1<br>Osjap12g02610.1 0.002 1 0.998 0.002 1	1<br>Osind11g01990.1 0.002 1 0.998 0.002 1<br>Osind12g02780.1 0.002 1 0.998 0.002 1	1<br>Orufi11g02320.1 0.002 1 0.998 0.003 1<br>Orufi12g02480.1 0.002 1 0.998 0.002 1	1<br>Oglab11g02200.1 0.002 1 0.998 0.002 1<br>Oglab12g02130.1 0.002 1 0.998 0.002 1	1<br>Obart12g02080.1 0.022 1 0.978 0.002 1	0	0	0	0	0
	1<br>Osjap07g04360.1 0.031 0.64 0.609 0.018 0.925<br>Osjap12g12870.1 0.651 0.953 0.302 0.022 0.417	1<br>Osind11g02010.1 0.013 0.64 0.627 0.001 0.994<br>Osind12g10020.1 0.651 0.953 0.302 0.022 0.417	1<br>Orufi07g03650.1 0.031 0.64 0.609 0.019 0.976<br>Orufi12g10910.1 0.651 0.953 0.302 0.022 0.417	1<br>Oglab07g03810.1 0.031 0.64 0.609 0.019 0.987<br>Oglab12g09880.1 0.651 0.953 0.302 0.02 0.385	0<br>Obart11g02350.1 0.02 0.64 0.62 0.004 0.993	1<br>Olong_012584-R1 0.061 0.597 0.536 0.002 0.965<br>Olong_017316-R1 0.651 0.953 0.302 0.017 0.365	1<br>Opunc11g02190.1 0.002 0.64 0.638 0.003 0.989<br>Opunc12g08740.1 0.651 0.953 0.302 0.015 0.324	1<br>Obrac05g10860.1 0.047 0.64 0.593 0.097 0.987<br>Obrac12g07170.1 0.651 0.944 0.293 0.029 0.57	1<br>Lperr12g02190.1 0.002 0.64 0.638 0.003 0.989<br>Lperr12g08380.1 0.644 0.987 0.343 0.01 0.488	0<br>AT5G16080.1 0.047 0.644 0.597 0.081 0.994
Osind12g09530.1	1<br>Osjap12g12320.1 0.001 1 0.999 0.001 1	1<br>Osind12g09530.1 0.001 1 0.999 0.001 1	1<br>Orufi12g10460.1 0.001 1 0.999 0.001 1	1<br>Oglab12g09470.1 0.001 1 0.999 0.001 1	1<br>Obart12g09410.1 0.001 1 0.999 0.001 1	0	0	0	0	0
	1<br>Osjap01g44780.2 0.453 0.998 0.545 0.095 0.994<br>Osjap03g07600.1 0.001 0.292 0.291 0.004 0.996	1<br>Osind01g46040.1 0.453 0.998 0.545 0.151 0.995<br>Osind03g07670.1 0.001 0.292 0.291 0.004 0.996	1<br>Orufi01g42650.3 0.453 0.998 0.545 0.195 0.995<br>Orufi03g07180.1 0.001 0.292 0.291 0.004 0.996	0<br>Oglab01g34040.1 0.502 0.998 0.496 0.203 0.995<br>Oglab03g07280.1 0.001 0.292 0.291 0.004 0.996	1<br>Obart01g39260.3 0.469 0.998 0.529 0.041 0.994<br>Obart03g07210.1 0.001 0.292 0.291 0.004 0.996	1<br>Olong_021912-R1 0.47 1 0.53 0.099 1<br>Olong_021913-R1 0.001 0.291 0.29 0.004 0.996	1<br>Opunc12g08570.1 0.334 1 0.666 0.017 1<br>Opunc12g08580.1 0.001 0.291 0.29 0.004 0.996	1<br>Obrac03g05880.1 0.001 0.291 0.29 0.004 0.996<br>Obrac12g06500.1 0.475 1 0.525 0.103 1	1<br>Lperr01g33810.2 0.469 0.998 0.529 0.002 0.994<br>Lperr03g06600.1 0.001 0.291 0.29 0.004 0.996	0<br>AT4G34670.1 0.001 0.283 0.282 0.004 0.966<br>AT5G67540.2 0.502 0.993 0.491 0.053 1
Osind12g10670.1	1<br>Osjap01g31400.1 0.007 0.915 0.908 0.006 0.818<br>Osjap12g13600.1 0.007 0.993 0.986 0.007 0.993	1<br>Osind01g32860.1 0.007 0.915 0.908 0.007 0.854<br>Osind12g10660.1 0.007 0.993 0.986 0.007 0.993<br>Osind12g10670.1 0.007 1 0.993 0.007 1	1<br>Orufi01g30130.1 0.007 0.915 0.908 0.007 0.854	1<br>Oglab01g22960.1 0.071 0.915 0.844 0.194 0.874	1<br>Obart01g27080.1 0.071 0.915 0.844 0.194 0.874<br>Obart12g10510.1 0.007 0.993 0.986 0.012 0.988	0	1<br>Opunc10g03560.1 0.035 0.844 0.809 0.016 0.248	0	0	0
	1<br>Osjap01g05590.1 0.014 0.326 0.312 0.017 0.556<br>Osjap02g24490.1 0.369 0.908 0.539 0.591 0.779	1<br>Osind03g19770.1 0.007 0.411 0.404 0.002 0.412<br>Osind04g15200.1 0.44 0.972 0.532 0.042 0.368	0<br>Orufi12g11520.1 0.007 0.794 0.787 0.008 0.882	1<br>Oglab01g20040.1 0.035 0.34 0.305 0.022 0.11<br>Oglab06g05230.1 0.404 0.915 0.511 0.669 0.816	1<br>Obart01g16250.1 0.411 0.908 0.497 0.765 0.869<br>Obart03g17930.1 0.007 0.411 0.404 0.002 0.126	1<br>Olong_000447-R1 0.39 0.908 0.518 0.691 0.824<br>Olong_003316-R1 0.035 0.34 0.305 0.021 0.103	1<br>Opunc03g17210.1 0.007 0.333 0.326 0.002 0.095<br>Opunc06g05170.1 0.404 0.915 0.511 0.649 0.802	0<br>Obrac01g31700.1 0.184 0.908 0.724 0.829 0.924	1<br>Lperr03g05500.1 0.411 0.915 0.504 0.585 0.769<br>Lperr03g15640.1 0.007 0.333 0.326 0.002 0.09	1<br>AT2G36780.1 0.035 0.333 0.298 0.028 0.113<br>AT5G50380.1 0.355 0.908 0.553 0.706 0.82
Osind12g15880.1	1<br>Osjap12g19810.1 0.002 1 0.998 0.002 1	1<br>Osind12g15880.1 0.002 1 0.998 0.002 1	0	0	0	0	0	0	0	0

	jap	indica	rufi	glab	barthii	longi	punctata	brachy	leersia	tair
	1<br>Osjap07g08090.1 0.146 0.615 0.469 0.642 0.97<br>Osjap12g19770.1 0.659 1 0.341 0.006 1	0<br>Osind04g18390.2 0.13 0.643 0.513 0.648 0.994	1<br>Orufi12g17100.1 0.654 1 0.346 0.081 1<br>Orufi12g17130.1 0.178 0.654 0.476 0.004 0.991	1<br>Oglab06g04730.1 0.146 0.615 0.469 0.655 0.974<br>Oglab12g14370.2 0.654 1 0.346 0.126 1	1<br>Obart03g38270.2 0.146 0.615 0.469 0.61 0.968<br>Obart12g15400.1 0.659 1 0.341 0.006 1	1<br>Olong_001697-R1 0.659 1 0.341 0.006 1<br>Olong_021520-R2 0.139 0.609 0.47 0.684 0.963	1<br>Opunc03g35430.1 0.146 0.615 0.469 0.637 0.97<br>Opunc12g13920.1 0.63 1 0.37 0.003 1	1<br>Obrac06g03630.1 0.126 0.609 0.483 0.588 0.894<br>Obrac12g09830.1 0.652 1 0.348 0.003 1	0<br>Lperr12g12580.2 0.515 1 0.485 0.03 1	0<br>AT5G20250.4 0.146 0.615 0.469 0.681 0.972
Osind12g16420.1	0	1<br>Osind12g16420.1 0.002 1 0.998 0.002 1	0	0	0	0	0	0	0	0
	1<br>Osjap09g01510.1 0.002 0.212 0.21 0.397 0.804<br>Osjap12g20330.1 0.304 1 0.696 0.037 1	1<br>Osind02g10370.1 0.4 0.972 0.572 0.094 0.963<br>Osind06g09400.1 0.002 0.274 0.272 0.373 0.873	1<br>Orufi12g17680.1 0.002 0.265 0.263 0.005 1<br>Orufi12g17700.1 0.304 1 0.696 0.037 1	1<br>Oglab06g08180.1 0.002 0.274 0.272 0.373 0.873<br>Oglab12g14860.1 0.304 1 0.696 0.037 1	1<br>Obart09g01470.1 0.002 0.212 0.21 0.026 0.684<br>Obart12g15930.1 0.304 1 0.696 0.034 1	1<br>Olong_005120-R1 0.304 1 0.696 0.037 1<br>Olong_008551-R1 0.002 0.209 0.207 0.365 0.786	1<br>Opunc09g01310.1 0.002 0.212 0.21 0.379 0.821<br>Opunc12g14360.1 0.304 1 0.696 0.14 1	1<br>Obrac06g15120.1 0.002 0.252 0.25 0.308 0.869<br>Obrac12g10160.1 0.304 0.997 0.693 0.083 0.994	1<br>Lperr06g18350.1 0.002 0.252 0.25 0.391 0.883<br>Lperr12g13120.1 0.304 1 0.696 0.034 1	1<br>AT1G06990.1 0.005 0.265 0.26 0.386 0.908<br>AT1G09300.1 0.316 0.974 0.658 0.01 0.937

((((((_b1,_a2),longi),punctata),brachy),leersia),tair);

chimeric gene id	INGROUP			MIDGROUP				OUTGROUP		
	jap	indica	rufi	glab	barthii	longi	punctata	brachy	leersia	tair
Osind01g08490.1	1<br>Osjap01g08660.1 0.001 1 0.999 0.001 1	1<br>Osind01g08490.1 0.001 1 0.999 0.001 1	1<br>Orufi01g08070.1 0.001 1 0.999 0.001 1	1<br>Oglab01g06470.1 0.001 1 0.999 0.001 1	1<br>Obart01g07430.1 0.017 1 0.983 0.001 1	1<br>Olong_011567-R1 0.07 1 0.93 0.001 1	0	0	0	0
	1<br>Osjap01g29230.2 0.114 0.581 0.467 0.25 0.983<br>Osjap05g00560.2 0.647 0.997 0.35 0.146 1	1<br>Osind01g30690.1 0.114 0.581 0.467 0.267 0.982<br>Osind05g00540.1 0.647 0.997 0.35 0.126 1	1<br>Orufi01g27950.1 0.114 0.581 0.467 0.25 0.983<br>Orufi01g48560.2 0.647 0.98 0.333 0.085 0.984	1<br>Oglab01g21070.2 0.114 0.581 0.467 0.249 0.983<br>Oglab05g00530.1 0.647 0.997 0.35 0.146 1	1<br>Obart01g25090.1 0.114 0.581 0.467 0.219 0.982<br>Obart05g00540.1 0.647 0.997 0.35 0.146 1	0<br>Olong_003039-R1 0.647 0.997 0.35 0.128 1<br>Olong_003692-R2 0.153 0.581 0.428 0.244 0.979	1<br>Opunc01g07170.1 0.001 0.582 0.581 0.001 0.984<br>Opunc05g00510.2 0.647 0.997 0.35 0.15 1	1<br>Obrac01g05760.3 0.583 1 0.417 0.082 1<br>Obrac01g05770.3 0.001 0.582 0.581 0.001 0.985	1<br>Lperr01g07030.1 0.583 1 0.417 0.018 1<br>Lperr01g07040.2 0.001 0.582 0.581 0.001 0.989	1<br>AT1G54350.1 0.585 0.993 0.408 0.084 0.975<br>AT5G53430.1 0.024 0.581 0.557 0.09 0.984
Osind01g26900.1	1<br>Osjap01g25870.1 0.001 1 0.999 0.001 0.627	1<br>Osind01g26900.1 0.001 1 0.999 0.001 1	1<br>Orufi01g24680.1 0.006 1 0.994 0.207 1<br>Orufi01g24680.2 0.006 1 0.994 0.198 1	1<br>Oglab01g18120.1 0.006 0.994 0.988 0.149 0.728	0	0	0	0	0	0
	1<br>Osjap07g18880.1 0.033 0.582 0.549 0.391 0.604<br>Osjap11g19730.1 0.642 0.994 0.352 0.269 0.459	1<br>Osind01g26890.1 0.001 0.603 0.602 0.004 0.996<br>Osind11g14800.1 0.624 0.999 0.375 0.108 0.803	0<br>Orufi01g24710.1 0.233 0.517 0.284 0.005 1<br>Orufi11g18390.1 0.625 0.999 0.374 0.001 0.371	1<br>Oglab01g18150.1 0.001 0.517 0.516 0.003 1<br>Oglab11g15370.1 0.625 0.997 0.372 0.001 0.904	1<br>Obart01g21980.1 0.001 0.603 0.602 0.002 0.998<br>Obart11g17430.1 0.606 0.999 0.393 0.007 0.827	0<br>Olong_007897-R1 0.233 0.603 0.37 0.004 0.996<br>Olong_023940-R1 0.611 1 0.389 0.007 0.917	1<br>Opunc01g22160.1 0.001 0.592 0.591 0.002 0.973<br>Opunc10g14020.1 0.62 0.997 0.377 0.003 0.26	0<br>Obrac01g12530.1 0.056 0.481 0.425 0.016 0.79<br>Obrac10g00570.1 0.625 0.999 0.374 0.001 0.328	1<br>Lperr01g18740.1 0.004 0.603 0.599 0.004 0.998<br>Lperr01g18750.1 0.625 1 0.375 0.001 0.278	0<br>AT1G33590.1 0.625 0.996 0.371 0.002 0.551
Osind01g28070.1	0	1<br>Osind01g28070.1 0.002 1 0.998 0.002 1	1<br>Orufi01g25620.1 0.002 1 0.998 0.002 1	0	0	0	0	0	0	0
	1<br>Osjap01g26860.1 0.246 0.964 0.718 0.083 0.987<br>Osjap08g15630.1 0.002 0.246 0.244 0.007 0.912	1<br>Osind02g32380.1 0.002 0.246 0.244 0.009 1<br>Osind11g01570.1 0.246 0.893 0.647 0.489 0.955	1<br>Orufi12g02050.1 0.254 0.877 0.623 0.388 0.948<br>Orufi12g05600.1 0.002 0.246 0.244 0.459 0.725	1<br>Oglab11g01770.1 0.246 0.857 0.611 0.213 0.4<br>Oglab11g05250.1 0.005 0.246 0.241 0.43 0.731	1<br>Obart09g10970.1 0.002 0.202 0.2 0.007 0.743<br>Obart12g01860.1 0.232 0.973 0.741 0.088 0.703	1<br>Olong_007172-R1 0.005 0.246 0.241 0.341 0.684<br>Olong_015795-R1 0.246 0.877 0.631 0.506 0.958	1<br>Opunc09g15420.1 0.246 1 0.754 0.068 1<br>Opunc11g05440.1 0.005 0.246 0.241 0.431 0.728	1<br>Obrac09g10790.1 0.246 0.968 0.722 0.06 0.843<br>Obrac12g03740.1 0.005 0.246 0.241 0.35 0.667	1<br>Lperr09g13070.1 0.246 0.959 0.713 0.171 0.919<br>Lperr11g05150.1 0.005 0.246 0.241 0.444 0.715	1<br>AT1G77380.1 0.005 0.246 0.241 0.439 0.704<br>AT4G33495.1 0.246 0.914 0.668 0.103 0.988
Osind01g44140.1	1<br>Osjap01g42770.1 0.001 1 0.999 0.001 1	1<br>Osind01g44140.1 0.001 1 0.999 0.001 1	1<br>Orufi01g40790.1 0.001 1 0.999 0.001 1<br>Orufi01g40790.2 0.001 1 0.999 0.001 1<br>Orufi01g40790.3 0.001 1 0.999 0.001 1	0	0	1<br>Olong_024830-R1 0.001 1 0.999 0.001 1	0	0	0	0
	1<br>Osjap01g08100.1 0.013 0.278 0.265 0.133 0.963	1<br>Osind01g08020.1 0.013 0.278 0.265 0.133 0.963	1<br>Orufi02g20240.3 0.013 0.267 0.254 0.044 0.951	1<br>Oglab01g32220.1 0.001 0.266 0.265 0.004 0.934	1<br>Obart01g37410.1 0.001 0.266 0.265 0.004 0.934	1<br>Olong_010570-R1 0.412 0.995 0.583 0.115 0.915	1<br>Opunc01g36330.1 0.001 0.266 0.265 0.005 0.975	1<br>Obrac01g28830.1 0.001 0.264 0.263 0.004 0.978	1<br>Lperr01g32130.1 0.001 0.266 0.265 0.005 0.977	0<br>AT2G37970.1 0.001 0.266 0.265 0.049 0.978

	Osjap03g27560.1 0.412 0.995 0.583 0.127 0.95	Osind03g28340.1 0.412 0.995 0.583 0.127 0.95	Orufi03g26160.3 0.412 0.995 0.583 0.127 0.95	Oglab01g32230.1 0.363 0.999 0.636 0.002 0.955	Obart01g37420.1 0.363 1 0.637 0.002 1	Olong_011675-R1 0.013 0.266 0.253 0.517 0.972	Opunc01g36340.1 0.363 0.999 0.636 0.002 0.969	Obrac01g28840.1 0.365 1 0.635 0.002 1	Lperr01g32140.1 0.363 1 0.637 0.002 1	AT3G54090.1 0.475 1 0.525 0.123 1
Osind01g52750.1	1<br>Osjap01g51220.1 0.002 1 0.998 0.071 1	1<br>Osind01g52750.1 0.002 1 0.998 0.002 1	1<br>Orufi01g48620.1 0.043 1 0.957 0.124 1	0	0	0	0	0	0	0
	1<br>Osjap02g42170.1 0.002 0.698 0.696 0.362 0.927<br>Osjap05g12720.1 0.797 1 0.203 0.16 1	1<br>Osind02g41730.1 0.002 0.698 0.696 0.362 0.927<br>Osind05g13530.1 0.797 1 0.203 0.16 1	1<br>Orufi02g39420.1 0.002 0.698 0.696 0.362 0.927<br>Orufi05g12160.1 0.797 1 0.203 0.16 1	1<br>Oglab02g35020.1 0.002 0.698 0.696 0.352 0.901<br>Oglab05g10250.1 0.797 1 0.203 0.16 1	1<br>Obart02g37850.1 0.002 0.698 0.696 0.33 0.933<br>Obart05g11250.1 0.797 1 0.203 0.16 1	1<br>Olong_006258-R1 0.002 0.698 0.696 0.246 0.913<br>Olong_022231-R1 0.797 1 0.203 0.16 1	1<br>Opunc02g35080.1 0.002 0.698 0.696 0.366 0.926<br>Opunc05g09730.1 0.797 1 0.203 0.594 1	1<br>Obrac01g35490.1 0.797 1 0.203 0.395 1<br>Obrac02g28040.1 0.002 0.698 0.696 0.366 0.928	0<br>Lperr01g39540.1 0.797 1 0.203 0.152 1<br>Lperr02g31330.1 0.005 0.538 0.533 0.364 0.762	0<br>AT5G24900.1 0.029 0.534 0.505 0.408 0.785
Osind02g26640.2	1<br>Osjap02g26750.1 0.001 0.953 0.952 0.001 0.993<br>Osjap02g26750.2 0.001 0.953 0.952 0.001 0.993<br>Osjap02g26750.4 0.001 0.953 0.952 0.001 0.799	1<br>Osind02g26640.2 0.001 1 0.999 0.001 1	1<br>Orufi02g24900.1 0.01 0.958 0.948 0.06 0.859<br>Orufi02g24900.3 0.001 0.958 0.957 0.001 0.74<br>Orufi02g24900.4 0.001 0.958 0.957 0.001 0.737	1<br>Oglab02g22130.2 0.001 1 0.999 0.001 1	1<br>Obart02g23460.2 0.001 1 0.999 0.001 1	0	1<br>Opunc02g21410.1 0.01 0.975 0.965 0.116 0.996<br>Opunc02g21410.2 0.01 0.975 0.965 0.114 0.996<br>Opunc02g21410.3 0.01 0.975 0.965 0.115 0.996<br>Opunc02g21410.4 0.01 0.975 0.965 0.114 0.996	0	0	0
	1<br>Osjap02g26590.1 0.497 0.986 0.489 0.033 0.996<br>Osjap02g26750.5 0.01 0.413 0.403 0.238 0.968	1<br>Osind02g26450.1 0.497 0.986 0.489 0.023 0.992<br>Osind02g26620.1 0.01 0.413 0.403 0.495 0.978	1<br>Orufi02g24730.1 0.497 0.986 0.489 0.031 0.996<br>Orufi02g24880.2 0.01 0.413 0.403 0.18 0.964	1<br>Oglab02g21930.1 0.497 1 0.503 0.047 1<br>Oglab02g22110.2 0.01 0.413 0.403 0.532 0.976	1<br>Obart02g23290.1 0.497 1 0.503 0.029 0.997<br>Obart02g23440.1 0.01 0.413 0.403 0.492 0.978	0<br>Olong_012377-R1 0.651 0.957 0.306 0.444 0.915<br>Olong_024345-R1 0.01 0.434 0.424 0.442 0.991	1<br>Opunc02g21230.1 0.504 0.96 0.456 0.003 0.934<br>Opunc02g21240.1 0.01 0.422 0.412 0.121 0.972	1<br>Obrac02g16670.1 0.503 0.957 0.454 0.005 0.995<br>Obrac02g16700.1 0.01 0.422 0.412 0.114 0.947	1<br>Lperr02g18740.1 0.512 0.975 0.463 0.059 0.982<br>Lperr02g19120.1 0.01 0.443 0.433 0.183 0.978	0<br>AT1G47230.2 0.577 0.953 0.376 0.095 0.857<br>AT4G31860.1 0.013 0.411 0.398 0.123 0.989
Osind02g39460.2	0	1<br>Osind02g39460.1 0.001 1 0.999 0.001 1<br>Osind02g39460.2 0.001 1 0.999 0.001 1	1<br>Orufi02g37290.1 0.028 0.883 0.855 0.031 1	0	0	0	0	0	0	0
	1<br>Osjap01g00320.1 0.613 0.883 0.27 0.256 1<br>Osjap02g39920.1 0.001 0.572 0.571 0.001 0.971	1<br>Osind01g00350.1 0.613 0.883 0.27 0.037 1<br>Osind09g08880.1 0.02 0.567 0.547 0.201 0.99	1<br>Orufi01g00280.2 0.613 0.883 0.27 0.039 1<br>Orufi02g37310.1 0.001 0.572 0.571 0.002 0.995	1<br>Oglab02g32990.1 0.612 1 0.388 0.217 1<br>Oglab02g33000.1 0.001 0.606 0.605 0.001 0.986	1<br>Obart02g35820.1 0.612 1 0.388 0.223 1<br>Obart02g35830.2 0.001 0.606 0.605 0.001 0.985	1<br>Olong_003837-R1 0.614 0.883 0.269 0.003 1<br>Olong_003838-R2 0.001 0.572 0.571 0.014 1	1<br>Opunc01g00290.1 0.614 0.883 0.269 0.003 1<br>Opunc02g33020.1 0.013 0.595 0.582 0.032 0.997	1<br>Obrac01g00240.4 0.609 0.883 0.274 0.056 1<br>Obrac02g26250.1 0.015 0.573 0.558 0.059 0.992	1<br>Lperr01g00240.1 0.574 0.883 0.309 0.039 1<br>Lperr02g29510.1 0.001 0.572 0.571 0.034 1	1<br>AT3G54360.1 0.614 0.887 0.273 0.064 1<br>AT4G23370.1 0.006 0.567 0.561 0.179 0.996
Osind03g00740.1	1<br>Osjap03g00680.1 0.001 0.996 0.995 0.001 0.994<br>Osjap03g00680.2 0.001 0.996 0.995 0.001 0.994<br>Osjap03g00680.3 0.001 0.996 0.995 0.001 0.994	1<br>Osind03g00740.1 0.001 1 0.999 0.001 1	1<br>Orufi03g12660.1 0.001 0.996 0.995 0.001 0.994<br>Orufi03g12660.2 0.001 0.996 0.995 0.001 0.994	1<br>Oglab03g00390.1 0.001 0.996 0.995 0.001 0.994	1<br>Obart03g00550.1 0.001 1 0.999 0.001 1	0	0	0	0	0
	1<br>Osjap02g42090.1 0.052 0.678 0.626 0.065 1<br>Osjap12g08580.1 0.708 0.996 0.288 0.011 0.977	1<br>Osind02g41670.1 0.062 0.678 0.616 0.131 1<br>Osind03g02220.1 0.698 0.996 0.298 0.015 0.982	1<br>Orufi02g39350.1 0.062 0.678 0.616 0.131 1<br>Orufi09g03230.3 0.732 0.99 0.258 0.053 0.963	1<br>Oglab02g34920.1 0.062 0.678 0.616 0.131 1<br>Oglab03g02050.1 0.698 0.996 0.298 0.214 0.986	1<br>Obart02g37790.1 0.062 0.648 0.586 0.133 0.977<br>Obart12g06510.1 0.676 0.996 0.32 0.052 0.984	0<br>Olong_013175-R1 0.271 0.904 0.633 0.002 0.961	1<br>Opunc03g00470.1 0.001 0.78 0.779 0.002 1<br>Opunc11g04590.1 0.78 0.996 0.216 0.055 0.982	0<br>Obrac02g27970.1 0.137 0.678 0.541 0.105 1<br>Obrac05g17660.1 0.743 0.995 0.252 0.425 0.997	1<br>Lperr03g00570.1 0.001 0.678 0.677 0.002 1<br>Lperr04g11360.1 0.747 0.996 0.249 0.415 0.99	0<br>AT5G04460.1 0.018 0.678 0.66 0.081 1
Osind03g12930.1	1<br>Osjap03g13100.1 0.028 1 0.972 0.001 1	1<br>Osind03g12930.1 0.001 1 0.999 0.001 1	0	1<br>Oglab03g12120.1 0.001 1 0.999 0.001 1	1<br>Obart03g12120.1 0.001 1 0.999 0.001 1	0	0	0	0	0
	1<br>Osjap04g18730.1 0.64 0.998 0.358 0.015 0.863<br>Osjap12g01140.1 0.109 0.633 0.524 0.498 0.981	1<br>Osind06g01770.1 0.642 0.998 0.356 0.212 0.912<br>Osind12g01290.1 0.109 0.633 0.524 0.49 0.981	1<br>Orufi06g01460.3 0.642 0.998 0.356 0.212 0.912<br>Orufi12g01130.1 0.109 0.633 0.524 0.498 0.981	1<br>Oglab06g01620.1 0.642 0.998 0.356 0.233 0.912<br>Oglab12g00800.1 0.114 0.633 0.519 0.483 0.983	1<br>Obart06g01490.1 0.642 0.998 0.356 0.233 0.912<br>Obart11g01060.1 0.114 0.633 0.519 0.471 0.982	1<br>Olong_007714-R9 0.642 1 0.358 0.002 1<br>Olong_007715-R1 0.125 0.633 0.508 0.007 0.965	1<br>Opunc01g28650.1 0.646 0.998 0.352 0.094 0.923<br>Opunc03g11680.1 0.028 0.633 0.605 0.001 0.971	1<br>Obrac03g09720.1 0.025 0.633 0.608 0.036 0.971<br>Obrac04g09430.1 0.64 0.998 0.358 0.014 0.863	1<br>Lperr03g10720.1 0.028 0.633 0.605 0.001 0.934<br>Lperr03g17470.1 0.633 0.998 0.365 0.119 0.828	1<br>AT3G46740.1 0.122 0.633 0.511 0.158 0.971<br>AT3G46790.1 0.648 0.998 0.35 0.068 0.807
Osind03g31500.1	1	1	1	0	1	0	0	0	0	0

	Osjap03g30460.1 0.025 1 0.975 0.022 1	Osind03g31500.1 0.001 1 0.999 0.001 1	Orufi03g29110.1 0.003 1 0.997 0.276 1		Obart03g27980.1 0.089 1 0.911 0.016 1<br>Obart03g27980.2 0.089 1 0.911 0.015 1					
	1<br>Osjap03g30380.1 0.557 0.999 0.442 0.059 0.866<br>Osjap12g24400.2 0.092 0.528 0.436 0.012 0.988	1<br>Osind08g14630.1 0.557 0.999 0.442 0.069 0.96<br>Osind12g20440.1 0.092 0.528 0.436 0.01 0.99	1<br>Orufi03g29970.1 0.557 0.999 0.442 0.035 0.998<br>Orufi12g21550.1 0.092 0.528 0.436 0.01 0.99	1<br>Oglab03g27330.1 0.557 1 0.443 0.036 1<br>Oglab12g17460.4 0.092 0.528 0.436 0.012 0.996	1<br>Obart03g27900.1 0.557 0.999 0.442 0.053 0.865<br>Obart12g19300.1 0.092 0.528 0.436 0.01 0.99	1<br>Olong_002926-R1 0.097 0.529 0.432 0.002 0.996<br>Olong_002936-R1 0.557 0.999 0.442 0.059 0.866	1<br>Opunc03g25470.1 0.01 0.529 0.519 0.001 0.997<br>Opunc06g15370.1 0.557 0.999 0.442 0.035 0.871	0<br>Obrac03g20710.1 0.094 0.529 0.435 0.028 0.997	1<br>Lperr03g23190.1 0.557 0.999 0.442 0.03 0.867<br>Lperr03g23200.1 0.079 0.529 0.45 0.002 0.997	0<br>AT5G36940.1 0.093 0.531 0.438 0.003 0.998
Osind03g34150.1	1<br>Osjap03g33110.1 0.003 1 0.997 0.003 1	1<br>Osind03g34150.1 0.003 1 0.997 0.003 1	1<br>Orufi03g31780.1 0.003 1 0.997 0.003 1	1<br>Oglab03g29380.1 0.003 0.987 0.984 0.002 0.888	1<br>Obart03g30540.1 0.003 0.987 0.984 0.003 0.954	0	0	0	0	0
	1<br>Osjap03g34570.1 0.007 0.441 0.434 0.083 0.217<br>Osjap05g02750.1 0.474 0.899 0.425 0.399 0.778	1<br>Osind05g02740.1 0.474 0.899 0.425 0.399 0.778<br>Osind10g10410.1 0.007 0.444 0.437 0.286 0.81	0<br>Orufi05g02620.1 0.435 0.899 0.464 0.348 0.77<br>Orufi10g11920.1 0.007 0.255 0.248 0.424 0.927	0<br>Oglab01g05160.1 0.569 0.895 0.326 0.472 0.764<br>Oglab11g10860.1 0.007 0.454 0.447 0.051 0.155	1<br>Obart05g02540.1 0.474 0.922 0.448 0.344 0.808<br>Obart10g08010.1 0.01 0.444 0.434 0.07 0.19	1<br>Olong_003965-R1 0.01 0.526 0.516 0.054 0.186<br>Olong_027259-R1 0.588 0.892 0.304 0.303 0.684	1<br>Opunc01g05410.1 0.474 0.899 0.425 0.138 0.664<br>Opunc11g10570.1 0.007 0.454 0.447 0.066 0.214	1<br>Obrac01g04280.1 0.474 0.895 0.421 0.415 0.77<br>Obrac04g21450.1 0.007 0.441 0.434 0.038 0.183	1<br>Lperr03g25260.1 0.003 0.444 0.441 0.173 0.514<br>Lperr06g16660.1 0.497 0.899 0.402 0.431 0.779	1<br>AT2G01820.1 0.01 0.542 0.532 0.07 0.239<br>AT3G62160.1 0.605 0.892 0.287 0.507 0.764
Osind03g35550.1	1<br>Osjap03g34330.1 0.039 1 0.961 0.001 1	1<br>Osind03g35550.1 0.001 1 0.999 0.001 1	1<br>Orufi03g33060.1 0.039 1 0.961 0.001 1	1<br>Oglab03g30830.1 0.039 1 0.961 0.001 1	1<br>Obart03g31750.1 0.039 1 0.961 0.046 1	0	0	0	0	0
	1<br>Osjap03g39110.1 0.646 1 0.354 0.036 1<br>Osjap10g05370.1 0.026 0.622 0.596 0.051 0.963	1<br>Osind01g46540.1 0.646 0.999 0.353 0.065 0.994<br>Osind10g03770.1 0.026 0.622 0.596 0.057 0.96	1<br>Orufi10g04850.1 0.026 0.622 0.596 0.054 0.963<br>Orufi11g12520.1 0.647 1 0.353 0.038 0.991	1<br>Oglab03g34460.1 0.622 0.997 0.375 0.016 0.993<br>Oglab10g04340.1 0.02 0.622 0.602 0.058 0.963	1<br>Obart03g36200.1 0.646 1 0.354 0.016 0.997<br>Obart10g04600.1 0.051 0.622 0.571 0.106 0.962	1<br>Olong_014796-R1 0.039 0.532 0.493 0.002 0.95<br>Olong_027495-R1 0.622 0.995 0.373 0.005 0.998	1<br>Opunc01g42030.1 0.646 1 0.354 0.28 0.872<br>Opunc10g04080.2 0.033 0.622 0.589 0.084 0.959	1<br>Obrac02g16280.1 0.64 0.981 0.341 0.063 0.949<br>Obrac03g23440.1 0.039 0.622 0.583 0.002 0.964	1<br>Lperr03g30520.1 0.638 1 0.362 0.003 0.997<br>Lperr10g03360.1 0.004 0.622 0.618 0.009 0.962	0<br>AT1G70730.3 0.039 0.622 0.583 0.118 0.967
Osind03g45590.1	1<br>Osjap03g44530.1 0.002 1 0.998 0.001 1	1<br>Osind03g45590.1 0.002 1 0.998 0.002 1	0	0	0	1<br>Olong_013357-R1 0.002 1 0.998 0.002 1	0	1<br>Obrac03g30850.1 0.008 1 0.992 0.023 1	0	0
	1<br>Osjap03g41540.2 0.589 0.998 0.409 0.064 0.993<br>Osjap07g07980.2 0.005 0.556 0.551 0.225 0.99	1<br>Osind03g42620.1 0.589 0.998 0.409 0.054 0.993<br>Osind07g07170.1 0.002 0.556 0.554 0.002 0.987	1<br>Orufi03g42810.1 0.577 1 0.423 0.004 1<br>Orufi07g06860.4 0.002 0.556 0.554 0.002 0.987	1<br>Oglab03g38980.1 0.569 1 0.431 0.004 1<br>Oglab03g38990.1 0.002 0.542 0.54 0.002 0.968	1<br>Obart03g41020.1 0.569 1 0.431 0.004 1<br>Obart03g41030.1 0.002 0.542 0.54 0.002 0.967	1<br>Olong_020188-R1 0.002 0.556 0.554 0.002 0.987<br>Olong_021244-R1 0.563 0.992 0.429 0.283 0.929	1<br>Opunc03g38110.1 0.581 1 0.419 0.007 1<br>Opunc07g06610.1 0.002 0.561 0.559 0.002 0.993	1<br>Obrac03g30850.2 0.008 0.542 0.534 0.037 0.969<br>Obrac04g17280.1 0.569 0.992 0.423 0.003 0.906	1<br>Lperr03g34950.2 0.577 1 0.423 0.129 0.987<br>Lperr07g06010.1 0.002 0.561 0.559 0.002 0.991	1<br>AT3G01980.3 0.589 1 0.411 0.017 1<br>AT5G46410.2 0.145 0.539 0.394 0.206 0.991
Osind04g18090.1	1<br>Osjap04g19350.1 0.001 1 0.999 0.001 1	1<br>Osind04g18090.1 0.001 1 0.999 0.001 1	1<br>Orufi04g17470.1 0.03 1 0.97 0.001 1	0	0	1<br>Olong_005904-R1 0.032 1 0.968 0.021 1	0	0	1<br>Lperr04g12800.1 0.004 0.999 0.995 0.032 0.999	0
	1<br>Osjap02g23820.1 0.486 0.982 0.496 0.12 0.898<br>Osjap02g25840.1 0.034 0.4 0.366 0.013 0.995	1<br>Osind02g23720.1 0.486 0.982 0.496 0.123 0.899<br>Osind02g25560.1 0.034 0.4 0.366 0.013 0.995	1<br>Orufi02g23890.1 0.034 0.4 0.366 0.013 0.995<br>Orufi06g19770.1 0.487 1 0.513 0.115 0.977	1<br>Oglab04g14400.1 0.45 1 0.55 0.002 1<br>Oglab04g14410.1 0.027 0.404 0.377 0.064 1	1<br>Obart04g16100.1 0.45 1 0.55 0.002 1<br>Obart04g16110.1 0.027 0.404 0.377 0.064 1	1<br>Olong_013421-R1 0.507 1 0.493 0.002 0.946<br>Olong_021848-R1 0.034 0.4 0.366 0.013 0.995	1<br>Opunc04g13970.1 0.45 0.992 0.542 0.002 0.985<br>Opunc04g13980.1 0.03 0.404 0.374 0.003 1	1<br>Obrac04g09860.1 0.032 0.404 0.372 0.008 1<br>Obrac07g00130.3 0.452 0.983 0.531 0.001 0.49	1<br>Lperr02g18380.1 0.034 0.4 0.366 0.012 0.995<br>Lperr07g00080.1 0.451 0.999 0.548 0.004 0.892	0<br>AT5G01110.1 0.405 0.992 0.587 0.111 0.996
Osind05g00510.1	1<br>Osjap05g00530.1 0.001 1 0.999 0.131 1<br>Osjap05g00530.2 0.001 1 0.999 0.16 1<br>Osjap05g00530.3 0.001 1 0.999 0.161 1<br>Osjap05g00530.4 0.001 1 0.999 0.142 1	1<br>Osind05g00510.1 0.001 1 0.999 0.001 1	1<br>Orufi05g00500.1 0.001 1 0.999 0.131 1	0	1<br>Obart05g00520.1 0.001 0.999 0.998 0.049 0.993<br>Obart05g00520.2 0.001 1 0.999 0.049 1	1<br>Olong_022405-R1 0.001 1 0.999 0.001 1	0	0	0	0
	0<br>Osjap02g42390.1 0.326 0.993 0.667 0.001 0.983	1<br>Osind01g11620.1 0.002 0.275 0.273 0.271 0.988<br>Osind09g15830.1 0.31 1 0.69 0.029 1	1<br>Orufi07g24760.1 0.001 0.258 0.257 0.267 0.834<br>Orufi09g16480.1 0.31 1 0.69 0.029 1	1<br>Oglab05g00500.1 0.001 0.26 0.259 0.344 0.982<br>Oglab09g13820.1 0.291 1 0.709 0.064 1	1<br>Obart01g04950.1 0.001 0.258 0.257 0.193 0.858<br>Obart07g24200.1 0.302 0.998 0.696 0.005 0.907	1<br>Olong_004108-R1 0.004 0.275 0.271 0.098 0.928<br>Olong_023813-R1 0.303 0.997 0.694 0.001 0.979	1<br>Opunc05g00480.1 0.298 0.999 0.701 0.007 0.998<br>Opunc05g00490.1 0.001 0.26 0.259 0.352 0.982	1<br>Obrac02g14170.4 0.004 0.267 0.263 0.164 0.843<br>Obrac03g06070.3 0.31 0.998 0.688 0.023 0.996	1<br>Lperr02g16220.1 0.004 0.267 0.263 0.238 0.857<br>Lperr05g00500.1 0.301 1 0.699 0.009 1	0<br>AT4G14820.1 0.001 0.267 0.266 0.107 0.878

Osind05g02510.1	1<br>Osjap05g02530.1 0.001 0.987 0.986 0.001 0.998	1<br>Osind05g02510.1 0.001 1 0.999 0.001 1	0	1<br>Oglab05g02190.1 0.002 0.987 0.985 0.079 0.998	1<br>Obart05g02340.1 0.002 0.987 0.985 0.076 0.998	0	0	0	0	0
	0<br>Osjap03g00960.1 0.533 0.984 0.451 0.327 0.996<br>Osjap08g22320.1 0.005 0.352 0.347 0.174 0.689	1<br>Osind07g26860.1 0.391 0.987 0.596 0.035 1<br>Osind12g11780.1 0.001 0.35 0.349 0.224 0.839	1<br>Orufi05g02460.2 0.349 0.988 0.639 0.003 0.979<br>Orufi08g23390.1 0.005 0.349 0.344 0.011 0.994	0<br>Oglab03g00660.1 0.533 0.984 0.451 0.321 0.996<br>Oglab07g20370.2 0.005 0.352 0.347 0.007 0.626	1<br>Obart08g20910.1 0.005 0.352 0.347 0.006 0.539<br>Obart11g00590.1 0.408 0.963 0.555 0.146 0.917	1<br>Olong_014752-R1 0.008 0.346 0.338 0.016 0.959<br>Olong_026687-R1 0.348 0.988 0.64 0.088 0.98	1<br>Opunc02g12280.1 0.001 0.349 0.348 0.004 0.985<br>Opunc05g02220.2 0.349 0.987 0.638 0.003 0.977	0<br>Obrac05g01840.1 0.348 0.987 0.639 0.168 1	1<br>Lperr03g00840.1 0.346 0.987 0.641 0.107 0.719<br>Lperr11g18770.2 0.006 0.296 0.29 0.428 0.781	0<br>AT5G15380.1 0.369 0.981 0.612 0.066 0.987
Osind05g04070.1	1<br>Osjap05g04120.1 0.001 1 0.999 0.008 1<br>Osjap05g04120.2 0.001 1 0.999 0.008 1	1<br>Osind05g04070.1 0.001 1 0.999 0.001 1<br>Osind05g04070.2 0.001 1 0.999 0.001 1	1<br>Orufi05g03950.1 0.001 1 0.999 0.008 1<br>Orufi05g03950.2 0.001 1 0.999 0.008 1	1<br>Oglab05g03490.1 0.001 1 0.999 0.008 1<br>Oglab05g03490.2 0.001 1 0.999 0.008 1	1<br>Obart05g03830.1 0.001 1 0.999 0.008 1<br>Obart05g03830.2 0.001 1 0.999 0.008 1	1<br>Olong_016739-R1 0.069 1 0.931 0.001 1	1<br>Opunc05g03540.1 0.001 1 0.999 0.008 1<br>Opunc05g03540.2 0.001 1 0.999 0.008 1	1<br>Obrac05g02930.1 0.069 1 0.931 0.001 1	0	0
	1<br>Osjap01g27410.1 0.086 0.735 0.649 0.066 0.992<br>Osjap03g15140.2 0.738 1 0.262 0.01 0.192	1<br>Osind01g28630.1 0.086 0.735 0.649 0.066 0.992<br>Osind03g15230.1 0.783 1 0.217 0.03 1	1<br>Orufi01g26130.1 0.086 0.735 0.649 0.066 0.992<br>Orufi03g14720.1 0.783 1 0.217 0.03 1	1<br>Oglab01g19490.1 0.086 0.735 0.649 0.066 0.992<br>Oglab03g14060.1 0.783 1 0.217 0.03 1	1<br>Obart01g23310.1 0.086 0.735 0.649 0.066 0.992<br>Obart03g14050.1 0.783 1 0.217 0.03 1	1<br>Olong_003347-R2 0.086 0.735 0.649 0.047 0.992<br>Olong_009943-R1 0.783 1 0.217 0.03 1	1<br>Opunc01g23460.1 0.086 0.735 0.649 0.064 0.991<br>Opunc03g13590.1 0.783 1 0.217 0.002 0.545	1<br>Obrac01g17590.1 0.086 0.735 0.649 0.067 0.991<br>Obrac03g11330.1 0.783 1 0.217 0.03 1	0<br>Lperr05g03460.1 0.001 0.736 0.735 0.011 0.994	1<br>AT2G47580.1 0.768 1 0.232 0.008 1<br>AT3G16630.1 0.001 0.736 0.735 0.039 0.994
Osind05g04070.2	1<br>Osjap05g04120.1 0.001 1 0.999 0.008 1<br>Osjap05g04120.2 0.001 1 0.999 0.008 1	1<br>Osind05g04070.1 0.001 1 0.999 0.001 1<br>Osind05g04070.2 0.001 1 0.999 0.001 1	1<br>Orufi05g03950.1 0.001 1 0.999 0.008 1<br>Orufi05g03950.2 0.001 1 0.999 0.008 1	1<br>Oglab05g03490.1 0.001 1 0.999 0.008 1<br>Oglab05g03490.2 0.001 1 0.999 0.008 1	1<br>Obart05g03830.1 0.001 1 0.999 0.008 1<br>Obart05g03830.2 0.001 1 0.999 0.008 1	1<br>Olong_016739-R1 0.068 1 0.932 0.001 1	1<br>Opunc05g03540.1 0.001 1 0.999 0.008 1<br>Opunc05g03540.2 0.001 1 0.999 0.008 1	1<br>Obrac05g02930.1 0.068 1 0.932 0.001 1	0	0
	1<br>Osjap01g27410.1 0.084 0.738 0.654 0.066 0.992<br>Osjap03g15140.2 0.742 1 0.258 0.01 0.192	1<br>Osind01g28630.1 0.084 0.738 0.654 0.066 0.992<br>Osind03g15230.1 0.786 1 0.214 0.03 1	1<br>Orufi01g26130.1 0.084 0.738 0.654 0.066 0.992<br>Orufi03g14720.1 0.786 1 0.214 0.03 1	1<br>Oglab01g19490.1 0.084 0.738 0.654 0.066 0.992<br>Oglab03g14060.1 0.786 1 0.214 0.03 1	1<br>Obart01g23310.1 0.084 0.738 0.654 0.066 0.992<br>Obart03g14050.1 0.786 1 0.214 0.03 1	1<br>Olong_003347-R2 0.084 0.738 0.654 0.047 0.992<br>Olong_009943-R1 0.786 1 0.214 0.03 1	1<br>Opunc01g23460.1 0.084 0.738 0.654 0.064 0.991<br>Opunc03g13590.1 0.786 1 0.214 0.002 0.545	1<br>Obrac01g17590.1 0.084 0.738 0.654 0.067 0.991<br>Obrac03g11330.1 0.786 1 0.214 0.03 1	0<br>Lperr05g03460.1 0.001 0.74 0.739 0.011 0.994	1<br>AT2G47580.1 0.771 1 0.229 0.008 1<br>AT3G16630.1 0.001 0.74 0.739 0.039 0.994
Osind05g12550.1	1<br>Osjap05g11740.1 0.005 1 0.995 0.388 1<br>Osjap05g11740.2 0.005 1 0.995 0.432 1	1<br>Osind05g12550.1 0.005 1 0.995 0.005 1	1<br>Orufi05g11190.1 0.005 1 0.995 0.005 1	1<br>Oglab05g09590.1 0.005 1 0.995 0.005 1<br>Oglab05g09590.2 0.005 1 0.995 0.005 1	0	0	0	0	0	0
	0<br>Osjap11g19150.1 0.005 0.532 0.527 0.37 0.489	1<br>Osind08g13680.1 0.537 0.886 0.349 0.748 0.825<br>Osind11g14080.1 0.005 0.532 0.527 0.003 0.309	1<br>Orufi08g12780.1 0.537 0.886 0.349 0.769 0.839<br>Orufi11g17770.2 0.005 0.532 0.527 0.003 0.356	0<br>Oglab11g01510.4 0.507 0.886 0.379 0.681 0.789	1<br>Obart09g04270.2 0.537 0.92 0.383 0.847 0.93<br>Obart11g16680.1 0.065 0.532 0.467 0.051 0.311	1<br>Olong_007188-R1 0.537 0.886 0.349 0.744 0.822<br>Olong_026954-R1 0.045 0.532 0.487 0.016 0.497	1<br>Opunc08g10590.1 0.537 0.886 0.349 0.768 0.839<br>Opunc11g13660.1 0.065 0.532 0.467 0.051 0.311	0<br>Obrac12g01300.1 0.507 0.886 0.379 0.428 0.614	1<br>Lperr08g09600.2 0.537 0.886 0.349 0.759 0.833<br>Lperr11g13130.1 0.005 0.532 0.527 0.003 0.303	1<br>AT3G47630.2 0.005 0.532 0.527 0.003 0.298<br>AT4G19710.2 0.537 0.886 0.349 0.767 0.838
Osind05g28520.1	1<br>Osjap05g28230.1 0.001 1 0.999 0.001 1	1<br>Osind05g28520.1 0.001 1 0.999 0.001 1	1<br>Orufi05g26870.1 0.001 1 0.999 0.001 1	1<br>Oglab05g23160.1 0.001 1 0.999 0.001 1	1<br>Obart05g25070.1 0.001 1 0.999 0.001 1	0	1<br>Opunc05g22640.1 0.001 1 0.999 0.001 1	0	1<br>Lperr01g22990.1 0.001 1 0.999 0.027 1	0
	1<br>Osjap01g31140.1 0.006 0.43 0.424 0.093 0.995<br>Osjap01g31150.1 0.486 0.999 0.513 0.009 0.999	1<br>Osind01g32600.1 0.001 0.43 0.429 0.002 0.995<br>Osind01g32610.1 0.486 0.999 0.513 0.009 0.999	1<br>Orufi01g29880.1 0.001 0.43 0.429 0.075 0.995<br>Orufi01g29890.1 0.486 0.999 0.513 0.009 0.999	1<br>Oglab01g22740.1 0.091 0.431 0.34 0.213 0.996<br>Oglab01g22750.1 0.486 0.999 0.513 0.009 0.999	1<br>Obart01g26870.1 0.005 0.43 0.425 0.117 0.995<br>Obart01g26880.1 0.486 0.999 0.513 0.009 0.999	1<br>Olong_000367-R1 0.1 0.431 0.331 0.002 0.995<br>Olong_000368-R1 0.461 0.999 0.538 0.069 0.999	1<br>Opunc01g26820.1 0.091 0.431 0.34 0.118 0.996<br>Opunc01g26830.1 0.486 1 0.514 0.009 1	1<br>Obrac01g20400.1 0.091 0.431 0.34 0.125 0.996<br>Obrac05g17560.2 0.431 1 0.569 0.109 1	1<br>Lperr05g20850.2 0.491 1 0.509 0.002 1<br>Lperr05g20860.1 0.091 0.431 0.34 0.215 0.996	1<br>AT5G08680.1 0.022 0.43 0.408 0.064 0.995<br>AT5G51600.1 0.486 0.999 0.513 0.008 0.996
Osind06g23650.1	0	1<br>Osind06g23650.1 0.001 1 0.999 0.001 1	1<br>Orufi06g22110.1 0.001 1 0.999 0.011 1	0	1<br>Obart06g20680.1 0.001 1 0.999 0.001 1	0	0	0	0	0
	1<br>Osjap06g24310.1 0.001 0.693 0.692 0.059 0.986<br>Osjap06g24320.3 0.713 1 0.287 0.019 1	1<br>Osind02g08380.1 0.714 0.999 0.285 0.031 0.997<br>Osind09g17960.1 0.005 0.693 0.688 0.008 0.978	1<br>Orufi02g07600.1 0.714 0.999 0.285 0.031 0.997<br>Orufi09g15100.1 0.01 0.693 0.683 0.029 0.975	1<br>Oglab06g18700.1 0.001 0.693 0.692 0.016 0.986<br>Oglab06g18710.1 0.713 1 0.287 0.019 1	1<br>Obart02g07500.3 0.714 0.999 0.285 0.031 0.997<br>Obart04g22320.1 0.005 0.691 0.686 0.017 0.982	1<br>Olong_021346-R1 0.001 0.693 0.692 0.01 0.982<br>Olong_021347-R1 0.715 1 0.285 0.003 1	1<br>Opunc01g29350.1 0.001 0.691 0.69 0.004 0.986<br>Opunc06g18860.1 0.713 1 0.287 0.009 0.497	1<br>Obrac04g14990.1 0.005 0.691 0.686 0.017 0.982<br>Obrac06g14090.3 0.712 1 0.288 0.338 1	1<br>Lperr01g25410.2 0.001 0.691 0.69 0.009 0.986<br>Lperr02g06450.6 0.714 0.999 0.285 0.032 0.997	1<br>AT4G21160.1 0.716 1 0.284 0.047 1<br>AT5G51750.1 0.005 0.693 0.688 0.021 0.985

Osind06g27610.1	1<br>Osjap06g28190.1 0.003 0.841 0.838 0.002 0.813<br>Osjap06g28190.2 0.003 0.859 0.856 0.002 0.837	1<br>Osind06g27610.1 0.003 1 0.997 0.003 1<br>Osind06g27610.2 0.003 1 0.997 0.003 1<br>Osind06g27610.3 0.003 1 0.997 0.002 1<br>Osind06g27610.4 0.003 1 0.997 0.003 1<br>Osind06g27610.5 0.003 1 0.997 0.004 1	0	0	0	0	0	0	0	0
	1<br>Osjap03g05350.1 0.463 1 0.537 0.247 1<br>Osjap06g28190.3 0.003 0.46 0.457 0.004 0.706	1<br>Osind03g05580.1 0.527 1 0.473 0.332 1<br>Osind04g28280.1 0.026 0.46 0.434 0.004 0.685	0<br>Orufi07g22480.1 0.026 0.476 0.45 0.003 0.619	1<br>Oglab02g05550.2 0.026 0.46 0.434 0.004 0.646<br>Oglab03g05170.2 0.463 0.997 0.534 0.204 0.997	1<br>Obart06g20620.2 0.522 1 0.478 0.413 1<br>Obart06g24100.1 0.026 0.425 0.399 0.008 0.867	1<br>Olong_011724-R1 0.026 0.46 0.434 0.004 0.679<br>Olong_012117-R1 0.547 1 0.453 0.573 0.86	0<br>Opunc06g21840.1 0.026 0.46 0.434 0.004 0.718	0<br>Obrac04g17290.1 0.026 0.524 0.498 0.004 0.782	0<br>Lperr01g34040.1 0.026 0.463 0.437 0.005 0.923	0<br>AT5G62165.1 0.026 0.501 0.475 0.005 0.881
Osind06g27610.2	1<br>Osjap06g28190.1 0.003 0.839 0.836 0.002 0.813<br>Osjap06g28190.2 0.003 0.857 0.854 0.002 0.837	1<br>Osind06g27610.1 0.003 1 0.997 0.003 1<br>Osind06g27610.2 0.003 1 0.997 0.003 1<br>Osind06g27610.3 0.003 1 0.997 0.002 1<br>Osind06g27610.4 0.003 1 0.997 0.003 1<br>Osind06g27610.5 0.003 1 0.997 0.004 1	0	0	0	0	0	0	0	0
	1<br>Osjap03g05350.1 0.471 1 0.529 0.247 1<br>Osjap06g28190.3 0.003 0.469 0.466 0.004 0.706	1<br>Osind03g05580.1 0.536 1 0.464 0.332 1<br>Osind04g28280.1 0.026 0.469 0.443 0.004 0.685	0<br>Orufi07g22480.1 0.026 0.484 0.458 0.003 0.619	1<br>Oglab02g05550.2 0.026 0.469 0.443 0.004 0.646<br>Oglab03g05170.2 0.471 0.997 0.526 0.204 0.997	1<br>Obart06g20620.2 0.531 1 0.469 0.413 1<br>Obart06g24100.1 0.026 0.432 0.406 0.008 0.867	1<br>Olong_011724-R1 0.026 0.469 0.443 0.004 0.679<br>Olong_012117-R1 0.557 1 0.443 0.573 0.86	0<br>Opunc06g21840.1 0.026 0.469 0.443 0.004 0.718	0<br>Obrac04g17290.1 0.026 0.534 0.508 0.004 0.782	0<br>Lperr01g34040.1 0.026 0.471 0.445 0.005 0.923	0<br>AT5G62165.1 0.026 0.51 0.484 0.005 0.881
Osind06g27610.4	1<br>Osjap06g28190.1 0.003 0.858 0.855 0.002 0.793<br>Osjap06g28190.2 0.003 0.858 0.855 0.002 0.802	1<br>Osind06g27610.1 0.003 1 0.997 0.003 1<br>Osind06g27610.2 0.003 1 0.997 0.003 1<br>Osind06g27610.3 0.003 1 0.997 0.002 1<br>Osind06g27610.4 0.003 1 0.997 0.003 1<br>Osind06g27610.5 0.003 1 0.997 0.004 1	0	0	0	0	0	0	0	0
	1<br>Osjap03g05350.1 0.516 1 0.484 0.247 1<br>Osjap06g28190.3 0.003 0.513 0.51 0.004 0.706	1<br>Osind03g05580.1 0.587 1 0.413 0.332 1<br>Osind07g21840.1 0.028 0.513 0.485 0.004 0.638	0<br>Orufi07g22480.1 0.028 0.53 0.502 0.003 0.619	1<br>Oglab02g05550.2 0.028 0.513 0.485 0.004 0.646<br>Oglab03g05170.3 0.516 0.997 0.481 0.234 0.996	1<br>Obart06g24100.1 0.028 0.473 0.445 0.008 0.867<br>Obart12g13810.1 0.513 1 0.487 0.443 1	1<br>Olong_011724-R1 0.028 0.513 0.485 0.004 0.679<br>Olong_012117-R2 0.61 1 0.39 0.626 0.847	0<br>Opunc06g21840.1 0.028 0.513 0.485 0.004 0.718	0<br>Obrac04g17290.1 0.028 0.584 0.556 0.004 0.782	0<br>Lperr01g34040.1 0.028 0.516 0.488 0.005 0.923	0<br>AT5G62165.1 0.028 0.558 0.53 0.005 0.881
Osind07g02300.3	1<br>Osjap07g02600.3 0.002 0.978 0.976 0.478 0.853<br>Osjap07g02600.4 0.002 1 0.998 0.555 1<br>Osjap07g02600.5 0.002 1 0.998 0.002 1	1<br>Osind07g02300.3 0.002 1 0.998 0.002 1<br>Osind07g02300.4 0.002 1 0.998 0.002 1	0	0	0	0	0	0	0	0
	1<br>Osjap01g07190.1 0.002 0.254 0.252 0.692 0.935<br>Osjap07g02840.1 0.298 0.976 0.678 0.411 0.823	1<br>Osind01g07120.1 0.002 0.254 0.252 0.692 0.935<br>Osind07g02530.1 0.298 0.976 0.678 0.411 0.823	1<br>Orufi07g02320.1 0.298 0.976 0.678 0.411 0.823<br>Orufi12g02700.1 0.007 0.259 0.252 0.518 0.746	1<br>Oglab01g05790.1 0.002 0.254 0.252 0.692 0.935<br>Oglab07g02390.2 0.298 0.976 0.678 0.41 0.823	1<br>Obart01g06210.1 0.002 0.254 0.252 0.692 0.935<br>Obart07g02450.1 0.298 0.976 0.678 0.377 0.809	1<br>Olong_003501-R1 0.002 0.254 0.252 0.634 0.923<br>Olong_009970-R1 0.298 0.976 0.678 0.411 0.823	1<br>Opunc01g05980.1 0.002 0.254 0.252 0.689 0.934<br>Opunc07g02500.8 0.298 0.976 0.678 0.472 0.79	1<br>Obrac05g04770.1 0.002 0.254 0.252 0.594 0.923<br>Obrac07g01520.1 0.298 0.99 0.692 0.386 0.893	1<br>Lperr07g02310.1 0.298 0.976 0.678 0.431 0.845<br>Lperr11g02330.1 0.007 0.257 0.25 0.682 0.933	0<br>AT4G36380.1 0.002 0.308 0.306 0.691 0.966
Osind07g02300.4	1<br>Osjap07g02600.3 0.002 0.978 0.976 0.478 0.853<br>Osjap07g02600.4 0.002 1 0.998 0.555 1<br>Osjap07g02600.5 0.002 1 0.998 0.002 1	1<br>Osind07g02300.3 0.002 1 0.998 0.002 1<br>Osind07g02300.4 0.002 1 0.998 0.002 1	0	0	0	0	0	0	0	0

	1<br>Osjap01g07190.1 0.002 0.254 0.252 0.692 0.935<br>Osjap07g02840.1 0.298 0.976 0.678 0.411 0.823	1<br>Osind01g07120.1 0.002 0.254 0.252 0.692 0.935<br>Osind07g02530.1 0.298 0.976 0.678 0.411 0.823	1<br>Orufi07g02320.1 0.298 0.976 0.678 0.411 0.823<br>Orufi12g02700.1 0.007 0.259 0.252 0.518 0.746	1<br>Oglab01g05790.1 0.002 0.254 0.252 0.692 0.935<br>Oglab07g02390.2 0.298 0.976 0.678 0.41 0.823	1<br>Obart01g06210.1 0.002 0.254 0.252 0.692 0.935<br>Obart07g02450.1 0.298 0.976 0.678 0.377 0.809	1<br>Olong_003501-R1 0.002 0.254 0.252 0.634 0.923<br>Olong_009970-R1 0.298 0.976 0.678 0.411 0.823	1<br>Opunc01g05980.1 0.002 0.254 0.252 0.689 0.934<br>Opunc07g02500.8 0.298 0.976 0.678 0.472 0.79	1<br>Obrac05g04770.1 0.002 0.254 0.252 0.594 0.923<br>Obrac07g01520.1 0.298 0.99 0.692 0.386 0.893	1<br>Lperr07g02310.1 0.298 0.976 0.678 0.431 0.845<br>Lperr11g02330.1 0.007 0.257 0.25 0.682 0.933	0<br>AT4G36380.1 0.002 0.308 0.306 0.691 0.966
Osind07g04880.1	1<br>Osjap07g05600.1 0.002 1 0.998 0.002 1	1<br>Osind07g04880.1 0.002 1 0.998 0.002 1	1<br>Orufi07g04740.1 0.002 0.94 0.938 0.002 0.996	0	0	0	0	0	0	0
	1<br>Osjap04g15050.1 0.356 0.865 0.509 0.315 0.932<br>Osjap07g05610.1 0.036 0.333 0.297 0.005 0.444	1<br>Osind04g13510.1 0.356 0.865 0.509 0.318 0.938<br>Osind07g04890.1 0.002 0.333 0.331 0.006 0.696	1<br>Orufi04g13400.1 0.356 0.865 0.509 0.318 0.938<br>Orufi07g04750.1 0.036 0.333 0.297 0.007 0.667	1<br>Oglab04g10600.1 0.356 0.865 0.509 0.323 0.938<br>Oglab07g04740.1 0.002 0.32 0.318 0.004 0.866	1<br>Obart02g19560.1 0.361 0.865 0.504 0.151 0.92<br>Obart07g04940.1 0.002 0.32 0.318 0.007 0.757	0<br>Olong_011399-R1 0.002 0.601 0.599 0.004 0.978	1<br>Opunc02g17460.1 0.361 0.873 0.512 0.326 0.95<br>Opunc08g05640.1 0.002 0.333 0.331 0.006 0.705	0<br>Obrac02g20740.1 0.569 0.908 0.339 0.635 0.984	0<br>Lperr02g15690.1 0.356 0.865 0.509 0.32 0.935	0<br>AT3G52450.1 0.567 0.865 0.298 0.618 0.936
Osind07g27670.1	1<br>Osjap07g31020.1 0.008 1 0.992 0.404 1	1<br>Osind07g27670.1 0.008 1 0.992 0.008 1<br>Osind07g27730.1 0.008 1 0.992 0.404 1	0	1<br>Oglab07g23640.1 0.016 1 0.984 0.801 0.998<br>Oglab07g23640.2 0.016 1 0.984 0.813 0.998	1<br>Obart07g27040.1 0.016 1 0.984 0.144 0.32<br>Obart07g27040.2 0.016 1 0.984 0.118 0.299	0	0	0	0	0
	1<br>Osjap03g15620.2 0.73 1 0.27 0.934 0.97<br>Osjap07g30980.1 0.008 0.639 0.631 0.199 0.358	1<br>Osind07g27700.1 0.008 0.664 0.656 0.124 0.258<br>Osind07g27740.1 0.73 1 0.27 0.939 0.972	1<br>Orufi03g15210.1 0.73 1 0.27 0.939 0.972<br>Orufi07g27980.1 0.008 0.664 0.656 0.221 0.34	1<br>Oglab03g14540.1 0.73 1 0.27 0.938 0.971<br>Oglab07g23620.1 0.008 0.664 0.656 0.124 0.258	1<br>Obart07g27030.1 0.008 0.664 0.656 0.425 0.51<br>Obart07g27050.1 0.73 1 0.27 0.939 0.972	1<br>Olong_008697-R2 0.73 1 0.27 0.936 0.971<br>Olong_008701-R1 0.008 0.664 0.656 0.124 0.258	1<br>Opunc07g25410.1 0.008 0.664 0.656 0.129 0.268<br>Opunc07g25450.1 0.73 1 0.27 0.937 0.971	1<br>Obrac03g11770.2 0.73 1 0.27 0.931 0.968<br>Obrac07g18100.1 0.008 0.664 0.656 0.131 0.271	1<br>Lperr03g12860.1 0.73 1 0.27 0.937 0.971<br>Lperr07g23810.1 0.008 0.664 0.656 0.123 0.256	0<br>AT3G66658.2 0.008 0.754 0.746 0.124 0.273
Osind08g06190.1	0	1<br>Osind08g06190.1 0.001 1 0.999 0.001 1<br>Osind08g06240.1 0.002 0.996 0.994 0.013 0.991	1<br>Orufi08g06360.1 0.002 1 0.998 0.014 0.984<br>Orufi08g06360.2 0.002 1 0.998 0.014 0.984	1<br>Oglab08g05190.1 0.002 0.996 0.994 0.001 0.998<br>Oglab08g05190.2 0.002 0.996 0.994 0.001 0.998<br>Oglab08g05190.3 0.002 0.996 0.994 0.001 0.998<br>Oglab08g05190.4 0.002 0.996 0.994 0.002 0.998<br>Oglab08g05190.5 0.002 0.996 0.994 0.002 0.998	0	1<br>Olong_020302-R1 0.002 0.942 0.94 0.002 0.986	0	0	0	0
	0<br>Osjap08g06250.1 0.002 0.649 0.647 0.089 0.974	1<br>Osind03g20810.1 0.011 0.625 0.614 0.145 0.862<br>Osind09g08750.1 0.634 0.994 0.36 0.112 0.893	1<br>Orufi03g23480.1 0.011 0.625 0.614 0.451 0.911<br>Orufi12g06630.1 0.636 0.969 0.333 0.308 0.98	0<br>Oglab10g08280.1 0.627 0.996 0.369 0.109 0.936	1<br>Obart03g22810.1 0.011 0.625 0.614 0.32 0.89<br>Obart09g08510.1 0.634 0.997 0.363 0.027 0.97	1<br>Olong_021247-R1 0.692 0.993 0.301 0.293 0.93<br>Olong_024935-R1 0.04 0.638 0.598 0.344 0.987	1<br>Opunc03g21490.1 0.011 0.625 0.614 0.177 0.734<br>Opunc08g04700.1 0.632 0.872 0.24 0.34 0.958	1<br>Obrac04g06380.1 0.635 0.994 0.359 0.156 0.825<br>Obrac07g13250.1 0.019 0.625 0.606 0.158 0.752	0<br>Lperr03g19020.1 0.017 0.625 0.608 0.28 0.884	0<br>AT3G42170.1 0.377 0.993 0.616 0.089 0.968
Osind08g06790.1	1<br>Osjap08g06650.1 0.002 1 0.998 0.002 1	1<br>Osind08g06790.1 0.002 1 0.998 0.002 1	1<br>Orufi08g06840.1 0.002 1 0.998 0.002 1	1<br>Oglab08g05640.1 0.002 1 0.998 0.002 1	1<br>Obart08g06070.1 0.002 1 0.998 0.002 1	0	1<br>Opunc08g05850.1 0.047 1 0.953 0.068 1	0	0	0
	1<br>Osjap04g21220.1 0.047 0.275 0.228 0.21 0.936<br>Osjap09g20870.1 0.359 0.984 0.625 0.208 0.966	1<br>Osind04g20250.1 0.047 0.275 0.228 0.198 0.94<br>Osind09g20960.1 0.359 0.984 0.625 0.208 0.966	1<br>Orufi04g19250.2 0.047 0.275 0.228 0.21 0.936<br>Orufi09g21960.1 0.359 0.984 0.625 0.2 0.965	1<br>Oglab02g26360.1 0.348 0.996 0.648 0.01 0.994<br>Oglab04g16180.1 0.047 0.275 0.228 0.198 0.94	1<br>Obart02g28440.1 0.348 0.996 0.648 0.093 0.994<br>Obart04g17770.1 0.047 0.275 0.228 0.198 0.94	1<br>Olong_008030-R1 0.047 0.275 0.228 0.232 0.937<br>Olong_008031-R2 0.277 1 0.723 0.106 1	1<br>Opunc04g15550.1 0.047 0.275 0.228 0.206 0.938<br>Opunc04g18740.1 0.295 0.984 0.689 0.088 0.994	1<br>Obrac08g04780.1 0.277 1 0.723 0.098 1<br>Obrac08g04800.1 0.002 0.275 0.273 0.006 0.943	1<br>Lperr08g05690.1 0.277 1 0.723 0.1 1<br>Lperr08g05700.1 0.002 0.275 0.273 0.006 0.943	1<br>AT1G05310.1 0.33 0.982 0.652 0.183 0.99<br>AT4G30800.1 0.047 0.275 0.228 0.208 0.943
Osind08g20190.1	0	1<br>Osind08g20180.1 0.139 0.975 0.836 0.237 0.449<br>Osind08g20190.1 0.008 1 0.992 0.008 1	1<br>Orufi08g10060.1 0.189 0.992 0.803 0.353 0.575<br>Orufi08g19130.1 0.131 1 0.869 0.305 0.472<br>Orufi08g19130.2 0.131 1 0.869 0.315 0.487	0	1<br>Obart08g17200.1 0.148 1 0.852 0.302 0.839	1<br>Olong_011913-R1 0.131 1 0.869 0.145 0.69	0	0	0	0
	1<br>Osjap08g09660.1 0.238 0.992 0.754 0.336 0.525<br>Osjap08g18160.1 0.033 0.238 0.205 0.876 0.905	0<br>Osind08g20150.1 0.221 0.992 0.771 0.385 0.557	1<br>Orufi08g10050.1 0.238 0.992 0.754 0.395 0.567<br>Orufi08g19160.1 0.033 0.238 0.205 0.88 0.908	1<br>Oglab08g16020.1 0.238 0.992 0.754 0.596 0.856<br>Oglab08g16050.1 0.033 0.238 0.205 0.88 0.908	1<br>Obart08g08800.1 0.246 1 0.754 0.337 0.537<br>Obart08g17210.1 0.033 0.238 0.205 0.852 0.886	1<br>Olong_011909-R1 0.033 0.238 0.205 0.792 0.849<br>Olong_011910-R1 0.238 0.992 0.754 0.097 0.617	0<br>Opunc08g15040.1 0.213 1 0.787 0.345 0.531	1<br>Obrac08g11180.1 0.238 1 0.762 0.39 0.522<br>Obrac08g11210.1 0.033 0.238 0.205 0.822 0.854	0<br>Lperr08g14190.1 0.23 0.943 0.713 0.469 0.652	0

Osind08g22530.1	1<br>Osjap08g20640.1 0.111 0.994 0.883 0.199 0.933	1<br>Osind08g22530.1 0.002 1 0.998 0.002 1<br>Osind08g22680.1 0.111 0.994 0.883 0.199 0.933	1<br>Orufi08g21480.1 0.002 1 0.998 0.001 0.97<br>Orufi08g21480.2 0.002 1 0.998 0.003 0.952<br>Orufi08g21640.1 0.111 0.994 0.883 0.199 0.933	0	0	1<br>Olong_020048-R1 0.002 1 0.998 0.003 0.94	0	0	0	0
	1<br>Osjap05g14370.1 0.491 0.912 0.421 0.004 1<br>Osjap12g00550.1 0.091 0.48 0.389 0.127 0.648	1<br>Osind12g07100.1 0.009 0.471 0.462 0.042 0.813<br>Osind12g11660.1 0.491 0.927 0.436 0.003 1	1<br>Orufi05g13530.1 0.491 0.912 0.421 0.004 1<br>Orufi12g00580.1 0.091 0.48 0.389 0.145 0.74	1<br>Oglab02g26530.1 0.491 0.927 0.436 0.003 1<br>Oglab09g10810.1 0.009 0.486 0.477 0.017 0.871	1<br>Obart09g11820.1 0.009 0.486 0.477 0.017 0.871<br>Obart11g19340.1 0.491 0.927 0.436 0.004 1	1<br>Olong_005330-R1 0.002 0.486 0.484 0.003 0.821<br>Olong_028575-R1 0.514 0.927 0.413 0.004 1	0<br>Opunc08g17290.1 0.204 1 0.796 0.002 0.47	1<br>Obrac03g12080.1 0.551 0.923 0.372 0.173 0.986<br>Obrac12g00030.1 0.026 0.486 0.46 0.436 0.966	0<br>Lperr08g16100.3 0.005 0.569 0.564 0.013 0.982	0
Osind09g02760.1	1<br>Osjap04g11160.1 0.001 1 0.999 0.001 1	1<br>Osind09g02760.1 0.001 1 0.999 0.001 1	1<br>Orufi04g09840.1 0.001 1 0.999 0.001 1	0	0	0	1<br>Opunc04g07220.1 0.017 0.988 0.971 0.005 1	0	1<br>Lperr04g05910.1 0.066 0.993 0.927 0.022 0.997	0
	1<br>Osjap09g02370.1 0.585 0.999 0.414 0.006 0.928<br>Osjap11g14300.1 0.008 0.524 0.516 0.04 0.878	1<br>Osind01g17770.1 0.585 0.999 0.414 0.006 0.928<br>Osind08g20650.1 0.008 0.524 0.516 0.04 0.878	1<br>Orufi10g19500.1 0.563 0.988 0.425 0.022 0.976<br>Orufi11g12980.1 0.008 0.524 0.516 0.039 0.881	1<br>Oglab01g12700.1 0.585 0.999 0.414 0.006 0.928<br>Oglab11g10800.1 0.008 0.524 0.516 0.04 0.878	1<br>Obart04g08610.1 0.544 1 0.456 0.001 1<br>Obart11g12300.1 0.008 0.524 0.516 0.04 0.878	1<br>Olong_024054-R1 0.585 1 0.415 0.002 1<br>Olong_024055-R1 0.01 0.548 0.538 0.001 0.96	1<br>Opunc08g10560.1 0.563 0.986 0.423 0.07 0.993<br>Opunc11g10540.3 0.008 0.524 0.516 0.087 0.889	1<br>Obrac04g04850.1 0.008 0.556 0.548 0.043 0.975<br>Obrac10g11900.4 0.567 0.982 0.415 0.03 0.966	1<br>Lperr02g04400.1 0.555 0.967 0.412 0.048 0.961<br>Lperr11g10010.2 0.008 0.524 0.516 0.037 0.886	1<br>AT1G25360.1 0.57 0.996 0.426 0.019 0.914<br>AT5G44240.1 0.008 0.524 0.516 0.066 0.889
Osind09g09040.1	1<br>Osjap11g06710.1 0.001 1 0.999 0.067 0.999<br>Osjap11g06720.1 0.001 0.999 0.998 0.054 0.981	1<br>Osind09g09040.1 0.001 1 0.999 0.001 1	1<br>Orufi11g05930.1 0.001 1 0.999 0.09 1<br>Orufi11g05940.1 0.001 1 0.999 0.002 1	1<br>Oglab11g05440.1 0.001 0.995 0.994 0.001 0.998	1<br>Obart11g05910.1 0.001 0.88 0.879 0.05 1	0	0	0	0	0
	1<br>Osjap01g24690.1 0.525 0.989 0.464 0.014 0.934<br>Osjap11g06740.8 0.022 0.484 0.462 0.013 0.907	1<br>Osind09g09050.1 0.001 0.481 0.48 0.104 0.994<br>Osind11g05650.1 0.522 1 0.478 0.002 1	1<br>Orufi11g05890.1 0.522 1 0.478 0.003 0.997<br>Orufi11g05900.2 0.001 0.481 0.48 0.002 0.978	1<br>Oglab08g10820.1 0.522 0.955 0.433 0.002 0.867<br>Oglab11g05430.1 0.001 0.503 0.502 0.049 0.944	1<br>Obart01g20930.1 0.525 0.989 0.464 0.006 0.498<br>Obart11g05930.1 0.001 0.481 0.48 0.002 0.998	1<br>Olong_000002-R1 0.001 0.517 0.516 0.117 0.996<br>Olong_005787-R1 0.522 1 0.478 0.002 1	0<br>Opunc11g05960.1 0.425 1 0.575 0.002 1	1<br>Obrac01g15800.1 0.521 0.989 0.468 0.004 0.915<br>Obrac11g03930.1 0.001 0.482 0.481 0.002 0.939	1<br>Lperr05g13220.1 0.522 1 0.478 0.002 1<br>Lperr11g05360.1 0.001 0.481 0.48 0.044 0.995	0<br>AT4G38130.1 0.522 0.955 0.433 0.002 0.782
Osind09g09810.2	1<br>Osjap09g09660.1 0.001 0.984 0.983 0.001 0.615<br>Osjap09g09660.2 0.001 1 0.999 0.001 1<br>Osjap09g09660.3 0.001 1 0.999 0.001 1<br>Osjap09g09660.4 0.001 1 0.999 0.001 1	1<br>Osind09g09810.1 0.001 0.984 0.983 0.001 0.605<br>Osind09g09810.2 0.001 1 0.999 0.001 1	1<br>Orufi09g10340.1 0.001 0.984 0.983 0.001 0.67<br>Orufi09g10340.2 0.001 1 0.999 0.001 1<br>Orufi09g10340.3 0.001 1 0.999 0.001 1	0	0	0	1<br>Opunc09g08150.1 0.001 1 0.999 0.001 1	0	1<br>Lperr09g06610.1 0.001 1 0.999 0.001 0.802<br>Lperr09g06610.2 0.001 1 0.999 0.001 1	0
	1<br>Osjap02g00030.1 0.593 0.846 0.253 0.196 0.692<br>Osjap08g16360.1 0.001 0.563 0.562 0.002 0.984	1<br>Osind02g00030.1 0.593 0.846 0.253 0.188 0.689<br>Osind08g18230.1 0.001 0.563 0.562 0.002 0.984	1<br>Orufi02g00020.1 0.593 0.846 0.253 0.282 0.725<br>Orufi08g17320.1 0.001 0.563 0.562 0.002 0.984	1<br>Oglab01g06450.1 0.656 0.997 0.341 0.674 0.999<br>Oglab08g14350.1 0.001 0.563 0.562 0.002 0.984	1<br>Obart08g15380.1 0.001 0.563 0.562 0.002 0.984<br>Obart09g09660.3 0.566 1 0.434 0.136 1	1<br>Olong_009135-R1 0.001 0.564 0.563 0.086 0.862<br>Olong_009135-R6 0.592 0.99 0.398 0.003 0.983	0<br>Opunc08g12760.1 0.001 0.563 0.562 0.002 0.984<br>Opunc11g05680.1 0.593 0.818 0.225 0.151 0.422	1<br>Obrac09g05000.3 0.592 1 0.408 0.174 1<br>Obrac09g05010.1 0.001 0.564 0.563 0.002 0.988	1<br>Lperr02g00030.2 0.593 0.846 0.253 0.27 0.728<br>Lperr08g12650.1 0.001 0.56 0.559 0.003 0.96	0<br>AT3G17880.1 0.608 1 0.392 0.195 0.997
Osind09g15570.1	1<br>Osjap09g15430.2 0.002 1 0.998 0.002 1<br>Osjap09g15430.3 0.002 1 0.998 0.002 1	1<br>Osind09g15570.1 0.002 1 0.998 0.002 1<br>Osind09g15570.2 0.002 1 0.998 0.002 1	1<br>Orufi09g16160.1 0.002 1 0.998 0.002 1<br>Orufi09g16160.2 0.002 1 0.998 0.002 1	1<br>Oglab09g13510.1 0.002 1 0.998 0.002 1<br>Oglab09g13510.2 0.002 1 0.998 0.002 1<br>Oglab09g13510.3 0.002 1 0.998 0.002 1<br>Oglab09g13510.4 0.002 1 0.998 0.002 1	1<br>Obart09g14980.1 0.002 1 0.998 0.002 1<br>Obart09g14980.2 0.002 1 0.998 0.002 1	0	1<br>Opunc09g13420.1 0.002 1 0.998 0.002 1<br>Opunc09g13420.2 0.002 1 0.998 0.002 1	1<br>Obrac09g09090.1 0.002 1 0.998 0.002 1	0	0
	1<br>Osjap02g06650.1 0.01 0.54 0.53 0.01 0.997<br>Osjap09g15430.1 0.544 1 0.456 0.131 1	1<br>Osind02g06940.1 0.01 0.54 0.53 0.01 0.997<br>Osind08g23920.1 0.544 0.993 0.449 0.131 0.988	1<br>Orufi04g24220.1 0.013 0.542 0.529 0.165 1<br>Orufi08g22880.1 0.544 0.993 0.449 0.131 0.988	1<br>Oglab08g19240.1 0.544 0.993 0.449 0.143 0.986<br>Oglab12g18680.1 0.002 0.542 0.54 0.003 1	1<br>Obart02g06180.1 0.01 0.54 0.53 0.01 0.997<br>Obart08g20390.1 0.544 0.993 0.449 0.631 0.988	1<br>Olong_013054-R1 0.547 0.992 0.445 0.149 0.979<br>Olong_024186-R1 0.002 0.542 0.54 0.003 1	1<br>Opunc04g20190.1 0.007 0.542 0.535 0.006 1<br>Opunc08g18560.1 0.544 0.993 0.449 0.128 0.969	1<br>Obrac02g04550.3 0.008 0.54 0.532 0.214 0.997<br>Obrac09g00560.1 0.548 0.998 0.45 0.164 0.966	1<br>Lperr04g18350.1 0.002 0.542 0.54 0.021 1<br>Lperr09g11120.1 0.544 1 0.456 0.156 1	1<br>AT1G68540.1 0.542 0.992 0.45 0.112 1<br>AT2G21270.3 0.003 0.54 0.537 0.059 0.997

Osind09g15570.2	1<br>Osjap09g15430.2 0.002 1 0.998 0.002 1<br>Osjap09g15430.3 0.002 1 0.998 0.002 1	1<br>Osind09g15570.1 0.002 1 0.998 0.002 1<br>Osind09g15570.2 0.002 1 0.998 0.002 1	1<br>Orufi09g16160.1 0.002 1 0.998 0.002 1<br>Orufi09g16160.2 0.002 1 0.998 0.002 1	1<br>Oglab09g13510.1 0.002 1 0.998 0.002 1<br>Oglab09g13510.2 0.002 1 0.998 0.002 1<br>Oglab09g13510.3 0.002 1 0.998 0.002 1<br>Oglab09g13510.4 0.002 1 0.998 0.002 1	1<br>Obart09g14980.1 0.002 1 0.998 0.002 1<br>Obart09g14980.2 0.002 1 0.998 0.002 1	0	1<br>Opunc09g13420.1 0.002 1 0.998 0.002 1<br>Opunc09g13420.2 0.002 1 0.998 0.002 1	1<br>Obrac09g09090.1 0.002 1 0.998 0.002 1	0	0
	1<br>Osjap02g06650.1 0.01 0.565 0.555 0.01 0.997<br>Osjap09g15430.1 0.569 1 0.431 0.131 1	1<br>Osind02g06940.1 0.01 0.565 0.555 0.01 0.997<br>Osind08g23920.1 0.569 0.993 0.424 0.131 0.988	1<br>Orufi04g24220.1 0.014 0.567 0.553 0.165 1<br>Orufi08g22880.1 0.569 0.993 0.424 0.131 0.988	1<br>Oglab08g19240.1 0.569 0.993 0.424 0.143 0.986<br>Oglab12g18680.1 0.002 0.567 0.565 0.003 1	1<br>Obart02g06180.1 0.01 0.565 0.555 0.01 0.997<br>Obart08g20390.1 0.569 0.993 0.424 0.631 0.988	1<br>Olong_008093-R2 0.596 0.991 0.395 0.25 0.996<br>Olong_024186-R1 0.002 0.567 0.565 0.003 1	1<br>Opunc04g20190.1 0.007 0.567 0.56 0.006 1<br>Opunc08g18560.1 0.569 0.993 0.424 0.128 0.969	1<br>Obrac01g09090.1 0.605 0.998 0.393 0.299 0.982<br>Obrac02g04550.3 0.009 0.565 0.556 0.214 0.997	1<br>Lperr04g18350.1 0.002 0.567 0.565 0.021 1<br>Lperr09g11120.1 0.569 1 0.431 0.156 1	1<br>AT1G68540.1 0.567 0.991 0.424 0.112 1<br>AT2G21270.3 0.003 0.565 0.562 0.059 0.997
Osind09g15930.1	0	1<br>Osind09g15930.1 0.001 1 0.999 0.001 1	1<br>Orufi09g16580.1 0.001 1 0.999 0.061 1	1<br>Oglab09g13920.1 0.001 1 0.999 0.061 1	1<br>Obart09g15360.1 0.057 1 0.943 0.001 1	0	0	0	0	0
	1<br>Osjap03g04840.3 0.544 0.887 0.343 0.018 0.769<br>Osjap09g15790.1 0.001 0.544 0.543 0.155 0.993	1<br>Osind08g24670.1 0.004 0.544 0.54 0.138 0.995<br>Osind09g02290.1 0.544 0.887 0.343 0.018 0.769	1<br>Orufi03g04550.5 0.544 0.887 0.343 0.017 0.733<br>Orufi08g23480.1 0.004 0.544 0.54 0.146 0.995	1<br>Oglab02g15910.1 0.037 0.512 0.475 0.001 0.988<br>Oglab09g02290.1 0.544 0.887 0.343 0.02 0.789	1<br>Obart03g04820.1 0.569 0.887 0.318 0.1 0.796<br>Obart08g21020.1 0.056 0.544 0.488 0.086 0.994	1<br>Olong_025005-R2 0.571 0.98 0.409 0.001 0.955<br>Olong_025006-R1 0.105 0.544 0.439 0.031 0.99	1<br>Opunc08g19030.2 0.006 0.544 0.538 0.071 0.976<br>Opunc09g13790.1 0.544 0.98 0.436 0.055 0.958	1<br>Obrac08g14010.2 0.036 0.544 0.508 0.001 0.989<br>Obrac09g09380.2 0.544 0.98 0.436 0.01 0.954	1<br>Lperr09g11500.1 0.001 0.544 0.543 0.105 0.992<br>Lperr09g11510.2 0.544 0.98 0.436 0.01 0.958	1<br>AT2G32000.1 0.544 0.978 0.434 0.012 0.961<br>AT2G43900.1 0.002 0.519 0.517 0.083 0.843
Osind09g18210.1	1<br>Osjap09g17990.1 0.002 1 0.998 0.157 1	1<br>Osind09g18210.1 0.002 1 0.998 0.002 1	0	1<br>Oglab09g15840.1 0.002 1 0.998 0.002 1	1<br>Obart09g17630.1 0.002 1 0.998 0.153 1	0	0	0	0	0
	0<br>Osjap01g39390.1 0.508 0.89 0.382 0.144 0.713	1<br>Osind01g40800.1 0.508 0.89 0.382 0.104 0.917<br>Osind08g26570.1 0.002 0.476 0.474 0.004 1	1<br>Orufi09g19030.1 0.002 0.476 0.474 0.004 1<br>Orufi09g19040.1 0.489 1 0.511 0.003 1	0<br>Oglab06g16980.1 0.489 0.897 0.408 0.005 0.903	1<br>Obart06g18820.1 0.489 0.897 0.408 0.005 0.903<br>Obart08g22690.1 0.016 0.476 0.46 0.265 1	1<br>Olong_007765-R1 0.002 0.476 0.474 0.004 1<br>Olong_023444-R1 0.534 0.89 0.356 0.35 0.752	1<br>Opunc01g33270.1 0.508 0.89 0.382 0.155 0.871<br>Opunc08g20790.1 0.002 0.476 0.474 0.004 1	1<br>Obrac01g26370.4 0.508 0.884 0.376 0.313 0.74<br>Obrac09g11280.2 0.002 0.476 0.474 0.004 1	1<br>Lperr01g29580.1 0.508 0.89 0.382 0.162 0.862<br>Lperr09g13540.1 0.002 0.476 0.474 0.273 1	1<br>AT1G75760.1 0.002 0.476 0.474 0.004 1<br>AT3G48520.1 0.508 0.888 0.38 0.316 0.725
Osind09g20560.1	1<br>Osjap09g20460.1 0.001 0.998 0.997 0.001 0.986	1<br>Osind09g20560.1 0.001 1 0.999 0.001 1	1<br>Orufi09g21510.1 0.001 0.978 0.977 0.001 0.999	1<br>Oglab09g18200.1 0.001 0.998 0.997 0.001 0.985	1<br>Obart09g19980.1 0.067 0.998 0.931 0.165 0.99<br>Obart09g19980.2 0.001 0.998 0.997 0.001 0.984	1<br>Olong_014589-R1 0.117 0.985 0.868 0.018 0.991<br>Olong_014589-R2 0.117 0.985 0.868 0.018 0.991	0	0	0	0
	1<br>Osjap02g21530.2 0.024 0.581 0.557 0.096 0.943<br>Osjap09g20430.1 0.623 0.998 0.375 0.085 0.962	1<br>Osind02g21430.2 0.024 0.581 0.557 0.096 0.943<br>Osind09g20550.1 0.623 0.998 0.375 0.032 0.982	1<br>Orufi02g19810.2 0.024 0.581 0.557 0.096 0.943<br>Orufi09g21500.1 0.623 0.998 0.375 0.085 0.962	1<br>Oglab02g17850.1 0.024 0.581 0.557 0.095 0.914<br>Oglab09g18170.1 0.623 0.998 0.375 0.079 0.966	1<br>Obart04g25370.1 0.047 0.589 0.542 0.176 0.967<br>Obart09g19950.1 0.623 0.998 0.375 0.079 0.966	1<br>Olong_014116-R1 0.064 0.589 0.525 0.069 0.957<br>Olong_027417-R1 0.625 0.982 0.357 0.135 0.711	1<br>Opunc09g18280.1 0.618 0.998 0.38 0.058 0.841<br>Opunc09g18290.2 0.001 0.59 0.589 0.001 0.97	1<br>Obrac09g13140.3 0.623 0.998 0.375 0.049 0.806<br>Obrac09g13160.3 0.001 0.596 0.595 0.001 0.996	1<br>Lperr09g15890.1 0.622 0.999 0.377 0.048 0.95<br>Lperr09g15920.1 0.001 0.59 0.589 0.001 0.957	0<br>AT1G18160.1 0.021 0.589 0.568 0.064 0.958
Osind10g19230.1	0	1<br>Osind10g19230.1 0.002 1 0.998 0.002 1	1<br>Orufi10g20960.1 0.002 1 0.998 0.002 1	1<br>Oglab10g16090.1 0.002 0.992 0.99 0.001 0.861	1<br>Obart10g19720.1 0.002 1 0.998 0.002 1	0	1<br>Opunc10g18180.1 0.002 0.992 0.99 0.002 0.85	0	0	0
	1<br>Osjap10g22750.1 0.509 0.992 0.483 0.061 0.836<br>Osjap10g22760.1 0.002 0.486 0.484 0.003 0.993	1<br>Osind01g20890.1 0.519 0.992 0.473 0.154 0.668<br>Osind08g03990.1 0.046 0.464 0.418 0.057 0.95	1<br>Orufi01g19490.1 0.519 0.992 0.473 0.171 0.72<br>Orufi08g04140.1 0.046 0.464 0.418 0.057 0.95	1<br>Oglab01g14760.1 0.519 0.992 0.473 0.2 0.827<br>Oglab08g03250.2 0.046 0.464 0.418 0.052 0.955	1<br>Obart01g17550.1 0.519 0.992 0.473 0.174 0.721<br>Obart08g03750.1 0.046 0.464 0.418 0.057 0.95	1<br>Olong_006312-R1 0.516 0.986 0.47 0.067 0.835<br>Olong_006313-R1 0.002 0.486 0.484 0.003 0.993	1<br>Opunc01g18040.1 0.519 0.992 0.473 0.174 0.822<br>Opunc08g03700.1 0.017 0.464 0.447 0.011 0.951	1<br>Obrac01g13330.1 0.525 0.992 0.467 0.196 0.825<br>Obrac10g13120.1 0.002 0.486 0.484 0.004 0.993	1<br>Lperr10g15590.1 0.516 0.992 0.476 0.07 0.79<br>Lperr10g15600.1 0.002 0.482 0.48 0.003 0.979	1<br>AT1G32080.1 0.521 0.988 0.467 0.188 0.82<br>AT2G32260.1 0.044 0.465 0.421 0.078 0.825
Osind11g00690.1	1<br>Osjap11g01220.1 0.001 0.999 0.998 0.001 0.955<br>Osjap12g01140.1 0.001 0.999 0.998 0.001 0.953	1<br>Osind11g00690.1 0.001 1 0.999 0.001 1<br>Osind12g01290.1 0.001 0.999 0.998 0.001 0.954	1<br>Orufi11g01000.1 0.001 0.999 0.998 0.001 0.951<br>Orufi12g01130.1 0.001 0.999 0.998 0.001 0.953	1<br>Oglab12g00800.1 0.001 0.999 0.998 0.001 0.956	1<br>Obart11g01060.1 0.001 0.999 0.998 0.001 0.954	0	1<br>Opunc12g00940.1 0.001 0.999 0.998 0.001 0.937	0	0	0
	1<br>Osjap03g13100.1 0.523 0.999 0.476 0.097 0.593	1<br>Osind03g12930.1 0.523 0.999 0.476 0.121 0.604	1<br>Orufi03g17220.1 0.53 0.999 0.469 0.027 0.912	1<br>Oglab03g12120.1 0.523 0.999 0.476 0.121 0.604	1<br>Obart03g12120.1 0.523 0.999 0.476 0.121 0.604	1<br>Olong_007715-R1 0.527 0.999 0.472 0.009 0.911	1<br>Opunc03g11680.1 0.523 0.999 0.476 0.148 0.924	1<br>Obrac03g09720.2 0.523 0.999 0.476 0.142 0.923	0<br>Lperr11g00920.1 0.001 0.462 0.461 0.002 0.949	1<br>AT2G34940.1 0.001 0.462 0.461 0.002 0.955

	Osjap07g30390.1 0.01 0.462 0.452 0.013 0.944	Osind07g27070.1 0.007 0.462 0.455 0.006 0.946	Orufi07g27420.1 0.01 0.462 0.452 0.013 0.946	Oglab07g22980.1 0.007 0.462 0.455 0.007 0.94	Obart12g00790.1 0.001 0.462 0.461 0.002 0.949	Olong_008211-R1 0.007 0.462 0.455 0.006 0.946	Opunc11g00920.1 0.001 0.462 0.461 0.142 0.951	Obrac12g00780.1 0.001 0.462 0.461 0.11 0.953		AT3G46740.1 0.526 1 0.474 0.161 0.928
Osind11g00780.1	0	1<br>Osind11g00780.1 0.001 1 0.999 0.001 1<br>Osind12g01380.1 0.003 0.881 0.878 0.211 0.964	1<br>Orufi11g01080.1 0.028 1 0.972 0.002 1	0	0	0	0	0	0	0
	1<br>Osjap11g01340.1 0.569 1 0.431 0.04 1<br>Osjap12g01290.2 0.003 0.488 0.485 0.011 1	0<br>Osind05g13850.1 0.632 0.999 0.367 0.2 0.925<br>Osind08g02040.1 0.008 0.351 0.343 0.041 0.637	1<br>Orufi12g01220.1 0.624 1 0.376 0.003 1<br>Orufi12g01240.1 0.028 0.488 0.46 0.002 1	1<br>Oglab12g00910.1 0.622 1 0.378 0.11 1<br>Oglab12g00920.1 0.031 0.488 0.457 0.05 1	1<br>Obart12g00900.1 0.622 1 0.378 0.083 1<br>Obart12g00910.1 0.003 0.488 0.485 0.015 1	1<br>Olong_005170-R1 0.634 0.991 0.357 0.123 0.942<br>Olong_028818-R1 0.003 0.488 0.485 0.014 1	1<br>Opunc12g01050.1 0.618 0.996 0.378 0.092 0.988<br>Opunc12g01060.1 0.003 0.488 0.485 0.015 1	0<br>Obrac12g00870.5 0.618 0.999 0.381 0.095 0.997	1<br>Lperr11g01030.1 0.003 0.488 0.485 0.098 1<br>Lperr12g01130.1 0.593 0.998 0.405 0.01 0.99	1<br>AT2G30520.1 0.003 0.488 0.485 0.042 1<br>AT5G01930.1 0.625 0.992 0.367 0.085 0.915
Osind11g01990.1	1<br>Osjap11g02640.1 0.002 1 0.998 0.002 1<br>Osjap12g02610.1 0.002 1 0.998 0.002 1	1<br>Osind11g01990.1 0.002 1 0.998 0.002 1<br>Osind12g02780.1 0.002 1 0.998 0.002 1	1<br>Orufi11g02320.1 0.002 1 0.998 0.013 1<br>Orufi12g02480.1 0.002 1 0.998 0.002 1	1<br>Oglab11g02200.1 0.002 1 0.998 0.002 1<br>Oglab12g02130.1 0.002 1 0.998 0.002 1	1<br>Obart12g02080.1 0.023 1 0.977 0.002 1	0	0	0	0	0
	1<br>Osjap07g04360.1 0.033 0.669 0.636 0.018 0.925<br>Osjap12g12870.1 0.673 0.949 0.276 0.055 0.417	1<br>Osind11g02010.1 0.014 0.669 0.655 0.001 0.994<br>Osind12g10020.1 0.673 0.949 0.276 0.055 0.417	1<br>Orufi12g02470.1 0.045 0.669 0.624 0.04 0.988<br>Orufi12g10750.1 0.671 0.949 0.278 0.045 0.364	1<br>Oglab07g03810.1 0.033 0.669 0.636 0.019 0.987<br>Oglab12g09880.1 0.673 0.949 0.276 0.051 0.385	0<br>Obart11g02350.1 0.021 0.669 0.648 0.024 0.993	1<br>Olong_012584-R1 0.066 0.622 0.556 0.002 0.965<br>Olong_017316-R1 0.671 0.949 0.278 0.045 0.365	1<br>Opunc11g02190.1 0.002 0.669 0.667 0.003 0.989<br>Opunc12g08740.1 0.671 0.949 0.278 0.04 0.324	1<br>Obrac05g10860.1 0.051 0.669 0.618 0.097 0.987<br>Obrac12g07170.1 0.671 0.94 0.269 0.071 0.57	1<br>Lperr12g02190.1 0.002 0.669 0.667 0.003 0.989<br>Lperr12g08370.1 0.669 0.949 0.28 0.047 0.391	0<br>AT5G27320.1 0.014 0.643 0.629 0.003 0.98
Osind11g09560.1	1<br>Osjap11g12460.1 0.009 0.896 0.887 0.338 0.846	1<br>Osind11g09560.1 0.009 1 0.991 0.009 1	1<br>Orufi11g11390.1 0.009 0.896 0.887 0.338 0.846	1<br>Oglab11g09700.1 0.009 0.896 0.887 0.34 0.85	1<br>Obart11g10770.1 0.009 0.896 0.887 0.29 0.839	0	0	0	0	0
	1<br>Osjap05g02540.1 0.304 0.974 0.67 0.002 0.202<br>Osjap10g10280.2 0.009 0.252 0.243 0.604 0.743	1<br>Osind05g02520.1 0.304 0.974 0.67 0.002 0.202<br>Osind10g07670.1 0.009 0.252 0.243 0.63 0.759	0<br>Orufi01g10400.1 0.243 0.939 0.696 0.168 0.326	1<br>Oglab03g23910.1 0.009 0.252 0.243 0.814 0.982<br>Oglab05g02200.1 0.304 0.974 0.67 0.002 0.202	1<br>Obart03g24070.1 0.009 0.252 0.243 0.551 0.957<br>Obart05g02350.1 0.304 0.974 0.67 0.002 0.202	1<br>Olong_021023-R2 0.009 0.252 0.243 0.018 0.518<br>Olong_025943-R1 0.304 1 0.696 0.003 0.247	0<br>Opunc05g02230.1 0.304 0.974 0.67 0.002 0.202	0<br>Obrac03g30060.1 0.148 0.939 0.791 0.046 0.255	0<br>Lperr01g11350.1 0.304 0.939 0.635 0.003 0.195	0<br>AT5G26751.1 0.304 0.974 0.67 0.002 0.19
Osind11g17120.1	1<br>Osjap11g23010.1 0.004 0.992 0.988 0.002 0.589	1<br>Osind11g17120.1 0.004 1 0.996 0.004 1	1<br>Orufi11g21270.1 0.004 0.992 0.988 0.002 0.538	0	0	0	0	0	0	0
	1<br>Osjap10g07190.1 0.012 0.669 0.657 0.132 0.882<br>Osjap11g23280.1 0.669 0.992 0.323 0.285 0.467	1<br>Osind07g22620.1 0.062 0.619 0.557 0.157 0.93<br>Osind11g17350.1 0.623 0.992 0.369 0.433 0.864	1<br>Orufi10g06540.1 0.012 0.669 0.657 0.132 0.882<br>Orufi11g21510.1 0.669 0.992 0.323 0.285 0.467	1<br>Oglab10g05750.1 0.012 0.669 0.657 0.128 0.882<br>Oglab11g17490.1 0.669 0.992 0.323 0.285 0.467	1<br>Obart04g09320.1 0.767 0.992 0.225 0.376 0.428<br>Obart10g06170.1 0.012 0.669 0.657 0.132 0.882	1<br>Olong_020397-R1 0.004 0.584 0.58 0.006 0.787<br>Olong_023942-R1 0.669 0.992 0.323 0.349 0.515	1<br>Opunc06g04020.1 0.77 0.996 0.226 0.534 0.607<br>Opunc10g05480.1 0.012 0.669 0.657 0.132 0.882	1<br>Obrac04g04990.1 0.759 0.992 0.233 0.158 0.31<br>Obrac10g03800.1 0.012 0.611 0.599 0.155 0.885	1<br>Lperr04g06250.2 0.759 0.992 0.233 0.709 0.762<br>Lperr11g04260.1 0.027 0.626 0.599 0.249 0.427	0<br>AT2G21110.1 0.031 0.615 0.584 0.129 0.941
Osind11g17390.1	0	1<br>Osind11g17350.1 0.07 0.98 0.91 0.001 0.992<br>Osind11g17390.1 0.002 1 0.998 0.002 1	1<br>Orufi11g21490.1 0.002 0.997 0.995 0.088 0.76	0	0	0	0	0	0	0
	1<br>Osjap11g23270.1 0.449 0.997 0.548 0.184 0.651<br>Osjap11g23280.1 0.07 0.429 0.359 0.456 0.955	1<br>Osind11g17360.1 0.449 0.997 0.548 0.156 0.559<br>Osind11g17370.1 0.07 0.429 0.359 0.24 0.938	1<br>Orufi10g18830.1 0.076 0.437 0.361 0.48 0.821<br>Orufi11g21400.1 0.443 0.974 0.531 0.187 0.524	1<br>Oglab11g17470.1 0.429 0.98 0.551 0.042 0.513<br>Oglab11g17490.1 0.047 0.429 0.382 0.42 0.955	1<br>Obart02g27080.1 0.049 0.437 0.388 0.303 0.902<br>Obart03g04520.1 0.46 0.983 0.523 0.004 0.325	1<br>Olong_023942-R1 0.07 0.429 0.359 0.506 0.959<br>Olong_027580-R1 0.429 0.98 0.551 0.054 0.519	1<br>Opunc10g16000.1 0.073 0.423 0.35 0.642 0.876<br>Opunc11g04080.1 0.423 0.983 0.56 0.038 0.488	1<br>Obrac09g13360.3 0.061 0.431 0.37 0.63 0.894<br>Obrac11g11670.1 0.455 0.973 0.518 0.002 0.429	0<br>Lperr11g16280.5 0.426 0.992 0.566 0.046 0.496	0<br>AT1G16760.1 0.023 0.429 0.406 0.611 0.933
Osind12g01290.1	1<br>Osjap11g01220.1 0.001 1 0.999 0.001 1<br>Osjap12g01140.1 0.001 1 0.999 0.001 1	1<br>Osind11g00690.1 0.001 0.954 0.953 0.001 0.999<br>Osind12g01290.1 0.001 1 0.999 0.001 1	1<br>Orufi11g01000.1 0.001 1 0.999 0.001 1<br>Orufi12g01130.1 0.001 1 0.999 0.001 1	1<br>Oglab12g00800.1 0.001 1 0.999 0.001 1	1<br>Obart11g01060.1 0.001 1 0.999 0.001 1	0	1<br>Opunc12g00940.1 0.001 1 0.999 0.001 1	0	0	0
	1	1	1	1	1	1	1	1	1	1

	Osjap03g13100.1 0.502 0.981 0.479 0.097 0.622<br>Osjap07g30390.1 0.01 0.366 0.356 0.013 0.798	Osind03g12930.1 0.502 0.981 0.479 0.121 0.633<br>Osind07g27070.1 0.007 0.37 0.363 0.006 0.783	Orufi03g17220.1 0.509 1 0.491 0.027 1<br>Orufi07g27420.1 0.01 0.37 0.36 0.013 0.783	Oglab03g12120.1 0.502 0.981 0.479 0.121 0.633<br>Oglab07g22980.1 0.007 0.366 0.359 0.007 0.783	Obart03g12120.1 0.502 0.981 0.479 0.121 0.633<br>Obart10g06750.1 0.021 0.401 0.38 0.151 0.885	Olong_007715-R1 0.505 1 0.495 0.007 1<br>Olong_008211-R1 0.007 0.37 0.363 0.006 0.783	Opunc03g11680.1 0.502 1 0.498 0.148 1<br>Opunc11g00920.1 0.001 0.37 0.369 0.142 0.816	Obrac03g09720.2 0.502 1 0.498 0.142 1<br>Obrac10g04150.1 0.019 0.398 0.379 0.036 0.868	Lperr03g10720.1 0.502 0.993 0.491 0.148 0.951<br>Lperr11g00920.1 0.001 0.37 0.369 0.002 0.792	AT2G34940.1 0.001 0.37 0.369 0.002 0.793<br>AT3G46740.1 0.505 1 0.495 0.161 1
Osind12g02780.1	1<br>Osjap11g02640.1 0.002 1 0.998 0.002 1<br>Osjap12g02610.1 0.002 1 0.998 0.002 1	1<br>Osind11g01990.1 0.002 1 0.998 0.002 1<br>Osind12g02780.1 0.002 1 0.998 0.002 1	1<br>Orufi11g02320.1 0.002 1 0.998 0.003 1<br>Orufi12g02480.1 0.002 1 0.998 0.002 1	1<br>Oglab11g02200.1 0.002 1 0.998 0.002 1<br>Oglab12g02130.1 0.002 1 0.998 0.002 1	1<br>Obart12g02080.1 0.022 1 0.978 0.002 1	0	0	0	0	0
	1<br>Osjap07g04360.1 0.031 0.64 0.609 0.018 0.925<br>Osjap12g12870.1 0.651 0.953 0.302 0.022 0.417	1<br>Osind11g02010.1 0.013 0.64 0.627 0.001 0.994<br>Osind12g10020.1 0.651 0.953 0.302 0.022 0.417	1<br>Orufi07g03650.1 0.031 0.64 0.609 0.019 0.976<br>Orufi12g10910.1 0.651 0.953 0.302 0.022 0.417	1<br>Oglab07g03810.1 0.031 0.64 0.609 0.019 0.987<br>Oglab12g09880.1 0.651 0.953 0.302 0.02 0.385	0<br>Obart11g02350.1 0.02 0.64 0.62 0.004 0.993	1<br>Olong_012584-R1 0.061 0.597 0.536 0.002 0.965<br>Olong_017316-R1 0.651 0.953 0.302 0.017 0.365	1<br>Opunc11g02190.1 0.002 0.64 0.638 0.003 0.989<br>Opunc12g08740.1 0.651 0.953 0.302 0.015 0.324	1<br>Obrac05g10860.1 0.047 0.64 0.593 0.097 0.987<br>Obrac12g07170.1 0.651 0.944 0.293 0.029 0.57	1<br>Lperr12g02190.1 0.002 0.64 0.638 0.003 0.989<br>Lperr12g08380.1 0.644 0.987 0.343 0.01 0.488	0<br>AT5G16080.1 0.047 0.644 0.597 0.081 0.994
Osind12g09530.1	1<br>Osjap12g12320.1 0.001 1 0.999 0.001 1	1<br>Osind12g09530.1 0.001 1 0.999 0.001 1	1<br>Orufi12g10460.1 0.001 1 0.999 0.001 1	1<br>Oglab12g09470.1 0.001 1 0.999 0.001 1	1<br>Obart12g09410.1 0.001 1 0.999 0.001 1	0	0	0	0	0
	1<br>Osjap01g44780.2 0.453 0.998 0.545 0.095 0.994<br>Osjap03g07600.1 0.001 0.292 0.291 0.004 0.996	1<br>Osind01g46040.1 0.453 0.998 0.545 0.151 0.995<br>Osind03g07670.1 0.001 0.292 0.291 0.004 0.996	1<br>Orufi01g42650.3 0.453 0.998 0.545 0.195 0.995<br>Orufi03g07180.1 0.001 0.292 0.291 0.004 0.996	0<br>Oglab01g34040.1 0.502 0.998 0.496 0.203 0.995<br>Oglab03g07280.1 0.001 0.292 0.291 0.004 0.996	1<br>Obart01g39260.3 0.469 0.998 0.529 0.041 0.994<br>Obart03g07210.1 0.001 0.292 0.291 0.004 0.996	1<br>Olong_021912-R1 0.47 1 0.53 0.099 1<br>Olong_021913-R1 0.001 0.291 0.29 0.004 0.996	1<br>Opunc12g08570.1 0.334 1 0.666 0.017 1<br>Opunc12g08580.1 0.001 0.291 0.29 0.004 0.996	1<br>Obrac03g05880.1 0.001 0.291 0.29 0.004 0.996<br>Obrac12g06500.1 0.475 1 0.525 0.103 1	1<br>Lperr01g33810.2 0.469 0.998 0.529 0.002 0.994<br>Lperr03g06600.1 0.001 0.291 0.29 0.004 0.996	0<br>AT4G34670.1 0.001 0.283 0.282 0.004 0.966<br>AT5G67540.2 0.502 0.993 0.491 0.053 1
Osind12g10670.1	1<br>Osjap01g31400.1 0.007 0.915 0.908 0.006 0.818<br>Osjap12g13600.1 0.007 0.993 0.986 0.007 0.993	1<br>Osind01g32860.1 0.007 0.915 0.908 0.007 0.854<br>Osind12g10660.1 0.007 0.993 0.986 0.007 0.993<br>Osind12g10670.1 0.007 1 0.993 0.007 1	1<br>Orufi01g30130.1 0.007 0.915 0.908 0.007 0.854	1<br>Oglab01g22960.1 0.071 0.915 0.844 0.194 0.874	1<br>Obart01g27080.1 0.071 0.915 0.844 0.194 0.874<br>Obart12g10510.1 0.007 0.993 0.986 0.012 0.988	0	1<br>Opunc10g03560.1 0.035 0.844 0.809 0.016 0.248	0	0	0
	1<br>Osjap01g05590.1 0.014 0.326 0.312 0.017 0.556<br>Osjap02g24490.1 0.369 0.908 0.539 0.591 0.779	1<br>Osind03g19770.1 0.007 0.411 0.404 0.002 0.412<br>Osind04g15200.1 0.44 0.972 0.532 0.042 0.368	0<br>Orufi12g11520.1 0.007 0.794 0.787 0.008 0.882	1<br>Oglab01g20040.1 0.035 0.34 0.305 0.022 0.11<br>Oglab06g05230.1 0.404 0.915 0.511 0.669 0.816	1<br>Obart01g16250.1 0.411 0.908 0.497 0.765 0.869<br>Obart03g17930.1 0.007 0.411 0.404 0.002 0.126	1<br>Olong_000447-R1 0.39 0.908 0.518 0.691 0.824<br>Olong_003316-R1 0.035 0.34 0.305 0.021 0.103	1<br>Opunc03g17210.1 0.007 0.333 0.326 0.002 0.095<br>Opunc06g05170.1 0.404 0.915 0.511 0.649 0.802	0<br>Obrac01g31700.1 0.184 0.908 0.724 0.829 0.924	1<br>Lperr03g05500.1 0.411 0.915 0.504 0.585 0.769<br>Lperr03g15640.1 0.007 0.333 0.326 0.002 0.09	1<br>AT2G36780.1 0.035 0.333 0.298 0.028 0.113<br>AT5G50380.1 0.355 0.908 0.553 0.706 0.82
Osind12g15880.1	1<br>Osjap12g19810.1 0.002 1 0.998 0.002 1	1<br>Osind12g15880.1 0.002 1 0.998 0.002 1	0	0	0	0	0	0	0	0
	1<br>Osjap07g08090.1 0.146 0.615 0.469 0.642 0.97<br>Osjap12g19770.1 0.659 1 0.341 0.006 1	0<br>Osind04g18390.2 0.13 0.643 0.513 0.648 0.994	1<br>Orufi12g17100.1 0.654 1 0.346 0.081 1<br>Orufi12g17130.1 0.178 0.654 0.476 0.004 0.991	1<br>Oglab06g04730.1 0.146 0.615 0.469 0.655 0.974<br>Oglab12g14370.2 0.654 1 0.346 0.126 1	1<br>Obart03g38270.2 0.146 0.615 0.469 0.61 0.968<br>Obart12g15400.1 0.659 1 0.341 0.006 1	1<br>Olong_001697-R1 0.659 1 0.341 0.006 1<br>Olong_021520-R2 0.139 0.609 0.47 0.684 0.963	1<br>Opunc03g35430.1 0.146 0.615 0.469 0.637 0.97<br>Opunc12g13920.1 0.63 1 0.37 0.003 1	1<br>Obrac06g03630.1 0.126 0.609 0.483 0.588 0.894<br>Obrac12g09830.1 0.652 1 0.348 0.003 1	0<br>Lperr12g12580.2 0.515 1 0.485 0.03 1	0<br>AT5G20250.4 0.146 0.615 0.469 0.681 0.972

(((((_c1,longi),punctata),brachy),leersia),tair);

chimeric gene id	INGROUP					MIDGROUP		OUTGROUP		
	jap	indica	rufi	glab	barthii	longi	punctata	brachy	leersia	tair
Osind01g08490.1	1<br>Osjap01g08660.1 0.001 1 0.999 0.001 1	1<br>Osind01g08490.1 0.001 1 0.999 0.001 1	1<br>Orufi01g08070.1 0.001 1 0.999 0.001 1	1<br>Oglab01g06470.1 0.001 1 0.999 0.001 1	1<br>Obart01g07430.1 0.017 1 0.983 0.001 1	1<br>Olong_011567-R1 0.07 1 0.93 0.001 1	0	0	0	0
	1<br>Osjap01g29230.2 0.114 0.581 0.467 0.25 0.983<br>Osjap05g00560.2 0.647 0.997 0.35 0.146 1	1<br>Osind01g30690.1 0.114 0.581 0.467 0.267 0.982<br>Osind05g00540.1 0.647 0.997 0.35 0.126 1	1<br>Orufi01g27950.1 0.114 0.581 0.467 0.25 0.983<br>Orufi01g48560.2 0.647 0.98 0.333 0.085 0.984	1<br>Oglab01g21070.2 0.114 0.581 0.467 0.249 0.983<br>Oglab05g00530.1 0.647 0.997 0.35 0.146 1	1<br>Obart01g25090.1 0.114 0.581 0.467 0.219 0.982<br>Obart05g00540.1 0.647 0.997 0.35 0.146 1	0<br>Olong_003039-R1 0.647 0.997 0.35 0.128 1<br>Olong_003692-R2 0.153 0.581 0.428 0.244 0.979	1<br>Opunc01g07170.1 0.001 0.582 0.581 0.001 0.984<br>Opunc05g00510.2 0.647 0.997 0.35 0.15 1	1<br>Obrac01g05760.3 0.583 1 0.417 0.082 1<br>Obrac01g05770.3 0.001 0.582 0.581 0.001 0.985	1<br>Lperr01g07030.1 0.583 1 0.417 0.018 1<br>Lperr01g07040.2 0.001 0.582 0.581 0.001 0.989	1<br>AT1G54350.1 0.585 0.993 0.408 0.084 0.975<br>AT5G53430.1 0.024 0.581 0.557 0.09 0.984

Osind01g26900.1	1<br>Osjap01g25870.1 0.001 1 0.999 0.001 0.627	1<br>Osind01g26900.1 0.001 1 0.999 0.001 1	1<br>Orufi01g24680.1 0.006 1 0.994 0.207 1<br>Orufi01g24680.2 0.006 1 0.994 0.198 1	1<br>Oglab01g18120.1 0.006 0.994 0.988 0.149 0.728	0	0	0	0	0	0
	1<br>Osjap07g18880.1 0.033 0.582 0.549 0.391 0.604<br>Osjap11g19730.1 0.642 0.994 0.352 0.269 0.459	1<br>Osind01g26890.1 0.001 0.603 0.602 0.004 0.996<br>Osind11g14800.1 0.624 0.999 0.375 0.108 0.803	0<br>Orufi01g24710.1 0.233 0.517 0.284 0.005 1<br>Orufi11g18390.1 0.625 0.999 0.374 0.001 0.371	1<br>Oglab01g18150.1 0.001 0.517 0.516 0.003 1<br>Oglab11g15370.1 0.625 0.997 0.372 0.001 0.904	1<br>Obart01g21980.1 0.001 0.603 0.602 0.002 0.998<br>Obart11g17430.1 0.606 0.999 0.393 0.007 0.827	0<br>Olong_007897-R1 0.233 0.603 0.37 0.004 0.996<br>Olong_023940-R1 0.611 1 0.389 0.007 0.917	1<br>Opunc01g22160.1 0.001 0.592 0.591 0.002 0.973<br>Opunc10g14020.1 0.62 0.997 0.377 0.003 0.26	0<br>Obrac01g12530.1 0.056 0.481 0.425 0.016 0.79<br>Obrac10g00570.1 0.625 0.999 0.374 0.001 0.328	1<br>Lperr01g18740.1 0.004 0.603 0.599 0.004 0.998<br>Lperr01g18750.1 0.625 1 0.375 0.001 0.278	0<br>AT1G33590.1 0.625 0.996 0.371 0.002 0.551
Osind02g26640.2	1<br>Osjap02g26750.1 0.001 0.953 0.952 0.001 0.993<br>Osjap02g26750.2 0.001 0.953 0.952 0.001 0.993<br>Osjap02g26750.4 0.001 0.953 0.952 0.001 0.799	1<br>Osind02g26640.2 0.001 1 0.999 0.001 1	1<br>Orufi02g24900.1 0.01 0.958 0.948 0.06 0.859<br>Orufi02g24900.3 0.001 0.958 0.957 0.001 0.74<br>Orufi02g24900.4 0.001 0.958 0.957 0.001 0.737	1<br>Oglab02g22130.2 0.001 1 0.999 0.001 1	1<br>Obart02g23460.2 0.001 1 0.999 0.001 1	0	1<br>Opunc02g21410.1 0.01 0.975 0.965 0.116 0.996<br>Opunc02g21410.2 0.01 0.975 0.965 0.114 0.996<br>Opunc02g21410.3 0.01 0.975 0.965 0.115 0.996<br>Opunc02g21410.4 0.01 0.975 0.965 0.114 0.996	0	0	0
	1<br>Osjap02g26590.1 0.497 0.986 0.489 0.033 0.996<br>Osjap02g26750.5 0.01 0.413 0.403 0.238 0.968	1<br>Osind02g26450.1 0.497 0.986 0.489 0.023 0.992<br>Osind02g26620.1 0.01 0.413 0.403 0.495 0.978	1<br>Orufi02g24730.1 0.497 0.986 0.489 0.031 0.996<br>Orufi02g24880.2 0.01 0.413 0.403 0.18 0.964	1<br>Oglab02g21930.1 0.497 1 0.503 0.047 1<br>Oglab02g22110.2 0.01 0.413 0.403 0.532 0.976	1<br>Obart02g23290.1 0.497 1 0.503 0.029 0.997<br>Obart02g23440.1 0.01 0.413 0.403 0.492 0.978	0<br>Olong_012377-R1 0.651 0.957 0.306 0.444 0.915<br>Olong_024345-R1 0.01 0.434 0.424 0.442 0.991	1<br>Opunc02g21230.1 0.504 0.96 0.456 0.003 0.934<br>Opunc02g21240.1 0.01 0.422 0.412 0.121 0.972	1<br>Obrac02g16670.1 0.503 0.957 0.454 0.005 0.995<br>Obrac02g16700.1 0.01 0.422 0.412 0.114 0.947	1<br>Lperr02g18740.1 0.512 0.975 0.463 0.059 0.982<br>Lperr02g19120.1 0.01 0.443 0.433 0.183 0.978	0<br>AT1G47230.2 0.577 0.953 0.376 0.095 0.857<br>AT4G31860.1 0.013 0.411 0.398 0.123 0.989
Osind03g00740.1	1<br>Osjap03g00680.1 0.001 0.996 0.995 0.001 0.994<br>Osjap03g00680.2 0.001 0.996 0.995 0.001 0.994<br>Osjap03g00680.3 0.001 0.996 0.995 0.001 0.994	1<br>Osind03g00740.1 0.001 1 0.999 0.001 1	1<br>Orufi03g12660.1 0.001 0.996 0.995 0.001 0.994<br>Orufi03g12660.2 0.001 0.996 0.995 0.001 0.994	1<br>Oglab03g00390.1 0.001 0.996 0.995 0.001 0.994	1<br>Obart03g00550.1 0.001 1 0.999 0.001 1	0	0	0	0	0
	1<br>Osjap02g42090.1 0.052 0.678 0.626 0.065 1<br>Osjap12g08580.1 0.708 0.996 0.288 0.011 0.977	1<br>Osind02g41670.1 0.062 0.678 0.616 0.131 1<br>Osind03g02220.1 0.698 0.996 0.298 0.015 0.982	1<br>Orufi02g39350.1 0.062 0.678 0.616 0.131 1<br>Orufi09g03230.3 0.732 0.99 0.258 0.053 0.963	1<br>Oglab02g34920.1 0.062 0.678 0.616 0.131 1<br>Oglab03g02050.1 0.698 0.996 0.298 0.214 0.986	1<br>Obart02g37790.1 0.062 0.648 0.586 0.133 0.977<br>Obart12g06510.1 0.676 0.996 0.32 0.052 0.984	0<br>Olong_013175-R1 0.271 0.904 0.633 0.002 0.961	1<br>Opunc03g00470.1 0.001 0.78 0.779 0.002 1<br>Opunc11g04590.1 0.78 0.996 0.216 0.055 0.982	0<br>Obrac02g27970.1 0.137 0.678 0.541 0.105 1<br>Obrac05g17660.1 0.743 0.995 0.252 0.425 0.997	1<br>Lperr03g00570.1 0.001 0.678 0.677 0.002 1<br>Lperr04g11360.1 0.747 0.996 0.249 0.415 0.99	0<br>AT5G04460.1 0.018 0.678 0.66 0.081 1
Osind03g12930.1	1<br>Osjap03g13100.1 0.028 1 0.972 0.001 1	1<br>Osind03g12930.1 0.001 1 0.999 0.001 1	0	1<br>Oglab03g12120.1 0.001 1 0.999 0.001 1	1<br>Obart03g12120.1 0.001 1 0.999 0.001 1	0	0	0	0	0
	1<br>Osjap04g18730.1 0.64 0.998 0.358 0.015 0.863<br>Osjap12g01140.1 0.109 0.633 0.524 0.498 0.981	1<br>Osind06g01770.1 0.642 0.998 0.356 0.212 0.912<br>Osind12g01290.1 0.109 0.633 0.524 0.49 0.981	1<br>Orufi06g01460.3 0.642 0.998 0.356 0.212 0.912<br>Orufi12g01130.1 0.109 0.633 0.524 0.498 0.981	1<br>Oglab06g01620.1 0.642 0.998 0.356 0.233 0.912<br>Oglab12g00800.1 0.114 0.633 0.519 0.483 0.983	1<br>Obart06g01490.1 0.642 0.998 0.356 0.233 0.912<br>Obart11g01060.1 0.114 0.633 0.519 0.471 0.982	1<br>Olong_007714-R9 0.642 1 0.358 0.002 1<br>Olong_007715-R1 0.125 0.633 0.508 0.007 0.965	1<br>Opunc01g28650.1 0.646 0.998 0.352 0.094 0.923<br>Opunc03g11680.1 0.028 0.633 0.605 0.001 0.971	1<br>Obrac03g09720.1 0.025 0.633 0.608 0.036 0.971<br>Obrac04g09430.1 0.64 0.998 0.358 0.014 0.863	1<br>Lperr03g10720.1 0.028 0.633 0.605 0.001 0.934<br>Lperr03g17470.1 0.633 0.998 0.365 0.119 0.828	1<br>AT3G46740.1 0.122 0.633 0.511 0.158 0.971<br>AT3G46790.1 0.648 0.998 0.35 0.068 0.807
Osind03g31500.1	1<br>Osjap03g30460.1 0.025 1 0.975 0.022 1	1<br>Osind03g31500.1 0.001 1 0.999 0.001 1	1<br>Orufi03g29110.1 0.003 1 0.997 0.276 1	0	1<br>Obart03g27980.1 0.089 1 0.911 0.016 1<br>Obart03g27980.2 0.089 1 0.911 0.015 1	0	0	0	0	0
	1<br>Osjap03g30380.1 0.557 0.999 0.442 0.059 0.866<br>Osjap12g24400.2 0.092 0.528 0.436 0.012 0.988	1<br>Osind08g14630.1 0.557 0.999 0.442 0.069 0.96<br>Osind12g20440.1 0.092 0.528 0.436 0.01 0.99	1<br>Orufi03g29970.1 0.557 0.999 0.442 0.035 0.998<br>Orufi12g21550.1 0.092 0.528 0.436 0.01 0.99	1<br>Oglab03g27330.1 0.557 1 0.443 0.036 1<br>Oglab12g17460.4 0.092 0.528 0.436 0.012 0.996	1<br>Obart03g27900.1 0.557 0.999 0.442 0.053 0.865<br>Obart12g19300.1 0.092 0.528 0.436 0.01 0.99	1<br>Olong_002926-R1 0.097 0.529 0.432 0.002 0.996<br>Olong_002936-R1 0.557 0.999 0.442 0.059 0.866	1<br>Opunc03g25470.1 0.01 0.529 0.519 0.001 0.997<br>Opunc06g15370.1 0.557 0.999 0.442 0.035 0.871	0<br>Obrac03g20710.1 0.094 0.529 0.435 0.028 0.997	1<br>Lperr03g23190.1 0.557 0.999 0.442 0.03 0.867<br>Lperr03g23200.1 0.079 0.529 0.45 0.002 0.997	0<br>AT5G36940.1 0.093 0.531 0.438 0.003 0.998
Osind03g35550.1	1	1	1	1	1	0	0	0	0	0

	Osjap03g34330.1 0.039 1 0.961 0.001 1	Osind03g35550.1 0.001 1 0.999 0.001 1	Orufi03g33060.1 0.039 1 0.961 0.001 1	Oglab03g30830.1 0.039 1 0.961 0.001 1	Obart03g31750.1 0.039 1 0.961 0.046 1					
	1<br>Osjap03g39110.1 0.646 1 0.354 0.036 1<br>Osjap10g05370.1 0.026 0.622 0.596 0.051 0.963	1<br>Osind01g46540.1 0.646 0.999 0.353 0.065 0.994<br>Osind10g03770.1 0.026 0.622 0.596 0.057 0.96	1<br>Orufi10g04850.1 0.026 0.622 0.596 0.054 0.963<br>Orufi11g12520.1 0.647 1 0.353 0.038 0.991	1<br>Oglab03g34460.1 0.622 0.997 0.375 0.016 0.993<br>Oglab10g04340.1 0.02 0.622 0.602 0.058 0.963	1<br>Obart03g36200.1 0.646 1 0.354 0.016 0.997<br>Obart10g04600.1 0.051 0.622 0.571 0.106 0.962	1<br>Olong_014796-R1 0.039 0.532 0.493 0.002 0.95<br>Olong_027495-R1 0.622 0.995 0.373 0.005 0.998	1<br>Opunc01g42030.1 0.646 1 0.354 0.28 0.872<br>Opunc10g04080.2 0.033 0.622 0.589 0.084 0.959	1<br>Obrac02g16280.1 0.64 0.981 0.341 0.063 0.949<br>Obrac03g23440.1 0.039 0.622 0.583 0.002 0.964	1<br>Lperr03g30520.1 0.638 1 0.362 0.003 0.997<br>Lperr10g03360.1 0.004 0.622 0.618 0.009 0.962	0<br>AT1G70730.3 0.039 0.622 0.583 0.118 0.967
Osind05g00510.1	1<br>Osjap05g00530.1 0.001 1 0.999 0.131 1<br>Osjap05g00530.2 0.001 1 0.999 0.16 1<br>Osjap05g00530.3 0.001 1 0.999 0.161 1<br>Osjap05g00530.4 0.001 1 0.999 0.142 1	1<br>Osind05g00510.1 0.001 1 0.999 0.001 1	1<br>Orufi05g00500.1 0.001 1 0.999 0.131 1	0	1<br>Obart05g00520.1 0.001 0.999 0.998 0.049 0.993<br>Obart05g00520.2 0.001 1 0.999 0.049 1	1<br>Olong_022405-R1 0.001 1 0.999 0.001 1	0	0	0	0
	0<br>Osjap02g42390.1 0.326 0.993 0.667 0.001 0.983	1<br>Osind01g11620.1 0.002 0.275 0.273 0.271 0.988<br>Osind09g15830.1 0.31 1 0.69 0.029 1	1<br>Orufi07g24760.1 0.001 0.258 0.257 0.267 0.834<br>Orufi09g16480.1 0.31 1 0.69 0.029 1	1<br>Oglab05g00500.1 0.001 0.26 0.259 0.344 0.982<br>Oglab09g13820.1 0.291 1 0.709 0.064 1	1<br>Obart01g04950.1 0.001 0.258 0.257 0.193 0.858<br>Obart07g24200.1 0.302 0.998 0.696 0.005 0.907	1<br>Olong_004108-R1 0.004 0.275 0.271 0.098 0.928<br>Olong_023813-R1 0.303 0.997 0.694 0.001 0.979	1<br>Opunc05g00480.1 0.298 0.999 0.701 0.007 0.998<br>Opunc05g00490.1 0.001 0.26 0.259 0.352 0.982	1<br>Obrac02g14170.4 0.004 0.267 0.263 0.164 0.843<br>Obrac03g06070.3 0.31 0.998 0.688 0.023 0.996	1<br>Lperr02g16220.1 0.004 0.267 0.263 0.238 0.857<br>Lperr05g00500.1 0.301 1 0.699 0.009 1	0<br>AT4G14820.1 0.001 0.267 0.266 0.107 0.878
Osind05g04070.1	1<br>Osjap05g04120.1 0.001 1 0.999 0.008 1<br>Osjap05g04120.2 0.001 1 0.999 0.008 1	1<br>Osind05g04070.1 0.001 1 0.999 0.001 1<br>Osind05g04070.2 0.001 1 0.999 0.001 1	1<br>Orufi05g03950.1 0.001 1 0.999 0.008 1<br>Orufi05g03950.2 0.001 1 0.999 0.008 1	1<br>Oglab05g03490.1 0.001 1 0.999 0.008 1<br>Oglab05g03490.2 0.001 1 0.999 0.008 1	1<br>Obart05g03830.1 0.001 1 0.999 0.008 1<br>Obart05g03830.2 0.001 1 0.999 0.008 1	1<br>Olong_016739-R1 0.069 1 0.931 0.001 1	1<br>Opunc05g03540.1 0.001 1 0.999 0.008 1<br>Opunc05g03540.2 0.001 1 0.999 0.008 1	1<br>Obrac05g02930.1 0.069 1 0.931 0.001 1	0	0
	1<br>Osjap01g27410.1 0.086 0.735 0.649 0.066 0.992<br>Osjap03g15140.2 0.738 1 0.262 0.01 0.192	1<br>Osind01g28630.1 0.086 0.735 0.649 0.066 0.992<br>Osind03g15230.1 0.783 1 0.217 0.03 1	1<br>Orufi01g26130.1 0.086 0.735 0.649 0.066 0.992<br>Orufi03g14720.1 0.783 1 0.217 0.03 1	1<br>Oglab01g19490.1 0.086 0.735 0.649 0.066 0.992<br>Oglab03g14060.1 0.783 1 0.217 0.03 1	1<br>Obart01g23310.1 0.086 0.735 0.649 0.066 0.992<br>Obart03g14050.1 0.783 1 0.217 0.03 1	1<br>Olong_003347-R2 0.086 0.735 0.649 0.047 0.992<br>Olong_009943-R1 0.783 1 0.217 0.03 1	1<br>Opunc01g23460.1 0.086 0.735 0.649 0.064 0.991<br>Opunc03g13590.1 0.783 1 0.217 0.002 0.545	1<br>Obrac01g17590.1 0.086 0.735 0.649 0.067 0.991<br>Obrac03g11330.1 0.783 1 0.217 0.03 1	0<br>Lperr05g03460.1 0.001 0.736 0.735 0.011 0.994	1<br>AT2G47580.1 0.768 1 0.232 0.008 1<br>AT3G16630.1 0.001 0.736 0.735 0.039 0.994
Osind05g04070.2	1<br>Osjap05g04120.1 0.001 1 0.999 0.008 1<br>Osjap05g04120.2 0.001 1 0.999 0.008 1	1<br>Osind05g04070.1 0.001 1 0.999 0.001 1<br>Osind05g04070.2 0.001 1 0.999 0.001 1	1<br>Orufi05g03950.1 0.001 1 0.999 0.008 1<br>Orufi05g03950.2 0.001 1 0.999 0.008 1	1<br>Oglab05g03490.1 0.001 1 0.999 0.008 1<br>Oglab05g03490.2 0.001 1 0.999 0.008 1	1<br>Obart05g03830.1 0.001 1 0.999 0.008 1<br>Obart05g03830.2 0.001 1 0.999 0.008 1	1<br>Olong_016739-R1 0.068 1 0.932 0.001 1	1<br>Opunc05g03540.1 0.001 1 0.999 0.008 1<br>Opunc05g03540.2 0.001 1 0.999 0.008 1	1<br>Obrac05g02930.1 0.068 1 0.932 0.001 1	0	0
	1<br>Osjap01g27410.1 0.084 0.738 0.654 0.066 0.992<br>Osjap03g15140.2 0.742 1 0.258 0.01 0.192	1<br>Osind01g28630.1 0.084 0.738 0.654 0.066 0.992<br>Osind03g15230.1 0.786 1 0.214 0.03 1	1<br>Orufi01g26130.1 0.084 0.738 0.654 0.066 0.992<br>Orufi03g14720.1 0.786 1 0.214 0.03 1	1<br>Oglab01g19490.1 0.084 0.738 0.654 0.066 0.992<br>Oglab03g14060.1 0.786 1 0.214 0.03 1	1<br>Obart01g23310.1 0.084 0.738 0.654 0.066 0.992<br>Obart03g14050.1 0.786 1 0.214 0.03 1	1<br>Olong_003347-R2 0.084 0.738 0.654 0.047 0.992<br>Olong_009943-R1 0.786 1 0.214 0.03 1	1<br>Opunc01g23460.1 0.084 0.738 0.654 0.064 0.991<br>Opunc03g13590.1 0.786 1 0.214 0.002 0.545	1<br>Obrac01g17590.1 0.084 0.738 0.654 0.067 0.991<br>Obrac03g11330.1 0.786 1 0.214 0.03 1	0<br>Lperr05g03460.1 0.001 0.74 0.739 0.011 0.994	1<br>AT2G47580.1 0.771 1 0.229 0.008 1<br>AT3G16630.1 0.001 0.74 0.739 0.039 0.994
Osind05g28520.1	1<br>Osjap05g28230.1 0.001 1 0.999 0.001 1	1<br>Osind05g28520.1 0.001 1 0.999 0.001 1	1<br>Orufi05g26870.1 0.001 1 0.999 0.001 1	1<br>Oglab05g23160.1 0.001 1 0.999 0.001 1	1<br>Obart05g25070.1 0.001 1 0.999 0.001 1	0	1<br>Opunc05g22640.1 0.001 1 0.999 0.001 1	0	1<br>Lperr01g22990.1 0.001 1 0.999 0.027 1	0
	1<br>Osjap01g31140.1 0.006 0.43 0.424 0.093 0.995<br>Osjap01g31150.1 0.486 0.999 0.513 0.009 0.999	1<br>Osind01g32600.1 0.001 0.43 0.429 0.002 0.995<br>Osind01g32610.1 0.486 0.999 0.513 0.009 0.999	1<br>Orufi01g29880.1 0.001 0.43 0.429 0.075 0.995<br>Orufi01g29890.1 0.486 0.999 0.513 0.009 0.999	1<br>Oglab01g22740.1 0.091 0.431 0.34 0.213 0.996<br>Oglab01g22750.1 0.486 0.999 0.513 0.009 0.999	1<br>Obart01g26870.1 0.005 0.43 0.425 0.117 0.995<br>Obart01g26880.1 0.486 0.999 0.513 0.009 0.999	1<br>Olong_000367-R1 0.1 0.431 0.331 0.002 0.995<br>Olong_000368-R1 0.461 0.999 0.538 0.069 0.999	1<br>Opunc01g26820.1 0.091 0.431 0.34 0.118 0.996<br>Opunc01g26830.1 0.486 1 0.514 0.009 1	1<br>Obrac01g20400.1 0.091 0.431 0.34 0.125 0.996<br>Obrac05g17560.2 0.431 1 0.569 0.109 1	1<br>Lperr05g20850.2 0.491 1 0.509 0.002 1<br>Lperr05g20860.1 0.091 0.431 0.34 0.215 0.996	1<br>AT5G08680.1 0.022 0.43 0.408 0.064 0.995<br>AT5G51600.1 0.486 0.999 0.513 0.008 0.996
Osind07g21110.1	1<br>Osjap01g22010.1 0.107 0.994 0.887 0.18 0.603<br>Osjap05g18970.1 0.136 1 0.864 0.019 0.4<br>Osjap06g16240.1 0.006 1 0.994 0.009 0.498<br>Osjap10g11020.1 0.006 1 0.994 0.059 0.471	1<br>Osind02g13760.1 0.006 1 0.994 0.117 0.528<br>Osind07g21110.1 0.006 1 0.994 0.006 1	1<br>Orufi01g20970.1 0.107 0.994 0.887 0.18 0.603<br>Orufi06g15090.1 0.006 0.972 0.966 0.011 0.59	1<br>Oglab06g11250.1 0.006 0.994 0.988 0.265 0.612<br>Oglab06g13690.1 0.006 1 0.994 0.011 0.604<br>Oglab10g08950.1 0.006 1 0.994 0.08 0.631	1<br>Obart06g14180.1 0.006 1 0.994 0.019 0.924	0	0	0	1<br>Lperr02g15640.1 0.006 0.91 0.904 0.002 0.429<br>Lperr04g04630.1 0.006 0.989 0.983 0.002 0.542<br>Lperr07g12330.1 0.017 0.864 0.847 0.009 0.912	0
	1	1	1	0	1	0	1	0	0	0

	Osjap02g04690.1 0.006 0.237 0.231 0.045 0.511<br>Osjap07g27990.1 0.243 1 0.757 0.144 0.295	Osind02g04960.1 0.006 0.237 0.231 0.415 0.748<br>Osind07g24640.1 0.243 1 0.757 0.202 0.414	Orufi01g02310.1 0.243 1 0.757 0.185 0.366<br>Orufi07g21730.1 0.006 0.237 0.231 0.051 0.577	Oglab07g03540.1 0.243 1 0.757 0.201 0.412	Obart06g11400.1 0.006 0.237 0.231 0.396 0.681<br>Obart07g03670.1 0.243 1 0.757 0.201 0.412	Olong_018765-R1 0.22 1 0.78 0.09 0.844	Opunc07g02910.1 0.249 1 0.751 0.37 0.949<br>Opunc10g08340.1 0.006 0.215 0.209 0.015 0.157	Obrac07g08420.1 0.288 0.492 0.204 0.184 0.291	Lperr03g29540.1 0.017 0.78 0.763 0.005 0.272	
Osind07g27670.1	1<br>Osjap07g31020.1 0.008 1 0.992 0.404 1	1<br>Osind07g27670.1 0.008 1 0.992 0.008 1<br>Osind07g27730.1 0.008 1 0.992 0.404 1	0	1<br>Oglab07g23640.1 0.016 1 0.984 0.801 0.998<br>Oglab07g23640.2 0.016 1 0.984 0.813 0.998	1<br>Obart07g27040.1 0.016 1 0.984 0.144 0.32<br>Obart07g27040.2 0.016 1 0.984 0.118 0.299	0	0	0	0	0
	1<br>Osjap03g15620.2 0.73 1 0.27 0.934 0.97<br>Osjap07g30980.1 0.008 0.639 0.631 0.199 0.358	1<br>Osind07g27700.1 0.008 0.664 0.656 0.124 0.258<br>Osind07g27740.1 0.73 1 0.27 0.939 0.972	1<br>Orufi03g15210.1 0.73 1 0.27 0.939 0.972<br>Orufi07g27980.1 0.008 0.664 0.656 0.221 0.34	1<br>Oglab03g14540.1 0.73 1 0.27 0.938 0.971<br>Oglab07g23620.1 0.008 0.664 0.656 0.124 0.258	1<br>Obart07g27030.1 0.008 0.664 0.656 0.425 0.51<br>Obart07g27050.1 0.73 1 0.27 0.939 0.972	1<br>Olong_008697-R2 0.73 1 0.27 0.936 0.971<br>Olong_008701-R1 0.008 0.664 0.656 0.124 0.258	1<br>Opunc07g25410.1 0.008 0.664 0.656 0.129 0.268<br>Opunc07g25450.1 0.73 1 0.27 0.937 0.971	1<br>Obrac03g11770.2 0.73 1 0.27 0.931 0.968<br>Obrac07g18100.1 0.008 0.664 0.656 0.131 0.271	1<br>Lperr03g12860.1 0.73 1 0.27 0.937 0.971<br>Lperr07g23810.1 0.008 0.664 0.656 0.123 0.256	0<br>AT3G66658.2 0.008 0.754 0.746 0.124 0.273
Osind08g06790.1	1<br>Osjap08g06650.1 0.002 1 0.998 0.002 1	1<br>Osind08g06790.1 0.002 1 0.998 0.002 1	1<br>Orufi08g06840.1 0.002 1 0.998 0.002 1	1<br>Oglab08g05640.1 0.002 1 0.998 0.002 1	1<br>Obart08g06070.1 0.002 1 0.998 0.002 1	0	1<br>Opunc08g05850.1 0.047 1 0.953 0.068 1	0	0	0
	1<br>Osjap04g21220.1 0.047 0.275 0.228 0.21 0.936<br>Osjap09g20870.1 0.359 0.984 0.625 0.208 0.966	1<br>Osind04g20250.1 0.047 0.275 0.228 0.198 0.94<br>Osind09g20960.1 0.359 0.984 0.625 0.208 0.966	1<br>Orufi04g19250.2 0.047 0.275 0.228 0.21 0.936<br>Orufi09g21960.1 0.359 0.984 0.625 0.2 0.965	1<br>Oglab02g26360.1 0.348 0.996 0.648 0.01 0.994<br>Oglab04g16180.1 0.047 0.275 0.228 0.198 0.94	1<br>Obart02g28440.1 0.348 0.996 0.648 0.093 0.994<br>Obart04g17770.1 0.047 0.275 0.228 0.198 0.94	1<br>Olong_008030-R1 0.047 0.275 0.228 0.232 0.937<br>Olong_008031-R2 0.277 1 0.723 0.106 1	1<br>Opunc04g15550.1 0.047 0.275 0.228 0.206 0.938<br>Opunc04g18740.1 0.295 0.984 0.689 0.088 0.994	1<br>Obrac08g04780.1 0.277 1 0.723 0.098 1<br>Obrac08g04800.1 0.002 0.275 0.273 0.006 0.943	1<br>Lperr08g05690.1 0.277 1 0.723 0.1 1<br>Lperr08g05700.1 0.002 0.275 0.273 0.006 0.943	1<br>AT1G05310.1 0.33 0.982 0.652 0.183 0.99<br>AT4G30800.1 0.047 0.275 0.228 0.208 0.943
Osind08g23660.1	1<br>Osjap06g14410.1 0.004 0.824 0.82 0.003 0.972<br>Osjap06g14410.2 0.004 0.828 0.824 0.004 0.975<br>Osjap06g14410.3 0.004 0.824 0.82 0.004 0.969<br>Osjap06g14410.4 0.004 0.824 0.82 0.004 0.97	1<br>Osind06g14230.1 0.004 0.824 0.82 0.003 0.972<br>Osind08g23660.1 0.004 1 0.996 0.004 1	1<br>Orufi06g13260.1 0.004 0.824 0.82 0.003 0.972<br>Orufi08g22580.1 0.004 1 0.996 0.004 1<br>Orufi08g22580.2 0.004 1 0.996 0.004 1	1<br>Oglab06g12260.1 0.004 0.941 0.937 0.004 0.913	1<br>Obart06g12530.1 0.105 0.941 0.836 0.173 0.937<br>Obart08g20150.1 0.004 1 0.996 0.005 1	0	0	0	0	0
	1<br>Osjap03g19840.3 0.004 0.371 0.367 0.028 0.331<br>Osjap03g44720.1 0.488 0.957 0.469 0.561 0.813	1<br>Osind03g45770.1 0.488 0.957 0.469 0.496 0.785<br>Osind07g24350.1 0.004 0.371 0.367 0.013 0.297	1<br>Orufi03g42970.1 0.488 0.957 0.469 0.503 0.788<br>Orufi07g25160.1 0.004 0.371 0.367 0.013 0.291	1<br>Oglab03g39140.1 0.488 0.957 0.469 0.561 0.813<br>Oglab07g20500.1 0.004 0.371 0.367 0.013 0.297	0<br>Obart03g41170.1 0.488 0.957 0.469 0.479 0.778	0<br>Olong_013371-R1 0.488 0.957 0.469 0.357 0.726	1<br>Opunc03g17540.4 0.004 0.391 0.387 0.019 0.333<br>Opunc03g38260.1 0.488 0.957 0.469 0.351 0.723	1<br>Obrac03g31000.1 0.488 0.922 0.434 0.483 0.759<br>Obrac07g15830.2 0.004 0.371 0.367 0.014 0.273	0<br>Lperr03g35120.1 0.488 0.922 0.434 0.449 0.742	0<br>AT5G50970.1 0.465 0.957 0.492 0.443 0.768
Osind09g09040.1	1<br>Osjap11g06710.1 0.001 1 0.999 0.067 0.999<br>Osjap11g06720.1 0.001 0.999 0.998 0.054 0.981	1<br>Osind09g09040.1 0.001 1 0.999 0.001 1	1<br>Orufi11g05930.1 0.001 1 0.999 0.09 1<br>Orufi11g05940.1 0.001 1 0.999 0.002 1	1<br>Oglab11g05440.1 0.001 0.995 0.994 0.001 0.998	1<br>Obart11g05910.1 0.001 0.88 0.879 0.05 1	0	0	0	0	0
	1<br>Osjap01g24690.1 0.525 0.989 0.464 0.014 0.934<br>Osjap11g06740.8 0.022 0.484 0.462 0.013 0.907	1<br>Osind09g09050.1 0.001 0.481 0.48 0.104 0.994<br>Osind11g05650.1 0.522 1 0.478 0.002 1	1<br>Orufi11g05890.1 0.522 1 0.478 0.003 0.997<br>Orufi11g05900.2 0.001 0.481 0.48 0.002 0.978	1<br>Oglab08g10820.1 0.522 0.955 0.433 0.002 0.867<br>Oglab11g05430.1 0.001 0.503 0.502 0.049 0.944	1<br>Obart01g20930.1 0.525 0.989 0.464 0.006 0.498<br>Obart11g05930.1 0.001 0.481 0.48 0.002 0.998	1<br>Olong_000002-R1 0.001 0.517 0.516 0.117 0.996<br>Olong_005787-R1 0.522 1 0.478 0.002 1	0<br>Opunc11g05960.1 0.425 1 0.575 0.002 1	1<br>Obrac01g15800.1 0.521 0.989 0.468 0.004 0.915<br>Obrac11g03930.1 0.001 0.482 0.481 0.002 0.939	1<br>Lperr05g13220.1 0.522 1 0.478 0.002 1<br>Lperr11g05360.1 0.001 0.481 0.48 0.044 0.995	0<br>AT4G38130.1 0.522 0.955 0.433 0.002 0.782
Osind09g15570.1	1<br>Osjap09g15430.2 0.002 1 0.998 0.002 1<br>Osjap09g15430.3 0.002 1 0.998 0.002 1	1<br>Osind09g15570.1 0.002 1 0.998 0.002 1<br>Osind09g15570.2 0.002 1 0.998 0.002 1	1<br>Orufi09g16160.1 0.002 1 0.998 0.002 1<br>Orufi09g16160.2 0.002 1 0.998 0.002 1	1<br>Oglab09g13510.1 0.002 1 0.998 0.002 1<br>Oglab09g13510.2 0.002 1 0.998 0.002 1<br>Oglab09g13510.3 0.002 1 0.998 0.002 1<br>Oglab09g13510.4 0.002 1 0.998 0.002 1	1<br>Obart09g14980.1 0.002 1 0.998 0.002 1<br>Obart09g14980.2 0.002 1 0.998 0.002 1	0	1<br>Opunc09g13420.1 0.002 1 0.998 0.002 1<br>Opunc09g13420.2 0.002 1 0.998 0.002 1	1<br>Obrac09g09090.1 0.002 1 0.998 0.002 1	0	0
	1<br>Osjap02g06650.1 0.01 0.54 0.53 0.01 0.997<br>Osjap09g15430.1 0.544 1 0.456 0.131 1	1<br>Osind02g06940.1 0.01 0.54 0.53 0.01 0.997<br>Osind08g23920.1 0.544 0.993 0.449 0.131 0.988	1<br>Orufi04g24220.1 0.013 0.542 0.529 0.165 1<br>Orufi08g22880.1 0.544 0.993 0.449 0.131 0.988	1<br>Oglab08g19240.1 0.544 0.993 0.449 0.143 0.986<br>Oglab12g18680.1 0.002 0.542 0.54 0.003 1	1<br>Obart02g06180.1 0.01 0.54 0.53 0.01 0.997<br>Obart08g20390.1 0.544 0.993 0.449 0.631 0.988	1<br>Olong_013054-R1 0.547 0.992 0.445 0.149 0.979<br>Olong_024186-R1 0.002 0.542 0.54 0.003 1	1<br>Opunc04g20190.1 0.007 0.542 0.535 0.006 1<br>Opunc08g18560.1 0.544 0.993 0.449 0.128 0.969	1<br>Obrac02g04550.3 0.008 0.54 0.532 0.214 0.997<br>Obrac09g00560.1 0.548 0.998 0.45 0.164 0.966	1<br>Lperr04g18350.1 0.002 0.542 0.54 0.021 1<br>Lperr09g11120.1 0.544 1 0.456 0.156 1	1<br>AT1G68540.1 0.542 0.992 0.45 0.112 1<br>AT2G21270.3 0.003 0.54 0.537 0.059 0.997

Osind09g15570.2	1<br>Osjap09g15430.2 0.002 1 0.998 0.002 1<br>Osjap09g15430.3 0.002 1 0.998 0.002 1	1<br>Osind09g15570.1 0.002 1 0.998 0.002 1<br>Osind09g15570.2 0.002 1 0.998 0.002 1	1<br>Orufi09g16160.1 0.002 1 0.998 0.002 1<br>Orufi09g16160.2 0.002 1 0.998 0.002 1	1<br>Oglab09g13510.1 0.002 1 0.998 0.002 1<br>Oglab09g13510.2 0.002 1 0.998 0.002 1<br>Oglab09g13510.3 0.002 1 0.998 0.002 1<br>Oglab09g13510.4 0.002 1 0.998 0.002 1	1<br>Obart09g14980.1 0.002 1 0.998 0.002 1<br>Obart09g14980.2 0.002 1 0.998 0.002 1	0	1<br>Opunc09g13420.1 0.002 1 0.998 0.002 1<br>Opunc09g13420.2 0.002 1 0.998 0.002 1	1<br>Obrac09g09090.1 0.002 1 0.998 0.002 1	0	0
	1<br>Osjap02g06650.1 0.01 0.565 0.555 0.01 0.997<br>Osjap09g15430.1 0.569 1 0.431 0.131 1	1<br>Osind02g06940.1 0.01 0.565 0.555 0.01 0.997<br>Osind08g23920.1 0.569 0.993 0.424 0.131 0.988	1<br>Orufi04g24220.1 0.014 0.567 0.553 0.165 1<br>Orufi08g22880.1 0.569 0.993 0.424 0.131 0.988	1<br>Oglab08g19240.1 0.569 0.993 0.424 0.143 0.986<br>Oglab12g18680.1 0.002 0.567 0.565 0.003 1	1<br>Obart02g06180.1 0.01 0.565 0.555 0.01 0.997<br>Obart08g20390.1 0.569 0.993 0.424 0.631 0.988	1<br>Olong_008093-R2 0.596 0.991 0.395 0.25 0.996<br>Olong_024186-R1 0.002 0.567 0.565 0.003 1	1<br>Opunc04g20190.1 0.007 0.567 0.56 0.006 1<br>Opunc08g18560.1 0.569 0.993 0.424 0.128 0.969	1<br>Obrac01g09090.1 0.605 0.998 0.393 0.299 0.982<br>Obrac02g04550.3 0.009 0.565 0.556 0.214 0.997	1<br>Lperr04g18350.1 0.002 0.567 0.565 0.021 1<br>Lperr09g11120.1 0.569 1 0.431 0.156 1	1<br>AT1G68540.1 0.567 0.991 0.424 0.112 1<br>AT2G21270.3 0.003 0.565 0.562 0.059 0.997
Osind09g15930.1	0	1<br>Osind09g15930.1 0.001 1 0.999 0.001 1	1<br>Orufi09g16580.1 0.001 1 0.999 0.061 1	1<br>Oglab09g13920.1 0.001 1 0.999 0.061 1	1<br>Obart09g15360.1 0.057 1 0.943 0.001 1	0	0	0	0	0
	1<br>Osjap03g04840.3 0.544 0.887 0.343 0.018 0.769<br>Osjap09g15790.1 0.001 0.544 0.543 0.155 0.993	1<br>Osind08g24670.1 0.004 0.544 0.54 0.138 0.995<br>Osind09g02290.1 0.544 0.887 0.343 0.018 0.769	1<br>Orufi03g04550.5 0.544 0.887 0.343 0.017 0.733<br>Orufi08g23480.1 0.004 0.544 0.54 0.146 0.995	1<br>Oglab02g15910.1 0.037 0.512 0.475 0.001 0.988<br>Oglab09g02290.1 0.544 0.887 0.343 0.02 0.789	1<br>Obart03g04820.1 0.569 0.887 0.318 0.1 0.796<br>Obart08g21020.1 0.056 0.544 0.488 0.086 0.994	1<br>Olong_025005-R2 0.571 0.98 0.409 0.001 0.955<br>Olong_025006-R1 0.105 0.544 0.439 0.031 0.99	1<br>Opunc08g19030.2 0.006 0.544 0.538 0.071 0.976<br>Opunc09g13790.1 0.544 0.98 0.436 0.055 0.958	1<br>Obrac08g14010.2 0.036 0.544 0.508 0.001 0.989<br>Obrac09g09380.2 0.544 0.98 0.436 0.01 0.954	1<br>Lperr09g11500.1 0.001 0.544 0.543 0.105 0.992<br>Lperr09g11510.2 0.544 0.98 0.436 0.01 0.958	1<br>AT2G32000.1 0.544 0.978 0.434 0.012 0.961<br>AT2G43900.1 0.002 0.519 0.517 0.083 0.843
Osind09g20560.1	1<br>Osjap09g20460.1 0.001 0.998 0.997 0.001 0.986	1<br>Osind09g20560.1 0.001 1 0.999 0.001 1	1<br>Orufi09g21510.1 0.001 0.978 0.977 0.001 0.999	1<br>Oglab09g18200.1 0.001 0.998 0.997 0.001 0.985	1<br>Obart09g19980.1 0.067 0.998 0.931 0.165 0.99<br>Obart09g19980.2 0.001 0.998 0.997 0.001 0.984	1<br>Olong_014589-R1 0.117 0.985 0.868 0.018 0.991<br>Olong_014589-R2 0.117 0.985 0.868 0.018 0.991	0	0	0	0
	1<br>Osjap02g21530.2 0.024 0.581 0.557 0.096 0.943<br>Osjap09g20430.1 0.623 0.998 0.375 0.085 0.962	1<br>Osind02g21430.2 0.024 0.581 0.557 0.096 0.943<br>Osind09g20550.1 0.623 0.998 0.375 0.032 0.982	1<br>Orufi02g19810.2 0.024 0.581 0.557 0.096 0.943<br>Orufi09g21500.1 0.623 0.998 0.375 0.085 0.962	1<br>Oglab02g17850.1 0.024 0.581 0.557 0.095 0.914<br>Oglab09g18170.1 0.623 0.998 0.375 0.079 0.966	1<br>Obart04g25370.1 0.047 0.589 0.542 0.176 0.967<br>Obart09g19950.1 0.623 0.998 0.375 0.079 0.966	1<br>Olong_014116-R1 0.064 0.589 0.525 0.069 0.957<br>Olong_027417-R1 0.625 0.982 0.357 0.135 0.711	1<br>Opunc09g18280.1 0.618 0.998 0.38 0.058 0.841<br>Opunc09g18290.2 0.001 0.59 0.589 0.001 0.97	1<br>Obrac09g13140.3 0.623 0.998 0.375 0.049 0.806<br>Obrac09g13160.3 0.001 0.596 0.595 0.001 0.996	1<br>Lperr09g15890.1 0.622 0.999 0.377 0.048 0.95<br>Lperr09g15920.1 0.001 0.59 0.589 0.001 0.957	0<br>AT1G18160.1 0.021 0.589 0.568 0.064 0.958
Osind10g19230.1	0	1<br>Osind10g19230.1 0.002 1 0.998 0.002 1	1<br>Orufi10g20960.1 0.002 1 0.998 0.002 1	1<br>Oglab10g16090.1 0.002 0.992 0.99 0.001 0.861	1<br>Obart10g19720.1 0.002 1 0.998 0.002 1	0	1<br>Opunc10g18180.1 0.002 0.992 0.99 0.002 0.85	0	0	0
	1<br>Osjap10g22750.1 0.509 0.992 0.483 0.061 0.836<br>Osjap10g22760.1 0.002 0.486 0.484 0.003 0.993	1<br>Osind01g20890.1 0.519 0.992 0.473 0.154 0.668<br>Osind08g03990.1 0.046 0.464 0.418 0.057 0.95	1<br>Orufi01g19490.1 0.519 0.992 0.473 0.171 0.72<br>Orufi08g04140.1 0.046 0.464 0.418 0.057 0.95	1<br>Oglab01g14760.1 0.519 0.992 0.473 0.2 0.827<br>Oglab08g03250.2 0.046 0.464 0.418 0.052 0.955	1<br>Obart01g17550.1 0.519 0.992 0.473 0.174 0.721<br>Obart08g03750.1 0.046 0.464 0.418 0.057 0.95	1<br>Olong_006312-R1 0.516 0.986 0.47 0.067 0.835<br>Olong_006313-R1 0.002 0.486 0.484 0.003 0.993	1<br>Opunc01g18040.1 0.519 0.992 0.473 0.174 0.822<br>Opunc08g03700.1 0.017 0.464 0.447 0.011 0.951	1<br>Obrac01g13330.1 0.525 0.992 0.467 0.196 0.825<br>Obrac10g13120.1 0.002 0.486 0.484 0.004 0.993	1<br>Lperr10g15590.1 0.516 0.992 0.476 0.07 0.79<br>Lperr10g15600.1 0.002 0.482 0.48 0.003 0.979	1<br>AT1G32080.1 0.521 0.988 0.467 0.188 0.82<br>AT2G32260.1 0.044 0.465 0.421 0.078 0.825
Osind11g00690.1	1<br>Osjap11g01220.1 0.001 0.999 0.998 0.001 0.955<br>Osjap12g01140.1 0.001 0.999 0.998 0.001 0.953	1<br>Osind11g00690.1 0.001 1 0.999 0.001 1<br>Osind12g01290.1 0.001 0.999 0.998 0.001 0.954	1<br>Orufi11g01000.1 0.001 0.999 0.998 0.001 0.951<br>Orufi12g01130.1 0.001 0.999 0.998 0.001 0.953	1<br>Oglab12g00800.1 0.001 0.999 0.998 0.001 0.956	1<br>Obart11g01060.1 0.001 0.999 0.998 0.001 0.954	0	1<br>Opunc12g00940.1 0.001 0.999 0.998 0.001 0.937	0	0	0
	1<br>Osjap03g13100.1 0.523 0.999 0.476 0.097 0.593<br>Osjap07g30390.1 0.01 0.462 0.452 0.013 0.944	1<br>Osind03g12930.1 0.523 0.999 0.476 0.121 0.604<br>Osind07g27070.1 0.007 0.462 0.455 0.006 0.946	1<br>Orufi03g17220.1 0.53 0.999 0.469 0.027 0.912<br>Orufi07g27420.1 0.01 0.462 0.452 0.013 0.946	1<br>Oglab03g12120.1 0.523 0.999 0.476 0.121 0.604<br>Oglab07g22980.1 0.007 0.462 0.455 0.007 0.94	1<br>Obart03g12120.1 0.523 0.999 0.476 0.121 0.604<br>Obart12g00790.1 0.001 0.462 0.461 0.002 0.949	1<br>Olong_007715-R1 0.527 0.999 0.472 0.009 0.911<br>Olong_008211-R1 0.007 0.462 0.455 0.006 0.946	1<br>Opunc03g11680.1 0.523 0.999 0.476 0.148 0.924<br>Opunc11g00920.1 0.001 0.462 0.461 0.142 0.951	1<br>Obrac03g09720.2 0.523 0.999 0.476 0.142 0.923<br>Obrac12g00780.1 0.001 0.462 0.461 0.11 0.953	0<br>Lperr11g00920.1 0.001 0.462 0.461 0.002 0.949	1<br>AT2G34940.1 0.001 0.462 0.461 0.002 0.955<br>AT3G46740.1 0.526 1 0.474 0.161 0.928
Osind11g01990.1	1<br>Osjap11g02640.1 0.002 1 0.998 0.002 1<br>Osjap12g02610.1 0.002 1 0.998 0.002 1	1<br>Osind11g01990.1 0.002 1 0.998 0.002 1<br>Osind12g02780.1 0.002 1 0.998 0.002 1	1<br>Orufi11g02320.1 0.002 1 0.998 0.013 1<br>Orufi12g02480.1 0.002 1 0.998 0.002 1	1<br>Oglab11g02200.1 0.002 1 0.998 0.002 1<br>Oglab12g02130.1 0.002 1 0.998 0.002 1	1<br>Obart12g02080.1 0.023 1 0.977 0.002 1	0	0	0	0	0

	1<br>Osjap07g04360.1 0.033 0.669 0.636 0.018 0.925<br>Osjap12g12870.1 0.673 0.949 0.276 0.055 0.417	1<br>Osind11g02010.1 0.014 0.669 0.655 0.001 0.994<br>Osind12g10020.1 0.673 0.949 0.276 0.055 0.417	1<br>Orufi12g02470.1 0.045 0.669 0.624 0.04 0.988<br>Orufi12g10750.1 0.671 0.949 0.278 0.045 0.364	1<br>Oglab07g03810.1 0.033 0.669 0.636 0.019 0.987<br>Oglab12g09880.1 0.673 0.949 0.276 0.051 0.385	0<br>Obart11g02350.1 0.021 0.669 0.648 0.024 0.993	1<br>Olong_012584-R1 0.066 0.622 0.556 0.002 0.965<br>Olong_017316-R1 0.671 0.949 0.278 0.045 0.365	1<br>Opunc11g02190.1 0.002 0.669 0.667 0.003 0.989<br>Opunc12g08740.1 0.671 0.949 0.278 0.04 0.324	1<br>Obrac05g10860.1 0.051 0.669 0.618 0.097 0.987<br>Obrac12g07170.1 0.671 0.94 0.269 0.071 0.57	1<br>Lperr12g02190.1 0.002 0.669 0.667 0.003 0.989<br>Lperr12g08370.1 0.669 0.949 0.28 0.047 0.391	0<br>AT5G27320.1 0.014 0.643 0.629 0.003 0.98
Osind11g09560.1	1<br>Osjap11g12460.1 0.009 0.896 0.887 0.338 0.846	1<br>Osind11g09560.1 0.009 1 0.991 0.009 1	1<br>Orufi11g11390.1 0.009 0.896 0.887 0.338 0.846	1<br>Oglab11g09700.1 0.009 0.896 0.887 0.34 0.85	1<br>Obart11g10770.1 0.009 0.896 0.887 0.29 0.839	0	0	0	0	0
	1<br>Osjap05g02540.1 0.304 0.974 0.67 0.002 0.202<br>Osjap10g10280.2 0.009 0.252 0.243 0.604 0.743	1<br>Osind05g02520.1 0.304 0.974 0.67 0.002 0.202<br>Osind10g07670.1 0.009 0.252 0.243 0.63 0.759	0<br>Orufi01g10400.1 0.243 0.939 0.696 0.168 0.326	1<br>Oglab03g23910.1 0.009 0.252 0.243 0.814 0.982<br>Oglab05g02200.1 0.304 0.974 0.67 0.002 0.202	1<br>Obart03g24070.1 0.009 0.252 0.243 0.551 0.957<br>Obart05g02350.1 0.304 0.974 0.67 0.002 0.202	1<br>Olong_021023-R2 0.009 0.252 0.243 0.018 0.518<br>Olong_025943-R1 0.304 1 0.696 0.003 0.247	0<br>Opunc05g02230.1 0.304 0.974 0.67 0.002 0.202	0<br>Obrac03g30060.1 0.148 0.939 0.791 0.046 0.255	0<br>Lperr01g11350.1 0.304 0.939 0.635 0.003 0.195	0<br>AT5G26751.1 0.304 0.974 0.67 0.002 0.19
Osind12g01290.1	1<br>Osjap11g01220.1 0.001 1 0.999 0.001 1<br>Osjap12g01140.1 0.001 1 0.999 0.001 1	1<br>Osind11g00690.1 0.001 0.954 0.953 0.001 0.999<br>Osind12g01290.1 0.001 1 0.999 0.001 1	1<br>Orufi11g01000.1 0.001 1 0.999 0.001 1<br>Orufi12g01130.1 0.001 1 0.999 0.001 1	1<br>Oglab12g00800.1 0.001 1 0.999 0.001 1	1<br>Obart11g01060.1 0.001 1 0.999 0.001 1	0	1<br>Opunc12g00940.1 0.001 1 0.999 0.001 1	0	0	0
	1<br>Osjap03g13100.1 0.502 0.981 0.479 0.097 0.622<br>Osjap07g30390.1 0.01 0.366 0.356 0.013 0.798	1<br>Osind03g12930.1 0.502 0.981 0.479 0.121 0.633<br>Osind07g27070.1 0.007 0.37 0.363 0.006 0.783	1<br>Orufi03g17220.1 0.509 1 0.491 0.027 1<br>Orufi07g27420.1 0.01 0.37 0.36 0.013 0.783	1<br>Oglab03g12120.1 0.502 0.981 0.479 0.121 0.633<br>Oglab07g22980.1 0.007 0.366 0.359 0.007 0.783	1<br>Obart03g12120.1 0.502 0.981 0.479 0.121 0.633<br>Obart10g06750.1 0.021 0.401 0.38 0.151 0.885	1<br>Olong_007715-R1 0.505 1 0.495 0.007 1<br>Olong_008211-R1 0.007 0.37 0.363 0.006 0.783	1<br>Opunc03g11680.1 0.502 1 0.498 0.148 1<br>Opunc11g00920.1 0.001 0.37 0.369 0.142 0.816	1<br>Obrac03g09720.2 0.502 1 0.498 0.142 1<br>Obrac10g04150.1 0.019 0.398 0.379 0.036 0.868	1<br>Lperr03g10720.1 0.502 0.993 0.491 0.148 0.951<br>Lperr11g00920.1 0.001 0.37 0.369 0.002 0.792	1<br>AT2G34940.1 0.001 0.37 0.369 0.002 0.793<br>AT3G46740.1 0.505 1 0.495 0.161 1
Osind12g02780.1	1<br>Osjap11g02640.1 0.002 1 0.998 0.002 1<br>Osjap12g02610.1 0.002 1 0.998 0.002 1	1<br>Osind11g01990.1 0.002 1 0.998 0.002 1<br>Osind12g02780.1 0.002 1 0.998 0.002 1	1<br>Orufi11g02320.1 0.002 1 0.998 0.003 1<br>Orufi12g02480.1 0.002 1 0.998 0.002 1	1<br>Oglab11g02200.1 0.002 1 0.998 0.002 1<br>Oglab12g02130.1 0.002 1 0.998 0.002 1	1<br>Obart12g02080.1 0.022 1 0.978 0.002 1	0	0	0	0	0
	1<br>Osjap07g04360.1 0.031 0.64 0.609 0.018 0.925<br>Osjap12g12870.1 0.651 0.953 0.302 0.022 0.417	1<br>Osind11g02010.1 0.013 0.64 0.627 0.001 0.994<br>Osind12g10020.1 0.651 0.953 0.302 0.022 0.417	1<br>Orufi07g03650.1 0.031 0.64 0.609 0.019 0.976<br>Orufi12g10910.1 0.651 0.953 0.302 0.022 0.417	1<br>Oglab07g03810.1 0.031 0.64 0.609 0.019 0.987<br>Oglab12g09880.1 0.651 0.953 0.302 0.02 0.385	0<br>Obart11g02350.1 0.02 0.64 0.62 0.004 0.993	1<br>Olong_012584-R1 0.061 0.597 0.536 0.002 0.965<br>Olong_017316-R1 0.651 0.953 0.302 0.017 0.365	1<br>Opunc11g02190.1 0.002 0.64 0.638 0.003 0.989<br>Opunc12g08740.1 0.651 0.953 0.302 0.015 0.324	1<br>Obrac05g10860.1 0.047 0.64 0.593 0.097 0.987<br>Obrac12g07170.1 0.651 0.944 0.293 0.029 0.57	1<br>Lperr12g02190.1 0.002 0.64 0.638 0.003 0.989<br>Lperr12g08380.1 0.644 0.987 0.343 0.01 0.488	0<br>AT5G16080.1 0.047 0.644 0.597 0.081 0.994
Osind12g09530.1	1<br>Osjap12g12320.1 0.001 1 0.999 0.001 1	1<br>Osind12g09530.1 0.001 1 0.999 0.001 1	1<br>Orufi12g10460.1 0.001 1 0.999 0.001 1	1<br>Oglab12g09470.1 0.001 1 0.999 0.001 1	1<br>Obart12g09410.1 0.001 1 0.999 0.001 1	0	0	0	0	0
	1<br>Osjap01g44780.2 0.453 0.998 0.545 0.095 0.994<br>Osjap03g07600.1 0.001 0.292 0.291 0.004 0.996	1<br>Osind01g46040.1 0.453 0.998 0.545 0.151 0.995<br>Osind03g07670.1 0.001 0.292 0.291 0.004 0.996	1<br>Orufi01g42650.3 0.453 0.998 0.545 0.195 0.995<br>Orufi03g07180.1 0.001 0.292 0.291 0.004 0.996	0<br>Oglab01g34040.1 0.502 0.998 0.496 0.203 0.995<br>Oglab03g07280.1 0.001 0.292 0.291 0.004 0.996	1<br>Obart01g39260.3 0.469 0.998 0.529 0.041 0.994<br>Obart03g07210.1 0.001 0.292 0.291 0.004 0.996	1<br>Olong_021912-R1 0.47 1 0.53 0.099 1<br>Olong_021913-R1 0.001 0.291 0.29 0.004 0.996	1<br>Opunc12g08570.1 0.334 1 0.666 0.017 1<br>Opunc12g08580.1 0.001 0.291 0.29 0.004 0.996	1<br>Obrac03g05880.1 0.001 0.291 0.29 0.004 0.996<br>Obrac12g06500.1 0.475 1 0.525 0.103 1	1<br>Lperr01g33810.2 0.469 0.998 0.529 0.002 0.994<br>Lperr03g06600.1 0.001 0.291 0.29 0.004 0.996	0<br>AT4G34670.1 0.001 0.283 0.282 0.004 0.966<br>AT5G67540.2 0.502 0.993 0.491 0.053 1
Osind12g10670.1	1<br>Osjap01g31400.1 0.007 0.915 0.908 0.006 0.818<br>Osjap12g13600.1 0.007 0.993 0.986 0.007 0.993	1<br>Osind01g32860.1 0.007 0.915 0.908 0.007 0.854<br>Osind12g10660.1 0.007 0.993 0.986 0.007 0.993<br>Osind12g10670.1 0.007 1 0.993 0.007 1	1<br>Orufi01g30130.1 0.007 0.915 0.908 0.007 0.854	1<br>Oglab01g22960.1 0.071 0.915 0.844 0.194 0.874	1<br>Obart01g27080.1 0.071 0.915 0.844 0.194 0.874<br>Obart12g10510.1 0.007 0.993 0.986 0.012 0.988	0	1<br>Opunc10g03560.1 0.035 0.844 0.809 0.016 0.248	0	0	0
	1<br>Osjap01g05590.1 0.014 0.326 0.312 0.017 0.556<br>Osjap02g24490.1 0.369 0.908 0.539 0.591 0.779	1<br>Osind03g19770.1 0.007 0.411 0.404 0.002 0.412<br>Osind04g15200.1 0.44 0.972 0.532 0.042 0.368	0<br>Orufi12g11520.1 0.007 0.794 0.787 0.008 0.882	1<br>Oglab01g20040.1 0.035 0.34 0.305 0.022 0.11<br>Oglab06g05230.1 0.404 0.915 0.511 0.669 0.816	1<br>Obart01g16250.1 0.411 0.908 0.497 0.765 0.869<br>Obart03g17930.1 0.007 0.411 0.404 0.002 0.126	1<br>Olong_000447-R1 0.39 0.908 0.518 0.691 0.824<br>Olong_003316-R1 0.035 0.34 0.305 0.021 0.103	1<br>Opunc03g17210.1 0.007 0.333 0.326 0.002 0.095<br>Opunc06g05170.1 0.404 0.915 0.511 0.649 0.802	0<br>Obrac01g31700.1 0.184 0.908 0.724 0.829 0.924	1<br>Lperr03g05500.1 0.411 0.915 0.504 0.585 0.769<br>Lperr03g15640.1 0.007 0.333 0.326 0.002 0.09	1<br>AT2G36780.1 0.035 0.333 0.298 0.028 0.113<br>AT5G50380.1 0.355 0.908 0.553 0.706 0.82

((((_d1,punctata),brachy),leersia),tair);

chimeric gene id	INGROUP						MIDGROUP	OUTGROUP		
	jap	indica	rufi	glab	barthii	longi	punctata	brachy	leersia	tair
Osind01g08490.1	1<br>Osjap01g08660.1 0.001 1 0.999 0.001 1	1<br>Osind01g08490.1 0.001 1 0.999 0.001 1	1<br>Orufi01g08070.1 0.001 1 0.999 0.001 1	1<br>Oglab01g06470.1 0.001 1 0.999 0.001 1	1<br>Obart01g07430.1 0.017 1 0.983 0.001 1	1<br>Olong_011567-R1 0.07 1 0.93 0.001 1	0	0	0	0
	1<br>Osjap01g29230.2 0.114 0.581 0.467 0.25 0.983<br>Osjap05g00560.2 0.647 0.997 0.35 0.146 1	1<br>Osind01g30690.1 0.114 0.581 0.467 0.267 0.982<br>Osind05g00540.1 0.647 0.997 0.35 0.126 1	1<br>Orufi01g27950.1 0.114 0.581 0.467 0.25 0.983<br>Orufi01g48560.2 0.647 0.98 0.333 0.085 0.984	1<br>Oglab01g21070.2 0.114 0.581 0.467 0.249 0.983<br>Oglab05g00530.1 0.647 0.997 0.35 0.146 1	1<br>Obart01g25090.1 0.114 0.581 0.467 0.219 0.982<br>Obart05g00540.1 0.647 0.997 0.35 0.146 1	0<br>Olong_003039-R1 0.647 0.997 0.35 0.128 1<br>Olong_003692-R2 0.153 0.581 0.428 0.244 0.979	1<br>Opunc01g07170.1 0.001 0.582 0.581 0.001 0.984<br>Opunc05g00510.2 0.647 0.997 0.35 0.15 1	1<br>Obrac01g05760.3 0.583 1 0.417 0.082 1<br>Obrac01g05770.3 0.001 0.582 0.581 0.001 0.985	1<br>Lperr01g07030.1 0.583 1 0.417 0.018 1<br>Lperr01g07040.2 0.001 0.582 0.581 0.001 0.989	1<br>AT1G54350.1 0.585 0.993 0.408 0.084 0.975<br>AT5G53430.1 0.024 0.581 0.557 0.09 0.984
Osind02g26640.2	1<br>Osjap02g26750.1 0.001 0.953 0.952 0.001 0.993<br>Osjap02g26750.2 0.001 0.953 0.952 0.001 0.993<br>Osjap02g26750.4 0.001 0.953 0.952 0.001 0.799	1<br>Osind02g26640.2 0.001 1 0.999 0.001 1	1<br>Orufi02g24900.1 0.01 0.958 0.948 0.06 0.859<br>Orufi02g24900.3 0.001 0.958 0.957 0.001 0.74<br>Orufi02g24900.4 0.001 0.958 0.957 0.001 0.737	1<br>Oglab02g22130.2 0.001 1 0.999 0.001 1	1<br>Obart02g23460.2 0.001 1 0.999 0.001 1	0	1<br>Opunc02g21410.1 0.01 0.975 0.965 0.116 0.996<br>Opunc02g21410.2 0.01 0.975 0.965 0.114 0.996<br>Opunc02g21410.3 0.01 0.975 0.965 0.115 0.996<br>Opunc02g21410.4 0.01 0.975 0.965 0.114 0.996	0	0	0
	1<br>Osjap02g26590.1 0.497 0.986 0.489 0.033 0.996<br>Osjap02g26750.5 0.01 0.413 0.403 0.238 0.968	1<br>Osind02g26450.1 0.497 0.986 0.489 0.023 0.992<br>Osind02g26620.1 0.01 0.413 0.403 0.495 0.978	1<br>Orufi02g24730.1 0.497 0.986 0.489 0.031 0.996<br>Orufi02g24880.2 0.01 0.413 0.403 0.18 0.964	1<br>Oglab02g21930.1 0.497 1 0.503 0.047 1<br>Oglab02g22110.2 0.01 0.413 0.403 0.532 0.976	1<br>Obart02g23290.1 0.497 1 0.503 0.029 0.997<br>Obart02g23440.1 0.01 0.413 0.403 0.492 0.978	0<br>Olong_012377-R1 0.651 0.957 0.306 0.444 0.915<br>Olong_024345-R1 0.01 0.434 0.424 0.442 0.991	1<br>Opunc02g21230.1 0.504 0.96 0.456 0.003 0.934<br>Opunc02g21240.1 0.01 0.422 0.412 0.121 0.972	1<br>Obrac02g16670.1 0.503 0.957 0.454 0.005 0.995<br>Obrac02g16700.1 0.01 0.422 0.412 0.114 0.947	1<br>Lperr02g18740.1 0.512 0.975 0.463 0.059 0.982<br>Lperr02g19120.1 0.01 0.443 0.433 0.183 0.978	0<br>AT1G47230.2 0.577 0.953 0.376 0.095 0.857<br>AT4G31860.1 0.013 0.411 0.398 0.123 0.989
Osind03g00740.1	1<br>Osjap03g00680.1 0.001 0.996 0.995 0.001 0.994<br>Osjap03g00680.2 0.001 0.996 0.995 0.001 0.994<br>Osjap03g00680.3 0.001 0.996 0.995 0.001 0.994	1<br>Osind03g00740.1 0.001 1 0.999 0.001 1	1<br>Orufi03g12660.1 0.001 0.996 0.995 0.001 0.994<br>Orufi03g12660.2 0.001 0.996 0.995 0.001 0.994	1<br>Oglab03g00390.1 0.001 0.996 0.995 0.001 0.994	1<br>Obart03g00550.1 0.001 1 0.999 0.001 1	0	0	0	0	0
	1<br>Osjap02g42090.1 0.052 0.678 0.626 0.065 1<br>Osjap12g08580.1 0.708 0.996 0.288 0.011 0.977	1<br>Osind02g41670.1 0.062 0.678 0.616 0.131 1<br>Osind03g02220.1 0.698 0.996 0.298 0.015 0.982	1<br>Orufi02g39350.1 0.062 0.678 0.616 0.131 1<br>Orufi09g03230.3 0.732 0.99 0.258 0.053 0.963	1<br>Oglab02g34920.1 0.062 0.678 0.616 0.131 1<br>Oglab03g02050.1 0.698 0.996 0.298 0.214 0.986	1<br>Obart02g37790.1 0.062 0.648 0.586 0.133 0.977<br>Obart12g06510.1 0.676 0.996 0.32 0.052 0.984	0<br>Olong_013175-R1 0.271 0.904 0.633 0.002 0.961	1<br>Opunc03g00470.1 0.001 0.78 0.779 0.002 1<br>Opunc11g04590.1 0.78 0.996 0.216 0.055 0.982	0<br>Obrac02g27970.1 0.137 0.678 0.541 0.105 1<br>Obrac05g17660.1 0.743 0.995 0.252 0.425 0.997	1<br>Lperr03g00570.1 0.001 0.678 0.677 0.002 1<br>Lperr04g11360.1 0.747 0.996 0.249 0.415 0.99	0<br>AT5G04460.1 0.018 0.678 0.66 0.081 1
Osind03g35550.1	1<br>Osjap03g34330.1 0.039 1 0.961 0.001 1	1<br>Osind03g35550.1 0.001 1 0.999 0.001 1	1<br>Orufi03g33060.1 0.039 1 0.961 0.001 1	1<br>Oglab03g30830.1 0.039 1 0.961 0.001 1	1<br>Obart03g31750.1 0.039 1 0.961 0.046 1	0	0	0	0	0
	1<br>Osjap03g39110.1 0.646 1 0.354 0.036 1<br>Osjap10g05370.1 0.026 0.622 0.596 0.051 0.963	1<br>Osind01g46540.1 0.646 0.999 0.353 0.065 0.994<br>Osind10g03770.1 0.026 0.622 0.596 0.057 0.96	1<br>Orufi10g04850.1 0.026 0.622 0.596 0.054 0.963<br>Orufi11g12520.1 0.647 1 0.353 0.038 0.991	1<br>Oglab03g34460.1 0.622 0.997 0.375 0.016 0.993<br>Oglab10g04340.1 0.02 0.622 0.602 0.058 0.963	1<br>Obart03g36200.1 0.646 1 0.354 0.016 0.997<br>Obart10g04600.1 0.051 0.622 0.571 0.106 0.962	1<br>Olong_014796-R1 0.039 0.532 0.493 0.002 0.95<br>Olong_027495-R1 0.622 0.995 0.373 0.005 0.998	1<br>Opunc01g42030.1 0.646 1 0.354 0.28 0.872<br>Opunc10g04080.2 0.033 0.622 0.589 0.084 0.959	1<br>Obrac02g16280.1 0.64 0.981 0.341 0.063 0.949<br>Obrac03g23440.1 0.039 0.622 0.583 0.002 0.964	1<br>Lperr03g30520.1 0.638 1 0.362 0.003 0.997<br>Lperr10g03360.1 0.004 0.622 0.618 0.009 0.962	0<br>AT1G70730.3 0.039 0.622 0.583 0.118 0.967
Osind05g00510.1	1<br>Osjap05g00530.1 0.001 1 0.999 0.131 1<br>Osjap05g00530.2 0.001 1 0.999 0.16 1<br>Osjap05g00530.3 0.001 1 0.999 0.161 1<br>Osjap05g00530.4 0.001 1 0.999 0.142 1	1<br>Osind05g00510.1 0.001 1 0.999 0.001 1	1<br>Orufi05g00500.1 0.001 1 0.999 0.131 1	0	1<br>Obart05g00520.1 0.001 0.999 0.998 0.049 0.993<br>Obart05g00520.2 0.001 1 0.999 0.049 1	1<br>Olong_022405-R1 0.001 1 0.999 0.001 1	0	0	0	0
	0<br>Osjap02g42390.1 0.326 0.993 0.667 0.001 0.983	1<br>Osind01g11620.1 0.002 0.275 0.273 0.271 0.988	1<br>Orufi07g24760.1 0.001 0.258 0.257 0.267 0.834	1<br>Oglab05g00500.1 0.001 0.26 0.259 0.344 0.982	1<br>Obart01g04950.1 0.001 0.258 0.257 0.193 0.858	1<br>Olong_004108-R1 0.004 0.275 0.271 0.098 0.928	1<br>Opunc05g00480.1 0.298 0.999 0.701 0.007 0.998	1<br>Obrac02g14170.4 0.004 0.267 0.263 0.164 0.843	1<br>Lperr02g16220.1 0.004 0.267 0.263 0.238 0.857	0<br>AT4G14820.1 0.001 0.267 0.266 0.107 0.878

		Osind09g15830.1 0.31 1 0.69 0.029 1	Orufi09g16480.1 0.31 1 0.69 0.029 1	Oglab09g13820.1 0.291 1 0.709 0.064 1	Obart07g24200.1 0.302 0.998 0.696 0.005 0.907	Olong_023813-R1 0.303 0.997 0.694 0.001 0.979	Opunc05g00490.1 0.001 0.26 0.259 0.352 0.982	Obrac03g06070.3 0.31 0.998 0.688 0.023 0.996	Lperr05g00500.1 0.301 1 0.699 0.009 1	
Osind05g04070.1	1<br>Osjap05g04120.1 0.001 1 0.999 0.008 1<br>Osjap05g04120.2 0.001 1 0.999 0.008 1	1<br>Osind05g04070.1 0.001 1 0.999 0.001 1<br>Osind05g04070.2 0.001 1 0.999 0.001 1	1<br>Orufi05g03950.1 0.001 1 0.999 0.008 1<br>Orufi05g03950.2 0.001 1 0.999 0.008 1	1<br>Oglab05g03490.1 0.001 1 0.999 0.008 1<br>Oglab05g03490.2 0.001 1 0.999 0.008 1	1<br>Obart05g03830.1 0.001 1 0.999 0.008 1<br>Obart05g03830.2 0.001 1 0.999 0.008 1	1<br>Olong_016739-R1 0.069 1 0.931 0.001 1	1<br>Opunc05g03540.1 0.001 1 0.999 0.008 1<br>Opunc05g03540.2 0.001 1 0.999 0.008 1	1<br>Obrac05g02930.1 0.069 1 0.931 0.001 1	0	0
	1<br>Osjap01g27410.1 0.086 0.735 0.649 0.066 0.992<br>Osjap03g15140.2 0.738 1 0.262 0.01 0.192	1<br>Osind01g28630.1 0.086 0.735 0.649 0.066 0.992<br>Osind03g15230.1 0.783 1 0.217 0.03 1	1<br>Orufi01g26130.1 0.086 0.735 0.649 0.066 0.992<br>Orufi03g14720.1 0.783 1 0.217 0.03 1	1<br>Oglab01g19490.1 0.086 0.735 0.649 0.066 0.992<br>Oglab03g14060.1 0.783 1 0.217 0.03 1	1<br>Obart01g23310.1 0.086 0.735 0.649 0.066 0.992<br>Obart03g14050.1 0.783 1 0.217 0.03 1	1<br>Olong_003347-R2 0.086 0.735 0.649 0.047 0.992<br>Olong_009943-R1 0.783 1 0.217 0.03 1	1<br>Opunc01g23460.1 0.086 0.735 0.649 0.064 0.991<br>Opunc03g13590.1 0.783 1 0.217 0.002 0.545	1<br>Obrac01g17590.1 0.086 0.735 0.649 0.067 0.991<br>Obrac03g11330.1 0.783 1 0.217 0.03 1	0<br>Lperr05g03460.1 0.001 0.736 0.735 0.011 0.994	1<br>AT2G47580.1 0.768 1 0.232 0.008 1<br>AT3G16630.1 0.001 0.736 0.735 0.039 0.994
Osind05g04070.2	1<br>Osjap05g04120.1 0.001 1 0.999 0.008 1<br>Osjap05g04120.2 0.001 1 0.999 0.008 1	1<br>Osind05g04070.1 0.001 1 0.999 0.001 1<br>Osind05g04070.2 0.001 1 0.999 0.001 1	1<br>Orufi05g03950.1 0.001 1 0.999 0.008 1<br>Orufi05g03950.2 0.001 1 0.999 0.008 1	1<br>Oglab05g03490.1 0.001 1 0.999 0.008 1<br>Oglab05g03490.2 0.001 1 0.999 0.008 1	1<br>Obart05g03830.1 0.001 1 0.999 0.008 1<br>Obart05g03830.2 0.001 1 0.999 0.008 1	1<br>Olong_016739-R1 0.068 1 0.932 0.001 1	1<br>Opunc05g03540.1 0.001 1 0.999 0.008 1<br>Opunc05g03540.2 0.001 1 0.999 0.008 1	1<br>Obrac05g02930.1 0.068 1 0.932 0.001 1	0	0
	1<br>Osjap01g27410.1 0.084 0.738 0.654 0.066 0.992<br>Osjap03g15140.2 0.742 1 0.258 0.01 0.192	1<br>Osind01g28630.1 0.084 0.738 0.654 0.066 0.992<br>Osind03g15230.1 0.786 1 0.214 0.03 1	1<br>Orufi01g26130.1 0.084 0.738 0.654 0.066 0.992<br>Orufi03g14720.1 0.786 1 0.214 0.03 1	1<br>Oglab01g19490.1 0.084 0.738 0.654 0.066 0.992<br>Oglab03g14060.1 0.786 1 0.214 0.03 1	1<br>Obart01g23310.1 0.084 0.738 0.654 0.066 0.992<br>Obart03g14050.1 0.786 1 0.214 0.03 1	1<br>Olong_003347-R2 0.084 0.738 0.654 0.047 0.992<br>Olong_009943-R1 0.786 1 0.214 0.03 1	1<br>Opunc01g23460.1 0.084 0.738 0.654 0.064 0.991<br>Opunc03g13590.1 0.786 1 0.214 0.002 0.545	1<br>Obrac01g17590.1 0.084 0.738 0.654 0.067 0.991<br>Obrac03g11330.1 0.786 1 0.214 0.03 1	0<br>Lperr05g03460.1 0.001 0.74 0.739 0.011 0.994	1<br>AT2G47580.1 0.771 1 0.229 0.008 1<br>AT3G16630.1 0.001 0.74 0.739 0.039 0.994
Osind05g28520.1	1<br>Osjap05g28230.1 0.001 1 0.999 0.001 1	1<br>Osind05g28520.1 0.001 1 0.999 0.001 1	1<br>Orufi05g26870.1 0.001 1 0.999 0.001 1	1<br>Oglab05g23160.1 0.001 1 0.999 0.001 1	1<br>Obart05g25070.1 0.001 1 0.999 0.001 1	0	1<br>Opunc05g22640.1 0.001 1 0.999 0.001 1	0	1<br>Lperr01g22990.1 0.001 1 0.999 0.027 1	0
	1<br>Osjap01g31140.1 0.006 0.43 0.424 0.093 0.995<br>Osjap01g31150.1 0.486 0.999 0.513 0.009 0.999	1<br>Osind01g32600.1 0.001 0.43 0.429 0.002 0.995<br>Osind01g32610.1 0.486 0.999 0.513 0.009 0.999	1<br>Orufi01g29880.1 0.001 0.43 0.429 0.075 0.995<br>Orufi01g29890.1 0.486 0.999 0.513 0.009 0.999	1<br>Oglab01g22740.1 0.091 0.431 0.34 0.213 0.996<br>Oglab01g22750.1 0.486 0.999 0.513 0.009 0.999	1<br>Obart01g26870.1 0.005 0.43 0.425 0.117 0.995<br>Obart01g26880.1 0.486 0.999 0.513 0.009 0.999	1<br>Olong_000367-R1 0.1 0.431 0.331 0.002 0.995<br>Olong_000368-R1 0.461 0.999 0.538 0.069 0.999	1<br>Opunc01g26820.1 0.091 0.431 0.34 0.118 0.996<br>Opunc01g26830.1 0.486 1 0.514 0.009 1	1<br>Obrac01g20400.1 0.091 0.431 0.34 0.125 0.996<br>Obrac05g17560.2 0.431 1 0.569 0.109 1	1<br>Lperr05g20850.2 0.491 1 0.509 0.002 1<br>Lperr05g20860.1 0.091 0.431 0.34 0.215 0.996	1<br>AT5G08680.1 0.022 0.43 0.408 0.064 0.995<br>AT5G51600.1 0.486 0.999 0.513 0.008 0.996
Osind08g06790.1	1<br>Osjap08g06650.1 0.002 1 0.998 0.002 1	1<br>Osind08g06790.1 0.002 1 0.998 0.002 1	1<br>Orufi08g06840.1 0.002 1 0.998 0.002 1	1<br>Oglab08g05640.1 0.002 1 0.998 0.002 1	1<br>Obart08g06070.1 0.002 1 0.998 0.002 1	0	1<br>Opunc08g05850.1 0.047 1 0.953 0.068 1	0	0	0
	1<br>Osjap04g21220.1 0.047 0.275 0.228 0.21 0.936<br>Osjap09g20870.1 0.359 0.984 0.625 0.208 0.966	1<br>Osind04g20250.1 0.047 0.275 0.228 0.198 0.94<br>Osind09g20960.1 0.359 0.984 0.625 0.208 0.966	1<br>Orufi04g19250.2 0.047 0.275 0.228 0.21 0.936<br>Orufi09g21960.1 0.359 0.984 0.625 0.2 0.965	1<br>Oglab02g26360.1 0.348 0.996 0.648 0.01 0.994<br>Oglab04g16180.1 0.047 0.275 0.228 0.198 0.94	1<br>Obart02g28440.1 0.348 0.996 0.648 0.093 0.994<br>Obart04g17770.1 0.047 0.275 0.228 0.198 0.94	1<br>Olong_008030-R1 0.047 0.275 0.228 0.232 0.937<br>Olong_008031-R2 0.277 1 0.723 0.106 1	1<br>Opunc04g15550.1 0.047 0.275 0.228 0.206 0.938<br>Opunc04g18740.1 0.295 0.984 0.689 0.088 0.994	1<br>Obrac08g04780.1 0.277 1 0.723 0.098 1<br>Obrac08g04800.1 0.002 0.275 0.273 0.006 0.943	1<br>Lperr08g05690.1 0.277 1 0.723 0.1 1<br>Lperr08g05700.1 0.002 0.275 0.273 0.006 0.943	1<br>AT1G05310.1 0.33 0.982 0.652 0.183 0.99<br>AT4G30800.1 0.047 0.275 0.228 0.208 0.943
Osind09g15570.1	1<br>Osjap09g15430.2 0.002 1 0.998 0.002 1<br>Osjap09g15430.3 0.002 1 0.998 0.002 1	1<br>Osind09g15570.1 0.002 1 0.998 0.002 1<br>Osind09g15570.2 0.002 1 0.998 0.002 1	1<br>Orufi09g16160.1 0.002 1 0.998 0.002 1<br>Orufi09g16160.2 0.002 1 0.998 0.002 1	1<br>Oglab09g13510.1 0.002 1 0.998 0.002 1<br>Oglab09g13510.2 0.002 1 0.998 0.002 1<br>Oglab09g13510.3 0.002 1 0.998 0.002 1<br>Oglab09g13510.4 0.002 1 0.998 0.002 1	1<br>Obart09g14980.1 0.002 1 0.998 0.002 1<br>Obart09g14980.2 0.002 1 0.998 0.002 1	0	1<br>Opunc09g13420.1 0.002 1 0.998 0.002 1<br>Opunc09g13420.2 0.002 1 0.998 0.002 1	1<br>Obrac09g09090.1 0.002 1 0.998 0.002 1	0	0
	1<br>Osjap02g06650.1 0.01 0.54 0.53 0.01 0.997<br>Osjap09g15430.1 0.544 1 0.456 0.131 1	1<br>Osind02g06940.1 0.01 0.54 0.53 0.01 0.997<br>Osind08g23920.1 0.544 0.993 0.449 0.131 0.988	1<br>Orufi04g24220.1 0.013 0.542 0.529 0.165 1<br>Orufi08g22880.1 0.544 0.993 0.449 0.131 0.988	1<br>Oglab08g19240.1 0.544 0.993 0.449 0.143 0.986<br>Oglab12g18680.1 0.002 0.542 0.54 0.003 1	1<br>Obart02g06180.1 0.01 0.54 0.53 0.01 0.997<br>Obart08g20390.1 0.544 0.993 0.449 0.631 0.988	1<br>Olong_013054-R1 0.547 0.992 0.445 0.149 0.979<br>Olong_024186-R1 0.002 0.542 0.54 0.003 1	1<br>Opunc04g20190.1 0.007 0.542 0.535 0.006 1<br>Opunc08g18560.1 0.544 0.993 0.449 0.128 0.969	1<br>Obrac02g04550.3 0.008 0.54 0.532 0.214 0.997<br>Obrac09g00560.1 0.548 0.998 0.45 0.164 0.966	1<br>Lperr04g18350.1 0.002 0.542 0.54 0.021 1<br>Lperr09g11120.1 0.544 1 0.456 0.156 1	1<br>AT1G68540.1 0.542 0.992 0.45 0.112 1<br>AT2G21270.3 0.003 0.54 0.537 0.059 0.997
Osind09g15570.2	1<br>Osjap09g15430.2 0.002 1 0.998 0.002 1	1<br>Osind09g15570.1 0.002 1 0.998 0.002 1	1<br>Orufi09g16160.1 0.002 1 0.998 0.002 1	1<br>Oglab09g13510.1 0.002 1 0.998 0.002 1	1<br>Obart09g14980.1 0.002 1 0.998 0.002 1	0	1<br>Opunc09g13420.1 0.002 1 0.998 0.002 1	1<br>Obrac09g09090.1 0.002 1 0.998 0.002 1	0	0

	Osjap09g15430.3 0.002 1 0.998 0.002 1	Osind09g15570.2 0.002 1 0.998 0.002 1	Orufi09g16160.2 0.002 1 0.998 0.002 1	Oglab09g13510.2 0.002 1 0.998 0.002 1<br>Oglab09g13510.3 0.002 1 0.998 0.002 1<br>Oglab09g13510.4 0.002 1 0.998 0.002 1	Obart09g14980.2 0.002 1 0.998 0.002 1		Opunc09g13420.2 0.002 1 0.998 0.002 1			
	1<br>Osjap02g06650.1 0.01 0.565 0.555 0.01 0.997<br>Osjap09g15430.1 0.569 1 0.431 0.131 1	1<br>Osind02g06940.1 0.01 0.565 0.555 0.01 0.997<br>Osind08g23920.1 0.569 0.993 0.424 0.131 0.988	1<br>Orufi04g24220.1 0.014 0.567 0.553 0.165 1<br>Orufi08g22880.1 0.569 0.993 0.424 0.131 0.988	1<br>Oglab08g19240.1 0.569 0.993 0.424 0.143 0.986<br>Oglab12g18680.1 0.002 0.567 0.565 0.003 1	1<br>Obart02g06180.1 0.01 0.565 0.555 0.01 0.997<br>Obart08g20390.1 0.569 0.993 0.424 0.631 0.988	1<br>Olong_008093-R2 0.596 0.991 0.395 0.25 0.996<br>Olong_024186-R1 0.002 0.567 0.565 0.003 1	1<br>Opunc04g20190.1 0.007 0.567 0.56 0.006 1<br>Opunc08g18560.1 0.569 0.993 0.424 0.128 0.969	1<br>Obrac01g09090.1 0.605 0.998 0.393 0.299 0.982<br>Obrac02g04550.3 0.009 0.565 0.556 0.214 0.997	1<br>Lperr04g18350.1 0.002 0.567 0.565 0.021 1<br>Lperr09g11120.1 0.569 1 0.431 0.156 1	1<br>AT1G68540.1 0.567 0.991 0.424 0.112 1<br>AT2G21270.3 0.003 0.565 0.562 0.059 0.997
Osind09g20560.1	1<br>Osjap09g20460.1 0.001 0.998 0.997 0.001 0.986	1<br>Osind09g20560.1 0.001 1 0.999 0.001 1	1<br>Orufi09g21510.1 0.001 0.978 0.977 0.001 0.999	1<br>Oglab09g18200.1 0.001 0.998 0.997 0.001 0.985	1<br>Obart09g19980.1 0.067 0.998 0.931 0.165 0.99<br>Obart09g19980.2 0.001 0.998 0.997 0.001 0.984	1<br>Olong_014589-R1 0.117 0.985 0.868 0.018 0.991<br>Olong_014589-R2 0.117 0.985 0.868 0.018 0.991	0	0	0	0
	1<br>Osjap02g21530.2 0.024 0.581 0.557 0.096 0.943<br>Osjap09g20430.1 0.623 0.998 0.375 0.085 0.962	1<br>Osind02g21430.2 0.024 0.581 0.557 0.096 0.943<br>Osind09g20550.1 0.623 0.998 0.375 0.032 0.982	1<br>Orufi02g19810.2 0.024 0.581 0.557 0.096 0.943<br>Orufi09g21500.1 0.623 0.998 0.375 0.085 0.962	1<br>Oglab02g17850.1 0.024 0.581 0.557 0.095 0.914<br>Oglab09g18170.1 0.623 0.998 0.375 0.079 0.966	1<br>Obart04g25370.1 0.047 0.589 0.542 0.176 0.967<br>Obart09g19950.1 0.623 0.998 0.375 0.079 0.966	1<br>Olong_014116-R1 0.064 0.589 0.525 0.069 0.957<br>Olong_027417-R1 0.625 0.982 0.357 0.135 0.711	1<br>Opunc09g18280.1 0.618 0.998 0.38 0.058 0.841<br>Opunc09g18290.2 0.001 0.59 0.589 0.001 0.97	1<br>Obrac09g13140.3 0.623 0.998 0.375 0.049 0.806<br>Obrac09g13160.3 0.001 0.596 0.595 0.001 0.996	1<br>Lperr09g15890.1 0.622 0.999 0.377 0.048 0.95<br>Lperr09g15920.1 0.001 0.59 0.589 0.001 0.957	0<br>AT1G18160.1 0.021 0.589 0.568 0.064 0.958
Osind11g00690.1	1<br>Osjap11g01220.1 0.001 0.999 0.998 0.001 0.955<br>Osjap12g01140.1 0.001 0.999 0.998 0.001 0.953	1<br>Osind11g00690.1 0.001 1 0.999 0.001 1<br>Osind12g01290.1 0.001 0.999 0.998 0.001 0.954	1<br>Orufi11g01000.1 0.001 0.999 0.998 0.001 0.951<br>Orufi12g01130.1 0.001 0.999 0.998 0.001 0.953	1<br>Oglab12g00800.1 0.001 0.999 0.998 0.001 0.956	1<br>Obart11g01060.1 0.001 0.999 0.998 0.001 0.954	0	1<br>Opunc12g00940.1 0.001 0.999 0.998 0.001 0.937	0	0	0
	1<br>Osjap03g13100.1 0.523 0.999 0.476 0.097 0.593<br>Osjap07g30390.1 0.01 0.462 0.452 0.013 0.944	1<br>Osind03g12930.1 0.523 0.999 0.476 0.121 0.604<br>Osind07g27070.1 0.007 0.462 0.455 0.006 0.946	1<br>Orufi03g17220.1 0.53 0.999 0.469 0.027 0.912<br>Orufi07g27420.1 0.01 0.462 0.452 0.013 0.946	1<br>Oglab03g12120.1 0.523 0.999 0.476 0.121 0.604<br>Oglab07g22980.1 0.007 0.462 0.455 0.007 0.94	1<br>Obart03g12120.1 0.523 0.999 0.476 0.121 0.604<br>Obart12g00790.1 0.001 0.462 0.461 0.002 0.949	1<br>Olong_007715-R1 0.527 0.999 0.472 0.009 0.911<br>Olong_008211-R1 0.007 0.462 0.455 0.006 0.946	1<br>Opunc03g11680.1 0.523 0.999 0.476 0.148 0.924<br>Opunc11g00920.1 0.001 0.462 0.461 0.142 0.951	1<br>Obrac03g09720.2 0.523 0.999 0.476 0.142 0.923<br>Obrac12g00780.1 0.001 0.462 0.461 0.11 0.953	0<br>Lperr11g00920.1 0.001 0.462 0.461 0.002 0.949	1<br>AT2G34940.1 0.001 0.462 0.461 0.002 0.955<br>AT3G46740.1 0.526 1 0.474 0.161 0.928
Osind11g01990.1	1<br>Osjap11g02640.1 0.002 1 0.998 0.002 1<br>Osjap12g02610.1 0.002 1 0.998 0.002 1	1<br>Osind11g01990.1 0.002 1 0.998 0.002 1<br>Osind12g02780.1 0.002 1 0.998 0.002 1	1<br>Orufi11g02320.1 0.002 1 0.998 0.013 1<br>Orufi12g02480.1 0.002 1 0.998 0.002 1	1<br>Oglab11g02200.1 0.002 1 0.998 0.002 1<br>Oglab12g02130.1 0.002 1 0.998 0.002 1	1<br>Obart12g02080.1 0.023 1 0.977 0.002 1	0	0	0	0	0
	1<br>Osjap07g04360.1 0.033 0.669 0.636 0.018 0.925<br>Osjap12g12870.1 0.673 0.949 0.276 0.055 0.417	1<br>Osind11g02010.1 0.014 0.669 0.655 0.001 0.994<br>Osind12g10020.1 0.673 0.949 0.276 0.055 0.417	1<br>Orufi12g02470.1 0.045 0.669 0.624 0.04 0.988<br>Orufi12g10750.1 0.671 0.949 0.278 0.045 0.364	1<br>Oglab07g03810.1 0.033 0.669 0.636 0.019 0.987<br>Oglab12g09880.1 0.673 0.949 0.276 0.051 0.385	0<br>Obart11g02350.1 0.021 0.669 0.648 0.024 0.993	1<br>Olong_012584-R1 0.066 0.622 0.556 0.002 0.965<br>Olong_017316-R1 0.671 0.949 0.278 0.045 0.365	1<br>Opunc11g02190.1 0.002 0.669 0.667 0.003 0.989<br>Opunc12g08740.1 0.671 0.949 0.278 0.04 0.324	1<br>Obrac05g10860.1 0.051 0.669 0.618 0.097 0.987<br>Obrac12g07170.1 0.671 0.94 0.269 0.071 0.57	1<br>Lperr12g02190.1 0.002 0.669 0.667 0.003 0.989<br>Lperr12g08370.1 0.669 0.949 0.28 0.047 0.391	0<br>AT5G27320.1 0.014 0.643 0.629 0.003 0.98
Osind12g01290.1	1<br>Osjap11g01220.1 0.001 1 0.999 0.001 1<br>Osjap12g01140.1 0.001 1 0.999 0.001 1	1<br>Osind11g00690.1 0.001 0.954 0.953 0.001 0.999<br>Osind12g01290.1 0.001 1 0.999 0.001 1	1<br>Orufi11g01000.1 0.001 1 0.999 0.001 1<br>Orufi12g01130.1 0.001 1 0.999 0.001 1	1<br>Oglab12g00800.1 0.001 1 0.999 0.001 1	1<br>Obart11g01060.1 0.001 1 0.999 0.001 1	0	1<br>Opunc12g00940.1 0.001 1 0.999 0.001 1	0	0	0
	1<br>Osjap03g13100.1 0.502 0.981 0.479 0.097 0.622<br>Osjap07g30390.1 0.01 0.366 0.356 0.013 0.798	1<br>Osind03g12930.1 0.502 0.981 0.479 0.121 0.633<br>Osind07g27070.1 0.007 0.37 0.363 0.006 0.783	1<br>Orufi03g17220.1 0.509 1 0.491 0.027 1<br>Orufi07g27420.1 0.01 0.37 0.36 0.013 0.783	1<br>Oglab03g12120.1 0.502 0.981 0.479 0.121 0.633<br>Oglab07g22980.1 0.007 0.366 0.359 0.007 0.783	1<br>Obart03g12120.1 0.502 0.981 0.479 0.121 0.633<br>Obart10g06750.1 0.021 0.401 0.38 0.151 0.885	1<br>Olong_007715-R1 0.505 1 0.495 0.007 1<br>Olong_008211-R1 0.007 0.37 0.363 0.006 0.783	1<br>Opunc03g11680.1 0.502 1 0.498 0.148 1<br>Opunc11g00920.1 0.001 0.37 0.369 0.142 0.816	1<br>Obrac03g09720.2 0.502 1 0.498 0.142 1<br>Obrac10g04150.1 0.019 0.398 0.379 0.036 0.868	1<br>Lperr03g10720.1 0.502 0.993 0.491 0.148 0.951<br>Lperr11g00920.1 0.001 0.37 0.369 0.002 0.792	1<br>AT2G34940.1 0.001 0.37 0.369 0.002 0.793<br>AT3G46740.1 0.505 1 0.495 0.161 1
Osind12g02780.1	1<br>Osjap11g02640.1 0.002 1 0.998 0.002 1<br>Osjap12g02610.1 0.002 1 0.998 0.002 1	1<br>Osind11g01990.1 0.002 1 0.998 0.002 1<br>Osind12g02780.1 0.002 1 0.998 0.002 1	1<br>Orufi11g02320.1 0.002 1 0.998 0.003 1<br>Orufi12g02480.1 0.002 1 0.998 0.002 1	1<br>Oglab11g02200.1 0.002 1 0.998 0.002 1<br>Oglab12g02130.1 0.002 1 0.998 0.002 1	1<br>Obart12g02080.1 0.022 1 0.978 0.002 1	0	0	0	0	0
	1<br>Osjap07g04360.1 0.031 0.64 0.609 0.018 0.925	1<br>Osind11g02010.1 0.013 0.64 0.627 0.001 0.994	1<br>Orufi07g03650.1 0.031 0.64 0.609 0.019 0.976	1<br>Oglab07g03810.1 0.031 0.64 0.609 0.019 0.987	0<br>Obart11g02350.1 0.02 0.64 0.62 0.004 0.993	1<br>Olong_012584-R1 0.061 0.597 0.536 0.002 0.965	1<br>Opunc11g02190.1 0.002 0.64 0.638 0.003 0.989	1<br>Obrac05g10860.1 0.047 0.64 0.593 0.097 0.987	1<br>Lperr12g02190.1 0.002 0.64 0.638 0.003 0.989	0<br>AT5G16080.1 0.047 0.644 0.597 0.081 0.994

	Osjap12g12870.1 0.651 0.953 0.302 0.022 0.417	Osind12g10020.1 0.651 0.953 0.302 0.022 0.417	Orufi12g10910.1 0.651 0.953 0.302 0.022 0.417	Oglab12g09880.1 0.651 0.953 0.302 0.02 0.385		Olong_017316-R1 0.651 0.953 0.302 0.017 0.365	Opunc12g08740.1 0.651 0.953 0.302 0.015 0.324	Obrac12g07170.1 0.651 0.944 0.293 0.029 0.57	Lperr12g08380.1 0.644 0.987 0.343 0.01 0.488	
Osind12g09530.1	1<br>Osjap12g12320.1 0.001 1 0.999 0.001 1	1<br>Osind12g09530.1 0.001 1 0.999 0.001 1	1<br>Orufi12g10460.1 0.001 1 0.999 0.001 1	1<br>Oglab12g09470.1 0.001 1 0.999 0.001 1	1<br>Obart12g09410.1 0.001 1 0.999 0.001 1	0	0	0	0	0
	1<br>Osjap01g44780.2 0.453 0.998 0.545 0.095 0.994<br>Osjap03g07600.1 0.001 0.292 0.291 0.004 0.996	1<br>Osind01g46040.1 0.453 0.998 0.545 0.151 0.995<br>Osind03g07670.1 0.001 0.292 0.291 0.004 0.996	1<br>Orufi01g42650.3 0.453 0.998 0.545 0.195 0.995<br>Orufi03g07180.1 0.001 0.292 0.291 0.004 0.996	0<br>Oglab01g34040.1 0.502 0.998 0.496 0.203 0.995<br>Oglab03g07280.1 0.001 0.292 0.291 0.004 0.996	1<br>Obart01g39260.3 0.469 0.998 0.529 0.041 0.994<br>Obart03g07210.1 0.001 0.292 0.291 0.004 0.996	1<br>Olong_021912-R1 0.47 1 0.53 0.099 1<br>Olong_021913-R1 0.001 0.291 0.29 0.004 0.996	1<br>Opunc12g08570.1 0.334 1 0.666 0.017 1<br>Opunc12g08580.1 0.001 0.291 0.29 0.004 0.996	1<br>Obrac03g05880.1 0.001 0.291 0.29 0.004 0.996<br>Obrac12g06500.1 0.475 1 0.525 0.103 1	1<br>Lperr01g33810.2 0.469 0.998 0.529 0.002 0.994<br>Lperr03g06600.1 0.001 0.291 0.29 0.004 0.996	0<br>AT4G34670.1 0.001 0.283 0.282 0.004 0.966<br>AT5G67540.2 0.502 0.993 0.491 0.053 1
Osind12g10670.1	1<br>Osjap01g31400.1 0.007 0.915 0.908 0.006 0.818<br>Osjap12g13600.1 0.007 0.993 0.986 0.007 0.993	1<br>Osind01g32860.1 0.007 0.915 0.908 0.007 0.854<br>Osind12g10660.1 0.007 0.993 0.986 0.007 0.993<br>Osind12g10670.1 0.007 1 0.993 0.007 1	1<br>Orufi01g30130.1 0.007 0.915 0.908 0.007 0.854	1<br>Oglab01g22960.1 0.071 0.915 0.844 0.194 0.874	1<br>Obart01g27080.1 0.071 0.915 0.844 0.194 0.874<br>Obart12g10510.1 0.007 0.993 0.986 0.012 0.988	0	1<br>Opunc10g03560.1 0.035 0.844 0.809 0.016 0.248	0	0	0
	1<br>Osjap01g05590.1 0.014 0.326 0.312 0.017 0.556<br>Osjap02g24490.1 0.369 0.908 0.539 0.591 0.779	1<br>Osind03g19770.1 0.007 0.411 0.404 0.002 0.412<br>Osind04g15200.1 0.44 0.972 0.532 0.042 0.368	0<br>Orufi12g11520.1 0.007 0.794 0.787 0.008 0.882	1<br>Oglab01g20040.1 0.035 0.34 0.305 0.022 0.11<br>Oglab06g05230.1 0.404 0.915 0.511 0.669 0.816	1<br>Obart01g16250.1 0.411 0.908 0.497 0.765 0.869<br>Obart03g17930.1 0.007 0.411 0.404 0.002 0.126	1<br>Olong_000447-R1 0.39 0.908 0.518 0.691 0.824<br>Olong_003316-R1 0.035 0.34 0.305 0.021 0.103	1<br>Opunc03g17210.1 0.007 0.333 0.326 0.002 0.095<br>Opunc06g05170.1 0.404 0.915 0.511 0.649 0.802	0<br>Obrac01g31700.1 0.184 0.908 0.724 0.829 0.924	1<br>Lperr03g05500.1 0.411 0.915 0.504 0.585 0.769<br>Lperr03g15640.1 0.007 0.333 0.326 0.002 0.09	1<br>AT2G36780.1 0.035 0.333 0.298 0.028 0.113<br>AT5G50380.1 0.355 0.908 0.553 0.706 0.82

((((((((jap,indica),rufi),(glab,barthii)),longi),punctata),brachy),leersia),tair);

chimeric gene id	INGROUP	MIDGROUP						OUTGROUP		
	glab	jap	indica	rufi	barthii	longi	punctata	brachy	leersia	tair
Oglab01g01590.1	1<br>Oglab01g01590.1 0.001 1 0.999 0.001 1	0	1<br>Osind01g02220.1 0.001 0.999 0.998 0.056 0.987	0	1<br>Obart01g01710.1 0.001 0.987 0.986 0.001 0.986	1<br>Olong_021362-R1 0.001 0.999 0.998 0.048 0.986	1<br>Opunc01g01490.1 0.002 0.998 0.996 0.021 0.988<br>Opunc01g01490.2 0.002 0.998 0.996 0.024 0.988	0	0	0
	1<br>Oglab01g01570.1 0.537 0.999 0.462 0.043 0.998<br>Oglab05g03370.1 0.002 0.469 0.467 0.104 0.976	1<br>Osjap01g02100.1 0.001 0.469 0.468 0.002 0.973<br>Osjap01g02110.1 0.511 1 0.489 0.039 1	1<br>Osind01g02230.1 0.511 0.987 0.476 0.061 0.964<br>Osind05g03930.1 0.002 0.469 0.467 0.104 0.976	1<br>Orufi01g01810.1 0.001 0.469 0.468 0.002 0.973<br>Orufi01g01830.1 0.537 0.999 0.462 0.079 0.999	1<br>Obart01g01680.1 0.537 0.999 0.462 0.046 0.998<br>Obart05g03720.1 0.002 0.469 0.467 0.104 0.976	1<br>Olong_016471-R1 0.002 0.469 0.467 0.106 0.972<br>Olong_021361-R1 0.514 1 0.486 0.002 1	1<br>Opunc01g01490.3 0.537 0.993 0.456 0.056 0.981<br>Opunc05g03460.1 0.016 0.469 0.453 0.119 0.971	1<br>Obrac01g01340.1 0.485 0.998 0.513 0.001 0.999<br>Obrac01g17910.1 0.004 0.469 0.465 0.248 0.98	1<br>Lperr01g01640.1 0.509 0.999 0.49 0.051 0.998<br>Lperr05g03400.1 0.015 0.469 0.454 0.158 0.972	1<br>AT1G71040.1 0.526 0.999 0.473 0.021 0.998<br>AT3G48670.1 0.002 0.468 0.466 0.117 0.971
Oglab01g03790.1	1<br>Oglab01g03790.1 0.001 1 0.999 0.001 1	0	0	0	1<br>Obart01g04220.1 0.001 1 0.999 0.001 1	0	0	0	0	0
	1<br>Oglab05g04170.2 0.048 0.567 0.519 0.114 1<br>Oglab07g07650.1 0.62 0.997 0.377 0.002 0.778	1<br>Osjap01g04780.1 0.62 1 0.38 0.002 1<br>Osjap05g04880.1 0.048 0.567 0.519 0.008 1	1<br>Osind01g04880.1 0.62 1 0.38 0.002 1<br>Osind05g04760.1 0.048 0.567 0.519 0.008 1	1<br>Orufi01g04370.1 0.62 1 0.38 0.002 1<br>Orufi05g04740.1 0.048 0.567 0.519 0.008 1	1<br>Obart05g04510.1 0.036 0.567 0.531 0.012 1<br>Obart07g07920.1 0.62 0.997 0.377 0.002 0.778	1<br>Olong_014225-R1 0.044 0.563 0.519 0.001 0.984<br>Olong_014227-R1 0.62 1 0.38 0.002 1	1<br>Opunc01g03980.1 0.62 1 0.38 0.002 1<br>Opunc07g16230.1 0.042 0.556 0.514 0.014 0.972	1<br>Obrac01g03070.1 0.62 1 0.38 0.002 1<br>Obrac05g03450.2 0.039 0.567 0.528 0.003 1	1<br>Lperr01g03770.1 0.62 1 0.38 0.002 1<br>Lperr01g03780.1 0.039 0.561 0.522 0.002 0.979	1<br>AT5G01660.1 0.053 0.556 0.503 0.015 0.97<br>AT5G64190.1 0.62 0.997 0.377 0.002 0.998
Oglab01g06260.1	1<br>Oglab01g06260.1 0.001 1 0.999 0.001 1	0	0	0	0	0	0	0	0	0
	1<br>Oglab05g25190.3 0.025 0.527 0.502 0.041 0.991<br>Oglab06g15610.1 0.555 0.99 0.435 0.132 1	1<br>Osjap01g08460.1 0.561 0.991 0.43 0.03 0.997<br>Osjap05g30490.5 0.025 0.552 0.527 0.078 0.969	1<br>Osind01g08290.1 0.528 1 0.472 0.003 1<br>Osind05g30870.1 0.024 0.527 0.503 0.029 0.989	1<br>Orufi01g07850.1 0.021 0.536 0.515 0.039 0.998<br>Orufi06g18220.1 0.555 0.99 0.435 0.132 1	1<br>Obart01g07310.1 0.528 0.996 0.468 0.002 0.908<br>Obart05g27220.1 0.025 0.521 0.496 0.085 0.982	1<br>Olong_012629-R1 0.024 0.489 0.465 0.034 0.997<br>Olong_026782-R1 0.525 0.991 0.466 0.002 0.995	1<br>Opunc01g07000.1 0.53 0.977 0.447 0.032 0.987<br>Opunc05g24760.1 0.025 0.521 0.496 0.034 0.991	1<br>Obrac01g17790.1 0.024 0.519 0.495 0.078 0.962<br>Obrac05g15110.1 0.528 0.991 0.463 0.003 0.992	1<br>Lperr01g06950.1 0.561 0.995 0.434 0.078 1<br>Lperr05g22820.1 0.024 0.521 0.497 0.071 0.976	1<br>AT1G15000.1 0.024 0.531 0.507 0.07 0.977<br>AT1G28600.1 0.533 1 0.467 0.025 0.957
Oglab01g06470.1	1<br>Oglab01g06470.1 0.001 1 0.999 0.001 1	1<br>Osjap01g08660.1 0.001 1 0.999 0.001 1	1<br>Osind01g08490.1 0.001 1 0.999 0.001 1	1<br>Orufi01g08070.1 0.001 1 0.999 0.001 1	1<br>Obart01g07430.1 0.02 1 0.98 0.013 1	1<br>Olong_011567-R1 0.056 1 0.944 0.001 1	0	0	0	0
	1<br>Oglab01g21070.2 0.1 0.581 0.481 0.249 0.983<br>Oglab05g00530.1 0.647 0.997 0.35 0.146 1	1<br>Osjap01g29230.2 0.1 0.581 0.481 0.25 0.983<br>Osjap05g00560.2 0.647 0.997 0.35 0.146 1	1<br>Osind01g30690.1 0.1 0.581 0.481 0.267 0.982<br>Osind05g00540.1 0.647 0.997 0.35 0.126 1	1<br>Orufi01g27950.1 0.1 0.581 0.481 0.25 0.983<br>Orufi01g48560.2 0.647 0.98 0.333 0.085 0.984	1<br>Obart01g25090.1 0.1 0.581 0.481 0.219 0.982<br>Obart05g00540.1 0.647 0.997 0.35 0.146 1	0<br>Olong_003039-R1 0.647 0.997 0.35 0.128 1<br>Olong_003692-R2 0.139 0.581 0.442 0.244 0.979	1<br>Opunc01g07170.1 0.001 0.582 0.581 0.001 0.984<br>Opunc05g00510.2 0.647 0.997 0.35 0.15 1	1<br>Obrac01g05760.3 0.583 1 0.417 0.082 1<br>Obrac01g05770.3 0.001 0.582 0.581 0.001 0.985	1<br>Lperr01g07030.1 0.583 1 0.417 0.018 1<br>Lperr01g07040.2 0.001 0.582 0.581 0.054 0.989	1<br>AT1G54350.1 0.585 0.993 0.408 0.084 0.975<br>AT5G53430.1 0.071 0.581 0.51 0.164 0.984
Oglab01g37540.3	1<br>Oglab01g37540.1 0.002 0.969 0.967 0.002 0.971<br>Oglab01g37540.2 0.002 0.969 0.967 0.002 0.97<br>Oglab01g37540.3 0.002 1 0.998 0.002 1<br>Oglab01g37540.4 0.002 0.969 0.967 0.003 0.959	0	0	0	0	0	0	0	0	0
	1<br>Oglab02g03680.1 0.257 0.969 0.712 0.038 0.962<br>Oglab05g11700.1 0.002 0.215 0.213 0.005 0.575	1<br>Osjap02g03900.1 0.257 0.969 0.712 0.058 0.963<br>Osjap05g14440.1 0.002 0.215 0.213 0.005 0.575	1<br>Osind01g50140.1 0.002 0.218 0.216 0.008 0.829<br>Osind02g04170.1 0.257 0.969 0.712 0.036 0.962	1<br>Orufi01g46330.1 0.358 0.969 0.611 0.004 0.947<br>Orufi05g13610.1 0.002 0.215 0.213 0.005 0.575	1<br>Obart01g42890.1 0.358 0.969 0.611 0.004 0.946<br>Obart05g12620.1 0.002 0.215 0.213 0.005 0.575	0<br>Olong_010223-R1 0.004 0.224 0.22 0.014 0.53<br>Olong_014935-R1 0.259 0.527 0.268 0.336 0.935<br>Olong_014940-R4 0.533 0.782 0.249 0.02 0.141	1<br>Opunc02g02910.2 0.272 0.941 0.669 0.028 0.92<br>Opunc05g10670.1 0.002 0.218 0.216 0.005 0.58	1<br>Obrac02g02690.2 0.362 1 0.638 0.015 1<br>Obrac05g08200.1 0.002 0.228 0.226 0.008 0.953	0<br>Lperr02g03270.1 0.358 0.941 0.583 0.003 0.907<br>Lperr05g10020.1 0.002 0.211 0.209 0.005 0.608	0<br>AT2G28500.1 0.01 0.272 0.262 0.233 0.832<br>AT5G24630.6 0.636 0.868 0.232 0.599 0.857

Oglab02g22130.2	1<br>Oglab02g22130.2 0.001 1 0.999 0.001 1	1<br>Osjap02g26750.1 0.001 0.969 0.968 0.001 0.994<br>Osjap02g26750.2 0.001 0.969 0.968 0.001 0.994<br>Osjap02g26750.4 0.001 0.969 0.968 0.001 0.799	1<br>Osind02g26640.2 0.001 1 0.999 0.001 1	1<br>Orufi02g24900.1 0.011 0.974 0.963 0.06 0.859<br>Orufi02g24900.3 0.001 0.974 0.973 0.001 0.74<br>Orufi02g24900.4 0.001 0.974 0.973 0.001 0.737	1<br>Obart02g23460.2 0.001 1 0.999 0.001 1	0	1<br>Opunc02g21410.1 0.011 0.999 0.988 0.116 0.996<br>Opunc02g21410.2 0.011 0.999 0.988 0.114 0.996<br>Opunc02g21410.3 0.011 0.999 0.988 0.115 0.996<br>Opunc02g21410.4 0.011 0.999 0.988 0.114 0.996	0	1<br>Lperr02g18970.1 0.011 0.997 0.986 0.032 0.996	0
	1<br>Oglab02g21950.1 0.475 0.999 0.524 0.073 0.995<br>Oglab02g22110.2 0.011 0.459 0.448 0.532 0.98	1<br>Osjap02g26590.1 0.475 0.999 0.524 0.033 0.999<br>Osjap02g26750.5 0.011 0.459 0.448 0.238 0.973	1<br>Osind02g26450.1 0.475 0.999 0.524 0.025 0.997<br>Osind02g26620.1 0.011 0.459 0.448 0.495 0.981	1<br>Orufi02g24730.1 0.475 0.994 0.519 0.031 0.996<br>Orufi02g24880.2 0.011 0.459 0.448 0.18 0.97	1<br>Obart02g23290.1 0.475 0.999 0.524 0.029 0.999<br>Obart02g23440.1 0.011 0.459 0.448 0.492 0.981	0<br>Olong_024345-R1 0.011 0.474 0.463 0.442 0.991	1<br>Opunc02g21230.1 0.479 0.976 0.497 0.003 0.934<br>Opunc02g21240.1 0.011 0.461 0.45 0.121 0.972	1<br>Obrac02g16670.1 0.549 0.973 0.424 0.076 0.995<br>Obrac02g16700.1 0.011 0.461 0.45 0.114 0.947	1<br>Lperr02g18740.1 0.485 0.997 0.512 0.056 0.991<br>Lperr02g19120.1 0.011 0.484 0.473 0.183 0.978	1<br>AT4G31860.1 0.014 0.45 0.436 0.123 0.989<br>AT5G43080.1 0.626 1 0.374 0.194 0.932
Oglab02g33340.1	1<br>Oglab02g33340.1 0.004 1 0.996 0.004 1	1<br>Osjap02g40310.1 0.042 0.973 0.931 0.432 0.926<br>Osjap02g40340.1 0.042 1 0.958 0.361 1<br>Osjap02g40370.1 0.042 0.973 0.931 0.267 0.726	1<br>Osind02g39860.1 0.042 0.977 0.935 0.257 0.659<br>Osind02g39860.2 0.069 0.977 0.908 0.058 0.571<br>Osind02g39860.3 0.069 0.973 0.904 0.061 0.631<br>Osind02g39860.4 0.069 0.973 0.904 0.066 0.679	1<br>Orufi02g37680.1 0.069 0.989 0.92 0.067 0.86<br>Orufi02g37680.10 0.069 0.989 0.92 0.091 0.811<br>Orufi02g37680.2 0.069 0.989 0.92 0.07 0.854<br>Orufi02g37680.3 0.069 0.989 0.92 0.076 0.895<br>Orufi02g37680.4 0.069 0.989 0.92 0.073 0.849<br>Orufi02g37680.5 0.069 0.989 0.92 0.076 0.841<br>Orufi02g37680.6 0.069 0.989 0.92 0.088 0.879<br>Orufi02g37680.7 0.069 0.973 0.904 0.079 0.855<br>Orufi02g37680.9 0.069 0.973 0.904 0.086 0.843	1<br>Obart02g36180.1 0.069 0.989 0.92 0.061 0.778<br>Obart02g36180.2 0.069 0.989 0.92 0.073 0.734<br>Obart02g36180.3 0.069 0.989 0.92 0.076 0.724	0	0	0	0	0
	1<br>Oglab02g34400.1 0.483 0.992 0.509 0.434 0.804<br>Oglab03g25400.1 0.073 0.41 0.337 0.475 0.707	1<br>Osjap02g41540.1 0.483 0.992 0.509 0.434 0.804<br>Osjap05g23970.1 0.061 0.387 0.326 0.669 0.821	1<br>Osind02g41040.1 0.483 0.992 0.509 0.459 0.813<br>Osind05g24480.1 0.061 0.387 0.326 0.588 0.777	0<br>Orufi02g37680.8 0.372 0.989 0.617 0.159 0.801	1<br>Obart02g37290.1 0.483 0.992 0.509 0.434 0.804<br>Obart05g21320.1 0.061 0.387 0.326 0.588 0.777	1<br>Olong_011081-R1 0.479 1 0.521 0.05 1<br>Olong_025373-R1 0.073 0.41 0.337 0.236 0.574	1<br>Opunc02g34450.1 0.483 0.992 0.509 0.432 0.803<br>Opunc05g18980.1 0.061 0.383 0.322 0.588 0.771	1<br>Obrac02g27500.6 0.483 0.992 0.509 0.432 0.803<br>Obrac05g14470.1 0.061 0.383 0.322 0.588 0.771	1<br>Lperr02g30750.1 0.483 0.992 0.509 0.453 0.811<br>Lperr04g17770.1 0.038 0.383 0.345 0.48 0.678	1<br>AT1G16330.1 0.038 0.414 0.376 0.701 0.873<br>AT2G20360.1 0.483 0.992 0.509 0.423 0.799
Oglab03g00390.1	1<br>Oglab03g00390.1 0.001 1 0.999 0.001 1	1<br>Osjap03g00680.1 0.001 1 0.999 0.001 1<br>Osjap03g00680.2 0.001 1 0.999 0.001 1<br>Osjap03g00680.3 0.001 1 0.999 0.001 1	1<br>Osind03g00740.1 0.001 0.994 0.993 0.001 0.996	1<br>Orufi03g12660.1 0.001 1 0.999 0.001 1<br>Orufi03g12660.2 0.001 1 0.999 0.001 1	1<br>Obart03g00550.1 0.001 0.994 0.993 0.001 0.996	0	0	0	0	0
	1<br>Oglab02g34920.1 0.058 0.634 0.576 0.131 1<br>Oglab09g02040.2 0.696 1 0.304 0.07 1	1<br>Osjap02g42090.1 0.049 0.634 0.585 0.065 1<br>Osjap04g17560.1 0.696 0.998 0.302 0.466 0.996	1<br>Osind02g41670.1 0.058 0.634 0.576 0.131 1<br>Osind03g02220.1 0.652 1 0.348 0.015 1	1<br>Orufi02g39350.1 0.058 0.634 0.576 0.131 1<br>Orufi09g02220.1 0.696 1 0.304 0.072 1	1<br>Obart03g02140.1 0.652 1 0.348 0.013 1<br>Obart09g20260.1 0.152 0.634 0.482 0.12 1	0<br>Olong_013175-R1 0.253 0.906 0.653 0.002 0.961	1<br>Opunc03g00470.1 0.001 0.73 0.729 0.002 0.996<br>Opunc11g04590.1 0.783 0.998 0.215 0.023 1	0<br>Obrac02g10260.1 0.736 0.999 0.263 0.286 0.995<br>Obrac02g27970.1 0.128 0.634 0.506 0.105 1	1<br>Lperr03g00570.1 0.001 0.634 0.633 0.002 1<br>Lperr12g09110.1 0.699 0.998 0.299 0.295 0.967	0<br>AT5G04460.1 0.017 0.634 0.617 0.081 1
Oglab03g12120.1	1<br>Oglab03g12120.1 0.001 1 0.999 0.001 1	1<br>Osjap03g13100.1 0.028 1 0.972 0.001 1	1<br>Osind03g12930.1 0.001 1 0.999 0.001 1	0	1<br>Obart03g12120.1 0.001 1 0.999 0.001 1	0	0	0	0	0
	1<br>Oglab06g01620.1 0.642 0.998 0.356 0.212 0.912<br>Oglab12g00800.1 0.114 0.633 0.519 0.483 0.983	1<br>Osjap04g16240.1 0.642 0.998 0.356 0.067 0.921<br>Osjap12g01140.1 0.109 0.633 0.524 0.498 0.981	1<br>Osind04g14790.1 0.642 0.998 0.356 0.013 0.911<br>Osind12g01290.1 0.109 0.633 0.524 0.49 0.981	1<br>Orufi06g01460.3 0.642 0.998 0.356 0.212 0.912<br>Orufi12g01130.1 0.109 0.633 0.524 0.498 0.981	1<br>Obart04g15560.1 0.64 0.998 0.358 0.146 0.877<br>Obart11g01060.1 0.114 0.633 0.519 0.471 0.982	1<br>Olong_007714-R9 0.642 1 0.358 0.002 1<br>Olong_007715-R1 0.125 0.633 0.508 0.007 0.965	1<br>Opunc03g11680.1 0.028 0.633 0.605 0.001 0.971<br>Opunc04g11270.1 0.642 0.998 0.356 0.067 0.921	1<br>Obrac03g09720.1 0.025 0.633 0.608 0.036 0.971<br>Obrac04g09430.1 0.64 0.998 0.358 0.014 0.863	1<br>Lperr03g10720.1 0.028 0.633 0.605 0.001 0.934<br>Lperr03g17470.1 0.633 0.998 0.365 0.119 0.828	1<br>AT3G46740.1 0.122 0.633 0.511 0.158 0.971<br>AT3G53360.1 0.672 0.998 0.326 0.09 0.934
Oglab03g30830.1	1<br>Oglab03g30830.1 0.001 1 0.999 0.001 1	1<br>Osjap03g34330.1 0.001 1 0.999 0.001 1	1<br>Osind03g35550.1 0.001 1 0.999 0.039 1	1<br>Orufi03g33060.1 0.001 1 0.999 0.001 1	1<br>Obart03g31750.1 0.001 1 0.999 0.046 1	0	0	0	0	0
	1<br>Oglab03g34460.1 0.629 0.997 0.368 0.016 0.993	1<br>Osjap03g39110.1 0.652 1 0.348 0.036 1	1<br>Osind01g46540.1 0.652 0.999 0.347 0.065 0.994	1<br>Orufi10g04850.1 0.029 0.583 0.554 0.093 0.963	1<br>Obart03g36200.1 0.652 1 0.348 0.016 0.997	1<br>Olong_014796-R1 0.001 0.495 0.494 0.002 0.95	1<br>Opunc01g42030.1 0.652 1 0.348 0.28 0.872	1<br>Obrac02g16280.1 0.646 0.982 0.336 0.063 0.949	1<br>Lperr03g26400.1 0.001 0.6 0.599 0.002 0.995	0<br>AT1G70730.3 0.001 0.6 0.599 0.118 0.995

	Oglab10g04340.1 0.012 0.583 0.571 0.1 0.963	Osjap10g05370.1 0.012 0.583 0.571 0.1 0.963	Osind10g03770.1 0.012 0.583 0.571 0.11 0.96	Orufi11g12520.1 0.653 1 0.347 0.038 0.991	Obart10g04600.1 0.013 0.583 0.57 0.106 0.962	Olong_024872-R1 0.648 1 0.352 0.01 1	Opunc03g28940.2 0.001 0.6 0.599 0.002 0.995	Obrac03g23440.1 0.001 0.6 0.599 0.002 0.995	Lperr03g30520.1 0.644 1 0.356 0.003 0.997	
Oglab03g39920.1	1<br>Oglab03g39920.1 0.001 1 0.999 0.001 1	0	0	0	0	0	0	0	0	0
	1<br>Oglab02g12900.1 0.004 0.58 0.576 0.05 0.992<br>Oglab05g15580.1 0.616 0.996 0.38 0.208 0.946	1<br>Osjap02g14350.1 0.005 0.58 0.575 0.13 0.993<br>Osjap05g18940.1 0.616 0.996 0.38 0.208 0.946	1<br>Osind02g14610.1 0.005 0.58 0.575 0.13 0.993<br>Osind05g19660.1 0.616 0.996 0.38 0.208 0.946	1<br>Orufi01g35570.1 0.005 0.583 0.578 0.14 0.975<br>Orufi05g17840.1 0.616 0.996 0.38 0.208 0.946	1<br>Obart02g13170.1 0.005 0.58 0.575 0.13 0.993<br>Obart06g08490.1 0.619 1 0.381 0.002 1	1<br>Olong_004931-R1 0.621 1 0.379 0.184 0.927<br>Olong_023965-R1 0.005 0.58 0.575 0.13 0.993	1<br>Opunc02g11640.1 0.005 0.581 0.576 0.108 0.996<br>Opunc05g03040.1 0.621 1 0.379 0.181 0.929	1<br>Obrac02g12790.2 0.005 0.579 0.574 0.122 0.999<br>Obrac06g06500.1 0.591 0.997 0.406 0.026 0.978	1<br>Lperr01g27850.2 0.005 0.579 0.574 0.126 0.999<br>Lperr02g26310.1 0.581 0.997 0.416 0.038 0.979	1<br>AT1G65630.1 0.586 0.995 0.409 0.091 0.948<br>AT4G14990.1 0.005 0.576 0.571 0.142 0.995
Oglab04g27220.1	1<br>Oglab04g27220.1 0.001 1 0.999 0.001 1	0	0	0	0	0	0	0	0	0
	1<br>Oglab02g23520.1 0.544 0.999 0.455 0.028 0.993<br>Oglab06g09240.2 0.001 0.401 0.4 0.072 0.816	1<br>Osjap02g28270.1 0.544 0.999 0.455 0.034 0.993<br>Osjap04g33340.1 0.001 0.519 0.518 0.14 0.994	1<br>Osind02g04720.1 0.532 0.998 0.466 0.017 0.997<br>Osind04g32550.1 0.001 0.519 0.518 0.001 0.994	1<br>Orufi02g26250.1 0.544 0.999 0.455 0.034 0.993<br>Orufi04g30900.1 0.001 0.485 0.484 0.14 0.957	1<br>Obart02g04210.1 0.532 0.999 0.467 0.065 0.998<br>Obart04g29190.4 0.001 0.519 0.518 0.001 0.994	1<br>Olong_002807-R4 0.001 0.485 0.484 0.001 0.953<br>Olong_024919-R1 0.558 0.998 0.44 0.069 0.989	1<br>Opunc02g00670.1 0.545 1 0.455 0.043 0.999<br>Opunc04g26710.1 0.001 0.485 0.484 0.001 0.948	1<br>Obrac04g20580.2 0.001 0.509 0.508 0.017 0.994<br>Obrac06g17870.1 0.547 0.998 0.451 0.051 0.992	1<br>Lperr04g24690.2 0.001 0.489 0.488 0.115 0.915<br>Lperr07g00420.1 0.527 0.998 0.471 0.021 1	0<br>AT2G02220.1 0.551 0.998 0.447 0.047 1
Oglab05g03490.1	1<br>Oglab05g03490.1 0.001 1 0.999 0.001 1<br>Oglab05g03490.2 0.001 1 0.999 0.001 1	1<br>Osjap05g04120.1 0.001 1 0.999 0.001 1<br>Osjap05g04120.2 0.001 1 0.999 0.001 1	1<br>Osind05g04070.1 0.008 1 0.992 0.001 1<br>Osind05g04070.2 0.008 1 0.992 0.001 1	1<br>Orufi05g03950.1 0.001 1 0.999 0.001 1<br>Orufi05g03950.2 0.001 1 0.999 0.001 1	1<br>Obart05g03830.1 0.001 1 0.999 0.001 1<br>Obart05g03830.2 0.001 1 0.999 0.001 1	1<br>Olong_016739-R1 0.074 1 0.926 0.001 1	1<br>Opunc05g03540.1 0.001 1 0.999 0.001 1<br>Opunc05g03540.2 0.001 1 0.999 0.001 1	1<br>Obrac05g02930.1 0.074 1 0.926 0.001 1	0	0
	1<br>Oglab01g19490.1 0.09 0.723 0.633 0.066 0.996<br>Oglab03g14060.1 0.79 1 0.21 0.03 1	1<br>Osjap01g27410.1 0.09 0.723 0.633 0.066 0.996<br>Osjap03g15140.5 0.743 1 0.257 0.061 1	1<br>Osind01g28630.1 0.09 0.723 0.633 0.066 0.996<br>Osind03g15230.1 0.79 1 0.21 0.03 1	1<br>Orufi01g26130.1 0.09 0.723 0.633 0.066 0.996<br>Orufi03g14720.1 0.79 1 0.21 0.03 1	1<br>Obart01g23310.1 0.09 0.723 0.633 0.066 0.996<br>Obart03g14050.1 0.79 1 0.21 0.03 1	1<br>Olong_003347-R2 0.09 0.723 0.633 0.047 0.996<br>Olong_009943-R1 0.79 1 0.21 0.03 1	1<br>Opunc01g23460.1 0.09 0.723 0.633 0.064 0.996<br>Opunc03g13590.1 0.79 1 0.21 0.002 0.545	1<br>Obrac01g17590.1 0.09 0.723 0.633 0.067 0.996<br>Obrac03g11330.1 0.79 1 0.21 0.03 1	0<br>Lperr05g03460.1 0.001 0.721 0.72 0.001 0.993	1<br>AT2G47580.1 0.775 1 0.225 0.008 1<br>AT3G16630.1 0.003 0.721 0.718 0.031 0.992
Oglab05g03490.2	1<br>Oglab05g03490.1 0.001 1 0.999 0.001 1<br>Oglab05g03490.2 0.001 1 0.999 0.001 1	1<br>Osjap05g04120.1 0.001 1 0.999 0.001 1<br>Osjap05g04120.2 0.001 1 0.999 0.001 1	1<br>Osind05g04070.1 0.008 1 0.992 0.001 1<br>Osind05g04070.2 0.008 1 0.992 0.001 1	1<br>Orufi05g03950.1 0.001 1 0.999 0.001 1<br>Orufi05g03950.2 0.001 1 0.999 0.001 1	1<br>Obart05g03830.1 0.001 1 0.999 0.001 1<br>Obart05g03830.2 0.001 1 0.999 0.001 1	1<br>Olong_016739-R1 0.073 1 0.927 0.001 1	1<br>Opunc05g03540.1 0.001 1 0.999 0.001 1<br>Opunc05g03540.2 0.001 1 0.999 0.001 1	1<br>Obrac05g02930.1 0.073 1 0.927 0.001 1	0	0
	1<br>Oglab01g19490.1 0.089 0.726 0.637 0.066 0.996<br>Oglab03g14060.1 0.792 1 0.208 0.03 1	1<br>Osjap01g27410.1 0.089 0.726 0.637 0.066 0.996<br>Osjap03g15140.5 0.747 1 0.253 0.061 1	1<br>Osind01g28630.1 0.089 0.726 0.637 0.066 0.996<br>Osind03g15230.1 0.792 1 0.208 0.03 1	1<br>Orufi01g26130.1 0.089 0.726 0.637 0.066 0.996<br>Orufi03g14720.1 0.792 1 0.208 0.03 1	1<br>Obart01g23310.1 0.089 0.726 0.637 0.066 0.996<br>Obart03g14050.1 0.792 1 0.208 0.03 1	1<br>Olong_003347-R2 0.089 0.726 0.637 0.047 0.996<br>Olong_009943-R1 0.792 1 0.208 0.03 1	1<br>Opunc01g23460.1 0.089 0.726 0.637 0.064 0.996<br>Opunc03g13590.1 0.792 1 0.208 0.002 0.545	1<br>Obrac01g17590.1 0.089 0.726 0.637 0.067 0.996<br>Obrac03g11330.1 0.792 1 0.208 0.03 1	0<br>Lperr05g03460.1 0.001 0.725 0.724 0.001 0.993	1<br>AT2G47580.1 0.778 1 0.222 0.008 1<br>AT3G16630.1 0.003 0.725 0.722 0.031 0.992
Oglab05g04140.1	1<br>Oglab05g04140.1 0.002 1 0.998 0.002 1	0	0	0	1<br>Obart05g04470.1 0.002 1 0.998 0.002 1	0	0	0	0	0
	1<br>Oglab05g02840.1 0.362 0.977 0.615 0.395 0.582<br>Oglab06g15820.1 0.014 0.359 0.345 0.033 0.973	1<br>Osjap05g04830.1 0.002 0.35 0.348 0.006 0.86<br>Osjap08g03110.1 0.359 0.993 0.634 0.031 0.944	0<br>Osind05g03400.1 0.274 0.945 0.671 0.792 0.975	1<br>Orufi05g04690.1 0.002 0.35 0.348 0.006 0.86<br>Orufi05g04700.1 0.35 1 0.65 0.212 1	1<br>Obart05g03110.1 0.362 0.977 0.615 0.395 0.582<br>Obart06g05940.1 0.014 0.35 0.336 0.048 0.458	1<br>Olong_002683-R1 0.359 0.993 0.634 0.031 0.945<br>Olong_015421-R1 0.002 0.35 0.348 0.01 1	1<br>Opunc05g03750.1 0.362 0.984 0.622 0.659 0.972<br>Opunc05g04100.1 0.002 0.35 0.348 0.006 0.878	0<br>Obrac05g03390.1 0.346 0.986 0.64 0.441 0.983	1<br>Lperr03g03510.1 0.023 0.35 0.327 0.073 0.593<br>Lperr05g03760.1 0.5 0.984 0.484 0.279 0.955	0<br>AT3G16720.1 0.002 0.35 0.348 0.059 0.523
Oglab05g04450.2	1<br>Oglab05g04450.1 0.001 1 0.999 0.001 1<br>Oglab05g04450.2 0.001 1 0.999 0.001 1	0	1<br>Osind05g05280.1 0.181 0.99 0.809 0.001 0.981	0	1<br>Obart05g05010.1 0.001 1 0.999 0.01 1<br>Obart05g05010.2 0.001 1 0.999 0.011 1<br>Obart05g05010.3 0.001 1 0.999 0.011 1	0	0	0	0	0
	1	1	1	1	1	1	0	1	1	0

	Oglab05g04600.1 0.677 0.999 0.322 0.044 0.997<br>Oglab05g08330.1 0.001 0.628 0.627 0.001 0.997	Osjap05g10230.1 0.001 0.629 0.628 0.001 0.999<br>Osjap07g20060.1 0.666 0.999 0.333 0.002 0.991	Osind05g11170.1 0.665 0.999 0.334 0.02 0.998<br>Osind05g11190.1 0.001 0.629 0.628 0.001 0.999	Orufi05g05240.1 0.674 0.998 0.324 0.034 0.995<br>Orufi05g09830.2 0.001 0.629 0.628 0.001 0.999	Obart05g09170.2 0.001 0.628 0.627 0.001 0.997<br>Obart07g17280.1 0.667 0.995 0.328 0.002 0.977	Olong_008766-R1 0.666 0.998 0.332 0.005 0.995<br>Olong_015082-R1 0.001 0.625 0.624 0.001 0.993	Opunc05g04580.1 0.001 0.793 0.792 0.001 1	Obrac05g03760.1 0.664 0.998 0.334 0.003 0.995<br>Obrac11g11360.1 0.001 0.629 0.628 0.001 0.999	Lperr05g02380.1 0.67 1 0.33 0.003 1<br>Lperr11g15610.1 0.001 0.629 0.628 0.001 0.997	AT2G27090.1 0.001 0.622 0.621 0.001 0.985
Oglab05g07040.1	1<br>Oglab05g07040.1 0.004 1 0.996 0.004 1	0	0	1<br>Orufi05g08440.1 0.004 0.917 0.913 0.003 0.663	0	0	0	0	0	0
	1<br>Oglab01g16080.1 0.737 0.974 0.237 0.041 0.684<br>Oglab05g07020.1 0.004 0.718 0.714 0.004 0.838	1<br>Osjap05g08680.1 0.004 0.718 0.714 0.004 0.964<br>Osjap08g02020.1 0.737 0.974 0.237 0.02 0.34	1<br>Osind05g08590.1 0.004 0.718 0.714 0.004 0.964<br>Osind05g08610.1 0.756 1 0.244 0.015 1	0<br>Orufi01g21290.1 0.737 0.974 0.237 0.041 0.684<br>Orufi05g08430.1 0.086 0.62 0.534 0.156 0.574	1<br>Obart05g07850.1 0.004 0.718 0.714 0.004 0.87<br>Obart05g07870.1 0.756 1 0.244 0.015 1	1<br>Olong_012066-R1 0.004 0.711 0.707 0.004 0.964<br>Olong_015117-R1 0.759 0.974 0.215 0.009 0.523	1<br>Opunc02g01060.1 0.748 0.974 0.226 0.029 0.248<br>Opunc05g07190.1 0.004 0.711 0.707 0.003 0.978	0<br>Obrac07g06840.1 0.053 0.688 0.635 0.03 0.911	1<br>Lperr07g09890.1 0.03 0.643 0.613 0.006 0.891<br>Lperr08g02000.1 0.737 0.974 0.237 0.028 0.469	0<br>AT4G36020.1 0.752 0.977 0.225 0.043 0.244
Oglab05g23160.1	1<br>Oglab05g23160.1 0.001 1 0.999 0.001 1	1<br>Osjap05g28230.1 0.001 1 0.999 0.001 1	1<br>Osind05g28520.1 0.001 1 0.999 0.001 1	1<br>Orufi05g26870.1 0.001 1 0.999 0.001 1	1<br>Obart05g25070.1 0.001 1 0.999 0.001 1	0	1<br>Opunc05g22640.1 0.001 1 0.999 0.001 1	0	1<br>Lperr01g22990.1 0.001 1 0.999 0.027 1	0
	1<br>Oglab01g22740.1 0.028 0.451 0.423 0.061 0.995<br>Oglab01g22750.1 0.462 0.999 0.537 0.009 0.999	1<br>Osjap01g31140.1 0.007 0.451 0.444 0.093 0.995<br>Osjap01g31150.1 0.462 0.999 0.537 0.009 0.999	1<br>Osind01g32600.1 0.001 0.451 0.45 0.002 0.995<br>Osind01g32610.1 0.462 0.999 0.537 0.009 0.999	1<br>Orufi01g29880.1 0.001 0.451 0.45 0.075 0.995<br>Orufi01g29890.1 0.462 0.999 0.537 0.009 0.999	1<br>Obart01g26870.1 0.006 0.451 0.445 0.117 0.995<br>Obart01g26880.1 0.462 0.999 0.537 0.009 0.999	1<br>Olong_000367-R1 0.105 0.451 0.346 0.002 0.993<br>Olong_000368-R1 0.456 0.999 0.543 0.108 0.999	1<br>Opunc01g26820.1 0.049 0.451 0.402 0.002 0.994<br>Opunc01g26830.1 0.462 1 0.538 0.009 1	1<br>Obrac01g20400.1 0.048 0.451 0.403 0.006 0.994<br>Obrac05g17560.2 0.451 1 0.549 0.164 1	1<br>Lperr05g20850.2 0.467 1 0.533 0.002 1<br>Lperr05g20860.1 0.001 0.451 0.45 0.002 0.995	1<br>AT5G08690.1 0.012 0.451 0.439 0.034 0.995<br>AT5G51600.1 0.462 0.999 0.537 0.008 0.996
Oglab06g00840.1	1<br>Oglab06g00840.1 0.001 1 0.999 0.001 1	1<br>Osjap06g00940.1 0.04 1 0.96 0.085 1	0	1<br>Orufi06g00860.1 0.067 1 0.933 0.021 1	1<br>Obart06g00880.1 0.001 1 0.999 0.001 1	0	0	0	0	0
	1<br>Oglab06g00730.1 0.526 0.99 0.464 0.257 0.968<br>Oglab07g19660.1 0.033 0.458 0.425 0.142 0.938	1<br>Osjap06g00950.1 0.529 0.992 0.463 0.132 0.99<br>Osjap08g13790.1 0.051 0.458 0.407 0.005 1	1<br>Osind06g00910.1 0.526 1 0.474 0.068 1<br>Osind08g15520.1 0.051 0.458 0.407 0.005 1	1<br>Orufi06g00690.1 0.529 1 0.471 0.099 1<br>Orufi06g00860.2 0.001 0.445 0.444 0.005 0.98	1<br>Obart06g00690.1 0.529 1 0.471 0.099 1<br>Obart09g03180.1 0.021 0.458 0.437 0.145 1	0<br>Olong_023831-R1 0.053 0.583 0.53 0.001 0.373	1<br>Opunc06g00800.1 0.457 1 0.543 0.025 1<br>Opunc06g00830.1 0.05 0.457 0.407 0.006 1	1<br>Obrac06g00660.1 0.526 1 0.474 0.034 0.999<br>Obrac07g14790.1 0.058 0.503 0.445 0.093 0.948	1<br>Lperr01g40030.1 0.055 0.457 0.402 0.013 0.994<br>Lperr06g00740.1 0.531 0.999 0.468 0.078 0.997	0<br>AT4G34470.1 0.063 0.457 0.394 0.013 1
Oglab06g02870.1	1<br>Oglab06g02870.1 0.001 1 0.999 0.001 1	1<br>Osjap06g03080.1 0.001 1 0.999 0.001 1	0	1<br>Orufi06g02670.1 0.045 0.964 0.919 0.032 0.91	1<br>Obart06g02710.1 0.001 1 0.999 0.03 1	1<br>Olong_013693-R1 0.045 1 0.955 0.069 1	0	0	0	0
	1<br>Oglab01g27580.1 0.037 0.715 0.678 0.03 0.949<br>Oglab04g28300.1 0.767 0.968 0.201 0.466 0.888	1<br>Osjap01g37620.1 0.001 0.756 0.755 0.033 0.998<br>Osjap04g34350.1 0.767 0.968 0.201 0.466 0.888	1<br>Osind03g45110.1 0.04 0.75 0.71 0.141 0.984<br>Osind04g33620.1 0.767 0.968 0.201 0.438 0.875	0<br>Orufi01g36040.3 0.037 0.756 0.719 0.03 0.998	1<br>Obart01g06310.1 0.037 0.756 0.719 0.066 0.997<br>Obart04g30240.1 0.767 0.968 0.201 0.466 0.888	1<br>Olong_001861-R1 0.767 0.968 0.201 0.381 0.87<br>Olong_005208-R1 0.014 0.715 0.701 0.141 0.969	1<br>Opunc03g00730.1 0.001 0.756 0.755 0.004 1<br>Opunc04g27750.1 0.767 0.968 0.201 0.426 0.879	1<br>Obrac04g05640.6 0.014 0.705 0.691 0.262 0.995<br>Obrac04g21390.1 0.767 0.968 0.201 0.381 0.87	1<br>Lperr10g07060.1 0.04 0.722 0.682 0.115 0.964<br>Lperr12g00960.1 0.767 0.968 0.201 0.389 0.871	0<br>AT2G37990.1 0.767 0.968 0.201 0.425 0.893
Oglab06g21340.1	1<br>Oglab06g21340.1 0.002 1 0.998 0.002 1	0	0	0	0	0	0	0	0	0
	1<br>Oglab02g06010.1 0.01 0.597 0.587 0.147 0.863<br>Oglab05g08960.1 0.684 0.986 0.302 0.443 0.897	1<br>Osjap05g02420.1 0.692 0.966 0.274 0.167 0.443<br>Osjap06g27500.1 0.002 0.689 0.687 0.002 0.971	1<br>Osind05g02400.1 0.692 0.966 0.274 0.17 0.451<br>Osind06g26900.1 0.009 0.689 0.68 0.162 0.973	1<br>Orufi05g02370.1 0.692 0.966 0.274 0.167 0.443<br>Orufi06g25120.1 0.009 0.689 0.68 0.162 0.973	1<br>Obart05g02250.1 0.692 0.966 0.274 0.173 0.46<br>Obart06g23450.1 0.002 0.689 0.687 0.003 0.969	1<br>Olong_002211-R1 0.009 0.689 0.68 0.126 0.972<br>Olong_004663-R2 0.706 0.966 0.26 0.116 0.242	1<br>Opunc06g21170.1 0.009 0.689 0.68 0.236 0.976<br>Opunc08g00730.1 0.701 0.968 0.267 0.316 0.987	1<br>Obrac05g06640.1 0.72 0.986 0.266 0.346 0.873<br>Obrac08g07630.1 0.005 0.694 0.689 0.187 0.935	1<br>Lperr05g02030.1 0.692 0.966 0.274 0.158 0.439<br>Lperr06g19460.1 0.005 0.689 0.684 0.199 0.975	0<br>AT2G18940.1 0.73 0.986 0.256 0.749 0.901
Oglab06g22820.1	1<br>Oglab06g22820.1 0.005 1 0.995 0.005 1	0	0	0	1<br>Obart06g25430.1 0.005 1 0.995 0.004 1	0	0	0	0	0
	1<br>Oglab06g20540.1 0.005 0.675 0.67 0.625 0.997<br>Oglab09g12320.1 0.72 0.98 0.26 0.658 0.994	1<br>Osjap06g26550.1 0.005 0.465 0.46 0.698 0.988<br>Osjap06g29670.1 0.625 0.98 0.355 0.469 0.992	1<br>Osind06g29020.1 0.625 0.98 0.355 0.469 0.992<br>Osind06g29050.1 0.015 0.54 0.525 0.655 0.915	1<br>Orufi06g24280.1 0.005 0.525 0.52 0.654 0.959<br>Orufi06g27300.1 0.535 0.985 0.45 0.29 0.942	1<br>Obart06g22650.1 0.005 0.465 0.46 0.574 0.984<br>Obart06g25420.1 0.625 0.98 0.355 0.403 0.852	0<br>Olong_024432-R1 0.625 0.98 0.355 0.561 0.994	1<br>Opunc02g22800.1 0.015 0.525 0.51 0.695 0.966<br>Opunc06g23120.1 0.625 0.98 0.355 0.691 0.995	0<br>Obrac09g08010.1 0.72 0.98 0.26 0.586 0.992	0<br>Lperr09g09890.1 0.585 0.98 0.395 0.53 0.994	0<br>AT4G30380.1 0.73 0.97 0.24 0.585 0.984

Oglab06g24190.1	1<br>Oglab06g24190.1 0.001 1 0.999 0.001 1<br>Oglab06g24190.2 0.001 0.995 0.994 0.001 0.991	1<br>Osjap06g31210.1 0.001 0.995 0.994 0.008 1	1<br>Osind06g30430.1 0.001 0.995 0.994 0.008 1	1<br>Orufi06g28720.1 0.001 0.995 0.994 0.008 1	1<br>Obart06g26750.1 0.001 0.995 0.994 0.008 1	1<br>Olong_013658-R1 0.001 0.995 0.994 0.001 1	1<br>Opunc06g24460.1 0.001 0.995 0.994 0.008 1	0	1<br>Lperr06g22350.1 0.001 0.995 0.994 0.033 1	0
	1<br>Oglab02g02970.1 0.694 0.993 0.299 0.006 0.93<br>Oglab12g14250.1 0.001 0.62 0.619 0.001 0.743	1<br>Osjap02g03010.1 0.694 0.993 0.299 0.006 0.928<br>Osjap12g19610.1 0.001 0.62 0.619 0.001 0.675	1<br>Osind02g03310.1 0.694 0.993 0.299 0.006 0.928<br>Osind07g26390.1 0.001 0.59 0.589 0.074 0.935	1<br>Orufi02g02960.1 0.694 0.993 0.299 0.006 0.928<br>Orufi12g16970.1 0.001 0.62 0.619 0.001 0.674	1<br>Obart02g03050.1 0.694 0.993 0.299 0.006 0.93<br>Obart12g15260.1 0.001 0.602 0.601 0.001 0.686	1<br>Olong_017635-R1 0.694 0.993 0.299 0.006 0.93<br>Olong_024721-R1 0.01 0.608 0.598 0.05 0.932	1<br>Opunc02g02350.2 0.715 0.993 0.278 0.248 0.944<br>Opunc03g37840.1 0.001 0.622 0.621 0.047 0.706	1<br>Obrac02g02360.1 0.694 0.994 0.3 0.006 0.933<br>Obrac09g01040.1 0.001 0.591 0.59 0.026 0.872	1<br>Lperr02g02740.1 0.699 0.995 0.296 0.006 0.96<br>Lperr12g12430.1 0.001 0.602 0.601 0.001 0.854	1<br>AT3G10250.1 0.701 0.998 0.297 0.006 0.948<br>AT4G32430.1 0.005 0.61 0.605 0.051 0.976
Oglab06g24190.2	1<br>Oglab06g24190.1 0.001 0.991 0.99 0.001 0.995<br>Oglab06g24190.2 0.001 1 0.999 0.001 1	1<br>Osjap06g31210.1 0.001 0.991 0.99 0.008 1	1<br>Osind06g30430.1 0.001 0.991 0.99 0.008 1	1<br>Orufi06g28720.1 0.001 0.991 0.99 0.008 1	1<br>Obart06g26750.1 0.001 0.991 0.99 0.008 1	1<br>Olong_013658-R1 0.001 0.991 0.99 0.001 1	1<br>Opunc06g24460.1 0.001 0.991 0.99 0.008 1	0	1<br>Lperr06g22350.1 0.001 0.991 0.99 0.033 1	0
	1<br>Oglab02g02970.1 0.691 0.997 0.306 0.006 0.96<br>Oglab12g14250.1 0.001 0.617 0.616 0.001 0.743	1<br>Osjap02g03010.1 0.691 0.997 0.306 0.006 0.959<br>Osjap12g19610.1 0.001 0.617 0.616 0.001 0.675	1<br>Osind02g03310.1 0.691 0.997 0.306 0.006 0.959<br>Osind07g26390.1 0.001 0.587 0.586 0.074 0.935	1<br>Orufi02g02960.1 0.691 0.997 0.306 0.006 0.959<br>Orufi09g02510.1 0.001 0.589 0.588 0.014 0.762	1<br>Obart02g03050.1 0.691 0.997 0.306 0.006 0.96<br>Obart12g15260.1 0.001 0.617 0.616 0.001 0.686	1<br>Olong_017635-R1 0.691 0.997 0.306 0.006 0.96<br>Olong_024721-R1 0.01 0.605 0.595 0.05 0.932	1<br>Opunc02g02350.2 0.712 0.997 0.285 0.248 0.968<br>Opunc03g37840.1 0.001 0.619 0.618 0.047 0.706	1<br>Obrac02g02360.1 0.691 0.997 0.306 0.006 0.961<br>Obrac09g01040.1 0.001 0.588 0.587 0.026 0.872	1<br>Lperr02g02740.1 0.696 0.991 0.295 0.006 0.96<br>Lperr03g02630.1 0.001 0.584 0.583 0.105 0.84	1<br>AT3G10250.1 0.698 0.992 0.294 0.006 0.941<br>AT4G32430.1 0.005 0.607 0.602 0.051 0.976
Oglab06g25490.1	1<br>Oglab06g25490.1 0.001 1 0.999 0.001 1<br>Oglab09g15660.1 0.024 0.999 0.975 0.154 0.95	1<br>Osjap09g17800.1 0.024 1 0.976 0.113 0.974<br>Osjap09g17800.2 0.024 1 0.976 0.114 0.974<br>Osjap09g17800.3 0.024 0.997 0.973 0.187 0.947	1<br>Osind06g31620.1 0.001 1 0.999 0.001 1	1<br>Orufi06g29990.1 0.001 1 0.999 0.001 1<br>Orufi09g18790.1 0.024 1 0.976 0.107 0.97<br>Orufi09g18790.2 0.024 1 0.976 0.107 0.97<br>Orufi09g18790.3 0.024 1 0.976 0.108 0.969<br>Orufi09g18790.4 0.024 1 0.976 0.108 0.969<br>Orufi09g18790.5 0.024 0.976 0.952 0.17 0.937<br>Orufi09g18790.6 0.024 0.996 0.972 0.172 0.937	1<br>Obart06g28060.1 0.001 1 0.999 0.001 1<br>Obart09g17460.1 0.024 1 0.976 0.076 0.969<br>Obart09g17460.2 0.024 1 0.976 0.076 0.969	1<br>Olong_016700-R1 0.001 1 0.999 0.001 1	0	0	1<br>Lperr09g13380.1 0.084 0.982 0.898 0.166 0.951	0
	1<br>Oglab02g01250.1 0.066 0.406 0.34 0.221 0.93<br>Oglab10g06880.1 0.427 1 0.573 0.099 1	1<br>Osjap06g32830.1 0.427 1 0.573 0.085 1<br>Osjap06g32840.1 0.001 0.407 0.406 0.002 0.952	1<br>Osind04g24640.1 0.07 0.417 0.347 0.233 0.933<br>Osind06g30840.1 0.485 0.999 0.514 0.109 1	0<br>Orufi06g29150.4 0.485 0.999 0.514 0.122 1	1<br>Obart04g21770.1 0.07 0.417 0.347 0.232 0.933<br>Obart10g07310.1 0.427 1 0.573 0.099 1	1<br>Olong_000286-R1 0.066 0.406 0.34 0.258 0.928<br>Olong_026973-R2 0.427 1 0.573 0.099 1	1<br>Opunc06g25610.1 0.001 0.407 0.406 0.002 0.951<br>Opunc10g06480.1 0.427 0.998 0.571 0.098 0.984	1<br>Obrac06g20010.2 0.432 1 0.568 0.119 1<br>Obrac06g20020.1 0.001 0.407 0.406 0.002 0.952	1<br>Lperr02g22230.1 0.043 0.392 0.349 0.113 0.88<br>Lperr06g23510.1 0.402 1 0.598 0.074 1	1<br>AT1G76360.1 0.036 0.406 0.37 0.219 0.952<br>AT3G61130.1 0.465 0.999 0.534 0.207 1
Oglab06g25720.1	1<br>Oglab06g25720.1 0.001 1 0.999 0.001 1	0	0	0	0	0	0	0	0	0
	1<br>Oglab06g11430.1 0.001 0.629 0.628 0.001 0.997<br>Oglab06g12400.1 0.782 0.992 0.21 0.422 0.995	1<br>Osjap06g13090.1 0.004 0.67 0.666 0.003 0.966<br>Osjap06g14920.1 0.782 0.992 0.21 0.422 0.995	1<br>Osind06g13010.1 0.019 0.644 0.625 0.005 0.999<br>Osind06g14680.1 0.782 0.997 0.215 0.283 0.989	1<br>Orufi06g12040.1 0.004 0.67 0.666 0.003 0.966<br>Orufi06g13730.1 0.782 0.992 0.21 0.296 0.994	1<br>Obart06g11630.1 0.001 0.631 0.63 0.001 0.996<br>Obart06g12910.1 0.782 0.992 0.21 0.422 0.995	0<br>Olong_024681-R1 0.003 0.645 0.642 0.161 0.964	1<br>Opunc06g10960.1 0.004 0.67 0.666 0.003 0.97<br>Opunc06g12290.1 0.782 0.992 0.21 0.415 0.993	1<br>Obrac06g08260.2 0.001 0.646 0.645 0.037 0.965<br>Obrac08g03520.1 0.782 0.992 0.21 0.431 0.993	0<br>Lperr06g10400.1 0.001 0.632 0.631 0.002 0.983	0
Oglab07g20540.2	1<br>Oglab07g20540.1 0.001 1 0.999 0.001 1<br>Oglab07g20540.2 0.001 1 0.999 0.001 1	0	0	0	1<br>Obart07g23990.1 0.001 1 0.999 0.001 1	0	0	0	0	0
	1<br>Oglab05g21510.1 0.602 1 0.398 0.068 1<br>Oglab12g08980.2 0.019 0.597 0.578 0.031 0.953	1<br>Osjap06g10100.1 0.596 1 0.404 0.044 1<br>Osjap10g03880.1 0.028 0.583 0.555 0.047 0.967	1<br>Osind05g26020.2 0.602 1 0.398 0.068 1<br>Osind12g09130.1 0.031 0.597 0.566 0.009 0.945	1<br>Orufi05g24100.1 0.602 1 0.398 0.142 1<br>Orufi12g09900.2 0.031 0.597 0.566 0.057 0.953	1<br>Obart04g03800.1 0.59 1 0.41 0.022 1<br>Obart10g03330.1 0.031 0.583 0.552 0.052 0.967	0<br>Olong_008774-R1 0.577 1 0.423 0.006 1	1<br>Opunc10g02860.1 0.023 0.583 0.56 0.038 0.967<br>Opunc10g02870.1 0.59 1 0.41 0.025 0.989	1<br>Obrac07g07280.1 0.004 0.568 0.564 0.003 0.921<br>Obrac07g07970.1 0.578 1 0.422 0.013 1	1<br>Lperr05g10370.1 0.036 0.583 0.547 0.059 0.935<br>Lperr06g09490.1 0.59 1 0.41 0.024 1	0<br>AT2G37460.1 0.038 0.582 0.544 0.045 0.905

Oglab08g05640.1	1<br>Oglab08g05640.1 0.002 1 0.998 0.002 1	1<br>Osjap08g06650.1 0.002 1 0.998 0.002 1	1<br>Osind08g06790.1 0.002 1 0.998 0.002 1	1<br>Orufi08g06840.1 0.002 1 0.998 0.002 1	1<br>Obart08g06070.1 0.002 1 0.998 0.002 1	0	1<br>Opunc08g05850.1 0.047 1 0.953 0.068 1	0	0	0
	1<br>Oglab02g26360.1 0.348 0.996 0.648 0.01 0.994<br>Oglab04g16180.1 0.047 0.275 0.228 0.198 0.94	1<br>Osjap04g21220.1 0.047 0.275 0.228 0.21 0.936<br>Osjap09g20870.1 0.359 0.984 0.625 0.208 0.966	1<br>Osind04g20250.1 0.047 0.275 0.228 0.198 0.94<br>Osind09g20960.1 0.359 0.984 0.625 0.208 0.966	1<br>Orufi04g19250.2 0.047 0.275 0.228 0.21 0.936<br>Orufi09g21960.1 0.359 0.984 0.625 0.2 0.965	1<br>Obart02g28440.1 0.348 0.996 0.648 0.093 0.994<br>Obart04g17770.1 0.047 0.275 0.228 0.198 0.94	1<br>Olong_008030-R1 0.047 0.275 0.228 0.232 0.937<br>Olong_008031-R2 0.277 1 0.723 0.106 1	1<br>Opunc04g15550.1 0.047 0.275 0.228 0.206 0.938<br>Opunc04g18740.1 0.295 0.984 0.689 0.088 0.994	1<br>Obrac08g04780.1 0.277 1 0.723 0.098 1<br>Obrac08g04800.1 0.002 0.275 0.273 0.006 0.943	1<br>Lperr08g05690.1 0.277 1 0.723 0.1 1<br>Lperr08g05700.1 0.002 0.275 0.273 0.006 0.943	1<br>AT1G05310.1 0.33 0.982 0.652 0.183 0.99<br>AT4G30800.1 0.047 0.275 0.228 0.208 0.943
Oglab08g13140.1	1<br>Oglab08g13140.1 0.001 1 0.999 0.001 1	1<br>Osjap08g14980.1 0.001 0.819 0.818 0.347 0.856	0	0	0	0	0	0	0	0
	1<br>Oglab04g06580.1 0.001 0.443 0.442 0.399 0.772<br>Oglab08g13180.1 0.466 0.992 0.526 0.035 0.979	1<br>Osjap08g15020.1 0.466 0.992 0.526 0.035 0.979<br>Osjap12g14110.1 0.001 0.444 0.443 0.397 0.776	1<br>Osind12g11050.1 0.001 0.444 0.443 0.397 0.776<br>Osind12g18550.1 0.471 0.992 0.521 0.004 0.982	1<br>Orufi08g15830.1 0.466 0.992 0.526 0.004 0.982<br>Orufi12g12010.1 0.001 0.444 0.443 0.388 0.773	1<br>Obart08g14060.1 0.466 0.992 0.526 0.04 0.976<br>Obart12g10850.1 0.001 0.444 0.443 0.397 0.776	0<br>Olong_015986-R1 0.425 0.936 0.511 0.754 0.962	1<br>Opunc08g12640.1 0.459 0.992 0.533 0.004 0.981<br>Opunc12g09730.1 0.001 0.444 0.443 0.397 0.778	1<br>Obrac04g04430.1 0.001 0.443 0.442 0.399 0.772<br>Obrac08g09240.1 0.466 0.992 0.526 0.155 0.986	1<br>Lperr08g11630.1 0.451 0.992 0.541 0.002 0.987<br>Lperr12g09160.1 0.001 0.444 0.443 0.397 0.776	1<br>AT2G16050.1 0.471 0.992 0.521 0.004 0.975<br>AT4G10710.1 0.001 0.443 0.442 0.402 0.772
Oglab08g15520.1	1<br>Oglab01g06790.1 0.007 0.844 0.837 0.061 1<br>Oglab08g15520.1 0.007 1 0.993 0.007 1	1<br>Osjap01g09050.1 0.007 0.844 0.837 0.061 1	1<br>Osind01g08880.1 0.102 0.986 0.884 0.004 0.567	1<br>Orufi01g08450.1 0.007 0.973 0.966 0.026 0.487	1<br>Obart01g07700.1 0.007 0.844 0.837 0.061 1<br>Obart08g16670.1 0.007 0.993 0.986 0.007 0.993	1<br>Olong_003647-R1 0.007 0.83 0.823 0.061 0.985	0	0	0	0
	1<br>Oglab03g37410.1 0.714 1 0.286 0.049 0.141<br>Oglab11g05440.1 0.007 0.694 0.687 0.573 0.683	1<br>Osjap01g24690.1 0.007 0.694 0.687 0.104 0.321<br>Osjap07g09900.1 0.694 1 0.306 0.005 0.126	1<br>Osind07g08950.1 0.694 1 0.306 0.004 0.113<br>Osind11g05650.1 0.007 0.694 0.687 0.103 0.319	1<br>Orufi01g23650.1 0.007 0.694 0.687 0.106 0.307<br>Orufi07g08630.1 0.694 1 0.306 0.005 0.117	1<br>Obart07g08560.1 0.694 0.952 0.258 0.009 0.192<br>Obart11g05910.1 0.007 0.694 0.687 0.752 0.834	1<br>Olong_005787-R1 0.007 0.694 0.687 0.103 0.319<br>Olong_016241-R1 0.728 1 0.272 0.735 0.775	1<br>Opunc01g21000.1 0.007 0.694 0.687 0.114 0.334<br>Opunc07g08090.1 0.694 1 0.306 0.004 0.092	1<br>Obrac07g05110.1 0.728 0.993 0.265 0.077 0.151<br>Obrac11g03720.1 0.007 0.694 0.687 0.112 0.34	1<br>Lperr01g17850.1 0.007 0.694 0.687 0.105 0.313<br>Lperr07g08590.1 0.694 0.993 0.299 0.022 0.124	1<br>AT3G55700.1 0.728 0.973 0.245 0.015 0.093<br>AT4G38130.1 0.007 0.68 0.673 0.09 0.269
Oglab09g13510.1	1<br>Oglab09g13510.1 0.002 1 0.998 0.002 1<br>Oglab09g13510.2 0.002 1 0.998 0.002 1<br>Oglab09g13510.3 0.002 1 0.998 0.002 1<br>Oglab09g13510.4 0.002 1 0.998 0.002 1	1<br>Osjap09g15430.2 0.002 1 0.998 0.002 1<br>Osjap09g15430.3 0.002 1 0.998 0.002 1	1<br>Osind09g15570.1 0.002 1 0.998 0.002 1<br>Osind09g15570.2 0.002 1 0.998 0.002 1	1<br>Orufi09g16160.1 0.002 1 0.998 0.002 1<br>Orufi09g16160.2 0.002 1 0.998 0.002 1	1<br>Obart09g14980.1 0.002 1 0.998 0.002 1<br>Obart09g14980.2 0.002 1 0.998 0.002 1	0	1<br>Opunc09g13420.1 0.002 1 0.998 0.002 1<br>Opunc09g13420.2 0.002 1 0.998 0.002 1	1<br>Obrac09g09090.1 0.002 1 0.998 0.002 1	0	0
	1<br>Oglab08g19240.1 0.519 0.994 0.475 0.014 0.986<br>Oglab12g18680.1 0.002 0.501 0.499 0.003 0.997	1<br>Osjap02g06650.1 0.009 0.501 0.492 0.01 0.997<br>Osjap09g15430.1 0.514 1 0.486 0.003 1	1<br>Osind02g06940.1 0.009 0.501 0.492 0.01 0.997<br>Osind08g23920.1 0.519 0.994 0.475 0.012 0.988	1<br>Orufi04g24220.1 0.012 0.502 0.49 0.165 1<br>Orufi08g22880.1 0.519 0.994 0.475 0.012 0.988	1<br>Obart02g06180.1 0.009 0.501 0.492 0.01 0.997<br>Obart08g20390.1 0.501 0.994 0.493 0.566 0.988	1<br>Olong_018059-R1 0.518 0.992 0.474 0.046 0.957<br>Olong_024186-R1 0.002 0.501 0.499 0.003 0.997	1<br>Opunc02g05570.1 0.005 0.501 0.496 0.087 0.997<br>Opunc08g18560.1 0.519 0.994 0.475 0.012 0.969	0<br>Obrac08g13590.1 0.499 0.994 0.495 0.689 0.994	1<br>Lperr04g18350.1 0.002 0.501 0.499 0.021 0.997<br>Lperr08g17230.1 0.507 0.994 0.487 0.021 0.997	1<br>AT2G21270.3 0.003 0.501 0.498 0.059 0.997<br>AT5G42800.1 0.516 0.992 0.476 0.003 0.851
Oglab09g13510.2	1<br>Oglab09g13510.1 0.002 1 0.998 0.002 1<br>Oglab09g13510.2 0.002 1 0.998 0.002 1<br>Oglab09g13510.3 0.002 1 0.998 0.002 1<br>Oglab09g13510.4 0.002 1 0.998 0.002 1	1<br>Osjap09g15430.2 0.002 1 0.998 0.002 1<br>Osjap09g15430.3 0.002 1 0.998 0.002 1	1<br>Osind09g15570.1 0.002 1 0.998 0.002 1<br>Osind09g15570.2 0.002 1 0.998 0.002 1	1<br>Orufi09g16160.1 0.002 1 0.998 0.002 1<br>Orufi09g16160.2 0.002 1 0.998 0.002 1	1<br>Obart09g14980.1 0.002 1 0.998 0.002 1<br>Obart09g14980.2 0.002 1 0.998 0.002 1	0	1<br>Opunc09g13420.1 0.002 1 0.998 0.002 1<br>Opunc09g13420.2 0.002 1 0.998 0.002 1	1<br>Obrac09g09090.1 0.002 1 0.998 0.002 1	0	0
	1<br>Oglab08g19240.1 0.541 0.994 0.453 0.014 0.986<br>Oglab12g18680.1 0.002 0.522 0.52 0.003 0.997	1<br>Osjap02g06650.1 0.01 0.522 0.512 0.01 0.997<br>Osjap09g15430.1 0.537 1 0.463 0.003 1	1<br>Osind02g06940.1 0.01 0.522 0.512 0.01 0.997<br>Osind08g23920.1 0.541 0.994 0.453 0.012 0.988	1<br>Orufi04g24220.1 0.013 0.524 0.511 0.165 1<br>Orufi08g22880.1 0.541 0.994 0.453 0.012 0.988	1<br>Obart02g06180.1 0.01 0.522 0.512 0.01 0.997<br>Obart08g20390.1 0.522 0.994 0.472 0.566 0.988	1<br>Olong_014421-R1 0.537 0.992 0.455 0.002 0.984<br>Olong_024186-R1 0.002 0.522 0.52 0.003 0.997	1<br>Opunc02g05570.1 0.005 0.522 0.517 0.087 0.997<br>Opunc08g18560.1 0.541 0.994 0.453 0.012 0.969	1<br>Obrac02g04550.3 0.008 0.522 0.514 0.214 0.997<br>Obrac09g00560.1 0.54 0.992 0.452 0.043 0.954	1<br>Lperr04g18350.1 0.002 0.522 0.52 0.021 0.997<br>Lperr08g17230.1 0.529 0.994 0.465 0.021 0.997	1<br>AT1G15950.1 0.538 0.992 0.454 0.015 0.936<br>AT2G21270.3 0.003 0.522 0.519 0.059 0.997
Oglab09g13510.3	1<br>Oglab09g13510.1 0.002 1 0.998 0.002 1<br>Oglab09g13510.2 0.002 1 0.998 0.002 1	1<br>Osjap09g15430.2 0.002 1 0.998 0.002 1<br>Osjap09g15430.3 0.002 1 0.998 0.002 1	1<br>Osind09g15570.1 0.002 1 0.998 0.002 1<br>Osind09g15570.2 0.002 1 0.998 0.002 1	1<br>Orufi09g16160.1 0.002 1 0.998 0.002 1<br>Orufi09g16160.2 0.002 1 0.998 0.002 1	1<br>Obart09g14980.1 0.002 1 0.998 0.002 1<br>Obart09g14980.2 0.002 1 0.998 0.002 1	0	1<br>Opunc09g13420.1 0.002 1 0.998 0.002 1<br>Opunc09g13420.2 0.002 1 0.998 0.002 1	1<br>Obrac09g09090.1 0.002 1 0.998 0.002 1	0	0

	Oglab09g13510.3 0.002 1 0.998 0.002 1<br>Oglab09g13510.4 0.002 1 0.998 0.002 1									
	1<br>Oglab08g19240.1 0.544 0.993 0.449 0.143 0.986<br>Oglab12g18680.1 0.002 0.542 0.54 0.003 1	1<br>Osjap02g06650.1 0.01 0.54 0.53 0.01 0.997<br>Osjap09g15430.1 0.544 1 0.456 0.131 1	1<br>Osind02g06940.1 0.01 0.54 0.53 0.01 0.997<br>Osind08g23920.1 0.544 0.993 0.449 0.131 0.988	1<br>Orufi04g24220.1 0.013 0.542 0.529 0.165 1<br>Orufi08g22880.1 0.544 0.993 0.449 0.131 0.988	1<br>Obart02g06180.1 0.01 0.54 0.53 0.01 0.997<br>Obart08g20390.1 0.544 0.993 0.449 0.631 0.988	1<br>Olong_013054-R1 0.547 0.992 0.445 0.149 0.979<br>Olong_024186-R1 0.002 0.542 0.54 0.003 1	1<br>Opunc04g20190.1 0.007 0.542 0.535 0.006 1<br>Opunc08g18560.1 0.544 0.993 0.449 0.128 0.969	1<br>Obrac02g04550.3 0.008 0.54 0.532 0.214 0.997<br>Obrac09g00560.1 0.548 0.998 0.45 0.164 0.966	1<br>Lperr04g18350.1 0.002 0.542 0.54 0.021 1<br>Lperr09g11120.1 0.544 1 0.456 0.156 1	1<br>AT1G68540.1 0.542 0.992 0.45 0.112 1<br>AT2G21270.3 0.003 0.54 0.537 0.059 0.997
Oglab09g13510.4	1<br>Oglab09g13510.1 0.002 1 0.998 0.002 1<br>Oglab09g13510.2 0.002 1 0.998 0.002 1<br>Oglab09g13510.3 0.002 1 0.998 0.002 1<br>Oglab09g13510.4 0.002 1 0.998 0.002 1	1<br>Osjap09g15430.2 0.002 1 0.998 0.002 1<br>Osjap09g15430.3 0.002 1 0.998 0.002 1	1<br>Osind09g15570.1 0.002 1 0.998 0.002 1<br>Osind09g15570.2 0.002 1 0.998 0.002 1	1<br>Orufi09g16160.1 0.002 1 0.998 0.002 1<br>Orufi09g16160.2 0.002 1 0.998 0.002 1	1<br>Obart09g14980.1 0.002 1 0.998 0.002 1<br>Obart09g14980.2 0.002 1 0.998 0.002 1	0	1<br>Opunc09g13420.1 0.002 1 0.998 0.002 1<br>Opunc09g13420.2 0.002 1 0.998 0.002 1	1<br>Obrac09g09090.1 0.002 1 0.998 0.002 1	0	0
	1<br>Oglab08g19240.1 0.569 0.993 0.424 0.143 0.986<br>Oglab12g18680.1 0.002 0.567 0.565 0.003 1	1<br>Osjap02g06650.1 0.01 0.565 0.555 0.01 0.997<br>Osjap09g15430.1 0.569 1 0.431 0.131 1	1<br>Osind02g06940.1 0.01 0.565 0.555 0.01 0.997<br>Osind08g23920.1 0.569 0.993 0.424 0.131 0.988	1<br>Orufi04g24220.1 0.014 0.567 0.553 0.165 1<br>Orufi08g22880.1 0.569 0.993 0.424 0.131 0.988	1<br>Obart02g06180.1 0.01 0.565 0.555 0.01 0.997<br>Obart08g20390.1 0.569 0.993 0.424 0.631 0.988	1<br>Olong_008093-R2 0.596 0.991 0.395 0.25 0.996<br>Olong_024186-R1 0.002 0.567 0.565 0.003 1	1<br>Opunc04g20190.1 0.007 0.567 0.56 0.006 1<br>Opunc08g18560.1 0.569 0.993 0.424 0.128 0.969	1<br>Obrac01g09090.1 0.605 0.998 0.393 0.299 0.982<br>Obrac02g04550.3 0.009 0.565 0.556 0.214 0.997	1<br>Lperr04g18350.1 0.002 0.567 0.565 0.021 1<br>Lperr09g11120.1 0.569 1 0.431 0.156 1	1<br>AT1G68540.1 0.567 0.991 0.424 0.112 1<br>AT2G21270.3 0.003 0.565 0.562 0.059 0.997
Oglab09g14970.1	1<br>Oglab09g14970.1 0.001 1 0.999 0.001 1	0	0	0	0	0	0	0	0	0
	1<br>Oglab03g15980.2 0.767 0.971 0.204 0.004 0.818<br>Oglab12g01080.1 0.001 0.686 0.685 0.001 0.984	1<br>Osjap09g16930.1 0.713 0.971 0.258 0.077 0.857<br>Osjap09g16940.1 0.001 0.69 0.689 0.001 0.977	1<br>Osind09g17090.1 0.713 0.971 0.258 0.077 0.857<br>Osind09g17100.1 0.001 0.69 0.689 0.001 0.977	1<br>Orufi09g17780.1 0.713 0.971 0.258 0.077 0.857<br>Orufi09g17790.1 0.001 0.69 0.689 0.001 0.977	1<br>Obart09g16470.1 0.713 0.971 0.258 0.077 0.857<br>Obart09g16480.1 0.001 0.69 0.689 0.001 0.977	1<br>Olong_000845-R1 0.001 0.544 0.543 0.002 1<br>Olong_000846-R1 0.713 0.971 0.258 0.075 0.862	1<br>Opunc09g14900.1 0.713 0.971 0.258 0.077 0.857<br>Opunc09g14910.1 0.001 0.707 0.706 0.001 1	1<br>Obrac08g14920.1 0.001 0.706 0.705 0.18 0.999<br>Obrac09g10340.1 0.713 0.971 0.258 0.077 0.857	1<br>Lperr09g12580.2 0.713 0.971 0.258 0.075 0.857<br>Lperr09g12600.1 0.001 0.707 0.706 0.001 1	1<br>AT2G31350.1 0.708 0.971 0.263 0.046 0.852<br>AT3G19610.1 0.001 0.689 0.688 0.045 0.956
Oglab09g18200.1	1<br>Oglab09g18200.1 0.001 1 0.999 0.001 1	1<br>Osjap09g20460.1 0.001 0.999 0.998 0.001 0.999	1<br>Osind09g20560.1 0.001 0.985 0.984 0.001 0.998	1<br>Orufi09g21510.1 0.001 0.958 0.957 0.001 0.999	1<br>Obart09g19980.1 0.066 1 0.934 0.181 1<br>Obart09g19980.2 0.001 1 0.999 0.001 1	1<br>Olong_014589-R1 0.116 0.972 0.856 0.032 0.991<br>Olong_014589-R2 0.116 0.972 0.856 0.033 0.991	0	0	0	0
	1<br>Oglab02g17850.1 0.023 0.609 0.586 0.095 0.914<br>Oglab09g18170.1 0.619 0.999 0.38 0.13 0.998	1<br>Osjap02g21530.2 0.023 0.609 0.586 0.096 0.943<br>Osjap09g20430.1 0.649 0.999 0.35 0.151 0.998	1<br>Osind02g21430.2 0.023 0.609 0.586 0.096 0.943<br>Osind09g20550.1 0.619 0.999 0.38 0.058 0.999	1<br>Orufi02g19810.2 0.023 0.609 0.586 0.096 0.943<br>Orufi09g21500.1 0.649 0.999 0.35 0.151 0.998	1<br>Obart04g25370.1 0.047 0.617 0.57 0.176 0.967<br>Obart09g19950.1 0.619 0.999 0.38 0.13 0.998	0<br>Olong_014116-R1 0.063 0.617 0.554 0.069 0.957	1<br>Opunc09g18280.1 0.619 0.998 0.379 0.118 0.951<br>Opunc09g18290.2 0.001 0.618 0.617 0.001 0.971	1<br>Obrac09g13140.3 0.619 0.985 0.366 0.089 0.806<br>Obrac09g13160.1 0.001 0.618 0.617 0.001 0.953	1<br>Lperr09g15890.1 0.619 0.997 0.378 0.085 0.957<br>Lperr09g15920.1 0.001 0.618 0.617 0.001 0.958	0<br>AT1G18160.1 0.021 0.617 0.596 0.064 0.958
Oglab09g18980.1	1<br>Oglab09g18980.1 0.001 1 0.999 0.001 1	0	0	0	0	0	0	0	0	0
	1<br>Oglab01g27280.1 0.001 0.371 0.37 0.002 0.481<br>Oglab08g16490.1 0.403 1 0.597 0.001 1	1<br>Osjap01g37290.2 0.001 0.371 0.37 0.065 0.591<br>Osjap08g18690.1 0.45 1 0.55 0.006 1	1<br>Osind03g38060.1 0.025 0.39 0.365 0.05 0.573<br>Osind08g20790.1 0.442 1 0.558 0.001 1	1<br>Orufi01g35750.2 0.001 0.371 0.37 0.069 0.57<br>Orufi08g19800.1 0.45 1 0.55 0.006 1	1<br>Obart03g34250.1 0.025 0.39 0.365 0.05 0.573<br>Obart08g17650.1 0.403 1 0.597 0.001 1	1<br>Olong_000486-R1 0.403 1 0.597 0.077 1<br>Olong_000981-R1 0.001 0.371 0.37 0.002 0.539	1<br>Opunc01g31560.1 0.001 0.371 0.37 0.002 0.483<br>Opunc08g15500.1 0.458 1 0.542 0.028 1	1<br>Obrac03g25390.2 0.005 0.371 0.366 0.015 0.559<br>Obrac08g11470.1 0.439 1 0.561 0.004 1	1<br>Lperr03g28740.1 0.01 0.392 0.382 0.011 0.568<br>Lperr08g14590.1 0.48 1 0.52 0.103 1	1<br>AT2G03200.1 0.476 1 0.524 0.098 1<br>AT5G04885.1 0.006 0.397 0.391 0.011 0.55
Oglab10g03910.1	1<br>Oglab10g03910.1 0.01 1 0.99 0.01 1	1<br>Osjap10g04950.1 0.01 1 0.99 0.01 1<br>Osjap10g21210.1 0.01 1 0.99 0.477 1	1<br>Osind10g03420.1 0.01 1 0.99 0.558 1<br>Osind10g17700.1 0.01 1 0.99 0.477 1	1<br>Orufi10g04520.1 0.01 1 0.99 0.01 1<br>Orufi10g19420.1 0.01 1 0.99 0.477 1	1<br>Obart10g18220.1 0.01 1 0.99 0.392 0.823	1<br>Olong_009744-R1 0.01 1 0.99 0.651 1<br>Olong_009744-R2 0.01 1 0.99 0.641 1	0	0	0	0
	1<br>Oglab02g32260.1 0.361 1 0.639 0.838 0.924<br>Oglab04g27900.1 0.01 0.33 0.32 0.14 0.208	0<br>Osjap07g13660.9 0.361 1 0.639 0.733 0.947	1<br>Osind02g38620.1 0.361 1 0.639 0.835 0.923<br>Osind06g00010.1 0.01 0.361 0.351 0.609 0.978	1<br>Orufi02g36440.1 0.361 1 0.639 0.835 0.923<br>Orufi08g26670.5 0.01 0.351 0.341 0.583 0.749	1<br>Obart02g34980.1 0.361 1 0.639 0.838 0.924<br>Obart04g29850.1 0.01 0.33 0.32 0.131 0.196	1<br>Olong_008835-R2 0.361 1 0.639 0.809 0.91<br>Olong_022843-R2 0.01 0.33 0.32 0.14 0.208	1<br>Opunc02g32260.1 0.361 1 0.639 0.84 0.925<br>Opunc04g27380.2 0.01 0.33 0.32 0.14 0.208	1<br>Obrac02g25650.1 0.381 1 0.619 0.839 0.923<br>Obrac04g21100.1 0.01 0.33 0.32 0.145 0.217	1<br>Lperr02g28770.1 0.381 1 0.619 0.834 0.92<br>Lperr04g25370.2 0.01 0.33 0.32 0.151 0.225	0

Oglab10g08680.2	1<br>Oglab10g08680.1 0.009 1 0.991 0.008 1<br>Oglab10g08680.2 0.001 1 0.999 0.001 1	0	0	1<br>Orufi10g09480.1 0.009 1 0.991 0.008 1<br>Orufi10g09480.2 0.009 1 0.991 0.008 1<br>Orufi10g09480.3 0.001 1 0.999 0.001 1	1<br>Obart10g09110.1 0.009 1 0.991 0.008 1<br>Obart10g09110.2 0.001 1 0.999 0.001 1	1<br>Olong_027249-R1 0.03 1 0.97 0.001 1<br>Olong_027249-R2 0.03 1 0.97 0.001 1	0	0	0	0
	1<br>Oglab02g10660.1 0.625 0.999 0.374 0.129 0.991<br>Oglab12g06810.1 0.013 0.615 0.602 0.056 0.969	1<br>Osjap01g36630.1 0.626 1 0.374 0.222 1<br>Osjap10g10740.1 0.001 0.626 0.625 0.002 0.997	1<br>Osind10g08120.1 0.626 1 0.374 0.222 1<br>Osind10g08130.1 0.001 0.626 0.625 0.002 0.997	1<br>Orufi01g35060.1 0.626 1 0.374 0.222 1<br>Orufi12g07540.1 0.013 0.615 0.602 0.039 0.673	0<br>Obart02g10660.1 0.625 0.999 0.374 0.129 0.991	1<br>Olong_022306-R1 0.609 0.991 0.382 0.19 0.992<br>Olong_024721-R1 0.105 0.604 0.499 0.048 0.942	1<br>Opunc01g30900.1 0.626 1 0.374 0.222 1<br>Opunc10g07870.1 0.004 0.626 0.622 0.003 0.997	1<br>Obrac01g24330.1 0.626 1 0.374 0.221 1<br>Obrac12g04960.2 0.102 0.615 0.513 0.026 0.902	1<br>Lperr01g27340.1 0.626 1 0.374 0.228 1<br>Lperr09g14980.1 0.001 0.604 0.603 0.002 0.959	0<br>AT4G14820.1 0.001 0.614 0.613 0.004 0.86
Oglab10g14420.1	1<br>Oglab10g14420.1 0.007 1 0.993 0.007 1	1<br>Osjap04g26670.1 0.059 0.98 0.921 0.005 0.989<br>Osjap10g21180.1 0.046 1 0.954 0.495 1	0	1<br>Orufi04g24510.1 0.007 0.993 0.986 0.009 1<br>Orufi10g19400.1 0.039 1 0.961 0.009 1	0	0	1<br>Opunc06g20630.1 0.039 1 0.961 0.316 0.984	0	0	0
	1<br>Oglab04g27360.1 0.671 0.928 0.257 0.741 0.891<br>Oglab06g09210.2 0.039 0.586 0.547 0.082 0.173	1<br>Osjap02g36030.1 0.039 0.586 0.547 0.001 0.098<br>Osjap04g33480.1 0.671 0.928 0.257 0.741 0.891	1<br>Osind04g32680.1 0.671 0.928 0.257 0.741 0.891<br>Osind08g18030.1 0.039 0.586 0.547 0.001 0.106	1<br>Orufi02g33550.1 0.007 0.586 0.579 0.001 0.104<br>Orufi04g31030.1 0.671 0.928 0.257 0.741 0.891	1<br>Obart04g29320.1 0.671 0.928 0.257 0.741 0.891<br>Obart08g15190.1 0.039 0.586 0.547 0.001 0.106	0<br>Olong_004819-R1 0.026 0.362 0.336 0.231 0.615	1<br>Opunc02g29570.2 0.033 0.586 0.553 0.008 0.106<br>Opunc04g26840.1 0.671 0.928 0.257 0.741 0.891	0<br>Obrac02g23230.1 0.033 0.586 0.553 0.007 0.1	0<br>Lperr02g26070.3 0.007 0.586 0.579 0.001 0.112	0<br>AT1G59610.1 0.039 0.586 0.547 0.001 0.099
Oglab10g16090.1	1<br>Oglab10g16090.1 0.001 1 0.999 0.001 1	0	1<br>Osind10g19230.1 0.001 0.861 0.86 0.002 0.992	1<br>Orufi10g20960.1 0.001 0.861 0.86 0.002 0.992	1<br>Obart10g19720.1 0.001 0.861 0.86 0.002 0.992	0	1<br>Opunc10g18180.1 0.001 0.99 0.989 0.002 0.998	0	0	0
	1<br>Oglab01g14760.1 0.419 0.984 0.565 0.2 1<br>Oglab08g03250.2 0.037 0.374 0.337 0.052 0.955	1<br>Osjap10g22750.1 0.411 1 0.589 0.061 1<br>Osjap10g22760.1 0.001 0.392 0.391 0.003 0.993	1<br>Osind01g20890.1 0.419 0.977 0.558 0.154 0.802<br>Osind08g03990.1 0.037 0.374 0.337 0.057 0.95	1<br>Orufi01g19490.1 0.419 0.977 0.558 0.171 0.862<br>Orufi08g04140.1 0.037 0.374 0.337 0.057 0.95	1<br>Obart01g17550.1 0.419 0.977 0.558 0.174 0.863<br>Obart08g03750.1 0.037 0.374 0.337 0.057 0.95	1<br>Olong_006312-R1 0.416 1 0.584 0.067 1<br>Olong_006313-R1 0.001 0.392 0.391 0.003 0.993	1<br>Opunc01g18040.1 0.419 0.984 0.565 0.174 1<br>Opunc08g03700.1 0.014 0.374 0.36 0.011 0.951	1<br>Obrac01g13330.1 0.423 0.984 0.561 0.196 1<br>Obrac10g13120.1 0.001 0.392 0.391 0.004 0.993	1<br>Lperr10g15590.1 0.416 0.985 0.569 0.07 0.989<br>Lperr10g15600.1 0.001 0.389 0.388 0.003 0.979	1<br>AT1G32080.1 0.42 0.984 0.564 0.188 1<br>AT2G32260.1 0.035 0.375 0.34 0.078 0.825
Oglab11g01510.4	1<br>Oglab11g01510.1 0.002 1 0.998 0.14 1<br>Oglab11g01510.2 0.002 1 0.998 0.202 1<br>Oglab11g01510.3 0.002 1 0.998 0.203 1<br>Oglab11g01510.4 0.002 1 0.998 0.002 1<br>Oglab11g01510.5 0.002 1 0.998 0.002 1	1<br>Osjap11g01890.1 0.002 0.989 0.987 0.147 0.988<br>Osjap11g01890.2 0.002 1 0.998 0.143 1<br>Osjap11g01890.3 0.002 0.989 0.987 0.13 0.988<br>Osjap11g01890.4 0.002 0.989 0.987 0.149 0.988<br>Osjap11g01890.5 0.002 0.989 0.987 0.142 0.988<br>Osjap11g01890.6 0.002 1 0.998 0.146 1<br>Osjap11g01890.7 0.002 0.989 0.987 0.201 0.983	0	0	1<br>Obart11g01710.1 0.002 0.994 0.992 0.104 0.922<br>Obart11g01710.2 0.002 0.994 0.992 0.106 0.921<br>Obart11g01710.3 0.002 0.994 0.992 0.115 0.914<br>Obart11g01710.4 0.002 0.994 0.992 0.117 0.913	0	1<br>Opunc12g01520.7 0.002 0.989 0.987 0.23 0.981	0	1<br>Lperr12g01560.1 0.002 0.989 0.987 0.13 0.989	0
	1<br>Oglab12g01390.1 0.434 0.989 0.555 0.003 0.961<br>Oglab12g01400.2 0.002 0.408 0.406 0.291 0.961	1<br>Osjap12g01820.1 0.434 0.989 0.555 0.003 0.961<br>Osjap12g01870.1 0.002 0.408 0.406 0.341 0.93	1<br>Osind02g25820.1 0.434 0.989 0.555 0.003 0.961<br>Osind03g06850.1 0.005 0.415 0.41 0.359 1	1<br>Orufi03g27580.1 0.002 0.431 0.429 0.305 0.953<br>Orufi11g01580.1 0.434 0.989 0.555 0.003 0.961	1<br>Obart03g06450.1 0.005 0.415 0.41 0.359 1<br>Obart12g01440.2 0.434 0.989 0.555 0.003 0.961	0<br>Olong_024204-R1 0.002 0.409 0.407 0.36 0.998<br>Olong_028389-R1 0.659 0.989 0.33 0.008 0.936	1<br>Opunc11g01470.1 0.434 0.989 0.555 0.003 0.961<br>Opunc12g01520.9 0.002 0.397 0.395 0.371 0.992	1<br>Obrac03g18430.1 0.002 0.418 0.416 0.331 0.992<br>Obrac12g01300.1 0.434 0.989 0.555 0.003 0.961	1<br>Lperr03g19970.1 0.002 0.418 0.416 0.335 0.98<br>Lperr11g01360.1 0.434 0.989 0.555 0.003 0.962	1<br>AT1G33090.1 0.003 0.432 0.429 0.34 1<br>AT5G21060.2 0.446 0.989 0.543 0.011 0.968
Oglab11g01510.5	1<br>Oglab11g01510.1 0.002 1 0.998 0.14 1<br>Oglab11g01510.2 0.002 1 0.998 0.202 1<br>Oglab11g01510.3 0.002 1 0.998 0.203 1<br>Oglab11g01510.4 0.002 1 0.998 0.002 1<br>Oglab11g01510.5 0.002 1 0.998 0.002 1	1<br>Osjap11g01890.1 0.002 0.989 0.987 0.147 0.988<br>Osjap11g01890.2 0.002 1 0.998 0.143 1<br>Osjap11g01890.3 0.002 0.989 0.987 0.13 0.988<br>Osjap11g01890.4 0.002 0.989 0.987 0.149 0.988<br>Osjap11g01890.5 0.002 0.989 0.987 0.142 0.988	0	0	1<br>Obart11g01710.1 0.002 0.994 0.992 0.104 0.922<br>Obart11g01710.2 0.002 0.994 0.992 0.106 0.921<br>Obart11g01710.3 0.002 0.994 0.992 0.115 0.914<br>Obart11g01710.4 0.002 0.994 0.992 0.117 0.913	0	1<br>Opunc12g01520.7 0.002 0.989 0.987 0.23 0.981	0	1<br>Lperr12g01560.1 0.002 0.989 0.987 0.13 0.989	0

		Osjap11g01890.6 0.002 1 0.998 0.146 1<br>Osjap11g01890.7 0.002 0.989 0.987 0.201 0.983								
	1<br>Oglab12g01390.1 0.43 0.989 0.559 0.003 0.961<br>Oglab12g01400.2 0.002 0.404 0.402 0.291 0.961	1<br>Osjap12g01820.1 0.43 0.989 0.559 0.003 0.961<br>Osjap12g01870.1 0.002 0.404 0.402 0.341 0.93	1<br>Osind02g25820.1 0.43 0.989 0.559 0.003 0.961<br>Osind03g06850.1 0.005 0.412 0.407 0.359 1	1<br>Orufi03g24390.1 0.002 0.415 0.413 0.36 0.979<br>Orufi11g01580.1 0.43 0.989 0.559 0.003 0.961	1<br>Obart03g06450.1 0.005 0.412 0.407 0.359 1<br>Obart12g01440.2 0.43 0.989 0.559 0.003 0.961	0<br>Olong_024204-R1 0.002 0.406 0.404 0.36 0.998<br>Olong_028389-R1 0.657 0.989 0.332 0.008 0.936	1<br>Opunc11g01470.1 0.43 0.989 0.559 0.003 0.961<br>Opunc12g01520.9 0.002 0.394 0.392 0.371 0.992	1<br>Obrac03g18430.1 0.002 0.415 0.413 0.331 0.992<br>Obrac12g01300.1 0.43 0.989 0.559 0.003 0.961	1<br>Lperr03g05800.1 0.005 0.412 0.407 0.38 1<br>Lperr11g01360.1 0.43 0.989 0.559 0.003 0.962	1<br>AT1G33090.1 0.003 0.429 0.426 0.34 1<br>AT5G21060.2 0.442 0.989 0.547 0.011 0.968
Oglab11g02200.1	1<br>Oglab11g02200.1 0.002 1 0.998 0.002 1<br>Oglab12g02130.1 0.002 1 0.998 0.002 1	1<br>Osjap11g02640.1 0.002 1 0.998 0.002 1<br>Osjap12g02610.1 0.002 1 0.998 0.002 1	1<br>Osind11g01990.1 0.002 1 0.998 0.002 1<br>Osind12g02780.1 0.002 1 0.998 0.002 1	1<br>Orufi11g02320.1 0.002 1 0.998 0.003 1<br>Orufi12g02480.1 0.002 1 0.998 0.002 1	1<br>Obart12g02080.1 0.023 1 0.977 0.002 1	0	0	0	0	0
	1<br>Oglab07g03810.1 0.032 0.657 0.625 0.019 0.987<br>Oglab12g09880.1 0.66 0.951 0.291 0.051 0.385	1<br>Osjap07g04360.1 0.032 0.657 0.625 0.018 0.925<br>Osjap12g12870.1 0.66 0.951 0.291 0.055 0.417	1<br>Osind11g02010.1 0.013 0.657 0.644 0.001 0.994<br>Osind12g10020.1 0.66 0.951 0.291 0.055 0.417	1<br>Orufi07g03650.1 0.032 0.657 0.625 0.019 0.976<br>Orufi12g10750.1 0.658 0.951 0.293 0.045 0.364	0<br>Obart11g02350.1 0.021 0.657 0.636 0.004 0.993	1<br>Olong_012584-R1 0.064 0.611 0.547 0.002 0.965<br>Olong_017316-R1 0.658 0.951 0.293 0.045 0.365	1<br>Opunc11g02190.1 0.002 0.657 0.655 0.003 0.989<br>Opunc12g08740.1 0.658 0.951 0.293 0.04 0.324	1<br>Obrac05g10860.1 0.049 0.657 0.608 0.097 0.987<br>Obrac12g07170.1 0.658 0.942 0.284 0.071 0.57	1<br>Lperr12g02190.1 0.002 0.657 0.655 0.003 0.989<br>Lperr12g08370.1 0.657 0.951 0.294 0.047 0.391	0<br>AT5G27320.1 0.013 0.632 0.619 0.003 0.98
Oglab11g05440.1	1<br>Oglab11g05440.1 0.001 1 0.999 0.001 1	1<br>Osjap11g06710.1 0.001 0.998 0.997 0.067 0.993<br>Osjap11g06720.1 0.001 0.998 0.997 0.054 0.977	1<br>Osind09g09040.1 0.001 0.998 0.997 0.001 0.995	1<br>Orufi11g05930.1 0.001 0.998 0.997 0.09 0.992<br>Orufi11g05940.1 0.001 1 0.999 0.002 0.995	1<br>Obart11g05910.1 0.001 0.875 0.874 0.05 1	0	0	0	0	0
	1<br>Oglab08g10820.1 0.526 0.955 0.429 0.015 0.867<br>Oglab11g05700.1 0.001 0.51 0.509 0.001 0.966	1<br>Osjap01g24690.1 0.525 0.992 0.467 0.014 0.934<br>Osjap11g06740.8 0.024 0.509 0.485 0.013 0.579	1<br>Osind09g09050.1 0.001 0.502 0.501 0.104 0.994<br>Osind11g05650.1 0.521 0.998 0.477 0.002 0.989	1<br>Orufi11g05890.1 0.521 0.998 0.477 0.003 0.984<br>Orufi11g05900.2 0.001 0.509 0.508 0.002 0.991	1<br>Obart01g20930.1 0.525 0.992 0.467 0.006 0.498<br>Obart11g05930.1 0.001 0.502 0.501 0.002 0.998	1<br>Olong_005786-R3 0.007 0.511 0.504 0.006 0.948<br>Olong_005787-R1 0.521 0.998 0.477 0.002 0.989	0<br>Opunc11g05960.1 0.443 0.998 0.555 0.002 0.991	1<br>Obrac01g15800.1 0.52 0.992 0.472 0.004 0.915<br>Obrac11g03930.1 0.001 0.504 0.503 0.002 0.941	1<br>Lperr01g17850.1 0.521 0.992 0.471 0.002 0.973<br>Lperr11g05360.1 0.001 0.502 0.501 0.044 0.995	0<br>AT4G38130.1 0.521 0.955 0.434 0.002 0.782
Oglab11g06530.1	1<br>Oglab11g06530.1 0.01 1 0.99 0.01 1	0	1<br>Osind11g06680.1 0.01 0.942 0.932 0.004 0.411	1<br>Orufi11g07410.1 0.01 0.942 0.932 0.005 0.516	0	0	0	0	0	0
	1<br>Oglab01g35920.1 0.01 0.612 0.602 0.002 0.144<br>Oglab08g00300.1 0.612 0.981 0.369 0.487 0.537	1<br>Osjap01g46770.1 0.01 0.612 0.602 0.002 0.144<br>Osjap08g00940.1 0.612 0.981 0.369 0.487 0.537	1<br>Osind01g47980.1 0.01 0.612 0.602 0.002 0.142<br>Osind04g02960.1 0.612 0.981 0.369 0.501 0.562	1<br>Orufi01g44430.1 0.01 0.612 0.602 0.002 0.14<br>Orufi08g00930.1 0.612 0.981 0.369 0.487 0.537	1<br>Obart01g41090.1 0.01 0.612 0.602 0.002 0.144<br>Obart04g30900.2 0.612 0.981 0.369 0.451 0.513	0<br>Olong_019421-R2 0.398 0.981 0.583 0.431 0.523	1<br>Opunc01g39900.1 0.01 0.612 0.602 0.002 0.159<br>Opunc04g28500.1 0.612 0.981 0.369 0.467 0.523	0<br>Obrac12g04550.1 0.398 0.981 0.583 0.719 0.769	0<br>Lperr12g05120.1 0.388 0.981 0.593 0.461 0.561	0<br>AT1G57700.1 0.33 0.981 0.651 0.422 0.527
Oglab11g14350.2	1<br>Oglab11g14350.1 0.001 0.967 0.966 0.001 1<br>Oglab11g14350.2 0.001 1 0.999 0.001 1	0	0	0	1<br>Obart11g16070.1 0.001 1 0.999 0.013 1	0	0	0	0	0
	1<br>Oglab02g07220.2 0.597 1 0.403 0.003 1<br>Oglab11g18510.1 0.032 0.552 0.52 0.029 1	1<br>Osjap02g07570.1 0.597 1 0.403 0.002 1<br>Osjap11g18290.1 0.001 0.531 0.53 0.514 0.989	1<br>Osind11g13470.1 0.001 0.496 0.495 0.416 0.992<br>Osind11g13490.1 0.595 1 0.405 0.081 1	1<br>Orufi11g17130.1 0.001 0.531 0.53 0.522 0.989<br>Orufi11g17160.1 0.597 1 0.403 0.002 1	1<br>Obart02g07160.1 0.597 1 0.403 0.002 1<br>Obart08g10830.1 0.036 0.55 0.514 0.004 0.944	1<br>Olong_012209-R1 0.597 1 0.403 0.002 1<br>Olong_021538-R1 0.028 0.559 0.531 0.006 0.801	1<br>Opunc11g13170.1 0.597 1 0.403 0.002 1<br>Opunc11g17530.1 0.026 0.545 0.519 0.009 0.97	1<br>Obrac02g05260.1 0.597 1 0.403 0.002 1<br>Obrac10g05860.1 0.038 0.536 0.498 0.005 0.973	1<br>Lperr11g17450.1 0.034 0.537 0.503 0.003 0.875<br>Lperr12g06590.1 0.568 1 0.432 0.061 1	0<br>AT2G44350.2 0.597 1 0.403 0.002 0.998<br>AT5G54820.1 0.038 0.401 0.363 0.015 0.788
Oglab11g18520.1	1<br>Oglab11g18520.1 0.001 1 0.999 0.001 1	0	0	1<br>Orufi11g22790.1 0.013 0.977 0.964 0.006 0.971	0	0	0	0	0	0
	1<br>Oglab11g18510.1 0.013 0.512 0.499 0.037 0.942<br>Oglab11g18550.1 0.54 1 0.46 0.002 1	1<br>Osjap11g24530.1 0.013 0.536 0.523 0.038 0.993<br>Osjap11g24540.1 0.54 1 0.46 0.002 1	1<br>Osind06g03750.1 0.54 1 0.46 0.002 1<br>Osind10g01290.1 0.017 0.501 0.484 0.035 0.99	1<br>Orufi04g30270.1 0.54 0.992 0.452 0.002 0.984<br>Orufi11g22780.1 0.009 0.498 0.489 0.018 0.881	1<br>Obart11g20950.1 0.013 0.512 0.499 0.003 0.945<br>Obart11g20980.1 0.54 1 0.46 0.003 1	1<br>Olong_021538-R1 0.013 0.514 0.501 0.011 0.957<br>Olong_027904-R1 0.579 0.989 0.41 0.108 0.991	1<br>Opunc03g06070.1 0.54 0.992 0.452 0.002 0.98<br>Opunc11g13180.1 0.013 0.498 0.485 0.169 0.909	1<br>Obrac03g05030.1 0.54 0.992 0.452 0.003 0.967<br>Obrac11g09310.1 0.005 0.498 0.493 0.025 0.932	1<br>Lperr09g14070.1 0.54 0.991 0.451 0.007 1<br>Lperr11g12540.1 0.005 0.496 0.491 0.013 0.892	0<br>AT5G23940.1 0.547 0.991 0.444 0.012 0.921

Oglab11g20100.2	1<br>Oglab11g20100.1 0.001 0.998 0.997 0 0.998<br>Oglab11g20100.2 0.001 1 0.999 0.001 1	0	0	0	1<br>Obart11g23070.1 0.001 1 0.999 0.022 1	0	1<br>Opunc11g19450.1 0.001 0.979 0.978 0.001 0.995	0	0	0
	1<br>Oglab02g25180.1 0.548 0.976 0.428 0.006 0.938<br>Oglab05g19280.1 0.001 0.537 0.536 0.001 0.81	0<br>Osjap05g22990.2 0.001 0.544 0.543 0.001 0.886	1<br>Osind02g30490.1 0.548 0.98 0.432 0.006 1<br>Osind05g23630.1 0.001 0.544 0.543 0.001 0.886	1<br>Orufi02g28510.2 0.548 0.98 0.432 0.007 1<br>Orufi05g21610.1 0.001 0.536 0.535 0.001 0.97	1<br>Obart02g27080.1 0.548 0.976 0.428 0.006 0.938<br>Obart11g22940.1 0.001 0.536 0.535 0.146 0.791	1<br>Olong_005582-R1 0.546 0.986 0.44 0.145 0.995<br>Olong_026752-R1 0.001 0.539 0.538 0.001 0.973	1<br>Opunc02g24700.1 0.553 0.98 0.427 0.003 1<br>Opunc05g18050.1 0.001 0.536 0.535 0.001 0.848	1<br>Obrac02g19610.1 0.547 0.976 0.429 0.001 0.983<br>Obrac05g13790.3 0.001 0.53 0.529 0.092 0.901	0<br>Lperr11g19460.1 0.001 0.535 0.534 0.005 0.813	0<br>AT2G45910.1 0.585 0.976 0.391 0.054 0.994
Oglab12g00050.1	1<br>Oglab12g00050.1 0.003 1 0.997 0.003 1	0	0	0	0	0	0	0	0	0
	1<br>Oglab01g30880.1 0.335 0.911 0.576 0.03 0.464<br>Oglab11g00420.3 0.003 0.298 0.295 0.002 0.735	1<br>Osjap11g00430.1 0.003 0.298 0.295 0.004 0.425<br>Osjap12g00360.1 0.325 0.945 0.62 0.002 0.506	1<br>Osind12g00440.1 0.325 0.945 0.62 0.002 0.506<br>Osind12g00580.1 0.003 0.298 0.295 0.004 0.425	0<br>Orufi12g00380.1 0.325 0.945 0.62 0.002 0.506	1<br>Obart12g00130.1 0.325 0.945 0.62 0.002 0.509<br>Obart12g00140.5 0.003 0.298 0.295 0.004 0.46	1<br>Olong_014020-R1 0.359 0.937 0.578 0.055 0.353<br>Olong_016703-R1 0.003 0.298 0.295 0.004 0.423	1<br>Opunc05g17450.1 0.335 0.927 0.592 0.029 0.496<br>Opunc11g00340.1 0.003 0.291 0.288 0.007 0.696	0<br>Obrac05g13350.2 0.144 0.911 0.767 0.033 0.562	1<br>Lperr11g00380.1 0.003 0.298 0.295 0.004 0.481<br>Lperr12g00140.1 0.33 0.982 0.652 0.016 0.62	1<br>AT1G25260.1 0.003 0.293 0.29 0.004 0.409<br>AT3G24290.1 0.359 0.937 0.578 0.093 0.496
Oglab12g00800.1	1<br>Oglab12g00800.1 0.001 1 0.999 0.001 1	1<br>Osjap11g01220.1 0.001 1 0.999 0.001 1<br>Osjap12g01140.1 0.001 1 0.999 0.001 1	1<br>Osind11g00690.1 0.001 0.956 0.955 0.001 0.999<br>Osind12g01290.1 0.001 1 0.999 0.001 1	1<br>Orufi11g01000.1 0.001 1 0.999 0.001 1<br>Orufi12g01130.1 0.001 1 0.999 0.001 1	1<br>Obart11g01060.1 0.001 1 0.999 0.001 1	0	1<br>Opunc12g00940.1 0.001 1 0.999 0.001 1	0	0	0
	1<br>Oglab03g12120.1 0.49 0.983 0.493 0.121 0.633<br>Oglab07g22980.1 0.006 0.393 0.387 0.007 0.933	1<br>Osjap03g13100.1 0.49 0.983 0.493 0.097 0.622<br>Osjap07g30390.1 0.009 0.393 0.384 0.013 0.938	1<br>Osind03g12930.1 0.49 0.983 0.493 0.121 0.633<br>Osind07g27070.1 0.006 0.393 0.387 0.006 0.939	1<br>Orufi03g17220.1 0.496 1 0.504 0.027 1<br>Orufi07g27420.1 0.009 0.393 0.384 0.013 0.939	1<br>Obart03g12120.1 0.49 0.983 0.493 0.121 0.633<br>Obart12g00790.1 0.001 0.393 0.392 0.002 0.942	1<br>Olong_007715-R1 0.493 1 0.507 0.007 1<br>Olong_008211-R1 0.006 0.393 0.387 0.006 0.939	1<br>Opunc03g11680.1 0.49 1 0.51 0.148 1<br>Opunc11g00920.1 0.001 0.393 0.392 0.142 0.945	1<br>Obrac03g09720.2 0.49 1 0.51 0.142 1<br>Obrac06g16520.1 0.02 0.411 0.391 0.048 0.978	1<br>Lperr03g10720.1 0.49 0.996 0.506 0.148 0.954<br>Lperr11g00920.1 0.001 0.393 0.392 0.002 0.942	1<br>AT3G46740.1 0.493 1 0.507 0.161 1<br>AT4G20110.2 0.001 0.393 0.392 0.002 0.939
Oglab12g01430.1	1<br>Oglab12g01430.1 0.001 1 0.999 0.001 1	0	0	0	1<br>Obart12g01480.1 0.001 0.996 0.995 0.001 0.986	0	0	0	0	0
	1<br>Oglab01g17070.1 0.001 0.479 0.478 0.05 0.856<br>Oglab12g01740.1 0.587 1 0.413 0.007 0.993	1<br>Osjap01g23560.1 0.001 0.558 0.557 0.04 0.822<br>Osjap12g02170.3 0.587 1 0.413 0.007 1	1<br>Osind12g02430.1 0.001 0.587 0.586 0.002 0.95<br>Osind12g02460.1 0.587 1 0.413 0.006 0.994	1<br>Orufi01g22500.1 0.001 0.558 0.557 0.049 0.912<br>Orufi12g02140.1 0.587 1 0.413 0.007 0.993	1<br>Obart01g05330.1 0.123 0.598 0.475 0.101 0.965<br>Obart02g36960.1 0.606 0.996 0.39 0.27 0.993	1<br>Olong_015804-R1 0.001 0.59 0.589 0.002 0.976<br>Olong_027205-R1 0.592 0.996 0.404 0.004 0.84	1<br>Opunc02g34070.1 0.595 0.996 0.401 0.218 0.996<br>Opunc08g00050.1 0.001 0.479 0.478 0.03 0.777	1<br>Obrac03g08440.1 0.136 0.595 0.459 0.179 0.989<br>Obrac12g01540.1 0.606 0.996 0.39 0.017 0.983	0<br>Lperr02g30420.7 0.608 0.996 0.388 0.02 0.984<br>Lperr03g07390.1 0.174 0.562 0.388 0.175 0.868	0<br>AT2G02250.1 0.537 0.996 0.459 0.003 0.974
Oglab12g01890.1	1<br>Oglab12g01890.1 0.002 1 0.998 0.002 1	0	0	0	1<br>Obart12g01910.1 0.002 0.974 0.972 0.002 0.938	0	1<br>Opunc11g02000.1 0.002 1 0.998 0.002 0.907<br>Opunc12g01920.1 0.002 1 0.998 0.002 0.978	1<br>Obrac11g01380.1 0.023 0.972 0.949 0.036 0.95	0	0
	1<br>Oglab07g23480.1 0.737 0.998 0.261 0.002 0.386<br>Oglab11g01930.1 0.013 0.718 0.705 0.051 0.947	1<br>Osjap11g02310.2 0.739 1 0.261 0.006 1<br>Osjap12g02360.1 0.002 0.718 0.716 0.002 0.945	1<br>Osind07g27540.1 0.737 0.998 0.261 0.002 0.386<br>Osind12g02620.1 0.002 0.718 0.716 0.002 0.945	1<br>Orufi11g02050.1 0.739 1 0.261 0.006 1<br>Orufi12g02190.1 0.023 0.622 0.599 0.159 1	1<br>Obart07g26900.1 0.737 0.998 0.261 0.002 0.386<br>Obart11g02130.1 0.013 0.718 0.705 0.051 0.947	1<br>Olong_013437-R2 0.739 0.997 0.258 0.007 0.809<br>Olong_019069-R2 0.002 0.718 0.716 0.002 0.945	0<br>Opunc07g25270.1 0.737 0.998 0.261 0.003 0.43	1<br>Obrac01g08350.1 0.732 0.977 0.245 0.007 1<br>Obrac12g01700.2 0.002 0.718 0.716 0.002 0.948	1<br>Lperr03g13480.1 0.732 0.977 0.245 0.007 1<br>Lperr07g20930.1 0.026 0.718 0.692 0.09 0.956	1<br>AT2G19540.1 0.023 0.718 0.695 0.062 0.947<br>AT3G43810.1 0.732 0.977 0.245 0.007 1
Oglab12g02960.1	1<br>Oglab12g02960.1 0.001 1 0.999 0.001 1	0	0	0	0	0	1<br>Opunc12g02670.1 0.001 1 0.999 0.001 1	0	0	0
	1<br>Oglab06g24980.2 0.005 0.757 0.752 0.153 0.821<br>Oglab11g03020.1 0.782 1 0.218 0.005 1	1<br>Osjap11g03380.1 0.782 1 0.218 0.005 1<br>Osjap12g03480.1 0.001 0.738 0.737 0.002 0.965	1<br>Osind06g31110.1 0.005 0.757 0.752 0.152 0.823<br>Osind12g03520.1 0.782 1 0.218 0.008 1	1<br>Orufi12g03250.1 0.782 1 0.218 0.008 1<br>Orufi12g03260.1 0.001 0.738 0.737 0.002 0.965	1<br>Obart06g27500.1 0.005 0.757 0.752 0.152 0.823<br>Obart12g02850.1 0.782 1 0.218 0.008 1	1<br>Olong_004443-R1 0.782 1 0.218 0.01 1<br>Olong_023554-R1 0.005 0.757 0.752 0.125 0.818	1<br>Opunc06g25170.3 0.005 0.757 0.752 0.09 0.809<br>Opunc11g02740.1 0.782 1 0.218 0.005 1	1<br>Obrac06g19580.3 0.005 0.757 0.752 0.149 0.822<br>Obrac11g01880.1 0.782 0.997 0.215 0.01 0.99	1<br>Lperr01g19770.1 0.782 1 0.218 0.008 1<br>Lperr03g35610.1 0.012 0.763 0.751 0.026 0.955	0<br>AT5G04550.1 0.009 0.731 0.722 0.032 0.97

Oglab12g09470.1	1<br>Oglab12g09470.1 0.001 1 0.999 0.001 1	1<br>Osjap12g12320.1 0.001 1 0.999 0.001 1	1<br>Osind12g09530.1 0.001 1 0.999 0.001 1	1<br>Orufi12g10460.1 0.001 1 0.999 0.001 1	1<br>Obart12g09410.1 0.001 1 0.999 0.001 1	0	0	0	0	0
	1<br>Oglab01g34040.1 0.489 0.998 0.509 0.203 0.995<br>Oglab03g07280.1 0.001 0.295 0.294 0.004 0.981	1<br>Osjap01g44780.2 0.44 0.998 0.558 0.095 0.994<br>Osjap03g07600.1 0.001 0.295 0.294 0.004 0.981	1<br>Osind01g46040.1 0.44 0.998 0.558 0.151 0.995<br>Osind03g07670.1 0.001 0.295 0.294 0.004 0.981	1<br>Orufi01g42650.3 0.44 0.998 0.558 0.195 0.995<br>Orufi03g07180.1 0.001 0.295 0.294 0.004 0.981	1<br>Obart01g39260.3 0.456 0.998 0.542 0.041 0.994<br>Obart03g07210.1 0.001 0.295 0.294 0.004 0.981	1<br>Olong_021912-R1 0.457 1 0.543 0.099 1<br>Olong_021913-R1 0.001 0.296 0.295 0.004 0.988	1<br>Opunc12g08570.1 0.326 1 0.674 0.052 1<br>Opunc12g08580.1 0.001 0.296 0.295 0.004 0.988	1<br>Obrac03g05880.1 0.001 0.296 0.295 0.004 0.988<br>Obrac12g06500.1 0.461 1 0.539 0.103 1	1<br>Lperr01g33810.2 0.456 0.998 0.542 0.002 0.994<br>Lperr12g08300.1 0.001 0.296 0.295 0.004 0.985	0<br>AT4G34670.1 0.001 0.29 0.289 0.004 0.966<br>AT5G67540.2 0.489 0.993 0.504 0.053 1

(((((((_a1,rufi),_a2),longi),punctata),brachy),leersia),tair);

chimeric gene id	INGROUP		MIDGROUP					OUTGROUP		
	glab	barthii	jap	indica	rufi	longi	punctata	brachy	leersia	tair
Oglab01g01590.1	1<br>Oglab01g01590.1 0.001 1 0.999 0.001 1	1<br>Obart01g01710.1 0.001 0.987 0.986 0.001 0.986	0	1<br>Osind01g02220.1 0.001 0.999 0.998 0.056 0.987	0	1<br>Olong_021362-R1 0.001 0.999 0.998 0.048 0.986	1<br>Opunc01g01490.1 0.002 0.998 0.996 0.021 0.988<br>Opunc01g01490.2 0.002 0.998 0.996 0.024 0.988	0	0	0
	1<br>Oglab01g01570.1 0.537 0.999 0.462 0.043 0.998<br>Oglab05g03370.1 0.002 0.469 0.467 0.104 0.976	1<br>Obart01g01680.1 0.537 0.999 0.462 0.046 0.998<br>Obart05g03720.1 0.002 0.469 0.467 0.104 0.976	1<br>Osjap01g02100.1 0.001 0.469 0.468 0.002 0.973<br>Osjap01g02110.1 0.511 1 0.489 0.039 1	1<br>Osind01g02230.1 0.511 0.987 0.476 0.061 0.964<br>Osind05g03930.1 0.002 0.469 0.467 0.104 0.976	1<br>Orufi01g01810.1 0.001 0.469 0.468 0.002 0.973<br>Orufi01g01830.1 0.537 0.999 0.462 0.079 0.999	1<br>Olong_016471-R1 0.002 0.469 0.467 0.106 0.972<br>Olong_021361-R1 0.514 1 0.486 0.002 1	1<br>Opunc01g01490.3 0.537 0.993 0.456 0.056 0.981<br>Opunc05g03460.1 0.016 0.469 0.453 0.119 0.971	1<br>Obrac01g01340.1 0.485 0.998 0.513 0.001 0.999<br>Obrac01g17910.1 0.004 0.469 0.465 0.248 0.98	1<br>Lperr01g01640.1 0.509 0.999 0.49 0.051 0.998<br>Lperr05g03400.1 0.015 0.469 0.454 0.158 0.972	1<br>AT1G71040.1 0.526 0.999 0.473 0.021 0.998<br>AT3G48670.1 0.002 0.468 0.466 0.117 0.971
Oglab01g03790.1	1<br>Oglab01g03790.1 0.001 1 0.999 0.001 1	1<br>Obart01g04220.1 0.001 1 0.999 0.001 1	0	0	0	0	0	0	0	0
	1<br>Oglab05g04170.2 0.048 0.567 0.519 0.114 1<br>Oglab07g07650.1 0.62 0.997 0.377 0.002 0.778	1<br>Obart05g04510.1 0.036 0.567 0.531 0.012 1<br>Obart07g07920.1 0.62 0.997 0.377 0.002 0.778	1<br>Osjap01g04780.1 0.62 1 0.38 0.002 1<br>Osjap05g04880.1 0.048 0.567 0.519 0.008 1	1<br>Osind01g04880.1 0.62 1 0.38 0.002 1<br>Osind05g04760.1 0.048 0.567 0.519 0.008 1	1<br>Orufi01g04370.1 0.62 1 0.38 0.002 1<br>Orufi05g04740.1 0.048 0.567 0.519 0.008 1	1<br>Olong_014225-R1 0.044 0.563 0.519 0.001 0.984<br>Olong_014227-R1 0.62 1 0.38 0.002 1	1<br>Opunc01g03980.1 0.62 1 0.38 0.002 1<br>Opunc07g16230.1 0.042 0.556 0.514 0.014 0.972	1<br>Obrac01g03070.1 0.62 1 0.38 0.002 1<br>Obrac05g03450.2 0.039 0.567 0.528 0.003 1	1<br>Lperr01g03770.1 0.62 1 0.38 0.002 1<br>Lperr01g03780.1 0.039 0.561 0.522 0.002 0.979	1<br>AT5G01660.1 0.053 0.556 0.503 0.015 0.97<br>AT5G64190.1 0.62 0.997 0.377 0.002 0.998
Oglab01g06260.1	1<br>Oglab01g06260.1 0.001 1 0.999 0.001 1	0	0	0	0	0	0	0	0	0
	1<br>Oglab05g25190.3 0.025 0.527 0.502 0.041 0.991<br>Oglab06g15610.1 0.555 0.99 0.435 0.132 1	1<br>Obart01g07310.1 0.528 0.996 0.468 0.002 0.908<br>Obart05g27220.1 0.025 0.521 0.496 0.085 0.982	1<br>Osjap01g08460.1 0.561 0.991 0.43 0.03 0.997<br>Osjap05g30490.5 0.025 0.552 0.527 0.078 0.969	1<br>Osind01g08290.1 0.528 1 0.472 0.003 1<br>Osind05g30870.1 0.024 0.527 0.503 0.029 0.989	1<br>Orufi01g07850.1 0.021 0.536 0.515 0.039 0.998<br>Orufi06g18220.1 0.555 0.99 0.435 0.132 1	1<br>Olong_012629-R1 0.024 0.489 0.465 0.034 0.997<br>Olong_026782-R1 0.525 0.991 0.466 0.002 0.995	1<br>Opunc01g07000.1 0.53 0.977 0.447 0.032 0.987<br>Opunc05g24760.1 0.025 0.521 0.496 0.034 0.991	1<br>Obrac01g17790.1 0.024 0.519 0.495 0.078 0.962<br>Obrac05g15110.1 0.528 0.991 0.463 0.003 0.992	1<br>Lperr01g06950.1 0.561 0.995 0.434 0.078 1<br>Lperr05g22820.1 0.024 0.521 0.497 0.071 0.976	1<br>AT1G15000.1 0.024 0.531 0.507 0.07 0.977<br>AT1G28600.1 0.533 1 0.467 0.025 0.957
Oglab01g06470.1	1<br>Oglab01g06470.1 0.001 1 0.999 0.001 1	1<br>Obart01g07430.1 0.02 1 0.98 0.013 1	1<br>Osjap01g08660.1 0.001 1 0.999 0.001 1	1<br>Osind01g08490.1 0.001 1 0.999 0.001 1	1<br>Orufi01g08070.1 0.001 1 0.999 0.001 1	1<br>Olong_011567-R1 0.056 1 0.944 0.001 1	0	0	0	0
	1<br>Oglab01g21070.2 0.1 0.581 0.481 0.249 0.983<br>Oglab05g00530.1 0.647 0.997 0.35 0.146 1	1<br>Obart01g25090.1 0.1 0.581 0.481 0.219 0.982<br>Obart05g00540.1 0.647 0.997 0.35 0.146 1	1<br>Osjap01g29230.2 0.1 0.581 0.481 0.25 0.983<br>Osjap05g00560.2 0.647 0.997 0.35 0.146 1	1<br>Osind01g30690.1 0.1 0.581 0.481 0.267 0.982<br>Osind05g00540.1 0.647 0.997 0.35 0.126 1	1<br>Orufi01g27950.1 0.1 0.581 0.481 0.25 0.983<br>Orufi01g48560.2 0.647 0.98 0.333 0.085 0.984	0<br>Olong_003039-R1 0.647 0.997 0.35 0.128 1<br>Olong_003692-R2 0.139 0.581 0.442 0.244 0.979	1<br>Opunc01g07170.1 0.001 0.582 0.581 0.001 0.984<br>Opunc05g00510.2 0.647 0.997 0.35 0.15 1	1<br>Obrac01g05760.3 0.583 1 0.417 0.082 1<br>Obrac01g05770.3 0.001 0.582 0.581 0.001 0.985	1<br>Lperr01g07030.1 0.583 1 0.417 0.018 1<br>Lperr01g07040.2 0.001 0.582 0.581 0.054 0.989	1<br>AT1G54350.1 0.585 0.993 0.408 0.084 0.975<br>AT5G53430.1 0.071 0.581 0.51 0.164 0.984
Oglab01g10320.1	1<br>Oglab01g10320.1 0.001 1 0.999 0.001 1	0	1<br>Osjap01g13630.1 0.042 1 0.958 0.042 1	0	1<br>Orufi01g13080.1 0.042 1 0.958 0.043 1	0	0	1<br>Obrac01g09290.1 0.001 1 0.999 0.001 1	0	0
	0	1	1	1	1	1	1	0	1	1

	Oglab01g10320.2 0.463 1 0.537 0.002 1	Obart01g12120.1 0.534 1 0.466 0.002 1<br>Obart01g12130.1 0.042 0.489 0.447 0.087 0.994	Osjap01g13630.3 0.463 1 0.537 0.002 1<br>Osjap01g13640.1 0.001 0.45 0.449 0.002 0.99	Osind01g13830.1 0.534 1 0.466 0.002 1<br>Osind01g13840.1 0.001 0.45 0.449 0.002 0.99	Orufi01g13080.4 0.463 1 0.537 0.002 1<br>Orufi01g13090.1 0.001 0.45 0.449 0.002 0.99	Olong_021508-R1 0.53 1 0.47 0.064 1<br>Olong_021509-R1 0.077 0.452 0.375 0.005 0.995	Opunc01g11830.1 0.031 0.489 0.458 0.054 0.994<br>Opunc05g02420.2 0.534 1 0.466 0.002 1	Obrac05g02000.1 0.534 1 0.466 0.002 1	Lperr01g11080.1 0.001 0.45 0.449 0.002 0.989<br>Lperr05g21710.3 0.53 1 0.47 0.008 1	AT3G62130.1 0.025 0.446 0.421 0.018 0.993<br>AT4G04460.1 0.544 1 0.456 0.022 1
Oglab01g24700.1	1<br>Oglab01g24700.1 0.001 1 0.999 0.001 1<br>Oglab01g24700.2 0.001 1 0.999 0.001 1<br>Oglab01g24700.3 0.001 1 0.999 0.001 1	0	1<br>Osjap01g33510.1 0.001 1 0.999 0.022 1<br>Osjap01g33510.2 0.001 1 0.999 0.023 1<br>Osjap01g33510.3 0.001 1 0.999 0.023 1<br>Osjap01g33510.4 0.001 1 0.999 0.025 1	0	0	0	0	1<br>Obrac01g22030.1 0.002 1 0.998 0.02 0.999<br>Obrac01g22030.2 0.002 1 0.998 0.021 0.999	0	0
	0<br>Oglab02g12410.1 0.648 1 0.352 0.063 0.989<br>Oglab03g26370.1 0.242 0.511 0.269 0.103 0.969	1<br>Obart01g29130.1 0.057 0.626 0.569 0.002 0.994<br>Obart02g12550.1 0.648 1 0.352 0.063 0.989	0<br>Osjap03g28950.1 0.242 0.511 0.269 0.103 0.969<br>Osjap07g15680.1 0.64 1 0.36 0.058 0.982	1<br>Osind01g35000.1 0.057 0.626 0.569 0.002 0.994<br>Osind07g13570.1 0.643 1 0.357 0.011 0.981	1<br>Orufi01g32190.1 0.057 0.626 0.569 0.002 0.994<br>Orufi02g12700.1 0.648 1 0.352 0.063 0.989	1<br>Olong_006665-R1 0.131 0.626 0.495 0.012 0.993<br>Olong_027869-R1 0.648 1 0.352 0.064 0.997	1<br>Opunc01g28950.1 0.057 0.626 0.569 0.002 0.994<br>Opunc07g15610.1 0.642 1 0.358 0.313 0.993	0<br>Obrac02g08920.1 0.646 1 0.354 0.149 0.997<br>Obrac02g16330.1 0.249 0.476 0.227 0.21 0.922	0<br>Lperr01g24940.1 0.627 1 0.373 0.02 1<br>Lperr02g18750.1 0.249 0.476 0.227 0.461 0.947	1<br>AT3G24430.1 0.136 0.619 0.483 0.145 0.987<br>AT5G24230.1 0.648 1 0.352 0.098 0.995
Oglab01g24700.2	1<br>Oglab01g24700.1 0.001 1 0.999 0.001 1<br>Oglab01g24700.2 0.001 1 0.999 0.001 1<br>Oglab01g24700.3 0.001 1 0.999 0.001 1	0	1<br>Osjap01g33510.1 0.001 1 0.999 0.022 1<br>Osjap01g33510.2 0.001 1 0.999 0.023 1<br>Osjap01g33510.3 0.001 1 0.999 0.023 1<br>Osjap01g33510.4 0.001 1 0.999 0.025 1	0	0	0	0	1<br>Obrac01g22030.1 0.002 1 0.998 0.02 0.999<br>Obrac01g22030.2 0.002 1 0.998 0.021 0.999	0	0
	0<br>Oglab02g12410.1 0.643 1 0.357 0.063 0.989<br>Oglab03g26370.1 0.245 0.505 0.26 0.103 0.969	1<br>Obart01g29130.1 0.058 0.621 0.563 0.002 0.994<br>Obart02g12550.1 0.643 1 0.357 0.063 0.989	0<br>Osjap03g28950.1 0.245 0.505 0.26 0.103 0.969<br>Osjap07g15680.1 0.636 1 0.364 0.058 0.982	1<br>Osind01g35000.1 0.058 0.621 0.563 0.002 0.994<br>Osind07g13570.1 0.639 1 0.361 0.011 0.981	1<br>Orufi01g32190.1 0.058 0.621 0.563 0.002 0.994<br>Orufi02g12700.1 0.643 1 0.357 0.063 0.989	1<br>Olong_006665-R1 0.132 0.621 0.489 0.012 0.993<br>Olong_027869-R1 0.643 1 0.357 0.064 0.997	1<br>Opunc01g28950.1 0.058 0.621 0.563 0.002 0.994<br>Opunc07g15610.1 0.638 1 0.362 0.313 0.993	0<br>Obrac02g08920.1 0.642 1 0.358 0.149 0.997	0<br>Lperr01g24930.2 0.086 0.303 0.217 0.439 0.992<br>Lperr01g24940.1 0.623 1 0.377 0.02 1	1<br>AT3G24430.1 0.138 0.615 0.477 0.145 0.987<br>AT5G24230.1 0.643 1 0.357 0.098 0.995
Oglab01g28270.1	1<br>Oglab01g28270.1 0.001 1 0.999 0.001 1	1<br>Obart01g33610.1 0.022 1 0.978 0.001 1	0	0	0	0	0	0	0	0
	1<br>Oglab03g01040.1 0.021 0.23 0.209 0.029 0.99<br>Oglab10g10080.1 0.374 0.998 0.624 0.138 0.999	0<br>Obart10g10470.1 0.374 0.998 0.624 0.121 0.999	1<br>Osjap01g38450.1 0.309 1 0.691 0.022 1<br>Osjap03g01290.1 0.001 0.234 0.233 0.001 0.185	1<br>Osind01g39980.1 0.273 1 0.727 0.117 1<br>Osind01g39990.1 0.001 0.23 0.229 0.005 0.991	1<br>Orufi01g36790.1 0.309 1 0.691 0.022 1<br>Orufi01g36800.1 0.001 0.23 0.229 0.005 0.991	0<br>Olong_007589-R1 0.374 0.998 0.624 0.19 0.999	1<br>Opunc01g32680.1 0.309 1 0.691 0.022 1<br>Opunc03g01090.1 0.001 0.23 0.229 0.004 0.991	1<br>Obrac01g25800.1 0.309 1 0.691 0.022 1<br>Obrac01g25810.1 0.001 0.23 0.229 0.005 0.991	1<br>Lperr01g29020.1 0.294 1 0.706 0.001 1<br>Lperr01g29030.1 0.001 0.23 0.229 0.005 0.991	1<br>AT1G69290.1 0.33 1 0.67 0.036 1<br>AT3G13230.1 0.006 0.23 0.224 0.009 1
Oglab01g34910.1	1<br>Oglab01g34910.1 0.002 1 0.998 0.002 1	1<br>Obart01g40120.1 0.002 1 0.998 0.002 1<br>Obart01g40120.2 0.002 1 0.998 0.002 1	1<br>Osjap01g45670.1 0.002 1 0.998 0.003 1	1<br>Osind01g46990.1 0.002 1 0.998 0.002 1	1<br>Orufi01g43430.1 0.123 1 0.877 0.351 1	1<br>Olong_025215-R1 0.152 0.975 0.823 0.002 0.977	0	0	0	0
	1<br>Oglab01g34890.1 0.123 0.39 0.267 0.646 0.992<br>Oglab11g07730.1 0.439 0.993 0.554 0.021 0.416	0<br>Obart11g08370.2 0.377 0.993 0.616 0.022 0.446	1<br>Osjap01g45650.1 0.123 0.39 0.267 0.646 0.992<br>Osjap11g09760.2 0.439 0.993 0.554 0.021 0.416	1<br>Osind01g46870.1 0.002 0.285 0.283 0.007 0.986<br>Osind11g07730.1 0.453 0.993 0.54 0.002 0.401	0<br>Orufi01g43430.3 0.285 1 0.715 0.045 1	1<br>Olong_009328-R1 0.123 0.37 0.247 0.649 0.983<br>Olong_020581-R1 0.439 0.993 0.554 0.021 0.416	1<br>Opunc04g15200.1 0.123 0.37 0.247 0.687 0.984<br>Opunc11g08140.1 0.377 0.993 0.616 0.122 0.503	1<br>Obrac01g31030.2 0.07 0.381 0.311 0.454 0.952<br>Obrac11g05320.1 0.439 0.993 0.554 0.021 0.416	1<br>Lperr01g34610.3 0.123 0.39 0.267 0.645 0.992<br>Lperr11g07510.1 0.439 0.993 0.554 0.021 0.416	1<br>AT3G28450.1 0.439 0.998 0.559 0.023 0.443<br>AT5G16960.1 0.123 0.383 0.26 0.65 1
Oglab01g37540.3	1<br>Oglab01g37540.1 0.002 0.969 0.967 0.002 0.971<br>Oglab01g37540.2 0.002 0.969 0.967 0.002 0.97<br>Oglab01g37540.3 0.002 1 0.998 0.002 1<br>Oglab01g37540.4 0.002 0.969 0.967 0.003 0.959	0	0	0	0	0	0	0	0	0
	1	1	1	1	1	0	1	1	0	0

	Oglab02g03680.1 0.257 0.969 0.712 0.038 0.962<br>Oglab05g11700.1 0.002 0.215 0.213 0.005 0.575	Obart01g42890.1 0.358 0.969 0.611 0.004 0.946<br>Obart05g12620.1 0.002 0.215 0.213 0.005 0.575	Osjap02g03900.1 0.257 0.969 0.712 0.058 0.963<br>Osjap05g14440.1 0.002 0.215 0.213 0.005 0.575	Osind01g50140.1 0.002 0.218 0.216 0.008 0.829<br>Osind02g04170.1 0.257 0.969 0.712 0.036 0.962	Orufi01g46330.1 0.358 0.969 0.611 0.004 0.947<br>Orufi05g13610.1 0.002 0.215 0.213 0.005 0.575	Olong_010223-R1 0.004 0.224 0.22 0.014 0.53<br>Olong_014935-R1 0.259 0.527 0.268 0.336 0.935<br>Olong_014940-R4 0.533 0.782 0.249 0.02 0.141	Opunc02g02910.2 0.272 0.941 0.669 0.028 0.92<br>Opunc05g10670.1 0.002 0.218 0.216 0.005 0.58	Obrac02g02690.2 0.362 1 0.638 0.015 1<br>Obrac05g08200.1 0.002 0.228 0.226 0.008 0.953	Lperr02g03270.1 0.358 0.941 0.583 0.003 0.907<br>Lperr05g10020.1 0.002 0.211 0.209 0.005 0.608	AT2G28500.1 0.01 0.272 0.262 0.233 0.832<br>AT5G24630.6 0.636 0.868 0.232 0.599 0.857
Oglab02g00240.1	1<br>Oglab02g00240.1 0.001 1 0.999 0.001 1	1<br>Obart02g00330.1 0.001 1 0.999 0.011 1	1<br>Osjap02g00350.2 0.193 1 0.807 0.001 1	0	1<br>Orufi02g00370.1 0.019 0.959 0.94 0.382 0.979<br>Orufi02g00370.2 0.019 0.959 0.94 0.405 0.98	1<br>Olong_001783-R1 0.066 0.948 0.882 0.001 0.991	0	1<br>Obrac02g00300.1 0.155 0.983 0.828 0.005 0.995<br>Obrac02g00300.2 0.155 0.983 0.828 0.005 0.995	0	0
	1<br>Oglab04g13000.1 0.22 0.99 0.77 0.023 0.994<br>Oglab09g13130.1 0.001 0.203 0.202 0.053 0.995	0<br>Obart04g14710.1 0.22 0.99 0.77 0.025 0.994	1<br>Osjap02g00350.3 0.206 1 0.794 0.004 1<br>Osjap02g00360.1 0.001 0.204 0.203 0.052 1	1<br>Osind02g00340.1 0.213 1 0.787 0.001 1<br>Osind02g00350.1 0.001 0.204 0.203 0.052 1	1<br>Orufi04g16100.1 0.22 0.99 0.77 0.023 0.994<br>Orufi09g15710.1 0.001 0.203 0.202 0.053 0.995	0<br>Olong_004740-R1 0.212 0.992 0.78 0.004 0.996	1<br>Opunc02g00010.2 0.001 0.204 0.203 0.052 1<br>Opunc11g05460.1 0.217 1 0.783 0.002 1	1<br>Obrac09g06000.3 0.216 0.999 0.783 0.007 0.975<br>Obrac09g08720.1 0.001 0.203 0.202 0.053 0.995	1<br>Lperr02g00330.1 0.214 1 0.786 0.001 1<br>Lperr02g00340.1 0.001 0.204 0.203 0.053 1	1<br>AT1G33140.1 0.001 0.203 0.202 0.052 0.995<br>AT3G30340.1 0.22 0.996 0.776 0.027 0.995
Oglab02g12130.1	1<br>Oglab02g12130.1 0.002 1 0.998 0.002 1	0	0	0	0	0	0	0	1<br>Lperr02g10210.1 0.08 0.995 0.915 0.066 0.997	0
	0<br>Oglab02g12130.2 0.002 0.666 0.664 0.002 0.859	1<br>Obart02g12130.1 0.667 0.995 0.328 0.099 0.988<br>Obart02g12140.1 0.002 0.666 0.664 0.002 0.881	1<br>Osjap02g13370.1 0.667 0.995 0.328 0.09 0.899<br>Osjap02g13380.1 0.002 0.666 0.664 0.002 0.881	1<br>Osind02g13570.1 0.667 0.995 0.328 0.09 0.899<br>Osind02g13580.1 0.002 0.666 0.664 0.002 0.881	0<br>Orufi02g12280.1 0.002 0.666 0.664 0.002 0.881	1<br>Olong_006942-R1 0.667 0.995 0.328 0.077 0.991<br>Olong_006943-R1 0.157 0.666 0.509 0.003 0.853	1<br>Opunc02g10840.1 0.667 0.997 0.33 0.069 0.918<br>Opunc02g10850.2 0.082 0.666 0.584 0.104 0.883	1<br>Obrac02g08670.1 0.703 0.997 0.294 0.005 0.87<br>Obrac02g08690.1 0.157 0.666 0.509 0.003 0.847	0<br>Lperr02g10200.1 0.708 0.997 0.289 0.007 0.662	0<br>AT4G32770.1 0.023 0.777 0.754 0.043 0.986
Oglab02g22130.2	1<br>Oglab02g22130.2 0.001 1 0.999 0.001 1	1<br>Obart02g23460.2 0.001 1 0.999 0.001 1	1<br>Osjap02g26750.1 0.001 0.969 0.968 0.001 0.994<br>Osjap02g26750.2 0.001 0.969 0.968 0.001 0.994<br>Osjap02g26750.4 0.001 0.969 0.968 0.001 0.799	1<br>Osind02g26640.2 0.001 1 0.999 0.001 1	1<br>Orufi02g24900.1 0.011 0.974 0.963 0.06 0.859<br>Orufi02g24900.3 0.001 0.974 0.973 0.001 0.74<br>Orufi02g24900.4 0.001 0.974 0.973 0.001 0.737	0	1<br>Opunc02g21410.1 0.011 0.999 0.988 0.116 0.996<br>Opunc02g21410.2 0.011 0.999 0.988 0.114 0.996<br>Opunc02g21410.3 0.011 0.999 0.988 0.115 0.996<br>Opunc02g21410.4 0.011 0.999 0.988 0.114 0.996	0	1<br>Lperr02g18970.1 0.011 0.997 0.986 0.032 0.996	0
	1<br>Oglab02g21950.1 0.475 0.999 0.524 0.073 0.995<br>Oglab02g22110.2 0.011 0.459 0.448 0.532 0.98	1<br>Obart02g23290.1 0.475 0.999 0.524 0.029 0.999<br>Obart02g23440.1 0.011 0.459 0.448 0.492 0.981	1<br>Osjap02g26590.1 0.475 0.999 0.524 0.033 0.999<br>Osjap02g26750.5 0.011 0.459 0.448 0.238 0.973	1<br>Osind02g26450.1 0.475 0.999 0.524 0.025 0.997<br>Osind02g26620.1 0.011 0.459 0.448 0.495 0.981	1<br>Orufi02g24730.1 0.475 0.994 0.519 0.031 0.996<br>Orufi02g24880.2 0.011 0.459 0.448 0.18 0.97	0<br>Olong_024345-R1 0.011 0.474 0.463 0.442 0.991	1<br>Opunc02g21230.1 0.479 0.976 0.497 0.003 0.934<br>Opunc02g21240.1 0.011 0.461 0.45 0.121 0.972	1<br>Obrac02g16670.1 0.549 0.973 0.424 0.076 0.995<br>Obrac02g16700.1 0.011 0.461 0.45 0.114 0.947	1<br>Lperr02g18740.1 0.485 0.997 0.512 0.056 0.991<br>Lperr02g19120.1 0.011 0.484 0.473 0.183 0.978	1<br>AT4G31860.1 0.014 0.45 0.436 0.123 0.989<br>AT5G43080.1 0.626 1 0.374 0.194 0.932
Oglab02g33340.1	1<br>Oglab02g33340.1 0.004 1 0.996 0.004 1	1<br>Obart02g36180.1 0.069 0.989 0.92 0.061 0.778<br>Obart02g36180.2 0.069 0.989 0.92 0.073 0.734<br>Obart02g36180.3 0.069 0.989 0.92 0.076 0.724	1<br>Osjap02g40310.1 0.042 0.973 0.931 0.432 0.926<br>Osjap02g40340.1 0.042 1 0.958 0.361 1<br>Osjap02g40370.1 0.042 0.973 0.931 0.267 0.726	1<br>Osind02g39860.1 0.042 0.977 0.935 0.257 0.659<br>Osind02g39860.2 0.069 0.977 0.908 0.058 0.571<br>Osind02g39860.3 0.069 0.973 0.904 0.061 0.631<br>Osind02g39860.4 0.069 0.973 0.904 0.066 0.679	1<br>Orufi02g37680.1 0.069 0.989 0.92 0.067 0.86<br>Orufi02g37680.10 0.069 0.989 0.92 0.091 0.811<br>Orufi02g37680.2 0.069 0.989 0.92 0.07 0.854<br>Orufi02g37680.3 0.069 0.989 0.92 0.076 0.895<br>Orufi02g37680.4 0.069 0.989 0.92 0.073 0.849<br>Orufi02g37680.5 0.069 0.989 0.92 0.076 0.841<br>Orufi02g37680.6 0.069 0.989 0.92 0.088 0.879<br>Orufi02g37680.7 0.069 0.973 0.904 0.079 0.855<br>Orufi02g37680.9 0.069 0.973 0.904 0.086 0.843	0	0	0	0	0
	1<br>Oglab02g34400.1 0.483 0.992 0.509 0.434 0.804<br>Oglab03g25400.1 0.073 0.41 0.337 0.475 0.707	1<br>Obart02g37290.1 0.483 0.992 0.509 0.434 0.804<br>Obart05g21320.1 0.061 0.387 0.326 0.588 0.777	1<br>Osjap02g41540.1 0.483 0.992 0.509 0.434 0.804<br>Osjap05g23970.1 0.061 0.387 0.326 0.669 0.821	1<br>Osind02g41040.1 0.483 0.992 0.509 0.459 0.813<br>Osind05g24480.1 0.061 0.387 0.326 0.588 0.777	0<br>Orufi02g37680.8 0.372 0.989 0.617 0.159 0.801	1<br>Olong_011081-R1 0.479 1 0.521 0.05 1<br>Olong_025373-R1 0.073 0.41 0.337 0.236 0.574	1<br>Opunc02g34450.1 0.483 0.992 0.509 0.432 0.803<br>Opunc05g18980.1 0.061 0.383 0.322 0.588 0.771	1<br>Obrac02g27500.6 0.483 0.992 0.509 0.432 0.803<br>Obrac05g14470.1 0.061 0.383 0.322 0.588 0.771	1<br>Lperr02g30750.1 0.483 0.992 0.509 0.453 0.811<br>Lperr04g17770.1 0.038 0.383 0.345 0.48 0.678	1<br>AT1G16330.1 0.038 0.414 0.376 0.701 0.873<br>AT2G20360.1 0.483 0.992 0.509 0.423 0.799

Oglab02g34160.1	1<br>Oglab02g34160.1 0.001 1 0.999 0.001 1	0	0	0	0	0	0	0	0	0
	0	1<br>Obart02g37040.1 0.704 1 0.296 0.012 1<br>Obart02g37050.1 0.001 0.704 0.703 0.002 0.997	1<br>Osjap02g41280.1 0.001 0.672 0.671 0.002 0.99<br>Osjap10g15310.1 0.694 0.992 0.298 0.289 0.967	1<br>Osind02g40750.1 0.726 1 0.274 0.038 1<br>Osind02g40760.1 0.001 0.672 0.671 0.002 0.99	1<br>Orufi02g38510.1 0.704 1 0.296 0.016 1<br>Orufi02g38520.5 0.001 0.672 0.671 0.002 0.998	1<br>Olong_018845-R1 0.049 0.672 0.623 0.05 0.99<br>Olong_022698-R2 0.694 0.992 0.298 0.04 0.953	1<br>Opunc02g34200.1 0.049 0.673 0.624 0.021 0.916<br>Opunc10g11320.1 0.694 0.992 0.298 0.052 0.944	1<br>Obrac02g27240.1 0.704 1 0.296 0.012 1<br>Obrac02g27260.4 0.055 0.679 0.624 0.038 1	1<br>Lperr02g30470.1 0.704 1 0.296 0.011 1<br>Lperr02g30480.1 0.05 0.672 0.622 0.048 0.899	1<br>AT1G04010.1 0.017 0.671 0.654 0.011 0.913<br>AT3G07090.1 0.704 0.992 0.288 0.011 0.977
Oglab02g34920.2	1<br>Oglab02g34920.2 0.001 1 0.999 0.001 1	0	0	0	0	0	0	0	0	0
	0<br>Oglab02g34920.1 0.001 0.738 0.737 0.001 0.991	1<br>Obart02g37790.1 0.001 0.728 0.727 0.001 0.993<br>Obart12g07130.1 0.73 1 0.27 0.138 1	1<br>Osjap02g42090.2 0.001 0.738 0.737 0.001 0.991<br>Osjap04g03680.2 0.739 1 0.261 0.325 1	1<br>Osind02g41670.1 0.001 0.738 0.737 0.001 0.991<br>Osind04g00200.1 0.739 1 0.261 0.194 0.981	1<br>Orufi02g39350.1 0.001 0.738 0.737 0.001 0.991<br>Orufi02g39370.2 0.739 1 0.261 0.203 1	1<br>Olong_006251-R1 0.001 0.738 0.737 0.002 0.989<br>Olong_026422-R1 0.739 1 0.261 0.203 1	1<br>Opunc02g35010.1 0.001 0.738 0.737 0.001 0.991<br>Opunc08g03270.1 0.739 1 0.261 0.209 1	0<br>Obrac02g27970.1 0.204 0.738 0.534 0.105 0.989<br>Obrac04g01750.2 0.739 1 0.261 0.227 1	1<br>Lperr02g31260.2 0.001 0.738 0.737 0.001 0.991<br>Lperr04g01800.2 0.739 1 0.261 0.124 1	1<br>AT1G33030.1 0.739 1 0.261 0.185 0.994<br>AT5G04460.1 0.012 0.738 0.726 0.022 0.992
Oglab03g00390.1	1<br>Oglab03g00390.1 0.001 1 0.999 0.001 1	1<br>Obart03g00550.1 0.001 0.994 0.993 0.001 0.996	1<br>Osjap03g00680.1 0.001 1 0.999 0.001 1<br>Osjap03g00680.2 0.001 1 0.999 0.001 1<br>Osjap03g00680.3 0.001 1 0.999 0.001 1	1<br>Osind03g00740.1 0.001 0.994 0.993 0.001 0.996	1<br>Orufi03g12660.1 0.001 1 0.999 0.001 1<br>Orufi03g12660.2 0.001 1 0.999 0.001 1	0	0	0	0	0
	1<br>Oglab02g34920.1 0.058 0.634 0.576 0.131 1<br>Oglab09g02040.2 0.696 1 0.304 0.07 1	1<br>Obart03g02140.1 0.652 1 0.348 0.013 1<br>Obart09g20260.1 0.152 0.634 0.482 0.12 1	1<br>Osjap02g42090.1 0.049 0.634 0.585 0.065 1<br>Osjap04g17560.1 0.696 0.998 0.302 0.466 0.996	1<br>Osind02g41670.1 0.058 0.634 0.576 0.131 1<br>Osind03g02220.1 0.652 1 0.348 0.015 1	1<br>Orufi02g39350.1 0.058 0.634 0.576 0.131 1<br>Orufi09g02220.1 0.696 1 0.304 0.072 1	0<br>Olong_013175-R1 0.253 0.906 0.653 0.002 0.961	1<br>Opunc03g00470.1 0.001 0.73 0.729 0.002 0.996<br>Opunc11g04590.1 0.783 0.998 0.215 0.023 1	0<br>Obrac02g10260.1 0.736 0.999 0.263 0.286 0.995<br>Obrac02g27970.1 0.128 0.634 0.506 0.105 1	1<br>Lperr03g00570.1 0.001 0.634 0.633 0.002 1<br>Lperr12g09110.1 0.699 0.998 0.299 0.295 0.967	0<br>AT5G04460.1 0.017 0.634 0.617 0.081 1
Oglab03g05040.1	1<br>Oglab03g05040.1 0.001 1 0.999 0.001 1	0	0	0	0	0	0	0	1<br>Lperr03g04520.1 0.006 1 0.994 0.004 1	0
	0<br>Oglab01g32450.1 0.518 0.996 0.478 0.05 0.943	1<br>Obart03g05120.1 0.483 1 0.517 0.002 1<br>Obart03g05130.1 0.12 0.48 0.36 0.002 0.922	1<br>Osjap03g05170.1 0.483 1 0.517 0.002 1<br>Osjap03g05190.1 0.001 0.48 0.479 0.002 0.94	1<br>Osind03g05440.1 0.483 1 0.517 0.002 1<br>Osind03g05450.1 0.001 0.48 0.479 0.002 0.94	1<br>Orufi03g04890.1 0.483 1 0.517 0.002 1<br>Orufi03g04900.1 0.001 0.48 0.479 0.002 0.94	1<br>Olong_009894-R1 0.524 0.996 0.472 0.047 0.955<br>Olong_019322-R1 0.12 0.48 0.36 0.003 0.907	1<br>Opunc03g04830.1 0.483 1 0.517 0.002 1<br>Opunc03g04840.1 0.001 0.48 0.479 0.002 0.941	1<br>Obrac03g04160.1 0.001 0.48 0.479 0.002 0.926<br>Obrac10g10430.1 0.513 0.998 0.485 0.043 0.998	0<br>Lperr10g10310.1 0.491 0.997 0.506 0.042 0.987	1<br>AT1G26760.1 0.01 0.438 0.428 0.035 0.875<br>AT5G23190.1 0.5 0.996 0.496 0.064 0.961
Oglab03g11620.1	1<br>Oglab03g11620.1 0.001 1 0.999 0.001 1<br>Oglab03g11620.2 0.001 0.994 0.993 0.001 0.994<br>Oglab03g11620.3 0.001 0.994 0.993 0.001 0.994	0	0	0	0	1<br>Olong_011801-R1 0.001 0.994 0.993 0.001 0.993	0	0	1<br>Lperr03g10320.1 0.001 0.999 0.998 0.07 0.992	0
	0<br>Oglab02g22540.1 0.68 0.99 0.31 0.007 0.989	1<br>Obart03g11600.1 0.68 1 0.32 0.015 1<br>Obart03g11610.3 0.001 0.648 0.647 0.001 0.995	1<br>Osjap03g12550.2 0.68 0.994 0.314 0.013 0.86<br>Osjap03g12560.3 0.001 0.648 0.647 0.001 0.994	1<br>Osind03g12280.1 0.68 1 0.32 0.015 1<br>Osind03g12290.1 0.001 0.64 0.639 0.001 0.991	1<br>Orufi03g11950.1 0.68 1 0.32 0.015 1<br>Orufi03g11960.1 0.001 0.648 0.647 0.001 0.993	0	1<br>Opunc03g11160.1 0.001 0.648 0.647 0.001 0.995<br>Opunc07g10560.1 0.683 0.997 0.314 0.014 1	1<br>Obrac03g09320.1 0.68 0.995 0.315 0.015 0.996<br>Obrac03g09330.1 0.001 0.648 0.647 0.001 0.993	1<br>Lperr03g10320.2 0.001 0.648 0.647 0.098 0.989<br>Lperr07g09930.1 0.684 0.991 0.307 0.01 0.607	1<br>AT1G04730.1 0.016 0.648 0.632 0.023 0.994<br>AT3G09030.1 0.68 0.995 0.315 0.013 0.998
Oglab03g11620.2	1<br>Oglab03g11620.1 0.001 0.994 0.993 0.001 0.994<br>Oglab03g11620.2 0.001 1 0.999 0.001 1<br>Oglab03g11620.3 0.001 0.994 0.993 0.001 0.994	0	0	0	0	1<br>Olong_011801-R1 0.001 0.994 0.993 0.001 0.993	0	0	1<br>Lperr03g10320.1 0.001 0.999 0.998 0.07 0.992	0
	0<br>Oglab02g22540.1 0.676 0.99 0.314 0.007 0.989	1<br>Obart03g11600.1 0.675 1 0.325 0.015 1	1<br>Osjap03g12550.2 0.675 0.994 0.319 0.013 0.86	1<br>Osind03g12280.1 0.675 1 0.325 0.015 1	1<br>Orufi03g11950.1 0.675 1 0.325 0.015 1	0	1<br>Opunc03g11160.1 0.001 0.644 0.643 0.001 0.995	1<br>Obrac03g09320.1 0.675 0.995 0.32 0.015 0.996	1<br>Lperr03g10320.2 0.001 0.643 0.642 0.098 0.989	1<br>AT1G04730.1 0.016 0.643 0.627 0.023 0.994

		Obart03g11610.3 0.001 0.644 0.643 0.001 0.995	Osjap03g12560.3 0.001 0.644 0.643 0.001 0.994	Osind03g12290.1 0.001 0.635 0.634 0.001 0.991	Orufi03g11960.6 0.001 0.644 0.643 0.001 0.995		Opunc07g10560.1 0.679 0.997 0.318 0.014 1	Obrac03g09330.1 0.001 0.643 0.642 0.001 0.993	Lperr07g09930.1 0.679 0.99 0.311 0.01 0.607	AT3G09030.1 0.675 0.995 0.32 0.013 0.998
Oglab03g11620.3	1<br>Oglab03g11620.1 0.001 0.994 0.993 0.001 0.994<br>Oglab03g11620.2 0.001 0.994 0.993 0.001 0.994<br>Oglab03g11620.3 0.001 1 0.999 0.001 1	0	0	0	0	1<br>Olong_011801-R1 0.001 0.994 0.993 0.001 0.993	0	0	1<br>Lperr03g10320.1 0.001 0.999 0.998 0.07 0.992	0
	0<br>Oglab02g22540.1 0.662 0.989 0.327 0.007 0.989	1<br>Obart03g11600.1 0.661 1 0.339 0.015 1<br>Obart03g11610.3 0.001 0.628 0.627 0.001 0.995	1<br>Osjap03g12550.2 0.661 0.994 0.333 0.013 0.86<br>Osjap03g12560.3 0.001 0.628 0.627 0.001 0.994	1<br>Osind03g12280.1 0.661 1 0.339 0.015 1<br>Osind03g12290.1 0.001 0.62 0.619 0.001 0.991	1<br>Orufi03g11950.1 0.661 1 0.339 0.015 1<br>Orufi03g11960.1 0.001 0.628 0.627 0.001 0.993	0	1<br>Opunc03g11160.1 0.001 0.628 0.627 0.001 0.995<br>Opunc07g10560.1 0.665 0.996 0.331 0.014 1	1<br>Obrac03g09320.1 0.661 0.995 0.334 0.015 0.996<br>Obrac03g09330.1 0.001 0.628 0.627 0.001 0.993	1<br>Lperr03g10320.2 0.001 0.628 0.627 0.098 0.989<br>Lperr07g09930.1 0.665 0.99 0.325 0.01 0.607	1<br>AT1G04730.1 0.017 0.628 0.611 0.023 0.994<br>AT3G09030.1 0.661 0.995 0.334 0.013 0.998
Oglab03g12120.1	1<br>Oglab03g12120.1 0.001 1 0.999 0.001 1	1<br>Obart03g12120.1 0.001 1 0.999 0.001 1	1<br>Osjap03g13100.1 0.028 1 0.972 0.001 1	1<br>Osind03g12930.1 0.001 1 0.999 0.001 1	0	0	0	0	0	0
	1<br>Oglab06g01620.1 0.642 0.998 0.356 0.212 0.912<br>Oglab12g00800.1 0.114 0.633 0.519 0.483 0.983	1<br>Obart04g15560.1 0.64 0.998 0.358 0.146 0.877<br>Obart11g01060.1 0.114 0.633 0.519 0.471 0.982	1<br>Osjap04g16240.1 0.642 0.998 0.356 0.067 0.921<br>Osjap12g01140.1 0.109 0.633 0.524 0.498 0.981	1<br>Osind04g14790.1 0.642 0.998 0.356 0.013 0.911<br>Osind12g01290.1 0.109 0.633 0.524 0.49 0.981	1<br>Orufi06g01460.3 0.642 0.998 0.356 0.212 0.912<br>Orufi12g01130.1 0.109 0.633 0.524 0.498 0.981	1<br>Olong_007714-R9 0.642 1 0.358 0.002 1<br>Olong_007715-R1 0.125 0.633 0.508 0.007 0.965	1<br>Opunc03g11680.1 0.028 0.633 0.605 0.001 0.971<br>Opunc04g11270.1 0.642 0.998 0.356 0.067 0.921	1<br>Obrac03g09720.1 0.025 0.633 0.608 0.036 0.971<br>Obrac04g09430.1 0.64 0.998 0.358 0.014 0.863	1<br>Lperr03g10720.1 0.028 0.633 0.605 0.001 0.934<br>Lperr03g17470.1 0.633 0.998 0.365 0.119 0.828	1<br>AT3G46740.1 0.122 0.633 0.511 0.158 0.971<br>AT3G53360.1 0.672 0.998 0.326 0.09 0.934
Oglab03g30700.1	1<br>Oglab03g30700.1 0.001 1 0.999 0.001 1	0	0	0	0	0	0	0	0	0
	0<br>Oglab09g01300.1 0.363 0.989 0.626 0.247 0.934	1<br>Obart01g06810.1 0.109 0.347 0.238 0.004 0.594<br>Obart03g31630.1 0.363 0.989 0.626 0.159 0.946	1<br>Osjap01g13740.2 0.123 0.349 0.226 0.054 0.616<br>Osjap09g01290.1 0.363 0.989 0.626 0.355 0.949	1<br>Osind01g07780.1 0.109 0.347 0.238 0.004 0.594<br>Osind09g01320.1 0.363 0.989 0.626 0.247 0.934	1<br>Orufi01g07380.1 0.109 0.347 0.238 0.004 0.594<br>Orufi09g01300.1 0.363 0.989 0.626 0.247 0.934	1<br>Olong_000655-R2 0.112 0.339 0.227 0.012 0.53<br>Olong_014805-R1 0.363 0.989 0.626 0.189 0.924	1<br>Opunc03g28820.1 0.001 0.363 0.362 0.016 0.988<br>Opunc03g28830.1 0.363 0.989 0.626 0.247 0.934	0<br>Obrac05g04080.2 0.363 0.989 0.626 0.21 0.929	1<br>Lperr03g26260.1 0.363 0.989 0.626 0.247 0.934<br>Lperr12g17090.1 0.001 0.358 0.357 0.015 0.941	1<br>AT2G21390.1 0.363 0.989 0.626 0.247 0.933<br>AT5G60550.1 0.081 0.352 0.271 0.16 0.943
Oglab03g30830.1	1<br>Oglab03g30830.1 0.001 1 0.999 0.001 1	1<br>Obart03g31750.1 0.001 1 0.999 0.046 1	1<br>Osjap03g34330.1 0.001 1 0.999 0.001 1	1<br>Osind03g35550.1 0.001 1 0.999 0.039 1	1<br>Orufi03g33060.1 0.001 1 0.999 0.001 1	0	0	0	0	0
	1<br>Oglab03g34460.1 0.629 0.997 0.368 0.016 0.993<br>Oglab10g04340.1 0.012 0.583 0.571 0.1 0.963	1<br>Obart03g36200.1 0.652 1 0.348 0.016 0.997<br>Obart10g04600.1 0.013 0.583 0.57 0.106 0.962	1<br>Osjap03g39110.1 0.652 1 0.348 0.036 1<br>Osjap10g05370.1 0.012 0.583 0.571 0.1 0.963	1<br>Osind01g46540.1 0.652 0.999 0.347 0.065 0.994<br>Osind10g03770.1 0.012 0.583 0.571 0.11 0.96	1<br>Orufi10g04850.1 0.029 0.583 0.554 0.093 0.963<br>Orufi11g12520.1 0.653 1 0.347 0.038 0.991	1<br>Olong_014796-R1 0.001 0.495 0.494 0.002 0.95<br>Olong_024872-R1 0.648 1 0.352 0.01 1	1<br>Opunc01g42030.1 0.652 1 0.348 0.28 0.872<br>Opunc03g28940.2 0.001 0.6 0.599 0.002 0.995	1<br>Obrac02g16280.1 0.646 0.982 0.336 0.063 0.949<br>Obrac03g23440.1 0.001 0.6 0.599 0.002 0.995	1<br>Lperr03g26400.1 0.001 0.6 0.599 0.002 0.995<br>Lperr03g30520.1 0.644 1 0.356 0.003 0.997	0<br>AT1G70730.3 0.001 0.6 0.599 0.118 0.995
Oglab03g32170.1	1<br>Oglab03g32170.1 0.003 1 0.997 0.003 1<br>Oglab03g32170.2 0.003 1 0.997 0.003 1	1<br>Obart03g33760.1 0.003 1 0.997 0.003 1	1<br>Osjap03g36400.1 0.003 1 0.997 0.003 1	1<br>Osind03g37600.1 0.003 1 0.997 0.003 1	1<br>Orufi03g35150.1 0.003 1 0.997 0.003 1	1<br>Olong_002225-R1 0.003 1 0.997 0.136 1<br>Olong_002225-R2 0.003 1 0.997 0.003 1	1<br>Opunc03g30920.1 0.003 1 0.997 0.003 1	0	0	0
	0<br>Oglab05g19940.1 0.32 0.991 0.671 0.015 0.997	1<br>Obart01g34520.1 0.547 0.991 0.444 0.027 0.818<br>Obart03g04650.1 0.003 0.414 0.411 0.007 1	1<br>Osjap01g12980.2 0.547 0.979 0.432 0.032 0.961<br>Osjap03g04690.1 0.003 0.414 0.411 0.007 1	1<br>Osind03g04960.1 0.003 0.414 0.411 0.007 1<br>Osind12g02610.1 0.538 0.991 0.453 0.005 0.759	1<br>Orufi03g04350.1 0.003 0.414 0.411 0.007 1<br>Orufi05g22410.2 0.547 0.997 0.45 0.031 0.969	1<br>Olong_020510-R2 0.523 0.991 0.468 0.291 0.873<br>Olong_025165-R1 0.003 0.414 0.411 0.007 1	0<br>Opunc12g01920.1 0.526 0.973 0.447 0.663 0.947	0<br>Obrac03g25020.5 0.003 0.535 0.532 0.439 0.972	1<br>Lperr01g29830.2 0.532 0.991 0.459 0.203 0.858<br>Lperr03g28250.1 0.003 0.414 0.411 0.007 1	1<br>AT1G52740.1 0.003 0.414 0.411 0.007 1<br>AT4G14640.1 0.544 0.979 0.435 0.033 0.987
Oglab03g32170.2	1<br>Oglab03g32170.1 0.003 1 0.997 0.003 1<br>Oglab03g32170.2 0.003 1 0.997 0.003 1	1<br>Obart03g33760.1 0.003 1 0.997 0.003 1	1<br>Osjap03g36400.1 0.003 1 0.997 0.003 1	1<br>Osind03g37600.1 0.003 1 0.997 0.003 1	1<br>Orufi03g35150.1 0.003 1 0.997 0.003 1	1<br>Olong_002225-R1 0.003 1 0.997 0.136 1<br>Olong_002225-R2 0.003 1 0.997 0.003 1	1<br>Opunc03g30920.1 0.003 1 0.997 0.003 1	0	0	0
	0	1	1	1	1	1	0	0	1	1

	Oglab05g19940.1 0.32 0.991 0.671 0.015 0.997	Obart01g34520.1 0.547 0.991 0.444 0.027 0.818<br>Obart03g04650.1 0.003 0.414 0.411 0.007 1	Osjap01g12980.2 0.547 0.979 0.432 0.032 0.961<br>Osjap03g04690.1 0.003 0.414 0.411 0.007 1	Osind03g04960.1 0.003 0.414 0.411 0.007 1<br>Osind12g02610.1 0.538 0.991 0.453 0.005 0.759	Orufi03g04350.1 0.003 0.414 0.411 0.007 1<br>Orufi05g22410.2 0.547 0.997 0.45 0.031 0.969	Olong_020510-R2 0.523 0.991 0.468 0.291 0.873<br>Olong_025165-R1 0.003 0.414 0.411 0.007 1	Opunc12g01920.1 0.526 0.973 0.447 0.663 0.947	Obrac03g25020.5 0.003 0.535 0.532 0.439 0.972	Lperr01g29830.2 0.532 0.991 0.459 0.203 0.858<br>Lperr03g28250.1 0.003 0.414 0.411 0.007 1	AT1G52740.1 0.003 0.414 0.411 0.007 1<br>AT4G14640.1 0.544 0.979 0.435 0.033 0.987
Oglab03g35760.1	1<br>Oglab03g35760.1 0.001 1 0.999 0.001 1	0	0	0	0	0	0	0	0	0
	0<br>Oglab09g02410.1 0.47 0.957 0.487 0.443 0.943	1<br>Obart01g02390.1 0.506 0.997 0.491 0.153 0.308<br>Obart07g05930.1 0.001 0.483 0.482 0.168 0.985	1<br>Osjap01g02750.1 0.506 0.983 0.477 0.5 0.973<br>Osjap07g06820.1 0.001 0.483 0.482 0.003 0.982	1<br>Osind01g02910.1 0.506 0.997 0.491 0.232 0.458<br>Osind03g41900.1 0.001 0.481 0.48 0.003 0.977	1<br>Orufi01g02470.1 0.506 0.997 0.491 0.175 0.346<br>Orufi03g39370.2 0.001 0.481 0.48 0.003 0.977	1<br>Olong_013112-R3 0.502 0.957 0.455 0.202 1<br>Olong_024768-R1 0.001 0.481 0.48 0.005 0.942	1<br>Opunc01g02040.3 0.506 0.983 0.477 0.491 0.992<br>Opunc07g05660.1 0.001 0.483 0.482 0.16 0.948	1<br>Obrac01g01910.1 0.506 0.983 0.477 0.506 0.973<br>Obrac03g28110.2 0.001 0.483 0.482 0.003 0.986	1<br>Lperr01g02180.7 0.506 0.999 0.493 0.486 0.973<br>Lperr07g05150.1 0.001 0.483 0.482 0.134 0.953	1<br>AT1G66880.1 0.506 0.955 0.449 0.738 0.964<br>AT3G28630.1 0.001 0.483 0.482 0.099 0.982
Oglab03g38510.1	1<br>Oglab03g38510.1 0.002 1 0.998 0.002 1	0	0	0	0	0	0	0	0	0
	0<br>Oglab01g11460.1 0.647 1 0.353 0.543 0.951<br>Oglab12g00200.1 0.042 0.352 0.31 0.403 0.997	1<br>Obart03g40540.1 0.037 0.344 0.307 0.072 0.986<br>Obart03g40550.1 0.345 0.959 0.614 0.019 0.908	1<br>Osjap03g43930.1 0.037 0.344 0.307 0.072 0.986<br>Osjap03g43940.1 0.345 0.994 0.649 0.018 0.864	1<br>Osind03g44940.1 0.037 0.344 0.307 0.035 0.985<br>Osind03g44950.1 0.345 0.979 0.634 0.019 0.878	1<br>Orufi03g42280.1 0.037 0.344 0.307 0.072 0.986<br>Orufi03g42290.1 0.345 0.893 0.548 0.023 0.951	1<br>Olong_018753-R1 0.345 0.925 0.58 0.02 0.89<br>Olong_018754-R1 0.057 0.344 0.287 0.127 0.985	0<br>Opunc03g37710.1 0.083 0.344 0.261 0.15 0.984<br>Opunc07g07820.1 0.708 0.998 0.29 0.5 0.917	0<br>Obrac01g10510.1 0.616 0.997 0.381 0.488 0.935<br>Obrac03g30550.1 0.084 0.35 0.266 0.238 0.993	0<br>Lperr02g19630.1 0.679 1 0.321 0.436 0.924<br>Lperr03g34570.1 0.099 0.339 0.24 0.193 1	0
Oglab03g39920.1	1<br>Oglab03g39920.1 0.001 1 0.999 0.001 1	0	0	0	0	0	0	0	0	0
	1<br>Oglab02g12900.1 0.004 0.58 0.576 0.05 0.992<br>Oglab05g15580.1 0.616 0.996 0.38 0.208 0.946	1<br>Obart02g13170.1 0.005 0.58 0.575 0.13 0.993<br>Obart06g08490.1 0.619 1 0.381 0.002 1	1<br>Osjap02g14350.1 0.005 0.58 0.575 0.13 0.993<br>Osjap05g18940.1 0.616 0.996 0.38 0.208 0.946	1<br>Osind02g14610.1 0.005 0.58 0.575 0.13 0.993<br>Osind05g19660.1 0.616 0.996 0.38 0.208 0.946	1<br>Orufi01g35570.1 0.005 0.583 0.578 0.14 0.975<br>Orufi05g17840.1 0.616 0.996 0.38 0.208 0.946	1<br>Olong_004931-R1 0.621 1 0.379 0.184 0.927<br>Olong_023965-R1 0.005 0.58 0.575 0.13 0.993	1<br>Opunc02g11640.1 0.005 0.581 0.576 0.108 0.996<br>Opunc05g03040.1 0.621 1 0.379 0.181 0.929	1<br>Obrac02g12790.2 0.005 0.579 0.574 0.122 0.999<br>Obrac06g06500.1 0.591 0.997 0.406 0.026 0.978	1<br>Lperr01g27850.2 0.005 0.579 0.574 0.126 0.999<br>Lperr02g26310.1 0.581 0.997 0.416 0.038 0.979	1<br>AT1G65630.1 0.586 0.995 0.409 0.091 0.948<br>AT4G14990.1 0.005 0.576 0.571 0.142 0.995
Oglab04g03860.1	1<br>Oglab04g03860.1 0.004 1 0.996 0.004 1	1<br>Obart04g04460.1 0.004 0.996 0.992 0.005 0.96<br>Obart04g04460.2 0.004 0.885 0.881 0.006 0.982	1<br>Osjap04g05800.1 0.045 0.996 0.951 0.156 0.964	0	0	0	0	0	0	0
	1<br>Oglab05g07770.1 0.469 0.996 0.527 0.386 0.904<br>Oglab06g24390.1 0.008 0.383 0.375 0.275 0.736	0<br>Obart01g00430.1 0.008 0.383 0.375 0.278 0.737<br>Obart09g05770.4 0.691 0.996 0.305 0.445 0.956	1<br>Osjap01g00480.1 0.008 0.383 0.375 0.276 0.74<br>Osjap05g09580.1 0.469 0.996 0.527 0.385 0.903	1<br>Osind05g10500.1 0.469 0.996 0.527 0.387 0.903<br>Osind06g30600.1 0.008 0.383 0.375 0.275 0.736	1<br>Orufi01g00410.1 0.008 0.383 0.375 0.276 0.74<br>Orufi05g09280.1 0.469 0.996 0.527 0.387 0.903	0<br>Olong_011188-R1 0.56 0.996 0.436 0.056 0.935<br>Olong_021982-R1 0.008 0.28 0.272 0.455 1	1<br>Opunc01g00390.1 0.008 0.383 0.375 0.277 0.738<br>Opunc05g07720.1 0.469 0.996 0.527 0.392 0.903	1<br>Obrac05g05910.1 0.469 0.979 0.51 0.396 0.908<br>Obrac06g19120.5 0.008 0.383 0.375 0.209 0.755	0<br>Lperr08g08820.1 0.008 0.383 0.375 0.327 0.744	0<br>AT4G39220.1 0.008 0.383 0.375 0.277 0.743
Oglab04g27220.1	1<br>Oglab04g27220.1 0.001 1 0.999 0.001 1	0	0	0	0	0	0	0	0	0
	1<br>Oglab02g23520.1 0.544 0.999 0.455 0.028 0.993<br>Oglab06g09240.2 0.001 0.401 0.4 0.072 0.816	1<br>Obart02g04210.1 0.532 0.999 0.467 0.065 0.998<br>Obart04g29190.4 0.001 0.519 0.518 0.001 0.994	1<br>Osjap02g28270.1 0.544 0.999 0.455 0.034 0.993<br>Osjap04g33340.1 0.001 0.519 0.518 0.14 0.994	1<br>Osind02g04720.1 0.532 0.998 0.466 0.017 0.997<br>Osind04g32550.1 0.001 0.519 0.518 0.001 0.994	1<br>Orufi02g26250.1 0.544 0.999 0.455 0.034 0.993<br>Orufi04g30900.1 0.001 0.485 0.484 0.14 0.957	1<br>Olong_002807-R4 0.001 0.485 0.484 0.001 0.953<br>Olong_024919-R1 0.558 0.998 0.44 0.069 0.989	1<br>Opunc02g00670.1 0.545 1 0.455 0.043 0.999<br>Opunc04g26710.1 0.001 0.485 0.484 0.001 0.948	1<br>Obrac04g20580.2 0.001 0.509 0.508 0.017 0.994<br>Obrac06g17870.1 0.547 0.998 0.451 0.051 0.992	1<br>Lperr04g24690.2 0.001 0.489 0.488 0.115 0.915<br>Lperr07g00420.1 0.527 0.998 0.471 0.021 1	0<br>AT2G02220.1 0.551 0.998 0.447 0.047 1
Oglab05g02190.1	1<br>Oglab05g02190.1 0.001 1 0.999 0.001 1	1<br>Obart05g02340.1 0.001 1 0.999 0.001 1	1<br>Osjap05g02530.1 0.079 1 0.921 0.002 1	1<br>Osind05g02510.1 0.079 0.998 0.919 0.002 0.987	0	0	0	0	0	0
	0<br>Oglab03g00660.1 0.608 0.994 0.386 0.321 0.996<br>Oglab05g07310.1 0.082 0.433 0.351 0.013 0.935	1<br>Obart06g15630.1 0.079 0.433 0.354 0.004 0.955<br>Obart11g00590.1 0.501 0.977 0.476 0.146 0.917	1<br>Osjap11g00710.1 0.553 0.997 0.444 0.034 0.911<br>Osjap12g14320.1 0.084 0.441 0.357 0.575 0.91	1<br>Osind07g26860.1 0.487 0.997 0.51 0.035 1<br>Osind12g11780.1 0.03 0.433 0.403 0.012 0.947	1<br>Orufi05g02460.2 0.451 1 0.549 0.003 0.982<br>Orufi07g25010.2 0.082 0.427 0.345 0.007 0.691	1<br>Olong_016162-R1 0.079 0.433 0.354 0.004 0.954<br>Olong_026687-R1 0.451 1 0.549 0.09 0.983	1<br>Opunc05g02220.2 0.451 1 0.549 0.003 0.981<br>Opunc07g20770.2 0.082 0.432 0.35 0.009 0.832	1<br>Obrac05g01840.1 0.451 0.998 0.547 0.169 1<br>Obrac06g15870.1 0.082 0.434 0.352 0.015 0.84	1<br>Lperr05g02100.1 0.451 0.998 0.547 0.017 0.978<br>Lperr11g18770.1 0.027 0.304 0.277 0.358 0.699	0<br>AT5G15380.1 0.468 0.992 0.524 0.064 0.987

Oglab05g03490.1	1<br>Oglab05g03490.1 0.001 1 0.999 0.001 1<br>Oglab05g03490.2 0.001 1 0.999 0.001 1	1<br>Obart05g03830.1 0.001 1 0.999 0.001 1<br>Obart05g03830.2 0.001 1 0.999 0.001 1	1<br>Osjap05g04120.1 0.001 1 0.999 0.001 1<br>Osjap05g04120.2 0.001 1 0.999 0.001 1	1<br>Osind05g04070.1 0.008 1 0.992 0.001 1<br>Osind05g04070.2 0.008 1 0.992 0.001 1	1<br>Orufi05g03950.1 0.001 1 0.999 0.001 1<br>Orufi05g03950.2 0.001 1 0.999 0.001 1	1<br>Olong_016739-R1 0.074 1 0.926 0.001 1	1<br>Opunc05g03540.1 0.001 1 0.999 0.001 1<br>Opunc05g03540.2 0.001 1 0.999 0.001 1	1<br>Obrac05g02930.1 0.074 1 0.926 0.001 1	0	0
	1<br>Oglab01g19490.1 0.09 0.723 0.633 0.066 0.996<br>Oglab03g14060.1 0.79 1 0.21 0.03 1	1<br>Obart01g23310.1 0.09 0.723 0.633 0.066 0.996<br>Obart03g14050.1 0.79 1 0.21 0.03 1	1<br>Osjap01g27410.1 0.09 0.723 0.633 0.066 0.996<br>Osjap03g15140.5 0.743 1 0.257 0.061 1	1<br>Osind01g28630.1 0.09 0.723 0.633 0.066 0.996<br>Osind03g15230.1 0.79 1 0.21 0.03 1	1<br>Orufi01g26130.1 0.09 0.723 0.633 0.066 0.996<br>Orufi03g14720.1 0.79 1 0.21 0.03 1	1<br>Olong_003347-R2 0.09 0.723 0.633 0.047 0.996<br>Olong_009943-R1 0.79 1 0.21 0.03 1	1<br>Opunc01g23460.1 0.09 0.723 0.633 0.064 0.996<br>Opunc03g13590.1 0.79 1 0.21 0.002 0.545	1<br>Obrac01g17590.1 0.09 0.723 0.633 0.067 0.996<br>Obrac03g11330.1 0.79 1 0.21 0.03 1	0<br>Lperr05g03460.1 0.001 0.721 0.72 0.001 0.993	1<br>AT2G47580.1 0.775 1 0.225 0.008 1<br>AT3G16630.1 0.003 0.721 0.718 0.031 0.992
Oglab05g03490.2	1<br>Oglab05g03490.1 0.001 1 0.999 0.001 1<br>Oglab05g03490.2 0.001 1 0.999 0.001 1	1<br>Obart05g03830.1 0.001 1 0.999 0.001 1<br>Obart05g03830.2 0.001 1 0.999 0.001 1	1<br>Osjap05g04120.1 0.001 1 0.999 0.001 1<br>Osjap05g04120.2 0.001 1 0.999 0.001 1	1<br>Osind05g04070.1 0.008 1 0.992 0.001 1<br>Osind05g04070.2 0.008 1 0.992 0.001 1	1<br>Orufi05g03950.1 0.001 1 0.999 0.001 1<br>Orufi05g03950.2 0.001 1 0.999 0.001 1	1<br>Olong_016739-R1 0.073 1 0.927 0.001 1	1<br>Opunc05g03540.1 0.001 1 0.999 0.001 1<br>Opunc05g03540.2 0.001 1 0.999 0.001 1	1<br>Obrac05g02930.1 0.073 1 0.927 0.001 1	0	0
	1<br>Oglab01g19490.1 0.089 0.726 0.637 0.066 0.996<br>Oglab03g14060.1 0.792 1 0.208 0.03 1	1<br>Obart01g23310.1 0.089 0.726 0.637 0.066 0.996<br>Obart03g14050.1 0.792 1 0.208 0.03 1	1<br>Osjap01g27410.1 0.089 0.726 0.637 0.066 0.996<br>Osjap03g15140.5 0.747 1 0.253 0.061 1	1<br>Osind01g28630.1 0.089 0.726 0.637 0.066 0.996<br>Osind03g15230.1 0.792 1 0.208 0.03 1	1<br>Orufi01g26130.1 0.089 0.726 0.637 0.066 0.996<br>Orufi03g14720.1 0.792 1 0.208 0.03 1	1<br>Olong_003347-R2 0.089 0.726 0.637 0.047 0.996<br>Olong_009943-R1 0.792 1 0.208 0.03 1	1<br>Opunc01g23460.1 0.089 0.726 0.637 0.064 0.996<br>Opunc03g13590.1 0.792 1 0.208 0.002 0.545	1<br>Obrac01g17590.1 0.089 0.726 0.637 0.067 0.996<br>Obrac03g11330.1 0.792 1 0.208 0.03 1	0<br>Lperr05g03460.1 0.001 0.725 0.724 0.001 0.993	1<br>AT2G47580.1 0.778 1 0.222 0.008 1<br>AT3G16630.1 0.003 0.725 0.722 0.031 0.992
Oglab05g04140.1	1<br>Oglab05g04140.1 0.002 1 0.998 0.002 1	1<br>Obart05g04470.1 0.002 1 0.998 0.002 1	0	0	0	0	0	0	0	0
	1<br>Oglab05g02840.1 0.362 0.977 0.615 0.395 0.582<br>Oglab06g15820.1 0.014 0.359 0.345 0.033 0.973	1<br>Obart05g03110.1 0.362 0.977 0.615 0.395 0.582<br>Obart06g05940.1 0.014 0.35 0.336 0.048 0.458	1<br>Osjap05g04830.1 0.002 0.35 0.348 0.006 0.86<br>Osjap08g03110.1 0.359 0.993 0.634 0.031 0.944	0<br>Osind05g03400.1 0.274 0.945 0.671 0.792 0.975	1<br>Orufi05g04690.1 0.002 0.35 0.348 0.006 0.86<br>Orufi05g04700.1 0.35 1 0.65 0.212 1	1<br>Olong_002683-R1 0.359 0.993 0.634 0.031 0.945<br>Olong_015421-R1 0.002 0.35 0.348 0.01 1	1<br>Opunc05g03750.1 0.362 0.984 0.622 0.659 0.972<br>Opunc05g04100.1 0.002 0.35 0.348 0.006 0.878	0<br>Obrac05g03390.1 0.346 0.986 0.64 0.441 0.983	1<br>Lperr03g03510.1 0.023 0.35 0.327 0.073 0.593<br>Lperr05g03760.1 0.5 0.984 0.484 0.279 0.955	0<br>AT3G16720.1 0.002 0.35 0.348 0.059 0.523
Oglab05g04450.2	1<br>Oglab05g04450.1 0.001 1 0.999 0.001 1<br>Oglab05g04450.2 0.001 1 0.999 0.001 1	1<br>Obart05g05010.1 0.001 1 0.999 0.01 1<br>Obart05g05010.2 0.001 1 0.999 0.011 1<br>Obart05g05010.3 0.001 1 0.999 0.011 1	0	1<br>Osind05g05280.1 0.181 0.99 0.809 0.001 0.981	0	0	0	0	0	0
	1<br>Oglab05g04600.1 0.677 0.999 0.322 0.044 0.997<br>Oglab05g08330.1 0.001 0.628 0.627 0.001 0.997	1<br>Obart05g09170.2 0.001 0.628 0.627 0.001 0.997<br>Obart07g17280.1 0.667 0.995 0.328 0.002 0.977	1<br>Osjap05g10230.1 0.001 0.629 0.628 0.001 0.999<br>Osjap07g20060.1 0.666 0.999 0.333 0.002 0.991	1<br>Osind05g11170.1 0.665 0.999 0.334 0.02 0.998<br>Osind05g11190.1 0.001 0.629 0.628 0.001 0.999	1<br>Orufi05g05240.1 0.674 0.998 0.324 0.034 0.995<br>Orufi05g09830.2 0.001 0.629 0.628 0.001 0.999	1<br>Olong_008766-R1 0.666 0.998 0.332 0.005 0.995<br>Olong_015082-R1 0.001 0.625 0.624 0.001 0.993	0<br>Opunc05g04580.1 0.001 0.793 0.792 0.001 1	1<br>Obrac05g03760.1 0.664 0.998 0.334 0.003 0.995<br>Obrac11g11360.1 0.001 0.629 0.628 0.001 0.999	1<br>Lperr05g02380.1 0.67 1 0.33 0.003 1<br>Lperr11g15610.1 0.001 0.629 0.628 0.001 0.997	0<br>AT2G27090.1 0.001 0.622 0.621 0.001 0.985
Oglab05g07040.1	1<br>Oglab05g07040.1 0.004 1 0.996 0.004 1	0	0	0	1<br>Orufi05g08440.1 0.004 0.917 0.913 0.003 0.663	0	0	0	0	0
	1<br>Oglab01g16080.1 0.737 0.974 0.237 0.041 0.684<br>Oglab05g07020.1 0.004 0.718 0.714 0.004 0.838	1<br>Obart05g07850.1 0.004 0.718 0.714 0.004 0.87<br>Obart05g07870.1 0.756 1 0.244 0.015 1	1<br>Osjap05g08680.1 0.004 0.718 0.714 0.004 0.964<br>Osjap08g02020.1 0.737 0.974 0.237 0.02 0.34	1<br>Osind05g08590.1 0.004 0.718 0.714 0.004 0.964<br>Osind05g08610.1 0.756 1 0.244 0.015 1	0<br>Orufi01g21290.1 0.737 0.974 0.237 0.041 0.684<br>Orufi05g08430.1 0.086 0.62 0.534 0.156 0.574	1<br>Olong_012066-R1 0.004 0.711 0.707 0.004 0.964<br>Olong_015117-R1 0.759 0.974 0.215 0.009 0.523	1<br>Opunc02g01060.1 0.748 0.974 0.226 0.029 0.248<br>Opunc05g07190.1 0.004 0.711 0.707 0.003 0.978	0<br>Obrac07g06840.1 0.053 0.688 0.635 0.03 0.911	1<br>Lperr07g09890.1 0.03 0.643 0.613 0.006 0.891<br>Lperr08g02000.1 0.737 0.974 0.237 0.028 0.469	0<br>AT4G36020.1 0.752 0.977 0.225 0.043 0.244
Oglab05g09590.1	1<br>Oglab05g09590.1 0.005 1 0.995 0.005 1<br>Oglab05g09590.2 0.005 1 0.995 0.005 1	0	1<br>Osjap05g11740.1 0.005 1 0.995 0.388 1<br>Osjap05g11740.2 0.005 1 0.995 0.432 1	1<br>Osind05g12550.1 0.005 1 0.995 0.005 1	1<br>Orufi05g11190.1 0.005 1 0.995 0.005 1	0	0	0	0	0
	0	1	0	1	1	1	1	0	1	1

	Oglab11g01510.4 0.507 0.886 0.379 0.681 0.789	Obart09g04270.2 0.537 0.92 0.383 0.847 0.93<br>Obart11g16680.1 0.065 0.532 0.467 0.051 0.311	Osjap11g19150.1 0.005 0.532 0.527 0.37 0.489	Osind08g13680.1 0.537 0.886 0.349 0.748 0.825<br>Osind11g14080.1 0.005 0.532 0.527 0.003 0.309	Orufi08g12780.1 0.537 0.886 0.349 0.769 0.839<br>Orufi11g17770.2 0.005 0.532 0.527 0.003 0.356	Olong_007188-R1 0.537 0.886 0.349 0.744 0.822<br>Olong_026954-R1 0.045 0.532 0.487 0.016 0.497	Opunc08g10590.1 0.537 0.886 0.349 0.768 0.839<br>Opunc11g13660.1 0.065 0.532 0.467 0.051 0.311	Obrac12g01300.1 0.507 0.886 0.379 0.428 0.614	Lperr08g09600.2 0.537 0.886 0.349 0.759 0.833<br>Lperr11g13130.1 0.005 0.532 0.527 0.003 0.303	AT3G47630.2 0.005 0.532 0.527 0.003 0.298<br>AT4G19710.2 0.537 0.886 0.349 0.767 0.838
Oglab05g09590.2	1<br>Oglab05g09590.1 0.005 1 0.995 0.005 1<br>Oglab05g09590.2 0.005 1 0.995 0.005 1	0	1<br>Osjap05g11740.1 0.005 1 0.995 0.388 1<br>Osjap05g11740.2 0.005 1 0.995 0.432 1	1<br>Osind05g12550.1 0.005 1 0.995 0.005 1	1<br>Orufi05g11190.1 0.005 1 0.995 0.005 1	0	0	0	0	0
	0<br>Oglab11g01510.4 0.487 0.881 0.394 0.681 0.789	1<br>Obart09g04270.2 0.518 0.917 0.399 0.847 0.93<br>Obart11g16680.1 0.067 0.513 0.446 0.051 0.311	0<br>Osjap11g19150.1 0.005 0.513 0.508 0.37 0.489	1<br>Osind08g13680.1 0.518 0.881 0.363 0.748 0.825<br>Osind11g14080.1 0.005 0.513 0.508 0.003 0.309	1<br>Orufi08g12780.1 0.518 0.881 0.363 0.769 0.839<br>Orufi11g17770.2 0.005 0.513 0.508 0.003 0.356	1<br>Olong_007188-R1 0.518 0.881 0.363 0.744 0.822<br>Olong_026954-R1 0.047 0.513 0.466 0.016 0.497	1<br>Opunc08g10590.1 0.518 0.881 0.363 0.768 0.839<br>Opunc11g13660.1 0.067 0.513 0.446 0.051 0.311	0<br>Obrac12g01300.1 0.487 0.881 0.394 0.428 0.614	1<br>Lperr08g09600.2 0.518 0.881 0.363 0.759 0.833<br>Lperr11g13130.1 0.005 0.513 0.508 0.003 0.303	1<br>AT3G47630.2 0.005 0.513 0.508 0.003 0.298<br>AT4G19710.2 0.518 0.881 0.363 0.767 0.838
Oglab05g14020.2	1<br>Oglab05g14020.2 0.002 1 0.998 0.002 1	0	0	0	0	0	0	0	0	0
	0<br>Oglab05g14020.1 0.002 0.672 0.67 0.003 1	1<br>Obart01g44290.1 0.676 1 0.324 0.492 1<br>Obart05g14930.1 0.002 0.672 0.67 0.003 1	1<br>Osjap01g50270.1 0.676 1 0.324 0.553 1<br>Osjap05g16940.3 0.002 0.672 0.67 0.003 1	1<br>Osind01g51840.1 0.676 1 0.324 0.553 1<br>Osind05g17720.1 0.002 0.672 0.67 0.003 1	1<br>Orufi01g47720.1 0.676 1 0.324 0.492 1<br>Orufi05g16000.2 0.002 0.672 0.67 0.003 1	0<br>Olong_011178-R1 0.002 0.672 0.67 0.003 1	1<br>Opunc01g43220.1 0.676 1 0.324 0.085 1<br>Opunc05g13020.1 0.002 0.672 0.67 0.003 1	0<br>Obrac05g09800.2 0.124 0.672 0.548 0.014 1	1<br>Lperr01g38720.1 0.693 1 0.307 0.067 1<br>Lperr05g11830.1 0.002 0.672 0.67 0.003 1	1<br>AT2G41200.1 0.767 1 0.233 0.261 1<br>AT4G01220.1 0.006 0.672 0.666 0.019 0.997
Oglab05g20580.1	1<br>Oglab05g20580.1 0.002 1 0.998 0.002 1	0	0	0	0	0	0	0	0	0
	0	1<br>Obart05g21800.1 0.284 1 0.716 0.003 1<br>Obart05g21810.1 0.002 0.209 0.207 0.01 0.98	1<br>Osjap05g24470.1 0.284 1 0.716 0.003 1<br>Osjap05g24480.1 0.002 0.209 0.207 0.01 0.98	1<br>Osind05g24960.1 0.284 1 0.716 0.003 1<br>Osind05g24970.1 0.002 0.209 0.207 0.01 0.98	1<br>Orufi05g23060.1 0.284 1 0.716 0.003 1<br>Orufi05g23070.1 0.002 0.209 0.207 0.01 0.98	0<br>Olong_006515-R1 0.002 0.209 0.207 0.012 0.976<br>Olong_017564-R1 0.705 0.978 0.273 0.007 0.936	1<br>Opunc05g19390.1 0.284 0.996 0.712 0.003 0.997<br>Opunc05g19400.1 0.002 0.209 0.207 0.01 0.98	0<br>Obrac05g14860.1 0.486 0.998 0.512 0.012 1<br>Obrac05g14880.1 0.002 0.209 0.207 0.01 0.979	1<br>Lperr05g17700.1 0.284 0.998 0.714 0.003 1<br>Lperr05g17710.1 0.002 0.209 0.207 0.01 0.981	0<br>AT3G20680.1 0.43 0.989 0.559 0.225 1<br>AT3G59840.1 0.002 0.209 0.207 0.01 0.979
Oglab05g23160.1	1<br>Oglab05g23160.1 0.001 1 0.999 0.001 1	1<br>Obart05g25070.1 0.001 1 0.999 0.001 1	1<br>Osjap05g28230.1 0.001 1 0.999 0.001 1	1<br>Osind05g28520.1 0.001 1 0.999 0.001 1	1<br>Orufi05g26870.1 0.001 1 0.999 0.001 1	0	1<br>Opunc05g22640.1 0.001 1 0.999 0.001 1	0	1<br>Lperr01g22990.1 0.001 1 0.999 0.027 1	0
	1<br>Oglab01g22740.1 0.028 0.451 0.423 0.061 0.995<br>Oglab01g22750.1 0.462 0.999 0.537 0.009 0.999	1<br>Obart01g26870.1 0.006 0.451 0.445 0.117 0.995<br>Obart01g26880.1 0.462 0.999 0.537 0.009 0.999	1<br>Osjap01g31140.1 0.007 0.451 0.444 0.093 0.995<br>Osjap01g31150.1 0.462 0.999 0.537 0.009 0.999	1<br>Osind01g32600.1 0.001 0.451 0.45 0.002 0.995<br>Osind01g32610.1 0.462 0.999 0.537 0.009 0.999	1<br>Orufi01g29880.1 0.001 0.451 0.45 0.075 0.995<br>Orufi01g29890.1 0.462 0.999 0.537 0.009 0.999	1<br>Olong_000367-R1 0.105 0.451 0.346 0.002 0.993<br>Olong_000368-R1 0.456 0.999 0.543 0.108 0.999	1<br>Opunc01g26820.1 0.049 0.451 0.402 0.002 0.994<br>Opunc01g26830.1 0.462 1 0.538 0.009 1	1<br>Obrac01g20400.1 0.048 0.451 0.403 0.006 0.994<br>Obrac05g17560.2 0.451 1 0.549 0.164 1	1<br>Lperr05g20850.2 0.467 1 0.533 0.002 1<br>Lperr05g20860.1 0.001 0.451 0.45 0.002 0.995	1<br>AT5G08690.1 0.012 0.451 0.439 0.034 0.995<br>AT5G51600.1 0.462 0.999 0.537 0.008 0.996
Oglab06g00840.1	1<br>Oglab06g00840.1 0.001 1 0.999 0.001 1	1<br>Obart06g00880.1 0.001 1 0.999 0.001 1	1<br>Osjap06g00940.1 0.04 1 0.96 0.085 1	0	1<br>Orufi06g00860.1 0.067 1 0.933 0.021 1	0	0	0	0	0
	1<br>Oglab06g00730.1 0.526 0.99 0.464 0.257 0.968<br>Oglab07g19660.1 0.033 0.458 0.425 0.142 0.938	1<br>Obart06g00690.1 0.529 1 0.471 0.099 1<br>Obart09g03180.1 0.021 0.458 0.437 0.145 1	1<br>Osjap06g00950.1 0.529 0.992 0.463 0.132 0.99<br>Osjap08g13790.1 0.051 0.458 0.407 0.005 1	1<br>Osind06g00910.1 0.526 1 0.474 0.068 1<br>Osind08g15520.1 0.051 0.458 0.407 0.005 1	1<br>Orufi06g00690.1 0.529 1 0.471 0.099 1<br>Orufi06g00860.2 0.001 0.445 0.444 0.005 0.98	0<br>Olong_023831-R1 0.053 0.583 0.53 0.001 0.373	1<br>Opunc06g00800.1 0.457 1 0.543 0.025 1<br>Opunc06g00830.1 0.05 0.457 0.407 0.006 1	1<br>Obrac06g00660.1 0.526 1 0.474 0.034 0.999<br>Obrac07g14790.1 0.058 0.503 0.445 0.093 0.948	1<br>Lperr01g40030.1 0.055 0.457 0.402 0.013 0.994<br>Lperr06g00740.1 0.531 0.999 0.468 0.078 0.997	0<br>AT4G34470.1 0.063 0.457 0.394 0.013 1
Oglab06g02870.1	1<br>Oglab06g02870.1 0.001 1 0.999 0.001 1	1<br>Obart06g02710.1 0.001 1 0.999 0.03 1	1<br>Osjap06g03080.1 0.001 1 0.999 0.001 1	0	1<br>Orufi06g02670.1 0.045 0.964 0.919 0.032 0.91	1<br>Olong_013693-R1 0.045 1 0.955 0.069 1	0	0	0	0
	1<br>Oglab01g27580.1 0.037 0.715 0.678 0.03 0.949<br>Oglab04g28300.1 0.767 0.968 0.201 0.466 0.888	1<br>Obart01g06310.1 0.037 0.756 0.719 0.066 0.997<br>Obart04g30240.1 0.767 0.968 0.201 0.466 0.888	1<br>Osjap01g37620.1 0.001 0.756 0.755 0.033 0.998<br>Osjap04g34350.1 0.767 0.968 0.201 0.466 0.888	1<br>Osind03g45110.1 0.04 0.75 0.71 0.141 0.984<br>Osind04g33620.1 0.767 0.968 0.201 0.438 0.875	0<br>Orufi01g36040.3 0.037 0.756 0.719 0.03 0.998	1<br>Olong_001861-R1 0.767 0.968 0.201 0.381 0.87<br>Olong_005208-R1 0.014 0.715 0.701 0.141 0.969	1<br>Opunc03g00730.1 0.001 0.756 0.755 0.004 1<br>Opunc04g27750.1 0.767 0.968 0.201 0.426 0.879	1<br>Obrac04g05640.6 0.014 0.705 0.691 0.262 0.995<br>Obrac04g21390.1 0.767 0.968 0.201 0.381 0.87	1<br>Lperr10g07060.1 0.04 0.722 0.682 0.115 0.964<br>Lperr12g00960.1 0.767 0.968 0.201 0.389 0.871	0<br>AT2G37990.1 0.767 0.968 0.201 0.425 0.893

Oglab06g21340.1	1<br>Oglab06g21340.1 0.002 1 0.998 0.002 1	0	0	0	0	0	0	0	0	0
	1<br>Oglab02g06010.1 0.01 0.597 0.587 0.147 0.863<br>Oglab05g08960.1 0.684 0.986 0.302 0.443 0.897	1<br>Obart05g02250.1 0.692 0.966 0.274 0.173 0.46<br>Obart06g23450.1 0.002 0.689 0.687 0.003 0.969	1<br>Osjap05g02420.1 0.692 0.966 0.274 0.167 0.443<br>Osjap06g27500.1 0.002 0.689 0.687 0.002 0.971	1<br>Osind05g02400.1 0.692 0.966 0.274 0.17 0.451<br>Osind06g26900.1 0.009 0.689 0.68 0.162 0.973	1<br>Orufi05g02370.1 0.692 0.966 0.274 0.167 0.443<br>Orufi06g25120.1 0.009 0.689 0.68 0.162 0.973	1<br>Olong_002211-R1 0.009 0.689 0.68 0.126 0.972<br>Olong_004663-R2 0.706 0.966 0.26 0.116 0.242	1<br>Opunc06g21170.1 0.009 0.689 0.68 0.236 0.976<br>Opunc08g00730.1 0.701 0.968 0.267 0.316 0.987	1<br>Obrac05g06640.1 0.72 0.986 0.266 0.346 0.873<br>Obrac08g07630.1 0.005 0.694 0.689 0.187 0.935	1<br>Lperr05g02030.1 0.692 0.966 0.274 0.158 0.439<br>Lperr06g19460.1 0.005 0.689 0.684 0.199 0.975	0<br>AT2G18940.1 0.73 0.986 0.256 0.749 0.901
Oglab06g22820.1	1<br>Oglab06g22820.1 0.005 1 0.995 0.005 1	1<br>Obart06g25430.1 0.005 1 0.995 0.004 1	0	0	0	0	0	0	0	0
	1<br>Oglab06g20540.1 0.005 0.675 0.67 0.625 0.997<br>Oglab09g12320.1 0.72 0.98 0.26 0.658 0.994	1<br>Obart06g22650.1 0.005 0.465 0.46 0.574 0.984<br>Obart06g25420.1 0.625 0.98 0.355 0.403 0.852	1<br>Osjap06g26550.1 0.005 0.465 0.46 0.698 0.988<br>Osjap06g29670.1 0.625 0.98 0.355 0.469 0.992	1<br>Osind06g29020.1 0.625 0.98 0.355 0.469 0.992<br>Osind06g29050.1 0.015 0.54 0.525 0.655 0.915	1<br>Orufi06g24280.1 0.005 0.525 0.52 0.654 0.959<br>Orufi06g27300.1 0.535 0.985 0.45 0.29 0.942	0<br>Olong_024432-R1 0.625 0.98 0.355 0.561 0.994	1<br>Opunc02g22800.1 0.015 0.525 0.51 0.695 0.966<br>Opunc06g23120.1 0.625 0.98 0.355 0.691 0.995	0<br>Obrac09g08010.1 0.72 0.98 0.26 0.586 0.992	0<br>Lperr09g09890.1 0.585 0.98 0.395 0.53 0.994	0<br>AT4G30380.1 0.73 0.97 0.24 0.585 0.984
Oglab06g24190.1	1<br>Oglab06g24190.1 0.001 1 0.999 0.001 1<br>Oglab06g24190.2 0.001 0.995 0.994 0.001 0.991	1<br>Obart06g26750.1 0.001 0.995 0.994 0.008 1	1<br>Osjap06g31210.1 0.001 0.995 0.994 0.008 1	1<br>Osind06g30430.1 0.001 0.995 0.994 0.008 1	1<br>Orufi06g28720.1 0.001 0.995 0.994 0.008 1	1<br>Olong_013658-R1 0.001 0.995 0.994 0.001 1	1<br>Opunc06g24460.1 0.001 0.995 0.994 0.008 1	0	1<br>Lperr06g22350.1 0.001 0.995 0.994 0.033 1	0
	1<br>Oglab02g02970.1 0.694 0.993 0.299 0.006 0.93<br>Oglab12g14250.1 0.001 0.62 0.619 0.001 0.743	1<br>Obart02g03050.1 0.694 0.993 0.299 0.006 0.93<br>Obart12g15260.1 0.001 0.602 0.601 0.001 0.686	1<br>Osjap02g03010.1 0.694 0.993 0.299 0.006 0.928<br>Osjap12g19610.1 0.001 0.62 0.619 0.001 0.675	1<br>Osind02g03310.1 0.694 0.993 0.299 0.006 0.928<br>Osind07g26390.1 0.001 0.59 0.589 0.074 0.935	1<br>Orufi02g02960.1 0.694 0.993 0.299 0.006 0.928<br>Orufi12g16970.1 0.001 0.62 0.619 0.001 0.674	1<br>Olong_017635-R1 0.694 0.993 0.299 0.006 0.93<br>Olong_024721-R1 0.01 0.608 0.598 0.05 0.932	1<br>Opunc02g02350.2 0.715 0.993 0.278 0.248 0.944<br>Opunc03g37840.1 0.001 0.622 0.621 0.047 0.706	1<br>Obrac02g02360.1 0.694 0.994 0.3 0.006 0.933<br>Obrac09g01040.1 0.001 0.591 0.59 0.026 0.872	1<br>Lperr02g02740.1 0.699 0.995 0.296 0.006 0.96<br>Lperr12g12430.1 0.001 0.602 0.601 0.001 0.854	1<br>AT3G10250.1 0.701 0.998 0.297 0.006 0.948<br>AT4G32430.1 0.005 0.61 0.605 0.051 0.976
Oglab06g24190.2	1<br>Oglab06g24190.1 0.001 0.991 0.99 0.001 0.995<br>Oglab06g24190.2 0.001 1 0.999 0.001 1	1<br>Obart06g26750.1 0.001 0.991 0.99 0.008 1	1<br>Osjap06g31210.1 0.001 0.991 0.99 0.008 1	1<br>Osind06g30430.1 0.001 0.991 0.99 0.008 1	1<br>Orufi06g28720.1 0.001 0.991 0.99 0.008 1	1<br>Olong_013658-R1 0.001 0.991 0.99 0.001 1	1<br>Opunc06g24460.1 0.001 0.991 0.99 0.008 1	0	1<br>Lperr06g22350.1 0.001 0.991 0.99 0.033 1	0
	1<br>Oglab02g02970.1 0.691 0.997 0.306 0.006 0.96<br>Oglab12g14250.1 0.001 0.617 0.616 0.001 0.743	1<br>Obart02g03050.1 0.691 0.997 0.306 0.006 0.96<br>Obart12g15260.1 0.001 0.617 0.616 0.001 0.686	1<br>Osjap02g03010.1 0.691 0.997 0.306 0.006 0.959<br>Osjap12g19610.1 0.001 0.617 0.616 0.001 0.675	1<br>Osind02g03310.1 0.691 0.997 0.306 0.006 0.959<br>Osind07g26390.1 0.001 0.587 0.586 0.074 0.935	1<br>Orufi02g02960.1 0.691 0.997 0.306 0.006 0.959<br>Orufi09g02510.1 0.001 0.589 0.588 0.014 0.762	1<br>Olong_017635-R1 0.691 0.997 0.306 0.006 0.96<br>Olong_024721-R1 0.01 0.605 0.595 0.05 0.932	1<br>Opunc02g02350.2 0.712 0.997 0.285 0.248 0.968<br>Opunc03g37840.1 0.001 0.619 0.618 0.047 0.706	1<br>Obrac02g02360.1 0.691 0.997 0.306 0.006 0.961<br>Obrac09g01040.1 0.001 0.588 0.587 0.026 0.872	1<br>Lperr02g02740.1 0.696 0.991 0.295 0.006 0.96<br>Lperr03g02630.1 0.001 0.584 0.583 0.105 0.84	1<br>AT3G10250.1 0.698 0.992 0.294 0.006 0.941<br>AT4G32430.1 0.005 0.607 0.602 0.051 0.976
Oglab06g25490.1	1<br>Oglab06g25490.1 0.001 1 0.999 0.001 1<br>Oglab09g15660.1 0.024 0.999 0.975 0.154 0.95	1<br>Obart06g28060.1 0.001 1 0.999 0.001 1<br>Obart09g17460.1 0.024 1 0.976 0.076 0.969<br>Obart09g17460.2 0.024 1 0.976 0.076 0.969	1<br>Osjap09g17800.1 0.024 1 0.976 0.113 0.974<br>Osjap09g17800.2 0.024 1 0.976 0.114 0.974<br>Osjap09g17800.3 0.024 0.997 0.973 0.187 0.947	1<br>Osind06g31620.1 0.001 1 0.999 0.001 1	1<br>Orufi06g29990.1 0.001 1 0.999 0.001 1<br>Orufi09g18790.1 0.024 1 0.976 0.107 0.97<br>Orufi09g18790.2 0.024 1 0.976 0.107 0.97<br>Orufi09g18790.3 0.024 1 0.976 0.108 0.969<br>Orufi09g18790.4 0.024 1 0.976 0.108 0.969<br>Orufi09g18790.5 0.024 0.976 0.952 0.17 0.937<br>Orufi09g18790.6 0.024 0.996 0.972 0.172 0.937	1<br>Olong_016700-R1 0.001 1 0.999 0.001 1	0	0	1<br>Lperr09g13380.1 0.084 0.982 0.898 0.166 0.951	0
	1<br>Oglab02g01250.1 0.066 0.406 0.34 0.221 0.93<br>Oglab10g06880.1 0.427 1 0.573 0.099 1	1<br>Obart04g21770.1 0.07 0.417 0.347 0.232 0.933<br>Obart10g07310.1 0.427 1 0.573 0.099 1	1<br>Osjap06g32830.1 0.427 1 0.573 0.085 1<br>Osjap06g32840.1 0.001 0.407 0.406 0.002 0.952	1<br>Osind04g24640.1 0.07 0.417 0.347 0.233 0.933<br>Osind06g30840.1 0.485 0.999 0.514 0.109 1	0<br>Orufi06g29150.4 0.485 0.999 0.514 0.122 1	1<br>Olong_000286-R1 0.066 0.406 0.34 0.258 0.928<br>Olong_026973-R2 0.427 1 0.573 0.099 1	1<br>Opunc06g25610.1 0.001 0.407 0.406 0.002 0.951<br>Opunc10g06480.1 0.427 0.998 0.571 0.098 0.984	1<br>Obrac06g20010.2 0.432 1 0.568 0.119 1<br>Obrac06g20020.1 0.001 0.407 0.406 0.002 0.952	1<br>Lperr02g22230.1 0.043 0.392 0.349 0.113 0.88<br>Lperr06g23510.1 0.402 1 0.598 0.074 1	1<br>AT1G76360.1 0.036 0.406 0.37 0.219 0.952<br>AT3G61130.1 0.465 0.999 0.534 0.207 1

Oglab06g25720.1	1<br>Oglab06g25720.1 0.001 1 0.999 0.001 1	0	0	0	0	0	0	0	0	0
	1<br>Oglab06g11430.1 0.001 0.629 0.628 0.001 0.997<br>Oglab06g12400.1 0.782 0.992 0.21 0.422 0.995	1<br>Obart06g11630.1 0.001 0.631 0.63 0.001 0.996<br>Obart06g12910.1 0.782 0.992 0.21 0.422 0.995	1<br>Osjap06g13090.1 0.004 0.67 0.666 0.003 0.966<br>Osjap06g14920.1 0.782 0.992 0.21 0.422 0.995	1<br>Osind06g13010.1 0.019 0.644 0.625 0.005 0.999<br>Osind06g14680.1 0.782 0.997 0.215 0.283 0.989	1<br>Orufi06g12040.1 0.004 0.67 0.666 0.003 0.966<br>Orufi06g13730.1 0.782 0.992 0.21 0.296 0.994	0<br>Olong_024681-R1 0.003 0.645 0.642 0.161 0.964	1<br>Opunc06g10960.1 0.004 0.67 0.666 0.003 0.97<br>Opunc06g12290.1 0.782 0.992 0.21 0.415 0.993	1<br>Obrac06g08260.2 0.001 0.646 0.645 0.037 0.965<br>Obrac08g03520.1 0.782 0.992 0.21 0.431 0.993	0<br>Lperr06g10400.1 0.001 0.632 0.631 0.002 0.983	0
Oglab06g26450.1	1<br>Oglab06g26450.1 0.005 1 0.995 0.005 1	0	0	0	0	0	0	0	0	0
	0<br>Oglab06g11740.1 0.297 0.99 0.693 0.051 0.891	1<br>Obart06g11070.1 0.005 0.287 0.282 0.568 0.761<br>Obart06g11970.1 0.297 0.99 0.693 0.051 0.891	1<br>Osjap06g12660.1 0.005 0.446 0.441 0.568 0.895<br>Osjap06g13590.1 0.475 0.99 0.515 0.154 0.894	1<br>Osind06g12570.1 0.005 0.446 0.441 0.358 0.844<br>Osind06g13420.1 0.475 0.99 0.515 0.146 0.719	1<br>Orufi06g11690.1 0.005 0.446 0.441 0.574 0.905<br>Orufi06g12520.1 0.475 0.99 0.515 0.154 0.894	1<br>Olong_006141-R1 0.475 0.99 0.515 0.131 0.891<br>Olong_026282-R1 0.005 0.332 0.327 0.569 0.923	0<br>Opunc06g10640.1 0.005 0.54 0.535 0.43 0.835	0<br>Obrac06g08090.1 0.005 0.584 0.579 0.543 0.993	1<br>Lperr06g09740.1 0.005 0.361 0.356 0.509 0.758<br>Lperr06g10530.1 0.485 0.99 0.505 0.191 0.921	0
Oglab07g03220.1	1<br>Oglab07g03220.1 0.003 1 0.997 0.003 1	0	0	0	0	0	0	0	0	0
	0<br>Oglab04g16900.2 0.003 0.416 0.413 0.002 0.269	1<br>Obart07g03300.1 0.003 0.416 0.413 0.002 0.262<br>Obart07g03310.1 0.419 1 0.581 0.072 1	1<br>Osjap04g21970.2 0.003 0.416 0.413 0.002 0.253<br>Osjap07g03650.1 0.419 1 0.581 0.072 1	1<br>Osind04g21080.2 0.003 0.416 0.413 0.002 0.269<br>Osind07g03190.1 0.419 1 0.581 0.072 1	1<br>Orufi04g19960.1 0.003 0.416 0.413 0.002 0.269<br>Orufi07g02980.1 0.419 1 0.581 0.065 1	1<br>Olong_006794-R1 0.419 1 0.581 0.072 1<br>Olong_009547-R1 0.003 0.416 0.413 0.002 0.253	1<br>Opunc04g16180.3 0.003 0.416 0.413 0.002 0.269<br>Opunc07g03180.1 0.419 1 0.581 0.21 1	1<br>Obrac04g11780.1 0.003 0.416 0.413 0.002 0.259<br>Obrac07g01940.1 0.419 0.995 0.576 0.068 0.928	0<br>Lperr07g02800.1 0.003 0.416 0.413 0.002 0.273	1<br>AT1G34770.1 0.419 0.989 0.57 0.072 0.975<br>AT4G16480.1 0.003 0.416 0.413 0.003 0.27
Oglab07g20540.2	1<br>Oglab07g20540.1 0.001 1 0.999 0.001 1<br>Oglab07g20540.2 0.001 1 0.999 0.001 1	1<br>Obart07g23990.1 0.001 1 0.999 0.001 1	0	0	0	0	0	0	0	0
	1<br>Oglab05g21510.1 0.602 1 0.398 0.068 1<br>Oglab12g08980.2 0.019 0.597 0.578 0.031 0.953	1<br>Obart04g03800.1 0.59 1 0.41 0.022 1<br>Obart10g03330.1 0.031 0.583 0.552 0.052 0.967	1<br>Osjap06g10100.1 0.596 1 0.404 0.044 1<br>Osjap10g03880.1 0.028 0.583 0.555 0.047 0.967	1<br>Osind05g26020.2 0.602 1 0.398 0.068 1<br>Osind12g09130.1 0.031 0.597 0.566 0.009 0.945	1<br>Orufi05g24100.1 0.602 1 0.398 0.142 1<br>Orufi12g09900.2 0.031 0.597 0.566 0.057 0.953	0<br>Olong_008774-R1 0.577 1 0.423 0.006 1	1<br>Opunc10g02860.1 0.023 0.583 0.56 0.038 0.967<br>Opunc10g02870.1 0.59 1 0.41 0.025 0.989	1<br>Obrac07g07280.1 0.004 0.568 0.564 0.003 0.921<br>Obrac07g07970.1 0.578 1 0.422 0.013 1	1<br>Lperr05g10370.1 0.036 0.583 0.547 0.059 0.935<br>Lperr06g09490.1 0.59 1 0.41 0.024 1	0<br>AT2G37460.1 0.038 0.582 0.544 0.045 0.905
Oglab08g01630.1	1<br>Oglab08g01630.1 0.004 1 0.996 0.004 1	0	0	0	1<br>Orufi08g02390.1 0.004 0.989 0.985 0.004 0.996<br>Orufi08g02390.2 0.004 0.989 0.985 0.004 0.996<br>Orufi08g02390.3 0.004 0.993 0.989 0.003 0.887	0	0	0	0	0
	0<br>Oglab08g01670.1 0.658 0.978 0.32 0.418 0.942	1<br>Obart02g18480.1 0.069 0.611 0.542 0.625 0.865<br>Obart08g02150.1 0.658 0.993 0.335 0.181 0.774	1<br>Osjap02g21180.1 0.069 0.611 0.542 0.658 0.877<br>Osjap08g02350.1 0.658 0.993 0.335 0.181 0.774	1<br>Osind02g21110.1 0.069 0.611 0.542 0.612 0.86<br>Osind08g02280.1 0.658 0.978 0.32 0.494 0.95	1<br>Orufi02g19480.1 0.069 0.611 0.542 0.665 0.88<br>Orufi08g02440.1 0.658 0.978 0.32 0.462 0.946	0<br>Olong_001677-R1 0.658 0.978 0.32 0.235 0.924	1<br>Opunc02g16440.1 0.069 0.611 0.542 0.606 0.858<br>Opunc08g02010.1 0.658 0.975 0.317 0.329 0.956	1<br>Obrac02g12990.1 0.069 0.611 0.542 0.612 0.86<br>Obrac08g01890.2 0.658 0.971 0.313 0.325 0.924	0<br>Lperr08g02240.1 0.658 0.971 0.313 0.314 0.909	0
Oglab08g04500.1	1<br>Oglab08g04500.1 0.004 1 0.996 0.004 1	0	0	0	0	0	0	0	0	0
	0<br>Oglab08g04470.1 0.535 0.961 0.426 0.194 0.524	1<br>Obart08g05010.1 0.662 1 0.338 0.094 1<br>Obart08g05020.1 0.004 0.575 0.571 0.008 1	1<br>Osjap08g05460.1 0.004 0.575 0.571 0.007 1<br>Osjap09g20620.1 0.662 0.987 0.325 0.008 0.597	1<br>Osind08g05440.1 0.004 0.575 0.571 0.007 1<br>Osind09g14950.1 0.649 0.982 0.333 0.372 0.717	1<br>Orufi08g05620.1 0.004 0.575 0.571 0.007 1<br>Orufi09g11280.1 0.649 0.982 0.333 0.007 0.993	1<br>Olong_011729-R1 0.654 0.987 0.333 0.025 0.988<br>Olong_014757-R1 0.004 0.575 0.571 0.182 1	0<br>Opunc10g11500.5 0.491 0.961 0.47 0.143 0.519	0<br>Obrac09g13140.3 0.654 0.987 0.333 0.814 0.926	0<br>Lperr08g04770.1 0.36 0.952 0.592 0.125 0.592	0<br>AT1G31340.1 0.649 0.987 0.338 0.006 0.981

Oglab08g05640.1	1<br>Oglab08g05640.1 0.002 1 0.998 0.002 1	1<br>Obart08g06070.1 0.002 1 0.998 0.002 1	1<br>Osjap08g06650.1 0.002 1 0.998 0.002 1	1<br>Osind08g06790.1 0.002 1 0.998 0.002 1	1<br>Orufi08g06840.1 0.002 1 0.998 0.002 1	0	1<br>Opunc08g05850.1 0.047 1 0.953 0.068 1	0	0	0
	1<br>Oglab02g26360.1 0.348 0.996 0.648 0.01 0.994<br>Oglab04g16180.1 0.047 0.275 0.228 0.198 0.94	1<br>Obart02g28440.1 0.348 0.996 0.648 0.093 0.994<br>Obart04g17770.1 0.047 0.275 0.228 0.198 0.94	1<br>Osjap04g21220.1 0.047 0.275 0.228 0.21 0.936<br>Osjap09g20870.1 0.359 0.984 0.625 0.208 0.966	1<br>Osind04g20250.1 0.047 0.275 0.228 0.198 0.94<br>Osind09g20960.1 0.359 0.984 0.625 0.208 0.966	1<br>Orufi04g19250.2 0.047 0.275 0.228 0.21 0.936<br>Orufi09g21960.1 0.359 0.984 0.625 0.2 0.965	1<br>Olong_008030-R1 0.047 0.275 0.228 0.232 0.937<br>Olong_008031-R2 0.277 1 0.723 0.106 1	1<br>Opunc04g15550.1 0.047 0.275 0.228 0.206 0.938<br>Opunc04g18740.1 0.295 0.984 0.689 0.088 0.994	1<br>Obrac08g04780.1 0.277 1 0.723 0.098 1<br>Obrac08g04800.1 0.002 0.275 0.273 0.006 0.943	1<br>Lperr08g05690.1 0.277 1 0.723 0.1 1<br>Lperr08g05700.1 0.002 0.275 0.273 0.006 0.943	1<br>AT1G05310.1 0.33 0.982 0.652 0.183 0.99<br>AT4G30800.1 0.047 0.275 0.228 0.208 0.943
Oglab08g10710.1	1<br>Oglab08g10710.1 0.006 1 0.994 0.006 1	0	0	0	0	0	0	0	0	0
	0<br>Oglab05g14480.2 0.006 0.438 0.432 0.001 0.091	1<br>Obart01g20880.1 0.006 0.438 0.432 0.001 0.104<br>Obart09g19350.1 0.544 1 0.456 0.003 0.219	1<br>Osjap01g24620.1 0.006 0.438 0.432 0.001 0.104<br>Osjap09g19760.2 0.521 1 0.479 0.075 0.284	1<br>Osind03g16830.1 0.006 0.479 0.473 0.001 0.071<br>Osind09g19920.1 0.521 1 0.479 0.075 0.284	1<br>Orufi01g23550.1 0.006 0.438 0.432 0.001 0.104<br>Orufi09g20900.1 0.544 1 0.456 0.003 0.219	1<br>Olong_001741-R1 0.006 0.479 0.473 0.001 0.106<br>Olong_028599-R1 0.544 1 0.456 0.009 0.703	1<br>Opunc09g17720.1 0.521 1 0.479 0.08 0.288<br>Opunc10g17020.1 0.006 0.444 0.438 0.002 0.128	1<br>Obrac01g12860.1 0.006 0.355 0.349 0.001 0.073<br>Obrac09g12650.1 0.521 1 0.479 0.06 0.276	1<br>Lperr01g14840.1 0.006 0.402 0.396 0.001 0.093<br>Lperr09g15230.1 0.521 1 0.479 0.073 0.281	0<br>AT1G02110.1 0.006 0.479 0.473 0.001 0.115<br>AT2G24220.1 0.692 1 0.308 0.075 0.219
Oglab08g13140.1	1<br>Oglab08g13140.1 0.001 1 0.999 0.001 1	0	1<br>Osjap08g14980.1 0.001 0.819 0.818 0.347 0.856	0	0	0	0	0	0	0
	1<br>Oglab04g06580.1 0.001 0.443 0.442 0.399 0.772<br>Oglab08g13180.1 0.466 0.992 0.526 0.035 0.979	1<br>Obart08g14060.1 0.466 0.992 0.526 0.04 0.976<br>Obart12g10850.1 0.001 0.444 0.443 0.397 0.776	1<br>Osjap08g15020.1 0.466 0.992 0.526 0.035 0.979<br>Osjap12g14110.1 0.001 0.444 0.443 0.397 0.776	1<br>Osind12g11050.1 0.001 0.444 0.443 0.397 0.776<br>Osind12g18550.1 0.471 0.992 0.521 0.004 0.982	1<br>Orufi08g15830.1 0.466 0.992 0.526 0.004 0.982<br>Orufi12g12010.1 0.001 0.444 0.443 0.388 0.773	0<br>Olong_015986-R1 0.425 0.936 0.511 0.754 0.962	1<br>Opunc08g12640.1 0.459 0.992 0.533 0.004 0.981<br>Opunc12g09730.1 0.001 0.444 0.443 0.397 0.778	1<br>Obrac04g04430.1 0.001 0.443 0.442 0.399 0.772<br>Obrac08g09240.1 0.466 0.992 0.526 0.155 0.986	1<br>Lperr08g11630.1 0.451 0.992 0.541 0.002 0.987<br>Lperr12g09160.1 0.001 0.444 0.443 0.397 0.776	1<br>AT2G16050.1 0.471 0.992 0.521 0.004 0.975<br>AT4G10710.1 0.001 0.443 0.442 0.402 0.772
Oglab08g15520.1	1<br>Oglab01g06790.1 0.007 0.844 0.837 0.061 1<br>Oglab08g15520.1 0.007 1 0.993 0.007 1	1<br>Obart01g07700.1 0.007 0.844 0.837 0.061 1<br>Obart08g16670.1 0.007 0.993 0.986 0.007 0.993	1<br>Osjap01g09050.1 0.007 0.844 0.837 0.061 1	1<br>Osind01g08880.1 0.102 0.986 0.884 0.004 0.567	1<br>Orufi01g08450.1 0.007 0.973 0.966 0.026 0.487	1<br>Olong_003647-R1 0.007 0.83 0.823 0.061 0.985	0	0	0	0
	1<br>Oglab03g37410.1 0.714 1 0.286 0.049 0.141<br>Oglab11g05440.1 0.007 0.694 0.687 0.573 0.683	1<br>Obart07g08560.1 0.694 0.952 0.258 0.009 0.192<br>Obart11g05910.1 0.007 0.694 0.687 0.752 0.834	1<br>Osjap01g24690.1 0.007 0.694 0.687 0.104 0.321<br>Osjap07g09900.1 0.694 1 0.306 0.005 0.126	1<br>Osind07g08950.1 0.694 1 0.306 0.004 0.113<br>Osind11g05650.1 0.007 0.694 0.687 0.103 0.319	1<br>Orufi01g23650.1 0.007 0.694 0.687 0.106 0.307<br>Orufi07g08630.1 0.694 1 0.306 0.005 0.117	1<br>Olong_005787-R1 0.007 0.694 0.687 0.103 0.319<br>Olong_016241-R1 0.728 1 0.272 0.735 0.775	1<br>Opunc01g21000.1 0.007 0.694 0.687 0.114 0.334<br>Opunc07g08090.1 0.694 1 0.306 0.004 0.092	1<br>Obrac07g05110.1 0.728 0.993 0.265 0.077 0.151<br>Obrac11g03720.1 0.007 0.694 0.687 0.112 0.34	1<br>Lperr01g17850.1 0.007 0.694 0.687 0.105 0.313<br>Lperr07g08590.1 0.694 0.993 0.299 0.022 0.124	1<br>AT3G55700.1 0.728 0.973 0.245 0.015 0.093<br>AT4G38130.1 0.007 0.68 0.673 0.09 0.269
Oglab09g13510.1	1<br>Oglab09g13510.1 0.002 1 0.998 0.002 1<br>Oglab09g13510.2 0.002 1 0.998 0.002 1<br>Oglab09g13510.3 0.002 1 0.998 0.002 1<br>Oglab09g13510.4 0.002 1 0.998 0.002 1	1<br>Obart09g14980.1 0.002 1 0.998 0.002 1<br>Obart09g14980.2 0.002 1 0.998 0.002 1	1<br>Osjap09g15430.2 0.002 1 0.998 0.002 1<br>Osjap09g15430.3 0.002 1 0.998 0.002 1	1<br>Osind09g15570.1 0.002 1 0.998 0.002 1<br>Osind09g15570.2 0.002 1 0.998 0.002 1	1<br>Orufi09g16160.1 0.002 1 0.998 0.002 1<br>Orufi09g16160.2 0.002 1 0.998 0.002 1	0	1<br>Opunc09g13420.1 0.002 1 0.998 0.002 1<br>Opunc09g13420.2 0.002 1 0.998 0.002 1	1<br>Obrac09g09090.1 0.002 1 0.998 0.002 1	0	0
	1<br>Oglab08g19240.1 0.519 0.994 0.475 0.014 0.986<br>Oglab12g18680.1 0.002 0.501 0.499 0.003 0.997	1<br>Obart02g06180.1 0.009 0.501 0.492 0.01 0.997<br>Obart08g20390.1 0.501 0.994 0.493 0.566 0.988	1<br>Osjap02g06650.1 0.009 0.501 0.492 0.01 0.997<br>Osjap09g15430.1 0.514 1 0.486 0.003 1	1<br>Osind02g06940.1 0.009 0.501 0.492 0.01 0.997<br>Osind08g23920.1 0.519 0.994 0.475 0.012 0.988	1<br>Orufi04g24220.1 0.012 0.502 0.49 0.165 1<br>Orufi08g22880.1 0.519 0.994 0.475 0.012 0.988	1<br>Olong_018059-R1 0.518 0.992 0.474 0.046 0.957<br>Olong_024186-R1 0.002 0.501 0.499 0.003 0.997	1<br>Opunc02g05570.1 0.005 0.501 0.496 0.087 0.997<br>Opunc08g18560.1 0.519 0.994 0.475 0.012 0.969	0<br>Obrac08g13590.1 0.499 0.994 0.495 0.689 0.994	1<br>Lperr04g18350.1 0.002 0.501 0.499 0.021 0.997<br>Lperr08g17230.1 0.507 0.994 0.487 0.021 0.997	1<br>AT2G21270.3 0.003 0.501 0.498 0.059 0.997<br>AT5G42800.1 0.516 0.992 0.476 0.003 0.851
Oglab09g13510.2	1<br>Oglab09g13510.1 0.002 1 0.998 0.002 1<br>Oglab09g13510.2 0.002 1 0.998 0.002 1<br>Oglab09g13510.3 0.002 1 0.998 0.002 1<br>Oglab09g13510.4 0.002 1 0.998 0.002 1	1<br>Obart09g14980.1 0.002 1 0.998 0.002 1<br>Obart09g14980.2 0.002 1 0.998 0.002 1	1<br>Osjap09g15430.2 0.002 1 0.998 0.002 1<br>Osjap09g15430.3 0.002 1 0.998 0.002 1	1<br>Osind09g15570.1 0.002 1 0.998 0.002 1<br>Osind09g15570.2 0.002 1 0.998 0.002 1	1<br>Orufi09g16160.1 0.002 1 0.998 0.002 1<br>Orufi09g16160.2 0.002 1 0.998 0.002 1	0	1<br>Opunc09g13420.1 0.002 1 0.998 0.002 1<br>Opunc09g13420.2 0.002 1 0.998 0.002 1	1<br>Obrac09g09090.1 0.002 1 0.998 0.002 1	0	0
	1	1	1	1	1	1	1	1	1	1

	Oglab08g19240.1 0.541 0.994 0.453 0.014 0.986<br>Oglab12g18680.1 0.002 0.522 0.52 0.003 0.997	Obart02g06180.1 0.01 0.522 0.512 0.01 0.997<br>Obart08g20390.1 0.522 0.994 0.472 0.566 0.988	Osjap02g06650.1 0.01 0.522 0.512 0.01 0.997<br>Osjap09g15430.1 0.537 1 0.463 0.003 1	Osind02g06940.1 0.01 0.522 0.512 0.01 0.997<br>Osind08g23920.1 0.541 0.994 0.453 0.012 0.988	Orufi04g24220.1 0.013 0.524 0.511 0.165 1<br>Orufi08g22880.1 0.541 0.994 0.453 0.012 0.988	Olong_014421-R1 0.537 0.992 0.455 0.002 0.984<br>Olong_024186-R1 0.002 0.522 0.52 0.003 0.997	Opunc02g05570.1 0.005 0.522 0.517 0.087 0.997<br>Opunc08g18560.1 0.541 0.994 0.453 0.012 0.969	Obrac02g04550.3 0.008 0.522 0.514 0.214 0.997<br>Obrac09g00560.1 0.54 0.992 0.452 0.043 0.954	Lperr04g18350.1 0.002 0.522 0.52 0.021 0.997<br>Lperr08g17230.1 0.529 0.994 0.465 0.021 0.997	AT1G15950.1 0.538 0.992 0.454 0.015 0.936<br>AT2G21270.3 0.003 0.522 0.519 0.059 0.997
Oglab09g13510.3	1<br>Oglab09g13510.1 0.002 1 0.998 0.002 1<br>Oglab09g13510.2 0.002 1 0.998 0.002 1<br>Oglab09g13510.3 0.002 1 0.998 0.002 1<br>Oglab09g13510.4 0.002 1 0.998 0.002 1	1<br>Obart09g14980.1 0.002 1 0.998 0.002 1<br>Obart09g14980.2 0.002 1 0.998 0.002 1	1<br>Osjap09g15430.2 0.002 1 0.998 0.002 1<br>Osjap09g15430.3 0.002 1 0.998 0.002 1	1<br>Osind09g15570.1 0.002 1 0.998 0.002 1<br>Osind09g15570.2 0.002 1 0.998 0.002 1	1<br>Orufi09g16160.1 0.002 1 0.998 0.002 1<br>Orufi09g16160.2 0.002 1 0.998 0.002 1	0	1<br>Opunc09g13420.1 0.002 1 0.998 0.002 1<br>Opunc09g13420.2 0.002 1 0.998 0.002 1	1<br>Obrac09g09090.1 0.002 1 0.998 0.002 1	0	0
	1<br>Oglab08g19240.1 0.544 0.993 0.449 0.143 0.986<br>Oglab12g18680.1 0.002 0.542 0.54 0.003 1	1<br>Obart02g06180.1 0.01 0.54 0.53 0.01 0.997<br>Obart08g20390.1 0.544 0.993 0.449 0.631 0.988	1<br>Osjap02g06650.1 0.01 0.54 0.53 0.01 0.997<br>Osjap09g15430.1 0.544 1 0.456 0.131 1	1<br>Osind02g06940.1 0.01 0.54 0.53 0.01 0.997<br>Osind08g23920.1 0.544 0.993 0.449 0.131 0.988	1<br>Orufi04g24220.1 0.013 0.542 0.529 0.165 1<br>Orufi08g22880.1 0.544 0.993 0.449 0.131 0.988	1<br>Olong_013054-R1 0.547 0.992 0.445 0.149 0.979<br>Olong_024186-R1 0.002 0.542 0.54 0.003 1	1<br>Opunc04g20190.1 0.007 0.542 0.535 0.006 1<br>Opunc08g18560.1 0.544 0.993 0.449 0.128 0.969	1<br>Obrac02g04550.3 0.008 0.54 0.532 0.214 0.997<br>Obrac09g00560.1 0.548 0.998 0.45 0.164 0.966	1<br>Lperr04g18350.1 0.002 0.542 0.54 0.021 1<br>Lperr09g11120.1 0.544 1 0.456 0.156 1	1<br>AT1G68540.1 0.542 0.992 0.45 0.112 1<br>AT2G21270.3 0.003 0.54 0.537 0.059 0.997
Oglab09g13510.4	1<br>Oglab09g13510.1 0.002 1 0.998 0.002 1<br>Oglab09g13510.2 0.002 1 0.998 0.002 1<br>Oglab09g13510.3 0.002 1 0.998 0.002 1<br>Oglab09g13510.4 0.002 1 0.998 0.002 1	1<br>Obart09g14980.1 0.002 1 0.998 0.002 1<br>Obart09g14980.2 0.002 1 0.998 0.002 1	1<br>Osjap09g15430.2 0.002 1 0.998 0.002 1<br>Osjap09g15430.3 0.002 1 0.998 0.002 1	1<br>Osind09g15570.1 0.002 1 0.998 0.002 1<br>Osind09g15570.2 0.002 1 0.998 0.002 1	1<br>Orufi09g16160.1 0.002 1 0.998 0.002 1<br>Orufi09g16160.2 0.002 1 0.998 0.002 1	0	1<br>Opunc09g13420.1 0.002 1 0.998 0.002 1<br>Opunc09g13420.2 0.002 1 0.998 0.002 1	1<br>Obrac09g09090.1 0.002 1 0.998 0.002 1	0	0
	1<br>Oglab08g19240.1 0.569 0.993 0.424 0.143 0.986<br>Oglab12g18680.1 0.002 0.567 0.565 0.003 1	1<br>Obart02g06180.1 0.01 0.565 0.555 0.01 0.997<br>Obart08g20390.1 0.569 0.993 0.424 0.631 0.988	1<br>Osjap02g06650.1 0.01 0.565 0.555 0.01 0.997<br>Osjap09g15430.1 0.569 1 0.431 0.131 1	1<br>Osind02g06940.1 0.01 0.565 0.555 0.01 0.997<br>Osind08g23920.1 0.569 0.993 0.424 0.131 0.988	1<br>Orufi04g24220.1 0.014 0.567 0.553 0.165 1<br>Orufi08g22880.1 0.569 0.993 0.424 0.131 0.988	1<br>Olong_008093-R2 0.596 0.991 0.395 0.25 0.996<br>Olong_024186-R1 0.002 0.567 0.565 0.003 1	1<br>Opunc04g20190.1 0.007 0.567 0.56 0.006 1<br>Opunc08g18560.1 0.569 0.993 0.424 0.128 0.969	1<br>Obrac01g09090.1 0.605 0.998 0.393 0.299 0.982<br>Obrac02g04550.3 0.009 0.565 0.556 0.214 0.997	1<br>Lperr04g18350.1 0.002 0.567 0.565 0.021 1<br>Lperr09g11120.1 0.569 1 0.431 0.156 1	1<br>AT1G68540.1 0.567 0.991 0.424 0.112 1<br>AT2G21270.3 0.003 0.565 0.562 0.059 0.997
Oglab09g14970.1	1<br>Oglab09g14970.1 0.001 1 0.999 0.001 1	0	0	0	0	0	0	0	0	0
	1<br>Oglab03g15980.2 0.767 0.971 0.204 0.004 0.818<br>Oglab12g01080.1 0.001 0.686 0.685 0.001 0.984	1<br>Obart09g16470.1 0.713 0.971 0.258 0.077 0.857<br>Obart09g16480.1 0.001 0.69 0.689 0.001 0.977	1<br>Osjap09g16930.1 0.713 0.971 0.258 0.077 0.857<br>Osjap09g16940.1 0.001 0.69 0.689 0.001 0.977	1<br>Osind09g17090.1 0.713 0.971 0.258 0.077 0.857<br>Osind09g17100.1 0.001 0.69 0.689 0.001 0.977	1<br>Orufi09g17780.1 0.713 0.971 0.258 0.077 0.857<br>Orufi09g17790.1 0.001 0.69 0.689 0.001 0.977	1<br>Olong_000845-R1 0.001 0.544 0.543 0.002 1<br>Olong_000846-R1 0.713 0.971 0.258 0.075 0.862	1<br>Opunc09g14900.1 0.713 0.971 0.258 0.077 0.857<br>Opunc09g14910.1 0.001 0.707 0.706 0.001 1	1<br>Obrac08g14920.1 0.001 0.706 0.705 0.18 0.999<br>Obrac09g10340.1 0.713 0.971 0.258 0.077 0.857	1<br>Lperr09g12580.2 0.713 0.971 0.258 0.075 0.857<br>Lperr09g12600.1 0.001 0.707 0.706 0.001 1	1<br>AT2G31350.1 0.708 0.971 0.263 0.046 0.852<br>AT3G19610.1 0.001 0.689 0.688 0.045 0.956
Oglab09g17780.2	1<br>Oglab09g17780.1 0.001 1 0.999 0.484 1<br>Oglab09g17780.2 0.001 1 0.999 0.001 1<br>Oglab09g17780.3 0.001 1 0.999 0.001 1<br>Oglab09g17780.4 0.001 1 0.999 0.001 1<br>Oglab09g17780.5 0.001 1 0.999 0.001 1<br>Oglab09g17780.6 0.001 1 0.999 0.001 1<br>Oglab09g17780.7 0.001 0.987 0.986 0.001 0.979<br>Oglab09g17780.8 0.001 0.987 0.986 0.001 0.978	1<br>Obart09g19580.1 0.046 0.987 0.941 0.466 0.988	0	0	0	1<br>Olong_015753-R1 0.046 0.987 0.941 0.231 0.982	0	0	0	0
	0<br>Oglab06g21810.1 0.029 0.495 0.466 0.075 0.955	1<br>Obart05g23600.1 0.018 0.492 0.474 0.009 0.941<br>Obart09g19600.1 0.568 0.987 0.419 0.062 0.955	1<br>Osjap09g20010.5 0.001 0.515 0.514 0.003 0.997<br>Osjap09g20020.1 0.533 0.987 0.454 0.003 0.954	1<br>Osind09g20180.1 0.001 0.515 0.514 0.632 0.999<br>Osind09g20190.1 0.533 0.99 0.457 0.002 0.976	1<br>Orufi10g16450.1 0.533 1 0.467 0.003 0.812<br>Orufi10g16460.1 0.001 0.515 0.514 0.639 0.999	1<br>Olong_015754-R1 0.568 0.987 0.419 0.06 0.957<br>Olong_021159-R1 0.018 0.492 0.474 0.023 0.935	1<br>Opunc09g18000.1 0.001 0.493 0.492 0.003 0.965<br>Opunc09g18010.1 0.533 0.987 0.454 0.003 0.954	1<br>Obrac05g16330.2 0.018 0.492 0.474 0.011 0.928<br>Obrac09g12850.1 0.533 0.988 0.455 0.002 0.977	1<br>Lperr02g04840.1 0.04 0.511 0.471 0.265 0.995<br>Lperr09g15500.1 0.533 0.987 0.454 0.002 0.977	1<br>AT1G30910.1 0.563 0.985 0.422 0.05 0.95<br>AT3G12740.1 0.04 0.511 0.471 0.08 0.994

Oglab09g17780.3	1<br>Oglab09g17780.1 0.001 1 0.999 0.484 1<br>Oglab09g17780.2 0.001 1 0.999 0.001 1<br>Oglab09g17780.3 0.001 1 0.999 0.001 1<br>Oglab09g17780.4 0.001 1 0.999 0.001 1<br>Oglab09g17780.5 0.001 1 0.999 0.001 1<br>Oglab09g17780.6 0.001 1 0.999 0.001 1<br>Oglab09g17780.7 0.001 0.987 0.986 0.001 0.973<br>Oglab09g17780.8 0.001 0.987 0.986 0.001 0.972	1<br>Obart09g19580.1 0.046 0.987 0.941 0.466 0.985	0	0	0	1<br>Olong_015753-R1 0.046 0.987 0.941 0.231 0.977	0	0	0	0
	0<br>Oglab06g21810.1 0.03 0.498 0.468 0.075 0.955	1<br>Obart05g23600.1 0.018 0.495 0.477 0.009 0.941<br>Obart09g19600.1 0.572 0.987 0.415 0.062 0.944	1<br>Osjap09g20010.5 0.001 0.519 0.518 0.003 0.997<br>Osjap09g20020.1 0.536 0.987 0.451 0.003 0.941	1<br>Osind09g20180.1 0.001 0.519 0.518 0.632 0.999<br>Osind09g20190.1 0.536 0.987 0.451 0.002 0.969	1<br>Orufi10g16450.1 0.536 0.987 0.451 0.003 0.769<br>Orufi10g16460.1 0.001 0.519 0.518 0.639 0.999	1<br>Olong_005512-R4 0.573 0.991 0.418 0.623 0.984<br>Olong_021159-R1 0.018 0.495 0.477 0.023 0.935	1<br>Opunc09g18000.1 0.001 0.496 0.495 0.003 0.965<br>Opunc09g18010.1 0.536 0.987 0.451 0.003 0.941	1<br>Obrac05g16330.2 0.018 0.495 0.477 0.011 0.928<br>Obrac09g12850.1 0.536 0.987 0.451 0.002 0.971	1<br>Lperr02g04840.1 0.04 0.514 0.474 0.265 0.995<br>Lperr09g15500.1 0.536 0.987 0.451 0.002 0.971	1<br>AT1G30910.1 0.566 0.987 0.421 0.05 0.94<br>AT3G12740.1 0.04 0.514 0.474 0.08 0.994
Oglab09g17780.4	1<br>Oglab09g17780.1 0.001 1 0.999 0.484 1<br>Oglab09g17780.2 0.001 1 0.999 0.001 1<br>Oglab09g17780.3 0.001 1 0.999 0.001 1<br>Oglab09g17780.4 0.001 1 0.999 0.001 1<br>Oglab09g17780.5 0.001 1 0.999 0.001 1<br>Oglab09g17780.6 0.001 1 0.999 0.001 1<br>Oglab09g17780.7 0.001 0.987 0.986 0.001 0.979<br>Oglab09g17780.8 0.001 0.987 0.986 0.001 0.978	1<br>Obart09g19580.1 0.046 0.987 0.941 0.466 0.988	0	0	0	1<br>Olong_015753-R1 0.046 0.987 0.941 0.231 0.982	0	0	0	0
	0<br>Oglab06g21810.1 0.029 0.495 0.466 0.075 0.955	1<br>Obart05g23600.1 0.018 0.492 0.474 0.009 0.941<br>Obart09g19600.1 0.568 0.987 0.419 0.062 0.955	1<br>Osjap09g20010.5 0.001 0.515 0.514 0.003 0.997<br>Osjap09g20020.1 0.533 0.987 0.454 0.003 0.954	1<br>Osind09g20180.1 0.001 0.515 0.514 0.632 0.999<br>Osind09g20190.1 0.533 0.99 0.457 0.002 0.976	1<br>Orufi10g16450.1 0.533 1 0.467 0.003 0.812<br>Orufi10g16460.1 0.001 0.515 0.514 0.639 0.999	1<br>Olong_015754-R1 0.568 0.987 0.419 0.06 0.957<br>Olong_021159-R1 0.018 0.492 0.474 0.023 0.935	1<br>Opunc09g18000.1 0.001 0.493 0.492 0.003 0.965<br>Opunc09g18010.1 0.533 0.987 0.454 0.003 0.954	1<br>Obrac05g16330.2 0.018 0.492 0.474 0.011 0.928<br>Obrac09g12850.1 0.533 0.988 0.455 0.002 0.977	1<br>Lperr02g04840.1 0.04 0.511 0.471 0.265 0.995<br>Lperr09g15500.1 0.533 0.987 0.454 0.002 0.977	1<br>AT1G30910.1 0.563 0.985 0.422 0.05 0.95<br>AT3G12740.1 0.04 0.511 0.471 0.08 0.994
Oglab09g17780.5	1<br>Oglab09g17780.1 0.001 1 0.999 0.484 1<br>Oglab09g17780.2 0.001 1 0.999 0.001 1<br>Oglab09g17780.3 0.001 1 0.999 0.001 1<br>Oglab09g17780.4 0.001 1 0.999 0.001 1<br>Oglab09g17780.5 0.001 1 0.999 0.001 1<br>Oglab09g17780.6 0.001 1 0.999 0.001 1<br>Oglab09g17780.7 0.001 0.987 0.986 0.001 0.973<br>Oglab09g17780.8 0.001 0.987 0.986 0.001 0.972	1<br>Obart09g19580.1 0.046 0.987 0.941 0.466 0.985	0	0	0	1<br>Olong_015753-R1 0.046 0.987 0.941 0.231 0.977	0	0	0	0
	0<br>Oglab06g21810.1 0.03 0.498 0.468 0.075 0.955	1<br>Obart05g23600.1 0.018 0.495 0.477 0.009 0.941<br>Obart09g19600.1 0.572 0.987 0.415 0.062 0.944	1<br>Osjap09g20010.5 0.001 0.519 0.518 0.003 0.997<br>Osjap09g20020.1 0.536 0.987 0.451 0.003 0.941	1<br>Osind09g20180.1 0.001 0.519 0.518 0.632 0.999<br>Osind09g20190.1 0.536 0.987 0.451 0.002 0.969	1<br>Orufi10g16450.1 0.536 0.987 0.451 0.003 0.769<br>Orufi10g16460.1 0.001 0.519 0.518 0.639 0.999	1<br>Olong_005512-R4 0.573 0.991 0.418 0.623 0.984<br>Olong_021159-R1 0.018 0.495 0.477 0.023 0.935	1<br>Opunc09g18000.1 0.001 0.496 0.495 0.003 0.965<br>Opunc09g18010.1 0.536 0.987 0.451 0.003 0.941	1<br>Obrac05g16330.2 0.018 0.495 0.477 0.011 0.928<br>Obrac09g12850.1 0.536 0.987 0.451 0.002 0.971	1<br>Lperr02g04840.1 0.04 0.514 0.474 0.265 0.995<br>Lperr09g15500.1 0.536 0.987 0.451 0.002 0.971	1<br>AT1G30910.1 0.566 0.987 0.421 0.05 0.94<br>AT3G12740.1 0.04 0.514 0.474 0.08 0.994
Oglab09g17780.6	1<br>Oglab09g17780.1 0.001 1 0.999 0.484 1<br>Oglab09g17780.2 0.001 1 0.999 0.001 1<br>Oglab09g17780.3 0.001 1 0.999 0.001 1	1<br>Obart09g19580.1 0.046 0.987 0.941 0.466 0.985	0	0	0	1<br>Olong_015753-R1 0.046 0.987 0.941 0.231 0.977	0	0	0	0

	Oglab09g17780.4 0.001 1 0.999 0.001 1<br>Oglab09g17780.5 0.001 1 0.999 0.001 1<br>Oglab09g17780.6 0.001 1 0.999 0.001 1<br>Oglab09g17780.7 0.001 0.987 0.986 0.001 0.973<br>Oglab09g17780.8 0.001 0.987 0.986 0.001 0.972									
	0<br>Oglab06g21810.1 0.03 0.498 0.468 0.075 0.955	1<br>Obart05g23600.1 0.018 0.495 0.477 0.009 0.941<br>Obart09g19600.1 0.572 0.987 0.415 0.062 0.944	1<br>Osjap09g20010.5 0.001 0.519 0.518 0.003 0.997<br>Osjap09g20020.1 0.536 0.987 0.451 0.003 0.941	1<br>Osind09g20180.1 0.001 0.519 0.518 0.632 0.999<br>Osind09g20190.1 0.536 0.987 0.451 0.002 0.969	1<br>Orufi10g16450.1 0.536 0.987 0.451 0.003 0.769<br>Orufi10g16460.1 0.001 0.519 0.518 0.639 0.999	1<br>Olong_005512-R4 0.573 0.991 0.418 0.623 0.984<br>Olong_021159-R1 0.018 0.495 0.477 0.023 0.935	1<br>Opunc09g18000.1 0.001 0.496 0.495 0.003 0.965<br>Opunc09g18010.1 0.536 0.987 0.451 0.003 0.941	1<br>Obrac05g16330.2 0.018 0.495 0.477 0.011 0.928<br>Obrac09g12850.1 0.536 0.987 0.451 0.002 0.971	1<br>Lperr02g04840.1 0.04 0.514 0.474 0.265 0.995<br>Lperr09g15500.1 0.536 0.987 0.451 0.002 0.971	1<br>AT1G30910.1 0.566 0.987 0.421 0.05 0.94<br>AT3G12740.1 0.04 0.514 0.474 0.08 0.994
Oglab09g17780.7	1<br>Oglab09g17780.1 0.001 0.979 0.978 0.484 0.993<br>Oglab09g17780.2 0.001 0.979 0.978 0.001 0.987<br>Oglab09g17780.3 0.001 0.973 0.972 0.001 0.987<br>Oglab09g17780.4 0.001 0.979 0.978 0.001 0.987<br>Oglab09g17780.5 0.001 0.973 0.972 0.001 0.987<br>Oglab09g17780.6 0.001 0.973 0.972 0.001 0.987<br>Oglab09g17780.7 0.001 1 0.999 0.001 1<br>Oglab09g17780.8 0.001 1 0.999 0.001 1	1<br>Obart09g19580.1 0.044 1 0.956 0.466 1	0	0	0	1<br>Olong_015753-R1 0.044 1 0.956 0.231 1	0	0	0	0
	0<br>Oglab06g21810.1 0.029 0.481 0.452 0.075 0.955	1<br>Obart05g23600.1 0.017 0.478 0.461 0.009 0.941<br>Obart09g19600.1 0.552 1 0.448 0.062 1	1<br>Osjap09g20010.5 0.001 0.501 0.5 0.003 0.997<br>Osjap09g20020.1 0.518 1 0.482 0.003 1	1<br>Osind09g20180.1 0.001 0.501 0.5 0.632 0.999<br>Osind09g20190.2 0.535 1 0.465 0.034 1	1<br>Orufi10g16450.2 0.518 0.987 0.469 0.003 1<br>Orufi10g16460.1 0.001 0.501 0.5 0.639 0.999	1<br>Olong_015754-R1 0.552 1 0.448 0.06 1<br>Olong_021159-R1 0.017 0.478 0.461 0.023 0.935	1<br>Opunc09g18000.1 0.001 0.479 0.478 0.003 0.965<br>Opunc09g18010.1 0.518 1 0.482 0.003 1	1<br>Obrac05g16330.2 0.017 0.478 0.461 0.011 0.928<br>Obrac09g12850.1 0.518 1 0.482 0.002 1	1<br>Lperr02g04840.1 0.034 0.496 0.462 0.253 0.995<br>Lperr09g15500.1 0.518 1 0.482 0.002 1	1<br>AT1G30910.1 0.546 0.996 0.45 0.05 0.991<br>AT3G12740.1 0.034 0.496 0.462 0.066 0.994
Oglab09g17780.8	1<br>Oglab09g17780.1 0.001 0.978 0.977 0.484 0.993<br>Oglab09g17780.2 0.001 0.978 0.977 0.001 0.987<br>Oglab09g17780.3 0.001 0.972 0.971 0.001 0.987<br>Oglab09g17780.4 0.001 0.978 0.977 0.001 0.987<br>Oglab09g17780.5 0.001 0.972 0.971 0.001 0.987<br>Oglab09g17780.6 0.001 0.972 0.971 0.001 0.987<br>Oglab09g17780.7 0.001 1 0.999 0.001 1<br>Oglab09g17780.8 0.001 1 0.999 0.001 1	1<br>Obart09g19580.1 0.045 1 0.955 0.466 1	0	0	0	1<br>Olong_015753-R1 0.045 1 0.955 0.231 1	0	0	0	0
	0<br>Oglab06g21810.1 0.029 0.491 0.462 0.075 0.955	1<br>Obart05g23600.1 0.018 0.488 0.47 0.009 0.941<br>Obart09g19600.1 0.564 1 0.436 0.062 1	1<br>Osjap09g20010.5 0.001 0.511 0.51 0.003 0.997<br>Osjap09g20020.1 0.528 1 0.472 0.003 1	1<br>Osind09g20180.1 0.001 0.511 0.51 0.632 0.999<br>Osind09g20190.2 0.546 1 0.454 0.034 1	1<br>Orufi10g16450.2 0.528 0.987 0.459 0.003 1<br>Orufi10g16460.1 0.001 0.511 0.51 0.639 0.999	1<br>Olong_015754-R1 0.564 1 0.436 0.06 1<br>Olong_021159-R1 0.018 0.488 0.47 0.023 0.935	1<br>Opunc09g18000.1 0.001 0.489 0.488 0.003 0.965<br>Opunc09g18010.1 0.528 1 0.472 0.003 1	1<br>Obrac05g16330.2 0.018 0.488 0.47 0.011 0.928<br>Obrac09g12850.1 0.528 1 0.472 0.002 1	1<br>Lperr02g04840.1 0.035 0.507 0.472 0.253 0.995<br>Lperr09g15500.1 0.528 1 0.472 0.002 1	1<br>AT1G30910.1 0.558 0.996 0.438 0.05 0.991<br>AT3G12740.1 0.039 0.507 0.468 0.08 0.994
Oglab09g18200.1	1<br>Oglab09g18200.1 0.001 1 0.999 0.001 1	1<br>Obart09g19980.1 0.066 1 0.934 0.181 1<br>Obart09g19980.2 0.001 1 0.999 0.001 1	1<br>Osjap09g20460.1 0.001 0.999 0.998 0.001 0.999	1<br>Osind09g20560.1 0.001 0.985 0.984 0.001 0.998	1<br>Orufi09g21510.1 0.001 0.958 0.957 0.001 0.999	1<br>Olong_014589-R1 0.116 0.972 0.856 0.032 0.991<br>Olong_014589-R2 0.116 0.972 0.856 0.033 0.991	0	0	0	0
	1<br>Oglab02g17850.1 0.023 0.609 0.586 0.095 0.914<br>Oglab09g18170.1 0.619 0.999 0.38 0.13 0.998	1<br>Obart04g25370.1 0.047 0.617 0.57 0.176 0.967<br>Obart09g19950.1 0.619 0.999 0.38 0.13 0.998	1<br>Osjap02g21530.2 0.023 0.609 0.586 0.096 0.943<br>Osjap09g20430.1 0.649 0.999 0.35 0.151 0.998	1<br>Osind02g21430.2 0.023 0.609 0.586 0.096 0.943<br>Osind09g20550.1 0.619 0.999 0.38 0.058 0.999	1<br>Orufi02g19810.2 0.023 0.609 0.586 0.096 0.943<br>Orufi09g21500.1 0.649 0.999 0.35 0.151 0.998	0<br>Olong_014116-R1 0.063 0.617 0.554 0.069 0.957	1<br>Opunc09g18280.1 0.619 0.998 0.379 0.118 0.951<br>Opunc09g18290.2 0.001 0.618 0.617 0.001 0.971	1<br>Obrac09g13140.3 0.619 0.985 0.366 0.089 0.806<br>Obrac09g13160.1 0.001 0.618 0.617 0.001 0.953	1<br>Lperr09g15890.1 0.619 0.997 0.378 0.085 0.957<br>Lperr09g15920.1 0.001 0.618 0.617 0.001 0.958	0<br>AT1G18160.1 0.021 0.617 0.596 0.064 0.958

Oglab09g18980.1	1<br>Oglab09g18980.1 0.001 1 0.999 0.001 1	0	0	0	0	0	0	0	0	0
	1<br>Oglab01g27280.1 0.001 0.371 0.37 0.002 0.481<br>Oglab08g16490.1 0.403 1 0.597 0.001 1	1<br>Obart03g34250.1 0.025 0.39 0.365 0.05 0.573<br>Obart08g17650.1 0.403 1 0.597 0.001 1	1<br>Osjap01g37290.2 0.001 0.371 0.37 0.065 0.591<br>Osjap08g18690.1 0.45 1 0.55 0.006 1	1<br>Osind03g38060.1 0.025 0.39 0.365 0.05 0.573<br>Osind08g20790.1 0.442 1 0.558 0.001 1	1<br>Orufi01g35750.2 0.001 0.371 0.37 0.069 0.57<br>Orufi08g19800.1 0.45 1 0.55 0.006 1	1<br>Olong_000486-R1 0.403 1 0.597 0.077 1<br>Olong_000981-R1 0.001 0.371 0.37 0.002 0.539	1<br>Opunc01g31560.1 0.001 0.371 0.37 0.002 0.483<br>Opunc08g15500.1 0.458 1 0.542 0.028 1	1<br>Obrac03g25390.2 0.005 0.371 0.366 0.015 0.559<br>Obrac08g11470.1 0.439 1 0.561 0.004 1	1<br>Lperr03g28740.1 0.01 0.392 0.382 0.011 0.568<br>Lperr08g14590.1 0.48 1 0.52 0.103 1	1<br>AT2G03200.1 0.476 1 0.524 0.098 1<br>AT5G04885.1 0.006 0.397 0.391 0.011 0.55
Oglab10g02440.1	1<br>Oglab10g02440.1 0.001 1 0.999 0.001 1<br>Oglab10g02440.2 0.001 1 0.999 0.001 1	1<br>Obart10g02510.2 0.001 1 0.999 0.001 1	0	1<br>Osind10g02110.1 0.001 0.996 0.995 0.001 0.952	0	0	0	0	0	0
	0<br>Oglab10g02440.3 0.434 1 0.566 0.005 1	1<br>Obart10g02510.1 0.001 0.465 0.464 0.001 0.487<br>Obart11g08380.1 0.533 1 0.467 0.194 0.922	1<br>Osjap10g02940.1 0.02 0.375 0.355 0.027 0.984<br>Osjap10g02950.1 0.451 0.974 0.523 0.002 0.928	1<br>Osind10g00350.1 0.001 0.372 0.371 0.001 0.98<br>Osind11g07740.1 0.451 0.996 0.545 0.003 1	0<br>Orufi10g02530.1 0.213 0.996 0.783 0.284 1	1<br>Olong_016648-R1 0.213 0.996 0.783 0.265 1<br>Olong_020863-R1 0.004 0.212 0.208 0.007 0.797	1<br>Opunc10g02120.1 0.001 0.375 0.374 0.001 0.992<br>Opunc11g08150.1 0.451 1 0.549 0.003 0.911	1<br>Obrac03g10950.1 0.001 0.372 0.371 0.001 0.983<br>Obrac11g05340.1 0.451 1 0.549 0.002 0.922	1<br>Lperr10g00410.1 0.001 0.373 0.372 0.003 0.986<br>Lperr10g01950.1 0.451 1 0.549 0.003 0.895	0<br>AT1G21230.1 0.004 0.337 0.333 0.01 0.93<br>AT4G00830.1 0.528 0.974 0.446 0.194 0.891
Oglab10g02440.2	1<br>Oglab10g02440.1 0.001 1 0.999 0.001 1<br>Oglab10g02440.2 0.001 1 0.999 0.001 1	1<br>Obart10g02510.1 0.001 1 0.999 0.001 1	0	1<br>Osind10g02110.1 0.001 0.995 0.994 0.001 0.952	0	0	0	0	0	0
	0<br>Oglab10g02440.3 0.462 1 0.538 0.005 1	1<br>Obart10g02510.2 0.001 0.494 0.493 0.001 0.51<br>Obart11g08380.1 0.567 1 0.433 0.194 0.922	1<br>Osjap10g02940.1 0.021 0.399 0.378 0.027 0.984<br>Osjap10g02950.1 0.48 0.972 0.492 0.002 0.928	1<br>Osind10g00350.1 0.001 0.396 0.395 0.001 0.98<br>Osind11g07740.1 0.48 0.995 0.515 0.003 0.96	1<br>Orufi10g02520.1 0.021 0.225 0.204 0.065 0.758<br>Orufi10g02530.1 0.226 0.995 0.769 0.284 0.988	1<br>Olong_016648-R1 0.226 0.995 0.769 0.265 1<br>Olong_020863-R1 0.005 0.225 0.22 0.007 0.797	1<br>Opunc10g02120.1 0.001 0.399 0.398 0.001 0.992<br>Opunc11g08150.1 0.48 1 0.52 0.003 0.911	1<br>Obrac03g10950.1 0.001 0.396 0.395 0.001 0.983<br>Obrac11g05340.1 0.48 1 0.52 0.002 0.922	1<br>Lperr10g00410.1 0.001 0.397 0.396 0.003 0.986<br>Lperr10g01950.1 0.48 1 0.52 0.003 0.895	0<br>AT1G21230.1 0.005 0.358 0.353 0.01 0.93<br>AT4G00830.1 0.561 0.972 0.411 0.194 0.891
Oglab10g03910.1	1<br>Oglab10g03910.1 0.01 1 0.99 0.01 1	1<br>Obart10g18220.1 0.01 1 0.99 0.392 0.823	1<br>Osjap10g04950.1 0.01 1 0.99 0.01 1<br>Osjap10g21210.1 0.01 1 0.99 0.477 1	1<br>Osind10g03420.1 0.01 1 0.99 0.558 1<br>Osind10g17700.1 0.01 1 0.99 0.477 1	1<br>Orufi10g04520.1 0.01 1 0.99 0.01 1<br>Orufi10g19420.1 0.01 1 0.99 0.477 1	1<br>Olong_009744-R1 0.01 1 0.99 0.651 1<br>Olong_009744-R2 0.01 1 0.99 0.641 1	0	0	0	0
	1<br>Oglab02g32260.1 0.361 1 0.639 0.838 0.924<br>Oglab04g27900.1 0.01 0.33 0.32 0.14 0.208	1<br>Obart02g34980.1 0.361 1 0.639 0.838 0.924<br>Obart04g29850.1 0.01 0.33 0.32 0.131 0.196	0<br>Osjap07g13660.9 0.361 1 0.639 0.733 0.947	1<br>Osind02g38620.1 0.361 1 0.639 0.835 0.923<br>Osind06g00010.1 0.01 0.361 0.351 0.609 0.978	1<br>Orufi02g36440.1 0.361 1 0.639 0.835 0.923<br>Orufi08g26670.5 0.01 0.351 0.341 0.583 0.749	1<br>Olong_008835-R2 0.361 1 0.639 0.809 0.91<br>Olong_022843-R2 0.01 0.33 0.32 0.14 0.208	1<br>Opunc02g32260.1 0.361 1 0.639 0.84 0.925<br>Opunc04g27380.2 0.01 0.33 0.32 0.14 0.208	1<br>Obrac02g25650.1 0.381 1 0.619 0.839 0.923<br>Obrac04g21100.1 0.01 0.33 0.32 0.145 0.217	1<br>Lperr02g28770.1 0.381 1 0.619 0.834 0.92<br>Lperr04g25370.2 0.01 0.33 0.32 0.151 0.225	0
Oglab10g08680.2	1<br>Oglab10g08680.1 0.009 1 0.991 0.008 1<br>Oglab10g08680.2 0.001 1 0.999 0.001 1	1<br>Obart10g09110.1 0.009 1 0.991 0.008 1<br>Obart10g09110.2 0.001 1 0.999 0.001 1	0	0	1<br>Orufi10g09480.1 0.009 1 0.991 0.008 1<br>Orufi10g09480.2 0.009 1 0.991 0.008 1<br>Orufi10g09480.3 0.001 1 0.999 0.001 1	1<br>Olong_027249-R1 0.03 1 0.97 0.001 1<br>Olong_027249-R2 0.03 1 0.97 0.001 1	0	0	0	0
	1<br>Oglab02g10660.1 0.625 0.999 0.374 0.129 0.991<br>Oglab12g06810.1 0.013 0.615 0.602 0.056 0.969	0<br>Obart02g10660.1 0.625 0.999 0.374 0.129 0.991	1<br>Osjap01g36630.1 0.626 1 0.374 0.222 1<br>Osjap10g10740.1 0.001 0.626 0.625 0.002 0.997	1<br>Osind10g08120.1 0.626 1 0.374 0.222 1<br>Osind10g08130.1 0.001 0.626 0.625 0.002 0.997	1<br>Orufi01g35060.1 0.626 1 0.374 0.222 1<br>Orufi12g07540.1 0.013 0.615 0.602 0.039 0.673	1<br>Olong_022306-R1 0.609 0.991 0.382 0.19 0.992<br>Olong_024721-R1 0.105 0.604 0.499 0.048 0.942	1<br>Opunc01g30900.1 0.626 1 0.374 0.222 1<br>Opunc10g07870.1 0.004 0.626 0.622 0.003 0.997	1<br>Obrac01g24330.1 0.626 1 0.374 0.221 1<br>Obrac12g04960.2 0.102 0.615 0.513 0.026 0.902	1<br>Lperr01g27340.1 0.626 1 0.374 0.228 1<br>Lperr09g14980.1 0.001 0.604 0.603 0.002 0.959	0<br>AT4G14820.1 0.001 0.614 0.613 0.004 0.86
Oglab10g14420.1	1<br>Oglab10g14420.1 0.007 1 0.993 0.007 1	0	1<br>Osjap04g26670.1 0.059 0.98 0.921 0.005 0.989<br>Osjap10g21180.1 0.046 1 0.954 0.495 1	0	1<br>Orufi04g24510.1 0.007 0.993 0.986 0.009 1<br>Orufi10g19400.1 0.039 1 0.961 0.009 1	0	1<br>Opunc06g20630.1 0.039 1 0.961 0.316 0.984	0	0	0
	1<br>Oglab04g27360.1 0.671 0.928 0.257 0.741 0.891	1<br>Obart04g29320.1 0.671 0.928 0.257 0.741 0.891	1<br>Osjap02g36030.1 0.039 0.586 0.547 0.001 0.098	1<br>Osind04g32680.1 0.671 0.928 0.257 0.741 0.891	1<br>Orufi02g33550.1 0.007 0.586 0.579 0.001 0.104	0<br>Olong_004819-R1 0.026 0.362 0.336 0.231 0.615	1<br>Opunc02g29570.2 0.033 0.586 0.553 0.008 0.106	0<br>Obrac02g23230.1 0.033 0.586 0.553 0.007 0.1	0<br>Lperr02g26070.3 0.007 0.586 0.579 0.001 0.112	0<br>AT1G59610.1 0.039 0.586 0.547 0.001 0.099

	Oglab06g09210.2 0.039 0.586 0.547 0.082 0.173	Obart08g15190.1 0.039 0.586 0.547 0.001 0.106	Osjap04g33480.1 0.671 0.928 0.257 0.741 0.891	Osind08g18030.1 0.039 0.586 0.547 0.001 0.106	Orufi04g31030.1 0.671 0.928 0.257 0.741 0.891		Opunc04g26840.1 0.671 0.928 0.257 0.741 0.891			
Oglab10g16090.1	1<br>Oglab10g16090.1 0.001 1 0.999 0.001 1	1<br>Obart10g19720.1 0.001 0.861 0.86 0.002 0.992	0	1<br>Osind10g19230.1 0.001 0.861 0.86 0.002 0.992	1<br>Orufi10g20960.1 0.001 0.861 0.86 0.002 0.992	0	1<br>Opunc10g18180.1 0.001 0.99 0.989 0.002 0.998	0	0	0
	1<br>Oglab01g14760.1 0.419 0.984 0.565 0.2 1<br>Oglab08g03250.2 0.037 0.374 0.337 0.052 0.955	1<br>Obart01g17550.1 0.419 0.977 0.558 0.174 0.863<br>Obart08g03750.1 0.037 0.374 0.337 0.057 0.95	1<br>Osjap10g22750.1 0.411 1 0.589 0.061 1<br>Osjap10g22760.1 0.001 0.392 0.391 0.003 0.993	1<br>Osind01g20890.1 0.419 0.977 0.558 0.154 0.802<br>Osind08g03990.1 0.037 0.374 0.337 0.057 0.95	1<br>Orufi01g19490.1 0.419 0.977 0.558 0.171 0.862<br>Orufi08g04140.1 0.037 0.374 0.337 0.057 0.95	1<br>Olong_006312-R1 0.416 1 0.584 0.067 1<br>Olong_006313-R1 0.001 0.392 0.391 0.003 0.993	1<br>Opunc01g18040.1 0.419 0.984 0.565 0.174 1<br>Opunc08g03700.1 0.014 0.374 0.36 0.011 0.951	1<br>Obrac01g13330.1 0.423 0.984 0.561 0.196 1<br>Obrac10g13120.1 0.001 0.392 0.391 0.004 0.993	1<br>Lperr10g15590.1 0.416 0.985 0.569 0.07 0.989<br>Lperr10g15600.1 0.001 0.389 0.388 0.003 0.979	1<br>AT1G32080.1 0.42 0.984 0.564 0.188 1<br>AT2G32260.1 0.035 0.375 0.34 0.078 0.825
Oglab11g01340.2	1<br>Oglab11g01340.2 0.004 1 0.996 0.004 1	0	1<br>Osjap11g01730.2 0.004 1 0.996 0.004 1	0	1<br>Orufi11g01440.2 0.004 1 0.996 0.004 1	0	1<br>Opunc11g01300.1 0.004 0.989 0.985 0.309 1	0	0	0
	0<br>Oglab11g01340.3 0.442 0.925 0.483 0.004 0.929	1<br>Obart12g01240.1 0.494 0.993 0.499 0.002 0.566<br>Obart12g01250.1 0.004 0.438 0.434 0.57 0.939	0<br>Osjap11g01730.3 0.461 1 0.539 0.004 1	1<br>Osind11g01160.1 0.449 1 0.551 0.004 1<br>Osind12g01910.1 0.004 0.438 0.434 0.57 0.939	1<br>Orufi12g01510.1 0.446 1 0.554 0.007 1<br>Orufi12g01520.1 0.004 0.438 0.434 0.663 0.953	1<br>Olong_020639-R1 0.449 1 0.551 0.014 1<br>Olong_025830-R1 0.004 0.438 0.434 0.502 0.93	1<br>Opunc11g01300.2 0.004 0.438 0.434 0.538 0.935<br>Opunc12g01310.1 0.453 0.955 0.502 0.032 0.866	0<br>Obrac11g03920.2 0.004 0.446 0.442 0.631 0.971	0<br>Lperr11g01230.1 0.648 0.858 0.21 0.245 0.765<br>Lperr12g01420.1 0.004 0.438 0.434 0.547 0.936	0<br>AT1G04590.2 0.004 0.625 0.621 0.534 0.997
Oglab11g01510.4	1<br>Oglab11g01510.1 0.002 1 0.998 0.14 1<br>Oglab11g01510.2 0.002 1 0.998 0.202 1<br>Oglab11g01510.3 0.002 1 0.998 0.203 1<br>Oglab11g01510.4 0.002 1 0.998 0.002 1<br>Oglab11g01510.5 0.002 1 0.998 0.002 1	1<br>Obart11g01710.1 0.002 0.994 0.992 0.104 0.922<br>Obart11g01710.2 0.002 0.994 0.992 0.106 0.921<br>Obart11g01710.3 0.002 0.994 0.992 0.115 0.914<br>Obart11g01710.4 0.002 0.994 0.992 0.117 0.913	1<br>Osjap11g01890.1 0.002 0.989 0.987 0.147 0.988<br>Osjap11g01890.2 0.002 1 0.998 0.143 1<br>Osjap11g01890.3 0.002 0.989 0.987 0.13 0.988<br>Osjap11g01890.4 0.002 0.989 0.987 0.149 0.988<br>Osjap11g01890.5 0.002 0.989 0.987 0.142 0.988<br>Osjap11g01890.6 0.002 1 0.998 0.146 1<br>Osjap11g01890.7 0.002 0.989 0.987 0.201 0.983	0	0	0	1<br>Opunc12g01520.7 0.002 0.989 0.987 0.23 0.981	0	1<br>Lperr12g01560.1 0.002 0.989 0.987 0.13 0.989	0
	1<br>Oglab12g01390.1 0.434 0.989 0.555 0.003 0.961<br>Oglab12g01400.2 0.002 0.408 0.406 0.291 0.961	1<br>Obart03g06450.1 0.005 0.415 0.41 0.359 1<br>Obart12g01440.2 0.434 0.989 0.555 0.003 0.961	1<br>Osjap12g01820.1 0.434 0.989 0.555 0.003 0.961<br>Osjap12g01870.1 0.002 0.408 0.406 0.341 0.93	1<br>Osind02g25820.1 0.434 0.989 0.555 0.003 0.961<br>Osind03g06850.1 0.005 0.415 0.41 0.359 1	1<br>Orufi03g27580.1 0.002 0.431 0.429 0.305 0.953<br>Orufi11g01580.1 0.434 0.989 0.555 0.003 0.961	0<br>Olong_024204-R1 0.002 0.409 0.407 0.36 0.998<br>Olong_028389-R1 0.659 0.989 0.33 0.008 0.936	1<br>Opunc11g01470.1 0.434 0.989 0.555 0.003 0.961<br>Opunc12g01520.9 0.002 0.397 0.395 0.371 0.992	1<br>Obrac03g18430.1 0.002 0.418 0.416 0.331 0.992<br>Obrac12g01300.1 0.434 0.989 0.555 0.003 0.961	1<br>Lperr03g19970.1 0.002 0.418 0.416 0.335 0.98<br>Lperr11g01360.1 0.434 0.989 0.555 0.003 0.962	1<br>AT1G33090.1 0.003 0.432 0.429 0.34 1<br>AT5G21060.2 0.446 0.989 0.543 0.011 0.968
Oglab11g01510.5	1<br>Oglab11g01510.1 0.002 1 0.998 0.14 1<br>Oglab11g01510.2 0.002 1 0.998 0.202 1<br>Oglab11g01510.3 0.002 1 0.998 0.203 1<br>Oglab11g01510.4 0.002 1 0.998 0.002 1<br>Oglab11g01510.5 0.002 1 0.998 0.002 1	1<br>Obart11g01710.1 0.002 0.994 0.992 0.104 0.922<br>Obart11g01710.2 0.002 0.994 0.992 0.106 0.921<br>Obart11g01710.3 0.002 0.994 0.992 0.115 0.914<br>Obart11g01710.4 0.002 0.994 0.992 0.117 0.913	1<br>Osjap11g01890.1 0.002 0.989 0.987 0.147 0.988<br>Osjap11g01890.2 0.002 1 0.998 0.143 1<br>Osjap11g01890.3 0.002 0.989 0.987 0.13 0.988<br>Osjap11g01890.4 0.002 0.989 0.987 0.149 0.988<br>Osjap11g01890.5 0.002 0.989 0.987 0.142 0.988<br>Osjap11g01890.6 0.002 1 0.998 0.146 1<br>Osjap11g01890.7 0.002 0.989 0.987 0.201 0.983	0	0	0	1<br>Opunc12g01520.7 0.002 0.989 0.987 0.23 0.981	0	1<br>Lperr12g01560.1 0.002 0.989 0.987 0.13 0.989	0
	1<br>Oglab12g01390.1 0.43 0.989 0.559 0.003 0.961<br>Oglab12g01400.2 0.002 0.404 0.402 0.291 0.961	1<br>Obart03g06450.1 0.005 0.412 0.407 0.359 1<br>Obart12g01440.2 0.43 0.989 0.559 0.003 0.961	1<br>Osjap12g01820.1 0.43 0.989 0.559 0.003 0.961<br>Osjap12g01870.1 0.002 0.404 0.402 0.341 0.93	1<br>Osind02g25820.1 0.43 0.989 0.559 0.003 0.961<br>Osind03g06850.1 0.005 0.412 0.407 0.359 1	1<br>Orufi03g24390.1 0.002 0.415 0.413 0.36 0.979<br>Orufi11g01580.1 0.43 0.989 0.559 0.003 0.961	0<br>Olong_024204-R1 0.002 0.406 0.404 0.36 0.998<br>Olong_028389-R1 0.657 0.989 0.332 0.008 0.936	1<br>Opunc11g01470.1 0.43 0.989 0.559 0.003 0.961<br>Opunc12g01520.9 0.002 0.394 0.392 0.371 0.992	1<br>Obrac03g18430.1 0.002 0.415 0.413 0.331 0.992<br>Obrac12g01300.1 0.43 0.989 0.559 0.003 0.961	1<br>Lperr03g05800.1 0.005 0.412 0.407 0.38 1<br>Lperr11g01360.1 0.43 0.989 0.559 0.003 0.962	1<br>AT1G33090.1 0.003 0.429 0.426 0.34 1<br>AT5G21060.2 0.442 0.989 0.547 0.011 0.968
Oglab11g02200.1	1<br>Oglab11g02200.1 0.002 1 0.998 0.002 1	1<br>Obart12g02080.1 0.023 1 0.977 0.002 1	1<br>Osjap11g02640.1 0.002 1 0.998 0.002 1	1<br>Osind11g01990.1 0.002 1 0.998 0.002 1	1<br>Orufi11g02320.1 0.002 1 0.998 0.003 1	0	0	0	0	0

	Oglab12g02130.1 0.002 1 0.998 0.002 1		Osjap12g02610.1 0.002 1 0.998 0.002 1	Osind12g02780.1 0.002 1 0.998 0.002 1	Orufi12g02480.1 0.002 1 0.998 0.002 1					
	1<br>Oglab07g03810.1 0.032 0.657 0.625 0.019 0.987<br>Oglab12g09880.1 0.66 0.951 0.291 0.051 0.385	0<br>Obart11g02350.1 0.021 0.657 0.636 0.004 0.993	1<br>Osjap07g04360.1 0.032 0.657 0.625 0.018 0.925<br>Osjap12g12870.1 0.66 0.951 0.291 0.055 0.417	1<br>Osind11g02010.1 0.013 0.657 0.644 0.001 0.994<br>Osind12g10020.1 0.66 0.951 0.291 0.055 0.417	1<br>Orufi07g03650.1 0.032 0.657 0.625 0.019 0.976<br>Orufi12g10750.1 0.658 0.951 0.293 0.045 0.364	1<br>Olong_012584-R1 0.064 0.611 0.547 0.002 0.965<br>Olong_017316-R1 0.658 0.951 0.293 0.045 0.365	1<br>Opunc11g02190.1 0.002 0.657 0.655 0.003 0.989<br>Opunc12g08740.1 0.658 0.951 0.293 0.04 0.324	1<br>Obrac05g10860.1 0.049 0.657 0.608 0.097 0.987<br>Obrac12g07170.1 0.658 0.942 0.284 0.071 0.57	1<br>Lperr12g02190.1 0.002 0.657 0.655 0.003 0.989<br>Lperr12g08370.1 0.657 0.951 0.294 0.047 0.391	0<br>AT5G27320.1 0.013 0.632 0.619 0.003 0.98
Oglab11g05440.1	1<br>Oglab11g05440.1 0.001 1 0.999 0.001 1	1<br>Obart11g05910.1 0.001 0.875 0.874 0.05 1	1<br>Osjap11g06710.1 0.001 0.998 0.997 0.067 0.993<br>Osjap11g06720.1 0.001 0.998 0.997 0.054 0.977	1<br>Osind09g09040.1 0.001 0.998 0.997 0.001 0.995	1<br>Orufi11g05930.1 0.001 0.998 0.997 0.09 0.992<br>Orufi11g05940.1 0.001 1 0.999 0.002 0.995	0	0	0	0	0
	1<br>Oglab08g10820.1 0.526 0.955 0.429 0.015 0.867<br>Oglab11g05700.1 0.001 0.51 0.509 0.001 0.966	1<br>Obart01g20930.1 0.525 0.992 0.467 0.006 0.498<br>Obart11g05930.1 0.001 0.502 0.501 0.002 0.998	1<br>Osjap01g24690.1 0.525 0.992 0.467 0.014 0.934<br>Osjap11g06740.8 0.024 0.509 0.485 0.013 0.579	1<br>Osind09g09050.1 0.001 0.502 0.501 0.104 0.994<br>Osind11g05650.1 0.521 0.998 0.477 0.002 0.989	1<br>Orufi11g05890.1 0.521 0.998 0.477 0.003 0.984<br>Orufi11g05900.2 0.001 0.509 0.508 0.002 0.991	1<br>Olong_005786-R3 0.007 0.511 0.504 0.006 0.948<br>Olong_005787-R1 0.521 0.998 0.477 0.002 0.989	0<br>Opunc11g05960.1 0.443 0.998 0.555 0.002 0.991	1<br>Obrac01g15800.1 0.52 0.992 0.472 0.004 0.915<br>Obrac11g03930.1 0.001 0.504 0.503 0.002 0.941	1<br>Lperr01g17850.1 0.521 0.992 0.471 0.002 0.973<br>Lperr11g05360.1 0.001 0.502 0.501 0.044 0.995	0<br>AT4G38130.1 0.521 0.955 0.434 0.002 0.782
Oglab11g06530.1	1<br>Oglab11g06530.1 0.01 1 0.99 0.01 1	0	0	1<br>Osind11g06680.1 0.01 0.942 0.932 0.004 0.411	1<br>Orufi11g07410.1 0.01 0.942 0.932 0.005 0.516	0	0	0	0	0
	1<br>Oglab01g35920.1 0.01 0.612 0.602 0.002 0.144<br>Oglab08g00300.1 0.612 0.981 0.369 0.487 0.537	1<br>Obart01g41090.1 0.01 0.612 0.602 0.002 0.144<br>Obart04g30900.2 0.612 0.981 0.369 0.451 0.513	1<br>Osjap01g46770.1 0.01 0.612 0.602 0.002 0.144<br>Osjap08g00940.1 0.612 0.981 0.369 0.487 0.537	1<br>Osind01g47980.1 0.01 0.612 0.602 0.002 0.142<br>Osind04g02960.1 0.612 0.981 0.369 0.501 0.562	1<br>Orufi01g44430.1 0.01 0.612 0.602 0.002 0.14<br>Orufi08g00930.1 0.612 0.981 0.369 0.487 0.537	0<br>Olong_019421-R2 0.398 0.981 0.583 0.431 0.523	1<br>Opunc01g39900.1 0.01 0.612 0.602 0.002 0.159<br>Opunc04g28500.1 0.612 0.981 0.369 0.467 0.523	0<br>Obrac12g04550.1 0.398 0.981 0.583 0.719 0.769	0<br>Lperr12g05120.1 0.388 0.981 0.593 0.461 0.561	0<br>AT1G57700.1 0.33 0.981 0.651 0.422 0.527
Oglab11g14350.2	1<br>Oglab11g14350.1 0.001 0.967 0.966 0.001 1<br>Oglab11g14350.2 0.001 1 0.999 0.001 1	1<br>Obart11g16070.1 0.001 1 0.999 0.013 1	0	0	0	0	0	0	0	0
	1<br>Oglab02g07220.2 0.597 1 0.403 0.003 1<br>Oglab11g18510.1 0.032 0.552 0.52 0.029 1	1<br>Obart02g07160.1 0.597 1 0.403 0.002 1<br>Obart08g10830.1 0.036 0.55 0.514 0.004 0.944	1<br>Osjap02g07570.1 0.597 1 0.403 0.002 1<br>Osjap11g18290.1 0.001 0.531 0.53 0.514 0.989	1<br>Osind11g13470.1 0.001 0.496 0.495 0.416 0.992<br>Osind11g13490.1 0.595 1 0.405 0.081 1	1<br>Orufi11g17130.1 0.001 0.531 0.53 0.522 0.989<br>Orufi11g17160.1 0.597 1 0.403 0.002 1	1<br>Olong_012209-R1 0.597 1 0.403 0.002 1<br>Olong_021538-R1 0.028 0.559 0.531 0.006 0.801	1<br>Opunc11g13170.1 0.597 1 0.403 0.002 1<br>Opunc11g17530.1 0.026 0.545 0.519 0.009 0.97	1<br>Obrac02g05260.1 0.597 1 0.403 0.002 1<br>Obrac10g05860.1 0.038 0.536 0.498 0.005 0.973	1<br>Lperr11g17450.1 0.034 0.537 0.503 0.003 0.875<br>Lperr12g06590.1 0.568 1 0.432 0.061 1	0<br>AT2G44350.2 0.597 1 0.403 0.002 0.998<br>AT5G54820.1 0.038 0.401 0.363 0.015 0.788
Oglab11g18520.1	1<br>Oglab11g18520.1 0.001 1 0.999 0.001 1	0	0	0	1<br>Orufi11g22790.1 0.013 0.977 0.964 0.006 0.971	0	0	0	0	0
	1<br>Oglab11g18510.1 0.013 0.512 0.499 0.037 0.942<br>Oglab11g18550.1 0.54 1 0.46 0.002 1	1<br>Obart11g20950.1 0.013 0.512 0.499 0.003 0.945<br>Obart11g20980.1 0.54 1 0.46 0.003 1	1<br>Osjap11g24530.1 0.013 0.536 0.523 0.038 0.993<br>Osjap11g24540.1 0.54 1 0.46 0.002 1	1<br>Osind06g03750.1 0.54 1 0.46 0.002 1<br>Osind10g01290.1 0.017 0.501 0.484 0.035 0.99	1<br>Orufi04g30270.1 0.54 0.992 0.452 0.002 0.984<br>Orufi11g22780.1 0.009 0.498 0.489 0.018 0.881	1<br>Olong_021538-R1 0.013 0.514 0.501 0.011 0.957<br>Olong_027904-R1 0.579 0.989 0.41 0.108 0.991	1<br>Opunc03g06070.1 0.54 0.992 0.452 0.002 0.98<br>Opunc11g13180.1 0.013 0.498 0.485 0.169 0.909	1<br>Obrac03g05030.1 0.54 0.992 0.452 0.003 0.967<br>Obrac11g09310.1 0.005 0.498 0.493 0.025 0.932	1<br>Lperr09g14070.1 0.54 0.991 0.451 0.007 1<br>Lperr11g12540.1 0.005 0.496 0.491 0.013 0.892	0<br>AT5G23940.1 0.547 0.991 0.444 0.012 0.921
Oglab11g20100.2	1<br>Oglab11g20100.1 0.001 0.998 0.997 0 0.998<br>Oglab11g20100.2 0.001 1 0.999 0.001 1	1<br>Obart11g23070.1 0.001 1 0.999 0.022 1	0	0	0	0	1<br>Opunc11g19450.1 0.001 0.979 0.978 0.001 0.995	0	0	0
	1<br>Oglab02g25180.1 0.548 0.976 0.428 0.006 0.938<br>Oglab05g19280.1 0.001 0.537 0.536 0.001 0.81	1<br>Obart02g27080.1 0.548 0.976 0.428 0.006 0.938<br>Obart11g22940.1 0.001 0.536 0.535 0.146 0.791	0<br>Osjap05g22990.2 0.001 0.544 0.543 0.001 0.886	1<br>Osind02g30490.1 0.548 0.98 0.432 0.006 1<br>Osind05g23630.1 0.001 0.544 0.543 0.001 0.886	1<br>Orufi02g28510.2 0.548 0.98 0.432 0.007 1<br>Orufi05g21610.1 0.001 0.536 0.535 0.001 0.97	1<br>Olong_005582-R1 0.546 0.986 0.44 0.145 0.995<br>Olong_026752-R1 0.001 0.539 0.538 0.001 0.973	1<br>Opunc02g24700.1 0.553 0.98 0.427 0.003 1<br>Opunc05g18050.1 0.001 0.536 0.535 0.001 0.848	1<br>Obrac02g19610.1 0.547 0.976 0.429 0.001 0.983<br>Obrac05g13790.3 0.001 0.53 0.529 0.092 0.901	0<br>Lperr11g19460.1 0.001 0.535 0.534 0.005 0.813	0<br>AT2G45910.1 0.585 0.976 0.391 0.054 0.994
Oglab12g00050.1	1	0	0	0	0	0	0	0	0	0

	Oglab12g00050.1 0.003 1 0.997 0.003 1									
	1<br>Oglab01g30880.1 0.335 0.911 0.576 0.03 0.464<br>Oglab11g00420.3 0.003 0.298 0.295 0.002 0.735	1<br>Obart12g00130.1 0.325 0.945 0.62 0.002 0.509<br>Obart12g00140.5 0.003 0.298 0.295 0.004 0.46	1<br>Osjap11g00430.1 0.003 0.298 0.295 0.004 0.425<br>Osjap12g00360.1 0.325 0.945 0.62 0.002 0.506	1<br>Osind12g00440.1 0.325 0.945 0.62 0.002 0.506<br>Osind12g00580.1 0.003 0.298 0.295 0.004 0.425	0<br>Orufi12g00380.1 0.325 0.945 0.62 0.002 0.506	1<br>Olong_014020-R1 0.359 0.937 0.578 0.055 0.353<br>Olong_016703-R1 0.003 0.298 0.295 0.004 0.423	1<br>Opunc05g17450.1 0.335 0.927 0.592 0.029 0.496<br>Opunc11g00340.1 0.003 0.291 0.288 0.007 0.696	0<br>Obrac05g13350.2 0.144 0.911 0.767 0.033 0.562	1<br>Lperr11g00380.1 0.003 0.298 0.295 0.004 0.481<br>Lperr12g00140.1 0.33 0.982 0.652 0.016 0.62	1<br>AT1G25260.1 0.003 0.293 0.29 0.004 0.409<br>AT3G24290.1 0.359 0.937 0.578 0.093 0.496
Oglab12g00800.1	1<br>Oglab12g00800.1 0.001 1 0.999 0.001 1	1<br>Obart11g01060.1 0.001 1 0.999 0.001 1	1<br>Osjap11g01220.1 0.001 1 0.999 0.001 1<br>Osjap12g01140.1 0.001 1 0.999 0.001 1	1<br>Osind11g00690.1 0.001 0.956 0.955 0.001 0.999<br>Osind12g01290.1 0.001 1 0.999 0.001 1	1<br>Orufi11g01000.1 0.001 1 0.999 0.001 1<br>Orufi12g01130.1 0.001 1 0.999 0.001 1	0	1<br>Opunc12g00940.1 0.001 1 0.999 0.001 1	0	0	0
	1<br>Oglab03g12120.1 0.49 0.983 0.493 0.121 0.633<br>Oglab07g22980.1 0.006 0.393 0.387 0.007 0.933	1<br>Obart03g12120.1 0.49 0.983 0.493 0.121 0.633<br>Obart12g00790.1 0.001 0.393 0.392 0.002 0.942	1<br>Osjap03g13100.1 0.49 0.983 0.493 0.097 0.622<br>Osjap07g30390.1 0.009 0.393 0.384 0.013 0.938	1<br>Osind03g12930.1 0.49 0.983 0.493 0.121 0.633<br>Osind07g27070.1 0.006 0.393 0.387 0.006 0.939	1<br>Orufi03g17220.1 0.496 1 0.504 0.027 1<br>Orufi07g27420.1 0.009 0.393 0.384 0.013 0.939	1<br>Olong_007715-R1 0.493 1 0.507 0.007 1<br>Olong_008211-R1 0.006 0.393 0.387 0.006 0.939	1<br>Opunc03g11680.1 0.49 1 0.51 0.148 1<br>Opunc11g00920.1 0.001 0.393 0.392 0.142 0.945	1<br>Obrac03g09720.2 0.49 1 0.51 0.142 1<br>Obrac06g16520.1 0.02 0.411 0.391 0.048 0.978	1<br>Lperr03g10720.1 0.49 0.996 0.506 0.148 0.954<br>Lperr11g00920.1 0.001 0.393 0.392 0.002 0.942	1<br>AT3G46740.1 0.493 1 0.507 0.161 1<br>AT4G20110.2 0.001 0.393 0.392 0.002 0.939
Oglab12g01430.1	1<br>Oglab12g01430.1 0.001 1 0.999 0.001 1	1<br>Obart12g01480.1 0.001 0.996 0.995 0.001 0.986	0	0	0	0	0	0	0	0
	1<br>Oglab01g17070.1 0.001 0.479 0.478 0.05 0.856<br>Oglab12g01740.1 0.587 1 0.413 0.007 0.993	1<br>Obart01g05330.1 0.123 0.598 0.475 0.101 0.965<br>Obart02g36960.1 0.606 0.996 0.39 0.27 0.993	1<br>Osjap01g23560.1 0.001 0.558 0.557 0.04 0.822<br>Osjap12g02170.3 0.587 1 0.413 0.007 1	1<br>Osind12g02430.1 0.001 0.587 0.586 0.002 0.95<br>Osind12g02460.1 0.587 1 0.413 0.006 0.994	1<br>Orufi01g22500.1 0.001 0.558 0.557 0.049 0.912<br>Orufi12g02140.1 0.587 1 0.413 0.007 0.993	1<br>Olong_015804-R1 0.001 0.59 0.589 0.002 0.976<br>Olong_027205-R1 0.592 0.996 0.404 0.004 0.84	1<br>Opunc02g34070.1 0.595 0.996 0.401 0.218 0.996<br>Opunc08g00050.1 0.001 0.479 0.478 0.03 0.777	1<br>Obrac03g08440.1 0.136 0.595 0.459 0.179 0.989<br>Obrac12g01540.1 0.606 0.996 0.39 0.017 0.983	0<br>Lperr02g30420.7 0.608 0.996 0.388 0.02 0.984<br>Lperr03g07390.1 0.174 0.562 0.388 0.175 0.868	0<br>AT2G02250.1 0.537 0.996 0.459 0.003 0.974
Oglab12g01890.1	1<br>Oglab12g01890.1 0.002 1 0.998 0.002 1	1<br>Obart12g01910.1 0.002 0.974 0.972 0.002 0.938	0	0	0	0	1<br>Opunc11g02000.1 0.002 1 0.998 0.002 0.907<br>Opunc12g01920.1 0.002 1 0.998 0.002 0.978	1<br>Obrac11g01380.1 0.023 0.972 0.949 0.036 0.95	0	0
	1<br>Oglab07g23480.1 0.737 0.998 0.261 0.002 0.386<br>Oglab11g01930.1 0.013 0.718 0.705 0.051 0.947	1<br>Obart07g26900.1 0.737 0.998 0.261 0.002 0.386<br>Obart11g02130.1 0.013 0.718 0.705 0.051 0.947	1<br>Osjap11g02310.2 0.739 1 0.261 0.006 1<br>Osjap12g02360.1 0.002 0.718 0.716 0.002 0.945	1<br>Osind07g27540.1 0.737 0.998 0.261 0.002 0.386<br>Osind12g02620.1 0.002 0.718 0.716 0.002 0.945	1<br>Orufi11g02050.1 0.739 1 0.261 0.006 1<br>Orufi12g02190.1 0.023 0.622 0.599 0.159 1	1<br>Olong_013437-R2 0.739 0.997 0.258 0.007 0.809<br>Olong_019069-R2 0.002 0.718 0.716 0.002 0.945	0<br>Opunc07g25270.1 0.737 0.998 0.261 0.003 0.43	1<br>Obrac01g08350.1 0.732 0.977 0.245 0.007 1<br>Obrac12g01700.2 0.002 0.718 0.716 0.002 0.948	1<br>Lperr03g13480.1 0.732 0.977 0.245 0.007 1<br>Lperr07g20930.1 0.026 0.718 0.692 0.09 0.956	1<br>AT2G19540.1 0.023 0.718 0.695 0.062 0.947<br>AT3G43810.1 0.732 0.977 0.245 0.007 1
Oglab12g02130.1	1<br>Oglab11g02200.1 0.002 1 0.998 0.002 1<br>Oglab12g02130.1 0.002 1 0.998 0.002 1	1<br>Obart12g02080.1 0.022 1 0.978 0.002 1	1<br>Osjap11g02640.1 0.002 1 0.998 0.002 1<br>Osjap12g02610.1 0.002 1 0.998 0.002 1	1<br>Osind11g01990.1 0.002 1 0.998 0.002 1<br>Osind12g02780.1 0.002 1 0.998 0.002 1	1<br>Orufi11g02320.1 0.002 1 0.998 0.003 1<br>Orufi12g02480.1 0.002 1 0.998 0.002 1	0	0	0	0	0
	1<br>Oglab07g03810.1 0.031 0.64 0.609 0.019 0.987<br>Oglab12g09880.1 0.651 0.953 0.302 0.02 0.385	0<br>Obart11g02350.1 0.02 0.64 0.62 0.004 0.993	1<br>Osjap07g04360.1 0.031 0.64 0.609 0.018 0.925<br>Osjap12g12870.1 0.651 0.953 0.302 0.022 0.417	1<br>Osind11g02010.1 0.013 0.64 0.627 0.001 0.994<br>Osind12g10020.1 0.651 0.953 0.302 0.022 0.417	0<br>Orufi07g03640.1 0.047 0.655 0.608 0.028 0.997	1<br>Olong_012584-R1 0.061 0.597 0.536 0.002 0.965<br>Olong_017316-R1 0.651 0.953 0.302 0.017 0.365	1<br>Opunc11g02190.1 0.002 0.64 0.638 0.003 0.989<br>Opunc12g08740.1 0.651 0.953 0.302 0.015 0.324	1<br>Obrac05g10860.1 0.047 0.64 0.593 0.097 0.987<br>Obrac12g07170.1 0.651 0.944 0.293 0.029 0.57	1<br>Lperr12g02190.1 0.002 0.64 0.638 0.003 0.989<br>Lperr12g08380.1 0.644 0.987 0.343 0.01 0.488	0<br>AT5G16080.1 0.047 0.644 0.597 0.081 0.994
Oglab12g02960.1	1<br>Oglab12g02960.1 0.001 1 0.999 0.001 1	0	0	0	0	0	1<br>Opunc12g02670.1 0.001 1 0.999 0.001 1	0	0	0
	1<br>Oglab06g24980.2 0.005 0.757 0.752 0.153 0.821<br>Oglab11g03020.1 0.782 1 0.218 0.005 1	1<br>Obart06g27500.1 0.005 0.757 0.752 0.152 0.823<br>Obart12g02850.1 0.782 1 0.218 0.008 1	1<br>Osjap11g03380.1 0.782 1 0.218 0.005 1<br>Osjap12g03480.1 0.001 0.738 0.737 0.002 0.965	1<br>Osind06g31110.1 0.005 0.757 0.752 0.152 0.823<br>Osind12g03520.1 0.782 1 0.218 0.008 1	1<br>Orufi12g03250.1 0.782 1 0.218 0.008 1<br>Orufi12g03260.1 0.001 0.738 0.737 0.002 0.965	1<br>Olong_004443-R1 0.782 1 0.218 0.01 1<br>Olong_023554-R1 0.005 0.757 0.752 0.125 0.818	1<br>Opunc06g25170.3 0.005 0.757 0.752 0.09 0.809<br>Opunc11g02740.1 0.782 1 0.218 0.005 1	1<br>Obrac06g19580.3 0.005 0.757 0.752 0.149 0.822<br>Obrac11g01880.1 0.782 0.997 0.215 0.01 0.99	1<br>Lperr01g19770.1 0.782 1 0.218 0.008 1<br>Lperr03g35610.1 0.012 0.763 0.751 0.026 0.955	0<br>AT5G04550.1 0.009 0.731 0.722 0.032 0.97
Oglab12g03070.1	1	1	0	1	1	1	0	0	0	0

	Oglab12g03070.1 0.001 1 0.999 0.001 1	Obart12g02950.1 0.001 1 0.999 0.001 1		Osind12g03620.1 0.001 0.999 0.998 0.029 0.961	Orufi12g03350.1 0.012 0.999 0.987 0.044 0.966<br>Orufi12g03350.2 0.012 0.999 0.987 0.043 0.966	Olong_000549-R1 0.012 0.999 0.987 0.011 0.967				
	1<br>Oglab10g13340.3 0.546 0.99 0.444 0.213 0.951<br>Oglab12g02020.1 0.002 0.437 0.435 0.001 0.388	0<br>Obart11g02240.1 0.198 0.437 0.239 0.014 0.272<br>Obart11g21270.1 0.479 0.984 0.505 0.138 0.958	1<br>Osjap10g20010.1 0.483 0.99 0.507 0.022 0.946<br>Osjap12g02500.1 0.002 0.437 0.435 0.001 0.388	1<br>Osind01g01580.1 0.477 0.984 0.507 0.171 0.96<br>Osind12g02670.1 0.002 0.437 0.435 0.001 0.396	1<br>Orufi04g25720.1 0.483 0.867 0.384 0.002 0.207<br>Orufi11g02210.1 0.016 0.437 0.421 0.015 0.492	0<br>Olong_010277-R4 0.473 0.867 0.394 0.001 0.93<br>Olong_027241-R1 0.047 0.287 0.24 0.003 0.866	1<br>Opunc11g17890.2 0.476 0.984 0.508 0.189 0.952<br>Opunc12g02040.1 0.012 0.437 0.425 0.009 0.402	1<br>Obrac11g01490.1 0.002 0.437 0.435 0.029 0.403<br>Obrac11g12600.2 0.534 0.984 0.45 0.046 0.748	0<br>Lperr11g01950.1 0.012 0.235 0.223 0.015 0.297<br>Lperr11g17840.1 0.486 0.984 0.498 0.046 0.945	1<br>AT1G65810.1 0.483 0.867 0.384 0.021 0.596<br>AT2G26780.1 0.017 0.433 0.416 0.011 0.388
Oglab12g06650.1	1<br>Oglab12g06650.1 0.001 1 0.999 0.001 1	0	0	0	0	0	1<br>Opunc12g06220.1 0.01 1 0.99 0.004 1	0	0	0
	0<br>Oglab01g38670.1 0.784 0.993 0.209 0.076 0.968	1<br>Obart12g06540.1 0.795 1 0.205 0.035 1<br>Obart12g06550.1 0.058 0.795 0.737 0.09 0.962	1<br>Osjap01g49850.1 0.784 0.993 0.209 0.027 0.337<br>Osjap12g08640.3 0.026 0.752 0.726 0.12 0.985	1<br>Osind01g51390.1 0.784 0.993 0.209 0.076 0.968<br>Osind12g07610.1 0.026 0.752 0.726 0.037 0.985	1<br>Orufi01g47370.1 0.784 0.993 0.209 0.029 0.359<br>Orufi12g07360.1 0.026 0.752 0.726 0.037 0.985	1<br>Olong_017083-R1 0.793 0.999 0.206 0.373 1<br>Olong_026612-R3 0.078 0.752 0.674 0.067 0.981	0<br>Opunc11g02000.1 0.727 0.988 0.261 0.59 0.878	1<br>Obrac04g10690.1 0.793 0.999 0.206 0.375 1<br>Obrac12g04840.4 0.013 0.752 0.739 0.014 0.986	1<br>Lperr04g13760.1 0.793 0.999 0.206 0.321 1<br>Lperr12g05750.1 0.001 0.752 0.751 0.002 0.986	1<br>AT2G20210.1 0.082 0.749 0.667 0.053 0.98<br>AT3G07490.1 0.789 0.999 0.21 0.007 0.98
Oglab12g09470.1	1<br>Oglab12g09470.1 0.001 1 0.999 0.001 1	1<br>Obart12g09410.1 0.001 1 0.999 0.001 1	1<br>Osjap12g12320.1 0.001 1 0.999 0.001 1	1<br>Osind12g09530.1 0.001 1 0.999 0.001 1	1<br>Orufi12g10460.1 0.001 1 0.999 0.001 1	0	0	0	0	0
	1<br>Oglab01g34040.1 0.489 0.998 0.509 0.203 0.995<br>Oglab03g07280.1 0.001 0.295 0.294 0.004 0.981	1<br>Obart01g39260.3 0.456 0.998 0.542 0.041 0.994<br>Obart03g07210.1 0.001 0.295 0.294 0.004 0.981	1<br>Osjap01g44780.2 0.44 0.998 0.558 0.095 0.994<br>Osjap03g07600.1 0.001 0.295 0.294 0.004 0.981	1<br>Osind01g46040.1 0.44 0.998 0.558 0.151 0.995<br>Osind03g07670.1 0.001 0.295 0.294 0.004 0.981	1<br>Orufi01g42650.3 0.44 0.998 0.558 0.195 0.995<br>Orufi03g07180.1 0.001 0.295 0.294 0.004 0.981	1<br>Olong_021912-R1 0.457 1 0.543 0.099 1<br>Olong_021913-R1 0.001 0.296 0.295 0.004 0.988	1<br>Opunc12g08570.1 0.326 1 0.674 0.052 1<br>Opunc12g08580.1 0.001 0.296 0.295 0.004 0.988	1<br>Obrac03g05880.1 0.001 0.296 0.295 0.004 0.988<br>Obrac12g06500.1 0.461 1 0.539 0.103 1	1<br>Lperr01g33810.2 0.456 0.998 0.542 0.002 0.994<br>Lperr12g08300.1 0.001 0.296 0.295 0.004 0.985	0<br>AT4G34670.1 0.001 0.29 0.289 0.004 0.966<br>AT5G67540.2 0.489 0.993 0.504 0.053 1
Oglab12g11360.1	1<br>Oglab12g11360.1 0.005 1 0.995 0.005 1	0	0	0	0	1<br>Olong_021020-R1 0.005 1 0.995 0.005 1	0	0	0	0
	1<br>Oglab07g16860.1 0.491 1 0.509 0.207 1<br>Oglab12g09200.1 0.014 0.326 0.312 0.787 0.962	0<br>Obart07g18590.1 0.491 1 0.509 0.207 1	1<br>Osjap12g11960.1 0.014 0.335 0.321 0.341 0.911<br>Osjap12g14980.1 0.45 1 0.55 0.016 1	1<br>Osind10g17000.1 0.073 0.408 0.335 0.853 0.978<br>Osind12g08720.1 0.491 1 0.509 0.152 1	1<br>Orufi05g05820.1 0.491 1 0.509 0.152 1<br>Orufi12g10130.1 0.014 0.413 0.399 0.304 0.913	1<br>Olong_015025-R1 0.486 1 0.514 0.111 1<br>Olong_019160-R2 0.028 0.326 0.298 0.39 0.949	0<br>Opunc02g32830.1 0.349 1 0.651 0.039 1	0<br>Obrac08g07610.1 0.491 0.821 0.33 0.157 0.971	0<br>Lperr05g07610.1 0.422 0.775 0.353 0.328 0.867	0
Oglab12g13410.1	1<br>Oglab12g13410.1 0.005 1 0.995 0.005 1	0	0	0	0	0	0	0	0	0
	0<br>Oglab05g23160.1 0.362 1 0.638 0.119 0.225	1<br>Obart01g26870.1 0.362 1 0.638 0.347 0.552<br>Obart04g05090.1 0.005 0.357 0.352 0.664 0.96	1<br>Osjap01g31140.1 0.362 1 0.638 0.327 0.539<br>Osjap04g06490.1 0.005 0.221 0.216 0.706 0.899	1<br>Osind01g32600.1 0.362 1 0.638 0.269 0.498<br>Osind11g12290.1 0.005 0.221 0.216 0.706 0.899	1<br>Orufi01g29880.1 0.362 1 0.638 0.323 0.536<br>Orufi10g18640.1 0.005 0.221 0.216 0.014 0.662	0<br>Olong_000367-R1 0.362 1 0.638 0.042 0.344	1<br>Opunc05g22640.1 0.362 1 0.638 0.127 0.237<br>Opunc12g12730.1 0.005 0.249 0.244 0.421 0.963	0<br>Obrac05g17590.1 0.362 1 0.638 0.042 0.344	0<br>Lperr05g20860.1 0.362 1 0.638 0.265 0.496	0<br>ATCG00480.1 0.357 1 0.643 0.006 0.438

((((((_b1,_a2),longi),punctata),brachy),leersia),tair);

chimeric gene id	INGROUP		MIDGROUP					OUTGROUP		
	glab	barthii	jap	indica	rufi	longi	punctata	brachy	leersia	tair
Oglab01g01590.1	1<br>Oglab01g01590.1 0.001 1 0.999 0.001 1	1<br>Obart01g01710.1 0.001 0.987 0.986 0.001 0.986	0	1<br>Osind01g02220.1 0.001 0.999 0.998 0.056 0.987	0	1<br>Olong_021362-R1 0.001 0.999 0.998 0.048 0.986	1<br>Opunc01g01490.1 0.002 0.998 0.996 0.021 0.988<br>Opunc01g01490.2 0.002 0.998 0.996 0.024 0.988	0	0	0
	1	1	1	1	1	1	1	1	1	1

	Oglab01g01570.1 0.537 0.999 0.462 0.043 0.998<br>Oglab05g03370.1 0.002 0.469 0.467 0.104 0.976	Obart01g01680.1 0.537 0.999 0.462 0.046 0.998<br>Obart05g03720.1 0.002 0.469 0.467 0.104 0.976	Osjap01g02100.1 0.001 0.469 0.468 0.002 0.973<br>Osjap01g02110.1 0.511 1 0.489 0.039 1	Osind01g02230.1 0.511 0.987 0.476 0.061 0.964<br>Osind05g03930.1 0.002 0.469 0.467 0.104 0.976	Orufi01g01810.1 0.001 0.469 0.468 0.002 0.973<br>Orufi01g01830.1 0.537 0.999 0.462 0.079 0.999	Olong_016471-R1 0.002 0.469 0.467 0.106 0.972<br>Olong_021361-R1 0.514 1 0.486 0.002 1	Opunc01g01490.3 0.537 0.993 0.456 0.056 0.981<br>Opunc05g03460.1 0.016 0.469 0.453 0.119 0.971	Obrac01g01340.1 0.485 0.998 0.513 0.001 0.999<br>Obrac01g17910.1 0.004 0.469 0.465 0.248 0.98	Lperr01g01640.1 0.509 0.999 0.49 0.051 0.998<br>Lperr05g03400.1 0.015 0.469 0.454 0.158 0.972	AT1G71040.1 0.526 0.999 0.473 0.021 0.998<br>AT3G48670.1 0.002 0.468 0.466 0.117 0.971
Oglab01g03790.1	1<br>Oglab01g03790.1 0.001 1 0.999 0.001 1	1<br>Obart01g04220.1 0.001 1 0.999 0.001 1	0	0	0	0	0	0	0	0
	1<br>Oglab05g04170.2 0.048 0.567 0.519 0.114 1<br>Oglab07g07650.1 0.62 0.997 0.377 0.002 0.778	1<br>Obart05g04510.1 0.036 0.567 0.531 0.012 1<br>Obart07g07920.1 0.62 0.997 0.377 0.002 0.778	1<br>Osjap01g04780.1 0.62 1 0.38 0.002 1<br>Osjap05g04880.1 0.048 0.567 0.519 0.008 1	1<br>Osind01g04880.1 0.62 1 0.38 0.002 1<br>Osind05g04760.1 0.048 0.567 0.519 0.008 1	1<br>Orufi01g04370.1 0.62 1 0.38 0.002 1<br>Orufi05g04740.1 0.048 0.567 0.519 0.008 1	1<br>Olong_014225-R1 0.044 0.563 0.519 0.001 0.984<br>Olong_014227-R1 0.62 1 0.38 0.002 1	1<br>Opunc01g03980.1 0.62 1 0.38 0.002 1<br>Opunc07g16230.1 0.042 0.556 0.514 0.014 0.972	1<br>Obrac01g03070.1 0.62 1 0.38 0.002 1<br>Obrac05g03450.2 0.039 0.567 0.528 0.003 1	1<br>Lperr01g03770.1 0.62 1 0.38 0.002 1<br>Lperr01g03780.1 0.039 0.561 0.522 0.002 0.979	1<br>AT5G01660.1 0.053 0.556 0.503 0.015 0.97<br>AT5G64190.1 0.62 0.997 0.377 0.002 0.998
Oglab01g06260.1	1<br>Oglab01g06260.1 0.001 1 0.999 0.001 1	0	0	0	0	0	0	0	0	0
	1<br>Oglab05g25190.3 0.025 0.527 0.502 0.041 0.991<br>Oglab06g15610.1 0.555 0.99 0.435 0.132 1	1<br>Obart01g07310.1 0.528 0.996 0.468 0.002 0.908<br>Obart05g27220.1 0.025 0.521 0.496 0.085 0.982	1<br>Osjap01g08460.1 0.561 0.991 0.43 0.03 0.997<br>Osjap05g30490.5 0.025 0.552 0.527 0.078 0.969	1<br>Osind01g08290.1 0.528 1 0.472 0.003 1<br>Osind05g30870.1 0.024 0.527 0.503 0.029 0.989	1<br>Orufi01g07850.1 0.021 0.536 0.515 0.039 0.998<br>Orufi06g18220.1 0.555 0.99 0.435 0.132 1	1<br>Olong_012629-R1 0.024 0.489 0.465 0.034 0.997<br>Olong_026782-R1 0.525 0.991 0.466 0.002 0.995	1<br>Opunc01g07000.1 0.53 0.977 0.447 0.032 0.987<br>Opunc05g24760.1 0.025 0.521 0.496 0.034 0.991	1<br>Obrac01g17790.1 0.024 0.519 0.495 0.078 0.962<br>Obrac05g15110.1 0.528 0.991 0.463 0.003 0.992	1<br>Lperr01g06950.1 0.561 0.995 0.434 0.078 1<br>Lperr05g22820.1 0.024 0.521 0.497 0.071 0.976	1<br>AT1G15000.1 0.024 0.531 0.507 0.07 0.977<br>AT1G28600.1 0.533 1 0.467 0.025 0.957
Oglab01g06470.1	1<br>Oglab01g06470.1 0.001 1 0.999 0.001 1	1<br>Obart01g07430.1 0.02 1 0.98 0.013 1	1<br>Osjap01g08660.1 0.001 1 0.999 0.001 1	1<br>Osind01g08490.1 0.001 1 0.999 0.001 1	1<br>Orufi01g08070.1 0.001 1 0.999 0.001 1	1<br>Olong_011567-R1 0.056 1 0.944 0.001 1	0	0	0	0
	1<br>Oglab01g21070.2 0.1 0.581 0.481 0.249 0.983<br>Oglab05g00530.1 0.647 0.997 0.35 0.146 1	1<br>Obart01g25090.1 0.1 0.581 0.481 0.219 0.982<br>Obart05g00540.1 0.647 0.997 0.35 0.146 1	1<br>Osjap01g29230.2 0.1 0.581 0.481 0.25 0.983<br>Osjap05g00560.2 0.647 0.997 0.35 0.146 1	1<br>Osind01g30690.1 0.1 0.581 0.481 0.267 0.982<br>Osind05g00540.1 0.647 0.997 0.35 0.126 1	1<br>Orufi01g27950.1 0.1 0.581 0.481 0.25 0.983<br>Orufi01g48560.2 0.647 0.98 0.333 0.085 0.984	0<br>Olong_003039-R1 0.647 0.997 0.35 0.128 1<br>Olong_003692-R2 0.139 0.581 0.442 0.244 0.979	1<br>Opunc01g07170.1 0.001 0.582 0.581 0.001 0.984<br>Opunc05g00510.2 0.647 0.997 0.35 0.15 1	1<br>Obrac01g05760.3 0.583 1 0.417 0.082 1<br>Obrac01g05770.3 0.001 0.582 0.581 0.001 0.985	1<br>Lperr01g07030.1 0.583 1 0.417 0.018 1<br>Lperr01g07040.2 0.001 0.582 0.581 0.054 0.989	1<br>AT1G54350.1 0.585 0.993 0.408 0.084 0.975<br>AT5G53430.1 0.071 0.581 0.51 0.164 0.984
Oglab01g10320.1	1<br>Oglab01g10320.1 0.001 1 0.999 0.001 1	0	1<br>Osjap01g13630.1 0.042 1 0.958 0.042 1	0	1<br>Orufi01g13080.1 0.042 1 0.958 0.043 1	0	0	1<br>Obrac01g09290.1 0.001 1 0.999 0.001 1	0	0
	0<br>Oglab01g10320.2 0.463 1 0.537 0.002 1	1<br>Obart01g12120.1 0.534 1 0.466 0.002 1<br>Obart01g12130.1 0.042 0.489 0.447 0.087 0.994	1<br>Osjap01g13630.3 0.463 1 0.537 0.002 1<br>Osjap01g13640.1 0.001 0.45 0.449 0.002 0.99	1<br>Osind01g13830.1 0.534 1 0.466 0.002 1<br>Osind01g13840.1 0.001 0.45 0.449 0.002 0.99	1<br>Orufi01g13080.4 0.463 1 0.537 0.002 1<br>Orufi01g13090.1 0.001 0.45 0.449 0.002 0.99	1<br>Olong_021508-R1 0.53 1 0.47 0.064 1<br>Olong_021509-R1 0.077 0.452 0.375 0.005 0.995	1<br>Opunc01g11830.1 0.031 0.489 0.458 0.054 0.994<br>Opunc05g02420.2 0.534 1 0.466 0.002 1	0<br>Obrac05g02000.1 0.534 1 0.466 0.002 1	1<br>Lperr01g11080.1 0.001 0.45 0.449 0.002 0.989<br>Lperr05g21710.3 0.53 1 0.47 0.008 1	1<br>AT3G62130.1 0.025 0.446 0.421 0.018 0.993<br>AT4G04460.1 0.544 1 0.456 0.022 1
Oglab01g24700.1	1<br>Oglab01g24700.1 0.001 1 0.999 0.001 1<br>Oglab01g24700.2 0.001 1 0.999 0.001 1<br>Oglab01g24700.3 0.001 1 0.999 0.001 1	0	1<br>Osjap01g33510.1 0.001 1 0.999 0.022 1<br>Osjap01g33510.2 0.001 1 0.999 0.023 1<br>Osjap01g33510.3 0.001 1 0.999 0.023 1<br>Osjap01g33510.4 0.001 1 0.999 0.025 1	0	0	0	0	1<br>Obrac01g22030.1 0.002 1 0.998 0.02 0.999<br>Obrac01g22030.2 0.002 1 0.998 0.021 0.999	0	0
	0<br>Oglab02g12410.1 0.648 1 0.352 0.063 0.989<br>Oglab03g26370.1 0.242 0.511 0.269 0.103 0.969	1<br>Obart01g29130.1 0.057 0.626 0.569 0.002 0.994<br>Obart02g12550.1 0.648 1 0.352 0.063 0.989	0<br>Osjap03g28950.1 0.242 0.511 0.269 0.103 0.969<br>Osjap07g15680.1 0.64 1 0.36 0.058 0.982	1<br>Osind01g35000.1 0.057 0.626 0.569 0.002 0.994<br>Osind07g13570.1 0.643 1 0.357 0.011 0.981	1<br>Orufi01g32190.1 0.057 0.626 0.569 0.002 0.994<br>Orufi02g12700.1 0.648 1 0.352 0.063 0.989	1<br>Olong_006665-R1 0.131 0.626 0.495 0.012 0.993<br>Olong_027869-R1 0.648 1 0.352 0.064 0.997	1<br>Opunc01g28950.1 0.057 0.626 0.569 0.002 0.994<br>Opunc07g15610.1 0.642 1 0.358 0.313 0.993	0<br>Obrac02g08920.1 0.646 1 0.354 0.149 0.997<br>Obrac02g16330.1 0.249 0.476 0.227 0.21 0.922	0<br>Lperr01g24940.1 0.627 1 0.373 0.02 1<br>Lperr02g18750.1 0.249 0.476 0.227 0.461 0.947	1<br>AT3G24430.1 0.136 0.619 0.483 0.145 0.987<br>AT5G24230.1 0.648 1 0.352 0.098 0.995
Oglab01g24700.2	1<br>Oglab01g24700.1 0.001 1 0.999 0.001 1<br>Oglab01g24700.2 0.001 1 0.999 0.001 1	0	1<br>Osjap01g33510.1 0.001 1 0.999 0.022 1<br>Osjap01g33510.2 0.001 1 0.999 0.023 1	0	0	0	0	1<br>Obrac01g22030.1 0.002 1 0.998 0.02 0.999<br>Obrac01g22030.2 0.002 1 0.998 0.021 0.999	0	0

	Oglab01g24700.3 0.001 1 0.999 0.001 1		Osjap01g33510.3 0.001 1 0.999 0.023 1<br>Osjap01g33510.4 0.001 1 0.999 0.025 1							
	0<br>Oglab02g12410.1 0.643 1 0.357 0.063 0.989<br>Oglab03g26370.1 0.245 0.505 0.26 0.103 0.969	1<br>Obart01g29130.1 0.058 0.621 0.563 0.002 0.994<br>Obart02g12550.1 0.643 1 0.357 0.063 0.989	0<br>Osjap03g28950.1 0.245 0.505 0.26 0.103 0.969<br>Osjap07g15680.1 0.636 1 0.364 0.058 0.982	1<br>Osind01g35000.1 0.058 0.621 0.563 0.002 0.994<br>Osind07g13570.1 0.639 1 0.361 0.011 0.981	1<br>Orufi01g32190.1 0.058 0.621 0.563 0.002 0.994<br>Orufi02g12700.1 0.643 1 0.357 0.063 0.989	1<br>Olong_006665-R1 0.132 0.621 0.489 0.012 0.993<br>Olong_027869-R1 0.643 1 0.357 0.064 0.997	1<br>Opunc01g28950.1 0.058 0.621 0.563 0.002 0.994<br>Opunc07g15610.1 0.638 1 0.362 0.313 0.993	0<br>Obrac02g08920.1 0.642 1 0.358 0.149 0.997	0<br>Lperr01g24930.2 0.086 0.303 0.217 0.439 0.992<br>Lperr01g24940.1 0.623 1 0.377 0.02 1	1<br>AT3G24430.1 0.138 0.615 0.477 0.145 0.987<br>AT5G24230.1 0.643 1 0.357 0.098 0.995
Oglab01g28270.1	1<br>Oglab01g28270.1 0.001 1 0.999 0.001 1	1<br>Obart01g33610.1 0.022 1 0.978 0.001 1	0	0	0	0	0	0	0	0
	1<br>Oglab03g01040.1 0.021 0.23 0.209 0.029 0.99<br>Oglab10g10080.1 0.374 0.998 0.624 0.138 0.999	0<br>Obart10g10470.1 0.374 0.998 0.624 0.121 0.999	1<br>Osjap01g38450.1 0.309 1 0.691 0.022 1<br>Osjap03g01290.1 0.001 0.234 0.233 0.001 0.185	1<br>Osind01g39980.1 0.273 1 0.727 0.117 1<br>Osind01g39990.1 0.001 0.23 0.229 0.005 0.991	1<br>Orufi01g36790.1 0.309 1 0.691 0.022 1<br>Orufi01g36800.1 0.001 0.23 0.229 0.005 0.991	0<br>Olong_007589-R1 0.374 0.998 0.624 0.19 0.999	1<br>Opunc01g32680.1 0.309 1 0.691 0.022 1<br>Opunc03g01090.1 0.001 0.23 0.229 0.004 0.991	1<br>Obrac01g25800.1 0.309 1 0.691 0.022 1<br>Obrac01g25810.1 0.001 0.23 0.229 0.005 0.991	1<br>Lperr01g29020.1 0.294 1 0.706 0.001 1<br>Lperr01g29030.1 0.001 0.23 0.229 0.005 0.991	1<br>AT1G69290.1 0.33 1 0.67 0.036 1<br>AT3G13230.1 0.006 0.23 0.224 0.009 1
Oglab01g34910.1	1<br>Oglab01g34910.1 0.002 1 0.998 0.002 1	1<br>Obart01g40120.1 0.002 1 0.998 0.002 1<br>Obart01g40120.2 0.002 1 0.998 0.002 1	1<br>Osjap01g45670.1 0.002 1 0.998 0.003 1	1<br>Osind01g46990.1 0.002 1 0.998 0.002 1	1<br>Orufi01g43430.1 0.123 1 0.877 0.351 1	1<br>Olong_025215-R1 0.152 0.975 0.823 0.002 0.977	0	0	0	0
	1<br>Oglab01g34890.1 0.123 0.39 0.267 0.646 0.992<br>Oglab11g07730.1 0.439 0.993 0.554 0.021 0.416	0<br>Obart11g08370.2 0.377 0.993 0.616 0.022 0.446	1<br>Osjap01g45650.1 0.123 0.39 0.267 0.646 0.992<br>Osjap11g09760.2 0.439 0.993 0.554 0.021 0.416	1<br>Osind01g46870.1 0.002 0.285 0.283 0.007 0.986<br>Osind11g07730.1 0.453 0.993 0.54 0.002 0.401	0<br>Orufi01g43430.3 0.285 1 0.715 0.045 1	1<br>Olong_009328-R1 0.123 0.37 0.247 0.649 0.983<br>Olong_020581-R1 0.439 0.993 0.554 0.021 0.416	1<br>Opunc04g15200.1 0.123 0.37 0.247 0.687 0.984<br>Opunc11g08140.1 0.377 0.993 0.616 0.122 0.503	1<br>Obrac01g31030.2 0.07 0.381 0.311 0.454 0.952<br>Obrac11g05320.1 0.439 0.993 0.554 0.021 0.416	1<br>Lperr01g34610.3 0.123 0.39 0.267 0.645 0.992<br>Lperr11g07510.1 0.439 0.993 0.554 0.021 0.416	1<br>AT3G28450.1 0.439 0.998 0.559 0.023 0.443<br>AT5G16960.1 0.123 0.383 0.26 0.65 1
Oglab01g37540.3	1<br>Oglab01g37540.1 0.002 0.969 0.967 0.002 0.971<br>Oglab01g37540.2 0.002 0.969 0.967 0.002 0.97<br>Oglab01g37540.3 0.002 1 0.998 0.002 1<br>Oglab01g37540.4 0.002 0.969 0.967 0.003 0.959	0	0	0	0	0	0	0	0	0
	1<br>Oglab02g03680.1 0.257 0.969 0.712 0.038 0.962<br>Oglab05g11700.1 0.002 0.215 0.213 0.005 0.575	1<br>Obart01g42890.1 0.358 0.969 0.611 0.004 0.946<br>Obart05g12620.1 0.002 0.215 0.213 0.005 0.575	1<br>Osjap02g03900.1 0.257 0.969 0.712 0.058 0.963<br>Osjap05g14440.1 0.002 0.215 0.213 0.005 0.575	1<br>Osind01g50140.1 0.002 0.218 0.216 0.008 0.829<br>Osind02g04170.1 0.257 0.969 0.712 0.036 0.962	1<br>Orufi01g46330.1 0.358 0.969 0.611 0.004 0.947<br>Orufi05g13610.1 0.002 0.215 0.213 0.005 0.575	0<br>Olong_010223-R1 0.004 0.224 0.22 0.014 0.53<br>Olong_014935-R1 0.259 0.527 0.268 0.336 0.935<br>Olong_014940-R4 0.533 0.782 0.249 0.02 0.141	1<br>Opunc02g02910.2 0.272 0.941 0.669 0.028 0.92<br>Opunc05g10670.1 0.002 0.218 0.216 0.005 0.58	1<br>Obrac02g02690.2 0.362 1 0.638 0.015 1<br>Obrac05g08200.1 0.002 0.228 0.226 0.008 0.953	0<br>Lperr02g03270.1 0.358 0.941 0.583 0.003 0.907<br>Lperr05g10020.1 0.002 0.211 0.209 0.005 0.608	0<br>AT2G28500.1 0.01 0.272 0.262 0.233 0.832<br>AT5G24630.6 0.636 0.868 0.232 0.599 0.857
Oglab02g00240.1	1<br>Oglab02g00240.1 0.001 1 0.999 0.001 1	1<br>Obart02g00330.1 0.001 1 0.999 0.011 1	1<br>Osjap02g00350.2 0.193 1 0.807 0.001 1	0	1<br>Orufi02g00370.1 0.019 0.959 0.94 0.382 0.979<br>Orufi02g00370.2 0.019 0.959 0.94 0.405 0.98	1<br>Olong_001783-R1 0.066 0.948 0.882 0.001 0.991	0	1<br>Obrac02g00300.1 0.155 0.983 0.828 0.005 0.995<br>Obrac02g00300.2 0.155 0.983 0.828 0.005 0.995	0	0
	1<br>Oglab04g13000.1 0.22 0.99 0.77 0.023 0.994<br>Oglab09g13130.1 0.001 0.203 0.202 0.053 0.995	0<br>Obart04g14710.1 0.22 0.99 0.77 0.025 0.994	1<br>Osjap02g00350.3 0.206 1 0.794 0.004 1<br>Osjap02g00360.1 0.001 0.204 0.203 0.052 1	1<br>Osind02g00340.1 0.213 1 0.787 0.001 1<br>Osind02g00350.1 0.001 0.204 0.203 0.052 1	1<br>Orufi04g16100.1 0.22 0.99 0.77 0.023 0.994<br>Orufi09g15710.1 0.001 0.203 0.202 0.053 0.995	0<br>Olong_004740-R1 0.212 0.992 0.78 0.004 0.996	1<br>Opunc02g00010.2 0.001 0.204 0.203 0.052 1<br>Opunc11g05460.1 0.217 1 0.783 0.002 1	1<br>Obrac09g06000.3 0.216 0.999 0.783 0.007 0.975<br>Obrac09g08720.1 0.001 0.203 0.202 0.053 0.995	1<br>Lperr02g00330.1 0.214 1 0.786 0.001 1<br>Lperr02g00340.1 0.001 0.204 0.203 0.053 1	1<br>AT1G33140.1 0.001 0.203 0.202 0.052 0.995<br>AT3G30340.1 0.22 0.996 0.776 0.027 0.995
Oglab02g12130.1	1<br>Oglab02g12130.1 0.002 1 0.998 0.002 1	0	0	0	0	0	0	0	1<br>Lperr02g10210.1 0.08 0.995 0.915 0.066 0.997	0
	0<br>Oglab02g12130.2 0.002 0.666 0.664 0.002 0.859	1<br>Obart02g12130.1 0.667 0.995 0.328 0.099 0.988	1<br>Osjap02g13370.1 0.667 0.995 0.328 0.09 0.899	1<br>Osind02g13570.1 0.667 0.995 0.328 0.09 0.899	0<br>Orufi02g12280.1 0.002 0.666 0.664 0.002 0.881	1<br>Olong_006942-R1 0.667 0.995 0.328 0.077 0.991	1<br>Opunc02g10840.1 0.667 0.997 0.33 0.069 0.918	1<br>Obrac02g08670.1 0.703 0.997 0.294 0.005 0.87	0<br>Lperr02g10200.1 0.708 0.997 0.289 0.007 0.662	0<br>AT4G32770.1 0.023 0.777 0.754 0.043 0.986

		Obart02g12140.1 0.002 0.666 0.664 0.002 0.881	Osjap02g13380.1 0.002 0.666 0.664 0.002 0.881	Osind02g13580.1 0.002 0.666 0.664 0.002 0.881		Olong_006943-R1 0.157 0.666 0.509 0.003 0.853	Opunc02g10850.2 0.082 0.666 0.584 0.104 0.883	Obrac02g08690.1 0.157 0.666 0.509 0.003 0.847		
Oglab02g22130.2	1<br>Oglab02g22130.2 0.001 1 0.999 0.001 1	1<br>Obart02g23460.2 0.001 1 0.999 0.001 1	1<br>Osjap02g26750.1 0.001 0.969 0.968 0.001 0.994<br>Osjap02g26750.2 0.001 0.969 0.968 0.001 0.994<br>Osjap02g26750.4 0.001 0.969 0.968 0.001 0.799	1<br>Osind02g26640.2 0.001 1 0.999 0.001 1	1<br>Orufi02g24900.1 0.011 0.974 0.963 0.06 0.859<br>Orufi02g24900.3 0.001 0.974 0.973 0.001 0.74<br>Orufi02g24900.4 0.001 0.974 0.973 0.001 0.737	0	1<br>Opunc02g21410.1 0.011 0.999 0.988 0.116 0.996<br>Opunc02g21410.2 0.011 0.999 0.988 0.114 0.996<br>Opunc02g21410.3 0.011 0.999 0.988 0.115 0.996<br>Opunc02g21410.4 0.011 0.999 0.988 0.114 0.996	0	1<br>Lperr02g18970.1 0.011 0.997 0.986 0.032 0.996	0
	1<br>Oglab02g21950.1 0.475 0.999 0.524 0.073 0.995<br>Oglab02g22110.2 0.011 0.459 0.448 0.532 0.98	1<br>Obart02g23290.1 0.475 0.999 0.524 0.029 0.999<br>Obart02g23440.1 0.011 0.459 0.448 0.492 0.981	1<br>Osjap02g26590.1 0.475 0.999 0.524 0.033 0.999<br>Osjap02g26750.5 0.011 0.459 0.448 0.238 0.973	1<br>Osind02g26450.1 0.475 0.999 0.524 0.025 0.997<br>Osind02g26620.1 0.011 0.459 0.448 0.495 0.981	1<br>Orufi02g24730.1 0.475 0.994 0.519 0.031 0.996<br>Orufi02g24880.2 0.011 0.459 0.448 0.18 0.97	0<br>Olong_024345-R1 0.011 0.474 0.463 0.442 0.991	1<br>Opunc02g21230.1 0.479 0.976 0.497 0.003 0.934<br>Opunc02g21240.1 0.011 0.461 0.45 0.121 0.972	1<br>Obrac02g16670.1 0.549 0.973 0.424 0.076 0.995<br>Obrac02g16700.1 0.011 0.461 0.45 0.114 0.947	1<br>Lperr02g18740.1 0.485 0.997 0.512 0.056 0.991<br>Lperr02g19120.1 0.011 0.484 0.473 0.183 0.978	1<br>AT4G31860.1 0.014 0.45 0.436 0.123 0.989<br>AT5G43080.1 0.626 1 0.374 0.194 0.932
Oglab02g33340.1	1<br>Oglab02g33340.1 0.004 1 0.996 0.004 1	1<br>Obart02g36180.1 0.069 0.989 0.92 0.061 0.778<br>Obart02g36180.2 0.069 0.989 0.92 0.073 0.734<br>Obart02g36180.3 0.069 0.989 0.92 0.076 0.724	1<br>Osjap02g40310.1 0.042 0.973 0.931 0.432 0.926<br>Osjap02g40340.1 0.042 1 0.958 0.361 1<br>Osjap02g40370.1 0.042 0.973 0.931 0.267 0.726	1<br>Osind02g39860.1 0.042 0.977 0.935 0.257 0.659<br>Osind02g39860.2 0.069 0.977 0.908 0.058 0.571<br>Osind02g39860.3 0.069 0.973 0.904 0.061 0.631<br>Osind02g39860.4 0.069 0.973 0.904 0.066 0.679	1<br>Orufi02g37680.1 0.069 0.989 0.92 0.067 0.86<br>Orufi02g37680.10 0.069 0.989 0.92 0.091 0.811<br>Orufi02g37680.2 0.069 0.989 0.92 0.07 0.854<br>Orufi02g37680.3 0.069 0.989 0.92 0.076 0.895<br>Orufi02g37680.4 0.069 0.989 0.92 0.073 0.849<br>Orufi02g37680.5 0.069 0.989 0.92 0.076 0.841<br>Orufi02g37680.6 0.069 0.989 0.92 0.088 0.879<br>Orufi02g37680.7 0.069 0.973 0.904 0.079 0.855<br>Orufi02g37680.9 0.069 0.973 0.904 0.086 0.843	0	0	0	0	0
	1<br>Oglab02g34400.1 0.483 0.992 0.509 0.434 0.804<br>Oglab03g25400.1 0.073 0.41 0.337 0.475 0.707	1<br>Obart02g37290.1 0.483 0.992 0.509 0.434 0.804<br>Obart05g21320.1 0.061 0.387 0.326 0.588 0.777	1<br>Osjap02g41540.1 0.483 0.992 0.509 0.434 0.804<br>Osjap05g23970.1 0.061 0.387 0.326 0.669 0.821	1<br>Osind02g41040.1 0.483 0.992 0.509 0.459 0.813<br>Osind05g24480.1 0.061 0.387 0.326 0.588 0.777	0<br>Orufi02g37680.8 0.372 0.989 0.617 0.159 0.801	1<br>Olong_011081-R1 0.479 1 0.521 0.05 1<br>Olong_025373-R1 0.073 0.41 0.337 0.236 0.574	1<br>Opunc02g34450.1 0.483 0.992 0.509 0.432 0.803<br>Opunc05g18980.1 0.061 0.383 0.322 0.588 0.771	1<br>Obrac02g27500.6 0.483 0.992 0.509 0.432 0.803<br>Obrac05g14470.1 0.061 0.383 0.322 0.588 0.771	1<br>Lperr02g30750.1 0.483 0.992 0.509 0.453 0.811<br>Lperr04g17770.1 0.038 0.383 0.345 0.48 0.678	1<br>AT1G16330.1 0.038 0.414 0.376 0.701 0.873<br>AT2G20360.1 0.483 0.992 0.509 0.423 0.799
Oglab02g34160.1	1<br>Oglab02g34160.1 0.001 1 0.999 0.001 1	0	0	0	0	0	0	0	0	0
	0	1<br>Obart02g37040.1 0.704 1 0.296 0.012 1<br>Obart02g37050.1 0.001 0.704 0.703 0.002 0.997	1<br>Osjap02g41280.1 0.001 0.672 0.671 0.002 0.99<br>Osjap10g15310.1 0.694 0.992 0.298 0.289 0.967	1<br>Osind02g40750.1 0.726 1 0.274 0.038 1<br>Osind02g40760.1 0.001 0.672 0.671 0.002 0.99	1<br>Orufi02g38510.1 0.704 1 0.296 0.016 1<br>Orufi02g38520.5 0.001 0.672 0.671 0.002 0.998	1<br>Olong_018845-R1 0.049 0.672 0.623 0.05 0.99<br>Olong_022698-R2 0.694 0.992 0.298 0.04 0.953	1<br>Opunc02g34200.1 0.049 0.673 0.624 0.021 0.916<br>Opunc10g11320.1 0.694 0.992 0.298 0.052 0.944	1<br>Obrac02g27240.1 0.704 1 0.296 0.012 1<br>Obrac02g27260.4 0.055 0.679 0.624 0.038 1	1<br>Lperr02g30470.1 0.704 1 0.296 0.011 1<br>Lperr02g30480.1 0.05 0.672 0.622 0.048 0.899	1<br>AT1G04010.1 0.017 0.671 0.654 0.011 0.913<br>AT3G07090.1 0.704 0.992 0.288 0.011 0.977
Oglab02g34920.2	1<br>Oglab02g34920.2 0.001 1 0.999 0.001 1	0	0	0	0	0	0	0	0	0
	0<br>Oglab02g34920.1 0.001 0.738 0.737 0.001 0.991	1<br>Obart02g37790.1 0.001 0.728 0.727 0.001 0.993<br>Obart12g07130.1 0.73 1 0.27 0.138 1	1<br>Osjap02g42090.2 0.001 0.738 0.737 0.001 0.991<br>Osjap04g03680.2 0.739 1 0.261 0.325 1	1<br>Osind02g41670.1 0.001 0.738 0.737 0.001 0.991<br>Osind04g00200.1 0.739 1 0.261 0.194 0.981	1<br>Orufi02g39350.1 0.001 0.738 0.737 0.001 0.991<br>Orufi02g39370.2 0.739 1 0.261 0.203 1	1<br>Olong_006251-R1 0.001 0.738 0.737 0.002 0.989<br>Olong_026422-R1 0.739 1 0.261 0.203 1	1<br>Opunc02g35010.1 0.001 0.738 0.737 0.001 0.991<br>Opunc08g03270.1 0.739 1 0.261 0.209 1	0<br>Obrac02g27970.1 0.204 0.738 0.534 0.105 0.989<br>Obrac04g01750.2 0.739 1 0.261 0.227 1	1<br>Lperr02g31260.2 0.001 0.738 0.737 0.001 0.991<br>Lperr04g01800.2 0.739 1 0.261 0.124 1	1<br>AT1G33030.1 0.739 1 0.261 0.185 0.994<br>AT5G04460.1 0.012 0.738 0.726 0.022 0.992
Oglab03g00390.1	1<br>Oglab03g00390.1 0.001 1 0.999 0.001 1	1<br>Obart03g00550.1 0.001 0.994 0.993 0.001 0.996	1<br>Osjap03g00680.1 0.001 1 0.999 0.001 1<br>Osjap03g00680.2 0.001 1 0.999 0.001 1	1<br>Osind03g00740.1 0.001 0.994 0.993 0.001 0.996	1<br>Orufi03g12660.1 0.001 1 0.999 0.001 1<br>Orufi03g12660.2 0.001 1 0.999 0.001 1	0	0	0	0	0

			Osjap03g00680.3 0.001 1 0.999 0.001 1							
	1<br>Oglab02g34920.1 0.058 0.634 0.576 0.131 1<br>Oglab09g02040.2 0.696 1 0.304 0.07 1	1<br>Obart03g02140.1 0.652 1 0.348 0.013 1<br>Obart09g20260.1 0.152 0.634 0.482 0.12 1	1<br>Osjap02g42090.1 0.049 0.634 0.585 0.065 1<br>Osjap04g17560.1 0.696 0.998 0.302 0.466 0.996	1<br>Osind02g41670.1 0.058 0.634 0.576 0.131 1<br>Osind03g02220.1 0.652 1 0.348 0.015 1	1<br>Orufi02g39350.1 0.058 0.634 0.576 0.131 1<br>Orufi09g02220.1 0.696 1 0.304 0.072 1	0<br>Olong_013175-R1 0.253 0.906 0.653 0.002 0.961	1<br>Opunc03g00470.1 0.001 0.73 0.729 0.002 0.996<br>Opunc11g04590.1 0.783 0.998 0.215 0.023 1	0<br>Obrac02g10260.1 0.736 0.999 0.263 0.286 0.995<br>Obrac02g27970.1 0.128 0.634 0.506 0.105 1	1<br>Lperr03g00570.1 0.001 0.634 0.633 0.002 1<br>Lperr12g09110.1 0.699 0.998 0.299 0.295 0.967	0<br>AT5G04460.1 0.017 0.634 0.617 0.081 1
Oglab03g05040.1	1<br>Oglab03g05040.1 0.001 1 0.999 0.001 1	0	0	0	0	0	0	0	1<br>Lperr03g04520.1 0.006 1 0.994 0.004 1	0
	0<br>Oglab01g32450.1 0.518 0.996 0.478 0.05 0.943	1<br>Obart03g05120.1 0.483 1 0.517 0.002 1<br>Obart03g05130.1 0.12 0.48 0.36 0.002 0.922	1<br>Osjap03g05170.1 0.483 1 0.517 0.002 1<br>Osjap03g05190.1 0.001 0.48 0.479 0.002 0.94	1<br>Osind03g05440.1 0.483 1 0.517 0.002 1<br>Osind03g05450.1 0.001 0.48 0.479 0.002 0.94	1<br>Orufi03g04890.1 0.483 1 0.517 0.002 1<br>Orufi03g04900.1 0.001 0.48 0.479 0.002 0.94	1<br>Olong_009894-R1 0.524 0.996 0.472 0.047 0.955<br>Olong_019322-R1 0.12 0.48 0.36 0.003 0.907	1<br>Opunc03g04830.1 0.483 1 0.517 0.002 1<br>Opunc03g04840.1 0.001 0.48 0.479 0.002 0.941	1<br>Obrac03g04160.1 0.001 0.48 0.479 0.002 0.926<br>Obrac10g10430.1 0.513 0.998 0.485 0.043 0.998	0<br>Lperr10g10310.1 0.491 0.997 0.506 0.042 0.987	1<br>AT1G26760.1 0.01 0.438 0.428 0.035 0.875<br>AT5G23190.1 0.5 0.996 0.496 0.064 0.961
Oglab03g11620.1	1<br>Oglab03g11620.1 0.001 1 0.999 0.001 1<br>Oglab03g11620.2 0.001 0.994 0.993 0.001 0.994<br>Oglab03g11620.3 0.001 0.994 0.993 0.001 0.994	0	0	0	0	1<br>Olong_011801-R1 0.001 0.994 0.993 0.001 0.993	0	0	1<br>Lperr03g10320.1 0.001 0.999 0.998 0.07 0.992	0
	0<br>Oglab02g22540.1 0.68 0.99 0.31 0.007 0.989	1<br>Obart03g11600.1 0.68 1 0.32 0.015 1<br>Obart03g11610.3 0.001 0.648 0.647 0.001 0.995	1<br>Osjap03g12550.2 0.68 0.994 0.314 0.013 0.86<br>Osjap03g12560.3 0.001 0.648 0.647 0.001 0.994	1<br>Osind03g12280.1 0.68 1 0.32 0.015 1<br>Osind03g12290.1 0.001 0.64 0.639 0.001 0.991	1<br>Orufi03g11950.1 0.68 1 0.32 0.015 1<br>Orufi03g11960.1 0.001 0.648 0.647 0.001 0.993	0	1<br>Opunc03g11160.1 0.001 0.648 0.647 0.001 0.995<br>Opunc07g10560.1 0.683 0.997 0.314 0.014 1	1<br>Obrac03g09320.1 0.68 0.995 0.315 0.015 0.996<br>Obrac03g09330.1 0.001 0.648 0.647 0.001 0.993	1<br>Lperr03g10320.2 0.001 0.648 0.647 0.098 0.989<br>Lperr07g09930.1 0.684 0.991 0.307 0.01 0.607	1<br>AT1G04730.1 0.016 0.648 0.632 0.023 0.994<br>AT3G09030.1 0.68 0.995 0.315 0.013 0.998
Oglab03g11620.2	1<br>Oglab03g11620.1 0.001 0.994 0.993 0.001 0.994<br>Oglab03g11620.2 0.001 1 0.999 0.001 1<br>Oglab03g11620.3 0.001 0.994 0.993 0.001 0.994	0	0	0	0	1<br>Olong_011801-R1 0.001 0.994 0.993 0.001 0.993	0	0	1<br>Lperr03g10320.1 0.001 0.999 0.998 0.07 0.992	0
	0<br>Oglab02g22540.1 0.676 0.99 0.314 0.007 0.989	1<br>Obart03g11600.1 0.675 1 0.325 0.015 1<br>Obart03g11610.3 0.001 0.644 0.643 0.001 0.995	1<br>Osjap03g12550.2 0.675 0.994 0.319 0.013 0.86<br>Osjap03g12560.3 0.001 0.644 0.643 0.001 0.994	1<br>Osind03g12280.1 0.675 1 0.325 0.015 1<br>Osind03g12290.1 0.001 0.635 0.634 0.001 0.991	1<br>Orufi03g11950.1 0.675 1 0.325 0.015 1<br>Orufi03g11960.6 0.001 0.644 0.643 0.001 0.995	0	1<br>Opunc03g11160.1 0.001 0.644 0.643 0.001 0.995<br>Opunc07g10560.1 0.679 0.997 0.318 0.014 1	1<br>Obrac03g09320.1 0.675 0.995 0.32 0.015 0.996<br>Obrac03g09330.1 0.001 0.643 0.642 0.001 0.993	1<br>Lperr03g10320.2 0.001 0.643 0.642 0.098 0.989<br>Lperr07g09930.1 0.679 0.99 0.311 0.01 0.607	1<br>AT1G04730.1 0.016 0.643 0.627 0.023 0.994<br>AT3G09030.1 0.675 0.995 0.32 0.013 0.998
Oglab03g11620.3	1<br>Oglab03g11620.1 0.001 0.994 0.993 0.001 0.994<br>Oglab03g11620.2 0.001 0.994 0.993 0.001 0.994<br>Oglab03g11620.3 0.001 1 0.999 0.001 1	0	0	0	0	1<br>Olong_011801-R1 0.001 0.994 0.993 0.001 0.993	0	0	1<br>Lperr03g10320.1 0.001 0.999 0.998 0.07 0.992	0
	0<br>Oglab02g22540.1 0.662 0.989 0.327 0.007 0.989	1<br>Obart03g11600.1 0.661 1 0.339 0.015 1<br>Obart03g11610.3 0.001 0.628 0.627 0.001 0.995	1<br>Osjap03g12550.2 0.661 0.994 0.333 0.013 0.86<br>Osjap03g12560.3 0.001 0.628 0.627 0.001 0.994	1<br>Osind03g12280.1 0.661 1 0.339 0.015 1<br>Osind03g12290.1 0.001 0.62 0.619 0.001 0.991	1<br>Orufi03g11950.1 0.661 1 0.339 0.015 1<br>Orufi03g11960.1 0.001 0.628 0.627 0.001 0.993	0	1<br>Opunc03g11160.1 0.001 0.628 0.627 0.001 0.995<br>Opunc07g10560.1 0.665 0.996 0.331 0.014 1	1<br>Obrac03g09320.1 0.661 0.995 0.334 0.015 0.996<br>Obrac03g09330.1 0.001 0.628 0.627 0.001 0.993	1<br>Lperr03g10320.2 0.001 0.628 0.627 0.098 0.989<br>Lperr07g09930.1 0.665 0.99 0.325 0.01 0.607	1<br>AT1G04730.1 0.017 0.628 0.611 0.023 0.994<br>AT3G09030.1 0.661 0.995 0.334 0.013 0.998
Oglab03g12120.1	1<br>Oglab03g12120.1 0.001 1 0.999 0.001 1	1<br>Obart03g12120.1 0.001 1 0.999 0.001 1	1<br>Osjap03g13100.1 0.028 1 0.972 0.001 1	1<br>Osind03g12930.1 0.001 1 0.999 0.001 1	0	0	0	0	0	0
	1<br>Oglab06g01620.1 0.642 0.998 0.356 0.212 0.912<br>Oglab12g00800.1 0.114 0.633 0.519 0.483 0.983	1<br>Obart04g15560.1 0.64 0.998 0.358 0.146 0.877<br>Obart11g01060.1 0.114 0.633 0.519 0.471 0.982	1<br>Osjap04g16240.1 0.642 0.998 0.356 0.067 0.921<br>Osjap12g01140.1 0.109 0.633 0.524 0.498 0.981	1<br>Osind04g14790.1 0.642 0.998 0.356 0.013 0.911<br>Osind12g01290.1 0.109 0.633 0.524 0.49 0.981	1<br>Orufi06g01460.3 0.642 0.998 0.356 0.212 0.912<br>Orufi12g01130.1 0.109 0.633 0.524 0.498 0.981	1<br>Olong_007714-R9 0.642 1 0.358 0.002 1<br>Olong_007715-R1 0.125 0.633 0.508 0.007 0.965	1<br>Opunc03g11680.1 0.028 0.633 0.605 0.001 0.971<br>Opunc04g11270.1 0.642 0.998 0.356 0.067 0.921	1<br>Obrac03g09720.1 0.025 0.633 0.608 0.036 0.971<br>Obrac04g09430.1 0.64 0.998 0.358 0.014 0.863	1<br>Lperr03g10720.1 0.028 0.633 0.605 0.001 0.934<br>Lperr03g17470.1 0.633 0.998 0.365 0.119 0.828	1<br>AT3G46740.1 0.122 0.633 0.511 0.158 0.971<br>AT3G53360.1 0.672 0.998 0.326 0.09 0.934

Oglab03g30700.1	1<br>Oglab03g30700.1 0.001 1 0.999 0.001 1	0	0	0	0	0	0	0	0	0
	0<br>Oglab09g01300.1 0.363 0.989 0.626 0.247 0.934	1<br>Obart01g06810.1 0.109 0.347 0.238 0.004 0.594<br>Obart03g31630.1 0.363 0.989 0.626 0.159 0.946	1<br>Osjap01g13740.2 0.123 0.349 0.226 0.054 0.616<br>Osjap09g01290.1 0.363 0.989 0.626 0.355 0.949	1<br>Osind01g07780.1 0.109 0.347 0.238 0.004 0.594<br>Osind09g01320.1 0.363 0.989 0.626 0.247 0.934	1<br>Orufi01g07380.1 0.109 0.347 0.238 0.004 0.594<br>Orufi09g01300.1 0.363 0.989 0.626 0.247 0.934	1<br>Olong_000655-R2 0.112 0.339 0.227 0.012 0.53<br>Olong_014805-R1 0.363 0.989 0.626 0.189 0.924	1<br>Opunc03g28820.1 0.001 0.363 0.362 0.016 0.988<br>Opunc03g28830.1 0.363 0.989 0.626 0.247 0.934	0<br>Obrac05g04080.2 0.363 0.989 0.626 0.21 0.929	1<br>Lperr03g26260.1 0.363 0.989 0.626 0.247 0.934<br>Lperr12g17090.1 0.001 0.358 0.357 0.015 0.941	1<br>AT2G21390.1 0.363 0.989 0.626 0.247 0.933<br>AT5G60550.1 0.081 0.352 0.271 0.16 0.943
Oglab03g30830.1	1<br>Oglab03g30830.1 0.001 1 0.999 0.001 1	1<br>Obart03g31750.1 0.001 1 0.999 0.046 1	1<br>Osjap03g34330.1 0.001 1 0.999 0.001 1	1<br>Osind03g35550.1 0.001 1 0.999 0.039 1	1<br>Orufi03g33060.1 0.001 1 0.999 0.001 1	0	0	0	0	0
	1<br>Oglab03g34460.1 0.629 0.997 0.368 0.016 0.993<br>Oglab10g04340.1 0.012 0.583 0.571 0.1 0.963	1<br>Obart03g36200.1 0.652 1 0.348 0.016 0.997<br>Obart10g04600.1 0.013 0.583 0.57 0.106 0.962	1<br>Osjap03g39110.1 0.652 1 0.348 0.036 1<br>Osjap10g05370.1 0.012 0.583 0.571 0.1 0.963	1<br>Osind01g46540.1 0.652 0.999 0.347 0.065 0.994<br>Osind10g03770.1 0.012 0.583 0.571 0.11 0.96	1<br>Orufi10g04850.1 0.029 0.583 0.554 0.093 0.963<br>Orufi11g12520.1 0.653 1 0.347 0.038 0.991	1<br>Olong_014796-R1 0.001 0.495 0.494 0.002 0.95<br>Olong_024872-R1 0.648 1 0.352 0.01 1	1<br>Opunc01g42030.1 0.652 1 0.348 0.28 0.872<br>Opunc03g28940.2 0.001 0.6 0.599 0.002 0.995	1<br>Obrac02g16280.1 0.646 0.982 0.336 0.063 0.949<br>Obrac03g23440.1 0.001 0.6 0.599 0.002 0.995	1<br>Lperr03g26400.1 0.001 0.6 0.599 0.002 0.995<br>Lperr03g30520.1 0.644 1 0.356 0.003 0.997	0<br>AT1G70730.3 0.001 0.6 0.599 0.118 0.995
Oglab03g32170.1	1<br>Oglab03g32170.1 0.003 1 0.997 0.003 1<br>Oglab03g32170.2 0.003 1 0.997 0.003 1	1<br>Obart03g33760.1 0.003 1 0.997 0.003 1	1<br>Osjap03g36400.1 0.003 1 0.997 0.003 1	1<br>Osind03g37600.1 0.003 1 0.997 0.003 1	1<br>Orufi03g35150.1 0.003 1 0.997 0.003 1	1<br>Olong_002225-R1 0.003 1 0.997 0.136 1<br>Olong_002225-R2 0.003 1 0.997 0.003 1	1<br>Opunc03g30920.1 0.003 1 0.997 0.003 1	0	0	0
	0<br>Oglab05g19940.1 0.32 0.991 0.671 0.015 0.997	1<br>Obart01g34520.1 0.547 0.991 0.444 0.027 0.818<br>Obart03g04650.1 0.003 0.414 0.411 0.007 1	1<br>Osjap01g12980.2 0.547 0.979 0.432 0.032 0.961<br>Osjap03g04690.1 0.003 0.414 0.411 0.007 1	1<br>Osind03g04960.1 0.003 0.414 0.411 0.007 1<br>Osind12g02610.1 0.538 0.991 0.453 0.005 0.759	1<br>Orufi03g04350.1 0.003 0.414 0.411 0.007 1<br>Orufi05g22410.2 0.547 0.997 0.45 0.031 0.969	1<br>Olong_020510-R2 0.523 0.991 0.468 0.291 0.873<br>Olong_025165-R1 0.003 0.414 0.411 0.007 1	0<br>Opunc12g01920.1 0.526 0.973 0.447 0.663 0.947	0<br>Obrac03g25020.5 0.003 0.535 0.532 0.439 0.972	1<br>Lperr01g29830.2 0.532 0.991 0.459 0.203 0.858<br>Lperr03g28250.1 0.003 0.414 0.411 0.007 1	1<br>AT1G52740.1 0.003 0.414 0.411 0.007 1<br>AT4G14640.1 0.544 0.979 0.435 0.033 0.987
Oglab03g32170.2	1<br>Oglab03g32170.1 0.003 1 0.997 0.003 1<br>Oglab03g32170.2 0.003 1 0.997 0.003 1	1<br>Obart03g33760.1 0.003 1 0.997 0.003 1	1<br>Osjap03g36400.1 0.003 1 0.997 0.003 1	1<br>Osind03g37600.1 0.003 1 0.997 0.003 1	1<br>Orufi03g35150.1 0.003 1 0.997 0.003 1	1<br>Olong_002225-R1 0.003 1 0.997 0.136 1<br>Olong_002225-R2 0.003 1 0.997 0.003 1	1<br>Opunc03g30920.1 0.003 1 0.997 0.003 1	0	0	0
	0<br>Oglab05g19940.1 0.32 0.991 0.671 0.015 0.997	1<br>Obart01g34520.1 0.547 0.991 0.444 0.027 0.818<br>Obart03g04650.1 0.003 0.414 0.411 0.007 1	1<br>Osjap01g12980.2 0.547 0.979 0.432 0.032 0.961<br>Osjap03g04690.1 0.003 0.414 0.411 0.007 1	1<br>Osind03g04960.1 0.003 0.414 0.411 0.007 1<br>Osind12g02610.1 0.538 0.991 0.453 0.005 0.759	1<br>Orufi03g04350.1 0.003 0.414 0.411 0.007 1<br>Orufi05g22410.2 0.547 0.997 0.45 0.031 0.969	1<br>Olong_020510-R2 0.523 0.991 0.468 0.291 0.873<br>Olong_025165-R1 0.003 0.414 0.411 0.007 1	0<br>Opunc12g01920.1 0.526 0.973 0.447 0.663 0.947	0<br>Obrac03g25020.5 0.003 0.535 0.532 0.439 0.972	1<br>Lperr01g29830.2 0.532 0.991 0.459 0.203 0.858<br>Lperr03g28250.1 0.003 0.414 0.411 0.007 1	1<br>AT1G52740.1 0.003 0.414 0.411 0.007 1<br>AT4G14640.1 0.544 0.979 0.435 0.033 0.987
Oglab03g35760.1	1<br>Oglab03g35760.1 0.001 1 0.999 0.001 1	0	0	0	0	0	0	0	0	0
	0<br>Oglab09g02410.1 0.47 0.957 0.487 0.443 0.943	1<br>Obart01g02390.1 0.506 0.997 0.491 0.153 0.308<br>Obart07g05930.1 0.001 0.483 0.482 0.168 0.985	1<br>Osjap01g02750.1 0.506 0.983 0.477 0.5 0.973<br>Osjap07g06820.1 0.001 0.483 0.482 0.003 0.982	1<br>Osind01g02910.1 0.506 0.997 0.491 0.232 0.458<br>Osind03g41900.1 0.001 0.481 0.48 0.003 0.977	1<br>Orufi01g02470.1 0.506 0.997 0.491 0.175 0.346<br>Orufi03g39370.2 0.001 0.481 0.48 0.003 0.977	1<br>Olong_013112-R3 0.502 0.957 0.455 0.202 1<br>Olong_024768-R1 0.001 0.481 0.48 0.005 0.942	1<br>Opunc01g02040.3 0.506 0.983 0.477 0.491 0.992<br>Opunc07g05660.1 0.001 0.483 0.482 0.16 0.948	1<br>Obrac01g01910.1 0.506 0.983 0.477 0.506 0.973<br>Obrac03g28110.2 0.001 0.483 0.482 0.003 0.986	1<br>Lperr01g02180.7 0.506 0.999 0.493 0.486 0.973<br>Lperr07g05150.1 0.001 0.483 0.482 0.134 0.953	1<br>AT1G66880.1 0.506 0.955 0.449 0.738 0.964<br>AT3G28630.1 0.001 0.483 0.482 0.099 0.982
Oglab03g38510.1	1<br>Oglab03g38510.1 0.002 1 0.998 0.002 1	0	0	0	0	0	0	0	0	0
	0<br>Oglab01g11460.1 0.647 1 0.353 0.543 0.951<br>Oglab12g00200.1 0.042 0.352 0.31 0.403 0.997	1<br>Obart03g40540.1 0.037 0.344 0.307 0.072 0.986<br>Obart03g40550.1 0.345 0.959 0.614 0.019 0.908	1<br>Osjap03g43930.1 0.037 0.344 0.307 0.072 0.986<br>Osjap03g43940.1 0.345 0.994 0.649 0.018 0.864	1<br>Osind03g44940.1 0.037 0.344 0.307 0.035 0.985<br>Osind03g44950.1 0.345 0.979 0.634 0.019 0.878	1<br>Orufi03g42280.1 0.037 0.344 0.307 0.072 0.986<br>Orufi03g42290.1 0.345 0.893 0.548 0.023 0.951	1<br>Olong_018753-R1 0.345 0.925 0.58 0.02 0.89<br>Olong_018754-R1 0.057 0.344 0.287 0.127 0.985	0<br>Opunc03g37710.1 0.083 0.344 0.261 0.15 0.984<br>Opunc07g07820.1 0.708 0.998 0.29 0.5 0.917	0<br>Obrac01g10510.1 0.616 0.997 0.381 0.488 0.935<br>Obrac03g30550.1 0.084 0.35 0.266 0.238 0.993	0<br>Lperr02g19630.1 0.679 1 0.321 0.436 0.924<br>Lperr03g34570.1 0.099 0.339 0.24 0.193 1	0

Oglab03g39920.1	1<br>Oglab03g39920.1 0.001 1 0.999 0.001 1	0	0	0	0	0	0	0	0	0
	1<br>Oglab02g12900.1 0.004 0.58 0.576 0.05 0.992<br>Oglab05g15580.1 0.616 0.996 0.38 0.208 0.946	1<br>Obart02g13170.1 0.005 0.58 0.575 0.13 0.993<br>Obart06g08490.1 0.619 1 0.381 0.002 1	1<br>Osjap02g14350.1 0.005 0.58 0.575 0.13 0.993<br>Osjap05g18940.1 0.616 0.996 0.38 0.208 0.946	1<br>Osind02g14610.1 0.005 0.58 0.575 0.13 0.993<br>Osind05g19660.1 0.616 0.996 0.38 0.208 0.946	1<br>Orufi01g35570.1 0.005 0.583 0.578 0.14 0.975<br>Orufi05g17840.1 0.616 0.996 0.38 0.208 0.946	1<br>Olong_004931-R1 0.621 1 0.379 0.184 0.927<br>Olong_023965-R1 0.005 0.58 0.575 0.13 0.993	1<br>Opunc02g11640.1 0.005 0.581 0.576 0.108 0.996<br>Opunc05g03040.1 0.621 1 0.379 0.181 0.929	1<br>Obrac02g12790.2 0.005 0.579 0.574 0.122 0.999<br>Obrac06g06500.1 0.591 0.997 0.406 0.026 0.978	1<br>Lperr01g27850.2 0.005 0.579 0.574 0.126 0.999<br>Lperr02g26310.1 0.581 0.997 0.416 0.038 0.979	1<br>AT1G65630.1 0.586 0.995 0.409 0.091 0.948<br>AT4G14990.1 0.005 0.576 0.571 0.142 0.995
Oglab04g03860.1	1<br>Oglab04g03860.1 0.004 1 0.996 0.004 1	1<br>Obart04g04460.1 0.004 0.996 0.992 0.005 0.96<br>Obart04g04460.2 0.004 0.885 0.881 0.006 0.982	1<br>Osjap04g05800.1 0.045 0.996 0.951 0.156 0.964	0	0	0	0	0	0	0
	1<br>Oglab05g07770.1 0.469 0.996 0.527 0.386 0.904<br>Oglab06g24390.1 0.008 0.383 0.375 0.275 0.736	0<br>Obart01g00430.1 0.008 0.383 0.375 0.278 0.737<br>Obart09g05770.4 0.691 0.996 0.305 0.445 0.956	1<br>Osjap01g00480.1 0.008 0.383 0.375 0.276 0.74<br>Osjap05g09580.1 0.469 0.996 0.527 0.385 0.903	1<br>Osind05g10500.1 0.469 0.996 0.527 0.387 0.903<br>Osind06g30600.1 0.008 0.383 0.375 0.275 0.736	1<br>Orufi01g00410.1 0.008 0.383 0.375 0.276 0.74<br>Orufi05g09280.1 0.469 0.996 0.527 0.387 0.903	0<br>Olong_011188-R1 0.56 0.996 0.436 0.056 0.935<br>Olong_021982-R1 0.008 0.28 0.272 0.455 1	1<br>Opunc01g00390.1 0.008 0.383 0.375 0.277 0.738<br>Opunc05g07720.1 0.469 0.996 0.527 0.392 0.903	1<br>Obrac05g05910.1 0.469 0.979 0.51 0.396 0.908<br>Obrac06g19120.5 0.008 0.383 0.375 0.209 0.755	0<br>Lperr08g08820.1 0.008 0.383 0.375 0.327 0.744	0<br>AT4G39220.1 0.008 0.383 0.375 0.277 0.743
Oglab04g27220.1	1<br>Oglab04g27220.1 0.001 1 0.999 0.001 1	0	0	0	0	0	0	0	0	0
	1<br>Oglab02g23520.1 0.544 0.999 0.455 0.028 0.993<br>Oglab06g09240.2 0.001 0.401 0.4 0.072 0.816	1<br>Obart02g04210.1 0.532 0.999 0.467 0.065 0.998<br>Obart04g29190.4 0.001 0.519 0.518 0.001 0.994	1<br>Osjap02g28270.1 0.544 0.999 0.455 0.034 0.993<br>Osjap04g33340.1 0.001 0.519 0.518 0.14 0.994	1<br>Osind02g04720.1 0.532 0.998 0.466 0.017 0.997<br>Osind04g32550.1 0.001 0.519 0.518 0.001 0.994	1<br>Orufi02g26250.1 0.544 0.999 0.455 0.034 0.993<br>Orufi04g30900.1 0.001 0.485 0.484 0.14 0.957	1<br>Olong_002807-R4 0.001 0.485 0.484 0.001 0.953<br>Olong_024919-R1 0.558 0.998 0.44 0.069 0.989	1<br>Opunc02g00670.1 0.545 1 0.455 0.043 0.999<br>Opunc04g26710.1 0.001 0.485 0.484 0.001 0.948	1<br>Obrac04g20580.2 0.001 0.509 0.508 0.017 0.994<br>Obrac06g17870.1 0.547 0.998 0.451 0.051 0.992	1<br>Lperr04g24690.2 0.001 0.489 0.488 0.115 0.915<br>Lperr07g00420.1 0.527 0.998 0.471 0.021 1	0<br>AT2G02220.1 0.551 0.998 0.447 0.047 1
Oglab05g02190.1	1<br>Oglab05g02190.1 0.001 1 0.999 0.001 1	1<br>Obart05g02340.1 0.001 1 0.999 0.001 1	1<br>Osjap05g02530.1 0.079 1 0.921 0.002 1	1<br>Osind05g02510.1 0.079 0.998 0.919 0.002 0.987	0	0	0	0	0	0
	0<br>Oglab03g00660.1 0.608 0.994 0.386 0.321 0.996<br>Oglab05g07310.1 0.082 0.433 0.351 0.013 0.935	1<br>Obart06g15630.1 0.079 0.433 0.354 0.004 0.955<br>Obart11g00590.1 0.501 0.977 0.476 0.146 0.917	1<br>Osjap11g00710.1 0.553 0.997 0.444 0.034 0.911<br>Osjap12g14320.1 0.084 0.441 0.357 0.575 0.91	1<br>Osind07g26860.1 0.487 0.997 0.51 0.035 1<br>Osind12g11780.1 0.03 0.433 0.403 0.012 0.947	1<br>Orufi05g02460.2 0.451 1 0.549 0.003 0.982<br>Orufi07g25010.2 0.082 0.427 0.345 0.007 0.691	1<br>Olong_016162-R1 0.079 0.433 0.354 0.004 0.954<br>Olong_026687-R1 0.451 1 0.549 0.09 0.983	1<br>Opunc05g02220.2 0.451 1 0.549 0.003 0.981<br>Opunc07g20770.2 0.082 0.432 0.35 0.009 0.832	1<br>Obrac05g01840.1 0.451 0.998 0.547 0.169 1<br>Obrac06g15870.1 0.082 0.434 0.352 0.015 0.84	1<br>Lperr05g02100.1 0.451 0.998 0.547 0.017 0.978<br>Lperr11g18770.1 0.027 0.304 0.277 0.358 0.699	0<br>AT5G15380.1 0.468 0.992 0.524 0.064 0.987
Oglab05g03490.1	1<br>Oglab05g03490.1 0.001 1 0.999 0.001 1<br>Oglab05g03490.2 0.001 1 0.999 0.001 1	1<br>Obart05g03830.1 0.001 1 0.999 0.001 1<br>Obart05g03830.2 0.001 1 0.999 0.001 1	1<br>Osjap05g04120.1 0.001 1 0.999 0.001 1<br>Osjap05g04120.2 0.001 1 0.999 0.001 1	1<br>Osind05g04070.1 0.008 1 0.992 0.001 1<br>Osind05g04070.2 0.008 1 0.992 0.001 1	1<br>Orufi05g03950.1 0.001 1 0.999 0.001 1<br>Orufi05g03950.2 0.001 1 0.999 0.001 1	1<br>Olong_016739-R1 0.074 1 0.926 0.001 1	1<br>Opunc05g03540.1 0.001 1 0.999 0.001 1<br>Opunc05g03540.2 0.001 1 0.999 0.001 1	1<br>Obrac05g02930.1 0.074 1 0.926 0.001 1	0	0
	1<br>Oglab01g19490.1 0.09 0.723 0.633 0.066 0.996<br>Oglab03g14060.1 0.79 1 0.21 0.03 1	1<br>Obart01g23310.1 0.09 0.723 0.633 0.066 0.996<br>Obart03g14050.1 0.79 1 0.21 0.03 1	1<br>Osjap01g27410.1 0.09 0.723 0.633 0.066 0.996<br>Osjap03g15140.5 0.743 1 0.257 0.061 1	1<br>Osind01g28630.1 0.09 0.723 0.633 0.066 0.996<br>Osind03g15230.1 0.79 1 0.21 0.03 1	1<br>Orufi01g26130.1 0.09 0.723 0.633 0.066 0.996<br>Orufi03g14720.1 0.79 1 0.21 0.03 1	1<br>Olong_003347-R2 0.09 0.723 0.633 0.047 0.996<br>Olong_009943-R1 0.79 1 0.21 0.03 1	1<br>Opunc01g23460.1 0.09 0.723 0.633 0.064 0.996<br>Opunc03g13590.1 0.79 1 0.21 0.002 0.545	1<br>Obrac01g17590.1 0.09 0.723 0.633 0.067 0.996<br>Obrac03g11330.1 0.79 1 0.21 0.03 1	0<br>Lperr05g03460.1 0.001 0.721 0.72 0.001 0.993	1<br>AT2G47580.1 0.775 1 0.225 0.008 1<br>AT3G16630.1 0.003 0.721 0.718 0.031 0.992
Oglab05g03490.2	1<br>Oglab05g03490.1 0.001 1 0.999 0.001 1<br>Oglab05g03490.2 0.001 1 0.999 0.001 1	1<br>Obart05g03830.1 0.001 1 0.999 0.001 1<br>Obart05g03830.2 0.001 1 0.999 0.001 1	1<br>Osjap05g04120.1 0.001 1 0.999 0.001 1<br>Osjap05g04120.2 0.001 1 0.999 0.001 1	1<br>Osind05g04070.1 0.008 1 0.992 0.001 1<br>Osind05g04070.2 0.008 1 0.992 0.001 1	1<br>Orufi05g03950.1 0.001 1 0.999 0.001 1<br>Orufi05g03950.2 0.001 1 0.999 0.001 1	1<br>Olong_016739-R1 0.073 1 0.927 0.001 1	1<br>Opunc05g03540.1 0.001 1 0.999 0.001 1<br>Opunc05g03540.2 0.001 1 0.999 0.001 1	1<br>Obrac05g02930.1 0.073 1 0.927 0.001 1	0	0
	1<br>Oglab01g19490.1 0.089 0.726 0.637 0.066 0.996<br>Oglab03g14060.1 0.792 1 0.208 0.03 1	1<br>Obart01g23310.1 0.089 0.726 0.637 0.066 0.996<br>Obart03g14050.1 0.792 1 0.208 0.03 1	1<br>Osjap01g27410.1 0.089 0.726 0.637 0.066 0.996<br>Osjap03g15140.5 0.747 1 0.253 0.061 1	1<br>Osind01g28630.1 0.089 0.726 0.637 0.066 0.996<br>Osind03g15230.1 0.792 1 0.208 0.03 1	1<br>Orufi01g26130.1 0.089 0.726 0.637 0.066 0.996<br>Orufi03g14720.1 0.792 1 0.208 0.03 1	1<br>Olong_003347-R2 0.089 0.726 0.637 0.047 0.996<br>Olong_009943-R1 0.792 1 0.208 0.03 1	1<br>Opunc01g23460.1 0.089 0.726 0.637 0.064 0.996<br>Opunc03g13590.1 0.792 1 0.208 0.002 0.545	1<br>Obrac01g17590.1 0.089 0.726 0.637 0.067 0.996<br>Obrac03g11330.1 0.792 1 0.208 0.03 1	0<br>Lperr05g03460.1 0.001 0.725 0.724 0.001 0.993	1<br>AT2G47580.1 0.778 1 0.222 0.008 1<br>AT3G16630.1 0.003 0.725 0.722 0.031 0.992

Oglab05g04140.1	1<br>Oglab05g04140.1 0.002 1 0.998 0.002 1	1<br>Obart05g04470.1 0.002 1 0.998 0.002 1	0	0	0	0	0	0	0	0
	1<br>Oglab05g02840.1 0.362 0.977 0.615 0.395 0.582<br>Oglab06g15820.1 0.014 0.359 0.345 0.033 0.973	1<br>Obart05g03110.1 0.362 0.977 0.615 0.395 0.582<br>Obart06g05940.1 0.014 0.35 0.336 0.048 0.458	1<br>Osjap05g04830.1 0.002 0.35 0.348 0.006 0.86<br>Osjap08g03110.1 0.359 0.993 0.634 0.031 0.944	0<br>Osind05g03400.1 0.274 0.945 0.671 0.792 0.975	1<br>Orufi05g04690.1 0.002 0.35 0.348 0.006 0.86<br>Orufi05g04700.1 0.35 1 0.65 0.212 1	1<br>Olong_002683-R1 0.359 0.993 0.634 0.031 0.945<br>Olong_015421-R1 0.002 0.35 0.348 0.01 1	1<br>Opunc05g03750.1 0.362 0.984 0.622 0.659 0.972<br>Opunc05g04100.1 0.002 0.35 0.348 0.006 0.878	0<br>Obrac05g03390.1 0.346 0.986 0.64 0.441 0.983	1<br>Lperr03g03510.1 0.023 0.35 0.327 0.073 0.593<br>Lperr05g03760.1 0.5 0.984 0.484 0.279 0.955	0<br>AT3G16720.1 0.002 0.35 0.348 0.059 0.523
Oglab05g04450.2	1<br>Oglab05g04450.1 0.001 1 0.999 0.001 1<br>Oglab05g04450.2 0.001 1 0.999 0.001 1	1<br>Obart05g05010.1 0.001 1 0.999 0.01 1<br>Obart05g05010.2 0.001 1 0.999 0.011 1<br>Obart05g05010.3 0.001 1 0.999 0.011 1	0	1<br>Osind05g05280.1 0.181 0.99 0.809 0.001 0.981	0	0	0	0	0	0
	1<br>Oglab05g04600.1 0.677 0.999 0.322 0.044 0.997<br>Oglab05g08330.1 0.001 0.628 0.627 0.001 0.997	1<br>Obart05g09170.2 0.001 0.628 0.627 0.001 0.997<br>Obart07g17280.1 0.667 0.995 0.328 0.002 0.977	1<br>Osjap05g10230.1 0.001 0.629 0.628 0.001 0.999<br>Osjap07g20060.1 0.666 0.999 0.333 0.002 0.991	1<br>Osind05g11170.1 0.665 0.999 0.334 0.02 0.998<br>Osind05g11190.1 0.001 0.629 0.628 0.001 0.999	1<br>Orufi05g05240.1 0.674 0.998 0.324 0.034 0.995<br>Orufi05g09830.2 0.001 0.629 0.628 0.001 0.999	1<br>Olong_008766-R1 0.666 0.998 0.332 0.005 0.995<br>Olong_015082-R1 0.001 0.625 0.624 0.001 0.993	0<br>Opunc05g04580.1 0.001 0.793 0.792 0.001 1	1<br>Obrac05g03760.1 0.664 0.998 0.334 0.003 0.995<br>Obrac11g11360.1 0.001 0.629 0.628 0.001 0.999	1<br>Lperr05g02380.1 0.67 1 0.33 0.003 1<br>Lperr11g15610.1 0.001 0.629 0.628 0.001 0.997	0<br>AT2G27090.1 0.001 0.622 0.621 0.001 0.985
Oglab05g07040.1	1<br>Oglab05g07040.1 0.004 1 0.996 0.004 1	0	0	0	1<br>Orufi05g08440.1 0.004 0.917 0.913 0.003 0.663	0	0	0	0	0
	1<br>Oglab01g16080.1 0.737 0.974 0.237 0.041 0.684<br>Oglab05g07020.1 0.004 0.718 0.714 0.004 0.838	1<br>Obart05g07850.1 0.004 0.718 0.714 0.004 0.87<br>Obart05g07870.1 0.756 1 0.244 0.015 1	1<br>Osjap05g08680.1 0.004 0.718 0.714 0.004 0.964<br>Osjap08g02020.1 0.737 0.974 0.237 0.02 0.34	1<br>Osind05g08590.1 0.004 0.718 0.714 0.004 0.964<br>Osind05g08610.1 0.756 1 0.244 0.015 1	0<br>Orufi01g21290.1 0.737 0.974 0.237 0.041 0.684<br>Orufi05g08430.1 0.086 0.62 0.534 0.156 0.574	1<br>Olong_012066-R1 0.004 0.711 0.707 0.004 0.964<br>Olong_015117-R1 0.759 0.974 0.215 0.009 0.523	1<br>Opunc02g01060.1 0.748 0.974 0.226 0.029 0.248<br>Opunc05g07190.1 0.004 0.711 0.707 0.003 0.978	0<br>Obrac07g06840.1 0.053 0.688 0.635 0.03 0.911	1<br>Lperr07g09890.1 0.03 0.643 0.613 0.006 0.891<br>Lperr08g02000.1 0.737 0.974 0.237 0.028 0.469	0<br>AT4G36020.1 0.752 0.977 0.225 0.043 0.244
Oglab05g09590.1	1<br>Oglab05g09590.1 0.005 1 0.995 0.005 1<br>Oglab05g09590.2 0.005 1 0.995 0.005 1	0	1<br>Osjap05g11740.1 0.005 1 0.995 0.388 1<br>Osjap05g11740.2 0.005 1 0.995 0.432 1	1<br>Osind05g12550.1 0.005 1 0.995 0.005 1	1<br>Orufi05g11190.1 0.005 1 0.995 0.005 1	0	0	0	0	0
	0<br>Oglab11g01510.4 0.507 0.886 0.379 0.681 0.789	1<br>Obart09g04270.2 0.537 0.92 0.383 0.847 0.93<br>Obart11g16680.1 0.065 0.532 0.467 0.051 0.311	0<br>Osjap11g19150.1 0.005 0.532 0.527 0.37 0.489	1<br>Osind08g13680.1 0.537 0.886 0.349 0.748 0.825<br>Osind11g14080.1 0.005 0.532 0.527 0.003 0.309	1<br>Orufi08g12780.1 0.537 0.886 0.349 0.769 0.839<br>Orufi11g17770.2 0.005 0.532 0.527 0.003 0.356	1<br>Olong_007188-R1 0.537 0.886 0.349 0.744 0.822<br>Olong_026954-R1 0.045 0.532 0.487 0.016 0.497	1<br>Opunc08g10590.1 0.537 0.886 0.349 0.768 0.839<br>Opunc11g13660.1 0.065 0.532 0.467 0.051 0.311	0<br>Obrac12g01300.1 0.507 0.886 0.379 0.428 0.614	1<br>Lperr08g09600.2 0.537 0.886 0.349 0.759 0.833<br>Lperr11g13130.1 0.005 0.532 0.527 0.003 0.303	1<br>AT3G47630.2 0.005 0.532 0.527 0.003 0.298<br>AT4G19710.2 0.537 0.886 0.349 0.767 0.838
Oglab05g09590.2	1<br>Oglab05g09590.1 0.005 1 0.995 0.005 1<br>Oglab05g09590.2 0.005 1 0.995 0.005 1	0	1<br>Osjap05g11740.1 0.005 1 0.995 0.388 1<br>Osjap05g11740.2 0.005 1 0.995 0.432 1	1<br>Osind05g12550.1 0.005 1 0.995 0.005 1	1<br>Orufi05g11190.1 0.005 1 0.995 0.005 1	0	0	0	0	0
	0<br>Oglab11g01510.4 0.487 0.881 0.394 0.681 0.789	1<br>Obart09g04270.2 0.518 0.917 0.399 0.847 0.93<br>Obart11g16680.1 0.067 0.513 0.446 0.051 0.311	0<br>Osjap11g19150.1 0.005 0.513 0.508 0.37 0.489	1<br>Osind08g13680.1 0.518 0.881 0.363 0.748 0.825<br>Osind11g14080.1 0.005 0.513 0.508 0.003 0.309	1<br>Orufi08g12780.1 0.518 0.881 0.363 0.769 0.839<br>Orufi11g17770.2 0.005 0.513 0.508 0.003 0.356	1<br>Olong_007188-R1 0.518 0.881 0.363 0.744 0.822<br>Olong_026954-R1 0.047 0.513 0.466 0.016 0.497	1<br>Opunc08g10590.1 0.518 0.881 0.363 0.768 0.839<br>Opunc11g13660.1 0.067 0.513 0.446 0.051 0.311	0<br>Obrac12g01300.1 0.487 0.881 0.394 0.428 0.614	1<br>Lperr08g09600.2 0.518 0.881 0.363 0.759 0.833<br>Lperr11g13130.1 0.005 0.513 0.508 0.003 0.303	1<br>AT3G47630.2 0.005 0.513 0.508 0.003 0.298<br>AT4G19710.2 0.518 0.881 0.363 0.767 0.838
Oglab05g14020.2	1<br>Oglab05g14020.2 0.002 1 0.998 0.002 1	0	0	0	0	0	0	0	0	0
	0<br>Oglab05g14020.1 0.002 0.672 0.67 0.003 1	1<br>Obart01g44290.1 0.676 1 0.324 0.492 1<br>Obart05g14930.1 0.002 0.672 0.67 0.003 1	1<br>Osjap01g50270.1 0.676 1 0.324 0.553 1<br>Osjap05g16940.3 0.002 0.672 0.67 0.003 1	1<br>Osind01g51840.1 0.676 1 0.324 0.553 1<br>Osind05g17720.1 0.002 0.672 0.67 0.003 1	1<br>Orufi01g47720.1 0.676 1 0.324 0.492 1<br>Orufi05g16000.2 0.002 0.672 0.67 0.003 1	0<br>Olong_011178-R1 0.002 0.672 0.67 0.003 1	1<br>Opunc01g43220.1 0.676 1 0.324 0.085 1<br>Opunc05g13020.1 0.002 0.672 0.67 0.003 1	0<br>Obrac05g09800.2 0.124 0.672 0.548 0.014 1	1<br>Lperr01g38720.1 0.693 1 0.307 0.067 1<br>Lperr05g11830.1 0.002 0.672 0.67 0.003 1	1<br>AT2G41200.1 0.767 1 0.233 0.261 1<br>AT4G01220.1 0.006 0.672 0.666 0.019 0.997

Oglab05g20580.1	1<br>Oglab05g20580.1 0.002 1 0.998 0.002 1	0	0	0	0	0	0	0	0	0
	0	1<br>Obart05g21800.1 0.284 1 0.716 0.003 1<br>Obart05g21810.1 0.002 0.209 0.207 0.01 0.98	1<br>Osjap05g24470.1 0.284 1 0.716 0.003 1<br>Osjap05g24480.1 0.002 0.209 0.207 0.01 0.98	1<br>Osind05g24960.1 0.284 1 0.716 0.003 1<br>Osind05g24970.1 0.002 0.209 0.207 0.01 0.98	1<br>Orufi05g23060.1 0.284 1 0.716 0.003 1<br>Orufi05g23070.1 0.002 0.209 0.207 0.01 0.98	0<br>Olong_006515-R1 0.002 0.209 0.207 0.012 0.976<br>Olong_017564-R1 0.705 0.978 0.273 0.007 0.936	1<br>Opunc05g19390.1 0.284 0.996 0.712 0.003 0.997<br>Opunc05g19400.1 0.002 0.209 0.207 0.01 0.98	0<br>Obrac05g14860.1 0.486 0.998 0.512 0.012 1<br>Obrac05g14880.1 0.002 0.209 0.207 0.01 0.979	1<br>Lperr05g17700.1 0.284 0.998 0.714 0.003 1<br>Lperr05g17710.1 0.002 0.209 0.207 0.01 0.981	0<br>AT3G20680.1 0.43 0.989 0.559 0.225 1<br>AT3G59840.1 0.002 0.209 0.207 0.01 0.979
Oglab05g23160.1	1<br>Oglab05g23160.1 0.001 1 0.999 0.001 1	1<br>Obart05g25070.1 0.001 1 0.999 0.001 1	1<br>Osjap05g28230.1 0.001 1 0.999 0.001 1	1<br>Osind05g28520.1 0.001 1 0.999 0.001 1	1<br>Orufi05g26870.1 0.001 1 0.999 0.001 1	0	1<br>Opunc05g22640.1 0.001 1 0.999 0.001 1	0	1<br>Lperr01g22990.1 0.001 1 0.999 0.027 1	0
	1<br>Oglab01g22740.1 0.028 0.451 0.423 0.061 0.995<br>Oglab01g22750.1 0.462 0.999 0.537 0.009 0.999	1<br>Obart01g26870.1 0.006 0.451 0.445 0.117 0.995<br>Obart01g26880.1 0.462 0.999 0.537 0.009 0.999	1<br>Osjap01g31140.1 0.007 0.451 0.444 0.093 0.995<br>Osjap01g31150.1 0.462 0.999 0.537 0.009 0.999	1<br>Osind01g32600.1 0.001 0.451 0.45 0.002 0.995<br>Osind01g32610.1 0.462 0.999 0.537 0.009 0.999	1<br>Orufi01g29880.1 0.001 0.451 0.45 0.075 0.995<br>Orufi01g29890.1 0.462 0.999 0.537 0.009 0.999	1<br>Olong_000367-R1 0.105 0.451 0.346 0.002 0.993<br>Olong_000368-R1 0.456 0.999 0.543 0.108 0.999	1<br>Opunc01g26820.1 0.049 0.451 0.402 0.002 0.994<br>Opunc01g26830.1 0.462 1 0.538 0.009 1	1<br>Obrac01g20400.1 0.048 0.451 0.403 0.006 0.994<br>Obrac05g17560.2 0.451 1 0.549 0.164 1	1<br>Lperr05g20850.2 0.467 1 0.533 0.002 1<br>Lperr05g20860.1 0.001 0.451 0.45 0.002 0.995	1<br>AT5G08690.1 0.012 0.451 0.439 0.034 0.995<br>AT5G51600.1 0.462 0.999 0.537 0.008 0.996
Oglab06g00840.1	1<br>Oglab06g00840.1 0.001 1 0.999 0.001 1	1<br>Obart06g00880.1 0.001 1 0.999 0.001 1	1<br>Osjap06g00940.1 0.04 1 0.96 0.085 1	0	1<br>Orufi06g00860.1 0.067 1 0.933 0.021 1	0	0	0	0	0
	1<br>Oglab06g00730.1 0.526 0.99 0.464 0.257 0.968<br>Oglab07g19660.1 0.033 0.458 0.425 0.142 0.938	1<br>Obart06g00690.1 0.529 1 0.471 0.099 1<br>Obart09g03180.1 0.021 0.458 0.437 0.145 1	1<br>Osjap06g00950.1 0.529 0.992 0.463 0.132 0.99<br>Osjap08g13790.1 0.051 0.458 0.407 0.005 1	1<br>Osind06g00910.1 0.526 1 0.474 0.068 1<br>Osind08g15520.1 0.051 0.458 0.407 0.005 1	1<br>Orufi06g00690.1 0.529 1 0.471 0.099 1<br>Orufi06g00860.2 0.001 0.445 0.444 0.005 0.98	0<br>Olong_023831-R1 0.053 0.583 0.53 0.001 0.373	1<br>Opunc06g00800.1 0.457 1 0.543 0.025 1<br>Opunc06g00830.1 0.05 0.457 0.407 0.006 1	1<br>Obrac06g00660.1 0.526 1 0.474 0.034 0.999<br>Obrac07g14790.1 0.058 0.503 0.445 0.093 0.948	1<br>Lperr01g40030.1 0.055 0.457 0.402 0.013 0.994<br>Lperr06g00740.1 0.531 0.999 0.468 0.078 0.997	0<br>AT4G34470.1 0.063 0.457 0.394 0.013 1
Oglab06g02870.1	1<br>Oglab06g02870.1 0.001 1 0.999 0.001 1	1<br>Obart06g02710.1 0.001 1 0.999 0.03 1	1<br>Osjap06g03080.1 0.001 1 0.999 0.001 1	0	1<br>Orufi06g02670.1 0.045 0.964 0.919 0.032 0.91	1<br>Olong_013693-R1 0.045 1 0.955 0.069 1	0	0	0	0
	1<br>Oglab01g27580.1 0.037 0.715 0.678 0.03 0.949<br>Oglab04g28300.1 0.767 0.968 0.201 0.466 0.888	1<br>Obart01g06310.1 0.037 0.756 0.719 0.066 0.997<br>Obart04g30240.1 0.767 0.968 0.201 0.466 0.888	1<br>Osjap01g37620.1 0.001 0.756 0.755 0.033 0.998<br>Osjap04g34350.1 0.767 0.968 0.201 0.466 0.888	1<br>Osind03g45110.1 0.04 0.75 0.71 0.141 0.984<br>Osind04g33620.1 0.767 0.968 0.201 0.438 0.875	0<br>Orufi01g36040.3 0.037 0.756 0.719 0.03 0.998	1<br>Olong_001861-R1 0.767 0.968 0.201 0.381 0.87<br>Olong_005208-R1 0.014 0.715 0.701 0.141 0.969	1<br>Opunc03g00730.1 0.001 0.756 0.755 0.004 1<br>Opunc04g27750.1 0.767 0.968 0.201 0.426 0.879	1<br>Obrac04g05640.6 0.014 0.705 0.691 0.262 0.995<br>Obrac04g21390.1 0.767 0.968 0.201 0.381 0.87	1<br>Lperr10g07060.1 0.04 0.722 0.682 0.115 0.964<br>Lperr12g00960.1 0.767 0.968 0.201 0.389 0.871	0<br>AT2G37990.1 0.767 0.968 0.201 0.425 0.893
Oglab06g21340.1	1<br>Oglab06g21340.1 0.002 1 0.998 0.002 1	0	0	0	0	0	0	0	0	0
	1<br>Oglab02g06010.1 0.01 0.597 0.587 0.147 0.863<br>Oglab05g08960.1 0.684 0.986 0.302 0.443 0.897	1<br>Obart05g02250.1 0.692 0.966 0.274 0.173 0.46<br>Obart06g23450.1 0.002 0.689 0.687 0.003 0.969	1<br>Osjap05g02420.1 0.692 0.966 0.274 0.167 0.443<br>Osjap06g27500.1 0.002 0.689 0.687 0.002 0.971	1<br>Osind05g02400.1 0.692 0.966 0.274 0.17 0.451<br>Osind06g26900.1 0.009 0.689 0.68 0.162 0.973	1<br>Orufi05g02370.1 0.692 0.966 0.274 0.167 0.443<br>Orufi06g25120.1 0.009 0.689 0.68 0.162 0.973	1<br>Olong_002211-R1 0.009 0.689 0.68 0.126 0.972<br>Olong_004663-R2 0.706 0.966 0.26 0.116 0.242	1<br>Opunc06g21170.1 0.009 0.689 0.68 0.236 0.976<br>Opunc08g00730.1 0.701 0.968 0.267 0.316 0.987	1<br>Obrac05g06640.1 0.72 0.986 0.266 0.346 0.873<br>Obrac08g07630.1 0.005 0.694 0.689 0.187 0.935	1<br>Lperr05g02030.1 0.692 0.966 0.274 0.158 0.439<br>Lperr06g19460.1 0.005 0.689 0.684 0.199 0.975	0<br>AT2G18940.1 0.73 0.986 0.256 0.749 0.901
Oglab06g22820.1	1<br>Oglab06g22820.1 0.005 1 0.995 0.005 1	1<br>Obart06g25430.1 0.005 1 0.995 0.004 1	0	0	0	0	0	0	0	0
	1<br>Oglab06g20540.1 0.005 0.675 0.67 0.625 0.997<br>Oglab09g12320.1 0.72 0.98 0.26 0.658 0.994	1<br>Obart06g22650.1 0.005 0.465 0.46 0.574 0.984<br>Obart06g25420.1 0.625 0.98 0.355 0.403 0.852	1<br>Osjap06g26550.1 0.005 0.465 0.46 0.698 0.988<br>Osjap06g29670.1 0.625 0.98 0.355 0.469 0.992	1<br>Osind06g29020.1 0.625 0.98 0.355 0.469 0.992<br>Osind06g29050.1 0.015 0.54 0.525 0.655 0.915	1<br>Orufi06g24280.1 0.005 0.525 0.52 0.654 0.959<br>Orufi06g27300.1 0.535 0.985 0.45 0.29 0.942	0<br>Olong_024432-R1 0.625 0.98 0.355 0.561 0.994	1<br>Opunc02g22800.1 0.015 0.525 0.51 0.695 0.966<br>Opunc06g23120.1 0.625 0.98 0.355 0.691 0.995	0<br>Obrac09g08010.1 0.72 0.98 0.26 0.586 0.992	0<br>Lperr09g09890.1 0.585 0.98 0.395 0.53 0.994	0<br>AT4G30380.1 0.73 0.97 0.24 0.585 0.984

Oglab06g24190.1	1<br>Oglab06g24190.1 0.001 1 0.999 0.001 1<br>Oglab06g24190.2 0.001 0.995 0.994 0.001 0.991	1<br>Obart06g26750.1 0.001 0.995 0.994 0.008 1	1<br>Osjap06g31210.1 0.001 0.995 0.994 0.008 1	1<br>Osind06g30430.1 0.001 0.995 0.994 0.008 1	1<br>Orufi06g28720.1 0.001 0.995 0.994 0.008 1	1<br>Olong_013658-R1 0.001 0.995 0.994 0.001 1	1<br>Opunc06g24460.1 0.001 0.995 0.994 0.008 1	0	1<br>Lperr06g22350.1 0.001 0.995 0.994 0.033 1	0
	1<br>Oglab02g02970.1 0.694 0.993 0.299 0.006 0.93<br>Oglab12g14250.1 0.001 0.62 0.619 0.001 0.743	1<br>Obart02g03050.1 0.694 0.993 0.299 0.006 0.93<br>Obart12g15260.1 0.001 0.602 0.601 0.001 0.686	1<br>Osjap02g03010.1 0.694 0.993 0.299 0.006 0.928<br>Osjap12g19610.1 0.001 0.62 0.619 0.001 0.675	1<br>Osind02g03310.1 0.694 0.993 0.299 0.006 0.928<br>Osind07g26390.1 0.001 0.59 0.589 0.074 0.935	1<br>Orufi02g02960.1 0.694 0.993 0.299 0.006 0.928<br>Orufi12g16970.1 0.001 0.62 0.619 0.001 0.674	1<br>Olong_017635-R1 0.694 0.993 0.299 0.006 0.93<br>Olong_024721-R1 0.01 0.608 0.598 0.05 0.932	1<br>Opunc02g02350.2 0.715 0.993 0.278 0.248 0.944<br>Opunc03g37840.1 0.001 0.622 0.621 0.047 0.706	1<br>Obrac02g02360.1 0.694 0.994 0.3 0.006 0.933<br>Obrac09g01040.1 0.001 0.591 0.59 0.026 0.872	1<br>Lperr02g02740.1 0.699 0.995 0.296 0.006 0.96<br>Lperr12g12430.1 0.001 0.602 0.601 0.001 0.854	1<br>AT3G10250.1 0.701 0.998 0.297 0.006 0.948<br>AT4G32430.1 0.005 0.61 0.605 0.051 0.976
Oglab06g24190.2	1<br>Oglab06g24190.1 0.001 0.991 0.99 0.001 0.995<br>Oglab06g24190.2 0.001 1 0.999 0.001 1	1<br>Obart06g26750.1 0.001 0.991 0.99 0.008 1	1<br>Osjap06g31210.1 0.001 0.991 0.99 0.008 1	1<br>Osind06g30430.1 0.001 0.991 0.99 0.008 1	1<br>Orufi06g28720.1 0.001 0.991 0.99 0.008 1	1<br>Olong_013658-R1 0.001 0.991 0.99 0.001 1	1<br>Opunc06g24460.1 0.001 0.991 0.99 0.008 1	0	1<br>Lperr06g22350.1 0.001 0.991 0.99 0.033 1	0
	1<br>Oglab02g02970.1 0.691 0.997 0.306 0.006 0.96<br>Oglab12g14250.1 0.001 0.617 0.616 0.001 0.743	1<br>Obart02g03050.1 0.691 0.997 0.306 0.006 0.96<br>Obart12g15260.1 0.001 0.617 0.616 0.001 0.686	1<br>Osjap02g03010.1 0.691 0.997 0.306 0.006 0.959<br>Osjap12g19610.1 0.001 0.617 0.616 0.001 0.675	1<br>Osind02g03310.1 0.691 0.997 0.306 0.006 0.959<br>Osind07g26390.1 0.001 0.587 0.586 0.074 0.935	1<br>Orufi02g02960.1 0.691 0.997 0.306 0.006 0.959<br>Orufi09g02510.1 0.001 0.589 0.588 0.014 0.762	1<br>Olong_017635-R1 0.691 0.997 0.306 0.006 0.96<br>Olong_024721-R1 0.01 0.605 0.595 0.05 0.932	1<br>Opunc02g02350.2 0.712 0.997 0.285 0.248 0.968<br>Opunc03g37840.1 0.001 0.619 0.618 0.047 0.706	1<br>Obrac02g02360.1 0.691 0.997 0.306 0.006 0.961<br>Obrac09g01040.1 0.001 0.588 0.587 0.026 0.872	1<br>Lperr02g02740.1 0.696 0.991 0.295 0.006 0.96<br>Lperr03g02630.1 0.001 0.584 0.583 0.105 0.84	1<br>AT3G10250.1 0.698 0.992 0.294 0.006 0.941<br>AT4G32430.1 0.005 0.607 0.602 0.051 0.976
Oglab06g25490.1	1<br>Oglab06g25490.1 0.001 1 0.999 0.001 1<br>Oglab09g15660.1 0.024 0.999 0.975 0.154 0.95	1<br>Obart06g28060.1 0.001 1 0.999 0.001 1<br>Obart09g17460.1 0.024 1 0.976 0.076 0.969<br>Obart09g17460.2 0.024 1 0.976 0.076 0.969	1<br>Osjap09g17800.1 0.024 1 0.976 0.113 0.974<br>Osjap09g17800.2 0.024 1 0.976 0.114 0.974<br>Osjap09g17800.3 0.024 0.997 0.973 0.187 0.947	1<br>Osind06g31620.1 0.001 1 0.999 0.001 1	1<br>Orufi06g29990.1 0.001 1 0.999 0.001 1<br>Orufi09g18790.1 0.024 1 0.976 0.107 0.97<br>Orufi09g18790.2 0.024 1 0.976 0.107 0.97<br>Orufi09g18790.3 0.024 1 0.976 0.108 0.969<br>Orufi09g18790.4 0.024 1 0.976 0.108 0.969<br>Orufi09g18790.5 0.024 0.976 0.952 0.17 0.937<br>Orufi09g18790.6 0.024 0.996 0.972 0.172 0.937	1<br>Olong_016700-R1 0.001 1 0.999 0.001 1	0	0	1<br>Lperr09g13380.1 0.084 0.982 0.898 0.166 0.951	0
	1<br>Oglab02g01250.1 0.066 0.406 0.34 0.221 0.93<br>Oglab10g06880.1 0.427 1 0.573 0.099 1	1<br>Obart04g21770.1 0.07 0.417 0.347 0.232 0.933<br>Obart10g07310.1 0.427 1 0.573 0.099 1	1<br>Osjap06g32830.1 0.427 1 0.573 0.085 1<br>Osjap06g32840.1 0.001 0.407 0.406 0.002 0.952	1<br>Osind04g24640.1 0.07 0.417 0.347 0.233 0.933<br>Osind06g30840.1 0.485 0.999 0.514 0.109 1	0<br>Orufi06g29150.4 0.485 0.999 0.514 0.122 1	1<br>Olong_000286-R1 0.066 0.406 0.34 0.258 0.928<br>Olong_026973-R2 0.427 1 0.573 0.099 1	1<br>Opunc06g25610.1 0.001 0.407 0.406 0.002 0.951<br>Opunc10g06480.1 0.427 0.998 0.571 0.098 0.984	1<br>Obrac06g20010.2 0.432 1 0.568 0.119 1<br>Obrac06g20020.1 0.001 0.407 0.406 0.002 0.952	1<br>Lperr02g22230.1 0.043 0.392 0.349 0.113 0.88<br>Lperr06g23510.1 0.402 1 0.598 0.074 1	1<br>AT1G76360.1 0.036 0.406 0.37 0.219 0.952<br>AT3G61130.1 0.465 0.999 0.534 0.207 1
Oglab06g25720.1	1<br>Oglab06g25720.1 0.001 1 0.999 0.001 1	0	0	0	0	0	0	0	0	0
	1<br>Oglab06g11430.1 0.001 0.629 0.628 0.001 0.997<br>Oglab06g12400.1 0.782 0.992 0.21 0.422 0.995	1<br>Obart06g11630.1 0.001 0.631 0.63 0.001 0.996<br>Obart06g12910.1 0.782 0.992 0.21 0.422 0.995	1<br>Osjap06g13090.1 0.004 0.67 0.666 0.003 0.966<br>Osjap06g14920.1 0.782 0.992 0.21 0.422 0.995	1<br>Osind06g13010.1 0.019 0.644 0.625 0.005 0.999<br>Osind06g14680.1 0.782 0.997 0.215 0.283 0.989	1<br>Orufi06g12040.1 0.004 0.67 0.666 0.003 0.966<br>Orufi06g13730.1 0.782 0.992 0.21 0.296 0.994	0<br>Olong_024681-R1 0.003 0.645 0.642 0.161 0.964	1<br>Opunc06g10960.1 0.004 0.67 0.666 0.003 0.97<br>Opunc06g12290.1 0.782 0.992 0.21 0.415 0.993	1<br>Obrac06g08260.2 0.001 0.646 0.645 0.037 0.965<br>Obrac08g03520.1 0.782 0.992 0.21 0.431 0.993	0<br>Lperr06g10400.1 0.001 0.632 0.631 0.002 0.983	0
Oglab06g26450.1	1<br>Oglab06g26450.1 0.005 1 0.995 0.005 1	0	0	0	0	0	0	0	0	0
	0<br>Oglab06g11740.1 0.297 0.99 0.693 0.051 0.891	1<br>Obart06g11070.1 0.005 0.287 0.282 0.568 0.761<br>Obart06g11970.1 0.297 0.99 0.693 0.051 0.891	1<br>Osjap06g12660.1 0.005 0.446 0.441 0.568 0.895<br>Osjap06g13590.1 0.475 0.99 0.515 0.154 0.894	1<br>Osind06g12570.1 0.005 0.446 0.441 0.358 0.844<br>Osind06g13420.1 0.475 0.99 0.515 0.146 0.719	1<br>Orufi06g11690.1 0.005 0.446 0.441 0.574 0.905<br>Orufi06g12520.1 0.475 0.99 0.515 0.154 0.894	1<br>Olong_006141-R1 0.475 0.99 0.515 0.131 0.891<br>Olong_026282-R1 0.005 0.332 0.327 0.569 0.923	0<br>Opunc06g10640.1 0.005 0.54 0.535 0.43 0.835	0<br>Obrac06g08090.1 0.005 0.584 0.579 0.543 0.993	1<br>Lperr06g09740.1 0.005 0.361 0.356 0.509 0.758<br>Lperr06g10530.1 0.485 0.99 0.505 0.191 0.921	0
Oglab07g03220.1	1<br>Oglab07g03220.1 0.003 1 0.997 0.003 1	0	0	0	0	0	0	0	0	0

	0<br>Oglab04g16900.2 0.003 0.416 0.413 0.002 0.269	1<br>Obart07g03300.1 0.003 0.416 0.413 0.002 0.262<br>Obart07g03310.1 0.419 1 0.581 0.072 1	1<br>Osjap04g21970.2 0.003 0.416 0.413 0.002 0.253<br>Osjap07g03650.1 0.419 1 0.581 0.072 1	1<br>Osind04g21080.2 0.003 0.416 0.413 0.002 0.269<br>Osind07g03190.1 0.419 1 0.581 0.072 1	1<br>Orufi04g19960.1 0.003 0.416 0.413 0.002 0.269<br>Orufi07g02980.1 0.419 1 0.581 0.065 1	1<br>Olong_006794-R1 0.419 1 0.581 0.072 1<br>Olong_009547-R1 0.003 0.416 0.413 0.002 0.253	1<br>Opunc04g16180.3 0.003 0.416 0.413 0.002 0.269<br>Opunc07g03180.1 0.419 1 0.581 0.21 1	1<br>Obrac04g11780.1 0.003 0.416 0.413 0.002 0.259<br>Obrac07g01940.1 0.419 0.995 0.576 0.068 0.928	0<br>Lperr07g02800.1 0.003 0.416 0.413 0.002 0.273	1<br>AT1G34770.1 0.419 0.989 0.57 0.072 0.975<br>AT4G16480.1 0.003 0.416 0.413 0.003 0.27
Oglab07g20540.2	1<br>Oglab07g20540.1 0.001 1 0.999 0.001 1<br>Oglab07g20540.2 0.001 1 0.999 0.001 1	1<br>Obart07g23990.1 0.001 1 0.999 0.001 1	0	0	0	0	0	0	0	0
	1<br>Oglab05g21510.1 0.602 1 0.398 0.068 1<br>Oglab12g08980.2 0.019 0.597 0.578 0.031 0.953	1<br>Obart04g03800.1 0.59 1 0.41 0.022 1<br>Obart10g03330.1 0.031 0.583 0.552 0.052 0.967	1<br>Osjap06g10100.1 0.596 1 0.404 0.044 1<br>Osjap10g03880.1 0.028 0.583 0.555 0.047 0.967	1<br>Osind05g26020.2 0.602 1 0.398 0.068 1<br>Osind12g09130.1 0.031 0.597 0.566 0.009 0.945	1<br>Orufi05g24100.1 0.602 1 0.398 0.142 1<br>Orufi12g09900.2 0.031 0.597 0.566 0.057 0.953	0<br>Olong_008774-R1 0.577 1 0.423 0.006 1	1<br>Opunc10g02860.1 0.023 0.583 0.56 0.038 0.967<br>Opunc10g02870.1 0.59 1 0.41 0.025 0.989	1<br>Obrac07g07280.1 0.004 0.568 0.564 0.003 0.921<br>Obrac07g07970.1 0.578 1 0.422 0.013 1	1<br>Lperr05g10370.1 0.036 0.583 0.547 0.059 0.935<br>Lperr06g09490.1 0.59 1 0.41 0.024 1	0<br>AT2G37460.1 0.038 0.582 0.544 0.045 0.905
Oglab08g01630.1	1<br>Oglab08g01630.1 0.004 1 0.996 0.004 1	0	0	0	1<br>Orufi08g02390.1 0.004 0.989 0.985 0.004 0.996<br>Orufi08g02390.2 0.004 0.989 0.985 0.004 0.996<br>Orufi08g02390.3 0.004 0.993 0.989 0.003 0.887	0	0	0	0	0
	0<br>Oglab08g01670.1 0.658 0.978 0.32 0.418 0.942	1<br>Obart02g18480.1 0.069 0.611 0.542 0.625 0.865<br>Obart08g02150.1 0.658 0.993 0.335 0.181 0.774	1<br>Osjap02g21180.1 0.069 0.611 0.542 0.658 0.877<br>Osjap08g02350.1 0.658 0.993 0.335 0.181 0.774	1<br>Osind02g21110.1 0.069 0.611 0.542 0.612 0.86<br>Osind08g02280.1 0.658 0.978 0.32 0.494 0.95	1<br>Orufi02g19480.1 0.069 0.611 0.542 0.665 0.88<br>Orufi08g02440.1 0.658 0.978 0.32 0.462 0.946	0<br>Olong_001677-R1 0.658 0.978 0.32 0.235 0.924	1<br>Opunc02g16440.1 0.069 0.611 0.542 0.606 0.858<br>Opunc08g02010.1 0.658 0.975 0.317 0.329 0.956	1<br>Obrac02g12990.1 0.069 0.611 0.542 0.612 0.86<br>Obrac08g01890.2 0.658 0.971 0.313 0.325 0.924	0<br>Lperr08g02240.1 0.658 0.971 0.313 0.314 0.909	0
Oglab08g04500.1	1<br>Oglab08g04500.1 0.004 1 0.996 0.004 1	0	0	0	0	0	0	0	0	0
	0<br>Oglab08g04470.1 0.535 0.961 0.426 0.194 0.524	1<br>Obart08g05010.1 0.662 1 0.338 0.094 1<br>Obart08g05020.1 0.004 0.575 0.571 0.008 1	1<br>Osjap08g05460.1 0.004 0.575 0.571 0.007 1<br>Osjap09g20620.1 0.662 0.987 0.325 0.008 0.597	1<br>Osind08g05440.1 0.004 0.575 0.571 0.007 1<br>Osind09g14950.1 0.649 0.982 0.333 0.372 0.717	1<br>Orufi08g05620.1 0.004 0.575 0.571 0.007 1<br>Orufi09g11280.1 0.649 0.982 0.333 0.007 0.993	1<br>Olong_011729-R1 0.654 0.987 0.333 0.025 0.988<br>Olong_014757-R1 0.004 0.575 0.571 0.182 1	0<br>Opunc10g11500.5 0.491 0.961 0.47 0.143 0.519	0<br>Obrac09g13140.3 0.654 0.987 0.333 0.814 0.926	0<br>Lperr08g04770.1 0.36 0.952 0.592 0.125 0.592	0<br>AT1G31340.1 0.649 0.987 0.338 0.006 0.981
Oglab08g05640.1	1<br>Oglab08g05640.1 0.002 1 0.998 0.002 1	1<br>Obart08g06070.1 0.002 1 0.998 0.002 1	1<br>Osjap08g06650.1 0.002 1 0.998 0.002 1	1<br>Osind08g06790.1 0.002 1 0.998 0.002 1	1<br>Orufi08g06840.1 0.002 1 0.998 0.002 1	0	1<br>Opunc08g05850.1 0.047 1 0.953 0.068 1	0	0	0
	1<br>Oglab02g26360.1 0.348 0.996 0.648 0.01 0.994<br>Oglab04g16180.1 0.047 0.275 0.228 0.198 0.94	1<br>Obart02g28440.1 0.348 0.996 0.648 0.093 0.994<br>Obart04g17770.1 0.047 0.275 0.228 0.198 0.94	1<br>Osjap04g21220.1 0.047 0.275 0.228 0.21 0.936<br>Osjap09g20870.1 0.359 0.984 0.625 0.208 0.966	1<br>Osind04g20250.1 0.047 0.275 0.228 0.198 0.94<br>Osind09g20960.1 0.359 0.984 0.625 0.208 0.966	1<br>Orufi04g19250.2 0.047 0.275 0.228 0.21 0.936<br>Orufi09g21960.1 0.359 0.984 0.625 0.2 0.965	1<br>Olong_008030-R1 0.047 0.275 0.228 0.232 0.937<br>Olong_008031-R2 0.277 1 0.723 0.106 1	1<br>Opunc04g15550.1 0.047 0.275 0.228 0.206 0.938<br>Opunc04g18740.1 0.295 0.984 0.689 0.088 0.994	1<br>Obrac08g04780.1 0.277 1 0.723 0.098 1<br>Obrac08g04800.1 0.002 0.275 0.273 0.006 0.943	1<br>Lperr08g05690.1 0.277 1 0.723 0.1 1<br>Lperr08g05700.1 0.002 0.275 0.273 0.006 0.943	1<br>AT1G05310.1 0.33 0.982 0.652 0.183 0.99<br>AT4G30800.1 0.047 0.275 0.228 0.208 0.943
Oglab08g10710.1	1<br>Oglab08g10710.1 0.006 1 0.994 0.006 1	0	0	0	0	0	0	0	0	0
	0<br>Oglab05g14480.2 0.006 0.438 0.432 0.001 0.091	1<br>Obart01g20880.1 0.006 0.438 0.432 0.001 0.104<br>Obart09g19350.1 0.544 1 0.456 0.003 0.219	1<br>Osjap01g24620.1 0.006 0.438 0.432 0.001 0.104<br>Osjap09g19760.2 0.521 1 0.479 0.075 0.284	1<br>Osind03g16830.1 0.006 0.479 0.473 0.001 0.071<br>Osind09g19920.1 0.521 1 0.479 0.075 0.284	1<br>Orufi01g23550.1 0.006 0.438 0.432 0.001 0.104<br>Orufi09g20900.1 0.544 1 0.456 0.003 0.219	1<br>Olong_001741-R1 0.006 0.479 0.473 0.001 0.106<br>Olong_028599-R1 0.544 1 0.456 0.009 0.703	1<br>Opunc09g17720.1 0.521 1 0.479 0.08 0.288<br>Opunc10g17020.1 0.006 0.444 0.438 0.002 0.128	1<br>Obrac01g12860.1 0.006 0.355 0.349 0.001 0.073<br>Obrac09g12650.1 0.521 1 0.479 0.06 0.276	1<br>Lperr01g14840.1 0.006 0.402 0.396 0.001 0.093<br>Lperr09g15230.1 0.521 1 0.479 0.073 0.281	0<br>AT1G02110.1 0.006 0.479 0.473 0.001 0.115<br>AT2G24220.1 0.692 1 0.308 0.075 0.219
Oglab08g13140.1	1<br>Oglab08g13140.1 0.001 1 0.999 0.001 1	0	1<br>Osjap08g14980.1 0.001 0.819 0.818 0.347 0.856	0	0	0	0	0	0	0

	1<br>Oglab04g06580.1 0.001 0.443 0.442 0.399 0.772<br>Oglab08g13180.1 0.466 0.992 0.526 0.035 0.979	1<br>Obart08g14060.1 0.466 0.992 0.526 0.04 0.976<br>Obart12g10850.1 0.001 0.444 0.443 0.397 0.776	1<br>Osjap08g15020.1 0.466 0.992 0.526 0.035 0.979<br>Osjap12g14110.1 0.001 0.444 0.443 0.397 0.776	1<br>Osind12g11050.1 0.001 0.444 0.443 0.397 0.776<br>Osind12g18550.1 0.471 0.992 0.521 0.004 0.982	1<br>Orufi08g15830.1 0.466 0.992 0.526 0.004 0.982<br>Orufi12g12010.1 0.001 0.444 0.443 0.388 0.773	0<br>Olong_015986-R1 0.425 0.936 0.511 0.754 0.962	1<br>Opunc08g12640.1 0.459 0.992 0.533 0.004 0.981<br>Opunc12g09730.1 0.001 0.444 0.443 0.397 0.778	1<br>Obrac04g04430.1 0.001 0.443 0.442 0.399 0.772<br>Obrac08g09240.1 0.466 0.992 0.526 0.155 0.986	1<br>Lperr08g11630.1 0.451 0.992 0.541 0.002 0.987<br>Lperr12g09160.1 0.001 0.444 0.443 0.397 0.776	1<br>AT2G16050.1 0.471 0.992 0.521 0.004 0.975<br>AT4G10710.1 0.001 0.443 0.442 0.402 0.772
Oglab08g15520.1	1<br>Oglab01g06790.1 0.007 0.844 0.837 0.061 1<br>Oglab08g15520.1 0.007 1 0.993 0.007 1	1<br>Obart01g07700.1 0.007 0.844 0.837 0.061 1<br>Obart08g16670.1 0.007 0.993 0.986 0.007 0.993	1<br>Osjap01g09050.1 0.007 0.844 0.837 0.061 1	1<br>Osind01g08880.1 0.102 0.986 0.884 0.004 0.567	1<br>Orufi01g08450.1 0.007 0.973 0.966 0.026 0.487	1<br>Olong_003647-R1 0.007 0.83 0.823 0.061 0.985	0	0	0	0
	1<br>Oglab03g37410.1 0.714 1 0.286 0.049 0.141<br>Oglab11g05440.1 0.007 0.694 0.687 0.573 0.683	1<br>Obart07g08560.1 0.694 0.952 0.258 0.009 0.192<br>Obart11g05910.1 0.007 0.694 0.687 0.752 0.834	1<br>Osjap01g24690.1 0.007 0.694 0.687 0.104 0.321<br>Osjap07g09900.1 0.694 1 0.306 0.005 0.126	1<br>Osind07g08950.1 0.694 1 0.306 0.004 0.113<br>Osind11g05650.1 0.007 0.694 0.687 0.103 0.319	1<br>Orufi01g23650.1 0.007 0.694 0.687 0.106 0.307<br>Orufi07g08630.1 0.694 1 0.306 0.005 0.117	1<br>Olong_005787-R1 0.007 0.694 0.687 0.103 0.319<br>Olong_016241-R1 0.728 1 0.272 0.735 0.775	1<br>Opunc01g21000.1 0.007 0.694 0.687 0.114 0.334<br>Opunc07g08090.1 0.694 1 0.306 0.004 0.092	1<br>Obrac07g05110.1 0.728 0.993 0.265 0.077 0.151<br>Obrac11g03720.1 0.007 0.694 0.687 0.112 0.34	1<br>Lperr01g17850.1 0.007 0.694 0.687 0.105 0.313<br>Lperr07g08590.1 0.694 0.993 0.299 0.022 0.124	1<br>AT3G55700.1 0.728 0.973 0.245 0.015 0.093<br>AT4G38130.1 0.007 0.68 0.673 0.09 0.269
Oglab09g13510.1	1<br>Oglab09g13510.1 0.002 1 0.998 0.002 1<br>Oglab09g13510.2 0.002 1 0.998 0.002 1<br>Oglab09g13510.3 0.002 1 0.998 0.002 1<br>Oglab09g13510.4 0.002 1 0.998 0.002 1	1<br>Obart09g14980.1 0.002 1 0.998 0.002 1<br>Obart09g14980.2 0.002 1 0.998 0.002 1	1<br>Osjap09g15430.2 0.002 1 0.998 0.002 1<br>Osjap09g15430.3 0.002 1 0.998 0.002 1	1<br>Osind09g15570.1 0.002 1 0.998 0.002 1<br>Osind09g15570.2 0.002 1 0.998 0.002 1	1<br>Orufi09g16160.1 0.002 1 0.998 0.002 1<br>Orufi09g16160.2 0.002 1 0.998 0.002 1	0	1<br>Opunc09g13420.1 0.002 1 0.998 0.002 1<br>Opunc09g13420.2 0.002 1 0.998 0.002 1	1<br>Obrac09g09090.1 0.002 1 0.998 0.002 1	0	0
	1<br>Oglab08g19240.1 0.519 0.994 0.475 0.014 0.986<br>Oglab12g18680.1 0.002 0.501 0.499 0.003 0.997	1<br>Obart02g06180.1 0.009 0.501 0.492 0.01 0.997<br>Obart08g20390.1 0.501 0.994 0.493 0.566 0.988	1<br>Osjap02g06650.1 0.009 0.501 0.492 0.01 0.997<br>Osjap09g15430.1 0.514 1 0.486 0.003 1	1<br>Osind02g06940.1 0.009 0.501 0.492 0.01 0.997<br>Osind08g23920.1 0.519 0.994 0.475 0.012 0.988	1<br>Orufi04g24220.1 0.012 0.502 0.49 0.165 1<br>Orufi08g22880.1 0.519 0.994 0.475 0.012 0.988	1<br>Olong_018059-R1 0.518 0.992 0.474 0.046 0.957<br>Olong_024186-R1 0.002 0.501 0.499 0.003 0.997	1<br>Opunc02g05570.1 0.005 0.501 0.496 0.087 0.997<br>Opunc08g18560.1 0.519 0.994 0.475 0.012 0.969	0<br>Obrac08g13590.1 0.499 0.994 0.495 0.689 0.994	1<br>Lperr04g18350.1 0.002 0.501 0.499 0.021 0.997<br>Lperr08g17230.1 0.507 0.994 0.487 0.021 0.997	1<br>AT2G21270.3 0.003 0.501 0.498 0.059 0.997<br>AT5G42800.1 0.516 0.992 0.476 0.003 0.851
Oglab09g13510.2	1<br>Oglab09g13510.1 0.002 1 0.998 0.002 1<br>Oglab09g13510.2 0.002 1 0.998 0.002 1<br>Oglab09g13510.3 0.002 1 0.998 0.002 1<br>Oglab09g13510.4 0.002 1 0.998 0.002 1	1<br>Obart09g14980.1 0.002 1 0.998 0.002 1<br>Obart09g14980.2 0.002 1 0.998 0.002 1	1<br>Osjap09g15430.2 0.002 1 0.998 0.002 1<br>Osjap09g15430.3 0.002 1 0.998 0.002 1	1<br>Osind09g15570.1 0.002 1 0.998 0.002 1<br>Osind09g15570.2 0.002 1 0.998 0.002 1	1<br>Orufi09g16160.1 0.002 1 0.998 0.002 1<br>Orufi09g16160.2 0.002 1 0.998 0.002 1	0	1<br>Opunc09g13420.1 0.002 1 0.998 0.002 1<br>Opunc09g13420.2 0.002 1 0.998 0.002 1	1<br>Obrac09g09090.1 0.002 1 0.998 0.002 1	0	0
	1<br>Oglab08g19240.1 0.541 0.994 0.453 0.014 0.986<br>Oglab12g18680.1 0.002 0.522 0.52 0.003 0.997	1<br>Obart02g06180.1 0.01 0.522 0.512 0.01 0.997<br>Obart08g20390.1 0.522 0.994 0.472 0.566 0.988	1<br>Osjap02g06650.1 0.01 0.522 0.512 0.01 0.997<br>Osjap09g15430.1 0.537 1 0.463 0.003 1	1<br>Osind02g06940.1 0.01 0.522 0.512 0.01 0.997<br>Osind08g23920.1 0.541 0.994 0.453 0.012 0.988	1<br>Orufi04g24220.1 0.013 0.524 0.511 0.165 1<br>Orufi08g22880.1 0.541 0.994 0.453 0.012 0.988	1<br>Olong_014421-R1 0.537 0.992 0.455 0.002 0.984<br>Olong_024186-R1 0.002 0.522 0.52 0.003 0.997	1<br>Opunc02g05570.1 0.005 0.522 0.517 0.087 0.997<br>Opunc08g18560.1 0.541 0.994 0.453 0.012 0.969	1<br>Obrac02g04550.3 0.008 0.522 0.514 0.214 0.997<br>Obrac09g00560.1 0.54 0.992 0.452 0.043 0.954	1<br>Lperr04g18350.1 0.002 0.522 0.52 0.021 0.997<br>Lperr08g17230.1 0.529 0.994 0.465 0.021 0.997	1<br>AT1G15950.1 0.538 0.992 0.454 0.015 0.936<br>AT2G21270.3 0.003 0.522 0.519 0.059 0.997
Oglab09g13510.3	1<br>Oglab09g13510.1 0.002 1 0.998 0.002 1<br>Oglab09g13510.2 0.002 1 0.998 0.002 1<br>Oglab09g13510.3 0.002 1 0.998 0.002 1<br>Oglab09g13510.4 0.002 1 0.998 0.002 1	1<br>Obart09g14980.1 0.002 1 0.998 0.002 1<br>Obart09g14980.2 0.002 1 0.998 0.002 1	1<br>Osjap09g15430.2 0.002 1 0.998 0.002 1<br>Osjap09g15430.3 0.002 1 0.998 0.002 1	1<br>Osind09g15570.1 0.002 1 0.998 0.002 1<br>Osind09g15570.2 0.002 1 0.998 0.002 1	1<br>Orufi09g16160.1 0.002 1 0.998 0.002 1<br>Orufi09g16160.2 0.002 1 0.998 0.002 1	0	1<br>Opunc09g13420.1 0.002 1 0.998 0.002 1<br>Opunc09g13420.2 0.002 1 0.998 0.002 1	1<br>Obrac09g09090.1 0.002 1 0.998 0.002 1	0	0
	1<br>Oglab08g19240.1 0.544 0.993 0.449 0.143 0.986<br>Oglab12g18680.1 0.002 0.542 0.54 0.003 1	1<br>Obart02g06180.1 0.01 0.54 0.53 0.01 0.997<br>Obart08g20390.1 0.544 0.993 0.449 0.631 0.988	1<br>Osjap02g06650.1 0.01 0.54 0.53 0.01 0.997<br>Osjap09g15430.1 0.544 1 0.456 0.131 1	1<br>Osind02g06940.1 0.01 0.54 0.53 0.01 0.997<br>Osind08g23920.1 0.544 0.993 0.449 0.131 0.988	1<br>Orufi04g24220.1 0.013 0.542 0.529 0.165 1<br>Orufi08g22880.1 0.544 0.993 0.449 0.131 0.988	1<br>Olong_013054-R1 0.547 0.992 0.445 0.149 0.979<br>Olong_024186-R1 0.002 0.542 0.54 0.003 1	1<br>Opunc04g20190.1 0.007 0.542 0.535 0.006 1<br>Opunc08g18560.1 0.544 0.993 0.449 0.128 0.969	1<br>Obrac02g04550.3 0.008 0.54 0.532 0.214 0.997<br>Obrac09g00560.1 0.548 0.998 0.45 0.164 0.966	1<br>Lperr04g18350.1 0.002 0.542 0.54 0.021 1<br>Lperr09g11120.1 0.544 1 0.456 0.156 1	1<br>AT1G68540.1 0.542 0.992 0.45 0.112 1<br>AT2G21270.3 0.003 0.54 0.537 0.059 0.997
Oglab09g13510.4	1<br>Oglab09g13510.1 0.002 1 0.998 0.002 1	1<br>Obart09g14980.1 0.002 1 0.998 0.002 1	1<br>Osjap09g15430.2 0.002 1 0.998 0.002 1	1<br>Osind09g15570.1 0.002 1 0.998 0.002 1	1<br>Orufi09g16160.1 0.002 1 0.998 0.002 1	0	1<br>Opunc09g13420.1 0.002 1 0.998 0.002 1	1<br>Obrac09g09090.1 0.002 1 0.998 0.002 1	0	0

	Oglab09g13510.2 0.002 1 0.998 0.002 1<br>Oglab09g13510.3 0.002 1 0.998 0.002 1<br>Oglab09g13510.4 0.002 1 0.998 0.002 1	Obart09g14980.2 0.002 1 0.998 0.002 1	Osjap09g15430.3 0.002 1 0.998 0.002 1	Osind09g15570.2 0.002 1 0.998 0.002 1	Orufi09g16160.2 0.002 1 0.998 0.002 1		Opunc09g13420.2 0.002 1 0.998 0.002 1			
	1<br>Oglab08g19240.1 0.569 0.993 0.424 0.143 0.986<br>Oglab12g18680.1 0.002 0.567 0.565 0.003 1	1<br>Obart02g06180.1 0.01 0.565 0.555 0.01 0.997<br>Obart08g20390.1 0.569 0.993 0.424 0.631 0.988	1<br>Osjap02g06650.1 0.01 0.565 0.555 0.01 0.997<br>Osjap09g15430.1 0.569 1 0.431 0.131 1	1<br>Osind02g06940.1 0.01 0.565 0.555 0.01 0.997<br>Osind08g23920.1 0.569 0.993 0.424 0.131 0.988	1<br>Orufi04g24220.1 0.014 0.567 0.553 0.165 1<br>Orufi08g22880.1 0.569 0.993 0.424 0.131 0.988	1<br>Olong_008093-R2 0.596 0.991 0.395 0.25 0.996<br>Olong_024186-R1 0.002 0.567 0.565 0.003 1	1<br>Opunc04g20190.1 0.007 0.567 0.56 0.006 1<br>Opunc08g18560.1 0.569 0.993 0.424 0.128 0.969	1<br>Obrac01g09090.1 0.605 0.998 0.393 0.299 0.982<br>Obrac02g04550.3 0.009 0.565 0.556 0.214 0.997	1<br>Lperr04g18350.1 0.002 0.567 0.565 0.021 1<br>Lperr09g11120.1 0.569 1 0.431 0.156 1	1<br>AT1G68540.1 0.567 0.991 0.424 0.112 1<br>AT2G21270.3 0.003 0.565 0.562 0.059 0.997
Oglab09g14970.1	1<br>Oglab09g14970.1 0.001 1 0.999 0.001 1	0	0	0	0	0	0	0	0	0
	1<br>Oglab03g15980.2 0.767 0.971 0.204 0.004 0.818<br>Oglab12g01080.1 0.001 0.686 0.685 0.001 0.984	1<br>Obart09g16470.1 0.713 0.971 0.258 0.077 0.857<br>Obart09g16480.1 0.001 0.69 0.689 0.001 0.977	1<br>Osjap09g16930.1 0.713 0.971 0.258 0.077 0.857<br>Osjap09g16940.1 0.001 0.69 0.689 0.001 0.977	1<br>Osind09g17090.1 0.713 0.971 0.258 0.077 0.857<br>Osind09g17100.1 0.001 0.69 0.689 0.001 0.977	1<br>Orufi09g17780.1 0.713 0.971 0.258 0.077 0.857<br>Orufi09g17790.1 0.001 0.69 0.689 0.001 0.977	1<br>Olong_000845-R1 0.001 0.544 0.543 0.002 1<br>Olong_000846-R1 0.713 0.971 0.258 0.075 0.862	1<br>Opunc09g14900.1 0.713 0.971 0.258 0.077 0.857<br>Opunc09g14910.1 0.001 0.707 0.706 0.001 1	1<br>Obrac08g14920.1 0.001 0.706 0.705 0.18 0.999<br>Obrac09g10340.1 0.713 0.971 0.258 0.077 0.857	1<br>Lperr09g12580.2 0.713 0.971 0.258 0.075 0.857<br>Lperr09g12600.1 0.001 0.707 0.706 0.001 1	1<br>AT2G31350.1 0.708 0.971 0.263 0.046 0.852<br>AT3G19610.1 0.001 0.689 0.688 0.045 0.956
Oglab09g17780.2	1<br>Oglab09g17780.1 0.001 1 0.999 0.484 1<br>Oglab09g17780.2 0.001 1 0.999 0.001 1<br>Oglab09g17780.3 0.001 1 0.999 0.001 1<br>Oglab09g17780.4 0.001 1 0.999 0.001 1<br>Oglab09g17780.5 0.001 1 0.999 0.001 1<br>Oglab09g17780.6 0.001 1 0.999 0.001 1<br>Oglab09g17780.7 0.001 0.987 0.986 0.001 0.979<br>Oglab09g17780.8 0.001 0.987 0.986 0.001 0.978	1<br>Obart09g19580.1 0.046 0.987 0.941 0.466 0.988	0	0	0	1<br>Olong_015753-R1 0.046 0.987 0.941 0.231 0.982	0	0	0	0
	0<br>Oglab06g21810.1 0.029 0.495 0.466 0.075 0.955	1<br>Obart05g23600.1 0.018 0.492 0.474 0.009 0.941<br>Obart09g19600.1 0.568 0.987 0.419 0.062 0.955	1<br>Osjap09g20010.5 0.001 0.515 0.514 0.003 0.997<br>Osjap09g20020.1 0.533 0.987 0.454 0.003 0.954	1<br>Osind09g20180.1 0.001 0.515 0.514 0.632 0.999<br>Osind09g20190.1 0.533 0.99 0.457 0.002 0.976	1<br>Orufi10g16450.1 0.533 1 0.467 0.003 0.812<br>Orufi10g16460.1 0.001 0.515 0.514 0.639 0.999	1<br>Olong_015754-R1 0.568 0.987 0.419 0.06 0.957<br>Olong_021159-R1 0.018 0.492 0.474 0.023 0.935	1<br>Opunc09g18000.1 0.001 0.493 0.492 0.003 0.965<br>Opunc09g18010.1 0.533 0.987 0.454 0.003 0.954	1<br>Obrac05g16330.2 0.018 0.492 0.474 0.011 0.928<br>Obrac09g12850.1 0.533 0.988 0.455 0.002 0.977	1<br>Lperr02g04840.1 0.04 0.511 0.471 0.265 0.995<br>Lperr09g15500.1 0.533 0.987 0.454 0.002 0.977	1<br>AT1G30910.1 0.563 0.985 0.422 0.05 0.95<br>AT3G12740.1 0.04 0.511 0.471 0.08 0.994
Oglab09g17780.3	1<br>Oglab09g17780.1 0.001 1 0.999 0.484 1<br>Oglab09g17780.2 0.001 1 0.999 0.001 1<br>Oglab09g17780.3 0.001 1 0.999 0.001 1<br>Oglab09g17780.4 0.001 1 0.999 0.001 1<br>Oglab09g17780.5 0.001 1 0.999 0.001 1<br>Oglab09g17780.6 0.001 1 0.999 0.001 1<br>Oglab09g17780.7 0.001 0.987 0.986 0.001 0.973<br>Oglab09g17780.8 0.001 0.987 0.986 0.001 0.972	1<br>Obart09g19580.1 0.046 0.987 0.941 0.466 0.985	0	0	0	1<br>Olong_015753-R1 0.046 0.987 0.941 0.231 0.977	0	0	0	0
	0<br>Oglab06g21810.1 0.03 0.498 0.468 0.075 0.955	1<br>Obart05g23600.1 0.018 0.495 0.477 0.009 0.941<br>Obart09g19600.1 0.572 0.987 0.415 0.062 0.944	1<br>Osjap09g20010.5 0.001 0.519 0.518 0.003 0.997<br>Osjap09g20020.1 0.536 0.987 0.451 0.003 0.941	1<br>Osind09g20180.1 0.001 0.519 0.518 0.632 0.999<br>Osind09g20190.1 0.536 0.987 0.451 0.002 0.969	1<br>Orufi10g16450.1 0.536 0.987 0.451 0.003 0.769<br>Orufi10g16460.1 0.001 0.519 0.518 0.639 0.999	1<br>Olong_005512-R4 0.573 0.991 0.418 0.623 0.984<br>Olong_021159-R1 0.018 0.495 0.477 0.023 0.935	1<br>Opunc09g18000.1 0.001 0.496 0.495 0.003 0.965<br>Opunc09g18010.1 0.536 0.987 0.451 0.003 0.941	1<br>Obrac05g16330.2 0.018 0.495 0.477 0.011 0.928<br>Obrac09g12850.1 0.536 0.987 0.451 0.002 0.971	1<br>Lperr02g04840.1 0.04 0.514 0.474 0.265 0.995<br>Lperr09g15500.1 0.536 0.987 0.451 0.002 0.971	1<br>AT1G30910.1 0.566 0.987 0.421 0.05 0.94<br>AT3G12740.1 0.04 0.514 0.474 0.08 0.994
Oglab09g17780.4	1<br>Oglab09g17780.1 0.001 1 0.999 0.484 1<br>Oglab09g17780.2 0.001 1 0.999 0.001 1	1<br>Obart09g19580.1 0.046 0.987 0.941 0.466 0.988	0	0	0	1<br>Olong_015753-R1 0.046 0.987 0.941 0.231 0.982	0	0	0	0

	Oglab09g17780.3 0.001 1 0.999 0.001 1<br>Oglab09g17780.4 0.001 1 0.999 0.001 1<br>Oglab09g17780.5 0.001 1 0.999 0.001 1<br>Oglab09g17780.6 0.001 1 0.999 0.001 1<br>Oglab09g17780.7 0.001 0.987 0.986 0.001 0.979<br>Oglab09g17780.8 0.001 0.987 0.986 0.001 0.978									
	0<br>Oglab06g21810.1 0.029 0.495 0.466 0.075 0.955	1<br>Obart05g23600.1 0.018 0.492 0.474 0.009 0.941<br>Obart09g19600.1 0.568 0.987 0.419 0.062 0.955	1<br>Osjap09g20010.5 0.001 0.515 0.514 0.003 0.997<br>Osjap09g20020.1 0.533 0.987 0.454 0.003 0.954	1<br>Osind09g20180.1 0.001 0.515 0.514 0.632 0.999<br>Osind09g20190.1 0.533 0.99 0.457 0.002 0.976	1<br>Orufi10g16450.1 0.533 1 0.467 0.003 0.812<br>Orufi10g16460.1 0.001 0.515 0.514 0.639 0.999	1<br>Olong_015754-R1 0.568 0.987 0.419 0.06 0.957<br>Olong_021159-R1 0.018 0.492 0.474 0.023 0.935	1<br>Opunc09g18000.1 0.001 0.493 0.492 0.003 0.965<br>Opunc09g18010.1 0.533 0.987 0.454 0.003 0.954	1<br>Obrac05g16330.2 0.018 0.492 0.474 0.011 0.928<br>Obrac09g12850.1 0.533 0.988 0.455 0.002 0.977	1<br>Lperr02g04840.1 0.04 0.511 0.471 0.265 0.995<br>Lperr09g15500.1 0.533 0.987 0.454 0.002 0.977	1<br>AT1G30910.1 0.563 0.985 0.422 0.05 0.95<br>AT3G12740.1 0.04 0.511 0.471 0.08 0.994
Oglab09g17780.5	1<br>Oglab09g17780.1 0.001 1 0.999 0.484 1<br>Oglab09g17780.2 0.001 1 0.999 0.001 1<br>Oglab09g17780.3 0.001 1 0.999 0.001 1<br>Oglab09g17780.4 0.001 1 0.999 0.001 1<br>Oglab09g17780.5 0.001 1 0.999 0.001 1<br>Oglab09g17780.6 0.001 1 0.999 0.001 1<br>Oglab09g17780.7 0.001 0.987 0.986 0.001 0.973<br>Oglab09g17780.8 0.001 0.987 0.986 0.001 0.972	1<br>Obart09g19580.1 0.046 0.987 0.941 0.466 0.985	0	0	0	1<br>Olong_015753-R1 0.046 0.987 0.941 0.231 0.977	0	0	0	0
	0<br>Oglab06g21810.1 0.03 0.498 0.468 0.075 0.955	1<br>Obart05g23600.1 0.018 0.495 0.477 0.009 0.941<br>Obart09g19600.1 0.572 0.987 0.415 0.062 0.944	1<br>Osjap09g20010.5 0.001 0.519 0.518 0.003 0.997<br>Osjap09g20020.1 0.536 0.987 0.451 0.003 0.941	1<br>Osind09g20180.1 0.001 0.519 0.518 0.632 0.999<br>Osind09g20190.1 0.536 0.987 0.451 0.002 0.969	1<br>Orufi10g16450.1 0.536 0.987 0.451 0.003 0.769<br>Orufi10g16460.1 0.001 0.519 0.518 0.639 0.999	1<br>Olong_005512-R4 0.573 0.991 0.418 0.623 0.984<br>Olong_021159-R1 0.018 0.495 0.477 0.023 0.935	1<br>Opunc09g18000.1 0.001 0.496 0.495 0.003 0.965<br>Opunc09g18010.1 0.536 0.987 0.451 0.003 0.941	1<br>Obrac05g16330.2 0.018 0.495 0.477 0.011 0.928<br>Obrac09g12850.1 0.536 0.987 0.451 0.002 0.971	1<br>Lperr02g04840.1 0.04 0.514 0.474 0.265 0.995<br>Lperr09g15500.1 0.536 0.987 0.451 0.002 0.971	1<br>AT1G30910.1 0.566 0.987 0.421 0.05 0.94<br>AT3G12740.1 0.04 0.514 0.474 0.08 0.994
Oglab09g17780.6	1<br>Oglab09g17780.1 0.001 1 0.999 0.484 1<br>Oglab09g17780.2 0.001 1 0.999 0.001 1<br>Oglab09g17780.3 0.001 1 0.999 0.001 1<br>Oglab09g17780.4 0.001 1 0.999 0.001 1<br>Oglab09g17780.5 0.001 1 0.999 0.001 1<br>Oglab09g17780.6 0.001 1 0.999 0.001 1<br>Oglab09g17780.7 0.001 0.987 0.986 0.001 0.973<br>Oglab09g17780.8 0.001 0.987 0.986 0.001 0.972	1<br>Obart09g19580.1 0.046 0.987 0.941 0.466 0.985	0	0	0	1<br>Olong_015753-R1 0.046 0.987 0.941 0.231 0.977	0	0	0	0
	0<br>Oglab06g21810.1 0.03 0.498 0.468 0.075 0.955	1<br>Obart05g23600.1 0.018 0.495 0.477 0.009 0.941<br>Obart09g19600.1 0.572 0.987 0.415 0.062 0.944	1<br>Osjap09g20010.5 0.001 0.519 0.518 0.003 0.997<br>Osjap09g20020.1 0.536 0.987 0.451 0.003 0.941	1<br>Osind09g20180.1 0.001 0.519 0.518 0.632 0.999<br>Osind09g20190.1 0.536 0.987 0.451 0.002 0.969	1<br>Orufi10g16450.1 0.536 0.987 0.451 0.003 0.769<br>Orufi10g16460.1 0.001 0.519 0.518 0.639 0.999	1<br>Olong_005512-R4 0.573 0.991 0.418 0.623 0.984<br>Olong_021159-R1 0.018 0.495 0.477 0.023 0.935	1<br>Opunc09g18000.1 0.001 0.496 0.495 0.003 0.965<br>Opunc09g18010.1 0.536 0.987 0.451 0.003 0.941	1<br>Obrac05g16330.2 0.018 0.495 0.477 0.011 0.928<br>Obrac09g12850.1 0.536 0.987 0.451 0.002 0.971	1<br>Lperr02g04840.1 0.04 0.514 0.474 0.265 0.995<br>Lperr09g15500.1 0.536 0.987 0.451 0.002 0.971	1<br>AT1G30910.1 0.566 0.987 0.421 0.05 0.94<br>AT3G12740.1 0.04 0.514 0.474 0.08 0.994
Oglab09g17780.7	1<br>Oglab09g17780.1 0.001 0.979 0.978 0.484 0.993<br>Oglab09g17780.2 0.001 0.979 0.978 0.001 0.987<br>Oglab09g17780.3 0.001 0.973 0.972 0.001 0.987<br>Oglab09g17780.4 0.001 0.979 0.978 0.001 0.987<br>Oglab09g17780.5 0.001 0.973 0.972 0.001 0.987<br>Oglab09g17780.6 0.001 0.973 0.972 0.001 0.987	1<br>Obart09g19580.1 0.044 1 0.956 0.466 1	0	0	0	1<br>Olong_015753-R1 0.044 1 0.956 0.231 1	0	0	0	0

	Oglab09g17780.7 0.001 1 0.999 0.001 1<br>Oglab09g17780.8 0.001 1 0.999 0.001 1									
	0<br>Oglab06g21810.1 0.029 0.481 0.452 0.075 0.955	1<br>Obart05g23600.1 0.017 0.478 0.461 0.009 0.941<br>Obart09g19600.1 0.552 1 0.448 0.062 1	1<br>Osjap09g20010.5 0.001 0.501 0.5 0.003 0.997<br>Osjap09g20020.1 0.518 1 0.482 0.003 1	1<br>Osind09g20180.1 0.001 0.501 0.5 0.632 0.999<br>Osind09g20190.2 0.535 1 0.465 0.034 1	1<br>Orufi10g16450.2 0.518 0.987 0.469 0.003 1<br>Orufi10g16460.1 0.001 0.501 0.5 0.639 0.999	1<br>Olong_015754-R1 0.552 1 0.448 0.06 1<br>Olong_021159-R1 0.017 0.478 0.461 0.023 0.935	1<br>Opunc09g18000.1 0.001 0.479 0.478 0.003 0.965<br>Opunc09g18010.1 0.518 1 0.482 0.003 1	1<br>Obrac05g16330.2 0.017 0.478 0.461 0.011 0.928<br>Obrac09g12850.1 0.518 1 0.482 0.002 1	1<br>Lperr02g04840.1 0.034 0.496 0.462 0.253 0.995<br>Lperr09g15500.1 0.518 1 0.482 0.002 1	1<br>AT1G30910.1 0.546 0.996 0.45 0.05 0.991<br>AT3G12740.1 0.034 0.496 0.462 0.066 0.994
Oglab09g17780.8	1<br>Oglab09g17780.1 0.001 0.978 0.977 0.484 0.993<br>Oglab09g17780.2 0.001 0.978 0.977 0.001 0.987<br>Oglab09g17780.3 0.001 0.972 0.971 0.001 0.987<br>Oglab09g17780.4 0.001 0.978 0.977 0.001 0.987<br>Oglab09g17780.5 0.001 0.972 0.971 0.001 0.987<br>Oglab09g17780.6 0.001 0.972 0.971 0.001 0.987<br>Oglab09g17780.7 0.001 1 0.999 0.001 1<br>Oglab09g17780.8 0.001 1 0.999 0.001 1	1<br>Obart09g19580.1 0.045 1 0.955 0.466 1	0	0	0	1<br>Olong_015753-R1 0.045 1 0.955 0.231 1	0	0	0	0
	0<br>Oglab06g21810.1 0.029 0.491 0.462 0.075 0.955	1<br>Obart05g23600.1 0.018 0.488 0.47 0.009 0.941<br>Obart09g19600.1 0.564 1 0.436 0.062 1	1<br>Osjap09g20010.5 0.001 0.511 0.51 0.003 0.997<br>Osjap09g20020.1 0.528 1 0.472 0.003 1	1<br>Osind09g20180.1 0.001 0.511 0.51 0.632 0.999<br>Osind09g20190.2 0.546 1 0.454 0.034 1	1<br>Orufi10g16450.2 0.528 0.987 0.459 0.003 1<br>Orufi10g16460.1 0.001 0.511 0.51 0.639 0.999	1<br>Olong_015754-R1 0.564 1 0.436 0.06 1<br>Olong_021159-R1 0.018 0.488 0.47 0.023 0.935	1<br>Opunc09g18000.1 0.001 0.489 0.488 0.003 0.965<br>Opunc09g18010.1 0.528 1 0.472 0.003 1	1<br>Obrac05g16330.2 0.018 0.488 0.47 0.011 0.928<br>Obrac09g12850.1 0.528 1 0.472 0.002 1	1<br>Lperr02g04840.1 0.035 0.507 0.472 0.253 0.995<br>Lperr09g15500.1 0.528 1 0.472 0.002 1	1<br>AT1G30910.1 0.558 0.996 0.438 0.05 0.991<br>AT3G12740.1 0.039 0.507 0.468 0.08 0.994
Oglab09g18200.1	1<br>Oglab09g18200.1 0.001 1 0.999 0.001 1	1<br>Obart09g19980.1 0.066 1 0.934 0.181 1<br>Obart09g19980.2 0.001 1 0.999 0.001 1	1<br>Osjap09g20460.1 0.001 0.999 0.998 0.001 0.999	1<br>Osind09g20560.1 0.001 0.985 0.984 0.001 0.998	1<br>Orufi09g21510.1 0.001 0.958 0.957 0.001 0.999	1<br>Olong_014589-R1 0.116 0.972 0.856 0.032 0.991<br>Olong_014589-R2 0.116 0.972 0.856 0.033 0.991	0	0	0	0
	1<br>Oglab02g17850.1 0.023 0.609 0.586 0.095 0.914<br>Oglab09g18170.1 0.619 0.999 0.38 0.13 0.998	1<br>Obart04g25370.1 0.047 0.617 0.57 0.176 0.967<br>Obart09g19950.1 0.619 0.999 0.38 0.13 0.998	1<br>Osjap02g21530.2 0.023 0.609 0.586 0.096 0.943<br>Osjap09g20430.1 0.649 0.999 0.35 0.151 0.998	1<br>Osind02g21430.2 0.023 0.609 0.586 0.096 0.943<br>Osind09g20550.1 0.619 0.999 0.38 0.058 0.999	1<br>Orufi02g19810.2 0.023 0.609 0.586 0.096 0.943<br>Orufi09g21500.1 0.649 0.999 0.35 0.151 0.998	0<br>Olong_014116-R1 0.063 0.617 0.554 0.069 0.957	1<br>Opunc09g18280.1 0.619 0.998 0.379 0.118 0.951<br>Opunc09g18290.2 0.001 0.618 0.617 0.001 0.971	1<br>Obrac09g13140.3 0.619 0.985 0.366 0.089 0.806<br>Obrac09g13160.1 0.001 0.618 0.617 0.001 0.953	1<br>Lperr09g15890.1 0.619 0.997 0.378 0.085 0.957<br>Lperr09g15920.1 0.001 0.618 0.617 0.001 0.958	0<br>AT1G18160.1 0.021 0.617 0.596 0.064 0.958
Oglab09g18980.1	1<br>Oglab09g18980.1 0.001 1 0.999 0.001 1	0	0	0	0	0	0	0	0	0
	1<br>Oglab01g27280.1 0.001 0.371 0.37 0.002 0.481<br>Oglab08g16490.1 0.403 1 0.597 0.001 1	1<br>Obart03g34250.1 0.025 0.39 0.365 0.05 0.573<br>Obart08g17650.1 0.403 1 0.597 0.001 1	1<br>Osjap01g37290.2 0.001 0.371 0.37 0.065 0.591<br>Osjap08g18690.1 0.45 1 0.55 0.006 1	1<br>Osind03g38060.1 0.025 0.39 0.365 0.05 0.573<br>Osind08g20790.1 0.442 1 0.558 0.001 1	1<br>Orufi01g35750.2 0.001 0.371 0.37 0.069 0.57<br>Orufi08g19800.1 0.45 1 0.55 0.006 1	1<br>Olong_000486-R1 0.403 1 0.597 0.077 1<br>Olong_000981-R1 0.001 0.371 0.37 0.002 0.539	1<br>Opunc01g31560.1 0.001 0.371 0.37 0.002 0.483<br>Opunc08g15500.1 0.458 1 0.542 0.028 1	1<br>Obrac03g25390.2 0.005 0.371 0.366 0.015 0.559<br>Obrac08g11470.1 0.439 1 0.561 0.004 1	1<br>Lperr03g28740.1 0.01 0.392 0.382 0.011 0.568<br>Lperr08g14590.1 0.48 1 0.52 0.103 1	1<br>AT2G03200.1 0.476 1 0.524 0.098 1<br>AT5G04885.1 0.006 0.397 0.391 0.011 0.55
Oglab10g02440.1	1<br>Oglab10g02440.1 0.001 1 0.999 0.001 1<br>Oglab10g02440.2 0.001 1 0.999 0.001 1	1<br>Obart10g02510.2 0.001 1 0.999 0.001 1	0	1<br>Osind10g02110.1 0.001 0.996 0.995 0.001 0.952	0	0	0	0	0	0
	0<br>Oglab10g02440.3 0.434 1 0.566 0.005 1	1<br>Obart10g02510.1 0.001 0.465 0.464 0.001 0.487<br>Obart11g08380.1 0.533 1 0.467 0.194 0.922	1<br>Osjap10g02940.1 0.02 0.375 0.355 0.027 0.984<br>Osjap10g02950.1 0.451 0.974 0.523 0.002 0.928	1<br>Osind10g00350.1 0.001 0.372 0.371 0.001 0.98<br>Osind11g07740.1 0.451 0.996 0.545 0.003 1	0<br>Orufi10g02530.1 0.213 0.996 0.783 0.284 1	1<br>Olong_016648-R1 0.213 0.996 0.783 0.265 1<br>Olong_020863-R1 0.004 0.212 0.208 0.007 0.797	1<br>Opunc10g02120.1 0.001 0.375 0.374 0.001 0.992<br>Opunc11g08150.1 0.451 1 0.549 0.003 0.911	1<br>Obrac03g10950.1 0.001 0.372 0.371 0.001 0.983<br>Obrac11g05340.1 0.451 1 0.549 0.002 0.922	1<br>Lperr10g00410.1 0.001 0.373 0.372 0.003 0.986<br>Lperr10g01950.1 0.451 1 0.549 0.003 0.895	0<br>AT1G21230.1 0.004 0.337 0.333 0.01 0.93<br>AT4G00830.1 0.528 0.974 0.446 0.194 0.891
Oglab10g02440.2	1	1	0	1	0	0	0	0	0	0

	Oglab10g02440.1 0.001 1 0.999 0.001 1<br>Oglab10g02440.2 0.001 1 0.999 0.001 1	Obart10g02510.1 0.001 1 0.999 0.001 1		Osind10g02110.1 0.001 0.995 0.994 0.001 0.952						
	0<br>Oglab10g02440.3 0.462 1 0.538 0.005 1	1<br>Obart10g02510.2 0.001 0.494 0.493 0.001 0.51<br>Obart11g08380.1 0.567 1 0.433 0.194 0.922	1<br>Osjap10g02940.1 0.021 0.399 0.378 0.027 0.984<br>Osjap10g02950.1 0.48 0.972 0.492 0.002 0.928	1<br>Osind10g00350.1 0.001 0.396 0.395 0.001 0.98<br>Osind11g07740.1 0.48 0.995 0.515 0.003 0.96	1<br>Orufi10g02520.1 0.021 0.225 0.204 0.065 0.758<br>Orufi10g02530.1 0.226 0.995 0.769 0.284 0.988	1<br>Olong_016648-R1 0.226 0.995 0.769 0.265 1<br>Olong_020863-R1 0.005 0.225 0.22 0.007 0.797	1<br>Opunc10g02120.1 0.001 0.399 0.398 0.001 0.992<br>Opunc11g08150.1 0.48 1 0.52 0.003 0.911	1<br>Obrac03g10950.1 0.001 0.396 0.395 0.001 0.983<br>Obrac11g05340.1 0.48 1 0.52 0.002 0.922	1<br>Lperr10g00410.1 0.001 0.397 0.396 0.003 0.986<br>Lperr10g01950.1 0.48 1 0.52 0.003 0.895	0<br>AT1G21230.1 0.005 0.358 0.353 0.01 0.93<br>AT4G00830.1 0.561 0.972 0.411 0.194 0.891
Oglab10g03910.1	1<br>Oglab10g03910.1 0.01 1 0.99 0.01 1	1<br>Obart10g18220.1 0.01 1 0.99 0.392 0.823	1<br>Osjap10g04950.1 0.01 1 0.99 0.01 1<br>Osjap10g21210.1 0.01 1 0.99 0.477 1	1<br>Osind10g03420.1 0.01 1 0.99 0.558 1<br>Osind10g17700.1 0.01 1 0.99 0.477 1	1<br>Orufi10g04520.1 0.01 1 0.99 0.01 1<br>Orufi10g19420.1 0.01 1 0.99 0.477 1	1<br>Olong_009744-R1 0.01 1 0.99 0.651 1<br>Olong_009744-R2 0.01 1 0.99 0.641 1	0	0	0	0
	1<br>Oglab02g32260.1 0.361 1 0.639 0.838 0.924<br>Oglab04g27900.1 0.01 0.33 0.32 0.14 0.208	1<br>Obart02g34980.1 0.361 1 0.639 0.838 0.924<br>Obart04g29850.1 0.01 0.33 0.32 0.131 0.196	0<br>Osjap07g13660.9 0.361 1 0.639 0.733 0.947	1<br>Osind02g38620.1 0.361 1 0.639 0.835 0.923<br>Osind06g00010.1 0.01 0.361 0.351 0.609 0.978	1<br>Orufi02g36440.1 0.361 1 0.639 0.835 0.923<br>Orufi08g26670.5 0.01 0.351 0.341 0.583 0.749	1<br>Olong_008835-R2 0.361 1 0.639 0.809 0.91<br>Olong_022843-R2 0.01 0.33 0.32 0.14 0.208	1<br>Opunc02g32260.1 0.361 1 0.639 0.84 0.925<br>Opunc04g27380.2 0.01 0.33 0.32 0.14 0.208	1<br>Obrac02g25650.1 0.381 1 0.619 0.839 0.923<br>Obrac04g21100.1 0.01 0.33 0.32 0.145 0.217	1<br>Lperr02g28770.1 0.381 1 0.619 0.834 0.92<br>Lperr04g25370.2 0.01 0.33 0.32 0.151 0.225	0
Oglab10g08680.2	1<br>Oglab10g08680.1 0.009 1 0.991 0.008 1<br>Oglab10g08680.2 0.001 1 0.999 0.001 1	1<br>Obart10g09110.1 0.009 1 0.991 0.008 1<br>Obart10g09110.2 0.001 1 0.999 0.001 1	0	0	1<br>Orufi10g09480.1 0.009 1 0.991 0.008 1<br>Orufi10g09480.2 0.009 1 0.991 0.008 1<br>Orufi10g09480.3 0.001 1 0.999 0.001 1	1<br>Olong_027249-R1 0.03 1 0.97 0.001 1<br>Olong_027249-R2 0.03 1 0.97 0.001 1	0	0	0	0
	1<br>Oglab02g10660.1 0.625 0.999 0.374 0.129 0.991<br>Oglab12g06810.1 0.013 0.615 0.602 0.056 0.969	0<br>Obart02g10660.1 0.625 0.999 0.374 0.129 0.991	1<br>Osjap01g36630.1 0.626 1 0.374 0.222 1<br>Osjap10g10740.1 0.001 0.626 0.625 0.002 0.997	1<br>Osind10g08120.1 0.626 1 0.374 0.222 1<br>Osind10g08130.1 0.001 0.626 0.625 0.002 0.997	1<br>Orufi01g35060.1 0.626 1 0.374 0.222 1<br>Orufi12g07540.1 0.013 0.615 0.602 0.039 0.673	1<br>Olong_022306-R1 0.609 0.991 0.382 0.19 0.992<br>Olong_024721-R1 0.105 0.604 0.499 0.048 0.942	1<br>Opunc01g30900.1 0.626 1 0.374 0.222 1<br>Opunc10g07870.1 0.004 0.626 0.622 0.003 0.997	1<br>Obrac01g24330.1 0.626 1 0.374 0.221 1<br>Obrac12g04960.2 0.102 0.615 0.513 0.026 0.902	1<br>Lperr01g27340.1 0.626 1 0.374 0.228 1<br>Lperr09g14980.1 0.001 0.604 0.603 0.002 0.959	0<br>AT4G14820.1 0.001 0.614 0.613 0.004 0.86
Oglab10g14420.1	1<br>Oglab10g14420.1 0.007 1 0.993 0.007 1	0	1<br>Osjap04g26670.1 0.059 0.98 0.921 0.005 0.989<br>Osjap10g21180.1 0.046 1 0.954 0.495 1	0	1<br>Orufi04g24510.1 0.007 0.993 0.986 0.009 1<br>Orufi10g19400.1 0.039 1 0.961 0.009 1	0	1<br>Opunc06g20630.1 0.039 1 0.961 0.316 0.984	0	0	0
	1<br>Oglab04g27360.1 0.671 0.928 0.257 0.741 0.891<br>Oglab06g09210.2 0.039 0.586 0.547 0.082 0.173	1<br>Obart04g29320.1 0.671 0.928 0.257 0.741 0.891<br>Obart08g15190.1 0.039 0.586 0.547 0.001 0.106	1<br>Osjap02g36030.1 0.039 0.586 0.547 0.001 0.098<br>Osjap04g33480.1 0.671 0.928 0.257 0.741 0.891	1<br>Osind04g32680.1 0.671 0.928 0.257 0.741 0.891<br>Osind08g18030.1 0.039 0.586 0.547 0.001 0.106	1<br>Orufi02g33550.1 0.007 0.586 0.579 0.001 0.104<br>Orufi04g31030.1 0.671 0.928 0.257 0.741 0.891	0<br>Olong_004819-R1 0.026 0.362 0.336 0.231 0.615	1<br>Opunc02g29570.2 0.033 0.586 0.553 0.008 0.106<br>Opunc04g26840.1 0.671 0.928 0.257 0.741 0.891	0<br>Obrac02g23230.1 0.033 0.586 0.553 0.007 0.1	0<br>Lperr02g26070.3 0.007 0.586 0.579 0.001 0.112	0<br>AT1G59610.1 0.039 0.586 0.547 0.001 0.099
Oglab10g16090.1	1<br>Oglab10g16090.1 0.001 1 0.999 0.001 1	1<br>Obart10g19720.1 0.001 0.861 0.86 0.002 0.992	0	1<br>Osind10g19230.1 0.001 0.861 0.86 0.002 0.992	1<br>Orufi10g20960.1 0.001 0.861 0.86 0.002 0.992	0	1<br>Opunc10g18180.1 0.001 0.99 0.989 0.002 0.998	0	0	0
	1<br>Oglab01g14760.1 0.419 0.984 0.565 0.2 1<br>Oglab08g03250.2 0.037 0.374 0.337 0.052 0.955	1<br>Obart01g17550.1 0.419 0.977 0.558 0.174 0.863<br>Obart08g03750.1 0.037 0.374 0.337 0.057 0.95	1<br>Osjap10g22750.1 0.411 1 0.589 0.061 1<br>Osjap10g22760.1 0.001 0.392 0.391 0.003 0.993	1<br>Osind01g20890.1 0.419 0.977 0.558 0.154 0.802<br>Osind08g03990.1 0.037 0.374 0.337 0.057 0.95	1<br>Orufi01g19490.1 0.419 0.977 0.558 0.171 0.862<br>Orufi08g04140.1 0.037 0.374 0.337 0.057 0.95	1<br>Olong_006312-R1 0.416 1 0.584 0.067 1<br>Olong_006313-R1 0.001 0.392 0.391 0.003 0.993	1<br>Opunc01g18040.1 0.419 0.984 0.565 0.174 1<br>Opunc08g03700.1 0.014 0.374 0.36 0.011 0.951	1<br>Obrac01g13330.1 0.423 0.984 0.561 0.196 1<br>Obrac10g13120.1 0.001 0.392 0.391 0.004 0.993	1<br>Lperr10g15590.1 0.416 0.985 0.569 0.07 0.989<br>Lperr10g15600.1 0.001 0.389 0.388 0.003 0.979	1<br>AT1G32080.1 0.42 0.984 0.564 0.188 1<br>AT2G32260.1 0.035 0.375 0.34 0.078 0.825
Oglab11g01340.2	1<br>Oglab11g01340.2 0.004 1 0.996 0.004 1	0	1<br>Osjap11g01730.2 0.004 1 0.996 0.004 1	0	1<br>Orufi11g01440.2 0.004 1 0.996 0.004 1	0	1<br>Opunc11g01300.1 0.004 0.989 0.985 0.309 1	0	0	0
	0<br>Oglab11g01340.3 0.442 0.925 0.483 0.004 0.929	1<br>Obart12g01240.1 0.494 0.993 0.499 0.002 0.566<br>Obart12g01250.1 0.004 0.438 0.434 0.57 0.939	0<br>Osjap11g01730.3 0.461 1 0.539 0.004 1	1<br>Osind11g01160.1 0.449 1 0.551 0.004 1<br>Osind12g01910.1 0.004 0.438 0.434 0.57 0.939	1<br>Orufi12g01510.1 0.446 1 0.554 0.007 1<br>Orufi12g01520.1 0.004 0.438 0.434 0.663 0.953	1<br>Olong_020639-R1 0.449 1 0.551 0.014 1<br>Olong_025830-R1 0.004 0.438 0.434 0.502 0.93	1<br>Opunc11g01300.2 0.004 0.438 0.434 0.538 0.935<br>Opunc12g01310.1 0.453 0.955 0.502 0.032 0.866	0<br>Obrac11g03920.2 0.004 0.446 0.442 0.631 0.971	0<br>Lperr11g01230.1 0.648 0.858 0.21 0.245 0.765<br>Lperr12g01420.1 0.004 0.438 0.434 0.547 0.936	0<br>AT1G04590.2 0.004 0.625 0.621 0.534 0.997

Oglab11g01510.4	1<br>Oglab11g01510.1 0.002 1 0.998 0.14 1<br>Oglab11g01510.2 0.002 1 0.998 0.202 1<br>Oglab11g01510.3 0.002 1 0.998 0.203 1<br>Oglab11g01510.4 0.002 1 0.998 0.002 1<br>Oglab11g01510.5 0.002 1 0.998 0.002 1	1<br>Obart11g01710.1 0.002 0.994 0.992 0.104 0.922<br>Obart11g01710.2 0.002 0.994 0.992 0.106 0.921<br>Obart11g01710.3 0.002 0.994 0.992 0.115 0.914<br>Obart11g01710.4 0.002 0.994 0.992 0.117 0.913	1<br>Osjap11g01890.1 0.002 0.989 0.987 0.147 0.988<br>Osjap11g01890.2 0.002 1 0.998 0.143 1<br>Osjap11g01890.3 0.002 0.989 0.987 0.13 0.988<br>Osjap11g01890.4 0.002 0.989 0.987 0.149 0.988<br>Osjap11g01890.5 0.002 0.989 0.987 0.142 0.988<br>Osjap11g01890.6 0.002 1 0.998 0.146 1<br>Osjap11g01890.7 0.002 0.989 0.987 0.201 0.983	0	0	0	1<br>Opunc12g01520.7 0.002 0.989 0.987 0.23 0.981	0	1<br>Lperr12g01560.1 0.002 0.989 0.987 0.13 0.989	0
	1<br>Oglab12g01390.1 0.434 0.989 0.555 0.003 0.961<br>Oglab12g01400.2 0.002 0.408 0.406 0.291 0.961	1<br>Obart03g06450.1 0.005 0.415 0.41 0.359 1<br>Obart12g01440.2 0.434 0.989 0.555 0.003 0.961	1<br>Osjap12g01820.1 0.434 0.989 0.555 0.003 0.961<br>Osjap12g01870.1 0.002 0.408 0.406 0.341 0.93	1<br>Osind02g25820.1 0.434 0.989 0.555 0.003 0.961<br>Osind03g06850.1 0.005 0.415 0.41 0.359 1	1<br>Orufi03g27580.1 0.002 0.431 0.429 0.305 0.953<br>Orufi11g01580.1 0.434 0.989 0.555 0.003 0.961	0<br>Olong_024204-R1 0.002 0.409 0.407 0.36 0.998<br>Olong_028389-R1 0.659 0.989 0.33 0.008 0.936	1<br>Opunc11g01470.1 0.434 0.989 0.555 0.003 0.961<br>Opunc12g01520.9 0.002 0.397 0.395 0.371 0.992	1<br>Obrac03g18430.1 0.002 0.418 0.416 0.331 0.992<br>Obrac12g01300.1 0.434 0.989 0.555 0.003 0.961	1<br>Lperr03g19970.1 0.002 0.418 0.416 0.335 0.98<br>Lperr11g01360.1 0.434 0.989 0.555 0.003 0.962	1<br>AT1G33090.1 0.003 0.432 0.429 0.34 1<br>AT5G21060.2 0.446 0.989 0.543 0.011 0.968
Oglab11g01510.5	1<br>Oglab11g01510.1 0.002 1 0.998 0.14 1<br>Oglab11g01510.2 0.002 1 0.998 0.202 1<br>Oglab11g01510.3 0.002 1 0.998 0.203 1<br>Oglab11g01510.4 0.002 1 0.998 0.002 1<br>Oglab11g01510.5 0.002 1 0.998 0.002 1	1<br>Obart11g01710.1 0.002 0.994 0.992 0.104 0.922<br>Obart11g01710.2 0.002 0.994 0.992 0.106 0.921<br>Obart11g01710.3 0.002 0.994 0.992 0.115 0.914<br>Obart11g01710.4 0.002 0.994 0.992 0.117 0.913	1<br>Osjap11g01890.1 0.002 0.989 0.987 0.147 0.988<br>Osjap11g01890.2 0.002 1 0.998 0.143 1<br>Osjap11g01890.3 0.002 0.989 0.987 0.13 0.988<br>Osjap11g01890.4 0.002 0.989 0.987 0.149 0.988<br>Osjap11g01890.5 0.002 0.989 0.987 0.142 0.988<br>Osjap11g01890.6 0.002 1 0.998 0.146 1<br>Osjap11g01890.7 0.002 0.989 0.987 0.201 0.983	0	0	0	1<br>Opunc12g01520.7 0.002 0.989 0.987 0.23 0.981	0	1<br>Lperr12g01560.1 0.002 0.989 0.987 0.13 0.989	0
	1<br>Oglab12g01390.1 0.43 0.989 0.559 0.003 0.961<br>Oglab12g01400.2 0.002 0.404 0.402 0.291 0.961	1<br>Obart03g06450.1 0.005 0.412 0.407 0.359 1<br>Obart12g01440.2 0.43 0.989 0.559 0.003 0.961	1<br>Osjap12g01820.1 0.43 0.989 0.559 0.003 0.961<br>Osjap12g01870.1 0.002 0.404 0.402 0.341 0.93	1<br>Osind02g25820.1 0.43 0.989 0.559 0.003 0.961<br>Osind03g06850.1 0.005 0.412 0.407 0.359 1	1<br>Orufi03g24390.1 0.002 0.415 0.413 0.36 0.979<br>Orufi11g01580.1 0.43 0.989 0.559 0.003 0.961	0<br>Olong_024204-R1 0.002 0.406 0.404 0.36 0.998<br>Olong_028389-R1 0.657 0.989 0.332 0.008 0.936	1<br>Opunc11g01470.1 0.43 0.989 0.559 0.003 0.961<br>Opunc12g01520.9 0.002 0.394 0.392 0.371 0.992	1<br>Obrac03g18430.1 0.002 0.415 0.413 0.331 0.992<br>Obrac12g01300.1 0.43 0.989 0.559 0.003 0.961	1<br>Lperr03g05800.1 0.005 0.412 0.407 0.38 1<br>Lperr11g01360.1 0.43 0.989 0.559 0.003 0.962	1<br>AT1G33090.1 0.003 0.429 0.426 0.34 1<br>AT5G21060.2 0.442 0.989 0.547 0.011 0.968
Oglab11g02200.1	1<br>Oglab11g02200.1 0.002 1 0.998 0.002 1<br>Oglab12g02130.1 0.002 1 0.998 0.002 1	1<br>Obart12g02080.1 0.023 1 0.977 0.002 1	1<br>Osjap11g02640.1 0.002 1 0.998 0.002 1<br>Osjap12g02610.1 0.002 1 0.998 0.002 1	1<br>Osind11g01990.1 0.002 1 0.998 0.002 1<br>Osind12g02780.1 0.002 1 0.998 0.002 1	1<br>Orufi11g02320.1 0.002 1 0.998 0.003 1<br>Orufi12g02480.1 0.002 1 0.998 0.002 1	0	0	0	0	0
	1<br>Oglab07g03810.1 0.032 0.657 0.625 0.019 0.987<br>Oglab12g09880.1 0.66 0.951 0.291 0.051 0.385	0<br>Obart11g02350.1 0.021 0.657 0.636 0.004 0.993	1<br>Osjap07g04360.1 0.032 0.657 0.625 0.018 0.925<br>Osjap12g12870.1 0.66 0.951 0.291 0.055 0.417	1<br>Osind11g02010.1 0.013 0.657 0.644 0.001 0.994<br>Osind12g10020.1 0.66 0.951 0.291 0.055 0.417	1<br>Orufi07g03650.1 0.032 0.657 0.625 0.019 0.976<br>Orufi12g10750.1 0.658 0.951 0.293 0.045 0.364	1<br>Olong_012584-R1 0.064 0.611 0.547 0.002 0.965<br>Olong_017316-R1 0.658 0.951 0.293 0.045 0.365	1<br>Opunc11g02190.1 0.002 0.657 0.655 0.003 0.989<br>Opunc12g08740.1 0.658 0.951 0.293 0.04 0.324	1<br>Obrac05g10860.1 0.049 0.657 0.608 0.097 0.987<br>Obrac12g07170.1 0.658 0.942 0.284 0.071 0.57	1<br>Lperr12g02190.1 0.002 0.657 0.655 0.003 0.989<br>Lperr12g08370.1 0.657 0.951 0.294 0.047 0.391	0<br>AT5G27320.1 0.013 0.632 0.619 0.003 0.98
Oglab11g05440.1	1<br>Oglab11g05440.1 0.001 1 0.999 0.001 1	1<br>Obart11g05910.1 0.001 0.875 0.874 0.05 1	1<br>Osjap11g06710.1 0.001 0.998 0.997 0.067 0.993<br>Osjap11g06720.1 0.001 0.998 0.997 0.054 0.977	1<br>Osind09g09040.1 0.001 0.998 0.997 0.001 0.995	1<br>Orufi11g05930.1 0.001 0.998 0.997 0.09 0.992<br>Orufi11g05940.1 0.001 1 0.999 0.002 0.995	0	0	0	0	0
	1<br>Oglab08g10820.1 0.526 0.955 0.429 0.015 0.867<br>Oglab11g05700.1 0.001 0.51 0.509 0.001 0.966	1<br>Obart01g20930.1 0.525 0.992 0.467 0.006 0.498<br>Obart11g05930.1 0.001 0.502 0.501 0.002 0.998	1<br>Osjap01g24690.1 0.525 0.992 0.467 0.014 0.934<br>Osjap11g06740.8 0.024 0.509 0.485 0.013 0.579	1<br>Osind09g09050.1 0.001 0.502 0.501 0.104 0.994<br>Osind11g05650.1 0.521 0.998 0.477 0.002 0.989	1<br>Orufi11g05890.1 0.521 0.998 0.477 0.003 0.984<br>Orufi11g05900.2 0.001 0.509 0.508 0.002 0.991	1<br>Olong_005786-R3 0.007 0.511 0.504 0.006 0.948<br>Olong_005787-R1 0.521 0.998 0.477 0.002 0.989	0<br>Opunc11g05960.1 0.443 0.998 0.555 0.002 0.991	1<br>Obrac01g15800.1 0.52 0.992 0.472 0.004 0.915<br>Obrac11g03930.1 0.001 0.504 0.503 0.002 0.941	1<br>Lperr01g17850.1 0.521 0.992 0.471 0.002 0.973<br>Lperr11g05360.1 0.001 0.502 0.501 0.044 0.995	0<br>AT4G38130.1 0.521 0.955 0.434 0.002 0.782
Oglab11g06530.1	1<br>Oglab11g06530.1 0.01 1 0.99 0.01 1	0	0	1<br>Osind11g06680.1 0.01 0.942 0.932 0.004 0.411	1<br>Orufi11g07410.1 0.01 0.942 0.932 0.005 0.516	0	0	0	0	0

	1<br>Oglab01g35920.1 0.01 0.612 0.602 0.002 0.144<br>Oglab08g00300.1 0.612 0.981 0.369 0.487 0.537	1<br>Obart01g41090.1 0.01 0.612 0.602 0.002 0.144<br>Obart04g30900.2 0.612 0.981 0.369 0.451 0.513	1<br>Osjap01g46770.1 0.01 0.612 0.602 0.002 0.144<br>Osjap08g00940.1 0.612 0.981 0.369 0.487 0.537	1<br>Osind01g47980.1 0.01 0.612 0.602 0.002 0.142<br>Osind04g02960.1 0.612 0.981 0.369 0.501 0.562	1<br>Orufi01g44430.1 0.01 0.612 0.602 0.002 0.14<br>Orufi08g00930.1 0.612 0.981 0.369 0.487 0.537	0<br>Olong_019421-R2 0.398 0.981 0.583 0.431 0.523	1<br>Opunc01g39900.1 0.01 0.612 0.602 0.002 0.159<br>Opunc04g28500.1 0.612 0.981 0.369 0.467 0.523	0<br>Obrac12g04550.1 0.398 0.981 0.583 0.719 0.769	0<br>Lperr12g05120.1 0.388 0.981 0.593 0.461 0.561	0<br>AT1G57700.1 0.33 0.981 0.651 0.422 0.527
Oglab11g14350.2	1<br>Oglab11g14350.1 0.001 0.967 0.966 0.001 1<br>Oglab11g14350.2 0.001 1 0.999 0.001 1	1<br>Obart11g16070.1 0.001 1 0.999 0.013 1	0	0	0	0	0	0	0	0
	1<br>Oglab02g07220.2 0.597 1 0.403 0.003 1<br>Oglab11g18510.1 0.032 0.552 0.52 0.029 1	1<br>Obart02g07160.1 0.597 1 0.403 0.002 1<br>Obart08g10830.1 0.036 0.55 0.514 0.004 0.944	1<br>Osjap02g07570.1 0.597 1 0.403 0.002 1<br>Osjap11g18290.1 0.001 0.531 0.53 0.514 0.989	1<br>Osind11g13470.1 0.001 0.496 0.495 0.416 0.992<br>Osind11g13490.1 0.595 1 0.405 0.081 1	1<br>Orufi11g17130.1 0.001 0.531 0.53 0.522 0.989<br>Orufi11g17160.1 0.597 1 0.403 0.002 1	1<br>Olong_012209-R1 0.597 1 0.403 0.002 1<br>Olong_021538-R1 0.028 0.559 0.531 0.006 0.801	1<br>Opunc11g13170.1 0.597 1 0.403 0.002 1<br>Opunc11g17530.1 0.026 0.545 0.519 0.009 0.97	1<br>Obrac02g05260.1 0.597 1 0.403 0.002 1<br>Obrac10g05860.1 0.038 0.536 0.498 0.005 0.973	1<br>Lperr11g17450.1 0.034 0.537 0.503 0.003 0.875<br>Lperr12g06590.1 0.568 1 0.432 0.061 1	0<br>AT2G44350.2 0.597 1 0.403 0.002 0.998<br>AT5G54820.1 0.038 0.401 0.363 0.015 0.788
Oglab11g18520.1	1<br>Oglab11g18520.1 0.001 1 0.999 0.001 1	0	0	0	1<br>Orufi11g22790.1 0.013 0.977 0.964 0.006 0.971	0	0	0	0	0
	1<br>Oglab11g18510.1 0.013 0.512 0.499 0.037 0.942<br>Oglab11g18550.1 0.54 1 0.46 0.002 1	1<br>Obart11g20950.1 0.013 0.512 0.499 0.003 0.945<br>Obart11g20980.1 0.54 1 0.46 0.003 1	1<br>Osjap11g24530.1 0.013 0.536 0.523 0.038 0.993<br>Osjap11g24540.1 0.54 1 0.46 0.002 1	1<br>Osind06g03750.1 0.54 1 0.46 0.002 1<br>Osind10g01290.1 0.017 0.501 0.484 0.035 0.99	1<br>Orufi04g30270.1 0.54 0.992 0.452 0.002 0.984<br>Orufi11g22780.1 0.009 0.498 0.489 0.018 0.881	1<br>Olong_021538-R1 0.013 0.514 0.501 0.011 0.957<br>Olong_027904-R1 0.579 0.989 0.41 0.108 0.991	1<br>Opunc03g06070.1 0.54 0.992 0.452 0.002 0.98<br>Opunc11g13180.1 0.013 0.498 0.485 0.169 0.909	1<br>Obrac03g05030.1 0.54 0.992 0.452 0.003 0.967<br>Obrac11g09310.1 0.005 0.498 0.493 0.025 0.932	1<br>Lperr09g14070.1 0.54 0.991 0.451 0.007 1<br>Lperr11g12540.1 0.005 0.496 0.491 0.013 0.892	0<br>AT5G23940.1 0.547 0.991 0.444 0.012 0.921
Oglab11g20100.2	1<br>Oglab11g20100.1 0.001 0.998 0.997 0 0.998<br>Oglab11g20100.2 0.001 1 0.999 0.001 1	1<br>Obart11g23070.1 0.001 1 0.999 0.022 1	0	0	0	0	1<br>Opunc11g19450.1 0.001 0.979 0.978 0.001 0.995	0	0	0
	1<br>Oglab02g25180.1 0.548 0.976 0.428 0.006 0.938<br>Oglab05g19280.1 0.001 0.537 0.536 0.001 0.81	1<br>Obart02g27080.1 0.548 0.976 0.428 0.006 0.938<br>Obart11g22940.1 0.001 0.536 0.535 0.146 0.791	0<br>Osjap05g22990.2 0.001 0.544 0.543 0.001 0.886	1<br>Osind02g30490.1 0.548 0.98 0.432 0.006 1<br>Osind05g23630.1 0.001 0.544 0.543 0.001 0.886	1<br>Orufi02g28510.2 0.548 0.98 0.432 0.007 1<br>Orufi05g21610.1 0.001 0.536 0.535 0.001 0.97	1<br>Olong_005582-R1 0.546 0.986 0.44 0.145 0.995<br>Olong_026752-R1 0.001 0.539 0.538 0.001 0.973	1<br>Opunc02g24700.1 0.553 0.98 0.427 0.003 1<br>Opunc05g18050.1 0.001 0.536 0.535 0.001 0.848	1<br>Obrac02g19610.1 0.547 0.976 0.429 0.001 0.983<br>Obrac05g13790.3 0.001 0.53 0.529 0.092 0.901	0<br>Lperr11g19460.1 0.001 0.535 0.534 0.005 0.813	0<br>AT2G45910.1 0.585 0.976 0.391 0.054 0.994
Oglab12g00050.1	1<br>Oglab12g00050.1 0.003 1 0.997 0.003 1	0	0	0	0	0	0	0	0	0
	1<br>Oglab01g30880.1 0.335 0.911 0.576 0.03 0.464<br>Oglab11g00420.3 0.003 0.298 0.295 0.002 0.735	1<br>Obart12g00130.1 0.325 0.945 0.62 0.002 0.509<br>Obart12g00140.5 0.003 0.298 0.295 0.004 0.46	1<br>Osjap11g00430.1 0.003 0.298 0.295 0.004 0.425<br>Osjap12g00360.1 0.325 0.945 0.62 0.002 0.506	1<br>Osind12g00440.1 0.325 0.945 0.62 0.002 0.506<br>Osind12g00580.1 0.003 0.298 0.295 0.004 0.425	0<br>Orufi12g00380.1 0.325 0.945 0.62 0.002 0.506	1<br>Olong_014020-R1 0.359 0.937 0.578 0.055 0.353<br>Olong_016703-R1 0.003 0.298 0.295 0.004 0.423	1<br>Opunc05g17450.1 0.335 0.927 0.592 0.029 0.496<br>Opunc11g00340.1 0.003 0.291 0.288 0.007 0.696	0<br>Obrac05g13350.2 0.144 0.911 0.767 0.033 0.562	1<br>Lperr11g00380.1 0.003 0.298 0.295 0.004 0.481<br>Lperr12g00140.1 0.33 0.982 0.652 0.016 0.62	1<br>AT1G25260.1 0.003 0.293 0.29 0.004 0.409<br>AT3G24290.1 0.359 0.937 0.578 0.093 0.496
Oglab12g00800.1	1<br>Oglab12g00800.1 0.001 1 0.999 0.001 1	1<br>Obart11g01060.1 0.001 1 0.999 0.001 1	1<br>Osjap11g01220.1 0.001 1 0.999 0.001 1<br>Osjap12g01140.1 0.001 1 0.999 0.001 1	1<br>Osind11g00690.1 0.001 0.956 0.955 0.001 0.999<br>Osind12g01290.1 0.001 1 0.999 0.001 1	1<br>Orufi11g01000.1 0.001 1 0.999 0.001 1<br>Orufi12g01130.1 0.001 1 0.999 0.001 1	0	1<br>Opunc12g00940.1 0.001 1 0.999 0.001 1	0	0	0
	1<br>Oglab03g12120.1 0.49 0.983 0.493 0.121 0.633<br>Oglab07g22980.1 0.006 0.393 0.387 0.007 0.933	1<br>Obart03g12120.1 0.49 0.983 0.493 0.121 0.633<br>Obart12g00790.1 0.001 0.393 0.392 0.002 0.942	1<br>Osjap03g13100.1 0.49 0.983 0.493 0.097 0.622<br>Osjap07g30390.1 0.009 0.393 0.384 0.013 0.938	1<br>Osind03g12930.1 0.49 0.983 0.493 0.121 0.633<br>Osind07g27070.1 0.006 0.393 0.387 0.006 0.939	1<br>Orufi03g17220.1 0.496 1 0.504 0.027 1<br>Orufi07g27420.1 0.009 0.393 0.384 0.013 0.939	1<br>Olong_007715-R1 0.493 1 0.507 0.007 1<br>Olong_008211-R1 0.006 0.393 0.387 0.006 0.939	1<br>Opunc03g11680.1 0.49 1 0.51 0.148 1<br>Opunc11g00920.1 0.001 0.393 0.392 0.142 0.945	1<br>Obrac03g09720.2 0.49 1 0.51 0.142 1<br>Obrac06g16520.1 0.02 0.411 0.391 0.048 0.978	1<br>Lperr03g10720.1 0.49 0.996 0.506 0.148 0.954<br>Lperr11g00920.1 0.001 0.393 0.392 0.002 0.942	1<br>AT3G46740.1 0.493 1 0.507 0.161 1<br>AT4G20110.2 0.001 0.393 0.392 0.002 0.939
Oglab12g01430.1	1<br>Oglab12g01430.1 0.001 1 0.999 0.001 1	1<br>Obart12g01480.1 0.001 0.996 0.995 0.001 0.986	0	0	0	0	0	0	0	0

	1<br>Oglab01g17070.1 0.001 0.479 0.478 0.05 0.856<br>Oglab12g01740.1 0.587 1 0.413 0.007 0.993	1<br>Obart01g05330.1 0.123 0.598 0.475 0.101 0.965<br>Obart02g36960.1 0.606 0.996 0.39 0.27 0.993	1<br>Osjap01g23560.1 0.001 0.558 0.557 0.04 0.822<br>Osjap12g02170.3 0.587 1 0.413 0.007 1	1<br>Osind12g02430.1 0.001 0.587 0.586 0.002 0.95<br>Osind12g02460.1 0.587 1 0.413 0.006 0.994	1<br>Orufi01g22500.1 0.001 0.558 0.557 0.049 0.912<br>Orufi12g02140.1 0.587 1 0.413 0.007 0.993	1<br>Olong_015804-R1 0.001 0.59 0.589 0.002 0.976<br>Olong_027205-R1 0.592 0.996 0.404 0.004 0.84	1<br>Opunc02g34070.1 0.595 0.996 0.401 0.218 0.996<br>Opunc08g00050.1 0.001 0.479 0.478 0.03 0.777	1<br>Obrac03g08440.1 0.136 0.595 0.459 0.179 0.989<br>Obrac12g01540.1 0.606 0.996 0.39 0.017 0.983	0<br>Lperr02g30420.7 0.608 0.996 0.388 0.02 0.984<br>Lperr03g07390.1 0.174 0.562 0.388 0.175 0.868	0<br>AT2G02250.1 0.537 0.996 0.459 0.003 0.974
Oglab12g01890.1	1<br>Oglab12g01890.1 0.002 1 0.998 0.002 1	1<br>Obart12g01910.1 0.002 0.974 0.972 0.002 0.938	0	0	0	0	1<br>Opunc11g02000.1 0.002 1 0.998 0.002 0.907<br>Opunc12g01920.1 0.002 1 0.998 0.002 0.978	1<br>Obrac11g01380.1 0.023 0.972 0.949 0.036 0.95	0	0
	1<br>Oglab07g23480.1 0.737 0.998 0.261 0.002 0.386<br>Oglab11g01930.1 0.013 0.718 0.705 0.051 0.947	1<br>Obart07g26900.1 0.737 0.998 0.261 0.002 0.386<br>Obart11g02130.1 0.013 0.718 0.705 0.051 0.947	1<br>Osjap11g02310.2 0.739 1 0.261 0.006 1<br>Osjap12g02360.1 0.002 0.718 0.716 0.002 0.945	1<br>Osind07g27540.1 0.737 0.998 0.261 0.002 0.386<br>Osind12g02620.1 0.002 0.718 0.716 0.002 0.945	1<br>Orufi11g02050.1 0.739 1 0.261 0.006 1<br>Orufi12g02190.1 0.023 0.622 0.599 0.159 1	1<br>Olong_013437-R2 0.739 0.997 0.258 0.007 0.809<br>Olong_019069-R2 0.002 0.718 0.716 0.002 0.945	0<br>Opunc07g25270.1 0.737 0.998 0.261 0.003 0.43	1<br>Obrac01g08350.1 0.732 0.977 0.245 0.007 1<br>Obrac12g01700.2 0.002 0.718 0.716 0.002 0.948	1<br>Lperr03g13480.1 0.732 0.977 0.245 0.007 1<br>Lperr07g20930.1 0.026 0.718 0.692 0.09 0.956	1<br>AT2G19540.1 0.023 0.718 0.695 0.062 0.947<br>AT3G43810.1 0.732 0.977 0.245 0.007 1
Oglab12g02130.1	1<br>Oglab11g02200.1 0.002 1 0.998 0.002 1<br>Oglab12g02130.1 0.002 1 0.998 0.002 1	1<br>Obart12g02080.1 0.022 1 0.978 0.002 1	1<br>Osjap11g02640.1 0.002 1 0.998 0.002 1<br>Osjap12g02610.1 0.002 1 0.998 0.002 1	1<br>Osind11g01990.1 0.002 1 0.998 0.002 1<br>Osind12g02780.1 0.002 1 0.998 0.002 1	1<br>Orufi11g02320.1 0.002 1 0.998 0.003 1<br>Orufi12g02480.1 0.002 1 0.998 0.002 1	0	0	0	0	0
	1<br>Oglab07g03810.1 0.031 0.64 0.609 0.019 0.987<br>Oglab12g09880.1 0.651 0.953 0.302 0.02 0.385	0<br>Obart11g02350.1 0.02 0.64 0.62 0.004 0.993	1<br>Osjap07g04360.1 0.031 0.64 0.609 0.018 0.925<br>Osjap12g12870.1 0.651 0.953 0.302 0.022 0.417	1<br>Osind11g02010.1 0.013 0.64 0.627 0.001 0.994<br>Osind12g10020.1 0.651 0.953 0.302 0.022 0.417	0<br>Orufi07g03640.1 0.047 0.655 0.608 0.028 0.997	1<br>Olong_012584-R1 0.061 0.597 0.536 0.002 0.965<br>Olong_017316-R1 0.651 0.953 0.302 0.017 0.365	1<br>Opunc11g02190.1 0.002 0.64 0.638 0.003 0.989<br>Opunc12g08740.1 0.651 0.953 0.302 0.015 0.324	1<br>Obrac05g10860.1 0.047 0.64 0.593 0.097 0.987<br>Obrac12g07170.1 0.651 0.944 0.293 0.029 0.57	1<br>Lperr12g02190.1 0.002 0.64 0.638 0.003 0.989<br>Lperr12g08380.1 0.644 0.987 0.343 0.01 0.488	0<br>AT5G16080.1 0.047 0.644 0.597 0.081 0.994
Oglab12g02960.1	1<br>Oglab12g02960.1 0.001 1 0.999 0.001 1	0	0	0	0	0	1<br>Opunc12g02670.1 0.001 1 0.999 0.001 1	0	0	0
	1<br>Oglab06g24980.2 0.005 0.757 0.752 0.153 0.821<br>Oglab11g03020.1 0.782 1 0.218 0.005 1	1<br>Obart06g27500.1 0.005 0.757 0.752 0.152 0.823<br>Obart12g02850.1 0.782 1 0.218 0.008 1	1<br>Osjap11g03380.1 0.782 1 0.218 0.005 1<br>Osjap12g03480.1 0.001 0.738 0.737 0.002 0.965	1<br>Osind06g31110.1 0.005 0.757 0.752 0.152 0.823<br>Osind12g03520.1 0.782 1 0.218 0.008 1	1<br>Orufi12g03250.1 0.782 1 0.218 0.008 1<br>Orufi12g03260.1 0.001 0.738 0.737 0.002 0.965	1<br>Olong_004443-R1 0.782 1 0.218 0.01 1<br>Olong_023554-R1 0.005 0.757 0.752 0.125 0.818	1<br>Opunc06g25170.3 0.005 0.757 0.752 0.09 0.809<br>Opunc11g02740.1 0.782 1 0.218 0.005 1	1<br>Obrac06g19580.3 0.005 0.757 0.752 0.149 0.822<br>Obrac11g01880.1 0.782 0.997 0.215 0.01 0.99	1<br>Lperr01g19770.1 0.782 1 0.218 0.008 1<br>Lperr03g35610.1 0.012 0.763 0.751 0.026 0.955	0<br>AT5G04550.1 0.009 0.731 0.722 0.032 0.97
Oglab12g03070.1	1<br>Oglab12g03070.1 0.001 1 0.999 0.001 1	1<br>Obart12g02950.1 0.001 1 0.999 0.001 1	0	1<br>Osind12g03620.1 0.001 0.999 0.998 0.029 0.961	1<br>Orufi12g03350.1 0.012 0.999 0.987 0.044 0.966<br>Orufi12g03350.2 0.012 0.999 0.987 0.043 0.966	1<br>Olong_000549-R1 0.012 0.999 0.987 0.011 0.967	0	0	0	0
	1<br>Oglab10g13340.3 0.546 0.99 0.444 0.213 0.951<br>Oglab12g02020.1 0.002 0.437 0.435 0.001 0.388	0<br>Obart11g02240.1 0.198 0.437 0.239 0.014 0.272<br>Obart11g21270.1 0.479 0.984 0.505 0.138 0.958	1<br>Osjap10g20010.1 0.483 0.99 0.507 0.022 0.946<br>Osjap12g02500.1 0.002 0.437 0.435 0.001 0.388	1<br>Osind01g01580.1 0.477 0.984 0.507 0.171 0.96<br>Osind12g02670.1 0.002 0.437 0.435 0.001 0.396	1<br>Orufi04g25720.1 0.483 0.867 0.384 0.002 0.207<br>Orufi11g02210.1 0.016 0.437 0.421 0.015 0.492	0<br>Olong_010277-R4 0.473 0.867 0.394 0.001 0.93<br>Olong_027241-R1 0.047 0.287 0.24 0.003 0.866	1<br>Opunc11g17890.2 0.476 0.984 0.508 0.189 0.952<br>Opunc12g02040.1 0.012 0.437 0.425 0.009 0.402	1<br>Obrac11g01490.1 0.002 0.437 0.435 0.029 0.403<br>Obrac11g12600.2 0.534 0.984 0.45 0.046 0.748	0<br>Lperr11g01950.1 0.012 0.235 0.223 0.015 0.297<br>Lperr11g17840.1 0.486 0.984 0.498 0.046 0.945	1<br>AT1G65810.1 0.483 0.867 0.384 0.021 0.596<br>AT2G26780.1 0.017 0.433 0.416 0.011 0.388
Oglab12g06650.1	1<br>Oglab12g06650.1 0.001 1 0.999 0.001 1	0	0	0	0	0	1<br>Opunc12g06220.1 0.01 1 0.99 0.004 1	0	0	0
	0<br>Oglab01g38670.1 0.784 0.993 0.209 0.076 0.968	1<br>Obart12g06540.1 0.795 1 0.205 0.035 1<br>Obart12g06550.1 0.058 0.795 0.737 0.09 0.962	1<br>Osjap01g49850.1 0.784 0.993 0.209 0.027 0.337<br>Osjap12g08640.3 0.026 0.752 0.726 0.12 0.985	1<br>Osind01g51390.1 0.784 0.993 0.209 0.076 0.968<br>Osind12g07610.1 0.026 0.752 0.726 0.037 0.985	1<br>Orufi01g47370.1 0.784 0.993 0.209 0.029 0.359<br>Orufi12g07360.1 0.026 0.752 0.726 0.037 0.985	1<br>Olong_017083-R1 0.793 0.999 0.206 0.373 1<br>Olong_026612-R3 0.078 0.752 0.674 0.067 0.981	0<br>Opunc11g02000.1 0.727 0.988 0.261 0.59 0.878	1<br>Obrac04g10690.1 0.793 0.999 0.206 0.375 1<br>Obrac12g04840.4 0.013 0.752 0.739 0.014 0.986	1<br>Lperr04g13760.1 0.793 0.999 0.206 0.321 1<br>Lperr12g05750.1 0.001 0.752 0.751 0.002 0.986	1<br>AT2G20210.1 0.082 0.749 0.667 0.053 0.98<br>AT3G07490.1 0.789 0.999 0.21 0.007 0.98
Oglab12g09470.1	1<br>Oglab12g09470.1 0.001 1 0.999 0.001 1	1<br>Obart12g09410.1 0.001 1 0.999 0.001 1	1<br>Osjap12g12320.1 0.001 1 0.999 0.001 1	1<br>Osind12g09530.1 0.001 1 0.999 0.001 1	1<br>Orufi12g10460.1 0.001 1 0.999 0.001 1	0	0	0	0	0

	1<br>Oglab01g34040.1 0.489 0.998 0.509 0.203 0.995<br>Oglab03g07280.1 0.001 0.295 0.294 0.004 0.981	1<br>Obart01g39260.3 0.456 0.998 0.542 0.041 0.994<br>Obart03g07210.1 0.001 0.295 0.294 0.004 0.981	1<br>Osjap01g44780.2 0.44 0.998 0.558 0.095 0.994<br>Osjap03g07600.1 0.001 0.295 0.294 0.004 0.981	1<br>Osind01g46040.1 0.44 0.998 0.558 0.151 0.995<br>Osind03g07670.1 0.001 0.295 0.294 0.004 0.981	1<br>Orufi01g42650.3 0.44 0.998 0.558 0.195 0.995<br>Orufi03g07180.1 0.001 0.295 0.294 0.004 0.981	1<br>Olong_021912-R1 0.457 1 0.543 0.099 1<br>Olong_021913-R1 0.001 0.296 0.295 0.004 0.988	1<br>Opunc12g08570.1 0.326 1 0.674 0.052 1<br>Opunc12g08580.1 0.001 0.296 0.295 0.004 0.988	1<br>Obrac03g05880.1 0.001 0.296 0.295 0.004 0.988<br>Obrac12g06500.1 0.461 1 0.539 0.103 1	1<br>Lperr01g33810.2 0.456 0.998 0.542 0.002 0.994<br>Lperr12g08300.1 0.001 0.296 0.295 0.004 0.985	0<br>AT4G34670.1 0.001 0.29 0.289 0.004 0.966<br>AT5G67540.2 0.489 0.993 0.504 0.053 1
Oglab12g11360.1	1<br>Oglab12g11360.1 0.005 1 0.995 0.005 1	0	0	0	0	1<br>Olong_021020-R1 0.005 1 0.995 0.005 1	0	0	0	0
	1<br>Oglab07g16860.1 0.491 1 0.509 0.207 1<br>Oglab12g09200.1 0.014 0.326 0.312 0.787 0.962	0<br>Obart07g18590.1 0.491 1 0.509 0.207 1	1<br>Osjap12g11960.1 0.014 0.335 0.321 0.341 0.911<br>Osjap12g14980.1 0.45 1 0.55 0.016 1	1<br>Osind10g17000.1 0.073 0.408 0.335 0.853 0.978<br>Osind12g08720.1 0.491 1 0.509 0.152 1	1<br>Orufi05g05820.1 0.491 1 0.509 0.152 1<br>Orufi12g10130.1 0.014 0.413 0.399 0.304 0.913	1<br>Olong_015025-R1 0.486 1 0.514 0.111 1<br>Olong_019160-R2 0.028 0.326 0.298 0.39 0.949	0<br>Opunc02g32830.1 0.349 1 0.651 0.039 1	0<br>Obrac08g07610.1 0.491 0.821 0.33 0.157 0.971	0<br>Lperr05g07610.1 0.422 0.775 0.353 0.328 0.867	0
Oglab12g13410.1	1<br>Oglab12g13410.1 0.005 1 0.995 0.005 1	0	0	0	0	0	0	0	0	0
	0<br>Oglab05g23160.1 0.362 1 0.638 0.119 0.225	1<br>Obart01g26870.1 0.362 1 0.638 0.347 0.552<br>Obart04g05090.1 0.005 0.357 0.352 0.664 0.96	1<br>Osjap01g31140.1 0.362 1 0.638 0.327 0.539<br>Osjap04g06490.1 0.005 0.221 0.216 0.706 0.899	1<br>Osind01g32600.1 0.362 1 0.638 0.269 0.498<br>Osind11g12290.1 0.005 0.221 0.216 0.706 0.899	1<br>Orufi01g29880.1 0.362 1 0.638 0.323 0.536<br>Orufi10g18640.1 0.005 0.221 0.216 0.014 0.662	0<br>Olong_000367-R1 0.362 1 0.638 0.042 0.344	1<br>Opunc05g22640.1 0.362 1 0.638 0.127 0.237<br>Opunc12g12730.1 0.005 0.249 0.244 0.421 0.963	0<br>Obrac05g17590.1 0.362 1 0.638 0.042 0.344	0<br>Lperr05g20860.1 0.362 1 0.638 0.265 0.496	0<br>ATCG00480.1 0.357 1 0.643 0.006 0.438

(((((_c1,longi),punctata),brachy),leersia),tair);

chimeric gene id	INGROUP					MIDGROUP		OUTGROUP		
	jap	indica	rufi	glab	barthii	longi	punctata	brachy	leersia	tair
Oglab01g06470.1	1<br>Osjap01g08660.1 0.001 1 0.999 0.001 1	1<br>Osind01g08490.1 0.001 1 0.999 0.001 1	1<br>Orufi01g08070.1 0.001 1 0.999 0.001 1	1<br>Oglab01g06470.1 0.001 1 0.999 0.001 1	1<br>Obart01g07430.1 0.02 1 0.98 0.013 1	1<br>Olong_011567-R1 0.056 1 0.944 0.001 1	0	0	0	0
	1<br>Osjap01g29230.2 0.1 0.581 0.481 0.25 0.983<br>Osjap05g00560.2 0.647 0.997 0.35 0.146 1	1<br>Osind01g30690.1 0.1 0.581 0.481 0.267 0.982<br>Osind05g00540.1 0.647 0.997 0.35 0.126 1	1<br>Orufi01g27950.1 0.1 0.581 0.481 0.25 0.983<br>Orufi01g48560.2 0.647 0.98 0.333 0.085 0.984	1<br>Oglab01g21070.2 0.1 0.581 0.481 0.249 0.983<br>Oglab05g00530.1 0.647 0.997 0.35 0.146 1	1<br>Obart01g25090.1 0.1 0.581 0.481 0.219 0.982<br>Obart05g00540.1 0.647 0.997 0.35 0.146 1	0<br>Olong_003039-R1 0.647 0.997 0.35 0.128 1<br>Olong_003692-R2 0.139 0.581 0.442 0.244 0.979	1<br>Opunc01g07170.1 0.001 0.582 0.581 0.001 0.984<br>Opunc05g00510.2 0.647 0.997 0.35 0.15 1	1<br>Obrac01g05760.3 0.583 1 0.417 0.082 1<br>Obrac01g05770.3 0.001 0.582 0.581 0.001 0.985	1<br>Lperr01g07030.1 0.583 1 0.417 0.018 1<br>Lperr01g07040.2 0.001 0.582 0.581 0.054 0.989	1<br>AT1G54350.1 0.585 0.993 0.408 0.084 0.975<br>AT5G53430.1 0.071 0.581 0.51 0.164 0.984
Oglab02g00240.1	1<br>Osjap02g00350.2 0.193 1 0.807 0.001 1	0	1<br>Orufi02g00370.1 0.019 0.959 0.94 0.382 0.979<br>Orufi02g00370.2 0.019 0.959 0.94 0.405 0.98	1<br>Oglab02g00240.1 0.001 1 0.999 0.001 1	1<br>Obart02g00330.1 0.001 1 0.999 0.011 1	1<br>Olong_001783-R1 0.066 0.948 0.882 0.001 0.991	0	1<br>Obrac02g00300.1 0.155 0.983 0.828 0.005 0.995<br>Obrac02g00300.2 0.155 0.983 0.828 0.005 0.995	0	0
	1<br>Osjap02g00350.3 0.206 1 0.794 0.004 1<br>Osjap02g00360.1 0.001 0.204 0.203 0.052 1	1<br>Osind02g00340.1 0.213 1 0.787 0.001 1<br>Osind02g00350.1 0.001 0.204 0.203 0.052 1	1<br>Orufi04g16100.1 0.22 0.99 0.77 0.023 0.994<br>Orufi09g15710.1 0.001 0.203 0.202 0.053 0.995	1<br>Oglab04g13000.1 0.22 0.99 0.77 0.023 0.994<br>Oglab09g13130.1 0.001 0.203 0.202 0.053 0.995	0<br>Obart04g14710.1 0.22 0.99 0.77 0.025 0.994	0<br>Olong_004740-R1 0.212 0.992 0.78 0.004 0.996	1<br>Opunc02g00010.2 0.001 0.204 0.203 0.052 1<br>Opunc11g05460.1 0.217 1 0.783 0.002 1	1<br>Obrac09g06000.3 0.216 0.999 0.783 0.007 0.975<br>Obrac09g08720.1 0.001 0.203 0.202 0.053 0.995	1<br>Lperr02g00330.1 0.214 1 0.786 0.001 1<br>Lperr02g00340.1 0.001 0.204 0.203 0.053 1	1<br>AT1G33140.1 0.001 0.203 0.202 0.052 0.995<br>AT3G30340.1 0.22 0.996 0.776 0.027 0.995
Oglab02g22130.2	1<br>Osjap02g26750.1 0.001 0.969 0.968 0.001 0.994<br>Osjap02g26750.2 0.001 0.969 0.968 0.001 0.994<br>Osjap02g26750.4 0.001 0.969 0.968 0.001 0.799	1<br>Osind02g26640.2 0.001 1 0.999 0.001 1	1<br>Orufi02g24900.1 0.011 0.974 0.963 0.06 0.859<br>Orufi02g24900.3 0.001 0.974 0.973 0.001 0.74<br>Orufi02g24900.4 0.001 0.974 0.973 0.001 0.737	1<br>Oglab02g22130.2 0.001 1 0.999 0.001 1	1<br>Obart02g23460.2 0.001 1 0.999 0.001 1	0	1<br>Opunc02g21410.1 0.011 0.999 0.988 0.116 0.996<br>Opunc02g21410.2 0.011 0.999 0.988 0.114 0.996<br>Opunc02g21410.3 0.011 0.999 0.988 0.115 0.996<br>Opunc02g21410.4 0.011 0.999 0.988 0.114 0.996	0	1<br>Lperr02g18970.1 0.011 0.997 0.986 0.032 0.996	0
	1	1	1	1	1	0	1	1	1	1

	Osjap02g26590.1 0.475 0.999 0.524 0.033 0.999<br>Osjap02g26750.5 0.011 0.459 0.448 0.238 0.973	Osind02g26450.1 0.475 0.999 0.524 0.025 0.997<br>Osind02g26620.1 0.011 0.459 0.448 0.495 0.981	Orufi02g24730.1 0.475 0.994 0.519 0.031 0.996<br>Orufi02g24880.2 0.011 0.459 0.448 0.18 0.97	Oglab02g21950.1 0.475 0.999 0.524 0.073 0.995<br>Oglab02g22110.2 0.011 0.459 0.448 0.532 0.98	Obart02g23290.1 0.475 0.999 0.524 0.029 0.999<br>Obart02g23440.1 0.011 0.459 0.448 0.492 0.981	Olong_024345-R1 0.011 0.474 0.463 0.442 0.991	Opunc02g21230.1 0.479 0.976 0.497 0.003 0.934<br>Opunc02g21240.1 0.011 0.461 0.45 0.121 0.972	Obrac02g16670.1 0.549 0.973 0.424 0.076 0.995<br>Obrac02g16700.1 0.011 0.461 0.45 0.114 0.947	Lperr02g18740.1 0.485 0.997 0.512 0.056 0.991<br>Lperr02g19120.1 0.011 0.484 0.473 0.183 0.978	AT4G31860.1 0.014 0.45 0.436 0.123 0.989<br>AT5G43080.1 0.626 1 0.374 0.194 0.932
Oglab02g33340.1	1<br>Osjap02g40310.1 0.042 0.973 0.931 0.432 0.926<br>Osjap02g40340.1 0.042 1 0.958 0.361 1<br>Osjap02g40370.1 0.042 0.973 0.931 0.267 0.726	1<br>Osind02g39860.1 0.042 0.977 0.935 0.257 0.659<br>Osind02g39860.2 0.069 0.977 0.908 0.058 0.571<br>Osind02g39860.3 0.069 0.973 0.904 0.061 0.631<br>Osind02g39860.4 0.069 0.973 0.904 0.066 0.679	1<br>Orufi02g37680.1 0.069 0.989 0.92 0.067 0.86<br>Orufi02g37680.10 0.069 0.989 0.92 0.091 0.811<br>Orufi02g37680.2 0.069 0.989 0.92 0.07 0.854<br>Orufi02g37680.3 0.069 0.989 0.92 0.076 0.895<br>Orufi02g37680.4 0.069 0.989 0.92 0.073 0.849<br>Orufi02g37680.5 0.069 0.989 0.92 0.076 0.841<br>Orufi02g37680.6 0.069 0.989 0.92 0.088 0.879<br>Orufi02g37680.7 0.069 0.973 0.904 0.079 0.855<br>Orufi02g37680.9 0.069 0.973 0.904 0.086 0.843	1<br>Oglab02g33340.1 0.004 1 0.996 0.004 1	1<br>Obart02g36180.1 0.069 0.989 0.92 0.061 0.778<br>Obart02g36180.2 0.069 0.989 0.92 0.073 0.734<br>Obart02g36180.3 0.069 0.989 0.92 0.076 0.724	0	0	0	0	0
	1<br>Osjap02g41540.1 0.483 0.992 0.509 0.434 0.804<br>Osjap05g23970.1 0.061 0.387 0.326 0.669 0.821	1<br>Osind02g41040.1 0.483 0.992 0.509 0.459 0.813<br>Osind05g24480.1 0.061 0.387 0.326 0.588 0.777	0<br>Orufi02g37680.8 0.372 0.989 0.617 0.159 0.801	1<br>Oglab02g34400.1 0.483 0.992 0.509 0.434 0.804<br>Oglab03g25400.1 0.073 0.41 0.337 0.475 0.707	1<br>Obart02g37290.1 0.483 0.992 0.509 0.434 0.804<br>Obart05g21320.1 0.061 0.387 0.326 0.588 0.777	1<br>Olong_011081-R1 0.479 1 0.521 0.05 1<br>Olong_025373-R1 0.073 0.41 0.337 0.236 0.574	1<br>Opunc02g34450.1 0.483 0.992 0.509 0.432 0.803<br>Opunc05g18980.1 0.061 0.383 0.322 0.588 0.771	1<br>Obrac02g27500.6 0.483 0.992 0.509 0.432 0.803<br>Obrac05g14470.1 0.061 0.383 0.322 0.588 0.771	1<br>Lperr02g30750.1 0.483 0.992 0.509 0.453 0.811<br>Lperr04g17770.1 0.038 0.383 0.345 0.48 0.678	1<br>AT1G16330.1 0.038 0.414 0.376 0.701 0.873<br>AT2G20360.1 0.483 0.992 0.509 0.423 0.799
Oglab03g00390.1	1<br>Osjap03g00680.1 0.001 1 0.999 0.001 1<br>Osjap03g00680.2 0.001 1 0.999 0.001 1<br>Osjap03g00680.3 0.001 1 0.999 0.001 1	1<br>Osind03g00740.1 0.001 0.994 0.993 0.001 0.996	1<br>Orufi03g12660.1 0.001 1 0.999 0.001 1<br>Orufi03g12660.2 0.001 1 0.999 0.001 1	1<br>Oglab03g00390.1 0.001 1 0.999 0.001 1	1<br>Obart03g00550.1 0.001 0.994 0.993 0.001 0.996	0	0	0	0	0
	1<br>Osjap02g42090.1 0.049 0.634 0.585 0.065 1<br>Osjap04g17560.1 0.696 0.998 0.302 0.466 0.996	1<br>Osind02g41670.1 0.058 0.634 0.576 0.131 1<br>Osind03g02220.1 0.652 1 0.348 0.015 1	1<br>Orufi02g39350.1 0.058 0.634 0.576 0.131 1<br>Orufi09g02220.1 0.696 1 0.304 0.072 1	1<br>Oglab02g34920.1 0.058 0.634 0.576 0.131 1<br>Oglab09g02040.2 0.696 1 0.304 0.07 1	1<br>Obart03g02140.1 0.652 1 0.348 0.013 1<br>Obart09g20260.1 0.152 0.634 0.482 0.12 1	0<br>Olong_013175-R1 0.253 0.906 0.653 0.002 0.961	1<br>Opunc03g00470.1 0.001 0.73 0.729 0.002 0.996<br>Opunc11g04590.1 0.783 0.998 0.215 0.023 1	0<br>Obrac02g10260.1 0.736 0.999 0.263 0.286 0.995<br>Obrac02g27970.1 0.128 0.634 0.506 0.105 1	1<br>Lperr03g00570.1 0.001 0.634 0.633 0.002 1<br>Lperr12g09110.1 0.699 0.998 0.299 0.295 0.967	0<br>AT5G04460.1 0.017 0.634 0.617 0.081 1
Oglab03g12120.1	1<br>Osjap03g13100.1 0.028 1 0.972 0.001 1	1<br>Osind03g12930.1 0.001 1 0.999 0.001 1	0	1<br>Oglab03g12120.1 0.001 1 0.999 0.001 1	1<br>Obart03g12120.1 0.001 1 0.999 0.001 1	0	0	0	0	0
	1<br>Osjap04g16240.1 0.642 0.998 0.356 0.067 0.921<br>Osjap12g01140.1 0.109 0.633 0.524 0.498 0.981	1<br>Osind04g14790.1 0.642 0.998 0.356 0.013 0.911<br>Osind12g01290.1 0.109 0.633 0.524 0.49 0.981	1<br>Orufi06g01460.3 0.642 0.998 0.356 0.212 0.912<br>Orufi12g01130.1 0.109 0.633 0.524 0.498 0.981	1<br>Oglab06g01620.1 0.642 0.998 0.356 0.212 0.912<br>Oglab12g00800.1 0.114 0.633 0.519 0.483 0.983	1<br>Obart04g15560.1 0.64 0.998 0.358 0.146 0.877<br>Obart11g01060.1 0.114 0.633 0.519 0.471 0.982	1<br>Olong_007714-R9 0.642 1 0.358 0.002 1<br>Olong_007715-R1 0.125 0.633 0.508 0.007 0.965	1<br>Opunc03g11680.1 0.028 0.633 0.605 0.001 0.971<br>Opunc04g11270.1 0.642 0.998 0.356 0.067 0.921	1<br>Obrac03g09720.1 0.025 0.633 0.608 0.036 0.971<br>Obrac04g09430.1 0.64 0.998 0.358 0.014 0.863	1<br>Lperr03g10720.1 0.028 0.633 0.605 0.001 0.934<br>Lperr03g17470.1 0.633 0.998 0.365 0.119 0.828	1<br>AT3G46740.1 0.122 0.633 0.511 0.158 0.971<br>AT3G53360.1 0.672 0.998 0.326 0.09 0.934
Oglab03g30830.1	1<br>Osjap03g34330.1 0.001 1 0.999 0.001 1	1<br>Osind03g35550.1 0.001 1 0.999 0.039 1	1<br>Orufi03g33060.1 0.001 1 0.999 0.001 1	1<br>Oglab03g30830.1 0.001 1 0.999 0.001 1	1<br>Obart03g31750.1 0.001 1 0.999 0.046 1	0	0	0	0	0
	1<br>Osjap03g39110.1 0.652 1 0.348 0.036 1<br>Osjap10g05370.1 0.012 0.583 0.571 0.1 0.963	1<br>Osind01g46540.1 0.652 0.999 0.347 0.065 0.994<br>Osind10g03770.1 0.012 0.583 0.571 0.11 0.96	1<br>Orufi10g04850.1 0.029 0.583 0.554 0.093 0.963<br>Orufi11g12520.1 0.653 1 0.347 0.038 0.991	1<br>Oglab03g34460.1 0.629 0.997 0.368 0.016 0.993<br>Oglab10g04340.1 0.012 0.583 0.571 0.1 0.963	1<br>Obart03g36200.1 0.652 1 0.348 0.016 0.997<br>Obart10g04600.1 0.013 0.583 0.57 0.106 0.962	1<br>Olong_014796-R1 0.001 0.495 0.494 0.002 0.95<br>Olong_024872-R1 0.648 1 0.352 0.01 1	1<br>Opunc01g42030.1 0.652 1 0.348 0.28 0.872<br>Opunc03g28940.2 0.001 0.6 0.599 0.002 0.995	1<br>Obrac02g16280.1 0.646 0.982 0.336 0.063 0.949<br>Obrac03g23440.1 0.001 0.6 0.599 0.002 0.995	1<br>Lperr03g26400.1 0.001 0.6 0.599 0.002 0.995<br>Lperr03g30520.1 0.644 1 0.356 0.003 0.997	0<br>AT1G70730.3 0.001 0.6 0.599 0.118 0.995
Oglab03g32170.1	1<br>Osjap03g36400.1 0.003 1 0.997 0.003 1	1<br>Osind03g37600.1 0.003 1 0.997 0.003 1	1<br>Orufi03g35150.1 0.003 1 0.997 0.003 1	1<br>Oglab03g32170.1 0.003 1 0.997 0.003 1<br>Oglab03g32170.2 0.003 1 0.997 0.003 1	1<br>Obart03g33760.1 0.003 1 0.997 0.003 1	1<br>Olong_002225-R1 0.003 1 0.997 0.136 1<br>Olong_002225-R2 0.003 1 0.997 0.003 1	1<br>Opunc03g30920.1 0.003 1 0.997 0.003 1	0	0	0

	1<br>Osjap01g12980.2 0.547 0.979 0.432 0.032 0.961<br>Osjap03g04690.1 0.003 0.414 0.411 0.007 1	1<br>Osind03g04960.1 0.003 0.414 0.411 0.007 1<br>Osind12g02610.1 0.538 0.991 0.453 0.005 0.759	1<br>Orufi03g04350.1 0.003 0.414 0.411 0.007 1<br>Orufi05g22410.2 0.547 0.997 0.45 0.031 0.969	0<br>Oglab05g19940.1 0.32 0.991 0.671 0.015 0.997	1<br>Obart01g34520.1 0.547 0.991 0.444 0.027 0.818<br>Obart03g04650.1 0.003 0.414 0.411 0.007 1
Oglab03g32170.2	1<br>Osjap03g36400.1 0.003 1 0.997 0.003 1	1<br>Osind03g37600.1 0.003 1 0.997 0.003 1	1<br>Orufi03g35150.1 0.003 1 0.997 0.003 1	1<br>Oglab03g32170.1 0.003 1 0.997 0.003 1<br>Oglab03g32170.2 0.003 1 0.997 0.003 1	1<br>Obart03g33760.1 0.003 1 0.997 0.003 1
	1<br>Osjap01g12980.2 0.547 0.979 0.432 0.032 0.961<br>Osjap03g04690.1 0.003 0.414 0.411 0.007 1	1<br>Osind03g04960.1 0.003 0.414 0.411 0.007 1<br>Osind12g02610.1 0.538 0.991 0.453 0.005 0.759	1<br>Orufi03g04350.1 0.003 0.414 0.411 0.007 1<br>Orufi05g22410.2 0.547 0.997 0.45 0.031 0.969	0<br>Oglab05g19940.1 0.32 0.991 0.671 0.015 0.997	1<br>Obart01g34520.1 0.547 0.991 0.444 0.027 0.818<br>Obart03g04650.1 0.003 0.414 0.411 0.007 1
Oglab05g02190.1	1<br>Osjap05g02530.1 0.079 1 0.921 0.002 1	1<br>Osind05g02510.1 0.079 0.998 0.919 0.002 0.987	0	1<br>Oglab05g02190.1 0.001 1 0.999 0.001 1	1<br>Obart05g02340.1 0.001 1 0.999 0.001 1
	1<br>Osjap11g00710.1 0.553 0.997 0.444 0.034 0.911<br>Osjap12g14320.1 0.084 0.441 0.357 0.575 0.91	1<br>Osind07g26860.1 0.487 0.997 0.51 0.035 1<br>Osind12g11780.1 0.03 0.433 0.403 0.012 0.947	1<br>Orufi05g02460.2 0.451 1 0.549 0.003 0.982<br>Orufi07g25010.2 0.082 0.427 0.345 0.007 0.691	0<br>Oglab03g00660.1 0.608 0.994 0.386 0.321 0.996<br>Oglab05g07310.1 0.082 0.433 0.351 0.013 0.935	1<br>Obart06g15630.1 0.079 0.433 0.354 0.004 0.955<br>Obart11g00590.1 0.501 0.977 0.476 0.146 0.917
Oglab05g03490.1	1<br>Osjap05g04120.1 0.001 1 0.999 0.001 1<br>Osjap05g04120.2 0.001 1 0.999 0.001 1	1<br>Osind05g04070.1 0.008 1 0.992 0.001 1<br>Osind05g04070.2 0.008 1 0.992 0.001 1	1<br>Orufi05g03950.1 0.001 1 0.999 0.001 1<br>Orufi05g03950.2 0.001 1 0.999 0.001 1	1<br>Oglab05g03490.1 0.001 1 0.999 0.001 1<br>Oglab05g03490.2 0.001 1 0.999 0.001 1	1<br>Obart05g03830.1 0.001 1 0.999 0.001 1<br>Obart05g03830.2 0.001 1 0.999 0.001 1
	1<br>Osjap01g27410.1 0.09 0.723 0.633 0.066 0.996<br>Osjap03g15140.5 0.743 1 0.257 0.061 1	1<br>Osind01g28630.1 0.09 0.723 0.633 0.066 0.996<br>Osind03g15230.1 0.79 1 0.21 0.03 1	1<br>Orufi01g26130.1 0.09 0.723 0.633 0.066 0.996<br>Orufi03g14720.1 0.79 1 0.21 0.03 1	1<br>Oglab01g19490.1 0.09 0.723 0.633 0.066 0.996<br>Oglab03g14060.1 0.79 1 0.21 0.03 1	1<br>Obart01g23310.1 0.09 0.723 0.633 0.066 0.996<br>Obart03g14050.1 0.79 1 0.21 0.03 1
Oglab05g03490.2	1<br>Osjap05g04120.1 0.001 1 0.999 0.001 1<br>Osjap05g04120.2 0.001 1 0.999 0.001 1	1<br>Osind05g04070.1 0.008 1 0.992 0.001 1<br>Osind05g04070.2 0.008 1 0.992 0.001 1	1<br>Orufi05g03950.1 0.001 1 0.999 0.001 1<br>Orufi05g03950.2 0.001 1 0.999 0.001 1	1<br>Oglab05g03490.1 0.001 1 0.999 0.001 1<br>Oglab05g03490.2 0.001 1 0.999 0.001 1	1<br>Obart05g03830.1 0.001 1 0.999 0.001 1<br>Obart05g03830.2 0.001 1 0.999 0.001 1
	1<br>Osjap01g27410.1 0.089 0.726 0.637 0.066 0.996<br>Osjap03g15140.5 0.747 1 0.253 0.061 1	1<br>Osind01g28630.1 0.089 0.726 0.637 0.066 0.996<br>Osind03g15230.1 0.792 1 0.208 0.03 1	1<br>Orufi01g26130.1 0.089 0.726 0.637 0.066 0.996<br>Orufi03g14720.1 0.792 1 0.208 0.03 1	1<br>Oglab01g19490.1 0.089 0.726 0.637 0.066 0.996<br>Oglab03g14060.1 0.792 1 0.208 0.03 1	1<br>Obart01g23310.1 0.089 0.726 0.637 0.066 0.996<br>Obart03g14050.1 0.792 1 0.208 0.03 1
Oglab05g23160.1	1<br>Osjap05g28230.1 0.001 1 0.999 0.001 1	1<br>Osind05g28520.1 0.001 1 0.999 0.001 1	1<br>Orufi05g26870.1 0.001 1 0.999 0.001 1	1<br>Oglab05g23160.1 0.001 1 0.999 0.001 1	1<br>Obart05g25070.1 0.001 1 0.999 0.001 1
	1<br>Osjap01g31140.1 0.007 0.451 0.444 0.093 0.995<br>Osjap01g31150.1 0.462 0.999 0.537 0.009 0.999	1<br>Osind01g32600.1 0.001 0.451 0.45 0.002 0.995<br>Osind01g32610.1 0.462 0.999 0.537 0.009 0.999	1<br>Orufi01g29880.1 0.001 0.451 0.45 0.075 0.995<br>Orufi01g29890.1 0.462 0.999 0.537 0.009 0.999	1<br>Oglab01g22740.1 0.028 0.451 0.423 0.061 0.995<br>Oglab01g22750.1 0.462 0.999 0.537 0.009 0.999	1<br>Obart01g26870.1 0.006 0.451 0.445 0.117 0.995<br>Obart01g26880.1 0.462 0.999 0.537 0.009 0.999
Oglab06g00840.1	1<br>Osjap06g00940.1 0.04 1 0.96 0.085 1	0	1<br>Orufi06g00860.1 0.067 1 0.933 0.021 1	1<br>Oglab06g00840.1 0.001 1 0.999 0.001 1	1<br>Obart06g00880.1 0.001 1 0.999 0.001 1

	1<br>Olong_020510-R2 0.523 0.991 0.468 0.291 0.873<br>Olong_025165-R1 0.003 0.414 0.411 0.007 1	0<br>Opunc12g01920.1 0.526 0.973 0.447 0.663 0.947	0<br>Obrac03g25020.5 0.003 0.535 0.532 0.439 0.972	1<br>Lperr01g29830.2 0.532 0.991 0.459 0.203 0.858<br>Lperr03g28250.1 0.003 0.414 0.411 0.007 1	1<br>AT1G52740.1 0.003 0.414 0.411 0.007 1<br>AT4G14640.1 0.544 0.979 0.435 0.033 0.987
Oglab03g32170.2	1<br>Olong_002225-R1 0.003 1 0.997 0.136 1<br>Olong_002225-R2 0.003 1 0.997 0.003 1	1<br>Opunc03g30920.1 0.003 1 0.997 0.003 1	0	0	0
	1<br>Olong_020510-R2 0.523 0.991 0.468 0.291 0.873<br>Olong_025165-R1 0.003 0.414 0.411 0.007 1	0<br>Opunc12g01920.1 0.526 0.973 0.447 0.663 0.947	0<br>Obrac03g25020.5 0.003 0.535 0.532 0.439 0.972	1<br>Lperr01g29830.2 0.532 0.991 0.459 0.203 0.858<br>Lperr03g28250.1 0.003 0.414 0.411 0.007 1	1<br>AT1G52740.1 0.003 0.414 0.411 0.007 1<br>AT4G14640.1 0.544 0.979 0.435 0.033 0.987
Oglab05g02190.1	0	0	0	0	0
	1<br>Olong_016162-R1 0.079 0.433 0.354 0.004 0.954<br>Olong_026687-R1 0.451 1 0.549 0.09 0.983	1<br>Opunc05g02220.2 0.451 1 0.549 0.003 0.981<br>Opunc07g20770.2 0.082 0.432 0.35 0.009 0.832	1<br>Obrac05g01840.1 0.451 0.998 0.547 0.169 1<br>Obrac06g15870.1 0.082 0.434 0.352 0.015 0.84	1<br>Lperr05g02100.1 0.451 0.998 0.547 0.017 0.978<br>Lperr11g18770.1 0.027 0.304 0.277 0.358 0.699	0<br>AT5G15380.1 0.468 0.992 0.524 0.064 0.987
Oglab05g03490.1	1<br>Olong_016739-R1 0.074 1 0.926 0.001 1	1<br>Opunc05g03540.1 0.001 1 0.999 0.001 1<br>Opunc05g03540.2 0.001 1 0.999 0.001 1	1<br>Obrac05g02930.1 0.074 1 0.926 0.001 1	0	0
	1<br>Olong_003347-R2 0.09 0.723 0.633 0.047 0.996<br>Olong_009943-R1 0.79 1 0.21 0.03 1	1<br>Opunc01g23460.1 0.09 0.723 0.633 0.064 0.996<br>Opunc03g13590.1 0.79 1 0.21 0.002 0.545	1<br>Obrac01g17590.1 0.09 0.723 0.633 0.067 0.996<br>Obrac03g11330.1 0.79 1 0.21 0.03 1	0<br>Lperr05g03460.1 0.001 0.721 0.72 0.001 0.993	1<br>AT2G47580.1 0.775 1 0.225 0.008 1<br>AT3G16630.1 0.003 0.721 0.718 0.031 0.992
Oglab05g03490.2	1<br>Olong_016739-R1 0.073 1 0.927 0.001 1	1<br>Opunc05g03540.1 0.001 1 0.999 0.001 1<br>Opunc05g03540.2 0.001 1 0.999 0.001 1	1<br>Obrac05g02930.1 0.073 1 0.927 0.001 1	0	0
	1<br>Olong_003347-R2 0.089 0.726 0.637 0.047 0.996<br>Olong_009943-R1 0.792 1 0.208 0.03 1	1<br>Opunc01g23460.1 0.089 0.726 0.637 0.064 0.996<br>Opunc03g13590.1 0.792 1 0.208 0.002 0.545	1<br>Obrac01g17590.1 0.089 0.726 0.637 0.067 0.996<br>Obrac03g11330.1 0.792 1 0.208 0.03 1	0<br>Lperr05g03460.1 0.001 0.725 0.724 0.001 0.993	1<br>AT2G47580.1 0.778 1 0.222 0.008 1<br>AT3G16630.1 0.003 0.725 0.722 0.031 0.992
Oglab05g23160.1	0	1<br>Opunc05g22640.1 0.001 1 0.999 0.001 1	0	1<br>Lperr01g22990.1 0.001 1 0.999 0.027 1	0
	1<br>Olong_000367-R1 0.105 0.451 0.346 0.002 0.993<br>Olong_000368-R1 0.456 0.999 0.543 0.108 0.999	1<br>Opunc01g26820.1 0.049 0.451 0.402 0.002 0.994<br>Opunc01g26830.1 0.462 1 0.538 0.009 1	1<br>Obrac01g20400.1 0.048 0.451 0.403 0.006 0.994<br>Obrac05g17560.2 0.451 1 0.549 0.164 1	1<br>Lperr05g20850.2 0.467 1 0.533 0.002 1<br>Lperr05g20860.1 0.001 0.451 0.45 0.002 0.995	1<br>AT5G08690.1 0.012 0.451 0.439 0.034 0.995<br>AT5G51600.1 0.462 0.999 0.537 0.008 0.996
Oglab06g00840.1	0	0	0	0	0

	1<br>Osjap06g00950.1 0.529 0.992 0.463 0.132 0.99<br>Osjap08g13790.1 0.051 0.458 0.407 0.005 1	1<br>Osind06g00910.1 0.526 1 0.474 0.068 1<br>Osind08g15520.1 0.051 0.458 0.407 0.005 1	1<br>Orufi06g00690.1 0.529 1 0.471 0.099 1<br>Orufi06g00860.2 0.001 0.445 0.444 0.005 0.98	1<br>Oglab06g00730.1 0.526 0.99 0.464 0.257 0.968<br>Oglab07g19660.1 0.033 0.458 0.425 0.142 0.938	1<br>Obart06g00690.1 0.529 1 0.471 0.099 1<br>Obart09g03180.1 0.021 0.458 0.437 0.145 1	0<br>Olong_023831-R1 0.053 0.583 0.53 0.001 0.373	1<br>Opunc06g00800.1 0.457 1 0.543 0.025 1<br>Opunc06g00830.1 0.05 0.457 0.407 0.006 1	1<br>Obrac06g00660.1 0.526 1 0.474 0.034 0.999<br>Obrac07g14790.1 0.058 0.503 0.445 0.093 0.948	1<br>Lperr01g40030.1 0.055 0.457 0.402 0.013 0.994<br>Lperr06g00740.1 0.531 0.999 0.468 0.078 0.997	0<br>AT4G34470.1 0.063 0.457 0.394 0.013 1
Oglab06g02870.1	1<br>Osjap06g03080.1 0.001 1 0.999 0.001 1	0	1<br>Orufi06g02670.1 0.045 0.964 0.919 0.032 0.91	1<br>Oglab06g02870.1 0.001 1 0.999 0.001 1	1<br>Obart06g02710.1 0.001 1 0.999 0.03 1	1<br>Olong_013693-R1 0.045 1 0.955 0.069 1	0	0	0	0
	1<br>Osjap01g37620.1 0.001 0.756 0.755 0.033 0.998<br>Osjap04g34350.1 0.767 0.968 0.201 0.466 0.888	1<br>Osind03g45110.1 0.04 0.75 0.71 0.141 0.984<br>Osind04g33620.1 0.767 0.968 0.201 0.438 0.875	0<br>Orufi01g36040.3 0.037 0.756 0.719 0.03 0.998	1<br>Oglab01g27580.1 0.037 0.715 0.678 0.03 0.949<br>Oglab04g28300.1 0.767 0.968 0.201 0.466 0.888	1<br>Obart01g06310.1 0.037 0.756 0.719 0.066 0.997<br>Obart04g30240.1 0.767 0.968 0.201 0.466 0.888	1<br>Olong_001861-R1 0.767 0.968 0.201 0.381 0.87<br>Olong_005208-R1 0.014 0.715 0.701 0.141 0.969	1<br>Opunc03g00730.1 0.001 0.756 0.755 0.004 1<br>Opunc04g27750.1 0.767 0.968 0.201 0.426 0.879	1<br>Obrac04g05640.6 0.014 0.705 0.691 0.262 0.995<br>Obrac04g21390.1 0.767 0.968 0.201 0.381 0.87	1<br>Lperr10g07060.1 0.04 0.722 0.682 0.115 0.964<br>Lperr12g00960.1 0.767 0.968 0.201 0.389 0.871	0<br>AT2G37990.1 0.767 0.968 0.201 0.425 0.893
Oglab06g24190.1	1<br>Osjap06g31210.1 0.001 0.995 0.994 0.008 1	1<br>Osind06g30430.1 0.001 0.995 0.994 0.008 1	1<br>Orufi06g28720.1 0.001 0.995 0.994 0.008 1	1<br>Oglab06g24190.1 0.001 1 0.999 0.001 1<br>Oglab06g24190.2 0.001 0.995 0.994 0.001 0.991	1<br>Obart06g26750.1 0.001 0.995 0.994 0.008 1	1<br>Olong_013658-R1 0.001 0.995 0.994 0.001 1	1<br>Opunc06g24460.1 0.001 0.995 0.994 0.008 1	0	1<br>Lperr06g22350.1 0.001 0.995 0.994 0.033 1	0
	1<br>Osjap02g03010.1 0.694 0.993 0.299 0.006 0.928<br>Osjap12g19610.1 0.001 0.62 0.619 0.001 0.675	1<br>Osind02g03310.1 0.694 0.993 0.299 0.006 0.928<br>Osind07g26390.1 0.001 0.59 0.589 0.074 0.935	1<br>Orufi02g02960.1 0.694 0.993 0.299 0.006 0.928<br>Orufi12g16970.1 0.001 0.62 0.619 0.001 0.674	1<br>Oglab02g02970.1 0.694 0.993 0.299 0.006 0.93<br>Oglab12g14250.1 0.001 0.62 0.619 0.001 0.743	1<br>Obart02g03050.1 0.694 0.993 0.299 0.006 0.93<br>Obart12g15260.1 0.001 0.602 0.601 0.001 0.686	1<br>Olong_017635-R1 0.694 0.993 0.299 0.006 0.93<br>Olong_024721-R1 0.01 0.608 0.598 0.05 0.932	1<br>Opunc02g02350.2 0.715 0.993 0.278 0.248 0.944<br>Opunc03g37840.1 0.001 0.622 0.621 0.047 0.706	1<br>Obrac02g02360.1 0.694 0.994 0.3 0.006 0.933<br>Obrac09g01040.1 0.001 0.591 0.59 0.026 0.872	1<br>Lperr02g02740.1 0.699 0.995 0.296 0.006 0.96<br>Lperr12g12430.1 0.001 0.602 0.601 0.001 0.854	1<br>AT3G10250.1 0.701 0.998 0.297 0.006 0.948<br>AT4G32430.1 0.005 0.61 0.605 0.051 0.976
Oglab06g24190.2	1<br>Osjap06g31210.1 0.001 0.991 0.99 0.008 1	1<br>Osind06g30430.1 0.001 0.991 0.99 0.008 1	1<br>Orufi06g28720.1 0.001 0.991 0.99 0.008 1	1<br>Oglab06g24190.1 0.001 0.991 0.99 0.001 0.995<br>Oglab06g24190.2 0.001 1 0.999 0.001 1	1<br>Obart06g26750.1 0.001 0.991 0.99 0.008 1	1<br>Olong_013658-R1 0.001 0.991 0.99 0.001 1	1<br>Opunc06g24460.1 0.001 0.991 0.99 0.008 1	0	1<br>Lperr06g22350.1 0.001 0.991 0.99 0.033 1	0
	1<br>Osjap02g03010.1 0.691 0.997 0.306 0.006 0.959<br>Osjap12g19610.1 0.001 0.617 0.616 0.001 0.675	1<br>Osind02g03310.1 0.691 0.997 0.306 0.006 0.959<br>Osind07g26390.1 0.001 0.587 0.586 0.074 0.935	1<br>Orufi02g02960.1 0.691 0.997 0.306 0.006 0.959<br>Orufi09g02510.1 0.001 0.589 0.588 0.014 0.762	1<br>Oglab02g02970.1 0.691 0.997 0.306 0.006 0.96<br>Oglab12g14250.1 0.001 0.617 0.616 0.001 0.743	1<br>Obart02g03050.1 0.691 0.997 0.306 0.006 0.96<br>Obart12g15260.1 0.001 0.617 0.616 0.001 0.686	1<br>Olong_017635-R1 0.691 0.997 0.306 0.006 0.96<br>Olong_024721-R1 0.01 0.605 0.595 0.05 0.932	1<br>Opunc02g02350.2 0.712 0.997 0.285 0.248 0.968<br>Opunc03g37840.1 0.001 0.619 0.618 0.047 0.706	1<br>Obrac02g02360.1 0.691 0.997 0.306 0.006 0.961<br>Obrac09g01040.1 0.001 0.588 0.587 0.026 0.872	1<br>Lperr02g02740.1 0.696 0.991 0.295 0.006 0.96<br>Lperr03g02630.1 0.001 0.584 0.583 0.105 0.84	1<br>AT3G10250.1 0.698 0.992 0.294 0.006 0.941<br>AT4G32430.1 0.005 0.607 0.602 0.051 0.976
Oglab06g25490.1	1<br>Osjap09g17800.1 0.024 1 0.976 0.113 0.974<br>Osjap09g17800.2 0.024 1 0.976 0.114 0.974<br>Osjap09g17800.3 0.024 0.997 0.973 0.187 0.947	1<br>Osind06g31620.1 0.001 1 0.999 0.001 1	1<br>Orufi06g29990.1 0.001 1 0.999 0.001 1<br>Orufi09g18790.1 0.024 1 0.976 0.107 0.97<br>Orufi09g18790.2 0.024 1 0.976 0.107 0.97<br>Orufi09g18790.3 0.024 1 0.976 0.108 0.969<br>Orufi09g18790.4 0.024 1 0.976 0.108 0.969<br>Orufi09g18790.5 0.024 0.976 0.952 0.17 0.937<br>Orufi09g18790.6 0.024 0.996 0.972 0.172 0.937	1<br>Oglab06g25490.1 0.001 1 0.999 0.001 1<br>Oglab09g15660.1 0.024 0.999 0.975 0.154 0.95	1<br>Obart06g28060.1 0.001 1 0.999 0.001 1<br>Obart09g17460.1 0.024 1 0.976 0.076 0.969<br>Obart09g17460.2 0.024 1 0.976 0.076 0.969	1<br>Olong_016700-R1 0.001 1 0.999 0.001 1	0	0	1<br>Lperr09g13380.1 0.084 0.982 0.898 0.166 0.951	0
	1<br>Osjap06g32830.1 0.427 1 0.573 0.085 1<br>Osjap06g32840.1 0.001 0.407 0.406 0.002 0.952	1<br>Osind04g24640.1 0.07 0.417 0.347 0.233 0.933<br>Osind06g30840.1 0.485 0.999 0.514 0.109 1	0<br>Orufi06g29150.4 0.485 0.999 0.514 0.122 1	1<br>Oglab02g01250.1 0.066 0.406 0.34 0.221 0.93<br>Oglab10g06880.1 0.427 1 0.573 0.099 1	1<br>Obart04g21770.1 0.07 0.417 0.347 0.232 0.933<br>Obart10g07310.1 0.427 1 0.573 0.099 1	1<br>Olong_000286-R1 0.066 0.406 0.34 0.258 0.928<br>Olong_026973-R2 0.427 1 0.573 0.099 1	1<br>Opunc06g25610.1 0.001 0.407 0.406 0.002 0.951<br>Opunc10g06480.1 0.427 0.998 0.571 0.098 0.984	1<br>Obrac06g20010.2 0.432 1 0.568 0.119 1<br>Obrac06g20020.1 0.001 0.407 0.406 0.002 0.952	1<br>Lperr02g22230.1 0.043 0.392 0.349 0.113 0.88<br>Lperr06g23510.1 0.402 1 0.598 0.074 1	1<br>AT1G76360.1 0.036 0.406 0.37 0.219 0.952<br>AT3G61130.1 0.465 0.999 0.534 0.207 1
Oglab08g05640.1	1<br>Osjap08g06650.1 0.002 1 0.998 0.002 1	1<br>Osind08g06790.1 0.002 1 0.998 0.002 1	1<br>Orufi08g06840.1 0.002 1 0.998 0.002 1	1<br>Oglab08g05640.1 0.002 1 0.998 0.002 1	1<br>Obart08g06070.1 0.002 1 0.998 0.002 1	0	1<br>Opunc08g05850.1 0.047 1 0.953 0.068 1	0	0	0
	1<br>Osjap04g21220.1 0.047 0.275 0.228 0.21 0.936	1<br>Osind04g20250.1 0.047 0.275 0.228 0.198 0.94	1<br>Orufi04g19250.2 0.047 0.275 0.228 0.21 0.936	1<br>Oglab02g26360.1 0.348 0.996 0.648 0.01 0.994	1<br>Obart02g28440.1 0.348 0.996 0.648 0.093 0.994	1<br>Olong_008030-R1 0.047 0.275 0.228 0.232 0.937	1<br>Opunc04g15550.1 0.047 0.275 0.228 0.206 0.938	1<br>Obrac08g04780.1 0.277 1 0.723 0.098 1	1<br>Lperr08g05690.1 0.277 1 0.723 0.1 1	1<br>AT1G05310.1 0.33 0.982 0.652 0.183 0.99

	Osjap09g20870.1 0.359 0.984 0.625 0.208 0.966	Osind09g20960.1 0.359 0.984 0.625 0.208 0.966	Orufi09g21960.1 0.359 0.984 0.625 0.2 0.965	Oglab04g16180.1 0.047 0.275 0.228 0.198 0.94	Obart04g17770.1 0.047 0.275 0.228 0.198 0.94	Olong_008031-R2 0.277 1 0.723 0.106 1	Opunc04g18740.1 0.295 0.984 0.689 0.088 0.994	Obrac08g04800.1 0.002 0.275 0.273 0.006 0.943	Lperr08g05700.1 0.002 0.275 0.273 0.006 0.943	AT4G30800.1 0.047 0.275 0.228 0.208 0.943
Oglab08g15520.1	1<br>Osjap01g09050.1 0.007 0.844 0.837 0.061 1	1<br>Osind01g08880.1 0.102 0.986 0.884 0.004 0.567	1<br>Orufi01g08450.1 0.007 0.973 0.966 0.026 0.487	1<br>Oglab01g06790.1 0.007 0.844 0.837 0.061 1<br>Oglab08g15520.1 0.007 1 0.993 0.007 1	1<br>Obart01g07700.1 0.007 0.844 0.837 0.061 1<br>Obart08g16670.1 0.007 0.993 0.986 0.007 0.993	1<br>Olong_003647-R1 0.007 0.83 0.823 0.061 0.985	0	0	0	0
	1<br>Osjap01g24690.1 0.007 0.694 0.687 0.104 0.321<br>Osjap07g09900.1 0.694 1 0.306 0.005 0.126	1<br>Osind07g08950.1 0.694 1 0.306 0.004 0.113<br>Osind11g05650.1 0.007 0.694 0.687 0.103 0.319	1<br>Orufi01g23650.1 0.007 0.694 0.687 0.106 0.307<br>Orufi07g08630.1 0.694 1 0.306 0.005 0.117	1<br>Oglab03g37410.1 0.714 1 0.286 0.049 0.141<br>Oglab11g05440.1 0.007 0.694 0.687 0.573 0.683	1<br>Obart07g08560.1 0.694 0.952 0.258 0.009 0.192<br>Obart11g05910.1 0.007 0.694 0.687 0.752 0.834	1<br>Olong_005787-R1 0.007 0.694 0.687 0.103 0.319<br>Olong_016241-R1 0.728 1 0.272 0.735 0.775	1<br>Opunc01g21000.1 0.007 0.694 0.687 0.114 0.334<br>Opunc07g08090.1 0.694 1 0.306 0.004 0.092	1<br>Obrac07g05110.1 0.728 0.993 0.265 0.077 0.151<br>Obrac11g03720.1 0.007 0.694 0.687 0.112 0.34	1<br>Lperr01g17850.1 0.007 0.694 0.687 0.105 0.313<br>Lperr07g08590.1 0.694 0.993 0.299 0.022 0.124	1<br>AT3G55700.1 0.728 0.973 0.245 0.015 0.093<br>AT4G38130.1 0.007 0.68 0.673 0.09 0.269
Oglab09g13510.1	1<br>Osjap09g15430.2 0.002 1 0.998 0.002 1<br>Osjap09g15430.3 0.002 1 0.998 0.002 1	1<br>Osind09g15570.1 0.002 1 0.998 0.002 1<br>Osind09g15570.2 0.002 1 0.998 0.002 1	1<br>Orufi09g16160.1 0.002 1 0.998 0.002 1<br>Orufi09g16160.2 0.002 1 0.998 0.002 1	1<br>Oglab09g13510.1 0.002 1 0.998 0.002 1<br>Oglab09g13510.2 0.002 1 0.998 0.002 1<br>Oglab09g13510.3 0.002 1 0.998 0.002 1<br>Oglab09g13510.4 0.002 1 0.998 0.002 1	1<br>Obart09g14980.1 0.002 1 0.998 0.002 1<br>Obart09g14980.2 0.002 1 0.998 0.002 1	0	1<br>Opunc09g13420.1 0.002 1 0.998 0.002 1<br>Opunc09g13420.2 0.002 1 0.998 0.002 1	1<br>Obrac09g09090.1 0.002 1 0.998 0.002 1	0	0
	1<br>Osjap02g06650.1 0.009 0.501 0.492 0.01 0.997<br>Osjap09g15430.1 0.514 1 0.486 0.003 1	1<br>Osind02g06940.1 0.009 0.501 0.492 0.01 0.997<br>Osind08g23920.1 0.519 0.994 0.475 0.012 0.988	1<br>Orufi04g24220.1 0.012 0.502 0.49 0.165 1<br>Orufi08g22880.1 0.519 0.994 0.475 0.012 0.988	1<br>Oglab08g19240.1 0.519 0.994 0.475 0.014 0.986<br>Oglab12g18680.1 0.002 0.501 0.499 0.003 0.997	1<br>Obart02g06180.1 0.009 0.501 0.492 0.01 0.997<br>Obart08g20390.1 0.501 0.994 0.493 0.566 0.988	1<br>Olong_018059-R1 0.518 0.992 0.474 0.046 0.957<br>Olong_024186-R1 0.002 0.501 0.499 0.003 0.997	1<br>Opunc02g05570.1 0.005 0.501 0.496 0.087 0.997<br>Opunc08g18560.1 0.519 0.994 0.475 0.012 0.969	0<br>Obrac08g13590.1 0.499 0.994 0.495 0.689 0.994	1<br>Lperr04g18350.1 0.002 0.501 0.499 0.021 0.997<br>Lperr08g17230.1 0.507 0.994 0.487 0.021 0.997	1<br>AT2G21270.3 0.003 0.501 0.498 0.059 0.997<br>AT5G42800.1 0.516 0.992 0.476 0.003 0.851
Oglab09g13510.2	1<br>Osjap09g15430.2 0.002 1 0.998 0.002 1<br>Osjap09g15430.3 0.002 1 0.998 0.002 1	1<br>Osind09g15570.1 0.002 1 0.998 0.002 1<br>Osind09g15570.2 0.002 1 0.998 0.002 1	1<br>Orufi09g16160.1 0.002 1 0.998 0.002 1<br>Orufi09g16160.2 0.002 1 0.998 0.002 1	1<br>Oglab09g13510.1 0.002 1 0.998 0.002 1<br>Oglab09g13510.2 0.002 1 0.998 0.002 1<br>Oglab09g13510.3 0.002 1 0.998 0.002 1<br>Oglab09g13510.4 0.002 1 0.998 0.002 1	1<br>Obart09g14980.1 0.002 1 0.998 0.002 1<br>Obart09g14980.2 0.002 1 0.998 0.002 1	0	1<br>Opunc09g13420.1 0.002 1 0.998 0.002 1<br>Opunc09g13420.2 0.002 1 0.998 0.002 1	1<br>Obrac09g09090.1 0.002 1 0.998 0.002 1	0	0
	1<br>Osjap02g06650.1 0.01 0.522 0.512 0.01 0.997<br>Osjap09g15430.1 0.537 1 0.463 0.003 1	1<br>Osind02g06940.1 0.01 0.522 0.512 0.01 0.997<br>Osind08g23920.1 0.541 0.994 0.453 0.012 0.988	1<br>Orufi04g24220.1 0.013 0.524 0.511 0.165 1<br>Orufi08g22880.1 0.541 0.994 0.453 0.012 0.988	1<br>Oglab08g19240.1 0.541 0.994 0.453 0.014 0.986<br>Oglab12g18680.1 0.002 0.522 0.52 0.003 0.997	1<br>Obart02g06180.1 0.01 0.522 0.512 0.01 0.997<br>Obart08g20390.1 0.522 0.994 0.472 0.566 0.988	1<br>Olong_014421-R1 0.537 0.992 0.455 0.002 0.984<br>Olong_024186-R1 0.002 0.522 0.52 0.003 0.997	1<br>Opunc02g05570.1 0.005 0.522 0.517 0.087 0.997<br>Opunc08g18560.1 0.541 0.994 0.453 0.012 0.969	1<br>Obrac02g04550.3 0.008 0.522 0.514 0.214 0.997<br>Obrac09g00560.1 0.54 0.992 0.452 0.043 0.954	1<br>Lperr04g18350.1 0.002 0.522 0.52 0.021 0.997<br>Lperr08g17230.1 0.529 0.994 0.465 0.021 0.997	1<br>AT1G15950.1 0.538 0.992 0.454 0.015 0.936<br>AT2G21270.3 0.003 0.522 0.519 0.059 0.997
Oglab09g13510.3	1<br>Osjap09g15430.2 0.002 1 0.998 0.002 1<br>Osjap09g15430.3 0.002 1 0.998 0.002 1	1<br>Osind09g15570.1 0.002 1 0.998 0.002 1<br>Osind09g15570.2 0.002 1 0.998 0.002 1	1<br>Orufi09g16160.1 0.002 1 0.998 0.002 1<br>Orufi09g16160.2 0.002 1 0.998 0.002 1	1<br>Oglab09g13510.1 0.002 1 0.998 0.002 1<br>Oglab09g13510.2 0.002 1 0.998 0.002 1<br>Oglab09g13510.3 0.002 1 0.998 0.002 1<br>Oglab09g13510.4 0.002 1 0.998 0.002 1	1<br>Obart09g14980.1 0.002 1 0.998 0.002 1<br>Obart09g14980.2 0.002 1 0.998 0.002 1	0	1<br>Opunc09g13420.1 0.002 1 0.998 0.002 1<br>Opunc09g13420.2 0.002 1 0.998 0.002 1	1<br>Obrac09g09090.1 0.002 1 0.998 0.002 1	0	0
	1<br>Osjap02g06650.1 0.01 0.54 0.53 0.01 0.997<br>Osjap09g15430.1 0.544 1 0.456 0.131 1	1<br>Osind02g06940.1 0.01 0.54 0.53 0.01 0.997<br>Osind08g23920.1 0.544 0.993 0.449 0.131 0.988	1<br>Orufi04g24220.1 0.013 0.542 0.529 0.165 1<br>Orufi08g22880.1 0.544 0.993 0.449 0.131 0.988	1<br>Oglab08g19240.1 0.544 0.993 0.449 0.143 0.986<br>Oglab12g18680.1 0.002 0.542 0.54 0.003 1	1<br>Obart02g06180.1 0.01 0.54 0.53 0.01 0.997<br>Obart08g20390.1 0.544 0.993 0.449 0.631 0.988	1<br>Olong_013054-R1 0.547 0.992 0.445 0.149 0.979<br>Olong_024186-R1 0.002 0.542 0.54 0.003 1	1<br>Opunc04g20190.1 0.007 0.542 0.535 0.006 1<br>Opunc08g18560.1 0.544 0.993 0.449 0.128 0.969	1<br>Obrac02g04550.3 0.008 0.54 0.532 0.214 0.997<br>Obrac09g00560.1 0.548 0.998 0.45 0.164 0.966	1<br>Lperr04g18350.1 0.002 0.542 0.54 0.021 1<br>Lperr09g11120.1 0.544 1 0.456 0.156 1	1<br>AT1G68540.1 0.542 0.992 0.45 0.112 1<br>AT2G21270.3 0.003 0.54 0.537 0.059 0.997
Oglab09g13510.4	1<br>Osjap09g15430.2 0.002 1 0.998 0.002 1<br>Osjap09g15430.3 0.002 1 0.998 0.002 1	1<br>Osind09g15570.1 0.002 1 0.998 0.002 1<br>Osind09g15570.2 0.002 1 0.998 0.002 1	1<br>Orufi09g16160.1 0.002 1 0.998 0.002 1<br>Orufi09g16160.2 0.002 1 0.998 0.002 1	1<br>Oglab09g13510.1 0.002 1 0.998 0.002 1<br>Oglab09g13510.2 0.002 1 0.998 0.002 1<br>Oglab09g13510.3 0.002 1 0.998 0.002 1	1<br>Obart09g14980.1 0.002 1 0.998 0.002 1<br>Obart09g14980.2 0.002 1 0.998 0.002 1	0	1<br>Opunc09g13420.1 0.002 1 0.998 0.002 1<br>Opunc09g13420.2 0.002 1 0.998 0.002 1	1<br>Obrac09g09090.1 0.002 1 0.998 0.002 1	0	0

				Oglab09g13510.4 0.002 1 0.998 0.002 1						
	1<br>Osjap02g06650.1 0.01 0.565 0.555 0.01 0.997<br>Osjap09g15430.1 0.569 1 0.431 0.131 1	1<br>Osind02g06940.1 0.01 0.565 0.555 0.01 0.997<br>Osind08g23920.1 0.569 0.993 0.424 0.131 0.988	1<br>Orufi04g24220.1 0.014 0.567 0.553 0.165 1<br>Orufi08g22880.1 0.569 0.993 0.424 0.131 0.988	1<br>Oglab08g19240.1 0.569 0.993 0.424 0.143 0.986<br>Oglab12g18680.1 0.002 0.567 0.565 0.003 1	1<br>Obart02g06180.1 0.01 0.565 0.555 0.01 0.997<br>Obart08g20390.1 0.569 0.993 0.424 0.631 0.988	1<br>Olong_008093-R2 0.596 0.991 0.395 0.25 0.996<br>Olong_024186-R1 0.002 0.567 0.565 0.003 1	1<br>Opunc04g20190.1 0.007 0.567 0.56 0.006 1<br>Opunc08g18560.1 0.569 0.993 0.424 0.128 0.969	1<br>Obrac01g09090.1 0.605 0.998 0.393 0.299 0.982<br>Obrac02g04550.3 0.009 0.565 0.556 0.214 0.997	1<br>Lperr04g18350.1 0.002 0.567 0.565 0.021 1<br>Lperr09g11120.1 0.569 1 0.431 0.156 1	1<br>AT1G68540.1 0.567 0.991 0.424 0.112 1<br>AT2G21270.3 0.003 0.565 0.562 0.059 0.997
Oglab09g18200.1	1<br>Osjap09g20460.1 0.001 0.999 0.998 0.001 0.999	1<br>Osind09g20560.1 0.001 0.985 0.984 0.001 0.998	1<br>Orufi09g21510.1 0.001 0.958 0.957 0.001 0.999	1<br>Oglab09g18200.1 0.001 1 0.999 0.001 1	1<br>Obart09g19980.1 0.066 1 0.934 0.181 1<br>Obart09g19980.2 0.001 1 0.999 0.001 1	1<br>Olong_014589-R1 0.116 0.972 0.856 0.032 0.991<br>Olong_014589-R2 0.116 0.972 0.856 0.033 0.991	0	0	0	0
	1<br>Osjap02g21530.2 0.023 0.609 0.586 0.096 0.943<br>Osjap09g20430.1 0.649 0.999 0.35 0.151 0.998	1<br>Osind02g21430.2 0.023 0.609 0.586 0.096 0.943<br>Osind09g20550.1 0.619 0.999 0.38 0.058 0.999	1<br>Orufi02g19810.2 0.023 0.609 0.586 0.096 0.943<br>Orufi09g21500.1 0.649 0.999 0.35 0.151 0.998	1<br>Oglab02g17850.1 0.023 0.609 0.586 0.095 0.914<br>Oglab09g18170.1 0.619 0.999 0.38 0.13 0.998	1<br>Obart04g25370.1 0.047 0.617 0.57 0.176 0.967<br>Obart09g19950.1 0.619 0.999 0.38 0.13 0.998	0<br>Olong_014116-R1 0.063 0.617 0.554 0.069 0.957	1<br>Opunc09g18280.1 0.619 0.998 0.379 0.118 0.951<br>Opunc09g18290.2 0.001 0.618 0.617 0.001 0.971	1<br>Obrac09g13140.3 0.619 0.985 0.366 0.089 0.806<br>Obrac09g13160.1 0.001 0.618 0.617 0.001 0.953	1<br>Lperr09g15890.1 0.619 0.997 0.378 0.085 0.957<br>Lperr09g15920.1 0.001 0.618 0.617 0.001 0.958	0<br>AT1G18160.1 0.021 0.617 0.596 0.064 0.958
Oglab10g03910.1	1<br>Osjap10g04950.1 0.01 1 0.99 0.01 1<br>Osjap10g21210.1 0.01 1 0.99 0.477 1	1<br>Osind10g03420.1 0.01 1 0.99 0.558 1<br>Osind10g17700.1 0.01 1 0.99 0.477 1	1<br>Orufi10g04520.1 0.01 1 0.99 0.01 1<br>Orufi10g19420.1 0.01 1 0.99 0.477 1	1<br>Oglab10g03910.1 0.01 1 0.99 0.01 1	1<br>Obart10g18220.1 0.01 1 0.99 0.392 0.823	1<br>Olong_009744-R1 0.01 1 0.99 0.651 1<br>Olong_009744-R2 0.01 1 0.99 0.641 1	0	0	0	0
	0<br>Osjap07g13660.9 0.361 1 0.639 0.733 0.947	1<br>Osind02g38620.1 0.361 1 0.639 0.835 0.923<br>Osind06g00010.1 0.01 0.361 0.351 0.609 0.978	1<br>Orufi02g36440.1 0.361 1 0.639 0.835 0.923<br>Orufi08g26670.5 0.01 0.351 0.341 0.583 0.749	1<br>Oglab02g32260.1 0.361 1 0.639 0.838 0.924<br>Oglab04g27900.1 0.01 0.33 0.32 0.14 0.208	1<br>Obart02g34980.1 0.361 1 0.639 0.838 0.924<br>Obart04g29850.1 0.01 0.33 0.32 0.131 0.196	1<br>Olong_008835-R2 0.361 1 0.639 0.809 0.91<br>Olong_022843-R2 0.01 0.33 0.32 0.14 0.208	1<br>Opunc02g32260.1 0.361 1 0.639 0.84 0.925<br>Opunc04g27380.2 0.01 0.33 0.32 0.14 0.208	1<br>Obrac02g25650.1 0.381 1 0.619 0.839 0.923<br>Obrac04g21100.1 0.01 0.33 0.32 0.145 0.217	1<br>Lperr02g28770.1 0.381 1 0.619 0.834 0.92<br>Lperr04g25370.2 0.01 0.33 0.32 0.151 0.225	0
Oglab10g16090.1	0	1<br>Osind10g19230.1 0.001 0.861 0.86 0.002 0.992	1<br>Orufi10g20960.1 0.001 0.861 0.86 0.002 0.992	1<br>Oglab10g16090.1 0.001 1 0.999 0.001 1	1<br>Obart10g19720.1 0.001 0.861 0.86 0.002 0.992	0	1<br>Opunc10g18180.1 0.001 0.99 0.989 0.002 0.998	0	0	0
	1<br>Osjap10g22750.1 0.411 1 0.589 0.061 1<br>Osjap10g22760.1 0.001 0.392 0.391 0.003 0.993	1<br>Osind01g20890.1 0.419 0.977 0.558 0.154 0.802<br>Osind08g03990.1 0.037 0.374 0.337 0.057 0.95	1<br>Orufi01g19490.1 0.419 0.977 0.558 0.171 0.862<br>Orufi08g04140.1 0.037 0.374 0.337 0.057 0.95	1<br>Oglab01g14760.1 0.419 0.984 0.565 0.2 1<br>Oglab08g03250.2 0.037 0.374 0.337 0.052 0.955	1<br>Obart01g17550.1 0.419 0.977 0.558 0.174 0.863<br>Obart08g03750.1 0.037 0.374 0.337 0.057 0.95	1<br>Olong_006312-R1 0.416 1 0.584 0.067 1<br>Olong_006313-R1 0.001 0.392 0.391 0.003 0.993	1<br>Opunc01g18040.1 0.419 0.984 0.565 0.174 1<br>Opunc08g03700.1 0.014 0.374 0.36 0.011 0.951	1<br>Obrac01g13330.1 0.423 0.984 0.561 0.196 1<br>Obrac10g13120.1 0.001 0.392 0.391 0.004 0.993	1<br>Lperr10g15590.1 0.416 0.985 0.569 0.07 0.989<br>Lperr10g15600.1 0.001 0.389 0.388 0.003 0.979	1<br>AT1G32080.1 0.42 0.984 0.564 0.188 1<br>AT2G32260.1 0.035 0.375 0.34 0.078 0.825
Oglab11g02200.1	1<br>Osjap11g02640.1 0.002 1 0.998 0.002 1<br>Osjap12g02610.1 0.002 1 0.998 0.002 1	1<br>Osind11g01990.1 0.002 1 0.998 0.002 1<br>Osind12g02780.1 0.002 1 0.998 0.002 1	1<br>Orufi11g02320.1 0.002 1 0.998 0.003 1<br>Orufi12g02480.1 0.002 1 0.998 0.002 1	1<br>Oglab11g02200.1 0.002 1 0.998 0.002 1<br>Oglab12g02130.1 0.002 1 0.998 0.002 1	1<br>Obart12g02080.1 0.023 1 0.977 0.002 1	0	0	0	0	0
	1<br>Osjap07g04360.1 0.032 0.657 0.625 0.018 0.925<br>Osjap12g12870.1 0.66 0.951 0.291 0.055 0.417	1<br>Osind11g02010.1 0.013 0.657 0.644 0.001 0.994<br>Osind12g10020.1 0.66 0.951 0.291 0.055 0.417	1<br>Orufi07g03650.1 0.032 0.657 0.625 0.019 0.976<br>Orufi12g10750.1 0.658 0.951 0.293 0.045 0.364	1<br>Oglab07g03810.1 0.032 0.657 0.625 0.019 0.987<br>Oglab12g09880.1 0.66 0.951 0.291 0.051 0.385	0<br>Obart11g02350.1 0.021 0.657 0.636 0.004 0.993	1<br>Olong_012584-R1 0.064 0.611 0.547 0.002 0.965<br>Olong_017316-R1 0.658 0.951 0.293 0.045 0.365	1<br>Opunc11g02190.1 0.002 0.657 0.655 0.003 0.989<br>Opunc12g08740.1 0.658 0.951 0.293 0.04 0.324	1<br>Obrac05g10860.1 0.049 0.657 0.608 0.097 0.987<br>Obrac12g07170.1 0.658 0.942 0.284 0.071 0.57	1<br>Lperr12g02190.1 0.002 0.657 0.655 0.003 0.989<br>Lperr12g08370.1 0.657 0.951 0.294 0.047 0.391	0<br>AT5G27320.1 0.013 0.632 0.619 0.003 0.98
Oglab11g05440.1	1<br>Osjap11g06710.1 0.001 0.998 0.997 0.067 0.993<br>Osjap11g06720.1 0.001 0.998 0.997 0.054 0.977	1<br>Osind09g09040.1 0.001 0.998 0.997 0.001 0.995	1<br>Orufi11g05930.1 0.001 0.998 0.997 0.09 0.992<br>Orufi11g05940.1 0.001 1 0.999 0.002 0.995	1<br>Oglab11g05440.1 0.001 1 0.999 0.001 1	1<br>Obart11g05910.1 0.001 0.875 0.874 0.05 1	0	0	0	0	0
	1<br>Osjap01g24690.1 0.525 0.992 0.467 0.014 0.934<br>Osjap11g06740.8 0.024 0.509 0.485 0.013 0.579	1<br>Osind09g09050.1 0.001 0.502 0.501 0.104 0.994<br>Osind11g05650.1 0.521 0.998 0.477 0.002 0.989	1<br>Orufi11g05890.1 0.521 0.998 0.477 0.003 0.984<br>Orufi11g05900.2 0.001 0.509 0.508 0.002 0.991	1<br>Oglab08g10820.1 0.526 0.955 0.429 0.015 0.867<br>Oglab11g05700.1 0.001 0.51 0.509 0.001 0.966	1<br>Obart01g20930.1 0.525 0.992 0.467 0.006 0.498<br>Obart11g05930.1 0.001 0.502 0.501 0.002 0.998	1<br>Olong_005786-R3 0.007 0.511 0.504 0.006 0.948<br>Olong_005787-R1 0.521 0.998 0.477 0.002 0.989	0<br>Opunc11g05960.1 0.443 0.998 0.555 0.002 0.991	1<br>Obrac01g15800.1 0.52 0.992 0.472 0.004 0.915<br>Obrac11g03930.1 0.001 0.504 0.503 0.002 0.941	1<br>Lperr01g17850.1 0.521 0.992 0.471 0.002 0.973<br>Lperr11g05360.1 0.001 0.502 0.501 0.044 0.995	0<br>AT4G38130.1 0.521 0.955 0.434 0.002 0.782

Oglab12g00800.1	1<br>Osjap11g01220.1 0.001 1 0.999 0.001 1<br>Osjap12g01140.1 0.001 1 0.999 0.001 1	1<br>Osind11g00690.1 0.001 0.956 0.955 0.001 0.999<br>Osind12g01290.1 0.001 1 0.999 0.001 1	1<br>Orufi11g01000.1 0.001 1 0.999 0.001 1<br>Orufi12g01130.1 0.001 1 0.999 0.001 1	1<br>Oglab12g00800.1 0.001 1 0.999 0.001 1	1<br>Obart11g01060.1 0.001 1 0.999 0.001 1	0	1<br>Opunc12g00940.1 0.001 1 0.999 0.001 1	0	0	0
	1<br>Osjap03g13100.1 0.49 0.983 0.493 0.097 0.622<br>Osjap07g30390.1 0.009 0.393 0.384 0.013 0.938	1<br>Osind03g12930.1 0.49 0.983 0.493 0.121 0.633<br>Osind07g27070.1 0.006 0.393 0.387 0.006 0.939	1<br>Orufi03g17220.1 0.496 1 0.504 0.027 1<br>Orufi07g27420.1 0.009 0.393 0.384 0.013 0.939	1<br>Oglab03g12120.1 0.49 0.983 0.493 0.121 0.633<br>Oglab07g22980.1 0.006 0.393 0.387 0.007 0.933	1<br>Obart03g12120.1 0.49 0.983 0.493 0.121 0.633<br>Obart12g00790.1 0.001 0.393 0.392 0.002 0.942	1<br>Olong_007715-R1 0.493 1 0.507 0.007 1<br>Olong_008211-R1 0.006 0.393 0.387 0.006 0.939	1<br>Opunc03g11680.1 0.49 1 0.51 0.148 1<br>Opunc11g00920.1 0.001 0.393 0.392 0.142 0.945	1<br>Obrac03g09720.2 0.49 1 0.51 0.142 1<br>Obrac06g16520.1 0.02 0.411 0.391 0.048 0.978	1<br>Lperr03g10720.1 0.49 0.996 0.506 0.148 0.954<br>Lperr11g00920.1 0.001 0.393 0.392 0.002 0.942	1<br>AT3G46740.1 0.493 1 0.507 0.161 1<br>AT4G20110.2 0.001 0.393 0.392 0.002 0.939
Oglab12g03070.1	0	1<br>Osind12g03620.1 0.001 0.999 0.998 0.029 0.961	1<br>Orufi12g03350.1 0.012 0.999 0.987 0.044 0.966<br>Orufi12g03350.2 0.012 0.999 0.987 0.043 0.966	1<br>Oglab12g03070.1 0.001 1 0.999 0.001 1	1<br>Obart12g02950.1 0.001 1 0.999 0.001 1	1<br>Olong_000549-R1 0.012 0.999 0.987 0.011 0.967	0	0	0	0
	1<br>Osjap10g20010.1 0.483 0.99 0.507 0.022 0.946<br>Osjap12g02500.1 0.002 0.437 0.435 0.001 0.388	1<br>Osind01g01580.1 0.477 0.984 0.507 0.171 0.96<br>Osind12g02670.1 0.002 0.437 0.435 0.001 0.396	1<br>Orufi04g25720.1 0.483 0.867 0.384 0.002 0.207<br>Orufi11g02210.1 0.016 0.437 0.421 0.015 0.492	1<br>Oglab10g13340.3 0.546 0.99 0.444 0.213 0.951<br>Oglab12g02020.1 0.002 0.437 0.435 0.001 0.388	0<br>Obart11g02240.1 0.198 0.437 0.239 0.014 0.272<br>Obart11g21270.1 0.479 0.984 0.505 0.138 0.958	0<br>Olong_010277-R4 0.473 0.867 0.394 0.001 0.93<br>Olong_027241-R1 0.047 0.287 0.24 0.003 0.866	1<br>Opunc11g17890.2 0.476 0.984 0.508 0.189 0.952<br>Opunc12g02040.1 0.012 0.437 0.425 0.009 0.402	1<br>Obrac11g01490.1 0.002 0.437 0.435 0.029 0.403<br>Obrac11g12600.2 0.534 0.984 0.45 0.046 0.748	0<br>Lperr11g01950.1 0.012 0.235 0.223 0.015 0.297<br>Lperr11g17840.1 0.486 0.984 0.498 0.046 0.945	1<br>AT1G65810.1 0.483 0.867 0.384 0.021 0.596<br>AT2G26780.1 0.017 0.433 0.416 0.011 0.388
Oglab12g09470.1	1<br>Osjap12g12320.1 0.001 1 0.999 0.001 1	1<br>Osind12g09530.1 0.001 1 0.999 0.001 1	1<br>Orufi12g10460.1 0.001 1 0.999 0.001 1	1<br>Oglab12g09470.1 0.001 1 0.999 0.001 1	1<br>Obart12g09410.1 0.001 1 0.999 0.001 1	0	0	0	0	0
	1<br>Osjap01g44780.2 0.44 0.998 0.558 0.095 0.994<br>Osjap03g07600.1 0.001 0.295 0.294 0.004 0.981	1<br>Osind01g46040.1 0.44 0.998 0.558 0.151 0.995<br>Osind03g07670.1 0.001 0.295 0.294 0.004 0.981	1<br>Orufi01g42650.3 0.44 0.998 0.558 0.195 0.995<br>Orufi03g07180.1 0.001 0.295 0.294 0.004 0.981	1<br>Oglab01g34040.1 0.489 0.998 0.509 0.203 0.995<br>Oglab03g07280.1 0.001 0.295 0.294 0.004 0.981	1<br>Obart01g39260.3 0.456 0.998 0.542 0.041 0.994<br>Obart03g07210.1 0.001 0.295 0.294 0.004 0.981	1<br>Olong_021912-R1 0.457 1 0.543 0.099 1<br>Olong_021913-R1 0.001 0.296 0.295 0.004 0.988	1<br>Opunc12g08570.1 0.326 1 0.674 0.052 1<br>Opunc12g08580.1 0.001 0.296 0.295 0.004 0.988	1<br>Obrac03g05880.1 0.001 0.296 0.295 0.004 0.988<br>Obrac12g06500.1 0.461 1 0.539 0.103 1	1<br>Lperr01g33810.2 0.456 0.998 0.542 0.002 0.994<br>Lperr12g08300.1 0.001 0.296 0.295 0.004 0.985	0<br>AT4G34670.1 0.001 0.29 0.289 0.004 0.966<br>AT5G67540.2 0.489 0.993 0.504 0.053 1

((((_d1,punctata),brachy),leersia),tair);

chimeric gene id	INGROUP						MIDGROUP	OUTGROUP		
	jap	indica	rufi	glab	barthii	longi	punctata	brachy	leersia	tair
Oglab01g06470.1	1<br>Osjap01g08660.1 0.001 1 0.999 0.001 1	1<br>Osind01g08490.1 0.001 1 0.999 0.001 1	1<br>Orufi01g08070.1 0.001 1 0.999 0.001 1	1<br>Oglab01g06470.1 0.001 1 0.999 0.001 1	1<br>Obart01g07430.1 0.02 1 0.98 0.013 1	1<br>Olong_011567-R1 0.056 1 0.944 0.001 1	0	0	0	0
	1<br>Osjap01g29230.2 0.1 0.581 0.481 0.25 0.983<br>Osjap05g00560.2 0.647 0.997 0.35 0.146 1	1<br>Osind01g30690.1 0.1 0.581 0.481 0.267 0.982<br>Osind05g00540.1 0.647 0.997 0.35 0.126 1	1<br>Orufi01g27950.1 0.1 0.581 0.481 0.25 0.983<br>Orufi01g48560.2 0.647 0.98 0.333 0.085 0.984	1<br>Oglab01g21070.2 0.1 0.581 0.481 0.249 0.983<br>Oglab05g00530.1 0.647 0.997 0.35 0.146 1	1<br>Obart01g25090.1 0.1 0.581 0.481 0.219 0.982<br>Obart05g00540.1 0.647 0.997 0.35 0.146 1	0<br>Olong_003039-R1 0.647 0.997 0.35 0.128 1<br>Olong_003692-R2 0.139 0.581 0.442 0.244 0.979	1<br>Opunc01g07170.1 0.001 0.582 0.581 0.001 0.984<br>Opunc05g00510.2 0.647 0.997 0.35 0.15 1	1<br>Obrac01g05760.3 0.583 1 0.417 0.082 1<br>Obrac01g05770.3 0.001 0.582 0.581 0.001 0.985	1<br>Lperr01g07030.1 0.583 1 0.417 0.018 1<br>Lperr01g07040.2 0.001 0.582 0.581 0.054 0.989	1<br>AT1G54350.1 0.585 0.993 0.408 0.084 0.975<br>AT5G53430.1 0.071 0.581 0.51 0.164 0.984
Oglab02g22130.2	1<br>Osjap02g26750.1 0.001 0.969 0.968 0.001 0.994<br>Osjap02g26750.2 0.001 0.969 0.968 0.001 0.994<br>Osjap02g26750.4 0.001 0.969 0.968 0.001 0.799	1<br>Osind02g26640.2 0.001 1 0.999 0.001 1	1<br>Orufi02g24900.1 0.011 0.974 0.963 0.06 0.859<br>Orufi02g24900.3 0.001 0.974 0.973 0.001 0.74<br>Orufi02g24900.4 0.001 0.974 0.973 0.001 0.737	1<br>Oglab02g22130.2 0.001 1 0.999 0.001 1	1<br>Obart02g23460.2 0.001 1 0.999 0.001 1	0	1<br>Opunc02g21410.1 0.011 0.999 0.988 0.116 0.996<br>Opunc02g21410.2 0.011 0.999 0.988 0.114 0.996<br>Opunc02g21410.3 0.011 0.999 0.988 0.115 0.996<br>Opunc02g21410.4 0.011 0.999 0.988 0.114 0.996	0	1<br>Lperr02g18970.1 0.011 0.997 0.986 0.032 0.996	0
	1<br>Osjap02g26590.1 0.475 0.999 0.524 0.033 0.999<br>Osjap02g26750.5 0.011 0.459 0.448 0.238 0.973	1<br>Osind02g26450.1 0.475 0.999 0.524 0.025 0.997<br>Osind02g26620.1 0.011 0.459 0.448 0.495 0.981	1<br>Orufi02g24730.1 0.475 0.994 0.519 0.031 0.996<br>Orufi02g24880.2 0.011 0.459 0.448 0.18 0.97	1<br>Oglab02g21950.1 0.475 0.999 0.524 0.073 0.995<br>Oglab02g22110.2 0.011 0.459 0.448 0.532 0.98	1<br>Obart02g23290.1 0.475 0.999 0.524 0.029 0.999<br>Obart02g23440.1 0.011 0.459 0.448 0.492 0.981	0<br>Olong_024345-R1 0.011 0.474 0.463 0.442 0.991	1<br>Opunc02g21230.1 0.479 0.976 0.497 0.003 0.934<br>Opunc02g21240.1 0.011 0.461 0.45 0.121 0.972	1<br>Obrac02g16670.1 0.549 0.973 0.424 0.076 0.995<br>Obrac02g16700.1 0.011 0.461 0.45 0.114 0.947	1<br>Lperr02g18740.1 0.485 0.997 0.512 0.056 0.991<br>Lperr02g19120.1 0.011 0.484 0.473 0.183 0.978	1<br>AT4G31860.1 0.014 0.45 0.436 0.123 0.989<br>AT5G43080.1 0.626 1 0.374 0.194 0.932

Oglab02g33340.1	1<br>Osjap02g40310.1 0.042 0.973 0.931 0.432 0.926<br>Osjap02g40340.1 0.042 1 0.958 0.361 1<br>Osjap02g40370.1 0.042 0.973 0.931 0.267 0.726	1<br>Osind02g39860.1 0.042 0.977 0.935 0.257 0.659<br>Osind02g39860.2 0.069 0.977 0.908 0.058 0.571<br>Osind02g39860.3 0.069 0.973 0.904 0.061 0.631<br>Osind02g39860.4 0.069 0.973 0.904 0.066 0.679	1<br>Orufi02g37680.1 0.069 0.989 0.92 0.067 0.86<br>Orufi02g37680.10 0.069 0.989 0.92 0.091 0.811<br>Orufi02g37680.2 0.069 0.989 0.92 0.07 0.854<br>Orufi02g37680.3 0.069 0.989 0.92 0.076 0.895<br>Orufi02g37680.4 0.069 0.989 0.92 0.073 0.849<br>Orufi02g37680.5 0.069 0.989 0.92 0.076 0.841<br>Orufi02g37680.6 0.069 0.989 0.92 0.088 0.879<br>Orufi02g37680.7 0.069 0.973 0.904 0.079 0.855<br>Orufi02g37680.9 0.069 0.973 0.904 0.086 0.843	1<br>Oglab02g33340.1 0.004 1 0.996 0.004 1	1<br>Obart02g36180.1 0.069 0.989 0.92 0.061 0.778<br>Obart02g36180.2 0.069 0.989 0.92 0.073 0.734<br>Obart02g36180.3 0.069 0.989 0.92 0.076 0.724	0	0	0	0	0
	1<br>Osjap02g41540.1 0.483 0.992 0.509 0.434 0.804<br>Osjap05g23970.1 0.061 0.387 0.326 0.669 0.821	1<br>Osind02g41040.1 0.483 0.992 0.509 0.459 0.813<br>Osind05g24480.1 0.061 0.387 0.326 0.588 0.777	0<br>Orufi02g37680.8 0.372 0.989 0.617 0.159 0.801	1<br>Oglab02g34400.1 0.483 0.992 0.509 0.434 0.804<br>Oglab03g25400.1 0.073 0.41 0.337 0.475 0.707	1<br>Obart02g37290.1 0.483 0.992 0.509 0.434 0.804<br>Obart05g21320.1 0.061 0.387 0.326 0.588 0.777	1<br>Olong_011081-R1 0.479 1 0.521 0.05 1<br>Olong_025373-R1 0.073 0.41 0.337 0.236 0.574	1<br>Opunc02g34450.1 0.483 0.992 0.509 0.432 0.803<br>Opunc05g18980.1 0.061 0.383 0.322 0.588 0.771	1<br>Obrac02g27500.6 0.483 0.992 0.509 0.432 0.803<br>Obrac05g14470.1 0.061 0.383 0.322 0.588 0.771	1<br>Lperr02g30750.1 0.483 0.992 0.509 0.453 0.811<br>Lperr04g17770.1 0.038 0.383 0.345 0.48 0.678	1<br>AT1G16330.1 0.038 0.414 0.376 0.701 0.873<br>AT2G20360.1 0.483 0.992 0.509 0.423 0.799
Oglab03g00390.1	1<br>Osjap03g00680.1 0.001 1 0.999 0.001 1<br>Osjap03g00680.2 0.001 1 0.999 0.001 1<br>Osjap03g00680.3 0.001 1 0.999 0.001 1	1<br>Osind03g00740.1 0.001 0.994 0.993 0.001 0.996	1<br>Orufi03g12660.1 0.001 1 0.999 0.001 1<br>Orufi03g12660.2 0.001 1 0.999 0.001 1	1<br>Oglab03g00390.1 0.001 1 0.999 0.001 1	1<br>Obart03g00550.1 0.001 0.994 0.993 0.001 0.996	0	0	0	0	0
	1<br>Osjap02g42090.1 0.049 0.634 0.585 0.065 1<br>Osjap04g17560.1 0.696 0.998 0.302 0.466 0.996	1<br>Osind02g41670.1 0.058 0.634 0.576 0.131 1<br>Osind03g02220.1 0.652 1 0.348 0.015 1	1<br>Orufi02g39350.1 0.058 0.634 0.576 0.131 1<br>Orufi09g02220.1 0.696 1 0.304 0.072 1	1<br>Oglab02g34920.1 0.058 0.634 0.576 0.131 1<br>Oglab09g02040.2 0.696 1 0.304 0.07 1	1<br>Obart03g02140.1 0.652 1 0.348 0.013 1<br>Obart09g20260.1 0.152 0.634 0.482 0.12 1	0<br>Olong_013175-R1 0.253 0.906 0.653 0.002 0.961	1<br>Opunc03g00470.1 0.001 0.73 0.729 0.002 0.996<br>Opunc11g04590.1 0.783 0.998 0.215 0.023 1	0<br>Obrac02g10260.1 0.736 0.999 0.263 0.286 0.995<br>Obrac02g27970.1 0.128 0.634 0.506 0.105 1	1<br>Lperr03g00570.1 0.001 0.634 0.633 0.002 1<br>Lperr12g09110.1 0.699 0.998 0.299 0.295 0.967	0<br>AT5G04460.1 0.017 0.634 0.617 0.081 1
Oglab03g30830.1	1<br>Osjap03g34330.1 0.001 1 0.999 0.001 1	1<br>Osind03g35550.1 0.001 1 0.999 0.039 1	1<br>Orufi03g33060.1 0.001 1 0.999 0.001 1	1<br>Oglab03g30830.1 0.001 1 0.999 0.001 1	1<br>Obart03g31750.1 0.001 1 0.999 0.046 1	0	0	0	0	0
	1<br>Osjap03g39110.1 0.652 1 0.348 0.036 1<br>Osjap10g05370.1 0.012 0.583 0.571 0.1 0.963	1<br>Osind01g46540.1 0.652 0.999 0.347 0.065 0.994<br>Osind10g03770.1 0.012 0.583 0.571 0.11 0.96	1<br>Orufi10g04850.1 0.029 0.583 0.554 0.093 0.963<br>Orufi11g12520.1 0.653 1 0.347 0.038 0.991	1<br>Oglab03g34460.1 0.629 0.997 0.368 0.016 0.993<br>Oglab10g04340.1 0.012 0.583 0.571 0.1 0.963	1<br>Obart03g36200.1 0.652 1 0.348 0.016 0.997<br>Obart10g04600.1 0.013 0.583 0.57 0.106 0.962	1<br>Olong_014796-R1 0.001 0.495 0.494 0.002 0.95<br>Olong_024872-R1 0.648 1 0.352 0.01 1	1<br>Opunc01g42030.1 0.652 1 0.348 0.28 0.872<br>Opunc03g28940.2 0.001 0.6 0.599 0.002 0.995	1<br>Obrac02g16280.1 0.646 0.982 0.336 0.063 0.949<br>Obrac03g23440.1 0.001 0.6 0.599 0.002 0.995	1<br>Lperr03g26400.1 0.001 0.6 0.599 0.002 0.995<br>Lperr03g30520.1 0.644 1 0.356 0.003 0.997	0<br>AT1G70730.3 0.001 0.6 0.599 0.118 0.995
Oglab05g03490.1	1<br>Osjap05g04120.1 0.001 1 0.999 0.001 1<br>Osjap05g04120.2 0.001 1 0.999 0.001 1	1<br>Osind05g04070.1 0.008 1 0.992 0.001 1<br>Osind05g04070.2 0.008 1 0.992 0.001 1	1<br>Orufi05g03950.1 0.001 1 0.999 0.001 1<br>Orufi05g03950.2 0.001 1 0.999 0.001 1	1<br>Oglab05g03490.1 0.001 1 0.999 0.001 1<br>Oglab05g03490.2 0.001 1 0.999 0.001 1	1<br>Obart05g03830.1 0.001 1 0.999 0.001 1<br>Obart05g03830.2 0.001 1 0.999 0.001 1	1<br>Olong_016739-R1 0.074 1 0.926 0.001 1	1<br>Opunc05g03540.1 0.001 1 0.999 0.001 1<br>Opunc05g03540.2 0.001 1 0.999 0.001 1	1<br>Obrac05g02930.1 0.074 1 0.926 0.001 1	0	0
	1<br>Osjap01g27410.1 0.09 0.723 0.633 0.066 0.996<br>Osjap03g15140.5 0.743 1 0.257 0.061 1	1<br>Osind01g28630.1 0.09 0.723 0.633 0.066 0.996<br>Osind03g15230.1 0.79 1 0.21 0.03 1	1<br>Orufi01g26130.1 0.09 0.723 0.633 0.066 0.996<br>Orufi03g14720.1 0.79 1 0.21 0.03 1	1<br>Oglab01g19490.1 0.09 0.723 0.633 0.066 0.996<br>Oglab03g14060.1 0.79 1 0.21 0.03 1	1<br>Obart01g23310.1 0.09 0.723 0.633 0.066 0.996<br>Obart03g14050.1 0.79 1 0.21 0.03 1	1<br>Olong_003347-R2 0.09 0.723 0.633 0.047 0.996<br>Olong_009943-R1 0.79 1 0.21 0.03 1	1<br>Opunc01g23460.1 0.09 0.723 0.633 0.064 0.996<br>Opunc03g13590.1 0.79 1 0.21 0.002 0.545	1<br>Obrac01g17590.1 0.09 0.723 0.633 0.067 0.996<br>Obrac03g11330.1 0.79 1 0.21 0.03 1	0<br>Lperr05g03460.1 0.001 0.721 0.72 0.001 0.993	1<br>AT2G47580.1 0.775 1 0.225 0.008 1<br>AT3G16630.1 0.003 0.721 0.718 0.031 0.992
Oglab05g03490.2	1<br>Osjap05g04120.1 0.001 1 0.999 0.001 1<br>Osjap05g04120.2 0.001 1 0.999 0.001 1	1<br>Osind05g04070.1 0.008 1 0.992 0.001 1<br>Osind05g04070.2 0.008 1 0.992 0.001 1	1<br>Orufi05g03950.1 0.001 1 0.999 0.001 1<br>Orufi05g03950.2 0.001 1 0.999 0.001 1	1<br>Oglab05g03490.1 0.001 1 0.999 0.001 1<br>Oglab05g03490.2 0.001 1 0.999 0.001 1	1<br>Obart05g03830.1 0.001 1 0.999 0.001 1<br>Obart05g03830.2 0.001 1 0.999 0.001 1	1<br>Olong_016739-R1 0.073 1 0.927 0.001 1	1<br>Opunc05g03540.1 0.001 1 0.999 0.001 1<br>Opunc05g03540.2 0.001 1 0.999 0.001 1	1<br>Obrac05g02930.1 0.073 1 0.927 0.001 1	0	0
	1<br>Osjap01g27410.1 0.089 0.726 0.637 0.066 0.996<br>Osjap03g15140.5 0.747 1 0.253 0.061 1	1<br>Osind01g28630.1 0.089 0.726 0.637 0.066 0.996<br>Osind03g15230.1 0.792 1 0.208 0.03 1	1<br>Orufi01g26130.1 0.089 0.726 0.637 0.066 0.996<br>Orufi03g14720.1 0.792 1 0.208 0.03 1	1<br>Oglab01g19490.1 0.089 0.726 0.637 0.066 0.996<br>Oglab03g14060.1 0.792 1 0.208 0.03 1	1<br>Obart01g23310.1 0.089 0.726 0.637 0.066 0.996<br>Obart03g14050.1 0.792 1 0.208 0.03 1	1<br>Olong_003347-R2 0.089 0.726 0.637 0.047 0.996<br>Olong_009943-R1 0.792 1 0.208 0.03 1	1<br>Opunc01g23460.1 0.089 0.726 0.637 0.064 0.996<br>Opunc03g13590.1 0.792 1 0.208 0.002 0.545	1<br>Obrac01g17590.1 0.089 0.726 0.637 0.067 0.996<br>Obrac03g11330.1 0.792 1 0.208 0.03 1	0<br>Lperr05g03460.1 0.001 0.725 0.724 0.001 0.993	1<br>AT2G47580.1 0.778 1 0.222 0.008 1<br>AT3G16630.1 0.003 0.725 0.722 0.031 0.992

Oglab05g23160.1	1<br>Osjap05g28230.1 0.001 1 0.999 0.001 1	1<br>Osind05g28520.1 0.001 1 0.999 0.001 1	1<br>Orufi05g26870.1 0.001 1 0.999 0.001 1	1<br>Oglab05g23160.1 0.001 1 0.999 0.001 1	1<br>Obart05g25070.1 0.001 1 0.999 0.001 1	0	1<br>Opunc05g22640.1 0.001 1 0.999 0.001 1	0	1<br>Lperr01g22990.1 0.001 1 0.999 0.027 1	0
	1<br>Osjap01g31140.1 0.007 0.451 0.444 0.093 0.995<br>Osjap01g31150.1 0.462 0.999 0.537 0.009 0.999	1<br>Osind01g32600.1 0.001 0.451 0.45 0.002 0.995<br>Osind01g32610.1 0.462 0.999 0.537 0.009 0.999	1<br>Orufi01g29880.1 0.001 0.451 0.45 0.075 0.995<br>Orufi01g29890.1 0.462 0.999 0.537 0.009 0.999	1<br>Oglab01g22740.1 0.028 0.451 0.423 0.061 0.995<br>Oglab01g22750.1 0.462 0.999 0.537 0.009 0.999	1<br>Obart01g26870.1 0.006 0.451 0.445 0.117 0.995<br>Obart01g26880.1 0.462 0.999 0.537 0.009 0.999	1<br>Olong_000367-R1 0.105 0.451 0.346 0.002 0.993<br>Olong_000368-R1 0.456 0.999 0.543 0.108 0.999	1<br>Opunc01g26820.1 0.049 0.451 0.402 0.002 0.994<br>Opunc01g26830.1 0.462 1 0.538 0.009 1	1<br>Obrac01g20400.1 0.048 0.451 0.403 0.006 0.994<br>Obrac05g17560.2 0.451 1 0.549 0.164 1	1<br>Lperr05g20850.2 0.467 1 0.533 0.002 1<br>Lperr05g20860.1 0.001 0.451 0.45 0.002 0.995	1<br>AT5G08690.1 0.012 0.451 0.439 0.034 0.995<br>AT5G51600.1 0.462 0.999 0.537 0.008 0.996
Oglab06g02870.1	1<br>Osjap06g03080.1 0.001 1 0.999 0.001 1	0	1<br>Orufi06g02670.1 0.045 0.964 0.919 0.032 0.91	1<br>Oglab06g02870.1 0.001 1 0.999 0.001 1	1<br>Obart06g02710.1 0.001 1 0.999 0.03 1	1<br>Olong_013693-R1 0.045 1 0.955 0.069 1	0	0	0	0
	1<br>Osjap01g37620.1 0.001 0.756 0.755 0.033 0.998<br>Osjap04g34350.1 0.767 0.968 0.201 0.466 0.888	1<br>Osind03g45110.1 0.04 0.75 0.71 0.141 0.984<br>Osind04g33620.1 0.767 0.968 0.201 0.438 0.875	0<br>Orufi01g36040.3 0.037 0.756 0.719 0.03 0.998	1<br>Oglab01g27580.1 0.037 0.715 0.678 0.03 0.949<br>Oglab04g28300.1 0.767 0.968 0.201 0.466 0.888	1<br>Obart01g06310.1 0.037 0.756 0.719 0.066 0.997<br>Obart04g30240.1 0.767 0.968 0.201 0.466 0.888	1<br>Olong_001861-R1 0.767 0.968 0.201 0.381 0.87<br>Olong_005208-R1 0.014 0.715 0.701 0.141 0.969	1<br>Opunc03g00730.1 0.001 0.756 0.755 0.004 1<br>Opunc04g27750.1 0.767 0.968 0.201 0.426 0.879	1<br>Obrac04g05640.6 0.014 0.705 0.691 0.262 0.995<br>Obrac04g21390.1 0.767 0.968 0.201 0.381 0.87	1<br>Lperr10g07060.1 0.04 0.722 0.682 0.115 0.964<br>Lperr12g00960.1 0.767 0.968 0.201 0.389 0.871	0<br>AT2G37990.1 0.767 0.968 0.201 0.425 0.893
Oglab06g24190.1	1<br>Osjap06g31210.1 0.001 0.995 0.994 0.008 1	1<br>Osind06g30430.1 0.001 0.995 0.994 0.008 1	1<br>Orufi06g28720.1 0.001 0.995 0.994 0.008 1	1<br>Oglab06g24190.1 0.001 1 0.999 0.001 1<br>Oglab06g24190.2 0.001 0.995 0.994 0.001 0.991	1<br>Obart06g26750.1 0.001 0.995 0.994 0.008 1	1<br>Olong_013658-R1 0.001 0.995 0.994 0.001 1	1<br>Opunc06g24460.1 0.001 0.995 0.994 0.008 1	0	1<br>Lperr06g22350.1 0.001 0.995 0.994 0.033 1	0
	1<br>Osjap02g03010.1 0.694 0.993 0.299 0.006 0.928<br>Osjap12g19610.1 0.001 0.62 0.619 0.001 0.675	1<br>Osind02g03310.1 0.694 0.993 0.299 0.006 0.928<br>Osind07g26390.1 0.001 0.59 0.589 0.074 0.935	1<br>Orufi02g02960.1 0.694 0.993 0.299 0.006 0.928<br>Orufi12g16970.1 0.001 0.62 0.619 0.001 0.674	1<br>Oglab02g02970.1 0.694 0.993 0.299 0.006 0.93<br>Oglab12g14250.1 0.001 0.62 0.619 0.001 0.743	1<br>Obart02g03050.1 0.694 0.993 0.299 0.006 0.93<br>Obart12g15260.1 0.001 0.602 0.601 0.001 0.686	1<br>Olong_017635-R1 0.694 0.993 0.299 0.006 0.93<br>Olong_024721-R1 0.01 0.608 0.598 0.05 0.932	1<br>Opunc02g02350.2 0.715 0.993 0.278 0.248 0.944<br>Opunc03g37840.1 0.001 0.622 0.621 0.047 0.706	1<br>Obrac02g02360.1 0.694 0.994 0.3 0.006 0.933<br>Obrac09g01040.1 0.001 0.591 0.59 0.026 0.872	1<br>Lperr02g02740.1 0.699 0.995 0.296 0.006 0.96<br>Lperr12g12430.1 0.001 0.602 0.601 0.001 0.854	1<br>AT3G10250.1 0.701 0.998 0.297 0.006 0.948<br>AT4G32430.1 0.005 0.61 0.605 0.051 0.976
Oglab06g24190.2	1<br>Osjap06g31210.1 0.001 0.991 0.99 0.008 1	1<br>Osind06g30430.1 0.001 0.991 0.99 0.008 1	1<br>Orufi06g28720.1 0.001 0.991 0.99 0.008 1	1<br>Oglab06g24190.1 0.001 0.991 0.99 0.001 0.995<br>Oglab06g24190.2 0.001 1 0.999 0.001 1	1<br>Obart06g26750.1 0.001 0.991 0.99 0.008 1	1<br>Olong_013658-R1 0.001 0.991 0.99 0.001 1	1<br>Opunc06g24460.1 0.001 0.991 0.99 0.008 1	0	1<br>Lperr06g22350.1 0.001 0.991 0.99 0.033 1	0
	1<br>Osjap02g03010.1 0.691 0.997 0.306 0.006 0.959<br>Osjap12g19610.1 0.001 0.617 0.616 0.001 0.675	1<br>Osind02g03310.1 0.691 0.997 0.306 0.006 0.959<br>Osind07g26390.1 0.001 0.587 0.586 0.074 0.935	1<br>Orufi02g02960.1 0.691 0.997 0.306 0.006 0.959<br>Orufi09g02510.1 0.001 0.589 0.588 0.014 0.762	1<br>Oglab02g02970.1 0.691 0.997 0.306 0.006 0.96<br>Oglab12g14250.1 0.001 0.617 0.616 0.001 0.743	1<br>Obart02g03050.1 0.691 0.997 0.306 0.006 0.96<br>Obart12g15260.1 0.001 0.617 0.616 0.001 0.686	1<br>Olong_017635-R1 0.691 0.997 0.306 0.006 0.96<br>Olong_024721-R1 0.01 0.605 0.595 0.05 0.932	1<br>Opunc02g02350.2 0.712 0.997 0.285 0.248 0.968<br>Opunc03g37840.1 0.001 0.619 0.618 0.047 0.706	1<br>Obrac02g02360.1 0.691 0.997 0.306 0.006 0.961<br>Obrac09g01040.1 0.001 0.588 0.587 0.026 0.872	1<br>Lperr02g02740.1 0.696 0.991 0.295 0.006 0.96<br>Lperr03g02630.1 0.001 0.584 0.583 0.105 0.84	1<br>AT3G10250.1 0.698 0.992 0.294 0.006 0.941<br>AT4G32430.1 0.005 0.607 0.602 0.051 0.976
Oglab06g25490.1	1<br>Osjap09g17800.1 0.024 1 0.976 0.113 0.974<br>Osjap09g17800.2 0.024 1 0.976 0.114 0.974<br>Osjap09g17800.3 0.024 0.997 0.973 0.187 0.947	1<br>Osind06g31620.1 0.001 1 0.999 0.001 1	1<br>Orufi06g29990.1 0.001 1 0.999 0.001 1<br>Orufi09g18790.1 0.024 1 0.976 0.107 0.97<br>Orufi09g18790.2 0.024 1 0.976 0.107 0.97<br>Orufi09g18790.3 0.024 1 0.976 0.108 0.969<br>Orufi09g18790.4 0.024 1 0.976 0.108 0.969<br>Orufi09g18790.5 0.024 0.976 0.952 0.17 0.937<br>Orufi09g18790.6 0.024 0.996 0.972 0.172 0.937	1<br>Oglab06g25490.1 0.001 1 0.999 0.001 1<br>Oglab09g15660.1 0.024 0.999 0.975 0.154 0.95	1<br>Obart06g28060.1 0.001 1 0.999 0.001 1<br>Obart09g17460.1 0.024 1 0.976 0.076 0.969<br>Obart09g17460.2 0.024 1 0.976 0.076 0.969	1<br>Olong_016700-R1 0.001 1 0.999 0.001 1	0	0	1<br>Lperr09g13380.1 0.084 0.982 0.898 0.166 0.951	0
	1<br>Osjap06g32830.1 0.427 1 0.573 0.085 1<br>Osjap06g32840.1 0.001 0.407 0.406 0.002 0.952	1<br>Osind04g24640.1 0.07 0.417 0.347 0.233 0.933<br>Osind06g30840.1 0.485 0.999 0.514 0.109 1	0<br>Orufi06g29150.4 0.485 0.999 0.514 0.122 1	1<br>Oglab02g01250.1 0.066 0.406 0.34 0.221 0.93<br>Oglab10g06880.1 0.427 1 0.573 0.099 1	1<br>Obart04g21770.1 0.07 0.417 0.347 0.232 0.933<br>Obart10g07310.1 0.427 1 0.573 0.099 1	1<br>Olong_000286-R1 0.066 0.406 0.34 0.258 0.928<br>Olong_026973-R2 0.427 1 0.573 0.099 1	1<br>Opunc06g25610.1 0.001 0.407 0.406 0.002 0.951<br>Opunc10g06480.1 0.427 0.998 0.571 0.098 0.984	1<br>Obrac06g20010.2 0.432 1 0.568 0.119 1<br>Obrac06g20020.1 0.001 0.407 0.406 0.002 0.952	1<br>Lperr02g22230.1 0.043 0.392 0.349 0.113 0.88<br>Lperr06g23510.1 0.402 1 0.598 0.074 1	1<br>AT1G76360.1 0.036 0.406 0.37 0.219 0.952<br>AT3G61130.1 0.465 0.999 0.534 0.207 1

Oglab08g05640.1	1<br>Osjap08g06650.1 0.002 1 0.998 0.002 1	1<br>Osind08g06790.1 0.002 1 0.998 0.002 1	1<br>Orufi08g06840.1 0.002 1 0.998 0.002 1	1<br>Oglab08g05640.1 0.002 1 0.998 0.002 1	1<br>Obart08g06070.1 0.002 1 0.998 0.002 1	0	1<br>Opunc08g05850.1 0.047 1 0.953 0.068 1	0	0	0
	1<br>Osjap04g21220.1 0.047 0.275 0.228 0.21 0.936<br>Osjap09g20870.1 0.359 0.984 0.625 0.208 0.966	1<br>Osind04g20250.1 0.047 0.275 0.228 0.198 0.94<br>Osind09g20960.1 0.359 0.984 0.625 0.208 0.966	1<br>Orufi04g19250.2 0.047 0.275 0.228 0.21 0.936<br>Orufi09g21960.1 0.359 0.984 0.625 0.2 0.965	1<br>Oglab02g26360.1 0.348 0.996 0.648 0.01 0.994<br>Oglab04g16180.1 0.047 0.275 0.228 0.198 0.94	1<br>Obart02g28440.1 0.348 0.996 0.648 0.093 0.994<br>Obart04g17770.1 0.047 0.275 0.228 0.198 0.94	1<br>Olong_008030-R1 0.047 0.275 0.228 0.232 0.937<br>Olong_008031-R2 0.277 1 0.723 0.106 1	1<br>Opunc04g15550.1 0.047 0.275 0.228 0.206 0.938<br>Opunc04g18740.1 0.295 0.984 0.689 0.088 0.994	1<br>Obrac08g04780.1 0.277 1 0.723 0.098 1<br>Obrac08g04800.1 0.002 0.275 0.273 0.006 0.943	1<br>Lperr08g05690.1 0.277 1 0.723 0.1 1<br>Lperr08g05700.1 0.002 0.275 0.273 0.006 0.943	1<br>AT1G05310.1 0.33 0.982 0.652 0.183 0.99<br>AT4G30800.1 0.047 0.275 0.228 0.208 0.943
Oglab08g15520.1	1<br>Osjap01g09050.1 0.007 0.844 0.837 0.061 1	1<br>Osind01g08880.1 0.102 0.986 0.884 0.004 0.567	1<br>Orufi01g08450.1 0.007 0.973 0.966 0.026 0.487	1<br>Oglab01g06790.1 0.007 0.844 0.837 0.061 1<br>Oglab08g15520.1 0.007 1 0.993 0.007 1	1<br>Obart01g07700.1 0.007 0.844 0.837 0.061 1<br>Obart08g16670.1 0.007 0.993 0.986 0.007 0.993	1<br>Olong_003647-R1 0.007 0.83 0.823 0.061 0.985	0	0	0	0
	1<br>Osjap01g24690.1 0.007 0.694 0.687 0.104 0.321<br>Osjap07g09900.1 0.694 1 0.306 0.005 0.126	1<br>Osind07g08950.1 0.694 1 0.306 0.004 0.113<br>Osind11g05650.1 0.007 0.694 0.687 0.103 0.319	1<br>Orufi01g23650.1 0.007 0.694 0.687 0.106 0.307<br>Orufi07g08630.1 0.694 1 0.306 0.005 0.117	1<br>Oglab03g37410.1 0.714 1 0.286 0.049 0.141<br>Oglab11g05440.1 0.007 0.694 0.687 0.573 0.683	1<br>Obart07g08560.1 0.694 0.952 0.258 0.009 0.192<br>Obart11g05910.1 0.007 0.694 0.687 0.752 0.834	1<br>Olong_005787-R1 0.007 0.694 0.687 0.103 0.319<br>Olong_016241-R1 0.728 1 0.272 0.735 0.775	1<br>Opunc01g21000.1 0.007 0.694 0.687 0.114 0.334<br>Opunc07g08090.1 0.694 1 0.306 0.004 0.092	1<br>Obrac07g05110.1 0.728 0.993 0.265 0.077 0.151<br>Obrac11g03720.1 0.007 0.694 0.687 0.112 0.34	1<br>Lperr01g17850.1 0.007 0.694 0.687 0.105 0.313<br>Lperr07g08590.1 0.694 0.993 0.299 0.022 0.124	1<br>AT3G55700.1 0.728 0.973 0.245 0.015 0.093<br>AT4G38130.1 0.007 0.68 0.673 0.09 0.269
Oglab08g20310.1	1<br>Osjap08g25130.1 0.007 1 0.993 0.007 1	1<br>Osind08g27620.1 0.007 1 0.993 0.009 1	1<br>Orufi08g26140.1 0.007 1 0.993 0.007 1	1<br>Oglab08g20310.1 0.007 1 0.993 0.007 1	1<br>Obart08g23660.1 0.007 1 0.993 0.007 1	1<br>Olong_025249-R1 0.051 0.964 0.913 0.654 0.984	0	0	0	0
	1<br>Osjap03g41270.1 0.007 0.297 0.29 0.191 0.227<br>Osjap03g43190.1 0.297 0.993 0.696 0.742 0.993	1<br>Osind03g42330.1 0.007 0.297 0.29 0.211 0.259<br>Osind03g44220.1 0.297 0.993 0.696 0.734 0.993	1<br>Orufi03g39770.1 0.007 0.297 0.29 0.204 0.242<br>Orufi03g41620.1 0.297 0.855 0.558 0.779 0.998	1<br>Oglab03g36140.1 0.007 0.297 0.29 0.191 0.227<br>Oglab03g37860.1 0.297 0.993 0.696 0.742 0.993	1<br>Obart03g38160.1 0.007 0.297 0.29 0.203 0.242<br>Obart03g39810.1 0.297 0.993 0.696 0.742 0.993	1<br>Olong_002950-R1 0.036 0.471 0.435 0.593 0.685<br>Olong_020567-R1 0.529 1 0.471 0.328 1	1<br>Opunc03g35260.1 0.007 0.297 0.29 0.248 0.294<br>Opunc03g37040.1 0.297 0.964 0.667 0.742 0.984	1<br>Obrac03g29980.1 0.297 0.957 0.66 0.725 0.981<br>Obrac08g08500.1 0.007 0.297 0.29 0.058 0.116	0<br>Lperr03g33960.1 0.196 0.964 0.768 0.723 0.985	0<br>AT1G08450.1 0.196 0.667 0.471 0.745 0.901
Oglab09g13510.1	1<br>Osjap09g15430.2 0.002 1 0.998 0.002 1<br>Osjap09g15430.3 0.002 1 0.998 0.002 1	1<br>Osind09g15570.1 0.002 1 0.998 0.002 1<br>Osind09g15570.2 0.002 1 0.998 0.002 1	1<br>Orufi09g16160.1 0.002 1 0.998 0.002 1<br>Orufi09g16160.2 0.002 1 0.998 0.002 1	1<br>Oglab09g13510.1 0.002 1 0.998 0.002 1<br>Oglab09g13510.2 0.002 1 0.998 0.002 1<br>Oglab09g13510.3 0.002 1 0.998 0.002 1<br>Oglab09g13510.4 0.002 1 0.998 0.002 1	1<br>Obart09g14980.1 0.002 1 0.998 0.002 1<br>Obart09g14980.2 0.002 1 0.998 0.002 1	0	1<br>Opunc09g13420.1 0.002 1 0.998 0.002 1<br>Opunc09g13420.2 0.002 1 0.998 0.002 1	1<br>Obrac09g09090.1 0.002 1 0.998 0.002 1	0	0
	1<br>Osjap02g06650.1 0.009 0.501 0.492 0.01 0.997<br>Osjap09g15430.1 0.514 1 0.486 0.003 1	1<br>Osind02g06940.1 0.009 0.501 0.492 0.01 0.997<br>Osind08g23920.1 0.519 0.994 0.475 0.012 0.988	1<br>Orufi04g24220.1 0.012 0.502 0.49 0.165 1<br>Orufi08g22880.1 0.519 0.994 0.475 0.012 0.988	1<br>Oglab08g19240.1 0.519 0.994 0.475 0.014 0.986<br>Oglab12g18680.1 0.002 0.501 0.499 0.003 0.997	1<br>Obart02g06180.1 0.009 0.501 0.492 0.01 0.997<br>Obart08g20390.1 0.501 0.994 0.493 0.566 0.988	1<br>Olong_018059-R1 0.518 0.992 0.474 0.046 0.957<br>Olong_024186-R1 0.002 0.501 0.499 0.003 0.997	1<br>Opunc02g05570.1 0.005 0.501 0.496 0.087 0.997<br>Opunc08g18560.1 0.519 0.994 0.475 0.012 0.969	0<br>Obrac08g13590.1 0.499 0.994 0.495 0.689 0.994	1<br>Lperr04g18350.1 0.002 0.501 0.499 0.021 0.997<br>Lperr08g17230.1 0.507 0.994 0.487 0.021 0.997	1<br>AT2G21270.3 0.003 0.501 0.498 0.059 0.997<br>AT5G42800.1 0.516 0.992 0.476 0.003 0.851
Oglab09g13510.2	1<br>Osjap09g15430.2 0.002 1 0.998 0.002 1<br>Osjap09g15430.3 0.002 1 0.998 0.002 1	1<br>Osind09g15570.1 0.002 1 0.998 0.002 1<br>Osind09g15570.2 0.002 1 0.998 0.002 1	1<br>Orufi09g16160.1 0.002 1 0.998 0.002 1<br>Orufi09g16160.2 0.002 1 0.998 0.002 1	1<br>Oglab09g13510.1 0.002 1 0.998 0.002 1<br>Oglab09g13510.2 0.002 1 0.998 0.002 1<br>Oglab09g13510.3 0.002 1 0.998 0.002 1<br>Oglab09g13510.4 0.002 1 0.998 0.002 1	1<br>Obart09g14980.1 0.002 1 0.998 0.002 1<br>Obart09g14980.2 0.002 1 0.998 0.002 1	0	1<br>Opunc09g13420.1 0.002 1 0.998 0.002 1<br>Opunc09g13420.2 0.002 1 0.998 0.002 1	1<br>Obrac09g09090.1 0.002 1 0.998 0.002 1	0	0
	1<br>Osjap02g06650.1 0.01 0.522 0.512 0.01 0.997<br>Osjap09g15430.1 0.537 1 0.463 0.003 1	1<br>Osind02g06940.1 0.01 0.522 0.512 0.01 0.997<br>Osind08g23920.1 0.541 0.994 0.453 0.012 0.988	1<br>Orufi04g24220.1 0.013 0.524 0.511 0.165 1<br>Orufi08g22880.1 0.541 0.994 0.453 0.012 0.988	1<br>Oglab08g19240.1 0.541 0.994 0.453 0.014 0.986<br>Oglab12g18680.1 0.002 0.522 0.52 0.003 0.997	1<br>Obart02g06180.1 0.01 0.522 0.512 0.01 0.997<br>Obart08g20390.1 0.522 0.994 0.472 0.566 0.988	1<br>Olong_014421-R1 0.537 0.992 0.455 0.002 0.984<br>Olong_024186-R1 0.002 0.522 0.52 0.003 0.997	1<br>Opunc02g05570.1 0.005 0.522 0.517 0.087 0.997<br>Opunc08g18560.1 0.541 0.994 0.453 0.012 0.969	1<br>Obrac02g04550.3 0.008 0.522 0.514 0.214 0.997<br>Obrac09g00560.1 0.54 0.992 0.452 0.043 0.954	1<br>Lperr04g18350.1 0.002 0.522 0.52 0.021 0.997<br>Lperr08g17230.1 0.529 0.994 0.465 0.021 0.997	1<br>AT1G15950.1 0.538 0.992 0.454 0.015 0.936<br>AT2G21270.3 0.003 0.522 0.519 0.059 0.997
Oglab09g13510.3	1<br>Osjap09g15430.2 0.002 1 0.998 0.002 1<br>Osjap09g15430.3 0.002 1 0.998 0.002 1	1<br>Osind09g15570.1 0.002 1 0.998 0.002 1<br>Osind09g15570.2 0.002 1 0.998 0.002 1	1<br>Orufi09g16160.1 0.002 1 0.998 0.002 1<br>Orufi09g16160.2 0.002 1 0.998 0.002 1	1<br>Oglab09g13510.1 0.002 1 0.998 0.002 1<br>Oglab09g13510.2 0.002 1 0.998 0.002 1	1<br>Obart09g14980.1 0.002 1 0.998 0.002 1<br>Obart09g14980.2 0.002 1 0.998 0.002 1	0	1<br>Opunc09g13420.1 0.002 1 0.998 0.002 1<br>Opunc09g13420.2 0.002 1 0.998 0.002 1	1<br>Obrac09g09090.1 0.002 1 0.998 0.002 1	0	0

				Oglab09g13510.3 0.002 1 0.998 0.002 1<br>Oglab09g13510.4 0.002 1 0.998 0.002 1						
	1<br>Osjap02g06650.1 0.01 0.54 0.53 0.01 0.997<br>Osjap09g15430.1 0.544 1 0.456 0.131 1	1<br>Osind02g06940.1 0.01 0.54 0.53 0.01 0.997<br>Osind08g23920.1 0.544 0.993 0.449 0.131 0.988	1<br>Orufi04g24220.1 0.013 0.542 0.529 0.165 1<br>Orufi08g22880.1 0.544 0.993 0.449 0.131 0.988	1<br>Oglab08g19240.1 0.544 0.993 0.449 0.143 0.986<br>Oglab12g18680.1 0.002 0.542 0.54 0.003 1	1<br>Obart02g06180.1 0.01 0.54 0.53 0.01 0.997<br>Obart08g20390.1 0.544 0.993 0.449 0.631 0.988	1<br>Olong_013054-R1 0.547 0.992 0.445 0.149 0.979<br>Olong_024186-R1 0.002 0.542 0.54 0.003 1	1<br>Opunc04g20190.1 0.007 0.542 0.535 0.006 1<br>Opunc08g18560.1 0.544 0.993 0.449 0.128 0.969	1<br>Obrac02g04550.3 0.008 0.54 0.532 0.214 0.997<br>Obrac09g00560.1 0.548 0.998 0.45 0.164 0.966	1<br>Lperr04g18350.1 0.002 0.542 0.54 0.021 1<br>Lperr09g11120.1 0.544 1 0.456 0.156 1	1<br>AT1G68540.1 0.542 0.992 0.45 0.112 1<br>AT2G21270.3 0.003 0.54 0.537 0.059 0.997
Oglab09g13510.4	1<br>Osjap09g15430.2 0.002 1 0.998 0.002 1<br>Osjap09g15430.3 0.002 1 0.998 0.002 1	1<br>Osind09g15570.1 0.002 1 0.998 0.002 1<br>Osind09g15570.2 0.002 1 0.998 0.002 1	1<br>Orufi09g16160.1 0.002 1 0.998 0.002 1<br>Orufi09g16160.2 0.002 1 0.998 0.002 1	1<br>Oglab09g13510.1 0.002 1 0.998 0.002 1<br>Oglab09g13510.2 0.002 1 0.998 0.002 1<br>Oglab09g13510.3 0.002 1 0.998 0.002 1<br>Oglab09g13510.4 0.002 1 0.998 0.002 1	1<br>Obart09g14980.1 0.002 1 0.998 0.002 1<br>Obart09g14980.2 0.002 1 0.998 0.002 1	0	1<br>Opunc09g13420.1 0.002 1 0.998 0.002 1<br>Opunc09g13420.2 0.002 1 0.998 0.002 1	1<br>Obrac09g09090.1 0.002 1 0.998 0.002 1	0	0
	1<br>Osjap02g06650.1 0.01 0.565 0.555 0.01 0.997<br>Osjap09g15430.1 0.569 1 0.431 0.131 1	1<br>Osind02g06940.1 0.01 0.565 0.555 0.01 0.997<br>Osind08g23920.1 0.569 0.993 0.424 0.131 0.988	1<br>Orufi04g24220.1 0.014 0.567 0.553 0.165 1<br>Orufi08g22880.1 0.569 0.993 0.424 0.131 0.988	1<br>Oglab08g19240.1 0.569 0.993 0.424 0.143 0.986<br>Oglab12g18680.1 0.002 0.567 0.565 0.003 1	1<br>Obart02g06180.1 0.01 0.565 0.555 0.01 0.997<br>Obart08g20390.1 0.569 0.993 0.424 0.631 0.988	1<br>Olong_008093-R2 0.596 0.991 0.395 0.25 0.996<br>Olong_024186-R1 0.002 0.567 0.565 0.003 1	1<br>Opunc04g20190.1 0.007 0.567 0.56 0.006 1<br>Opunc08g18560.1 0.569 0.993 0.424 0.128 0.969	1<br>Obrac01g09090.1 0.605 0.998 0.393 0.299 0.982<br>Obrac02g04550.3 0.009 0.565 0.556 0.214 0.997	1<br>Lperr04g18350.1 0.002 0.567 0.565 0.021 1<br>Lperr09g11120.1 0.569 1 0.431 0.156 1	1<br>AT1G68540.1 0.567 0.991 0.424 0.112 1<br>AT2G21270.3 0.003 0.565 0.562 0.059 0.997
Oglab09g18200.1	1<br>Osjap09g20460.1 0.001 0.999 0.998 0.001 0.999	1<br>Osind09g20560.1 0.001 0.985 0.984 0.001 0.998	1<br>Orufi09g21510.1 0.001 0.958 0.957 0.001 0.999	1<br>Oglab09g18200.1 0.001 1 0.999 0.001 1	1<br>Obart09g19980.1 0.066 1 0.934 0.181 1<br>Obart09g19980.2 0.001 1 0.999 0.001 1	1<br>Olong_014589-R1 0.116 0.972 0.856 0.032 0.991<br>Olong_014589-R2 0.116 0.972 0.856 0.033 0.991	0	0	0	0
	1<br>Osjap02g21530.2 0.023 0.609 0.586 0.096 0.943<br>Osjap09g20430.1 0.649 0.999 0.35 0.151 0.998	1<br>Osind02g21430.2 0.023 0.609 0.586 0.096 0.943<br>Osind09g20550.1 0.619 0.999 0.38 0.058 0.999	1<br>Orufi02g19810.2 0.023 0.609 0.586 0.096 0.943<br>Orufi09g21500.1 0.649 0.999 0.35 0.151 0.998	1<br>Oglab02g17850.1 0.023 0.609 0.586 0.095 0.914<br>Oglab09g18170.1 0.619 0.999 0.38 0.13 0.998	1<br>Obart04g25370.1 0.047 0.617 0.57 0.176 0.967<br>Obart09g19950.1 0.619 0.999 0.38 0.13 0.998	0<br>Olong_014116-R1 0.063 0.617 0.554 0.069 0.957	1<br>Opunc09g18280.1 0.619 0.998 0.379 0.118 0.951<br>Opunc09g18290.2 0.001 0.618 0.617 0.001 0.971	1<br>Obrac09g13140.3 0.619 0.985 0.366 0.089 0.806<br>Obrac09g13160.1 0.001 0.618 0.617 0.001 0.953	1<br>Lperr09g15890.1 0.619 0.997 0.378 0.085 0.957<br>Lperr09g15920.1 0.001 0.618 0.617 0.001 0.958	0<br>AT1G18160.1 0.021 0.617 0.596 0.064 0.958
Oglab10g03910.1	1<br>Osjap10g04950.1 0.01 1 0.99 0.01 1<br>Osjap10g21210.1 0.01 1 0.99 0.477 1	1<br>Osind10g03420.1 0.01 1 0.99 0.558 1<br>Osind10g17700.1 0.01 1 0.99 0.477 1	1<br>Orufi10g04520.1 0.01 1 0.99 0.01 1<br>Orufi10g19420.1 0.01 1 0.99 0.477 1	1<br>Oglab10g03910.1 0.01 1 0.99 0.01 1	1<br>Obart10g18220.1 0.01 1 0.99 0.392 0.823	1<br>Olong_009744-R1 0.01 1 0.99 0.651 1<br>Olong_009744-R2 0.01 1 0.99 0.641 1	0	0	0	0
	0<br>Osjap07g13660.9 0.361 1 0.639 0.733 0.947	1<br>Osind02g38620.1 0.361 1 0.639 0.835 0.923<br>Osind06g00010.1 0.01 0.361 0.351 0.609 0.978	1<br>Orufi02g36440.1 0.361 1 0.639 0.835 0.923<br>Orufi08g26670.5 0.01 0.351 0.341 0.583 0.749	1<br>Oglab02g32260.1 0.361 1 0.639 0.838 0.924<br>Oglab04g27900.1 0.01 0.33 0.32 0.14 0.208	1<br>Obart02g34980.1 0.361 1 0.639 0.838 0.924<br>Obart04g29850.1 0.01 0.33 0.32 0.131 0.196	1<br>Olong_008835-R2 0.361 1 0.639 0.809 0.91<br>Olong_022843-R2 0.01 0.33 0.32 0.14 0.208	1<br>Opunc02g32260.1 0.361 1 0.639 0.84 0.925<br>Opunc04g27380.2 0.01 0.33 0.32 0.14 0.208	1<br>Obrac02g25650.1 0.381 1 0.619 0.839 0.923<br>Obrac04g21100.1 0.01 0.33 0.32 0.145 0.217	1<br>Lperr02g28770.1 0.381 1 0.619 0.834 0.92<br>Lperr04g25370.2 0.01 0.33 0.32 0.151 0.225	0
Oglab11g02200.1	1<br>Osjap11g02640.1 0.002 1 0.998 0.002 1<br>Osjap12g02610.1 0.002 1 0.998 0.002 1	1<br>Osind11g01990.1 0.002 1 0.998 0.002 1<br>Osind12g02780.1 0.002 1 0.998 0.002 1	1<br>Orufi11g02320.1 0.002 1 0.998 0.003 1<br>Orufi12g02480.1 0.002 1 0.998 0.002 1	1<br>Oglab11g02200.1 0.002 1 0.998 0.002 1<br>Oglab12g02130.1 0.002 1 0.998 0.002 1	1<br>Obart12g02080.1 0.023 1 0.977 0.002 1	0	0	0	0	0
	1<br>Osjap07g04360.1 0.032 0.657 0.625 0.018 0.925<br>Osjap12g12870.1 0.66 0.951 0.291 0.055 0.417	1<br>Osind11g02010.1 0.013 0.657 0.644 0.001 0.994<br>Osind12g10020.1 0.66 0.951 0.291 0.055 0.417	1<br>Orufi07g03650.1 0.032 0.657 0.625 0.019 0.976<br>Orufi12g10750.1 0.658 0.951 0.293 0.045 0.364	1<br>Oglab07g03810.1 0.032 0.657 0.625 0.019 0.987<br>Oglab12g09880.1 0.66 0.951 0.291 0.051 0.385	0<br>Obart11g02350.1 0.021 0.657 0.636 0.004 0.993	1<br>Olong_012584-R1 0.064 0.611 0.547 0.002 0.965<br>Olong_017316-R1 0.658 0.951 0.293 0.045 0.365	1<br>Opunc11g02190.1 0.002 0.657 0.655 0.003 0.989<br>Opunc12g08740.1 0.658 0.951 0.293 0.04 0.324	1<br>Obrac05g10860.1 0.049 0.657 0.608 0.097 0.987<br>Obrac12g07170.1 0.658 0.942 0.284 0.071 0.57	1<br>Lperr12g02190.1 0.002 0.657 0.655 0.003 0.989<br>Lperr12g08370.1 0.657 0.951 0.294 0.047 0.391	0<br>AT5G27320.1 0.013 0.632 0.619 0.003 0.98
Oglab12g00800.1	1<br>Osjap11g01220.1 0.001 1 0.999 0.001 1<br>Osjap12g01140.1 0.001 1 0.999 0.001 1	1<br>Osind11g00690.1 0.001 0.956 0.955 0.001 0.999<br>Osind12g01290.1 0.001 1 0.999 0.001 1	1<br>Orufi11g01000.1 0.001 1 0.999 0.001 1<br>Orufi12g01130.1 0.001 1 0.999 0.001 1	1<br>Oglab12g00800.1 0.001 1 0.999 0.001 1	1<br>Obart11g01060.1 0.001 1 0.999 0.001 1	0	1<br>Opunc12g00940.1 0.001 1 0.999 0.001 1	0	0	0
	1	1	1	1	1	1	1	1	1	1

	Osjap03g13100.1 0.49 0.983 0.493 0.097 0.622<br>Osjap07g30390.1 0.009 0.393 0.384 0.013 0.938	Osind03g12930.1 0.49 0.983 0.493 0.121 0.633<br>Osind07g27070.1 0.006 0.393 0.387 0.006 0.939	Orufi03g17220.1 0.496 1 0.504 0.027 1<br>Orufi07g27420.1 0.009 0.393 0.384 0.013 0.939	Oglab03g12120.1 0.49 0.983 0.493 0.121 0.633<br>Oglab07g22980.1 0.006 0.393 0.387 0.007 0.933	Obart03g12120.1 0.49 0.983 0.493 0.121 0.633<br>Obart12g00790.1 0.001 0.393 0.392 0.002 0.942	Olong_007715-R1 0.493 1 0.507 0.007 1<br>Olong_008211-R1 0.006 0.393 0.387 0.006 0.939	Opunc03g11680.1 0.49 1 0.51 0.148 1<br>Opunc11g00920.1 0.001 0.393 0.392 0.142 0.945	Obrac03g09720.2 0.49 1 0.51 0.142 1<br>Obrac06g16520.1 0.02 0.411 0.391 0.048 0.978	Lperr03g10720.1 0.49 0.996 0.506 0.148 0.954<br>Lperr11g00920.1 0.001 0.393 0.392 0.002 0.942	AT3G46740.1 0.493 1 0.507 0.161 1<br>AT4G20110.2 0.001 0.393 0.392 0.002 0.939
Oglab12g09470.1	1<br>Osjap12g12320.1 0.001 1 0.999 0.001 1	1<br>Osind12g09530.1 0.001 1 0.999 0.001 1	1<br>Orufi12g10460.1 0.001 1 0.999 0.001 1	1<br>Oglab12g09470.1 0.001 1 0.999 0.001 1	1<br>Obart12g09410.1 0.001 1 0.999 0.001 1	0	0	0	0	0
	1<br>Osjap01g44780.2 0.44 0.998 0.558 0.095 0.994<br>Osjap03g07600.1 0.001 0.295 0.294 0.004 0.981	1<br>Osind01g46040.1 0.44 0.998 0.558 0.151 0.995<br>Osind03g07670.1 0.001 0.295 0.294 0.004 0.981	1<br>Orufi01g42650.3 0.44 0.998 0.558 0.195 0.995<br>Orufi03g07180.1 0.001 0.295 0.294 0.004 0.981	1<br>Oglab01g34040.1 0.489 0.998 0.509 0.203 0.995<br>Oglab03g07280.1 0.001 0.295 0.294 0.004 0.981	1<br>Obart01g39260.3 0.456 0.998 0.542 0.041 0.994<br>Obart03g07210.1 0.001 0.295 0.294 0.004 0.981	1<br>Olong_021912-R1 0.457 1 0.543 0.099 1<br>Olong_021913-R1 0.001 0.296 0.295 0.004 0.988	1<br>Opunc12g08570.1 0.326 1 0.674 0.052 1<br>Opunc12g08580.1 0.001 0.296 0.295 0.004 0.988	1<br>Obrac03g05880.1 0.001 0.296 0.295 0.004 0.988<br>Obrac12g06500.1 0.461 1 0.539 0.103 1	1<br>Lperr01g33810.2 0.456 0.998 0.542 0.002 0.994<br>Lperr12g08300.1 0.001 0.296 0.295 0.004 0.985	0<br>AT4G34670.1 0.001 0.29 0.289 0.004 0.966<br>AT5G67540.2 0.489 0.993 0.504 0.053 1

((((((((jap,indica),rufi),(glab,barthii)),longi),punctata),brachy),leersia),tair);

chimeric gene id	INGROUP	MIDGROUP						OUTGROUP		
	barthii	jap	indica	rufi	glab	longi	punctata	brachy	leersia	tair
Obart01g01710.1	1<br>Obart01g01710.1 0.001 1 0.999 0.001 1	0	1<br>Osind01g02220.1 0.001 0.986 0.985 0.056 0.987	0	1<br>Oglab01g01590.1 0.001 0.986 0.985 0.001 0.987	1<br>Olong_021362-R1 0.001 0.986 0.985 0.048 0.986	1<br>Opunc01g01490.1 0.002 1 0.998 0.021 0.999<br>Opunc01g01490.2 0.002 1 0.998 0.024 0.999	0	0	0
	1<br>Obart01g01680.1 0.539 0.986 0.447 0.046 0.97<br>Obart05g03720.1 0.002 0.47 0.468 0.104 0.976	1<br>Osjap01g02100.1 0.001 0.47 0.469 0.002 0.973<br>Osjap01g02110.1 0.513 0.986 0.473 0.039 0.974	1<br>Osind01g02230.1 0.513 0.999 0.486 0.061 0.989<br>Osind05g03930.1 0.002 0.47 0.468 0.104 0.976	1<br>Orufi01g01810.1 0.001 0.47 0.469 0.002 0.973<br>Orufi01g01830.1 0.539 0.986 0.447 0.079 0.987	1<br>Oglab01g01570.1 0.539 0.986 0.447 0.043 0.973<br>Oglab05g03370.1 0.002 0.47 0.468 0.104 0.976	1<br>Olong_016471-R1 0.002 0.47 0.468 0.106 0.972<br>Olong_021361-R1 0.515 0.955 0.44 0.002 0.971	1<br>Opunc01g01490.3 0.539 0.986 0.447 0.056 0.97<br>Opunc05g03460.1 0.016 0.47 0.454 0.119 0.971	1<br>Obrac01g01340.1 0.486 0.986 0.5 0.001 0.999<br>Obrac01g17910.1 0.004 0.47 0.466 0.248 0.98	1<br>Lperr01g01640.1 0.51 0.986 0.476 0.051 0.975<br>Lperr05g03400.1 0.015 0.47 0.455 0.158 0.972	1<br>AT1G71040.1 0.528 0.986 0.458 0.021 0.972<br>AT3G48670.1 0.002 0.47 0.468 0.117 0.971
Obart01g04220.1	1<br>Obart01g04220.1 0.001 1 0.999 0.001 1	0	0	0	1<br>Oglab01g03790.1 0.001 1 0.999 0.001 1	0	0	0	0	0
	1<br>Obart05g04510.1 0.036 0.567 0.531 0.012 1<br>Obart07g07920.1 0.62 0.997 0.377 0.002 0.778	1<br>Osjap01g04780.1 0.62 1 0.38 0.002 1<br>Osjap05g04880.1 0.048 0.567 0.519 0.008 1	1<br>Osind01g04880.1 0.62 1 0.38 0.002 1<br>Osind05g04760.1 0.048 0.567 0.519 0.008 1	1<br>Orufi01g04370.1 0.62 1 0.38 0.002 1<br>Orufi05g04740.1 0.048 0.567 0.519 0.008 1	1<br>Oglab05g04170.2 0.048 0.567 0.519 0.114 1<br>Oglab07g07650.1 0.62 0.997 0.377 0.002 0.778	1<br>Olong_014225-R1 0.044 0.563 0.519 0.001 0.984<br>Olong_014227-R1 0.62 1 0.38 0.002 1	1<br>Opunc01g03980.1 0.62 1 0.38 0.002 1<br>Opunc07g16230.1 0.042 0.556 0.514 0.014 0.972	1<br>Obrac01g03070.1 0.62 1 0.38 0.002 1<br>Obrac05g03450.2 0.039 0.567 0.528 0.003 1	1<br>Lperr01g03770.1 0.62 1 0.38 0.002 1<br>Lperr01g03780.1 0.039 0.561 0.522 0.002 0.979	1<br>AT5G01660.1 0.053 0.556 0.503 0.015 0.97<br>AT5G64190.1 0.62 0.997 0.377 0.002 0.998
Obart01g07430.1	1<br>Obart01g07430.1 0.001 1 0.999 0.001 1	1<br>Osjap01g08660.1 0.007 1 0.993 0.026 1	1<br>Osind01g08490.1 0.007 1 0.993 0.026 1	1<br>Orufi01g08070.1 0.007 1 0.993 0.026 1	1<br>Oglab01g06470.1 0.013 1 0.987 0.02 1	1<br>Olong_011567-R1 0.036 1 0.964 0.001 1	0	0	0	0
	1<br>Obart01g25090.1 0.078 0.648 0.57 0.219 0.982<br>Obart05g00540.1 0.713 0.997 0.284 0.146 1	1<br>Osjap01g29230.2 0.078 0.648 0.57 0.25 0.983<br>Osjap05g00560.2 0.713 0.997 0.284 0.146 1	1<br>Osind01g30690.1 0.078 0.648 0.57 0.267 0.982<br>Osind05g00540.1 0.713 0.997 0.284 0.126 1	1<br>Orufi01g27950.1 0.078 0.648 0.57 0.25 0.983<br>Orufi01g48560.2 0.713 0.983 0.27 0.085 0.984	1<br>Oglab01g21070.2 0.078 0.648 0.57 0.249 0.983<br>Oglab05g00530.1 0.713 0.997 0.284 0.146 1	1<br>Olong_003039-R1 0.713 0.997 0.284 0.128 1<br>Olong_003692-R2 0.109 0.648 0.539 0.244 0.979	1<br>Opunc01g07160.1 0.656 1 0.344 0.111 1<br>Opunc01g07170.1 0.003 0.649 0.646 0.034 0.984	1<br>Obrac01g05760.3 0.66 1 0.34 0.082 1<br>Obrac01g18840.2 0.001 0.648 0.647 0.062 0.974	1<br>Lperr01g07030.1 0.66 1 0.34 0.018 1<br>Lperr01g07040.2 0.002 0.649 0.647 0.013 0.989	1<br>AT1G54350.1 0.659 0.994 0.335 0.076 0.975<br>AT3G61740.1 0.009 0.648 0.639 0.077 0.983
Obart01g26420.1	1<br>Obart01g26420.1 0.002 1 0.998 0.002 1<br>Obart01g26420.2 0.002 1 0.998 0.002 1	0	0	1<br>Orufi01g29390.1 0.002 1 0.998 0.002 1	0	0	0	0	0	0
	1<br>Obart07g20970.1 0.588 0.828 0.24 0.185 0.779<br>Obart11g13020.1 0.002 0.574 0.572 0.006 1	1<br>Osjap01g30690.1 0.002 0.585 0.583 0.003 0.997<br>Osjap07g24440.1 0.588 0.907 0.319 0.16 0.718	1<br>Osind01g32110.1 0.002 0.585 0.583 0.003 0.997<br>Osind07g21300.1 0.588 0.907 0.319 0.159 0.713	1<br>Orufi07g21990.1 0.588 0.852 0.264 0.175 0.751<br>Orufi11g13800.1 0.002 0.574 0.572 0.006 1	1<br>Oglab01g22320.1 0.002 0.585 0.583 0.003 0.997<br>Oglab07g18060.1 0.588 0.907 0.319 0.202 0.904	1<br>Olong_006531-R1 0.588 0.907 0.319 0.176 0.786<br>Olong_023995-R1 0.002 0.585 0.583 0.005 0.995	1<br>Opunc01g26380.1 0.002 0.585 0.583 0.003 0.997<br>Opunc07g12980.1 0.588 0.878 0.29 0.158 0.699	1<br>Obrac01g20020.1 0.002 0.585 0.583 0.003 0.997<br>Obrac02g00620.1 0.59 0.83 0.24 0.16 0.42	1<br>Lperr01g22600.1 0.002 0.585 0.583 0.003 0.997<br>Lperr07g18200.1 0.587 0.852 0.265 0.16 0.7	0<br>AT1G52155.1 0.013 0.553 0.54 0.022 0.997<br>AT4G20140.1 0.579 0.82 0.241 0.155 0.649
Obart01g26420.2	1<br>Obart01g26420.1 0.002 1 0.998 0.002 1<br>Obart01g26420.2 0.002 1 0.998 0.002 1	0	0	1<br>Orufi01g29390.1 0.002 1 0.998 0.002 1	0	0	0	0	0	0
	1<br>Obart07g20970.1 0.566 0.819 0.253 0.185 0.779<br>Obart11g13020.1 0.002 0.551 0.549 0.006 1	1<br>Osjap01g30690.1 0.002 0.563 0.561 0.003 0.997<br>Osjap07g24440.1 0.566 0.902 0.336 0.16 0.718	1<br>Osind01g32110.1 0.002 0.563 0.561 0.003 0.997<br>Osind07g21300.1 0.566 0.902 0.336 0.159 0.713	1<br>Orufi07g21990.1 0.566 0.844 0.278 0.175 0.751<br>Orufi11g13800.1 0.002 0.551 0.549 0.006 1	1<br>Oglab01g22320.1 0.002 0.563 0.561 0.003 0.997<br>Oglab07g18060.1 0.566 0.902 0.336 0.202 0.904	1<br>Olong_006531-R1 0.566 0.902 0.336 0.176 0.786<br>Olong_023995-R1 0.002 0.563 0.561 0.005 0.995	1<br>Opunc01g26380.1 0.002 0.563 0.561 0.003 0.997<br>Opunc07g12980.1 0.564 0.871 0.307 0.134 0.699	1<br>Obrac01g20020.1 0.002 0.563 0.561 0.003 0.997<br>Obrac02g00620.1 0.568 0.82 0.252 0.16 0.42	0<br>Lperr01g22600.1 0.002 0.563 0.561 0.003 0.997<br>Lperr08g10350.1 0.564 0.768 0.204 0.191 0.711	0<br>AT1G52155.1 0.014 0.529 0.515 0.022 0.997<br>AT4G20140.1 0.556 0.81 0.254 0.155 0.649
Obart01g26930.1	1<br>Obart01g26930.1 0.001 1 0.999 0.001 1	0	0	0	0	0	0	0	0	0

	1<br>Obart03g32590.1 0.001 0.39 0.389 0.002 0.921<br>Obart07g24100.1 0.404 0.927 0.523 0.166 0.828	1<br>Osjap01g31200.1 0.001 0.395 0.394 0.002 1<br>Osjap01g31210.1 0.404 0.959 0.555 0.2 0.999	1<br>Osind01g32660.1 0.001 0.395 0.394 0.002 0.972<br>Osind01g32670.1 0.404 0.959 0.555 0.198 0.999	1<br>Orufi01g29940.1 0.001 0.395 0.394 0.002 1<br>Orufi07g25280.1 0.404 0.927 0.523 0.17 0.829	1<br>Oglab01g22800.1 0.001 0.395 0.394 0.002 1<br>Oglab01g22810.1 0.406 0.959 0.553 0.148 0.999	1<br>Olong_000370-R1 0.001 0.395 0.394 0.426 1<br>Olong_012653-R1 0.404 0.927 0.523 0.168 0.829	1<br>Opunc01g26880.1 0.404 0.962 0.558 0.076 1<br>Opunc03g31650.1 0.01 0.393 0.383 0.017 0.936	1<br>Obrac01g20460.1 0.404 0.959 0.555 0.01 0.998<br>Obrac03g24030.1 0.001 0.366 0.365 0.002 0.841	1<br>Lperr03g07390.1 0.099 0.378 0.279 0.169 0.842<br>Lperr06g07900.1 0.404 0.927 0.523 0.173 0.936	0<br>AT5G28740.1 0.404 0.927 0.523 0.149 0.83
Obart01g35030.1	1<br>Obart01g35030.1 0.002 1 0.998 0.002 1	0	1<br>Osind01g41670.1 0.005 1 0.995 0.009 1	1<br>Orufi01g38340.1 0.005 1 0.995 0.01 1	0	1<br>Olong_021176-R1 0.005 0.998 0.993 0.007 0.998	1<br>Opunc01g34010.1 0.023 0.906 0.883 0 0.263<br>Opunc01g34010.2 0.023 0.906 0.883 0 0.259<br>Opunc01g34010.3 0.023 0.906 0.883 0 0.257	0	0	0
	1<br>Obart01g35020.1 0.028 0.743 0.715 0.001 0.174<br>Obart04g22680.1 0.743 1 0.257 0.597 1	1<br>Osjap01g40250.2 0.005 0.743 0.738 0.002 0.17<br>Osjap06g18760.1 0.745 0.995 0.25 0.361 0.988	1<br>Osind01g41660.1 0.028 0.743 0.715 0.001 0.162<br>Osind05g11680.1 0.743 0.991 0.248 0.378 0.977	1<br>Orufi01g38330.1 0.028 0.743 0.715 0.001 0.174<br>Orufi06g17080.1 0.745 0.995 0.25 0.312 0.987	1<br>Oglab01g29710.1 0.037 0.743 0.706 0.024 0.188<br>Oglab04g21270.1 0.745 1 0.255 0.564 1	1<br>Olong_027118-R1 0.028 0.743 0.715 0.001 0.248<br>Olong_027361-R1 0.745 1 0.255 0.255 1	0<br>Opunc05g18500.1 0.034 0.564 0.53 0.029 0.929<br>Opunc07g02150.1 0.741 0.995 0.254 0.35 0.983	1<br>Obrac01g26970.1 0.028 0.743 0.715 0.001 0.169<br>Obrac05g03410.2 0.743 0.998 0.255 0.551 0.953	0<br>Lperr01g30210.1 0.028 0.743 0.715 0.001 0.167	0<br>AT2G35630.1 0.028 0.743 0.715 0.001 0.168
Obart01g40910.1	1<br>Obart01g40910.1 0.005 1 0.995 0.005 1	0	0	0	1<br>Oglab01g35730.1 0.005 1 0.995 0.005 1	0	0	0	0	0
	1<br>Obart02g07460.1 0.475 1 0.525 0.531 1<br>Obart08g06610.1 0.13 0.405 0.275 0.364 0.944	1<br>Osjap01g46570.1 0.41 1 0.59 0.156 1<br>Osjap10g12600.2 0.1 0.405 0.305 0.235 0.978	1<br>Osind01g47760.1 0.41 1 0.59 0.156 1<br>Osind08g01920.1 0.12 0.405 0.285 0.622 0.841	1<br>Orufi01g44230.1 0.41 1 0.59 0.156 1<br>Orufi08g02060.1 0.12 0.405 0.285 0.622 0.841	1<br>Oglab02g07520.1 0.475 1 0.525 0.255 1<br>Oglab07g00120.1 0.13 0.405 0.275 0.696 0.841	0<br>Olong_004144-R1 0.39 1 0.61 0.008 1	1<br>Opunc02g06610.1 0.475 1 0.525 0.245 1<br>Opunc08g01790.1 0.12 0.405 0.285 0.404 0.94	1<br>Obrac02g05500.1 0.475 1 0.525 0.234 1<br>Obrac03g27510.3 0.13 0.405 0.275 0.748 0.87	1<br>Lperr01g35480.1 0.475 1 0.525 0.228 1<br>Lperr03g31300.1 0.13 0.405 0.275 0.754 0.873	0<br>AT3G03740.1 0.13 0.405 0.275 0.662 0.781<br>AT3G27340.1 0.47 0.98 0.51 0.234 0.957
Obart02g00480.1	1<br>Obart02g00480.1 0.002 1 0.998 0.002 1	0	0	0	0	0	0	0	0	0
	1<br>Obart01g15210.1 0.002 0.438 0.436 0.004 0.86<br>Obart05g02080.1 0.458 0.91 0.452 0.062 0.875	1<br>Osjap01g17630.1 0.002 0.438 0.436 0.004 0.86<br>Osjap02g00500.2 0.471 0.91 0.439 0.068 0.877	1<br>Osind01g18080.1 0.002 0.438 0.436 0.004 0.86<br>Osind03g09080.1 0.45 0.91 0.46 0.144 0.919	1<br>Orufi01g16820.1 0.002 0.438 0.436 0.004 0.86<br>Orufi02g00500.5 0.471 0.91 0.439 0.075 0.757	1<br>Oglab01g12840.1 0.002 0.438 0.436 0.004 0.86<br>Oglab02g00410.1 0.458 1 0.542 0.069 1	1<br>Olong_016589-R3 0.458 0.91 0.452 0.126 0.885<br>Olong_022005-R1 0.002 0.438 0.436 0.004 0.95	1<br>Opunc02g00150.1 0.002 0.438 0.436 0.026 0.863<br>Opunc09g13920.1 0.444 0.908 0.464 0.008 0.876	1<br>Obrac02g00390.2 0.002 0.438 0.436 0.114 0.876<br>Obrac08g15590.5 0.498 0.91 0.412 0.138 0.797	1<br>Lperr01g13600.1 0.002 0.438 0.436 0.004 0.86<br>Lperr02g00490.1 0.458 0.912 0.454 0.091 0.863	0<br>AT5G07090.1 0.002 0.438 0.436 0.004 0.87
Obart02g11640.1	1<br>Obart02g11640.1 0.001 1 0.999 0.001 1	0	0	0	0	0	0	0	0	0
	1<br>Obart02g10250.1 0.001 0.476 0.475 0.002 0.996<br>Obart03g04220.1 0.479 1 0.521 0.048 0.998	1<br>Osjap02g11160.1 0.001 0.476 0.475 0.002 0.996<br>Osjap03g04250.1 0.479 0.999 0.52 0.067 0.996	1<br>Osind02g12980.1 0.497 1 0.503 0.03 1<br>Osind02g13000.1 0.001 0.476 0.475 0.002 1	1<br>Orufi02g10270.1 0.001 0.476 0.475 0.002 0.996<br>Orufi02g11670.1 0.487 1 0.513 0.012 1	1<br>Oglab02g10250.1 0.001 0.476 0.475 0.002 0.996<br>Oglab02g11670.1 0.483 1 0.517 0.002 1	1<br>Olong_009803-R1 0.479 1 0.521 0.002 0.998<br>Olong_026120-R1 0.001 0.476 0.475 0.002 0.996	1<br>Opunc02g10280.1 0.479 1 0.521 0.002 1<br>Opunc02g10300.1 0.001 0.475 0.474 0.002 0.998	1<br>Obrac02g07710.1 0.001 0.476 0.475 0.002 1<br>Obrac03g03340.1 0.52 1 0.48 0.136 0.998	0<br>Lperr10g08090.1 0.386 1 0.614 0.063 0.998	1<br>AT1G03890.1 0.001 0.457 0.456 0.002 0.991<br>AT2G37640.1 0.486 0.996 0.51 0.011 0.985
Obart02g11910.1	1<br>Obart02g11910.1 0.001 1 0.999 0.001 1	0	0	0	0	0	0	0	0	0
	1<br>Obart11g09030.1 0.004 0.616 0.612 0.054 0.994<br>Obart11g20370.3 0.649 0.995 0.346 0.001 0.275	1<br>Osjap02g13100.1 0.649 0.996 0.347 0.001 0.242<br>Osjap02g13110.1 0.001 0.616 0.615 0.002 0.998	1<br>Osind02g13300.1 0.001 0.616 0.615 0.2 0.993<br>Osind12g16510.1 0.641 0.996 0.355 0.002 0.632	1<br>Orufi02g11960.1 0.001 0.616 0.615 0.172 0.992<br>Orufi08g05130.1 0.624 0.974 0.35 0 0.595	1<br>Oglab02g11870.3 0.001 0.616 0.615 0.018 0.991<br>Oglab02g11880.1 0.649 0.996 0.347 0.001 0.282	1<br>Olong_006337-R1 0.004 0.616 0.612 0.108 0.998<br>Olong_026902-R1 0.647 0.996 0.349 0.073 0.821	1<br>Opunc02g10610.1 0.649 0.987 0.338 0.004 0.983<br>Opunc02g10620.1 0.001 0.616 0.615 0.042 0.998	0<br>Obrac12g13260.3 0.001 0.619 0.618 0.002 0.998	1<br>Lperr11g07730.1 0.649 0.996 0.347 0.001 0.325<br>Lperr11g07990.1 0.001 0.616 0.615 0.002 0.988	1<br>AT1G50180.1 0.649 0.978 0.329 0.001 0.285<br>AT4G26510.1 0.001 0.596 0.595 0.002 0.998
Obart02g23460.2	1<br>Obart02g23460.2 0.001 1 0.999 0.001 1	1<br>Osjap02g26750.1 0.001 0.969 0.968 0.001 0.994<br>Osjap02g26750.2 0.001 0.969 0.968 0.001 0.994	1<br>Osind02g26640.2 0.001 1 0.999 0.001 1	1<br>Orufi02g24900.1 0.011 0.974 0.963 0.06 0.923<br>Orufi02g24900.3 0.001 0.974 0.973 0.001 0.857	1<br>Oglab02g22130.2 0.001 1 0.999 0.001 1	0	1<br>Opunc02g21410.1 0.011 0.999 0.988 0.116 0.996<br>Opunc02g21410.2 0.011 0.999 0.988 0.114 0.996	0	1<br>Lperr02g18970.1 0.011 0.997 0.986 0.032 0.996	0

		Osjap02g26750.4 0.001 0.969 0.968 0.001 0.799		Orufi02g24900.4 0.001 0.974 0.973 0.001 0.856			Opunc02g21410.3 0.011 0.999 0.988 0.115 0.996<br>Opunc02g21410.4 0.011 0.999 0.988 0.114 0.996			
	1<br>Obart02g23290.1 0.475 0.999 0.524 0.029 0.999<br>Obart02g23440.1 0.011 0.459 0.448 0.492 0.981	1<br>Osjap02g26590.1 0.475 0.999 0.524 0.033 0.999<br>Osjap02g26750.5 0.011 0.459 0.448 0.238 0.973	1<br>Osind02g26450.1 0.475 0.999 0.524 0.025 0.997<br>Osind02g26620.1 0.011 0.459 0.448 0.495 0.981	1<br>Orufi02g24730.1 0.475 0.994 0.519 0.031 0.996<br>Orufi02g24880.2 0.011 0.459 0.448 0.18 0.97	1<br>Oglab02g21950.1 0.475 0.999 0.524 0.073 0.995<br>Oglab02g22110.2 0.011 0.459 0.448 0.532 0.98	0<br>Olong_024345-R1 0.011 0.474 0.463 0.442 0.991	1<br>Opunc02g21230.1 0.479 0.976 0.497 0.003 0.934<br>Opunc02g21240.1 0.011 0.461 0.45 0.121 0.972	1<br>Obrac02g16670.1 0.549 0.973 0.424 0.076 0.995<br>Obrac02g16700.1 0.011 0.461 0.45 0.114 0.947	1<br>Lperr02g18740.1 0.485 0.997 0.512 0.056 0.991<br>Lperr02g19120.1 0.011 0.484 0.473 0.183 0.978	1<br>AT4G31860.1 0.014 0.45 0.436 0.123 0.989<br>AT5G43080.1 0.626 1 0.374 0.194 0.932
Obart03g00550.1	1<br>Obart03g00550.1 0.001 1 0.999 0.001 1	1<br>Osjap03g00680.1 0.001 0.996 0.995 0.001 0.994<br>Osjap03g00680.2 0.001 0.996 0.995 0.001 0.994<br>Osjap03g00680.3 0.001 0.996 0.995 0.001 0.994	1<br>Osind03g00740.1 0.001 1 0.999 0.001 1	1<br>Orufi03g12660.1 0.001 0.996 0.995 0.001 0.994<br>Orufi03g12660.2 0.001 0.996 0.995 0.001 0.994	1<br>Oglab03g00390.1 0.001 0.996 0.995 0.001 0.994	0	0	0	0	0
	1<br>Obart02g37790.1 0.061 0.645 0.584 0.133 0.977<br>Obart12g06510.1 0.673 0.996 0.323 0.052 0.984	1<br>Osjap02g42090.1 0.052 0.675 0.623 0.065 1<br>Osjap12g08580.1 0.706 0.996 0.29 0.011 0.977	1<br>Osind02g41670.1 0.061 0.675 0.614 0.131 1<br>Osind03g02220.1 0.695 0.996 0.301 0.015 0.982	1<br>Orufi02g39350.1 0.061 0.675 0.614 0.131 1<br>Orufi09g02220.2 0.742 0.996 0.254 0.095 0.975	1<br>Oglab02g34920.1 0.061 0.675 0.614 0.131 1<br>Oglab04g12740.1 0.715 0.996 0.281 0.411 0.989	0<br>Olong_013175-R1 0.27 0.904 0.634 0.002 0.961	1<br>Opunc03g00470.1 0.001 0.78 0.779 0.002 1<br>Opunc03g03130.2 0.78 0.996 0.216 0.443 0.984	0<br>Obrac02g27970.1 0.136 0.675 0.539 0.105 1<br>Obrac05g17660.1 0.747 0.995 0.248 0.427 0.997	1<br>Lperr03g00570.1 0.001 0.675 0.674 0.002 1<br>Lperr04g11360.1 0.748 0.996 0.248 0.415 0.99	0<br>AT5G04460.1 0.018 0.675 0.657 0.081 1
Obart03g01190.1	1<br>Obart03g01190.1 0.002 1 0.998 0.002 1	0	0	0	0	0	0	0	0	0
	1<br>Obart01g33610.1 0.706 0.993 0.287 0.002 0.17<br>Obart03g14810.2 0.061 0.593 0.532 0.198 0.987	1<br>Osjap03g01290.1 0.655 0.993 0.338 0.001 0.15<br>Osjap03g01300.2 0.002 0.633 0.631 0.002 0.996	1<br>Osind03g01350.1 0.694 0.993 0.299 0.005 0.796<br>Osind07g20900.1 0.035 0.606 0.571 0.217 0.955	1<br>Orufi03g00970.1 0.655 0.993 0.338 0.004 0.815<br>Orufi03g00980.1 0.057 0.611 0.554 0.002 0.982	1<br>Oglab03g01040.1 0.694 0.993 0.299 0.005 0.796<br>Oglab03g01050.1 0.002 0.611 0.609 0.002 0.983	0<br>Olong_020901-R3 0.066 0.593 0.527 0.009 0.744	1<br>Opunc03g01090.1 0.655 0.993 0.338 0.004 0.814<br>Opunc03g01100.1 0.013 0.611 0.598 0.01 0.983	1<br>Obrac02g06700.4 0.011 0.593 0.582 0.435 0.868<br>Obrac03g00890.1 0.694 0.993 0.299 0.001 0.149	1<br>Lperr03g01130.1 0.694 0.993 0.299 0.001 0.131<br>Lperr03g01140.1 0.009 0.617 0.608 0.005 0.858	1<br>AT3G13230.1 0.712 0.993 0.281 0.102 0.814<br>AT3G53170.1 0.004 0.593 0.589 0.064 0.772
Obart03g02800.1	1<br>Obart03g02800.1 0.001 1 0.999 0.001 1	0	0	0	0	0	0	0	0	0
	1<br>Obart03g02810.1 0.589 0.978 0.389 0.002 0.993<br>Obart03g02820.1 0.01 0.583 0.573 0.013 0.989	1<br>Osjap03g02800.1 0.589 0.978 0.389 0.005 0.968<br>Osjap03g02820.1 0.01 0.583 0.573 0.013 0.989	1<br>Osind03g02900.1 0.01 0.583 0.573 0.013 0.989<br>Osind10g16860.1 0.589 0.978 0.389 0.004 0.992	1<br>Orufi01g17470.2 0.591 0.996 0.405 0.002 0.996<br>Orufi03g02520.1 0.017 0.583 0.566 0.025 0.989	1<br>Oglab03g02720.1 0.589 1 0.411 0.002 1<br>Oglab03g02730.1 0.01 0.583 0.573 0.013 0.989	1<br>Olong_005980-R1 0.589 0.978 0.389 0.002 0.989<br>Olong_005981-R1 0.017 0.583 0.566 0.03 0.987	1<br>Opunc03g02530.1 0.589 0.979 0.39 0.002 0.912<br>Opunc03g02550.1 0.017 0.583 0.566 0.02 0.989	1<br>Obrac03g02200.1 0.017 0.588 0.571 0.034 0.997<br>Obrac10g11140.1 0.589 0.979 0.39 0.003 0.993	1<br>Lperr03g02550.1 0.01 0.583 0.573 0.012 0.989<br>Lperr10g13340.1 0.589 0.987 0.398 0.004 0.996	1<br>AT2G17020.1 0.022 0.58 0.558 0.04 0.97<br>AT3G62760.1 0.592 0.983 0.391 0.014 1
Obart03g07660.1	1<br>Obart03g07660.1 0.001 1 0.999 0.001 1	0	0	0	0	0	0	0	0	0
	1<br>Obart08g08450.1 0.001 0.267 0.266 0.154 0.956<br>Obart09g05350.1 0.373 0.998 0.625 0.123 0.988	1<br>Osjap03g08130.1 0.298 1 0.702 0.015 1<br>Osjap10g07700.1 0.001 0.267 0.266 0.203 0.959	1<br>Osind03g08180.1 0.329 1 0.671 0.001 1<br>Osind10g05560.1 0.001 0.267 0.266 0.003 0.945	1<br>Orufi03g07710.1 0.329 1 0.671 0.001 1<br>Orufi03g07730.2 0.001 0.275 0.274 0.003 0.994	1<br>Oglab03g07740.1 0.298 1 0.702 0.015 1<br>Oglab03g07760.1 0.001 0.275 0.274 0.003 0.994	1<br>Olong_012932-R1 0.001 0.275 0.274 0.003 0.994<br>Olong_012934-R1 0.329 1 0.671 0.001 1	1<br>Opunc01g43280.1 0.379 0.998 0.619 0.226 0.944<br>Opunc03g07460.1 0.001 0.275 0.274 0.003 0.994	1<br>Obrac03g06230.1 0.329 1 0.671 0.001 1<br>Obrac10g03970.1 0.001 0.277 0.276 0.003 0.94	1<br>Lperr03g06980.1 0.329 1 0.671 0.001 1<br>Lperr10g04560.2 0.001 0.275 0.274 0.003 0.976	0<br>AT4G27190.1 0.347 0.997 0.65 0.122 0.982
Obart03g09160.3	1<br>Obart03g09160.1 0 1 1 0 1<br>Obart03g09160.2 0 1 1 0 1<br>Obart03g09160.3 0 1 1 0 1	1<br>Osjap03g09700.1 0 1 1 0.046 1<br>Osjap03g09700.2 0 1 1 0.045 1	1<br>Osind03g09660.1 0 1 1 0.044 1	1<br>Orufi03g09320.1 0 1 1 0.045 1<br>Orufi03g09320.2 0 1 1 0.046 1<br>Orufi03g09320.3 0 1 1 0.047 1<br>Orufi03g09320.4 0 1 1 0.048 1	1<br>Oglab03g09270.1 0 1 1 0.001 1<br>Oglab03g09270.2 0 1 1 0.001 1	1<br>Olong_002353-R1 0.021 1 0.979 0.001 1	1<br>Opunc03g08800.1 0.028 1 0.972 0.026 1<br>Opunc03g08800.2 0.028 1 0.972 0.026 1<br>Opunc03g08800.3 0.028 1 0.972 0.027 1	0	0	0
	1<br>Obart10g00240.1 0.578 0.988 0.41 0.179 0.96<br>Obart10g00250.1 0.069 0.469 0.4 0.072 1	1<br>Osjap05g08590.1 0.095 0.459 0.364 0.436 0.995<br>Osjap06g17700.1 0.55 0.988 0.438 0.321 0.983	1<br>Osind01g21860.1 0.069 0.469 0.4 0.075 1<br>Osind06g17200.1 0.55 0.988 0.438 0.318 0.983	1<br>Orufi10g00220.1 0.578 0.988 0.41 0.171 0.96<br>Orufi10g00230.1 0.069 0.469 0.4 0.074 1	1<br>Oglab10g00260.1 0.578 0.988 0.41 0.179 0.96<br>Oglab10g00270.1 0.069 0.469 0.4 0.072 1	1<br>Olong_008994-R1 0.069 0.469 0.4 0.07 0.999<br>Olong_023066-R1 0.581 0.995 0.414 0.021 0.97	0<br>Opunc03g08800.7 0.349 1 0.651 0.004 1	1<br>Obrac03g07350.1 0.548 1 0.452 0.114 1<br>Obrac10g00260.1 0.092 0.469 0.377 0.03 0.999	1<br>Lperr01g01500.1 0.082 0.471 0.389 0.001 1<br>Lperr03g08210.1 0.546 1 0.454 0.133 1	1<br>AT1G69770.1 0.089 0.459 0.37 0.027 0.988<br>AT4G34440.1 0.525 0.992 0.467 0.122 0.975

Obart03g10480.1	1<br>Obart03g10480.1 0.004 1 0.996 0.004 1	1<br>Osjap03g11290.1 0.004 1 0.996 0.004 1	0	0	0	0	0	0	0	0
	1<br>Obart01g05330.1 0.495 0.996 0.501 0.64 0.945<br>Obart03g10500.1 0.004 0.305 0.301 0.011 0.989	0<br>Osjap01g06090.1 0.495 0.996 0.501 0.64 0.945<br>Osjap03g11310.1 0.004 0.283 0.279 0.005 0.398	1<br>Osind03g11120.1 0.004 0.509 0.505 0.007 1<br>Osind03g11140.1 0.527 1 0.473 0.668 0.954	1<br>Orufi03g10700.1 0.004 0.509 0.505 0.007 1<br>Orufi03g10740.1 0.527 1 0.473 0.664 0.947	1<br>Oglab01g01190.1 0.534 0.996 0.462 0.656 0.959<br>Oglab03g10450.1 0.004 0.509 0.505 0.007 1	1<br>Olong_001134-R1 0.53 0.828 0.298 0.477 0.77<br>Olong_001982-R1 0.004 0.509 0.505 0.007 1	1<br>Opunc03g10110.1 0.004 0.509 0.505 0.007 1<br>Opunc03g10130.1 0.527 1 0.473 0.686 0.985	0<br>Obrac03g08430.5 0.527 1 0.473 0.284 0.412	0<br>Lperr03g09350.4 0.004 0.509 0.505 0.005 1	0
Obart03g12120.1	1<br>Obart03g12120.1 0.001 1 0.999 0.001 1	1<br>Osjap03g13100.1 0.028 1 0.972 0.001 1	1<br>Osind03g12930.1 0.001 1 0.999 0.001 1	0	1<br>Oglab03g12120.1 0.001 1 0.999 0.001 1	0	0	0	0	0
	1<br>Obart04g15560.1 0.64 0.998 0.358 0.146 0.877<br>Obart11g01060.1 0.114 0.633 0.519 0.471 0.982	1<br>Osjap04g16240.1 0.642 0.998 0.356 0.067 0.921<br>Osjap12g01140.1 0.109 0.633 0.524 0.498 0.981	1<br>Osind04g14790.1 0.642 0.998 0.356 0.013 0.911<br>Osind12g01290.1 0.109 0.633 0.524 0.49 0.981	1<br>Orufi06g01460.3 0.642 0.998 0.356 0.212 0.912<br>Orufi12g01130.1 0.109 0.633 0.524 0.498 0.981	1<br>Oglab06g01620.1 0.642 0.998 0.356 0.212 0.912<br>Oglab12g00800.1 0.114 0.633 0.519 0.483 0.983	1<br>Olong_007714-R9 0.642 1 0.358 0.002 1<br>Olong_007715-R1 0.125 0.633 0.508 0.007 0.965	1<br>Opunc03g11680.1 0.028 0.633 0.605 0.001 0.971<br>Opunc04g11270.1 0.642 0.998 0.356 0.067 0.921	1<br>Obrac03g09720.1 0.025 0.633 0.608 0.036 0.971<br>Obrac04g09430.1 0.64 0.998 0.358 0.014 0.863	1<br>Lperr03g10720.1 0.028 0.633 0.605 0.001 0.934<br>Lperr03g17470.1 0.633 0.998 0.365 0.119 0.828	1<br>AT3G46740.1 0.122 0.633 0.511 0.158 0.971<br>AT3G53360.1 0.672 0.998 0.326 0.09 0.934
Obart03g20630.1	1<br>Obart03g20630.1 0.001 1 0.999 0.001 1	1<br>Osjap03g22210.1 0.009 0.991 0.982 0.567 0.972	1<br>Osind03g22510.1 0.017 1 0.983 0.552 1	1<br>Orufi03g21250.1 0.185 0.995 0.81 0.02 0.991<br>Orufi03g21250.2 0.185 0.995 0.81 0.019 0.991<br>Orufi03g21250.3 0.189 0.995 0.806 0.021 0.99<br>Orufi03g21250.4 0.185 0.995 0.81 0.021 0.99	1<br>Oglab03g20370.1 0.073 1 0.927 0.087 1	0	0	0	0	0
	1<br>Obart06g03480.2 0.051 0.556 0.505 0.002 0.826<br>Obart09g16880.1 0.557 0.931 0.374 0.091 0.964	1<br>Osjap05g27870.1 0.044 0.547 0.503 0.075 0.982<br>Osjap12g12160.2 0.556 0.931 0.375 0.083 0.997	1<br>Osind02g20400.1 0.607 0.931 0.324 0.137 0.985<br>Osind05g28150.1 0.044 0.547 0.503 0.075 0.982	1<br>Orufi05g26490.1 0.044 0.553 0.509 0.075 0.989<br>Orufi09g01530.2 0.557 0.931 0.374 0.087 0.965	1<br>Oglab05g22780.1 0.044 0.547 0.503 0.077 0.982<br>Oglab08g00090.1 0.557 0.931 0.374 0.117 0.976	1<br>Olong_013421-R1 0.051 0.556 0.505 0.002 0.877<br>Olong_028288-R1 0.607 1 0.393 0.003 1	1<br>Opunc05g00870.2 0.567 0.996 0.429 0.145 0.973<br>Opunc05g22250.1 0.044 0.547 0.503 0.075 0.982	0<br>Obrac09g04370.1 0.08 0.572 0.492 0.034 0.858	0<br>Lperr05g06040.4 0.557 0.992 0.435 0.084 0.556	0<br>AT1G07740.1 0.025 0.531 0.506 0.083 0.967
Obart03g27980.1	1<br>Obart03g27980.1 0.001 1 0.999 0.001 1<br>Obart03g27980.2 0.015 1 0.985 0.015 1	1<br>Osjap03g30460.1 0.021 1 0.979 0.085 1	1<br>Osind03g31500.1 0.021 1 0.979 0.094 1	1<br>Orufi03g29110.1 0.021 1 0.979 0.341 1	0	0	0	0	0	0
	1<br>Obart01g35640.1 0.509 0.998 0.489 0.063 0.982<br>Obart12g19300.1 0.019 0.475 0.456 0.01 0.99	1<br>Osjap03g31370.3 0.509 0.998 0.489 0.042 0.887<br>Osjap12g24400.2 0.019 0.475 0.456 0.012 0.988	1<br>Osind04g00720.3 0.509 0.999 0.49 0.064 0.919<br>Osind12g20440.1 0.019 0.475 0.456 0.01 0.99	1<br>Orufi03g30600.1 0.509 0.999 0.49 0.05 0.885<br>Orufi12g21550.1 0.019 0.475 0.456 0.01 0.99	1<br>Oglab03g27330.1 0.51 1 0.49 0.043 1<br>Oglab03g27340.1 0.008 0.476 0.468 0.008 0.997	1<br>Olong_006906-R1 0.04 0.468 0.428 0.071 1<br>Olong_007449-R1 0.509 0.999 0.49 0.065 0.909	1<br>Opunc03g25490.1 0.021 0.486 0.465 0.525 0.8<br>Opunc03g26940.1 0.509 0.999 0.49 0.052 0.885	1<br>Obrac03g20710.1 0.021 0.476 0.455 0.028 0.997<br>Obrac03g21410.1 0.574 0.995 0.421 0.09 0.91	1<br>Lperr03g23190.1 0.51 0.999 0.489 0.039 0.863<br>Lperr03g23200.1 0.009 0.476 0.467 0.008 0.997	0<br>AT5G36940.1 0.019 0.478 0.459 0.003 0.998
Obart03g27980.2	1<br>Obart03g27980.1 0.015 1 0.985 0.015 1<br>Obart03g27980.2 0.001 1 0.999 0.001 1	1<br>Osjap03g30460.1 0.021 1 0.979 0.085 1	1<br>Osind03g31500.1 0.021 1 0.979 0.094 1	1<br>Orufi03g29110.1 0.021 1 0.979 0.341 1	0	0	0	0	0	0
	1<br>Obart01g35640.1 0.516 0.998 0.482 0.063 0.982<br>Obart12g19300.1 0.018 0.483 0.465 0.01 0.99	1<br>Osjap03g31370.3 0.516 0.998 0.482 0.042 0.887<br>Osjap12g24400.2 0.018 0.483 0.465 0.012 0.988	1<br>Osind04g00720.3 0.516 0.999 0.483 0.064 0.919<br>Osind12g20440.1 0.018 0.483 0.465 0.01 0.99	1<br>Orufi03g30600.1 0.516 0.999 0.483 0.05 0.885<br>Orufi12g21550.1 0.018 0.483 0.465 0.01 0.99	1<br>Oglab03g27330.1 0.517 1 0.483 0.043 1<br>Oglab12g17460.4 0.018 0.483 0.465 0.012 0.996	1<br>Olong_002926-R1 0.024 0.484 0.46 0.002 0.996<br>Olong_007449-R1 0.516 0.999 0.483 0.065 0.909	1<br>Opunc03g25490.2 0.021 0.487 0.466 0.543 0.811<br>Opunc03g26940.1 0.516 0.999 0.483 0.052 0.885	1<br>Obrac03g20710.1 0.021 0.484 0.463 0.028 0.997<br>Obrac03g21410.1 0.58 0.995 0.415 0.09 0.91	1<br>Lperr03g23190.1 0.517 0.999 0.482 0.039 0.895<br>Lperr03g23200.1 0.008 0.484 0.476 0.008 0.997	0<br>AT5G36940.1 0.019 0.486 0.467 0.003 0.998
Obart03g31750.1	1<br>Obart03g31750.1 0.001 1 0.999 0.001 1	1<br>Osjap03g34330.1 0.046 1 0.954 0.001 1	1<br>Osind03g35550.1 0.046 1 0.954 0.039 1	1<br>Orufi03g33060.1 0.046 1 0.954 0.001 1	1<br>Oglab03g30830.1 0.046 1 0.954 0.001 1	0	0	0	0	0
	1	1	1	1	1	1	1	1	1	0

	Obart03g36200.1 0.667 1 0.333 0.016 0.997<br>Obart10g04600.1 0.058 0.602 0.544 0.106 0.962	Osjap03g39110.1 0.667 1 0.333 0.036 1<br>Osjap10g05370.1 0.034 0.602 0.568 0.051 0.963	Osind01g46540.1 0.667 0.999 0.332 0.065 0.994<br>Osind10g03770.1 0.034 0.602 0.568 0.057 0.96	Orufi10g04850.1 0.034 0.602 0.568 0.054 0.963<br>Orufi11g12520.1 0.668 1 0.332 0.038 0.991	Oglab03g34460.1 0.646 0.997 0.351 0.016 0.993<br>Oglab10g04340.1 0.028 0.602 0.574 0.058 0.963	Olong_014796-R1 0.046 0.518 0.472 0.002 0.95<br>Olong_024872-R1 0.664 1 0.336 0.01 1	Opunc01g42030.1 0.667 1 0.333 0.28 0.872<br>Opunc03g28940.1 0.041 0.62 0.579 0.049 0.998	Obrac02g16280.1 0.662 0.982 0.32 0.063 0.949<br>Obrac03g23440.1 0.046 0.62 0.574 0.002 0.998	Lperr03g30520.1 0.66 1 0.34 0.003 0.997<br>Lperr10g03360.1 0.013 0.602 0.589 0.009 0.962	AT1G70730.3 0.046 0.618 0.572 0.118 0.995
Obart04g09560.1	1<br>Obart04g09560.1 0.001 1 0.999 0.001 1	0	0	0	0	0	0	0	0	0
	1<br>Obart02g17390.1 0.456 1 0.544 0.031 1<br>Obart03g27200.1 0.058 0.44 0.382 0.321 0.866	1<br>Osjap04g12100.1 0.456 1 0.544 0.047 1<br>Osjap06g32310.5 0.054 0.456 0.402 0.325 0.956	1<br>Osind03g31020.1 0.058 0.44 0.382 0.321 0.866<br>Osind04g10510.1 0.456 1 0.544 0.002 1	1<br>Orufi03g28280.1 0.058 0.44 0.382 0.321 0.866<br>Orufi04g10530.1 0.449 1 0.551 0.008 1	1<br>Oglab02g16770.1 0.456 1 0.544 0.031 1<br>Oglab04g07880.1 0.001 0.432 0.431 0.002 0.931	1<br>Olong_002589-R1 0.058 0.44 0.382 0.263 0.855<br>Olong_009593-R1 0.456 1 0.544 0.034 1	1<br>Opunc03g24840.1 0.058 0.44 0.382 0.321 0.869<br>Opunc04g07630.1 0.455 1 0.545 0.046 1	1<br>Obrac03g20160.2 0.058 0.449 0.391 0.318 0.911<br>Obrac04g05060.1 0.451 1 0.549 0.05 1	1<br>Lperr03g22530.1 0.058 0.44 0.382 0.325 0.867<br>Lperr04g06480.1 0.455 1 0.545 0.045 1	1<br>AT1G30490.1 0.059 0.446 0.387 0.316 0.866<br>AT3G25150.2 0.456 0.989 0.533 0.201 0.961
Obart04g26710.1	1<br>Obart04g26710.1 0.001 1 0.999 0.001 1<br>Obart04g26710.2 0.001 1 0.999 0.001 1	1<br>Osjap04g30120.1 0.001 1 0.999 0.001 1<br>Osjap04g30120.3 0.001 1 0.999 0.001 1<br>Osjap04g30120.4 0.001 1 0.999 0.001 1	0	1<br>Orufi04g27870.1 0.001 1 0.999 0.001 1<br>Orufi04g27870.2 0.001 1 0.999 0.001 1	0	1<br>Olong_014711-R2 0.001 0.849 0.848 0.001 0.977	0	1<br>Obrac04g18040.3 0.001 1 0.999 0.001 1	0	0
	1<br>Obart05g21760.1 0.657 0.996 0.339 0.024 0.993<br>Obart10g13990.1 0.007 0.632 0.625 0.016 0.827	1<br>Osjap04g16240.1 0.007 0.623 0.616 0.07 0.895<br>Osjap05g24420.1 0.657 0.996 0.339 0.024 0.993	1<br>Osind04g29430.2 0.636 1 0.364 0.003 1<br>Osind10g13390.1 0.007 0.632 0.625 0.023 0.826	1<br>Orufi05g23020.1 0.657 0.996 0.339 0.024 0.993<br>Orufi10g14810.1 0.007 0.632 0.625 0.016 0.827	1<br>Oglab06g14840.1 0.01 0.632 0.622 0.059 0.882<br>Oglab11g19190.1 0.648 1 0.352 0.042 1	0<br>Olong_014711-R1 0.001 0.645 0.644 0.002 0.837	1<br>Opunc04g23700.2 0.633 1 0.367 0.003 1<br>Opunc05g09290.1 0.003 0.63 0.627 0.038 0.861	0<br>Obrac04g07720.1 0.007 0.633 0.626 0.076 0.934	1<br>Lperr07g00250.2 0.665 1 0.335 0.015 0.99<br>Lperr10g10450.1 0.007 0.632 0.625 0.009 0.826	1<br>AT1G03540.1 0.007 0.623 0.616 0.066 0.995<br>AT4G11290.1 0.643 1 0.357 0.012 1
Obart05g00520.1	1<br>Obart05g00520.1 0.001 1 0.999 0.001 1<br>Obart05g00520.2 0.001 0.995 0.994 0.001 0.999	1<br>Osjap05g00530.1 0.001 0.993 0.992 0.084 0.999<br>Osjap05g00530.2 0.001 0.993 0.992 0.103 0.999<br>Osjap05g00530.3 0.001 0.993 0.992 0.104 0.999<br>Osjap05g00530.4 0.001 0.993 0.992 0.091 0.999	1<br>Osind05g00510.1 0.049 0.993 0.944 0.001 0.999	1<br>Orufi05g00500.1 0.001 0.993 0.992 0.084 0.999	0	1<br>Olong_022405-R1 0.049 0.993 0.944 0.001 0.999	0	0	0	0
	1<br>Obart06g01490.1 0.017 0.293 0.276 0.2 0.982<br>Obart09g15270.1 0.34 0.993 0.653 0.029 0.998	1<br>Osjap02g39750.1 0.337 0.988 0.651 0.004 0.956<br>Osjap07g27300.1 0.003 0.291 0.288 0.116 0.834	1<br>Osind09g15830.1 0.34 0.993 0.653 0.029 0.998<br>Osind10g10530.3 0.001 0.336 0.335 0.088 0.826	1<br>Orufi07g24760.1 0.003 0.291 0.288 0.116 0.834<br>Orufi09g16480.1 0.34 0.993 0.653 0.029 0.998	1<br>Oglab05g00500.1 0.001 0.294 0.293 0.218 0.982<br>Oglab09g13820.1 0.322 0.993 0.671 0.064 0.998	1<br>Olong_018361-R1 0.34 0.993 0.653 0.012 0.998<br>Olong_020432-R1 0.017 0.325 0.308 0.115 0.666	1<br>Opunc05g00480.1 0.329 0.993 0.664 0.007 0.998<br>Opunc05g00490.1 0.001 0.294 0.293 0.228 0.982	1<br>Obrac02g14170.4 0.001 0.3 0.299 0.03 0.843<br>Obrac02g28140.1 0.342 0.989 0.647 0.039 0.973	1<br>Lperr05g00500.1 0.331 0.993 0.662 0.024 0.994<br>Lperr10g08300.1 0.001 0.291 0.29 0.117 0.971	0<br>AT5G44230.1 0.001 0.298 0.297 0.155 0.872
Obart05g00520.2	1<br>Obart05g00520.1 0.001 0.999 0.998 0.001 0.995<br>Obart05g00520.2 0.001 1 0.999 0.001 1	1<br>Osjap05g00530.1 0.001 1 0.999 0.084 1<br>Osjap05g00530.2 0.001 1 0.999 0.103 1<br>Osjap05g00530.3 0.001 1 0.999 0.104 1<br>Osjap05g00530.4 0.001 1 0.999 0.091 1	1<br>Osind05g00510.1 0.049 1 0.951 0.001 1	1<br>Orufi05g00500.1 0.001 1 0.999 0.084 1	0	1<br>Olong_022405-R1 0.049 1 0.951 0.001 1	0	0	0	0
	1<br>Obart06g01490.1 0.017 0.295 0.278 0.2 0.982<br>Obart09g15270.1 0.342 1 0.658 0.029 1	1<br>Osjap02g39750.1 0.339 0.994 0.655 0.004 0.956<br>Osjap07g27300.1 0.003 0.293 0.29 0.116 0.834	1<br>Osind09g15830.1 0.342 1 0.658 0.029 1<br>Osind10g10530.1 0.001 0.293 0.292 0.108 0.858	1<br>Orufi07g24760.1 0.003 0.293 0.29 0.116 0.834<br>Orufi09g16480.1 0.342 1 0.658 0.029 1	1<br>Oglab05g00500.1 0.001 0.295 0.294 0.218 0.982<br>Oglab09g13820.1 0.324 1 0.676 0.064 1	1<br>Olong_018361-R1 0.342 1 0.658 0.012 1<br>Olong_020432-R1 0.017 0.327 0.31 0.115 0.666	1<br>Opunc05g00480.1 0.331 0.999 0.668 0.007 0.998<br>Opunc05g00490.1 0.001 0.295 0.294 0.228 0.982	1<br>Obrac02g14170.4 0.001 0.302 0.301 0.03 0.843<br>Obrac02g28140.1 0.344 0.995 0.651 0.039 0.973	1<br>Lperr05g00500.1 0.334 1 0.666 0.024 0.996<br>Lperr10g08300.1 0.001 0.293 0.292 0.117 0.971	0<br>AT5G44230.1 0.001 0.3 0.299 0.155 0.872
Obart05g03830.1	1<br>Obart05g03830.1 0.001 1 0.999 0.001 1<br>Obart05g03830.2 0.001 1 0.999 0.001 1	1<br>Osjap05g04120.1 0.001 1 0.999 0.001 1<br>Osjap05g04120.2 0.001 1 0.999 0.001 1	1<br>Osind05g04070.1 0.008 1 0.992 0.001 1<br>Osind05g04070.2 0.008 1 0.992 0.001 1	1<br>Orufi05g03950.1 0.001 1 0.999 0.001 1<br>Orufi05g03950.2 0.001 1 0.999 0.001 1	1<br>Oglab05g03490.1 0.001 1 0.999 0.001 1<br>Oglab05g03490.2 0.001 1 0.999 0.001 1	1<br>Olong_016739-R1 0.074 1 0.926 0.001 1	1<br>Opunc05g03540.1 0.001 1 0.999 0.001 1<br>Opunc05g03540.2 0.001 1 0.999 0.001 1	1<br>Obrac05g02930.1 0.074 1 0.926 0.001 1	0	0
	1<br>Obart01g23310.1 0.09 0.723 0.633 0.066 0.996	1<br>Osjap01g27410.1 0.09 0.723 0.633 0.066 0.996	1<br>Osind01g28630.1 0.09 0.723 0.633 0.066 0.996	1<br>Orufi01g26130.1 0.09 0.723 0.633 0.066 0.996	1<br>Oglab01g19490.1 0.09 0.723 0.633 0.066 0.996	1<br>Olong_003347-R2 0.09 0.723 0.633 0.047 0.996	1<br>Opunc01g23460.1 0.09 0.723 0.633 0.064 0.996	1<br>Obrac01g17590.1 0.09 0.723 0.633 0.067 0.996	0<br>Lperr05g03460.1 0.001 0.721 0.72 0.001 0.993	1<br>AT2G47580.1 0.775 1 0.225 0.008 1

	Obart03g14050.1 0.79 1 0.21 0.03 1	Osjap03g15140.5 0.743 1 0.257 0.061 1	Osind03g15230.1 0.79 1 0.21 0.03 1	Orufi03g14720.1 0.79 1 0.21 0.03 1	Oglab03g14060.1 0.79 1 0.21 0.03 1	Olong_009943-R1 0.79 1 0.21 0.03 1	Opunc03g13590.1 0.79 1 0.21 0.002 0.545	Obrac03g11330.1 0.79 1 0.21 0.03 1		AT3G16630.1 0.003 0.721 0.718 0.031 0.992
Obart05g03830.2	1<br>Obart05g03830.1 0.001 1 0.999 0.001 1<br>Obart05g03830.2 0.001 1 0.999 0.001 1	1<br>Osjap05g04120.1 0.001 1 0.999 0.001 1<br>Osjap05g04120.2 0.001 1 0.999 0.001 1	1<br>Osind05g04070.1 0.008 1 0.992 0.001 1<br>Osind05g04070.2 0.008 1 0.992 0.001 1	1<br>Orufi05g03950.1 0.001 1 0.999 0.001 1<br>Orufi05g03950.2 0.001 1 0.999 0.001 1	1<br>Oglab05g03490.1 0.001 1 0.999 0.001 1<br>Oglab05g03490.2 0.001 1 0.999 0.001 1	1<br>Olong_016739-R1 0.073 1 0.927 0.001 1	1<br>Opunc05g03540.1 0.001 1 0.999 0.001 1<br>Opunc05g03540.2 0.001 1 0.999 0.001 1	1<br>Obrac05g02930.1 0.073 1 0.927 0.001 1	0	0
	1<br>Obart01g23310.1 0.089 0.726 0.637 0.066 0.996<br>Obart03g14050.1 0.792 1 0.208 0.03 1	1<br>Osjap01g27410.1 0.089 0.726 0.637 0.066 0.996<br>Osjap03g15140.5 0.747 1 0.253 0.061 1	1<br>Osind01g28630.1 0.089 0.726 0.637 0.066 0.996<br>Osind03g15230.1 0.792 1 0.208 0.03 1	1<br>Orufi01g26130.1 0.089 0.726 0.637 0.066 0.996<br>Orufi03g14720.1 0.792 1 0.208 0.03 1	1<br>Oglab01g19490.1 0.089 0.726 0.637 0.066 0.996<br>Oglab03g14060.1 0.792 1 0.208 0.03 1	1<br>Olong_003347-R2 0.089 0.726 0.637 0.047 0.996<br>Olong_009943-R1 0.792 1 0.208 0.03 1	1<br>Opunc01g23460.1 0.089 0.726 0.637 0.064 0.996<br>Opunc03g13590.1 0.792 1 0.208 0.002 0.545	1<br>Obrac01g17590.1 0.089 0.726 0.637 0.067 0.996<br>Obrac03g11330.1 0.792 1 0.208 0.03 1	0<br>Lperr05g03460.1 0.001 0.725 0.724 0.001 0.993	1<br>AT2G47580.1 0.778 1 0.222 0.008 1<br>AT3G16630.1 0.003 0.725 0.722 0.031 0.992
Obart05g04470.1	1<br>Obart05g04470.1 0.002 1 0.998 0.002 1	0	0	0	1<br>Oglab05g04140.1 0.002 1 0.998 0.002 1	0	0	0	0	0
	1<br>Obart05g03110.1 0.362 0.977 0.615 0.395 0.582<br>Obart06g05940.1 0.014 0.35 0.336 0.048 0.458	1<br>Osjap05g04830.1 0.002 0.35 0.348 0.006 0.86<br>Osjap08g03110.1 0.359 0.993 0.634 0.031 0.944	0<br>Osind05g03400.1 0.274 0.945 0.671 0.792 0.975	1<br>Orufi05g04690.1 0.002 0.35 0.348 0.006 0.86<br>Orufi05g04700.1 0.35 1 0.65 0.212 1	1<br>Oglab05g02840.1 0.362 0.977 0.615 0.395 0.582<br>Oglab06g15820.1 0.014 0.359 0.345 0.033 0.973	1<br>Olong_002683-R1 0.359 0.993 0.634 0.031 0.945<br>Olong_015421-R1 0.002 0.35 0.348 0.01 1	1<br>Opunc05g03750.1 0.362 0.984 0.622 0.659 0.972<br>Opunc05g04100.1 0.002 0.35 0.348 0.006 0.878	0<br>Obrac05g03390.1 0.346 0.986 0.64 0.441 0.983	1<br>Lperr03g03510.1 0.023 0.35 0.327 0.073 0.593<br>Lperr05g03760.1 0.5 0.984 0.484 0.279 0.955	0<br>AT3G16720.1 0.002 0.35 0.348 0.059 0.523
Obart05g17330.1	1<br>Obart04g00290.1 0.003 1 0.997 0.084 0.835<br>Obart05g17330.1 0.003 1 0.997 0.003 1	0	0	0	0	0	0	0	0	0
	1<br>Obart01g38930.1 0.629 1 0.371 0.221 0.478<br>Obart07g15280.1 0.003 0.558 0.555 0.231 0.531	1<br>Osjap01g02610.1 0.003 0.613 0.61 0.138 0.36<br>Osjap05g19900.1 0.681 1 0.319 0.173 0.422	1<br>Osind01g45750.2 0.629 0.955 0.326 0.265 0.54<br>Osind07g15230.1 0.003 0.565 0.562 0.101 0.359	1<br>Orufi03g06810.1 0.003 0.635 0.632 0.254 0.985<br>Orufi05g18690.1 0.681 1 0.319 0.19 0.434	1<br>Oglab05g16420.1 0.681 1 0.319 0.186 0.432<br>Oglab12g04370.1 0.003 0.613 0.61 0.242 0.582	1<br>Olong_017674-R1 0.674 1 0.326 0.128 0.394<br>Olong_018765-R1 0.006 0.613 0.607 0.066 0.849	1<br>Opunc02g12800.1 0.006 0.6 0.594 0.212 0.976<br>Opunc07g08640.2 0.674 1 0.326 0.196 0.424	0<br>Obrac05g11690.1 0.681 1 0.319 0.199 0.437	0<br>Lperr01g19800.1 0.003 0.29 0.287 0.354 0.818<br>Lperr05g13860.1 0.677 1 0.323 0.279 0.498	0<br>AT1G72040.1 0.706 1 0.294 0.378 0.559
Obart05g20080.1	1<br>Obart05g20080.1 0.001 1 0.999 0.001 1	0	0	0	0	0	0	0	0	0
	1<br>Obart07g26300.1 0.026 0.661 0.635 0.033 0.967<br>Obart09g11580.1 0.666 0.994 0.328 0.208 0.959	1<br>Osjap05g22670.1 0.001 0.664 0.663 0.063 0.939<br>Osjap09g11800.1 0.666 0.994 0.328 0.217 0.958	1<br>Osind07g26930.1 0.026 0.661 0.635 0.033 0.967<br>Osind09g11940.1 0.666 0.994 0.328 0.258 0.969	1<br>Orufi05g21340.1 0.001 0.664 0.663 0.002 0.935<br>Orufi09g12430.2 0.666 0.994 0.328 0.217 0.958	1<br>Oglab05g19000.1 0.001 0.664 0.663 0.066 0.939<br>Oglab09g10550.1 0.666 0.994 0.328 0.217 0.958	1<br>Olong_005541-R1 0.03 0.624 0.594 0.039 0.917<br>Olong_025141-R1 0.659 0.996 0.337 0.279 1	1<br>Opunc05g17810.1 0.009 0.664 0.655 0.075 0.945<br>Opunc09g10120.1 0.666 0.994 0.328 0.399 0.973	1<br>Obrac07g17500.1 0.013 0.66 0.647 0.034 0.94<br>Obrac09g06450.1 0.666 0.994 0.328 0.182 0.942	1<br>Lperr05g16000.1 0.023 0.664 0.641 0.022 0.942<br>Lperr09g08210.1 0.666 0.994 0.328 0.253 0.964	1<br>AT2G26830.1 0.666 0.994 0.328 0.278 0.971<br>AT2G40480.1 0.016 0.661 0.645 0.081 0.969
Obart05g25070.1	1<br>Obart05g25070.1 0.001 1 0.999 0.001 1	1<br>Osjap05g28230.1 0.001 1 0.999 0.001 1	1<br>Osind05g28520.1 0.001 1 0.999 0.001 1	1<br>Orufi05g26870.1 0.001 1 0.999 0.001 1	1<br>Oglab05g23160.1 0.001 1 0.999 0.001 1	0	1<br>Opunc05g22640.1 0.001 1 0.999 0.001 1	0	1<br>Lperr01g22990.1 0.001 1 0.999 0.027 1	0
	1<br>Obart01g26870.1 0.006 0.451 0.445 0.117 0.995<br>Obart01g26880.1 0.462 0.999 0.537 0.009 0.999	1<br>Osjap01g31140.1 0.007 0.451 0.444 0.093 0.995<br>Osjap01g31150.1 0.462 0.999 0.537 0.009 0.999	1<br>Osind01g32600.1 0.001 0.451 0.45 0.002 0.995<br>Osind01g32610.1 0.462 0.999 0.537 0.009 0.999	1<br>Orufi01g29880.1 0.001 0.451 0.45 0.075 0.995<br>Orufi01g29890.1 0.462 0.999 0.537 0.009 0.999	1<br>Oglab01g22740.1 0.028 0.451 0.423 0.061 0.995<br>Oglab01g22750.1 0.462 0.999 0.537 0.009 0.999	1<br>Olong_000367-R1 0.105 0.451 0.346 0.002 0.993<br>Olong_000368-R1 0.456 0.999 0.543 0.108 0.999	1<br>Opunc01g26820.1 0.049 0.451 0.402 0.002 0.994<br>Opunc01g26830.1 0.462 1 0.538 0.009 1	1<br>Obrac01g20400.1 0.046 0.451 0.405 0.004 0.994<br>Obrac05g17560.2 0.451 1 0.549 0.164 1	1<br>Lperr05g20850.2 0.467 1 0.533 0.002 1<br>Lperr05g20860.1 0.001 0.451 0.45 0.002 0.995	1<br>AT5G08690.1 0.012 0.451 0.439 0.034 0.995<br>AT5G51600.1 0.462 0.999 0.537 0.008 0.996
Obart05g27940.1	1<br>Obart05g27940.1 0.001 1 0.999 0.001 1	0	1<br>Osind05g31580.1 0.001 1 0.999 0.024 1	0	0	1<br>Olong_007981-R1 0.059 1 0.941 0.001 1	0	0	0	0
	1<br>Obart01g20890.1 0.589 0.98 0.391 0.063 0.943<br>Obart07g06670.1 0.158 0.582 0.424 0.36 0.978	1<br>Osjap01g24630.2 0.589 0.98 0.391 0.156 0.951<br>Osjap05g31230.3 0.001 0.581 0.58 0.041 0.982	1<br>Osind01g25470.1 0.002 0.536 0.534 0.073 0.967<br>Osind01g25480.1 0.589 0.954 0.365 0.063 0.877	1<br>Orufi01g23560.1 0.589 0.98 0.391 0.144 0.949<br>Orufi05g29910.3 0.001 0.581 0.58 0.036 0.982	1<br>Oglab05g25920.1 0.001 0.581 0.58 0.035 0.982<br>Oglab05g25930.1 0.589 1 0.411 0.07 1	1<br>Olong_011279-R1 0.589 0.991 0.402 0.063 0.978<br>Olong_011281-R2 0.002 0.565 0.563 0.019 1	1<br>Opunc05g25540.1 0.001 0.581 0.58 0.037 0.983<br>Opunc05g25550.1 0.589 1 0.411 0.067 1	0<br>Obrac05g19820.2 0.001 0.581 0.58 0.002 0.982	1<br>Lperr01g17820.1 0.002 0.582 0.58 0.023 0.973<br>Lperr01g17840.1 0.589 0.998 0.409 0.166 0.978	1<br>AT1G07990.1 0.589 0.917 0.328 0.061 0.782<br>AT4G05420.1 0.001 0.581 0.58 0.002 0.982

Obart06g00880.1	1<br>Obart06g00880.1 0.001 1 0.999 0.001 1	1<br>Osjap06g00940.1 0.04 1 0.96 0.085 1	0	1<br>Orufi06g00860.1 0.067 1 0.933 0.021 1	1<br>Oglab06g00840.1 0.001 1 0.999 0.001 1	0	0	0	0	0
	1<br>Obart06g00690.1 0.529 1 0.471 0.099 1<br>Obart09g03180.1 0.021 0.458 0.437 0.145 1	1<br>Osjap06g00950.1 0.529 0.992 0.463 0.132 0.99<br>Osjap08g13790.1 0.051 0.458 0.407 0.005 1	1<br>Osind06g00910.1 0.526 1 0.474 0.068 1<br>Osind08g15520.1 0.051 0.458 0.407 0.005 1	1<br>Orufi06g00690.1 0.529 1 0.471 0.099 1<br>Orufi06g00860.2 0.001 0.445 0.444 0.005 0.98	1<br>Oglab06g00730.1 0.526 0.99 0.464 0.257 0.968<br>Oglab07g19660.1 0.033 0.458 0.425 0.142 0.938	0<br>Olong_023831-R1 0.053 0.583 0.53 0.001 0.373	1<br>Opunc06g00800.1 0.457 1 0.543 0.025 1<br>Opunc06g00830.1 0.05 0.457 0.407 0.006 1	1<br>Obrac06g00660.1 0.526 1 0.474 0.034 0.999<br>Obrac07g14790.1 0.058 0.503 0.445 0.093 0.948	1<br>Lperr01g40030.1 0.055 0.457 0.402 0.013 0.994<br>Lperr06g00740.1 0.531 0.999 0.468 0.078 0.997	0<br>AT4G34470.1 0.063 0.457 0.394 0.013 1
Obart06g03390.1	1<br>Obart06g03390.1 0.001 1 0.999 0.001 1	0	0	0	0	0	0	0	0	0
	1<br>Obart01g10160.1 0.421 0.979 0.558 0.025 0.943<br>Obart06g25350.1 0.019 0.331 0.312 0.24 0.91	1<br>Osjap06g03820.1 0.411 1 0.589 0.002 0.797<br>Osjap06g03830.1 0.001 0.322 0.321 0.003 0.924	1<br>Osind06g04060.1 0.412 1 0.588 0.005 0.871<br>Osind06g04070.1 0.001 0.322 0.321 0.004 0.923	1<br>Orufi06g03390.1 0.411 1 0.589 0.002 1<br>Orufi06g03400.1 0.001 0.322 0.321 0.003 0.924	1<br>Oglab06g03530.1 0.411 1 0.589 0.002 1<br>Oglab06g03540.1 0.001 0.322 0.321 0.004 0.923	1<br>Olong_004095-R1 0.421 0.974 0.553 0.015 0.951<br>Olong_008568-R1 0.001 0.316 0.315 0.004 0.888	1<br>Opunc06g03190.1 0.419 1 0.581 0.004 1<br>Opunc06g03210.1 0.001 0.319 0.318 0.345 0.985	1<br>Obrac06g02440.1 0.419 1 0.581 0.004 1<br>Obrac06g02460.1 0.001 0.389 0.388 0.499 0.972	1<br>Lperr06g03190.1 0.415 1 0.585 0.004 1<br>Lperr06g03200.1 0.001 0.315 0.314 0.083 0.862	1<br>AT3G13700.1 0.014 0.347 0.333 0.02 0.99<br>AT5G16410.1 0.423 0.979 0.556 0.069 1
Obart06g20680.1	1<br>Obart06g20680.1 0.001 1 0.999 0.001 1	0	1<br>Osind06g23650.1 0.001 1 0.999 0.001 1	1<br>Orufi06g22110.1 0.001 1 0.999 0.011 1	0	0	0	0	0	0
	1<br>Obart01g27850.1 0.111 0.61 0.499 0.006 0.954<br>Obart05g14650.1 0.625 1 0.375 0.094 1	1<br>Osjap02g34710.2 0.637 0.998 0.361 0.12 0.997<br>Osjap06g24310.1 0.001 0.623 0.622 0.059 0.986	1<br>Osind05g17380.1 0.625 1 0.375 0.094 1<br>Osind09g14580.1 0.026 0.623 0.597 0.005 0.975	1<br>Orufi05g15730.1 0.625 1 0.375 0.094 1<br>Orufi09g15100.1 0.012 0.623 0.611 0.029 0.975	1<br>Oglab05g13700.1 0.625 1 0.375 0.094 1<br>Oglab06g18700.1 0.001 0.623 0.622 0.016 0.986	1<br>Olong_017051-R1 0.625 1 0.375 0.104 1<br>Olong_021346-R1 0.001 0.623 0.622 0.01 0.982	1<br>Opunc03g03090.1 0.016 0.639 0.623 0.028 1<br>Opunc05g12780.1 0.641 1 0.359 0.139 1	1<br>Obrac02g22310.1 0.628 0.998 0.37 0.012 0.997<br>Obrac09g08370.1 0.021 0.618 0.597 0.023 0.961	1<br>Lperr02g25000.1 0.628 0.998 0.37 0.012 0.997<br>Lperr07g09420.1 0.029 0.627 0.598 0.034 0.995	1<br>AT3G07940.1 0.637 1 0.363 0.094 1<br>AT5G58830.1 0.008 0.623 0.615 0.019 0.984
Obart06g25430.1	1<br>Obart06g25430.1 0.004 1 0.996 0.004 1	0	0	0	1<br>Oglab06g22820.1 0.004 1 0.996 0.005 1	0	0	0	0	0
	1<br>Obart06g25600.1 0.059 0.447 0.388 0.721 0.989<br>Obart09g13660.1 0.447 0.984 0.537 0.022 0.993	1<br>Osjap06g26550.1 0.004 0.368 0.364 0.698 0.988<br>Osjap09g14010.1 0.447 0.984 0.537 0.022 0.993	1<br>Osind06g29050.1 0.012 0.419 0.407 0.655 0.91<br>Osind09g14180.1 0.455 0.992 0.537 0.013 0.886	1<br>Orufi06g27530.1 0.059 0.447 0.388 0.721 0.989<br>Orufi09g14650.1 0.447 0.984 0.537 0.186 0.994	1<br>Oglab06g20540.1 0.004 0.419 0.415 0.625 0.915<br>Oglab09g12320.1 0.447 0.984 0.537 0.186 0.994	0<br>Olong_024432-R1 0.474 0.984 0.51 0.174 0.994	1<br>Opunc06g23340.1 0.004 0.289 0.285 0.367 0.975<br>Opunc09g12070.1 0.455 0.988 0.533 0.014 0.916	0<br>Obrac09g08010.1 0.443 0.984 0.541 0.008 0.992	0<br>Lperr09g09890.1 0.348 0.984 0.636 0.018 0.994	0<br>AT4G30380.1 0.494 0.976 0.482 0.033 0.984
Obart06g28060.1	1<br>Obart06g28060.1 0.001 1 0.999 0.001 1<br>Obart09g17460.1 0.024 1 0.976 0.076 0.969<br>Obart09g17460.2 0.024 1 0.976 0.076 0.969	1<br>Osjap09g17800.1 0.024 1 0.976 0.113 0.974<br>Osjap09g17800.2 0.024 1 0.976 0.114 0.974<br>Osjap09g17800.3 0.024 0.997 0.973 0.187 0.947	1<br>Osind06g31620.1 0.001 1 0.999 0.001 1	1<br>Orufi06g29990.1 0.001 1 0.999 0.001 1<br>Orufi09g18790.1 0.024 1 0.976 0.107 0.97<br>Orufi09g18790.2 0.024 1 0.976 0.107 0.97<br>Orufi09g18790.3 0.024 1 0.976 0.108 0.969<br>Orufi09g18790.4 0.024 1 0.976 0.108 0.969<br>Orufi09g18790.5 0.024 0.976 0.952 0.17 0.937<br>Orufi09g18790.6 0.024 0.996 0.972 0.172 0.937	1<br>Oglab06g25490.1 0.001 1 0.999 0.001 1<br>Oglab09g15660.1 0.024 0.999 0.975 0.154 0.95	1<br>Olong_016700-R1 0.001 1 0.999 0.001 1	0	0	1<br>Lperr09g13380.1 0.084 0.982 0.898 0.166 0.951	0
	1<br>Obart04g21770.1 0.07 0.417 0.347 0.232 0.933<br>Obart10g07310.1 0.427 1 0.573 0.099 1	1<br>Osjap06g32830.1 0.427 1 0.573 0.085 1<br>Osjap06g32840.1 0.001 0.407 0.406 0.002 0.952	1<br>Osind04g24640.1 0.07 0.417 0.347 0.233 0.933<br>Osind06g30840.1 0.485 0.999 0.514 0.109 1	0<br>Orufi06g29150.4 0.485 0.999 0.514 0.122 1	1<br>Oglab02g01250.1 0.066 0.406 0.34 0.221 0.93<br>Oglab10g06880.1 0.427 1 0.573 0.099 1	1<br>Olong_000286-R1 0.066 0.406 0.34 0.258 0.928<br>Olong_026973-R2 0.427 1 0.573 0.099 1	1<br>Opunc06g25610.1 0.001 0.407 0.406 0.002 0.951<br>Opunc10g06480.1 0.427 0.998 0.571 0.098 0.984	1<br>Obrac06g20010.2 0.432 1 0.568 0.119 1<br>Obrac06g20020.1 0.001 0.407 0.406 0.002 0.952	1<br>Lperr02g22230.1 0.043 0.392 0.349 0.113 0.88<br>Lperr06g23510.1 0.402 1 0.598 0.074 1	1<br>AT1G76360.1 0.036 0.406 0.37 0.219 0.952<br>AT3G61130.1 0.465 0.999 0.534 0.207 1
Obart07g03940.1	1<br>Obart07g03940.1 0.001 1 0.999 0.001 1	0	0	0	0	0	0	0	0	0

	1<br>Obart03g36870.1 0.038 0.393 0.355 0.002 0.889<br>Obart09g12990.1 0.441 0.99 0.549 0.054 0.997	1<br>Osjap01g04010.1 0.435 0.989 0.554 0.002 1<br>Osjap03g39900.1 0.038 0.429 0.391 0.002 1	1<br>Osind01g04300.1 0.441 0.998 0.557 0.001 0.994<br>Osind03g40960.1 0.038 0.429 0.391 0.002 1	1<br>Orufi03g38420.1 0.038 0.429 0.391 0.002 1<br>Orufi09g13930.1 0.441 0.99 0.549 0.079 1	1<br>Oglab01g03190.1 0.435 0.989 0.554 0.002 0.994<br>Oglab03g35120.1 0.038 0.429 0.391 0.002 1	1<br>Olong_012584-R1 0.463 0.991 0.528 0.017 0.998<br>Olong_020874-R1 0.038 0.339 0.301 0.004 1	1<br>Opunc01g03200.2 0.434 0.992 0.558 0.043 0.939<br>Opunc03g33860.1 0.044 0.395 0.351 0.012 0.894	0<br>Obrac09g07500.1 0.442 0.986 0.544 0.102 0.963	1<br>Lperr03g32440.1 0.074 0.414 0.34 0.073 0.966<br>Lperr07g03510.1 0.42 0.989 0.569 0.011 0.992	0<br>AT5G06570.1 0.45 0.989 0.539 0.003 1
Obart07g19760.1	1<br>Obart07g19760.1 0.002 1 0.998 0.002 1	0	0	0	0	0	1<br>Opunc07g18680.1 0.002 1 0.998 0.125 1<br>Opunc07g18680.2 0.002 1 0.998 0.12 1<br>Opunc07g18700.1 0.002 1 0.998 0.138 1<br>Opunc07g18700.2 0.002 1 0.998 0.115 1	0	1<br>Lperr07g17150.1 0.002 1 0.998 0.111 1	0
	1<br>Obart07g04680.1 0.337 0.998 0.661 0.003 0.994<br>Obart09g04230.1 0.002 0.265 0.263 0.074 0.973	1<br>Osjap07g05300.1 0.319 0.998 0.679 0.079 0.994<br>Osjap09g04300.1 0.002 0.265 0.263 0.074 0.973	1<br>Osind07g19860.1 0.002 0.269 0.267 0.402 0.996<br>Osind07g19870.1 0.337 1 0.663 0.003 1	1<br>Orufi07g04510.1 0.319 0.998 0.679 0.079 0.994<br>Orufi09g04740.1 0.002 0.265 0.263 0.074 0.973	1<br>Oglab07g04490.1 0.331 0.998 0.667 0.117 0.993<br>Oglab09g04130.1 0.002 0.265 0.263 0.074 0.973	1<br>Olong_011782-R1 0.002 0.265 0.263 0.074 0.973<br>Olong_015820-R2 0.337 1 0.663 0.003 1	1<br>Opunc07g04580.1 0.337 0.998 0.661 0.003 0.994<br>Opunc09g03700.1 0.002 0.265 0.263 0.074 0.973	1<br>Obrac07g12480.1 0.39 1 0.61 0.003 1<br>Obrac09g02110.1 0.002 0.265 0.263 0.074 0.973	0<br>Lperr09g02940.1 0.002 0.265 0.263 0.074 0.973	0<br>AT3G08690.1 0.012 0.273 0.261 0.128 1
Obart07g23980.1	1<br>Obart07g23980.1 0.003 1 0.997 0.003 1	0	0	0	0	0	0	0	0	0
	1<br>Obart03g38900.1 0.003 0.276 0.273 0.322 0.644<br>Obart09g15250.1 0.295 1 0.705 0.284 1	1<br>Osjap03g42080.1 0.003 0.292 0.289 0.432 0.749<br>Osjap09g15670.1 0.295 1 0.705 0.334 1	1<br>Osind09g15810.1 0.295 1 0.705 0.34 1<br>Osind11g00110.1 0.003 0.287 0.284 0.555 0.776	1<br>Orufi03g40560.1 0.003 0.276 0.273 0.492 0.733<br>Orufi09g16460.1 0.295 1 0.705 0.339 1	1<br>Oglab03g36900.1 0.003 0.276 0.273 0.59 0.785<br>Oglab09g13800.1 0.295 1 0.705 0.34 1	0<br>Olong_003491-R1 0.003 0.357 0.354 0.375 0.732	1<br>Opunc05g11140.1 0.003 0.276 0.273 0.544 0.745<br>Opunc07g22640.1 0.295 1 0.705 0.325 1	1<br>Obrac03g29030.2 0.003 0.276 0.273 0.463 0.718<br>Obrac09g09270.1 0.279 0.981 0.702 0.174 0.993	0<br>Lperr01g12120.1 0.239 0.979 0.74 0.278 0.995	1<br>AT2G24580.1 0.292 0.987 0.695 0.325 0.969<br>AT2G40320.1 0.003 0.282 0.279 0.485 0.734
Obart07g27040.1	1<br>Obart07g27040.1 0.002 1 0.998 0.002 1<br>Obart07g27040.2 0.002 1 0.998 0.002 1	0	0	0	0	0	0	0	0	0
	1<br>Obart07g27030.1 0.002 0.249 0.247 0.348 0.51<br>Obart08g16640.1 0.4 0.974 0.574 0.035 0.802	1<br>Osjap07g30980.1 0.002 0.244 0.242 0.003 0.358<br>Osjap07g31000.1 0.318 0.974 0.656 0.002 0.608	1<br>Osind07g27700.1 0.002 0.249 0.247 0.002 0.258<br>Osind07g27720.1 0.318 0.974 0.656 0.012 0.493	0<br>Orufi03g37620.1 0.237 0.884 0.647 0.006 0.997	1<br>Oglab07g23640.2 0.002 0.324 0.322 0.813 0.998<br>Oglab08g15450.1 0.4 0.974 0.574 0.067 0.483	1<br>Olong_008699-R1 0.315 0.974 0.659 0.003 0.523<br>Olong_008701-R1 0.002 0.249 0.247 0.002 0.258	1<br>Opunc07g25410.1 0.002 0.249 0.247 0.002 0.268<br>Opunc08g14550.1 0.4 0.974 0.574 0.08 0.527	1<br>Obrac07g18100.1 0.002 0.249 0.247 0.004 0.271<br>Obrac08g10910.1 0.4 0.974 0.574 0.075 0.529	0<br>Lperr07g23810.2 0.322 0.974 0.652 0.009 0.348	0<br>AT3G66658.2 0.002 0.268 0.266 0.002 0.273
Obart07g27040.2	1<br>Obart07g27040.1 0.002 1 0.998 0.002 1<br>Obart07g27040.2 0.002 1 0.998 0.002 1	0	0	0	0	0	0	0	0	0
	1<br>Obart07g27030.1 0.002 0.226 0.224 0.348 0.51<br>Obart08g16640.1 0.381 0.973 0.592 0.035 0.802	1<br>Osjap07g30980.1 0.002 0.221 0.219 0.003 0.358<br>Osjap07g31000.1 0.298 0.973 0.675 0.002 0.608	1<br>Osind07g27700.1 0.002 0.226 0.224 0.002 0.258<br>Osind07g27720.1 0.298 0.973 0.675 0.012 0.493	1<br>Orufi03g37620.1 0.258 0.881 0.623 0.006 0.998<br>Orufi07g27980.2 0.002 0.226 0.224 0.019 0.253	1<br>Oglab07g23640.2 0.002 0.303 0.301 0.813 0.998<br>Oglab08g15450.1 0.381 0.973 0.592 0.067 0.483	1<br>Olong_008699-R1 0.294 0.973 0.679 0.003 0.523<br>Olong_008701-R1 0.002 0.226 0.224 0.002 0.258	1<br>Opunc07g25410.1 0.002 0.226 0.224 0.002 0.268<br>Opunc08g14550.1 0.381 0.973 0.592 0.08 0.527	1<br>Obrac07g18100.1 0.002 0.226 0.224 0.004 0.271<br>Obrac08g10910.1 0.381 0.973 0.592 0.075 0.529	0<br>Lperr07g23810.2 0.301 0.973 0.672 0.009 0.348	0<br>AT3G66658.2 0.002 0.246 0.244 0.002 0.273
Obart07g27300.1	1<br>Obart07g27300.1 0.001 1 0.999 0.001 1	0	0	0	0	0	0	0	0	0
	1<br>Obart03g13590.1 0.028 0.527 0.499 0.001 0.952<br>Obart05g12700.1 0.592 1 0.408 0.017 1	1<br>Osjap07g31290.1 0.001 0.524 0.523 0.033 0.951<br>Osjap07g31300.1 0.555 1 0.445 0.007 1	1<br>Osind03g14630.1 0.028 0.527 0.499 0.001 0.952<br>Osind07g28000.1 0.555 1 0.445 0.046 1	1<br>Orufi03g14250.1 0.028 0.527 0.499 0.001 0.952<br>Orufi07g28280.2 0.555 1 0.445 0.046 1	1<br>Oglab07g23910.1 0.002 0.524 0.522 0.008 0.948<br>Oglab07g23920.1 0.527 1 0.473 0.009 1	1<br>Olong_003210-R1 0.002 0.524 0.522 0.015 0.943<br>Olong_003211-R1 0.535 1 0.465 0.026 1	1<br>Opunc03g13100.1 0.028 0.527 0.499 0.001 0.948<br>Opunc07g25690.1 0.555 0.999 0.444 0.007 0.983	1<br>Obrac07g18370.1 0.013 0.524 0.511 0.064 0.951<br>Obrac07g18380.4 0.555 1 0.445 0.007 0.994	1<br>Lperr05g10220.2 0.555 1 0.445 0.014 1<br>Lperr07g24070.2 0.028 0.524 0.496 0.001 0.948	1<br>AT2G38280.1 0.556 0.999 0.443 0.01 0.998<br>AT3G08960.1 0.028 0.527 0.499 0.001 0.952

Obart08g05380.1	1<br>Obart08g05380.1 0.004 1 0.996 0.004 1	0	0	0	0	0	0	0	0	0
	1<br>Obart07g07210.1 0.353 1 0.647 0.038 0.703<br>Obart08g05390.1 0.004 0.298 0.294 0.012 1	1<br>Osjap08g05840.2 0.353 1 0.647 0.065 0.75<br>Osjap08g05860.1 0.004 0.305 0.301 0.012 1	1<br>Osind07g07530.1 0.353 1 0.647 0.038 0.703<br>Osind08g05840.1 0.004 0.305 0.301 0.012 1	1<br>Orufi07g07230.1 0.353 1 0.647 0.037 0.706<br>Orufi08g05980.1 0.004 0.305 0.301 0.012 1	1<br>Oglab08g04820.1 0.331 1 0.669 0.203 1<br>Oglab08g04830.1 0.004 0.298 0.294 0.012 1	1<br>Olong_004294-R1 0.004 0.305 0.301 0.012 1<br>Olong_026698-R1 0.353 1 0.647 0.037 0.706	1<br>Opunc07g06940.1 0.356 1 0.644 0.004 0.695<br>Opunc08g05230.1 0.036 0.305 0.269 0.013 1	1<br>Obrac07g04400.3 0.345 1 0.655 0.015 0.7<br>Obrac08g04320.1 0.004 0.305 0.301 0.012 1	1<br>Lperr07g06270.1 0.356 1 0.644 0.004 0.68<br>Lperr08g05140.1 0.036 0.305 0.269 0.013 1	0<br>AT4G33690.1 0.356 0.924 0.568 0.004 0.691
Obart08g06070.1	1<br>Obart08g06070.1 0.002 1 0.998 0.002 1	1<br>Osjap08g06650.1 0.002 1 0.998 0.002 1	1<br>Osind08g06790.1 0.002 1 0.998 0.002 1	1<br>Orufi08g06840.1 0.002 1 0.998 0.002 1	1<br>Oglab08g05640.1 0.002 1 0.998 0.002 1	0	1<br>Opunc08g05850.1 0.047 1 0.953 0.068 1	0	0	0
	1<br>Obart02g28440.1 0.348 0.996 0.648 0.093 0.994<br>Obart04g17770.1 0.047 0.275 0.228 0.198 0.94	1<br>Osjap04g21220.1 0.047 0.275 0.228 0.21 0.936<br>Osjap09g20870.1 0.359 0.984 0.625 0.208 0.966	1<br>Osind04g20250.1 0.047 0.275 0.228 0.198 0.94<br>Osind09g20960.1 0.359 0.984 0.625 0.208 0.966	1<br>Orufi04g19250.2 0.047 0.275 0.228 0.21 0.936<br>Orufi09g21960.1 0.359 0.984 0.625 0.2 0.965	1<br>Oglab02g26360.1 0.348 0.996 0.648 0.01 0.994<br>Oglab04g16180.1 0.047 0.275 0.228 0.198 0.94	1<br>Olong_008030-R1 0.047 0.275 0.228 0.232 0.937<br>Olong_008031-R2 0.277 1 0.723 0.106 1	1<br>Opunc04g15550.1 0.047 0.275 0.228 0.206 0.938<br>Opunc04g18740.1 0.295 0.984 0.689 0.088 0.994	1<br>Obrac08g04780.1 0.277 1 0.723 0.098 1<br>Obrac08g04800.1 0.002 0.275 0.273 0.006 0.943	1<br>Lperr08g05690.1 0.277 1 0.723 0.1 1<br>Lperr08g05700.1 0.002 0.275 0.273 0.006 0.943	1<br>AT1G05310.1 0.33 0.982 0.652 0.183 0.99<br>AT4G30800.1 0.047 0.275 0.228 0.208 0.943
Obart08g11880.1	1<br>Obart08g11880.1 0.009 1 0.991 0.009 1<br>Obart10g13080.1 0.017 0.966 0.949 0.213 0.999	0	0	0	0	0	0	0	0	0
	1<br>Obart02g24490.1 0.333 1 0.667 0.82 1<br>Obart08g11890.1 0.009 0.333 0.324 0.523 0.701	1<br>Osjap02g27820.1 0.333 1 0.667 0.899 1<br>Osjap08g12720.1 0.009 0.333 0.324 0.01 0.379	1<br>Osind02g27730.1 0.333 1 0.667 0.899 1<br>Osind08g14210.1 0.009 0.333 0.324 0.536 0.709	1<br>Orufi02g25850.1 0.333 1 0.667 0.899 1<br>Orufi08g13360.1 0.009 0.333 0.324 0.349 0.792	1<br>Oglab02g23150.2 0.333 1 0.667 0.901 1<br>Oglab08g11080.1 0.009 0.333 0.324 0.523 0.701	1<br>Olong_025435-R1 0.342 0.966 0.624 0.075 0.988<br>Olong_027114-R1 0.009 0.333 0.324 0.007 0.277	1<br>Opunc08g10810.1 0.009 0.333 0.324 0.01 0.386<br>Opunc10g11490.1 0.333 0.966 0.633 0.867 0.998	1<br>Obrac02g17520.1 0.333 1 0.667 0.868 0.994<br>Obrac08g08040.1 0.009 0.316 0.307 0.012 0.435	1<br>Lperr02g20030.1 0.333 0.983 0.65 0.899 0.997<br>Lperr08g09830.2 0.009 0.333 0.324 0.011 0.437	0<br>AT3G23410.1 0.248 0.966 0.718 0.878 0.993
Obart08g16670.1	1<br>Obart01g07700.1 0.007 0.844 0.837 0.061 1<br>Obart08g16670.1 0.007 1 0.993 0.007 1	1<br>Osjap01g09050.1 0.007 0.844 0.837 0.061 1	1<br>Osind01g08880.1 0.102 0.986 0.884 0.004 0.567	1<br>Orufi01g08450.1 0.007 0.973 0.966 0.026 0.487	1<br>Oglab01g06790.1 0.007 0.844 0.837 0.061 1<br>Oglab08g15520.1 0.007 0.993 0.986 0.007 0.993	1<br>Olong_003647-R1 0.007 0.83 0.823 0.061 0.985	0	0	0	0
	1<br>Obart07g08560.1 0.694 0.952 0.258 0.009 0.192<br>Obart11g05910.1 0.007 0.694 0.687 0.752 0.834	1<br>Osjap01g24690.1 0.007 0.694 0.687 0.104 0.321<br>Osjap07g09900.1 0.694 0.993 0.299 0.005 0.123	1<br>Osind07g08950.1 0.694 0.993 0.299 0.004 0.111<br>Osind11g05650.1 0.007 0.694 0.687 0.103 0.319	1<br>Orufi01g23650.1 0.007 0.694 0.687 0.106 0.307<br>Orufi07g08630.1 0.694 0.993 0.299 0.005 0.115	1<br>Oglab07g08260.1 0.694 0.973 0.279 0.004 0.099<br>Oglab11g05440.1 0.007 0.694 0.687 0.573 0.683	1<br>Olong_005787-R1 0.007 0.694 0.687 0.103 0.319<br>Olong_016241-R1 0.728 0.932 0.204 0.735 0.768	1<br>Opunc01g21000.1 0.007 0.694 0.687 0.114 0.334<br>Opunc07g08090.1 0.694 0.993 0.299 0.004 0.09	1<br>Obrac07g05080.4 0.728 0.993 0.265 0.102 0.171<br>Obrac11g03720.1 0.007 0.694 0.687 0.112 0.34	1<br>Lperr01g17850.1 0.007 0.694 0.687 0.105 0.313<br>Lperr07g08590.1 0.694 0.993 0.299 0.022 0.124	1<br>AT3G55700.1 0.728 0.973 0.245 0.015 0.093<br>AT4G38130.1 0.007 0.68 0.673 0.09 0.269
Obart08g18520.1	1<br>Obart08g18520.1 0.001 1 0.999 0.001 1	0	0	0	0	0	0	0	0	0
	1<br>Obart08g18530.1 0.313 1 0.687 0.004 1<br>Obart08g18540.1 0.013 0.313 0.3 0.264 0.98	1<br>Osjap08g19640.1 0.313 1 0.687 0.004 1<br>Osjap08g19650.1 0.014 0.313 0.299 0.283 0.98	1<br>Osind08g21740.1 0.313 1 0.687 0.004 1<br>Osind10g18270.1 0.008 0.308 0.3 0.277 0.981	1<br>Orufi08g20720.1 0.313 1 0.687 0.004 1<br>Orufi08g20730.1 0.013 0.313 0.3 0.264 0.98	1<br>Oglab08g17140.1 0.313 1 0.687 0.004 1<br>Oglab08g17150.1 0.014 0.313 0.299 0.283 0.98	1<br>Olong_006289-R1 0.313 1 0.687 0.004 1<br>Olong_006290-R1 0.014 0.313 0.299 0.142 0.976	1<br>Opunc08g16450.1 0.313 1 0.687 0.004 1<br>Opunc08g16460.1 0.014 0.313 0.299 0.287 0.98	1<br>Obrac08g12190.5 0.313 1 0.687 0.004 1<br>Obrac08g12200.1 0.014 0.313 0.299 0.285 0.98	1<br>Lperr08g15390.1 0.313 0.999 0.686 0.004 0.998<br>Lperr08g15400.1 0.014 0.313 0.299 0.283 0.98	1<br>AT4G39980.1 0.014 0.31 0.296 0.335 1<br>AT4G40000.1 0.332 0.986 0.654 0.003 0.978
Obart08g23110.1	1<br>Obart08g23110.1 0.001 1 0.999 0.001 1	0	0	1<br>Orufi08g25640.1 0.014 1 0.986 0.056 1<br>Orufi08g25640.2 0.014 1 0.986 0.056 1<br>Orufi08g25640.3 0.014 1 0.986 0.056 1	0	0	0	0	1<br>Lperr08g19910.1 0.014 0.964 0.95 0.065 0.996<br>Lperr08g19910.2 0.014 0.964 0.95 0.065 0.996<br>Lperr08g19910.3 0.014 0.964 0.95 0.065 0.996	0
	1<br>Obart08g23140.1 0.487 0.964 0.477 0.03 0.992<br>Obart09g18630.1 0.056 0.444 0.388 0.43 0.984	1<br>Osjap08g24670.1 0.487 0.964 0.477 0.03 0.992<br>Osjap09g19050.1 0.056 0.444 0.388 0.464 0.985	1<br>Osind08g26930.1 0.001 0.445 0.444 0.035 0.978<br>Osind08g26940.1 0.487 0.964 0.477 0.046 0.992	1<br>Orufi08g25640.4 0.487 0.964 0.477 0.047 0.992<br>Orufi09g20160.1 0.056 0.444 0.388 0.464 0.985	1<br>Oglab09g16750.1 0.056 0.444 0.388 0.48 0.983<br>Oglab11g01890.1 0.491 0.968 0.477 0.08 0.987	0<br>Olong_020799-R2 0.051 0.385 0.334 0.252 0.989<br>Olong_024387-R1 0.54 0.968 0.428 0.002 0.982	1<br>Opunc08g21230.4 0.487 0.964 0.477 0.081 0.992<br>Opunc09g17040.1 0.003 0.444 0.441 0.044 0.975	1<br>Obrac08g15620.1 0.488 0.964 0.476 0.051 0.992<br>Obrac09g12080.1 0.056 0.444 0.388 0.147 0.976	1<br>Lperr09g14540.3 0.056 0.444 0.388 0.164 0.976<br>Lperr12g01980.1 0.49 0.972 0.482 0.082 1	1<br>AT1G09910.1 0.472 0.964 0.492 0.041 0.994<br>AT1G76270.1 0.001 0.424 0.423 0.038 0.944

Obart09g14980.1	1<br>Obart09g14980.1 0.002 1 0.998 0.002 1<br>Obart09g14980.2 0.002 1 0.998 0.002 1	1<br>Osjap09g15430.2 0.002 1 0.998 0.002 1<br>Osjap09g15430.3 0.002 1 0.998 0.002 1	1<br>Osind09g15570.1 0.002 1 0.998 0.002 1<br>Osind09g15570.2 0.002 1 0.998 0.002 1	1<br>Orufi09g16160.1 0.002 1 0.998 0.002 1<br>Orufi09g16160.2 0.002 1 0.998 0.002 1	1<br>Oglab09g13510.1 0.002 1 0.998 0.002 1<br>Oglab09g13510.2 0.002 1 0.998 0.002 1<br>Oglab09g13510.3 0.002 1 0.998 0.002 1<br>Oglab09g13510.4 0.002 1 0.998 0.002 1	0	1<br>Opunc09g13420.1 0.002 1 0.998 0.002 1<br>Opunc09g13420.2 0.002 1 0.998 0.002 1	1<br>Obrac09g09090.1 0.002 1 0.998 0.002 1	0	0
	1<br>Obart02g06180.1 0.01 0.565 0.555 0.01 0.997<br>Obart08g20390.1 0.569 0.993 0.424 0.631 0.988	1<br>Osjap02g06650.1 0.01 0.565 0.555 0.01 0.997<br>Osjap09g15430.1 0.569 1 0.431 0.131 1	1<br>Osind02g06940.1 0.01 0.565 0.555 0.01 0.997<br>Osind08g23920.1 0.569 0.993 0.424 0.131 0.988	1<br>Orufi04g24220.1 0.014 0.567 0.553 0.165 1<br>Orufi08g22880.1 0.569 0.993 0.424 0.131 0.988	1<br>Oglab08g19240.1 0.569 0.993 0.424 0.143 0.986<br>Oglab12g18680.1 0.002 0.567 0.565 0.003 1	1<br>Olong_008093-R2 0.596 0.991 0.395 0.25 0.996<br>Olong_024186-R1 0.002 0.567 0.565 0.003 1	1<br>Opunc04g20190.1 0.007 0.567 0.56 0.006 1<br>Opunc08g18560.1 0.569 0.993 0.424 0.128 0.969	1<br>Obrac01g09090.1 0.605 0.998 0.393 0.299 0.982<br>Obrac02g04550.3 0.009 0.565 0.556 0.214 0.997	1<br>Lperr04g18350.1 0.002 0.567 0.565 0.021 1<br>Lperr09g11120.1 0.569 1 0.431 0.156 1	1<br>AT1G68540.1 0.567 0.991 0.424 0.112 1<br>AT2G21270.3 0.003 0.565 0.562 0.059 0.997
Obart09g14980.2	1<br>Obart09g14980.1 0.002 1 0.998 0.002 1<br>Obart09g14980.2 0.002 1 0.998 0.002 1	1<br>Osjap09g15430.2 0.002 1 0.998 0.002 1<br>Osjap09g15430.3 0.002 1 0.998 0.002 1	1<br>Osind09g15570.1 0.002 1 0.998 0.002 1<br>Osind09g15570.2 0.002 1 0.998 0.002 1	1<br>Orufi09g16160.1 0.002 1 0.998 0.002 1<br>Orufi09g16160.2 0.002 1 0.998 0.002 1	1<br>Oglab09g13510.1 0.002 1 0.998 0.002 1<br>Oglab09g13510.2 0.002 1 0.998 0.002 1<br>Oglab09g13510.3 0.002 1 0.998 0.002 1<br>Oglab09g13510.4 0.002 1 0.998 0.002 1	0	1<br>Opunc09g13420.1 0.002 1 0.998 0.002 1<br>Opunc09g13420.2 0.002 1 0.998 0.002 1	1<br>Obrac09g09090.1 0.002 1 0.998 0.002 1	0	0
	1<br>Obart02g06180.1 0.01 0.54 0.53 0.01 0.997<br>Obart08g20390.1 0.544 0.993 0.449 0.631 0.988	1<br>Osjap02g06650.1 0.01 0.54 0.53 0.01 0.997<br>Osjap09g15430.1 0.544 1 0.456 0.131 1	1<br>Osind02g06940.1 0.01 0.54 0.53 0.01 0.997<br>Osind08g23920.1 0.544 0.993 0.449 0.131 0.988	1<br>Orufi04g24220.1 0.013 0.542 0.529 0.165 1<br>Orufi08g22880.1 0.544 0.993 0.449 0.131 0.988	1<br>Oglab08g19240.1 0.544 0.993 0.449 0.143 0.986<br>Oglab12g18680.1 0.002 0.542 0.54 0.003 1	1<br>Olong_013054-R1 0.547 0.992 0.445 0.149 0.979<br>Olong_024186-R1 0.002 0.542 0.54 0.003 1	1<br>Opunc04g20190.1 0.007 0.542 0.535 0.006 1<br>Opunc08g18560.1 0.544 0.993 0.449 0.128 0.969	1<br>Obrac02g04550.3 0.008 0.54 0.532 0.214 0.997<br>Obrac09g00560.1 0.548 0.998 0.45 0.164 0.966	1<br>Lperr04g18350.1 0.002 0.542 0.54 0.021 1<br>Lperr09g11120.1 0.544 1 0.456 0.156 1	1<br>AT1G68540.1 0.542 0.992 0.45 0.112 1<br>AT2G21270.3 0.003 0.54 0.537 0.059 0.997
Obart09g15360.1	1<br>Obart09g15360.1 0.001 1 0.999 0.001 1	0	1<br>Osind09g15930.1 0.001 1 0.999 0.057 1	1<br>Orufi09g16580.1 0.001 1 0.999 0.114 1	1<br>Oglab09g13920.1 0.001 1 0.999 0.114 1	0	0	0	0	0
	1<br>Obart03g04820.3 0.535 0.883 0.348 0.001 0.781<br>Obart08g21020.1 0.001 0.507 0.506 0.088 0.994	1<br>Osjap03g04840.3 0.507 0.883 0.376 0.018 0.769<br>Osjap09g15790.1 0.001 0.507 0.506 0.238 0.993	1<br>Osind08g24670.1 0.001 0.507 0.506 0.221 0.995<br>Osind09g02290.1 0.507 0.883 0.376 0.018 0.769	1<br>Orufi03g04550.5 0.507 0.883 0.376 0.017 0.733<br>Orufi08g23480.1 0.001 0.507 0.506 0.228 0.995	1<br>Oglab02g15910.1 0.003 0.474 0.471 0.048 0.988<br>Oglab09g02290.1 0.507 0.883 0.376 0.02 0.789	1<br>Olong_025005-R2 0.535 0.979 0.444 0.001 0.955<br>Olong_025006-R1 0.05 0.507 0.457 0.031 0.99	1<br>Opunc08g19030.2 0.001 0.507 0.506 0.156 0.976<br>Opunc09g13790.1 0.507 0.979 0.472 0.055 0.958	0<br>Obrac08g14010.2 0.001 0.508 0.507 0.044 0.989	1<br>Lperr08g17810.1 0.001 0.507 0.506 0.122 0.994<br>Lperr09g11510.2 0.507 0.979 0.472 0.01 0.958	1<br>AT2G32000.1 0.507 0.978 0.471 0.012 0.961<br>AT2G43900.1 0.003 0.481 0.478 0.165 0.843
Obart09g15430.1	1<br>Obart09g15430.1 0.003 1 0.997 0.003 1	0	1<br>Osind09g16000.1 0.003 0.943 0.94 0.002 1	0	0	0	0	0	0	0
	1<br>Obart01g41710.1 0.547 0.953 0.406 0.165 0.937<br>Obart09g15390.1 0.003 0.443 0.44 0.008 1	1<br>Osjap08g22450.1 0.003 0.443 0.44 0.254 1<br>Osjap09g15860.1 0.49 1 0.51 0.006 1	1<br>Osind01g48630.1 0.547 0.953 0.406 0.165 0.937<br>Osind08g24700.1 0.003 0.443 0.44 0.008 1	1<br>Orufi09g16660.1 0.49 1 0.51 0.006 1<br>Orufi09g16670.1 0.003 0.477 0.474 0.007 1	1<br>Oglab09g13980.1 0.003 0.443 0.44 0.008 1<br>Oglab09g13990.1 0.49 1 0.51 0.006 1	1<br>Olong_001207-R1 0.547 0.953 0.406 0.165 0.937<br>Olong_017992-R1 0.003 0.443 0.44 0.008 1	1<br>Opunc09g13840.1 0.003 0.443 0.44 0.008 1<br>Opunc09g13850.1 0.49 0.967 0.477 0.007 0.934	1<br>Obrac09g09430.1 0.003 0.443 0.44 0.008 1<br>Obrac09g09440.1 0.49 1 0.51 0.007 1	1<br>Lperr03g16320.1 0.003 0.443 0.44 0.007 1<br>Lperr09g11590.1 0.49 1 0.51 0.007 1	1<br>AT4G18100.1 0.003 0.443 0.44 0.008 1<br>AT5G45680.1 0.467 0.94 0.473 0.284 0.99
Obart09g16160.1	1<br>Obart09g16160.1 0.001 1 0.999 0.001 1	0	0	0	0	0	0	0	0	0
	1<br>Obart04g25680.1 0.485 0.974 0.489 0.159 0.786<br>Obart08g22080.1 0.004 0.441 0.437 0.04 0.979	1<br>Osjap09g16620.1 0.001 0.441 0.44 0.003 0.979<br>Osjap09g16630.1 0.485 1 0.515 0.2 1	1<br>Osind10g01650.1 0.001 0.441 0.44 0.003 0.979<br>Osind10g01660.1 0.485 1 0.515 0.245 1	1<br>Orufi09g17470.1 0.001 0.441 0.44 0.003 0.979<br>Orufi09g17480.1 0.485 1 0.515 0.2 1	1<br>Oglab09g14660.1 0.001 0.441 0.44 0.003 0.979<br>Oglab09g14670.1 0.485 1 0.515 0.2 1	1<br>Olong_000869-R1 0.485 1 0.515 0.072 1<br>Olong_000870-R1 0.001 0.441 0.44 0.003 0.979	1<br>Opunc09g14580.1 0.001 0.441 0.44 0.003 0.979<br>Opunc09g14590.1 0.485 1 0.515 0.205 1	1<br>Obrac09g10080.1 0.001 0.441 0.44 0.262 0.984<br>Obrac09g10090.2 0.485 0.999 0.514 0.083 1	1<br>Lperr09g12290.1 0.001 0.441 0.44 0.003 0.979<br>Lperr09g12300.1 0.485 0.999 0.514 0.268 0.998	1<br>AT2G17390.1 0.003 0.441 0.438 0.061 0.98<br>AT3G03310.1 0.485 0.995 0.51 0.157 0.996
Obart09g16460.1	1<br>Obart09g16460.1 0.001 1 0.999 0.001 1	0	0	0	0	0	0	0	0	0
	1	1	1	1	1	0	1	0	1	0

	Obart01g21490.2 0.727 1 0.273 0.17 0.551<br>Obart03g20890.1 0.025 0.659 0.634 0.028 0.915	Osjap02g19520.2 0.731 1 0.269 0.056 0.184<br>Osjap09g16920.1 0.001 0.721 0.72 0.002 0.914	Osind01g26310.1 0.727 0.997 0.27 0.344 0.652<br>Osind09g17080.1 0.001 0.721 0.72 0.002 0.918	Orufi01g24210.2 0.727 0.997 0.27 0.04 0.462<br>Orufi09g17770.1 0.001 0.721 0.72 0.002 0.918	Oglab01g17730.1 0.727 0.997 0.27 0.15 0.536<br>Oglab09g14960.1 0.001 0.721 0.72 0.002 0.929	Olong_025574-R2 0.098 0.659 0.561 0.059 0.907	Opunc01g21720.2 0.727 1 0.273 0.051 0.595<br>Opunc09g14890.1 0.001 0.721 0.72 0.002 0.917	Obrac09g10330.1 0.025 0.721 0.696 0.032 0.901	Lperr02g05180.1 0.731 1 0.269 0.121 0.552<br>Lperr09g12570.1 0.023 0.721 0.698 0.013 0.312	AT2G26260.1 0.025 0.73 0.705 0.021 0.92
Obart09g18850.1	1<br>Obart09g18850.1 0.001 1 0.999 0.001 1	0	0	0	0	0	0	0	0	0
	1<br>Obart02g30770.1 0.056 0.712 0.656 0.002 0.998<br>Obart09g19080.1 0.713 0.996 0.283 0.01 0.943	1<br>Osjap02g34900.1 0.001 0.712 0.711 0.001 0.999<br>Osjap09g18210.1 0.713 0.995 0.282 0.014 0.875	1<br>Osind09g18400.1 0.713 0.995 0.282 0.014 0.873<br>Osind09g19410.2 0.001 0.713 0.712 0.001 1	1<br>Orufi09g19250.1 0.713 0.995 0.282 0.014 0.873<br>Orufi09g20400.1 0.001 0.713 0.712 0.118 1	1<br>Oglab02g28320.1 0.001 0.712 0.711 0.001 0.999<br>Oglab09g16980.1 0.712 0.998 0.286 0.011 0.742	1<br>Olong_009633-R1 0.011 0.702 0.691 0.036 1<br>Olong_028691-R1 0.719 0.995 0.276 0.004 0.861	0<br>Opunc09g17250.1 0.001 0.712 0.711 0.001 0.999	0<br>Obrac09g12310.2 0.002 0.712 0.71 0.004 0.999	1<br>Lperr05g22000.1 0.713 0.988 0.275 0.036 1<br>Lperr09g14830.1 0.001 0.711 0.71 0.001 0.962	0<br>AT4G26600.1 0.115 0.601 0.486 0.165 0.902
Obart10g02510.1	1<br>Obart10g02510.1 0.001 1 0.999 0.001 1<br>Obart10g02510.2 0.001 1 0.999 0.001 1	0	1<br>Osind10g02110.1 0.001 0.995 0.994 0.001 0.952	0	1<br>Oglab10g02440.1 0.001 1 0.999 0.001 1<br>Oglab10g02440.2 0.001 1 0.999 0.001 1	0	0	0	0	0
	1<br>Obart10g00500.1 0.001 0.391 0.39 0.001 0.993<br>Obart11g08380.1 0.528 1 0.472 0.194 0.922	1<br>Osjap10g02940.1 0.021 0.393 0.372 0.027 0.984<br>Osjap10g02950.1 0.472 0.972 0.5 0.002 0.928	1<br>Osind10g00350.1 0.001 0.39 0.389 0.001 0.98<br>Osind11g07740.1 0.472 0.995 0.523 0.003 1	1<br>Orufi10g02520.1 0.021 0.222 0.201 0.065 0.758<br>Orufi10g02530.1 0.223 0.995 0.772 0.284 1	0<br>Oglab10g02440.3 0.455 1 0.545 0.005 1	1<br>Olong_016648-R1 0.223 0.995 0.772 0.265 1<br>Olong_020863-R1 0.005 0.222 0.217 0.007 0.797	1<br>Opunc10g02120.1 0.001 0.393 0.392 0.001 0.992<br>Opunc11g08150.1 0.472 1 0.528 0.003 0.911	1<br>Obrac03g10950.1 0.001 0.39 0.389 0.001 0.983<br>Obrac11g05340.1 0.472 1 0.528 0.002 0.922	1<br>Lperr10g00410.1 0.001 0.391 0.39 0.003 0.986<br>Lperr10g01950.1 0.472 1 0.528 0.003 0.895	0<br>AT1G21230.1 0.005 0.353 0.348 0.01 0.93<br>AT4G00830.1 0.523 0.972 0.449 0.194 0.891
Obart10g02510.2	1<br>Obart10g02510.1 0.001 1 0.999 0.001 1<br>Obart10g02510.2 0.001 1 0.999 0.001 1	0	1<br>Osind10g02110.1 0.001 0.995 0.994 0.001 0.952	0	1<br>Oglab10g02440.1 0.001 1 0.999 0.001 1<br>Oglab10g02440.2 0.001 1 0.999 0.001 1	0	0	0	0	0
	1<br>Obart10g00500.1 0.001 0.409 0.408 0.001 0.993<br>Obart11g08380.1 0.553 1 0.447 0.194 0.922	1<br>Osjap10g02940.1 0.022 0.411 0.389 0.027 0.984<br>Osjap10g02950.1 0.495 0.971 0.476 0.002 0.928	1<br>Osind10g00350.1 0.001 0.408 0.407 0.001 0.98<br>Osind11g07740.1 0.495 0.995 0.5 0.003 0.96	1<br>Orufi10g02520.1 0.022 0.232 0.21 0.065 0.758<br>Orufi10g02530.1 0.233 0.995 0.762 0.284 0.988	0<br>Oglab10g02440.3 0.476 1 0.524 0.005 1	1<br>Olong_016648-R1 0.233 0.995 0.762 0.265 1<br>Olong_028710-R1 0.031 0.232 0.201 0.004 0.754	1<br>Opunc10g02120.1 0.001 0.411 0.41 0.001 0.992<br>Opunc11g08150.1 0.495 1 0.505 0.003 0.911	1<br>Obrac03g10950.1 0.001 0.408 0.407 0.001 0.983<br>Obrac11g05340.1 0.495 1 0.505 0.002 0.922	1<br>Lperr10g00410.1 0.001 0.409 0.408 0.003 0.986<br>Lperr10g01950.1 0.495 1 0.505 0.003 0.895	0<br>AT1G21230.1 0.005 0.37 0.365 0.01 0.93<br>AT4G00830.1 0.548 0.971 0.423 0.194 0.891
Obart10g19720.1	1<br>Obart10g19720.1 0.002 1 0.998 0.002 1	0	1<br>Osind10g19230.1 0.002 1 0.998 0.002 1	1<br>Orufi10g20960.1 0.002 1 0.998 0.002 1	1<br>Oglab10g16090.1 0.002 0.992 0.99 0.001 0.861	0	1<br>Opunc10g18180.1 0.002 0.992 0.99 0.002 0.85	0	0	0
	1<br>Obart01g17550.1 0.483 0.992 0.509 0.174 0.721<br>Obart08g03750.1 0.042 0.431 0.389 0.057 0.95	1<br>Osjap10g22750.1 0.473 0.992 0.519 0.061 0.836<br>Osjap10g22760.1 0.002 0.451 0.449 0.003 0.993	1<br>Osind01g20890.1 0.483 0.992 0.509 0.154 0.668<br>Osind08g03990.1 0.042 0.431 0.389 0.057 0.95	1<br>Orufi01g19490.1 0.483 0.992 0.509 0.171 0.72<br>Orufi08g04140.1 0.042 0.431 0.389 0.057 0.95	1<br>Oglab01g14760.1 0.483 0.992 0.509 0.2 0.827<br>Oglab08g03250.2 0.042 0.431 0.389 0.052 0.955	1<br>Olong_006312-R1 0.48 0.987 0.507 0.067 0.835<br>Olong_006313-R1 0.002 0.451 0.449 0.003 0.993	1<br>Opunc01g18040.1 0.483 0.992 0.509 0.174 0.822<br>Opunc08g03700.1 0.016 0.431 0.415 0.011 0.951	1<br>Obrac01g13330.1 0.487 0.992 0.505 0.196 0.825<br>Obrac10g13120.1 0.002 0.451 0.449 0.004 0.993	1<br>Lperr10g15590.1 0.48 0.992 0.512 0.07 0.79<br>Lperr10g15600.1 0.002 0.448 0.446 0.003 0.979	1<br>AT1G32080.1 0.484 0.989 0.505 0.188 0.82<br>AT2G32260.1 0.041 0.432 0.391 0.078 0.825
Obart11g00370.1	1<br>Obart11g00370.1 0.001 1 0.999 0.001 1	0	0	0	0	0	0	0	0	0
	1<br>Obart12g00180.1 0.631 1 0.369 0.003 1<br>Obart12g00190.6 0.001 0.568 0.567 0.049 0.954	1<br>Osjap12g00400.1 0.631 1 0.369 0.003 1<br>Osjap12g00410.6 0.001 0.568 0.567 0.058 0.955	1<br>Osind12g00490.1 0.631 1 0.369 0.003 1<br>Osind12g00500.2 0.001 0.568 0.567 0.18 0.961	1<br>Orufi05g21080.1 0.055 0.568 0.513 0.019 0.946<br>Orufi12g00420.1 0.631 1 0.369 0.003 1	1<br>Oglab12g00090.1 0.631 1 0.369 0.003 1<br>Oglab12g00100.6 0.001 0.568 0.567 0.19 0.961	0<br>Olong_018889-R1 0.049 0.568 0.519 0.09 0.95	1<br>Opunc05g17550.1 0.049 0.568 0.519 0.084 0.95<br>Opunc12g00380.3 0.658 1 0.342 0.064 1	1<br>Obrac11g00100.1 0.636 1 0.364 0.013 1<br>Obrac11g00110.4 0.047 0.568 0.521 0.076 0.945	1<br>Lperr05g15790.1 0.049 0.568 0.519 0.088 0.95<br>Lperr11g00400.1 0.638 1 0.362 0.006 1	0<br>AT3G11070.1 0.041 0.578 0.537 0.088 0.988
Obart11g01060.1	1<br>Obart11g01060.1 0.001 1 0.999 0.001 1	1<br>Osjap11g01220.1 0.001 1 0.999 0.001 1<br>Osjap12g01140.1 0.001 1 0.999 0.001 1	1<br>Osind11g00690.1 0.001 0.954 0.953 0.001 0.999<br>Osind12g01290.1 0.001 1 0.999 0.001 1	1<br>Orufi11g01000.1 0.001 1 0.999 0.001 1<br>Orufi12g01130.1 0.001 1 0.999 0.001 1	1<br>Oglab12g00800.1 0.001 1 0.999 0.001 1	0	1<br>Opunc12g00940.1 0.001 1 0.999 0.001 1	0	0	0
	1	1	1	1	1	1	1	1	1	1

	Obart03g12120.1 0.479 0.982 0.503 0.121 0.633<br>Obart12g00790.1 0.001 0.42 0.419 0.002 0.949	Osjap03g13100.1 0.479 0.982 0.503 0.097 0.622<br>Osjap07g30390.1 0.01 0.42 0.41 0.013 0.944	Osind03g12930.1 0.479 0.982 0.503 0.121 0.633<br>Osind07g27070.1 0.007 0.42 0.413 0.006 0.946	Orufi03g17220.1 0.485 1 0.515 0.027 1<br>Orufi07g27420.1 0.01 0.42 0.41 0.013 0.946	Oglab03g12120.1 0.479 0.982 0.503 0.121 0.633<br>Oglab07g22980.1 0.007 0.42 0.413 0.007 0.94	Olong_007715-R1 0.482 1 0.518 0.007 1<br>Olong_008211-R1 0.007 0.42 0.413 0.006 0.946	Opunc03g11680.1 0.479 1 0.521 0.148 1<br>Opunc11g00920.1 0.001 0.42 0.419 0.142 0.951	Obrac03g09720.2 0.479 1 0.521 0.142 1<br>Obrac12g00780.1 0.001 0.42 0.419 0.11 0.953	Lperr03g10720.1 0.479 0.996 0.517 0.148 0.954<br>Lperr11g00920.1 0.001 0.42 0.419 0.002 0.949	AT3G46740.1 0.482 1 0.518 0.161 1<br>AT4G20110.2 0.001 0.42 0.419 0.002 0.946
Obart11g04380.1	1<br>Obart11g04380.1 0.001 1 0.999 0.001 1	0	0	0	0	0	0	0	0	0
	1<br>Obart03g11190.1 0.061 0.586 0.525 0.048 0.746<br>Obart03g32320.1 0.609 0.926 0.317 0.383 0.899	1<br>Osjap03g34930.1 0.609 0.926 0.317 0.383 0.899<br>Osjap11g04880.1 0.001 0.601 0.6 0.002 0.78	1<br>Osind03g34710.1 0.609 0.926 0.317 0.383 0.899<br>Osind11g04140.1 0.001 0.601 0.6 0.002 0.78	1<br>Orufi03g33710.1 0.609 0.926 0.317 0.383 0.899<br>Orufi11g04260.1 0.001 0.601 0.6 0.002 0.738	0<br>Oglab11g04130.1 0.001 0.601 0.6 0.002 0.78	1<br>Olong_004085-R1 0.061 0.586 0.525 0.048 0.744<br>Olong_009498-R1 0.609 0.926 0.317 0.13 0.858	1<br>Opunc03g29490.1 0.609 0.926 0.317 0.377 0.904<br>Opunc11g04020.1 0.001 0.601 0.6 0.002 0.777	1<br>Obrac03g08970.1 0.071 0.617 0.546 0.061 0.804<br>Obrac11g02780.1 0.724 1 0.276 0.005 0.985	1<br>Lperr03g26910.1 0.609 0.926 0.317 0.377 0.904<br>Lperr11g03800.1 0.001 0.601 0.6 0.002 0.77	1<br>AT3G57620.1 0.054 0.587 0.533 0.044 0.762<br>AT5G64090.1 0.587 1 0.413 0.395 1
Obart11g16070.1	1<br>Obart11g16070.1 0.001 1 0.999 0.001 1	0	0	0	1<br>Oglab11g14350.1 0.013 0.975 0.962 0.001 1<br>Oglab11g14350.2 0.013 1 0.987 0.001 1	0	0	0	0	0
	1<br>Obart02g07160.1 0.685 1 0.315 0.002 1<br>Obart08g10830.1 0.037 0.654 0.617 0.004 0.995	1<br>Osjap02g07570.1 0.685 1 0.315 0.002 1<br>Osjap11g18290.1 0.001 0.632 0.631 0.006 0.989	1<br>Osind11g13470.1 0.001 0.605 0.604 0.005 0.997<br>Osind11g13490.1 0.684 1 0.316 0.081 1	1<br>Orufi11g17130.1 0.001 0.632 0.631 0.006 0.989<br>Orufi11g17160.1 0.685 1 0.315 0.002 1	1<br>Oglab02g07220.2 0.685 1 0.315 0.003 1<br>Oglab11g18500.1 0.036 0.633 0.597 0.003 0.966	1<br>Olong_012209-R1 0.685 1 0.315 0.002 1<br>Olong_012210-R1 0.001 0.632 0.631 0.01 0.981	1<br>Opunc11g13170.1 0.685 1 0.315 0.002 1<br>Opunc11g13180.1 0.033 0.634 0.601 0.145 0.975	1<br>Obrac02g05260.1 0.685 1 0.315 0.002 1<br>Obrac10g05860.1 0.038 0.627 0.589 0.005 0.946	1<br>Lperr11g12540.1 0.035 0.63 0.595 0.002 0.969<br>Lperr12g06590.1 0.674 1 0.326 0.078 1	0<br>AT2G44350.2 0.685 1 0.315 0.002 0.998
Obart11g22080.1	1<br>Obart11g22080.1 0.001 1 0.999 0.001 1<br>Obart11g23070.1 0.024 0.973 0.949 0.038 0.936	0	0	0	1<br>Oglab11g20100.1 0.024 0.953 0.929 0.012 0.992	1<br>Olong_001283-R1 0.004 0.94 0.936 0.012 0.965	1<br>Opunc11g18660.1 0.001 0.987 0.986 0.001 0.924<br>Opunc11g18660.2 0.001 0.987 0.986 0.001 0.925<br>Opunc11g18660.3 0.001 0.987 0.986 0.001 0.89<br>Opunc11g19450.1 0.024 0.973 0.949 0.016 0.933	0	1<br>Lperr11g13500.1 0.008 0.944 0.936 0.003 0.998	0
	1<br>Obart11g12900.1 0.008 0.474 0.466 0.002 0.766<br>Obart11g22110.1 0.506 0.988 0.482 0.003 0.891	1<br>Osjap10g09150.6 0.007 0.461 0.454 0.005 0.72<br>Osjap11g25990.5 0.5 0.987 0.487 0.086 0.998	0<br>Osind05g25410.1 0.007 0.534 0.527 0.001 0.979	1<br>Orufi11g13640.1 0.008 0.474 0.466 0.002 0.766<br>Orufi11g24060.2 0.49 0.993 0.503 0.001 0.999	1<br>Oglab11g11420.1 0.008 0.461 0.453 0.002 0.759<br>Oglab11g19250.1 0.498 0.987 0.489 0.002 0.999	1<br>Olong_000091-R1 0.502 0.986 0.484 0.001 0.997<br>Olong_000093-R1 0.001 0.494 0.493 0.001 1	1<br>Opunc05g18050.1 0.024 0.492 0.468 0.031 0.839<br>Opunc11g18620.1 0.505 0.987 0.482 0.004 0.998	1<br>Obrac11g13000.2 0.506 0.989 0.483 0.006 0.999<br>Obrac11g13510.3 0.024 0.478 0.454 0.023 0.869	1<br>Lperr11g18720.1 0.505 0.995 0.49 0.002 0.997<br>Lperr11g19280.1 0.006 0.455 0.449 0.016 0.849	0<br>AT5G02800.1 0.585 0.998 0.413 0.153 0.989
Obart11g22180.1	1<br>Obart11g22180.1 0.001 1 0.999 0.001 1	0	1<br>Osind06g17590.1 0.038 0.984 0.946 0.037 0.999<br>Osind08g03120.1 0.028 0.98 0.952 0.059 0.995	0	0	1<br>Olong_000078-R1 0.029 0.872 0.843 0.013 0.995	0	0	0	0
	1<br>Obart11g22190.1 0.001 0.37 0.369 0.029 0.928<br>Obart11g22210.1 0.373 0.971 0.598 0.002 0.971	1<br>Osjap11g26070.1 0.374 1 0.626 0.095 1<br>Osjap11g26080.1 0.001 0.374 0.373 0.002 0.909	1<br>Osind01g01210.1 0.374 1 0.626 0.091 1<br>Osind01g30060.1 0.001 0.374 0.373 0.002 0.909	1<br>Orufi09g00060.1 0.374 1 0.626 0.11 1<br>Orufi09g00070.1 0.001 0.374 0.373 0.022 0.913	1<br>Oglab11g19350.1 0.001 0.374 0.373 0.002 0.896<br>Oglab11g19360.1 0.374 1 0.626 0.091 1	1<br>Olong_022538-R1 0.001 0.374 0.373 0.002 0.903<br>Olong_022539-R1 0.391 1 0.609 0.1 1	1<br>Opunc11g18540.2 0.009 0.369 0.36 0.022 0.956<br>Opunc11g18800.1 0.369 0.971 0.602 0.09 0.976	1<br>Obrac11g13060.1 0.004 0.374 0.37 0.251 0.937<br>Obrac11g13070.1 0.374 0.968 0.594 0.016 0.953	0<br>Lperr11g18890.1 0.374 0.977 0.603 0.119 0.991	0<br>AT5G57580.1 0.498 0.936 0.438 0.134 0.737
Obart12g01910.1	1<br>Obart12g01910.1 0.002 1 0.998 0.002 1	0	0	0	1<br>Oglab12g01890.1 0.002 0.938 0.936 0.002 0.974	0	1<br>Opunc11g02000.1 0.002 0.938 0.936 0.002 0.883<br>Opunc12g01920.1 0.002 0.938 0.936 0.002 0.951	1<br>Obrac11g01380.1 0.022 0.945 0.923 0.036 0.949	0	0
	1<br>Obart01g34520.1 0.71 1 0.29 0.005 1<br>Obart11g02130.1 0.013 0.691 0.678 0.051 0.947	1<br>Osjap11g02310.1 0.712 1 0.288 0.005 1<br>Osjap12g02360.1 0.002 0.691 0.689 0.002 0.945	1<br>Osind11g01670.1 0.712 0.997 0.285 0.004 0.742<br>Osind12g02620.1 0.002 0.691 0.689 0.002 0.945	1<br>Orufi01g37930.2 0.71 1 0.29 0.005 1<br>Orufi12g02190.1 0.022 0.6 0.578 0.159 1	1<br>Oglab11g01920.1 0.712 1 0.288 0.005 1<br>Oglab11g01930.1 0.013 0.691 0.678 0.051 0.947	1<br>Olong_019069-R2 0.002 0.691 0.689 0.002 0.945<br>Olong_020510-R1 0.71 1 0.29 0.005 1	0<br>Opunc03g30920.1 0.688 0.949 0.261 0.471 0.991	1<br>Obrac12g01690.1 0.712 1 0.288 0.005 1<br>Obrac12g01700.2 0.002 0.691 0.689 0.002 0.948	1<br>Lperr07g20930.1 0.025 0.691 0.666 0.09 0.956<br>Lperr12g02000.1 0.709 1 0.291 0.866 1	1<br>AT2G19540.1 0.022 0.691 0.669 0.062 0.947<br>AT2G27030.3 0.71 1 0.29 0.006 1

Obart12g02370.1	1<br>Obart12g02370.1 0.001 1 0.999 0.001 1	0	0	0	0	0	0	0	0	0
	1<br>Obart11g02670.1 0.492 1 0.508 0.277 1<br>Obart11g02690.1 0.001 0.434 0.433 0.003 0.982	1<br>Osjap11g03000.1 0.001 0.434 0.433 0.002 0.986<br>Osjap12g02930.1 0.492 1 0.508 0.286 1	1<br>Osind12g02950.1 0.492 1 0.508 0.286 1<br>Osind12g02970.1 0.001 0.434 0.433 0.002 0.935	1<br>Orufi11g02630.1 0.492 1 0.508 0.063 1<br>Orufi12g02750.1 0.075 0.434 0.359 0.003 0.907	1<br>Oglab12g02420.1 0.492 1 0.508 0.283 1<br>Oglab12g02460.1 0.001 0.434 0.433 0.002 0.822	0<br>Olong_011875-R1 0.103 0.411 0.308 0.161 0.908<br>Olong_028720-R1 0.557 1 0.443 0.003 1	1<br>Opunc11g02440.1 0.492 1 0.508 0.284 1<br>Opunc11g02450.1 0.001 0.434 0.433 0.002 0.932	0<br>Obrac11g01640.1 0.51 1 0.49 0.034 0.766	0<br>Lperr12g02500.1 0.147 0.432 0.285 0.144 0.868	0<br>AT3G47930.1 0.492 0.993 0.501 0.311 0.995
Obart12g02600.1	1<br>Obart12g02600.1 0.003 1 0.997 0.003 1	0	0	1<br>Orufi12g03000.1 0.003 0.978 0.975 0.004 0.823	1<br>Oglab12g02710.1 0.003 1 0.997 0.004 1	0	0	0	0	0
	1<br>Obart01g16130.1 0.588 1 0.412 0.209 1<br>Obart12g02460.1 0.009 0.581 0.572 0.02 0.975	1<br>Osjap01g18900.1 0.588 1 0.412 0.308 1<br>Osjap12g03070.1 0.009 0.581 0.572 0.02 0.975	1<br>Osind12g03230.1 0.606 1 0.394 0.292 1<br>Osind12g03240.1 0.003 0.588 0.585 0.008 1	1<br>Orufi02g31540.1 0.744 0.988 0.244 0.399 0.957<br>Orufi12g02840.1 0.009 0.581 0.572 0.02 0.975	1<br>Oglab11g14940.1 0.653 0.959 0.306 0.011 1<br>Oglab12g02550.1 0.009 0.581 0.572 0.02 0.975	1<br>Olong_021782-R1 0.713 0.975 0.262 0.051 0.859<br>Olong_027258-R1 0.009 0.581 0.572 0.28 0.981	0<br>Opunc02g10450.1 0.694 1 0.306 0.24 1<br>Opunc10g12380.1 0.297 0.641 0.344 0.499 0.843	0<br>Obrac11g01600.1 0.231 0.578 0.347 0.076 0.91	0<br>Lperr02g24330.1 0.694 0.919 0.225 0.287 0.816<br>Lperr02g27700.1 0.297 0.603 0.306 0.415 0.71	0<br>AT1G68180.1 0.272 0.584 0.312 0.31 0.746
Obart12g09410.1	1<br>Obart12g09410.1 0.001 1 0.999 0.001 1	1<br>Osjap12g12320.1 0.001 1 0.999 0.001 1	1<br>Osind12g09530.1 0.001 1 0.999 0.001 1	1<br>Orufi12g10460.1 0.001 1 0.999 0.001 1	1<br>Oglab12g09470.1 0.001 1 0.999 0.001 1	0	0	0	0	0
	1<br>Obart01g39260.2 0.452 0.998 0.546 0.017 0.994<br>Obart03g07210.1 0.001 0.306 0.305 0.004 0.996	1<br>Osjap01g44780.3 0.452 0.998 0.546 0.017 0.994<br>Osjap03g07600.1 0.001 0.306 0.305 0.004 0.996	1<br>Osind01g46040.1 0.452 0.998 0.546 0.186 0.995<br>Osind03g07670.1 0.001 0.306 0.305 0.004 0.996	1<br>Orufi01g42650.3 0.452 0.998 0.546 0.228 0.995<br>Orufi03g07180.1 0.001 0.306 0.305 0.004 0.996	1<br>Oglab01g34040.1 0.477 0.998 0.521 0.203 0.995<br>Oglab03g07280.1 0.001 0.306 0.305 0.004 0.996	1<br>Olong_021912-R1 0.376 1 0.624 0.002 1<br>Olong_021913-R1 0.001 0.305 0.304 0.004 0.996	1<br>Opunc12g08570.1 0.406 1 0.594 0.152 1<br>Opunc12g08580.1 0.001 0.305 0.304 0.004 0.996	1<br>Obrac12g06490.1 0.001 0.305 0.304 0.004 0.996<br>Obrac12g06500.1 0.449 1 0.551 0.103 1	1<br>Lperr03g06600.1 0.001 0.304 0.303 0.004 0.992<br>Lperr12g08310.1 0.452 0.999 0.547 0.002 0.998	1<br>AT4G34670.1 0.001 0.297 0.296 0.004 0.966<br>AT5G67540.2 0.477 0.993 0.516 0.053 1
Obart12g13650.1	1<br>Obart12g13650.1 0.004 1 0.996 0.004 1	0	0	0	0	0	0	0	0	0
	1<br>Obart03g06960.1 0.028 0.762 0.734 0.145 0.991<br>Obart11g20740.1 0.79 1 0.21 0.514 1	1<br>Osjap07g23370.2 0.79 0.992 0.202 0.086 0.168<br>Osjap12g17390.1 0.004 0.786 0.782 0.005 1	1<br>Osind11g16870.1 0.79 0.996 0.206 0.143 0.968<br>Osind12g14200.1 0.004 0.786 0.782 0.005 1	1<br>Orufi07g20930.2 0.79 0.992 0.202 0.087 0.169<br>Orufi12g14980.1 0.004 0.786 0.782 0.005 1	1<br>Oglab11g18280.1 0.79 1 0.21 0.514 1<br>Oglab12g12900.1 0.004 0.786 0.782 0.005 1	0<br>Olong_014123-R1 0.024 0.69 0.666 0.098 0.995	1<br>Opunc07g18980.2 0.79 1 0.21 0.014 0.105<br>Opunc12g12320.1 0.012 0.786 0.774 0.01 1	1<br>Obrac03g05620.1 0.056 0.706 0.65 0.158 0.937<br>Obrac12g08990.2 0.754 0.976 0.222 0.26 0.844	1<br>Lperr11g17030.1 0.79 1 0.21 0.116 0.884<br>Lperr12g11520.1 0.02 0.698 0.678 0.005 0.88	0<br>AT4G25830.1 0.04 0.746 0.706 0.017 1
Obart12g18260.1	1<br>Obart12g18260.1 0.002 1 0.998 0.002 1	0	0	0	0	0	1<br>Opunc12g16510.1 0.029 1 0.971 0.374 1	0	1<br>Lperr12g15270.1 0.002 1 0.998 0.002 1<br>Lperr12g15270.2 0.002 1 0.998 0.003 1	0
	1<br>Obart05g22220.1 0.431 1 0.569 0.47 1<br>Obart09g04390.1 0.002 0.282 0.28 0.02 0.77	1<br>Osjap03g34300.1 0.002 0.282 0.28 0.437 0.827<br>Osjap12g23030.1 0.402 1 0.598 0.42 1	1<br>Osind12g19080.1 0.402 1 0.598 0.42 1<br>Osind12g19090.1 0.002 0.282 0.28 0.007 0.767	1<br>Orufi09g04980.1 0.002 0.282 0.28 0.02 0.77<br>Orufi12g20240.1 0.402 1 0.598 0.42 1	1<br>Oglab03g30800.1 0.002 0.282 0.28 0.007 0.762<br>Oglab12g16450.1 0.402 1 0.598 0.42 1	1<br>Olong_014799-R1 0.002 0.282 0.28 0.007 0.8<br>Olong_025924-R1 0.402 1 0.598 0.195 1	1<br>Opunc05g19900.1 0.431 1 0.569 0.452 1<br>Opunc09g03850.1 0.002 0.282 0.28 0.02 0.77	1<br>Obrac03g23420.1 0.002 0.282 0.28 0.006 0.696<br>Obrac12g11780.1 0.404 1 0.596 0.236 1	1<br>Lperr03g26330.1 0.002 0.282 0.28 0.339 0.837<br>Lperr05g18130.1 0.431 1 0.569 0.387 1	0<br>AT1G23260.1 0.01 0.282 0.272 0.013 0.696<br>AT4G14770.1 0.431 0.652 0.221 0.682 0.818

((((((_a1,rufi),_a2),longi),punctata),brachy),leersia),tair);

chimeric gene id	INGROUP		MIDGROUP					OUTGROUP		
	glab	barthii	jap	indica	rufi	longi	punctata	brachy	leersia	tair
Obart01g01710.1	1<br>Oglab01g01590.1 0.001 0.986 0.985 0.001 0.987	1<br>Obart01g01710.1 0.001 1 0.999 0.001 1	0	1<br>Osind01g02220.1 0.001 0.986 0.985 0.056 0.987	0	1<br>Olong_021362-R1 0.001 0.986 0.985 0.048 0.986	1<br>Opunc01g01490.1 0.002 1 0.998 0.021 0.999<br>Opunc01g01490.2 0.002 1 0.998 0.024 0.999	0	0	0

	1<br>Oglab01g01570.1 0.539 0.986 0.447 0.043 0.973<br>Oglab05g03370.1 0.002 0.47 0.468 0.104 0.976	1<br>Obart01g01680.1 0.539 0.986 0.447 0.046 0.97<br>Obart05g03720.1 0.002 0.47 0.468 0.104 0.976	1<br>Osjap01g02100.1 0.001 0.47 0.469 0.002 0.973<br>Osjap01g02110.1 0.513 0.986 0.473 0.039 0.974	1<br>Osind01g02230.1 0.513 0.999 0.486 0.061 0.989<br>Osind05g03930.1 0.002 0.47 0.468 0.104 0.976	1<br>Orufi01g01810.1 0.001 0.47 0.469 0.002 0.973<br>Orufi01g01830.1 0.539 0.986 0.447 0.079 0.987	1<br>Olong_016471-R1 0.002 0.47 0.468 0.106 0.972<br>Olong_021361-R1 0.515 0.955 0.44 0.002 0.971	1<br>Opunc01g01490.3 0.539 0.986 0.447 0.056 0.97<br>Opunc05g03460.1 0.016 0.47 0.454 0.119 0.971	1<br>Obrac01g01340.1 0.486 0.986 0.5 0.001 0.999<br>Obrac01g17910.1 0.004 0.47 0.466 0.248 0.98	1<br>Lperr01g01640.1 0.51 0.986 0.476 0.051 0.975<br>Lperr05g03400.1 0.015 0.47 0.455 0.158 0.972	1<br>AT1G71040.1 0.528 0.986 0.458 0.021 0.972<br>AT3G48670.1 0.002 0.47 0.468 0.117 0.971
Obart01g02790.1	1<br>Oglab01g02400.1 0.03 1 0.97 0.135 1	1<br>Obart01g02790.1 0.001 1 0.999 0.001 1	0	0	0	0	1<br>Opunc01g02430.1 0.011 0.932 0.921 0.058 0.93	0	0	0
	1<br>Oglab01g02390.1 0.001 0.222 0.221 0.004 0.992<br>Oglab09g10930.1 0.236 0.954 0.718 0.008 0.94	0<br>Obart09g11930.1 0.236 0.954 0.718 0.008 0.94	1<br>Osjap01g03230.1 0.234 1 0.766 0.011 1<br>Osjap01g03250.1 0.001 0.223 0.222 0.004 0.996	1<br>Osind01g03370.1 0.001 0.222 0.221 0.004 0.992<br>Osind09g12280.1 0.236 0.954 0.718 0.008 0.948	1<br>Orufi01g02840.1 0.237 1 0.763 0.001 1<br>Orufi01g02850.1 0.001 0.223 0.222 0.004 0.996	0<br>Olong_016492-R1 0.041 0.82 0.779 0.023 0.979	1<br>Opunc01g02420.1 0.001 0.222 0.221 0.004 0.992<br>Opunc02g11760.1 0.239 0.947 0.708 0.026 0.984	0<br>Obrac01g02250.1 0.234 0.975 0.741 0.004 0.987	0<br>Lperr01g02550.1 0.237 0.975 0.738 0.01 0.997	0<br>AT2G39795.1 0.002 0.224 0.222 0.1 1<br>AT5G45480.1 0.671 0.956 0.285 0.656 0.991
Obart01g03110.1	0	1<br>Obart01g03110.1 0.001 1 0.999 0.001 1	0	0	0	0	0	0	1<br>Lperr01g02790.1 0.063 1 0.937 0.043 1<br>Lperr01g02790.2 0.063 1 0.937 0.042 1	0
	1<br>Oglab01g02690.1 0.425 1 0.575 0.077 1<br>Oglab01g02700.1 0.063 0.341 0.278 0.13 0.996	0<br>Obart09g17680.1 0.072 0.338 0.266 0.289 0.98<br>Obart10g18430.1 0.766 0.999 0.233 0.695 0.986	1<br>Osjap01g03540.1 0.395 1 0.605 0.016 1<br>Osjap01g03550.1 0.063 0.341 0.278 0.13 0.996	1<br>Osind01g03690.1 0.425 1 0.575 0.072 1<br>Osind01g03700.1 0.063 0.341 0.278 0.13 0.996	1<br>Orufi01g03140.1 0.425 1 0.575 0.073 1<br>Orufi01g03150.1 0.063 0.341 0.278 0.13 0.996	1<br>Olong_018924-R1 0.425 1 0.575 0.002 1<br>Olong_018925-R1 0.063 0.341 0.278 0.13 0.996	1<br>Opunc01g02730.1 0.423 0.959 0.536 0.077 0.955<br>Opunc09g16060.1 0.072 0.338 0.266 0.363 0.982	1<br>Obrac01g02390.1 0.412 0.956 0.544 0.071 0.967<br>Obrac01g28390.1 0.066 0.341 0.275 0.141 0.992	0<br>Lperr09g09500.1 0.782 0.997 0.215 0.722 0.997<br>Lperr09g13570.1 0.072 0.338 0.266 0.299 0.981	1<br>AT2G39690.1 0.458 1 0.542 0.006 1<br>AT3G55440.1 0.067 0.339 0.272 0.142 0.984
Obart01g04220.1	1<br>Oglab01g03790.1 0.001 1 0.999 0.001 1	1<br>Obart01g04220.1 0.001 1 0.999 0.001 1	0	0	0	0	0	0	0	0
	1<br>Oglab05g04170.2 0.048 0.567 0.519 0.114 1<br>Oglab07g07650.1 0.62 0.997 0.377 0.002 0.778	1<br>Obart05g04510.1 0.036 0.567 0.531 0.012 1<br>Obart07g07920.1 0.62 0.997 0.377 0.002 0.778	1<br>Osjap01g04780.1 0.62 1 0.38 0.002 1<br>Osjap05g04880.1 0.048 0.567 0.519 0.008 1	1<br>Osind01g04880.1 0.62 1 0.38 0.002 1<br>Osind05g04760.1 0.048 0.567 0.519 0.008 1	1<br>Orufi01g04370.1 0.62 1 0.38 0.002 1<br>Orufi05g04740.1 0.048 0.567 0.519 0.008 1	1<br>Olong_014225-R1 0.044 0.563 0.519 0.001 0.984<br>Olong_014227-R1 0.62 1 0.38 0.002 1	1<br>Opunc01g03980.1 0.62 1 0.38 0.002 1<br>Opunc07g16230.1 0.042 0.556 0.514 0.014 0.972	1<br>Obrac01g03070.1 0.62 1 0.38 0.002 1<br>Obrac05g03450.2 0.039 0.567 0.528 0.003 1	1<br>Lperr01g03770.1 0.62 1 0.38 0.002 1<br>Lperr01g03780.1 0.039 0.561 0.522 0.002 0.979	1<br>AT5G01660.1 0.053 0.556 0.503 0.015 0.97<br>AT5G64190.1 0.62 0.997 0.377 0.002 0.998
Obart01g07430.1	1<br>Oglab01g06470.1 0.013 1 0.987 0.02 1	1<br>Obart01g07430.1 0.001 1 0.999 0.001 1	1<br>Osjap01g08660.1 0.007 1 0.993 0.026 1	1<br>Osind01g08490.1 0.007 1 0.993 0.026 1	1<br>Orufi01g08070.1 0.007 1 0.993 0.026 1	1<br>Olong_011567-R1 0.036 1 0.964 0.001 1	0	0	0	0
	1<br>Oglab01g21070.2 0.078 0.648 0.57 0.249 0.983<br>Oglab05g00530.1 0.713 0.997 0.284 0.146 1	1<br>Obart01g25090.1 0.078 0.648 0.57 0.219 0.982<br>Obart05g00540.1 0.713 0.997 0.284 0.146 1	1<br>Osjap01g29230.2 0.078 0.648 0.57 0.25 0.983<br>Osjap05g00560.2 0.713 0.997 0.284 0.146 1	1<br>Osind01g30690.1 0.078 0.648 0.57 0.267 0.982<br>Osind05g00540.1 0.713 0.997 0.284 0.126 1	1<br>Orufi01g27950.1 0.078 0.648 0.57 0.25 0.983<br>Orufi01g48560.2 0.713 0.983 0.27 0.085 0.984	1<br>Olong_003039-R1 0.713 0.997 0.284 0.128 1<br>Olong_003692-R2 0.109 0.648 0.539 0.244 0.979	1<br>Opunc01g07160.1 0.656 1 0.344 0.111 1<br>Opunc01g07170.1 0.003 0.649 0.646 0.034 0.984	1<br>Obrac01g05760.3 0.66 1 0.34 0.082 1<br>Obrac01g18840.2 0.001 0.648 0.647 0.062 0.974	1<br>Lperr01g07030.1 0.66 1 0.34 0.018 1<br>Lperr01g07040.2 0.002 0.649 0.647 0.013 0.989	1<br>AT1G54350.1 0.659 0.994 0.335 0.076 0.975<br>AT3G61740.1 0.009 0.648 0.639 0.077 0.983
Obart01g25040.1	0	1<br>Obart01g25040.1 0.003 1 0.997 0.003 1	0	1<br>Osind01g30660.1 0.003 1 0.997 0.003 1	1<br>Orufi01g27920.1 0.003 0.894 0.891 0.003 0.862	1<br>Olong_017584-R1 0.171 1 0.829 0.059 1	0	0	0	0
	0<br>Oglab01g21050.2 0.003 0.711 0.708 0.005 0.995	1<br>Obart01g25070.1 0.284 0.987 0.703 0.125 0.979<br>Obart03g33060.1 0.003 0.227 0.224 0.013 0.961	1<br>Osjap01g29220.1 0.284 0.987 0.703 0.12 0.979<br>Osjap03g35690.1 0.003 0.227 0.224 0.013 0.961	1<br>Osind01g30680.1 0.284 0.987 0.703 0.12 0.979<br>Osind03g36920.1 0.003 0.227 0.224 0.013 0.961	1<br>Orufi01g27940.1 0.284 0.987 0.703 0.12 0.979<br>Orufi03g34430.1 0.003 0.227 0.224 0.013 0.961	0<br>Olong_003693-R1 0.284 0.987 0.703 0.127 0.978	1<br>Opunc01g24980.1 0.3 0.987 0.687 0.174 0.975<br>Opunc03g30200.1 0.003 0.227 0.224 0.013 0.961	1<br>Obrac01g18810.1 0.258 0.99 0.732 0.103 0.996<br>Obrac03g24520.1 0.003 0.227 0.224 0.013 0.961	0	0
Obart01g26420.1	0	1<br>Obart01g26420.1 0.002 1 0.998 0.002 1<br>Obart01g26420.2 0.002 1 0.998 0.002 1	0	0	1<br>Orufi01g29390.1 0.002 1 0.998 0.002 1	0	0	0	0	0
	1	1	1	1	1	1	1	1	1	0

	Oglab01g22320.1 0.002 0.585 0.583 0.003 0.997<br>Oglab07g18060.1 0.588 0.907 0.319 0.202 0.904	Obart07g20970.1 0.588 0.828 0.24 0.185 0.779<br>Obart11g13020.1 0.002 0.574 0.572 0.006 1	Osjap01g30690.1 0.002 0.585 0.583 0.003 0.997<br>Osjap07g24440.1 0.588 0.907 0.319 0.16 0.718	Osind01g32110.1 0.002 0.585 0.583 0.003 0.997<br>Osind07g21300.1 0.588 0.907 0.319 0.159 0.713	Orufi07g21990.1 0.588 0.852 0.264 0.175 0.751<br>Orufi11g13800.1 0.002 0.574 0.572 0.006 1	Olong_006531-R1 0.588 0.907 0.319 0.176 0.786<br>Olong_023995-R1 0.002 0.585 0.583 0.005 0.995	Opunc01g26380.1 0.002 0.585 0.583 0.003 0.997<br>Opunc07g12980.1 0.588 0.878 0.29 0.158 0.699	Obrac01g20020.1 0.002 0.585 0.583 0.003 0.997<br>Obrac02g00620.1 0.59 0.83 0.24 0.16 0.42	Lperr01g22600.1 0.002 0.585 0.583 0.003 0.997<br>Lperr07g18200.1 0.587 0.852 0.265 0.16 0.7	AT1G52155.1 0.013 0.553 0.54 0.022 0.997<br>AT4G20140.1 0.579 0.82 0.241 0.155 0.649
Obart01g26420.2	0	1<br>Obart01g26420.1 0.002 1 0.998 0.002 1<br>Obart01g26420.2 0.002 1 0.998 0.002 1	0	0	1<br>Orufi01g29390.1 0.002 1 0.998 0.002 1	0	0	0	0	0
	1<br>Oglab01g22320.1 0.002 0.563 0.561 0.003 0.997<br>Oglab07g18060.1 0.566 0.902 0.336 0.202 0.904	1<br>Obart07g20970.1 0.566 0.819 0.253 0.185 0.779<br>Obart11g13020.1 0.002 0.551 0.549 0.006 1	1<br>Osjap01g30690.1 0.002 0.563 0.561 0.003 0.997<br>Osjap07g24440.1 0.566 0.902 0.336 0.16 0.718	1<br>Osind01g32110.1 0.002 0.563 0.561 0.003 0.997<br>Osind07g21300.1 0.566 0.902 0.336 0.159 0.713	1<br>Orufi07g21990.1 0.566 0.844 0.278 0.175 0.751<br>Orufi11g13800.1 0.002 0.551 0.549 0.006 1	1<br>Olong_006531-R1 0.566 0.902 0.336 0.176 0.786<br>Olong_023995-R1 0.002 0.563 0.561 0.005 0.995	1<br>Opunc01g26380.1 0.002 0.563 0.561 0.003 0.997<br>Opunc07g12980.1 0.564 0.871 0.307 0.134 0.699	1<br>Obrac01g20020.1 0.002 0.563 0.561 0.003 0.997<br>Obrac02g00620.1 0.568 0.82 0.252 0.16 0.42	0<br>Lperr01g22600.1 0.002 0.563 0.561 0.003 0.997<br>Lperr08g10350.1 0.564 0.768 0.204 0.191 0.711	0<br>AT1G52155.1 0.014 0.529 0.515 0.022 0.997<br>AT4G20140.1 0.556 0.81 0.254 0.155 0.649
Obart01g26930.1	0	1<br>Obart01g26930.1 0.001 1 0.999 0.001 1	0	0	0	0	0	0	0	0
	1<br>Oglab01g22800.1 0.001 0.395 0.394 0.002 1<br>Oglab01g22810.1 0.406 0.959 0.553 0.148 0.999	1<br>Obart03g32590.1 0.001 0.39 0.389 0.002 0.921<br>Obart07g24100.1 0.404 0.927 0.523 0.166 0.828	1<br>Osjap01g31200.1 0.001 0.395 0.394 0.002 1<br>Osjap01g31210.1 0.404 0.959 0.555 0.2 0.999	1<br>Osind01g32660.1 0.001 0.395 0.394 0.002 0.972<br>Osind01g32670.1 0.404 0.959 0.555 0.198 0.999	1<br>Orufi01g29940.1 0.001 0.395 0.394 0.002 1<br>Orufi07g25280.1 0.404 0.927 0.523 0.17 0.829	1<br>Olong_000370-R1 0.001 0.395 0.394 0.426 1<br>Olong_012653-R1 0.404 0.927 0.523 0.168 0.829	1<br>Opunc01g26880.1 0.404 0.962 0.558 0.076 1<br>Opunc03g31650.1 0.01 0.393 0.383 0.017 0.936	1<br>Obrac01g20460.1 0.404 0.959 0.555 0.01 0.998<br>Obrac03g24030.1 0.001 0.366 0.365 0.002 0.841	1<br>Lperr03g07390.1 0.099 0.378 0.279 0.169 0.842<br>Lperr06g07900.1 0.404 0.927 0.523 0.173 0.936	0<br>AT5G28740.1 0.404 0.927 0.523 0.149 0.83
Obart01g27860.1	0	1<br>Obart01g27860.1 0.001 1 0.999 0.001 1<br>Obart01g27860.2 0.001 1 0.999 0.001 1	0	0	0	0	0	0	1<br>Lperr01g24010.1 0.006 0.997 0.991 0.012 0.792<br>Lperr01g24010.2 0.006 0.997 0.991 0.012 0.788	0
	1<br>Oglab01g23700.4 0.001 0.743 0.742 0.001 0.999<br>Oglab08g19780.2 0.744 0.999 0.255 0.079 0.791	0<br>Obart05g24550.1 0.734 0.997 0.263 0.065 0.923	1<br>Osjap01g32230.6 0.001 0.743 0.742 0.001 0.999<br>Osjap08g22350.1 0.744 0.999 0.255 0.077 0.797	1<br>Osind01g33730.2 0.001 0.743 0.742 0.001 0.999<br>Osind08g24520.1 0.744 0.999 0.255 0.079 0.791	0<br>Orufi01g30920.4 0.001 0.743 0.742 0.001 0.999	1<br>Olong_024789-R1 0.001 0.743 0.742 0.001 0.999<br>Olong_025647-R1 0.744 0.999 0.255 0.083 0.778	1<br>Opunc01g27750.2 0.001 0.743 0.742 0.001 0.999<br>Opunc09g13460.1 0.744 0.999 0.255 0.081 0.783	1<br>Obrac01g21170.1 0.001 0.743 0.742 0.001 0.999<br>Obrac08g13970.1 0.744 0.999 0.255 0.077 0.793	0<br>Lperr09g11160.1 0.744 0.999 0.255 0.075 0.831	1<br>AT3G49400.1 0.001 0.743 0.742 0.001 0.999<br>AT4G39150.1 0.744 0.997 0.253 0.087 0.907
Obart01g27860.2	0	1<br>Obart01g27860.1 0.001 1 0.999 0.001 1<br>Obart01g27860.2 0.001 1 0.999 0.001 1	0	0	0	0	0	0	1<br>Lperr01g24010.1 0.006 0.997 0.991 0.012 0.792<br>Lperr01g24010.2 0.006 0.997 0.991 0.012 0.788	0
	1<br>Oglab01g23700.4 0.001 0.743 0.742 0.001 0.999<br>Oglab08g19780.2 0.744 0.999 0.255 0.079 0.791	0<br>Obart05g24550.1 0.734 0.997 0.263 0.065 0.923	1<br>Osjap01g32230.6 0.001 0.743 0.742 0.001 0.999<br>Osjap08g22350.1 0.744 0.999 0.255 0.077 0.797	1<br>Osind01g33730.2 0.001 0.743 0.742 0.001 0.999<br>Osind08g24520.1 0.744 0.999 0.255 0.079 0.791	0<br>Orufi01g30920.4 0.001 0.743 0.742 0.001 0.999	1<br>Olong_024789-R1 0.001 0.743 0.742 0.001 0.999<br>Olong_025647-R1 0.744 0.999 0.255 0.083 0.778	1<br>Opunc01g27750.2 0.001 0.743 0.742 0.001 0.999<br>Opunc09g13460.1 0.744 0.999 0.255 0.081 0.783	1<br>Obrac01g21170.1 0.001 0.743 0.742 0.001 0.999<br>Obrac08g13970.1 0.744 0.999 0.255 0.077 0.793	0<br>Lperr09g11160.1 0.744 0.999 0.255 0.075 0.831	1<br>AT3G49400.1 0.001 0.743 0.742 0.001 0.999<br>AT4G39150.1 0.744 0.997 0.253 0.087 0.907
Obart01g33610.1	1<br>Oglab01g28270.1 0.001 1 0.999 0.022 1	1<br>Obart01g33610.1 0.001 1 0.999 0.001 1	0	0	0	0	0	0	0	0
	1<br>Oglab03g01040.1 0.002 0.213 0.211 0.039 0.99<br>Oglab10g10080.1 0.361 0.998 0.637 0.138 0.999	0<br>Obart10g10470.1 0.361 0.998 0.637 0.121 0.999	1<br>Osjap01g38450.1 0.294 1 0.706 0.022 1<br>Osjap03g01290.1 0.002 0.218 0.216 0.023 0.185	1<br>Osind01g39980.1 0.258 1 0.742 0.117 1<br>Osind01g39990.1 0.001 0.213 0.212 0.095 0.991	1<br>Orufi01g36790.1 0.294 1 0.706 0.022 1<br>Orufi01g36800.1 0.001 0.213 0.212 0.095 0.991	0<br>Olong_007589-R1 0.361 0.998 0.637 0.19 0.999	1<br>Opunc01g32680.1 0.294 1 0.706 0.022 1<br>Opunc01g32690.1 0.001 0.213 0.212 0.1 0.991	1<br>Obrac01g25800.1 0.294 1 0.706 0.022 1<br>Obrac03g00890.1 0.002 0.221 0.219 0.007 0.192	1<br>Lperr01g29020.1 0.279 1 0.721 0.001 1<br>Lperr01g29030.1 0.001 0.213 0.212 0.091 0.991	1<br>AT1G69290.1 0.316 1 0.684 0.036 1<br>AT3G13230.1 0.005 0.213 0.208 0.102 1
Obart01g40120.2	1<br>Oglab01g34910.1 0.002 1 0.998 0.002 1	1<br>Obart01g40120.1 0.002 1 0.998 0.002 1<br>Obart01g40120.2 0.002 1 0.998 0.002 1	1<br>Osjap01g45670.1 0.002 1 0.998 0.003 1	1<br>Osind01g46990.1 0.002 1 0.998 0.002 1	1<br>Orufi01g43430.1 0.123 1 0.877 0.351 1	1<br>Olong_025215-R1 0.152 0.975 0.823 0.002 0.977	0	0	0	0

	1<br>Oglab01g34890.1 0.123 0.39 0.267 0.646 0.992<br>Oglab11g07730.1 0.439 0.993 0.554 0.021 0.416	0<br>Obart11g08370.2 0.377 0.993 0.616 0.022 0.446	1<br>Osjap01g45650.1 0.123 0.39 0.267 0.646 0.992<br>Osjap11g09760.2 0.439 0.993 0.554 0.021 0.416	1<br>Osind01g46870.1 0.002 0.285 0.283 0.007 0.986<br>Osind11g07730.1 0.453 0.993 0.54 0.002 0.401	0<br>Orufi01g43430.3 0.285 1 0.715 0.045 1	1<br>Olong_009328-R1 0.123 0.37 0.247 0.649 0.983<br>Olong_020581-R1 0.439 0.993 0.554 0.021 0.416	1<br>Opunc04g15200.1 0.123 0.37 0.247 0.687 0.984<br>Opunc11g08140.1 0.377 0.993 0.616 0.122 0.503	1<br>Obrac01g31030.2 0.07 0.381 0.311 0.454 0.952<br>Obrac11g05320.1 0.439 0.993 0.554 0.021 0.416	1<br>Lperr01g34610.3 0.123 0.39 0.267 0.645 0.992<br>Lperr11g07510.1 0.439 0.993 0.554 0.021 0.416	1<br>AT3G28450.1 0.439 0.998 0.559 0.023 0.443<br>AT5G16960.1 0.123 0.383 0.26 0.65 1
Obart01g40910.1	1<br>Oglab01g35730.1 0.005 1 0.995 0.005 1	1<br>Obart01g40910.1 0.005 1 0.995 0.005 1	0	0	0	0	0	0	0	0
	1<br>Oglab02g07520.1 0.475 1 0.525 0.255 1<br>Oglab07g00120.1 0.13 0.405 0.275 0.696 0.841	1<br>Obart02g07460.1 0.475 1 0.525 0.531 1<br>Obart08g06610.1 0.13 0.405 0.275 0.364 0.944	1<br>Osjap01g46570.1 0.41 1 0.59 0.156 1<br>Osjap10g12600.2 0.1 0.405 0.305 0.235 0.978	1<br>Osind01g47760.1 0.41 1 0.59 0.156 1<br>Osind08g01920.1 0.12 0.405 0.285 0.622 0.841	1<br>Orufi01g44230.1 0.41 1 0.59 0.156 1<br>Orufi08g02060.1 0.12 0.405 0.285 0.622 0.841	0<br>Olong_004144-R1 0.39 1 0.61 0.008 1	1<br>Opunc02g06610.1 0.475 1 0.525 0.245 1<br>Opunc08g01790.1 0.12 0.405 0.285 0.404 0.94	1<br>Obrac02g05500.1 0.475 1 0.525 0.234 1<br>Obrac03g27510.3 0.13 0.405 0.275 0.748 0.87	1<br>Lperr01g35480.1 0.475 1 0.525 0.228 1<br>Lperr03g31300.1 0.13 0.405 0.275 0.754 0.873	0<br>AT3G03740.1 0.13 0.405 0.275 0.662 0.781<br>AT3G27340.1 0.47 0.98 0.51 0.234 0.957
Obart01g43280.1	1<br>Oglab01g37970.1 0 0.999 0.999 0 0.999<br>Oglab01g37970.2 0 0.999 0.999 0 0.999<br>Oglab01g37970.3 0.028 0.999 0.971 0 0.999	1<br>Obart01g43280.1 0 1 1 0 1<br>Obart01g43280.2 0.028 1 0.972 0 1	1<br>Osjap01g49090.1 0.028 0.999 0.971 0.001 0.999	1<br>Osind01g50590.1 0.028 0.999 0.971 0.001 0.999	1<br>Orufi01g46760.2 0.026 0.999 0.973 0.001 0.999	1<br>Olong_026121-R2 0.026 0.886 0.86 0 1<br>Olong_026121-R3 0.028 0.886 0.858 0.001 1	1<br>Opunc01g42300.1 0.027 0.999 0.972 0.043 0.999	1<br>Obrac01g33850.1 0.027 0.896 0.869 0 0.979<br>Obrac01g33850.2 0.027 0.896 0.869 0 0.979<br>Obrac01g33850.3 0.027 0.896 0.869 0 0.999	0	0
	1<br>Oglab01g37960.1 0.438 1 0.562 0.009 0.999<br>Oglab01g37980.1 0.028 0.435 0.407 0.001 1	0<br>Obart01g43290.1 0.027 0.496 0.469 0.033 0.976	1<br>Osjap01g49070.1 0.447 1 0.553 0.002 1<br>Osjap01g49110.1 0.027 0.435 0.408 0.074 1	1<br>Osind01g50550.1 0.443 1 0.557 0.001 1<br>Osind02g04390.1 0.028 0.429 0.401 0.001 0.995	1<br>Orufi01g46740.1 0.446 1 0.554 0.013 1<br>Orufi11g06790.1 0.027 0.425 0.398 0.002 1	0<br>Olong_026121-R1 0.007 0.436 0.429 0.001 0.988	1<br>Opunc01g42270.1 0.433 1 0.567 0.005 1<br>Opunc11g06550.1 0.028 0.433 0.405 0 0.984	1<br>Obrac01g33820.1 0.446 1 0.554 0.008 1<br>Obrac02g02870.1 0.027 0.431 0.404 0.244 1	1<br>Lperr01g37690.1 0.445 1 0.555 0.022 1<br>Lperr11g05950.1 0.026 0.439 0.413 0 0.968	1<br>AT1G03220.1 0.446 0.999 0.553 0.067 0.993<br>AT3G14460.1 0.042 0.432 0.39 0.034 0.999
Obart02g00480.1	0	1<br>Obart02g00480.1 0.002 1 0.998 0.002 1	0	0	0	0	0	0	0	0
	1<br>Oglab01g12840.1 0.002 0.438 0.436 0.004 0.86<br>Oglab02g00410.1 0.458 1 0.542 0.069 1	1<br>Obart01g15210.1 0.002 0.438 0.436 0.004 0.86<br>Obart05g02080.1 0.458 0.91 0.452 0.062 0.875	1<br>Osjap01g17630.1 0.002 0.438 0.436 0.004 0.86<br>Osjap02g00500.2 0.471 0.91 0.439 0.068 0.877	1<br>Osind01g18080.1 0.002 0.438 0.436 0.004 0.86<br>Osind03g09080.1 0.45 0.91 0.46 0.144 0.919	1<br>Orufi01g16820.1 0.002 0.438 0.436 0.004 0.86<br>Orufi02g00500.5 0.471 0.91 0.439 0.075 0.757	1<br>Olong_016589-R3 0.458 0.91 0.452 0.126 0.885<br>Olong_022005-R1 0.002 0.438 0.436 0.004 0.95	1<br>Opunc02g00150.1 0.002 0.438 0.436 0.026 0.863<br>Opunc09g13920.1 0.444 0.908 0.464 0.008 0.876	1<br>Obrac02g00390.2 0.002 0.438 0.436 0.114 0.876<br>Obrac08g15590.5 0.498 0.91 0.412 0.138 0.797	1<br>Lperr01g13600.1 0.002 0.438 0.436 0.004 0.86<br>Lperr02g00490.1 0.458 0.912 0.454 0.091 0.863	0<br>AT5G07090.1 0.002 0.438 0.436 0.004 0.87
Obart02g01820.1	0	1<br>Obart02g01820.1 0.001 1 0.999 0.001 1	0	0	0	0	0	0	0	0
	1<br>Oglab02g01740.1 0.341 1 0.659 0.041 1<br>Oglab02g01750.1 0.001 0.313 0.312 0.003 0.995	0<br>Obart10g00600.1 0.046 0.316 0.27 0.025 0.993	1<br>Osjap02g01820.1 0.322 1 0.678 0.028 1<br>Osjap02g01830.1 0.001 0.313 0.312 0.003 0.995	1<br>Osind02g02050.1 0.322 1 0.678 0.028 1<br>Osind02g02060.1 0.001 0.313 0.312 0.003 0.995	1<br>Orufi02g01810.1 0.322 1 0.678 0.028 1<br>Orufi02g01820.1 0.001 0.313 0.312 0.003 0.995	1<br>Olong_003544-R1 0.001 0.313 0.312 0.003 0.995<br>Olong_003545-R1 0.362 0.983 0.621 0.001 0.999	1<br>Opunc02g01290.1 0.341 0.983 0.642 0.043 0.999<br>Opunc02g01300.1 0.04 0.313 0.273 0.003 0.994	1<br>Obrac02g01430.2 0.341 0.983 0.642 0.089 0.999<br>Obrac02g01440.4 0.029 0.313 0.284 0.054 0.99	1<br>Lperr02g01630.1 0.341 0.983 0.642 0.045 0.999<br>Lperr02g01640.2 0.042 0.313 0.271 0.006 0.994	0<br>AT1G59960.1 0.051 0.323 0.272 0.028 0.963<br>AT1G73970.1 0.463 0.958 0.495 0.229 0.956
Obart02g06500.1	0	1<br>Obart02g06500.1 0.001 1 0.999 0.001 1	0	0	0	0	0	0	0	0
	1<br>Oglab02g06600.1 0.307 1 0.693 0.033 1<br>Oglab02g06610.1 0.003 0.289 0.286 0.172 0.979	0<br>Obart06g04490.1 0.003 0.274 0.271 0.196 0.978	1<br>Osjap02g06980.1 0.307 1 0.693 0.033 1<br>Osjap02g06990.1 0.003 0.289 0.286 0.172 0.979	1<br>Osind02g07300.1 0.312 1 0.688 0.001 1<br>Osind02g07310.1 0.003 0.289 0.286 0.172 0.979	1<br>Orufi02g06640.1 0.307 1 0.693 0.033 1<br>Orufi02g06650.1 0.003 0.289 0.286 0.172 0.979	1<br>Olong_019072-R1 0.321 1 0.679 0.001 1<br>Olong_027167-R1 0.001 0.289 0.288 0.003 0.975	1<br>Opunc02g05730.1 0.307 0.998 0.691 0.034 1<br>Opunc02g05740.1 0.003 0.289 0.286 0.166 0.944	1<br>Obrac02g04810.1 0.313 1 0.687 0.034 1<br>Obrac02g04830.1 0.003 0.289 0.286 0.172 0.979	1<br>Lperr02g05650.1 0.307 0.999 0.692 0.041 0.971<br>Lperr02g05670.1 0.003 0.289 0.286 0.175 0.979	1<br>AT3G24550.1 0.003 0.277 0.274 0.466 0.903<br>AT4G32190.1 0.302 0.997 0.695 0.024 0.987
Obart02g11640.1	0	1<br>Obart02g11640.1 0.001 1 0.999 0.001 1	0	0	0	0	0	0	0	0
	1	1	1	1	1	1	1	1	0	1

	Oglab02g10250.1 0.001 0.476 0.475 0.002 0.996<br>Oglab02g11670.1 0.483 1 0.517 0.002 1	Obart02g10250.1 0.001 0.476 0.475 0.002 0.996<br>Obart03g04220.1 0.479 1 0.521 0.048 0.998	Osjap02g11160.1 0.001 0.476 0.475 0.002 0.996<br>Osjap03g04250.1 0.479 0.999 0.52 0.067 0.996	Osind02g12980.1 0.497 1 0.503 0.03 1<br>Osind02g13000.1 0.001 0.476 0.475 0.002 1	Orufi02g10270.1 0.001 0.476 0.475 0.002 0.996<br>Orufi02g11670.1 0.487 1 0.513 0.012 1	Olong_009803-R1 0.479 1 0.521 0.002 0.998<br>Olong_026120-R1 0.001 0.476 0.475 0.002 0.996	Opunc02g10280.1 0.479 1 0.521 0.002 1<br>Opunc02g10300.1 0.001 0.475 0.474 0.002 0.998	Obrac02g07710.1 0.001 0.476 0.475 0.002 1<br>Obrac03g03340.1 0.52 1 0.48 0.136 0.998	Lperr10g08090.1 0.386 1 0.614 0.063 0.998	AT1G03890.1 0.001 0.457 0.456 0.002 0.991<br>AT2G37640.1 0.486 0.996 0.51 0.011 0.985
Obart02g11910.1	0	1<br>Obart02g11910.1 0.001 1 0.999 0.001 1	0	0	0	0	0	0	0	0
	1<br>Oglab02g11870.3 0.001 0.616 0.615 0.018 0.991<br>Oglab02g11880.1 0.649 0.996 0.347 0.001 0.282	1<br>Obart11g09030.1 0.004 0.616 0.612 0.054 0.994<br>Obart11g20370.3 0.649 0.995 0.346 0.001 0.275	1<br>Osjap02g13100.1 0.649 0.996 0.347 0.001 0.242<br>Osjap02g13110.1 0.001 0.616 0.615 0.002 0.998	1<br>Osind02g13300.1 0.001 0.616 0.615 0.2 0.993<br>Osind12g16510.1 0.641 0.996 0.355 0.002 0.632	1<br>Orufi02g11960.1 0.001 0.616 0.615 0.172 0.992<br>Orufi08g05130.1 0.624 0.974 0.35 0 0.595	1<br>Olong_006337-R1 0.004 0.616 0.612 0.108 0.998<br>Olong_026902-R1 0.647 0.996 0.349 0.073 0.821	1<br>Opunc02g10610.1 0.649 0.987 0.338 0.004 0.983<br>Opunc02g10620.1 0.001 0.616 0.615 0.042 0.998	0<br>Obrac12g13260.3 0.001 0.619 0.618 0.002 0.998	1<br>Lperr11g07730.1 0.649 0.996 0.347 0.001 0.325<br>Lperr11g07990.1 0.001 0.616 0.615 0.002 0.988	1<br>AT1G50180.1 0.649 0.978 0.329 0.001 0.285<br>AT4G26510.1 0.001 0.596 0.595 0.002 0.998
Obart02g23460.2	1<br>Oglab02g22130.2 0.001 1 0.999 0.001 1	1<br>Obart02g23460.2 0.001 1 0.999 0.001 1	1<br>Osjap02g26750.1 0.001 0.969 0.968 0.001 0.994<br>Osjap02g26750.2 0.001 0.969 0.968 0.001 0.994<br>Osjap02g26750.4 0.001 0.969 0.968 0.001 0.799	1<br>Osind02g26640.2 0.001 1 0.999 0.001 1	1<br>Orufi02g24900.1 0.011 0.974 0.963 0.06 0.923<br>Orufi02g24900.3 0.001 0.974 0.973 0.001 0.857<br>Orufi02g24900.4 0.001 0.974 0.973 0.001 0.856	0	1<br>Opunc02g21410.1 0.011 0.999 0.988 0.116 0.996<br>Opunc02g21410.2 0.011 0.999 0.988 0.114 0.996<br>Opunc02g21410.3 0.011 0.999 0.988 0.115 0.996<br>Opunc02g21410.4 0.011 0.999 0.988 0.114 0.996	0	1<br>Lperr02g18970.1 0.011 0.997 0.986 0.032 0.996	0
	1<br>Oglab02g21950.1 0.475 0.999 0.524 0.073 0.995<br>Oglab02g22110.2 0.011 0.459 0.448 0.532 0.98	1<br>Obart02g23290.1 0.475 0.999 0.524 0.029 0.999<br>Obart02g23440.1 0.011 0.459 0.448 0.492 0.981	1<br>Osjap02g26590.1 0.475 0.999 0.524 0.033 0.999<br>Osjap02g26750.5 0.011 0.459 0.448 0.238 0.973	1<br>Osind02g26450.1 0.475 0.999 0.524 0.025 0.997<br>Osind02g26620.1 0.011 0.459 0.448 0.495 0.981	1<br>Orufi02g24730.1 0.475 0.994 0.519 0.031 0.996<br>Orufi02g24880.2 0.011 0.459 0.448 0.18 0.97	0<br>Olong_024345-R1 0.011 0.474 0.463 0.442 0.991	1<br>Opunc02g21230.1 0.479 0.976 0.497 0.003 0.934<br>Opunc02g21240.1 0.011 0.461 0.45 0.121 0.972	1<br>Obrac02g16670.1 0.549 0.973 0.424 0.076 0.995<br>Obrac02g16700.1 0.011 0.461 0.45 0.114 0.947	1<br>Lperr02g18740.1 0.485 0.997 0.512 0.056 0.991<br>Lperr02g19120.1 0.011 0.484 0.473 0.183 0.978	1<br>AT4G31860.1 0.014 0.45 0.436 0.123 0.989<br>AT5G43080.1 0.626 1 0.374 0.194 0.932
Obart02g24930.1	0	1<br>Obart02g24930.1 0.007 1 0.993 0.007 1	0	0	0	0	0	0	0	0
	1<br>Oglab02g23510.1 0.007 0.345 0.338 0.546 0.803<br>Oglab04g03020.1 0.432 1 0.568 0.013 1	0<br>Obart05g18450.1 0.396 1 0.604 0.315 1	1<br>Osjap02g28260.2 0.007 0.345 0.338 0.012 0.571<br>Osjap04g22620.1 0.432 1 0.568 0.012 1	1<br>Osind02g28120.1 0.007 0.338 0.331 0.619 0.906<br>Osind04g04600.1 0.338 1 0.662 0.189 1	1<br>Orufi02g26240.6 0.007 0.345 0.338 0.012 0.571<br>Orufi04g20540.1 0.432 1 0.568 0.012 1	1<br>Olong_000623-R2 0.007 0.345 0.338 0.589 0.822<br>Olong_023182-R1 0.432 1 0.568 0.012 1	0<br>Opunc04g03300.1 0.432 1 0.568 0.013 1	0<br>Obrac02g17870.2 0.432 1 0.568 0.013 1	0<br>Lperr01g37400.3 0.252 1 0.748 0.758 0.84	0<br>AT2G31953.1 0.432 1 0.568 0.012 0.919
Obart02g31950.1	0	1<br>Obart02g31950.1 0.001 1 0.999 0.001 1<br>Obart02g31950.2 0.001 1 0.999 0.001 1	0	0	0	0	0	0	1<br>Lperr02g26150.1 0.001 1 0.999 0.001 1<br>Lperr02g26150.2 0.001 1 0.999 0.001 1<br>Lperr02g26150.3 0.001 1 0.999 0.002 1	0
	1<br>Oglab02g29520.1 0.426 1 0.574 0.039 1<br>Oglab02g29530.1 0.001 0.374 0.373 0.003 1	0<br>Obart06g27910.1 0.001 0.371 0.37 0.036 0.987	1<br>Osjap02g36120.2 0.426 0.933 0.507 0.039 0.865<br>Osjap02g36130.1 0.001 0.374 0.373 0.003 1	1<br>Osind02g35590.1 0.426 1 0.574 0.039 1<br>Osind02g35610.1 0.001 0.374 0.373 0.003 1	1<br>Orufi02g33640.1 0.426 0.933 0.507 0.039 0.865<br>Orufi02g33650.1 0.001 0.374 0.373 0.003 1	1<br>Olong_014377-R1 0.426 1 0.574 0.04 1<br>Olong_014378-R1 0.001 0.374 0.373 0.003 1	1<br>Opunc02g17770.1 0.001 0.374 0.373 0.003 1<br>Opunc02g17780.1 0.427 1 0.573 0.109 1	1<br>Obrac02g23310.1 0.436 1 0.564 0.063 1<br>Obrac02g23320.1 0.001 0.374 0.373 0.003 1	0<br>Lperr06g23320.1 0.002 0.402 0.4 0.006 0.997	1<br>AT4G04180.1 0.478 0.993 0.515 0.123 0.977<br>AT5G08460.1 0.001 0.371 0.37 0.031 0.992
Obart02g31950.2	0	1<br>Obart02g31950.1 0.001 1 0.999 0.001 1<br>Obart02g31950.2 0.001 1 0.999 0.001 1	0	0	0	0	0	0	1<br>Lperr02g26150.1 0.001 1 0.999 0.001 1<br>Lperr02g26150.2 0.001 1 0.999 0.001 1<br>Lperr02g26150.3 0.001 1 0.999 0.002 1	0
	1<br>Oglab02g29520.1 0.428 1 0.572 0.039 1<br>Oglab02g29530.1 0.001 0.375 0.374 0.003 1	0<br>Obart06g27910.1 0.001 0.372 0.371 0.036 0.987	1<br>Osjap02g36120.2 0.428 0.932 0.504 0.039 0.865<br>Osjap02g36130.1 0.001 0.375 0.374 0.003 1	1<br>Osind02g35590.1 0.428 1 0.572 0.039 1<br>Osind02g35610.1 0.001 0.375 0.374 0.003 1	1<br>Orufi02g33640.1 0.428 0.932 0.504 0.039 0.865<br>Orufi02g33650.1 0.001 0.375 0.374 0.003 1	1<br>Olong_014377-R1 0.428 1 0.572 0.04 1<br>Olong_014378-R1 0.001 0.375 0.374 0.003 1	1<br>Opunc02g17770.1 0.001 0.375 0.374 0.003 1<br>Opunc02g17780.1 0.429 1 0.571 0.109 1	1<br>Obrac02g23310.1 0.438 1 0.562 0.063 1<br>Obrac02g23320.1 0.001 0.375 0.374 0.003 1	0<br>Lperr06g23320.1 0.002 0.404 0.402 0.006 0.997	1<br>AT4G04180.1 0.48 0.993 0.513 0.123 0.977<br>AT5G08460.1 0.001 0.372 0.371 0.031 0.992

Obart02g38050.2	1<br>Oglab02g35180.1 0.001 0.976 0.975 0.001 0.919<br>Oglab02g35180.2 0.001 1 0.999 0.001 1	1<br>Obart02g38050.1 0.001 0.976 0.975 0.001 0.919<br>Obart02g38050.2 0.001 1 0.999 0.001 1	0	1<br>Osind02g41950.1 0.071 0.95 0.879 0.368 0.955	1<br>Orufi02g39760.1 0.001 1 0.999 0.001 0.95<br>Orufi02g39760.2 0.001 1 0.999 0.001 1	0	0	0	0	0
	1<br>Oglab02g35180.3 0.001 0.231 0.23 0.005 0.885<br>Oglab07g10660.4 0.263 0.996 0.733 0.085 0.801	0<br>Obart03g04840.1 0.32 0.974 0.654 0.007 0.995	1<br>Osjap05g10980.2 0.237 0.997 0.76 0.066 0.962<br>Osjap05g11860.1 0.001 0.209 0.208 0.078 0.911	1<br>Osind05g11850.1 0.237 0.997 0.76 0.066 0.974<br>Osind06g11870.1 0.001 0.209 0.208 0.078 0.911	1<br>Orufi02g39760.3 0.001 0.231 0.23 0.005 0.886<br>Orufi05g10490.3 0.237 0.949 0.712 0.064 0.962	1<br>Olong_016606-R1 0.31 0.996 0.686 0.008 0.889<br>Olong_027192-R1 0.001 0.209 0.208 0.078 0.911	0<br>Opunc02g35330.1 0.208 1 0.792 0.014 1	1<br>Obrac05g06630.3 0.242 0.971 0.729 0.081 0.95<br>Obrac06g07800.1 0.001 0.209 0.208 0.078 0.911	0<br>Lperr02g31540.1 0.208 1 0.792 0.017 1	0<br>AT5G38730.1 0.202 0.99 0.788 0.017 0.998
Obart03g00550.1	1<br>Oglab03g00390.1 0.001 0.996 0.995 0.001 0.994	1<br>Obart03g00550.1 0.001 1 0.999 0.001 1	1<br>Osjap03g00680.1 0.001 0.996 0.995 0.001 0.994<br>Osjap03g00680.2 0.001 0.996 0.995 0.001 0.994<br>Osjap03g00680.3 0.001 0.996 0.995 0.001 0.994	1<br>Osind03g00740.1 0.001 1 0.999 0.001 1	1<br>Orufi03g12660.1 0.001 0.996 0.995 0.001 0.994<br>Orufi03g12660.2 0.001 0.996 0.995 0.001 0.994	0	0	0	0	0
	1<br>Oglab02g34920.1 0.061 0.675 0.614 0.131 1<br>Oglab04g12740.1 0.715 0.996 0.281 0.411 0.989	1<br>Obart02g37790.1 0.061 0.645 0.584 0.133 0.977<br>Obart12g06510.1 0.673 0.996 0.323 0.052 0.984	1<br>Osjap02g42090.1 0.052 0.675 0.623 0.065 1<br>Osjap12g08580.1 0.706 0.996 0.29 0.011 0.977	1<br>Osind02g41670.1 0.061 0.675 0.614 0.131 1<br>Osind03g02220.1 0.695 0.996 0.301 0.015 0.982	1<br>Orufi02g39350.1 0.061 0.675 0.614 0.131 1<br>Orufi09g02220.2 0.742 0.996 0.254 0.095 0.975	0<br>Olong_013175-R1 0.27 0.904 0.634 0.002 0.961	1<br>Opunc03g00470.1 0.001 0.78 0.779 0.002 1<br>Opunc03g03130.2 0.78 0.996 0.216 0.443 0.984	0<br>Obrac02g27970.1 0.136 0.675 0.539 0.105 1<br>Obrac05g17660.1 0.747 0.995 0.248 0.427 0.997	1<br>Lperr03g00570.1 0.001 0.675 0.674 0.002 1<br>Lperr04g11360.1 0.748 0.996 0.248 0.415 0.99	0<br>AT5G04460.1 0.018 0.675 0.657 0.081 1
Obart03g01190.1	0	1<br>Obart03g01190.1 0.002 1 0.998 0.002 1	0	0	0	0	0	0	0	0
	1<br>Oglab03g01040.1 0.694 0.993 0.299 0.005 0.796<br>Oglab03g01050.1 0.002 0.611 0.609 0.002 0.983	1<br>Obart01g33610.1 0.706 0.993 0.287 0.002 0.17<br>Obart03g14810.2 0.061 0.593 0.532 0.198 0.987	1<br>Osjap03g01290.1 0.655 0.993 0.338 0.001 0.15<br>Osjap03g01300.2 0.002 0.633 0.631 0.002 0.996	1<br>Osind03g01350.1 0.694 0.993 0.299 0.005 0.796<br>Osind07g20900.1 0.035 0.606 0.571 0.217 0.955	1<br>Orufi03g00970.1 0.655 0.993 0.338 0.004 0.815<br>Orufi03g00980.1 0.057 0.611 0.554 0.002 0.982	0<br>Olong_020901-R3 0.066 0.593 0.527 0.009 0.744	1<br>Opunc03g01090.1 0.655 0.993 0.338 0.004 0.814<br>Opunc03g01100.1 0.013 0.611 0.598 0.01 0.983	1<br>Obrac02g06700.4 0.011 0.593 0.582 0.435 0.868<br>Obrac03g00890.1 0.694 0.993 0.299 0.001 0.149	1<br>Lperr03g01130.1 0.694 0.993 0.299 0.001 0.131<br>Lperr03g01140.1 0.009 0.617 0.608 0.005 0.858	1<br>AT3G13230.1 0.712 0.993 0.281 0.102 0.814<br>AT3G53170.1 0.004 0.593 0.589 0.064 0.772
Obart03g02800.1	0	1<br>Obart03g02800.1 0.001 1 0.999 0.001 1	0	0	0	0	0	0	0	0
	1<br>Oglab03g02720.1 0.589 1 0.411 0.002 1<br>Oglab03g02730.1 0.01 0.583 0.573 0.013 0.989	1<br>Obart03g02810.1 0.589 0.978 0.389 0.002 0.993<br>Obart03g02820.1 0.01 0.583 0.573 0.013 0.989	1<br>Osjap03g02800.1 0.589 0.978 0.389 0.005 0.968<br>Osjap03g02820.1 0.01 0.583 0.573 0.013 0.989	1<br>Osind03g02900.1 0.01 0.583 0.573 0.013 0.989<br>Osind10g16860.1 0.589 0.978 0.389 0.004 0.992	1<br>Orufi01g17470.2 0.591 0.996 0.405 0.002 0.996<br>Orufi03g02520.1 0.017 0.583 0.566 0.025 0.989	1<br>Olong_005980-R1 0.589 0.978 0.389 0.002 0.989<br>Olong_005981-R1 0.017 0.583 0.566 0.03 0.987	1<br>Opunc03g02530.1 0.589 0.979 0.39 0.002 0.912<br>Opunc03g02550.1 0.017 0.583 0.566 0.02 0.989	1<br>Obrac03g02200.1 0.017 0.588 0.571 0.034 0.997<br>Obrac10g11140.1 0.589 0.979 0.39 0.003 0.993	1<br>Lperr03g02550.1 0.01 0.583 0.573 0.012 0.989<br>Lperr10g13340.1 0.589 0.987 0.398 0.004 0.996	1<br>AT2G17020.1 0.022 0.58 0.558 0.04 0.97<br>AT3G62760.1 0.592 0.983 0.391 0.014 1
Obart03g07090.1	0	1<br>Obart03g07090.1 0.001 1 0.999 0.001 1<br>Obart03g07090.2 0.001 1 0.999 0.001 1	0	0	0	0	0	0	0	0
	1<br>Oglab03g07150.1 0.045 0.61 0.565 0.002 0.995<br>Oglab03g07160.4 0.61 1 0.39 0.003 1	0<br>Obart03g07090.4 0.001 0.61 0.609 0.002 0.978	1<br>Osjap01g23040.3 0.61 1 0.39 0.003 1<br>Osjap03g07460.1 0.045 0.61 0.565 0.002 0.995	1<br>Osind03g07550.1 0.001 0.61 0.609 0.002 0.995<br>Osind03g07560.1 0.61 1 0.39 0.003 1	1<br>Orufi01g22080.2 0.61 1 0.39 0.002 1<br>Orufi03g07040.1 0.045 0.61 0.565 0.002 0.995	1<br>Olong_017037-R1 0.61 1 0.39 0.003 1<br>Olong_026139-R1 0.093 0.61 0.517 0.002 0.995	1<br>Opunc01g19950.1 0.61 1 0.39 0.003 1<br>Opunc03g06910.1 0.033 0.61 0.577 0.054 0.995	0<br>Obrac03g05750.1 0.055 0.634 0.579 0.023 0.991	1<br>Lperr03g06490.1 0.045 0.61 0.565 0.002 0.995<br>Lperr03g06500.1 0.61 1 0.39 0.08 1	1<br>AT1G75100.1 0.055 0.61 0.555 0.046 0.995<br>AT5G27470.1 0.61 0.988 0.378 0.002 0.967
Obart03g07090.2	0	1<br>Obart03g07090.1 0.001 1 0.999 0.001 1<br>Obart03g07090.2 0.001 1 0.999 0.001 1	0	0	0	0	0	0	0	0
	1<br>Oglab03g07150.1 0.045 0.61 0.565 0.002 0.995	0<br>Obart03g07090.4 0.001 0.61 0.609 0.002 0.978	1<br>Osjap01g23040.3 0.61 1 0.39 0.003 1	1<br>Osind03g07550.1 0.001 0.61 0.609 0.002 0.995	1<br>Orufi01g22080.2 0.61 1 0.39 0.002 1	1<br>Olong_017037-R1 0.61 1 0.39 0.003 1	1<br>Opunc01g19950.1 0.61 1 0.39 0.003 1	0<br>Obrac03g05750.1 0.055 0.634 0.579 0.023 0.991	1<br>Lperr03g06490.1 0.045 0.61 0.565 0.002 0.995	1<br>AT1G75100.1 0.055 0.61 0.555 0.046 0.995

	Oglab03g07160.4 0.61 1 0.39 0.003 1		Osjap03g07460.1 0.045 0.61 0.565 0.002 0.995	Osind03g07560.1 0.61 1 0.39 0.003 1	Orufi03g07040.1 0.045 0.61 0.565 0.002 0.995	Olong_026139-R1 0.093 0.61 0.517 0.002 0.995	Opunc03g06910.1 0.033 0.61 0.577 0.054 0.995		Lperr03g06500.1 0.61 1 0.39 0.08 1	AT5G27470.1 0.61 0.988 0.378 0.002 0.967
Obart03g07660.1	0	1<br>Obart03g07660.1 0.001 1 0.999 0.001 1	0	0	0	0	0	0	0	0
	1<br>Oglab03g07740.1 0.298 1 0.702 0.015 1<br>Oglab03g07760.1 0.001 0.275 0.274 0.003 0.994	1<br>Obart08g08450.1 0.001 0.267 0.266 0.154 0.956<br>Obart09g05350.1 0.373 0.998 0.625 0.123 0.988	1<br>Osjap03g08130.1 0.298 1 0.702 0.015 1<br>Osjap10g07700.1 0.001 0.267 0.266 0.203 0.959	1<br>Osind03g08180.1 0.329 1 0.671 0.001 1<br>Osind10g05560.1 0.001 0.267 0.266 0.003 0.945	1<br>Orufi03g07710.1 0.329 1 0.671 0.001 1<br>Orufi03g07730.2 0.001 0.275 0.274 0.003 0.994	1<br>Olong_012932-R1 0.001 0.275 0.274 0.003 0.994<br>Olong_012934-R1 0.329 1 0.671 0.001 1	1<br>Opunc01g43280.1 0.379 0.998 0.619 0.226 0.944<br>Opunc03g07460.1 0.001 0.275 0.274 0.003 0.994	1<br>Obrac03g06230.1 0.329 1 0.671 0.001 1<br>Obrac10g03970.1 0.001 0.277 0.276 0.003 0.94	1<br>Lperr03g06980.1 0.329 1 0.671 0.001 1<br>Lperr10g04560.2 0.001 0.275 0.274 0.003 0.976	0<br>AT4G27190.1 0.347 0.997 0.65 0.122 0.982
Obart03g09160.3	1<br>Oglab03g09270.1 0 1 1 0.001 1<br>Oglab03g09270.2 0 1 1 0.001 1	1<br>Obart03g09160.1 0 1 1 0 1<br>Obart03g09160.2 0 1 1 0 1<br>Obart03g09160.3 0 1 1 0 1	1<br>Osjap03g09700.1 0 1 1 0.046 1<br>Osjap03g09700.2 0 1 1 0.045 1	1<br>Osind03g09660.1 0 1 1 0.044 1	1<br>Orufi03g09320.1 0 1 1 0.045 1<br>Orufi03g09320.2 0 1 1 0.046 1<br>Orufi03g09320.3 0 1 1 0.047 1<br>Orufi03g09320.4 0 1 1 0.048 1	1<br>Olong_002353-R1 0.021 1 0.979 0.001 1	1<br>Opunc03g08800.1 0.028 1 0.972 0.026 1<br>Opunc03g08800.2 0.028 1 0.972 0.026 1<br>Opunc03g08800.3 0.028 1 0.972 0.027 1	0	0	0
	1<br>Oglab10g00260.1 0.578 0.988 0.41 0.179 0.96<br>Oglab10g00270.1 0.069 0.469 0.4 0.072 1	1<br>Obart10g00240.1 0.578 0.988 0.41 0.179 0.96<br>Obart10g00250.1 0.069 0.469 0.4 0.072 1	1<br>Osjap05g08590.1 0.095 0.459 0.364 0.436 0.995<br>Osjap06g17700.1 0.55 0.988 0.438 0.321 0.983	1<br>Osind01g21860.1 0.069 0.469 0.4 0.075 1<br>Osind06g17200.1 0.55 0.988 0.438 0.318 0.983	1<br>Orufi10g00220.1 0.578 0.988 0.41 0.171 0.96<br>Orufi10g00230.1 0.069 0.469 0.4 0.074 1	1<br>Olong_008994-R1 0.069 0.469 0.4 0.07 0.999<br>Olong_023066-R1 0.581 0.995 0.414 0.021 0.97	0<br>Opunc03g08800.7 0.349 1 0.651 0.004 1	1<br>Obrac03g07350.1 0.548 1 0.452 0.114 1<br>Obrac10g00260.1 0.092 0.469 0.377 0.03 0.999	1<br>Lperr01g01500.1 0.082 0.471 0.389 0.001 1<br>Lperr03g08210.1 0.546 1 0.454 0.133 1	1<br>AT1G69770.1 0.089 0.459 0.37 0.027 0.988<br>AT4G34440.1 0.525 0.992 0.467 0.122 0.975
Obart03g10140.1	0	1<br>Obart03g10140.1 0.001 1 0.999 0.001 1	0	0	0	0	0	0	0	0
	1<br>Oglab03g10120.1 0.001 0.621 0.62 0.204 0.905<br>Oglab03g10130.1 0.621 1 0.379 0.15 1	0<br>Obart03g10140.2 0.001 0.621 0.62 0.002 0.881	1<br>Osjap03g10880.1 0.001 0.621 0.62 0.204 0.905<br>Osjap10g01900.1 0.621 1 0.379 0.143 1	1<br>Osind03g10780.1 0.001 0.448 0.447 0.193 0.979<br>Osind03g10790.1 0.621 1 0.379 0.145 1	1<br>Orufi03g10350.1 0.001 0.621 0.62 0.204 0.905<br>Orufi10g01680.1 0.621 1 0.379 0.143 1	1<br>Olong_000838-R1 0.02 0.621 0.601 0.002 0.868<br>Olong_000839-R1 0.621 1 0.379 0.071 1	1<br>Opunc03g09740.1 0.001 0.623 0.622 0.132 0.9<br>Opunc03g09750.2 0.636 1 0.364 0.076 1	1<br>Obrac03g08150.1 0.001 0.621 0.62 0.133 0.899<br>Obrac03g08160.1 0.621 1 0.379 0.142 1	1<br>Lperr03g09060.1 0.001 0.621 0.62 0.133 0.896<br>Lperr03g09070.3 0.621 1 0.379 0.141 1	1<br>AT5G64813.1 0.621 1 0.379 0.129 1<br>AT5G66130.1 0.005 0.615 0.61 0.115 0.883
Obart03g10480.1	0	1<br>Obart03g10480.1 0.004 1 0.996 0.004 1	1<br>Osjap03g11290.1 0.004 1 0.996 0.004 1	0	0	0	0	0	0	0
	1<br>Oglab01g01190.1 0.534 0.996 0.462 0.656 0.959<br>Oglab03g10450.1 0.004 0.509 0.505 0.007 1	1<br>Obart01g05330.1 0.495 0.996 0.501 0.64 0.945<br>Obart03g10500.1 0.004 0.305 0.301 0.011 0.989	0<br>Osjap01g06090.1 0.495 0.996 0.501 0.64 0.945<br>Osjap03g11310.1 0.004 0.283 0.279 0.005 0.398	1<br>Osind03g11120.1 0.004 0.509 0.505 0.007 1<br>Osind03g11140.1 0.527 1 0.473 0.668 0.954	1<br>Orufi03g10700.1 0.004 0.509 0.505 0.007 1<br>Orufi03g10740.1 0.527 1 0.473 0.664 0.947	1<br>Olong_001134-R1 0.53 0.828 0.298 0.477 0.77<br>Olong_001982-R1 0.004 0.509 0.505 0.007 1	1<br>Opunc03g10110.1 0.004 0.509 0.505 0.007 1<br>Opunc03g10130.1 0.527 1 0.473 0.686 0.985	0<br>Obrac03g08430.5 0.527 1 0.473 0.284 0.412	0<br>Lperr03g09350.4 0.004 0.509 0.505 0.005 1	0
Obart03g10890.2	1<br>Oglab03g10840.1 0.002 1 0.998 0.277 1<br>Oglab03g10840.4 0.002 1 0.998 0.26 1	1<br>Obart03g10890.2 0.002 1 0.998 0.002 1	1<br>Osjap03g11750.1 0.002 1 0.998 0.275 1<br>Osjap03g11750.2 0.002 0.991 0.989 0.257 0.987	0	0	0	0	0	0	0
	1<br>Oglab03g10840.2 0.002 0.601 0.599 0.271 0.735<br>Oglab09g00870.1 0.612 0.867 0.255 0.072 0.594	0<br>Obart03g10890.1 0.002 0.601 0.599 0.002 0.639	1<br>Osjap03g11750.3 0.002 0.601 0.599 0.35 0.953<br>Osjap05g12220.1 0.612 0.916 0.304 0.057 0.857	1<br>Osind03g11510.1 0.608 1 0.392 0.043 1<br>Osind03g11520.1 0.002 0.601 0.599 0.367 0.998	1<br>Orufi03g11130.1 0.608 1 0.392 0.043 1<br>Orufi03g11140.1 0.002 0.601 0.599 0.367 0.998	0<br>Olong_015766-R1 0.02 0.78 0.76 0.003 0.991	1<br>Opunc03g10480.1 0.61 0.953 0.343 0.045 0.829<br>Opunc03g10490.1 0.002 0.601 0.599 0.362 0.998	1<br>Obrac03g08730.1 0.608 0.927 0.319 0.052 0.981<br>Obrac03g08740.1 0.02 0.601 0.581 0.004 0.996	1<br>Lperr01g40130.1 0.612 0.929 0.317 0.063 0.769<br>Lperr03g09630.1 0.002 0.601 0.599 0.369 0.998	1<br>AT1G33520.1 0.002 0.601 0.599 0.284 0.998<br>AT4G36590.1 0.612 0.887 0.275 0.032 0.484
Obart03g12120.1	1<br>Oglab03g12120.1 0.001 1 0.999 0.001 1	1<br>Obart03g12120.1 0.001 1 0.999 0.001 1	1<br>Osjap03g13100.1 0.028 1 0.972 0.001 1	1<br>Osind03g12930.1 0.001 1 0.999 0.001 1	0	0	0	0	0	0
	1	1	1	1	1	1	1	1	1	1

	Oglab06g01620.1 0.642 0.998 0.356 0.212 0.912<br>Oglab12g00800.1 0.114 0.633 0.519 0.483 0.983	Obart04g15560.1 0.64 0.998 0.358 0.146 0.877<br>Obart11g01060.1 0.114 0.633 0.519 0.471 0.982	Osjap04g16240.1 0.642 0.998 0.356 0.067 0.921<br>Osjap12g01140.1 0.109 0.633 0.524 0.498 0.981	Osind04g14790.1 0.642 0.998 0.356 0.013 0.911<br>Osind12g01290.1 0.109 0.633 0.524 0.49 0.981	Orufi06g01460.3 0.642 0.998 0.356 0.212 0.912<br>Orufi12g01130.1 0.109 0.633 0.524 0.498 0.981	Olong_007714-R9 0.642 1 0.358 0.002 1<br>Olong_007715-R1 0.125 0.633 0.508 0.007 0.965	Opunc03g11680.1 0.028 0.633 0.605 0.001 0.971<br>Opunc04g11270.1 0.642 0.998 0.356 0.067 0.921	Obrac03g09720.1 0.025 0.633 0.608 0.036 0.971<br>Obrac04g09430.1 0.64 0.998 0.358 0.014 0.863	Lperr03g10720.1 0.028 0.633 0.605 0.001 0.934<br>Lperr03g17470.1 0.633 0.998 0.365 0.119 0.828	AT3G46740.1 0.122 0.633 0.511 0.158 0.971<br>AT3G53360.1 0.672 0.998 0.326 0.09 0.934
Obart03g20630.1	1<br>Oglab03g20370.1 0.073 1 0.927 0.087 1	1<br>Obart03g20630.1 0.001 1 0.999 0.001 1	1<br>Osjap03g22210.1 0.009 0.991 0.982 0.567 0.972	1<br>Osind03g22510.1 0.017 1 0.983 0.552 1	1<br>Orufi03g21250.1 0.185 0.995 0.81 0.02 0.991<br>Orufi03g21250.2 0.185 0.995 0.81 0.019 0.991<br>Orufi03g21250.3 0.189 0.995 0.806 0.021 0.99<br>Orufi03g21250.4 0.185 0.995 0.81 0.021 0.99	0	0	0	0	0
	1<br>Oglab05g22780.1 0.044 0.547 0.503 0.077 0.982<br>Oglab08g00090.1 0.557 0.931 0.374 0.117 0.976	1<br>Obart06g03480.2 0.051 0.556 0.505 0.002 0.826<br>Obart09g16880.1 0.557 0.931 0.374 0.091 0.964	1<br>Osjap05g27870.1 0.044 0.547 0.503 0.075 0.982<br>Osjap12g12160.2 0.556 0.931 0.375 0.083 0.997	1<br>Osind02g20400.1 0.607 0.931 0.324 0.137 0.985<br>Osind05g28150.1 0.044 0.547 0.503 0.075 0.982	1<br>Orufi05g26490.1 0.044 0.553 0.509 0.075 0.989<br>Orufi09g01530.2 0.557 0.931 0.374 0.087 0.965	1<br>Olong_013421-R1 0.051 0.556 0.505 0.002 0.877<br>Olong_028288-R1 0.607 1 0.393 0.003 1	1<br>Opunc05g00870.2 0.567 0.996 0.429 0.145 0.973<br>Opunc05g22250.1 0.044 0.547 0.503 0.075 0.982	0<br>Obrac09g04370.1 0.08 0.572 0.492 0.034 0.858	0<br>Lperr05g06040.4 0.557 0.992 0.435 0.084 0.556	0<br>AT1G07740.1 0.025 0.531 0.506 0.083 0.967
Obart03g27980.1	0	1<br>Obart03g27980.1 0.001 1 0.999 0.001 1<br>Obart03g27980.2 0.015 1 0.985 0.015 1	1<br>Osjap03g30460.1 0.021 1 0.979 0.085 1	1<br>Osind03g31500.1 0.021 1 0.979 0.094 1	1<br>Orufi03g29110.1 0.021 1 0.979 0.341 1	0	0	0	0	0
	1<br>Oglab03g27330.1 0.51 1 0.49 0.043 1<br>Oglab03g27340.1 0.008 0.476 0.468 0.008 0.997	1<br>Obart01g35640.1 0.509 0.998 0.489 0.063 0.982<br>Obart12g19300.1 0.019 0.475 0.456 0.01 0.99	1<br>Osjap03g31370.3 0.509 0.998 0.489 0.042 0.887<br>Osjap12g24400.2 0.019 0.475 0.456 0.012 0.988	1<br>Osind04g00720.3 0.509 0.999 0.49 0.064 0.919<br>Osind12g20440.1 0.019 0.475 0.456 0.01 0.99	1<br>Orufi03g30600.1 0.509 0.999 0.49 0.05 0.885<br>Orufi12g21550.1 0.019 0.475 0.456 0.01 0.99	1<br>Olong_006906-R1 0.04 0.468 0.428 0.071 1<br>Olong_007449-R1 0.509 0.999 0.49 0.065 0.909	1<br>Opunc03g25490.1 0.021 0.486 0.465 0.525 0.8<br>Opunc03g26940.1 0.509 0.999 0.49 0.052 0.885	1<br>Obrac03g20710.1 0.021 0.476 0.455 0.028 0.997<br>Obrac03g21410.1 0.574 0.995 0.421 0.09 0.91	1<br>Lperr03g23190.1 0.51 0.999 0.489 0.039 0.863<br>Lperr03g23200.1 0.009 0.476 0.467 0.008 0.997	0<br>AT5G36940.1 0.019 0.478 0.459 0.003 0.998
Obart03g27980.2	0	1<br>Obart03g27980.1 0.015 1 0.985 0.015 1<br>Obart03g27980.2 0.001 1 0.999 0.001 1	1<br>Osjap03g30460.1 0.021 1 0.979 0.085 1	1<br>Osind03g31500.1 0.021 1 0.979 0.094 1	1<br>Orufi03g29110.1 0.021 1 0.979 0.341 1	0	0	0	0	0
	1<br>Oglab03g27330.1 0.517 1 0.483 0.043 1<br>Oglab12g17460.4 0.018 0.483 0.465 0.012 0.996	1<br>Obart01g35640.1 0.516 0.998 0.482 0.063 0.982<br>Obart12g19300.1 0.018 0.483 0.465 0.01 0.99	1<br>Osjap03g31370.3 0.516 0.998 0.482 0.042 0.887<br>Osjap12g24400.2 0.018 0.483 0.465 0.012 0.988	1<br>Osind04g00720.3 0.516 0.999 0.483 0.064 0.919<br>Osind12g20440.1 0.018 0.483 0.465 0.01 0.99	1<br>Orufi03g30600.1 0.516 0.999 0.483 0.05 0.885<br>Orufi12g21550.1 0.018 0.483 0.465 0.01 0.99	1<br>Olong_002926-R1 0.024 0.484 0.46 0.002 0.996<br>Olong_007449-R1 0.516 0.999 0.483 0.065 0.909	1<br>Opunc03g25490.2 0.021 0.487 0.466 0.543 0.811<br>Opunc03g26940.1 0.516 0.999 0.483 0.052 0.885	1<br>Obrac03g20710.1 0.021 0.484 0.463 0.028 0.997<br>Obrac03g21410.1 0.58 0.995 0.415 0.09 0.91	1<br>Lperr03g23190.1 0.517 0.999 0.482 0.039 0.895<br>Lperr03g23200.1 0.008 0.484 0.476 0.008 0.997	0<br>AT5G36940.1 0.019 0.486 0.467 0.003 0.998
Obart03g31750.1	1<br>Oglab03g30830.1 0.046 1 0.954 0.001 1	1<br>Obart03g31750.1 0.001 1 0.999 0.001 1	1<br>Osjap03g34330.1 0.046 1 0.954 0.001 1	1<br>Osind03g35550.1 0.046 1 0.954 0.039 1	1<br>Orufi03g33060.1 0.046 1 0.954 0.001 1	0	0	0	0	0
	1<br>Oglab03g34460.1 0.646 0.997 0.351 0.016 0.993<br>Oglab10g04340.1 0.028 0.602 0.574 0.058 0.963	1<br>Obart03g36200.1 0.667 1 0.333 0.016 0.997<br>Obart10g04600.1 0.058 0.602 0.544 0.106 0.962	1<br>Osjap03g39110.1 0.667 1 0.333 0.036 1<br>Osjap10g05370.1 0.034 0.602 0.568 0.051 0.963	1<br>Osind01g46540.1 0.667 0.999 0.332 0.065 0.994<br>Osind10g03770.1 0.034 0.602 0.568 0.057 0.96	1<br>Orufi10g04850.1 0.034 0.602 0.568 0.054 0.963<br>Orufi11g12520.1 0.668 1 0.332 0.038 0.991	1<br>Olong_014796-R1 0.046 0.518 0.472 0.002 0.95<br>Olong_024872-R1 0.664 1 0.336 0.01 1	1<br>Opunc01g42030.1 0.667 1 0.333 0.28 0.872<br>Opunc03g28940.1 0.041 0.62 0.579 0.049 0.998	1<br>Obrac02g16280.1 0.662 0.982 0.32 0.063 0.949<br>Obrac03g23440.1 0.046 0.62 0.574 0.002 0.998	1<br>Lperr03g30520.1 0.66 1 0.34 0.003 0.997<br>Lperr10g03360.1 0.013 0.602 0.589 0.009 0.962	0<br>AT1G70730.3 0.046 0.618 0.572 0.118 0.995
Obart03g33760.1	1<br>Oglab03g32170.1 0.003 1 0.997 0.003 1<br>Oglab03g32170.2 0.003 1 0.997 0.003 1	1<br>Obart03g33760.1 0.003 1 0.997 0.003 1	1<br>Osjap03g36400.1 0.003 1 0.997 0.003 1	1<br>Osind03g37600.1 0.003 1 0.997 0.003 1	1<br>Orufi03g35150.1 0.003 1 0.997 0.003 1	1<br>Olong_002225-R1 0.003 1 0.997 0.136 1<br>Olong_002225-R2 0.003 1 0.997 0.003 1	1<br>Opunc03g30920.1 0.003 1 0.997 0.003 1	0	0	0
	0<br>Oglab05g19940.1 0.32 0.991 0.671 0.015 0.997	1<br>Obart01g34520.1 0.547 0.991 0.444 0.027 0.818<br>Obart03g04650.1 0.003 0.414 0.411 0.007 1	1<br>Osjap01g12980.2 0.547 0.979 0.432 0.032 0.961<br>Osjap03g04690.1 0.003 0.414 0.411 0.007 1	1<br>Osind03g04960.1 0.003 0.414 0.411 0.007 1<br>Osind12g02610.1 0.538 0.991 0.453 0.005 0.759	1<br>Orufi03g04350.1 0.003 0.414 0.411 0.007 1<br>Orufi05g22410.2 0.547 0.997 0.45 0.031 0.969	1<br>Olong_020510-R2 0.523 0.991 0.468 0.291 0.873<br>Olong_025165-R1 0.003 0.414 0.411 0.007 1	0<br>Opunc12g01920.1 0.526 0.973 0.447 0.663 0.947	0<br>Obrac03g25020.5 0.003 0.535 0.532 0.439 0.972	1<br>Lperr01g29830.2 0.532 0.991 0.459 0.203 0.858<br>Lperr03g28250.1 0.003 0.414 0.411 0.007 1	1<br>AT1G52740.1 0.003 0.414 0.411 0.007 1<br>AT4G14640.1 0.544 0.979 0.435 0.033 0.987

Obart03g38310.1	0	1<br>Obart03g38310.1 0.001 1 0.999 0.001 1	1<br>Osjap03g41450.1 0.076 1 0.924 0.097 1	0	0	0	0	1<br>Obrac03g28610.1 0.065 0.997 0.932 0.081 1<br>Obrac03g28610.2 0.065 0.997 0.932 0.081 1<br>Obrac03g28610.3 0.065 0.997 0.932 0.087 1	0	0
	1<br>Oglab03g36310.1 0.655 1 0.345 0.17 1<br>Oglab03g36320.1 0.064 0.655 0.591 0.137 0.994	0<br>Obart10g07730.1 0.78 0.995 0.215 0.458 0.989	0<br>Osjap10g09140.1 0.78 0.995 0.215 0.458 0.989	1<br>Osind03g42510.1 0.655 1 0.345 0.17 1<br>Osind03g42520.1 0.065 0.655 0.59 0.007 0.993	1<br>Orufi03g39950.1 0.655 1 0.345 0.17 1<br>Orufi03g39960.1 0.076 0.655 0.579 0.155 0.994	1<br>Olong_012011-R1 0.074 0.655 0.581 0.096 0.993<br>Olong_012012-R1 0.655 1 0.345 0.17 1	1<br>Opunc03g35470.1 0.655 1 0.345 0.17 1<br>Opunc03g35480.1 0.064 0.655 0.591 0.144 0.993	0<br>Obrac02g18090.1 0.731 0.997 0.266 0.034 0.998	1<br>Lperr03g32550.1 0.655 1 0.345 0.166 1<br>Lperr03g32560.1 0.072 0.655 0.583 0.144 0.994	0<br>AT1G75150.1 0.144 0.653 0.509 0.255 0.98<br>AT5G66720.1 0.748 0.997 0.249 0.321 1
Obart03g39770.1	0	1<br>Obart03g39770.1 0.002 1 0.998 0.002 1	0	0	0	0	0	0	0	0
	1<br>Oglab03g37820.1 0.363 1 0.637 0.276 1<br>Oglab09g02110.1 0.002 0.343 0.341 0.295 0.798	0<br>Obart09g01500.1 0.002 0.52 0.518 0.287 0.991	1<br>Osjap03g43150.1 0.363 1 0.637 0.276 1<br>Osjap09g02090.1 0.002 0.343 0.341 0.295 0.798	1<br>Osind03g44180.1 0.363 1 0.637 0.276 1<br>Osind09g02120.1 0.002 0.343 0.341 0.295 0.798	1<br>Orufi03g41570.1 0.363 1 0.637 0.276 1<br>Orufi09g02260.1 0.002 0.343 0.341 0.295 0.798	1<br>Olong_008554-R1 0.007 0.338 0.331 0.277 0.777<br>Olong_023902-R1 0.363 1 0.637 0.117 1	1<br>Opunc03g36930.2 0.363 1 0.637 0.292 1<br>Opunc09g01800.1 0.002 0.343 0.341 0.293 0.797	1<br>Obrac03g29930.1 0.363 1 0.637 0.177 1<br>Obrac06g09700.1 0.002 0.334 0.332 0.197 0.885	1<br>Lperr02g05900.1 0.002 0.343 0.341 0.156 0.883<br>Lperr03g33930.1 0.363 1 0.637 0.248 1	1<br>AT2G04570.1 0.002 0.336 0.334 0.283 0.786<br>AT3G55010.1 0.363 0.998 0.635 0.265 0.997
Obart03g41250.1	0	1<br>Obart03g41250.1 0.004 1 0.996 0.004 1	0	0	0	0	0	0	0	0
	1<br>Oglab03g39220.1 0.617 1 0.383 0.136 1<br>Oglab03g39230.1 0.004 0.571 0.567 0.006 0.962	0<br>Obart08g12230.2 0.165 0.481 0.316 0.191 0.832	1<br>Osjap03g44790.1 0.628 1 0.372 0.548 1<br>Osjap03g44800.1 0.004 0.571 0.567 0.006 0.962	1<br>Osind03g45850.1 0.628 1 0.372 0.548 1<br>Osind03g45860.1 0.004 0.571 0.567 0.006 0.962	1<br>Orufi03g43040.1 0.628 1 0.372 0.548 1<br>Orufi03g43050.1 0.004 0.571 0.567 0.006 0.962	0<br>Olong_023863-R1 0.162 0.462 0.3 0.047 0.774	1<br>Opunc03g38330.1 0.699 1 0.301 0.588 1<br>Opunc03g38340.1 0.004 0.571 0.567 0.006 0.962	1<br>Obrac03g31060.1 0.699 1 0.301 0.623 1<br>Obrac03g31070.1 0.004 0.571 0.567 0.006 0.962	1<br>Lperr03g35210.1 0.703 1 0.297 0.632 1<br>Lperr03g35220.1 0.004 0.571 0.567 0.006 0.968	0<br>AT1G73940.1 0.086 0.53 0.444 0.192 0.974<br>AT2G28440.1 0.703 0.977 0.274 0.701 0.981
Obart04g08760.3	0	1<br>Obart04g08760.1 0.001 1 0.999 0.001 1<br>Obart04g08760.2 0.001 1 0.999 0.001 1<br>Obart04g08760.3 0.001 1 0.999 0.001 1	0	0	0	0	0	0	0	0
	1<br>Oglab04g07860.1 0.642 1 0.358 0.134 0.995<br>Oglab05g17370.1 0.161 0.611 0.45 0.43 0.988	0<br>Obart03g10740.1 0.178 0.609 0.431 0.239 0.956<br>Obart04g08830.1 0.659 1 0.341 0.009 1	1<br>Osjap04g11380.1 0.003 0.609 0.606 0.001 0.962<br>Osjap04g11410.1 0.659 1 0.341 0.002 1	1<br>Osind04g09980.1 0.07 0.609 0.539 0.044 0.956<br>Osind04g10500.1 0.642 1 0.358 0.282 0.996	1<br>Orufi04g09970.1 0.07 0.609 0.539 0.042 0.958<br>Orufi04g10520.1 0.642 1 0.358 0.256 0.996	0<br>Olong_005665-R1 0.173 0.611 0.438 0.439 0.988<br>Olong_018692-R1 0.659 1 0.341 0.003 1	1<br>Opunc04g07340.1 0.031 0.609 0.578 0.045 0.96<br>Opunc04g07350.1 0.659 1 0.341 0.003 1	1<br>Obrac04g04890.2 0.023 0.609 0.586 0.039 0.959<br>Obrac04g05050.1 0.659 1 0.341 0.015 0.994	1<br>Lperr04g06080.1 0.145 0.609 0.464 0.09 0.965<br>Lperr09g11650.1 0.655 1 0.345 0.056 0.989	1<br>AT1G23740.1 0.663 0.999 0.336 0.194 0.99<br>AT2G36490.1 0.144 0.609 0.465 0.324 0.985
Obart04g09560.1	0	1<br>Obart04g09560.1 0.001 1 0.999 0.001 1	0	0	0	0	0	0	0	0
	1<br>Oglab02g16770.1 0.456 1 0.544 0.031 1<br>Oglab04g07880.1 0.001 0.432 0.431 0.002 0.931	1<br>Obart02g17390.1 0.456 1 0.544 0.031 1<br>Obart03g27200.1 0.058 0.44 0.382 0.321 0.866	1<br>Osjap04g12100.1 0.456 1 0.544 0.047 1<br>Osjap06g32310.5 0.054 0.456 0.402 0.325 0.956	1<br>Osind03g31020.1 0.058 0.44 0.382 0.321 0.866<br>Osind04g10510.1 0.456 1 0.544 0.002 1	1<br>Orufi03g28280.1 0.058 0.44 0.382 0.321 0.866<br>Orufi04g10530.1 0.449 1 0.551 0.008 1	1<br>Olong_002589-R1 0.058 0.44 0.382 0.263 0.855<br>Olong_009593-R1 0.456 1 0.544 0.034 1	1<br>Opunc03g24840.1 0.058 0.44 0.382 0.321 0.869<br>Opunc04g07630.1 0.455 1 0.545 0.046 1	1<br>Obrac03g20160.2 0.058 0.449 0.391 0.318 0.911<br>Obrac04g05060.1 0.451 1 0.549 0.05 1	1<br>Lperr03g22530.1 0.058 0.44 0.382 0.325 0.867<br>Lperr04g06480.1 0.455 1 0.545 0.045 1	1<br>AT1G30490.1 0.059 0.446 0.387 0.316 0.866<br>AT3G25150.2 0.456 0.989 0.533 0.201 0.961
Obart04g11380.1	0	1<br>Obart04g11380.1 0.001 1 0.999 0.001 1	0	0	0	0	0	0	1<br>Lperr04g08120.1 0.001 0.843 0.842 0.001 0.992<br>Lperr04g08120.2 0.001 0.843 0.842 0.001 0.992	0
	1<br>Oglab04g09690.1 0.001 0.26 0.259 0.002 1<br>Oglab04g09700.1 0.273 0.998 0.725 0.046 0.999	0<br>Obart09g12790.1 0.016 0.268 0.252 0.127 0.989	1<br>Osjap04g14100.1 0.273 0.998 0.725 0.051 0.973<br>Osjap09g13100.1 0.016 0.266 0.25 0.02 0.985	1<br>Osind04g12510.1 0.273 0.843 0.57 0.064 0.987<br>Osind09g13310.1 0.016 0.266 0.25 0.018 0.994	1<br>Orufi04g12550.1 0.001 0.26 0.259 0.002 1<br>Orufi04g12560.1 0.273 0.843 0.57 0.063 0.987	0<br>Olong_012592-R1 0.016 0.261 0.245 0.071 0.973<br>Olong_028740-R2 0.471 0.678 0.207 0.022 0.899	1<br>Opunc04g09410.1 0.001 0.26 0.259 0.002 0.998<br>Opunc04g09420.1 0.283 0.999 0.716 0.063 0.999	1<br>Obrac04g06150.1 0.282 1 0.718 0.065 1<br>Obrac09g07340.6 0.016 0.261 0.245 0.059 0.926	0<br>Lperr04g02100.1 0.003 0.26 0.257 0.01 1	0<br>AT1G76130.1 0.011 0.26 0.249 0.044 1<br>AT5G07400.1 0.411 0.811 0.4 0.335 0.944

Obart04g12680.3	0	1<br>Obart04g12680.1 0.001 1 0.999 0.001 1<br>Obart04g12680.2 0.001 1 0.999 0.001 1<br>Obart04g12680.3 0.001 1 0.999 0.001 1	1<br>Osjap04g15460.1 0.001 1 0.999 0.067 1<br>Osjap04g15460.2 0.001 1 0.999 0.068 1<br>Osjap04g15460.3 0.001 1 0.999 0.066 1<br>Osjap04g15460.4 0.001 1 0.999 0.066 1<br>Osjap04g15460.5 0.001 1 0.999 0.067 1<br>Osjap04g15460.6 0.001 1 0.999 0.068 1<br>Osjap04g15460.7 0.001 1 0.999 0.069 1<br>Osjap04g15460.8 0.001 1 0.999 0.068 1	0	0	0	1<br>Opunc04g07830.1 0.001 0.817 0.816 0.085 0.995	0	1<br>Lperr04g09380.1 0.001 0.817 0.816 0.097 0.994	0
	1<br>Oglab04g11000.3 0.329 1 0.671 0.001 1<br>Oglab04g11010.1 0.001 0.286 0.285 0.218 1	0<br>Obart02g19720.1 0.001 0.286 0.285 0.011 1	0<br>Osjap02g22530.1 0.006 0.276 0.27 0.225 0.958	1<br>Osind04g14040.2 0.329 1 0.671 0.001 1<br>Osind04g14050.1 0.001 0.286 0.285 0.218 1	1<br>Orufi04g13810.4 0.329 1 0.671 0.001 1<br>Orufi04g13820.1 0.001 0.286 0.285 0.207 1	1<br>Olong_025780-R1 0.001 0.286 0.285 0.003 1<br>Olong_025781-R3 0.299 0.817 0.518 0.024 0.991	1<br>Opunc04g10610.4 0.329 1 0.671 0.001 0.898<br>Opunc04g10620.1 0.001 0.286 0.285 0.186 1	0<br>Obrac04g07110.3 0.278 1 0.722 0.014 0.916	0<br>Lperr02g15820.1 0.001 0.286 0.285 0.2 1	0<br>AT1G43245.1 0.33 0.813 0.483 0.002 0.998<br>AT1G43580.1 0.001 0.286 0.285 0.164 1
Obart04g26710.1	0	1<br>Obart04g26710.1 0.001 1 0.999 0.001 1<br>Obart04g26710.2 0.001 1 0.999 0.001 1	1<br>Osjap04g30120.1 0.001 1 0.999 0.001 1<br>Osjap04g30120.3 0.001 1 0.999 0.001 1<br>Osjap04g30120.4 0.001 1 0.999 0.001 1	0	1<br>Orufi04g27870.1 0.001 1 0.999 0.001 1<br>Orufi04g27870.2 0.001 1 0.999 0.001 1	1<br>Olong_014711-R2 0.001 0.849 0.848 0.001 0.977	0	1<br>Obrac04g18040.3 0.001 1 0.999 0.001 1	0	0
	1<br>Oglab06g14840.1 0.01 0.632 0.622 0.059 0.882<br>Oglab11g19190.1 0.648 1 0.352 0.042 1	1<br>Obart05g21760.1 0.657 0.996 0.339 0.024 0.993<br>Obart10g13990.1 0.007 0.632 0.625 0.016 0.827	1<br>Osjap04g16240.1 0.007 0.623 0.616 0.07 0.895<br>Osjap05g24420.1 0.657 0.996 0.339 0.024 0.993	1<br>Osind04g29430.2 0.636 1 0.364 0.003 1<br>Osind10g13390.1 0.007 0.632 0.625 0.023 0.826	1<br>Orufi05g23020.1 0.657 0.996 0.339 0.024 0.993<br>Orufi10g14810.1 0.007 0.632 0.625 0.016 0.827	0<br>Olong_014711-R1 0.001 0.645 0.644 0.002 0.837	1<br>Opunc04g23700.2 0.633 1 0.367 0.003 1<br>Opunc05g09290.1 0.003 0.63 0.627 0.038 0.861	0<br>Obrac04g07720.1 0.007 0.633 0.626 0.076 0.934	1<br>Lperr07g00250.2 0.665 1 0.335 0.015 0.99<br>Lperr10g10450.1 0.007 0.632 0.625 0.009 0.826	1<br>AT1G03540.1 0.007 0.623 0.616 0.066 0.995<br>AT4G11290.1 0.643 1 0.357 0.012 1
Obart05g00520.1	0	1<br>Obart05g00520.1 0.001 1 0.999 0.001 1<br>Obart05g00520.2 0.001 0.995 0.994 0.001 0.999	1<br>Osjap05g00530.1 0.001 0.993 0.992 0.084 0.999<br>Osjap05g00530.2 0.001 0.993 0.992 0.103 0.999<br>Osjap05g00530.3 0.001 0.993 0.992 0.104 0.999<br>Osjap05g00530.4 0.001 0.993 0.992 0.091 0.999	1<br>Osind05g00510.1 0.049 0.993 0.944 0.001 0.999	1<br>Orufi05g00500.1 0.001 0.993 0.992 0.084 0.999	1<br>Olong_022405-R1 0.049 0.993 0.944 0.001 0.999	0	0	0	0
	1<br>Oglab05g00500.1 0.001 0.294 0.293 0.218 0.982<br>Oglab09g13820.1 0.322 0.993 0.671 0.064 0.998	1<br>Obart06g01490.1 0.017 0.293 0.276 0.2 0.982<br>Obart09g15270.1 0.34 0.993 0.653 0.029 0.998	1<br>Osjap02g39750.1 0.337 0.988 0.651 0.004 0.956<br>Osjap07g27300.1 0.003 0.291 0.288 0.116 0.834	1<br>Osind09g15830.1 0.34 0.993 0.653 0.029 0.998<br>Osind10g10530.3 0.001 0.336 0.335 0.088 0.826	1<br>Orufi07g24760.1 0.003 0.291 0.288 0.116 0.834<br>Orufi09g16480.1 0.34 0.993 0.653 0.029 0.998	1<br>Olong_018361-R1 0.34 0.993 0.653 0.012 0.998<br>Olong_020432-R1 0.017 0.325 0.308 0.115 0.666	1<br>Opunc05g00480.1 0.329 0.993 0.664 0.007 0.998<br>Opunc05g00490.1 0.001 0.294 0.293 0.228 0.982	1<br>Obrac02g14170.4 0.001 0.3 0.299 0.03 0.843<br>Obrac02g28140.1 0.342 0.989 0.647 0.039 0.973	1<br>Lperr05g00500.1 0.331 0.993 0.662 0.024 0.994<br>Lperr10g08300.1 0.001 0.291 0.29 0.117 0.971	0<br>AT5G44230.1 0.001 0.298 0.297 0.155 0.872
Obart05g00520.2	0	1<br>Obart05g00520.1 0.001 0.999 0.998 0.001 0.995<br>Obart05g00520.2 0.001 1 0.999 0.001 1	1<br>Osjap05g00530.1 0.001 1 0.999 0.084 1<br>Osjap05g00530.2 0.001 1 0.999 0.103 1<br>Osjap05g00530.3 0.001 1 0.999 0.104 1<br>Osjap05g00530.4 0.001 1 0.999 0.091 1	1<br>Osind05g00510.1 0.049 1 0.951 0.001 1	1<br>Orufi05g00500.1 0.001 1 0.999 0.084 1	1<br>Olong_022405-R1 0.049 1 0.951 0.001 1	0	0	0	0
	1<br>Oglab05g00500.1 0.001 0.295 0.294 0.218 0.982<br>Oglab09g13820.1 0.324 1 0.676 0.064 1	1<br>Obart06g01490.1 0.017 0.295 0.278 0.2 0.982<br>Obart09g15270.1 0.342 1 0.658 0.029 1	1<br>Osjap02g39750.1 0.339 0.994 0.655 0.004 0.956<br>Osjap07g27300.1 0.003 0.293 0.29 0.116 0.834	1<br>Osind09g15830.1 0.342 1 0.658 0.029 1<br>Osind10g10530.1 0.001 0.293 0.292 0.108 0.858	1<br>Orufi07g24760.1 0.003 0.293 0.29 0.116 0.834<br>Orufi09g16480.1 0.342 1 0.658 0.029 1	1<br>Olong_018361-R1 0.342 1 0.658 0.012 1<br>Olong_020432-R1 0.017 0.327 0.31 0.115 0.666	1<br>Opunc05g00480.1 0.331 0.999 0.668 0.007 0.998<br>Opunc05g00490.1 0.001 0.295 0.294 0.228 0.982	1<br>Obrac02g14170.4 0.001 0.302 0.301 0.03 0.843<br>Obrac02g28140.1 0.344 0.995 0.651 0.039 0.973	1<br>Lperr05g00500.1 0.334 1 0.666 0.024 0.996<br>Lperr10g08300.1 0.001 0.293 0.292 0.117 0.971	0<br>AT5G44230.1 0.001 0.3 0.299 0.155 0.872

Obart05g03830.1	1<br>Oglab05g03490.1 0.001 1 0.999 0.001 1<br>Oglab05g03490.2 0.001 1 0.999 0.001 1	1<br>Obart05g03830.1 0.001 1 0.999 0.001 1<br>Obart05g03830.2 0.001 1 0.999 0.001 1	1<br>Osjap05g04120.1 0.001 1 0.999 0.001 1<br>Osjap05g04120.2 0.001 1 0.999 0.001 1	1<br>Osind05g04070.1 0.008 1 0.992 0.001 1<br>Osind05g04070.2 0.008 1 0.992 0.001 1	1<br>Orufi05g03950.1 0.001 1 0.999 0.001 1<br>Orufi05g03950.2 0.001 1 0.999 0.001 1	1<br>Olong_016739-R1 0.074 1 0.926 0.001 1	1<br>Opunc05g03540.1 0.001 1 0.999 0.001 1<br>Opunc05g03540.2 0.001 1 0.999 0.001 1	1<br>Obrac05g02930.1 0.074 1 0.926 0.001 1	0	0
	1<br>Oglab01g19490.1 0.09 0.723 0.633 0.066 0.996<br>Oglab03g14060.1 0.79 1 0.21 0.03 1	1<br>Obart01g23310.1 0.09 0.723 0.633 0.066 0.996<br>Obart03g14050.1 0.79 1 0.21 0.03 1	1<br>Osjap01g27410.1 0.09 0.723 0.633 0.066 0.996<br>Osjap03g15140.5 0.743 1 0.257 0.061 1	1<br>Osind01g28630.1 0.09 0.723 0.633 0.066 0.996<br>Osind03g15230.1 0.79 1 0.21 0.03 1	1<br>Orufi01g26130.1 0.09 0.723 0.633 0.066 0.996<br>Orufi03g14720.1 0.79 1 0.21 0.03 1	1<br>Olong_003347-R2 0.09 0.723 0.633 0.047 0.996<br>Olong_009943-R1 0.79 1 0.21 0.03 1	1<br>Opunc01g23460.1 0.09 0.723 0.633 0.064 0.996<br>Opunc03g13590.1 0.79 1 0.21 0.002 0.545	1<br>Obrac01g17590.1 0.09 0.723 0.633 0.067 0.996<br>Obrac03g11330.1 0.79 1 0.21 0.03 1	0<br>Lperr05g03460.1 0.001 0.721 0.72 0.001 0.993	1<br>AT2G47580.1 0.775 1 0.225 0.008 1<br>AT3G16630.1 0.003 0.721 0.718 0.031 0.992
Obart05g03830.2	1<br>Oglab05g03490.1 0.001 1 0.999 0.001 1<br>Oglab05g03490.2 0.001 1 0.999 0.001 1	1<br>Obart05g03830.1 0.001 1 0.999 0.001 1<br>Obart05g03830.2 0.001 1 0.999 0.001 1	1<br>Osjap05g04120.1 0.001 1 0.999 0.001 1<br>Osjap05g04120.2 0.001 1 0.999 0.001 1	1<br>Osind05g04070.1 0.008 1 0.992 0.001 1<br>Osind05g04070.2 0.008 1 0.992 0.001 1	1<br>Orufi05g03950.1 0.001 1 0.999 0.001 1<br>Orufi05g03950.2 0.001 1 0.999 0.001 1	1<br>Olong_016739-R1 0.073 1 0.927 0.001 1	1<br>Opunc05g03540.1 0.001 1 0.999 0.001 1<br>Opunc05g03540.2 0.001 1 0.999 0.001 1	1<br>Obrac05g02930.1 0.073 1 0.927 0.001 1	0	0
	1<br>Oglab01g19490.1 0.089 0.726 0.637 0.066 0.996<br>Oglab03g14060.1 0.792 1 0.208 0.03 1	1<br>Obart01g23310.1 0.089 0.726 0.637 0.066 0.996<br>Obart03g14050.1 0.792 1 0.208 0.03 1	1<br>Osjap01g27410.1 0.089 0.726 0.637 0.066 0.996<br>Osjap03g15140.5 0.747 1 0.253 0.061 1	1<br>Osind01g28630.1 0.089 0.726 0.637 0.066 0.996<br>Osind03g15230.1 0.792 1 0.208 0.03 1	1<br>Orufi01g26130.1 0.089 0.726 0.637 0.066 0.996<br>Orufi03g14720.1 0.792 1 0.208 0.03 1	1<br>Olong_003347-R2 0.089 0.726 0.637 0.047 0.996<br>Olong_009943-R1 0.792 1 0.208 0.03 1	1<br>Opunc01g23460.1 0.089 0.726 0.637 0.064 0.996<br>Opunc03g13590.1 0.792 1 0.208 0.002 0.545	1<br>Obrac01g17590.1 0.089 0.726 0.637 0.067 0.996<br>Obrac03g11330.1 0.792 1 0.208 0.03 1	0<br>Lperr05g03460.1 0.001 0.725 0.724 0.001 0.993	1<br>AT2G47580.1 0.778 1 0.222 0.008 1<br>AT3G16630.1 0.003 0.725 0.722 0.031 0.992
Obart05g04470.1	1<br>Oglab05g04140.1 0.002 1 0.998 0.002 1	1<br>Obart05g04470.1 0.002 1 0.998 0.002 1	0	0	0	0	0	0	0	0
	1<br>Oglab05g02840.1 0.362 0.977 0.615 0.395 0.582<br>Oglab06g15820.1 0.014 0.359 0.345 0.033 0.973	1<br>Obart05g03110.1 0.362 0.977 0.615 0.395 0.582<br>Obart06g05940.1 0.014 0.35 0.336 0.048 0.458	1<br>Osjap05g04830.1 0.002 0.35 0.348 0.006 0.86<br>Osjap08g03110.1 0.359 0.993 0.634 0.031 0.944	0<br>Osind05g03400.1 0.274 0.945 0.671 0.792 0.975	1<br>Orufi05g04690.1 0.002 0.35 0.348 0.006 0.86<br>Orufi05g04700.1 0.35 1 0.65 0.212 1	1<br>Olong_002683-R1 0.359 0.993 0.634 0.031 0.945<br>Olong_015421-R1 0.002 0.35 0.348 0.01 1	1<br>Opunc05g03750.1 0.362 0.984 0.622 0.659 0.972<br>Opunc05g04100.1 0.002 0.35 0.348 0.006 0.878	0<br>Obrac05g03390.1 0.346 0.986 0.64 0.441 0.983	1<br>Lperr03g03510.1 0.023 0.35 0.327 0.073 0.593<br>Lperr05g03760.1 0.5 0.984 0.484 0.279 0.955	0<br>AT3G16720.1 0.002 0.35 0.348 0.059 0.523
Obart05g13050.1	0	1<br>Obart05g13050.1 0.003 1 0.997 0.003 1	0	0	0	0	0	0	0	0
	1<br>Oglab05g12100.1 0.003 0.701 0.698 0.005 0.976<br>Oglab07g01620.1 0.712 0.997 0.285 0.021 0.894	0<br>Obart01g09660.1 0.035 0.458 0.423 0.069 0.806<br>Obart07g01650.1 0.712 0.997 0.285 0.021 0.894	1<br>Osjap05g06280.3 0.712 1 0.288 0.007 0.318<br>Osjap05g14940.1 0.003 0.701 0.698 0.005 0.967	1<br>Osind05g06280.1 0.712 1 0.288 0.023 0.977<br>Osind05g15640.1 0.003 0.701 0.698 0.005 0.967	1<br>Orufi05g14100.1 0.003 0.701 0.698 0.005 0.967<br>Orufi07g05140.1 0.705 1 0.295 0.18 1	0<br>Olong_024811-R1 0.003 0.701 0.698 0.006 0.956	1<br>Opunc05g11230.1 0.003 0.656 0.653 0.005 0.9<br>Opunc11g14700.1 0.712 1 0.288 0.021 0.904	0<br>Obrac05g08550.1 0.003 0.625 0.622 0.005 0.851	0<br>Lperr05g10310.1 0.125 0.694 0.569 0.005 0.962	0<br>AT5G49350.1 0.122 0.549 0.427 0.593 0.967
Obart05g17330.1	0	1<br>Obart04g00290.1 0.003 1 0.997 0.084 0.835<br>Obart05g17330.1 0.003 1 0.997 0.003 1	0	0	0	0	0	0	0	0
	1<br>Oglab05g16420.1 0.681 1 0.319 0.186 0.432<br>Oglab12g04370.1 0.003 0.613 0.61 0.242 0.582	1<br>Obart01g38930.1 0.629 1 0.371 0.221 0.478<br>Obart07g15280.1 0.003 0.558 0.555 0.231 0.531	1<br>Osjap01g02610.1 0.003 0.613 0.61 0.138 0.36<br>Osjap05g19900.1 0.681 1 0.319 0.173 0.422	1<br>Osind01g45750.2 0.629 0.955 0.326 0.265 0.54<br>Osind07g15230.1 0.003 0.565 0.562 0.101 0.359	1<br>Orufi03g06810.1 0.003 0.635 0.632 0.254 0.985<br>Orufi05g18690.1 0.681 1 0.319 0.19 0.434	1<br>Olong_017674-R1 0.674 1 0.326 0.128 0.394<br>Olong_018765-R1 0.006 0.613 0.607 0.066 0.849	1<br>Opunc02g12800.1 0.006 0.6 0.594 0.212 0.976<br>Opunc07g08640.2 0.674 1 0.326 0.196 0.424	0<br>Obrac05g11690.1 0.681 1 0.319 0.199 0.437	0<br>Lperr01g19800.1 0.003 0.29 0.287 0.354 0.818<br>Lperr05g13860.1 0.677 1 0.323 0.279 0.498	0<br>AT1G72040.1 0.706 1 0.294 0.378 0.559
Obart05g20080.1	0	1<br>Obart05g20080.1 0.001 1 0.999 0.001 1	0	0	0	0	0	0	0	0
	1<br>Oglab05g19000.1 0.001 0.664 0.663 0.066 0.939<br>Oglab09g10550.1 0.666 0.994 0.328 0.217 0.958	1<br>Obart07g26300.1 0.026 0.661 0.635 0.033 0.967<br>Obart09g11580.1 0.666 0.994 0.328 0.208 0.959	1<br>Osjap05g22670.1 0.001 0.664 0.663 0.063 0.939<br>Osjap09g11800.1 0.666 0.994 0.328 0.217 0.958	1<br>Osind07g26930.1 0.026 0.661 0.635 0.033 0.967<br>Osind09g11940.1 0.666 0.994 0.328 0.258 0.969	1<br>Orufi05g21340.1 0.001 0.664 0.663 0.002 0.935<br>Orufi09g12430.2 0.666 0.994 0.328 0.217 0.958	1<br>Olong_005541-R1 0.03 0.624 0.594 0.039 0.917<br>Olong_025141-R1 0.659 0.996 0.337 0.279 1	1<br>Opunc05g17810.1 0.009 0.664 0.655 0.075 0.945<br>Opunc09g10120.1 0.666 0.994 0.328 0.399 0.973	1<br>Obrac07g17500.1 0.013 0.66 0.647 0.034 0.94<br>Obrac09g06450.1 0.666 0.994 0.328 0.182 0.942	1<br>Lperr05g16000.1 0.023 0.664 0.641 0.022 0.942<br>Lperr09g08210.1 0.666 0.994 0.328 0.253 0.964	1<br>AT2G26830.1 0.666 0.994 0.328 0.278 0.971<br>AT2G40480.1 0.016 0.661 0.645 0.081 0.969

Obart05g25070.1	1<br>Oglab05g23160.1 0.001 1 0.999 0.001 1	1<br>Obart05g25070.1 0.001 1 0.999 0.001 1	1<br>Osjap05g28230.1 0.001 1 0.999 0.001 1	1<br>Osind05g28520.1 0.001 1 0.999 0.001 1	1<br>Orufi05g26870.1 0.001 1 0.999 0.001 1	0	1<br>Opunc05g22640.1 0.001 1 0.999 0.001 1	0	1<br>Lperr01g22990.1 0.001 1 0.999 0.027 1	0
	1<br>Oglab01g22740.1 0.028 0.451 0.423 0.061 0.995<br>Oglab01g22750.1 0.462 0.999 0.537 0.009 0.999	1<br>Obart01g26870.1 0.006 0.451 0.445 0.117 0.995<br>Obart01g26880.1 0.462 0.999 0.537 0.009 0.999	1<br>Osjap01g31140.1 0.007 0.451 0.444 0.093 0.995<br>Osjap01g31150.1 0.462 0.999 0.537 0.009 0.999	1<br>Osind01g32600.1 0.001 0.451 0.45 0.002 0.995<br>Osind01g32610.1 0.462 0.999 0.537 0.009 0.999	1<br>Orufi01g29880.1 0.001 0.451 0.45 0.075 0.995<br>Orufi01g29890.1 0.462 0.999 0.537 0.009 0.999	1<br>Olong_000367-R1 0.105 0.451 0.346 0.002 0.993<br>Olong_000368-R1 0.456 0.999 0.543 0.108 0.999	1<br>Opunc01g26820.1 0.049 0.451 0.402 0.002 0.994<br>Opunc01g26830.1 0.462 1 0.538 0.009 1	1<br>Obrac01g20400.1 0.046 0.451 0.405 0.004 0.994<br>Obrac05g17560.2 0.451 1 0.549 0.164 1	1<br>Lperr05g20850.2 0.467 1 0.533 0.002 1<br>Lperr05g20860.1 0.001 0.451 0.45 0.002 0.995	1<br>AT5G08690.1 0.012 0.451 0.439 0.034 0.995<br>AT5G51600.1 0.462 0.999 0.537 0.008 0.996
Obart05g27940.1	0	1<br>Obart05g27940.1 0.001 1 0.999 0.001 1	0	1<br>Osind05g31580.1 0.001 1 0.999 0.024 1	0	1<br>Olong_007981-R1 0.059 1 0.941 0.001 1	0	0	0	0
	1<br>Oglab05g25920.1 0.001 0.581 0.58 0.035 0.982<br>Oglab05g25930.1 0.589 1 0.411 0.07 1	1<br>Obart01g20890.1 0.589 0.98 0.391 0.063 0.943<br>Obart07g06670.1 0.158 0.582 0.424 0.36 0.978	1<br>Osjap01g24630.2 0.589 0.98 0.391 0.156 0.951<br>Osjap05g31230.3 0.001 0.581 0.58 0.041 0.982	1<br>Osind01g25470.1 0.002 0.536 0.534 0.073 0.967<br>Osind01g25480.1 0.589 0.954 0.365 0.063 0.877	1<br>Orufi01g23560.1 0.589 0.98 0.391 0.144 0.949<br>Orufi05g29910.3 0.001 0.581 0.58 0.036 0.982	1<br>Olong_011279-R1 0.589 0.991 0.402 0.063 0.978<br>Olong_011281-R2 0.002 0.565 0.563 0.019 1	1<br>Opunc05g25540.1 0.001 0.581 0.58 0.037 0.983<br>Opunc05g25550.1 0.589 1 0.411 0.067 1	0<br>Obrac05g19820.2 0.001 0.581 0.58 0.002 0.982	1<br>Lperr01g17820.1 0.002 0.582 0.58 0.023 0.973<br>Lperr01g17840.1 0.589 0.998 0.409 0.166 0.978	1<br>AT1G07990.1 0.589 0.917 0.328 0.061 0.782<br>AT4G05420.1 0.001 0.581 0.58 0.002 0.982
Obart06g00880.1	1<br>Oglab06g00840.1 0.001 1 0.999 0.001 1	1<br>Obart06g00880.1 0.001 1 0.999 0.001 1	1<br>Osjap06g00940.1 0.04 1 0.96 0.085 1	0	1<br>Orufi06g00860.1 0.067 1 0.933 0.021 1	0	0	0	0	0
	1<br>Oglab06g00730.1 0.526 0.99 0.464 0.257 0.968<br>Oglab07g19660.1 0.033 0.458 0.425 0.142 0.938	1<br>Obart06g00690.1 0.529 1 0.471 0.099 1<br>Obart09g03180.1 0.021 0.458 0.437 0.145 1	1<br>Osjap06g00950.1 0.529 0.992 0.463 0.132 0.99<br>Osjap08g13790.1 0.051 0.458 0.407 0.005 1	1<br>Osind06g00910.1 0.526 1 0.474 0.068 1<br>Osind08g15520.1 0.051 0.458 0.407 0.005 1	1<br>Orufi06g00690.1 0.529 1 0.471 0.099 1<br>Orufi06g00860.2 0.001 0.445 0.444 0.005 0.98	0<br>Olong_023831-R1 0.053 0.583 0.53 0.001 0.373	1<br>Opunc06g00800.1 0.457 1 0.543 0.025 1<br>Opunc06g00830.1 0.05 0.457 0.407 0.006 1	1<br>Obrac06g00660.1 0.526 1 0.474 0.034 0.999<br>Obrac07g14790.1 0.058 0.503 0.445 0.093 0.948	1<br>Lperr01g40030.1 0.055 0.457 0.402 0.013 0.994<br>Lperr06g00740.1 0.531 0.999 0.468 0.078 0.997	0<br>AT4G34470.1 0.063 0.457 0.394 0.013 1
Obart06g03390.1	0	1<br>Obart06g03390.1 0.001 1 0.999 0.001 1	0	0	0	0	0	0	0	0
	1<br>Oglab06g03530.1 0.411 1 0.589 0.002 1<br>Oglab06g03540.1 0.001 0.322 0.321 0.004 0.923	1<br>Obart01g10160.1 0.421 0.979 0.558 0.025 0.943<br>Obart06g25350.1 0.019 0.331 0.312 0.24 0.91	1<br>Osjap06g03820.1 0.411 1 0.589 0.002 0.797<br>Osjap06g03830.1 0.001 0.322 0.321 0.003 0.924	1<br>Osind06g04060.1 0.412 1 0.588 0.005 0.871<br>Osind06g04070.1 0.001 0.322 0.321 0.004 0.923	1<br>Orufi06g03390.1 0.411 1 0.589 0.002 1<br>Orufi06g03400.1 0.001 0.322 0.321 0.003 0.924	1<br>Olong_004095-R1 0.421 0.974 0.553 0.015 0.951<br>Olong_008568-R1 0.001 0.316 0.315 0.004 0.888	1<br>Opunc06g03190.1 0.419 1 0.581 0.004 1<br>Opunc06g03210.1 0.001 0.319 0.318 0.345 0.985	1<br>Obrac06g02440.1 0.419 1 0.581 0.004 1<br>Obrac06g02460.1 0.001 0.389 0.388 0.499 0.972	1<br>Lperr06g03190.1 0.415 1 0.585 0.004 1<br>Lperr06g03200.1 0.001 0.315 0.314 0.083 0.862	1<br>AT3G13700.1 0.014 0.347 0.333 0.02 0.99<br>AT5G16410.1 0.423 0.979 0.556 0.069 1
Obart06g20680.1	0	1<br>Obart06g20680.1 0.001 1 0.999 0.001 1	0	1<br>Osind06g23650.1 0.001 1 0.999 0.001 1	1<br>Orufi06g22110.1 0.001 1 0.999 0.011 1	0	0	0	0	0
	1<br>Oglab05g13700.1 0.625 1 0.375 0.094 1<br>Oglab06g18700.1 0.001 0.623 0.622 0.016 0.986	1<br>Obart01g27850.1 0.111 0.61 0.499 0.006 0.954<br>Obart05g14650.1 0.625 1 0.375 0.094 1	1<br>Osjap02g34710.2 0.637 0.998 0.361 0.12 0.997<br>Osjap06g24310.1 0.001 0.623 0.622 0.059 0.986	1<br>Osind05g17380.1 0.625 1 0.375 0.094 1<br>Osind09g14580.1 0.026 0.623 0.597 0.005 0.975	1<br>Orufi05g15730.1 0.625 1 0.375 0.094 1<br>Orufi09g15100.1 0.012 0.623 0.611 0.029 0.975	1<br>Olong_017051-R1 0.625 1 0.375 0.104 1<br>Olong_021346-R1 0.001 0.623 0.622 0.01 0.982	1<br>Opunc03g03090.1 0.016 0.639 0.623 0.028 1<br>Opunc05g12780.1 0.641 1 0.359 0.139 1	1<br>Obrac02g22310.1 0.628 0.998 0.37 0.012 0.997<br>Obrac09g08370.1 0.021 0.618 0.597 0.023 0.961	1<br>Lperr02g25000.1 0.628 0.998 0.37 0.012 0.997<br>Lperr07g09420.1 0.029 0.627 0.598 0.034 0.995	1<br>AT3G07940.1 0.637 1 0.363 0.094 1<br>AT5G58830.1 0.008 0.623 0.615 0.019 0.984
Obart06g24730.4	0	1<br>Obart06g24730.4 0.002 1 0.998 0.002 1	0	0	0	0	0	0	0	0
	1<br>Oglab02g04920.1 0.002 0.695 0.693 0 0.105<br>Oglab11g03050.1 0.727 0.952 0.225 0.06 0.354	0<br>Obart06g24730.1 0.002 0.695 0.693 0.001 0.313	1<br>Osjap02g05090.1 0.002 0.695 0.693 0 0.105<br>Osjap11g03420.1 0.727 0.952 0.225 0.066 0.358	1<br>Osind02g05400.1 0.002 0.695 0.693 0.001 0.305<br>Osind11g02780.1 0.727 0.952 0.225 0.06 0.354	1<br>Orufi02g04910.1 0.002 0.695 0.693 0 0.115<br>Orufi12g03280.1 0.701 0.952 0.251 0.039 0.373	1<br>Olong_000683-R1 0.727 0.952 0.225 0.06 0.354<br>Olong_011615-R1 0.002 0.695 0.693 0 0.112	1<br>Opunc02g04040.3 0.002 0.695 0.693 0 0.113<br>Opunc11g02770.1 0.727 0.952 0.225 0.06 0.356	0<br>Obrac06g17230.1 0.102 0.695 0.593 0.002 0.106	1<br>Lperr02g04220.2 0.002 0.695 0.693 0 0.112<br>Lperr12g02790.1 0.715 0.952 0.237 0.047 0.353	0
Obart06g25430.1	1<br>Oglab06g22820.1 0.004 1 0.996 0.005 1	1<br>Obart06g25430.1 0.004 1 0.996 0.004 1	0	0	0	0	0	0	0	0

	1<br>Oglab06g20540.1 0.004 0.419 0.415 0.625 0.915<br>Oglab09g12320.1 0.447 0.984 0.537 0.186 0.994	1<br>Obart06g25600.1 0.059 0.447 0.388 0.721 0.989<br>Obart09g13660.1 0.447 0.984 0.537 0.022 0.993	1<br>Osjap06g26550.1 0.004 0.368 0.364 0.698 0.988<br>Osjap09g14010.1 0.447 0.984 0.537 0.022 0.993	1<br>Osind06g29050.1 0.012 0.419 0.407 0.655 0.91<br>Osind09g14180.1 0.455 0.992 0.537 0.013 0.886	1<br>Orufi06g27530.1 0.059 0.447 0.388 0.721 0.989<br>Orufi09g14650.1 0.447 0.984 0.537 0.186 0.994	0<br>Olong_024432-R1 0.474 0.984 0.51 0.174 0.994	1<br>Opunc06g23340.1 0.004 0.289 0.285 0.367 0.975<br>Opunc09g12070.1 0.455 0.988 0.533 0.014 0.916	0<br>Obrac09g08010.1 0.443 0.984 0.541 0.008 0.992	0<br>Lperr09g09890.1 0.348 0.984 0.636 0.018 0.994	0<br>AT4G30380.1 0.494 0.976 0.482 0.033 0.984
Obart06g28060.1	1<br>Oglab06g25490.1 0.001 1 0.999 0.001 1<br>Oglab09g15660.1 0.024 0.999 0.975 0.154 0.95	1<br>Obart06g28060.1 0.001 1 0.999 0.001 1<br>Obart09g17460.1 0.024 1 0.976 0.076 0.969<br>Obart09g17460.2 0.024 1 0.976 0.076 0.969	1<br>Osjap09g17800.1 0.024 1 0.976 0.113 0.974<br>Osjap09g17800.2 0.024 1 0.976 0.114 0.974<br>Osjap09g17800.3 0.024 0.997 0.973 0.187 0.947	1<br>Osind06g31620.1 0.001 1 0.999 0.001 1	1<br>Orufi06g29990.1 0.001 1 0.999 0.001 1<br>Orufi09g18790.1 0.024 1 0.976 0.107 0.97<br>Orufi09g18790.2 0.024 1 0.976 0.107 0.97<br>Orufi09g18790.3 0.024 1 0.976 0.108 0.969<br>Orufi09g18790.4 0.024 1 0.976 0.108 0.969<br>Orufi09g18790.5 0.024 0.976 0.952 0.17 0.937<br>Orufi09g18790.6 0.024 0.996 0.972 0.172 0.937	1<br>Olong_016700-R1 0.001 1 0.999 0.001 1	0	0	1<br>Lperr09g13380.1 0.084 0.982 0.898 0.166 0.951	0
	1<br>Oglab02g01250.1 0.066 0.406 0.34 0.221 0.93<br>Oglab10g06880.1 0.427 1 0.573 0.099 1	1<br>Obart04g21770.1 0.07 0.417 0.347 0.232 0.933<br>Obart10g07310.1 0.427 1 0.573 0.099 1	1<br>Osjap06g32830.1 0.427 1 0.573 0.085 1<br>Osjap06g32840.1 0.001 0.407 0.406 0.002 0.952	1<br>Osind04g24640.1 0.07 0.417 0.347 0.233 0.933<br>Osind06g30840.1 0.485 0.999 0.514 0.109 1	0<br>Orufi06g29150.4 0.485 0.999 0.514 0.122 1	1<br>Olong_000286-R1 0.066 0.406 0.34 0.258 0.928<br>Olong_026973-R2 0.427 1 0.573 0.099 1	1<br>Opunc06g25610.1 0.001 0.407 0.406 0.002 0.951<br>Opunc10g06480.1 0.427 0.998 0.571 0.098 0.984	1<br>Obrac06g20010.2 0.432 1 0.568 0.119 1<br>Obrac06g20020.1 0.001 0.407 0.406 0.002 0.952	1<br>Lperr02g22230.1 0.043 0.392 0.349 0.113 0.88<br>Lperr06g23510.1 0.402 1 0.598 0.074 1	1<br>AT1G76360.1 0.036 0.406 0.37 0.219 0.952<br>AT3G61130.1 0.465 0.999 0.534 0.207 1
Obart07g03720.1	0	1<br>Obart07g03720.1 0.004 1 0.996 0.004 1	0	0	0	0	0	0	0	0
	1<br>Oglab07g03590.1 0.363 1 0.637 0.007 1<br>Oglab07g03600.1 0.004 0.329 0.325 0.004 0.308	0<br>Obart07g03710.1 0.363 1 0.637 0.007 1	1<br>Osjap07g04140.1 0.363 1 0.637 0.007 1<br>Osjap07g04150.1 0.004 0.329 0.325 0.004 0.305	1<br>Osind07g03650.1 0.363 1 0.637 0.007 1<br>Osind07g03660.1 0.004 0.329 0.325 0.004 0.305	1<br>Orufi07g03430.1 0.363 1 0.637 0.007 1<br>Orufi07g03440.1 0.004 0.329 0.325 0.001 0.07	0<br>Olong_008621-R1 0.464 1 0.536 0.181 1	1<br>Opunc07g03640.1 0.363 1 0.637 0.007 1<br>Opunc07g03650.1 0.004 0.329 0.325 0.004 0.306	0<br>Obrac07g02220.1 0.46 1 0.54 0.174 1	1<br>Lperr07g03220.1 0.363 1 0.637 0.007 1<br>Lperr07g03230.1 0.004 0.308 0.304 0.004 0.24	0<br>AT3G44100.1 0.338 0.962 0.624 0.026 0.941
Obart07g03940.1	0	1<br>Obart07g03940.1 0.001 1 0.999 0.001 1	0	0	0	0	0	0	0	0
	1<br>Oglab01g03190.1 0.435 0.989 0.554 0.002 0.994<br>Oglab03g35120.1 0.038 0.429 0.391 0.002 1	1<br>Obart03g36870.1 0.038 0.393 0.355 0.002 0.889<br>Obart09g12990.1 0.441 0.99 0.549 0.054 0.997	1<br>Osjap01g04010.1 0.435 0.989 0.554 0.002 1<br>Osjap03g39900.1 0.038 0.429 0.391 0.002 1	1<br>Osind01g04300.1 0.441 0.998 0.557 0.001 0.994<br>Osind03g40960.1 0.038 0.429 0.391 0.002 1	1<br>Orufi03g38420.1 0.038 0.429 0.391 0.002 1<br>Orufi09g13930.1 0.441 0.99 0.549 0.079 1	1<br>Olong_012584-R1 0.463 0.991 0.528 0.017 0.998<br>Olong_020874-R1 0.038 0.339 0.301 0.004 1	1<br>Opunc01g03200.2 0.434 0.992 0.558 0.043 0.939<br>Opunc03g33860.1 0.044 0.395 0.351 0.012 0.894	0<br>Obrac09g07500.1 0.442 0.986 0.544 0.102 0.963	1<br>Lperr03g32440.1 0.074 0.414 0.34 0.073 0.966<br>Lperr07g03510.1 0.42 0.989 0.569 0.011 0.992	0<br>AT5G06570.1 0.45 0.989 0.539 0.003 1
Obart07g07590.1	0	1<br>Obart07g07590.1 0.001 1 0.999 0.001 1	0	0	0	0	0	0	0	0
	1<br>Oglab07g07310.1 0.001 0.435 0.434 0.002 0.88<br>Oglab07g07330.1 0.576 1 0.424 0.104 0.867	0<br>Obart03g10940.1 0.611 0.991 0.38 0.085 0.864	1<br>Osjap07g08860.1 0.001 0.435 0.434 0.002 0.88<br>Osjap07g08870.1 0.437 1 0.563 0.125 0.908	1<br>Osind07g07980.1 0.001 0.435 0.434 0.002 0.88<br>Osind07g08000.1 0.576 1 0.424 0.104 0.867	1<br>Orufi07g07560.1 0.001 0.435 0.434 0.002 0.88<br>Orufi07g07580.1 0.576 1 0.424 0.104 0.867	0<br>Olong_025526-R1 0.205 0.435 0.23 0.097 0.801<br>Olong_025527-R1 0.635 1 0.365 0.002 0.889	1<br>Opunc07g07290.1 0.001 0.435 0.434 0.002 0.881<br>Opunc07g07300.1 0.577 1 0.423 0.125 1	1<br>Obrac03g08780.1 0.612 0.991 0.379 0.015 0.852<br>Obrac07g04600.1 0.001 0.435 0.434 0.002 0.88	1<br>Lperr07g06420.1 0.001 0.465 0.464 0.002 0.935<br>Lperr07g06430.1 0.57 1 0.43 0.101 0.879	1<br>AT2G33260.1 0.615 1 0.385 0.028 0.943<br>AT2G47990.1 0.01 0.446 0.436 0.034 0.902
Obart07g11000.2	1<br>Oglab07g10660.1 0.001 0.923 0.922 0.1 0.995<br>Oglab07g10660.3 0.001 0.923 0.922 0.106 0.995<br>Oglab07g10660.4 0.001 0.923 0.922 0.109 0.995	1<br>Obart07g11000.1 0.001 1 0.999 0.001 1<br>Obart07g11000.2 0.001 1 0.999 0.001 1<br>Obart07g11000.3 0.001 1 0.999 0.001 1	1<br>Osjap07g12650.1 0.001 1 0.999 0.094 1<br>Osjap07g12650.2 0.001 1 0.999 0.099 1<br>Osjap07g12650.3 0.001 1 0.999 0.102 1	1<br>Osind03g00080.1 0.001 1 0.999 0.001 1	1<br>Orufi07g11170.1 0.001 1 0.999 0.1 1<br>Orufi07g11170.2 0.001 1 0.999 0.096 1<br>Orufi07g11170.3 0.001 1 0.999 0.098 1<br>Orufi07g11170.4 0.001 1 0.999 0.101 1<br>Orufi07g11170.5 0.001 1 0.999 0.102 1	1<br>Olong_020395-R1 0.001 1 0.999 0.001 1	0	0	0	0

	0<br>Oglab07g10660.2 0.001 0.745 0.744 0.128 0.998	1<br>Obart09g19100.1 0.729 0.974 0.245 0.319 1<br>Obart10g14260.1 0.002 0.716 0.714 0.13 0.992	1<br>Osjap01g03910.2 0.027 0.717 0.69 0.126 0.736<br>Osjap09g19490.1 0.729 0.974 0.245 0.193 1	1<br>Osind08g00800.1 0.036 0.726 0.69 0.111 0.925<br>Osind09g19670.1 0.729 0.974 0.245 0.184 1	1<br>Orufi05g10490.3 0.028 0.707 0.679 0.103 0.96<br>Orufi09g20690.1 0.729 0.974 0.245 0.319 1	1<br>Olong_002950-R1 0.002 0.711 0.709 0.027 0.982<br>Olong_014211-R1 0.729 0.941 0.212 0.332 0.998	0<br>Opunc06g16990.1 0.018 0.76 0.742 0.098 0.997	1<br>Obrac05g06630.4 0.008 0.713 0.705 0.114 0.869<br>Obrac09g12470.1 0.729 0.974 0.245 0.318 1	1<br>Lperr03g21150.4 0.008 0.713 0.705 0.06 0.973<br>Lperr09g14930.2 0.729 0.974 0.245 0.317 1	0<br>AT5G61990.1 0.001 0.744 0.743 0.044 0.999
Obart07g19760.1	0	1<br>Obart07g19760.1 0.002 1 0.998 0.002 1	0	0	0	0	1<br>Opunc07g18680.1 0.002 1 0.998 0.125 1<br>Opunc07g18680.2 0.002 1 0.998 0.12 1<br>Opunc07g18700.1 0.002 1 0.998 0.138 1<br>Opunc07g18700.2 0.002 1 0.998 0.115 1	0	1<br>Lperr07g17150.1 0.002 1 0.998 0.111 1	0
	1<br>Oglab07g04490.1 0.331 0.998 0.667 0.117 0.993<br>Oglab09g04130.1 0.002 0.265 0.263 0.074 0.973	1<br>Obart07g04680.1 0.337 0.998 0.661 0.003 0.994<br>Obart09g04230.1 0.002 0.265 0.263 0.074 0.973	1<br>Osjap07g05300.1 0.319 0.998 0.679 0.079 0.994<br>Osjap09g04300.1 0.002 0.265 0.263 0.074 0.973	1<br>Osind07g19860.1 0.002 0.269 0.267 0.402 0.996<br>Osind07g19870.1 0.337 1 0.663 0.003 1	1<br>Orufi07g04510.1 0.319 0.998 0.679 0.079 0.994<br>Orufi09g04740.1 0.002 0.265 0.263 0.074 0.973	1<br>Olong_011782-R1 0.002 0.265 0.263 0.074 0.973<br>Olong_015820-R2 0.337 1 0.663 0.003 1	1<br>Opunc07g04580.1 0.337 0.998 0.661 0.003 0.994<br>Opunc09g03700.1 0.002 0.265 0.263 0.074 0.973	1<br>Obrac07g12480.1 0.39 1 0.61 0.003 1<br>Obrac09g02110.1 0.002 0.265 0.263 0.074 0.973	0<br>Lperr09g02940.1 0.002 0.265 0.263 0.074 0.973	0<br>AT3G08690.1 0.012 0.273 0.261 0.128 1
Obart07g21100.1	0	1<br>Obart07g21100.1 0.002 1 0.998 0.002 1<br>Obart07g21100.2 0.002 1 0.998 0.001 1	0	0	0	0	0	0	1<br>Lperr07g18240.1 0.153 0.994 0.841 0.123 0.997	0
	0<br>Oglab07g18200.1 0.232 1 0.768 0.193 1	1<br>Obart03g02790.1 0.002 0.223 0.221 0.004 0.649<br>Obart07g08840.2 0.227 0.954 0.727 0.132 0.896	1<br>Osjap03g02790.1 0.002 0.223 0.221 0.004 0.649<br>Osjap07g24570.1 0.232 1 0.768 0.193 1	1<br>Osind03g02880.1 0.002 0.223 0.221 0.004 0.649<br>Osind07g21400.2 0.232 0.998 0.766 0.491 0.995	0<br>Orufi07g22120.1 0.232 1 0.768 0.193 1	1<br>Olong_005980-R1 0.002 0.223 0.221 0.002 0.878<br>Olong_006540-R1 0.243 1 0.757 0.062 1	1<br>Opunc03g02540.1 0.002 0.221 0.219 0.002 0.817<br>Opunc07g13100.1 0.232 1 0.768 0.191 1	1<br>Obrac03g02180.3 0.002 0.223 0.221 0.004 0.649<br>Obrac07g13370.3 0.24 0.994 0.754 0.002 0.994	1<br>Lperr08g11570.3 0.27 0.962 0.692 0.228 0.789<br>Lperr10g13340.1 0.002 0.247 0.245 0.004 0.62	0<br>AT1G26460.1 0.232 0.985 0.753 0.203 0.989
Obart07g23980.1	0	1<br>Obart07g23980.1 0.003 1 0.997 0.003 1	0	0	0	0	0	0	0	0
	1<br>Oglab03g36900.1 0.003 0.276 0.273 0.59 0.785<br>Oglab09g13800.1 0.295 1 0.705 0.34 1	1<br>Obart03g38900.1 0.003 0.276 0.273 0.322 0.644<br>Obart09g15250.1 0.295 1 0.705 0.284 1	1<br>Osjap03g42080.1 0.003 0.292 0.289 0.432 0.749<br>Osjap09g15670.1 0.295 1 0.705 0.334 1	1<br>Osind09g15810.1 0.295 1 0.705 0.34 1<br>Osind11g00110.1 0.003 0.287 0.284 0.555 0.776	1<br>Orufi03g40560.1 0.003 0.276 0.273 0.492 0.733<br>Orufi09g16460.1 0.295 1 0.705 0.339 1	0<br>Olong_003491-R1 0.003 0.357 0.354 0.375 0.732	1<br>Opunc05g11140.1 0.003 0.276 0.273 0.544 0.745<br>Opunc07g22640.1 0.295 1 0.705 0.325 1	1<br>Obrac03g29030.2 0.003 0.276 0.273 0.463 0.718<br>Obrac09g09270.1 0.279 0.981 0.702 0.174 0.993	0<br>Lperr01g12120.1 0.239 0.979 0.74 0.278 0.995	1<br>AT2G24580.1 0.292 0.987 0.695 0.325 0.969<br>AT2G40320.1 0.003 0.282 0.279 0.485 0.734
Obart07g27030.1	0	1<br>Obart07g27030.1 0.001 1 0.999 0.001 1<br>Obart07g27030.2 0.001 1 0.999 0.001 1	0	0	0	0	0	0	1<br>Lperr07g23810.2 0.001 1 0.999 0.381 1<br>Lperr07g23810.3 0.001 0.946 0.945 0.424 0.986	0
	1<br>Oglab07g23620.1 0.348 1 0.652 0.002 1<br>Oglab07g23630.1 0.001 0.311 0.31 0.574 0.982	0<br>Obart06g10380.1 0.374 0.927 0.553 0.107 0.984	1<br>Osjap07g30980.1 0.348 1 0.652 0.003 1<br>Osjap07g31000.1 0.001 0.222 0.221 0.659 0.973	1<br>Osind07g27700.1 0.348 1 0.652 0.002 1<br>Osind07g27720.1 0.001 0.333 0.332 0.537 0.945	0<br>Orufi07g27980.1 0.333 1 0.667 0.091 1	1<br>Olong_001492-R1 0.002 0.333 0.331 0.173 0.995<br>Olong_008701-R1 0.348 1 0.652 0.002 1	1<br>Opunc07g25410.1 0.348 1 0.652 0.002 1<br>Opunc07g25420.1 0.066 0.29 0.224 0.005 0.995	0<br>Obrac07g18100.1 0.348 0.978 0.63 0.004 0.967	0<br>Lperr07g23810.1 0.348 0.98 0.632 0.002 0.96	0<br>AT3G66658.2 0.348 0.997 0.649 0.002 0.997
Obart07g27030.2	0	1<br>Obart07g27030.1 0.001 1 0.999 0.001 1<br>Obart07g27030.2 0.001 1 0.999 0.001 1	0	0	0	0	0	0	1<br>Lperr07g23810.2 0.001 1 0.999 0.381 1<br>Lperr07g23810.3 0.001 0.945 0.944 0.424 0.986	0
	1<br>Oglab07g23620.1 0.354 1 0.646 0.002 1<br>Oglab07g23630.1 0.001 0.317 0.316 0.574 0.982	0<br>Obart06g10380.1 0.381 0.925 0.544 0.107 0.984	1<br>Osjap07g30980.1 0.354 1 0.646 0.003 1<br>Osjap07g31000.1 0.001 0.226 0.225 0.659 0.973	1<br>Osind07g27700.1 0.354 1 0.646 0.002 1<br>Osind07g27720.1 0.001 0.339 0.338 0.537 0.945	0<br>Orufi07g27980.1 0.336 1 0.664 0.086 1	1<br>Olong_001492-R1 0.002 0.339 0.337 0.173 0.995<br>Olong_008701-R1 0.354 1 0.646 0.002 1	1<br>Opunc07g25410.1 0.354 1 0.646 0.002 1<br>Opunc07g25420.1 0.067 0.296 0.229 0.005 0.995	0<br>Obrac07g18100.1 0.354 0.977 0.623 0.004 0.967	0<br>Lperr07g23810.1 0.354 0.979 0.625 0.002 0.96	0<br>AT3G66658.2 0.354 0.997 0.643 0.002 0.997

Obart07g27040.1	0	1<br>Obart07g27040.1 0.002 1 0.998 0.002 1<br>Obart07g27040.2 0.002 1 0.998 0.002 1	0	0	0	0	0	0	0	0
	1<br>Oglab07g23640.2 0.002 0.324 0.322 0.813 0.998<br>Oglab08g15450.1 0.4 0.974 0.574 0.067 0.483	1<br>Obart07g27030.1 0.002 0.249 0.247 0.348 0.51<br>Obart08g16640.1 0.4 0.974 0.574 0.035 0.802	1<br>Osjap07g30980.1 0.002 0.244 0.242 0.003 0.358<br>Osjap07g31000.1 0.318 0.974 0.656 0.002 0.608	1<br>Osind07g27700.1 0.002 0.249 0.247 0.002 0.258<br>Osind07g27720.1 0.318 0.974 0.656 0.012 0.493	0<br>Orufi03g37620.1 0.237 0.884 0.647 0.006 0.997	1<br>Olong_008699-R1 0.315 0.974 0.659 0.003 0.523<br>Olong_008701-R1 0.002 0.249 0.247 0.002 0.258	1<br>Opunc07g25410.1 0.002 0.249 0.247 0.002 0.268<br>Opunc08g14550.1 0.4 0.974 0.574 0.08 0.527	1<br>Obrac07g18100.1 0.002 0.249 0.247 0.004 0.271<br>Obrac08g10910.1 0.4 0.974 0.574 0.075 0.529	0<br>Lperr07g23810.2 0.322 0.974 0.652 0.009 0.348	0<br>AT3G66658.2 0.002 0.268 0.266 0.002 0.273
Obart07g27040.2	0	1<br>Obart07g27040.1 0.002 1 0.998 0.002 1<br>Obart07g27040.2 0.002 1 0.998 0.002 1	0	0	0	0	0	0	0	0
	1<br>Oglab07g23640.2 0.002 0.303 0.301 0.813 0.998<br>Oglab08g15450.1 0.381 0.973 0.592 0.067 0.483	1<br>Obart07g27030.1 0.002 0.226 0.224 0.348 0.51<br>Obart08g16640.1 0.381 0.973 0.592 0.035 0.802	1<br>Osjap07g30980.1 0.002 0.221 0.219 0.003 0.358<br>Osjap07g31000.1 0.298 0.973 0.675 0.002 0.608	1<br>Osind07g27700.1 0.002 0.226 0.224 0.002 0.258<br>Osind07g27720.1 0.298 0.973 0.675 0.012 0.493	1<br>Orufi03g37620.1 0.258 0.881 0.623 0.006 0.998<br>Orufi07g27980.2 0.002 0.226 0.224 0.019 0.253	1<br>Olong_008699-R1 0.294 0.973 0.679 0.003 0.523<br>Olong_008701-R1 0.002 0.226 0.224 0.002 0.258	1<br>Opunc07g25410.1 0.002 0.226 0.224 0.002 0.268<br>Opunc08g14550.1 0.381 0.973 0.592 0.08 0.527	1<br>Obrac07g18100.1 0.002 0.226 0.224 0.004 0.271<br>Obrac08g10910.1 0.381 0.973 0.592 0.075 0.529	0<br>Lperr07g23810.2 0.301 0.973 0.672 0.009 0.348	0<br>AT3G66658.2 0.002 0.246 0.244 0.002 0.273
Obart07g27300.1	0	1<br>Obart07g27300.1 0.001 1 0.999 0.001 1	0	0	0	0	0	0	0	0
	1<br>Oglab07g23910.1 0.002 0.524 0.522 0.008 0.948<br>Oglab07g23920.1 0.527 1 0.473 0.009 1	1<br>Obart03g13590.1 0.028 0.527 0.499 0.001 0.952<br>Obart05g12700.1 0.592 1 0.408 0.017 1	1<br>Osjap07g31290.1 0.001 0.524 0.523 0.033 0.951<br>Osjap07g31300.1 0.555 1 0.445 0.007 1	1<br>Osind03g14630.1 0.028 0.527 0.499 0.001 0.952<br>Osind07g28000.1 0.555 1 0.445 0.046 1	1<br>Orufi03g14250.1 0.028 0.527 0.499 0.001 0.952<br>Orufi07g28280.2 0.555 1 0.445 0.046 1	1<br>Olong_003210-R1 0.002 0.524 0.522 0.015 0.943<br>Olong_003211-R1 0.535 1 0.465 0.026 1	1<br>Opunc03g13100.1 0.028 0.527 0.499 0.001 0.948<br>Opunc07g25690.1 0.555 0.999 0.444 0.007 0.983	1<br>Obrac07g18370.1 0.013 0.524 0.511 0.064 0.951<br>Obrac07g18380.4 0.555 1 0.445 0.007 0.994	1<br>Lperr05g10220.2 0.555 1 0.445 0.014 1<br>Lperr07g24070.2 0.028 0.524 0.496 0.001 0.948	1<br>AT2G38280.1 0.556 0.999 0.443 0.01 0.998<br>AT3G08960.1 0.028 0.527 0.499 0.001 0.952
Obart08g02200.1	0	1<br>Obart08g02200.1 0.001 1 0.999 0.001 1	0	0	0	0	0	0	0	0
	1<br>Oglab03g29880.1 0.022 0.343 0.321 0.079 0.884<br>Oglab08g01680.7 0.344 0.99 0.646 0.109 1	0<br>Obart08g02200.5 0.345 1 0.655 0.01 1	1<br>Osjap03g33630.1 0.022 0.343 0.321 0.079 0.884<br>Osjap08g02400.4 0.344 0.99 0.646 0.106 1	1<br>Osind03g34740.1 0.022 0.343 0.321 0.079 0.884<br>Osind08g02290.1 0.372 0.99 0.618 0.002 1	1<br>Orufi03g32290.1 0.022 0.343 0.321 0.079 0.884<br>Orufi08g02450.3 0.344 0.99 0.646 0.1 1	1<br>Olong_001678-R3 0.368 0.99 0.622 0.039 1<br>Olong_002107-R1 0.028 0.346 0.318 0.11 0.973	1<br>Opunc08g02010.1 0.001 0.34 0.339 0.003 0.997<br>Opunc08g02020.4 0.368 0.99 0.622 0.005 1	1<br>Obrac08g01890.1 0.001 0.34 0.339 0.003 0.896<br>Obrac08g01910.2 0.345 0.99 0.645 0.1 1	1<br>Lperr08g02240.1 0.001 0.339 0.338 0.003 0.949<br>Lperr08g02260.1 0.36 0.99 0.63 0.003 1	1<br>AT3G07060.1 0.388 0.989 0.601 0.11 1<br>AT4G32390.1 0.022 0.354 0.332 0.109 0.98
Obart08g05380.1	0	1<br>Obart08g05380.1 0.004 1 0.996 0.004 1	0	0	0	0	0	0	0	0
	1<br>Oglab08g04820.1 0.331 1 0.669 0.203 1<br>Oglab08g04830.1 0.004 0.298 0.294 0.012 1	1<br>Obart07g07210.1 0.353 1 0.647 0.038 0.703<br>Obart08g05390.1 0.004 0.298 0.294 0.012 1	1<br>Osjap08g05840.2 0.353 1 0.647 0.065 0.75<br>Osjap08g05860.1 0.004 0.305 0.301 0.012 1	1<br>Osind07g07530.1 0.353 1 0.647 0.038 0.703<br>Osind08g05840.1 0.004 0.305 0.301 0.012 1	1<br>Orufi07g07230.1 0.353 1 0.647 0.037 0.706<br>Orufi08g05980.1 0.004 0.305 0.301 0.012 1	1<br>Olong_004294-R1 0.004 0.305 0.301 0.012 1<br>Olong_026698-R1 0.353 1 0.647 0.037 0.706	1<br>Opunc07g06940.1 0.356 1 0.644 0.004 0.695<br>Opunc08g05230.1 0.036 0.305 0.269 0.013 1	1<br>Obrac07g04400.3 0.345 1 0.655 0.015 0.7<br>Obrac08g04320.1 0.004 0.305 0.301 0.012 1	1<br>Lperr07g06270.1 0.356 1 0.644 0.004 0.68<br>Lperr08g05140.1 0.036 0.305 0.269 0.013 1	0<br>AT4G33690.1 0.356 0.924 0.568 0.004 0.691
Obart08g06070.1	1<br>Oglab08g05640.1 0.002 1 0.998 0.002 1	1<br>Obart08g06070.1 0.002 1 0.998 0.002 1	1<br>Osjap08g06650.1 0.002 1 0.998 0.002 1	1<br>Osind08g06790.1 0.002 1 0.998 0.002 1	1<br>Orufi08g06840.1 0.002 1 0.998 0.002 1	0	1<br>Opunc08g05850.1 0.047 1 0.953 0.068 1	0	0	0
	1<br>Oglab02g26360.1 0.348 0.996 0.648 0.01 0.994<br>Oglab04g16180.1 0.047 0.275 0.228 0.198 0.94	1<br>Obart02g28440.1 0.348 0.996 0.648 0.093 0.994<br>Obart04g17770.1 0.047 0.275 0.228 0.198 0.94	1<br>Osjap04g21220.1 0.047 0.275 0.228 0.21 0.936<br>Osjap09g20870.1 0.359 0.984 0.625 0.208 0.966	1<br>Osind04g20250.1 0.047 0.275 0.228 0.198 0.94<br>Osind09g20960.1 0.359 0.984 0.625 0.208 0.966	1<br>Orufi04g19250.2 0.047 0.275 0.228 0.21 0.936<br>Orufi09g21960.1 0.359 0.984 0.625 0.2 0.965	1<br>Olong_008030-R1 0.047 0.275 0.228 0.232 0.937<br>Olong_008031-R2 0.277 1 0.723 0.106 1	1<br>Opunc04g15550.1 0.047 0.275 0.228 0.206 0.938<br>Opunc04g18740.1 0.295 0.984 0.689 0.088 0.994	1<br>Obrac08g04780.1 0.277 1 0.723 0.098 1<br>Obrac08g04800.1 0.002 0.275 0.273 0.006 0.943	1<br>Lperr08g05690.1 0.277 1 0.723 0.1 1<br>Lperr08g05700.1 0.002 0.275 0.273 0.006 0.943	1<br>AT1G05310.1 0.33 0.982 0.652 0.183 0.99<br>AT4G30800.1 0.047 0.275 0.228 0.208 0.943

Obart08g11690.1	0	1<br>Obart08g11690.1 0.001 1 0.999 0.001 1	0	0	0	0	0	0	0	0
	1<br>Oglab08g10860.1 0.525 0.967 0.442 0.003 0.97<br>Oglab11g06870.1 0.008 0.511 0.503 0.007 0.988	0<br>Obart11g07530.1 0.001 0.567 0.566 0.476 0.957	1<br>Osjap03g01130.1 0.007 0.547 0.54 0.029 0.989<br>Osjap04g17040.5 0.561 0.975 0.414 0.224 0.844	1<br>Osind03g01180.1 0.007 0.547 0.54 0.029 0.989<br>Osind04g15750.1 0.561 0.975 0.414 0.254 0.907	1<br>Orufi03g00810.1 0.007 0.547 0.54 0.033 0.987<br>Orufi04g15370.3 0.561 0.975 0.414 0.243 0.917	1<br>Olong_005467-R1 0.01 0.556 0.546 0.049 0.974<br>Olong_007202-R1 0.56 1 0.44 0.342 1	1<br>Opunc06g03930.1 0.561 0.985 0.424 0.35 0.919<br>Opunc12g06580.1 0.001 0.517 0.516 0.036 0.951	1<br>Obrac07g02330.1 0.561 0.971 0.41 0.284 0.872<br>Obrac08g01810.1 0.008 0.517 0.509 0.44 0.989	0<br>Lperr11g13180.1 0.013 0.511 0.498 0.129 0.937	0
Obart08g11880.1	0	1<br>Obart08g11880.1 0.009 1 0.991 0.009 1<br>Obart10g13080.1 0.017 0.966 0.949 0.213 0.999	0	0	0	0	0	0	0	0
	1<br>Oglab02g23150.2 0.333 1 0.667 0.901 1<br>Oglab08g11080.1 0.009 0.333 0.324 0.523 0.701	1<br>Obart02g24490.1 0.333 1 0.667 0.82 1<br>Obart08g11890.1 0.009 0.333 0.324 0.523 0.701	1<br>Osjap02g27820.1 0.333 1 0.667 0.899 1<br>Osjap08g12720.1 0.009 0.333 0.324 0.01 0.379	1<br>Osind02g27730.1 0.333 1 0.667 0.899 1<br>Osind08g14210.1 0.009 0.333 0.324 0.536 0.709	1<br>Orufi02g25850.1 0.333 1 0.667 0.899 1<br>Orufi08g13360.1 0.009 0.333 0.324 0.349 0.792	1<br>Olong_025435-R1 0.342 0.966 0.624 0.075 0.988<br>Olong_027114-R1 0.009 0.333 0.324 0.007 0.277	1<br>Opunc08g10810.1 0.009 0.333 0.324 0.01 0.386<br>Opunc10g11490.1 0.333 0.966 0.633 0.867 0.998	1<br>Obrac02g17520.1 0.333 1 0.667 0.868 0.994<br>Obrac08g08040.1 0.009 0.316 0.307 0.012 0.435	1<br>Lperr02g20030.1 0.333 0.983 0.65 0.899 0.997<br>Lperr08g09830.2 0.009 0.333 0.324 0.011 0.437	0<br>AT3G23410.1 0.248 0.966 0.718 0.878 0.993
Obart08g16670.1	1<br>Oglab01g06790.1 0.007 0.844 0.837 0.061 1<br>Oglab08g15520.1 0.007 0.993 0.986 0.007 0.993	1<br>Obart01g07700.1 0.007 0.844 0.837 0.061 1<br>Obart08g16670.1 0.007 1 0.993 0.007 1	1<br>Osjap01g09050.1 0.007 0.844 0.837 0.061 1	1<br>Osind01g08880.1 0.102 0.986 0.884 0.004 0.567	1<br>Orufi01g08450.1 0.007 0.973 0.966 0.026 0.487	1<br>Olong_003647-R1 0.007 0.83 0.823 0.061 0.985	0	0	0	0
	1<br>Oglab07g08260.1 0.694 0.973 0.279 0.004 0.099<br>Oglab11g05440.1 0.007 0.694 0.687 0.573 0.683	1<br>Obart07g08560.1 0.694 0.952 0.258 0.009 0.192<br>Obart11g05910.1 0.007 0.694 0.687 0.752 0.834	1<br>Osjap01g24690.1 0.007 0.694 0.687 0.104 0.321<br>Osjap07g09900.1 0.694 0.993 0.299 0.005 0.123	1<br>Osind07g08950.1 0.694 0.993 0.299 0.004 0.111<br>Osind11g05650.1 0.007 0.694 0.687 0.103 0.319	1<br>Orufi01g23650.1 0.007 0.694 0.687 0.106 0.307<br>Orufi07g08630.1 0.694 0.993 0.299 0.005 0.115	1<br>Olong_005787-R1 0.007 0.694 0.687 0.103 0.319<br>Olong_016241-R1 0.728 0.932 0.204 0.735 0.768	1<br>Opunc01g21000.1 0.007 0.694 0.687 0.114 0.334<br>Opunc07g08090.1 0.694 0.993 0.299 0.004 0.09	1<br>Obrac07g05080.4 0.728 0.993 0.265 0.102 0.171<br>Obrac11g03720.1 0.007 0.694 0.687 0.112 0.34	1<br>Lperr01g17850.1 0.007 0.694 0.687 0.105 0.313<br>Lperr07g08590.1 0.694 0.993 0.299 0.022 0.124	1<br>AT3G55700.1 0.728 0.973 0.245 0.015 0.093<br>AT4G38130.1 0.007 0.68 0.673 0.09 0.269
Obart08g18520.1	0	1<br>Obart08g18520.1 0.001 1 0.999 0.001 1	0	0	0	0	0	0	0	0
	1<br>Oglab08g17140.1 0.313 1 0.687 0.004 1<br>Oglab08g17150.1 0.014 0.313 0.299 0.283 0.98	1<br>Obart08g18530.1 0.313 1 0.687 0.004 1<br>Obart08g18540.1 0.013 0.313 0.3 0.264 0.98	1<br>Osjap08g19640.1 0.313 1 0.687 0.004 1<br>Osjap08g19650.1 0.014 0.313 0.299 0.283 0.98	1<br>Osind08g21740.1 0.313 1 0.687 0.004 1<br>Osind10g18270.1 0.008 0.308 0.3 0.277 0.981	1<br>Orufi08g20720.1 0.313 1 0.687 0.004 1<br>Orufi08g20730.1 0.013 0.313 0.3 0.264 0.98	1<br>Olong_006289-R1 0.313 1 0.687 0.004 1<br>Olong_006290-R1 0.014 0.313 0.299 0.142 0.976	1<br>Opunc08g16450.1 0.313 1 0.687 0.004 1<br>Opunc08g16460.1 0.014 0.313 0.299 0.287 0.98	1<br>Obrac08g12190.5 0.313 1 0.687 0.004 1<br>Obrac08g12200.1 0.014 0.313 0.299 0.285 0.98	1<br>Lperr08g15390.1 0.313 0.999 0.686 0.004 0.998<br>Lperr08g15400.1 0.014 0.313 0.299 0.283 0.98	1<br>AT4G39980.1 0.014 0.31 0.296 0.335 1<br>AT4G40000.1 0.332 0.986 0.654 0.003 0.978
Obart08g20390.1	0	1<br>Obart08g20390.1 0.001 1 0.999 0.001 1	0	0	0	0	0	0	0	0
	1<br>Oglab08g19250.1 0.001 0.564 0.563 0.304 0.851<br>Oglab09g13510.1 0.566 0.988 0.422 0.501 0.994	0<br>Obart01g18290.1 0.588 0.987 0.399 0.113 0.99	1<br>Osjap08g21820.1 0.577 0.991 0.414 0.003 0.997<br>Osjap08g21830.1 0.001 0.564 0.563 0.303 0.851	1<br>Osind08g23920.1 0.577 0.992 0.415 0.003 0.997<br>Osind08g23930.1 0.001 0.564 0.563 0.303 0.851	1<br>Orufi08g22890.1 0.001 0.564 0.563 0.303 0.851<br>Orufi09g16160.1 0.566 0.988 0.422 0.501 0.994	0<br>Olong_014421-R1 0.553 0.995 0.442 0.015 0.99	1<br>Opunc08g18560.1 0.577 0.991 0.414 0.003 0.976<br>Opunc08g18570.1 0.001 0.564 0.563 0.307 0.852	1<br>Obrac08g13590.1 0.001 0.564 0.563 0.162 0.593<br>Obrac09g09090.1 0.57 0.988 0.418 0.501 0.993	1<br>Lperr01g20410.1 0.577 0.997 0.42 0.003 0.913<br>Lperr08g17250.1 0.001 0.564 0.563 0.303 0.849	1<br>AT5G19440.1 0.577 0.986 0.409 0.003 0.991<br>AT5G25070.1 0.008 0.57 0.562 0.255 0.852
Obart08g23110.1	0	1<br>Obart08g23110.1 0.001 1 0.999 0.001 1	0	0	1<br>Orufi08g25640.1 0.014 1 0.986 0.056 1<br>Orufi08g25640.2 0.014 1 0.986 0.056 1<br>Orufi08g25640.3 0.014 1 0.986 0.056 1	0	0	0	1<br>Lperr08g19910.1 0.014 0.964 0.95 0.065 0.996<br>Lperr08g19910.2 0.014 0.964 0.95 0.065 0.996<br>Lperr08g19910.3 0.014 0.964 0.95 0.065 0.996	0
	1<br>Oglab09g16750.1 0.056 0.444 0.388 0.48 0.983<br>Oglab11g01890.1 0.491 0.968 0.477 0.08 0.987	1<br>Obart08g23140.1 0.487 0.964 0.477 0.03 0.992<br>Obart09g18630.1 0.056 0.444 0.388 0.43 0.984	1<br>Osjap08g24670.1 0.487 0.964 0.477 0.03 0.992<br>Osjap09g19050.1 0.056 0.444 0.388 0.464 0.985	1<br>Osind08g26930.1 0.001 0.445 0.444 0.035 0.978<br>Osind08g26940.1 0.487 0.964 0.477 0.046 0.992	1<br>Orufi08g25640.4 0.487 0.964 0.477 0.047 0.992<br>Orufi09g20160.1 0.056 0.444 0.388 0.464 0.985	0<br>Olong_020799-R2 0.051 0.385 0.334 0.252 0.989<br>Olong_024387-R1 0.54 0.968 0.428 0.002 0.982	1<br>Opunc08g21230.4 0.487 0.964 0.477 0.081 0.992<br>Opunc09g17040.1 0.003 0.444 0.441 0.044 0.975	1<br>Obrac08g15620.1 0.488 0.964 0.476 0.051 0.992<br>Obrac09g12080.1 0.056 0.444 0.388 0.147 0.976	1<br>Lperr09g14540.3 0.056 0.444 0.388 0.164 0.976<br>Lperr12g01980.1 0.49 0.972 0.482 0.082 1	1<br>AT1G09910.1 0.472 0.964 0.492 0.041 0.994<br>AT1G76270.1 0.001 0.424 0.423 0.038 0.944

Obart09g14980.1	1<br>Oglab09g13510.1 0.002 1 0.998 0.002 1<br>Oglab09g13510.2 0.002 1 0.998 0.002 1<br>Oglab09g13510.3 0.002 1 0.998 0.002 1<br>Oglab09g13510.4 0.002 1 0.998 0.002 1	1<br>Obart09g14980.1 0.002 1 0.998 0.002 1<br>Obart09g14980.2 0.002 1 0.998 0.002 1	1<br>Osjap09g15430.2 0.002 1 0.998 0.002 1<br>Osjap09g15430.3 0.002 1 0.998 0.002 1	1<br>Osind09g15570.1 0.002 1 0.998 0.002 1<br>Osind09g15570.2 0.002 1 0.998 0.002 1	1<br>Orufi09g16160.1 0.002 1 0.998 0.002 1<br>Orufi09g16160.2 0.002 1 0.998 0.002 1	0	1<br>Opunc09g13420.1 0.002 1 0.998 0.002 1<br>Opunc09g13420.2 0.002 1 0.998 0.002 1	1<br>Obrac09g09090.1 0.002 1 0.998 0.002 1	0	0
	1<br>Oglab08g19240.1 0.569 0.993 0.424 0.143 0.986<br>Oglab12g18680.1 0.002 0.567 0.565 0.003 1	1<br>Obart02g06180.1 0.01 0.565 0.555 0.01 0.997<br>Obart08g20390.1 0.569 0.993 0.424 0.631 0.988	1<br>Osjap02g06650.1 0.01 0.565 0.555 0.01 0.997<br>Osjap09g15430.1 0.569 1 0.431 0.131 1	1<br>Osind02g06940.1 0.01 0.565 0.555 0.01 0.997<br>Osind08g23920.1 0.569 0.993 0.424 0.131 0.988	1<br>Orufi04g24220.1 0.014 0.567 0.553 0.165 1<br>Orufi08g22880.1 0.569 0.993 0.424 0.131 0.988	1<br>Olong_008093-R2 0.596 0.991 0.395 0.25 0.996<br>Olong_024186-R1 0.002 0.567 0.565 0.003 1	1<br>Opunc04g20190.1 0.007 0.567 0.56 0.006 1<br>Opunc08g18560.1 0.569 0.993 0.424 0.128 0.969	1<br>Obrac01g09090.1 0.605 0.998 0.393 0.299 0.982<br>Obrac02g04550.3 0.009 0.565 0.556 0.214 0.997	1<br>Lperr04g18350.1 0.002 0.567 0.565 0.021 1<br>Lperr09g11120.1 0.569 1 0.431 0.156 1	1<br>AT1G68540.1 0.567 0.991 0.424 0.112 1<br>AT2G21270.3 0.003 0.565 0.562 0.059 0.997
Obart09g14980.2	1<br>Oglab09g13510.1 0.002 1 0.998 0.002 1<br>Oglab09g13510.2 0.002 1 0.998 0.002 1<br>Oglab09g13510.3 0.002 1 0.998 0.002 1<br>Oglab09g13510.4 0.002 1 0.998 0.002 1	1<br>Obart09g14980.1 0.002 1 0.998 0.002 1<br>Obart09g14980.2 0.002 1 0.998 0.002 1	1<br>Osjap09g15430.2 0.002 1 0.998 0.002 1<br>Osjap09g15430.3 0.002 1 0.998 0.002 1	1<br>Osind09g15570.1 0.002 1 0.998 0.002 1<br>Osind09g15570.2 0.002 1 0.998 0.002 1	1<br>Orufi09g16160.1 0.002 1 0.998 0.002 1<br>Orufi09g16160.2 0.002 1 0.998 0.002 1	0	1<br>Opunc09g13420.1 0.002 1 0.998 0.002 1<br>Opunc09g13420.2 0.002 1 0.998 0.002 1	1<br>Obrac09g09090.1 0.002 1 0.998 0.002 1	0	0
	1<br>Oglab08g19240.1 0.544 0.993 0.449 0.143 0.986<br>Oglab12g18680.1 0.002 0.542 0.54 0.003 1	1<br>Obart02g06180.1 0.01 0.54 0.53 0.01 0.997<br>Obart08g20390.1 0.544 0.993 0.449 0.631 0.988	1<br>Osjap02g06650.1 0.01 0.54 0.53 0.01 0.997<br>Osjap09g15430.1 0.544 1 0.456 0.131 1	1<br>Osind02g06940.1 0.01 0.54 0.53 0.01 0.997<br>Osind08g23920.1 0.544 0.993 0.449 0.131 0.988	1<br>Orufi04g24220.1 0.013 0.542 0.529 0.165 1<br>Orufi08g22880.1 0.544 0.993 0.449 0.131 0.988	1<br>Olong_013054-R1 0.547 0.992 0.445 0.149 0.979<br>Olong_024186-R1 0.002 0.542 0.54 0.003 1	1<br>Opunc04g20190.1 0.007 0.542 0.535 0.006 1<br>Opunc08g18560.1 0.544 0.993 0.449 0.128 0.969	1<br>Obrac02g04550.3 0.008 0.54 0.532 0.214 0.997<br>Obrac09g00560.1 0.548 0.998 0.45 0.164 0.966	1<br>Lperr04g18350.1 0.002 0.542 0.54 0.021 1<br>Lperr09g11120.1 0.544 1 0.456 0.156 1	1<br>AT1G68540.1 0.542 0.992 0.45 0.112 1<br>AT2G21270.3 0.003 0.54 0.537 0.059 0.997
Obart09g15000.1	0	1<br>Obart09g15000.1 0.001 1 0.999 0.001 1	0	0	0	0	0	0	0	0
	1<br>Oglab09g13530.1 0.362 1 0.638 0.006 1<br>Oglab09g13540.1 0.001 0.351 0.35 0.003 1	0<br>Obart08g20950.1 0.001 0.314 0.313 0.003 0.87	1<br>Osjap09g15450.1 0.362 0.931 0.569 0.005 0.707<br>Osjap09g15460.1 0.001 0.351 0.35 0.003 1	1<br>Osind09g15590.1 0.362 1 0.638 0.006 1<br>Osind09g15600.1 0.001 0.351 0.35 0.003 1	1<br>Orufi08g23430.1 0.001 0.314 0.313 0.003 0.87<br>Orufi09g16180.1 0.329 1 0.671 0.007 1	1<br>Olong_025646-R1 0.484 0.996 0.512 0.002 0.977<br>Olong_025647-R1 0.001 0.353 0.352 0.003 0.972	1<br>Opunc09g13450.1 0.362 1 0.638 0.006 1<br>Opunc09g13460.1 0.001 0.353 0.352 0.003 0.973	1<br>Obrac09g09110.1 0.362 0.995 0.633 0.003 1<br>Obrac09g09120.2 0.001 0.351 0.35 0.003 1	1<br>Lperr08g17690.1 0.001 0.333 0.332 0.253 0.945<br>Lperr09g11150.1 0.362 0.995 0.633 0.006 1	1<br>AT1G34180.2 0.369 0.984 0.615 0.031 0.977<br>AT1G76700.1 0.001 0.326 0.325 0.003 0.917
Obart09g15360.1	1<br>Oglab09g13920.1 0.001 1 0.999 0.114 1	1<br>Obart09g15360.1 0.001 1 0.999 0.001 1	0	1<br>Osind09g15930.1 0.001 1 0.999 0.057 1	1<br>Orufi09g16580.1 0.001 1 0.999 0.114 1	0	0	0	0	0
	1<br>Oglab02g15910.1 0.003 0.474 0.471 0.048 0.988<br>Oglab09g02290.1 0.507 0.883 0.376 0.02 0.789	1<br>Obart03g04820.3 0.535 0.883 0.348 0.001 0.781<br>Obart08g21020.1 0.001 0.507 0.506 0.088 0.994	1<br>Osjap03g04840.3 0.507 0.883 0.376 0.018 0.769<br>Osjap09g15790.1 0.001 0.507 0.506 0.238 0.993	1<br>Osind08g24670.1 0.001 0.507 0.506 0.221 0.995<br>Osind09g02290.1 0.507 0.883 0.376 0.018 0.769	1<br>Orufi03g04550.5 0.507 0.883 0.376 0.017 0.733<br>Orufi08g23480.1 0.001 0.507 0.506 0.228 0.995	1<br>Olong_025005-R2 0.535 0.979 0.444 0.001 0.955<br>Olong_025006-R1 0.05 0.507 0.457 0.031 0.99	1<br>Opunc08g19030.2 0.001 0.507 0.506 0.156 0.976<br>Opunc09g13790.1 0.507 0.979 0.472 0.055 0.958	0<br>Obrac08g14010.2 0.001 0.508 0.507 0.044 0.989	1<br>Lperr08g17810.1 0.001 0.507 0.506 0.122 0.994<br>Lperr09g11510.2 0.507 0.979 0.472 0.01 0.958	1<br>AT2G32000.1 0.507 0.978 0.471 0.012 0.961<br>AT2G43900.1 0.003 0.481 0.478 0.165 0.843
Obart09g15430.1	0	1<br>Obart09g15430.1 0.003 1 0.997 0.003 1	0	1<br>Osind09g16000.1 0.003 0.943 0.94 0.002 1	0	0	0	0	0	0
	1<br>Oglab09g13980.1 0.003 0.443 0.44 0.008 1<br>Oglab09g13990.1 0.49 1 0.51 0.006 1	1<br>Obart01g41710.1 0.547 0.953 0.406 0.165 0.937<br>Obart09g15390.1 0.003 0.443 0.44 0.008 1	1<br>Osjap08g22450.1 0.003 0.443 0.44 0.254 1<br>Osjap09g15860.1 0.49 1 0.51 0.006 1	1<br>Osind01g48630.1 0.547 0.953 0.406 0.165 0.937<br>Osind08g24700.1 0.003 0.443 0.44 0.008 1	1<br>Orufi09g16660.1 0.49 1 0.51 0.006 1<br>Orufi09g16670.1 0.003 0.477 0.474 0.007 1	1<br>Olong_001207-R1 0.547 0.953 0.406 0.165 0.937<br>Olong_017992-R1 0.003 0.443 0.44 0.008 1	1<br>Opunc09g13840.1 0.003 0.443 0.44 0.008 1<br>Opunc09g13850.1 0.49 0.967 0.477 0.007 0.934	1<br>Obrac09g09430.1 0.003 0.443 0.44 0.008 1<br>Obrac09g09440.1 0.49 1 0.51 0.007 1	1<br>Lperr03g16320.1 0.003 0.443 0.44 0.007 1<br>Lperr09g11590.1 0.49 1 0.51 0.007 1	1<br>AT4G18100.1 0.003 0.443 0.44 0.008 1<br>AT5G45680.1 0.467 0.94 0.473 0.284 0.99
Obart09g16160.1	0	1<br>Obart09g16160.1 0.001 1 0.999 0.001 1	0	0	0	0	0	0	0	0
	1	1	1	1	1	1	1	1	1	1

	Oglab09g14660.1 0.001 0.441 0.44 0.003 0.979<br>Oglab09g14670.1 0.485 1 0.515 0.2 1	Obart04g25680.1 0.485 0.974 0.489 0.159 0.786<br>Obart08g22080.1 0.004 0.441 0.437 0.04 0.979	Osjap09g16620.1 0.001 0.441 0.44 0.003 0.979<br>Osjap09g16630.1 0.485 1 0.515 0.2 1	Osind10g01650.1 0.001 0.441 0.44 0.003 0.979<br>Osind10g01660.1 0.485 1 0.515 0.245 1	Orufi09g17470.1 0.001 0.441 0.44 0.003 0.979<br>Orufi09g17480.1 0.485 1 0.515 0.2 1	Olong_000869-R1 0.485 1 0.515 0.072 1<br>Olong_000870-R1 0.001 0.441 0.44 0.003 0.979	Opunc09g14580.1 0.001 0.441 0.44 0.003 0.979<br>Opunc09g14590.1 0.485 1 0.515 0.205 1	Obrac09g10080.1 0.001 0.441 0.44 0.262 0.984<br>Obrac09g10090.2 0.485 0.999 0.514 0.083 1	Lperr09g12290.1 0.001 0.441 0.44 0.003 0.979<br>Lperr09g12300.1 0.485 0.999 0.514 0.268 0.998	AT2G17390.1 0.003 0.441 0.438 0.061 0.98<br>AT3G03310.1 0.485 0.995 0.51 0.157 0.996
Obart09g16460.1	0	1<br>Obart09g16460.1 0.001 1 0.999 0.001 1	0	0	0	0	0	0	0	0
	1<br>Oglab01g17730.1 0.727 0.997 0.27 0.15 0.536<br>Oglab09g14960.1 0.001 0.721 0.72 0.002 0.929	1<br>Obart01g21490.2 0.727 1 0.273 0.17 0.551<br>Obart03g20890.1 0.025 0.659 0.634 0.028 0.915	1<br>Osjap02g19520.2 0.731 1 0.269 0.056 0.184<br>Osjap09g16920.1 0.001 0.721 0.72 0.002 0.914	1<br>Osind01g26310.1 0.727 0.997 0.27 0.344 0.652<br>Osind09g17080.1 0.001 0.721 0.72 0.002 0.918	1<br>Orufi01g24210.2 0.727 0.997 0.27 0.04 0.462<br>Orufi09g17770.1 0.001 0.721 0.72 0.002 0.918	0<br>Olong_025574-R2 0.098 0.659 0.561 0.059 0.907	1<br>Opunc01g21720.2 0.727 1 0.273 0.051 0.595<br>Opunc09g14890.1 0.001 0.721 0.72 0.002 0.917	0<br>Obrac09g10330.1 0.025 0.721 0.696 0.032 0.901	1<br>Lperr02g05180.1 0.731 1 0.269 0.121 0.552<br>Lperr09g12570.1 0.023 0.721 0.698 0.013 0.312	0<br>AT2G26260.1 0.025 0.73 0.705 0.021 0.92
Obart09g18850.1	0	1<br>Obart09g18850.1 0.001 1 0.999 0.001 1	0	0	0	0	0	0	0	0
	1<br>Oglab02g28320.1 0.001 0.712 0.711 0.001 0.999<br>Oglab09g16980.1 0.712 0.998 0.286 0.011 0.742	1<br>Obart02g30770.1 0.056 0.712 0.656 0.002 0.998<br>Obart09g19080.1 0.713 0.996 0.283 0.01 0.943	1<br>Osjap02g34900.1 0.001 0.712 0.711 0.001 0.999<br>Osjap09g18210.1 0.713 0.995 0.282 0.014 0.875	1<br>Osind09g18400.1 0.713 0.995 0.282 0.014 0.873<br>Osind09g19410.2 0.001 0.713 0.712 0.001 1	1<br>Orufi09g19250.1 0.713 0.995 0.282 0.014 0.873<br>Orufi09g20400.1 0.001 0.713 0.712 0.118 1	1<br>Olong_009633-R1 0.011 0.702 0.691 0.036 1<br>Olong_028691-R1 0.719 0.995 0.276 0.004 0.861	0<br>Opunc09g17250.1 0.001 0.712 0.711 0.001 0.999	0<br>Obrac09g12310.2 0.002 0.712 0.71 0.004 0.999	1<br>Lperr05g22000.1 0.713 0.988 0.275 0.036 1<br>Lperr09g14830.1 0.001 0.711 0.71 0.001 0.962	0<br>AT4G26600.1 0.115 0.601 0.486 0.165 0.902
Obart10g02510.1	1<br>Oglab10g02440.1 0.001 1 0.999 0.001 1<br>Oglab10g02440.2 0.001 1 0.999 0.001 1	1<br>Obart10g02510.1 0.001 1 0.999 0.001 1<br>Obart10g02510.2 0.001 1 0.999 0.001 1	0	1<br>Osind10g02110.1 0.001 0.995 0.994 0.001 0.952	0	0	0	0	0	0
	0<br>Oglab10g02440.3 0.455 1 0.545 0.005 1	1<br>Obart10g00500.1 0.001 0.391 0.39 0.001 0.993<br>Obart11g08380.1 0.528 1 0.472 0.194 0.922	1<br>Osjap10g02940.1 0.021 0.393 0.372 0.027 0.984<br>Osjap10g02950.1 0.472 0.972 0.5 0.002 0.928	1<br>Osind10g00350.1 0.001 0.39 0.389 0.001 0.98<br>Osind11g07740.1 0.472 0.995 0.523 0.003 1	1<br>Orufi10g02520.1 0.021 0.222 0.201 0.065 0.758<br>Orufi10g02530.1 0.223 0.995 0.772 0.284 1	1<br>Olong_016648-R1 0.223 0.995 0.772 0.265 1<br>Olong_020863-R1 0.005 0.222 0.217 0.007 0.797	1<br>Opunc10g02120.1 0.001 0.393 0.392 0.001 0.992<br>Opunc11g08150.1 0.472 1 0.528 0.003 0.911	1<br>Obrac03g10950.1 0.001 0.39 0.389 0.001 0.983<br>Obrac11g05340.1 0.472 1 0.528 0.002 0.922	1<br>Lperr10g00410.1 0.001 0.391 0.39 0.003 0.986<br>Lperr10g01950.1 0.472 1 0.528 0.003 0.895	0<br>AT1G21230.1 0.005 0.353 0.348 0.01 0.93<br>AT4G00830.1 0.523 0.972 0.449 0.194 0.891
Obart10g02510.2	1<br>Oglab10g02440.1 0.001 1 0.999 0.001 1<br>Oglab10g02440.2 0.001 1 0.999 0.001 1	1<br>Obart10g02510.1 0.001 1 0.999 0.001 1<br>Obart10g02510.2 0.001 1 0.999 0.001 1	0	1<br>Osind10g02110.1 0.001 0.995 0.994 0.001 0.952	0	0	0	0	0	0
	0<br>Oglab10g02440.3 0.476 1 0.524 0.005 1	1<br>Obart10g00500.1 0.001 0.409 0.408 0.001 0.993<br>Obart11g08380.1 0.553 1 0.447 0.194 0.922	1<br>Osjap10g02940.1 0.022 0.411 0.389 0.027 0.984<br>Osjap10g02950.1 0.495 0.971 0.476 0.002 0.928	1<br>Osind10g00350.1 0.001 0.408 0.407 0.001 0.98<br>Osind11g07740.1 0.495 0.995 0.5 0.003 0.96	1<br>Orufi10g02520.1 0.022 0.232 0.21 0.065 0.758<br>Orufi10g02530.1 0.233 0.995 0.762 0.284 0.988	1<br>Olong_016648-R1 0.233 0.995 0.762 0.265 1<br>Olong_028710-R1 0.031 0.232 0.201 0.004 0.754	1<br>Opunc10g02120.1 0.001 0.411 0.41 0.001 0.992<br>Opunc11g08150.1 0.495 1 0.505 0.003 0.911	1<br>Obrac03g10950.1 0.001 0.408 0.407 0.001 0.983<br>Obrac11g05340.1 0.495 1 0.505 0.002 0.922	1<br>Lperr10g00410.1 0.001 0.409 0.408 0.003 0.986<br>Lperr10g01950.1 0.495 1 0.505 0.003 0.895	0<br>AT1G21230.1 0.005 0.37 0.365 0.01 0.93<br>AT4G00830.1 0.548 0.971 0.423 0.194 0.891
Obart10g09110.2	1<br>Oglab10g08680.1 0.009 1 0.991 0.008 1<br>Oglab10g08680.2 0.001 1 0.999 0.001 1	1<br>Obart10g09110.1 0.009 1 0.991 0.008 1<br>Obart10g09110.2 0.001 1 0.999 0.001 1	0	0	1<br>Orufi10g09480.1 0.009 1 0.991 0.008 1<br>Orufi10g09480.2 0.009 1 0.991 0.008 1<br>Orufi10g09480.3 0.001 1 0.999 0.001 1	1<br>Olong_027249-R1 0.03 1 0.97 0.001 1<br>Olong_027249-R2 0.03 1 0.97 0.001 1	0	0	0	0
	1<br>Oglab02g10660.1 0.625 0.999 0.374 0.129 0.991<br>Oglab12g06810.1 0.013 0.615 0.602 0.056 0.969	0<br>Obart02g10660.1 0.625 0.999 0.374 0.129 0.991	1<br>Osjap01g36630.1 0.626 1 0.374 0.222 1<br>Osjap10g10740.1 0.001 0.626 0.625 0.002 0.997	1<br>Osind10g08120.1 0.626 1 0.374 0.222 1<br>Osind10g08130.1 0.001 0.626 0.625 0.002 0.997	1<br>Orufi01g35060.1 0.626 1 0.374 0.222 1<br>Orufi12g07540.1 0.013 0.615 0.602 0.039 0.673	1<br>Olong_022306-R1 0.609 0.991 0.382 0.19 0.992<br>Olong_024721-R1 0.105 0.604 0.499 0.048 0.942	1<br>Opunc01g30900.1 0.626 1 0.374 0.222 1<br>Opunc10g07870.1 0.004 0.626 0.622 0.003 0.997	1<br>Obrac01g24330.1 0.626 1 0.374 0.221 1<br>Obrac12g04960.2 0.102 0.615 0.513 0.026 0.902	1<br>Lperr01g27340.1 0.626 1 0.374 0.228 1<br>Lperr09g14980.1 0.001 0.604 0.603 0.002 0.959	0<br>AT4G14820.1 0.001 0.614 0.613 0.004 0.86
Obart10g12420.1	0	1<br>Obart10g12420.1 0.001 1 0.999 0.001 1	0	0	0	1<br>Olong_012076-R1 0.032 1 0.968 0.001 1	0	0	1<br>Lperr10g09020.1 0.032 1 0.968 0.001 1	0

	1<br>Oglab10g11980.3 0.017 0.579 0.562 0.038 1<br>Oglab10g11990.1 0.58 1 0.42 0.024 1	0<br>Obart10g12420.3 0.001 0.579 0.578 0.001 1	1<br>Osjap10g14690.1 0.008 0.579 0.571 0.082 1<br>Osjap10g14700.1 0.58 1 0.42 0.117 1	1<br>Osind10g11740.1 0.053 0.579 0.526 0.002 1<br>Osind10g11750.1 0.58 1 0.42 0.024 1	1<br>Orufi10g13110.1 0.008 0.579 0.571 0.096 1<br>Orufi10g13120.1 0.58 1 0.42 0.117 1	0<br>Olong_001780-R1 0.573 1 0.427 0.049 0.996	1<br>Opunc10g10660.1 0.008 0.579 0.571 0.078 1<br>Opunc10g10670.1 0.58 1 0.42 0.129 1	1<br>Obrac10g07370.1 0.032 0.58 0.548 0.001 0.999<br>Obrac10g07380.1 0.58 1 0.42 0.117 1	0<br>Lperr02g00290.1 0.573 0.996 0.423 0.016 0.342	0<br>AT3G04260.1 0.008 0.376 0.368 0.074 0.681<br>AT3G13860.1 0.584 0.997 0.413 0.033 0.998
Obart10g19720.1	1<br>Oglab10g16090.1 0.002 0.992 0.99 0.001 0.861	1<br>Obart10g19720.1 0.002 1 0.998 0.002 1	0	1<br>Osind10g19230.1 0.002 1 0.998 0.002 1	1<br>Orufi10g20960.1 0.002 1 0.998 0.002 1	0	1<br>Opunc10g18180.1 0.002 0.992 0.99 0.002 0.85	0	0	0
	1<br>Oglab01g14760.1 0.483 0.992 0.509 0.2 0.827<br>Oglab08g03250.2 0.042 0.431 0.389 0.052 0.955	1<br>Obart01g17550.1 0.483 0.992 0.509 0.174 0.721<br>Obart08g03750.1 0.042 0.431 0.389 0.057 0.95	1<br>Osjap10g22750.1 0.473 0.992 0.519 0.061 0.836<br>Osjap10g22760.1 0.002 0.451 0.449 0.003 0.993	1<br>Osind01g20890.1 0.483 0.992 0.509 0.154 0.668<br>Osind08g03990.1 0.042 0.431 0.389 0.057 0.95	1<br>Orufi01g19490.1 0.483 0.992 0.509 0.171 0.72<br>Orufi08g04140.1 0.042 0.431 0.389 0.057 0.95	1<br>Olong_006312-R1 0.48 0.987 0.507 0.067 0.835<br>Olong_006313-R1 0.002 0.451 0.449 0.003 0.993	1<br>Opunc01g18040.1 0.483 0.992 0.509 0.174 0.822<br>Opunc08g03700.1 0.016 0.431 0.415 0.011 0.951	1<br>Obrac01g13330.1 0.487 0.992 0.505 0.196 0.825<br>Obrac10g13120.1 0.002 0.451 0.449 0.004 0.993	1<br>Lperr10g15590.1 0.48 0.992 0.512 0.07 0.79<br>Lperr10g15600.1 0.002 0.448 0.446 0.003 0.979	1<br>AT1G32080.1 0.484 0.989 0.505 0.188 0.82<br>AT2G32260.1 0.041 0.432 0.391 0.078 0.825
Obart11g00370.1	0	1<br>Obart11g00370.1 0.001 1 0.999 0.001 1	0	0	0	0	0	0	0	0
	1<br>Oglab12g00090.1 0.631 1 0.369 0.003 1<br>Oglab12g00100.6 0.001 0.568 0.567 0.19 0.961	1<br>Obart12g00180.1 0.631 1 0.369 0.003 1<br>Obart12g00190.6 0.001 0.568 0.567 0.049 0.954	1<br>Osjap12g00400.1 0.631 1 0.369 0.003 1<br>Osjap12g00410.6 0.001 0.568 0.567 0.058 0.955	1<br>Osind12g00490.1 0.631 1 0.369 0.003 1<br>Osind12g00500.2 0.001 0.568 0.567 0.18 0.961	1<br>Orufi05g21080.1 0.055 0.568 0.513 0.019 0.946<br>Orufi12g00420.1 0.631 1 0.369 0.003 1	0<br>Olong_018889-R1 0.049 0.568 0.519 0.09 0.95	1<br>Opunc05g17550.1 0.049 0.568 0.519 0.084 0.95<br>Opunc12g00380.3 0.658 1 0.342 0.064 1	1<br>Obrac11g00100.1 0.636 1 0.364 0.013 1<br>Obrac11g00110.4 0.047 0.568 0.521 0.076 0.945	1<br>Lperr05g15790.1 0.049 0.568 0.519 0.088 0.95<br>Lperr11g00400.1 0.638 1 0.362 0.006 1	0<br>AT3G11070.1 0.041 0.578 0.537 0.088 0.988
Obart11g00980.1	0	1<br>Obart11g00980.1 0.001 1 0.999 0.001 1	0	0	0	0	0	0	0	0
	1<br>Oglab12g00700.1 0.664 0.973 0.309 0.121 0.761<br>Oglab12g00710.1 0.001 0.659 0.658 0.113 0.984	0<br>Obart03g07950.1 0.022 0.293 0.271 0.017 0.284<br>Obart05g00580.2 0.665 0.93 0.265 0.242 0.747	1<br>Osjap11g01110.2 0.001 0.659 0.658 0.001 0.983<br>Osjap12g01040.1 0.664 0.973 0.309 0.121 0.761	1<br>Osind11g00570.2 0.664 0.973 0.309 0.059 0.705<br>Osind11g00580.1 0.001 0.659 0.658 0.001 0.983	1<br>Orufi11g00880.1 0.664 0.973 0.309 0.105 0.755<br>Orufi11g00890.2 0.001 0.659 0.658 0.001 0.983	0<br>Olong_003043-R1 0.665 0.972 0.307 0.191 0.804<br>Olong_023565-R1 0.057 0.332 0.275 0.007 0.39	1<br>Opunc11g00810.1 0.664 0.973 0.309 0.087 0.793<br>Opunc11g00820.2 0.001 0.659 0.658 0.166 0.985	1<br>Obrac03g06410.1 0.022 0.344 0.322 0.018 0.356<br>Obrac11g00470.1 0.664 0.973 0.309 0.142 0.792<br>Obrac11g00480.1 0.401 0.656 0.255 0.003 0.947	1<br>Lperr12g00840.2 0.664 0.973 0.309 0.125 0.763<br>Lperr12g00850.1 0.001 0.659 0.658 0.001 0.982	0<br>AT3G51630.1 0.664 0.945 0.281 0.118 0.791<br>AT3G56320.1 0.014 0.332 0.318 0.046 0.73
Obart11g01060.1	1<br>Oglab12g00800.1 0.001 1 0.999 0.001 1	1<br>Obart11g01060.1 0.001 1 0.999 0.001 1	1<br>Osjap11g01220.1 0.001 1 0.999 0.001 1<br>Osjap12g01140.1 0.001 1 0.999 0.001 1	1<br>Osind11g00690.1 0.001 0.954 0.953 0.001 0.999<br>Osind12g01290.1 0.001 1 0.999 0.001 1	1<br>Orufi11g01000.1 0.001 1 0.999 0.001 1<br>Orufi12g01130.1 0.001 1 0.999 0.001 1	0	1<br>Opunc12g00940.1 0.001 1 0.999 0.001 1	0	0	0
	1<br>Oglab03g12120.1 0.479 0.982 0.503 0.121 0.633<br>Oglab07g22980.1 0.007 0.42 0.413 0.007 0.94	1<br>Obart03g12120.1 0.479 0.982 0.503 0.121 0.633<br>Obart12g00790.1 0.001 0.42 0.419 0.002 0.949	1<br>Osjap03g13100.1 0.479 0.982 0.503 0.097 0.622<br>Osjap07g30390.1 0.01 0.42 0.41 0.013 0.944	1<br>Osind03g12930.1 0.479 0.982 0.503 0.121 0.633<br>Osind07g27070.1 0.007 0.42 0.413 0.006 0.946	1<br>Orufi03g17220.1 0.485 1 0.515 0.027 1<br>Orufi07g27420.1 0.01 0.42 0.41 0.013 0.946	1<br>Olong_007715-R1 0.482 1 0.518 0.007 1<br>Olong_008211-R1 0.007 0.42 0.413 0.006 0.946	1<br>Opunc03g11680.1 0.479 1 0.521 0.148 1<br>Opunc11g00920.1 0.001 0.42 0.419 0.142 0.951	1<br>Obrac03g09720.2 0.479 1 0.521 0.142 1<br>Obrac12g00780.1 0.001 0.42 0.419 0.11 0.953	1<br>Lperr03g10720.1 0.479 0.996 0.517 0.148 0.954<br>Lperr11g00920.1 0.001 0.42 0.419 0.002 0.949	1<br>AT3G46740.1 0.482 1 0.518 0.161 1<br>AT4G20110.2 0.001 0.42 0.419 0.002 0.946
Obart11g04380.1	0	1<br>Obart11g04380.1 0.001 1 0.999 0.001 1	0	0	0	0	0	0	0	0
	0<br>Oglab11g04130.1 0.001 0.601 0.6 0.002 0.78	1<br>Obart03g11190.1 0.061 0.586 0.525 0.048 0.746<br>Obart03g32320.1 0.609 0.926 0.317 0.383 0.899	1<br>Osjap03g34930.1 0.609 0.926 0.317 0.383 0.899<br>Osjap11g04880.1 0.001 0.601 0.6 0.002 0.78	1<br>Osind03g34710.1 0.609 0.926 0.317 0.383 0.899<br>Osind11g04140.1 0.001 0.601 0.6 0.002 0.78	1<br>Orufi03g33710.1 0.609 0.926 0.317 0.383 0.899<br>Orufi11g04260.1 0.001 0.601 0.6 0.002 0.738	1<br>Olong_004085-R1 0.061 0.586 0.525 0.048 0.744<br>Olong_009498-R1 0.609 0.926 0.317 0.13 0.858	1<br>Opunc03g29490.1 0.609 0.926 0.317 0.377 0.904<br>Opunc11g04020.1 0.001 0.601 0.6 0.002 0.777	1<br>Obrac03g08970.1 0.071 0.617 0.546 0.061 0.804<br>Obrac11g02780.1 0.724 1 0.276 0.005 0.985	1<br>Lperr03g26910.1 0.609 0.926 0.317 0.377 0.904<br>Lperr11g03800.1 0.001 0.601 0.6 0.002 0.77	1<br>AT3G57620.1 0.054 0.587 0.533 0.044 0.762<br>AT5G64090.1 0.587 1 0.413 0.395 1
Obart11g16070.1	1<br>Oglab11g14350.1 0.013 0.975 0.962 0.001 1<br>Oglab11g14350.2 0.013 1 0.987 0.001 1	1<br>Obart11g16070.1 0.001 1 0.999 0.001 1	0	0	0	0	0	0	0	0

	1<br>Oglab02g07220.2 0.685 1 0.315 0.003 1<br>Oglab11g18500.1 0.036 0.633 0.597 0.003 0.966	1<br>Obart02g07160.1 0.685 1 0.315 0.002 1<br>Obart08g10830.1 0.037 0.654 0.617 0.004 0.995	1<br>Osjap02g07570.1 0.685 1 0.315 0.002 1<br>Osjap11g18290.1 0.001 0.632 0.631 0.006 0.989	1<br>Osind11g13470.1 0.001 0.605 0.604 0.005 0.997<br>Osind11g13490.1 0.684 1 0.316 0.081 1	1<br>Orufi11g17130.1 0.001 0.632 0.631 0.006 0.989<br>Orufi11g17160.1 0.685 1 0.315 0.002 1	1<br>Olong_012209-R1 0.685 1 0.315 0.002 1<br>Olong_012210-R1 0.001 0.632 0.631 0.01 0.981	1<br>Opunc11g13170.1 0.685 1 0.315 0.002 1<br>Opunc11g13180.1 0.033 0.634 0.601 0.145 0.975	1<br>Obrac02g05260.1 0.685 1 0.315 0.002 1<br>Obrac10g05860.1 0.038 0.627 0.589 0.005 0.946	1<br>Lperr11g12540.1 0.035 0.63 0.595 0.002 0.969<br>Lperr12g06590.1 0.674 1 0.326 0.078 1	0<br>AT2G44350.2 0.685 1 0.315 0.002 0.998
Obart11g22080.1	1<br>Oglab11g20100.1 0.024 0.953 0.929 0.012 0.992	1<br>Obart11g22080.1 0.001 1 0.999 0.001 1<br>Obart11g23070.1 0.024 0.973 0.949 0.038 0.936	0	0	0	1<br>Olong_001283-R1 0.004 0.94 0.936 0.012 0.965	1<br>Opunc11g18660.1 0.001 0.987 0.986 0.001 0.924<br>Opunc11g18660.2 0.001 0.987 0.986 0.001 0.925<br>Opunc11g18660.3 0.001 0.987 0.986 0.001 0.89<br>Opunc11g19450.1 0.024 0.973 0.949 0.016 0.933	0	1<br>Lperr11g13500.1 0.008 0.944 0.936 0.003 0.998	0
	1<br>Oglab11g11420.1 0.008 0.461 0.453 0.002 0.759<br>Oglab11g19250.1 0.498 0.987 0.489 0.002 0.999	1<br>Obart11g12900.1 0.008 0.474 0.466 0.002 0.766<br>Obart11g22110.1 0.506 0.988 0.482 0.003 0.891	1<br>Osjap10g09150.6 0.007 0.461 0.454 0.005 0.72<br>Osjap11g25990.5 0.5 0.987 0.487 0.086 0.998	0<br>Osind05g25410.1 0.007 0.534 0.527 0.001 0.979	1<br>Orufi11g13640.1 0.008 0.474 0.466 0.002 0.766<br>Orufi11g24060.2 0.49 0.993 0.503 0.001 0.999	1<br>Olong_000091-R1 0.502 0.986 0.484 0.001 0.997<br>Olong_000093-R1 0.001 0.494 0.493 0.001 1	1<br>Opunc05g18050.1 0.024 0.492 0.468 0.031 0.839<br>Opunc11g18620.1 0.505 0.987 0.482 0.004 0.998	1<br>Obrac11g13000.2 0.506 0.989 0.483 0.006 0.999<br>Obrac11g13510.3 0.024 0.478 0.454 0.023 0.869	1<br>Lperr11g18720.1 0.505 0.995 0.49 0.002 0.997<br>Lperr11g19280.1 0.006 0.455 0.449 0.016 0.849	0<br>AT5G02800.1 0.585 0.998 0.413 0.153 0.989
Obart11g22180.1	0	1<br>Obart11g22180.1 0.001 1 0.999 0.001 1	0	1<br>Osind06g17590.1 0.038 0.984 0.946 0.037 0.999<br>Osind08g03120.1 0.028 0.98 0.952 0.059 0.995	0	1<br>Olong_000078-R1 0.029 0.872 0.843 0.013 0.995	0	0	0	0
	1<br>Oglab11g19350.1 0.001 0.374 0.373 0.002 0.896<br>Oglab11g19360.1 0.374 1 0.626 0.091 1	1<br>Obart11g22190.1 0.001 0.37 0.369 0.029 0.928<br>Obart11g22210.1 0.373 0.971 0.598 0.002 0.971	1<br>Osjap11g26070.1 0.374 1 0.626 0.095 1<br>Osjap11g26080.1 0.001 0.374 0.373 0.002 0.909	1<br>Osind01g01210.1 0.374 1 0.626 0.091 1<br>Osind01g30060.1 0.001 0.374 0.373 0.002 0.909	1<br>Orufi09g00060.1 0.374 1 0.626 0.11 1<br>Orufi09g00070.1 0.001 0.374 0.373 0.022 0.913	1<br>Olong_022538-R1 0.001 0.374 0.373 0.002 0.903<br>Olong_022539-R1 0.391 1 0.609 0.1 1	1<br>Opunc11g18540.2 0.009 0.369 0.36 0.022 0.956<br>Opunc11g18800.1 0.369 0.971 0.602 0.09 0.976	1<br>Obrac11g13060.1 0.004 0.374 0.37 0.251 0.937<br>Obrac11g13070.1 0.374 0.968 0.594 0.016 0.953	0<br>Lperr11g18890.1 0.374 0.977 0.603 0.119 0.991	0<br>AT5G57580.1 0.498 0.936 0.438 0.134 0.737
Obart12g01910.1	1<br>Oglab12g01890.1 0.002 0.938 0.936 0.002 0.974	1<br>Obart12g01910.1 0.002 1 0.998 0.002 1	0	0	0	0	1<br>Opunc11g02000.1 0.002 0.938 0.936 0.002 0.883<br>Opunc12g01920.1 0.002 0.938 0.936 0.002 0.951	1<br>Obrac11g01380.1 0.022 0.945 0.923 0.036 0.949	0	0
	1<br>Oglab11g01920.1 0.712 1 0.288 0.005 1<br>Oglab11g01930.1 0.013 0.691 0.678 0.051 0.947	1<br>Obart01g34520.1 0.71 1 0.29 0.005 1<br>Obart11g02130.1 0.013 0.691 0.678 0.051 0.947	1<br>Osjap11g02310.1 0.712 1 0.288 0.005 1<br>Osjap12g02360.1 0.002 0.691 0.689 0.002 0.945	1<br>Osind11g01670.1 0.712 0.997 0.285 0.004 0.742<br>Osind12g02620.1 0.002 0.691 0.689 0.002 0.945	1<br>Orufi01g37930.2 0.71 1 0.29 0.005 1<br>Orufi12g02190.1 0.022 0.6 0.578 0.159 1	1<br>Olong_019069-R2 0.002 0.691 0.689 0.002 0.945<br>Olong_020510-R1 0.71 1 0.29 0.005 1	0<br>Opunc03g30920.1 0.688 0.949 0.261 0.471 0.991	1<br>Obrac12g01690.1 0.712 1 0.288 0.005 1<br>Obrac12g01700.2 0.002 0.691 0.689 0.002 0.948	1<br>Lperr07g20930.1 0.025 0.691 0.666 0.09 0.956<br>Lperr12g02000.1 0.709 1 0.291 0.866 1	1<br>AT2G19540.1 0.022 0.691 0.669 0.062 0.947<br>AT2G27030.3 0.71 1 0.29 0.006 1
Obart12g02080.1	1<br>Oglab11g02200.1 0.002 1 0.998 0.023 1<br>Oglab12g02130.1 0.002 1 0.998 0.022 1	1<br>Obart12g02080.1 0.002 1 0.998 0.002 1	1<br>Osjap11g02640.1 0.002 1 0.998 0.024 1<br>Osjap12g02610.1 0.002 1 0.998 0.022 1	1<br>Osind11g01990.1 0.002 1 0.998 0.023 1<br>Osind12g02780.1 0.002 1 0.998 0.022 1	1<br>Orufi11g02320.1 0.002 1 0.998 0.003 1<br>Orufi12g02480.1 0.002 1 0.998 0.021 1	0	0	0	0	0
	1<br>Oglab12g02120.1 0.002 0.64 0.638 0.006 1<br>Oglab12g09880.1 0.644 0.948 0.304 0.051 0.385	0<br>Obart11g02350.1 0.002 0.64 0.638 0.004 0.993	1<br>Osjap12g02600.1 0.002 0.64 0.638 0.006 1<br>Osjap12g12870.1 0.644 0.948 0.304 0.055 0.417	1<br>Osind11g01980.1 0.002 0.64 0.638 0.006 1<br>Osind12g10020.1 0.644 0.948 0.304 0.055 0.417	1<br>Orufi12g02470.1 0.002 0.64 0.638 0.006 1<br>Orufi12g10750.1 0.642 0.948 0.306 0.045 0.364	1<br>Olong_003435-R1 0.133 0.64 0.507 0.212 0.984<br>Olong_023179-R1 0.642 0.998 0.356 0.05 0.329	1<br>Opunc11g02180.1 0.002 0.64 0.638 0.028 0.992<br>Opunc12g08740.1 0.642 0.948 0.306 0.04 0.324	0<br>Obrac05g10860.1 0.072 0.523 0.451 0.097 0.99<br>Obrac12g07170.1 0.642 0.974 0.332 0.071 0.67	1<br>Lperr12g02190.1 0.002 0.64 0.638 0.033 0.992<br>Lperr12g08390.1 0.642 0.956 0.314 0.056 0.454	0<br>AT3G27320.1 0.052 0.421 0.369 0.596 0.978
Obart12g02370.1	0	1<br>Obart12g02370.1 0.001 1 0.999 0.001 1	0	0	0	0	0	0	0	0
	1<br>Oglab12g02420.1 0.492 1 0.508 0.283 1<br>Oglab12g02460.1 0.001 0.434 0.433 0.002 0.822	1<br>Obart11g02670.1 0.492 1 0.508 0.277 1<br>Obart11g02690.1 0.001 0.434 0.433 0.003 0.982	1<br>Osjap11g03000.1 0.001 0.434 0.433 0.002 0.986<br>Osjap12g02930.1 0.492 1 0.508 0.286 1	1<br>Osind12g02950.1 0.492 1 0.508 0.286 1<br>Osind12g02970.1 0.001 0.434 0.433 0.002 0.935	1<br>Orufi11g02630.1 0.492 1 0.508 0.063 1<br>Orufi12g02750.1 0.075 0.434 0.359 0.003 0.907	0<br>Olong_011875-R1 0.103 0.411 0.308 0.161 0.908<br>Olong_028720-R1 0.557 1 0.443 0.003 1	1<br>Opunc11g02440.1 0.492 1 0.508 0.284 1<br>Opunc11g02450.1 0.001 0.434 0.433 0.002 0.932	0<br>Obrac11g01640.1 0.51 1 0.49 0.034 0.766	0<br>Lperr12g02500.1 0.147 0.432 0.285 0.144 0.868	0<br>AT3G47930.1 0.492 0.993 0.501 0.311 0.995

Obart12g02600.1	1<br>Oglab12g02710.1 0.003 1 0.997 0.004 1	1<br>Obart12g02600.1 0.003 1 0.997 0.003 1	0	0	1<br>Orufi12g03000.1 0.003 0.978 0.975 0.004 0.823	0	0	0	0	0
	1<br>Oglab11g14940.1 0.653 0.959 0.306 0.011 1<br>Oglab12g02550.1 0.009 0.581 0.572 0.02 0.975	1<br>Obart01g16130.1 0.588 1 0.412 0.209 1<br>Obart12g02460.1 0.009 0.581 0.572 0.02 0.975	1<br>Osjap01g18900.1 0.588 1 0.412 0.308 1<br>Osjap12g03070.1 0.009 0.581 0.572 0.02 0.975	1<br>Osind12g03230.1 0.606 1 0.394 0.292 1<br>Osind12g03240.1 0.003 0.588 0.585 0.008 1	1<br>Orufi02g31540.1 0.744 0.988 0.244 0.399 0.957<br>Orufi12g02840.1 0.009 0.581 0.572 0.02 0.975	1<br>Olong_021782-R1 0.713 0.975 0.262 0.051 0.859<br>Olong_027258-R1 0.009 0.581 0.572 0.28 0.981	0<br>Opunc02g10450.1 0.694 1 0.306 0.24 1<br>Opunc10g12380.1 0.297 0.641 0.344 0.499 0.843	0<br>Obrac11g01600.1 0.231 0.578 0.347 0.076 0.91	0<br>Lperr02g24330.1 0.694 0.919 0.225 0.287 0.816<br>Lperr02g27700.1 0.297 0.603 0.306 0.415 0.71	0<br>AT1G68180.1 0.272 0.584 0.312 0.31 0.746
Obart12g02950.1	1<br>Oglab12g03070.1 0.001 1 0.999 0.001 1	1<br>Obart12g02950.1 0.001 1 0.999 0.001 1	0	1<br>Osind12g03620.1 0.001 0.999 0.998 0.029 0.961	1<br>Orufi12g03350.1 0.011 0.999 0.988 0.044 0.966<br>Orufi12g03350.2 0.011 0.999 0.988 0.043 0.966	1<br>Olong_000549-R1 0.011 0.999 0.988 0.011 0.967	0	0	0	0
	1<br>Oglab10g13340.3 0.559 0.991 0.432 0.213 0.951<br>Oglab12g02020.1 0.002 0.453 0.451 0.001 0.388	0<br>Obart11g02240.1 0.193 0.453 0.26 0.014 0.272<br>Obart11g21270.1 0.494 0.985 0.491 0.138 0.958	1<br>Osjap10g20010.1 0.498 0.991 0.493 0.022 0.946<br>Osjap12g02500.1 0.002 0.453 0.451 0.001 0.388	1<br>Osind01g01580.1 0.492 0.985 0.493 0.171 0.96<br>Osind12g02670.1 0.002 0.453 0.451 0.001 0.396	1<br>Orufi04g25720.1 0.498 0.871 0.373 0.002 0.207<br>Orufi11g02210.1 0.015 0.453 0.438 0.015 0.492	0<br>Olong_010277-R4 0.488 0.871 0.383 0.001 0.93<br>Olong_027241-R1 0.046 0.305 0.259 0.003 0.982	1<br>Opunc11g17890.2 0.491 0.985 0.494 0.189 0.952<br>Opunc12g02040.1 0.012 0.453 0.441 0.009 0.402	1<br>Obrac11g01490.1 0.002 0.453 0.451 0.029 0.403<br>Obrac11g12600.2 0.547 0.985 0.438 0.046 0.748	0<br>Lperr11g01950.1 0.011 0.228 0.217 0.015 0.297<br>Lperr11g17840.1 0.5 0.985 0.485 0.046 0.945	1<br>AT1G65810.1 0.498 0.871 0.373 0.021 0.596<br>AT2G26780.1 0.017 0.449 0.432 0.011 0.388
Obart12g03260.1	0	1<br>Obart12g03260.1 0.002 1 0.998 0.002 1	0	0	0	0	0	0	0	0
	1<br>Oglab03g28380.1 0.002 0.382 0.38 0.835 0.983<br>Oglab12g03430.1 0.382 0.835 0.453 0.004 0.758	0<br>Obart03g29120.1 0.002 0.382 0.38 0.835 0.983<br>Obart08g17720.1 0.434 0.833 0.399 0.105 0.76	1<br>Osjap03g31670.3 0.002 0.382 0.38 0.85 0.984<br>Osjap12g03930.1 0.382 0.835 0.453 0.004 0.758	1<br>Osind03g32640.1 0.002 0.382 0.38 0.835 0.983<br>Osind12g03880.1 0.382 0.835 0.453 0.004 0.758	0<br>Orufi12g03700.1 0.382 0.835 0.453 0.004 0.758	1<br>Olong_000521-R1 0.382 0.816 0.434 0.004 0.806<br>Olong_002508-R1 0.002 0.382 0.38 0.835 0.983	1<br>Opunc12g03040.1 0.002 0.382 0.38 0.823 0.967<br>Opunc12g03050.1 0.382 0.835 0.453 0.004 0.763	0<br>Obrac02g13530.3 0.468 0.891 0.423 0.273 0.927<br>Obrac12g02570.1 0.002 0.378 0.376 0.835 0.98	1<br>Lperr06g08630.1 0.002 0.382 0.38 0.835 0.983<br>Lperr12g03100.1 0.382 0.833 0.451 0.004 0.757	0<br>AT5G04180.1 0.391 0.833 0.442 0.007 0.722
Obart12g09410.1	1<br>Oglab12g09470.1 0.001 1 0.999 0.001 1	1<br>Obart12g09410.1 0.001 1 0.999 0.001 1	1<br>Osjap12g12320.1 0.001 1 0.999 0.001 1	1<br>Osind12g09530.1 0.001 1 0.999 0.001 1	1<br>Orufi12g10460.1 0.001 1 0.999 0.001 1	0	0	0	0	0
	1<br>Oglab01g34040.1 0.477 0.998 0.521 0.203 0.995<br>Oglab03g07280.1 0.001 0.306 0.305 0.004 0.996	1<br>Obart01g39260.2 0.452 0.998 0.546 0.017 0.994<br>Obart03g07210.1 0.001 0.306 0.305 0.004 0.996	1<br>Osjap01g44780.3 0.452 0.998 0.546 0.017 0.994<br>Osjap03g07600.1 0.001 0.306 0.305 0.004 0.996	1<br>Osind01g46040.1 0.452 0.998 0.546 0.186 0.995<br>Osind03g07670.1 0.001 0.306 0.305 0.004 0.996	1<br>Orufi01g42650.3 0.452 0.998 0.546 0.228 0.995<br>Orufi03g07180.1 0.001 0.306 0.305 0.004 0.996	1<br>Olong_021912-R1 0.376 1 0.624 0.002 1<br>Olong_021913-R1 0.001 0.305 0.304 0.004 0.996	1<br>Opunc12g08570.1 0.406 1 0.594 0.152 1<br>Opunc12g08580.1 0.001 0.305 0.304 0.004 0.996	1<br>Obrac12g06490.1 0.001 0.305 0.304 0.004 0.996<br>Obrac12g06500.1 0.449 1 0.551 0.103 1	1<br>Lperr03g06600.1 0.001 0.304 0.303 0.004 0.992<br>Lperr12g08310.1 0.452 0.999 0.547 0.002 0.998	1<br>AT4G34670.1 0.001 0.297 0.296 0.004 0.966<br>AT5G67540.2 0.477 0.993 0.516 0.053 1
Obart12g13650.1	0	1<br>Obart12g13650.1 0.004 1 0.996 0.004 1	0	0	0	0	0	0	0	0
	1<br>Oglab11g18280.1 0.79 1 0.21 0.514 1<br>Oglab12g12900.1 0.004 0.786 0.782 0.005 1	1<br>Obart03g06960.1 0.028 0.762 0.734 0.145 0.991<br>Obart11g20740.1 0.79 1 0.21 0.514 1	1<br>Osjap07g23370.2 0.79 0.992 0.202 0.086 0.168<br>Osjap12g17390.1 0.004 0.786 0.782 0.005 1	1<br>Osind11g16870.1 0.79 0.996 0.206 0.143 0.968<br>Osind12g14200.1 0.004 0.786 0.782 0.005 1	1<br>Orufi07g20930.2 0.79 0.992 0.202 0.087 0.169<br>Orufi12g14980.1 0.004 0.786 0.782 0.005 1	0<br>Olong_014123-R1 0.024 0.69 0.666 0.098 0.995	1<br>Opunc07g18980.2 0.79 1 0.21 0.014 0.105<br>Opunc12g12320.1 0.012 0.786 0.774 0.01 1	1<br>Obrac03g05620.1 0.056 0.706 0.65 0.158 0.937<br>Obrac12g08990.2 0.754 0.976 0.222 0.26 0.844	1<br>Lperr11g17030.1 0.79 1 0.21 0.116 0.884<br>Lperr12g11520.1 0.02 0.698 0.678 0.005 0.88	0<br>AT4G25830.1 0.04 0.746 0.706 0.017 1
Obart12g18260.1	0	1<br>Obart12g18260.1 0.002 1 0.998 0.002 1	0	0	0	0	1<br>Opunc12g16510.1 0.029 1 0.971 0.374 1	0	1<br>Lperr12g15270.1 0.002 1 0.998 0.002 1<br>Lperr12g15270.2 0.002 1 0.998 0.003 1	0
	1<br>Oglab03g30800.1 0.002 0.282 0.28 0.007 0.762<br>Oglab12g16450.1 0.402 1 0.598 0.42 1	1<br>Obart05g22220.1 0.431 1 0.569 0.47 1<br>Obart09g04390.1 0.002 0.282 0.28 0.02 0.77	1<br>Osjap03g34300.1 0.002 0.282 0.28 0.437 0.827<br>Osjap12g23030.1 0.402 1 0.598 0.42 1	1<br>Osind12g19080.1 0.402 1 0.598 0.42 1<br>Osind12g19090.1 0.002 0.282 0.28 0.007 0.767	1<br>Orufi09g04980.1 0.002 0.282 0.28 0.02 0.77<br>Orufi12g20240.1 0.402 1 0.598 0.42 1	1<br>Olong_014799-R1 0.002 0.282 0.28 0.007 0.8<br>Olong_025924-R1 0.402 1 0.598 0.195 1	1<br>Opunc05g19900.1 0.431 1 0.569 0.452 1<br>Opunc09g03850.1 0.002 0.282 0.28 0.02 0.77	1<br>Obrac03g23420.1 0.002 0.282 0.28 0.006 0.696<br>Obrac12g11780.1 0.404 1 0.596 0.236 1	1<br>Lperr03g26330.1 0.002 0.282 0.28 0.339 0.837<br>Lperr05g18130.1 0.431 1 0.569 0.387 1	0<br>AT1G23260.1 0.01 0.282 0.272 0.013 0.696<br>AT4G14770.1 0.431 0.652 0.221 0.682 0.818

((((((_b1,_a2),longi),punctata),brachy),leersia),tair);

chimeric gene id	INGROUP		MIDGROUP					OUTGROUP		
	glab	barthii	jap	indica	rufi	longi	punctata	brachy	leersia	tair
Obart01g01710.1	1<br>Oglab01g01590.1 0.001 0.986 0.985 0.001 0.987	1<br>Obart01g01710.1 0.001 1 0.999 0.001 1	0	1<br>Osind01g02220.1 0.001 0.986 0.985 0.056 0.987	0	1<br>Olong_021362-R1 0.001 0.986 0.985 0.048 0.986	1<br>Opunc01g01490.1 0.002 1 0.998 0.021 0.999<br>Opunc01g01490.2 0.002 1 0.998 0.024 0.999	0	0	0
	1<br>Oglab01g01570.1 0.539 0.986 0.447 0.043 0.973<br>Oglab05g03370.1 0.002 0.47 0.468 0.104 0.976	1<br>Obart01g01680.1 0.539 0.986 0.447 0.046 0.97<br>Obart05g03720.1 0.002 0.47 0.468 0.104 0.976	1<br>Osjap01g02100.1 0.001 0.47 0.469 0.002 0.973<br>Osjap01g02110.1 0.513 0.986 0.473 0.039 0.974	1<br>Osind01g02230.1 0.513 0.999 0.486 0.061 0.989<br>Osind05g03930.1 0.002 0.47 0.468 0.104 0.976	1<br>Orufi01g01810.1 0.001 0.47 0.469 0.002 0.973<br>Orufi01g01830.1 0.539 0.986 0.447 0.079 0.987	1<br>Olong_016471-R1 0.002 0.47 0.468 0.106 0.972<br>Olong_021361-R1 0.515 0.955 0.44 0.002 0.971	1<br>Opunc01g01490.3 0.539 0.986 0.447 0.056 0.97<br>Opunc05g03460.1 0.016 0.47 0.454 0.119 0.971	1<br>Obrac01g01340.1 0.486 0.986 0.5 0.001 0.999<br>Obrac01g17910.1 0.004 0.47 0.466 0.248 0.98	1<br>Lperr01g01640.1 0.51 0.986 0.476 0.051 0.975<br>Lperr05g03400.1 0.015 0.47 0.455 0.158 0.972	1<br>AT1G71040.1 0.528 0.986 0.458 0.021 0.972<br>AT3G48670.1 0.002 0.47 0.468 0.117 0.971
Obart01g02790.1	1<br>Oglab01g02400.1 0.03 1 0.97 0.135 1	1<br>Obart01g02790.1 0.001 1 0.999 0.001 1	0	0	0	0	1<br>Opunc01g02430.1 0.011 0.932 0.921 0.058 0.93	0	0	0
	1<br>Oglab01g02390.1 0.001 0.222 0.221 0.004 0.992<br>Oglab09g10930.1 0.236 0.954 0.718 0.008 0.94	0<br>Obart09g11930.1 0.236 0.954 0.718 0.008 0.94	1<br>Osjap01g03230.1 0.234 1 0.766 0.011 1<br>Osjap01g03250.1 0.001 0.223 0.222 0.004 0.996	1<br>Osind01g03370.1 0.001 0.222 0.221 0.004 0.992<br>Osind09g12280.1 0.236 0.954 0.718 0.008 0.948	1<br>Orufi01g02840.1 0.237 1 0.763 0.001 1<br>Orufi01g02850.1 0.001 0.223 0.222 0.004 0.996	0<br>Olong_016492-R1 0.041 0.82 0.779 0.023 0.979	1<br>Opunc01g02420.1 0.001 0.222 0.221 0.004 0.992<br>Opunc02g11760.1 0.239 0.947 0.708 0.026 0.984	0<br>Obrac01g02250.1 0.234 0.975 0.741 0.004 0.987	0<br>Lperr01g02550.1 0.237 0.975 0.738 0.01 0.997	0<br>AT2G39795.1 0.002 0.224 0.222 0.1 1<br>AT5G45480.1 0.671 0.956 0.285 0.656 0.991
Obart01g03110.1	0	1<br>Obart01g03110.1 0.001 1 0.999 0.001 1	0	0	0	0	0	0	1<br>Lperr01g02790.1 0.063 1 0.937 0.043 1<br>Lperr01g02790.2 0.063 1 0.937 0.042 1	0
	1<br>Oglab01g02690.1 0.425 1 0.575 0.077 1<br>Oglab01g02700.1 0.063 0.341 0.278 0.13 0.996	0<br>Obart09g17680.1 0.072 0.338 0.266 0.289 0.98<br>Obart10g18430.1 0.766 0.999 0.233 0.695 0.986	1<br>Osjap01g03540.1 0.395 1 0.605 0.016 1<br>Osjap01g03550.1 0.063 0.341 0.278 0.13 0.996	1<br>Osind01g03690.1 0.425 1 0.575 0.072 1<br>Osind01g03700.1 0.063 0.341 0.278 0.13 0.996	1<br>Orufi01g03140.1 0.425 1 0.575 0.073 1<br>Orufi01g03150.1 0.063 0.341 0.278 0.13 0.996	1<br>Olong_018924-R1 0.425 1 0.575 0.002 1<br>Olong_018925-R1 0.063 0.341 0.278 0.13 0.996	1<br>Opunc01g02730.1 0.423 0.959 0.536 0.077 0.955<br>Opunc09g16060.1 0.072 0.338 0.266 0.363 0.982	1<br>Obrac01g02390.1 0.412 0.956 0.544 0.071 0.967<br>Obrac01g28390.1 0.066 0.341 0.275 0.141 0.992	0<br>Lperr09g09500.1 0.782 0.997 0.215 0.722 0.997<br>Lperr09g13570.1 0.072 0.338 0.266 0.299 0.981	1<br>AT2G39690.1 0.458 1 0.542 0.006 1<br>AT3G55440.1 0.067 0.339 0.272 0.142 0.984
Obart01g04220.1	1<br>Oglab01g03790.1 0.001 1 0.999 0.001 1	1<br>Obart01g04220.1 0.001 1 0.999 0.001 1	0	0	0	0	0	0	0	0
	1<br>Oglab05g04170.2 0.048 0.567 0.519 0.114 1<br>Oglab07g07650.1 0.62 0.997 0.377 0.002 0.778	1<br>Obart05g04510.1 0.036 0.567 0.531 0.012 1<br>Obart07g07920.1 0.62 0.997 0.377 0.002 0.778	1<br>Osjap01g04780.1 0.62 1 0.38 0.002 1<br>Osjap05g04880.1 0.048 0.567 0.519 0.008 1	1<br>Osind01g04880.1 0.62 1 0.38 0.002 1<br>Osind05g04760.1 0.048 0.567 0.519 0.008 1	1<br>Orufi01g04370.1 0.62 1 0.38 0.002 1<br>Orufi05g04740.1 0.048 0.567 0.519 0.008 1	1<br>Olong_014225-R1 0.044 0.563 0.519 0.001 0.984<br>Olong_014227-R1 0.62 1 0.38 0.002 1	1<br>Opunc01g03980.1 0.62 1 0.38 0.002 1<br>Opunc07g16230.1 0.042 0.556 0.514 0.014 0.972	1<br>Obrac01g03070.1 0.62 1 0.38 0.002 1<br>Obrac05g03450.2 0.039 0.567 0.528 0.003 1	1<br>Lperr01g03770.1 0.62 1 0.38 0.002 1<br>Lperr01g03780.1 0.039 0.561 0.522 0.002 0.979	1<br>AT5G01660.1 0.053 0.556 0.503 0.015 0.97<br>AT5G64190.1 0.62 0.997 0.377 0.002 0.998
Obart01g07430.1	1<br>Oglab01g06470.1 0.013 1 0.987 0.02 1	1<br>Obart01g07430.1 0.001 1 0.999 0.001 1	1<br>Osjap01g08660.1 0.007 1 0.993 0.026 1	1<br>Osind01g08490.1 0.007 1 0.993 0.026 1	1<br>Orufi01g08070.1 0.007 1 0.993 0.026 1	1<br>Olong_011567-R1 0.036 1 0.964 0.001 1	0	0	0	0
	1<br>Oglab01g21070.2 0.078 0.648 0.57 0.249 0.983<br>Oglab05g00530.1 0.713 0.997 0.284 0.146 1	1<br>Obart01g25090.1 0.078 0.648 0.57 0.219 0.982<br>Obart05g00540.1 0.713 0.997 0.284 0.146 1	1<br>Osjap01g29230.2 0.078 0.648 0.57 0.25 0.983<br>Osjap05g00560.2 0.713 0.997 0.284 0.146 1	1<br>Osind01g30690.1 0.078 0.648 0.57 0.267 0.982<br>Osind05g00540.1 0.713 0.997 0.284 0.126 1	1<br>Orufi01g27950.1 0.078 0.648 0.57 0.25 0.983<br>Orufi01g48560.2 0.713 0.983 0.27 0.085 0.984	1<br>Olong_003039-R1 0.713 0.997 0.284 0.128 1<br>Olong_003692-R2 0.109 0.648 0.539 0.244 0.979	1<br>Opunc01g07160.1 0.656 1 0.344 0.111 1<br>Opunc01g07170.1 0.003 0.649 0.646 0.034 0.984	1<br>Obrac01g05760.3 0.66 1 0.34 0.082 1<br>Obrac01g18840.2 0.001 0.648 0.647 0.062 0.974	1<br>Lperr01g07030.1 0.66 1 0.34 0.018 1<br>Lperr01g07040.2 0.002 0.649 0.647 0.013 0.989	1<br>AT1G54350.1 0.659 0.994 0.335 0.076 0.975<br>AT3G61740.1 0.009 0.648 0.639 0.077 0.983
Obart01g25040.1	0	1	0	1	1	1	0	0	0	0

		Obart01g25040.1 0.003 1 0.997 0.003 1		Osind01g30660.1 0.003 1 0.997 0.003 1	Orufi01g27920.1 0.003 0.894 0.891 0.003 0.862	Olong_017584-R1 0.171 1 0.829 0.059 1				
	0<br>Oglab01g21050.2 0.003 0.711 0.708 0.005 0.995	1<br>Obart01g25070.1 0.284 0.987 0.703 0.125 0.979<br>Obart03g33060.1 0.003 0.227 0.224 0.013 0.961	1<br>Osjap01g29220.1 0.284 0.987 0.703 0.12 0.979<br>Osjap03g35690.1 0.003 0.227 0.224 0.013 0.961	1<br>Osind01g30680.1 0.284 0.987 0.703 0.12 0.979<br>Osind03g36920.1 0.003 0.227 0.224 0.013 0.961	1<br>Orufi01g27940.1 0.284 0.987 0.703 0.12 0.979<br>Orufi03g34430.1 0.003 0.227 0.224 0.013 0.961	0<br>Olong_003693-R1 0.284 0.987 0.703 0.127 0.978	1<br>Opunc01g24980.1 0.3 0.987 0.687 0.174 0.975<br>Opunc03g30200.1 0.003 0.227 0.224 0.013 0.961	1<br>Obrac01g18810.1 0.258 0.99 0.732 0.103 0.996<br>Obrac03g24520.1 0.003 0.227 0.224 0.013 0.961	0	0
Obart01g26420.1	0	1<br>Obart01g26420.1 0.002 1 0.998 0.002 1<br>Obart01g26420.2 0.002 1 0.998 0.002 1	0	0	1<br>Orufi01g29390.1 0.002 1 0.998 0.002 1	0	0	0	0	0
	1<br>Oglab01g22320.1 0.002 0.585 0.583 0.003 0.997<br>Oglab07g18060.1 0.588 0.907 0.319 0.202 0.904	1<br>Obart07g20970.1 0.588 0.828 0.24 0.185 0.779<br>Obart11g13020.1 0.002 0.574 0.572 0.006 1	1<br>Osjap01g30690.1 0.002 0.585 0.583 0.003 0.997<br>Osjap07g24440.1 0.588 0.907 0.319 0.16 0.718	1<br>Osind01g32110.1 0.002 0.585 0.583 0.003 0.997<br>Osind07g21300.1 0.588 0.907 0.319 0.159 0.713	1<br>Orufi07g21990.1 0.588 0.852 0.264 0.175 0.751<br>Orufi11g13800.1 0.002 0.574 0.572 0.006 1	1<br>Olong_006531-R1 0.588 0.907 0.319 0.176 0.786<br>Olong_023995-R1 0.002 0.585 0.583 0.005 0.995	1<br>Opunc01g26380.1 0.002 0.585 0.583 0.003 0.997<br>Opunc07g12980.1 0.588 0.878 0.29 0.158 0.699	1<br>Obrac01g20020.1 0.002 0.585 0.583 0.003 0.997<br>Obrac02g00620.1 0.59 0.83 0.24 0.16 0.42	1<br>Lperr01g22600.1 0.002 0.585 0.583 0.003 0.997<br>Lperr07g18200.1 0.587 0.852 0.265 0.16 0.7	0<br>AT1G52155.1 0.013 0.553 0.54 0.022 0.997<br>AT4G20140.1 0.579 0.82 0.241 0.155 0.649
Obart01g26420.2	0	1<br>Obart01g26420.1 0.002 1 0.998 0.002 1<br>Obart01g26420.2 0.002 1 0.998 0.002 1	0	0	1<br>Orufi01g29390.1 0.002 1 0.998 0.002 1	0	0	0	0	0
	1<br>Oglab01g22320.1 0.002 0.563 0.561 0.003 0.997<br>Oglab07g18060.1 0.566 0.902 0.336 0.202 0.904	1<br>Obart07g20970.1 0.566 0.819 0.253 0.185 0.779<br>Obart11g13020.1 0.002 0.551 0.549 0.006 1	1<br>Osjap01g30690.1 0.002 0.563 0.561 0.003 0.997<br>Osjap07g24440.1 0.566 0.902 0.336 0.16 0.718	1<br>Osind01g32110.1 0.002 0.563 0.561 0.003 0.997<br>Osind07g21300.1 0.566 0.902 0.336 0.159 0.713	1<br>Orufi07g21990.1 0.566 0.844 0.278 0.175 0.751<br>Orufi11g13800.1 0.002 0.551 0.549 0.006 1	1<br>Olong_006531-R1 0.566 0.902 0.336 0.176 0.786<br>Olong_023995-R1 0.002 0.563 0.561 0.005 0.995	1<br>Opunc01g26380.1 0.002 0.563 0.561 0.003 0.997<br>Opunc07g12980.1 0.564 0.871 0.307 0.134 0.699	1<br>Obrac01g20020.1 0.002 0.563 0.561 0.003 0.997<br>Obrac02g00620.1 0.568 0.82 0.252 0.16 0.42	0<br>Lperr01g22600.1 0.002 0.563 0.561 0.003 0.997<br>Lperr08g10350.1 0.564 0.768 0.204 0.191 0.711	0<br>AT1G52155.1 0.014 0.529 0.515 0.022 0.997<br>AT4G20140.1 0.556 0.81 0.254 0.155 0.649
Obart01g26930.1	0	1<br>Obart01g26930.1 0.001 1 0.999 0.001 1	0	0	0	0	0	0	0	0
	1<br>Oglab01g22800.1 0.001 0.395 0.394 0.002 1<br>Oglab01g22810.1 0.406 0.959 0.553 0.148 0.999	1<br>Obart03g32590.1 0.001 0.39 0.389 0.002 0.921<br>Obart07g24100.1 0.404 0.927 0.523 0.166 0.828	1<br>Osjap01g31200.1 0.001 0.395 0.394 0.002 1<br>Osjap01g31210.1 0.404 0.959 0.555 0.2 0.999	1<br>Osind01g32660.1 0.001 0.395 0.394 0.002 0.972<br>Osind01g32670.1 0.404 0.959 0.555 0.198 0.999	1<br>Orufi01g29940.1 0.001 0.395 0.394 0.002 1<br>Orufi07g25280.1 0.404 0.927 0.523 0.17 0.829	1<br>Olong_000370-R1 0.001 0.395 0.394 0.426 1<br>Olong_012653-R1 0.404 0.927 0.523 0.168 0.829	1<br>Opunc01g26880.1 0.404 0.962 0.558 0.076 1<br>Opunc03g31650.1 0.01 0.393 0.383 0.017 0.936	1<br>Obrac01g20460.1 0.404 0.959 0.555 0.01 0.998<br>Obrac03g24030.1 0.001 0.366 0.365 0.002 0.841	1<br>Lperr03g07390.1 0.099 0.378 0.279 0.169 0.842<br>Lperr06g07900.1 0.404 0.927 0.523 0.173 0.936	0<br>AT5G28740.1 0.404 0.927 0.523 0.149 0.83
Obart01g27860.1	0	1<br>Obart01g27860.1 0.001 1 0.999 0.001 1<br>Obart01g27860.2 0.001 1 0.999 0.001 1	0	0	0	0	0	0	1<br>Lperr01g24010.1 0.006 0.997 0.991 0.012 0.792<br>Lperr01g24010.2 0.006 0.997 0.991 0.012 0.788	0
	1<br>Oglab01g23700.4 0.001 0.743 0.742 0.001 0.999<br>Oglab08g19780.2 0.744 0.999 0.255 0.079 0.791	0<br>Obart05g24550.1 0.734 0.997 0.263 0.065 0.923	1<br>Osjap01g32230.6 0.001 0.743 0.742 0.001 0.999<br>Osjap08g22350.1 0.744 0.999 0.255 0.077 0.797	1<br>Osind01g33730.2 0.001 0.743 0.742 0.001 0.999<br>Osind08g24520.1 0.744 0.999 0.255 0.079 0.791	0<br>Orufi01g30920.4 0.001 0.743 0.742 0.001 0.999	1<br>Olong_024789-R1 0.001 0.743 0.742 0.001 0.999<br>Olong_025647-R1 0.744 0.999 0.255 0.083 0.778	1<br>Opunc01g27750.2 0.001 0.743 0.742 0.001 0.999<br>Opunc09g13460.1 0.744 0.999 0.255 0.081 0.783	1<br>Obrac01g21170.1 0.001 0.743 0.742 0.001 0.999<br>Obrac08g13970.1 0.744 0.999 0.255 0.077 0.793	0<br>Lperr09g11160.1 0.744 0.999 0.255 0.075 0.831	1<br>AT3G49400.1 0.001 0.743 0.742 0.001 0.999<br>AT4G39150.1 0.744 0.997 0.253 0.087 0.907
Obart01g27860.2	0	1<br>Obart01g27860.1 0.001 1 0.999 0.001 1<br>Obart01g27860.2 0.001 1 0.999 0.001 1	0	0	0	0	0	0	1<br>Lperr01g24010.1 0.006 0.997 0.991 0.012 0.792<br>Lperr01g24010.2 0.006 0.997 0.991 0.012 0.788	0
	1<br>Oglab01g23700.4 0.001 0.743 0.742 0.001 0.999<br>Oglab08g19780.2 0.744 0.999 0.255 0.079 0.791	0<br>Obart05g24550.1 0.734 0.997 0.263 0.065 0.923	1<br>Osjap01g32230.6 0.001 0.743 0.742 0.001 0.999<br>Osjap08g22350.1 0.744 0.999 0.255 0.077 0.797	1<br>Osind01g33730.2 0.001 0.743 0.742 0.001 0.999<br>Osind08g24520.1 0.744 0.999 0.255 0.079 0.791	0<br>Orufi01g30920.4 0.001 0.743 0.742 0.001 0.999	1<br>Olong_024789-R1 0.001 0.743 0.742 0.001 0.999<br>Olong_025647-R1 0.744 0.999 0.255 0.083 0.778	1<br>Opunc01g27750.2 0.001 0.743 0.742 0.001 0.999<br>Opunc09g13460.1 0.744 0.999 0.255 0.081 0.783	1<br>Obrac01g21170.1 0.001 0.743 0.742 0.001 0.999<br>Obrac08g13970.1 0.744 0.999 0.255 0.077 0.793	0<br>Lperr09g11160.1 0.744 0.999 0.255 0.075 0.831	1<br>AT3G49400.1 0.001 0.743 0.742 0.001 0.999<br>AT4G39150.1 0.744 0.997 0.253 0.087 0.907

Obart01g33610.1	1<br>Oglab01g28270.1 0.001 1 0.999 0.022 1	1<br>Obart01g33610.1 0.001 1 0.999 0.001 1	0	0	0	0	0	0	0	0
	1<br>Oglab03g01040.1 0.002 0.213 0.211 0.039 0.99<br>Oglab10g10080.1 0.361 0.998 0.637 0.138 0.999	0<br>Obart10g10470.1 0.361 0.998 0.637 0.121 0.999	1<br>Osjap01g38450.1 0.294 1 0.706 0.022 1<br>Osjap03g01290.1 0.002 0.218 0.216 0.023 0.185	1<br>Osind01g39980.1 0.258 1 0.742 0.117 1<br>Osind01g39990.1 0.001 0.213 0.212 0.095 0.991	1<br>Orufi01g36790.1 0.294 1 0.706 0.022 1<br>Orufi01g36800.1 0.001 0.213 0.212 0.095 0.991	0<br>Olong_007589-R1 0.361 0.998 0.637 0.19 0.999	1<br>Opunc01g32680.1 0.294 1 0.706 0.022 1<br>Opunc01g32690.1 0.001 0.213 0.212 0.1 0.991	1<br>Obrac01g25800.1 0.294 1 0.706 0.022 1<br>Obrac03g00890.1 0.002 0.221 0.219 0.007 0.192	1<br>Lperr01g29020.1 0.279 1 0.721 0.001 1<br>Lperr01g29030.1 0.001 0.213 0.212 0.091 0.991	1<br>AT1G69290.1 0.316 1 0.684 0.036 1<br>AT3G13230.1 0.005 0.213 0.208 0.102 1
Obart01g40120.2	1<br>Oglab01g34910.1 0.002 1 0.998 0.002 1	1<br>Obart01g40120.1 0.002 1 0.998 0.002 1<br>Obart01g40120.2 0.002 1 0.998 0.002 1	1<br>Osjap01g45670.1 0.002 1 0.998 0.003 1	1<br>Osind01g46990.1 0.002 1 0.998 0.002 1	1<br>Orufi01g43430.1 0.123 1 0.877 0.351 1	1<br>Olong_025215-R1 0.152 0.975 0.823 0.002 0.977	0	0	0	0
	1<br>Oglab01g34890.1 0.123 0.39 0.267 0.646 0.992<br>Oglab11g07730.1 0.439 0.993 0.554 0.021 0.416	0<br>Obart11g08370.2 0.377 0.993 0.616 0.022 0.446	1<br>Osjap01g45650.1 0.123 0.39 0.267 0.646 0.992<br>Osjap11g09760.2 0.439 0.993 0.554 0.021 0.416	1<br>Osind01g46870.1 0.002 0.285 0.283 0.007 0.986<br>Osind11g07730.1 0.453 0.993 0.54 0.002 0.401	0<br>Orufi01g43430.3 0.285 1 0.715 0.045 1	1<br>Olong_009328-R1 0.123 0.37 0.247 0.649 0.983<br>Olong_020581-R1 0.439 0.993 0.554 0.021 0.416	1<br>Opunc04g15200.1 0.123 0.37 0.247 0.687 0.984<br>Opunc11g08140.1 0.377 0.993 0.616 0.122 0.503	1<br>Obrac01g31030.2 0.07 0.381 0.311 0.454 0.952<br>Obrac11g05320.1 0.439 0.993 0.554 0.021 0.416	1<br>Lperr01g34610.3 0.123 0.39 0.267 0.645 0.992<br>Lperr11g07510.1 0.439 0.993 0.554 0.021 0.416	1<br>AT3G28450.1 0.439 0.998 0.559 0.023 0.443<br>AT5G16960.1 0.123 0.383 0.26 0.65 1
Obart01g40910.1	1<br>Oglab01g35730.1 0.005 1 0.995 0.005 1	1<br>Obart01g40910.1 0.005 1 0.995 0.005 1	0	0	0	0	0	0	0	0
	1<br>Oglab02g07520.1 0.475 1 0.525 0.255 1<br>Oglab07g00120.1 0.13 0.405 0.275 0.696 0.841	1<br>Obart02g07460.1 0.475 1 0.525 0.531 1<br>Obart08g06610.1 0.13 0.405 0.275 0.364 0.944	1<br>Osjap01g46570.1 0.41 1 0.59 0.156 1<br>Osjap10g12600.2 0.1 0.405 0.305 0.235 0.978	1<br>Osind01g47760.1 0.41 1 0.59 0.156 1<br>Osind08g01920.1 0.12 0.405 0.285 0.622 0.841	1<br>Orufi01g44230.1 0.41 1 0.59 0.156 1<br>Orufi08g02060.1 0.12 0.405 0.285 0.622 0.841	0<br>Olong_004144-R1 0.39 1 0.61 0.008 1	1<br>Opunc02g06610.1 0.475 1 0.525 0.245 1<br>Opunc08g01790.1 0.12 0.405 0.285 0.404 0.94	1<br>Obrac02g05500.1 0.475 1 0.525 0.234 1<br>Obrac03g27510.3 0.13 0.405 0.275 0.748 0.87	1<br>Lperr01g35480.1 0.475 1 0.525 0.228 1<br>Lperr03g31300.1 0.13 0.405 0.275 0.754 0.873	0<br>AT3G03740.1 0.13 0.405 0.275 0.662 0.781<br>AT3G27340.1 0.47 0.98 0.51 0.234 0.957
Obart01g43280.1	1<br>Oglab01g37970.1 0 0.999 0.999 0 0.999<br>Oglab01g37970.2 0 0.999 0.999 0 0.999<br>Oglab01g37970.3 0.028 0.999 0.971 0 0.999	1<br>Obart01g43280.1 0 1 1 0 1<br>Obart01g43280.2 0.028 1 0.972 0 1	1<br>Osjap01g49090.1 0.028 0.999 0.971 0.001 0.999	1<br>Osind01g50590.1 0.028 0.999 0.971 0.001 0.999	1<br>Orufi01g46760.2 0.026 0.999 0.973 0.001 0.999	1<br>Olong_026121-R2 0.026 0.886 0.86 0 1<br>Olong_026121-R3 0.028 0.886 0.858 0.001 1	1<br>Opunc01g42300.1 0.027 0.999 0.972 0.043 0.999	1<br>Obrac01g33850.1 0.027 0.896 0.869 0 0.979<br>Obrac01g33850.2 0.027 0.896 0.869 0 0.979<br>Obrac01g33850.3 0.027 0.896 0.869 0 0.999	0	0
	1<br>Oglab01g37960.1 0.438 1 0.562 0.009 0.999<br>Oglab01g37980.1 0.028 0.435 0.407 0.001 1	0<br>Obart01g43290.1 0.027 0.496 0.469 0.033 0.976	1<br>Osjap01g49070.1 0.447 1 0.553 0.002 1<br>Osjap01g49110.1 0.027 0.435 0.408 0.074 1	1<br>Osind01g50550.1 0.443 1 0.557 0.001 1<br>Osind02g04390.1 0.028 0.429 0.401 0.001 0.995	1<br>Orufi01g46740.1 0.446 1 0.554 0.013 1<br>Orufi11g06790.1 0.027 0.425 0.398 0.002 1	0<br>Olong_026121-R1 0.007 0.436 0.429 0.001 0.988	1<br>Opunc01g42270.1 0.433 1 0.567 0.005 1<br>Opunc11g06550.1 0.028 0.433 0.405 0 0.984	1<br>Obrac01g33820.1 0.446 1 0.554 0.008 1<br>Obrac02g02870.1 0.027 0.431 0.404 0.244 1	1<br>Lperr01g37690.1 0.445 1 0.555 0.022 1<br>Lperr11g05950.1 0.026 0.439 0.413 0 0.968	1<br>AT1G03220.1 0.446 0.999 0.553 0.067 0.993<br>AT3G14460.1 0.042 0.432 0.39 0.034 0.999
Obart02g00480.1	0	1<br>Obart02g00480.1 0.002 1 0.998 0.002 1	0	0	0	0	0	0	0	0
	1<br>Oglab01g12840.1 0.002 0.438 0.436 0.004 0.86<br>Oglab02g00410.1 0.458 1 0.542 0.069 1	1<br>Obart01g15210.1 0.002 0.438 0.436 0.004 0.86<br>Obart05g02080.1 0.458 0.91 0.452 0.062 0.875	1<br>Osjap01g17630.1 0.002 0.438 0.436 0.004 0.86<br>Osjap02g00500.2 0.471 0.91 0.439 0.068 0.877	1<br>Osind01g18080.1 0.002 0.438 0.436 0.004 0.86<br>Osind03g09080.1 0.45 0.91 0.46 0.144 0.919	1<br>Orufi01g16820.1 0.002 0.438 0.436 0.004 0.86<br>Orufi02g00500.5 0.471 0.91 0.439 0.075 0.757	1<br>Olong_016589-R3 0.458 0.91 0.452 0.126 0.885<br>Olong_022005-R1 0.002 0.438 0.436 0.004 0.95	1<br>Opunc02g00150.1 0.002 0.438 0.436 0.026 0.863<br>Opunc09g13920.1 0.444 0.908 0.464 0.008 0.876	1<br>Obrac02g00390.2 0.002 0.438 0.436 0.114 0.876<br>Obrac08g15590.5 0.498 0.91 0.412 0.138 0.797	1<br>Lperr01g13600.1 0.002 0.438 0.436 0.004 0.86<br>Lperr02g00490.1 0.458 0.912 0.454 0.091 0.863	0<br>AT5G07090.1 0.002 0.438 0.436 0.004 0.87
Obart02g01820.1	0	1<br>Obart02g01820.1 0.001 1 0.999 0.001 1	0	0	0	0	0	0	0	0
	1<br>Oglab02g01740.1 0.341 1 0.659 0.041 1<br>Oglab02g01750.1 0.001 0.313 0.312 0.003 0.995	0<br>Obart10g00600.1 0.046 0.316 0.27 0.025 0.993	1<br>Osjap02g01820.1 0.322 1 0.678 0.028 1<br>Osjap02g01830.1 0.001 0.313 0.312 0.003 0.995	1<br>Osind02g02050.1 0.322 1 0.678 0.028 1<br>Osind02g02060.1 0.001 0.313 0.312 0.003 0.995	1<br>Orufi02g01810.1 0.322 1 0.678 0.028 1<br>Orufi02g01820.1 0.001 0.313 0.312 0.003 0.995	1<br>Olong_003544-R1 0.001 0.313 0.312 0.003 0.995<br>Olong_003545-R1 0.362 0.983 0.621 0.001 0.999	1<br>Opunc02g01290.1 0.341 0.983 0.642 0.043 0.999<br>Opunc02g01300.1 0.04 0.313 0.273 0.003 0.994	1<br>Obrac02g01430.2 0.341 0.983 0.642 0.089 0.999<br>Obrac02g01440.4 0.029 0.313 0.284 0.054 0.99	1<br>Lperr02g01630.1 0.341 0.983 0.642 0.045 0.999<br>Lperr02g01640.2 0.042 0.313 0.271 0.006 0.994	0<br>AT1G59960.1 0.051 0.323 0.272 0.028 0.963<br>AT1G73970.1 0.463 0.958 0.495 0.229 0.956

Obart02g06500.1	0	1<br>Obart02g06500.1 0.001 1 0.999 0.001 1	0	0	0	0	0	0	0	0
	1<br>Oglab02g06600.1 0.307 1 0.693 0.033 1<br>Oglab02g06610.1 0.003 0.289 0.286 0.172 0.979	0<br>Obart06g04490.1 0.003 0.274 0.271 0.196 0.978	1<br>Osjap02g06980.1 0.307 1 0.693 0.033 1<br>Osjap02g06990.1 0.003 0.289 0.286 0.172 0.979	1<br>Osind02g07300.1 0.312 1 0.688 0.001 1<br>Osind02g07310.1 0.003 0.289 0.286 0.172 0.979	1<br>Orufi02g06640.1 0.307 1 0.693 0.033 1<br>Orufi02g06650.1 0.003 0.289 0.286 0.172 0.979	1<br>Olong_019072-R1 0.321 1 0.679 0.001 1<br>Olong_027167-R1 0.001 0.289 0.288 0.003 0.975	1<br>Opunc02g05730.1 0.307 0.998 0.691 0.034 1<br>Opunc02g05740.1 0.003 0.289 0.286 0.166 0.944	1<br>Obrac02g04810.1 0.313 1 0.687 0.034 1<br>Obrac02g04830.1 0.003 0.289 0.286 0.172 0.979	1<br>Lperr02g05650.1 0.307 0.999 0.692 0.041 0.971<br>Lperr02g05670.1 0.003 0.289 0.286 0.175 0.979	1<br>AT3G24550.1 0.003 0.277 0.274 0.466 0.903<br>AT4G32190.1 0.302 0.997 0.695 0.024 0.987
Obart02g11640.1	0	1<br>Obart02g11640.1 0.001 1 0.999 0.001 1	0	0	0	0	0	0	0	0
	1<br>Oglab02g10250.1 0.001 0.476 0.475 0.002 0.996<br>Oglab02g11670.1 0.483 1 0.517 0.002 1	1<br>Obart02g10250.1 0.001 0.476 0.475 0.002 0.996<br>Obart03g04220.1 0.479 1 0.521 0.048 0.998	1<br>Osjap02g11160.1 0.001 0.476 0.475 0.002 0.996<br>Osjap03g04250.1 0.479 0.999 0.52 0.067 0.996	1<br>Osind02g12980.1 0.497 1 0.503 0.03 1<br>Osind02g13000.1 0.001 0.476 0.475 0.002 1	1<br>Orufi02g10270.1 0.001 0.476 0.475 0.002 0.996<br>Orufi02g11670.1 0.487 1 0.513 0.012 1	1<br>Olong_009803-R1 0.479 1 0.521 0.002 0.998<br>Olong_026120-R1 0.001 0.476 0.475 0.002 0.996	1<br>Opunc02g10280.1 0.479 1 0.521 0.002 1<br>Opunc02g10300.1 0.001 0.475 0.474 0.002 0.998	1<br>Obrac02g07710.1 0.001 0.476 0.475 0.002 1<br>Obrac03g03340.1 0.52 1 0.48 0.136 0.998	0<br>Lperr10g08090.1 0.386 1 0.614 0.063 0.998	1<br>AT1G03890.1 0.001 0.457 0.456 0.002 0.991<br>AT2G37640.1 0.486 0.996 0.51 0.011 0.985
Obart02g11910.1	0	1<br>Obart02g11910.1 0.001 1 0.999 0.001 1	0	0	0	0	0	0	0	0
	1<br>Oglab02g11870.3 0.001 0.616 0.615 0.018 0.991<br>Oglab02g11880.1 0.649 0.996 0.347 0.001 0.282	1<br>Obart11g09030.1 0.004 0.616 0.612 0.054 0.994<br>Obart11g20370.3 0.649 0.995 0.346 0.001 0.275	1<br>Osjap02g13100.1 0.649 0.996 0.347 0.001 0.242<br>Osjap02g13110.1 0.001 0.616 0.615 0.002 0.998	1<br>Osind02g13300.1 0.001 0.616 0.615 0.2 0.993<br>Osind12g16510.1 0.641 0.996 0.355 0.002 0.632	1<br>Orufi02g11960.1 0.001 0.616 0.615 0.172 0.992<br>Orufi08g05130.1 0.624 0.974 0.35 0 0.595	1<br>Olong_006337-R1 0.004 0.616 0.612 0.108 0.998<br>Olong_026902-R1 0.647 0.996 0.349 0.073 0.821	1<br>Opunc02g10610.1 0.649 0.987 0.338 0.004 0.983<br>Opunc02g10620.1 0.001 0.616 0.615 0.042 0.998	0<br>Obrac12g13260.3 0.001 0.619 0.618 0.002 0.998	1<br>Lperr11g07730.1 0.649 0.996 0.347 0.001 0.325<br>Lperr11g07990.1 0.001 0.616 0.615 0.002 0.988	1<br>AT1G50180.1 0.649 0.978 0.329 0.001 0.285<br>AT4G26510.1 0.001 0.596 0.595 0.002 0.998
Obart02g23460.2	1<br>Oglab02g22130.2 0.001 1 0.999 0.001 1	1<br>Obart02g23460.2 0.001 1 0.999 0.001 1	1<br>Osjap02g26750.1 0.001 0.969 0.968 0.001 0.994<br>Osjap02g26750.2 0.001 0.969 0.968 0.001 0.994<br>Osjap02g26750.4 0.001 0.969 0.968 0.001 0.799	1<br>Osind02g26640.2 0.001 1 0.999 0.001 1	1<br>Orufi02g24900.1 0.011 0.974 0.963 0.06 0.923<br>Orufi02g24900.3 0.001 0.974 0.973 0.001 0.857<br>Orufi02g24900.4 0.001 0.974 0.973 0.001 0.856	0	1<br>Opunc02g21410.1 0.011 0.999 0.988 0.116 0.996<br>Opunc02g21410.2 0.011 0.999 0.988 0.114 0.996<br>Opunc02g21410.3 0.011 0.999 0.988 0.115 0.996<br>Opunc02g21410.4 0.011 0.999 0.988 0.114 0.996	0	1<br>Lperr02g18970.1 0.011 0.997 0.986 0.032 0.996	0
	1<br>Oglab02g21950.1 0.475 0.999 0.524 0.073 0.995<br>Oglab02g22110.2 0.011 0.459 0.448 0.532 0.98	1<br>Obart02g23290.1 0.475 0.999 0.524 0.029 0.999<br>Obart02g23440.1 0.011 0.459 0.448 0.492 0.981	1<br>Osjap02g26590.1 0.475 0.999 0.524 0.033 0.999<br>Osjap02g26750.5 0.011 0.459 0.448 0.238 0.973	1<br>Osind02g26450.1 0.475 0.999 0.524 0.025 0.997<br>Osind02g26620.1 0.011 0.459 0.448 0.495 0.981	1<br>Orufi02g24730.1 0.475 0.994 0.519 0.031 0.996<br>Orufi02g24880.2 0.011 0.459 0.448 0.18 0.97	0<br>Olong_024345-R1 0.011 0.474 0.463 0.442 0.991	1<br>Opunc02g21230.1 0.479 0.976 0.497 0.003 0.934<br>Opunc02g21240.1 0.011 0.461 0.45 0.121 0.972	1<br>Obrac02g16670.1 0.549 0.973 0.424 0.076 0.995<br>Obrac02g16700.1 0.011 0.461 0.45 0.114 0.947	1<br>Lperr02g18740.1 0.485 0.997 0.512 0.056 0.991<br>Lperr02g19120.1 0.011 0.484 0.473 0.183 0.978	1<br>AT4G31860.1 0.014 0.45 0.436 0.123 0.989<br>AT5G43080.1 0.626 1 0.374 0.194 0.932
Obart02g24930.1	0	1<br>Obart02g24930.1 0.007 1 0.993 0.007 1	0	0	0	0	0	0	0	0
	1<br>Oglab02g23510.1 0.007 0.345 0.338 0.546 0.803<br>Oglab04g03020.1 0.432 1 0.568 0.013 1	0<br>Obart05g18450.1 0.396 1 0.604 0.315 1	1<br>Osjap02g28260.2 0.007 0.345 0.338 0.012 0.571<br>Osjap04g22620.1 0.432 1 0.568 0.012 1	1<br>Osind02g28120.1 0.007 0.338 0.331 0.619 0.906<br>Osind04g04600.1 0.338 1 0.662 0.189 1	1<br>Orufi02g26240.6 0.007 0.345 0.338 0.012 0.571<br>Orufi04g20540.1 0.432 1 0.568 0.012 1	1<br>Olong_000623-R2 0.007 0.345 0.338 0.589 0.822<br>Olong_023182-R1 0.432 1 0.568 0.012 1	0<br>Opunc04g03300.1 0.432 1 0.568 0.013 1	0<br>Obrac02g17870.2 0.432 1 0.568 0.013 1	0<br>Lperr01g37400.3 0.252 1 0.748 0.758 0.84	0<br>AT2G31953.1 0.432 1 0.568 0.012 0.919
Obart02g31950.1	0	1<br>Obart02g31950.1 0.001 1 0.999 0.001 1<br>Obart02g31950.2 0.001 1 0.999 0.001 1	0	0	0	0	0	0	1<br>Lperr02g26150.1 0.001 1 0.999 0.001 1<br>Lperr02g26150.2 0.001 1 0.999 0.001 1<br>Lperr02g26150.3 0.001 1 0.999 0.002 1	0
	1<br>Oglab02g29520.1 0.426 1 0.574 0.039 1<br>Oglab02g29530.1 0.001 0.374 0.373 0.003 1	0<br>Obart06g27910.1 0.001 0.371 0.37 0.036 0.987	1<br>Osjap02g36120.2 0.426 0.933 0.507 0.039 0.865<br>Osjap02g36130.1 0.001 0.374 0.373 0.003 1	1<br>Osind02g35590.1 0.426 1 0.574 0.039 1<br>Osind02g35610.1 0.001 0.374 0.373 0.003 1	1<br>Orufi02g33640.1 0.426 0.933 0.507 0.039 0.865<br>Orufi02g33650.1 0.001 0.374 0.373 0.003 1	1<br>Olong_014377-R1 0.426 1 0.574 0.04 1<br>Olong_014378-R1 0.001 0.374 0.373 0.003 1	1<br>Opunc02g17770.1 0.001 0.374 0.373 0.003 1<br>Opunc02g17780.1 0.427 1 0.573 0.109 1	1<br>Obrac02g23310.1 0.436 1 0.564 0.063 1<br>Obrac02g23320.1 0.001 0.374 0.373 0.003 1	0<br>Lperr06g23320.1 0.002 0.402 0.4 0.006 0.997	1<br>AT4G04180.1 0.478 0.993 0.515 0.123 0.977<br>AT5G08460.1 0.001 0.371 0.37 0.031 0.992

Obart02g31950.2	0	1<br>Obart02g31950.1 0.001 1 0.999 0.001 1<br>Obart02g31950.2 0.001 1 0.999 0.001 1	0	0	0	0	0	0	1<br>Lperr02g26150.1 0.001 1 0.999 0.001 1<br>Lperr02g26150.2 0.001 1 0.999 0.001 1<br>Lperr02g26150.3 0.001 1 0.999 0.002 1	0
	1<br>Oglab02g29520.1 0.428 1 0.572 0.039 1<br>Oglab02g29530.1 0.001 0.375 0.374 0.003 1	0<br>Obart06g27910.1 0.001 0.372 0.371 0.036 0.987	1<br>Osjap02g36120.2 0.428 0.932 0.504 0.039 0.865<br>Osjap02g36130.1 0.001 0.375 0.374 0.003 1	1<br>Osind02g35590.1 0.428 1 0.572 0.039 1<br>Osind02g35610.1 0.001 0.375 0.374 0.003 1	1<br>Orufi02g33640.1 0.428 0.932 0.504 0.039 0.865<br>Orufi02g33650.1 0.001 0.375 0.374 0.003 1	1<br>Olong_014377-R1 0.428 1 0.572 0.04 1<br>Olong_014378-R1 0.001 0.375 0.374 0.003 1	1<br>Opunc02g17770.1 0.001 0.375 0.374 0.003 1<br>Opunc02g17780.1 0.429 1 0.571 0.109 1	1<br>Obrac02g23310.1 0.438 1 0.562 0.063 1<br>Obrac02g23320.1 0.001 0.375 0.374 0.003 1	0<br>Lperr06g23320.1 0.002 0.404 0.402 0.006 0.997	1<br>AT4G04180.1 0.48 0.993 0.513 0.123 0.977<br>AT5G08460.1 0.001 0.372 0.371 0.031 0.992
Obart02g38050.2	1<br>Oglab02g35180.1 0.001 0.976 0.975 0.001 0.919<br>Oglab02g35180.2 0.001 1 0.999 0.001 1	1<br>Obart02g38050.1 0.001 0.976 0.975 0.001 0.919<br>Obart02g38050.2 0.001 1 0.999 0.001 1	0	1<br>Osind02g41950.1 0.071 0.95 0.879 0.368 0.955	1<br>Orufi02g39760.1 0.001 1 0.999 0.001 0.95<br>Orufi02g39760.2 0.001 1 0.999 0.001 1	0	0	0	0	0
	1<br>Oglab02g35180.3 0.001 0.231 0.23 0.005 0.885<br>Oglab07g10660.4 0.263 0.996 0.733 0.085 0.801	0<br>Obart03g04840.1 0.32 0.974 0.654 0.007 0.995	1<br>Osjap05g10980.2 0.237 0.997 0.76 0.066 0.962<br>Osjap05g11860.1 0.001 0.209 0.208 0.078 0.911	1<br>Osind05g11850.1 0.237 0.997 0.76 0.066 0.974<br>Osind06g11870.1 0.001 0.209 0.208 0.078 0.911	1<br>Orufi02g39760.3 0.001 0.231 0.23 0.005 0.886<br>Orufi05g10490.3 0.237 0.949 0.712 0.064 0.962	1<br>Olong_016606-R1 0.31 0.996 0.686 0.008 0.889<br>Olong_027192-R1 0.001 0.209 0.208 0.078 0.911	0<br>Opunc02g35330.1 0.208 1 0.792 0.014 1	1<br>Obrac05g06630.3 0.242 0.971 0.729 0.081 0.95<br>Obrac06g07800.1 0.001 0.209 0.208 0.078 0.911	0<br>Lperr02g31540.1 0.208 1 0.792 0.017 1	0<br>AT5G38730.1 0.202 0.99 0.788 0.017 0.998
Obart03g00550.1	1<br>Oglab03g00390.1 0.001 0.996 0.995 0.001 0.994	1<br>Obart03g00550.1 0.001 1 0.999 0.001 1	1<br>Osjap03g00680.1 0.001 0.996 0.995 0.001 0.994<br>Osjap03g00680.2 0.001 0.996 0.995 0.001 0.994<br>Osjap03g00680.3 0.001 0.996 0.995 0.001 0.994	1<br>Osind03g00740.1 0.001 1 0.999 0.001 1	1<br>Orufi03g12660.1 0.001 0.996 0.995 0.001 0.994<br>Orufi03g12660.2 0.001 0.996 0.995 0.001 0.994	0	0	0	0	0
	1<br>Oglab02g34920.1 0.061 0.675 0.614 0.131 1<br>Oglab04g12740.1 0.715 0.996 0.281 0.411 0.989	1<br>Obart02g37790.1 0.061 0.645 0.584 0.133 0.977<br>Obart12g06510.1 0.673 0.996 0.323 0.052 0.984	1<br>Osjap02g42090.1 0.052 0.675 0.623 0.065 1<br>Osjap12g08580.1 0.706 0.996 0.29 0.011 0.977	1<br>Osind02g41670.1 0.061 0.675 0.614 0.131 1<br>Osind03g02220.1 0.695 0.996 0.301 0.015 0.982	1<br>Orufi02g39350.1 0.061 0.675 0.614 0.131 1<br>Orufi09g02220.2 0.742 0.996 0.254 0.095 0.975	0<br>Olong_013175-R1 0.27 0.904 0.634 0.002 0.961	1<br>Opunc03g00470.1 0.001 0.78 0.779 0.002 1<br>Opunc03g03130.2 0.78 0.996 0.216 0.443 0.984	0<br>Obrac02g27970.1 0.136 0.675 0.539 0.105 1<br>Obrac05g17660.1 0.747 0.995 0.248 0.427 0.997	1<br>Lperr03g00570.1 0.001 0.675 0.674 0.002 1<br>Lperr04g11360.1 0.748 0.996 0.248 0.415 0.99	0<br>AT5G04460.1 0.018 0.675 0.657 0.081 1
Obart03g01190.1	0	1<br>Obart03g01190.1 0.002 1 0.998 0.002 1	0	0	0	0	0	0	0	0
	1<br>Oglab03g01040.1 0.694 0.993 0.299 0.005 0.796<br>Oglab03g01050.1 0.002 0.611 0.609 0.002 0.983	1<br>Obart01g33610.1 0.706 0.993 0.287 0.002 0.17<br>Obart03g14810.2 0.061 0.593 0.532 0.198 0.987	1<br>Osjap03g01290.1 0.655 0.993 0.338 0.001 0.15<br>Osjap03g01300.2 0.002 0.633 0.631 0.002 0.996	1<br>Osind03g01350.1 0.694 0.993 0.299 0.005 0.796<br>Osind07g20900.1 0.035 0.606 0.571 0.217 0.955	1<br>Orufi03g00970.1 0.655 0.993 0.338 0.004 0.815<br>Orufi03g00980.1 0.057 0.611 0.554 0.002 0.982	0<br>Olong_020901-R3 0.066 0.593 0.527 0.009 0.744	1<br>Opunc03g01090.1 0.655 0.993 0.338 0.004 0.814<br>Opunc03g01100.1 0.013 0.611 0.598 0.01 0.983	1<br>Obrac02g06700.4 0.011 0.593 0.582 0.435 0.868<br>Obrac03g00890.1 0.694 0.993 0.299 0.001 0.149	1<br>Lperr03g01130.1 0.694 0.993 0.299 0.001 0.131<br>Lperr03g01140.1 0.009 0.617 0.608 0.005 0.858	1<br>AT3G13230.1 0.712 0.993 0.281 0.102 0.814<br>AT3G53170.1 0.004 0.593 0.589 0.064 0.772
Obart03g02800.1	0	1<br>Obart03g02800.1 0.001 1 0.999 0.001 1	0	0	0	0	0	0	0	0
	1<br>Oglab03g02720.1 0.589 1 0.411 0.002 1<br>Oglab03g02730.1 0.01 0.583 0.573 0.013 0.989	1<br>Obart03g02810.1 0.589 0.978 0.389 0.002 0.993<br>Obart03g02820.1 0.01 0.583 0.573 0.013 0.989	1<br>Osjap03g02800.1 0.589 0.978 0.389 0.005 0.968<br>Osjap03g02820.1 0.01 0.583 0.573 0.013 0.989	1<br>Osind03g02900.1 0.01 0.583 0.573 0.013 0.989<br>Osind10g16860.1 0.589 0.978 0.389 0.004 0.992	1<br>Orufi01g17470.2 0.591 0.996 0.405 0.002 0.996<br>Orufi03g02520.1 0.017 0.583 0.566 0.025 0.989	1<br>Olong_005980-R1 0.589 0.978 0.389 0.002 0.989<br>Olong_005981-R1 0.017 0.583 0.566 0.03 0.987	1<br>Opunc03g02530.1 0.589 0.979 0.39 0.002 0.912<br>Opunc03g02550.1 0.017 0.583 0.566 0.02 0.989	1<br>Obrac03g02200.1 0.017 0.588 0.571 0.034 0.997<br>Obrac10g11140.1 0.589 0.979 0.39 0.003 0.993	1<br>Lperr03g02550.1 0.01 0.583 0.573 0.012 0.989<br>Lperr10g13340.1 0.589 0.987 0.398 0.004 0.996	1<br>AT2G17020.1 0.022 0.58 0.558 0.04 0.97<br>AT3G62760.1 0.592 0.983 0.391 0.014 1
Obart03g07090.1	0	1<br>Obart03g07090.1 0.001 1 0.999 0.001 1<br>Obart03g07090.2 0.001 1 0.999 0.001 1	0	0	0	0	0	0	0	0

	1<br>Oglab03g07150.1 0.045 0.61 0.565 0.002 0.995<br>Oglab03g07160.4 0.61 1 0.39 0.003 1	0<br>Obart03g07090.4 0.001 0.61 0.609 0.002 0.978	1<br>Osjap01g23040.3 0.61 1 0.39 0.003 1<br>Osjap03g07460.1 0.045 0.61 0.565 0.002 0.995	1<br>Osind03g07550.1 0.001 0.61 0.609 0.002 0.995<br>Osind03g07560.1 0.61 1 0.39 0.003 1	1<br>Orufi01g22080.2 0.61 1 0.39 0.002 1<br>Orufi03g07040.1 0.045 0.61 0.565 0.002 0.995	1<br>Olong_017037-R1 0.61 1 0.39 0.003 1<br>Olong_026139-R1 0.093 0.61 0.517 0.002 0.995	1<br>Opunc01g19950.1 0.61 1 0.39 0.003 1<br>Opunc03g06910.1 0.033 0.61 0.577 0.054 0.995	0<br>Obrac03g05750.1 0.055 0.634 0.579 0.023 0.991	1<br>Lperr03g06490.1 0.045 0.61 0.565 0.002 0.995<br>Lperr03g06500.1 0.61 1 0.39 0.08 1	1<br>AT1G75100.1 0.055 0.61 0.555 0.046 0.995<br>AT5G27470.1 0.61 0.988 0.378 0.002 0.967
Obart03g07090.2	0	1<br>Obart03g07090.1 0.001 1 0.999 0.001 1<br>Obart03g07090.2 0.001 1 0.999 0.001 1	0	0	0	0	0	0	0	0
	1<br>Oglab03g07150.1 0.045 0.61 0.565 0.002 0.995<br>Oglab03g07160.4 0.61 1 0.39 0.003 1	0<br>Obart03g07090.4 0.001 0.61 0.609 0.002 0.978	1<br>Osjap01g23040.3 0.61 1 0.39 0.003 1<br>Osjap03g07460.1 0.045 0.61 0.565 0.002 0.995	1<br>Osind03g07550.1 0.001 0.61 0.609 0.002 0.995<br>Osind03g07560.1 0.61 1 0.39 0.003 1	1<br>Orufi01g22080.2 0.61 1 0.39 0.002 1<br>Orufi03g07040.1 0.045 0.61 0.565 0.002 0.995	1<br>Olong_017037-R1 0.61 1 0.39 0.003 1<br>Olong_026139-R1 0.093 0.61 0.517 0.002 0.995	1<br>Opunc01g19950.1 0.61 1 0.39 0.003 1<br>Opunc03g06910.1 0.033 0.61 0.577 0.054 0.995	0<br>Obrac03g05750.1 0.055 0.634 0.579 0.023 0.991	1<br>Lperr03g06490.1 0.045 0.61 0.565 0.002 0.995<br>Lperr03g06500.1 0.61 1 0.39 0.08 1	1<br>AT1G75100.1 0.055 0.61 0.555 0.046 0.995<br>AT5G27470.1 0.61 0.988 0.378 0.002 0.967
Obart03g07660.1	0	1<br>Obart03g07660.1 0.001 1 0.999 0.001 1	0	0	0	0	0	0	0	0
	1<br>Oglab03g07740.1 0.298 1 0.702 0.015 1<br>Oglab03g07760.1 0.001 0.275 0.274 0.003 0.994	1<br>Obart08g08450.1 0.001 0.267 0.266 0.154 0.956<br>Obart09g05350.1 0.373 0.998 0.625 0.123 0.988	1<br>Osjap03g08130.1 0.298 1 0.702 0.015 1<br>Osjap10g07700.1 0.001 0.267 0.266 0.203 0.959	1<br>Osind03g08180.1 0.329 1 0.671 0.001 1<br>Osind10g05560.1 0.001 0.267 0.266 0.003 0.945	1<br>Orufi03g07710.1 0.329 1 0.671 0.001 1<br>Orufi03g07730.2 0.001 0.275 0.274 0.003 0.994	1<br>Olong_012932-R1 0.001 0.275 0.274 0.003 0.994<br>Olong_012934-R1 0.329 1 0.671 0.001 1	1<br>Opunc01g43280.1 0.379 0.998 0.619 0.226 0.944<br>Opunc03g07460.1 0.001 0.275 0.274 0.003 0.994	1<br>Obrac03g06230.1 0.329 1 0.671 0.001 1<br>Obrac10g03970.1 0.001 0.277 0.276 0.003 0.94	1<br>Lperr03g06980.1 0.329 1 0.671 0.001 1<br>Lperr10g04560.2 0.001 0.275 0.274 0.003 0.976	0<br>AT4G27190.1 0.347 0.997 0.65 0.122 0.982
Obart03g09160.3	1<br>Oglab03g09270.1 0 1 1 0.001 1<br>Oglab03g09270.2 0 1 1 0.001 1	1<br>Obart03g09160.1 0 1 1 0 1<br>Obart03g09160.2 0 1 1 0 1<br>Obart03g09160.3 0 1 1 0 1	1<br>Osjap03g09700.1 0 1 1 0.046 1<br>Osjap03g09700.2 0 1 1 0.045 1	1<br>Osind03g09660.1 0 1 1 0.044 1	1<br>Orufi03g09320.1 0 1 1 0.045 1<br>Orufi03g09320.2 0 1 1 0.046 1<br>Orufi03g09320.3 0 1 1 0.047 1<br>Orufi03g09320.4 0 1 1 0.048 1	1<br>Olong_002353-R1 0.021 1 0.979 0.001 1	1<br>Opunc03g08800.1 0.028 1 0.972 0.026 1<br>Opunc03g08800.2 0.028 1 0.972 0.026 1<br>Opunc03g08800.3 0.028 1 0.972 0.027 1	0	0	0
	1<br>Oglab10g00260.1 0.578 0.988 0.41 0.179 0.96<br>Oglab10g00270.1 0.069 0.469 0.4 0.072 1	1<br>Obart10g00240.1 0.578 0.988 0.41 0.179 0.96<br>Obart10g00250.1 0.069 0.469 0.4 0.072 1	1<br>Osjap05g08590.1 0.095 0.459 0.364 0.436 0.995<br>Osjap06g17700.1 0.55 0.988 0.438 0.321 0.983	1<br>Osind01g21860.1 0.069 0.469 0.4 0.075 1<br>Osind06g17200.1 0.55 0.988 0.438 0.318 0.983	1<br>Orufi10g00220.1 0.578 0.988 0.41 0.171 0.96<br>Orufi10g00230.1 0.069 0.469 0.4 0.074 1	1<br>Olong_008994-R1 0.069 0.469 0.4 0.07 0.999<br>Olong_023066-R1 0.581 0.995 0.414 0.021 0.97	0<br>Opunc03g08800.7 0.349 1 0.651 0.004 1	1<br>Obrac03g07350.1 0.548 1 0.452 0.114 1<br>Obrac10g00260.1 0.092 0.469 0.377 0.03 0.999	1<br>Lperr01g01500.1 0.082 0.471 0.389 0.001 1<br>Lperr03g08210.1 0.546 1 0.454 0.133 1	1<br>AT1G69770.1 0.089 0.459 0.37 0.027 0.988<br>AT4G34440.1 0.525 0.992 0.467 0.122 0.975
Obart03g10140.1	0	1<br>Obart03g10140.1 0.001 1 0.999 0.001 1	0	0	0	0	0	0	0	0
	1<br>Oglab03g10120.1 0.001 0.621 0.62 0.204 0.905<br>Oglab03g10130.1 0.621 1 0.379 0.15 1	0<br>Obart03g10140.2 0.001 0.621 0.62 0.002 0.881	1<br>Osjap03g10880.1 0.001 0.621 0.62 0.204 0.905<br>Osjap10g01900.1 0.621 1 0.379 0.143 1	1<br>Osind03g10780.1 0.001 0.448 0.447 0.193 0.979<br>Osind03g10790.1 0.621 1 0.379 0.145 1	1<br>Orufi03g10350.1 0.001 0.621 0.62 0.204 0.905<br>Orufi10g01680.1 0.621 1 0.379 0.143 1	1<br>Olong_000838-R1 0.02 0.621 0.601 0.002 0.868<br>Olong_000839-R1 0.621 1 0.379 0.071 1	1<br>Opunc03g09740.1 0.001 0.623 0.622 0.132 0.9<br>Opunc03g09750.2 0.636 1 0.364 0.076 1	1<br>Obrac03g08150.1 0.001 0.621 0.62 0.133 0.899<br>Obrac03g08160.1 0.621 1 0.379 0.142 1	1<br>Lperr03g09060.1 0.001 0.621 0.62 0.133 0.896<br>Lperr03g09070.3 0.621 1 0.379 0.141 1	1<br>AT5G64813.1 0.621 1 0.379 0.129 1<br>AT5G66130.1 0.005 0.615 0.61 0.115 0.883
Obart03g10480.1	0	1<br>Obart03g10480.1 0.004 1 0.996 0.004 1	1<br>Osjap03g11290.1 0.004 1 0.996 0.004 1	0	0	0	0	0	0	0
	1<br>Oglab01g01190.1 0.534 0.996 0.462 0.656 0.959<br>Oglab03g10450.1 0.004 0.509 0.505 0.007 1	1<br>Obart01g05330.1 0.495 0.996 0.501 0.64 0.945<br>Obart03g10500.1 0.004 0.305 0.301 0.011 0.989	0<br>Osjap01g06090.1 0.495 0.996 0.501 0.64 0.945<br>Osjap03g11310.1 0.004 0.283 0.279 0.005 0.398	1<br>Osind03g11120.1 0.004 0.509 0.505 0.007 1<br>Osind03g11140.1 0.527 1 0.473 0.668 0.954	1<br>Orufi03g10700.1 0.004 0.509 0.505 0.007 1<br>Orufi03g10740.1 0.527 1 0.473 0.664 0.947	1<br>Olong_001134-R1 0.53 0.828 0.298 0.477 0.77<br>Olong_001982-R1 0.004 0.509 0.505 0.007 1	1<br>Opunc03g10110.1 0.004 0.509 0.505 0.007 1<br>Opunc03g10130.1 0.527 1 0.473 0.686 0.985	0<br>Obrac03g08430.5 0.527 1 0.473 0.284 0.412	0<br>Lperr03g09350.4 0.004 0.509 0.505 0.005 1	0
Obart03g10890.2	1	1	1	0	0	0	0	0	0	0

	Oglab03g10840.1 0.002 1 0.998 0.277 1<br>Oglab03g10840.4 0.002 1 0.998 0.26 1	Obart03g10890.2 0.002 1 0.998 0.002 1	Osjap03g11750.1 0.002 1 0.998 0.275 1<br>Osjap03g11750.2 0.002 0.991 0.989 0.257 0.987							
	1<br>Oglab03g10840.2 0.002 0.601 0.599 0.271 0.735<br>Oglab09g00870.1 0.612 0.867 0.255 0.072 0.594	0<br>Obart03g10890.1 0.002 0.601 0.599 0.002 0.639	1<br>Osjap03g11750.3 0.002 0.601 0.599 0.35 0.953<br>Osjap05g12220.1 0.612 0.916 0.304 0.057 0.857	1<br>Osind03g11510.1 0.608 1 0.392 0.043 1<br>Osind03g11520.1 0.002 0.601 0.599 0.367 0.998	1<br>Orufi03g11130.1 0.608 1 0.392 0.043 1<br>Orufi03g11140.1 0.002 0.601 0.599 0.367 0.998	0<br>Olong_015766-R1 0.02 0.78 0.76 0.003 0.991	1<br>Opunc03g10480.1 0.61 0.953 0.343 0.045 0.829<br>Opunc03g10490.1 0.002 0.601 0.599 0.362 0.998	1<br>Obrac03g08730.1 0.608 0.927 0.319 0.052 0.981<br>Obrac03g08740.1 0.02 0.601 0.581 0.004 0.996	1<br>Lperr01g40130.1 0.612 0.929 0.317 0.063 0.769<br>Lperr03g09630.1 0.002 0.601 0.599 0.369 0.998	1<br>AT1G33520.1 0.002 0.601 0.599 0.284 0.998<br>AT4G36590.1 0.612 0.887 0.275 0.032 0.484
Obart03g12120.1	1<br>Oglab03g12120.1 0.001 1 0.999 0.001 1	1<br>Obart03g12120.1 0.001 1 0.999 0.001 1	1<br>Osjap03g13100.1 0.028 1 0.972 0.001 1	1<br>Osind03g12930.1 0.001 1 0.999 0.001 1	0	0	0	0	0	0
	1<br>Oglab06g01620.1 0.642 0.998 0.356 0.212 0.912<br>Oglab12g00800.1 0.114 0.633 0.519 0.483 0.983	1<br>Obart04g15560.1 0.64 0.998 0.358 0.146 0.877<br>Obart11g01060.1 0.114 0.633 0.519 0.471 0.982	1<br>Osjap04g16240.1 0.642 0.998 0.356 0.067 0.921<br>Osjap12g01140.1 0.109 0.633 0.524 0.498 0.981	1<br>Osind04g14790.1 0.642 0.998 0.356 0.013 0.911<br>Osind12g01290.1 0.109 0.633 0.524 0.49 0.981	1<br>Orufi06g01460.3 0.642 0.998 0.356 0.212 0.912<br>Orufi12g01130.1 0.109 0.633 0.524 0.498 0.981	1<br>Olong_007714-R9 0.642 1 0.358 0.002 1<br>Olong_007715-R1 0.125 0.633 0.508 0.007 0.965	1<br>Opunc03g11680.1 0.028 0.633 0.605 0.001 0.971<br>Opunc04g11270.1 0.642 0.998 0.356 0.067 0.921	1<br>Obrac03g09720.1 0.025 0.633 0.608 0.036 0.971<br>Obrac04g09430.1 0.64 0.998 0.358 0.014 0.863	1<br>Lperr03g10720.1 0.028 0.633 0.605 0.001 0.934<br>Lperr03g17470.1 0.633 0.998 0.365 0.119 0.828	1<br>AT3G46740.1 0.122 0.633 0.511 0.158 0.971<br>AT3G53360.1 0.672 0.998 0.326 0.09 0.934
Obart03g20630.1	1<br>Oglab03g20370.1 0.073 1 0.927 0.087 1	1<br>Obart03g20630.1 0.001 1 0.999 0.001 1	1<br>Osjap03g22210.1 0.009 0.991 0.982 0.567 0.972	1<br>Osind03g22510.1 0.017 1 0.983 0.552 1	1<br>Orufi03g21250.1 0.185 0.995 0.81 0.02 0.991<br>Orufi03g21250.2 0.185 0.995 0.81 0.019 0.991<br>Orufi03g21250.3 0.189 0.995 0.806 0.021 0.99<br>Orufi03g21250.4 0.185 0.995 0.81 0.021 0.99	0	0	0	0	0
	1<br>Oglab05g22780.1 0.044 0.547 0.503 0.077 0.982<br>Oglab08g00090.1 0.557 0.931 0.374 0.117 0.976	1<br>Obart06g03480.2 0.051 0.556 0.505 0.002 0.826<br>Obart09g16880.1 0.557 0.931 0.374 0.091 0.964	1<br>Osjap05g27870.1 0.044 0.547 0.503 0.075 0.982<br>Osjap12g12160.2 0.556 0.931 0.375 0.083 0.997	1<br>Osind02g20400.1 0.607 0.931 0.324 0.137 0.985<br>Osind05g28150.1 0.044 0.547 0.503 0.075 0.982	1<br>Orufi05g26490.1 0.044 0.553 0.509 0.075 0.989<br>Orufi09g01530.2 0.557 0.931 0.374 0.087 0.965	1<br>Olong_013421-R1 0.051 0.556 0.505 0.002 0.877<br>Olong_028288-R1 0.607 1 0.393 0.003 1	1<br>Opunc05g00870.2 0.567 0.996 0.429 0.145 0.973<br>Opunc05g22250.1 0.044 0.547 0.503 0.075 0.982	0<br>Obrac09g04370.1 0.08 0.572 0.492 0.034 0.858	0<br>Lperr05g06040.4 0.557 0.992 0.435 0.084 0.556	0<br>AT1G07740.1 0.025 0.531 0.506 0.083 0.967
Obart03g27980.1	0	1<br>Obart03g27980.1 0.001 1 0.999 0.001 1<br>Obart03g27980.2 0.015 1 0.985 0.015 1	1<br>Osjap03g30460.1 0.021 1 0.979 0.085 1	1<br>Osind03g31500.1 0.021 1 0.979 0.094 1	1<br>Orufi03g29110.1 0.021 1 0.979 0.341 1	0	0	0	0	0
	1<br>Oglab03g27330.1 0.51 1 0.49 0.043 1<br>Oglab03g27340.1 0.008 0.476 0.468 0.008 0.997	1<br>Obart01g35640.1 0.509 0.998 0.489 0.063 0.982<br>Obart12g19300.1 0.019 0.475 0.456 0.01 0.99	1<br>Osjap03g31370.3 0.509 0.998 0.489 0.042 0.887<br>Osjap12g24400.2 0.019 0.475 0.456 0.012 0.988	1<br>Osind04g00720.3 0.509 0.999 0.49 0.064 0.919<br>Osind12g20440.1 0.019 0.475 0.456 0.01 0.99	1<br>Orufi03g30600.1 0.509 0.999 0.49 0.05 0.885<br>Orufi12g21550.1 0.019 0.475 0.456 0.01 0.99	1<br>Olong_006906-R1 0.04 0.468 0.428 0.071 1<br>Olong_007449-R1 0.509 0.999 0.49 0.065 0.909	1<br>Opunc03g25490.1 0.021 0.486 0.465 0.525 0.8<br>Opunc03g26940.1 0.509 0.999 0.49 0.052 0.885	1<br>Obrac03g20710.1 0.021 0.476 0.455 0.028 0.997<br>Obrac03g21410.1 0.574 0.995 0.421 0.09 0.91	1<br>Lperr03g23190.1 0.51 0.999 0.489 0.039 0.863<br>Lperr03g23200.1 0.009 0.476 0.467 0.008 0.997	0<br>AT5G36940.1 0.019 0.478 0.459 0.003 0.998
Obart03g27980.2	0	1<br>Obart03g27980.1 0.015 1 0.985 0.015 1<br>Obart03g27980.2 0.001 1 0.999 0.001 1	1<br>Osjap03g30460.1 0.021 1 0.979 0.085 1	1<br>Osind03g31500.1 0.021 1 0.979 0.094 1	1<br>Orufi03g29110.1 0.021 1 0.979 0.341 1	0	0	0	0	0
	1<br>Oglab03g27330.1 0.517 1 0.483 0.043 1<br>Oglab12g17460.4 0.018 0.483 0.465 0.012 0.996	1<br>Obart01g35640.1 0.516 0.998 0.482 0.063 0.982<br>Obart12g19300.1 0.018 0.483 0.465 0.01 0.99	1<br>Osjap03g31370.3 0.516 0.998 0.482 0.042 0.887<br>Osjap12g24400.2 0.018 0.483 0.465 0.012 0.988	1<br>Osind04g00720.3 0.516 0.999 0.483 0.064 0.919<br>Osind12g20440.1 0.018 0.483 0.465 0.01 0.99	1<br>Orufi03g30600.1 0.516 0.999 0.483 0.05 0.885<br>Orufi12g21550.1 0.018 0.483 0.465 0.01 0.99	1<br>Olong_002926-R1 0.024 0.484 0.46 0.002 0.996<br>Olong_007449-R1 0.516 0.999 0.483 0.065 0.909	1<br>Opunc03g25490.2 0.021 0.487 0.466 0.543 0.811<br>Opunc03g26940.1 0.516 0.999 0.483 0.052 0.885	1<br>Obrac03g20710.1 0.021 0.484 0.463 0.028 0.997<br>Obrac03g21410.1 0.58 0.995 0.415 0.09 0.91	1<br>Lperr03g23190.1 0.517 0.999 0.482 0.039 0.895<br>Lperr03g23200.1 0.008 0.484 0.476 0.008 0.997	0<br>AT5G36940.1 0.019 0.486 0.467 0.003 0.998
Obart03g31750.1	1<br>Oglab03g30830.1 0.046 1 0.954 0.001 1	1<br>Obart03g31750.1 0.001 1 0.999 0.001 1	1<br>Osjap03g34330.1 0.046 1 0.954 0.001 1	1<br>Osind03g35550.1 0.046 1 0.954 0.039 1	1<br>Orufi03g33060.1 0.046 1 0.954 0.001 1	0	0	0	0	0
	1<br>Oglab03g34460.1 0.646 0.997 0.351 0.016 0.993<br>Oglab10g04340.1 0.028 0.602 0.574 0.058 0.963	1<br>Obart03g36200.1 0.667 1 0.333 0.016 0.997<br>Obart10g04600.1 0.058 0.602 0.544 0.106 0.962	1<br>Osjap03g39110.1 0.667 1 0.333 0.036 1<br>Osjap10g05370.1 0.034 0.602 0.568 0.051 0.963	1<br>Osind01g46540.1 0.667 0.999 0.332 0.065 0.994<br>Osind10g03770.1 0.034 0.602 0.568 0.057 0.96	1<br>Orufi10g04850.1 0.034 0.602 0.568 0.054 0.963<br>Orufi11g12520.1 0.668 1 0.332 0.038 0.991	1<br>Olong_014796-R1 0.046 0.518 0.472 0.002 0.95<br>Olong_024872-R1 0.664 1 0.336 0.01 1	1<br>Opunc01g42030.1 0.667 1 0.333 0.28 0.872<br>Opunc03g28940.1 0.041 0.62 0.579 0.049 0.998	1<br>Obrac02g16280.1 0.662 0.982 0.32 0.063 0.949<br>Obrac03g23440.1 0.046 0.62 0.574 0.002 0.998	1<br>Lperr03g30520.1 0.66 1 0.34 0.003 0.997<br>Lperr10g03360.1 0.013 0.602 0.589 0.009 0.962	0<br>AT1G70730.3 0.046 0.618 0.572 0.118 0.995

Obart03g33760.1	1<br>Oglab03g32170.1 0.003 1 0.997 0.003 1<br>Oglab03g32170.2 0.003 1 0.997 0.003 1	1<br>Obart03g33760.1 0.003 1 0.997 0.003 1	1<br>Osjap03g36400.1 0.003 1 0.997 0.003 1	1<br>Osind03g37600.1 0.003 1 0.997 0.003 1	1<br>Orufi03g35150.1 0.003 1 0.997 0.003 1	1<br>Olong_002225-R1 0.003 1 0.997 0.136 1<br>Olong_002225-R2 0.003 1 0.997 0.003 1	1<br>Opunc03g30920.1 0.003 1 0.997 0.003 1	0	0	0
	0<br>Oglab05g19940.1 0.32 0.991 0.671 0.015 0.997	1<br>Obart01g34520.1 0.547 0.991 0.444 0.027 0.818<br>Obart03g04650.1 0.003 0.414 0.411 0.007 1	1<br>Osjap01g12980.2 0.547 0.979 0.432 0.032 0.961<br>Osjap03g04690.1 0.003 0.414 0.411 0.007 1	1<br>Osind03g04960.1 0.003 0.414 0.411 0.007 1<br>Osind12g02610.1 0.538 0.991 0.453 0.005 0.759	1<br>Orufi03g04350.1 0.003 0.414 0.411 0.007 1<br>Orufi05g22410.2 0.547 0.997 0.45 0.031 0.969	1<br>Olong_020510-R2 0.523 0.991 0.468 0.291 0.873<br>Olong_025165-R1 0.003 0.414 0.411 0.007 1	0<br>Opunc12g01920.1 0.526 0.973 0.447 0.663 0.947	0<br>Obrac03g25020.5 0.003 0.535 0.532 0.439 0.972	1<br>Lperr01g29830.2 0.532 0.991 0.459 0.203 0.858<br>Lperr03g28250.1 0.003 0.414 0.411 0.007 1	1<br>AT1G52740.1 0.003 0.414 0.411 0.007 1<br>AT4G14640.1 0.544 0.979 0.435 0.033 0.987
Obart03g38310.1	0	1<br>Obart03g38310.1 0.001 1 0.999 0.001 1	1<br>Osjap03g41450.1 0.076 1 0.924 0.097 1	0	0	0	0	1<br>Obrac03g28610.1 0.065 0.997 0.932 0.081 1<br>Obrac03g28610.2 0.065 0.997 0.932 0.081 1<br>Obrac03g28610.3 0.065 0.997 0.932 0.087 1	0	0
	1<br>Oglab03g36310.1 0.655 1 0.345 0.17 1<br>Oglab03g36320.1 0.064 0.655 0.591 0.137 0.994	0<br>Obart10g07730.1 0.78 0.995 0.215 0.458 0.989	0<br>Osjap10g09140.1 0.78 0.995 0.215 0.458 0.989	1<br>Osind03g42510.1 0.655 1 0.345 0.17 1<br>Osind03g42520.1 0.065 0.655 0.59 0.007 0.993	1<br>Orufi03g39950.1 0.655 1 0.345 0.17 1<br>Orufi03g39960.1 0.076 0.655 0.579 0.155 0.994	1<br>Olong_012011-R1 0.074 0.655 0.581 0.096 0.993<br>Olong_012012-R1 0.655 1 0.345 0.17 1	1<br>Opunc03g35470.1 0.655 1 0.345 0.17 1<br>Opunc03g35480.1 0.064 0.655 0.591 0.144 0.993	0<br>Obrac02g18090.1 0.731 0.997 0.266 0.034 0.998	1<br>Lperr03g32550.1 0.655 1 0.345 0.166 1<br>Lperr03g32560.1 0.072 0.655 0.583 0.144 0.994	0<br>AT1G75150.1 0.144 0.653 0.509 0.255 0.98<br>AT5G66720.1 0.748 0.997 0.249 0.321 1
Obart03g39770.1	0	1<br>Obart03g39770.1 0.002 1 0.998 0.002 1	0	0	0	0	0	0	0	0
	1<br>Oglab03g37820.1 0.363 1 0.637 0.276 1<br>Oglab09g02110.1 0.002 0.343 0.341 0.295 0.798	0<br>Obart09g01500.1 0.002 0.52 0.518 0.287 0.991	1<br>Osjap03g43150.1 0.363 1 0.637 0.276 1<br>Osjap09g02090.1 0.002 0.343 0.341 0.295 0.798	1<br>Osind03g44180.1 0.363 1 0.637 0.276 1<br>Osind09g02120.1 0.002 0.343 0.341 0.295 0.798	1<br>Orufi03g41570.1 0.363 1 0.637 0.276 1<br>Orufi09g02260.1 0.002 0.343 0.341 0.295 0.798	1<br>Olong_008554-R1 0.007 0.338 0.331 0.277 0.777<br>Olong_023902-R1 0.363 1 0.637 0.117 1	1<br>Opunc03g36930.2 0.363 1 0.637 0.292 1<br>Opunc09g01800.1 0.002 0.343 0.341 0.293 0.797	1<br>Obrac03g29930.1 0.363 1 0.637 0.177 1<br>Obrac06g09700.1 0.002 0.334 0.332 0.197 0.885	1<br>Lperr02g05900.1 0.002 0.343 0.341 0.156 0.883<br>Lperr03g33930.1 0.363 1 0.637 0.248 1	1<br>AT2G04570.1 0.002 0.336 0.334 0.283 0.786<br>AT3G55010.1 0.363 0.998 0.635 0.265 0.997
Obart03g41250.1	0	1<br>Obart03g41250.1 0.004 1 0.996 0.004 1	0	0	0	0	0	0	0	0
	1<br>Oglab03g39220.1 0.617 1 0.383 0.136 1<br>Oglab03g39230.1 0.004 0.571 0.567 0.006 0.962	0<br>Obart08g12230.2 0.165 0.481 0.316 0.191 0.832	1<br>Osjap03g44790.1 0.628 1 0.372 0.548 1<br>Osjap03g44800.1 0.004 0.571 0.567 0.006 0.962	1<br>Osind03g45850.1 0.628 1 0.372 0.548 1<br>Osind03g45860.1 0.004 0.571 0.567 0.006 0.962	1<br>Orufi03g43040.1 0.628 1 0.372 0.548 1<br>Orufi03g43050.1 0.004 0.571 0.567 0.006 0.962	0<br>Olong_023863-R1 0.162 0.462 0.3 0.047 0.774	1<br>Opunc03g38330.1 0.699 1 0.301 0.588 1<br>Opunc03g38340.1 0.004 0.571 0.567 0.006 0.962	1<br>Obrac03g31060.1 0.699 1 0.301 0.623 1<br>Obrac03g31070.1 0.004 0.571 0.567 0.006 0.962	1<br>Lperr03g35210.1 0.703 1 0.297 0.632 1<br>Lperr03g35220.1 0.004 0.571 0.567 0.006 0.968	0<br>AT1G73940.1 0.086 0.53 0.444 0.192 0.974<br>AT2G28440.1 0.703 0.977 0.274 0.701 0.981
Obart04g08760.3	0	1<br>Obart04g08760.1 0.001 1 0.999 0.001 1<br>Obart04g08760.2 0.001 1 0.999 0.001 1<br>Obart04g08760.3 0.001 1 0.999 0.001 1	0	0	0	0	0	0	0	0
	1<br>Oglab04g07860.1 0.642 1 0.358 0.134 0.995<br>Oglab05g17370.1 0.161 0.611 0.45 0.43 0.988	0<br>Obart03g10740.1 0.178 0.609 0.431 0.239 0.956<br>Obart04g08830.1 0.659 1 0.341 0.009 1	1<br>Osjap04g11380.1 0.003 0.609 0.606 0.001 0.962<br>Osjap04g11410.1 0.659 1 0.341 0.002 1	1<br>Osind04g09980.1 0.07 0.609 0.539 0.044 0.956<br>Osind04g10500.1 0.642 1 0.358 0.282 0.996	1<br>Orufi04g09970.1 0.07 0.609 0.539 0.042 0.958<br>Orufi04g10520.1 0.642 1 0.358 0.256 0.996	0<br>Olong_005665-R1 0.173 0.611 0.438 0.439 0.988<br>Olong_018692-R1 0.659 1 0.341 0.003 1	1<br>Opunc04g07340.1 0.031 0.609 0.578 0.045 0.96<br>Opunc04g07350.1 0.659 1 0.341 0.003 1	1<br>Obrac04g04890.2 0.023 0.609 0.586 0.039 0.959<br>Obrac04g05050.1 0.659 1 0.341 0.015 0.994	1<br>Lperr04g06080.1 0.145 0.609 0.464 0.09 0.965<br>Lperr09g11650.1 0.655 1 0.345 0.056 0.989	1<br>AT1G23740.1 0.663 0.999 0.336 0.194 0.99<br>AT2G36490.1 0.144 0.609 0.465 0.324 0.985
Obart04g09560.1	0	1<br>Obart04g09560.1 0.001 1 0.999 0.001 1	0	0	0	0	0	0	0	0
	1	1	1	1	1	1	1	1	1	1

	Oglab02g16770.1 0.456 1 0.544 0.031 1<br>Oglab04g07880.1 0.001 0.432 0.431 0.002 0.931	Obart02g17390.1 0.456 1 0.544 0.031 1<br>Obart03g27200.1 0.058 0.44 0.382 0.321 0.866	Osjap04g12100.1 0.456 1 0.544 0.047 1<br>Osjap06g32310.5 0.054 0.456 0.402 0.325 0.956	Osind03g31020.1 0.058 0.44 0.382 0.321 0.866<br>Osind04g10510.1 0.456 1 0.544 0.002 1	Orufi03g28280.1 0.058 0.44 0.382 0.321 0.866<br>Orufi04g10530.1 0.449 1 0.551 0.008 1	Olong_002589-R1 0.058 0.44 0.382 0.263 0.855<br>Olong_009593-R1 0.456 1 0.544 0.034 1	Opunc03g24840.1 0.058 0.44 0.382 0.321 0.869<br>Opunc04g07630.1 0.455 1 0.545 0.046 1	Obrac03g20160.2 0.058 0.449 0.391 0.318 0.911<br>Obrac04g05060.1 0.451 1 0.549 0.05 1	Lperr03g22530.1 0.058 0.44 0.382 0.325 0.867<br>Lperr04g06480.1 0.455 1 0.545 0.045 1	AT1G30490.1 0.059 0.446 0.387 0.316 0.866<br>AT3G25150.2 0.456 0.989 0.533 0.201 0.961
Obart04g11380.1	0	1<br>Obart04g11380.1 0.001 1 0.999 0.001 1	0	0	0	0	0	0	1<br>Lperr04g08120.1 0.001 0.843 0.842 0.001 0.992<br>Lperr04g08120.2 0.001 0.843 0.842 0.001 0.992	0
	1<br>Oglab04g09690.1 0.001 0.26 0.259 0.002 1<br>Oglab04g09700.1 0.273 0.998 0.725 0.046 0.999	0<br>Obart09g12790.1 0.016 0.268 0.252 0.127 0.989	1<br>Osjap04g14100.1 0.273 0.998 0.725 0.051 0.973<br>Osjap09g13100.1 0.016 0.266 0.25 0.02 0.985	1<br>Osind04g12510.1 0.273 0.843 0.57 0.064 0.987<br>Osind09g13310.1 0.016 0.266 0.25 0.018 0.994	1<br>Orufi04g12550.1 0.001 0.26 0.259 0.002 1<br>Orufi04g12560.1 0.273 0.843 0.57 0.063 0.987	0<br>Olong_012592-R1 0.016 0.261 0.245 0.071 0.973<br>Olong_028740-R2 0.471 0.678 0.207 0.022 0.899	1<br>Opunc04g09410.1 0.001 0.26 0.259 0.002 0.998<br>Opunc04g09420.1 0.283 0.999 0.716 0.063 0.999	1<br>Obrac04g06150.1 0.282 1 0.718 0.065 1<br>Obrac09g07340.6 0.016 0.261 0.245 0.059 0.926	0<br>Lperr04g02100.1 0.003 0.26 0.257 0.01 1	0<br>AT1G76130.1 0.011 0.26 0.249 0.044 1<br>AT5G07400.1 0.411 0.811 0.4 0.335 0.944
Obart04g12680.3	0	1<br>Obart04g12680.1 0.001 1 0.999 0.001 1<br>Obart04g12680.2 0.001 1 0.999 0.001 1<br>Obart04g12680.3 0.001 1 0.999 0.001 1	1<br>Osjap04g15460.1 0.001 1 0.999 0.067 1<br>Osjap04g15460.2 0.001 1 0.999 0.068 1<br>Osjap04g15460.3 0.001 1 0.999 0.066 1<br>Osjap04g15460.4 0.001 1 0.999 0.066 1<br>Osjap04g15460.5 0.001 1 0.999 0.067 1<br>Osjap04g15460.6 0.001 1 0.999 0.068 1<br>Osjap04g15460.7 0.001 1 0.999 0.069 1<br>Osjap04g15460.8 0.001 1 0.999 0.068 1	0	0	0	1<br>Opunc04g07830.1 0.001 0.817 0.816 0.085 0.995	0	1<br>Lperr04g09380.1 0.001 0.817 0.816 0.097 0.994	0
	1<br>Oglab04g11000.3 0.329 1 0.671 0.001 1<br>Oglab04g11010.1 0.001 0.286 0.285 0.218 1	0<br>Obart02g19720.1 0.001 0.286 0.285 0.011 1	0<br>Osjap02g22530.1 0.006 0.276 0.27 0.225 0.958	1<br>Osind04g14040.2 0.329 1 0.671 0.001 1<br>Osind04g14050.1 0.001 0.286 0.285 0.218 1	1<br>Orufi04g13810.4 0.329 1 0.671 0.001 1<br>Orufi04g13820.1 0.001 0.286 0.285 0.207 1	1<br>Olong_025780-R1 0.001 0.286 0.285 0.003 1<br>Olong_025781-R3 0.299 0.817 0.518 0.024 0.991	1<br>Opunc04g10610.4 0.329 1 0.671 0.001 0.898<br>Opunc04g10620.1 0.001 0.286 0.285 0.186 1	0<br>Obrac04g07110.3 0.278 1 0.722 0.014 0.916	0<br>Lperr02g15820.1 0.001 0.286 0.285 0.2 1	0<br>AT1G43245.1 0.33 0.813 0.483 0.002 0.998<br>AT1G43580.1 0.001 0.286 0.285 0.164 1
Obart04g26710.1	0	1<br>Obart04g26710.1 0.001 1 0.999 0.001 1<br>Obart04g26710.2 0.001 1 0.999 0.001 1	1<br>Osjap04g30120.1 0.001 1 0.999 0.001 1<br>Osjap04g30120.3 0.001 1 0.999 0.001 1<br>Osjap04g30120.4 0.001 1 0.999 0.001 1	0	1<br>Orufi04g27870.1 0.001 1 0.999 0.001 1<br>Orufi04g27870.2 0.001 1 0.999 0.001 1	1<br>Olong_014711-R2 0.001 0.849 0.848 0.001 0.977	0	1<br>Obrac04g18040.3 0.001 1 0.999 0.001 1	0	0
	1<br>Oglab06g14840.1 0.01 0.632 0.622 0.059 0.882<br>Oglab11g19190.1 0.648 1 0.352 0.042 1	1<br>Obart05g21760.1 0.657 0.996 0.339 0.024 0.993<br>Obart10g13990.1 0.007 0.632 0.625 0.016 0.827	1<br>Osjap04g16240.1 0.007 0.623 0.616 0.07 0.895<br>Osjap05g24420.1 0.657 0.996 0.339 0.024 0.993	1<br>Osind04g29430.2 0.636 1 0.364 0.003 1<br>Osind10g13390.1 0.007 0.632 0.625 0.023 0.826	1<br>Orufi05g23020.1 0.657 0.996 0.339 0.024 0.993<br>Orufi10g14810.1 0.007 0.632 0.625 0.016 0.827	0<br>Olong_014711-R1 0.001 0.645 0.644 0.002 0.837	1<br>Opunc04g23700.2 0.633 1 0.367 0.003 1<br>Opunc05g09290.1 0.003 0.63 0.627 0.038 0.861	0<br>Obrac04g07720.1 0.007 0.633 0.626 0.076 0.934	1<br>Lperr07g00250.2 0.665 1 0.335 0.015 0.99<br>Lperr10g10450.1 0.007 0.632 0.625 0.009 0.826	1<br>AT1G03540.1 0.007 0.623 0.616 0.066 0.995<br>AT4G11290.1 0.643 1 0.357 0.012 1
Obart05g00520.1	0	1<br>Obart05g00520.1 0.001 1 0.999 0.001 1<br>Obart05g00520.2 0.001 0.995 0.994 0.001 0.999	1<br>Osjap05g00530.1 0.001 0.993 0.992 0.084 0.999<br>Osjap05g00530.2 0.001 0.993 0.992 0.103 0.999<br>Osjap05g00530.3 0.001 0.993 0.992 0.104 0.999<br>Osjap05g00530.4 0.001 0.993 0.992 0.091 0.999	1<br>Osind05g00510.1 0.049 0.993 0.944 0.001 0.999	1<br>Orufi05g00500.1 0.001 0.993 0.992 0.084 0.999	1<br>Olong_022405-R1 0.049 0.993 0.944 0.001 0.999	0	0	0	0
	1<br>Oglab05g00500.1 0.001 0.294 0.293 0.218 0.982<br>Oglab09g13820.1 0.322 0.993 0.671 0.064 0.998	1<br>Obart06g01490.1 0.017 0.293 0.276 0.2 0.982<br>Obart09g15270.1 0.34 0.993 0.653 0.029 0.998	1<br>Osjap02g39750.1 0.337 0.988 0.651 0.004 0.956<br>Osjap07g27300.1 0.003 0.291 0.288 0.116 0.834	1<br>Osind09g15830.1 0.34 0.993 0.653 0.029 0.998<br>Osind10g10530.3 0.001 0.336 0.335 0.088 0.826	1<br>Orufi07g24760.1 0.003 0.291 0.288 0.116 0.834<br>Orufi09g16480.1 0.34 0.993 0.653 0.029 0.998	1<br>Olong_018361-R1 0.34 0.993 0.653 0.012 0.998<br>Olong_020432-R1 0.017 0.325 0.308 0.115 0.666	1<br>Opunc05g00480.1 0.329 0.993 0.664 0.007 0.998<br>Opunc05g00490.1 0.001 0.294 0.293 0.228 0.982	1<br>Obrac02g14170.4 0.001 0.3 0.299 0.03 0.843<br>Obrac02g28140.1 0.342 0.989 0.647 0.039 0.973	1<br>Lperr05g00500.1 0.331 0.993 0.662 0.024 0.994<br>Lperr10g08300.1 0.001 0.291 0.29 0.117 0.971	0<br>AT5G44230.1 0.001 0.298 0.297 0.155 0.872

Obart05g00520.2	0	1<br>Obart05g00520.1 0.001 0.999 0.998 0.001 0.995<br>Obart05g00520.2 0.001 1 0.999 0.001 1	1<br>Osjap05g00530.1 0.001 1 0.999 0.084 1<br>Osjap05g00530.2 0.001 1 0.999 0.103 1<br>Osjap05g00530.3 0.001 1 0.999 0.104 1<br>Osjap05g00530.4 0.001 1 0.999 0.091 1	1<br>Osind05g00510.1 0.049 1 0.951 0.001 1	1<br>Orufi05g00500.1 0.001 1 0.999 0.084 1	1<br>Olong_022405-R1 0.049 1 0.951 0.001 1	0	0	0	0
	1<br>Oglab05g00500.1 0.001 0.295 0.294 0.218 0.982<br>Oglab09g13820.1 0.324 1 0.676 0.064 1	1<br>Obart06g01490.1 0.017 0.295 0.278 0.2 0.982<br>Obart09g15270.1 0.342 1 0.658 0.029 1	1<br>Osjap02g39750.1 0.339 0.994 0.655 0.004 0.956<br>Osjap07g27300.1 0.003 0.293 0.29 0.116 0.834	1<br>Osind09g15830.1 0.342 1 0.658 0.029 1<br>Osind10g10530.1 0.001 0.293 0.292 0.108 0.858	1<br>Orufi07g24760.1 0.003 0.293 0.29 0.116 0.834<br>Orufi09g16480.1 0.342 1 0.658 0.029 1	1<br>Olong_018361-R1 0.342 1 0.658 0.012 1<br>Olong_020432-R1 0.017 0.327 0.31 0.115 0.666	1<br>Opunc05g00480.1 0.331 0.999 0.668 0.007 0.998<br>Opunc05g00490.1 0.001 0.295 0.294 0.228 0.982	1<br>Obrac02g14170.4 0.001 0.302 0.301 0.03 0.843<br>Obrac02g28140.1 0.344 0.995 0.651 0.039 0.973	1<br>Lperr05g00500.1 0.334 1 0.666 0.024 0.996<br>Lperr10g08300.1 0.001 0.293 0.292 0.117 0.971	0<br>AT5G44230.1 0.001 0.3 0.299 0.155 0.872
Obart05g03830.1	1<br>Oglab05g03490.1 0.001 1 0.999 0.001 1<br>Oglab05g03490.2 0.001 1 0.999 0.001 1	1<br>Obart05g03830.1 0.001 1 0.999 0.001 1<br>Obart05g03830.2 0.001 1 0.999 0.001 1	1<br>Osjap05g04120.1 0.001 1 0.999 0.001 1<br>Osjap05g04120.2 0.001 1 0.999 0.001 1	1<br>Osind05g04070.1 0.008 1 0.992 0.001 1<br>Osind05g04070.2 0.008 1 0.992 0.001 1	1<br>Orufi05g03950.1 0.001 1 0.999 0.001 1<br>Orufi05g03950.2 0.001 1 0.999 0.001 1	1<br>Olong_016739-R1 0.074 1 0.926 0.001 1	1<br>Opunc05g03540.1 0.001 1 0.999 0.001 1<br>Opunc05g03540.2 0.001 1 0.999 0.001 1	1<br>Obrac05g02930.1 0.074 1 0.926 0.001 1	0	0
	1<br>Oglab01g19490.1 0.09 0.723 0.633 0.066 0.996<br>Oglab03g14060.1 0.79 1 0.21 0.03 1	1<br>Obart01g23310.1 0.09 0.723 0.633 0.066 0.996<br>Obart03g14050.1 0.79 1 0.21 0.03 1	1<br>Osjap01g27410.1 0.09 0.723 0.633 0.066 0.996<br>Osjap03g15140.5 0.743 1 0.257 0.061 1	1<br>Osind01g28630.1 0.09 0.723 0.633 0.066 0.996<br>Osind03g15230.1 0.79 1 0.21 0.03 1	1<br>Orufi01g26130.1 0.09 0.723 0.633 0.066 0.996<br>Orufi03g14720.1 0.79 1 0.21 0.03 1	1<br>Olong_003347-R2 0.09 0.723 0.633 0.047 0.996<br>Olong_009943-R1 0.79 1 0.21 0.03 1	1<br>Opunc01g23460.1 0.09 0.723 0.633 0.064 0.996<br>Opunc03g13590.1 0.79 1 0.21 0.002 0.545	1<br>Obrac01g17590.1 0.09 0.723 0.633 0.067 0.996<br>Obrac03g11330.1 0.79 1 0.21 0.03 1	0<br>Lperr05g03460.1 0.001 0.721 0.72 0.001 0.993	1<br>AT2G47580.1 0.775 1 0.225 0.008 1<br>AT3G16630.1 0.003 0.721 0.718 0.031 0.992
Obart05g03830.2	1<br>Oglab05g03490.1 0.001 1 0.999 0.001 1<br>Oglab05g03490.2 0.001 1 0.999 0.001 1	1<br>Obart05g03830.1 0.001 1 0.999 0.001 1<br>Obart05g03830.2 0.001 1 0.999 0.001 1	1<br>Osjap05g04120.1 0.001 1 0.999 0.001 1<br>Osjap05g04120.2 0.001 1 0.999 0.001 1	1<br>Osind05g04070.1 0.008 1 0.992 0.001 1<br>Osind05g04070.2 0.008 1 0.992 0.001 1	1<br>Orufi05g03950.1 0.001 1 0.999 0.001 1<br>Orufi05g03950.2 0.001 1 0.999 0.001 1	1<br>Olong_016739-R1 0.073 1 0.927 0.001 1	1<br>Opunc05g03540.1 0.001 1 0.999 0.001 1<br>Opunc05g03540.2 0.001 1 0.999 0.001 1	1<br>Obrac05g02930.1 0.073 1 0.927 0.001 1	0	0
	1<br>Oglab01g19490.1 0.089 0.726 0.637 0.066 0.996<br>Oglab03g14060.1 0.792 1 0.208 0.03 1	1<br>Obart01g23310.1 0.089 0.726 0.637 0.066 0.996<br>Obart03g14050.1 0.792 1 0.208 0.03 1	1<br>Osjap01g27410.1 0.089 0.726 0.637 0.066 0.996<br>Osjap03g15140.5 0.747 1 0.253 0.061 1	1<br>Osind01g28630.1 0.089 0.726 0.637 0.066 0.996<br>Osind03g15230.1 0.792 1 0.208 0.03 1	1<br>Orufi01g26130.1 0.089 0.726 0.637 0.066 0.996<br>Orufi03g14720.1 0.792 1 0.208 0.03 1	1<br>Olong_003347-R2 0.089 0.726 0.637 0.047 0.996<br>Olong_009943-R1 0.792 1 0.208 0.03 1	1<br>Opunc01g23460.1 0.089 0.726 0.637 0.064 0.996<br>Opunc03g13590.1 0.792 1 0.208 0.002 0.545	1<br>Obrac01g17590.1 0.089 0.726 0.637 0.067 0.996<br>Obrac03g11330.1 0.792 1 0.208 0.03 1	0<br>Lperr05g03460.1 0.001 0.725 0.724 0.001 0.993	1<br>AT2G47580.1 0.778 1 0.222 0.008 1<br>AT3G16630.1 0.003 0.725 0.722 0.031 0.992
Obart05g04470.1	1<br>Oglab05g04140.1 0.002 1 0.998 0.002 1	1<br>Obart05g04470.1 0.002 1 0.998 0.002 1	0	0	0	0	0	0	0	0
	1<br>Oglab05g02840.1 0.362 0.977 0.615 0.395 0.582<br>Oglab06g15820.1 0.014 0.359 0.345 0.033 0.973	1<br>Obart05g03110.1 0.362 0.977 0.615 0.395 0.582<br>Obart06g05940.1 0.014 0.35 0.336 0.048 0.458	1<br>Osjap05g04830.1 0.002 0.35 0.348 0.006 0.86<br>Osjap08g03110.1 0.359 0.993 0.634 0.031 0.944	0<br>Osind05g03400.1 0.274 0.945 0.671 0.792 0.975	1<br>Orufi05g04690.1 0.002 0.35 0.348 0.006 0.86<br>Orufi05g04700.1 0.35 1 0.65 0.212 1	1<br>Olong_002683-R1 0.359 0.993 0.634 0.031 0.945<br>Olong_015421-R1 0.002 0.35 0.348 0.01 1	1<br>Opunc05g03750.1 0.362 0.984 0.622 0.659 0.972<br>Opunc05g04100.1 0.002 0.35 0.348 0.006 0.878	0<br>Obrac05g03390.1 0.346 0.986 0.64 0.441 0.983	1<br>Lperr03g03510.1 0.023 0.35 0.327 0.073 0.593<br>Lperr05g03760.1 0.5 0.984 0.484 0.279 0.955	0<br>AT3G16720.1 0.002 0.35 0.348 0.059 0.523
Obart05g13050.1	0	1<br>Obart05g13050.1 0.003 1 0.997 0.003 1	0	0	0	0	0	0	0	0
	1<br>Oglab05g12100.1 0.003 0.701 0.698 0.005 0.976<br>Oglab07g01620.1 0.712 0.997 0.285 0.021 0.894	0<br>Obart01g09660.1 0.035 0.458 0.423 0.069 0.806<br>Obart07g01650.1 0.712 0.997 0.285 0.021 0.894	1<br>Osjap05g06280.3 0.712 1 0.288 0.007 0.318<br>Osjap05g14940.1 0.003 0.701 0.698 0.005 0.967	1<br>Osind05g06280.1 0.712 1 0.288 0.023 0.977<br>Osind05g15640.1 0.003 0.701 0.698 0.005 0.967	1<br>Orufi05g14100.1 0.003 0.701 0.698 0.005 0.967<br>Orufi07g05140.1 0.705 1 0.295 0.18 1	0<br>Olong_024811-R1 0.003 0.701 0.698 0.006 0.956	1<br>Opunc05g11230.1 0.003 0.656 0.653 0.005 0.9<br>Opunc11g14700.1 0.712 1 0.288 0.021 0.904	0<br>Obrac05g08550.1 0.003 0.625 0.622 0.005 0.851	0<br>Lperr05g10310.1 0.125 0.694 0.569 0.005 0.962	0<br>AT5G49350.1 0.122 0.549 0.427 0.593 0.967
Obart05g17330.1	0	1<br>Obart04g00290.1 0.003 1 0.997 0.084 0.835<br>Obart05g17330.1 0.003 1 0.997 0.003 1	0	0	0	0	0	0	0	0
	1<br>Oglab05g16420.1 0.681 1 0.319 0.186 0.432	1<br>Obart01g38930.1 0.629 1 0.371 0.221 0.478	1<br>Osjap01g02610.1 0.003 0.613 0.61 0.138 0.36	1<br>Osind01g45750.2 0.629 0.955 0.326 0.265 0.54	1<br>Orufi03g06810.1 0.003 0.635 0.632 0.254 0.985	1<br>Olong_017674-R1 0.674 1 0.326 0.128 0.394	1<br>Opunc02g12800.1 0.006 0.6 0.594 0.212 0.976	0<br>Obrac05g11690.1 0.681 1 0.319 0.199 0.437	0<br>Lperr01g19800.1 0.003 0.29 0.287 0.354 0.818	0<br>AT1G72040.1 0.706 1 0.294 0.378 0.559

	Oglab12g04370.1 0.003 0.613 0.61 0.242 0.582	Obart07g15280.1 0.003 0.558 0.555 0.231 0.531	Osjap05g19900.1 0.681 1 0.319 0.173 0.422	Osind07g15230.1 0.003 0.565 0.562 0.101 0.359	Orufi05g18690.1 0.681 1 0.319 0.19 0.434	Olong_018765-R1 0.006 0.613 0.607 0.066 0.849	Opunc07g08640.2 0.674 1 0.326 0.196 0.424		Lperr05g13860.1 0.677 1 0.323 0.279 0.498	
Obart05g20080.1	0	1<br>Obart05g20080.1 0.001 1 0.999 0.001 1	0	0	0	0	0	0	0	0
	1<br>Oglab05g19000.1 0.001 0.664 0.663 0.066 0.939<br>Oglab09g10550.1 0.666 0.994 0.328 0.217 0.958	1<br>Obart07g26300.1 0.026 0.661 0.635 0.033 0.967<br>Obart09g11580.1 0.666 0.994 0.328 0.208 0.959	1<br>Osjap05g22670.1 0.001 0.664 0.663 0.063 0.939<br>Osjap09g11800.1 0.666 0.994 0.328 0.217 0.958	1<br>Osind07g26930.1 0.026 0.661 0.635 0.033 0.967<br>Osind09g11940.1 0.666 0.994 0.328 0.258 0.969	1<br>Orufi05g21340.1 0.001 0.664 0.663 0.002 0.935<br>Orufi09g12430.2 0.666 0.994 0.328 0.217 0.958	1<br>Olong_005541-R1 0.03 0.624 0.594 0.039 0.917<br>Olong_025141-R1 0.659 0.996 0.337 0.279 1	1<br>Opunc05g17810.1 0.009 0.664 0.655 0.075 0.945<br>Opunc09g10120.1 0.666 0.994 0.328 0.399 0.973	1<br>Obrac07g17500.1 0.013 0.66 0.647 0.034 0.94<br>Obrac09g06450.1 0.666 0.994 0.328 0.182 0.942	1<br>Lperr05g16000.1 0.023 0.664 0.641 0.022 0.942<br>Lperr09g08210.1 0.666 0.994 0.328 0.253 0.964	1<br>AT2G26830.1 0.666 0.994 0.328 0.278 0.971<br>AT2G40480.1 0.016 0.661 0.645 0.081 0.969
Obart05g25070.1	1<br>Oglab05g23160.1 0.001 1 0.999 0.001 1	1<br>Obart05g25070.1 0.001 1 0.999 0.001 1	1<br>Osjap05g28230.1 0.001 1 0.999 0.001 1	1<br>Osind05g28520.1 0.001 1 0.999 0.001 1	1<br>Orufi05g26870.1 0.001 1 0.999 0.001 1	0	1<br>Opunc05g22640.1 0.001 1 0.999 0.001 1	0	1<br>Lperr01g22990.1 0.001 1 0.999 0.027 1	0
	1<br>Oglab01g22740.1 0.028 0.451 0.423 0.061 0.995<br>Oglab01g22750.1 0.462 0.999 0.537 0.009 0.999	1<br>Obart01g26870.1 0.006 0.451 0.445 0.117 0.995<br>Obart01g26880.1 0.462 0.999 0.537 0.009 0.999	1<br>Osjap01g31140.1 0.007 0.451 0.444 0.093 0.995<br>Osjap01g31150.1 0.462 0.999 0.537 0.009 0.999	1<br>Osind01g32600.1 0.001 0.451 0.45 0.002 0.995<br>Osind01g32610.1 0.462 0.999 0.537 0.009 0.999	1<br>Orufi01g29880.1 0.001 0.451 0.45 0.075 0.995<br>Orufi01g29890.1 0.462 0.999 0.537 0.009 0.999	1<br>Olong_000367-R1 0.105 0.451 0.346 0.002 0.993<br>Olong_000368-R1 0.456 0.999 0.543 0.108 0.999	1<br>Opunc01g26820.1 0.049 0.451 0.402 0.002 0.994<br>Opunc01g26830.1 0.462 1 0.538 0.009 1	1<br>Obrac01g20400.1 0.046 0.451 0.405 0.004 0.994<br>Obrac05g17560.2 0.451 1 0.549 0.164 1	1<br>Lperr05g20850.2 0.467 1 0.533 0.002 1<br>Lperr05g20860.1 0.001 0.451 0.45 0.002 0.995	1<br>AT5G08690.1 0.012 0.451 0.439 0.034 0.995<br>AT5G51600.1 0.462 0.999 0.537 0.008 0.996
Obart05g27940.1	0	1<br>Obart05g27940.1 0.001 1 0.999 0.001 1	0	1<br>Osind05g31580.1 0.001 1 0.999 0.024 1	0	1<br>Olong_007981-R1 0.059 1 0.941 0.001 1	0	0	0	0
	1<br>Oglab05g25920.1 0.001 0.581 0.58 0.035 0.982<br>Oglab05g25930.1 0.589 1 0.411 0.07 1	1<br>Obart01g20890.1 0.589 0.98 0.391 0.063 0.943<br>Obart07g06670.1 0.158 0.582 0.424 0.36 0.978	1<br>Osjap01g24630.2 0.589 0.98 0.391 0.156 0.951<br>Osjap05g31230.3 0.001 0.581 0.58 0.041 0.982	1<br>Osind01g25470.1 0.002 0.536 0.534 0.073 0.967<br>Osind01g25480.1 0.589 0.954 0.365 0.063 0.877	1<br>Orufi01g23560.1 0.589 0.98 0.391 0.144 0.949<br>Orufi05g29910.3 0.001 0.581 0.58 0.036 0.982	1<br>Olong_011279-R1 0.589 0.991 0.402 0.063 0.978<br>Olong_011281-R2 0.002 0.565 0.563 0.019 1	1<br>Opunc05g25540.1 0.001 0.581 0.58 0.037 0.983<br>Opunc05g25550.1 0.589 1 0.411 0.067 1	0<br>Obrac05g19820.2 0.001 0.581 0.58 0.002 0.982	1<br>Lperr01g17820.1 0.002 0.582 0.58 0.023 0.973<br>Lperr01g17840.1 0.589 0.998 0.409 0.166 0.978	1<br>AT1G07990.1 0.589 0.917 0.328 0.061 0.782<br>AT4G05420.1 0.001 0.581 0.58 0.002 0.982
Obart06g00880.1	1<br>Oglab06g00840.1 0.001 1 0.999 0.001 1	1<br>Obart06g00880.1 0.001 1 0.999 0.001 1	1<br>Osjap06g00940.1 0.04 1 0.96 0.085 1	0	1<br>Orufi06g00860.1 0.067 1 0.933 0.021 1	0	0	0	0	0
	1<br>Oglab06g00730.1 0.526 0.99 0.464 0.257 0.968<br>Oglab07g19660.1 0.033 0.458 0.425 0.142 0.938	1<br>Obart06g00690.1 0.529 1 0.471 0.099 1<br>Obart09g03180.1 0.021 0.458 0.437 0.145 1	1<br>Osjap06g00950.1 0.529 0.992 0.463 0.132 0.99<br>Osjap08g13790.1 0.051 0.458 0.407 0.005 1	1<br>Osind06g00910.1 0.526 1 0.474 0.068 1<br>Osind08g15520.1 0.051 0.458 0.407 0.005 1	1<br>Orufi06g00690.1 0.529 1 0.471 0.099 1<br>Orufi06g00860.2 0.001 0.445 0.444 0.005 0.98	0<br>Olong_023831-R1 0.053 0.583 0.53 0.001 0.373	1<br>Opunc06g00800.1 0.457 1 0.543 0.025 1<br>Opunc06g00830.1 0.05 0.457 0.407 0.006 1	1<br>Obrac06g00660.1 0.526 1 0.474 0.034 0.999<br>Obrac07g14790.1 0.058 0.503 0.445 0.093 0.948	1<br>Lperr01g40030.1 0.055 0.457 0.402 0.013 0.994<br>Lperr06g00740.1 0.531 0.999 0.468 0.078 0.997	0<br>AT4G34470.1 0.063 0.457 0.394 0.013 1
Obart06g03390.1	0	1<br>Obart06g03390.1 0.001 1 0.999 0.001 1	0	0	0	0	0	0	0	0
	1<br>Oglab06g03530.1 0.411 1 0.589 0.002 1<br>Oglab06g03540.1 0.001 0.322 0.321 0.004 0.923	1<br>Obart01g10160.1 0.421 0.979 0.558 0.025 0.943<br>Obart06g25350.1 0.019 0.331 0.312 0.24 0.91	1<br>Osjap06g03820.1 0.411 1 0.589 0.002 0.797<br>Osjap06g03830.1 0.001 0.322 0.321 0.003 0.924	1<br>Osind06g04060.1 0.412 1 0.588 0.005 0.871<br>Osind06g04070.1 0.001 0.322 0.321 0.004 0.923	1<br>Orufi06g03390.1 0.411 1 0.589 0.002 1<br>Orufi06g03400.1 0.001 0.322 0.321 0.003 0.924	1<br>Olong_004095-R1 0.421 0.974 0.553 0.015 0.951<br>Olong_008568-R1 0.001 0.316 0.315 0.004 0.888	1<br>Opunc06g03190.1 0.419 1 0.581 0.004 1<br>Opunc06g03210.1 0.001 0.319 0.318 0.345 0.985	1<br>Obrac06g02440.1 0.419 1 0.581 0.004 1<br>Obrac06g02460.1 0.001 0.389 0.388 0.499 0.972	1<br>Lperr06g03190.1 0.415 1 0.585 0.004 1<br>Lperr06g03200.1 0.001 0.315 0.314 0.083 0.862	1<br>AT3G13700.1 0.014 0.347 0.333 0.02 0.99<br>AT5G16410.1 0.423 0.979 0.556 0.069 1
Obart06g20680.1	0	1<br>Obart06g20680.1 0.001 1 0.999 0.001 1	0	1<br>Osind06g23650.1 0.001 1 0.999 0.001 1	1<br>Orufi06g22110.1 0.001 1 0.999 0.011 1	0	0	0	0	0
	1<br>Oglab05g13700.1 0.625 1 0.375 0.094 1<br>Oglab06g18700.1 0.001 0.623 0.622 0.016 0.986	1<br>Obart01g27850.1 0.111 0.61 0.499 0.006 0.954<br>Obart05g14650.1 0.625 1 0.375 0.094 1	1<br>Osjap02g34710.2 0.637 0.998 0.361 0.12 0.997<br>Osjap06g24310.1 0.001 0.623 0.622 0.059 0.986	1<br>Osind05g17380.1 0.625 1 0.375 0.094 1<br>Osind09g14580.1 0.026 0.623 0.597 0.005 0.975	1<br>Orufi05g15730.1 0.625 1 0.375 0.094 1<br>Orufi09g15100.1 0.012 0.623 0.611 0.029 0.975	1<br>Olong_017051-R1 0.625 1 0.375 0.104 1<br>Olong_021346-R1 0.001 0.623 0.622 0.01 0.982	1<br>Opunc03g03090.1 0.016 0.639 0.623 0.028 1<br>Opunc05g12780.1 0.641 1 0.359 0.139 1	1<br>Obrac02g22310.1 0.628 0.998 0.37 0.012 0.997<br>Obrac09g08370.1 0.021 0.618 0.597 0.023 0.961	1<br>Lperr02g25000.1 0.628 0.998 0.37 0.012 0.997<br>Lperr07g09420.1 0.029 0.627 0.598 0.034 0.995	1<br>AT3G07940.1 0.637 1 0.363 0.094 1<br>AT5G58830.1 0.008 0.623 0.615 0.019 0.984

Obart06g24730.4	0	1<br>Obart06g24730.4 0.002 1 0.998 0.002 1	0	0	0	0	0	0	0	0
	1<br>Oglab02g04920.1 0.002 0.695 0.693 0 0.105<br>Oglab11g03050.1 0.727 0.952 0.225 0.06 0.354	0<br>Obart06g24730.1 0.002 0.695 0.693 0.001 0.313	1<br>Osjap02g05090.1 0.002 0.695 0.693 0 0.105<br>Osjap11g03420.1 0.727 0.952 0.225 0.066 0.358	1<br>Osind02g05400.1 0.002 0.695 0.693 0.001 0.305<br>Osind11g02780.1 0.727 0.952 0.225 0.06 0.354	1<br>Orufi02g04910.1 0.002 0.695 0.693 0 0.115<br>Orufi12g03280.1 0.701 0.952 0.251 0.039 0.373	1<br>Olong_000683-R1 0.727 0.952 0.225 0.06 0.354<br>Olong_011615-R1 0.002 0.695 0.693 0 0.112	1<br>Opunc02g04040.3 0.002 0.695 0.693 0 0.113<br>Opunc11g02770.1 0.727 0.952 0.225 0.06 0.356	0<br>Obrac06g17230.1 0.102 0.695 0.593 0.002 0.106	1<br>Lperr02g04220.2 0.002 0.695 0.693 0 0.112<br>Lperr12g02790.1 0.715 0.952 0.237 0.047 0.353	0
Obart06g25430.1	1<br>Oglab06g22820.1 0.004 1 0.996 0.005 1	1<br>Obart06g25430.1 0.004 1 0.996 0.004 1	0	0	0	0	0	0	0	0
	1<br>Oglab06g20540.1 0.004 0.419 0.415 0.625 0.915<br>Oglab09g12320.1 0.447 0.984 0.537 0.186 0.994	1<br>Obart06g25600.1 0.059 0.447 0.388 0.721 0.989<br>Obart09g13660.1 0.447 0.984 0.537 0.022 0.993	1<br>Osjap06g26550.1 0.004 0.368 0.364 0.698 0.988<br>Osjap09g14010.1 0.447 0.984 0.537 0.022 0.993	1<br>Osind06g29050.1 0.012 0.419 0.407 0.655 0.91<br>Osind09g14180.1 0.455 0.992 0.537 0.013 0.886	1<br>Orufi06g27530.1 0.059 0.447 0.388 0.721 0.989<br>Orufi09g14650.1 0.447 0.984 0.537 0.186 0.994	0<br>Olong_024432-R1 0.474 0.984 0.51 0.174 0.994	1<br>Opunc06g23340.1 0.004 0.289 0.285 0.367 0.975<br>Opunc09g12070.1 0.455 0.988 0.533 0.014 0.916	0<br>Obrac09g08010.1 0.443 0.984 0.541 0.008 0.992	0<br>Lperr09g09890.1 0.348 0.984 0.636 0.018 0.994	0<br>AT4G30380.1 0.494 0.976 0.482 0.033 0.984
Obart06g28060.1	1<br>Oglab06g25490.1 0.001 1 0.999 0.001 1<br>Oglab09g15660.1 0.024 0.999 0.975 0.154 0.95	1<br>Obart06g28060.1 0.001 1 0.999 0.001 1<br>Obart09g17460.1 0.024 1 0.976 0.076 0.969<br>Obart09g17460.2 0.024 1 0.976 0.076 0.969	1<br>Osjap09g17800.1 0.024 1 0.976 0.113 0.974<br>Osjap09g17800.2 0.024 1 0.976 0.114 0.974<br>Osjap09g17800.3 0.024 0.997 0.973 0.187 0.947	1<br>Osind06g31620.1 0.001 1 0.999 0.001 1	1<br>Orufi06g29990.1 0.001 1 0.999 0.001 1<br>Orufi09g18790.1 0.024 1 0.976 0.107 0.97<br>Orufi09g18790.2 0.024 1 0.976 0.107 0.97<br>Orufi09g18790.3 0.024 1 0.976 0.108 0.969<br>Orufi09g18790.4 0.024 1 0.976 0.108 0.969<br>Orufi09g18790.5 0.024 0.976 0.952 0.17 0.937<br>Orufi09g18790.6 0.024 0.996 0.972 0.172 0.937	1<br>Olong_016700-R1 0.001 1 0.999 0.001 1	0	0	1<br>Lperr09g13380.1 0.084 0.982 0.898 0.166 0.951	0
	1<br>Oglab02g01250.1 0.066 0.406 0.34 0.221 0.93<br>Oglab10g06880.1 0.427 1 0.573 0.099 1	1<br>Obart04g21770.1 0.07 0.417 0.347 0.232 0.933<br>Obart10g07310.1 0.427 1 0.573 0.099 1	1<br>Osjap06g32830.1 0.427 1 0.573 0.085 1<br>Osjap06g32840.1 0.001 0.407 0.406 0.002 0.952	1<br>Osind04g24640.1 0.07 0.417 0.347 0.233 0.933<br>Osind06g30840.1 0.485 0.999 0.514 0.109 1	0<br>Orufi06g29150.4 0.485 0.999 0.514 0.122 1	1<br>Olong_000286-R1 0.066 0.406 0.34 0.258 0.928<br>Olong_026973-R2 0.427 1 0.573 0.099 1	1<br>Opunc06g25610.1 0.001 0.407 0.406 0.002 0.951<br>Opunc10g06480.1 0.427 0.998 0.571 0.098 0.984	1<br>Obrac06g20010.2 0.432 1 0.568 0.119 1<br>Obrac06g20020.1 0.001 0.407 0.406 0.002 0.952	1<br>Lperr02g22230.1 0.043 0.392 0.349 0.113 0.88<br>Lperr06g23510.1 0.402 1 0.598 0.074 1	1<br>AT1G76360.1 0.036 0.406 0.37 0.219 0.952<br>AT3G61130.1 0.465 0.999 0.534 0.207 1
Obart07g03720.1	0	1<br>Obart07g03720.1 0.004 1 0.996 0.004 1	0	0	0	0	0	0	0	0
	1<br>Oglab07g03590.1 0.363 1 0.637 0.007 1<br>Oglab07g03600.1 0.004 0.329 0.325 0.004 0.308	0<br>Obart07g03710.1 0.363 1 0.637 0.007 1	1<br>Osjap07g04140.1 0.363 1 0.637 0.007 1<br>Osjap07g04150.1 0.004 0.329 0.325 0.004 0.305	1<br>Osind07g03650.1 0.363 1 0.637 0.007 1<br>Osind07g03660.1 0.004 0.329 0.325 0.004 0.305	1<br>Orufi07g03430.1 0.363 1 0.637 0.007 1<br>Orufi07g03440.1 0.004 0.329 0.325 0.001 0.07	0<br>Olong_008621-R1 0.464 1 0.536 0.181 1	1<br>Opunc07g03640.1 0.363 1 0.637 0.007 1<br>Opunc07g03650.1 0.004 0.329 0.325 0.004 0.306	0<br>Obrac07g02220.1 0.46 1 0.54 0.174 1	1<br>Lperr07g03220.1 0.363 1 0.637 0.007 1<br>Lperr07g03230.1 0.004 0.308 0.304 0.004 0.24	0<br>AT3G44100.1 0.338 0.962 0.624 0.026 0.941
Obart07g03940.1	0	1<br>Obart07g03940.1 0.001 1 0.999 0.001 1	0	0	0	0	0	0	0	0
	1<br>Oglab01g03190.1 0.435 0.989 0.554 0.002 0.994<br>Oglab03g35120.1 0.038 0.429 0.391 0.002 1	1<br>Obart03g36870.1 0.038 0.393 0.355 0.002 0.889<br>Obart09g12990.1 0.441 0.99 0.549 0.054 0.997	1<br>Osjap01g04010.1 0.435 0.989 0.554 0.002 1<br>Osjap03g39900.1 0.038 0.429 0.391 0.002 1	1<br>Osind01g04300.1 0.441 0.998 0.557 0.001 0.994<br>Osind03g40960.1 0.038 0.429 0.391 0.002 1	1<br>Orufi03g38420.1 0.038 0.429 0.391 0.002 1<br>Orufi09g13930.1 0.441 0.99 0.549 0.079 1	1<br>Olong_012584-R1 0.463 0.991 0.528 0.017 0.998<br>Olong_020874-R1 0.038 0.339 0.301 0.004 1	1<br>Opunc01g03200.2 0.434 0.992 0.558 0.043 0.939<br>Opunc03g33860.1 0.044 0.395 0.351 0.012 0.894	0<br>Obrac09g07500.1 0.442 0.986 0.544 0.102 0.963	1<br>Lperr03g32440.1 0.074 0.414 0.34 0.073 0.966<br>Lperr07g03510.1 0.42 0.989 0.569 0.011 0.992	0<br>AT5G06570.1 0.45 0.989 0.539 0.003 1
Obart07g07590.1	0	1<br>Obart07g07590.1 0.001 1 0.999 0.001 1	0	0	0	0	0	0	0	0
	1<br>Oglab07g07310.1 0.001 0.435 0.434 0.002 0.88	0<br>Obart03g10940.1 0.611 0.991 0.38 0.085 0.864	1<br>Osjap07g08860.1 0.001 0.435 0.434 0.002 0.88	1<br>Osind07g07980.1 0.001 0.435 0.434 0.002 0.88	1<br>Orufi07g07560.1 0.001 0.435 0.434 0.002 0.88	0<br>Olong_025526-R1 0.205 0.435 0.23 0.097 0.801	1<br>Opunc07g07290.1 0.001 0.435 0.434 0.002 0.881	1<br>Obrac03g08780.1 0.612 0.991 0.379 0.015 0.852	1<br>Lperr07g06420.1 0.001 0.465 0.464 0.002 0.935	1<br>AT2G33260.1 0.615 1 0.385 0.028 0.943

	Oglab07g07330.1 0.576 1 0.424 0.104 0.867		Osjap07g08870.1 0.437 1 0.563 0.125 0.908	Osind07g08000.1 0.576 1 0.424 0.104 0.867	Orufi07g07580.1 0.576 1 0.424 0.104 0.867	Olong_025527-R1 0.635 1 0.365 0.002 0.889	Opunc07g07300.1 0.577 1 0.423 0.125 1	Obrac07g04600.1 0.001 0.435 0.434 0.002 0.88	Lperr07g06430.1 0.57 1 0.43 0.101 0.879	AT2G47990.1 0.01 0.446 0.436 0.034 0.902
Obart07g11000.2	1<br>Oglab07g10660.1 0.001 0.923 0.922 0.1 0.995<br>Oglab07g10660.3 0.001 0.923 0.922 0.106 0.995<br>Oglab07g10660.4 0.001 0.923 0.922 0.109 0.995	1<br>Obart07g11000.1 0.001 1 0.999 0.001 1<br>Obart07g11000.2 0.001 1 0.999 0.001 1<br>Obart07g11000.3 0.001 1 0.999 0.001 1	1<br>Osjap07g12650.1 0.001 1 0.999 0.094 1<br>Osjap07g12650.2 0.001 1 0.999 0.099 1<br>Osjap07g12650.3 0.001 1 0.999 0.102 1	1<br>Osind03g00080.1 0.001 1 0.999 0.001 1	1<br>Orufi07g11170.1 0.001 1 0.999 0.1 1<br>Orufi07g11170.2 0.001 1 0.999 0.096 1<br>Orufi07g11170.3 0.001 1 0.999 0.098 1<br>Orufi07g11170.4 0.001 1 0.999 0.101 1<br>Orufi07g11170.5 0.001 1 0.999 0.102 1	1<br>Olong_020395-R1 0.001 1 0.999 0.001 1	0	0	0	0
	0<br>Oglab07g10660.2 0.001 0.745 0.744 0.128 0.998	1<br>Obart09g19100.1 0.729 0.974 0.245 0.319 1<br>Obart10g14260.1 0.002 0.716 0.714 0.13 0.992	1<br>Osjap01g03910.2 0.027 0.717 0.69 0.126 0.736<br>Osjap09g19490.1 0.729 0.974 0.245 0.193 1	1<br>Osind08g00800.1 0.036 0.726 0.69 0.111 0.925<br>Osind09g19670.1 0.729 0.974 0.245 0.184 1	1<br>Orufi05g10490.3 0.028 0.707 0.679 0.103 0.96<br>Orufi09g20690.1 0.729 0.974 0.245 0.319 1	1<br>Olong_002950-R1 0.002 0.711 0.709 0.027 0.982<br>Olong_014211-R1 0.729 0.941 0.212 0.332 0.998	0<br>Opunc06g16990.1 0.018 0.76 0.742 0.098 0.997	1<br>Obrac05g06630.4 0.008 0.713 0.705 0.114 0.869<br>Obrac09g12470.1 0.729 0.974 0.245 0.318 1	1<br>Lperr03g21150.4 0.008 0.713 0.705 0.06 0.973<br>Lperr09g14930.2 0.729 0.974 0.245 0.317 1	0<br>AT5G61990.1 0.001 0.744 0.743 0.044 0.999
Obart07g19760.1	0	1<br>Obart07g19760.1 0.002 1 0.998 0.002 1	0	0	0	0	1<br>Opunc07g18680.1 0.002 1 0.998 0.125 1<br>Opunc07g18680.2 0.002 1 0.998 0.12 1<br>Opunc07g18700.1 0.002 1 0.998 0.138 1<br>Opunc07g18700.2 0.002 1 0.998 0.115 1	0	1<br>Lperr07g17150.1 0.002 1 0.998 0.111 1	0
	1<br>Oglab07g04490.1 0.331 0.998 0.667 0.117 0.993<br>Oglab09g04130.1 0.002 0.265 0.263 0.074 0.973	1<br>Obart07g04680.1 0.337 0.998 0.661 0.003 0.994<br>Obart09g04230.1 0.002 0.265 0.263 0.074 0.973	1<br>Osjap07g05300.1 0.319 0.998 0.679 0.079 0.994<br>Osjap09g04300.1 0.002 0.265 0.263 0.074 0.973	1<br>Osind07g19860.1 0.002 0.269 0.267 0.402 0.996<br>Osind07g19870.1 0.337 1 0.663 0.003 1	1<br>Orufi07g04510.1 0.319 0.998 0.679 0.079 0.994<br>Orufi09g04740.1 0.002 0.265 0.263 0.074 0.973	1<br>Olong_011782-R1 0.002 0.265 0.263 0.074 0.973<br>Olong_015820-R2 0.337 1 0.663 0.003 1	1<br>Opunc07g04580.1 0.337 0.998 0.661 0.003 0.994<br>Opunc09g03700.1 0.002 0.265 0.263 0.074 0.973	1<br>Obrac07g12480.1 0.39 1 0.61 0.003 1<br>Obrac09g02110.1 0.002 0.265 0.263 0.074 0.973	0<br>Lperr09g02940.1 0.002 0.265 0.263 0.074 0.973	0<br>AT3G08690.1 0.012 0.273 0.261 0.128 1
Obart07g21100.1	0	1<br>Obart07g21100.1 0.002 1 0.998 0.002 1<br>Obart07g21100.2 0.002 1 0.998 0.001 1	0	0	0	0	0	0	1<br>Lperr07g18240.1 0.153 0.994 0.841 0.123 0.997	0
	0<br>Oglab07g18200.1 0.232 1 0.768 0.193 1	1<br>Obart03g02790.1 0.002 0.223 0.221 0.004 0.649<br>Obart07g08840.2 0.227 0.954 0.727 0.132 0.896	1<br>Osjap03g02790.1 0.002 0.223 0.221 0.004 0.649<br>Osjap07g24570.1 0.232 1 0.768 0.193 1	1<br>Osind03g02880.1 0.002 0.223 0.221 0.004 0.649<br>Osind07g21400.2 0.232 0.998 0.766 0.491 0.995	0<br>Orufi07g22120.1 0.232 1 0.768 0.193 1	1<br>Olong_005980-R1 0.002 0.223 0.221 0.002 0.878<br>Olong_006540-R1 0.243 1 0.757 0.062 1	1<br>Opunc03g02540.1 0.002 0.221 0.219 0.002 0.817<br>Opunc07g13100.1 0.232 1 0.768 0.191 1	1<br>Obrac03g02180.3 0.002 0.223 0.221 0.004 0.649<br>Obrac07g13370.3 0.24 0.994 0.754 0.002 0.994	1<br>Lperr08g11570.3 0.27 0.962 0.692 0.228 0.789<br>Lperr10g13340.1 0.002 0.247 0.245 0.004 0.62	0<br>AT1G26460.1 0.232 0.985 0.753 0.203 0.989
Obart07g23980.1	0	1<br>Obart07g23980.1 0.003 1 0.997 0.003 1	0	0	0	0	0	0	0	0
	1<br>Oglab03g36900.1 0.003 0.276 0.273 0.59 0.785<br>Oglab09g13800.1 0.295 1 0.705 0.34 1	1<br>Obart03g38900.1 0.003 0.276 0.273 0.322 0.644<br>Obart09g15250.1 0.295 1 0.705 0.284 1	1<br>Osjap03g42080.1 0.003 0.292 0.289 0.432 0.749<br>Osjap09g15670.1 0.295 1 0.705 0.334 1	1<br>Osind09g15810.1 0.295 1 0.705 0.34 1<br>Osind11g00110.1 0.003 0.287 0.284 0.555 0.776	1<br>Orufi03g40560.1 0.003 0.276 0.273 0.492 0.733<br>Orufi09g16460.1 0.295 1 0.705 0.339 1	0<br>Olong_003491-R1 0.003 0.357 0.354 0.375 0.732	1<br>Opunc05g11140.1 0.003 0.276 0.273 0.544 0.745<br>Opunc07g22640.1 0.295 1 0.705 0.325 1	1<br>Obrac03g29030.2 0.003 0.276 0.273 0.463 0.718<br>Obrac09g09270.1 0.279 0.981 0.702 0.174 0.993	0<br>Lperr01g12120.1 0.239 0.979 0.74 0.278 0.995	1<br>AT2G24580.1 0.292 0.987 0.695 0.325 0.969<br>AT2G40320.1 0.003 0.282 0.279 0.485 0.734
Obart07g27030.1	0	1<br>Obart07g27030.1 0.001 1 0.999 0.001 1<br>Obart07g27030.2 0.001 1 0.999 0.001 1	0	0	0	0	0	0	1<br>Lperr07g23810.2 0.001 1 0.999 0.381 1<br>Lperr07g23810.3 0.001 0.946 0.945 0.424 0.986	0
	1<br>Oglab07g23620.1 0.348 1 0.652 0.002 1<br>Oglab07g23630.1 0.001 0.311 0.31 0.574 0.982	0<br>Obart06g10380.1 0.374 0.927 0.553 0.107 0.984	1<br>Osjap07g30980.1 0.348 1 0.652 0.003 1<br>Osjap07g31000.1 0.001 0.222 0.221 0.659 0.973	1<br>Osind07g27700.1 0.348 1 0.652 0.002 1<br>Osind07g27720.1 0.001 0.333 0.332 0.537 0.945	0<br>Orufi07g27980.1 0.333 1 0.667 0.091 1	1<br>Olong_001492-R1 0.002 0.333 0.331 0.173 0.995<br>Olong_008701-R1 0.348 1 0.652 0.002 1	1<br>Opunc07g25410.1 0.348 1 0.652 0.002 1<br>Opunc07g25420.1 0.066 0.29 0.224 0.005 0.995	0<br>Obrac07g18100.1 0.348 0.978 0.63 0.004 0.967	0<br>Lperr07g23810.1 0.348 0.98 0.632 0.002 0.96	0<br>AT3G66658.2 0.348 0.997 0.649 0.002 0.997

Obart07g27030.2	0	1<br>Obart07g27030.1 0.001 1 0.999 0.001 1<br>Obart07g27030.2 0.001 1 0.999 0.001 1	0	0	0	0	0	0	1<br>Lperr07g23810.2 0.001 1 0.999 0.381 1<br>Lperr07g23810.3 0.001 0.945 0.944 0.424 0.986	0
	1<br>Oglab07g23620.1 0.354 1 0.646 0.002 1<br>Oglab07g23630.1 0.001 0.317 0.316 0.574 0.982	0<br>Obart06g10380.1 0.381 0.925 0.544 0.107 0.984	1<br>Osjap07g30980.1 0.354 1 0.646 0.003 1<br>Osjap07g31000.1 0.001 0.226 0.225 0.659 0.973	1<br>Osind07g27700.1 0.354 1 0.646 0.002 1<br>Osind07g27720.1 0.001 0.339 0.338 0.537 0.945	0<br>Orufi07g27980.1 0.336 1 0.664 0.086 1	1<br>Olong_001492-R1 0.002 0.339 0.337 0.173 0.995<br>Olong_008701-R1 0.354 1 0.646 0.002 1	1<br>Opunc07g25410.1 0.354 1 0.646 0.002 1<br>Opunc07g25420.1 0.067 0.296 0.229 0.005 0.995	0<br>Obrac07g18100.1 0.354 0.977 0.623 0.004 0.967	0<br>Lperr07g23810.1 0.354 0.979 0.625 0.002 0.96	0<br>AT3G66658.2 0.354 0.997 0.643 0.002 0.997
Obart07g27040.1	0	1<br>Obart07g27040.1 0.002 1 0.998 0.002 1<br>Obart07g27040.2 0.002 1 0.998 0.002 1	0	0	0	0	0	0	0	0
	1<br>Oglab07g23640.2 0.002 0.324 0.322 0.813 0.998<br>Oglab08g15450.1 0.4 0.974 0.574 0.067 0.483	1<br>Obart07g27030.1 0.002 0.249 0.247 0.348 0.51<br>Obart08g16640.1 0.4 0.974 0.574 0.035 0.802	1<br>Osjap07g30980.1 0.002 0.244 0.242 0.003 0.358<br>Osjap07g31000.1 0.318 0.974 0.656 0.002 0.608	1<br>Osind07g27700.1 0.002 0.249 0.247 0.002 0.258<br>Osind07g27720.1 0.318 0.974 0.656 0.012 0.493	0<br>Orufi03g37620.1 0.237 0.884 0.647 0.006 0.997	1<br>Olong_008699-R1 0.315 0.974 0.659 0.003 0.523<br>Olong_008701-R1 0.002 0.249 0.247 0.002 0.258	1<br>Opunc07g25410.1 0.002 0.249 0.247 0.002 0.268<br>Opunc08g14550.1 0.4 0.974 0.574 0.08 0.527	1<br>Obrac07g18100.1 0.002 0.249 0.247 0.004 0.271<br>Obrac08g10910.1 0.4 0.974 0.574 0.075 0.529	0<br>Lperr07g23810.2 0.322 0.974 0.652 0.009 0.348	0<br>AT3G66658.2 0.002 0.268 0.266 0.002 0.273
Obart07g27040.2	0	1<br>Obart07g27040.1 0.002 1 0.998 0.002 1<br>Obart07g27040.2 0.002 1 0.998 0.002 1	0	0	0	0	0	0	0	0
	1<br>Oglab07g23640.2 0.002 0.303 0.301 0.813 0.998<br>Oglab08g15450.1 0.381 0.973 0.592 0.067 0.483	1<br>Obart07g27030.1 0.002 0.226 0.224 0.348 0.51<br>Obart08g16640.1 0.381 0.973 0.592 0.035 0.802	1<br>Osjap07g30980.1 0.002 0.221 0.219 0.003 0.358<br>Osjap07g31000.1 0.298 0.973 0.675 0.002 0.608	1<br>Osind07g27700.1 0.002 0.226 0.224 0.002 0.258<br>Osind07g27720.1 0.298 0.973 0.675 0.012 0.493	1<br>Orufi03g37620.1 0.258 0.881 0.623 0.006 0.998<br>Orufi07g27980.2 0.002 0.226 0.224 0.019 0.253	1<br>Olong_008699-R1 0.294 0.973 0.679 0.003 0.523<br>Olong_008701-R1 0.002 0.226 0.224 0.002 0.258	1<br>Opunc07g25410.1 0.002 0.226 0.224 0.002 0.268<br>Opunc08g14550.1 0.381 0.973 0.592 0.08 0.527	1<br>Obrac07g18100.1 0.002 0.226 0.224 0.004 0.271<br>Obrac08g10910.1 0.381 0.973 0.592 0.075 0.529	0<br>Lperr07g23810.2 0.301 0.973 0.672 0.009 0.348	0<br>AT3G66658.2 0.002 0.246 0.244 0.002 0.273
Obart07g27300.1	0	1<br>Obart07g27300.1 0.001 1 0.999 0.001 1	0	0	0	0	0	0	0	0
	1<br>Oglab07g23910.1 0.002 0.524 0.522 0.008 0.948<br>Oglab07g23920.1 0.527 1 0.473 0.009 1	1<br>Obart03g13590.1 0.028 0.527 0.499 0.001 0.952<br>Obart05g12700.1 0.592 1 0.408 0.017 1	1<br>Osjap07g31290.1 0.001 0.524 0.523 0.033 0.951<br>Osjap07g31300.1 0.555 1 0.445 0.007 1	1<br>Osind03g14630.1 0.028 0.527 0.499 0.001 0.952<br>Osind07g28000.1 0.555 1 0.445 0.046 1	1<br>Orufi03g14250.1 0.028 0.527 0.499 0.001 0.952<br>Orufi07g28280.2 0.555 1 0.445 0.046 1	1<br>Olong_003210-R1 0.002 0.524 0.522 0.015 0.943<br>Olong_003211-R1 0.535 1 0.465 0.026 1	1<br>Opunc03g13100.1 0.028 0.527 0.499 0.001 0.948<br>Opunc07g25690.1 0.555 0.999 0.444 0.007 0.983	1<br>Obrac07g18370.1 0.013 0.524 0.511 0.064 0.951<br>Obrac07g18380.4 0.555 1 0.445 0.007 0.994	1<br>Lperr05g10220.2 0.555 1 0.445 0.014 1<br>Lperr07g24070.2 0.028 0.524 0.496 0.001 0.948	1<br>AT2G38280.1 0.556 0.999 0.443 0.01 0.998<br>AT3G08960.1 0.028 0.527 0.499 0.001 0.952
Obart08g02200.1	0	1<br>Obart08g02200.1 0.001 1 0.999 0.001 1	0	0	0	0	0	0	0	0
	1<br>Oglab03g29880.1 0.022 0.343 0.321 0.079 0.884<br>Oglab08g01680.7 0.344 0.99 0.646 0.109 1	0<br>Obart08g02200.5 0.345 1 0.655 0.01 1	1<br>Osjap03g33630.1 0.022 0.343 0.321 0.079 0.884<br>Osjap08g02400.4 0.344 0.99 0.646 0.106 1	1<br>Osind03g34740.1 0.022 0.343 0.321 0.079 0.884<br>Osind08g02290.1 0.372 0.99 0.618 0.002 1	1<br>Orufi03g32290.1 0.022 0.343 0.321 0.079 0.884<br>Orufi08g02450.3 0.344 0.99 0.646 0.1 1	1<br>Olong_001678-R3 0.368 0.99 0.622 0.039 1<br>Olong_002107-R1 0.028 0.346 0.318 0.11 0.973	1<br>Opunc08g02010.1 0.001 0.34 0.339 0.003 0.997<br>Opunc08g02020.4 0.368 0.99 0.622 0.005 1	1<br>Obrac08g01890.1 0.001 0.34 0.339 0.003 0.896<br>Obrac08g01910.2 0.345 0.99 0.645 0.1 1	1<br>Lperr08g02240.1 0.001 0.339 0.338 0.003 0.949<br>Lperr08g02260.1 0.36 0.99 0.63 0.003 1	1<br>AT3G07060.1 0.388 0.989 0.601 0.11 1<br>AT4G32390.1 0.022 0.354 0.332 0.109 0.98
Obart08g05380.1	0	1<br>Obart08g05380.1 0.004 1 0.996 0.004 1	0	0	0	0	0	0	0	0
	1<br>Oglab08g04820.1 0.331 1 0.669 0.203 1<br>Oglab08g04830.1 0.004 0.298 0.294 0.012 1	1<br>Obart07g07210.1 0.353 1 0.647 0.038 0.703<br>Obart08g05390.1 0.004 0.298 0.294 0.012 1	1<br>Osjap08g05840.2 0.353 1 0.647 0.065 0.75<br>Osjap08g05860.1 0.004 0.305 0.301 0.012 1	1<br>Osind07g07530.1 0.353 1 0.647 0.038 0.703<br>Osind08g05840.1 0.004 0.305 0.301 0.012 1	1<br>Orufi07g07230.1 0.353 1 0.647 0.037 0.706<br>Orufi08g05980.1 0.004 0.305 0.301 0.012 1	1<br>Olong_004294-R1 0.004 0.305 0.301 0.012 1<br>Olong_026698-R1 0.353 1 0.647 0.037 0.706	1<br>Opunc07g06940.1 0.356 1 0.644 0.004 0.695<br>Opunc08g05230.1 0.036 0.305 0.269 0.013 1	1<br>Obrac07g04400.3 0.345 1 0.655 0.015 0.7<br>Obrac08g04320.1 0.004 0.305 0.301 0.012 1	1<br>Lperr07g06270.1 0.356 1 0.644 0.004 0.68<br>Lperr08g05140.1 0.036 0.305 0.269 0.013 1	0<br>AT4G33690.1 0.356 0.924 0.568 0.004 0.691

Obart08g06070.1	1<br>Oglab08g05640.1 0.002 1 0.998 0.002 1	1<br>Obart08g06070.1 0.002 1 0.998 0.002 1	1<br>Osjap08g06650.1 0.002 1 0.998 0.002 1	1<br>Osind08g06790.1 0.002 1 0.998 0.002 1	1<br>Orufi08g06840.1 0.002 1 0.998 0.002 1	0	1<br>Opunc08g05850.1 0.047 1 0.953 0.068 1	0	0	0
	1<br>Oglab02g26360.1 0.348 0.996 0.648 0.01 0.994<br>Oglab04g16180.1 0.047 0.275 0.228 0.198 0.94	1<br>Obart02g28440.1 0.348 0.996 0.648 0.093 0.994<br>Obart04g17770.1 0.047 0.275 0.228 0.198 0.94	1<br>Osjap04g21220.1 0.047 0.275 0.228 0.21 0.936<br>Osjap09g20870.1 0.359 0.984 0.625 0.208 0.966	1<br>Osind04g20250.1 0.047 0.275 0.228 0.198 0.94<br>Osind09g20960.1 0.359 0.984 0.625 0.208 0.966	1<br>Orufi04g19250.2 0.047 0.275 0.228 0.21 0.936<br>Orufi09g21960.1 0.359 0.984 0.625 0.2 0.965	1<br>Olong_008030-R1 0.047 0.275 0.228 0.232 0.937<br>Olong_008031-R2 0.277 1 0.723 0.106 1	1<br>Opunc04g15550.1 0.047 0.275 0.228 0.206 0.938<br>Opunc04g18740.1 0.295 0.984 0.689 0.088 0.994	1<br>Obrac08g04780.1 0.277 1 0.723 0.098 1<br>Obrac08g04800.1 0.002 0.275 0.273 0.006 0.943	1<br>Lperr08g05690.1 0.277 1 0.723 0.1 1<br>Lperr08g05700.1 0.002 0.275 0.273 0.006 0.943	1<br>AT1G05310.1 0.33 0.982 0.652 0.183 0.99<br>AT4G30800.1 0.047 0.275 0.228 0.208 0.943
Obart08g11690.1	0	1<br>Obart08g11690.1 0.001 1 0.999 0.001 1	0	0	0	0	0	0	0	0
	1<br>Oglab08g10860.1 0.525 0.967 0.442 0.003 0.97<br>Oglab11g06870.1 0.008 0.511 0.503 0.007 0.988	0<br>Obart11g07530.1 0.001 0.567 0.566 0.476 0.957	1<br>Osjap03g01130.1 0.007 0.547 0.54 0.029 0.989<br>Osjap04g17040.5 0.561 0.975 0.414 0.224 0.844	1<br>Osind03g01180.1 0.007 0.547 0.54 0.029 0.989<br>Osind04g15750.1 0.561 0.975 0.414 0.254 0.907	1<br>Orufi03g00810.1 0.007 0.547 0.54 0.033 0.987<br>Orufi04g15370.3 0.561 0.975 0.414 0.243 0.917	1<br>Olong_005467-R1 0.01 0.556 0.546 0.049 0.974<br>Olong_007202-R1 0.56 1 0.44 0.342 1	1<br>Opunc06g03930.1 0.561 0.985 0.424 0.35 0.919<br>Opunc12g06580.1 0.001 0.517 0.516 0.036 0.951	1<br>Obrac07g02330.1 0.561 0.971 0.41 0.284 0.872<br>Obrac08g01810.1 0.008 0.517 0.509 0.44 0.989	0<br>Lperr11g13180.1 0.013 0.511 0.498 0.129 0.937	0
Obart08g11880.1	0	1<br>Obart08g11880.1 0.009 1 0.991 0.009 1<br>Obart10g13080.1 0.017 0.966 0.949 0.213 0.999	0	0	0	0	0	0	0	0
	1<br>Oglab02g23150.2 0.333 1 0.667 0.901 1<br>Oglab08g11080.1 0.009 0.333 0.324 0.523 0.701	1<br>Obart02g24490.1 0.333 1 0.667 0.82 1<br>Obart08g11890.1 0.009 0.333 0.324 0.523 0.701	1<br>Osjap02g27820.1 0.333 1 0.667 0.899 1<br>Osjap08g12720.1 0.009 0.333 0.324 0.01 0.379	1<br>Osind02g27730.1 0.333 1 0.667 0.899 1<br>Osind08g14210.1 0.009 0.333 0.324 0.536 0.709	1<br>Orufi02g25850.1 0.333 1 0.667 0.899 1<br>Orufi08g13360.1 0.009 0.333 0.324 0.349 0.792	1<br>Olong_025435-R1 0.342 0.966 0.624 0.075 0.988<br>Olong_027114-R1 0.009 0.333 0.324 0.007 0.277	1<br>Opunc08g10810.1 0.009 0.333 0.324 0.01 0.386<br>Opunc10g11490.1 0.333 0.966 0.633 0.867 0.998	1<br>Obrac02g17520.1 0.333 1 0.667 0.868 0.994<br>Obrac08g08040.1 0.009 0.316 0.307 0.012 0.435	1<br>Lperr02g20030.1 0.333 0.983 0.65 0.899 0.997<br>Lperr08g09830.2 0.009 0.333 0.324 0.011 0.437	0<br>AT3G23410.1 0.248 0.966 0.718 0.878 0.993
Obart08g16670.1	1<br>Oglab01g06790.1 0.007 0.844 0.837 0.061 1<br>Oglab08g15520.1 0.007 0.993 0.986 0.007 0.993	1<br>Obart01g07700.1 0.007 0.844 0.837 0.061 1<br>Obart08g16670.1 0.007 1 0.993 0.007 1	1<br>Osjap01g09050.1 0.007 0.844 0.837 0.061 1	1<br>Osind01g08880.1 0.102 0.986 0.884 0.004 0.567	1<br>Orufi01g08450.1 0.007 0.973 0.966 0.026 0.487	1<br>Olong_003647-R1 0.007 0.83 0.823 0.061 0.985	0	0	0	0
	1<br>Oglab07g08260.1 0.694 0.973 0.279 0.004 0.099<br>Oglab11g05440.1 0.007 0.694 0.687 0.573 0.683	1<br>Obart07g08560.1 0.694 0.952 0.258 0.009 0.192<br>Obart11g05910.1 0.007 0.694 0.687 0.752 0.834	1<br>Osjap01g24690.1 0.007 0.694 0.687 0.104 0.321<br>Osjap07g09900.1 0.694 0.993 0.299 0.005 0.123	1<br>Osind07g08950.1 0.694 0.993 0.299 0.004 0.111<br>Osind11g05650.1 0.007 0.694 0.687 0.103 0.319	1<br>Orufi01g23650.1 0.007 0.694 0.687 0.106 0.307<br>Orufi07g08630.1 0.694 0.993 0.299 0.005 0.115	1<br>Olong_005787-R1 0.007 0.694 0.687 0.103 0.319<br>Olong_016241-R1 0.728 0.932 0.204 0.735 0.768	1<br>Opunc01g21000.1 0.007 0.694 0.687 0.114 0.334<br>Opunc07g08090.1 0.694 0.993 0.299 0.004 0.09	1<br>Obrac07g05080.4 0.728 0.993 0.265 0.102 0.171<br>Obrac11g03720.1 0.007 0.694 0.687 0.112 0.34	1<br>Lperr01g17850.1 0.007 0.694 0.687 0.105 0.313<br>Lperr07g08590.1 0.694 0.993 0.299 0.022 0.124	1<br>AT3G55700.1 0.728 0.973 0.245 0.015 0.093<br>AT4G38130.1 0.007 0.68 0.673 0.09 0.269
Obart08g18520.1	0	1<br>Obart08g18520.1 0.001 1 0.999 0.001 1	0	0	0	0	0	0	0	0
	1<br>Oglab08g17140.1 0.313 1 0.687 0.004 1<br>Oglab08g17150.1 0.014 0.313 0.299 0.283 0.98	1<br>Obart08g18530.1 0.313 1 0.687 0.004 1<br>Obart08g18540.1 0.013 0.313 0.3 0.264 0.98	1<br>Osjap08g19640.1 0.313 1 0.687 0.004 1<br>Osjap08g19650.1 0.014 0.313 0.299 0.283 0.98	1<br>Osind08g21740.1 0.313 1 0.687 0.004 1<br>Osind10g18270.1 0.008 0.308 0.3 0.277 0.981	1<br>Orufi08g20720.1 0.313 1 0.687 0.004 1<br>Orufi08g20730.1 0.013 0.313 0.3 0.264 0.98	1<br>Olong_006289-R1 0.313 1 0.687 0.004 1<br>Olong_006290-R1 0.014 0.313 0.299 0.142 0.976	1<br>Opunc08g16450.1 0.313 1 0.687 0.004 1<br>Opunc08g16460.1 0.014 0.313 0.299 0.287 0.98	1<br>Obrac08g12190.5 0.313 1 0.687 0.004 1<br>Obrac08g12200.1 0.014 0.313 0.299 0.285 0.98	1<br>Lperr08g15390.1 0.313 0.999 0.686 0.004 0.998<br>Lperr08g15400.1 0.014 0.313 0.299 0.283 0.98	1<br>AT4G39980.1 0.014 0.31 0.296 0.335 1<br>AT4G40000.1 0.332 0.986 0.654 0.003 0.978
Obart08g20390.1	0	1<br>Obart08g20390.1 0.001 1 0.999 0.001 1	0	0	0	0	0	0	0	0
	1<br>Oglab08g19250.1 0.001 0.564 0.563 0.304 0.851<br>Oglab09g13510.1 0.566 0.988 0.422 0.501 0.994	0<br>Obart01g18290.1 0.588 0.987 0.399 0.113 0.99	1<br>Osjap08g21820.1 0.577 0.991 0.414 0.003 0.997<br>Osjap08g21830.1 0.001 0.564 0.563 0.303 0.851	1<br>Osind08g23920.1 0.577 0.992 0.415 0.003 0.997<br>Osind08g23930.1 0.001 0.564 0.563 0.303 0.851	1<br>Orufi08g22890.1 0.001 0.564 0.563 0.303 0.851<br>Orufi09g16160.1 0.566 0.988 0.422 0.501 0.994	0<br>Olong_014421-R1 0.553 0.995 0.442 0.015 0.99	1<br>Opunc08g18560.1 0.577 0.991 0.414 0.003 0.976<br>Opunc08g18570.1 0.001 0.564 0.563 0.307 0.852	1<br>Obrac08g13590.1 0.001 0.564 0.563 0.162 0.593<br>Obrac09g09090.1 0.57 0.988 0.418 0.501 0.993	1<br>Lperr01g20410.1 0.577 0.997 0.42 0.003 0.913<br>Lperr08g17250.1 0.001 0.564 0.563 0.303 0.849	1<br>AT5G19440.1 0.577 0.986 0.409 0.003 0.991<br>AT5G25070.1 0.008 0.57 0.562 0.255 0.852

Obart08g23110.1	0	1<br>Obart08g23110.1 0.001 1 0.999 0.001 1	0	0	1<br>Orufi08g25640.1 0.014 1 0.986 0.056 1<br>Orufi08g25640.2 0.014 1 0.986 0.056 1<br>Orufi08g25640.3 0.014 1 0.986 0.056 1	0	0	0	1<br>Lperr08g19910.1 0.014 0.964 0.95 0.065 0.996<br>Lperr08g19910.2 0.014 0.964 0.95 0.065 0.996<br>Lperr08g19910.3 0.014 0.964 0.95 0.065 0.996	0
	1<br>Oglab09g16750.1 0.056 0.444 0.388 0.48 0.983<br>Oglab11g01890.1 0.491 0.968 0.477 0.08 0.987	1<br>Obart08g23140.1 0.487 0.964 0.477 0.03 0.992<br>Obart09g18630.1 0.056 0.444 0.388 0.43 0.984	1<br>Osjap08g24670.1 0.487 0.964 0.477 0.03 0.992<br>Osjap09g19050.1 0.056 0.444 0.388 0.464 0.985	1<br>Osind08g26930.1 0.001 0.445 0.444 0.035 0.978<br>Osind08g26940.1 0.487 0.964 0.477 0.046 0.992	1<br>Orufi08g25640.4 0.487 0.964 0.477 0.047 0.992<br>Orufi09g20160.1 0.056 0.444 0.388 0.464 0.985	0<br>Olong_020799-R2 0.051 0.385 0.334 0.252 0.989<br>Olong_024387-R1 0.54 0.968 0.428 0.002 0.982	1<br>Opunc08g21230.4 0.487 0.964 0.477 0.081 0.992<br>Opunc09g17040.1 0.003 0.444 0.441 0.044 0.975	1<br>Obrac08g15620.1 0.488 0.964 0.476 0.051 0.992<br>Obrac09g12080.1 0.056 0.444 0.388 0.147 0.976	1<br>Lperr09g14540.3 0.056 0.444 0.388 0.164 0.976<br>Lperr12g01980.1 0.49 0.972 0.482 0.082 1	1<br>AT1G09910.1 0.472 0.964 0.492 0.041 0.994<br>AT1G76270.1 0.001 0.424 0.423 0.038 0.944
Obart09g14980.1	1<br>Oglab09g13510.1 0.002 1 0.998 0.002 1<br>Oglab09g13510.2 0.002 1 0.998 0.002 1<br>Oglab09g13510.3 0.002 1 0.998 0.002 1<br>Oglab09g13510.4 0.002 1 0.998 0.002 1	1<br>Obart09g14980.1 0.002 1 0.998 0.002 1<br>Obart09g14980.2 0.002 1 0.998 0.002 1	1<br>Osjap09g15430.2 0.002 1 0.998 0.002 1<br>Osjap09g15430.3 0.002 1 0.998 0.002 1	1<br>Osind09g15570.1 0.002 1 0.998 0.002 1<br>Osind09g15570.2 0.002 1 0.998 0.002 1	1<br>Orufi09g16160.1 0.002 1 0.998 0.002 1<br>Orufi09g16160.2 0.002 1 0.998 0.002 1	0	1<br>Opunc09g13420.1 0.002 1 0.998 0.002 1<br>Opunc09g13420.2 0.002 1 0.998 0.002 1	1<br>Obrac09g09090.1 0.002 1 0.998 0.002 1	0	0
	1<br>Oglab08g19240.1 0.569 0.993 0.424 0.143 0.986<br>Oglab12g18680.1 0.002 0.567 0.565 0.003 1	1<br>Obart02g06180.1 0.01 0.565 0.555 0.01 0.997<br>Obart08g20390.1 0.569 0.993 0.424 0.631 0.988	1<br>Osjap02g06650.1 0.01 0.565 0.555 0.01 0.997<br>Osjap09g15430.1 0.569 1 0.431 0.131 1	1<br>Osind02g06940.1 0.01 0.565 0.555 0.01 0.997<br>Osind08g23920.1 0.569 0.993 0.424 0.131 0.988	1<br>Orufi04g24220.1 0.014 0.567 0.553 0.165 1<br>Orufi08g22880.1 0.569 0.993 0.424 0.131 0.988	1<br>Olong_008093-R2 0.596 0.991 0.395 0.25 0.996<br>Olong_024186-R1 0.002 0.567 0.565 0.003 1	1<br>Opunc04g20190.1 0.007 0.567 0.56 0.006 1<br>Opunc08g18560.1 0.569 0.993 0.424 0.128 0.969	1<br>Obrac01g09090.1 0.605 0.998 0.393 0.299 0.982<br>Obrac02g04550.3 0.009 0.565 0.556 0.214 0.997	1<br>Lperr04g18350.1 0.002 0.567 0.565 0.021 1<br>Lperr09g11120.1 0.569 1 0.431 0.156 1	1<br>AT1G68540.1 0.567 0.991 0.424 0.112 1<br>AT2G21270.3 0.003 0.565 0.562 0.059 0.997
Obart09g14980.2	1<br>Oglab09g13510.1 0.002 1 0.998 0.002 1<br>Oglab09g13510.2 0.002 1 0.998 0.002 1<br>Oglab09g13510.3 0.002 1 0.998 0.002 1<br>Oglab09g13510.4 0.002 1 0.998 0.002 1	1<br>Obart09g14980.1 0.002 1 0.998 0.002 1<br>Obart09g14980.2 0.002 1 0.998 0.002 1	1<br>Osjap09g15430.2 0.002 1 0.998 0.002 1<br>Osjap09g15430.3 0.002 1 0.998 0.002 1	1<br>Osind09g15570.1 0.002 1 0.998 0.002 1<br>Osind09g15570.2 0.002 1 0.998 0.002 1	1<br>Orufi09g16160.1 0.002 1 0.998 0.002 1<br>Orufi09g16160.2 0.002 1 0.998 0.002 1	0	1<br>Opunc09g13420.1 0.002 1 0.998 0.002 1<br>Opunc09g13420.2 0.002 1 0.998 0.002 1	1<br>Obrac09g09090.1 0.002 1 0.998 0.002 1	0	0
	1<br>Oglab08g19240.1 0.544 0.993 0.449 0.143 0.986<br>Oglab12g18680.1 0.002 0.542 0.54 0.003 1	1<br>Obart02g06180.1 0.01 0.54 0.53 0.01 0.997<br>Obart08g20390.1 0.544 0.993 0.449 0.631 0.988	1<br>Osjap02g06650.1 0.01 0.54 0.53 0.01 0.997<br>Osjap09g15430.1 0.544 1 0.456 0.131 1	1<br>Osind02g06940.1 0.01 0.54 0.53 0.01 0.997<br>Osind08g23920.1 0.544 0.993 0.449 0.131 0.988	1<br>Orufi04g24220.1 0.013 0.542 0.529 0.165 1<br>Orufi08g22880.1 0.544 0.993 0.449 0.131 0.988	1<br>Olong_013054-R1 0.547 0.992 0.445 0.149 0.979<br>Olong_024186-R1 0.002 0.542 0.54 0.003 1	1<br>Opunc04g20190.1 0.007 0.542 0.535 0.006 1<br>Opunc08g18560.1 0.544 0.993 0.449 0.128 0.969	1<br>Obrac02g04550.3 0.008 0.54 0.532 0.214 0.997<br>Obrac09g00560.1 0.548 0.998 0.45 0.164 0.966	1<br>Lperr04g18350.1 0.002 0.542 0.54 0.021 1<br>Lperr09g11120.1 0.544 1 0.456 0.156 1	1<br>AT1G68540.1 0.542 0.992 0.45 0.112 1<br>AT2G21270.3 0.003 0.54 0.537 0.059 0.997
Obart09g15000.1	0	1<br>Obart09g15000.1 0.001 1 0.999 0.001 1	0	0	0	0	0	0	0	0
	1<br>Oglab09g13530.1 0.362 1 0.638 0.006 1<br>Oglab09g13540.1 0.001 0.351 0.35 0.003 1	0<br>Obart08g20950.1 0.001 0.314 0.313 0.003 0.87	1<br>Osjap09g15450.1 0.362 0.931 0.569 0.005 0.707<br>Osjap09g15460.1 0.001 0.351 0.35 0.003 1	1<br>Osind09g15590.1 0.362 1 0.638 0.006 1<br>Osind09g15600.1 0.001 0.351 0.35 0.003 1	1<br>Orufi08g23430.1 0.001 0.314 0.313 0.003 0.87<br>Orufi09g16180.1 0.329 1 0.671 0.007 1	1<br>Olong_025646-R1 0.484 0.996 0.512 0.002 0.977<br>Olong_025647-R1 0.001 0.353 0.352 0.003 0.972	1<br>Opunc09g13450.1 0.362 1 0.638 0.006 1<br>Opunc09g13460.1 0.001 0.353 0.352 0.003 0.973	1<br>Obrac09g09110.1 0.362 0.995 0.633 0.003 1<br>Obrac09g09120.2 0.001 0.351 0.35 0.003 1	1<br>Lperr08g17690.1 0.001 0.333 0.332 0.253 0.945<br>Lperr09g11150.1 0.362 0.995 0.633 0.006 1	1<br>AT1G34180.2 0.369 0.984 0.615 0.031 0.977<br>AT1G76700.1 0.001 0.326 0.325 0.003 0.917
Obart09g15360.1	1<br>Oglab09g13920.1 0.001 1 0.999 0.114 1	1<br>Obart09g15360.1 0.001 1 0.999 0.001 1	0	1<br>Osind09g15930.1 0.001 1 0.999 0.057 1	1<br>Orufi09g16580.1 0.001 1 0.999 0.114 1	0	0	0	0	0
	1<br>Oglab02g15910.1 0.003 0.474 0.471 0.048 0.988<br>Oglab09g02290.1 0.507 0.883 0.376 0.02 0.789	1<br>Obart03g04820.3 0.535 0.883 0.348 0.001 0.781<br>Obart08g21020.1 0.001 0.507 0.506 0.088 0.994	1<br>Osjap03g04840.3 0.507 0.883 0.376 0.018 0.769<br>Osjap09g15790.1 0.001 0.507 0.506 0.238 0.993	1<br>Osind08g24670.1 0.001 0.507 0.506 0.221 0.995<br>Osind09g02290.1 0.507 0.883 0.376 0.018 0.769	1<br>Orufi03g04550.5 0.507 0.883 0.376 0.017 0.733<br>Orufi08g23480.1 0.001 0.507 0.506 0.228 0.995	1<br>Olong_025005-R2 0.535 0.979 0.444 0.001 0.955<br>Olong_025006-R1 0.05 0.507 0.457 0.031 0.99	1<br>Opunc08g19030.2 0.001 0.507 0.506 0.156 0.976<br>Opunc09g13790.1 0.507 0.979 0.472 0.055 0.958	0<br>Obrac08g14010.2 0.001 0.508 0.507 0.044 0.989	1<br>Lperr08g17810.1 0.001 0.507 0.506 0.122 0.994<br>Lperr09g11510.2 0.507 0.979 0.472 0.01 0.958	1<br>AT2G32000.1 0.507 0.978 0.471 0.012 0.961<br>AT2G43900.1 0.003 0.481 0.478 0.165 0.843
Obart09g15430.1	0	1	0	1	0	0	0	0	0	0

		Obart09g15430.1 0.003 1 0.997 0.003 1		Osind09g16000.1 0.003 0.943 0.94 0.002 1						
	1<br>Oglab09g13980.1 0.003 0.443 0.44 0.008 1<br>Oglab09g13990.1 0.49 1 0.51 0.006 1	1<br>Obart01g41710.1 0.547 0.953 0.406 0.165 0.937<br>Obart09g15390.1 0.003 0.443 0.44 0.008 1	1<br>Osjap08g22450.1 0.003 0.443 0.44 0.254 1<br>Osjap09g15860.1 0.49 1 0.51 0.006 1	1<br>Osind01g48630.1 0.547 0.953 0.406 0.165 0.937<br>Osind08g24700.1 0.003 0.443 0.44 0.008 1	1<br>Orufi09g16660.1 0.49 1 0.51 0.006 1<br>Orufi09g16670.1 0.003 0.477 0.474 0.007 1	1<br>Olong_001207-R1 0.547 0.953 0.406 0.165 0.937<br>Olong_017992-R1 0.003 0.443 0.44 0.008 1	1<br>Opunc09g13840.1 0.003 0.443 0.44 0.008 1<br>Opunc09g13850.1 0.49 0.967 0.477 0.007 0.934	1<br>Obrac09g09430.1 0.003 0.443 0.44 0.008 1<br>Obrac09g09440.1 0.49 1 0.51 0.007 1	1<br>Lperr03g16320.1 0.003 0.443 0.44 0.007 1<br>Lperr09g11590.1 0.49 1 0.51 0.007 1	1<br>AT4G18100.1 0.003 0.443 0.44 0.008 1<br>AT5G45680.1 0.467 0.94 0.473 0.284 0.99
Obart09g16160.1	0	1<br>Obart09g16160.1 0.001 1 0.999 0.001 1	0	0	0	0	0	0	0	0
	1<br>Oglab09g14660.1 0.001 0.441 0.44 0.003 0.979<br>Oglab09g14670.1 0.485 1 0.515 0.2 1	1<br>Obart04g25680.1 0.485 0.974 0.489 0.159 0.786<br>Obart08g22080.1 0.004 0.441 0.437 0.04 0.979	1<br>Osjap09g16620.1 0.001 0.441 0.44 0.003 0.979<br>Osjap09g16630.1 0.485 1 0.515 0.2 1	1<br>Osind10g01650.1 0.001 0.441 0.44 0.003 0.979<br>Osind10g01660.1 0.485 1 0.515 0.245 1	1<br>Orufi09g17470.1 0.001 0.441 0.44 0.003 0.979<br>Orufi09g17480.1 0.485 1 0.515 0.2 1	1<br>Olong_000869-R1 0.485 1 0.515 0.072 1<br>Olong_000870-R1 0.001 0.441 0.44 0.003 0.979	1<br>Opunc09g14580.1 0.001 0.441 0.44 0.003 0.979<br>Opunc09g14590.1 0.485 1 0.515 0.205 1	1<br>Obrac09g10080.1 0.001 0.441 0.44 0.262 0.984<br>Obrac09g10090.2 0.485 0.999 0.514 0.083 1	1<br>Lperr09g12290.1 0.001 0.441 0.44 0.003 0.979<br>Lperr09g12300.1 0.485 0.999 0.514 0.268 0.998	1<br>AT2G17390.1 0.003 0.441 0.438 0.061 0.98<br>AT3G03310.1 0.485 0.995 0.51 0.157 0.996
Obart09g16460.1	0	1<br>Obart09g16460.1 0.001 1 0.999 0.001 1	0	0	0	0	0	0	0	0
	1<br>Oglab01g17730.1 0.727 0.997 0.27 0.15 0.536<br>Oglab09g14960.1 0.001 0.721 0.72 0.002 0.929	1<br>Obart01g21490.2 0.727 1 0.273 0.17 0.551<br>Obart03g20890.1 0.025 0.659 0.634 0.028 0.915	1<br>Osjap02g19520.2 0.731 1 0.269 0.056 0.184<br>Osjap09g16920.1 0.001 0.721 0.72 0.002 0.914	1<br>Osind01g26310.1 0.727 0.997 0.27 0.344 0.652<br>Osind09g17080.1 0.001 0.721 0.72 0.002 0.918	1<br>Orufi01g24210.2 0.727 0.997 0.27 0.04 0.462<br>Orufi09g17770.1 0.001 0.721 0.72 0.002 0.918	0<br>Olong_025574-R2 0.098 0.659 0.561 0.059 0.907	1<br>Opunc01g21720.2 0.727 1 0.273 0.051 0.595<br>Opunc09g14890.1 0.001 0.721 0.72 0.002 0.917	0<br>Obrac09g10330.1 0.025 0.721 0.696 0.032 0.901	1<br>Lperr02g05180.1 0.731 1 0.269 0.121 0.552<br>Lperr09g12570.1 0.023 0.721 0.698 0.013 0.312	0<br>AT2G26260.1 0.025 0.73 0.705 0.021 0.92
Obart09g18850.1	0	1<br>Obart09g18850.1 0.001 1 0.999 0.001 1	0	0	0	0	0	0	0	0
	1<br>Oglab02g28320.1 0.001 0.712 0.711 0.001 0.999<br>Oglab09g16980.1 0.712 0.998 0.286 0.011 0.742	1<br>Obart02g30770.1 0.056 0.712 0.656 0.002 0.998<br>Obart09g19080.1 0.713 0.996 0.283 0.01 0.943	1<br>Osjap02g34900.1 0.001 0.712 0.711 0.001 0.999<br>Osjap09g18210.1 0.713 0.995 0.282 0.014 0.875	1<br>Osind09g18400.1 0.713 0.995 0.282 0.014 0.873<br>Osind09g19410.2 0.001 0.713 0.712 0.001 1	1<br>Orufi09g19250.1 0.713 0.995 0.282 0.014 0.873<br>Orufi09g20400.1 0.001 0.713 0.712 0.118 1	1<br>Olong_009633-R1 0.011 0.702 0.691 0.036 1<br>Olong_028691-R1 0.719 0.995 0.276 0.004 0.861	0<br>Opunc09g17250.1 0.001 0.712 0.711 0.001 0.999	0<br>Obrac09g12310.2 0.002 0.712 0.71 0.004 0.999	1<br>Lperr05g22000.1 0.713 0.988 0.275 0.036 1<br>Lperr09g14830.1 0.001 0.711 0.71 0.001 0.962	0<br>AT4G26600.1 0.115 0.601 0.486 0.165 0.902
Obart10g02510.1	1<br>Oglab10g02440.1 0.001 1 0.999 0.001 1<br>Oglab10g02440.2 0.001 1 0.999 0.001 1	1<br>Obart10g02510.1 0.001 1 0.999 0.001 1<br>Obart10g02510.2 0.001 1 0.999 0.001 1	0	1<br>Osind10g02110.1 0.001 0.995 0.994 0.001 0.952	0	0	0	0	0	0
	0<br>Oglab10g02440.3 0.455 1 0.545 0.005 1	1<br>Obart10g00500.1 0.001 0.391 0.39 0.001 0.993<br>Obart11g08380.1 0.528 1 0.472 0.194 0.922	1<br>Osjap10g02940.1 0.021 0.393 0.372 0.027 0.984<br>Osjap10g02950.1 0.472 0.972 0.5 0.002 0.928	1<br>Osind10g00350.1 0.001 0.39 0.389 0.001 0.98<br>Osind11g07740.1 0.472 0.995 0.523 0.003 1	1<br>Orufi10g02520.1 0.021 0.222 0.201 0.065 0.758<br>Orufi10g02530.1 0.223 0.995 0.772 0.284 1	1<br>Olong_016648-R1 0.223 0.995 0.772 0.265 1<br>Olong_020863-R1 0.005 0.222 0.217 0.007 0.797	1<br>Opunc10g02120.1 0.001 0.393 0.392 0.001 0.992<br>Opunc11g08150.1 0.472 1 0.528 0.003 0.911	1<br>Obrac03g10950.1 0.001 0.39 0.389 0.001 0.983<br>Obrac11g05340.1 0.472 1 0.528 0.002 0.922	1<br>Lperr10g00410.1 0.001 0.391 0.39 0.003 0.986<br>Lperr10g01950.1 0.472 1 0.528 0.003 0.895	0<br>AT1G21230.1 0.005 0.353 0.348 0.01 0.93<br>AT4G00830.1 0.523 0.972 0.449 0.194 0.891
Obart10g02510.2	1<br>Oglab10g02440.1 0.001 1 0.999 0.001 1<br>Oglab10g02440.2 0.001 1 0.999 0.001 1	1<br>Obart10g02510.1 0.001 1 0.999 0.001 1<br>Obart10g02510.2 0.001 1 0.999 0.001 1	0	1<br>Osind10g02110.1 0.001 0.995 0.994 0.001 0.952	0	0	0	0	0	0
	0<br>Oglab10g02440.3 0.476 1 0.524 0.005 1	1<br>Obart10g00500.1 0.001 0.409 0.408 0.001 0.993<br>Obart11g08380.1 0.553 1 0.447 0.194 0.922	1<br>Osjap10g02940.1 0.022 0.411 0.389 0.027 0.984<br>Osjap10g02950.1 0.495 0.971 0.476 0.002 0.928	1<br>Osind10g00350.1 0.001 0.408 0.407 0.001 0.98<br>Osind11g07740.1 0.495 0.995 0.5 0.003 0.96	1<br>Orufi10g02520.1 0.022 0.232 0.21 0.065 0.758<br>Orufi10g02530.1 0.233 0.995 0.762 0.284 0.988	1<br>Olong_016648-R1 0.233 0.995 0.762 0.265 1<br>Olong_028710-R1 0.031 0.232 0.201 0.004 0.754	1<br>Opunc10g02120.1 0.001 0.411 0.41 0.001 0.992<br>Opunc11g08150.1 0.495 1 0.505 0.003 0.911	1<br>Obrac03g10950.1 0.001 0.408 0.407 0.001 0.983<br>Obrac11g05340.1 0.495 1 0.505 0.002 0.922	1<br>Lperr10g00410.1 0.001 0.409 0.408 0.003 0.986<br>Lperr10g01950.1 0.495 1 0.505 0.003 0.895	0<br>AT1G21230.1 0.005 0.37 0.365 0.01 0.93<br>AT4G00830.1 0.548 0.971 0.423 0.194 0.891
Obart10g09110.2	1<br>Oglab10g08680.1 0.009 1 0.991 0.008 1	1<br>Obart10g09110.1 0.009 1 0.991 0.008 1	0	0	1<br>Orufi10g09480.1 0.009 1 0.991 0.008 1	1<br>Olong_027249-R1 0.03 1 0.97 0.001 1	0	0	0	0

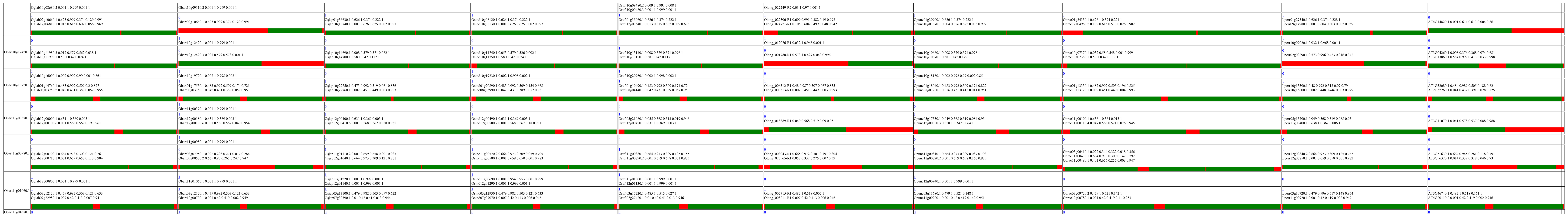
Obart10g12420.1
Obart10g19720.1
Obart11g00370.1
Obart11g00980.1
Obart11g01060.1
Obart11g04380.1

		Obart11g04380.1 0.001 1 0.999 0.001 1								
	0<br>Oglab11g04130.1 0.001 0.601 0.6 0.002 0.78	1<br>Obart03g11190.1 0.061 0.586 0.525 0.048 0.746<br>Obart03g32320.1 0.609 0.926 0.317 0.383 0.899	1<br>Osjap03g34930.1 0.609 0.926 0.317 0.383 0.899<br>Osjap11g04880.1 0.001 0.601 0.6 0.002 0.78	1<br>Osind03g34710.1 0.609 0.926 0.317 0.383 0.899<br>Osind11g04140.1 0.001 0.601 0.6 0.002 0.78	1<br>Orufi03g33710.1 0.609 0.926 0.317 0.383 0.899<br>Orufi11g04260.1 0.001 0.601 0.6 0.002 0.738	1<br>Olong_004085-R1 0.061 0.586 0.525 0.048 0.744<br>Olong_009498-R1 0.609 0.926 0.317 0.13 0.858	1<br>Opunc03g29490.1 0.609 0.926 0.317 0.377 0.904<br>Opunc11g04020.1 0.001 0.601 0.6 0.002 0.777	1<br>Obrac03g08970.1 0.071 0.617 0.546 0.061 0.804<br>Obrac11g02780.1 0.724 1 0.276 0.005 0.985	1<br>Lperr03g26910.1 0.609 0.926 0.317 0.377 0.904<br>Lperr11g03800.1 0.001 0.601 0.6 0.002 0.77	1<br>AT3G57620.1 0.054 0.587 0.533 0.044 0.762<br>AT5G64090.1 0.587 1 0.413 0.395 1
Obart11g16070.1	1<br>Oglab11g14350.1 0.013 0.975 0.962 0.001 1<br>Oglab11g14350.2 0.013 1 0.987 0.001 1	1<br>Obart11g16070.1 0.001 1 0.999 0.001 1	0	0	0	0	0	0	0	0
	1<br>Oglab02g07220.2 0.685 1 0.315 0.003 1<br>Oglab11g18500.1 0.036 0.633 0.597 0.003 0.966	1<br>Obart02g07160.1 0.685 1 0.315 0.002 1<br>Obart08g10830.1 0.037 0.654 0.617 0.004 0.995	1<br>Osjap02g07570.1 0.685 1 0.315 0.002 1<br>Osjap11g18290.1 0.001 0.632 0.631 0.006 0.989	1<br>Osind11g13470.1 0.001 0.605 0.604 0.005 0.997<br>Osind11g13490.1 0.684 1 0.316 0.081 1	1<br>Orufi11g17130.1 0.001 0.632 0.631 0.006 0.989<br>Orufi11g17160.1 0.685 1 0.315 0.002 1	1<br>Olong_012209-R1 0.685 1 0.315 0.002 1<br>Olong_012210-R1 0.001 0.632 0.631 0.01 0.981	1<br>Opunc11g13170.1 0.685 1 0.315 0.002 1<br>Opunc11g13180.1 0.033 0.634 0.601 0.145 0.975	1<br>Obrac02g05260.1 0.685 1 0.315 0.002 1<br>Obrac10g05860.1 0.038 0.627 0.589 0.005 0.946	1<br>Lperr11g12540.1 0.035 0.63 0.595 0.002 0.969<br>Lperr12g06590.1 0.674 1 0.326 0.078 1	0<br>AT2G44350.2 0.685 1 0.315 0.002 0.998
Obart11g22080.1	1<br>Oglab11g20100.1 0.024 0.953 0.929 0.012 0.992	1<br>Obart11g22080.1 0.001 1 0.999 0.001 1<br>Obart11g23070.1 0.024 0.973 0.949 0.038 0.936	0	0	0	1<br>Olong_001283-R1 0.004 0.94 0.936 0.012 0.965	1<br>Opunc11g18660.1 0.001 0.987 0.986 0.001 0.924<br>Opunc11g18660.2 0.001 0.987 0.986 0.001 0.925<br>Opunc11g18660.3 0.001 0.987 0.986 0.001 0.89<br>Opunc11g19450.1 0.024 0.973 0.949 0.016 0.933	0	1<br>Lperr11g13500.1 0.008 0.944 0.936 0.003 0.998	0
	1<br>Oglab11g11420.1 0.008 0.461 0.453 0.002 0.759<br>Oglab11g19250.1 0.498 0.987 0.489 0.002 0.999	1<br>Obart11g12900.1 0.008 0.474 0.466 0.002 0.766<br>Obart11g22110.1 0.506 0.988 0.482 0.003 0.891	1<br>Osjap10g09150.6 0.007 0.461 0.454 0.005 0.72<br>Osjap11g25990.5 0.5 0.987 0.487 0.086 0.998	0<br>Osind05g25410.1 0.007 0.534 0.527 0.001 0.979	1<br>Orufi11g13640.1 0.008 0.474 0.466 0.002 0.766<br>Orufi11g24060.2 0.49 0.993 0.503 0.001 0.999	1<br>Olong_000091-R1 0.502 0.986 0.484 0.001 0.997<br>Olong_000093-R1 0.001 0.494 0.493 0.001 1	1<br>Opunc05g18050.1 0.024 0.492 0.468 0.031 0.839<br>Opunc11g18620.1 0.505 0.987 0.482 0.004 0.998	1<br>Obrac11g13000.2 0.506 0.989 0.483 0.006 0.999<br>Obrac11g13510.3 0.024 0.478 0.454 0.023 0.869	1<br>Lperr11g18720.1 0.505 0.995 0.49 0.002 0.997<br>Lperr11g19280.1 0.006 0.455 0.449 0.016 0.849	0<br>AT5G02800.1 0.585 0.998 0.413 0.153 0.989
Obart11g22180.1	0	1<br>Obart11g22180.1 0.001 1 0.999 0.001 1	0	1<br>Osind06g17590.1 0.038 0.984 0.946 0.037 0.999<br>Osind08g03120.1 0.028 0.98 0.952 0.059 0.995	0	1<br>Olong_000078-R1 0.029 0.872 0.843 0.013 0.995	0	0	0	0
	1<br>Oglab11g19350.1 0.001 0.374 0.373 0.002 0.896<br>Oglab11g19360.1 0.374 1 0.626 0.091 1	1<br>Obart11g22190.1 0.001 0.37 0.369 0.029 0.928<br>Obart11g22210.1 0.373 0.971 0.598 0.002 0.971	1<br>Osjap11g26070.1 0.374 1 0.626 0.095 1<br>Osjap11g26080.1 0.001 0.374 0.373 0.002 0.909	1<br>Osind01g01210.1 0.374 1 0.626 0.091 1<br>Osind01g30060.1 0.001 0.374 0.373 0.002 0.909	1<br>Orufi09g00060.1 0.374 1 0.626 0.11 1<br>Orufi09g00070.1 0.001 0.374 0.373 0.022 0.913	1<br>Olong_022538-R1 0.001 0.374 0.373 0.002 0.903<br>Olong_022539-R1 0.391 1 0.609 0.1 1	1<br>Opunc11g18540.2 0.009 0.369 0.36 0.022 0.956<br>Opunc11g18800.1 0.369 0.971 0.602 0.09 0.976	1<br>Obrac11g13060.1 0.004 0.374 0.37 0.251 0.937<br>Obrac11g13070.1 0.374 0.968 0.594 0.016 0.953	0<br>Lperr11g18890.1 0.374 0.977 0.603 0.119 0.991	0<br>AT5G57580.1 0.498 0.936 0.438 0.134 0.737
Obart12g01910.1	1<br>Oglab12g01890.1 0.002 0.938 0.936 0.002 0.974	1<br>Obart12g01910.1 0.002 1 0.998 0.002 1	0	0	0	0	1<br>Opunc11g02000.1 0.002 0.938 0.936 0.002 0.883<br>Opunc12g01920.1 0.002 0.938 0.936 0.002 0.951	1<br>Obrac11g01380.1 0.022 0.945 0.923 0.036 0.949	0	0
	1<br>Oglab11g01920.1 0.712 1 0.288 0.005 1<br>Oglab11g01930.1 0.013 0.691 0.678 0.051 0.947	1<br>Obart01g34520.1 0.71 1 0.29 0.005 1<br>Obart11g02130.1 0.013 0.691 0.678 0.051 0.947	1<br>Osjap11g02310.1 0.712 1 0.288 0.005 1<br>Osjap12g02360.1 0.002 0.691 0.689 0.002 0.945	1<br>Osind11g01670.1 0.712 0.997 0.285 0.004 0.742<br>Osind12g02620.1 0.002 0.691 0.689 0.002 0.945	1<br>Orufi01g37930.2 0.71 1 0.29 0.005 1<br>Orufi12g02190.1 0.022 0.6 0.578 0.159 1	1<br>Olong_019069-R2 0.002 0.691 0.689 0.002 0.945<br>Olong_020510-R1 0.71 1 0.29 0.005 1	0<br>Opunc03g30920.1 0.688 0.949 0.261 0.471 0.991	1<br>Obrac12g01690.1 0.712 1 0.288 0.005 1<br>Obrac12g01700.2 0.002 0.691 0.689 0.002 0.948	1<br>Lperr07g20930.1 0.025 0.691 0.666 0.09 0.956<br>Lperr12g02000.1 0.709 1 0.291 0.866 1	1<br>AT2G19540.1 0.022 0.691 0.669 0.062 0.947<br>AT2G27030.3 0.71 1 0.29 0.006 1
Obart12g02080.1	1<br>Oglab11g02200.1 0.002 1 0.998 0.023 1<br>Oglab12g02130.1 0.002 1 0.998 0.022 1	1<br>Obart12g02080.1 0.002 1 0.998 0.002 1	1<br>Osjap11g02640.1 0.002 1 0.998 0.024 1<br>Osjap12g02610.1 0.002 1 0.998 0.022 1	1<br>Osind11g01990.1 0.002 1 0.998 0.023 1<br>Osind12g02780.1 0.002 1 0.998 0.022 1	1<br>Orufi11g02320.1 0.002 1 0.998 0.003 1<br>Orufi12g02480.1 0.002 1 0.998 0.021 1	0	0	0	0	0
	1<br>Oglab12g02120.1 0.002 0.64 0.638 0.006 1	0<br>Obart11g02350.1 0.002 0.64 0.638 0.004 0.993	1<br>Osjap12g02600.1 0.002 0.64 0.638 0.006 1	1<br>Osind11g01980.1 0.002 0.64 0.638 0.006 1	1<br>Orufi12g02470.1 0.002 0.64 0.638 0.006 1	1<br>Olong_003435-R1 0.133 0.64 0.507 0.212 0.984	1<br>Opunc11g02180.1 0.002 0.64 0.638 0.028 0.992	0<br>Obrac05g10860.1 0.072 0.523 0.451 0.097 0.99	1<br>Lperr12g02190.1 0.002 0.64 0.638 0.033 0.992	0<br>AT3G27320.1 0.052 0.421 0.369 0.596 0.978

	Oglab12g09880.1 0.644 0.948 0.304 0.051 0.385		Osjap12g12870.1 0.644 0.948 0.304 0.055 0.417	Osind12g10020.1 0.644 0.948 0.304 0.055 0.417	Orufi12g10750.1 0.642 0.948 0.306 0.045 0.364	Olong_023179-R1 0.642 0.998 0.356 0.05 0.329	Opunc12g08740.1 0.642 0.948 0.306 0.04 0.324	Obrac12g07170.1 0.642 0.974 0.332 0.071 0.67	Lperr12g08390.1 0.642 0.956 0.314 0.056 0.454	
Obart12g02370.1	0	1<br>Obart12g02370.1 0.001 1 0.999 0.001 1	0	0	0	0	0	0	0	0
	1<br>Oglab12g02420.1 0.492 1 0.508 0.283 1<br>Oglab12g02460.1 0.001 0.434 0.433 0.002 0.822	1<br>Obart11g02670.1 0.492 1 0.508 0.277 1<br>Obart11g02690.1 0.001 0.434 0.433 0.003 0.982	1<br>Osjap11g03000.1 0.001 0.434 0.433 0.002 0.986<br>Osjap12g02930.1 0.492 1 0.508 0.286 1	1<br>Osind12g02950.1 0.492 1 0.508 0.286 1<br>Osind12g02970.1 0.001 0.434 0.433 0.002 0.935	1<br>Orufi11g02630.1 0.492 1 0.508 0.063 1<br>Orufi12g02750.1 0.075 0.434 0.359 0.003 0.907	0<br>Olong_011875-R1 0.103 0.411 0.308 0.161 0.908<br>Olong_028720-R1 0.557 1 0.443 0.003 1	1<br>Opunc11g02440.1 0.492 1 0.508 0.284 1<br>Opunc11g02450.1 0.001 0.434 0.433 0.002 0.932	0<br>Obrac11g01640.1 0.51 1 0.49 0.034 0.766	0<br>Lperr12g02500.1 0.147 0.432 0.285 0.144 0.868	0<br>AT3G47930.1 0.492 0.993 0.501 0.311 0.995
Obart12g02600.1	1<br>Oglab12g02710.1 0.003 1 0.997 0.004 1	1<br>Obart12g02600.1 0.003 1 0.997 0.003 1	0	0	1<br>Orufi12g03000.1 0.003 0.978 0.975 0.004 0.823	0	0	0	0	0
	1<br>Oglab11g14940.1 0.653 0.959 0.306 0.011 1<br>Oglab12g02550.1 0.009 0.581 0.572 0.02 0.975	1<br>Obart01g16130.1 0.588 1 0.412 0.209 1<br>Obart12g02460.1 0.009 0.581 0.572 0.02 0.975	1<br>Osjap01g18900.1 0.588 1 0.412 0.308 1<br>Osjap12g03070.1 0.009 0.581 0.572 0.02 0.975	1<br>Osind12g03230.1 0.606 1 0.394 0.292 1<br>Osind12g03240.1 0.003 0.588 0.585 0.008 1	1<br>Orufi02g31540.1 0.744 0.988 0.244 0.399 0.957<br>Orufi12g02840.1 0.009 0.581 0.572 0.02 0.975	1<br>Olong_021782-R1 0.713 0.975 0.262 0.051 0.859<br>Olong_027258-R1 0.009 0.581 0.572 0.28 0.981	0<br>Opunc02g10450.1 0.694 1 0.306 0.24 1<br>Opunc10g12380.1 0.297 0.641 0.344 0.499 0.843	0<br>Obrac11g01600.1 0.231 0.578 0.347 0.076 0.91	0<br>Lperr02g24330.1 0.694 0.919 0.225 0.287 0.816<br>Lperr02g27700.1 0.297 0.603 0.306 0.415 0.71	0<br>AT1G68180.1 0.272 0.584 0.312 0.31 0.746
Obart12g02950.1	1<br>Oglab12g03070.1 0.001 1 0.999 0.001 1	1<br>Obart12g02950.1 0.001 1 0.999 0.001 1	0	1<br>Osind12g03620.1 0.001 0.999 0.998 0.029 0.961	1<br>Orufi12g03350.1 0.011 0.999 0.988 0.044 0.966<br>Orufi12g03350.2 0.011 0.999 0.988 0.043 0.966	1<br>Olong_000549-R1 0.011 0.999 0.988 0.011 0.967	0	0	0	0
	1<br>Oglab10g13340.3 0.559 0.991 0.432 0.213 0.951<br>Oglab12g02020.1 0.002 0.453 0.451 0.001 0.388	0<br>Obart11g02240.1 0.193 0.453 0.26 0.014 0.272<br>Obart11g21270.1 0.494 0.985 0.491 0.138 0.958	1<br>Osjap10g20010.1 0.498 0.991 0.493 0.022 0.946<br>Osjap12g02500.1 0.002 0.453 0.451 0.001 0.388	1<br>Osind01g01580.1 0.492 0.985 0.493 0.171 0.96<br>Osind12g02670.1 0.002 0.453 0.451 0.001 0.396	1<br>Orufi04g25720.1 0.498 0.871 0.373 0.002 0.207<br>Orufi11g02210.1 0.015 0.453 0.438 0.015 0.492	0<br>Olong_010277-R4 0.488 0.871 0.383 0.001 0.93<br>Olong_027241-R1 0.046 0.305 0.259 0.003 0.982	1<br>Opunc11g17890.2 0.491 0.985 0.494 0.189 0.952<br>Opunc12g02040.1 0.012 0.453 0.441 0.009 0.402	1<br>Obrac11g01490.1 0.002 0.453 0.451 0.029 0.403<br>Obrac11g12600.2 0.547 0.985 0.438 0.046 0.748	0<br>Lperr11g01950.1 0.011 0.228 0.217 0.015 0.297<br>Lperr11g17840.1 0.5 0.985 0.485 0.046 0.945	1<br>AT1G65810.1 0.498 0.871 0.373 0.021 0.596<br>AT2G26780.1 0.017 0.449 0.432 0.011 0.388
Obart12g03260.1	0	1<br>Obart12g03260.1 0.002 1 0.998 0.002 1	0	0	0	0	0	0	0	0
	1<br>Oglab03g28380.1 0.002 0.382 0.38 0.835 0.983<br>Oglab12g03430.1 0.382 0.835 0.453 0.004 0.758	0<br>Obart03g29120.1 0.002 0.382 0.38 0.835 0.983<br>Obart08g17720.1 0.434 0.833 0.399 0.105 0.76	1<br>Osjap03g31670.3 0.002 0.382 0.38 0.85 0.984<br>Osjap12g03930.1 0.382 0.835 0.453 0.004 0.758	1<br>Osind03g32640.1 0.002 0.382 0.38 0.835 0.983<br>Osind12g03880.1 0.382 0.835 0.453 0.004 0.758	0<br>Orufi12g03700.1 0.382 0.835 0.453 0.004 0.758	1<br>Olong_000521-R1 0.382 0.816 0.434 0.004 0.806<br>Olong_002508-R1 0.002 0.382 0.38 0.835 0.983	1<br>Opunc12g03040.1 0.002 0.382 0.38 0.823 0.967<br>Opunc12g03050.1 0.382 0.835 0.453 0.004 0.763	0<br>Obrac02g13530.3 0.468 0.891 0.423 0.273 0.927<br>Obrac12g02570.1 0.002 0.378 0.376 0.835 0.98	1<br>Lperr06g08630.1 0.002 0.382 0.38 0.835 0.983<br>Lperr12g03100.1 0.382 0.833 0.451 0.004 0.757	0<br>AT5G04180.1 0.391 0.833 0.442 0.007 0.722
Obart12g09410.1	1<br>Oglab12g09470.1 0.001 1 0.999 0.001 1	1<br>Obart12g09410.1 0.001 1 0.999 0.001 1	1<br>Osjap12g12320.1 0.001 1 0.999 0.001 1	1<br>Osind12g09530.1 0.001 1 0.999 0.001 1	1<br>Orufi12g10460.1 0.001 1 0.999 0.001 1	0	0	0	0	0
	1<br>Oglab01g34040.1 0.477 0.998 0.521 0.203 0.995<br>Oglab03g07280.1 0.001 0.306 0.305 0.004 0.996	1<br>Obart01g39260.2 0.452 0.998 0.546 0.017 0.994<br>Obart03g07210.1 0.001 0.306 0.305 0.004 0.996	1<br>Osjap01g44780.3 0.452 0.998 0.546 0.017 0.994<br>Osjap03g07600.1 0.001 0.306 0.305 0.004 0.996	1<br>Osind01g46040.1 0.452 0.998 0.546 0.186 0.995<br>Osind03g07670.1 0.001 0.306 0.305 0.004 0.996	1<br>Orufi01g42650.3 0.452 0.998 0.546 0.228 0.995<br>Orufi03g07180.1 0.001 0.306 0.305 0.004 0.996	1<br>Olong_021912-R1 0.376 1 0.624 0.002 1<br>Olong_021913-R1 0.001 0.305 0.304 0.004 0.996	1<br>Opunc12g08570.1 0.406 1 0.594 0.152 1<br>Opunc12g08580.1 0.001 0.305 0.304 0.004 0.996	1<br>Obrac12g06490.1 0.001 0.305 0.304 0.004 0.996<br>Obrac12g06500.1 0.449 1 0.551 0.103 1	1<br>Lperr03g06600.1 0.001 0.304 0.303 0.004 0.992<br>Lperr12g08310.1 0.452 0.999 0.547 0.002 0.998	1<br>AT4G34670.1 0.001 0.297 0.296 0.004 0.966<br>AT5G67540.2 0.477 0.993 0.516 0.053 1
Obart12g13650.1	0	1<br>Obart12g13650.1 0.004 1 0.996 0.004 1	0	0	0	0	0	0	0	0
	1<br>Oglab11g18280.1 0.79 1 0.21 0.514 1<br>Oglab12g12900.1 0.004 0.786 0.782 0.005 1	1<br>Obart03g06960.1 0.028 0.762 0.734 0.145 0.991<br>Obart11g20740.1 0.79 1 0.21 0.514 1	1<br>Osjap07g23370.2 0.79 0.992 0.202 0.086 0.168<br>Osjap12g17390.1 0.004 0.786 0.782 0.005 1	1<br>Osind11g16870.1 0.79 0.996 0.206 0.143 0.968<br>Osind12g14200.1 0.004 0.786 0.782 0.005 1	1<br>Orufi07g20930.2 0.79 0.992 0.202 0.087 0.169<br>Orufi12g14980.1 0.004 0.786 0.782 0.005 1	0<br>Olong_014123-R1 0.024 0.69 0.666 0.098 0.995	1<br>Opunc07g18980.2 0.79 1 0.21 0.014 0.105<br>Opunc12g12320.1 0.012 0.786 0.774 0.01 1	1<br>Obrac03g05620.1 0.056 0.706 0.65 0.158 0.937<br>Obrac12g08990.2 0.754 0.976 0.222 0.26 0.844	1<br>Lperr11g17030.1 0.79 1 0.21 0.116 0.884<br>Lperr12g11520.1 0.02 0.698 0.678 0.005 0.88	0<br>AT4G25830.1 0.04 0.746 0.706 0.017 1

Obart12g18260.1	0	1<br>Obart12g18260.1 0.002 1 0.998 0.002 1	0	0	0	0	1<br>Opunc12g16510.1 0.029 1 0.971 0.374 1	0	1<br>Lperr12g15270.1 0.002 1 0.998 0.002 1<br>Lperr12g15270.2 0.002 1 0.998 0.003 1	0
	1<br>Oglab03g30800.1 0.002 0.282 0.28 0.007 0.762<br>Oglab12g16450.1 0.402 1 0.598 0.42 1	1<br>Obart05g22220.1 0.431 1 0.569 0.47 1<br>Obart09g04390.1 0.002 0.282 0.28 0.02 0.77	1<br>Osjap03g34300.1 0.002 0.282 0.28 0.437 0.827<br>Osjap12g23030.1 0.402 1 0.598 0.42 1	1<br>Osind12g19080.1 0.402 1 0.598 0.42 1<br>Osind12g19090.1 0.002 0.282 0.28 0.007 0.767	1<br>Orufi09g04980.1 0.002 0.282 0.28 0.02 0.77<br>Orufi12g20240.1 0.402 1 0.598 0.42 1	1<br>Olong_014799-R1 0.002 0.282 0.28 0.007 0.8<br>Olong_025924-R1 0.402 1 0.598 0.195 1	1<br>Opunc05g19900.1 0.431 1 0.569 0.452 1<br>Opunc09g03850.1 0.002 0.282 0.28 0.02 0.77	1<br>Obrac03g23420.1 0.002 0.282 0.28 0.006 0.696<br>Obrac12g11780.1 0.404 1 0.596 0.236 1	1<br>Lperr03g26330.1 0.002 0.282 0.28 0.339 0.837<br>Lperr05g18130.1 0.431 1 0.569 0.387 1	0<br>AT1G23260.1 0.01 0.282 0.272 0.013 0.696<br>AT4G14770.1 0.431 0.652 0.221 0.682 0.818

(((((_c1,longi),punctata),brachy),leersia),tair);

chimeric gene id	INGROUP					MIDGROUP		OUTGROUP		
	jap	indica	rufi	glab	barthii	longi	punctata	brachy	leersia	tair
Obart01g07430.1	1<br>Osjap01g08660.1 0.007 1 0.993 0.026 1	1<br>Osind01g08490.1 0.007 1 0.993 0.026 1	1<br>Orufi01g08070.1 0.007 1 0.993 0.026 1	1<br>Oglab01g06470.1 0.013 1 0.987 0.02 1	1<br>Obart01g07430.1 0.001 1 0.999 0.001 1	1<br>Olong_011567-R1 0.036 1 0.964 0.001 1	0	0	0	0
	1<br>Osjap01g29230.2 0.078 0.648 0.57 0.25 0.983<br>Osjap05g00560.2 0.713 0.997 0.284 0.146 1	1<br>Osind01g30690.1 0.078 0.648 0.57 0.267 0.982<br>Osind05g00540.1 0.713 0.997 0.284 0.126 1	1<br>Orufi01g27950.1 0.078 0.648 0.57 0.25 0.983<br>Orufi01g48560.2 0.713 0.983 0.27 0.085 0.984	1<br>Oglab01g21070.2 0.078 0.648 0.57 0.249 0.983<br>Oglab05g00530.1 0.713 0.997 0.284 0.146 1	1<br>Obart01g25090.1 0.078 0.648 0.57 0.219 0.982<br>Obart05g00540.1 0.713 0.997 0.284 0.146 1	1<br>Olong_003039-R1 0.713 0.997 0.284 0.128 1<br>Olong_003692-R2 0.109 0.648 0.539 0.244 0.979	1<br>Opunc01g07160.1 0.656 1 0.344 0.111 1<br>Opunc01g07170.1 0.003 0.649 0.646 0.034 0.984	1<br>Obrac01g05760.3 0.66 1 0.34 0.082 1<br>Obrac01g18840.2 0.001 0.648 0.647 0.062 0.974	1<br>Lperr01g07030.1 0.66 1 0.34 0.018 1<br>Lperr01g07040.2 0.002 0.649 0.647 0.013 0.989	1<br>AT1G54350.1 0.659 0.994 0.335 0.076 0.975<br>AT3G61740.1 0.009 0.648 0.639 0.077 0.983
Obart01g43280.1	1<br>Osjap01g49090.1 0.028 0.999 0.971 0.001 0.999	1<br>Osind01g50590.1 0.028 0.999 0.971 0.001 0.999	1<br>Orufi01g46760.2 0.026 0.999 0.973 0.001 0.999	1<br>Oglab01g37970.1 0 0.999 0.999 0 0.999<br>Oglab01g37970.2 0 0.999 0.999 0 0.999<br>Oglab01g37970.3 0.028 0.999 0.971 0 0.999	1<br>Obart01g43280.1 0 1 1 0 1<br>Obart01g43280.2 0.028 1 0.972 0 1	1<br>Olong_026121-R2 0.026 0.886 0.86 0 1<br>Olong_026121-R3 0.028 0.886 0.858 0.001 1	1<br>Opunc01g42300.1 0.027 0.999 0.972 0.043 0.999	1<br>Obrac01g33850.1 0.027 0.896 0.869 0 0.979<br>Obrac01g33850.2 0.027 0.896 0.869 0 0.979<br>Obrac01g33850.3 0.027 0.896 0.869 0 0.999	0	0
	1<br>Osjap01g49070.1 0.447 1 0.553 0.002 1<br>Osjap01g49110.1 0.027 0.435 0.408 0.074 1	1<br>Osind01g50550.1 0.443 1 0.557 0.001 1<br>Osind02g04390.1 0.028 0.429 0.401 0.001 0.995	1<br>Orufi01g46740.1 0.446 1 0.554 0.013 1<br>Orufi11g06790.1 0.027 0.425 0.398 0.002 1	1<br>Oglab01g37960.1 0.438 1 0.562 0.009 0.999<br>Oglab01g37980.1 0.028 0.435 0.407 0.001 1	0<br>Obart01g43290.1 0.027 0.496 0.469 0.033 0.976	0<br>Olong_026121-R1 0.007 0.436 0.429 0.001 0.988	1<br>Opunc01g42270.1 0.433 1 0.567 0.005 1<br>Opunc11g06550.1 0.028 0.433 0.405 0 0.984	1<br>Obrac01g33820.1 0.446 1 0.554 0.008 1<br>Obrac02g02870.1 0.027 0.431 0.404 0.244 1	1<br>Lperr01g37690.1 0.445 1 0.555 0.022 1<br>Lperr11g05950.1 0.026 0.439 0.413 0 0.968	1<br>AT1G03220.1 0.446 0.999 0.553 0.067 0.993<br>AT3G14460.1 0.042 0.432 0.39 0.034 0.999
Obart02g23460.2	1<br>Osjap02g26750.1 0.001 0.969 0.968 0.001 0.994<br>Osjap02g26750.2 0.001 0.969 0.968 0.001 0.994<br>Osjap02g26750.4 0.001 0.969 0.968 0.001 0.799	1<br>Osind02g26640.2 0.001 1 0.999 0.001 1	1<br>Orufi02g24900.1 0.011 0.974 0.963 0.06 0.923<br>Orufi02g24900.3 0.001 0.974 0.973 0.001 0.857<br>Orufi02g24900.4 0.001 0.974 0.973 0.001 0.856	1<br>Oglab02g22130.2 0.001 1 0.999 0.001 1	1<br>Obart02g23460.2 0.001 1 0.999 0.001 1	0	1<br>Opunc02g21410.1 0.011 0.999 0.988 0.116 0.996<br>Opunc02g21410.2 0.011 0.999 0.988 0.114 0.996<br>Opunc02g21410.3 0.011 0.999 0.988 0.115 0.996<br>Opunc02g21410.4 0.011 0.999 0.988 0.114 0.996	0	1<br>Lperr02g18970.1 0.011 0.997 0.986 0.032 0.996	0
	1<br>Osjap02g26590.1 0.475 0.999 0.524 0.033 0.999<br>Osjap02g26750.5 0.011 0.459 0.448 0.238 0.973	1<br>Osind02g26450.1 0.475 0.999 0.524 0.025 0.997<br>Osind02g26620.1 0.011 0.459 0.448 0.495 0.981	1<br>Orufi02g24730.1 0.475 0.994 0.519 0.031 0.996<br>Orufi02g24880.2 0.011 0.459 0.448 0.18 0.97	1<br>Oglab02g21950.1 0.475 0.999 0.524 0.073 0.995<br>Oglab02g22110.2 0.011 0.459 0.448 0.532 0.98	1<br>Obart02g23290.1 0.475 0.999 0.524 0.029 0.999<br>Obart02g23440.1 0.011 0.459 0.448 0.492 0.981	0<br>Olong_024345-R1 0.011 0.474 0.463 0.442 0.991	1<br>Opunc02g21230.1 0.479 0.976 0.497 0.003 0.934<br>Opunc02g21240.1 0.011 0.461 0.45 0.121 0.972	1<br>Obrac02g16670.1 0.549 0.973 0.424 0.076 0.995<br>Obrac02g16700.1 0.011 0.461 0.45 0.114 0.947	1<br>Lperr02g18740.1 0.485 0.997 0.512 0.056 0.991<br>Lperr02g19120.1 0.011 0.484 0.473 0.183 0.978	1<br>AT4G31860.1 0.014 0.45 0.436 0.123 0.989<br>AT5G43080.1 0.626 1 0.374 0.194 0.932
Obart02g38050.2	0	1<br>Osind02g41950.1 0.071 0.95 0.879 0.368 0.955	1<br>Orufi02g39760.1 0.001 1 0.999 0.001 0.95<br>Orufi02g39760.2 0.001 1 0.999 0.001 1	1<br>Oglab02g35180.1 0.001 0.976 0.975 0.001 0.919<br>Oglab02g35180.2 0.001 1 0.999 0.001 1	1<br>Obart02g38050.1 0.001 0.976 0.975 0.001 0.919<br>Obart02g38050.2 0.001 1 0.999 0.001 1	0	0	0	0	0
	1<br>Osjap05g10980.2 0.237 0.997 0.76 0.066 0.962	1<br>Osind05g11850.1 0.237 0.997 0.76 0.066 0.974	1<br>Orufi02g39760.3 0.001 0.231 0.23 0.005 0.886	1<br>Oglab02g35180.3 0.001 0.231 0.23 0.005 0.885	0<br>Obart03g04840.1 0.32 0.974 0.654 0.007 0.995	1<br>Olong_016606-R1 0.31 0.996 0.686 0.008 0.889	0<br>Opunc02g35330.1 0.208 1 0.792 0.014 1	1<br>Obrac05g06630.3 0.242 0.971 0.729 0.081 0.95	0<br>Lperr02g31540.1 0.208 1 0.792 0.017 1	0<br>AT5G38730.1 0.202 0.99 0.788 0.017 0.998

	Osjap05g11860.1 0.001 0.209 0.208 0.078 0.911	Osind06g11870.1 0.001 0.209 0.208 0.078 0.911	Orufi05g10490.3 0.237 0.949 0.712 0.064 0.962	Oglab07g10660.4 0.263 0.996 0.733 0.085 0.801		Olong_027192-R1 0.001 0.209 0.208 0.078 0.911		Obrac06g07800.1 0.001 0.209 0.208 0.078 0.911		
Obart03g00550.1	1<br>Osjap03g00680.1 0.001 0.996 0.995 0.001 0.994<br>Osjap03g00680.2 0.001 0.996 0.995 0.001 0.994<br>Osjap03g00680.3 0.001 0.996 0.995 0.001 0.994	1<br>Osind03g00740.1 0.001 1 0.999 0.001 1	1<br>Orufi03g12660.1 0.001 0.996 0.995 0.001 0.994<br>Orufi03g12660.2 0.001 0.996 0.995 0.001 0.994	1<br>Oglab03g00390.1 0.001 0.996 0.995 0.001 0.994	1<br>Obart03g00550.1 0.001 1 0.999 0.001 1	0	0	0	0	0
	1<br>Osjap02g42090.1 0.052 0.675 0.623 0.065 1<br>Osjap12g08580.1 0.706 0.996 0.29 0.011 0.977	1<br>Osind02g41670.1 0.061 0.675 0.614 0.131 1<br>Osind03g02220.1 0.695 0.996 0.301 0.015 0.982	1<br>Orufi02g39350.1 0.061 0.675 0.614 0.131 1<br>Orufi09g02220.2 0.742 0.996 0.254 0.095 0.975	1<br>Oglab02g34920.1 0.061 0.675 0.614 0.131 1<br>Oglab04g12740.1 0.715 0.996 0.281 0.411 0.989	1<br>Obart02g37790.1 0.061 0.645 0.584 0.133 0.977<br>Obart12g06510.1 0.673 0.996 0.323 0.052 0.984	0<br>Olong_013175-R1 0.27 0.904 0.634 0.002 0.961	1<br>Opunc03g00470.1 0.001 0.78 0.779 0.002 1<br>Opunc03g03130.2 0.78 0.996 0.216 0.443 0.984	0<br>Obrac02g27970.1 0.136 0.675 0.539 0.105 1<br>Obrac05g17660.1 0.747 0.995 0.248 0.427 0.997	1<br>Lperr03g00570.1 0.001 0.675 0.674 0.002 1<br>Lperr04g11360.1 0.748 0.996 0.248 0.415 0.99	0<br>AT5G04460.1 0.018 0.675 0.657 0.081 1
Obart03g09160.1	1<br>Osjap03g09700.1 0 1 1 0.046 1<br>Osjap03g09700.2 0 1 1 0.045 1	1<br>Osind03g09660.1 0 1 1 0.044 1	1<br>Orufi03g09320.1 0 1 1 0.045 1<br>Orufi03g09320.2 0 1 1 0.046 1<br>Orufi03g09320.3 0 1 1 0.047 1<br>Orufi03g09320.4 0 1 1 0.048 1	1<br>Oglab03g09270.1 0 1 1 0.001 1<br>Oglab03g09270.2 0 1 1 0.001 1	1<br>Obart03g09160.1 0 1 1 0 1<br>Obart03g09160.2 0 1 1 0 1<br>Obart03g09160.3 0 1 1 0 1	1<br>Olong_002353-R1 0.021 1 0.979 0.001 1	1<br>Opunc03g08800.1 0.029 1 0.971 0.026 1<br>Opunc03g08800.2 0.029 1 0.971 0.026 1<br>Opunc03g08800.3 0.029 1 0.971 0.027 1	0	0	0
	0<br>Osjap05g08590.1 0.096 0.467 0.371 0.436 0.995<br>Osjap06g17700.1 0.559 0.987 0.428 0.321 0.983	1<br>Osind01g00960.1 0.537 0.995 0.458 0.006 0.975<br>Osind01g21860.1 0.07 0.476 0.406 0.075 0.999	1<br>Orufi10g00220.1 0.588 0.987 0.399 0.171 0.96<br>Orufi10g00230.1 0.07 0.477 0.407 0.074 1	1<br>Oglab10g00260.1 0.588 0.987 0.399 0.179 0.96<br>Oglab10g00270.1 0.07 0.477 0.407 0.072 1	1<br>Obart10g00240.1 0.588 0.987 0.399 0.179 0.96<br>Obart10g00250.1 0.07 0.477 0.407 0.072 1	1<br>Olong_008994-R1 0.07 0.476 0.406 0.07 0.999<br>Olong_023066-R1 0.591 0.995 0.404 0.021 0.97	0<br>Opunc03g08800.7 0.355 1 0.645 0.004 1	1<br>Obrac03g07350.1 0.557 1 0.443 0.114 1<br>Obrac10g00260.1 0.093 0.476 0.383 0.03 0.999	1<br>Lperr01g01500.1 0.083 0.479 0.396 0.001 1<br>Lperr03g08210.1 0.555 1 0.445 0.133 1	1<br>AT1G69770.1 0.091 0.467 0.376 0.027 0.988<br>AT4G34440.1 0.533 0.992 0.459 0.122 0.975
Obart03g09160.2	1<br>Osjap03g09700.1 0 1 1 0.046 1<br>Osjap03g09700.2 0 1 1 0.045 1	1<br>Osind03g09660.1 0 1 1 0.044 1	1<br>Orufi03g09320.1 0 1 1 0.045 1<br>Orufi03g09320.2 0 1 1 0.046 1<br>Orufi03g09320.3 0 1 1 0.047 1<br>Orufi03g09320.4 0 1 1 0.048 1	1<br>Oglab03g09270.1 0 1 1 0.001 1<br>Oglab03g09270.2 0 1 1 0.001 1	1<br>Obart03g09160.1 0 1 1 0 1<br>Obart03g09160.2 0 1 1 0 1<br>Obart03g09160.3 0 1 1 0 1	1<br>Olong_002353-R1 0.021 1 0.979 0.001 1	1<br>Opunc03g08800.1 0.029 1 0.971 0.026 1<br>Opunc03g08800.2 0.029 1 0.971 0.026 1<br>Opunc03g08800.3 0.029 1 0.971 0.027 1	0	0	0
	0<br>Osjap05g08590.1 0.096 0.467 0.371 0.436 0.995<br>Osjap06g17700.1 0.559 0.987 0.428 0.321 0.983	1<br>Osind01g21860.1 0.07 0.476 0.406 0.075 0.999<br>Osind06g17200.1 0.559 0.987 0.428 0.318 0.983	1<br>Orufi10g00220.1 0.588 0.987 0.399 0.171 0.96<br>Orufi10g00230.1 0.07 0.477 0.407 0.074 1	1<br>Oglab10g00260.1 0.588 0.987 0.399 0.179 0.96<br>Oglab10g00270.1 0.07 0.477 0.407 0.072 1	1<br>Obart10g00240.1 0.588 0.987 0.399 0.179 0.96<br>Obart10g00250.1 0.07 0.477 0.407 0.072 1	1<br>Olong_008994-R1 0.07 0.476 0.406 0.07 0.999<br>Olong_023066-R1 0.591 0.995 0.404 0.021 0.97	0<br>Opunc03g08800.7 0.355 1 0.645 0.004 1	1<br>Obrac03g07350.1 0.557 1 0.443 0.114 1<br>Obrac10g00260.1 0.093 0.476 0.383 0.03 0.999	1<br>Lperr01g01500.1 0.083 0.479 0.396 0.001 1<br>Lperr03g08210.1 0.555 1 0.445 0.133 1	1<br>AT1G69770.1 0.091 0.467 0.376 0.027 0.988<br>AT4G34440.1 0.533 0.992 0.459 0.122 0.975
Obart03g09160.3	1<br>Osjap03g09700.1 0 1 1 0.046 1<br>Osjap03g09700.2 0 1 1 0.045 1	1<br>Osind03g09660.1 0 1 1 0.044 1	1<br>Orufi03g09320.1 0 1 1 0.045 1<br>Orufi03g09320.2 0 1 1 0.046 1<br>Orufi03g09320.3 0 1 1 0.047 1<br>Orufi03g09320.4 0 1 1 0.048 1	1<br>Oglab03g09270.1 0 1 1 0.001 1<br>Oglab03g09270.2 0 1 1 0.001 1	1<br>Obart03g09160.1 0 1 1 0 1<br>Obart03g09160.2 0 1 1 0 1<br>Obart03g09160.3 0 1 1 0 1	1<br>Olong_002353-R1 0.021 1 0.979 0.001 1	1<br>Opunc03g08800.1 0.028 1 0.972 0.026 1<br>Opunc03g08800.2 0.028 1 0.972 0.026 1<br>Opunc03g08800.3 0.028 1 0.972 0.027 1	0	0	0
	1<br>Osjap05g08590.1 0.095 0.459 0.364 0.436 0.995<br>Osjap06g17700.1 0.55 0.988 0.438 0.321 0.983	1<br>Osind01g21860.1 0.069 0.469 0.4 0.075 1<br>Osind06g17200.1 0.55 0.988 0.438 0.318 0.983	1<br>Orufi10g00220.1 0.578 0.988 0.41 0.171 0.96<br>Orufi10g00230.1 0.069 0.469 0.4 0.074 1	1<br>Oglab10g00260.1 0.578 0.988 0.41 0.179 0.96<br>Oglab10g00270.1 0.069 0.469 0.4 0.072 1	1<br>Obart10g00240.1 0.578 0.988 0.41 0.179 0.96<br>Obart10g00250.1 0.069 0.469 0.4 0.072 1	1<br>Olong_008994-R1 0.069 0.469 0.4 0.07 0.999<br>Olong_023066-R1 0.581 0.995 0.414 0.021 0.97	0<br>Opunc03g08800.7 0.349 1 0.651 0.004 1	1<br>Obrac03g07350.1 0.548 1 0.452 0.114 1<br>Obrac10g00260.1 0.092 0.469 0.377 0.03 0.999	1<br>Lperr01g01500.1 0.082 0.471 0.389 0.001 1<br>Lperr03g08210.1 0.546 1 0.454 0.133 1	1<br>AT1G69770.1 0.089 0.459 0.37 0.027 0.988<br>AT4G34440.1 0.525 0.992 0.467 0.122 0.975
Obart03g12120.1	1<br>Osjap03g13100.1 0.028 1 0.972 0.001 1	1<br>Osind03g12930.1 0.001 1 0.999 0.001 1	0	1<br>Oglab03g12120.1 0.001 1 0.999 0.001 1	1<br>Obart03g12120.1 0.001 1 0.999 0.001 1	0	0	0	0	0
	1	1	1	1	1	1	1	1	1	1

	Osjap04g16240.1 0.642 0.998 0.356 0.067 0.921<br>Osjap12g01140.1 0.109 0.633 0.524 0.498 0.981	Osind04g14790.1 0.642 0.998 0.356 0.013 0.911<br>Osind12g01290.1 0.109 0.633 0.524 0.49 0.981	Orufi06g01460.3 0.642 0.998 0.356 0.212 0.912<br>Orufi12g01130.1 0.109 0.633 0.524 0.498 0.981	Oglab06g01620.1 0.642 0.998 0.356 0.212 0.912<br>Oglab12g00800.1 0.114 0.633 0.519 0.483 0.983	Obart04g15560.1 0.64 0.998 0.358 0.146 0.877<br>Obart11g01060.1 0.114 0.633 0.519 0.471 0.982	Olong_007714-R9 0.642 1 0.358 0.002 1<br>Olong_007715-R1 0.125 0.633 0.508 0.007 0.965	Opunc03g11680.1 0.028 0.633 0.605 0.001 0.971<br>Opunc04g11270.1 0.642 0.998 0.356 0.067 0.921	Obrac03g09720.1 0.025 0.633 0.608 0.036 0.971<br>Obrac04g09430.1 0.64 0.998 0.358 0.014 0.863	Lperr03g10720.1 0.028 0.633 0.605 0.001 0.934<br>Lperr03g17470.1 0.633 0.998 0.365 0.119 0.828	AT3G46740.1 0.122 0.633 0.511 0.158 0.971<br>AT3G53360.1 0.672 0.998 0.326 0.09 0.934
Obart03g20630.1	1<br>Osjap03g22210.1 0.009 0.991 0.982 0.567 0.972	1<br>Osind03g22510.1 0.017 1 0.983 0.552 1	1<br>Orufi03g21250.1 0.185 0.995 0.81 0.02 0.991<br>Orufi03g21250.2 0.185 0.995 0.81 0.019 0.991<br>Orufi03g21250.3 0.189 0.995 0.806 0.021 0.99<br>Orufi03g21250.4 0.185 0.995 0.81 0.021 0.99	1<br>Oglab03g20370.1 0.073 1 0.927 0.087 1	1<br>Obart03g20630.1 0.001 1 0.999 0.001 1	0	0	0	0	0
	1<br>Osjap05g27870.1 0.044 0.547 0.503 0.075 0.982<br>Osjap12g12160.2 0.556 0.931 0.375 0.083 0.997	1<br>Osind02g20400.1 0.607 0.931 0.324 0.137 0.985<br>Osind05g28150.1 0.044 0.547 0.503 0.075 0.982	1<br>Orufi05g26490.1 0.044 0.553 0.509 0.075 0.989<br>Orufi09g01530.2 0.557 0.931 0.374 0.087 0.965	1<br>Oglab05g22780.1 0.044 0.547 0.503 0.077 0.982<br>Oglab08g00090.1 0.557 0.931 0.374 0.117 0.976	1<br>Obart06g03480.2 0.051 0.556 0.505 0.002 0.826<br>Obart09g16880.1 0.557 0.931 0.374 0.091 0.964	1<br>Olong_013421-R1 0.051 0.556 0.505 0.002 0.877<br>Olong_028288-R1 0.607 1 0.393 0.003 1	1<br>Opunc05g00870.2 0.567 0.996 0.429 0.145 0.973<br>Opunc05g22250.1 0.044 0.547 0.503 0.075 0.982	0<br>Obrac09g04370.1 0.08 0.572 0.492 0.034 0.858	0<br>Lperr05g06040.4 0.557 0.992 0.435 0.084 0.556	0<br>AT1G07740.1 0.025 0.531 0.506 0.083 0.967
Obart03g27980.1	1<br>Osjap03g30460.1 0.021 1 0.979 0.085 1	1<br>Osind03g31500.1 0.021 1 0.979 0.094 1	1<br>Orufi03g29110.1 0.021 1 0.979 0.341 1	0	1<br>Obart03g27980.1 0.001 1 0.999 0.001 1<br>Obart03g27980.2 0.015 1 0.985 0.015 1	0	0	0	0	0
	1<br>Osjap03g31370.3 0.509 0.998 0.489 0.042 0.887<br>Osjap12g24400.2 0.019 0.475 0.456 0.012 0.988	1<br>Osind04g00720.3 0.509 0.999 0.49 0.064 0.919<br>Osind12g20440.1 0.019 0.475 0.456 0.01 0.99	1<br>Orufi03g30600.1 0.509 0.999 0.49 0.05 0.885<br>Orufi12g21550.1 0.019 0.475 0.456 0.01 0.99	1<br>Oglab03g27330.1 0.51 1 0.49 0.043 1<br>Oglab03g27340.1 0.008 0.476 0.468 0.008 0.997	1<br>Obart01g35640.1 0.509 0.998 0.489 0.063 0.982<br>Obart12g19300.1 0.019 0.475 0.456 0.01 0.99	1<br>Olong_006906-R1 0.04 0.468 0.428 0.071 1<br>Olong_007449-R1 0.509 0.999 0.49 0.065 0.909	1<br>Opunc03g25490.1 0.021 0.486 0.465 0.525 0.8<br>Opunc03g26940.1 0.509 0.999 0.49 0.052 0.885	1<br>Obrac03g20710.1 0.021 0.476 0.455 0.028 0.997<br>Obrac03g21410.1 0.574 0.995 0.421 0.09 0.91	1<br>Lperr03g23190.1 0.51 0.999 0.489 0.039 0.863<br>Lperr03g23200.1 0.009 0.476 0.467 0.008 0.997	0<br>AT5G36940.1 0.019 0.478 0.459 0.003 0.998
Obart03g27980.2	1<br>Osjap03g30460.1 0.021 1 0.979 0.085 1	1<br>Osind03g31500.1 0.021 1 0.979 0.094 1	1<br>Orufi03g29110.1 0.021 1 0.979 0.341 1	0	1<br>Obart03g27980.1 0.015 1 0.985 0.015 1<br>Obart03g27980.2 0.001 1 0.999 0.001 1	0	0	0	0	0
	1<br>Osjap03g31370.3 0.516 0.998 0.482 0.042 0.887<br>Osjap12g24400.2 0.018 0.483 0.465 0.012 0.988	1<br>Osind04g00720.3 0.516 0.999 0.483 0.064 0.919<br>Osind12g20440.1 0.018 0.483 0.465 0.01 0.99	1<br>Orufi03g30600.1 0.516 0.999 0.483 0.05 0.885<br>Orufi12g21550.1 0.018 0.483 0.465 0.01 0.99	1<br>Oglab03g27330.1 0.517 1 0.483 0.043 1<br>Oglab12g17460.4 0.018 0.483 0.465 0.012 0.996	1<br>Obart01g35640.1 0.516 0.998 0.482 0.063 0.982<br>Obart12g19300.1 0.018 0.483 0.465 0.01 0.99	1<br>Olong_002926-R1 0.024 0.484 0.46 0.002 0.996<br>Olong_007449-R1 0.516 0.999 0.483 0.065 0.909	1<br>Opunc03g25490.2 0.021 0.487 0.466 0.543 0.811<br>Opunc03g26940.1 0.516 0.999 0.483 0.052 0.885	1<br>Obrac03g20710.1 0.021 0.484 0.463 0.028 0.997<br>Obrac03g21410.1 0.58 0.995 0.415 0.09 0.91	1<br>Lperr03g23190.1 0.517 0.999 0.482 0.039 0.895<br>Lperr03g23200.1 0.008 0.484 0.476 0.008 0.997	0<br>AT5G36940.1 0.019 0.486 0.467 0.003 0.998
Obart03g31750.1	1<br>Osjap03g34330.1 0.046 1 0.954 0.001 1	1<br>Osind03g35550.1 0.046 1 0.954 0.039 1	1<br>Orufi03g33060.1 0.046 1 0.954 0.001 1	1<br>Oglab03g30830.1 0.046 1 0.954 0.001 1	1<br>Obart03g31750.1 0.001 1 0.999 0.001 1	0	0	0	0	0
	1<br>Osjap03g39110.1 0.667 1 0.333 0.036 1<br>Osjap10g05370.1 0.034 0.602 0.568 0.051 0.963	1<br>Osind01g46540.1 0.667 0.999 0.332 0.065 0.994<br>Osind10g03770.1 0.034 0.602 0.568 0.057 0.96	1<br>Orufi10g04850.1 0.034 0.602 0.568 0.054 0.963<br>Orufi11g12520.1 0.668 1 0.332 0.038 0.991	1<br>Oglab03g34460.1 0.646 0.997 0.351 0.016 0.993<br>Oglab10g04340.1 0.028 0.602 0.574 0.058 0.963	1<br>Obart03g36200.1 0.667 1 0.333 0.016 0.997<br>Obart10g04600.1 0.058 0.602 0.544 0.106 0.962	1<br>Olong_014796-R1 0.046 0.518 0.472 0.002 0.95<br>Olong_024872-R1 0.664 1 0.336 0.01 1	1<br>Opunc01g42030.1 0.667 1 0.333 0.28 0.872<br>Opunc03g28940.1 0.041 0.62 0.579 0.049 0.998	1<br>Obrac02g16280.1 0.662 0.982 0.32 0.063 0.949<br>Obrac03g23440.1 0.046 0.62 0.574 0.002 0.998	1<br>Lperr03g30520.1 0.66 1 0.34 0.003 0.997<br>Lperr10g03360.1 0.013 0.602 0.589 0.009 0.962	0<br>AT1G70730.3 0.046 0.618 0.572 0.118 0.995
Obart03g33760.1	1<br>Osjap03g36400.1 0.003 1 0.997 0.003 1	1<br>Osind03g37600.1 0.003 1 0.997 0.003 1	1<br>Orufi03g35150.1 0.003 1 0.997 0.003 1	1<br>Oglab03g32170.1 0.003 1 0.997 0.003 1<br>Oglab03g32170.2 0.003 1 0.997 0.003 1	1<br>Obart03g33760.1 0.003 1 0.997 0.003 1	1<br>Olong_002225-R1 0.003 1 0.997 0.136 1<br>Olong_002225-R2 0.003 1 0.997 0.003 1	1<br>Opunc03g30920.1 0.003 1 0.997 0.003 1	0	0	0
	1<br>Osjap01g12980.2 0.547 0.979 0.432 0.032 0.961<br>Osjap03g04690.1 0.003 0.414 0.411 0.007 1	1<br>Osind03g04960.1 0.003 0.414 0.411 0.007 1<br>Osind12g02610.1 0.538 0.991 0.453 0.005 0.759	1<br>Orufi03g04350.1 0.003 0.414 0.411 0.007 1<br>Orufi05g22410.2 0.547 0.997 0.45 0.031 0.969	0<br>Oglab05g19940.1 0.32 0.991 0.671 0.015 0.997	1<br>Obart01g34520.1 0.547 0.991 0.444 0.027 0.818<br>Obart03g04650.1 0.003 0.414 0.411 0.007 1	1<br>Olong_020510-R2 0.523 0.991 0.468 0.291 0.873<br>Olong_025165-R1 0.003 0.414 0.411 0.007 1	0<br>Opunc12g01920.1 0.526 0.973 0.447 0.663 0.947	0<br>Obrac03g25020.5 0.003 0.535 0.532 0.439 0.972	1<br>Lperr01g29830.2 0.532 0.991 0.459 0.203 0.858<br>Lperr03g28250.1 0.003 0.414 0.411 0.007 1	1<br>AT1G52740.1 0.003 0.414 0.411 0.007 1<br>AT4G14640.1 0.544 0.979 0.435 0.033 0.987

Obart05g00520.1	1<br>Osjap05g00530.1 0.001 0.993 0.992 0.084 0.999<br>Osjap05g00530.2 0.001 0.993 0.992 0.103 0.999<br>Osjap05g00530.3 0.001 0.993 0.992 0.104 0.999<br>Osjap05g00530.4 0.001 0.993 0.992 0.091 0.999	1<br>Osind05g00510.1 0.049 0.993 0.944 0.001 0.999	1<br>Orufi05g00500.1 0.001 0.993 0.992 0.084 0.999	0	1<br>Obart05g00520.1 0.001 1 0.999 0.001 1<br>Obart05g00520.2 0.001 0.995 0.994 0.001 0.999	1<br>Olong_022405-R1 0.049 0.993 0.944 0.001 0.999	0	0	0	0
	1<br>Osjap02g39750.1 0.337 0.988 0.651 0.004 0.956<br>Osjap07g27300.1 0.003 0.291 0.288 0.116 0.834	1<br>Osind09g15830.1 0.34 0.993 0.653 0.029 0.998<br>Osind10g10530.3 0.001 0.336 0.335 0.088 0.826	1<br>Orufi07g24760.1 0.003 0.291 0.288 0.116 0.834<br>Orufi09g16480.1 0.34 0.993 0.653 0.029 0.998	1<br>Oglab05g00500.1 0.001 0.294 0.293 0.218 0.982<br>Oglab09g13820.1 0.322 0.993 0.671 0.064 0.998	1<br>Obart06g01490.1 0.017 0.293 0.276 0.2 0.982<br>Obart09g15270.1 0.34 0.993 0.653 0.029 0.998	1<br>Olong_018361-R1 0.34 0.993 0.653 0.012 0.998<br>Olong_020432-R1 0.017 0.325 0.308 0.115 0.666	1<br>Opunc05g00480.1 0.329 0.993 0.664 0.007 0.998<br>Opunc05g00490.1 0.001 0.294 0.293 0.228 0.982	1<br>Obrac02g14170.4 0.001 0.3 0.299 0.03 0.843<br>Obrac02g28140.1 0.342 0.989 0.647 0.039 0.973	1<br>Lperr05g00500.1 0.331 0.993 0.662 0.024 0.994<br>Lperr10g08300.1 0.001 0.291 0.29 0.117 0.971	0<br>AT5G44230.1 0.001 0.298 0.297 0.155 0.872
Obart05g00520.2	1<br>Osjap05g00530.1 0.001 1 0.999 0.084 1<br>Osjap05g00530.2 0.001 1 0.999 0.103 1<br>Osjap05g00530.3 0.001 1 0.999 0.104 1<br>Osjap05g00530.4 0.001 1 0.999 0.091 1	1<br>Osind05g00510.1 0.049 1 0.951 0.001 1	1<br>Orufi05g00500.1 0.001 1 0.999 0.084 1	0	1<br>Obart05g00520.1 0.001 0.999 0.998 0.001 0.995<br>Obart05g00520.2 0.001 1 0.999 0.001 1	1<br>Olong_022405-R1 0.049 1 0.951 0.001 1	0	0	0	0
	1<br>Osjap02g39750.1 0.339 0.994 0.655 0.004 0.956<br>Osjap07g27300.1 0.003 0.293 0.29 0.116 0.834	1<br>Osind09g15830.1 0.342 1 0.658 0.029 1<br>Osind10g10530.1 0.001 0.293 0.292 0.108 0.858	1<br>Orufi07g24760.1 0.003 0.293 0.29 0.116 0.834<br>Orufi09g16480.1 0.342 1 0.658 0.029 1	1<br>Oglab05g00500.1 0.001 0.295 0.294 0.218 0.982<br>Oglab09g13820.1 0.324 1 0.676 0.064 1	1<br>Obart06g01490.1 0.017 0.295 0.278 0.2 0.982<br>Obart09g15270.1 0.342 1 0.658 0.029 1	1<br>Olong_018361-R1 0.342 1 0.658 0.012 1<br>Olong_020432-R1 0.017 0.327 0.31 0.115 0.666	1<br>Opunc05g00480.1 0.331 0.999 0.668 0.007 0.998<br>Opunc05g00490.1 0.001 0.295 0.294 0.228 0.982	1<br>Obrac02g14170.4 0.001 0.302 0.301 0.03 0.843<br>Obrac02g28140.1 0.344 0.995 0.651 0.039 0.973	1<br>Lperr05g00500.1 0.334 1 0.666 0.024 0.996<br>Lperr10g08300.1 0.001 0.293 0.292 0.117 0.971	0<br>AT5G44230.1 0.001 0.3 0.299 0.155 0.872
Obart05g03830.1	1<br>Osjap05g04120.1 0.001 1 0.999 0.001 1<br>Osjap05g04120.2 0.001 1 0.999 0.001 1	1<br>Osind05g04070.1 0.008 1 0.992 0.001 1<br>Osind05g04070.2 0.008 1 0.992 0.001 1	1<br>Orufi05g03950.1 0.001 1 0.999 0.001 1<br>Orufi05g03950.2 0.001 1 0.999 0.001 1	1<br>Oglab05g03490.1 0.001 1 0.999 0.001 1<br>Oglab05g03490.2 0.001 1 0.999 0.001 1	1<br>Obart05g03830.1 0.001 1 0.999 0.001 1<br>Obart05g03830.2 0.001 1 0.999 0.001 1	1<br>Olong_016739-R1 0.074 1 0.926 0.001 1	1<br>Opunc05g03540.1 0.001 1 0.999 0.001 1<br>Opunc05g03540.2 0.001 1 0.999 0.001 1	1<br>Obrac05g02930.1 0.074 1 0.926 0.001 1	0	0
	1<br>Osjap01g27410.1 0.09 0.723 0.633 0.066 0.996<br>Osjap03g15140.5 0.743 1 0.257 0.061 1	1<br>Osind01g28630.1 0.09 0.723 0.633 0.066 0.996<br>Osind03g15230.1 0.79 1 0.21 0.03 1	1<br>Orufi01g26130.1 0.09 0.723 0.633 0.066 0.996<br>Orufi03g14720.1 0.79 1 0.21 0.03 1	1<br>Oglab01g19490.1 0.09 0.723 0.633 0.066 0.996<br>Oglab03g14060.1 0.79 1 0.21 0.03 1	1<br>Obart01g23310.1 0.09 0.723 0.633 0.066 0.996<br>Obart03g14050.1 0.79 1 0.21 0.03 1	1<br>Olong_003347-R2 0.09 0.723 0.633 0.047 0.996<br>Olong_009943-R1 0.79 1 0.21 0.03 1	1<br>Opunc01g23460.1 0.09 0.723 0.633 0.064 0.996<br>Opunc03g13590.1 0.79 1 0.21 0.002 0.545	1<br>Obrac01g17590.1 0.09 0.723 0.633 0.067 0.996<br>Obrac03g11330.1 0.79 1 0.21 0.03 1	0<br>Lperr05g03460.1 0.001 0.721 0.72 0.001 0.993	1<br>AT2G47580.1 0.775 1 0.225 0.008 1<br>AT3G16630.1 0.003 0.721 0.718 0.031 0.992
Obart05g03830.2	1<br>Osjap05g04120.1 0.001 1 0.999 0.001 1<br>Osjap05g04120.2 0.001 1 0.999 0.001 1	1<br>Osind05g04070.1 0.008 1 0.992 0.001 1<br>Osind05g04070.2 0.008 1 0.992 0.001 1	1<br>Orufi05g03950.1 0.001 1 0.999 0.001 1<br>Orufi05g03950.2 0.001 1 0.999 0.001 1	1<br>Oglab05g03490.1 0.001 1 0.999 0.001 1<br>Oglab05g03490.2 0.001 1 0.999 0.001 1	1<br>Obart05g03830.1 0.001 1 0.999 0.001 1<br>Obart05g03830.2 0.001 1 0.999 0.001 1	1<br>Olong_016739-R1 0.073 1 0.927 0.001 1	1<br>Opunc05g03540.1 0.001 1 0.999 0.001 1<br>Opunc05g03540.2 0.001 1 0.999 0.001 1	1<br>Obrac05g02930.1 0.073 1 0.927 0.001 1	0	0
	1<br>Osjap01g27410.1 0.089 0.726 0.637 0.066 0.996<br>Osjap03g15140.5 0.747 1 0.253 0.061 1	1<br>Osind01g28630.1 0.089 0.726 0.637 0.066 0.996<br>Osind03g15230.1 0.792 1 0.208 0.03 1	1<br>Orufi01g26130.1 0.089 0.726 0.637 0.066 0.996<br>Orufi03g14720.1 0.792 1 0.208 0.03 1	1<br>Oglab01g19490.1 0.089 0.726 0.637 0.066 0.996<br>Oglab03g14060.1 0.792 1 0.208 0.03 1	1<br>Obart01g23310.1 0.089 0.726 0.637 0.066 0.996<br>Obart03g14050.1 0.792 1 0.208 0.03 1	1<br>Olong_003347-R2 0.089 0.726 0.637 0.047 0.996<br>Olong_009943-R1 0.792 1 0.208 0.03 1	1<br>Opunc01g23460.1 0.089 0.726 0.637 0.064 0.996<br>Opunc03g13590.1 0.792 1 0.208 0.002 0.545	1<br>Obrac01g17590.1 0.089 0.726 0.637 0.067 0.996<br>Obrac03g11330.1 0.792 1 0.208 0.03 1	0<br>Lperr05g03460.1 0.001 0.725 0.724 0.001 0.993	1<br>AT2G47580.1 0.778 1 0.222 0.008 1<br>AT3G16630.1 0.003 0.725 0.722 0.031 0.992
Obart05g25070.1	1<br>Osjap05g28230.1 0.001 1 0.999 0.001 1	1<br>Osind05g28520.1 0.001 1 0.999 0.001 1	1<br>Orufi05g26870.1 0.001 1 0.999 0.001 1	1<br>Oglab05g23160.1 0.001 1 0.999 0.001 1	1<br>Obart05g25070.1 0.001 1 0.999 0.001 1	0	1<br>Opunc05g22640.1 0.001 1 0.999 0.001 1	0	1<br>Lperr01g22990.1 0.001 1 0.999 0.027 1	0
	1<br>Osjap01g31140.1 0.007 0.451 0.444 0.093 0.995<br>Osjap01g31150.1 0.462 0.999 0.537 0.009 0.999	1<br>Osind01g32600.1 0.001 0.451 0.45 0.002 0.995<br>Osind01g32610.1 0.462 0.999 0.537 0.009 0.999	1<br>Orufi01g29880.1 0.001 0.451 0.45 0.075 0.995<br>Orufi01g29890.1 0.462 0.999 0.537 0.009 0.999	1<br>Oglab01g22740.1 0.028 0.451 0.423 0.061 0.995<br>Oglab01g22750.1 0.462 0.999 0.537 0.009 0.999	1<br>Obart01g26870.1 0.006 0.451 0.445 0.117 0.995<br>Obart01g26880.1 0.462 0.999 0.537 0.009 0.999	1<br>Olong_000367-R1 0.105 0.451 0.346 0.002 0.993<br>Olong_000368-R1 0.456 0.999 0.543 0.108 0.999	1<br>Opunc01g26820.1 0.049 0.451 0.402 0.002 0.994<br>Opunc01g26830.1 0.462 1 0.538 0.009 1	1<br>Obrac01g20400.1 0.046 0.451 0.405 0.004 0.994<br>Obrac05g17560.2 0.451 1 0.549 0.164 1	1<br>Lperr05g20850.2 0.467 1 0.533 0.002 1<br>Lperr05g20860.1 0.001 0.451 0.45 0.002 0.995	1<br>AT5G08690.1 0.012 0.451 0.439 0.034 0.995<br>AT5G51600.1 0.462 0.999 0.537 0.008 0.996
Obart06g00880.1	1<br>Osjap06g00940.1 0.04 1 0.96 0.085 1	0	1<br>Orufi06g00860.1 0.067 1 0.933 0.021 1	1<br>Oglab06g00840.1 0.001 1 0.999 0.001 1	1<br>Obart06g00880.1 0.001 1 0.999 0.001 1	0	0	0	0	0

	1<br>Osjap06g00950.1 0.529 0.992 0.463 0.132 0.99<br>Osjap08g13790.1 0.051 0.458 0.407 0.005 1	1<br>Osind06g00910.1 0.526 1 0.474 0.068 1<br>Osind08g15520.1 0.051 0.458 0.407 0.005 1	1<br>Orufi06g00690.1 0.529 1 0.471 0.099 1<br>Orufi06g00860.2 0.001 0.445 0.444 0.005 0.98	1<br>Oglab06g00730.1 0.526 0.99 0.464 0.257 0.968<br>Oglab07g19660.1 0.033 0.458 0.425 0.142 0.938	1<br>Obart06g00690.1 0.529 1 0.471 0.099 1<br>Obart09g03180.1 0.021 0.458 0.437 0.145 1	0<br>Olong_023831-R1 0.053 0.583 0.53 0.001 0.373	1<br>Opunc06g00800.1 0.457 1 0.543 0.025 1<br>Opunc06g00830.1 0.05 0.457 0.407 0.006 1	1<br>Obrac06g00660.1 0.526 1 0.474 0.034 0.999<br>Obrac07g14790.1 0.058 0.503 0.445 0.093 0.948	1<br>Lperr01g40030.1 0.055 0.457 0.402 0.013 0.994<br>Lperr06g00740.1 0.531 0.999 0.468 0.078 0.997	0<br>AT4G34470.1 0.063 0.457 0.394 0.013 1
Obart06g28060.1	1<br>Osjap09g17800.1 0.024 1 0.976 0.113 0.974<br>Osjap09g17800.2 0.024 1 0.976 0.114 0.974<br>Osjap09g17800.3 0.024 0.997 0.973 0.187 0.947	1<br>Osind06g31620.1 0.001 1 0.999 0.001 1	1<br>Orufi06g29990.1 0.001 1 0.999 0.001 1<br>Orufi09g18790.1 0.024 1 0.976 0.107 0.97<br>Orufi09g18790.2 0.024 1 0.976 0.107 0.97<br>Orufi09g18790.3 0.024 1 0.976 0.108 0.969<br>Orufi09g18790.4 0.024 1 0.976 0.108 0.969<br>Orufi09g18790.5 0.024 0.976 0.952 0.17 0.937<br>Orufi09g18790.6 0.024 0.996 0.972 0.172 0.937	1<br>Oglab06g25490.1 0.001 1 0.999 0.001 1<br>Oglab09g15660.1 0.024 0.999 0.975 0.154 0.95	1<br>Obart06g28060.1 0.001 1 0.999 0.001 1<br>Obart09g17460.1 0.024 1 0.976 0.076 0.969<br>Obart09g17460.2 0.024 1 0.976 0.076 0.969	1<br>Olong_016700-R1 0.001 1 0.999 0.001 1	0	0	1<br>Lperr09g13380.1 0.084 0.982 0.898 0.166 0.951	0
	1<br>Osjap06g32830.1 0.427 1 0.573 0.085 1<br>Osjap06g32840.1 0.001 0.407 0.406 0.002 0.952	1<br>Osind04g24640.1 0.07 0.417 0.347 0.233 0.933<br>Osind06g30840.1 0.485 0.999 0.514 0.109 1	0<br>Orufi06g29150.4 0.485 0.999 0.514 0.122 1	1<br>Oglab02g01250.1 0.066 0.406 0.34 0.221 0.93<br>Oglab10g06880.1 0.427 1 0.573 0.099 1	1<br>Obart04g21770.1 0.07 0.417 0.347 0.232 0.933<br>Obart10g07310.1 0.427 1 0.573 0.099 1	1<br>Olong_000286-R1 0.066 0.406 0.34 0.258 0.928<br>Olong_026973-R2 0.427 1 0.573 0.099 1	1<br>Opunc06g25610.1 0.001 0.407 0.406 0.002 0.951<br>Opunc10g06480.1 0.427 0.998 0.571 0.098 0.984	1<br>Obrac06g20010.2 0.432 1 0.568 0.119 1<br>Obrac06g20020.1 0.001 0.407 0.406 0.002 0.952	1<br>Lperr02g22230.1 0.043 0.392 0.349 0.113 0.88<br>Lperr06g23510.1 0.402 1 0.598 0.074 1	1<br>AT1G76360.1 0.036 0.406 0.37 0.219 0.952<br>AT3G61130.1 0.465 0.999 0.534 0.207 1
Obart07g11000.2	1<br>Osjap07g12650.1 0.001 1 0.999 0.094 1<br>Osjap07g12650.2 0.001 1 0.999 0.099 1<br>Osjap07g12650.3 0.001 1 0.999 0.102 1	1<br>Osind03g00080.1 0.001 1 0.999 0.001 1	1<br>Orufi07g11170.1 0.001 1 0.999 0.1 1<br>Orufi07g11170.2 0.001 1 0.999 0.096 1<br>Orufi07g11170.3 0.001 1 0.999 0.098 1<br>Orufi07g11170.4 0.001 1 0.999 0.101 1<br>Orufi07g11170.5 0.001 1 0.999 0.102 1	1<br>Oglab07g10660.1 0.001 0.923 0.922 0.1 0.995<br>Oglab07g10660.3 0.001 0.923 0.922 0.106 0.995<br>Oglab07g10660.4 0.001 0.923 0.922 0.109 0.995	1<br>Obart07g11000.1 0.001 1 0.999 0.001 1<br>Obart07g11000.2 0.001 1 0.999 0.001 1<br>Obart07g11000.3 0.001 1 0.999 0.001 1	1<br>Olong_020395-R1 0.001 1 0.999 0.001 1	0	0	0	0
	1<br>Osjap01g03910.2 0.027 0.717 0.69 0.126 0.736<br>Osjap09g19490.1 0.729 0.974 0.245 0.193 1	1<br>Osind08g00800.1 0.036 0.726 0.69 0.111 0.925<br>Osind09g19670.1 0.729 0.974 0.245 0.184 1	1<br>Orufi05g10490.3 0.028 0.707 0.679 0.103 0.96<br>Orufi09g20690.1 0.729 0.974 0.245 0.319 1	0<br>Oglab07g10660.2 0.001 0.745 0.744 0.128 0.998	1<br>Obart09g19100.1 0.729 0.974 0.245 0.319 1<br>Obart10g14260.1 0.002 0.716 0.714 0.13 0.992	1<br>Olong_002950-R1 0.002 0.711 0.709 0.027 0.982<br>Olong_014211-R1 0.729 0.941 0.212 0.332 0.998	0<br>Opunc06g16990.1 0.018 0.76 0.742 0.098 0.997	1<br>Obrac05g06630.4 0.008 0.713 0.705 0.114 0.869<br>Obrac09g12470.1 0.729 0.974 0.245 0.318 1	1<br>Lperr03g21150.4 0.008 0.713 0.705 0.06 0.973<br>Lperr09g14930.2 0.729 0.974 0.245 0.317 1	0<br>AT5G61990.1 0.001 0.744 0.743 0.044 0.999
Obart08g06070.1	1<br>Osjap08g06650.1 0.002 1 0.998 0.002 1	1<br>Osind08g06790.1 0.002 1 0.998 0.002 1	1<br>Orufi08g06840.1 0.002 1 0.998 0.002 1	1<br>Oglab08g05640.1 0.002 1 0.998 0.002 1	1<br>Obart08g06070.1 0.002 1 0.998 0.002 1	0	1<br>Opunc08g05850.1 0.047 1 0.953 0.068 1	0	0	0
	1<br>Osjap04g21220.1 0.047 0.275 0.228 0.21 0.936<br>Osjap09g20870.1 0.359 0.984 0.625 0.208 0.966	1<br>Osind04g20250.1 0.047 0.275 0.228 0.198 0.94<br>Osind09g20960.1 0.359 0.984 0.625 0.208 0.966	1<br>Orufi04g19250.2 0.047 0.275 0.228 0.21 0.936<br>Orufi09g21960.1 0.359 0.984 0.625 0.2 0.965	1<br>Oglab02g26360.1 0.348 0.996 0.648 0.01 0.994<br>Oglab04g16180.1 0.047 0.275 0.228 0.198 0.94	1<br>Obart02g28440.1 0.348 0.996 0.648 0.093 0.994<br>Obart04g17770.1 0.047 0.275 0.228 0.198 0.94	1<br>Olong_008030-R1 0.047 0.275 0.228 0.232 0.937<br>Olong_008031-R2 0.277 1 0.723 0.106 1	1<br>Opunc04g15550.1 0.047 0.275 0.228 0.206 0.938<br>Opunc04g18740.1 0.295 0.984 0.689 0.088 0.994	1<br>Obrac08g04780.1 0.277 1 0.723 0.098 1<br>Obrac08g04800.1 0.002 0.275 0.273 0.006 0.943	1<br>Lperr08g05690.1 0.277 1 0.723 0.1 1<br>Lperr08g05700.1 0.002 0.275 0.273 0.006 0.943	1<br>AT1G05310.1 0.33 0.982 0.652 0.183 0.99<br>AT4G30800.1 0.047 0.275 0.228 0.208 0.943
Obart08g16670.1	1<br>Osjap01g09050.1 0.007 0.844 0.837 0.061 1	1<br>Osind01g08880.1 0.102 0.986 0.884 0.004 0.567	1<br>Orufi01g08450.1 0.007 0.973 0.966 0.026 0.487	1<br>Oglab01g06790.1 0.007 0.844 0.837 0.061 1<br>Oglab08g15520.1 0.007 0.993 0.986 0.007 0.993	1<br>Obart01g07700.1 0.007 0.844 0.837 0.061 1<br>Obart08g16670.1 0.007 1 0.993 0.007 1	1<br>Olong_003647-R1 0.007 0.83 0.823 0.061 0.985	0	0	0	0
	1<br>Osjap01g24690.1 0.007 0.694 0.687 0.104 0.321<br>Osjap07g09900.1 0.694 0.993 0.299 0.005 0.123	1<br>Osind07g08950.1 0.694 0.993 0.299 0.004 0.111<br>Osind11g05650.1 0.007 0.694 0.687 0.103 0.319	1<br>Orufi01g23650.1 0.007 0.694 0.687 0.106 0.307<br>Orufi07g08630.1 0.694 0.993 0.299 0.005 0.115	1<br>Oglab07g08260.1 0.694 0.973 0.279 0.004 0.099<br>Oglab11g05440.1 0.007 0.694 0.687 0.573 0.683	1<br>Obart07g08560.1 0.694 0.952 0.258 0.009 0.192<br>Obart11g05910.1 0.007 0.694 0.687 0.752 0.834	1<br>Olong_005787-R1 0.007 0.694 0.687 0.103 0.319<br>Olong_016241-R1 0.728 0.932 0.204 0.735 0.768	1<br>Opunc01g21000.1 0.007 0.694 0.687 0.114 0.334<br>Opunc07g08090.1 0.694 0.993 0.299 0.004 0.09	1<br>Obrac07g05080.4 0.728 0.993 0.265 0.102 0.171<br>Obrac11g03720.1 0.007 0.694 0.687 0.112 0.34	1<br>Lperr01g17850.1 0.007 0.694 0.687 0.105 0.313<br>Lperr07g08590.1 0.694 0.993 0.299 0.022 0.124	1<br>AT3G55700.1 0.728 0.973 0.245 0.015 0.093<br>AT4G38130.1 0.007 0.68 0.673 0.09 0.269
Obart09g14980.1	1	1	1	1	1	0	1	1	0	0

	Osjap09g15430.2 0.002 1 0.998 0.002 1<br>Osjap09g15430.3 0.002 1 0.998 0.002 1	Osind09g15570.1 0.002 1 0.998 0.002 1<br>Osind09g15570.2 0.002 1 0.998 0.002 1	Orufi09g16160.1 0.002 1 0.998 0.002 1<br>Orufi09g16160.2 0.002 1 0.998 0.002 1	Oglab09g13510.1 0.002 1 0.998 0.002 1<br>Oglab09g13510.2 0.002 1 0.998 0.002 1<br>Oglab09g13510.3 0.002 1 0.998 0.002 1<br>Oglab09g13510.4 0.002 1 0.998 0.002 1	Obart09g14980.1 0.002 1 0.998 0.002 1<br>Obart09g14980.2 0.002 1 0.998 0.002 1		Opunc09g13420.1 0.002 1 0.998 0.002 1<br>Opunc09g13420.2 0.002 1 0.998 0.002 1	Obrac09g09090.1 0.002 1 0.998 0.002 1		
	1<br>Osjap02g06650.1 0.01 0.565 0.555 0.01 0.997<br>Osjap09g15430.1 0.569 1 0.431 0.131 1	1<br>Osind02g06940.1 0.01 0.565 0.555 0.01 0.997<br>Osind08g23920.1 0.569 0.993 0.424 0.131 0.988	1<br>Orufi04g24220.1 0.014 0.567 0.553 0.165 1<br>Orufi08g22880.1 0.569 0.993 0.424 0.131 0.988	1<br>Oglab08g19240.1 0.569 0.993 0.424 0.143 0.986<br>Oglab12g18680.1 0.002 0.567 0.565 0.003 1	1<br>Obart02g06180.1 0.01 0.565 0.555 0.01 0.997<br>Obart08g20390.1 0.569 0.993 0.424 0.631 0.988	1<br>Olong_008093-R2 0.596 0.991 0.395 0.25 0.996<br>Olong_024186-R1 0.002 0.567 0.565 0.003 1	1<br>Opunc04g20190.1 0.007 0.567 0.56 0.006 1<br>Opunc08g18560.1 0.569 0.993 0.424 0.128 0.969	1<br>Obrac01g09090.1 0.605 0.998 0.393 0.299 0.982<br>Obrac02g04550.3 0.009 0.565 0.556 0.214 0.997	1<br>Lperr04g18350.1 0.002 0.567 0.565 0.021 1<br>Lperr09g11120.1 0.569 1 0.431 0.156 1	1<br>AT1G68540.1 0.567 0.991 0.424 0.112 1<br>AT2G21270.3 0.003 0.565 0.562 0.059 0.997
Obart09g14980.2	1<br>Osjap09g15430.2 0.002 1 0.998 0.002 1<br>Osjap09g15430.3 0.002 1 0.998 0.002 1	1<br>Osind09g15570.1 0.002 1 0.998 0.002 1<br>Osind09g15570.2 0.002 1 0.998 0.002 1	1<br>Orufi09g16160.1 0.002 1 0.998 0.002 1<br>Orufi09g16160.2 0.002 1 0.998 0.002 1	1<br>Oglab09g13510.1 0.002 1 0.998 0.002 1<br>Oglab09g13510.2 0.002 1 0.998 0.002 1<br>Oglab09g13510.3 0.002 1 0.998 0.002 1<br>Oglab09g13510.4 0.002 1 0.998 0.002 1	1<br>Obart09g14980.1 0.002 1 0.998 0.002 1<br>Obart09g14980.2 0.002 1 0.998 0.002 1	0	1<br>Opunc09g13420.1 0.002 1 0.998 0.002 1<br>Opunc09g13420.2 0.002 1 0.998 0.002 1	1<br>Obrac09g09090.1 0.002 1 0.998 0.002 1	0	0
	1<br>Osjap02g06650.1 0.01 0.54 0.53 0.01 0.997<br>Osjap09g15430.1 0.544 1 0.456 0.131 1	1<br>Osind02g06940.1 0.01 0.54 0.53 0.01 0.997<br>Osind08g23920.1 0.544 0.993 0.449 0.131 0.988	1<br>Orufi04g24220.1 0.013 0.542 0.529 0.165 1<br>Orufi08g22880.1 0.544 0.993 0.449 0.131 0.988	1<br>Oglab08g19240.1 0.544 0.993 0.449 0.143 0.986<br>Oglab12g18680.1 0.002 0.542 0.54 0.003 1	1<br>Obart02g06180.1 0.01 0.54 0.53 0.01 0.997<br>Obart08g20390.1 0.544 0.993 0.449 0.631 0.988	1<br>Olong_013054-R1 0.547 0.992 0.445 0.149 0.979<br>Olong_024186-R1 0.002 0.542 0.54 0.003 1	1<br>Opunc04g20190.1 0.007 0.542 0.535 0.006 1<br>Opunc08g18560.1 0.544 0.993 0.449 0.128 0.969	1<br>Obrac02g04550.3 0.008 0.54 0.532 0.214 0.997<br>Obrac09g00560.1 0.548 0.998 0.45 0.164 0.966	1<br>Lperr04g18350.1 0.002 0.542 0.54 0.021 1<br>Lperr09g11120.1 0.544 1 0.456 0.156 1	1<br>AT1G68540.1 0.542 0.992 0.45 0.112 1<br>AT2G21270.3 0.003 0.54 0.537 0.059 0.997
Obart09g15360.1	0	1<br>Osind09g15930.1 0.001 1 0.999 0.057 1	1<br>Orufi09g16580.1 0.001 1 0.999 0.114 1	1<br>Oglab09g13920.1 0.001 1 0.999 0.114 1	1<br>Obart09g15360.1 0.001 1 0.999 0.001 1	0	0	0	0	0
	1<br>Osjap03g04840.3 0.507 0.883 0.376 0.018 0.769<br>Osjap09g15790.1 0.001 0.507 0.506 0.238 0.993	1<br>Osind08g24670.1 0.001 0.507 0.506 0.221 0.995<br>Osind09g02290.1 0.507 0.883 0.376 0.018 0.769	1<br>Orufi03g04550.5 0.507 0.883 0.376 0.017 0.733<br>Orufi08g23480.1 0.001 0.507 0.506 0.228 0.995	1<br>Oglab02g15910.1 0.003 0.474 0.471 0.048 0.988<br>Oglab09g02290.1 0.507 0.883 0.376 0.02 0.789	1<br>Obart03g04820.3 0.535 0.883 0.348 0.001 0.781<br>Obart08g21020.1 0.001 0.507 0.506 0.088 0.994	1<br>Olong_025005-R2 0.535 0.979 0.444 0.001 0.955<br>Olong_025006-R1 0.05 0.507 0.457 0.031 0.99	1<br>Opunc08g19030.2 0.001 0.507 0.506 0.156 0.976<br>Opunc09g13790.1 0.507 0.979 0.472 0.055 0.958	0<br>Obrac08g14010.2 0.001 0.508 0.507 0.044 0.989	1<br>Lperr08g17810.1 0.001 0.507 0.506 0.122 0.994<br>Lperr09g11510.2 0.507 0.979 0.472 0.01 0.958	1<br>AT2G32000.1 0.507 0.978 0.471 0.012 0.961<br>AT2G43900.1 0.003 0.481 0.478 0.165 0.843
Obart10g19720.1	0	1<br>Osind10g19230.1 0.002 1 0.998 0.002 1	1<br>Orufi10g20960.1 0.002 1 0.998 0.002 1	1<br>Oglab10g16090.1 0.002 0.992 0.99 0.001 0.861	1<br>Obart10g19720.1 0.002 1 0.998 0.002 1	0	1<br>Opunc10g18180.1 0.002 0.992 0.99 0.002 0.85	0	0	0
	1<br>Osjap10g22750.1 0.473 0.992 0.519 0.061 0.836<br>Osjap10g22760.1 0.002 0.451 0.449 0.003 0.993	1<br>Osind01g20890.1 0.483 0.992 0.509 0.154 0.668<br>Osind08g03990.1 0.042 0.431 0.389 0.057 0.95	1<br>Orufi01g19490.1 0.483 0.992 0.509 0.171 0.72<br>Orufi08g04140.1 0.042 0.431 0.389 0.057 0.95	1<br>Oglab01g14760.1 0.483 0.992 0.509 0.2 0.827<br>Oglab08g03250.2 0.042 0.431 0.389 0.052 0.955	1<br>Obart01g17550.1 0.483 0.992 0.509 0.174 0.721<br>Obart08g03750.1 0.042 0.431 0.389 0.057 0.95	1<br>Olong_006312-R1 0.48 0.987 0.507 0.067 0.835<br>Olong_006313-R1 0.002 0.451 0.449 0.003 0.993	1<br>Opunc01g18040.1 0.483 0.992 0.509 0.174 0.822<br>Opunc08g03700.1 0.016 0.431 0.415 0.011 0.951	1<br>Obrac01g13330.1 0.487 0.992 0.505 0.196 0.825<br>Obrac10g13120.1 0.002 0.451 0.449 0.004 0.993	1<br>Lperr10g15590.1 0.48 0.992 0.512 0.07 0.79<br>Lperr10g15600.1 0.002 0.448 0.446 0.003 0.979	1<br>AT1G32080.1 0.484 0.989 0.505 0.188 0.82<br>AT2G32260.1 0.041 0.432 0.391 0.078 0.825
Obart11g01060.1	1<br>Osjap11g01220.1 0.001 1 0.999 0.001 1<br>Osjap12g01140.1 0.001 1 0.999 0.001 1	1<br>Osind11g00690.1 0.001 0.954 0.953 0.001 0.999<br>Osind12g01290.1 0.001 1 0.999 0.001 1	1<br>Orufi11g01000.1 0.001 1 0.999 0.001 1<br>Orufi12g01130.1 0.001 1 0.999 0.001 1	1<br>Oglab12g00800.1 0.001 1 0.999 0.001 1	1<br>Obart11g01060.1 0.001 1 0.999 0.001 1	0	1<br>Opunc12g00940.1 0.001 1 0.999 0.001 1	0	0	0
	1<br>Osjap03g13100.1 0.479 0.982 0.503 0.097 0.622<br>Osjap07g30390.1 0.01 0.42 0.41 0.013 0.944	1<br>Osind03g12930.1 0.479 0.982 0.503 0.121 0.633<br>Osind07g27070.1 0.007 0.42 0.413 0.006 0.946	1<br>Orufi03g17220.1 0.485 1 0.515 0.027 1<br>Orufi07g27420.1 0.01 0.42 0.41 0.013 0.946	1<br>Oglab03g12120.1 0.479 0.982 0.503 0.121 0.633<br>Oglab07g22980.1 0.007 0.42 0.413 0.007 0.94	1<br>Obart03g12120.1 0.479 0.982 0.503 0.121 0.633<br>Obart12g00790.1 0.001 0.42 0.419 0.002 0.949	1<br>Olong_007715-R1 0.482 1 0.518 0.007 1<br>Olong_008211-R1 0.007 0.42 0.413 0.006 0.946	1<br>Opunc03g11680.1 0.479 1 0.521 0.148 1<br>Opunc11g00920.1 0.001 0.42 0.419 0.142 0.951	1<br>Obrac03g09720.2 0.479 1 0.521 0.142 1<br>Obrac12g00780.1 0.001 0.42 0.419 0.11 0.953	1<br>Lperr03g10720.1 0.479 0.996 0.517 0.148 0.954<br>Lperr11g00920.1 0.001 0.42 0.419 0.002 0.949	1<br>AT3G46740.1 0.482 1 0.518 0.161 1<br>AT4G20110.2 0.001 0.42 0.419 0.002 0.946
Obart12g00450.1	1<br>Osjap11g00780.1 0.001 1 0.999 0.003 0.978<br>Osjap12g00740.1 0.001 1 0.999 0.004 1	1<br>Osind05g13250.1 0.056 0.952 0.896 0.062 1<br>Osind05g13250.2 0.073 0.958 0.885 0.06 1	1<br>Orufi05g11850.1 0.073 0.958 0.885 0.034 1<br>Orufi05g11850.2 0.056 0.952 0.896 0.062 1<br>Orufi05g11850.3 0.073 0.958 0.885 0.06 1	1<br>Oglab05g10210.1 0.056 0.952 0.896 0.034 1<br>Oglab05g10210.2 0.073 0.958 0.885 0.034 1<br>Oglab05g10210.3 0.056 0.952 0.896 0.062 1	1<br>Obart05g10980.1 0.056 0.952 0.896 0.001 0.979<br>Obart12g00450.1 0.001 1 0.999 0.001 1	0	0	0	1<br>Lperr12g00660.1 0.001 0.995 0.994 0.009 0.943	0

				Oglab05g10210.4 0.073 0.958 0.885 0.06 1<br>Oglab11g00720.1 0.001 1 0.999 0.001 0.973						
	0<br>Osjap11g00780.4 0.334 1 0.666 0.024 0.939	1<br>Osind12g00970.1 0.338 1 0.662 0.059 1<br>Osind12g00980.1 0.001 0.311 0.31 0.005 0.988	1<br>Orufi11g00570.3 0.334 1 0.666 0.042 0.894<br>Orufi11g00580.5 0.073 0.308 0.235 0.001 0.959	1<br>Oglab02g19870.1 0.394 0.632 0.238 0.15 0.925<br>Oglab12g00450.1 0.633 1 0.367 0.003 1<br>Oglab12g00460.1 0.001 0.308 0.307 0.006 0.981	1<br>Obart11g00640.1 0.413 1 0.587 0.113 0.896<br>Obart11g00650.1 0.073 0.309 0.236 0.001 0.999	0<br>Olong_019743-R1 0.538 0.958 0.42 0.002 0.981	1<br>Opunc11g00610.1 0.001 0.311 0.31 0.343 0.999<br>Opunc12g00660.2 0.338 1 0.662 0.057 0.892	0<br>Obrac12g01610.1 0.363 0.647 0.284 0.04 0.995	1<br>Lperr11g00650.1 0.001 0.309 0.308 0.01 0.999<br>Lperr12g00660.4 0.338 0.995 0.657 0.061 0.902	0<br>AT1G09700.1 0.337 0.851 0.514 0.014 0.477<br>AT5G01770.1 0.042 0.308 0.266 0.046 0.998
Obart12g02080.1	1<br>Osjap11g02640.1 0.002 1 0.998 0.024 1<br>Osjap12g02610.1 0.002 1 0.998 0.022 1	1<br>Osind11g01990.1 0.002 1 0.998 0.023 1<br>Osind12g02780.1 0.002 1 0.998 0.022 1	1<br>Orufi11g02320.1 0.002 1 0.998 0.003 1<br>Orufi12g02480.1 0.002 1 0.998 0.021 1	1<br>Oglab11g02200.1 0.002 1 0.998 0.023 1<br>Oglab12g02130.1 0.002 1 0.998 0.022 1	1<br>Obart12g02080.1 0.002 1 0.998 0.002 1	0	0	0	0	0
	1<br>Osjap12g02600.1 0.002 0.64 0.638 0.006 1<br>Osjap12g12870.1 0.644 0.948 0.304 0.055 0.417	1<br>Osind11g01980.1 0.002 0.64 0.638 0.006 1<br>Osind12g10020.1 0.644 0.948 0.304 0.055 0.417	1<br>Orufi12g02470.1 0.002 0.64 0.638 0.006 1<br>Orufi12g10750.1 0.642 0.948 0.306 0.045 0.364	1<br>Oglab12g02120.1 0.002 0.64 0.638 0.006 1<br>Oglab12g09880.1 0.644 0.948 0.304 0.051 0.385	0<br>Obart11g02350.1 0.002 0.64 0.638 0.004 0.993	1<br>Olong_003435-R1 0.133 0.64 0.507 0.212 0.984<br>Olong_023179-R1 0.642 0.998 0.356 0.05 0.329	1<br>Opunc11g02180.1 0.002 0.64 0.638 0.028 0.992<br>Opunc12g08740.1 0.642 0.948 0.306 0.04 0.324	0<br>Obrac05g10860.1 0.072 0.523 0.451 0.097 0.99<br>Obrac12g07170.1 0.642 0.974 0.332 0.071 0.67	1<br>Lperr12g02190.1 0.002 0.64 0.638 0.033 0.992<br>Lperr12g08390.1 0.642 0.956 0.314 0.056 0.454	0<br>AT3G27320.1 0.052 0.421 0.369 0.596 0.978
Obart12g02950.1	0	1<br>Osind12g03620.1 0.001 0.999 0.998 0.029 0.961	1<br>Orufi12g03350.1 0.011 0.999 0.988 0.044 0.966<br>Orufi12g03350.2 0.011 0.999 0.988 0.043 0.966	1<br>Oglab12g03070.1 0.001 1 0.999 0.001 1	1<br>Obart12g02950.1 0.001 1 0.999 0.001 1	1<br>Olong_000549-R1 0.011 0.999 0.988 0.011 0.967	0	0	0	0
	1<br>Osjap10g20010.1 0.498 0.991 0.493 0.022 0.946<br>Osjap12g02500.1 0.002 0.453 0.451 0.001 0.388	1<br>Osind01g01580.1 0.492 0.985 0.493 0.171 0.96<br>Osind12g02670.1 0.002 0.453 0.451 0.001 0.396	1<br>Orufi04g25720.1 0.498 0.871 0.373 0.002 0.207<br>Orufi11g02210.1 0.015 0.453 0.438 0.015 0.492	1<br>Oglab10g13340.3 0.559 0.991 0.432 0.213 0.951<br>Oglab12g02020.1 0.002 0.453 0.451 0.001 0.388	0<br>Obart11g02240.1 0.193 0.453 0.26 0.014 0.272<br>Obart11g21270.1 0.494 0.985 0.491 0.138 0.958	0<br>Olong_010277-R4 0.488 0.871 0.383 0.001 0.93<br>Olong_027241-R1 0.046 0.305 0.259 0.003 0.982	1<br>Opunc11g17890.2 0.491 0.985 0.494 0.189 0.952<br>Opunc12g02040.1 0.012 0.453 0.441 0.009 0.402	1<br>Obrac11g01490.1 0.002 0.453 0.451 0.029 0.403<br>Obrac11g12600.2 0.547 0.985 0.438 0.046 0.748	0<br>Lperr11g01950.1 0.011 0.228 0.217 0.015 0.297<br>Lperr11g17840.1 0.5 0.985 0.485 0.046 0.945	1<br>AT1G65810.1 0.498 0.871 0.373 0.021 0.596<br>AT2G26780.1 0.017 0.449 0.432 0.011 0.388
Obart12g09410.1	1<br>Osjap12g12320.1 0.001 1 0.999 0.001 1	1<br>Osind12g09530.1 0.001 1 0.999 0.001 1	1<br>Orufi12g10460.1 0.001 1 0.999 0.001 1	1<br>Oglab12g09470.1 0.001 1 0.999 0.001 1	1<br>Obart12g09410.1 0.001 1 0.999 0.001 1	0	0	0	0	0
	1<br>Osjap01g44780.3 0.452 0.998 0.546 0.017 0.994<br>Osjap03g07600.1 0.001 0.306 0.305 0.004 0.996	1<br>Osind01g46040.1 0.452 0.998 0.546 0.186 0.995<br>Osind03g07670.1 0.001 0.306 0.305 0.004 0.996	1<br>Orufi01g42650.3 0.452 0.998 0.546 0.228 0.995<br>Orufi03g07180.1 0.001 0.306 0.305 0.004 0.996	1<br>Oglab01g34040.1 0.477 0.998 0.521 0.203 0.995<br>Oglab03g07280.1 0.001 0.306 0.305 0.004 0.996	1<br>Obart01g39260.2 0.452 0.998 0.546 0.017 0.994<br>Obart03g07210.1 0.001 0.306 0.305 0.004 0.996	1<br>Olong_021912-R1 0.376 1 0.624 0.002 1<br>Olong_021913-R1 0.001 0.305 0.304 0.004 0.996	1<br>Opunc12g08570.1 0.406 1 0.594 0.152 1<br>Opunc12g08580.1 0.001 0.305 0.304 0.004 0.996	1<br>Obrac12g06490.1 0.001 0.305 0.304 0.004 0.996<br>Obrac12g06500.1 0.449 1 0.551 0.103 1	1<br>Lperr03g06600.1 0.001 0.304 0.303 0.004 0.992<br>Lperr12g08310.1 0.452 0.999 0.547 0.002 0.998	1<br>AT4G34670.1 0.001 0.297 0.296 0.004 0.966<br>AT5G67540.2 0.477 0.993 0.516 0.053 1

((((_d1,punctata),brachy),leersia),tair);

chimeric gene id	INGROUP						MIDGROUP	OUTGROUP		
	jap	indica	rufi	glab	barthii	longi	punctata	brachy	leersia	tair
Obart01g07430.1	1<br>Osjap01g08660.1 0.007 1 0.993 0.026 1	1<br>Osind01g08490.1 0.007 1 0.993 0.026 1	1<br>Orufi01g08070.1 0.007 1 0.993 0.026 1	1<br>Oglab01g06470.1 0.013 1 0.987 0.02 1	1<br>Obart01g07430.1 0.001 1 0.999 0.001 1	1<br>Olong_011567-R1 0.036 1 0.964 0.001 1	0	0	0	0
	1<br>Osjap01g29230.2 0.078 0.648 0.57 0.25 0.983<br>Osjap05g00560.2 0.713 0.997 0.284 0.146 1	1<br>Osind01g30690.1 0.078 0.648 0.57 0.267 0.982<br>Osind05g00540.1 0.713 0.997 0.284 0.126 1	1<br>Orufi01g27950.1 0.078 0.648 0.57 0.25 0.983<br>Orufi01g48560.2 0.713 0.983 0.27 0.085 0.984	1<br>Oglab01g21070.2 0.078 0.648 0.57 0.249 0.983<br>Oglab05g00530.1 0.713 0.997 0.284 0.146 1	1<br>Obart01g25090.1 0.078 0.648 0.57 0.219 0.982<br>Obart05g00540.1 0.713 0.997 0.284 0.146 1	1<br>Olong_003039-R1 0.713 0.997 0.284 0.128 1<br>Olong_003692-R2 0.109 0.648 0.539 0.244 0.979	1<br>Opunc01g07160.1 0.656 1 0.344 0.111 1<br>Opunc01g07170.1 0.003 0.649 0.646 0.034 0.984	1<br>Obrac01g05760.3 0.66 1 0.34 0.082 1<br>Obrac01g18840.2 0.001 0.648 0.647 0.062 0.974	1<br>Lperr01g07030.1 0.66 1 0.34 0.018 1<br>Lperr01g07040.2 0.002 0.649 0.647 0.013 0.989	1<br>AT1G54350.1 0.659 0.994 0.335 0.076 0.975<br>AT3G61740.1 0.009 0.648 0.639 0.077 0.983
Obart02g23460.2	1<br>Osjap02g26750.1 0.001 0.969 0.968 0.001 0.994	1<br>Osind02g26640.2 0.001 1 0.999 0.001 1	1<br>Orufi02g24900.1 0.011 0.974 0.963 0.06 0.923	1<br>Oglab02g22130.2 0.001 1 0.999 0.001 1	1<br>Obart02g23460.2 0.001 1 0.999 0.001 1	0	1<br>Opunc02g21410.1 0.011 0.999 0.988 0.116 0.996	0	1<br>Lperr02g18970.1 0.011 0.997 0.986 0.032 0.996	0

	Osjap02g26750.2 0.001 0.969 0.968 0.001 0.994<br>Osjap02g26750.4 0.001 0.969 0.968 0.001 0.799		Orufi02g24900.3 0.001 0.974 0.973 0.001 0.857<br>Orufi02g24900.4 0.001 0.974 0.973 0.001 0.856				Opunc02g21410.2 0.011 0.999 0.988 0.114 0.996<br>Opunc02g21410.3 0.011 0.999 0.988 0.115 0.996<br>Opunc02g21410.4 0.011 0.999 0.988 0.114 0.996			
	1<br>Osjap02g26590.1 0.475 0.999 0.524 0.033 0.999<br>Osjap02g26750.5 0.011 0.459 0.448 0.238 0.973	1<br>Osind02g26450.1 0.475 0.999 0.524 0.025 0.997<br>Osind02g26620.1 0.011 0.459 0.448 0.495 0.981	1<br>Orufi02g24730.1 0.475 0.994 0.519 0.031 0.996<br>Orufi02g24880.2 0.011 0.459 0.448 0.18 0.97	1<br>Oglab02g21950.1 0.475 0.999 0.524 0.073 0.995<br>Oglab02g22110.2 0.011 0.459 0.448 0.532 0.98	1<br>Obart02g23290.1 0.475 0.999 0.524 0.029 0.999<br>Obart02g23440.1 0.011 0.459 0.448 0.492 0.981	0<br>Olong_024345-R1 0.011 0.474 0.463 0.442 0.991	1<br>Opunc02g21230.1 0.479 0.976 0.497 0.003 0.934<br>Opunc02g21240.1 0.011 0.461 0.45 0.121 0.972	1<br>Obrac02g16670.1 0.549 0.973 0.424 0.076 0.995<br>Obrac02g16700.1 0.011 0.461 0.45 0.114 0.947	1<br>Lperr02g18740.1 0.485 0.997 0.512 0.056 0.991<br>Lperr02g19120.1 0.011 0.484 0.473 0.183 0.978	1<br>AT4G31860.1 0.014 0.45 0.436 0.123 0.989<br>AT5G43080.1 0.626 1 0.374 0.194 0.932
Obart03g00550.1	1<br>Osjap03g00680.1 0.001 0.996 0.995 0.001 0.994<br>Osjap03g00680.2 0.001 0.996 0.995 0.001 0.994<br>Osjap03g00680.3 0.001 0.996 0.995 0.001 0.994	1<br>Osind03g00740.1 0.001 1 0.999 0.001 1	1<br>Orufi03g12660.1 0.001 0.996 0.995 0.001 0.994<br>Orufi03g12660.2 0.001 0.996 0.995 0.001 0.994	1<br>Oglab03g00390.1 0.001 0.996 0.995 0.001 0.994	1<br>Obart03g00550.1 0.001 1 0.999 0.001 1	0	0	0	0	0
	1<br>Osjap02g42090.1 0.052 0.675 0.623 0.065 1<br>Osjap12g08580.1 0.706 0.996 0.29 0.011 0.977	1<br>Osind02g41670.1 0.061 0.675 0.614 0.131 1<br>Osind03g02220.1 0.695 0.996 0.301 0.015 0.982	1<br>Orufi02g39350.1 0.061 0.675 0.614 0.131 1<br>Orufi09g02220.2 0.742 0.996 0.254 0.095 0.975	1<br>Oglab02g34920.1 0.061 0.675 0.614 0.131 1<br>Oglab04g12740.1 0.715 0.996 0.281 0.411 0.989	1<br>Obart02g37790.1 0.061 0.645 0.584 0.133 0.977<br>Obart12g06510.1 0.673 0.996 0.323 0.052 0.984	0<br>Olong_013175-R1 0.27 0.904 0.634 0.002 0.961	1<br>Opunc03g00470.1 0.001 0.78 0.779 0.002 1<br>Opunc03g03130.2 0.78 0.996 0.216 0.443 0.984	0<br>Obrac02g27970.1 0.136 0.675 0.539 0.105 1<br>Obrac05g17660.1 0.747 0.995 0.248 0.427 0.997	1<br>Lperr03g00570.1 0.001 0.675 0.674 0.002 1<br>Lperr04g11360.1 0.748 0.996 0.248 0.415 0.99	0<br>AT5G04460.1 0.018 0.675 0.657 0.081 1
Obart03g20630.1	1<br>Osjap03g22210.1 0.009 0.991 0.982 0.567 0.972	1<br>Osind03g22510.1 0.017 1 0.983 0.552 1	1<br>Orufi03g21250.1 0.185 0.995 0.81 0.02 0.991<br>Orufi03g21250.2 0.185 0.995 0.81 0.019 0.991<br>Orufi03g21250.3 0.189 0.995 0.806 0.021 0.99<br>Orufi03g21250.4 0.185 0.995 0.81 0.021 0.99	1<br>Oglab03g20370.1 0.073 1 0.927 0.087 1	1<br>Obart03g20630.1 0.001 1 0.999 0.001 1	0	0	0	0	0
	1<br>Osjap05g27870.1 0.044 0.547 0.503 0.075 0.982<br>Osjap12g12160.2 0.556 0.931 0.375 0.083 0.997	1<br>Osind02g20400.1 0.607 0.931 0.324 0.137 0.985<br>Osind05g28150.1 0.044 0.547 0.503 0.075 0.982	1<br>Orufi05g26490.1 0.044 0.553 0.509 0.075 0.989<br>Orufi09g01530.2 0.557 0.931 0.374 0.087 0.965	1<br>Oglab05g22780.1 0.044 0.547 0.503 0.077 0.982<br>Oglab08g00090.1 0.557 0.931 0.374 0.117 0.976	1<br>Obart06g03480.2 0.051 0.556 0.505 0.002 0.826<br>Obart09g16880.1 0.557 0.931 0.374 0.091 0.964	1<br>Olong_013421-R1 0.051 0.556 0.505 0.002 0.877<br>Olong_028288-R1 0.607 1 0.393 0.003 1	1<br>Opunc05g00870.2 0.567 0.996 0.429 0.145 0.973<br>Opunc05g22250.1 0.044 0.547 0.503 0.075 0.982	0<br>Obrac09g04370.1 0.08 0.572 0.492 0.034 0.858	0<br>Lperr05g06040.4 0.557 0.992 0.435 0.084 0.556	0<br>AT1G07740.1 0.025 0.531 0.506 0.083 0.967
Obart03g31750.1	1<br>Osjap03g34330.1 0.046 1 0.954 0.001 1	1<br>Osind03g35550.1 0.046 1 0.954 0.039 1	1<br>Orufi03g33060.1 0.046 1 0.954 0.001 1	1<br>Oglab03g30830.1 0.046 1 0.954 0.001 1	1<br>Obart03g31750.1 0.001 1 0.999 0.001 1	0	0	0	0	0
	1<br>Osjap03g39110.1 0.667 1 0.333 0.036 1<br>Osjap10g05370.1 0.034 0.602 0.568 0.051 0.963	1<br>Osind01g46540.1 0.667 0.999 0.332 0.065 0.994<br>Osind10g03770.1 0.034 0.602 0.568 0.057 0.96	1<br>Orufi10g04850.1 0.034 0.602 0.568 0.054 0.963<br>Orufi11g12520.1 0.668 1 0.332 0.038 0.991	1<br>Oglab03g34460.1 0.646 0.997 0.351 0.016 0.993<br>Oglab10g04340.1 0.028 0.602 0.574 0.058 0.963	1<br>Obart03g36200.1 0.667 1 0.333 0.016 0.997<br>Obart10g04600.1 0.058 0.602 0.544 0.106 0.962	1<br>Olong_014796-R1 0.046 0.518 0.472 0.002 0.95<br>Olong_024872-R1 0.664 1 0.336 0.01 1	1<br>Opunc01g42030.1 0.667 1 0.333 0.28 0.872<br>Opunc03g28940.1 0.041 0.62 0.579 0.049 0.998	1<br>Obrac02g16280.1 0.662 0.982 0.32 0.063 0.949<br>Obrac03g23440.1 0.046 0.62 0.574 0.002 0.998	1<br>Lperr03g30520.1 0.66 1 0.34 0.003 0.997<br>Lperr10g03360.1 0.013 0.602 0.589 0.009 0.962	0<br>AT1G70730.3 0.046 0.618 0.572 0.118 0.995
Obart05g00520.1	1<br>Osjap05g00530.1 0.001 0.993 0.992 0.084 0.999<br>Osjap05g00530.2 0.001 0.993 0.992 0.103 0.999<br>Osjap05g00530.3 0.001 0.993 0.992 0.104 0.999<br>Osjap05g00530.4 0.001 0.993 0.992 0.091 0.999	1<br>Osind05g00510.1 0.049 0.993 0.944 0.001 0.999	1<br>Orufi05g00500.1 0.001 0.993 0.992 0.084 0.999	0	1<br>Obart05g00520.1 0.001 1 0.999 0.001 1<br>Obart05g00520.2 0.001 0.995 0.994 0.001 0.999	1<br>Olong_022405-R1 0.049 0.993 0.944 0.001 0.999	0	0	0	0
	1<br>Osjap02g39750.1 0.337 0.988 0.651 0.004 0.956<br>Osjap07g27300.1 0.003 0.291 0.288 0.116 0.834	1<br>Osind09g15830.1 0.34 0.993 0.653 0.029 0.998<br>Osind10g10530.3 0.001 0.336 0.335 0.088 0.826	1<br>Orufi07g24760.1 0.003 0.291 0.288 0.116 0.834<br>Orufi09g16480.1 0.34 0.993 0.653 0.029 0.998	1<br>Oglab05g00500.1 0.001 0.294 0.293 0.218 0.982<br>Oglab09g13820.1 0.322 0.993 0.671 0.064 0.998	1<br>Obart06g01490.1 0.017 0.293 0.276 0.2 0.982<br>Obart09g15270.1 0.34 0.993 0.653 0.029 0.998	1<br>Olong_018361-R1 0.34 0.993 0.653 0.012 0.998<br>Olong_020432-R1 0.017 0.325 0.308 0.115 0.666	1<br>Opunc05g00480.1 0.329 0.993 0.664 0.007 0.998<br>Opunc05g00490.1 0.001 0.294 0.293 0.228 0.982	1<br>Obrac02g14170.4 0.001 0.3 0.299 0.03 0.843<br>Obrac02g28140.1 0.342 0.989 0.647 0.039 0.973	1<br>Lperr05g00500.1 0.331 0.993 0.662 0.024 0.994<br>Lperr10g08300.1 0.001 0.291 0.29 0.117 0.971	0<br>AT5G44230.1 0.001 0.298 0.297 0.155 0.872
Obart05g00520.2	1	1	1	0	1	1	0	0	0	0

	Osjap05g00530.1 0.001 1 0.999 0.084 1<br>Osjap05g00530.2 0.001 1 0.999 0.103 1<br>Osjap05g00530.3 0.001 1 0.999 0.104 1<br>Osjap05g00530.4 0.001 1 0.999 0.091 1	Osind05g00510.1 0.049 1 0.951 0.001 1	Orufi05g00500.1 0.001 1 0.999 0.084 1		Obart05g00520.1 0.001 0.999 0.998 0.001 0.995<br>Obart05g00520.2 0.001 1 0.999 0.001 1	Olong_022405-R1 0.049 1 0.951 0.001 1				
	1<br>Osjap02g39750.1 0.339 0.994 0.655 0.004 0.956<br>Osjap07g27300.1 0.003 0.293 0.29 0.116 0.834	1<br>Osind09g15830.1 0.342 1 0.658 0.029 1<br>Osind10g10530.1 0.001 0.293 0.292 0.108 0.858	1<br>Orufi07g24760.1 0.003 0.293 0.29 0.116 0.834<br>Orufi09g16480.1 0.342 1 0.658 0.029 1	1<br>Oglab05g00500.1 0.001 0.295 0.294 0.218 0.982<br>Oglab09g13820.1 0.324 1 0.676 0.064 1	1<br>Obart06g01490.1 0.017 0.295 0.278 0.2 0.982<br>Obart09g15270.1 0.342 1 0.658 0.029 1	1<br>Olong_018361-R1 0.342 1 0.658 0.012 1<br>Olong_020432-R1 0.017 0.327 0.31 0.115 0.666	1<br>Opunc05g00480.1 0.331 0.999 0.668 0.007 0.998<br>Opunc05g00490.1 0.001 0.295 0.294 0.228 0.982	1<br>Obrac02g14170.4 0.001 0.302 0.301 0.03 0.843<br>Obrac02g28140.1 0.344 0.995 0.651 0.039 0.973	1<br>Lperr05g00500.1 0.334 1 0.666 0.024 0.996<br>Lperr10g08300.1 0.001 0.293 0.292 0.117 0.971	0<br>AT5G44230.1 0.001 0.3 0.299 0.155 0.872
Obart05g03830.1	1<br>Osjap05g04120.1 0.001 1 0.999 0.001 1<br>Osjap05g04120.2 0.001 1 0.999 0.001 1	1<br>Osind05g04070.1 0.008 1 0.992 0.001 1<br>Osind05g04070.2 0.008 1 0.992 0.001 1	1<br>Orufi05g03950.1 0.001 1 0.999 0.001 1<br>Orufi05g03950.2 0.001 1 0.999 0.001 1	1<br>Oglab05g03490.1 0.001 1 0.999 0.001 1<br>Oglab05g03490.2 0.001 1 0.999 0.001 1	1<br>Obart05g03830.1 0.001 1 0.999 0.001 1<br>Obart05g03830.2 0.001 1 0.999 0.001 1	1<br>Olong_016739-R1 0.074 1 0.926 0.001 1	1<br>Opunc05g03540.1 0.001 1 0.999 0.001 1<br>Opunc05g03540.2 0.001 1 0.999 0.001 1	1<br>Obrac05g02930.1 0.074 1 0.926 0.001 1	0	0
	1<br>Osjap01g27410.1 0.09 0.723 0.633 0.066 0.996<br>Osjap03g15140.5 0.743 1 0.257 0.061 1	1<br>Osind01g28630.1 0.09 0.723 0.633 0.066 0.996<br>Osind03g15230.1 0.79 1 0.21 0.03 1	1<br>Orufi01g26130.1 0.09 0.723 0.633 0.066 0.996<br>Orufi03g14720.1 0.79 1 0.21 0.03 1	1<br>Oglab01g19490.1 0.09 0.723 0.633 0.066 0.996<br>Oglab03g14060.1 0.79 1 0.21 0.03 1	1<br>Obart01g23310.1 0.09 0.723 0.633 0.066 0.996<br>Obart03g14050.1 0.79 1 0.21 0.03 1	1<br>Olong_003347-R2 0.09 0.723 0.633 0.047 0.996<br>Olong_009943-R1 0.79 1 0.21 0.03 1	1<br>Opunc01g23460.1 0.09 0.723 0.633 0.064 0.996<br>Opunc03g13590.1 0.79 1 0.21 0.002 0.545	1<br>Obrac01g17590.1 0.09 0.723 0.633 0.067 0.996<br>Obrac03g11330.1 0.79 1 0.21 0.03 1	0<br>Lperr05g03460.1 0.001 0.721 0.72 0.001 0.993	1<br>AT2G47580.1 0.775 1 0.225 0.008 1<br>AT3G16630.1 0.003 0.721 0.718 0.031 0.992
Obart05g03830.2	1<br>Osjap05g04120.1 0.001 1 0.999 0.001 1<br>Osjap05g04120.2 0.001 1 0.999 0.001 1	1<br>Osind05g04070.1 0.008 1 0.992 0.001 1<br>Osind05g04070.2 0.008 1 0.992 0.001 1	1<br>Orufi05g03950.1 0.001 1 0.999 0.001 1<br>Orufi05g03950.2 0.001 1 0.999 0.001 1	1<br>Oglab05g03490.1 0.001 1 0.999 0.001 1<br>Oglab05g03490.2 0.001 1 0.999 0.001 1	1<br>Obart05g03830.1 0.001 1 0.999 0.001 1<br>Obart05g03830.2 0.001 1 0.999 0.001 1	1<br>Olong_016739-R1 0.073 1 0.927 0.001 1	1<br>Opunc05g03540.1 0.001 1 0.999 0.001 1<br>Opunc05g03540.2 0.001 1 0.999 0.001 1	1<br>Obrac05g02930.1 0.073 1 0.927 0.001 1	0	0
	1<br>Osjap01g27410.1 0.089 0.726 0.637 0.066 0.996<br>Osjap03g15140.5 0.747 1 0.253 0.061 1	1<br>Osind01g28630.1 0.089 0.726 0.637 0.066 0.996<br>Osind03g15230.1 0.792 1 0.208 0.03 1	1<br>Orufi01g26130.1 0.089 0.726 0.637 0.066 0.996<br>Orufi03g14720.1 0.792 1 0.208 0.03 1	1<br>Oglab01g19490.1 0.089 0.726 0.637 0.066 0.996<br>Oglab03g14060.1 0.792 1 0.208 0.03 1	1<br>Obart01g23310.1 0.089 0.726 0.637 0.066 0.996<br>Obart03g14050.1 0.792 1 0.208 0.03 1	1<br>Olong_003347-R2 0.089 0.726 0.637 0.047 0.996<br>Olong_009943-R1 0.792 1 0.208 0.03 1	1<br>Opunc01g23460.1 0.089 0.726 0.637 0.064 0.996<br>Opunc03g13590.1 0.792 1 0.208 0.002 0.545	1<br>Obrac01g17590.1 0.089 0.726 0.637 0.067 0.996<br>Obrac03g11330.1 0.792 1 0.208 0.03 1	0<br>Lperr05g03460.1 0.001 0.725 0.724 0.001 0.993	1<br>AT2G47580.1 0.778 1 0.222 0.008 1<br>AT3G16630.1 0.003 0.725 0.722 0.031 0.992
Obart05g25070.1	1<br>Osjap05g28230.1 0.001 1 0.999 0.001 1	1<br>Osind05g28520.1 0.001 1 0.999 0.001 1	1<br>Orufi05g26870.1 0.001 1 0.999 0.001 1	1<br>Oglab05g23160.1 0.001 1 0.999 0.001 1	1<br>Obart05g25070.1 0.001 1 0.999 0.001 1	0	1<br>Opunc05g22640.1 0.001 1 0.999 0.001 1	0	1<br>Lperr01g22990.1 0.001 1 0.999 0.027 1	0
	1<br>Osjap01g31140.1 0.007 0.451 0.444 0.093 0.995<br>Osjap01g31150.1 0.462 0.999 0.537 0.009 0.999	1<br>Osind01g32600.1 0.001 0.451 0.45 0.002 0.995<br>Osind01g32610.1 0.462 0.999 0.537 0.009 0.999	1<br>Orufi01g29880.1 0.001 0.451 0.45 0.075 0.995<br>Orufi01g29890.1 0.462 0.999 0.537 0.009 0.999	1<br>Oglab01g22740.1 0.028 0.451 0.423 0.061 0.995<br>Oglab01g22750.1 0.462 0.999 0.537 0.009 0.999	1<br>Obart01g26870.1 0.006 0.451 0.445 0.117 0.995<br>Obart01g26880.1 0.462 0.999 0.537 0.009 0.999	1<br>Olong_000367-R1 0.105 0.451 0.346 0.002 0.993<br>Olong_000368-R1 0.456 0.999 0.543 0.108 0.999	1<br>Opunc01g26820.1 0.049 0.451 0.402 0.002 0.994<br>Opunc01g26830.1 0.462 1 0.538 0.009 1	1<br>Obrac01g20400.1 0.046 0.451 0.405 0.004 0.994<br>Obrac05g17560.2 0.451 1 0.549 0.164 1	1<br>Lperr05g20850.2 0.467 1 0.533 0.002 1<br>Lperr05g20860.1 0.001 0.451 0.45 0.002 0.995	1<br>AT5G08690.1 0.012 0.451 0.439 0.034 0.995<br>AT5G51600.1 0.462 0.999 0.537 0.008 0.996
Obart06g28060.1	1<br>Osjap09g17800.1 0.024 1 0.976 0.113 0.974<br>Osjap09g17800.2 0.024 1 0.976 0.114 0.974<br>Osjap09g17800.3 0.024 0.997 0.973 0.187 0.947	1<br>Osind06g31620.1 0.001 1 0.999 0.001 1	1<br>Orufi06g29990.1 0.001 1 0.999 0.001 1<br>Orufi09g18790.1 0.024 1 0.976 0.107 0.97<br>Orufi09g18790.2 0.024 1 0.976 0.107 0.97<br>Orufi09g18790.3 0.024 1 0.976 0.108 0.969<br>Orufi09g18790.4 0.024 1 0.976 0.108 0.969<br>Orufi09g18790.5 0.024 0.976 0.952 0.17 0.937<br>Orufi09g18790.6 0.024 0.996 0.972 0.172 0.937	1<br>Oglab06g25490.1 0.001 1 0.999 0.001 1<br>Oglab09g15660.1 0.024 0.999 0.975 0.154 0.95	1<br>Obart06g28060.1 0.001 1 0.999 0.001 1<br>Obart09g17460.1 0.024 1 0.976 0.076 0.969<br>Obart09g17460.2 0.024 1 0.976 0.076 0.969	1<br>Olong_016700-R1 0.001 1 0.999 0.001 1	0	0	1<br>Lperr09g13380.1 0.084 0.982 0.898 0.166 0.951	0
	1<br>Osjap06g32830.1 0.427 1 0.573 0.085 1<br>Osjap06g32840.1 0.001 0.407 0.406 0.002 0.952	1<br>Osind04g24640.1 0.07 0.417 0.347 0.233 0.933<br>Osind06g30840.1 0.485 0.999 0.514 0.109 1	0<br>Orufi06g29150.4 0.485 0.999 0.514 0.122 1	1<br>Oglab02g01250.1 0.066 0.406 0.34 0.221 0.93<br>Oglab10g06880.1 0.427 1 0.573 0.099 1	1<br>Obart04g21770.1 0.07 0.417 0.347 0.232 0.933<br>Obart10g07310.1 0.427 1 0.573 0.099 1	1<br>Olong_000286-R1 0.066 0.406 0.34 0.258 0.928<br>Olong_026973-R2 0.427 1 0.573 0.099 1	1<br>Opunc06g25610.1 0.001 0.407 0.406 0.002 0.951<br>Opunc10g06480.1 0.427 0.998 0.571 0.098 0.984	1<br>Obrac06g20010.2 0.432 1 0.568 0.119 1<br>Obrac06g20020.1 0.001 0.407 0.406 0.002 0.952	1<br>Lperr02g22230.1 0.043 0.392 0.349 0.113 0.88<br>Lperr06g23510.1 0.402 1 0.598 0.074 1	1<br>AT1G76360.1 0.036 0.406 0.37 0.219 0.952<br>AT3G61130.1 0.465 0.999 0.534 0.207 1

Obart08g06070.1	1<br>Osjap08g06650.1 0.002 1 0.998 0.002 1	1<br>Osind08g06790.1 0.002 1 0.998 0.002 1	1<br>Orufi08g06840.1 0.002 1 0.998 0.002 1	1<br>Oglab08g05640.1 0.002 1 0.998 0.002 1	1<br>Obart08g06070.1 0.002 1 0.998 0.002 1	0	1<br>Opunc08g05850.1 0.047 1 0.953 0.068 1	0	0	0
	1<br>Osjap04g21220.1 0.047 0.275 0.228 0.21 0.936<br>Osjap09g20870.1 0.359 0.984 0.625 0.208 0.966	1<br>Osind04g20250.1 0.047 0.275 0.228 0.198 0.94<br>Osind09g20960.1 0.359 0.984 0.625 0.208 0.966	1<br>Orufi04g19250.2 0.047 0.275 0.228 0.21 0.936<br>Orufi09g21960.1 0.359 0.984 0.625 0.2 0.965	1<br>Oglab02g26360.1 0.348 0.996 0.648 0.01 0.994<br>Oglab04g16180.1 0.047 0.275 0.228 0.198 0.94	1<br>Obart02g28440.1 0.348 0.996 0.648 0.093 0.994<br>Obart04g17770.1 0.047 0.275 0.228 0.198 0.94	1<br>Olong_008030-R1 0.047 0.275 0.228 0.232 0.937<br>Olong_008031-R2 0.277 1 0.723 0.106 1	1<br>Opunc04g15550.1 0.047 0.275 0.228 0.206 0.938<br>Opunc04g18740.1 0.295 0.984 0.689 0.088 0.994	1<br>Obrac08g04780.1 0.277 1 0.723 0.098 1<br>Obrac08g04800.1 0.002 0.275 0.273 0.006 0.943	1<br>Lperr08g05690.1 0.277 1 0.723 0.1 1<br>Lperr08g05700.1 0.002 0.275 0.273 0.006 0.943	1<br>AT1G05310.1 0.33 0.982 0.652 0.183 0.99<br>AT4G30800.1 0.047 0.275 0.228 0.208 0.943
Obart08g16670.1	1<br>Osjap01g09050.1 0.007 0.844 0.837 0.061 1	1<br>Osind01g08880.1 0.102 0.986 0.884 0.004 0.567	1<br>Orufi01g08450.1 0.007 0.973 0.966 0.026 0.487	1<br>Oglab01g06790.1 0.007 0.844 0.837 0.061 1<br>Oglab08g15520.1 0.007 0.993 0.986 0.007 0.993	1<br>Obart01g07700.1 0.007 0.844 0.837 0.061 1<br>Obart08g16670.1 0.007 1 0.993 0.007 1	1<br>Olong_003647-R1 0.007 0.83 0.823 0.061 0.985	0	0	0	0
	1<br>Osjap01g24690.1 0.007 0.694 0.687 0.104 0.321<br>Osjap07g09900.1 0.694 0.993 0.299 0.005 0.123	1<br>Osind07g08950.1 0.694 0.993 0.299 0.004 0.111<br>Osind11g05650.1 0.007 0.694 0.687 0.103 0.319	1<br>Orufi01g23650.1 0.007 0.694 0.687 0.106 0.307<br>Orufi07g08630.1 0.694 0.993 0.299 0.005 0.115	1<br>Oglab07g08260.1 0.694 0.973 0.279 0.004 0.099<br>Oglab11g05440.1 0.007 0.694 0.687 0.573 0.683	1<br>Obart07g08560.1 0.694 0.952 0.258 0.009 0.192<br>Obart11g05910.1 0.007 0.694 0.687 0.752 0.834	1<br>Olong_005787-R1 0.007 0.694 0.687 0.103 0.319<br>Olong_016241-R1 0.728 0.932 0.204 0.735 0.768	1<br>Opunc01g21000.1 0.007 0.694 0.687 0.114 0.334<br>Opunc07g08090.1 0.694 0.993 0.299 0.004 0.09	1<br>Obrac07g05080.4 0.728 0.993 0.265 0.102 0.171<br>Obrac11g03720.1 0.007 0.694 0.687 0.112 0.34	1<br>Lperr01g17850.1 0.007 0.694 0.687 0.105 0.313<br>Lperr07g08590.1 0.694 0.993 0.299 0.022 0.124	1<br>AT3G55700.1 0.728 0.973 0.245 0.015 0.093<br>AT4G38130.1 0.007 0.68 0.673 0.09 0.269
Obart08g23660.1	1<br>Osjap08g25130.1 0.007 1 0.993 0.007 1	1<br>Osind08g27620.1 0.007 1 0.993 0.009 1	1<br>Orufi08g26140.1 0.007 1 0.993 0.007 1	1<br>Oglab08g20310.1 0.007 1 0.993 0.007 1	1<br>Obart08g23660.1 0.007 1 0.993 0.007 1	1<br>Olong_025249-R1 0.051 0.964 0.913 0.654 0.984	0	0	0	0
	1<br>Osjap03g41270.1 0.007 0.297 0.29 0.191 0.227<br>Osjap03g43190.1 0.297 0.993 0.696 0.742 0.993	1<br>Osind03g42330.1 0.007 0.297 0.29 0.211 0.259<br>Osind03g44220.1 0.297 0.993 0.696 0.734 0.993	1<br>Orufi03g39770.1 0.007 0.297 0.29 0.204 0.242<br>Orufi03g41620.1 0.297 0.855 0.558 0.779 0.998	1<br>Oglab03g36140.1 0.007 0.297 0.29 0.191 0.227<br>Oglab03g37860.1 0.297 0.993 0.696 0.742 0.993	1<br>Obart03g38160.1 0.007 0.297 0.29 0.203 0.242<br>Obart03g39810.1 0.297 0.993 0.696 0.742 0.993	1<br>Olong_002950-R1 0.036 0.471 0.435 0.593 0.685<br>Olong_020567-R1 0.529 1 0.471 0.328 1	1<br>Opunc03g35260.1 0.007 0.297 0.29 0.248 0.294<br>Opunc03g37040.1 0.297 0.964 0.667 0.742 0.984	1<br>Obrac03g29980.1 0.297 0.957 0.66 0.725 0.981<br>Obrac08g08500.1 0.007 0.297 0.29 0.058 0.116	0<br>Lperr03g33960.1 0.196 0.964 0.768 0.723 0.985	0<br>AT1G08450.1 0.196 0.667 0.471 0.745 0.901
Obart09g14980.1	1<br>Osjap09g15430.2 0.002 1 0.998 0.002 1<br>Osjap09g15430.3 0.002 1 0.998 0.002 1	1<br>Osind09g15570.1 0.002 1 0.998 0.002 1<br>Osind09g15570.2 0.002 1 0.998 0.002 1	1<br>Orufi09g16160.1 0.002 1 0.998 0.002 1<br>Orufi09g16160.2 0.002 1 0.998 0.002 1	1<br>Oglab09g13510.1 0.002 1 0.998 0.002 1<br>Oglab09g13510.2 0.002 1 0.998 0.002 1<br>Oglab09g13510.3 0.002 1 0.998 0.002 1<br>Oglab09g13510.4 0.002 1 0.998 0.002 1	1<br>Obart09g14980.1 0.002 1 0.998 0.002 1<br>Obart09g14980.2 0.002 1 0.998 0.002 1	0	1<br>Opunc09g13420.1 0.002 1 0.998 0.002 1<br>Opunc09g13420.2 0.002 1 0.998 0.002 1	1<br>Obrac09g09090.1 0.002 1 0.998 0.002 1	0	0
	1<br>Osjap02g06650.1 0.01 0.565 0.555 0.01 0.997<br>Osjap09g15430.1 0.569 1 0.431 0.131 1	1<br>Osind02g06940.1 0.01 0.565 0.555 0.01 0.997<br>Osind08g23920.1 0.569 0.993 0.424 0.131 0.988	1<br>Orufi04g24220.1 0.014 0.567 0.553 0.165 1<br>Orufi08g22880.1 0.569 0.993 0.424 0.131 0.988	1<br>Oglab08g19240.1 0.569 0.993 0.424 0.143 0.986<br>Oglab12g18680.1 0.002 0.567 0.565 0.003 1	1<br>Obart02g06180.1 0.01 0.565 0.555 0.01 0.997<br>Obart08g20390.1 0.569 0.993 0.424 0.631 0.988	1<br>Olong_008093-R2 0.596 0.991 0.395 0.25 0.996<br>Olong_024186-R1 0.002 0.567 0.565 0.003 1	1<br>Opunc04g20190.1 0.007 0.567 0.56 0.006 1<br>Opunc08g18560.1 0.569 0.993 0.424 0.128 0.969	1<br>Obrac01g09090.1 0.605 0.998 0.393 0.299 0.982<br>Obrac02g04550.3 0.009 0.565 0.556 0.214 0.997	1<br>Lperr04g18350.1 0.002 0.567 0.565 0.021 1<br>Lperr09g11120.1 0.569 1 0.431 0.156 1	1<br>AT1G68540.1 0.567 0.991 0.424 0.112 1<br>AT2G21270.3 0.003 0.565 0.562 0.059 0.997
Obart09g14980.2	1<br>Osjap09g15430.2 0.002 1 0.998 0.002 1<br>Osjap09g15430.3 0.002 1 0.998 0.002 1	1<br>Osind09g15570.1 0.002 1 0.998 0.002 1<br>Osind09g15570.2 0.002 1 0.998 0.002 1	1<br>Orufi09g16160.1 0.002 1 0.998 0.002 1<br>Orufi09g16160.2 0.002 1 0.998 0.002 1	1<br>Oglab09g13510.1 0.002 1 0.998 0.002 1<br>Oglab09g13510.2 0.002 1 0.998 0.002 1<br>Oglab09g13510.3 0.002 1 0.998 0.002 1<br>Oglab09g13510.4 0.002 1 0.998 0.002 1	1<br>Obart09g14980.1 0.002 1 0.998 0.002 1<br>Obart09g14980.2 0.002 1 0.998 0.002 1	0	1<br>Opunc09g13420.1 0.002 1 0.998 0.002 1<br>Opunc09g13420.2 0.002 1 0.998 0.002 1	1<br>Obrac09g09090.1 0.002 1 0.998 0.002 1	0	0
	1<br>Osjap02g06650.1 0.01 0.54 0.53 0.01 0.997<br>Osjap09g15430.1 0.544 1 0.456 0.131 1	1<br>Osind02g06940.1 0.01 0.54 0.53 0.01 0.997<br>Osind08g23920.1 0.544 0.993 0.449 0.131 0.988	1<br>Orufi04g24220.1 0.013 0.542 0.529 0.165 1<br>Orufi08g22880.1 0.544 0.993 0.449 0.131 0.988	1<br>Oglab08g19240.1 0.544 0.993 0.449 0.143 0.986<br>Oglab12g18680.1 0.002 0.542 0.54 0.003 1	1<br>Obart02g06180.1 0.01 0.54 0.53 0.01 0.997<br>Obart08g20390.1 0.544 0.993 0.449 0.631 0.988	1<br>Olong_013054-R1 0.547 0.992 0.445 0.149 0.979<br>Olong_024186-R1 0.002 0.542 0.54 0.003 1	1<br>Opunc04g20190.1 0.007 0.542 0.535 0.006 1<br>Opunc08g18560.1 0.544 0.993 0.449 0.128 0.969	1<br>Obrac02g04550.3 0.008 0.54 0.532 0.214 0.997<br>Obrac09g00560.1 0.548 0.998 0.45 0.164 0.966	1<br>Lperr04g18350.1 0.002 0.542 0.54 0.021 1<br>Lperr09g11120.1 0.544 1 0.456 0.156 1	1<br>AT1G68540.1 0.542 0.992 0.45 0.112 1<br>AT2G21270.3 0.003 0.54 0.537 0.059 0.997
Obart09g19980.2	1<br>Osjap09g20460.1 0.001 0.999 0.998 0.001 0.999	1<br>Osind09g20560.1 0.001 0.984 0.983 0.001 0.998	1<br>Orufi09g21510.1 0.001 0.956 0.955 0.001 0.999	1<br>Oglab09g18200.1 0.001 1 0.999 0.001 1	1<br>Obart09g19980.1 0.018 1 0.982 0.181 1<br>Obart09g19980.2 0.001 1 0.999 0.001 1	1<br>Olong_014589-R1 0.072 0.971 0.899 0.032 0.991<br>Olong_014589-R2 0.072 0.971 0.899 0.033 0.991	0	0	0	0

	1<br>Osjap04g28790.1 0.021 0.598 0.577 0.154 0.962<br>Osjap09g20430.1 0.631 0.999 0.368 0.151 0.998	1<br>Osind02g35910.1 0.02 0.598 0.578 0.165 0.967<br>Osind09g20550.1 0.6 0.999 0.399 0.058 0.999	1<br>Orufi02g09040.1 0.02 0.599 0.579 0.106 0.969<br>Orufi09g21500.1 0.631 0.999 0.368 0.151 0.998	1<br>Oglab02g09010.1 0.02 0.599 0.579 0.11 0.969<br>Oglab09g18170.1 0.6 0.999 0.399 0.13 0.998	1<br>Obart02g09010.1 0.02 0.599 0.579 0.014 0.966<br>Obart09g19950.1 0.6 0.999 0.399 0.13 0.998	0<br>Olong_022766-R1 0.02 0.598 0.578 0.112 0.96	1<br>Opunc09g18280.1 0.6 0.998 0.398 0.118 0.951<br>Opunc09g18290.2 0.001 0.599 0.598 0.001 0.971	0<br>Obrac09g13160.1 0.001 0.599 0.598 0.001 0.953	1<br>Lperr02g15070.1 0.016 0.614 0.598 0.138 0.986<br>Lperr09g15930.1 0.64 0.943 0.303 0.186 0.99	0<br>AT5G11850.1 0.002 0.602 0.6 0.09 0.965
Obart11g01060.1	1<br>Osjap11g01220.1 0.001 1 0.999 0.001 1<br>Osjap12g01140.1 0.001 1 0.999 0.001 1	1<br>Osind11g00690.1 0.001 0.954 0.953 0.001 0.999<br>Osind12g01290.1 0.001 1 0.999 0.001 1	1<br>Orufi11g01000.1 0.001 1 0.999 0.001 1<br>Orufi12g01130.1 0.001 1 0.999 0.001 1	1<br>Oglab12g00800.1 0.001 1 0.999 0.001 1	1<br>Obart11g01060.1 0.001 1 0.999 0.001 1	0	1<br>Opunc12g00940.1 0.001 1 0.999 0.001 1	0	0	0
	1<br>Osjap03g13100.1 0.479 0.982 0.503 0.097 0.622<br>Osjap07g30390.1 0.01 0.42 0.41 0.013 0.944	1<br>Osind03g12930.1 0.479 0.982 0.503 0.121 0.633<br>Osind07g27070.1 0.007 0.42 0.413 0.006 0.946	1<br>Orufi03g17220.1 0.485 1 0.515 0.027 1<br>Orufi07g27420.1 0.01 0.42 0.41 0.013 0.946	1<br>Oglab03g12120.1 0.479 0.982 0.503 0.121 0.633<br>Oglab07g22980.1 0.007 0.42 0.413 0.007 0.94	1<br>Obart03g12120.1 0.479 0.982 0.503 0.121 0.633<br>Obart12g00790.1 0.001 0.42 0.419 0.002 0.949	1<br>Olong_007715-R1 0.482 1 0.518 0.007 1<br>Olong_008211-R1 0.007 0.42 0.413 0.006 0.946	1<br>Opunc03g11680.1 0.479 1 0.521 0.148 1<br>Opunc11g00920.1 0.001 0.42 0.419 0.142 0.951	1<br>Obrac03g09720.2 0.479 1 0.521 0.142 1<br>Obrac12g00780.1 0.001 0.42 0.419 0.11 0.953	1<br>Lperr03g10720.1 0.479 0.996 0.517 0.148 0.954<br>Lperr11g00920.1 0.001 0.42 0.419 0.002 0.949	1<br>AT3G46740.1 0.482 1 0.518 0.161 1<br>AT4G20110.2 0.001 0.42 0.419 0.002 0.946
Obart11g19500.1	1<br>Osjap04g11870.1 0.011 0.997 0.986 0.001 0.995<br>Osjap11g22480.1 0.155 0.997 0.842 0.07 0.998<br>Osjap11g22480.2 0.024 0.997 0.973 0.022 0.999<br>Osjap11g22530.1 0.001 1 0.999 0.001 1<br>Osjap11g22560.1 0.001 1 0.999 0.002 0.998	1<br>Osind06g17670.1 0.001 0.997 0.996 0.001 0.996	1<br>Orufi04g10300.1 0.011 0.997 0.986 0.001 0.995<br>Orufi11g20790.1 0.059 0.997 0.938 0.006 0.999<br>Orufi11g20820.1 0.001 0.997 0.996 0.001 0.991<br>Orufi11g20870.1 0.001 1 0.999 0.002 0.998	1<br>Oglab11g17300.1 0.014 0.997 0.983 0.007 0.999<br>Oglab11g17330.1 0.001 1 0.999 0.001 1	1<br>Obart11g19430.1 0.001 0.997 0.996 0.002 0.992<br>Obart11g19470.1 0.014 0.997 0.983 0.007 0.996<br>Obart11g19500.1 0.001 1 0.999 0.001 1	1<br>Olong_018816-R1 0.014 0.997 0.983 0.008 0.887<br>Olong_019250-R1 0.055 0.997 0.942 0.044 1	0	0	0	0
	1<br>Osjap04g11850.1 0.001 0.314 0.313 0.001 0.968<br>Osjap11g17500.5 0.333 0.997 0.664 0.366 0.946	1<br>Osind04g10340.1 0.341 0.997 0.656 0.009 0.957<br>Osind11g06800.1 0.003 0.316 0.313 0.01 0.997	1<br>Orufi04g10280.1 0.001 0.314 0.313 0.001 0.972<br>Orufi11g16310.1 0.336 0.997 0.661 0.65 0.996	1<br>Oglab11g05980.1 0.012 0.299 0.287 0.046 0.527<br>Oglab11g13660.8 0.325 0.997 0.672 0.453 0.992	1<br>Obart11g06460.1 0.012 0.299 0.287 0.098 0.5<br>Obart11g15290.1 0.333 0.997 0.664 0.593 0.993	0<br>Olong_020133-R1 0.012 0.315 0.303 0.005 0.482	1<br>Opunc11g06610.1 0.012 0.315 0.303 0.005 0.493<br>Opunc11g12670.3 0.33 0.997 0.667 0.563 0.995	1<br>Obrac11g04140.4 0.012 0.312 0.3 0.002 0.8<br>Obrac11g09030.1 0.335 0.998 0.663 0.364 1	0<br>Lperr11g06010.1 0.026 0.328 0.302 0.007 0.319	0<br>AT4G21410.1 0.017 0.328 0.311 0.511 0.981
Obart12g02080.1	1<br>Osjap11g02640.1 0.002 1 0.998 0.024 1<br>Osjap12g02610.1 0.002 1 0.998 0.022 1	1<br>Osind11g01990.1 0.002 1 0.998 0.023 1<br>Osind12g02780.1 0.002 1 0.998 0.022 1	1<br>Orufi11g02320.1 0.002 1 0.998 0.003 1<br>Orufi12g02480.1 0.002 1 0.998 0.021 1	1<br>Oglab11g02200.1 0.002 1 0.998 0.023 1<br>Oglab12g02130.1 0.002 1 0.998 0.022 1	1<br>Obart12g02080.1 0.002 1 0.998 0.002 1	0	0	0	0	0
	1<br>Osjap12g02600.1 0.002 0.64 0.638 0.006 1<br>Osjap12g12870.1 0.644 0.948 0.304 0.055 0.417	1<br>Osind11g01980.1 0.002 0.64 0.638 0.006 1<br>Osind12g10020.1 0.644 0.948 0.304 0.055 0.417	1<br>Orufi12g02470.1 0.002 0.64 0.638 0.006 1<br>Orufi12g10750.1 0.642 0.948 0.306 0.045 0.364	1<br>Oglab12g02120.1 0.002 0.64 0.638 0.006 1<br>Oglab12g09880.1 0.644 0.948 0.304 0.051 0.385	0<br>Obart11g02350.1 0.002 0.64 0.638 0.004 0.993	1<br>Olong_003435-R1 0.133 0.64 0.507 0.212 0.984<br>Olong_023179-R1 0.642 0.998 0.356 0.05 0.329	1<br>Opunc11g02180.1 0.002 0.64 0.638 0.028 0.992<br>Opunc12g08740.1 0.642 0.948 0.306 0.04 0.324	0<br>Obrac05g10860.1 0.072 0.523 0.451 0.097 0.99<br>Obrac12g07170.1 0.642 0.974 0.332 0.071 0.67	1<br>Lperr12g02190.1 0.002 0.64 0.638 0.033 0.992<br>Lperr12g08390.1 0.642 0.956 0.314 0.056 0.454	0<br>AT3G27320.1 0.052 0.421 0.369 0.596 0.978
Obart12g09410.1	1<br>Osjap12g12320.1 0.001 1 0.999 0.001 1	1<br>Osind12g09530.1 0.001 1 0.999 0.001 1	1<br>Orufi12g10460.1 0.001 1 0.999 0.001 1	1<br>Oglab12g09470.1 0.001 1 0.999 0.001 1	1<br>Obart12g09410.1 0.001 1 0.999 0.001 1	0	0	0	0	0
	1<br>Osjap01g44780.3 0.452 0.998 0.546 0.017 0.994<br>Osjap03g07600.1 0.001 0.306 0.305 0.004 0.996	1<br>Osind01g46040.1 0.452 0.998 0.546 0.186 0.995<br>Osind03g07670.1 0.001 0.306 0.305 0.004 0.996	1<br>Orufi01g42650.3 0.452 0.998 0.546 0.228 0.995<br>Orufi03g07180.1 0.001 0.306 0.305 0.004 0.996	1<br>Oglab01g34040.1 0.477 0.998 0.521 0.203 0.995<br>Oglab03g07280.1 0.001 0.306 0.305 0.004 0.996	1<br>Obart01g39260.2 0.452 0.998 0.546 0.017 0.994<br>Obart03g07210.1 0.001 0.306 0.305 0.004 0.996	1<br>Olong_021912-R1 0.376 1 0.624 0.002 1<br>Olong_021913-R1 0.001 0.305 0.304 0.004 0.996	1<br>Opunc12g08570.1 0.406 1 0.594 0.152 1<br>Opunc12g08580.1 0.001 0.305 0.304 0.004 0.996	1<br>Obrac12g06490.1 0.001 0.305 0.304 0.004 0.996<br>Obrac12g06500.1 0.449 1 0.551 0.103 1	1<br>Lperr03g06600.1 0.001 0.304 0.303 0.004 0.992<br>Lperr12g08310.1 0.452 0.999 0.547 0.002 0.998	1<br>AT4G34670.1 0.001 0.297 0.296 0.004 0.966<br>AT5G67540.2 0.477 0.993 0.516 0.053 1
