# Supplementary material for: Gene fusion as an important mechanism to generate new genes in the genus Oryza
Source: Genome Biol. 2022 Jun 15;23:130. doi: 10.1186/s13059-022-02696-w (PMC9199173; doi:10.1186/s13059-022-02696-w)
Supplement: Supplementary file 11 — Additional file 11. Review history. [file 13059_2022_2696_MOESM11_ESM.docx]

Review History

**First round of review**

**Reviewer 1**

**Were you able to assess all statistics in the manuscript, including the appropriateness of statistical tests used?**

No, I do not feel adequately qualified to assess the statistics.

**Comments to author:**

This study designed a novel pipeline to identify fusion genes by conducting genome-wide interrogations of four Oryza genomes. The authors detected 310 fusion genes in four Oryza species and estimated that the rate of origination of fusion genes was as high as 63 fusion genes per species per million years in the rice tribe, two orders of magnitude higher than that estimated for flies. They also showed that over 44% of the fusion genes were expressed, with 90% of gene pairs showing strong signals of purifying selection. Using CRISPR/Cas9 knockout technique, they found that the new formed fusion genes mediated the phenotypes such as seed germination, shoot length and root length and implicated the functionality of these fusion genes. Overall, this study is a good attempt with an interesting topic (though not very novel) with a new designed method (GriffinDetector). The results were presented clearly and logically, and the manuscript was well written and concise. Despite these, I feel that this manuscript suffers from numerous weaknesses mentioned below and it's premature to publish this study until all the concerns and uncertainties were clarified and addressed.

Major concerns:

1) I acknowledged that it's an important topic to understand the origin, molecular mechanisms underlying and their evolutionary implications of new genes given that many interesting questions relevant are to be solved. Following previous studies by almost the same team, this study chose to focus on fusion genes, which was undoubtedly logical and encouraged. However, such studies on new genes require high-quality (de novo) genomes and related genomic resources from both the focus species and the closely related species. In this sense, it's bit disappointed that no any new data was generated in this work to add the current genomic resources and facilitate such a type of study because all genomic sequences and RNASeq datasets used in this work were downloaded from previous studies. In addition, the outgroups (Arabidopsis, maize and sorghum) used in this study were too far away (distinct) phylogenetically from the focus species (four Orzya species) to be appropriate although I understand that these outgroups have high-quality assembled genomes. To short, I feel that the genomic datasets used here seems not sufficient for an in-depth investigation and may include many false positives in various analyses. Major results and conclusions would be questionable before these basic technical issues were addressed.

2) This study identified 310 fusion genes across four Oryza species with most of them being species specific genes (page 6 and Table 1). This looks odd to me because gene fusion is one of numerous processes of new gene formation and Zhang et al. (2019) identified 175 new genes in Japonica (much less). How to explain that the number of fusion genes is larger than that of new genes in almost the same taxa (the four focus species were very closely related in the evolutionary time frame)? In addition, I wonder why the number of fusion genes are so different in four focus genomes (taxa) given all of them have a recent common ancestor relative to the outgroups? i.e., for each ingroup, more than half of the fusion genes are species-specific. It's very hard to explain! This raises a question regarding the accuracy mentioned in point 1) because of limited high-quality genomes and suitable outgroups. Ideally, at least one high-quality de novo genomes of an Oryza species should be used as an outgroup to ensure the feasibility and accuracy of this study. I guess this should be realistic given many de novo genomes in the rice genus have been completed during a recent decade.

3) Page 9. This section was a good attempt to use CRISPR technique to investigate the functional issues involving the evolutionary fates of fusion genes. However, many technical issues were unclear or equivocal. Say, why selected one single gene each from "neo-functionalization" and "subfunctionalization" genes? How many such genes in the fusion genes identified? Because 30 and 29 lines were obtained for the two genes, respectively, were three homozygous mutants from each gene sufficient for the results or conclusions?

4) Regarding the section "Fusion genes drive phenotype evolution", only two phenotypes (root length and shoot length of germinated seedlings) were measured "at five days after sowing". Could the results answer "whether these expressed fusion genes are associated with adaptive phenotypes" by focusing these two phenotypic traits? I agree they are functional but I did not find any proofs available for the argument that they were adaptive?

Minor points:

Page 4, lines 24-25. What that meant by "diverged from each other within <0.01-1" ? In fact, African cultivated rice O. glaberrima started to be domesticated roughly 4000 years before present.

Page 8, 1-2. Using RNA-seq data from root, leaf, and mixed stage panicle tissues of O. sativa v.g. japonica, O. barthii, O. glaberrima, O. punctata, O. brachyantha and L. perrieri [23]. Is any problems right here because only 80 out of 157 fusion genes in japonica were shared by all four focus species? Why not to use the shared 80 genes?

Page 10, 1-4. I am not sure how the comparisons were made. "… much lower that of the parental line Nipponbare", "… shorter than the wild type parental lines"? Which ones were the controls? And which was the wild type parental lines (wild type plants)?

Page 10, 8-10. I am not sure what that meant by "the parental gene status" because all the copies in the five species could be treated to be parental.

**Reviewer 2**

**Were you able to assess all statistics in the manuscript, including the appropriateness of statistical tests used?**

Yes, and I have assessed the statistics in my report.

**Comments to author:**

This study has developed a new pipline - GriffinDetector to identify fusion genes in Oryza genomes. Fusion genes were identified based on comparison of annotated genes from Oryza species and other out-group species. Gene knockout of two fusion genes via CRIPPR-cas technique showed that these fusion genes involved in the biological process, such as seed germination, shoot and root growth. The results are interesting, but I have some concerns about the existence of fusion genes before the publication of article in Genome Biology. They are listed as follows.

Major issues:

1. The fusion genes identification in this study mainly relied on the annotation results of these analyzed species in this study. However, fusion genes had low expression or no expression, the gene structures of which are usually lack of evidence. I suggested that the candidate fusion genes should be re-annotated.

2. Transcriptome data is an important evidence source for fusion genes, such as reads representing the fusion region of the identified genes. The authors could provide a set of discordant read pairs is mapped to two different genes, or when junction-spanning reads. Among fusion genes, 37 genes did not produce detectable FPKM signals, which would make them hard to be distinguished as functional gene.

3. It seems that the gene identity between parental genes and fusion genes are very high (Figure 4). Because some parental genes were not in the collinear region of fusion genes (Figure 6), the parental gene copies are co-existed with the fusion genes in the same genome. Please list fusions for this situation and add information for the target sites of sgRNA whether also targeted parental gene copies.

4. The authors present that an estimated 63 fusion genes could be generated in one species per million years. 63 fusion genes are a little scale of genes from the whole genome-wide perspective (63/52,424=0.0012) in 1 million years. My concern is about how many new genes would be formed by other ways like gene mutation, TE, recombination, etc., and comparing to these ways, whether new genes generated from gene fusion is a major mode for gene formation or at least one of the important ways. I think it's important for us to assess the value of this study, and authors should declare this question clear in Introduction or Discussion sections.

5. I wonder why choosing the 4 species of Oryza in this study, and how often fusion genes happened in other species in Oryza and also in other plants besides of Oryza.

6. The authors need to perform extra transformation by introducing the fusion gene into the crispr lines to elucidate the function of the candidate genes. Transgenic negative lines rather than wild type plants should be used as controls. Also, neither detailed description about the method nor the statistical for Fig. 5 was found.

Minor issues:

1. Uncontinuous line numbers over the whole MS make troubles for reviewing, the authors need add the continuous line number in the MS, for the ease of reviewing.

2. Methods described in Results section for the categorization of three major types of 69 expressed fusion genes should be moved to the Methods section.

3. Figure quality need to be improved; many figures are unclear.

4. Other errors:

1) Page 5, line 11, a redundant blank before 'However, it has been a challenge to understand the role ...'

2) Page 7, line 4, 'developed' should be 'Developed'

3) Arabic and English numbers used in MS is muddled, like page 9, line 2: '58 were shared between two to four species' English number 'two' used here; but at page 9, line 13 'with only 2 genes being 100%' Arabic number '2' used here; others like page 10, line 6, page 10, line 10; page 11, line 16, 22, 23; page 13 line 3, 9…

4) page 9, line 3 line 9: 'Supplementary Table 1', 'Table 1 and Fig. 3', used here; but at page 9, line 12, line 17 'Supplementary Table2' was used. Page 10 line 9, 'Fig1'; page 9 line 9 'Fig. 1'; page 10 line 9, 'Supplemental Fig1'; page 8 line 5, 'Fig. 2 A, B & C' line 8 '(Fig. 2 D & E) and group (Fig. 2 A & F)'; page 12 line 14 'Fig. 6A'; please use a unified format and revise across the entire MS.

5) page 14, line 28 'retrocopies they detected 1235 and 150, respectively', corrected to '1,235'.

6) page 18, line 20-22, error line spacing.

7) Page 24 line 18: corrected to bold front of 'Knockout mutant generated by CRISPR/Cas9'.

8) The marking of "sgRNA1" is missing in Figure 4A.

**Authors Response**

**Point-by-point responses to the reviewers’ comments:**

Reviewer #1:

This study designed a novel pipeline to identify fusion genes by conducting genome-wide interrogations of four Oryza genomes. The authors detected 310 fusion genes in four Oryza species and estimated that the rate of origination of fusion genes was as high as 63 fusion genes per species per million years in the rice tribe, two orders of magnitude higher than that estimated for flies. They also showed that over 44% of the fusion genes were expressed, with 90% of gene pairs showing strong signals of purifying selection. Using CRISPR/Cas9 knockout technique, they found that the new formed fusion genes mediated the phenotypes such as seed germination, shoot length and root length and implicated the functionality of these fusion genes. Overall, this study is a good attempt with an interesting topic (though not very novel) with a new designed method (GriffinDetector). The results were presented clearly and logically, and the manuscript was well written and concise. Despite these, I feel that this manuscript suffers from numerous weaknesses mentioned below and it's premature to publish this study until all the concerns and uncertainties were clarified and addressed.

Major concerns:

1) I acknowledged that it's an important topic to understand the origin, molecular mechanisms underlying and their evolutionary implications of new genes given that many interesting questions relevant are to be solved. Following previous studies by almost the same team, this study chose to focus on fusion genes, which was undoubtedly logical and encouraged. However, such studies on new genes require high-quality (de novo) genomes and related genomic resources from both the focus species and the closely related species. In this sense, it's bit disappointed that no any new data was generated in this work to add the current genomic resources and facilitate such a type of study because all genomic sequences and RNASeq datasets used in this work were downloaded from previous studies. In addition, the outgroups (Arabidopsis, maize and sorghum) used in this study were too far away (distinct) phylogenetically from the focus species (four Orzya species) to be appropriate although I understand that these outgroups have high-quality assembled genomes. To short, I feel that the genomic datasets used here seems not sufficient for an in-depth investigation and may include many false positives in various analyses. Major results and conclusions would be questionable before these basic technical issues were addressed.

*Response: Thanks very much for reviewer1’s positive views of evolutionary implications of new genes, it’s an interesting filed with plenty of hotspots, and it’s our pleasure to produce more works to share with the people who are interested in it.*

*Regarding the creation of new data, we would like to present following several lines of novelties from data to methodology to need of fields. First of all, besides we created the first set of gene editing data that are able to reveal previously rarely studied functionality in morphology, we would like to emphasize that the genome sequences we created are among of the best quality data that are most suitable to analyze gene evolution in the organism (e.g. Stein et al [1], 2018. Nature Ecology Evolution; Mortola and Long [3], 2021. American Scientist, as we cited in the manuscript). Furthermore, in this work, we pay more attention on the mechanism of the origination of fusion genes, all well as the fixation rate of new genes in the specific population. Moreover, since too many data and experiments are crossed to each other, it can hardly redo the analysis from the beginning part without the pipeline and algorithm as we created in this study. Since our work has proved the pipeline was eligible to run for detecting fusion genes, the colleagues in related genomic fields can use to analyze more data in the future.*

*Thanks for the warning that “the results are genome-quality dependent”. Before we discussed about the specifics in this study, we should point out the quality analysis on the genome sequences we created, which showed to be on the top of published genome data (Stein et al, 2018) especially for the analysis of new gene evolution (Zhang et al, 2019. Nature Ecology & Evolution). In this study, we further double checked our data. We found by the Blat analysis that (1) > 94% fusion genes detected by our work could be found in the new genome data. Such as in japonica, only 4 ouf 80 species specific fusion genes have 10-21 bp difference, which support our results is reliable. In additional, we also investigated the fixation frequency of fusion genes in large population data for each species, about 15.4%-93.0% fixation rate was found for four focus species, which suggests fusion genes are under selection to be fixed in the population. All these results are seminal and would provide a novel insight for new gene evolution. (2) On the other hand, species in the Mid-group will dynamically added to In-group in the pipeline, and finally at least one Mid-group was retained, this process will improve our accurate to remove genome annotation bias in focus species. Beside, as shown in line 486, O. brachyantha and Leersia perrieri were also fixed as Out-group, which are closely related species to rice. Thus, we have to respectfully point out that our research scheme has highly successfully avoided the deviation caused by genome quality.*

2) This study identified 310 fusion genes across four Oryza species with most of them being species specific genes (page 6 and Table 1). This looks odd to me because gene fusion is one of numerous processes of new gene formation and Zhang et al. (2019) identified 175 new genes in Japonica (much less). How to explain that the number of fusion genes is larger than that of new genes in almost the same taxa (the four focus species were very closely related in the evolutionary time frame)? In addition, I wonder why the number of fusion genes are so different in four focus genomes (taxa) given all of them have a recent common ancestor relative to the outgroups? i.e., for each ingroup, more than half of the fusion genes are species-specific. It's very hard to explain! This raises a question regarding the accuracy mentioned in point 1) because of limited high-quality genomes and suitable outgroups. Ideally, at least one high-quality de novo genomes of an Oryza species should be used as an outgroup to ensure the feasibility and accuracy of this study. I guess this should be realistic given many de novo genomes in the rice genus have been completed during a recent decade.

*Response: Thanks for the comments. We are sorry if we did not make it clear enough in last version for following points.*

*(1) Gene fusion is one type of new gene that is utterly different from the another type of new gene called de novo genes reported previously (Zhang et al, 2019. Nature Ecol Evol). However, the question why the fusion gene we reported is frequently higher than the de novo genes is a good question. One clear response can be found based on two classic references:*

*a. As Jacob (1977, Science. 196, 1161-1166) pointed out, the probability that orphan or noncoding sequences undergo fast turnover and become a functional new gene was thought to be extremely low.*

*b. Fusion gene originated from at least two independent parental genes, which largely depend on gene duplication driven by WGD, gene expansion, recombination or TE. Ohno S [4] proposed that gene duplication was the most important mechanism producing new gene copies. Works in humans and Drosophila also suggested that ~80% of genes are formed by DNA-based duplication, 5% - 10% by de novo duplication, and ~10% by retroposition [5].*

*Based on point (a) and (b), we think it was understandable that the number of fusion genes was much larger.*

*(2) As we had answered in the first question, we have fixed O. brachyantha and Leersia perrieri in the Out-group, which could largely reduce genome bias in our analysis. We also have checked the candidate fusion genes in newly generated genome and in big population data. Most of the fusion genes were available. Thus, for the “number of fusion genes are so different in four focus genomes” part, we think it was a great question” part, we agree it was a great question. We think the possible reason were (a) the fusion gene we detected might be fixed in different probabilities that may be determined by a series of factors, mainly including effective population size and selective strength, that can be different in the 4 species. (b) Different new gene of the focus species may be related to its functionality that defines changed phenotypic effects [6].*

3) Page 9. This section was a good attempt to use CRISPR technique to investigate the functional issues involving the evolutionary fates of fusion genes. However, many technical issues were unclear or equivocal. Say, why selected one single gene each from "neo-functionalization" and "subfunctionalization" genes? How many such genes in the fusion genes identified? Because 30 and 29 lines were obtained for the two genes, respectively, were three homozygous mutants from each gene sufficient for the results or conclusions?

*Response: Thanks for the comments. In japonica, we chose fusion genes with express level significantly correlated (R> 0.8, p<0.05) with parental gene to represent sub-functionalization, 18 fusion genes were obtained. Fusion genes without expression correlation (R< 0.5) to parental gene were treated as neo-functionalization, only 6 fusion genes were obtained. At first, we chose two representatives from each type for CRISPR/Cas9, but only one was successful, so we remain one for each type. Even though 30 and 29 lines were obtained for two fusion genes, but only 14 and 13 lines were edited, respectively. Apart from the same editing lines, there are only 9 and 7 edit types in total respectively. Apart from 2-3 complicated editing in both strand, we chose 3-5 lines for each fusion gene is applicable. In the functional study, 3 lines for each gene are generally acceptable, such as in Figure 4d of Wang et al [7] and Figure 1b of Dou et al [8].*

4) Regarding the section "Fusion genes drive phenotype evolution", only two phenotypes (root length and shoot length of germinated seedlings) were measured "at five days after sowing". Could the results answer "whether these expressed fusion genes are associated with adaptive phenotypes" by focusing these two phenotypic traits? I agree they are functional but I did not find any proofs available for the argument that they were adaptive?

*Response: Thanks for the question, we agree with this suggestion. We have revised this conclusion, we changed the subtitle to be “Fusion genes may drive phenotype evolution”. In additional, we search the expression data of two phenotypic fusion genes in rice stress database (http://bar.utoronto.ca/efprice/cgi-bin/efpWeb.cgi), Osjap07g28390 and Osjap09g15430 mainly response to drought stress. As reported in other species, drought often reduced germination rate, root length and shoot length [9, 10], thus the shorter root and lower germination in CRISPR mutant possibly reflect a result of drought adaptation.*

Minor points:

Page 4, lines 24-25. What that meant by "diverged from each other within <0.01-1"? In fact, African cultivated rice O. glaberrima started to be domesticated roughly 4000 years before present.

*Response: Thanks for the suggestion. We have changed as “that diverged from each other within 0.40 million years” in line 95.*

Page 8, 1-2. Using RNA-seq data from root, leaf, and mixed stage panicle tissues of O. sativa v.g. japonica, O. barthii, O. glaberrima, O. punctata, O. brachyantha and L. perrieri [23]. Is any problems right here because only 80 out of 157 fusion genes in japonica were shared by all four focus species? Why not to use the shared 80 genes?

*Response: Thanks for the comments. As reported, 114 fusion gene contain 157 transcripts were obtained in Japonica. Several isoform of fusion gene like Osjap01g06620 (line 12-17 in Table S3) and Osjap08g04940 (line 114-119 in Table S3) lead us to the final number of 157 gene transcripts. In this part, we only search homologous gene of japonica fusion gene, so it comes to 157 transcripts, not only the 80 species-specific gene in japonica.*

Page 10, 1-4. I am not sure how the comparisons were made. "… much lower that of the parental line Nipponbare", "… shorter than the wild type parental lines"? Which ones were the controls? And which was the wild type parental lines (wild type plants)?

*Response: Thanks for the good suggestion. Nipponbare was treated as control (wild type) in this study. Parental Nipponbare means the untransformed wild type. We have changed the sentence as “We found that the germination rate and root length of the three Osjap07g28390 lines was much lower that of the same parental line Nipponbare, and that root and shoot length of the three Osjap09g15430 edited lines were shorter (Figure 5) than the same parental line Nipponbare grown under identical conditions”.*

Page 10, 8-10. I am not sure what that meant by "the parental gene status" because all the copies in the five species could be treated to be parental.

*Response: In the origination pattern part of line 241, "the parental gene status" actually means the presence and location of parental gene.*

Reviewer #2:

This study has developed a new pipline - GriffinDetector to identify fusion genes in Oryza genomes. Fusion genes were identified based on comparison of annotated genes from Oryza species and other out-group species. Gene knockout of two fusion genes via CRIPPR-cas technique showed that these fusion genes involved in the biological process, such as seed germination, shoot and root growth. The results are interesting, but I have some concerns about the existence of fusion genes before the publication of article in Genome Biology. They are listed as follows.

Major issues:

1. The fusion genes identification in this study mainly relied on the annotation results of these analyzed species in this study. However, fusion genes had low expression or no expression, the gene structures of which are usually lack of evidence. I suggested that the candidate fusion genes should be re-annotated.

*Response: Yes, we have re-annotated the fusion genes for four focus species (indica, japonica, glaberrima and barthii). Only very little difference (10- 30bp) in 0%~6.5% fusion genes are found. On the other hand, we also investigated the fixation rate of fusion genes in large population sequence data. The result showed that 31.8%, 15.4%, 21.5% and 93.0% species specific fusion genes have been fixed in indica, japonica, glaberrima and barthii, respectively.*

2. Transcriptome data is an important evidence source for fusion genes, such as reads representing the fusion region of the identified genes. The authors could provide a set of discordant read pairs is mapped to two different genes, or when junction-spanning reads. Among fusion genes, 37 genes did not produce detectable FPKM signals, which would make them hard to be distinguished as functional gene.

*Response: Thanks for the comments. Illumina reads are good evidence of fusion gene. We chose two fusion genes for test. After import the bam file in IGV, we could found reads mapped to junction area for sub-functionalized Osjap09g15430 and neo-functionalized Osjap07g28390.*

3. It seems that the gene identity between parental genes and fusion genes are very high (Figure 4). Because some parental genes were not in the collinear region of fusion genes (Figure 6), the parental gene copies are co-existed with the fusion genes in the same genome. Please list fusions for this situation and add information for the target sites of sgRNA whether also targeted parental gene copies.

*Response: That is a good question. In fact, we designed the sgRNA on the consideration of specificity. Such as for Osjap07g28390, sgRNA was designed on fusion site (Figure 4A in manuscript) and a divergent region (see the figure below).*

4. The authors present that an estimated 63 fusion genes could be generated in one species per million years. 63 fusion genes are a little scale of genes from the whole genome-wide perspective (63/52,424=0.0012) in 1 million years. My concern is about how many new genes would be formed by other ways like gene mutation, TE, recombination, etc., and comparing to these ways, whether new genes generated from gene fusion is a major mode for gene formation or at least one of the important ways. I think it's important for us to assess the value of this study, and authors should declare this question clear in Introduction or Discussion sections.

*Response: Thanks very much for the good suggestion. We have added some discussion in line 291. As Ohno (1970) pointed out, gene duplication is the canonical model for origination of new genes. Fusion gene derived from gene duplication [11] or exon shuffling [12] could make use of existing nucleotide sequences, and was estimated to affect a wider range of phenotypic outcomes than gene duplication for potential larger genetic diversity [13]. In an influential essay, Jacob F [14] even stated “The probability that a functional protein would appear de novo by random association of amino acids is practically zero”.*

5. I wonder why choosing the 4 species of Oryza in this study, and how often fusion genes happened in other species in Oryza and also in other plants besides of Oryza.

*Response: It was a very interesting question. We would like to reveal the answer in the near future in other Oryza and in other genus. We chose these four species because previous studies have proved that O. Sativa L. v.g. japonica and O. Sativa L. v.g. indica split from their common ancestor less than 0.44 MY [15, 16], and O. barthii and O. glaberrima split less than 0.40 MY . Fusion genes (new genes) study should pay attentions to catch the very subtle traces to made the logical sense of how it evolved, in this article, we employed a very strict threshold to make sure the result of fusion gens are reliable. The shorter time, the more accurate data we will get. On the other hand, Long et al [5] also pointed out, studies on new genes must focus on recently formed ones, which carry all the signatures of initial evolutionary forces, thus provide a valid system to investigate the structural evolution and parental properties. Thus, we chose these four focus species.*

6. The authors need to perform extra transformation by introducing the fusion gene into the crispr lines to elucidate the function of the candidate genes. Transgenic negative lines rather than wild type plants should be used as controls. Also, neither detailed description about the method nor the statistical for Fig. 5 was found.

*Response: Thanks for the suggestions about rescue and same transgenic negative line as control. Yes, we used the same Nipponbare line as control and knockout experiments. We have added a detailed description including this information to the legend of Figure 5. We also germinated these genotypes with more seeds of each line. We think that our setting up such control was stringent enough to detect phenotypic effects¬.¬¬¬¬¬ Other experiment of functional studies support this design [7, 8].*

Minor issues:

1. Uncontinuous line numbers over the whole MS make troubles for reviewing, the authors need add the continuous line number in the MS, for the ease of reviewing.

*Response: Thanks for your good suggestion. We have added the continuous line number in the revised manuscript.*

2. Methods described in Results section for the categorization of three major types of 69 expressed fusion genes should be moved to the Methods section.

*Response: Thank a lot. Categorization of three major types of 69 expressed fusion genes is part of result to reveal formation pattern, which wholly based on the presence and location of parental genes. So, dispose it in result looks also sensible.*

3. Figure quality need to be improved; many figures are unclear.

*Response: yes, we have improved our figures in the new version.*

4. Other errors:

1) Page 5, line 11, a redundant blank before 'However, it has been a challenge to understand the role ...'

*Response: We have corrected it.*

2) Page 7, line 4, 'developed' should be 'Developed'

*Response: Yes, we have corrected it.*

3) Arabic and English numbers used in MS is muddled, like page 9, line 2: '58 were shared between two to four species' English number 'two' used here; but at page 9, line 13 'with only 2 genes being 100%' Arabic number '2' used here; others like page 10, line 6, page 10, line 10; page 11, line 16, 22, 23; page 13 line 3, 9…

*Response: yes, we have unified with Arabic throughout the full text.*

4) page 9, line 3 line 9: 'Supplementary Table 1', 'Table 1 and Fig. 3', used here; but at page 9, line 12, line 17 'Supplementary Table2' was used. Page 10 line 9, 'Fig1'; page 9 line 9 'Fig. 1'; page 10 line 9, 'Supplemental Fig1'; page 8 line 5, 'Fig. 2 A, B & C' line 8 '(Fig. 2 D & E) and group (Fig. 2 A & F)'; page 12 line 14 'Fig. 6A'; please use a unified format and revise across the entire MS.

*Response: Thanks a lot, we have unified the format across the entire MS.*

5) page 14, line 28 'retrocopies they detected 1235 and 150, respectively', corrected to '1,235'.

*Response: Thanks very much, we have corrected it in line 321.*

6) page 18, line 20-22, error line spacing.

R*esponse: We have corrected it in line 421-424.*

7) Page 24 line 18: corrected to bold front of 'Knockout mutant generated by CRISPR/Cas9'.

*Response: We have corrected it in line 567.*

8) The marking of "sgRNA1" is missing in Figure 4A.

*Response: We have added the marking of sgRNA1 in Figure 4A.*

*Reference:*

*1. Stein JC, Yu Y, Copetti D, Zwickl DJ, Zhang L, Zhang C, Chougule K, Gao D, Iwata A, Goicoechea JL, et al: Genomes of 13 domesticated and wild rice relatives highlight genetic conservation, turnover and innovation across the genus Oryza. Nature Genetics 2018, 50:285-296.*

*2. Zhang L, Ren Y, Yang T, Li G, Chen J, Gschwend AR, Yu Y, Hou G, Zi J, Zhou R, et al: Rapid evolution of protein diversity by de novo origination in Oryza. Nature Ecology & Evolution 2019, 3:679-690.*

*3. Mortola E, Long M: Turning Junk into Us: How Genes Are Born. American Scientist 2021, 109:174+.*

*4. Ohno S: Evolution by gene duplication. New York: Springer Science & Business Media; 1970.*

*5. Long M, VanKuren NW, Chen S, Vibranovski MD: New gene evolution: little did we know. Annual Review of Genetics 2013, 47:307-333.*

*6. Chen S, Krinsky BH, Long M: New genes as drivers of phenotypic evolution. Nature Reviews Genetics 2013, 14:645-660.*

*7. Wang P, Zhang J, Sun L, Ma Y, Xu J, Liang S, Deng J, Tan J, Zhang Q, Tu L, et al: High efficient multisites genome editing in allotetraploid cotton (Gossypium hirsutum) using CRISPR/Cas9 system. Plant Biotechnology Journal 2018, 16:137-150.*

*8. Dou S, Zhang T, Tu J, Shen J, Yi B, Wen J, Fu T, Dai C, Ma C: Generation of novel self-incompatible Brassica napus by CRISPR/Cas9. Plant biotechnology journal 2021, 19:875-877.*

*9. Khodarahmpour Z: Effect of drought stress induced by polyethylene glycol (PEG) on germination indices in corn (Zea mays L.) hybrids. African Journal of Biotechnology 2011, 10:18222-18227.*

*10. Queiroz MS, Oliveira CE, Steiner F, Zuffo AM, Zoz T, Vendruscolo EP, Silva MV, Mello B, Cabra R, Menis FT: Drought stresses on seed germination and early growth of maize and sorghum. Journal of Agricultural Science 2019, 11:310-318.*

*11. Rogers RL, Shao L, Thornton KR: Tandem duplications lead to novel expression patterns through exon shuffling in Drosophila yakuba. PLOS Genetics 2017, 13:e1006795.*

*12. Hillstrom C: Origins of New Genes: Exon Shuffling.*

*13. Rogers RL, Hartl DL: Chimeric genes as a source of rapid evolution in Drosophila melanogaster. Molecular Biology and Evolution 2012, 29:517-529.*

*14. Jacob F: Evolution and tinkering. Science 1977, 196:1161-1166.*

*15. Wei X, Wang R, Cao L, Yuan N, Huang J, Qiao W, Zhang W, Zeng H, Yang Q: Origin of Oryza sativa in China inferred by nucleotide polymorphisms of organelle DNA. PLoS One 2012, 7:e49546.*

*16. Ma J, Bennetzen JL: Rapid recent growth and divergence of rice nuclear genomes. Proceedings of the National Academy of Sciences of the United States of America 2004, 101:12404-12410.*

**Second round of review**

**Reviewer 1**

The authors addressed all my points very careful and comprehensively. They added some experiments and analyses to strengthen their conclusions, and clarified some uncertainties by additional descriptions. I have no further comments.

**Reviewer 3**

In this work, Zhou et al. developed a novel bioinformatic pipeline GriffinDetector for identifying fusion genes, and benchmarked the pipeline in four Oryza genomes. Gene fusion, either through mutations in the alternative splicing site, or through gene duplication followed by chimeric gene formation is one of the most important molecular processes for generating genetic novelties to be exposed further to the natural selection. I consider this work a significant and novel research, following authors' previous work in origin and evolution of new genes, or evolution of chimeric genes. This pipeline is expected to be widely used in other species for identifying lineage-specific new gene fusions. The highlights of this work, in my opinions, are two layers: 1) first, the pipeline takes multiple species' phylogenetic relationship into context. This is important as without cross-species comparison, it is difficult to infer the detected case represent a result of gene fusion or gene fission. 2) The authors made the effort to functionally verify the candidate fusion gene, and found they had a phenotype in the corresponding tissues where they are predominantly expressed. 3) The authors also inspected these fusion genes' population frequency, presumably if a gene reached a high population frequency (>80% defined by the author), it is very likely a functional gene favored by natural selection, even when the transcriptome data is unavailable. Apart from these highlights, I still have some comments which I hope the authors can address or improve, as detailed below:

First regarding the answers addressed to the reviewer 2's questions, as asked by the editor, I think the authors addressed most of the questions except for question 2, that the authors need to provide evidence of RNA-seq reads spanning the fusion genes not just for the two chosen fusion genes, but all the identified fusion genes. Also for question 3, the authors need to 'list fusions for this situation' as requested by the reviewer 2. That is, those cases that the fused genes show a high sequence identity with its parental copies.

My major comments are as follows:

1. At the first paragraph, the authors need to clearly define what is considered as a fusion gene, and what are the major mechanisms for generating them? At line 49 of the first paragraph, they wrote "New genes formed by gene fusion can occur between 2 ancestrally neighboring genes", this is certainly only one path of generating fusion gene, and can be misleading. At a much later position, the authors clarified that gene fusion can form through DNA- or RNA-based mechanism. In addition, did the authors get a chance to study the mechanisms of generating these gene fusions? The authors did categorize the transcribed fusion genes into three types, but it was unclear what are the proportions of fusion genes produced by DNA- or RNA-based mechanisms?

2. The pipeline relies on pre-existing annotations of the input species, if I understand it correctly, which could vary due to the assembly quality and the annotation method and transcriptome resources. What I found somewhat strange is that many fusion gene identified have at least one of their parental gene without any transcription evidence. The author need to discuss about this, and I agree with the reviewer 2 that the authors need to provide more RNA-seq evidence for the fusion junction so that we can actually call them a fusion gene. To be more accurate, the title of the first paragraph should also be "Predicting New Gene Fusions", and so do other parts in the main text.

3. Line 161, the fusion genes identified in O. barthii have a much lower fixation rate (21.5%) compared to those of O. glaberrima, do the authors have any explanation for this?

4. Line 178-183, what about the rest genes among the 157 genes, apart from the 37 fusion genes without detectable FPKM signals, and the 69 gene with FPKMs?

5. Line 195, how do you define "novel expression patterns", in terms of expressed tissues or expression levels?

6. Line 225-235, what are the parental genes' expression pattern for these two selected fusion genes?

Other minor comments:

1. Line 45, including gene duplication, de novo evolution and gene fusion etc. There should also a statement here, as suggested by reviewer 2, about brief introduction of which mechanism is the major mechanism for producing new genes.

2. Line 57, have deleterious consequences upon their birth

3. Line 81: the low expression…makes..difficult: how does this new pipeline overcome this difficulty?

4. Line 106: originated

5. Line 109: it is unclear whether the pipeline identifies fusion genes from, or not from gene duplications. If not, here it should not be called "paralogous genes"

6. Line 110, different stages of transcriptome data

7. Line 146, 4 focused genome

8. Line 152, could be properly mapped onto the tree. But here it is also unclear what are those cases that could not be mapped onto the tree?

9. Line 157, a great variation of fixation rate

10. Line 180, again, if the 37 genes do not have FPKM signals, how are they identified, or are they really a gene?

11. Line 193, is in accord with

12. Line 197: have undergone neofunctionalization or subfunctionalization

13. Line 210: remove "randomly", selected one gene that potentially

14. Line 211, whose expression level, there should be some brief description about how these two genes were generated, by alternative splicing site skipping or duplication?

15. Line 230: growing conditions

16. Line 255: what do you mean by "is moved", is it a transposition or duplication?

**Authors Response**

**Point-by-point responses to the reviewers’ comments:**

Reviewer #1:

The authors addressed all my points very careful and comprehensively. They added some experiments and analyses to strengthen their conclusions, and clarified some uncertainties by additional descriptions. I have no further comments.

*Response: Thanks very much for your review. We really learned a lot from your insightful comments.*

Reviewer #3:

In this work, Zhou et al. developed a novel bioinformatic pipeline GriffinDetector for identifying fusion genes, and benchmarked the pipeline in four Oryza genomes. Gene fusion, either through mutations in the alternative splicing site, or through gene duplication followed by chimeric gene formation is one of the most important molecular processes for generating genetic novelties to be exposed further to the natural selection. I consider this work a significant and novel research, following authors' previous work in origin and evolution of new genes, or evolution of chimeric genes. This pipeline is expected to be widely used in other species for identifying lineage-specific new gene fusions. The highlights of this work, in my opinions, are two layers: 1) first, the pipeline takes multiple species' phylogenetic relationship into context. This is important as without cross-species comparison, it is difficult to infer the detected case represent a result of gene fusion or gene fission. 2) The authors made the effort to functionally verify the candidate fusion gene, and found they had a phenotype in the corresponding tissues where they are predominantly expressed. 3) The authors also inspected these fusion genes' population frequency, presumably if a gene reached a high population frequency (>80% defined by the author), it is very likely a functional gene favored by natural selection, even when the transcriptome data is unavailable. Apart from these highlights, I still have some comments which I hope the authors can address or improve, as detailed below:

First regarding the answers addressed to the reviewer 2's questions, as asked by the editor, I think the authors addressed most of the questions except for question 2, that the authors need to provide evidence of RNA-seq reads spanning the fusion genes not just for the two chosen fusion genes, but all the identified fusion genes. Also for question 3, the authors need to 'list fusions for this situation' as requested by the reviewer 2. That is, those cases that the fused genes show a high sequence identity with its parental copies.

“Question 2: Transcriptome data is an important evidence source for fusion genes, such as reads representing the fusion region of the identified genes. The authors could provide a set of discordant read pairs is mapped to two different genes, or when junction-spanning reads.”

Response: Thank you for your positive feedback and give more suggestive comments. As we listed below, we found more RNA-seq reads spanning the fusion gene junction. Red arrow in each picture shows the fusion site, where reads are junction-spanning.

Osjap01g00640.1 Osjap01g00500.1 Osjap01g08660.1

Osjap01g31520.1 Osjap01g33510.1 Osjap10g04590.1

Osjap01g42770.1 Osjap02g23220.1 Osjap01g50410.1

Osjap09g15430.1 Osjap02g28800.3 Osjap02g30600.1

Osjap03g13100.1 Osjap03g34330.1 Osjap03g44070.1

Osjap03g44320.1 Osjap03g44530.1 Osjap04g02830.1

Osjap05g02530.1 Osjap05g04120.1 Osjap05g14570.1

Osjap05g20040.1 Osjap06g00390.1 Osjap06g08120.1

Osjap06g27840.1 Osjap06g31140.1 Osjap07g04240.1

Osjap07g25530.1 Osjap08g19480.1 Osjap09g00700.1

“Question 3: It seems that the gene identity between parental genes and fusion genes are very high (Figure 4). Because some parental genes were not in the collinear region of fusion genes (Figure 6), the parental gene copies are co-existed with the fusion genes in the same genome. Please list fusions for this situation and add information for the target sites of sgRNA whether also targeted parental gene copies.”

At the same time, we also recheck the primer of two CRISPR/Cas9 edited fusion gene (Osjap07g28390 and Osjap0915430 are fusion genes, Osjap04g19780 and Osjap0915440 are their parental genes respectively), it comes out as below:

My major comments are as follows:

1. At the first paragraph, the authors need to clearly define what is considered as a fusion gene, and what are the major mechanisms for generating them? At line 49 of the first paragraph, they wrote "New genes formed by gene fusion can occur between 2 ancestrally neighboring genes", this is certainly only one path of generating fusion gene, and can be misleading. At a much later position, the authors clarified that gene fusion can form through DNA- or RNA-based mechanism. In addition, did the authors get a chance to study the mechanisms of generating these gene fusions? The authors did categorize the transcribed fusion genes into three types, but it was unclear what are the proportions of fusion genes produced by DNA- or RNA-based mechanisms?

*Response: Thank you for the excellent suggestion. We have add the sentence “In term of the parental materials (DNA or RNA sequence) that produced to process, the fusion event results into fusion gene and retro-fusion gene, respectively” in first paragraph. We categorize the fusion genes into three types by the presence and location of parental gene, which can not discriminate the DNA- or RNA-based mechanisms clearly. After we check the presence of parental intron loss in genome, we only detected 2 cases of retro-fusion genes (Figure 8) out of a total of 69 fusion genes which have transcription evidence.*

2. The pipeline relies on pre-existing annotations of the input species, if I understand it correctly, which could vary due to the assembly quality and the annotation method and transcriptome resources. What I found somewhat strange is that many fusion gene identified have at least one of their parental gene without any transcription evidence. The author need to discuss about this, and I agree with the reviewer 2 that the authors need to provide more RNA-seq evidence for the fusion junction so that we can actually call them a fusion gene. To be more accurate, the title of the first paragraph should also be "Predicting New Gene Fusions", and so do other parts in the main text.

*Response: Thank you for the excellent suggestion. Yes, this pipeline relies on the pre-existing annotations of the input species. We divide all phylogenetic species into 3 group (in group, mid-group and out-group) to improve the robustness, which could avoid the annotation bias largely. Why many fusion gene have at least one of their parental gene without any transcription evidence is of interesting and maybe because of it is functional constraint. We also have added more RNA-seq evidence as aforementioned. We changed the title of first paragraph in Results as suggested “Predicting New Gene Fusions”.*

3. Line 161, the fusion genes identified in O. barthii have a much lower fixation rate (21.5%) compared to those of O. glaberrima, do the authors have any explanation for this?

*Response: Yes, we found O. glaberrima with very high fixation rate. After we discussed with O. glaberrima genome expert Dr. Muhua Wang, who conducted population genetics on O. glaberrima. He also found this interesting phenomenon, he and other studies found that it is a result of natural selection with bottleneck effect [1-4]. We also discussed that in Line 337-339.*

4. Line 178-183, what about the rest genes among the 157 genes, apart from the 37 fusion genes without detectable FPKM signals, and the 69 gene with FPKMs?

*Response: There are 97 long-copy gene isoforms have homologus gene in all six species, other 60 gene isoforms were absent in at least one species. Among the 97 gene isoforms identified in all species, 37 gene isoforms without detectable FPKM, 60 fusion genes with expression value in at least one tissue (Table S3). The 69 fusion genes that had detectable expression signals in O. sativa v.g. japonica specially refer to expression pattern of fusion genes in japonica, and not related to above-mentioned isoforms.*

5. Line 195, how do you define "novel expression patterns", in terms of expressed tissues or expression levels?

*Response: We define novel expression patterns by having no expression correlation between fusion gene and parental genes in different tissues.*

6. Line 225-235, what are the parental genes' expression pattern for these two selected fusion genes?

*Response: As indicated in Table S4, parental gene of Osjap09g15430 have similar expression pattern with Osjap09g15430 (both have highest expression in panicle); parental gene of Osjap07g28390 (expressed in panicle) have different expression with fusion gene Osjap07g28390 (expressed in root).*

Other minor comments:

1. Line 45, including gene duplication, de novo evolution and gene fusion etc. There should also a statement here, as suggested by reviewer 2, about brief introduction of which mechanism is the major mechanism for producing new genes.

*Response: Thank you for the excellent suggestion. We have changed the sentence as “a number of molecular mechanisms including gene duplication, de novo evolution and gene fusion”.*

2. Line 57, have deleterious consequences upon their birth.

*Response: We have change the sentence as “even though such fused genes may have deleterious consequences upon their birth”.*

3. Line 81: the low expression…makes..difficult: how does this new pipeline overcome this difficulty?

*Response: We used genome annotated gene models for chimeric gene identification in this pipeline, not the transcriptome based result, so this pipeline duck out this question smartly.*

4. Line 106: originated

*Response: We have changed the “originating” into “originated” in Line 103.*

5. Line 109: it is unclear whether the pipeline identifies fusion genes from, or not from gene duplications. If not, here it should not be called "paralogous genes".

*Response: Actually, this pipeline identifies fusion gene derived from gene duplication, so we keep it as “paralogous genes” in Line 110.*

6. Line 110, different stages of transcriptome data

*Response: We have changed it as “different stages of transcriptome data”.*

7. Line 146, 4 focused genome

*Response: We have changed it into “4 focused genome”.*

8. Line 152, could be properly mapped onto the tree. But here it is also unclear what are those cases that could not be mapped onto the tree?

*Response: Thanks for the good comments. Those fusion gene can not be mapped onto the tree maybe because reticular radiation evolution of plant kingdom, so the non-species specific fusion gene cannot totally mapped onto the simple species tree.*

9. Line 157, a great variation of fixation rate

*Response: Thanks so much. We have changed the sentence as “they showed a great variation of fixation rate”.*

10. Line 180, again, if the 37 genes do not have FPKM signals, how are they identified, or are they really a gene?

*Response: Yes. We detected the fusion gene by applied genome annotated genome model, so they can be identified without transcript signal. After finished, we can explore their expression level by transcriptome or gene id in other database.*

11. Line 193, is in accord with

*Response: Thank you. We have done that in Line 191.*

12. Line 197: have undergone neofunctionalization or subfunctionalization

*Response: We have changed the sentence as “over one third of the candidate fusion genes may have undergone neofunctionalization or subfunctionalization”.*

13. Line 210: remove "randomly", selected one gene that potentially

*Response: Thank you. We have deleted the “randomly”.*

14. Line 211, whose expression level, there should be some brief description about how these two genes were generated, by alternative splicing site skipping or duplication?

*Response: Thanks. We have changed it as “whose expression level” in Line 211, and the two fusion gene derived from duplication because they have existed parental gene.*

15. Line 230: growing conditions

*Response: We have changed it as “under identical growing”.*

16. Line 255: what do you mean by "is moved", is it a transposition or duplication?

*Response: Yes, “is moved” means transposition. We have changed as “one parental gene is transposed to an adjacent region of…”.*

*Reference:*

*1. Veltman MA, Flowers JM, van Andel TR, Schranz ME: Origins and geographic diversification of African rice (Oryza glaberrima). PLOS ONE 2019, 14:e0203508.*

*2. Semon M, Nielsen R, Jones MP, McCouch SR: The Population Structure of African Cultivated Rice Oryza glaberrima (Steud.): Evidence for Elevated Levels of Linkage Disequilibrium Caused by Admixture with O. sativa and Ecological Adaptation. Genetics 2005, 169:1639-1647.*

*3. Wang M, Yu Y, Haberer G, Marri PR, Fan C, Goicoechea JL, Zuccolo A, Song X, Kudrna D, Ammiraju JSS, et al: The genome sequence of African rice (Oryza glaberrima) and evidence for independent domestication. Nature Genetics 2014, 46:982-988.*

*4. Nabholz B, Sarah G, Sabot F, Ruiz M, Adam H, Nidelet S, Ghesquière A, Santoni S, David J, Glémin S: Transcriptome population genomics reveals severe bottleneck and domestication cost in the African rice (Oryza glaberrima). Molecular Ecology 2014, 23:2210-2227.*

**Third round of review**

**Reviewer 3**

The authors revised their manuscript accordingly, and I recommend accepting the paper after the authors addressing the minor issues that I detail below:

1. In one response to the question, the author didn't seem to finish the sentence: "At the same time, we also recheck the primer of two CRISPR/Cas9 edited fusion gene (Osjap07g28390 and Osjap0915430 are fusion genes, Osjap04g19780 and Osjap0915440 are their parental genes respectively), it comes out as below:

". What comes out as below?

**Authors Response**

**Point-by-point responses to the reviewers’ comments:**

Reviewer #3:

The authors revised their manuscript accordingly, and I recommend accepting the paper after the authors addressing the minor issues that I detail below:

1. In one response to the question, the author didn't seem to finish the sentence: "At the same time, we also recheck the primer of two CRISPR/Cas9 edited fusion gene (Osjap07g28390 and Osjap0915430 are fusion genes, Osjap04g19780 and Osjap0915440 are their parental genes respectively), it comes out as below:

". What comes out as below?

*Response: Thank you so much for the suggestion. We have changed it as “At the same time, we also recheck the primer of two CRISPR/Cas9 edited fusion gene (Osjap07g28390 and Osjap0915430 are fusion genes, Osjap04g19780 and Osjap0915440 are their parental genes respectively). The sgRNA comes out as below: the parental gene copy Osjap04g19780 was not in the collinear region of fusion gene Osjap07g28390 but divergent with fusion gene; parental gene copy Osjap09g15440 and Osjap09g15430 were located in the adjacent collinear region with different flanking sequence”.*
